# Changes in fatty acid composition as a response to glyphosate toxicity in *Pseudomonas fluorescens*

**DOI:** 10.1016/j.heliyon.2022.e09938

**Published:** 2022-07-13

**Authors:** Elizangela Paz de Oliveira, Kathleen Evelyn Marchi, Janaina Emiliano, Stella Marys Christóforo Hinojosa Salazar, Alisson Henrique Ferri, Rafael Mazer Etto, Péricles Martim Reche, Sônia Alvim Veiga Pileggi, Karlos Henrique Martins Kalks, Marcos Rogério Tótola, Zelinda Schemczssen-Graeff, Marcos Pileggi

**Affiliations:** a Department of Biotechnology, Genetics and Cell Biology, Maringá State University, Maringá, Paraná, Brazil; b Department of Structural and Molecular Biology and Genetics, Ponta Grossa State University, Ponta Grossa, Paraná, Brazil; c Department of Microbiology, Londrina State University, Londrina, Paraná, Brazil; d Department of Chemistry, Ponta Grossa State University, Ponta Grossa, Paraná, Brazil; e Department of Nursing and Public Health, Ponta Grossa State University, Ponta Grossa, Paraná, Brazil; f Department of Microbiology, Federal University of Viçosa, Viçosa, Minas Gerais, Brazil; g Department of Cellular Biology, Federal University of Paraná, Curitiba, Paraná, Brazil

**Keywords:** Herbicide, Oxidative enzyme, Physiological plasticity, Oxidative stress response, Lipidic peroxidation, Selective pressure

## Abstract

Excessive use of herbicides decreases soil biodiversity and fertility. The literature on the xenobiotic response by microorganisms is focused on herbicide biodegradation as a selective event. Non-degradation systems independent of selection could allow the survival of tolerant bacteria in contaminated environments, impacting xenobiotic turnover and, consequently, bioremediation strategies. However, it is uncertain whether the response based on these systems requires selective pressure to be effective. The objective here was to analyze non-degradation phenotypes, enzymatic and structural response systems, of *Pseudomonas fluorescens* CMA-55 strain, already investigated the production pattern of quorum sensing molecules in response to glyphosate, not present at the isolation site. One mode of response was associated with decrease in membrane permeability and effective antioxidative response for 0–2.30 mM glyphosate, at the mid-log growing phase, with higher activities of Mn-SOD, KatA, and KatB, and presence of fatty acids as nonadecylic acid, margaric and lauric acid. The second response system was characterized by lower antioxidative enzymes activity, presence of KatC isoform, and pelargonic, capric, myristic, stearic, palmitoleic and palmitic acid as principal fatty acids, allowing the strain to face stressful conditions in 9.20–11.50 mM glyphosate at the stationary phase. Therefore, the bacterial strain could modify the fatty acid composition and the permeability of membranes in two response modes according to the herbicide concentration, even glyphosate was not previously selective for *P. fluorescens*, featuring a generalist system based on physiological plasticity.

## Graphical abstract

Graphical representation of the main results showing the different response systems to the glyphosate herbicide of *Pseudomonas fluorescens* CMA-55 7729. This strain originated from biofilms formed in water storage tanks used for washing herbicide containers. No glyphosate container was washed with this water. The left panel represents the enzymatic responses with high activity of Mn-SOD and KatA, and KatB isoenzymes; and specific fatty acids produced at 0x, 1x, and 10x glyphosate concentrations, with high viability rates. The right panel represents the enzymatic responses with low activity of Mn-SOD and KatA, KatB, and KatC isoenzymes; and different fatty acids produced at 40x and 50x glyphosate concentrations, with low viability rates.

**Figure:**
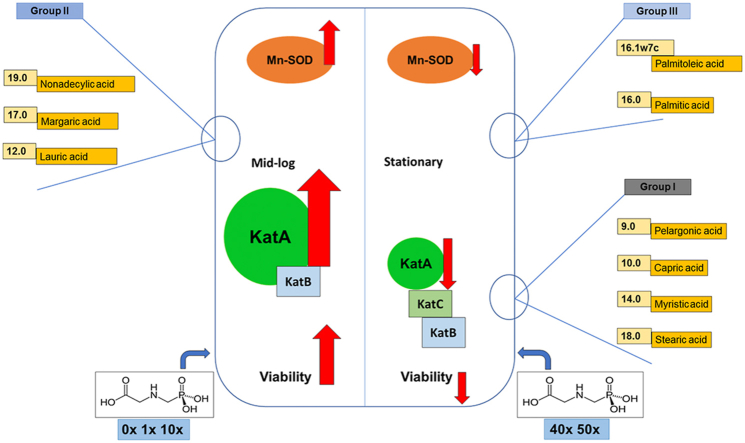

## 1 Introduction

Herbicides are important tools in promoting agricultural development, despite being the main class of pollutants in water (Huang et al., 2020). One of the best-known products, glyphosate [N-(Phosphonomethyl) glycine] is the active ingredient in several commercial herbicides, including Roundup, Clinc EV, Glyfos, Glyphogan, and Kapazino (Grube et al., 2019). Specifically, Monsanto’s product Roundup, had been utilized in soybean crop production which was genetically modified to be resistant to this herbicide; soybeans are now the most cultivated transgenic crop in the world (Kniss, 2018). Glyphosate inhibits the enzyme 5-enolpyruvyl-shikimate-3-phosphate synthase (EPSPS), a key enzyme in the synthesis of aromatic amino acids in the shikimic acid pathway, and increases reactive oxygen species (Sobjak et al., 2017). The enzyme inhibition impairs the synthesis of proteins necessary for various vital cellular processes (Camacho and Mejía, 2017).

The excessive use of glyphosate in crops has caused important problems for agriculture and the environment, as the selection of weed resistance to 5-enolpyruvyl-shikimate-3-phosphate synthase inhibitors (Alcántara-de la Cruz et al., 2019); and glyphosate dispersion through leaching (Singh et al., 2020).

An additional problem in being found in many environmental compartments is that glyphosate is toxic to non-target organisms. Native plant species from Chaco forests in Argentina presented situations of phytotoxicity, growth reduction, and sensitivity to this herbicide (Florencia et al., 2017). The common duckweed *Lemna minor*, presented a decrease in growth, yield, and photochemical activity of photosystem II (Sikorski et al., 2019). This herbicide causes moderate toxicity and high irritability in the Coleoptera *Cerotoma arcuata* (Pereira et al., 2018). Disruption in energy metabolism process and Ca^2+^ homeostasis, cell signaling, and endoplasmic reticulum stress response were observed in the marine bivalve *Mytilus galloprovincialis* by the presence of glyphosate (Milan et al., 2018). This herbicide is neurotoxic to rats, mice, *Caenorhabditis elegans*, zebrafish and humans, including their gut microbiota (Richardson et al., 2019). Glyphosate-based herbicides were classified as “probably carcinogenic to humans” by the International Cancer Research Agency in 2015 (Grube et al., 2019).

The herbicide can affect the diversity of soil microbiota, as he relative abundance of nitrogen-fixing bacteria (Yang et al., 2020), and by affecting some ecological and metabolic activities essential for soil maintenance and fertility (Marques et al., 2021). Glyphosate also reduced mycorrhizal colonization (Helander et al., 2018), phosphate solubilizing (Anzuay et al., 2017) and it had adverse effects over siderophore production by soil microorganisms, including *Pseudomonas fluorescens* (Kumar et al., 2019; Tyagi et al., 2020)*.*

Some of the harmful measures that herbicides cause are done by reactive oxygen species (ROS), which generate oxidative stress conditions that inhibit microbial growth by damaging membrane lipids and proteins. Oxidative stress occurs when there is a disruption in the balance between the effects of environmental pollutants and enzymatic and non-enzymatic antioxidant defenses (Choudhury et al., 2017). Microorganisms that are exposed to herbicides and oxidative stress conditions, activate antioxidative enzymes and other response systems (Pileggi et al., 2020; Rovida et al., 2021). Other biocide tolerance mechanisms are related to membrane lipid composition changes. *Pseudomonas putida*, for example, exhibits an adaptive response to quaternary ammonium compounds via impaired phospholipid enzymatic changes (Boeris et al., 2007).

The excessive use of different pesticides has the consequences of decreasing biodiversity and soil fertility (Baćmaga et al., 2019), and the literature on xenobiotic response systems is mainly focused on the presence of the selective agents in the environment, as in the case of herbicide degradation genes (Lima et al., 2020). Therefore, it is uncertain whether response systems based on non-degradation genes require specific selective pressure to be effective against contaminants.

Therefore, this work analyzed the antioxidative enzymes and fatty acid profiles as response systems of a bacterial strain of *Pseudomonas fluorescens* that had no previous contact with glyphosate, analyzing the hypothesis of being a generalist system.

## 2 Material and methods

### 2.1 Bacterial strains

The bacterium used in this study, *P. fluorescens* CMA-55, was selected for being an isolate to show the best growth rates at a concentration of 50x glyphosate. This strain was previously evaluated for the production of specific quorum sensing signaling molecules in response to herbicides, including glyphosate (Freitas et al., 2021). It was obtained from the Collection of Environmental Microorganisms at the Laboratory of Environmental Microbiology of Ponta Grossa State University, Brazil, which had been isolated from biofilms formed in storage tanks of water used for washing herbicide containers (Lima et al., 2020).

The bacterial strains were identified by sequencing of the 16S rRNA gene. Total DNA was extracted using a DNA isolation kit (Promega, Madison, WI, US) and 16S rRNA was amplified by PCR using universal primers 27F and 1492R (Lane, 1991). DNA was sequenced on an ABI 3500 xL Genetic Analyzer (Applied Biosystems, Foster City, CA, USA). The sequences were deposited in the GenBank database (http://www.ncbi.nlm.nih.gov) under accession number KY807296, which corresponds to *P. fluorescens*, with a 98.35% identity. This strain was deposited at the Johanna Döbereiner Biological Resources Center (CRB-JD/Embrapa, Brazil), under the code BR 14566.

### 2.2 Herbicide

The commercial formulation of glyphosate used was Roundup Transorb R (Monsanto, St. Louis, MO, USA), which contains 588 g/L of glyphosate potassium salt, with 480 g/L equivalent to N-phosphonomethyl glycine glyphosate and 820 g/L of inert ingredients.

### 2.3 Tolerance test

The tolerance test was performed in Petri dishes containing Luria Bertani Agar (LA: 10 g/L, tryptone, 5 g/L yeast extract, 10 g/L NaCl, 20 g/L agar). The bacteria were grown in plates containing various concentrations of glyphosate, to observe the growth capacity and tolerance levels to the herbicide: control, with no herbicide (0x); 0.23 mM glyphosate (1x concentration used in agriculture); 2.30 mM glyphosate (10x); 9.20 mM glyphosate (40x); and 11.50 mM glyphosate (50x). Strains were considered tolerant for forming colonies on media containing different concentrations of glyphosate.

### 2.4 Bacterial growth

The strain was grown, as a pre-inoculum, in Mineral Medium (MM: 10 mM potassium phosphate buffer, pH 7.0, 3 g/L NaNO_3,_ 0.5 g/L MgSO_4,_ 0.5 g/L KCl, 0.01 g/L FeSO_4,_ 0.04 g/L CaCl_2,_ 0.001 g/L MnSO_4_), with an addition of 20 g/L glucose, at 30 °C, for 20 h. After this period, the culture was inoculated in the following treatments, in triplicate: 0x, 1x, 10x, 40x, and 50x. The inoculants were standardized to start at an optical density (OD) of 0.05, and at an absorbance of 600 nm. The samples were diluted when they reached OD values of approximately 1.0, and the values were multiplied by the corresponding dilution factors.

### 2.5 Protein extraction for oxidative stress analysis

Bacterial cultures were grown as described (Section 3.4). The proteins were extracted in three periods: 20 h, (early-log phase), 30 h, (mid-log phase), and 40 h, (stationary phase). The material was centrifuged at 5,000 *g* for 15 min, and the precipitate macerated with liquid nitrogen and homogenized in 1:10 m/v of a 100 mM solution of potassium phosphate buffer (14.520 g/L K_2_HPO_4_; 2.260 g/L KH_2_PO_4_), pH 7.5; 0.372 g/L ethylenediamine tetraacetic acid (EDTA); 0.462 g/L DL-dithiothreitol; 5% (w/w) polyvinyl polypyrrolidone (PVPP, 10:1 volume of buffer: sample weight), at 4 °C, and centrifuged at 10,000 *g* for 30 min. The supernatant was stored at –80 °C. The protein concentrations were measured using the (Bradford, 1976) method, with bovine serum albumin as the standard. The results were expressed in μmol Protein/g of fresh weight (Rodrigues et al., 2010).

### 2.6 Hydrogen peroxide

The quantification of hydrogen peroxide (H_2_O_2_) was performed from the reaction of 200 μL of sample [100 mg of protein extract homogenized with 1 mL of 0.1% trichloroacetic acid (TCA), and centrifuged at 10,000 *g* for 15 min, at 4 °C], with 200 μL of potassium phosphate buffer (pH 7.5), and 800 μL of 1 M potassium iodide, for 1 h, on ice, in the dark. The iodine, released in this reaction, was quantified in a spectrophotometer at 390 nm. The results were expressed in μmol/g of fresh weight (Gravina et al., 2017).

### 2.7 Lipid peroxidation

Bacterial growth and pre- inoculation were performed as described previously (Section 3.4.). Lipid peroxidation was determined in a spectrophotometer at 530 and 600 nm, by the production of malondialdehyde (MDA), which is a metabolite reactive to 2-thiobarbituric acid (TBA). A volume of 250 μL of the sample (Section 2.6.), 200 μL of potassium phosphate buffer (pH 7.5) and 1 mL of 20% TCA +0.1% TBA, were added, which was maintained for 30 min in a water-bath at 97 °C. The sample remained for 10 min on ice before centrifugation at 10,000 *g* for 10 min, and the supernatant was read on a spectrophotometer. Calculations were made using an extinction coefficient of 155 mM cm^−1^. The amount of MDA was expressed as μmol MDA g^−1^ fresh weight.

### 2.8 Cell viability

The bacterial cultures were obtained under growing conditions with glyphosate treatments as described previously (Section 3.4). Cells were recovered by centrifugation and diluted in a NaCl 0.9% buffer to remove herbicide residues. The cultures, in triplicate, were incubated on LA plates. The dilutions were made until 25–300 colonies were obtained per plate, after an incubation at 30 °C for 24 h (Dobrzanski et al., 2018).

### 2.9 SOD isoforms

Isoforms of superoxide dismutase (SOD) were separated on 12% non-denaturing polyacrylamide electrophoresis (PAGE) gels (Dobrzanski et al., 2018). Isoforms were classified as Mn-SOD if resistant to KCN and H_2_O_2_ inhibitors, as Fe-SOD if resistant to KCN and inhibited by H_2_O_2_, and as Cu/Zn-SOD if inhibited by both substances (Azevedo et al., 1998).

### 2.10 SOD activity in non-denaturing PAGE

The activities of the SOD isoforms were evaluated using non-denaturing 12% PAGE, at a constant current of 15 mA for 3 h using 20 μg of each protein extract (Section 2.6) per lane. The gels were washed with deionized water and incubated in the dark at room temperature in a 50 mM potassium phosphate buffer (pH 7.8) containing 1 mM EDTA, 0.05 mM riboflavin, 0.1 mM nitroblue tetrazolium (NBT) and 0.3% N, N, N ′, N′-tetramethylethylenediamine (TEMED). This solution was discarded after 30 min of reaction. The gels were washed with deionized water and placed under fluorescent lighting to identify the bands (Cia et al., 2012).

### 2.11 Catalase activity in non-denaturing PAGE

Catalase (CAT) activity was determined using 12% non-denaturing PAGE as reported by (Dourado et al., 2015). A current of 15 mA per gel was applied for 17 h at 4 °C with 15 μg of protein from the samples (Section 2.6). The gels were washed with deionized water (3 times for 15 min) and incubated in 0.003% H_2_O_2_ for 10 min and transferred to a 1% (w/v) FeCl_3_ solution and 6 to 1% K_3_Fe (CN) solution (w/v) for 10 min for developing of bands.

### 2.12 Fatty acid analysis

#### 2.12.1 Sample preparation

Cells were grown in LB, as previously described (Section 3.4). Cultures e were centrifuged at 10,000 *g* for 5 min. The cell mass (40 mg) was saponified by the addition of 1 mL of a reagent solution containing 45 g NaOH, 150 mL methanol and 150 mL of deionized water. The samples were vortexed for 10 s, heated for 5 min in a water bath at 100 °C, stirred again for 10 s, and heated for 25 min at 100 °C. The samples were cooled in a water bath until reaching room temperature and transferred to 50-mL culture tubes.

The samples went through a fatty acid methylation process with the addition of a 2 mL solution containing 325 mL 6 M HCl and 275 mL methanol. The samples were vortexed for 10 s, heated to 80 °C for 10 min, and cooled in a water bath until reaching room temperature. The esters formed were extracted with 1.25 mL of a solvent solution, consisting of hexane and tert-butyl methyl ether (50:50), on a platform shaker for 10 min at low speed. The organic phase was separated into a 50 mL centrifuge tube, to which 3 mL of a 1.66 M NaOH solution was added and vortexed for 5 min. The tubes were centrifuged at 2,000 rpm for 3 min. The organic phase was separated and stored in a 2 mL vial (Sharmili and Ramasamy, 2016).

#### 2.12.2 Chromatographic analysis

Fatty acid samples, obtained as described in the previous section, were analyzed using a (CG) Sherlock MIS (Agilent Technologies 6890 or 6850 Santa Clara, CA, USA), gas chromatograph equipped with an automatic sampler, an Ultra 2 capillary column and fitted with a flame Ionization detector. Oven temperature was ramped up from 170 to 270 °C at 5 °C/min, as described by Sasser (2001).

### 2.13 Statistical analysis

The data referring to the bacterial growth (Supplementary Material 1) quantification of cell viability, H_2_O_2_, and MDA (Supplementary Material 2), were obtained in triplicate for each treatment, with the differences evaluated using Bonferroni's analysis of variance.

The membrane fatty acid compositions were analyzed using the Principal Component Analysis (PCA) on the correlation matrix, in the R program (Development Core Team, 2007) with the Vegan package (Oksanen et al., 2008). To analyze the linear correlations between the membrane fatty acid composition (results of various concentrations of glyphosate herbicide), cell H_2_O_2_ production and the MDA concentration of the first two axes of the PCA, the envfit()-function was applied, using 999 permutations. These linear correlations were subsequently plotted onto PCA-graphs (Supplementary Material 3).

## 3 Results and discussion

### 3.1 Glyphosate tolerance in *Pseudomonas fluorescens*

The excessive use of different pesticides has the consequences of decreasing biodiversity and soil fertility (Baćmaga et al., 2019). From the biofilms formed in storage tanks of water used for washing pesticide containers (Lima et al., 2020), 33 bacterial strains were isolated in our work, and 12 were considered sensitive to glyphosate, 21 were tolerant up to 1x the herbicide, 14 were tolerant up to 10x, and 6, including *P. fluorescens* CMA-55, were tolerant up to 50x the herbicide, even without prior selective pressure for this molecule in the water tanks where it was isolated. Concentrations above those used in agriculture constituted our strategy to test the limits of efficiency of bacterial response systems. Furthermore, high concentrations can be found in the environment, either by trying to effectively control resistant weeds, or by its stability inside the cells of plants with a slower metabolization of the herbicide (Fartyal et al., 2018).

The literature on xenobiotic response systems is mainly focused on the presence of the selective agent in the environment, as in the case of herbicide degradation genes (Lima et al., 2020). It is uncertain whether bacterial glyphosate tolerance is based on enzymes involved in antioxidative responses and whether changes in lipid saturation require specific selective pressure to be effective against this herbicide. A more accurate definition of this issue can improve the understanding of the turnover of glyphosate in environment and xenobiotic bioremediation strategies. The strain *P. fluorescens* CMA-55 was also studied by Freitas et al. (2021), noting that it had a profile of quorum sensing signaling molecules involved in controlling the production of reactive oxygen species for saflufenacil and glyphosate herbicides, and involved in different stages of biofilm formation in the presence of sulfentrazone, 2,4-D and dicamba herbicides.

**Figure 1:**
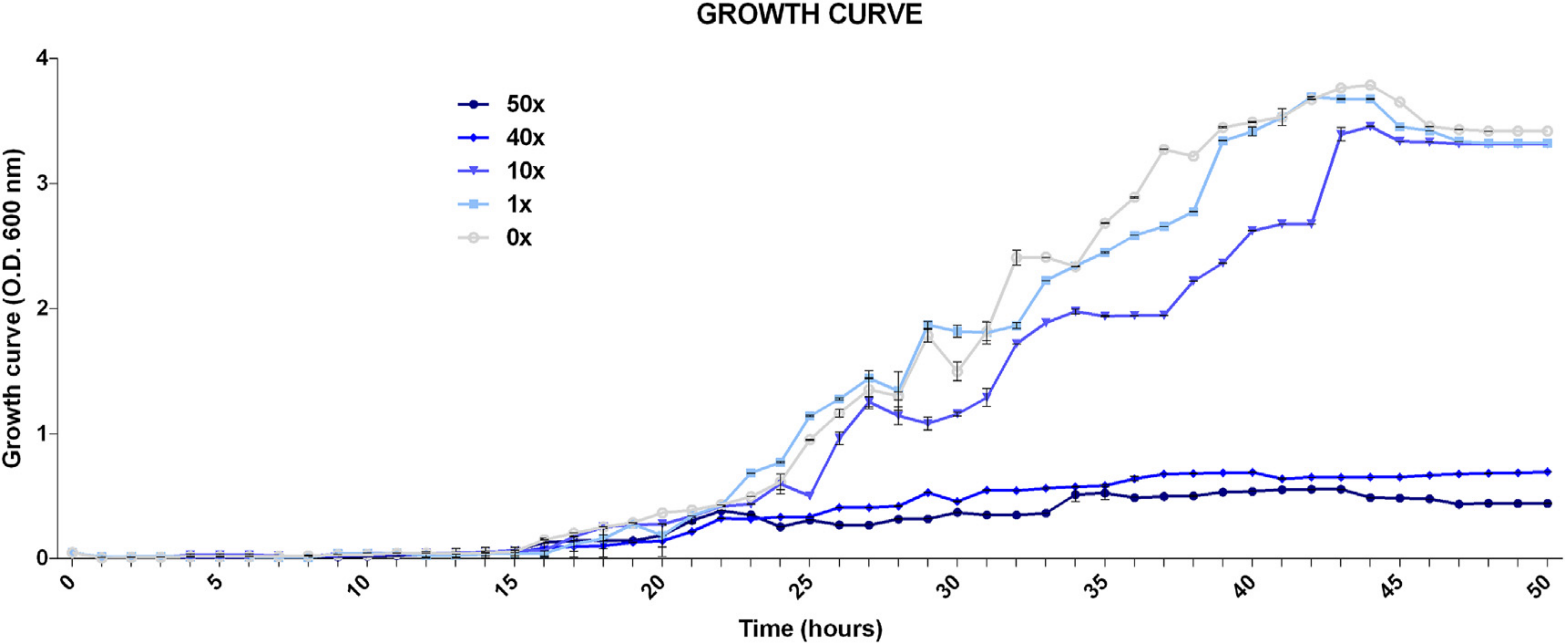
*P. fluorescens* CMA-55 growth curve in the treatments with 0x, 1x, 10x, 40x, and 50x the concentrations of glyphosate up to 50 h of incubation. Readings at 600 nm. Tukey’s test was used with p < 0.05 and the bars represent the standard error of the averages.

The growth curve of *P. fluorescens* CMA-55 in the 0x condition served as the baseline for determining the growth phases in which the data were obtained (Figure 1). In this way, the early-log phase was standardized occurring at 20 h of incubation, the mid-log phase at 30 h, and the stationary phase at 40 h. According to Figure 1, no significant differences in growth rates were found in 0x and 1x; however, there were significant differences among 0x and the treatments of 10x, 40x, and 50x, suggesting toxic effects of glyphosate for this strain at these higher concentrations.

Microorganisms present in aquatic environments may suffer selective pressure due to contact with herbicides (Iori et al., 2020). Bacteria can be selected for specific resistance to glyphosate through the elevation of the production of the EPSPS; herbicide degradation; detoxification by covalent modification; and decreasing uptake and increasing export of herbicide (Hertel et al., 2021). Nevertheless, we found no reports in the literature about response systems that were not induced by the selective pressure of specific xenobiotics.

### 3.2 Indicators of oxidative stress in *Pseudomonas fluorescens*

#### 3.2.1 Cell viability

**Figure 2:**
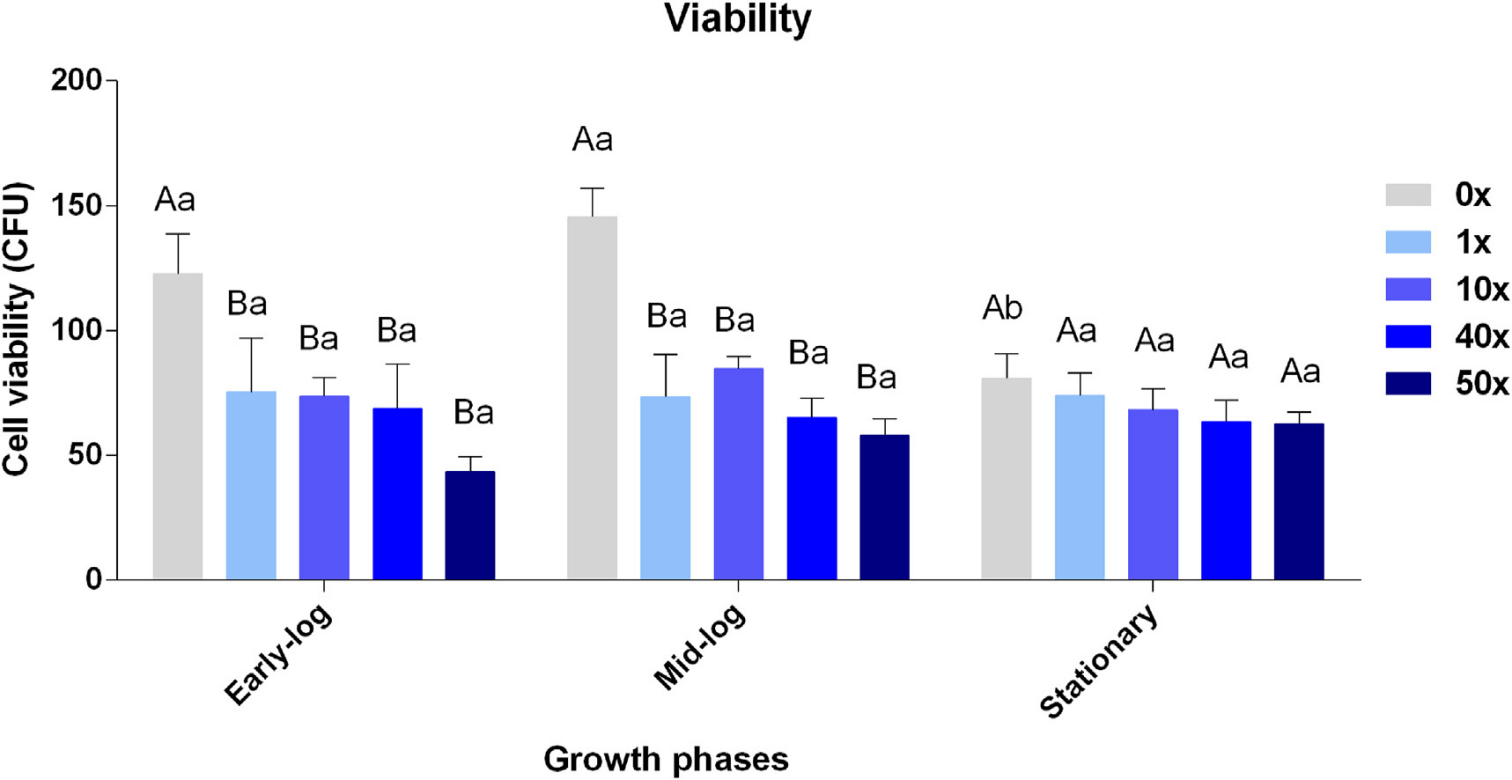
Cell viability in *P. fluorescens* CMA-55 in the treatments with 0x, 1x, 10x, 40x, and 50x glyphosate concentrations, in the early-log, mid-log, and stationary growing phases. The data were obtained in triplicates for each treatment and statistically analyzed using the complete block design through analysis of variance (two-way ANOVA), followed by Tukey’s post hoc test. Error bars represent statistically significant differences between treatments at the same time. Capital letters represent statistically significant differences between treatments at different times. Significance was set at p < 0.05.

Cell viability is a stress indicator characterized by the number of cells capable of dividing at a specified time and treatment. In this work, three time periods were established, as described before, in which the viability data in the control are significantly higher than the glyphosate treatments (Figure 2), indicating the toxic effect of this herbicide for *P. fluorescens* CMA-55, even being considered a tolerant strain. These phases are distinct in *Pseudomonas* because they are associated with gene regulation and metabolic changes in bacteria (Alkhalaf et al., 2021), allowing us to follow the evolution of the glyphosate toxicity response system.

The viability of *Pseudomonas* sp. isolated from soils decreased when exposed to glyphosate, as sensitivity was considered an inherent characteristic of this genus (Aristilde et al., 2017). However, *P. fluorescens* CMA-55, presented an efficient response system to glyphosate, tolerating concentrations of up to 10x (Figure 1).

#### 3.2.2 Quantification of hydrogen peroxide

**Figure 3:**
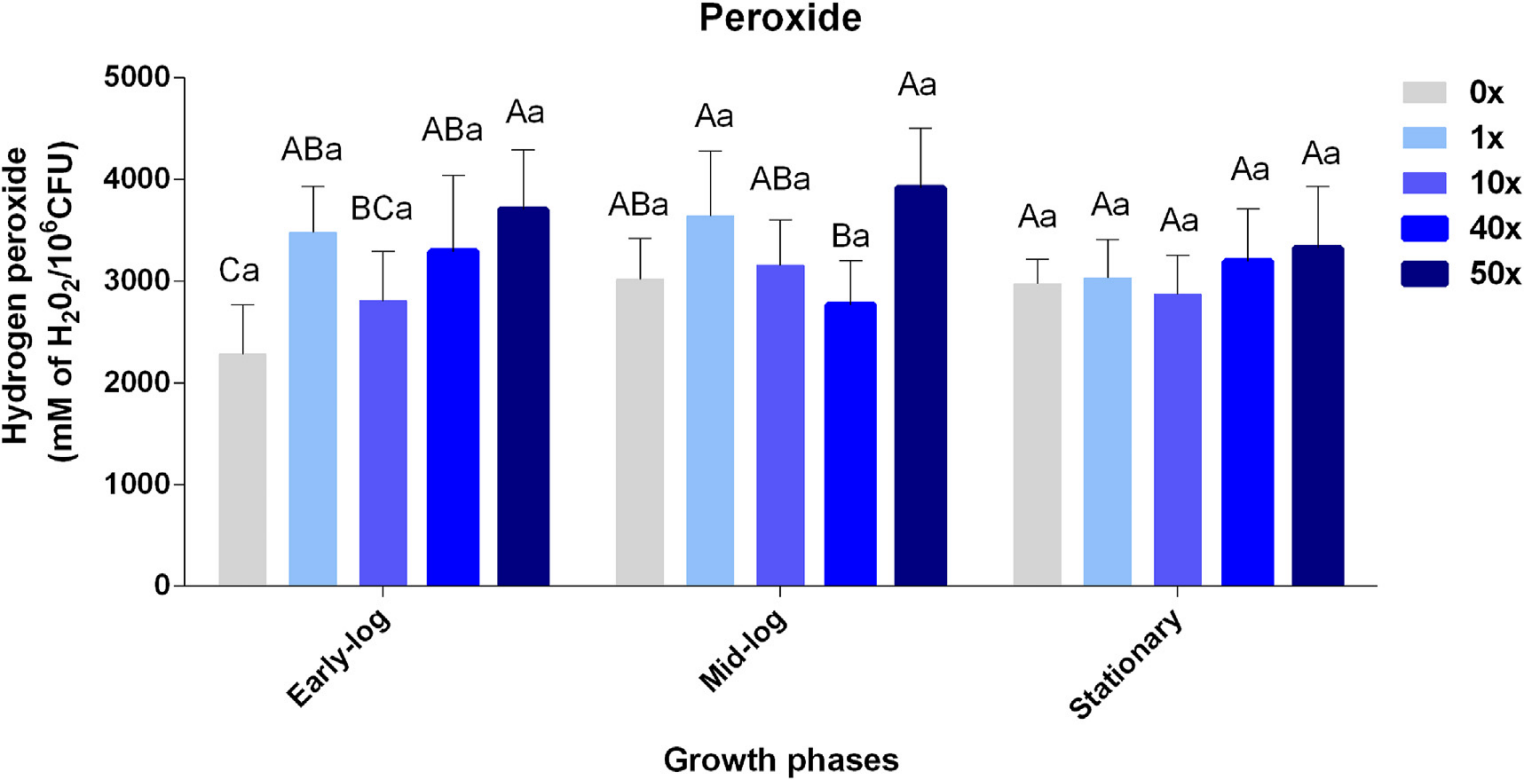
Quantification of H2O2 in *P. fluorescens* CMA-55 in the treatments with 0x, 1x, 10x, 40x, and 50x glyphosate concentrations, in the early-log, mid-log, and stationary growing phases. The data were obtained in triplicates for each treatment and analyzed statistically using the complete block design through analysis of variance (two-way ANOVA), followed by Tukey’s post hoc test. Error bars represent statistically significant differences between treatments at the same time. Capital letters represent statistically significant differences between treatments at different times. Significance was set at p < 0.05.

The results of quantification of H_2_O_2_ in *P. fluorescens* CMA-55 demonstrate different behaviors among the control and glyphosate treatments throughout the growth phases (Figure 3). In the early-log phase, significant increases in H_2_O_2_ production rates can be observed in glyphosate treatments (except for 10x), suggesting that herbicide induces increases in this stress indicator. In the mid-log and stationary phases, the production of H_2_O_2_ remained similar among the control and treatment groups. Generally, the H_2_O_2_ production data (Figure 3) was inversely proportional to the viability rates (Figure 2), as expected.

Similar data were found for *Pantoea ananatis* in treatments with the herbicide mesotrione, although no differences were found in growth rates among the control and treatment groups, with the data indicating the integration of enzymatic and structural responses (Prione et al., 2016).

#### 3.2.3 Quantification of MDA

**Figure 4:**
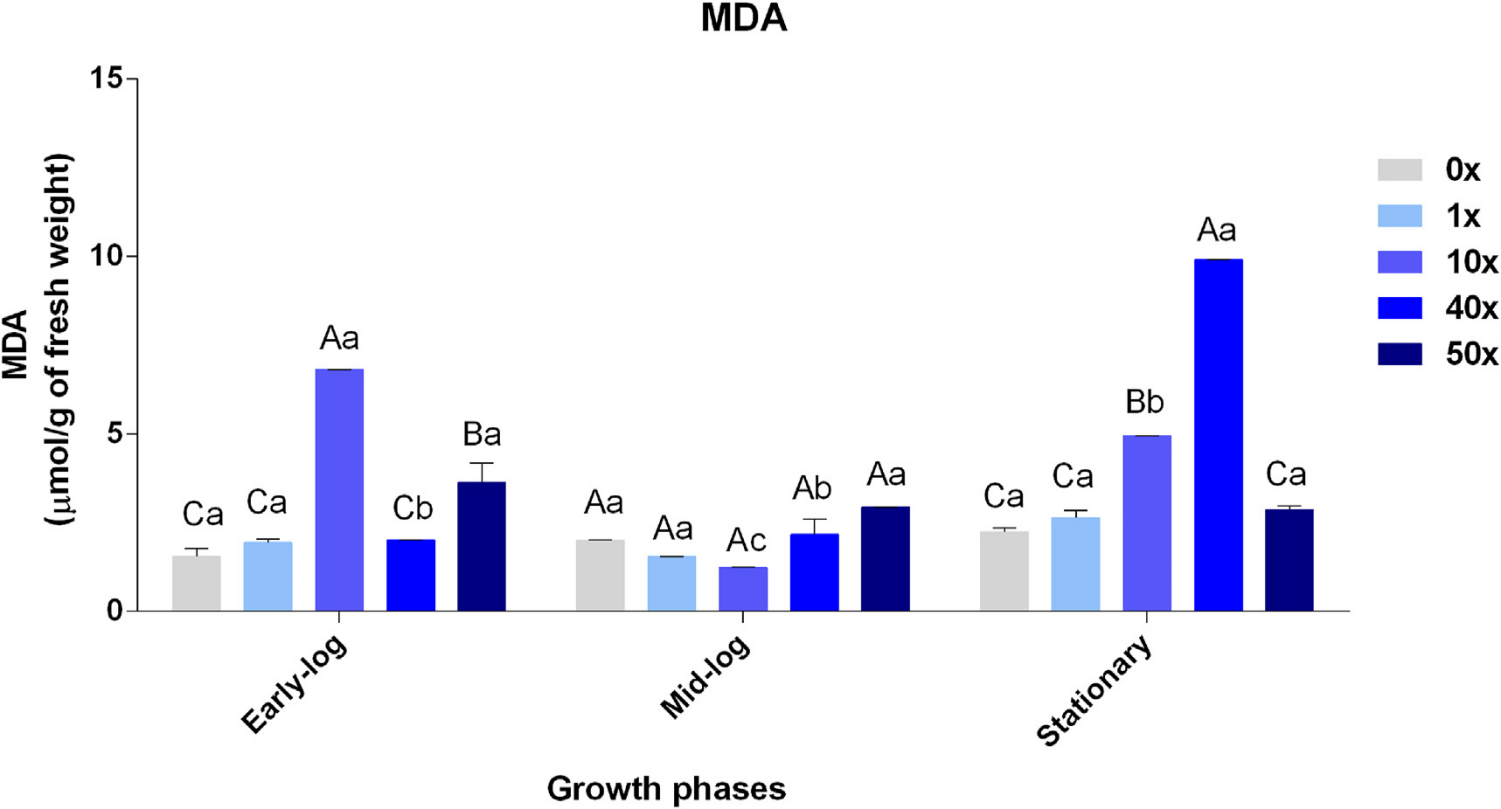
MDA quantification in *P. fluorescens* CMA-55 in the treatments with 0x, 1x, 10x, 40x, and 50x glyphosate concentrations, in the early-log, mid-log, and stationary growing phases. The data were obtained in triplicates for each treatment and analyzed statistically using the complete block design through analysis of variance (two-way ANOVA), followed by Tukey’s post hoc test. Error bars represent statistically significant differences between treatments at the same time. Capital letters represent statistically significant differences between treatments at different times. Significance was set at p < 0.05.

MDA is a dialdehyde formed as a secondary metabolite during the oxidation of polyunsaturated fatty acids and considered as an indicator of oxidative stress produced by herbicides in microorganisms (Du et al., 2017). Higher rates of ROS production are generally found in organisms that are in contact with toxic contaminants, and can cause damage to the membrane by reactions with these types of fatty acids, characterizing lipid peroxidation (Ni et al., 2018). Since there is no proportionality between the rates of MDA production (Figure 4) and those of H_2_O_2_ (Figure 3) in *P. fluorescens* CMA-55, and MDA is only produced when an ROS reacts with unsaturated lipids (Du et al., 2017), then probably occurred enzymatic changes in the fatty acid saturation pattern, resulting in changes in the permeability, as a way of responding to increasing glyphosate concentrations. The ability for enzymatic alterations in fatty acid profile has already been observed in the genus *Pseudomonas*, specifically in strain *Pseudomonas putida* KB3, which can change cell membrane saturation and permeability through the enzyme cyclopropane fatty acid synthase during phenol exposure (Nowak et al., 2021). Similar facts were observed in *Oenococcus oeni*, *a* bacterium with the capacity to adapt and withstand ethanol stresses through cell surface changes and membrane unsaturated/saturated fatty acid ratio (Bonomo et al., 2017).

Changes in the level of membrane fatty acid saturation, as cis-trans isomerization of unsaturated fatty acids and changes in phospholipid headgroups, were reported in the bacteria *Arthrobacter simplex* in response to elevated membrane fluidity caused by ethanol (Luo et al., 2018); and in *P. ananatis* (Prione et al., 2016) and *Bacillus megaterium* (Dobrzanski et al., 2018) to tolerate the herbicide Callisto.

### 3.3 Enzymatic responses to glyphosate in *Pseudomonas fluorescens*

#### 3.3.1 SOD activity

SOD is involved in antioxidative processes in aerobic or facultative organisms, catalyzing a dismutation of the superoxide radical (O_2_^–^) to H_2_O_2_ and O_2_, through different isoforms: Mn-SOD; Fe-SOD and Cu/Zn-SOD, have specific oxidation and reduction activities (Sheng et al., 2014).

Mn-SOD was the predominant isoform in *P. fluorescens* CMA-55, classified according to its sensitivity to H_2_O_2_ and KCN inhibitors (Supplementary Fig. A1). This isoform is located in the cytoplasm, where it responds to increased levels of the O_2_^-^ radical, and it is associated with the process of detoxifying herbicides in various organisms (Gravina et al., 2017).

**Figure 5:**
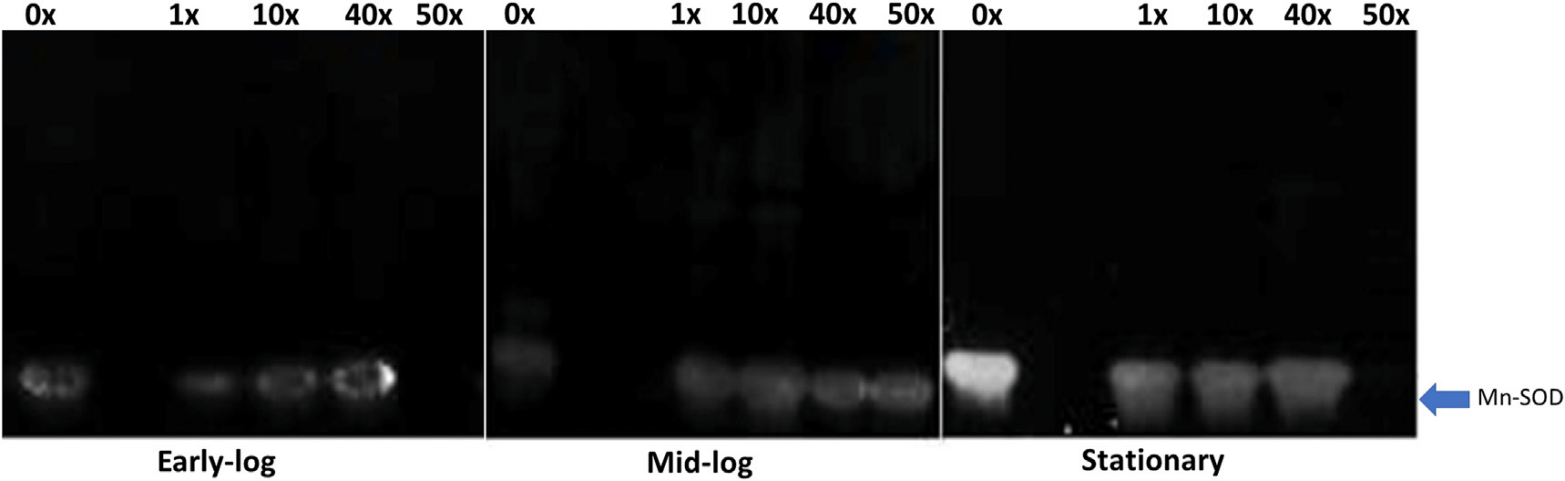
Characterization of SOD isoforms in PAGE, which were obtained from the extracts of *P. fluorescens* CMA-55, in PAGE, in the treatments with 0x, 1x, 10x, 40x, and 50x glyphosate concentrations, in the early-log, mid-log, and stationary growing phases. Non-adjusted images are shown in the Supplementary Material 4.

The activity of Mn-SOD was intense only in the control of the stationary phase, and in the 1x, 10x, and 40x treatments there is lower activity in all periods, with a slight increase in the stationary phase; the activity of this enzyme in the 50x treatment was only detected in the mid-log phase (Figure 5). At higher glyphosate concentrations, the Mn-SOD enzyme appears to be inhibited by the herbicide, limiting the efficiency in controlling ROS production (Figures 1 and 5). A similar situation was observed for the cyanobacterium *Microcystis* had SOD inhibited at higher concentrations of H_2_O_2_, but with CAT and reduced glutathione playing a more prominent role in oxidative stress control (Liu et al., 2017).

#### 3.3.2 CAT activity

CAT is an important antioxidant enzyme in cellular defense against oxidative stress, by converting the H_2_O_2_ molecule, produced by SOD, into H_2_O and O_2_. Its activity is increased with higher levels of H_2_O_2_ in cells (Imlay, 2013). Pezzoni et al. (2016) characterized three CAT isoforms in *Pseudomonas aeruginosa*: KatA, which are more active in all growth phases and more related to the control of high concentrations of H_2_O_2_; KatB, which is activated only in the presence of H_2_O_2_, but not fully involved in the control of oxidative stress; and KatC, which is unrelated to the control of oxidative stress.

**Figure 6:**
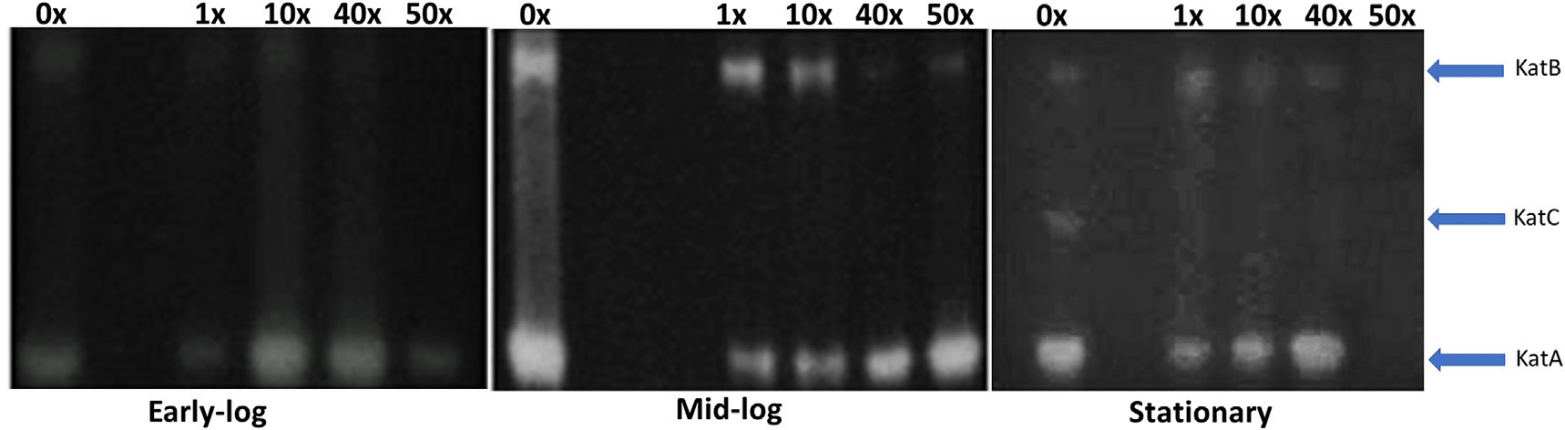
CAT gel activity in 0x, 1x, 10x, 40x, and 50x treatments, in the early-log and mid-log growing phases of *P. fluorescens* CMA-55. No band was observed at the 50x stationary growth phase. KatA, KatB, and additional band KatC isoforms were observed. Non-adjusted images are shown in the Supplementary Material 5.

The pattern of activity from CAT to *P. fluorescens* CMA-55 is shown in Figure 6. KatA presents an activity pattern in the largest number of treatments and with more intensity, KatB from mid-logs, and KatC only in the stationary phase. The pattern of variation in the activity of KatA and KatB, observed in Figure 6, is consistent with the production and need for H_2_O_2_ control in the different growth phases of *P. fluorescens* CMA-55 (Figure 3). In the stationary phase, there is an additional band, probably KatC (Figure 6). In *P. fluorescens* CMA-55, KatB is induced in response to glyphosate in the mid-log phase of growth, but it works in conjunction with KatA to control oxidative stress in the mid-log phase up to 10x the herbicide concentration (Figures 1 and 6). Notably that the three isoforms are inhibited by 50x in the Early-log and stationary phases, such as SOD. A generalized inhibition of the antioxidant enzymes was found in tomato plants, for instance, suggesting the occurrence of great redox disturbances by glyphosate (Soares et al., 2021).

Nevertheless, Olchanheski et al. (2014) observed that the activity of SOD and CAT in *E. coli* is fundamental for the maintenance of the mesotrione herbicide tolerance mechanism. Regarding *P. fluorescens* CMA-55, SOD (Figure 5) and CAT (Figure 6) acted in concert in the various growth phases, contributing to the control levels of H_2_O_2_ (Figure 3), MDA (Figure 4) and oxidative stress generated by glyphosate up to 10x treatment (Figure 1). These enzymes have been previously shown to act in sequence (Oliveira et al., 2021). Additionally, population density and quorum sensing, as N-dodecanoyl-homoserine lactone, which played important roles in the regulation of SOD and CAT enzymes in *Salmonella enteritidis* (Luiz de Freitas et al., 2021), and in response to herbicides, including the strain *P. fluorescens* CMA-55, studied in this work, in response to glyphosate (Freitas et al., 2021).

### 3.4 Fatty acid saturation and membrane permeability

Bacterial cell membranes are composed of lipids that participate in various physiological activities, as such as the well-studied transport and selectivity systems, and now the establishment of cellular organization, the central cellular scaffold that governs the cell’s inner architecture and guides its morphogenesis (Strahl and Errington, 2017), and antibiotic resistance (Salini and Pandian, 2015). However, the molecular interactions of lipopolysaccharides can be affected by environmental changes or toxic agents, such as herbicides, making membranes more permeable (Singh et al., 2017). In bacteria, the fatty acid composition can be enzymatically changed in response to temperature adaptation, through a DesK-mediated temperature sensing (Mendoza and Pilon, 2019).

In this study, data obtained using gas chromatography and mass spectrometry, we found saturation variations in different types of fatty acids in specific responses to the presence of glyphosate, to herbicide concentrations and to the growth phases (Table 1), suggesting a possible general strategy to sense and withstand the stress produced by glyphosate. The association of specific fatty acids was observed for 0x, 1x, and 10x glyphosate concentrations, such as the saturated nonadecylic acid (19.0), margaric acid (17.0) and lauric acid (12.0). We term this set of fatty acids associated with control and low concentrations of glyphosate Group II. Margaric acid was associated with a decrease in membrane permeability in *Pinus sylvestris* in response to aluminum contamination (Kalugina et al., 2021), whereas increases in the concentration of lauric acid were associated with decreased membrane integrity and permeability in *Bacillus subtilis* (Li et al., 2021).

**Table 1:** Correlations of the types of lipids identified by gas chromatography with glyphosate treatments (between []) in the three growing phases in *P. fluorescens* CMA-55. S = saturated lipid; U = unsaturated lipid; ∗ = 5% of statistical significance; ∗∗ = 1% of statistical significance; ∗∗∗ = 0.1% of statistical significance; inv. = statistical significance found for the concentrations located in the opposite quadrant in the PCA; x = lipid not found in the treatment.

Lipid type	Early-log significance	Mid-log significance	Stationary significance	Correlation group
9.0 S	[50x] ∗ inv.	[10x′40/50] ∗	x	
14.0 S	[40x] ∗∗∗	[0x] ∗	[50x] ∗	Group I
18.0 S	[40x] ∗∗∗	[0x] ∗∗∗	[10x] ∗∗	
10.0 S		[10x/40x/50x] ∗∗∗	[10x] ∗	
16.1w5c U	[50x] ∗∗ inv.	[40x] ∗	[50x] ∗∗	
12.0 S	[50x] ∗∗∗ inv.	∗∗∗	[0x/10x] ∗∗∗	
17.0 S	[50x] ∗∗ inv.	[1x] ∗∗	[10x] ∗∗∗	
19.0 S	[0x/1x] ∗∗∗	[0x] ∗∗∗	[10x] ∗∗∗	
10.0_3OH U	[50x] ∗∗∗ inv.	x	[0x/1x] ∗∗∗	
12.0_2OH U	[50x] ∗∗ inv.	x	[10x] ∗	Group II
17.0_iso U	[50x] ∗∗ inv.	[0x] ∗∗∗	[10x] ∗∗	
17.1w7c U	[0x/10x] ∗∗∗	[0x/1x/10x] ∗∗∗	[10x] ∗∗∗	
17.0cy U	[0x/1x] ∗∗	[1x] ∗∗∗	[1x] ∗∗∗	
18.1w7cM U	[50x] ∗∗ inv.	x	x	
18.2w6 U	[0x/10x] ∗∗∗	[0x] ∗∗∗	[10x] ∗∗∗	
19.0cy U	[0x/1x] ∗∗∗	[1x] ∗∗∗	[0x] ∗∗∗	
16.0 S	[50x] ∗∗∗	[40x] ∗	[40x] ∗	
16.1w7c U	[10x/40x] ∗∗∗	[10x/40x/50x] ∗∗∗	[40x] ∗∗∗	Group III
18.1w7c U	[10x/40x] ∗∗∗	[40x] ∗∗∗	[10x] ∗∗∗	

Group I fatty acids, were more associated with a transition of glyphosate concentrations, as examples the saturated fatty acids pelargonic acid (9.0), capric acid (10.0) myristic acid (14.0) and stearic acid (18.0), ranging from control to 50x. Of these fatty acids, steric acid is the one that presents some studies on the relationship of membrane permeability, saturation, and response to toxic substances in bacteria (Li et al., 2021). Group III, composed of unsaturated palmitoleic acid (16.1w.7c), and saturated palmitic acid (16.0), for example, was associated with 40x and 50x glyphosate concentrations. Palmitoleic acid was associated with changes in membrane permeability for erythromycin antibiotic in *Bacillus thuringiensis* (Zhou et al., 2018), and palmitic acid was associated with lauric acid in *Bacillus subtilis* (Li et al., 2021). Therefore, the strain *P. fluorescens* CMA-55 could modify the composition of its fatty acids and possibly the permeability of membranes in response to the differential toxicity of increasing glyphosate concentrations.

### 3.5 The response system analyzed for *Pseudomonas fluorescens* CMA-55

The ordering of the number of fatty acid types obtained from CG analysis, in the matrix correlations, indicated that some specific fatty acids were associated with stress situations in this study (Table 1). PCA showed that treatment groups were associated with growth phases, MDA and H_2_O_2_ in specific ways (Supplementary Material 3). The mid-log phase is characterized by the high activity of Mn-SOD (Figure 5) and KatA and KatB (Figure 6); and specific fatty acids (Group II, as discussed in the previous section) mainly produced at 0x, 1x, and 10x glyphosate concentrations. The specific set of fatty acids and the high activity of antioxidant enzymes led to a stress control condition, promoting higher rates of viability (Figure 2). The other phase with well-defined changes was the stationary phase, with low activity of Mn-SOD (Figure 5) and the three isoenzymes of CAT (Figure 6). The preponderant fatty acids belong to groups I and III and are linked to the highest concentrations of glyphosate at 40x and 50x, as demonstrated in the previous section. In this configuration, it appears that stress and permeability control were less efficient, resulting in lower viability rates.

Membrane homeostasis is a sense-and-response mechanism that monitors the membrane properties and adjusts its composition. These data indicated that bacteria adjust the properties of their membranes by regulating the biosynthesis of fatty acids and directly modifying membrane lipids. Neuberger et al. (2018) reported that changes in phospholipids cause changes and adaptations in bacteria when they are in sublethal processes in the presence of toxic compounds; this is a response that maintains the functionality of their membranes.

The sense-and-response seems to be involved with the antioxidant system of bacteria *P. fluorescens* CMA-55, in addition to changes in fatty acid saturation. For example, the proportionality between the amount of H_2_O_2_ and MDA in the control and 1x the glyphosate concentration occurs only in the stationary phase. There was no such correlation in other treatments and growth phases, in which the control of stress was more effective, suggesting that the bacteria were actively sensing different stress conditions as herbicide concentrations, modulating its fatty acid saturation pattern and antioxidative enzyme activities, possibly related to quorum sensing signaling molecules (Bollinger et al., 2001; Wang et al., 2020). There are reports of herbicides, as the herbicide Primextra Gold TZ (metolachlor and atrazine), having ecotoxicological and biochemical effects in various species in marine plankton, increasing the proportion of saturated fatty acids in response to toxicity (Filimonova et al., 2016).

The differential and integrated activities of SOD (Figure 5) and CAT (Figure 6), and the modulations of fatty acid types (Table 1) played important roles in the levels of glyphosate tolerance. Even through no prior selection by this herbicide in the environment where the *P. fluorescens* CMA-55 was isolated. These inherent mechanisms may include changes in fatty acid saturation of de novo synthetic processes via fatty acid synthetase enzyme and/or the action of desaturases enzymes that modify existing lipids (Mendoza and Pilon, 2019). Singh et al. (2017), stated that this phenomenon in pigmented bacteria, characterized by physiological plasticity, was responsible for the tolerance of temperature changes in the Arctic region. Our data suggest that specific fatty modifications represent a plasticity mechanism mediating changes in saturation and permeability to glyphosate in *P. fluorescens* CMA-55. This is representative of the adaptive potential of bacteria in environments subjected to intense variations of chemical substances, at toxic levels. Changes in the percentage of saturated fatty acids in biofilm bacterial cells, including *Pseudomonas aeruginosa*, were considered to adaptive stress responses and explain specific bacterial tolerance to biocides (Dubois-Brissonnet et al., 2016).

A question that can be raised from this study is related to the effect of physiological plasticity on a part of bacterial diversity to tolerate herbicides without selective pressure to degrade them, thus increasing the persistence of these xenobiotics in the environment. The persistence of these pesticides has been identified as a threat to non-target organisms that support important ecosystem functions (Thiour-Mauprivez et al., 2019). In this way, the signaling of specific tolerance and degradation responses of herbicides could be used to assemble more efficient bacterial consortia for bioremediation (Lima et al., 2020). Synthetic biology may provide tools to probe and manipulate quorum sensing behavior in natural bacterial communities; or to construct synthetic cocultures (Stephens and Bentley, 2020), or programming bacteria through quorum sensing controlled CRISPRi systems (Liu et al., 2020), to obtain desired behavior, as herbicide bioremediation.

## 4 Conclusions

*Pseudomonas fluorescens* CMA-55, isolated from an environment with high concentrations of various pesticides, but no glyphosate, presented two response system models, dependent on the concentration of this herbicide. One system was functional for low concentrations and mediated by the activities of Mn-SOD, KatA, and KatB, and specific fatty acids, as nonadecylic acid, margaric acid and lauric acid. The second, and not so efficient system, responded to high concentrations of glyphosate and had the appearance of an additional isoform, KatC, and pelargonic acid, capric acid, myristic acid, stearic acid, palmitoleic acid and palmitic acid as preponderant fatty acids. Therefore, the strain *P. fluorescens* CMA-55 could modulate the activity of different isoforms of antioxidant enzymes and the composition of fatty acids and membrane permeability in response to different glyphosate concentrations and toxicity levels, allowing the strain to grow under stressful conditions even at lower viability rates. Tolerance systems like these, based on physiological plasticity to non-selective xenobiotics, expand the potential for bacteria to survive in the presence of various toxic substances in the agricultural environment even without previous selective pressure, suggesting a possible general strategy to sense-and-response to the stress produced by xenobiotics. The challenge is to identify signaling mechanisms integrating these systems, possibly via quorum sensing, to manipulate bacterial populations by increasing the efficiency of bioremediation processes and by understanding their impact over microbiome diversity and functionality in agricultural soils.

## Declarations

### Author contribution statement

Rafael Mazer Etto; Péricles Martim Reche; Sônia Alvim Veiga Pileggi; Marcos Rogério Tótola; Marcos Pileggi: conceived and designed the experiments.

Elizangela Paz de Oliveira, Kathleen Evelyn Marchi Janaina Emiliano, Stella Marys Christóforo Hinojosa Salazar, Alisson Henrique Ferri, Karlos Henrique Martins Kalks: performed the experiments.

Elizangela Paz de Oliveira, Rafael Mazer Etto, Péricles Martim Reche, Sônia Alvim Veiga Pileggi, Marcos Rogério Tótola; Zelinda Schemczssen-Graeff, Marcos Pileggi: analyzed and interpreted the data.

Janaina Emiliano; Alisson Henrique Ferri, Rafael Mazer Etto, Péricles Martim Reche, Sônia Alvim Veiga Pileggi, Marcos Rogério Tótola, Zelinda Schemczssen-Graeff, Marcos Pileggi: contributed reagents, materials, analysis tools or data.

Elizangela Paz de Oliveira, Sônia Alvim Veiga Pileggi, Zelinda Schemczssen-Graeff, Marcos Pileggi: wrote the paper.

### Funding statement

This work was supported by the Coordination for the Improvement of Higher Level Personnel (10.13039/501100002322CAPES); and the National Council of Technological and Scientific Development (10.13039/501100003593CNPq).

### Data availability statement

Data associated with this study has been deposited at The sequence of 16S rRNA gene was deposited in the GenBank database (http://www.ncbi.nlm.nih.gov) under accession number KY807296.

### Declaration of interests statement

The authors declare no conflict of interest.

### Additional information

Supplementary content related to this article has been published online at https://doi.org/10.1016/j.heliyon.2022.e09938.
