# Supplementary material for: Changes in fatty acid composition as a response to glyphosate toxicity in Pseudomonas fluorescens
Source: Heliyon. 2022 Jul 13;8(8):e09938. doi: 10.1016/j.heliyon.2022.e09938 (PMC9364109; doi:10.1016/j.heliyon.2022.e09938)
Supplement: Multimedia component 1 [file mmc1.pdf]

Growth Curve

(2way ANOVA) Multiple Comparisons.

	2way ANOVA<br>Multiple comparisons	A<br>Data Set-A<br>Y	B<br>Data Set-B<br>Y	C<br>Data Set-C<br>Y
1	Compare cell means regardless of rows and columns			
2				
3	Number of families	1		
4	Number of comparisons per family	32385		
5	Alpha	0.05		
6				
7	Tukey's multiple comparisons test	Mean Diff.	95% CI of diff.	Significant?
8				
9	0:**0 X** vs. 0:**1 X**	0.0	-0.1440 to 0.1440	No
10	0:**0 X** vs. 0:**10 X**	0.0	-0.1440 to 0.1440	No
11	0:**0 X** vs. 0:**40 X**	0.0	-0.1440 to 0.1440	No
12	0:**0 X** vs. 0:**50 X**	0.0	-0.1440 to 0.1440	No
13	0:**0 X** vs. 1:**0 X**	0.04233	-0.1016 to 0.1863	No
14	0:**0 X** vs. 1:**1 X**	0.03467	-0.1093 to 0.1786	No
15	0:**0 X** vs. 1:**10 X**	0.0440	-0.09996 to 0.1880	No
16	0:**0 X** vs. 1:**40 X**	0.03833	-0.1056 to 0.1823	No
17	0:**0 X** vs. 1:**50 X**	0.0410	-0.1030 to 0.1850	No
18	0:**0 X** vs. 2:**0 X**	0.03767	-0.1063 to 0.1816	No
19	0:**0 X** vs. 2:**1 X**	0.03267	-0.1113 to 0.1766	No
20	0:**0 X** vs. 2:**10 X**	0.03867	-0.1053 to 0.1826	No
21	0:**0 X** vs. 2:**40 X**	0.0380	-0.1060 to 0.1820	No
22	0:**0 X** vs. 2:**50 X**	0.0410	-0.1030 to 0.1850	No
23	0:**0 X** vs. 3:**0 X**	0.03667	-0.1073 to 0.1806	No
24	0:**0 X** vs. 3:**1 X**	0.03433	-0.1096 to 0.1783	No
25	0:**0 X** vs. 3:**10 X**	0.0390	-0.1050 to 0.1830	No
26	0:**0 X** vs. 3:**40 X**	0.04133	-0.1026 to 0.1853	No
27	0:**0 X** vs. 3:**50 X**	0.04233	-0.1016 to 0.1863	No
28	0:**0 X** vs. 4:**0 X**	0.03233	-0.1116 to 0.1763	No
29	0:**0 X** vs. 4:**1 X**	0.03367	-0.1103 to 0.1776	No
30	0:**0 X** vs. 4:**10 X**	0.0190	-0.1250 to 0.1630	No
31	0:**0 X** vs. 4:**40 X**	0.03933	-0.1046 to 0.1833	No
32	0:**0 X** vs. 4:**50 X**	0.04133	-0.1026 to 0.1853	No
33	0:**0 X** vs. 5:**0 X**	0.03133	-0.1126 to 0.1753	No
34	0:**0 X** vs. 5:**1 X**	0.03333	-0.1106 to 0.1773	No
35	0:**0 X** vs. 5:**10 X**	0.01833	-0.1256 to 0.1623	No
36	0:**0 X** vs. 5:**40 X**	0.03933	-0.1046 to 0.1833	No
37	0:**0 X** vs. 5:**50 X**	0.04033	-0.1036 to 0.1843	No
38	0:**0 X** vs. 6:**0 X**	0.0320	-0.1120 to 0.1760	No
39	0:**0 X** vs. 6:**1 X**	0.0330	-0.1110 to 0.1770	No
40	0:**0 X** vs. 6:**10 X**	0.01833	-0.1256 to 0.1623	No
41	0:**0 X** vs. 6:**40 X**	0.0370	-0.1070 to 0.1810	No
42	0:**0 X** vs. 6:**50 X**	0.03867	-0.1053 to 0.1826	No
43	0:**0 X** vs. 7:**0 X**	0.0300	-0.1140 to 0.1740	No
44	0:**0 X** vs. 7:**1 X**	0.03867	-0.1053 to 0.1826	No
45	0:**0 X** vs. 7:**10 X**	0.02667	-0.1173 to 0.1706	No

	2way ANOVA Multiple comparisons	A	B	C
		Data Set-A	Data Set-B	Data Set-C
		Y	Y	Y
46	0:0 X vs. 7:40 X	0.03533	-0.1086 to 0.1793	No
47	0:0 X vs. 7:50 X	0.03733	-0.1066 to 0.1813	No
48	0:0 X vs. 8:0 X	0.02533	-0.1186 to 0.1693	No
49	0:0 X vs. 8:1 X	0.03867	-0.1053 to 0.1826	No
50	0:0 X vs. 8:10 X	0.03133	-0.1126 to 0.1753	No
51	0:0 X vs. 8:40 X	0.03067	-0.1133 to 0.1746	No
52	0:0 X vs. 8:50 X	0.03367	-0.1103 to 0.1776	No
53	0:0 X vs. 9:0 X	0.01733	-0.1266 to 0.1613	No
54	0:0 X vs. 9:1 X	0.009333	-0.1346 to 0.1533	No
55	0:0 X vs. 9:10 X	0.008333	-0.1356 to 0.1523	No
56	0:0 X vs. 9:40 X	0.0260	-0.1180 to 0.1700	No
57	0:0 X vs. 9:50 X	0.03267	-0.1113 to 0.1766	No
58	0:0 X vs. 10:0 X	0.01567	-0.1283 to 0.1596	No
59	0:0 X vs. 10:1 X	0.008667	-0.1353 to 0.1526	No
60	0:0 X vs. 10:10 X	0.0060	-0.1380 to 0.1500	No
61	0:0 X vs. 10:40 X	0.01633	-0.1276 to 0.1603	No
62	0:0 X vs. 10:50 X	0.02967	-0.1143 to 0.1736	No
63	0:0 X vs. 11:0 X	0.0060	-0.1380 to 0.1500	No
64	0:0 X vs. 11:1 X	0.004333	-0.1396 to 0.1483	No
65	0:0 X vs. 11:10 X	0.004000	-0.1400 to 0.1480	No
66	0:0 X vs. 11:40 X	0.01233	-0.1316 to 0.1563	No
67	0:0 X vs. 11:50 X	0.02433	-0.1196 to 0.1683	No
68	0:0 X vs. 12:0 X	0.005333	-0.1386 to 0.1493	No
69	0:0 X vs. 12:1 X	0.0230	-0.1210 to 0.1670	No
70	0:0 X vs. 12:10 X	0.005333	-0.1386 to 0.1493	No
71	0:0 X vs. 12:40 X	0.0190	-0.1250 to 0.1630	No
72	0:0 X vs. 12:50 X	0.01567	-0.1283 to 0.1596	No
73	0:0 X vs. 13:0 X	0.01133	-0.1326 to 0.1553	No
74	0:0 X vs. 13:1 X	0.0240	-0.1200 to 0.1680	No
75	0:0 X vs. 13:10 X	0.0020	-0.1420 to 0.1460	No
76	0:0 X vs. 13:40 X	0.005667	-0.1383 to 0.1496	No
77	0:0 X vs. 13:50 X	0.004667	-0.1393 to 0.1486	No
78	0:0 X vs. 14:0 X	0.002667	-0.1413 to 0.1466	No
79	0:0 X vs. 14:1 X	0.0110	-0.1330 to 0.1550	No
80	0:0 X vs. 14:10 X	-0.0030	-0.1470 to 0.1410	No
81	0:0 X vs. 14:40 X	0.0003333	-0.1436 to 0.1443	No
82	0:0 X vs. 14:50 X	-0.003667	-0.1476 to 0.1403	No
83	0:0 X vs. 15:0 X	-0.009667	-0.1536 to 0.1343	No
84	0:0 X vs. 15:1 X	0.01033	-0.1336 to 0.1543	No
85	0:0 X vs. 15:10 X	-0.0170	-0.1610 to 0.1270	No
86	0:0 X vs. 15:40 X	0.0006667	-0.1433 to 0.1446	No
87	0:0 X vs. 15:50 X	-0.0170	-0.1610 to 0.1270	No
88	0:0 X vs. 16:0 X	-0.1020	-0.2460 to 0.04196	No
89	0:0 X vs. 16:1 X	0.007000	-0.1370 to 0.1510	No
90	0:0 X vs. 16:10 X	-0.01867	-0.1626 to 0.1253	No

	**2way ANOVA**<br>Multiple comparisons	**A**<br>Data Set-A<br>**Y**	**B**<br>Data Set-B<br>**Y**	**C**<br>Data Set-C<br>**Y**
**91**	0:**0 X** vs. 16:**40 X**	-0.0380	-0.1820 to 0.1060	No
**92**	0:**0 X** vs. 16:**50 X**	-0.08033	-0.2243 to 0.06363	No
**93**	0:**0 X** vs. 17:**0 X**	-0.1547	-0.2986 to -0.01071	Yes
**94**	0:**0 X** vs. 17:**1 X**	-0.06900	-0.2130 to 0.07496	No
**95**	0:**0 X** vs. 17:**10 X**	-0.1257	-0.2696 to 0.01829	No
**96**	0:**0 X** vs. 17:**40 X**	-0.0490	-0.1930 to 0.09496	No
**97**	0:**0 X** vs. 17:**50 X**	-0.09433	-0.2383 to 0.04963	No
**98**	0:**0 X** vs. 18:**0 X**	-0.2033	-0.3473 to -0.05937	Yes
**99**	0:**0 X** vs. 18:**1 X**	-0.1117	-0.2556 to 0.03229	No
**100**	0:**0 X** vs. 18:**10 X**	-0.2040	-0.3480 to -0.06004	Yes
**101**	0:**0 X** vs. 18:**40 X**	-0.05333	-0.1973 to 0.09063	No
**102**	0:**0 X** vs. 18:**50 X**	-0.09833	-0.2423 to 0.04563	No
**103**	0:**0 X** vs. 19:**0 X**	-0.2383	-0.3823 to -0.09437	Yes
**104**	0:**0 X** vs. 19:**1 X**	-0.2263	-0.3703 to -0.08237	Yes
**105**	0:**0 X** vs. 19:**10 X**	-0.2240	-0.3680 to -0.08004	Yes
**106**	0:**0 X** vs. 19:**40 X**	-0.0820	-0.2260 to 0.06196	No
**107**	0:**0 X** vs. 19:**50 X**	-0.0930	-0.2370 to 0.05096	No
**108**	0:**0 X** vs. 20:**0 X**	-0.3180	-0.4620 to -0.1740	Yes
**109**	0:**0 X** vs. 20:**1 X**	-0.1373	-0.2813 to 0.006626	No
**110**	0:**0 X** vs. 20:**10 X**	-0.2263	-0.3703 to -0.08237	Yes
**111**	0:**0 X** vs. 20:**40 X**	-0.09367	-0.2376 to 0.05029	No
**112**	0:**0 X** vs. 20:**50 X**	-0.1377	-0.2816 to 0.006293	No
**113**	0:**0 X** vs. 21:**0 X**	-0.3413	-0.4853 to -0.1974	Yes
**114**	0:**0 X** vs. 21:**1 X**	-0.2943	-0.4383 to -0.1504	Yes
**115**	0:**0 X** vs. 21:**10 X**	-0.2913	-0.4353 to -0.1474	Yes
**116**	0:**0 X** vs. 21:**40 X**	-0.1667	-0.3106 to -0.02271	Yes
**117**	0:**0 X** vs. 21:**50 X**	-0.2570	-0.4010 to -0.1130	Yes
**118**	0:**0 X** vs. 22:**0 X**	-0.3830	-0.5270 to -0.2390	Yes
**119**	0:**0 X** vs. 22:**1 X**	-0.3777	-0.5216 to -0.2337	Yes
**120**	0:**0 X** vs. 22:**10 X**	-0.3730	-0.5170 to -0.2290	Yes
**121**	0:**0 X** vs. 22:**40 X**	-0.2733	-0.4173 to -0.1294	Yes
**122**	0:**0 X** vs. 22:**50 X**	-0.3360	-0.4800 to -0.1920	Yes
**123**	0:**0 X** vs. 23:**0 X**	-0.4483	-0.5923 to -0.3044	Yes
**124**	0:**0 X** vs. 23:**1 X**	-0.6380	-0.7990 to -0.4770	Yes
**125**	0:**0 X** vs. 23:**10 X**	-0.3877	-0.5316 to -0.2437	Yes
**126**	0:**0 X** vs. 23:**40 X**	-0.2680	-0.4290 to -0.1070	Yes
**127**	0:**0 X** vs. 23:**50 X**	-0.3020	-0.4460 to -0.1580	Yes
**128**	0:**0 X** vs. 24:**0 X**	-0.5657	-0.7096 to -0.4217	Yes
**129**	0:**0 X** vs. 24:**1 X**	-0.7220	-0.8830 to -0.5610	Yes
**130**	0:**0 X** vs. 24:**10 X**	-0.5495	-0.7105 to -0.3885	Yes
**131**	0:**0 X** vs. 24:**40 X**	-0.2843	-0.4283 to -0.1404	Yes
**132**	0:**0 X** vs. 24:**50 X**	-0.2060	-0.3500 to -0.06204	Yes
**133**	0:**0 X** vs. 25:**0 X**	-0.9030	-1.064 to -0.7420	Yes
**134**	0:**0 X** vs. 25:**1 X**	-1.093	-1.253 to -0.9315	Yes
**135**	0:**0 X** vs. 25:**10 X**	-0.4540	-0.6576 to -0.2504	Yes

	2way ANOVA<br>Multiple comparisons	A<br>Data Set-A<br>Y	B<br>Data Set-B<br>Y	C<br>Data Set-C<br>Y
136	0:**0 X** vs. 25:**40 X**	-0.2863	-0.4303 to -0.1424	Yes
137	0:**0 X** vs. 25:**50 X**	-0.2607	-0.4046 to -0.1167	Yes
138	0:**0 X** vs. 26:**0 X**	-1.116	-1.276 to -0.9545	Yes
139	0:**0 X** vs. 26:**1 X**	-1.229	-1.389 to -1.068	Yes
140	0:**0 X** vs. 26:**10 X**	-0.9150	-1.076 to -0.7540	Yes
141	0:**0 X** vs. 26:**40 X**	-0.3607	-0.5046 to -0.2167	Yes
142	0:**0 X** vs. 26:**50 X**	-0.2210	-0.3650 to -0.07704	Yes
143	0:**0 X** vs. 27:**0 X**	-1.303	-1.447 to -1.159	Yes
144	0:**0 X** vs. 27:**1 X**	-1.394	-1.555 to -1.233	Yes
145	0:**0 X** vs. 27:**10 X**	-1.206	-1.367 to -1.045	Yes
146	0:**0 X** vs. 27:**40 X**	-0.3607	-0.5046 to -0.2167	Yes
147	0:**0 X** vs. 27:**50 X**	-0.2203	-0.3643 to -0.07637	Yes
148	0:**0 X** vs. 28:**0 X**	-1.253	-1.413 to -1.092	Yes
149	0:**0 X** vs. 28:**1 X**	-1.293	-1.437 to -1.149	Yes
150	0:**0 X** vs. 28:**10 X**	-1.095	-1.256 to -0.9340	Yes
151	0:**0 X** vs. 28:**40 X**	-0.3730	-0.5170 to -0.2290	Yes
152	0:**0 X** vs. 28:**50 X**	-0.2683	-0.4123 to -0.1244	Yes
153	0:**0 X** vs. 29:**0 X**	-1.734	-1.895 to -1.573	Yes
154	0:**0 X** vs. 29:**1 X**	-1.819	-1.980 to -1.658	Yes
155	0:**0 X** vs. 29:**10 X**	-1.034	-1.194 to -0.8725	Yes
156	0:**0 X** vs. 29:**40 X**	-0.4810	-0.6250 to -0.3370	Yes
157	0:**0 X** vs. 29:**50 X**	-0.2697	-0.4136 to -0.1257	Yes
158	0:**0 X** vs. 30:**0 X**	-1.449	-1.609 to -1.288	Yes
159	0:**0 X** vs. 30:**1 X**	-1.770	-1.931 to -1.609	Yes
160	0:**0 X** vs. 30:**10 X**	-1.108	-1.269 to -0.9470	Yes
161	0:**0 X** vs. 30:**40 X**	-0.4080	-0.5520 to -0.2640	Yes
162	0:**0 X** vs. 30:**50 X**	-0.3220	-0.4660 to -0.1780	Yes
163	0:**0 X** vs. 31:**0 X**	-1.767	-1.927 to -1.606	Yes
164	0:**0 X** vs. 31:**1 X**	-1.757	-1.918 to -1.596	Yes
165	0:**0 X** vs. 31:**10 X**	-1.240	-1.400 to -1.079	Yes
166	0:**0 X** vs. 31:**40 X**	-0.4997	-0.6436 to -0.3557	Yes
167	0:**0 X** vs. 31:**50 X**	-0.3010	-0.4450 to -0.1570	Yes
168	0:**0 X** vs. 32:**0 X**	-2.358	-2.502 to -2.214	Yes
169	0:**0 X** vs. 32:**1 X**	-1.814	-1.975 to -1.653	Yes
170	0:**0 X** vs. 32:**10 X**	-1.670	-1.814 to -1.526	Yes
171	0:**0 X** vs. 32:**40 X**	-0.4960	-0.6400 to -0.3520	Yes
172	0:**0 X** vs. 32:**50 X**	-0.3007	-0.4446 to -0.1567	Yes
173	0:**0 X** vs. 33:**0 X**	-2.362	-2.506 to -2.218	Yes
174	0:**0 X** vs. 33:**1 X**	-2.176	-2.320 to -2.032	Yes
175	0:**0 X** vs. 33:**10 X**	-1.837	-1.981 to -1.693	Yes
176	0:**0 X** vs. 33:**40 X**	-0.5147	-0.6586 to -0.3707	Yes
177	0:**0 X** vs. 33:**50 X**	-0.3157	-0.4596 to -0.1717	Yes
178	0:**0 X** vs. 34:**0 X**	-2.286	-2.430 to -2.142	Yes
179	0:**0 X** vs. 34:**1 X**	-2.294	-2.438 to -2.150	Yes
180	0:**0 X** vs. 34:**10 X**	-1.927	-2.071 to -1.783	Yes

	2way ANOVA<br>Multiple comparisons	A<br>Data Set-A<br>Y	B<br>Data Set-B<br>Y	C<br>Data Set-C<br>Y
181	0:0 X vs. 34:40 X	-0.5267	-0.6706 to -0.3827	Yes
182	0:0 X vs. 34:50 X	-0.4647	-0.6086 to -0.3207	Yes
183	0:0 X vs. 35:0 X	-2.635	-2.779 to -2.491	Yes
184	0:0 X vs. 35:1 X	-2.401	-2.545 to -2.257	Yes
185	0:0 X vs. 35:10 X	-1.889	-2.033 to -1.745	Yes
186	0:0 X vs. 35:40 X	-0.5357	-0.6796 to -0.3917	Yes
187	0:0 X vs. 35:50 X	-0.4773	-0.6213 to -0.3334	Yes
188	0:0 X vs. 36:0 X	-2.842	-3.002 to -2.681	Yes
189	0:0 X vs. 36:1 X	-2.537	-2.681 to -2.393	Yes
190	0:0 X vs. 36:10 X	-1.894	-2.038 to -1.750	Yes
191	0:0 X vs. 36:40 X	-0.5910	-0.7350 to -0.4470	Yes
192	0:0 X vs. 36:50 X	-0.4407	-0.5846 to -0.2967	Yes
193	0:0 X vs. 37:0 X	-3.225	-3.369 to -3.081	Yes
194	0:0 X vs. 37:1 X	-2.607	-2.751 to -2.463	Yes
195	0:0 X vs. 37:10 X	-1.896	-2.040 to -1.752	Yes
196	0:0 X vs. 37:40 X	-0.6283	-0.7723 to -0.4844	Yes
197	0:0 X vs. 37:50 X	-0.4493	-0.5933 to -0.3054	Yes
198	0:0 X vs. 38:0 X	-3.170	-3.314 to -3.026	Yes
199	0:0 X vs. 38:1 X	-2.726	-2.870 to -2.582	Yes
200	0:0 X vs. 38:10 X	-2.171	-2.315 to -2.027	Yes
201	0:0 X vs. 38:40 X	-0.6327	-0.7766 to -0.4887	Yes
202	0:0 X vs. 38:50 X	-0.4533	-0.5973 to -0.3094	Yes
203	0:0 X vs. 39:0 X	-3.401	-3.545 to -3.257	Yes
204	0:0 X vs. 39:1 X	-3.293	-3.437 to -3.149	Yes
205	0:0 X vs. 39:10 X	-2.316	-2.460 to -2.172	Yes
206	0:0 X vs. 39:40 X	-0.6387	-0.7826 to -0.4947	Yes
207	0:0 X vs. 39:50 X	-0.4843	-0.6283 to -0.3404	Yes
208	0:0 X vs. 40:0 X	-3.442	-3.586 to -3.298	Yes
209	0:0 X vs. 40:1 X	-3.368	-3.512 to -3.224	Yes
210	0:0 X vs. 40:10 X	-2.573	-2.717 to -2.429	Yes
211	0:0 X vs. 40:40 X	-0.6433	-0.7873 to -0.4994	Yes
212	0:0 X vs. 40:50 X	-0.4907	-0.6346 to -0.3467	Yes
213	0:0 X vs. 41:0 X	-3.483	-3.627 to -3.339	Yes
214	0:0 X vs. 41:1 X	-3.482	-3.626 to -3.338	Yes
215	0:0 X vs. 41:10 X	-2.626	-2.770 to -2.482	Yes
216	0:0 X vs. 41:40 X	-0.5907	-0.7346 to -0.4467	Yes
217	0:0 X vs. 41:50 X	-0.5033	-0.6473 to -0.3594	Yes
218	0:0 X vs. 42:0 X	-3.625	-3.769 to -3.481	Yes
219	0:0 X vs. 42:1 X	-3.644	-3.788 to -3.500	Yes
220	0:0 X vs. 42:10 X	-2.627	-2.771 to -2.483	Yes
221	0:0 X vs. 42:40 X	-0.6033	-0.7473 to -0.4594	Yes
222	0:0 X vs. 42:50 X	-0.5067	-0.6506 to -0.3627	Yes
223	0:0 X vs. 43:0 X	-3.715	-3.859 to -3.571	Yes
224	0:0 X vs. 43:1 X	-3.629	-3.773 to -3.485	Yes
225	0:0 X vs. 43:10 X	-3.345	-3.489 to -3.201	Yes

	2way ANOVA<br>Multiple comparisons	A<br>Data Set-A<br>Y	B<br>Data Set-B<br>Y	C<br>Data Set-C<br>Y
226	0:**0 X** vs. 43:**40 X**	-0.6017	-0.7456 to -0.4577	Yes
227	0:**0 X** vs. 43:**50 X**	-0.5070	-0.6510 to -0.3630	Yes
228	0:**0 X** vs. 44:**0 X**	-3.739	-3.883 to -3.595	Yes
229	0:**0 X** vs. 44:**1 X**	-3.628	-3.772 to -3.484	Yes
230	0:**0 X** vs. 44:**10 X**	-3.408	-3.552 to -3.264	Yes
231	0:**0 X** vs. 44:**40 X**	-0.6037	-0.7476 to -0.4597	Yes
232	0:**0 X** vs. 44:**50 X**	-0.4393	-0.5833 to -0.2954	Yes
233	0:**0 X** vs. 45:**0 X**	-3.603	-3.747 to -3.459	Yes
234	0:**0 X** vs. 45:**1 X**	-3.405	-3.549 to -3.261	Yes
235	0:**0 X** vs. 45:**10 X**	-3.288	-3.432 to -3.144	Yes
236	0:**0 X** vs. 45:**40 X**	-0.6050	-0.7490 to -0.4610	Yes
237	0:**0 X** vs. 45:**50 X**	-0.4363	-0.5803 to -0.2924	Yes
238	0:**0 X** vs. 46:**0 X**	-3.408	-3.552 to -3.264	Yes
239	0:**0 X** vs. 46:**1 X**	-3.375	-3.519 to -3.231	Yes
240	0:**0 X** vs. 46:**10 X**	-3.283	-3.427 to -3.139	Yes
241	0:**0 X** vs. 46:**40 X**	-0.6187	-0.7626 to -0.4747	Yes
242	0:**0 X** vs. 46:**50 X**	-0.4293	-0.5733 to -0.2854	Yes
243	0:**0 X** vs. 47:**0 X**	-3.383	-3.527 to -3.239	Yes
244	0:**0 X** vs. 47:**1 X**	-3.287	-3.431 to -3.143	Yes
245	0:**0 X** vs. 47:**10 X**	-3.269	-3.413 to -3.125	Yes
246	0:**0 X** vs. 47:**40 X**	-0.6287	-0.7726 to -0.4847	Yes
247	0:**0 X** vs. 47:**50 X**	-0.3873	-0.5313 to -0.2434	Yes
248	0:**0 X** vs. 48:**0 X**	-3.371	-3.531 to -3.210	Yes
249	0:**0 X** vs. 48:**1 X**	-3.274	-3.418 to -3.130	Yes
250	0:**0 X** vs. 48:**10 X**	-3.269	-3.413 to -3.125	Yes
251	0:**0 X** vs. 48:**40 X**	-0.6350	-0.7790 to -0.4910	Yes
252	0:**0 X** vs. 48:**50 X**	-0.3947	-0.5386 to -0.2507	Yes
253	0:**0 X** vs. 49:**0 X**	-3.371	-3.515 to -3.227	Yes
254	0:**0 X** vs. 49:**1 X**	-3.274	-3.418 to -3.130	Yes
255	0:**0 X** vs. 49:**10 X**	-3.269	-3.413 to -3.125	Yes
256	0:**0 X** vs. 49:**40 X**	-0.6393	-0.7833 to -0.4954	Yes
257	0:**0 X** vs. 49:**50 X**	-0.3940	-0.5380 to -0.2500	Yes
258	0:**0 X** vs. 50:**0 X**	-3.371	-3.515 to -3.227	Yes
259	0:**0 X** vs. 50:**1 X**	-3.274	-3.418 to -3.130	Yes
260	0:**0 X** vs. 50:**10 X**	-3.269	-3.413 to -3.125	Yes
261	0:**0 X** vs. 50:**40 X**	-0.6470	-0.7910 to -0.5030	Yes
262	0:**0 X** vs. 50:**50 X**	-0.3937	-0.5376 to -0.2497	Yes
263	0:**1 X** vs. 0:**10 X**	0.0	-0.1440 to 0.1440	No
264	0:**1 X** vs. 0:**40 X**	0.0	-0.1440 to 0.1440	No
265	0:**1 X** vs. 0:**50 X**	0.0	-0.1440 to 0.1440	No
266	0:**1 X** vs. 1:**0 X**	0.04233	-0.1016 to 0.1863	No
267	0:**1 X** vs. 1:**1 X**	0.03467	-0.1093 to 0.1786	No
268	0:**1 X** vs. 1:**10 X**	0.0440	-0.09996 to 0.1880	No
269	0:**1 X** vs. 1:**40 X**	0.03833	-0.1056 to 0.1823	No
270	0:**1 X** vs. 1:**50 X**	0.0410	-0.1030 to 0.1850	No

	2way ANOVA<br>Multiple comparisons	A<br>Data Set-A<br>Y	B<br>Data Set-B<br>Y	C<br>Data Set-C<br>Y
**271**	0:**1 X** vs. 2:**0 X**	0.03767	-0.1063 to 0.1816	No
**272**	0:**1 X** vs. 2:**1 X**	0.03267	-0.1113 to 0.1766	No
**273**	0:**1 X** vs. 2:**10 X**	0.03867	-0.1053 to 0.1826	No
**274**	0:**1 X** vs. 2:**40 X**	0.0380	-0.1060 to 0.1820	No
**275**	0:**1 X** vs. 2:**50 X**	0.0410	-0.1030 to 0.1850	No
**276**	0:**1 X** vs. 3:**0 X**	0.03667	-0.1073 to 0.1806	No
**277**	0:**1 X** vs. 3:**1 X**	0.03433	-0.1096 to 0.1783	No
**278**	0:**1 X** vs. 3:**10 X**	0.0390	-0.1050 to 0.1830	No
**279**	0:**1 X** vs. 3:**40 X**	0.04133	-0.1026 to 0.1853	No
**280**	0:**1 X** vs. 3:**50 X**	0.04233	-0.1016 to 0.1863	No
**281**	0:**1 X** vs. 4:**0 X**	0.03233	-0.1116 to 0.1763	No
**282**	0:**1 X** vs. 4:**1 X**	0.03367	-0.1103 to 0.1776	No
**283**	0:**1 X** vs. 4:**10 X**	0.0190	-0.1250 to 0.1630	No
**284**	0:**1 X** vs. 4:**40 X**	0.03933	-0.1046 to 0.1833	No
**285**	0:**1 X** vs. 4:**50 X**	0.04133	-0.1026 to 0.1853	No
**286**	0:**1 X** vs. 5:**0 X**	0.03133	-0.1126 to 0.1753	No
**287**	0:**1 X** vs. 5:**1 X**	0.03333	-0.1106 to 0.1773	No
**288**	0:**1 X** vs. 5:**10 X**	0.01833	-0.1256 to 0.1623	No
**289**	0:**1 X** vs. 5:**40 X**	0.03933	-0.1046 to 0.1833	No
**290**	0:**1 X** vs. 5:**50 X**	0.04033	-0.1036 to 0.1843	No
**291**	0:**1 X** vs. 6:**0 X**	0.0320	-0.1120 to 0.1760	No
**292**	0:**1 X** vs. 6:**1 X**	0.0330	-0.1110 to 0.1770	No
**293**	0:**1 X** vs. 6:**10 X**	0.01833	-0.1256 to 0.1623	No
**294**	0:**1 X** vs. 6:**40 X**	0.0370	-0.1070 to 0.1810	No
**295**	0:**1 X** vs. 6:**50 X**	0.03867	-0.1053 to 0.1826	No
**296**	0:**1 X** vs. 7:**0 X**	0.0300	-0.1140 to 0.1740	No
**297**	0:**1 X** vs. 7:**1 X**	0.03867	-0.1053 to 0.1826	No
**298**	0:**1 X** vs. 7:**10 X**	0.02667	-0.1173 to 0.1706	No
**299**	0:**1 X** vs. 7:**40 X**	0.03533	-0.1086 to 0.1793	No
**300**	0:**1 X** vs. 7:**50 X**	0.03733	-0.1066 to 0.1813	No
**301**	0:**1 X** vs. 8:**0 X**	0.02533	-0.1186 to 0.1693	No
**302**	0:**1 X** vs. 8:**1 X**	0.03867	-0.1053 to 0.1826	No
**303**	0:**1 X** vs. 8:**10 X**	0.03133	-0.1126 to 0.1753	No
**304**	0:**1 X** vs. 8:**40 X**	0.03067	-0.1133 to 0.1746	No
**305**	0:**1 X** vs. 8:**50 X**	0.03367	-0.1103 to 0.1776	No
**306**	0:**1 X** vs. 9:**0 X**	0.01733	-0.1266 to 0.1613	No
**307**	0:**1 X** vs. 9:**1 X**	0.009333	-0.1346 to 0.1533	No
**308**	0:**1 X** vs. 9:**10 X**	0.008333	-0.1356 to 0.1523	No
**309**	0:**1 X** vs. 9:**40 X**	0.0260	-0.1180 to 0.1700	No
**310**	0:**1 X** vs. 9:**50 X**	0.03267	-0.1113 to 0.1766	No
**311**	0:**1 X** vs. 10:**0 X**	0.01567	-0.1283 to 0.1596	No
**312**	0:**1 X** vs. 10:**1 X**	0.008667	-0.1353 to 0.1526	No
**313**	0:**1 X** vs. 10:**10 X**	0.0060	-0.1380 to 0.1500	No
**314**	0:**1 X** vs. 10:**40 X**	0.01633	-0.1276 to 0.1603	No
**315**	0:**1 X** vs. 10:**50 X**	0.02967	-0.1143 to 0.1736	No

	2way ANOVA<br>Multiple comparisons	A<br>Data Set-A<br>Y	B<br>Data Set-B<br>Y	C<br>Data Set-C<br>Y
316	0:**1 X** vs. 11:**0 X**	0.0060	-0.1380 to 0.1500	No
317	0:**1 X** vs. 11:**1 X**	0.004333	-0.1396 to 0.1483	No
318	0:**1 X** vs. 11:**10 X**	0.004000	-0.1400 to 0.1480	No
319	0:**1 X** vs. 11:**40 X**	0.01233	-0.1316 to 0.1563	No
320	0:**1 X** vs. 11:**50 X**	0.02433	-0.1196 to 0.1683	No
321	0:**1 X** vs. 12:**0 X**	0.005333	-0.1386 to 0.1493	No
322	0:**1 X** vs. 12:**1 X**	0.0230	-0.1210 to 0.1670	No
323	0:**1 X** vs. 12:**10 X**	0.005333	-0.1386 to 0.1493	No
324	0:**1 X** vs. 12:**40 X**	0.0190	-0.1250 to 0.1630	No
325	0:**1 X** vs. 12:**50 X**	0.01567	-0.1283 to 0.1596	No
326	0:**1 X** vs. 13:**0 X**	0.01133	-0.1326 to 0.1553	No
327	0:**1 X** vs. 13:**1 X**	0.0240	-0.1200 to 0.1680	No
328	0:**1 X** vs. 13:**10 X**	0.0020	-0.1420 to 0.1460	No
329	0:**1 X** vs. 13:**40 X**	0.005667	-0.1383 to 0.1496	No
330	0:**1 X** vs. 13:**50 X**	0.004667	-0.1393 to 0.1486	No
331	0:**1 X** vs. 14:**0 X**	0.002667	-0.1413 to 0.1466	No
332	0:**1 X** vs. 14:**1 X**	0.0110	-0.1330 to 0.1550	No
333	0:**1 X** vs. 14:**10 X**	-0.0030	-0.1470 to 0.1410	No
334	0:**1 X** vs. 14:**40 X**	0.0003333	-0.1436 to 0.1443	No
335	0:**1 X** vs. 14:**50 X**	-0.003667	-0.1476 to 0.1403	No
336	0:**1 X** vs. 15:**0 X**	-0.009667	-0.1536 to 0.1343	No
337	0:**1 X** vs. 15:**1 X**	0.01033	-0.1336 to 0.1543	No
338	0:**1 X** vs. 15:**10 X**	-0.0170	-0.1610 to 0.1270	No
339	0:**1 X** vs. 15:**40 X**	0.0006667	-0.1433 to 0.1446	No
340	0:**1 X** vs. 15:**50 X**	-0.0170	-0.1610 to 0.1270	No
341	0:**1 X** vs. 16:**0 X**	-0.1020	-0.2460 to 0.04196	No
342	0:**1 X** vs. 16:**1 X**	0.007000	-0.1370 to 0.1510	No
343	0:**1 X** vs. 16:**10 X**	-0.01867	-0.1626 to 0.1253	No
344	0:**1 X** vs. 16:**40 X**	-0.0380	-0.1820 to 0.1060	No
345	0:**1 X** vs. 16:**50 X**	-0.08033	-0.2243 to 0.06363	No
346	0:**1 X** vs. 17:**0 X**	-0.1547	-0.2986 to -0.01071	Yes
347	0:**1 X** vs. 17:**1 X**	-0.06900	-0.2130 to 0.07496	No
348	0:**1 X** vs. 17:**10 X**	-0.1257	-0.2696 to 0.01829	No
349	0:**1 X** vs. 17:**40 X**	-0.0490	-0.1930 to 0.09496	No
350	0:**1 X** vs. 17:**50 X**	-0.09433	-0.2383 to 0.04963	No
351	0:**1 X** vs. 18:**0 X**	-0.2033	-0.3473 to -0.05937	Yes
352	0:**1 X** vs. 18:**1 X**	-0.1117	-0.2556 to 0.03229	No
353	0:**1 X** vs. 18:**10 X**	-0.2040	-0.3480 to -0.06004	Yes
354	0:**1 X** vs. 18:**40 X**	-0.05333	-0.1973 to 0.09063	No
355	0:**1 X** vs. 18:**50 X**	-0.09833	-0.2423 to 0.04563	No
356	0:**1 X** vs. 19:**0 X**	-0.2383	-0.3823 to -0.09437	Yes
357	0:**1 X** vs. 19:**1 X**	-0.2263	-0.3703 to -0.08237	Yes
358	0:**1 X** vs. 19:**10 X**	-0.2240	-0.3680 to -0.08004	Yes
359	0:**1 X** vs. 19:**40 X**	-0.0820	-0.2260 to 0.06196	No
360	0:**1 X** vs. 19:**50 X**	-0.0930	-0.2370 to 0.05096	No

	2way ANOVA Multiple comparisons	A Data Set-A Y	B Data Set-B Y	C Data Set-C Y
361	0:**1 X** vs. 20:**0 X**	-0.3180	-0.4620 to -0.1740	Yes
362	0:**1 X** vs. 20:**1 X**	-0.1373	-0.2813 to 0.006626	No
363	0:**1 X** vs. 20:**10 X**	-0.2263	-0.3703 to -0.08237	Yes
364	0:**1 X** vs. 20:**40 X**	-0.09367	-0.2376 to 0.05029	No
365	0:**1 X** vs. 20:**50 X**	-0.1377	-0.2816 to 0.006293	No
366	0:**1 X** vs. 21:**0 X**	-0.3413	-0.4853 to -0.1974	Yes
367	0:**1 X** vs. 21:**1 X**	-0.2943	-0.4383 to -0.1504	Yes
368	0:**1 X** vs. 21:**10 X**	-0.2913	-0.4353 to -0.1474	Yes
369	0:**1 X** vs. 21:**40 X**	-0.1667	-0.3106 to -0.02271	Yes
370	0:**1 X** vs. 21:**50 X**	-0.2570	-0.4010 to -0.1130	Yes
371	0:**1 X** vs. 22:**0 X**	-0.3830	-0.5270 to -0.2390	Yes
372	0:**1 X** vs. 22:**1 X**	-0.3777	-0.5216 to -0.2337	Yes
373	0:**1 X** vs. 22:**10 X**	-0.3730	-0.5170 to -0.2290	Yes
374	0:**1 X** vs. 22:**40 X**	-0.2733	-0.4173 to -0.1294	Yes
375	0:**1 X** vs. 22:**50 X**	-0.3360	-0.4800 to -0.1920	Yes
376	0:**1 X** vs. 23:**0 X**	-0.4483	-0.5923 to -0.3044	Yes
377	0:**1 X** vs. 23:**1 X**	-0.6380	-0.7990 to -0.4770	Yes
378	0:**1 X** vs. 23:**10 X**	-0.3877	-0.5316 to -0.2437	Yes
379	0:**1 X** vs. 23:**40 X**	-0.2680	-0.4290 to -0.1070	Yes
380	0:**1 X** vs. 23:**50 X**	-0.3020	-0.4460 to -0.1580	Yes
381	0:**1 X** vs. 24:**0 X**	-0.5657	-0.7096 to -0.4217	Yes
382	0:**1 X** vs. 24:**1 X**	-0.7220	-0.8830 to -0.5610	Yes
383	0:**1 X** vs. 24:**10 X**	-0.5495	-0.7105 to -0.3885	Yes
384	0:**1 X** vs. 24:**40 X**	-0.2843	-0.4283 to -0.1404	Yes
385	0:**1 X** vs. 24:**50 X**	-0.2060	-0.3500 to -0.06204	Yes
386	0:**1 X** vs. 25:**0 X**	-0.9030	-1.064 to -0.7420	Yes
387	0:**1 X** vs. 25:**1 X**	-1.093	-1.253 to -0.9315	Yes
388	0:**1 X** vs. 25:**10 X**	-0.4540	-0.6576 to -0.2504	Yes
389	0:**1 X** vs. 25:**40 X**	-0.2863	-0.4303 to -0.1424	Yes
390	0:**1 X** vs. 25:**50 X**	-0.2607	-0.4046 to -0.1167	Yes
391	0:**1 X** vs. 26:**0 X**	-1.116	-1.276 to -0.9545	Yes
392	0:**1 X** vs. 26:**1 X**	-1.229	-1.389 to -1.068	Yes
393	0:**1 X** vs. 26:**10 X**	-0.9150	-1.076 to -0.7540	Yes
394	0:**1 X** vs. 26:**40 X**	-0.3607	-0.5046 to -0.2167	Yes
395	0:**1 X** vs. 26:**50 X**	-0.2210	-0.3650 to -0.07704	Yes
396	0:**1 X** vs. 27:**0 X**	-1.303	-1.447 to -1.159	Yes
397	0:**1 X** vs. 27:**1 X**	-1.394	-1.555 to -1.233	Yes
398	0:**1 X** vs. 27:**10 X**	-1.206	-1.367 to -1.045	Yes
399	0:**1 X** vs. 27:**40 X**	-0.3607	-0.5046 to -0.2167	Yes
400	0:**1 X** vs. 27:**50 X**	-0.2203	-0.3643 to -0.07637	Yes
401	0:**1 X** vs. 28:**0 X**	-1.253	-1.413 to -1.092	Yes
402	0:**1 X** vs. 28:**1 X**	-1.293	-1.437 to -1.149	Yes
403	0:**1 X** vs. 28:**10 X**	-1.095	-1.256 to -0.9340	Yes
404	0:**1 X** vs. 28:**40 X**	-0.3730	-0.5170 to -0.2290	Yes
405	0:**1 X** vs. 28:**50 X**	-0.2683	-0.4123 to -0.1244	Yes

	2way ANOVA<br>Multiple comparisons	A<br>Data Set-A<br>Y	B<br>Data Set-B<br>Y	C<br>Data Set-C<br>Y
406	0:1 X vs. 29:0 X	-1.734	-1.895 to -1.573	Yes
407	0:1 X vs. 29:1 X	-1.819	-1.980 to -1.658	Yes
408	0:1 X vs. 29:10 X	-1.034	-1.194 to -0.8725	Yes
409	0:1 X vs. 29:40 X	-0.4810	-0.6250 to -0.3370	Yes
410	0:1 X vs. 29:50 X	-0.2697	-0.4136 to -0.1257	Yes
411	0:1 X vs. 30:0 X	-1.449	-1.609 to -1.288	Yes
412	0:1 X vs. 30:1 X	-1.770	-1.931 to -1.609	Yes
413	0:1 X vs. 30:10 X	-1.108	-1.269 to -0.9470	Yes
414	0:1 X vs. 30:40 X	-0.4080	-0.5520 to -0.2640	Yes
415	0:1 X vs. 30:50 X	-0.3220	-0.4660 to -0.1780	Yes
416	0:1 X vs. 31:0 X	-1.767	-1.927 to -1.606	Yes
417	0:1 X vs. 31:1 X	-1.757	-1.918 to -1.596	Yes
418	0:1 X vs. 31:10 X	-1.240	-1.400 to -1.079	Yes
419	0:1 X vs. 31:40 X	-0.4997	-0.6436 to -0.3557	Yes
420	0:1 X vs. 31:50 X	-0.3010	-0.4450 to -0.1570	Yes
421	0:1 X vs. 32:0 X	-2.358	-2.502 to -2.214	Yes
422	0:1 X vs. 32:1 X	-1.814	-1.975 to -1.653	Yes
423	0:1 X vs. 32:10 X	-1.670	-1.814 to -1.526	Yes
424	0:1 X vs. 32:40 X	-0.4960	-0.6400 to -0.3520	Yes
425	0:1 X vs. 32:50 X	-0.3007	-0.4446 to -0.1567	Yes
426	0:1 X vs. 33:0 X	-2.362	-2.506 to -2.218	Yes
427	0:1 X vs. 33:1 X	-2.176	-2.320 to -2.032	Yes
428	0:1 X vs. 33:10 X	-1.837	-1.981 to -1.693	Yes
429	0:1 X vs. 33:40 X	-0.5147	-0.6586 to -0.3707	Yes
430	0:1 X vs. 33:50 X	-0.3157	-0.4596 to -0.1717	Yes
431	0:1 X vs. 34:0 X	-2.286	-2.430 to -2.142	Yes
432	0:1 X vs. 34:1 X	-2.294	-2.438 to -2.150	Yes
433	0:1 X vs. 34:10 X	-1.927	-2.071 to -1.783	Yes
434	0:1 X vs. 34:40 X	-0.5267	-0.6706 to -0.3827	Yes
435	0:1 X vs. 34:50 X	-0.4647	-0.6086 to -0.3207	Yes
436	0:1 X vs. 35:0 X	-2.635	-2.779 to -2.491	Yes
437	0:1 X vs. 35:1 X	-2.401	-2.545 to -2.257	Yes
438	0:1 X vs. 35:10 X	-1.889	-2.033 to -1.745	Yes
439	0:1 X vs. 35:40 X	-0.5357	-0.6796 to -0.3917	Yes
440	0:1 X vs. 35:50 X	-0.4773	-0.6213 to -0.3334	Yes
441	0:1 X vs. 36:0 X	-2.842	-3.002 to -2.681	Yes
442	0:1 X vs. 36:1 X	-2.537	-2.681 to -2.393	Yes
443	0:1 X vs. 36:10 X	-1.894	-2.038 to -1.750	Yes
444	0:1 X vs. 36:40 X	-0.5910	-0.7350 to -0.4470	Yes
445	0:1 X vs. 36:50 X	-0.4407	-0.5846 to -0.2967	Yes
446	0:1 X vs. 37:0 X	-3.225	-3.369 to -3.081	Yes
447	0:1 X vs. 37:1 X	-2.607	-2.751 to -2.463	Yes
448	0:1 X vs. 37:10 X	-1.896	-2.040 to -1.752	Yes
449	0:1 X vs. 37:40 X	-0.6283	-0.7723 to -0.4844	Yes
450	0:1 X vs. 37:50 X	-0.4493	-0.5933 to -0.3054	Yes

	2way ANOVA<br>Multiple comparisons	A<br>Data Set-A<br>Y	B<br>Data Set-B<br>Y	C<br>Data Set-C<br>Y
**451**	0:**1 X** vs. 38:**0 X**	-3.170	-3.314 to -3.026	Yes
**452**	0:**1 X** vs. 38:**1 X**	-2.726	-2.870 to -2.582	Yes
**453**	0:**1 X** vs. 38:**10 X**	-2.171	-2.315 to -2.027	Yes
**454**	0:**1 X** vs. 38:**40 X**	-0.6327	-0.7766 to -0.4887	Yes
**455**	0:**1 X** vs. 38:**50 X**	-0.4533	-0.5973 to -0.3094	Yes
**456**	0:**1 X** vs. 39:**0 X**	-3.401	-3.545 to -3.257	Yes
**457**	0:**1 X** vs. 39:**1 X**	-3.293	-3.437 to -3.149	Yes
**458**	0:**1 X** vs. 39:**10 X**	-2.316	-2.460 to -2.172	Yes
**459**	0:**1 X** vs. 39:**40 X**	-0.6387	-0.7826 to -0.4947	Yes
**460**	0:**1 X** vs. 39:**50 X**	-0.4843	-0.6283 to -0.3404	Yes
**461**	0:**1 X** vs. 40:**0 X**	-3.442	-3.586 to -3.298	Yes
**462**	0:**1 X** vs. 40:**1 X**	-3.368	-3.512 to -3.224	Yes
**463**	0:**1 X** vs. 40:**10 X**	-2.573	-2.717 to -2.429	Yes
**464**	0:**1 X** vs. 40:**40 X**	-0.6433	-0.7873 to -0.4994	Yes
**465**	0:**1 X** vs. 40:**50 X**	-0.4907	-0.6346 to -0.3467	Yes
**466**	0:**1 X** vs. 41:**0 X**	-3.483	-3.627 to -3.339	Yes
**467**	0:**1 X** vs. 41:**1 X**	-3.482	-3.626 to -3.338	Yes
**468**	0:**1 X** vs. 41:**10 X**	-2.626	-2.770 to -2.482	Yes
**469**	0:**1 X** vs. 41:**40 X**	-0.5907	-0.7346 to -0.4467	Yes
**470**	0:**1 X** vs. 41:**50 X**	-0.5033	-0.6473 to -0.3594	Yes
**471**	0:**1 X** vs. 42:**0 X**	-3.625	-3.769 to -3.481	Yes
**472**	0:**1 X** vs. 42:**1 X**	-3.644	-3.788 to -3.500	Yes
**473**	0:**1 X** vs. 42:**10 X**	-2.627	-2.771 to -2.483	Yes
**474**	0:**1 X** vs. 42:**40 X**	-0.6033	-0.7473 to -0.4594	Yes
**475**	0:**1 X** vs. 42:**50 X**	-0.5067	-0.6506 to -0.3627	Yes
**476**	0:**1 X** vs. 43:**0 X**	-3.715	-3.859 to -3.571	Yes
**477**	0:**1 X** vs. 43:**1 X**	-3.629	-3.773 to -3.485	Yes
**478**	0:**1 X** vs. 43:**10 X**	-3.345	-3.489 to -3.201	Yes
**479**	0:**1 X** vs. 43:**40 X**	-0.6017	-0.7456 to -0.4577	Yes
**480**	0:**1 X** vs. 43:**50 X**	-0.5070	-0.6510 to -0.3630	Yes
**481**	0:**1 X** vs. 44:**0 X**	-3.739	-3.883 to -3.595	Yes
**482**	0:**1 X** vs. 44:**1 X**	-3.628	-3.772 to -3.484	Yes
**483**	0:**1 X** vs. 44:**10 X**	-3.408	-3.552 to -3.264	Yes
**484**	0:**1 X** vs. 44:**40 X**	-0.6037	-0.7476 to -0.4597	Yes
**485**	0:**1 X** vs. 44:**50 X**	-0.4393	-0.5833 to -0.2954	Yes
**486**	0:**1 X** vs. 45:**0 X**	-3.603	-3.747 to -3.459	Yes
**487**	0:**1 X** vs. 45:**1 X**	-3.405	-3.549 to -3.261	Yes
**488**	0:**1 X** vs. 45:**10 X**	-3.288	-3.432 to -3.144	Yes
**489**	0:**1 X** vs. 45:**40 X**	-0.6050	-0.7490 to -0.4610	Yes
**490**	0:**1 X** vs. 45:**50 X**	-0.4363	-0.5803 to -0.2924	Yes
**491**	0:**1 X** vs. 46:**0 X**	-3.408	-3.552 to -3.264	Yes
**492**	0:**1 X** vs. 46:**1 X**	-3.375	-3.519 to -3.231	Yes
**493**	0:**1 X** vs. 46:**10 X**	-3.283	-3.427 to -3.139	Yes
**494**	0:**1 X** vs. 46:**40 X**	-0.6187	-0.7626 to -0.4747	Yes
**495**	0:**1 X** vs. 46:**50 X**	-0.4293	-0.5733 to -0.2854	Yes

	2way ANOVA<br>Multiple comparisons	A<br>Data Set-A<br>Y	B<br>Data Set-B<br>Y	C<br>Data Set-C<br>Y
496	0:1 X vs. 47:0 X	-3.383	-3.527 to -3.239	Yes
497	0:1 X vs. 47:1 X	-3.287	-3.431 to -3.143	Yes
498	0:1 X vs. 47:10 X	-3.269	-3.413 to -3.125	Yes
499	0:1 X vs. 47:40 X	-0.6287	-0.7726 to -0.4847	Yes
500	0:1 X vs. 47:50 X	-0.3873	-0.5313 to -0.2434	Yes
501	0:1 X vs. 48:0 X	-3.371	-3.531 to -3.210	Yes
502	0:1 X vs. 48:1 X	-3.274	-3.418 to -3.130	Yes
503	0:1 X vs. 48:10 X	-3.269	-3.413 to -3.125	Yes
504	0:1 X vs. 48:40 X	-0.6350	-0.7790 to -0.4910	Yes
505	0:1 X vs. 48:50 X	-0.3947	-0.5386 to -0.2507	Yes
506	0:1 X vs. 49:0 X	-3.371	-3.515 to -3.227	Yes
507	0:1 X vs. 49:1 X	-3.274	-3.418 to -3.130	Yes
508	0:1 X vs. 49:10 X	-3.269	-3.413 to -3.125	Yes
509	0:1 X vs. 49:40 X	-0.6393	-0.7833 to -0.4954	Yes
510	0:1 X vs. 49:50 X	-0.3940	-0.5380 to -0.2500	Yes
511	0:1 X vs. 50:0 X	-3.371	-3.515 to -3.227	Yes
512	0:1 X vs. 50:1 X	-3.274	-3.418 to -3.130	Yes
513	0:1 X vs. 50:10 X	-3.269	-3.413 to -3.125	Yes
514	0:1 X vs. 50:40 X	-0.6470	-0.7910 to -0.5030	Yes
515	0:1 X vs. 50:50 X	-0.3937	-0.5376 to -0.2497	Yes
516	0:10 X vs. 0:40 X	0.0	-0.1440 to 0.1440	No
517	0:10 X vs. 0:50 X	0.0	-0.1440 to 0.1440	No
518	0:10 X vs. 1:0 X	0.04233	-0.1016 to 0.1863	No
519	0:10 X vs. 1:1 X	0.03467	-0.1093 to 0.1786	No
520	0:10 X vs. 1:10 X	0.0440	-0.09996 to 0.1880	No
521	0:10 X vs. 1:40 X	0.03833	-0.1056 to 0.1823	No
522	0:10 X vs. 1:50 X	0.0410	-0.1030 to 0.1850	No
523	0:10 X vs. 2:0 X	0.03767	-0.1063 to 0.1816	No
524	0:10 X vs. 2:1 X	0.03267	-0.1113 to 0.1766	No
525	0:10 X vs. 2:10 X	0.03867	-0.1053 to 0.1826	No
526	0:10 X vs. 2:40 X	0.0380	-0.1060 to 0.1820	No
527	0:10 X vs. 2:50 X	0.0410	-0.1030 to 0.1850	No
528	0:10 X vs. 3:0 X	0.03667	-0.1073 to 0.1806	No
529	0:10 X vs. 3:1 X	0.03433	-0.1096 to 0.1783	No
530	0:10 X vs. 3:10 X	0.0390	-0.1050 to 0.1830	No
531	0:10 X vs. 3:40 X	0.04133	-0.1026 to 0.1853	No
532	0:10 X vs. 3:50 X	0.04233	-0.1016 to 0.1863	No
533	0:10 X vs. 4:0 X	0.03233	-0.1116 to 0.1763	No
534	0:10 X vs. 4:1 X	0.03367	-0.1103 to 0.1776	No
535	0:10 X vs. 4:10 X	0.0190	-0.1250 to 0.1630	No
536	0:10 X vs. 4:40 X	0.03933	-0.1046 to 0.1833	No
537	0:10 X vs. 4:50 X	0.04133	-0.1026 to 0.1853	No
538	0:10 X vs. 5:0 X	0.03133	-0.1126 to 0.1753	No
539	0:10 X vs. 5:1 X	0.03333	-0.1106 to 0.1773	No
540	0:10 X vs. 5:10 X	0.01833	-0.1256 to 0.1623	No

	2way ANOVA<br>Multiple comparisons	A<br>Data Set-A<br>Y	B<br>Data Set-B<br>Y	C<br>Data Set-C<br>Y
541	0:10 X vs. 5:40 X	0.03933	-0.1046 to 0.1833	No
542	0:10 X vs. 5:50 X	0.04033	-0.1036 to 0.1843	No
543	0:10 X vs. 6:0 X	0.0320	-0.1120 to 0.1760	No
544	0:10 X vs. 6:1 X	0.0330	-0.1110 to 0.1770	No
545	0:10 X vs. 6:10 X	0.01833	-0.1256 to 0.1623	No
546	0:10 X vs. 6:40 X	0.0370	-0.1070 to 0.1810	No
547	0:10 X vs. 6:50 X	0.03867	-0.1053 to 0.1826	No
548	0:10 X vs. 7:0 X	0.0300	-0.1140 to 0.1740	No
549	0:10 X vs. 7:1 X	0.03867	-0.1053 to 0.1826	No
550	0:10 X vs. 7:10 X	0.02667	-0.1173 to 0.1706	No
551	0:10 X vs. 7:40 X	0.03533	-0.1086 to 0.1793	No
552	0:10 X vs. 7:50 X	0.03733	-0.1066 to 0.1813	No
553	0:10 X vs. 8:0 X	0.02533	-0.1186 to 0.1693	No
554	0:10 X vs. 8:1 X	0.03867	-0.1053 to 0.1826	No
555	0:10 X vs. 8:10 X	0.03133	-0.1126 to 0.1753	No
556	0:10 X vs. 8:40 X	0.03067	-0.1133 to 0.1746	No
557	0:10 X vs. 8:50 X	0.03367	-0.1103 to 0.1776	No
558	0:10 X vs. 9:0 X	0.01733	-0.1266 to 0.1613	No
559	0:10 X vs. 9:1 X	0.009333	-0.1346 to 0.1533	No
560	0:10 X vs. 9:10 X	0.008333	-0.1356 to 0.1523	No
561	0:10 X vs. 9:40 X	0.0260	-0.1180 to 0.1700	No
562	0:10 X vs. 9:50 X	0.03267	-0.1113 to 0.1766	No
563	0:10 X vs. 10:0 X	0.01567	-0.1283 to 0.1596	No
564	0:10 X vs. 10:1 X	0.008667	-0.1353 to 0.1526	No
565	0:10 X vs. 10:10 X	0.0060	-0.1380 to 0.1500	No
566	0:10 X vs. 10:40 X	0.01633	-0.1276 to 0.1603	No
567	0:10 X vs. 10:50 X	0.02967	-0.1143 to 0.1736	No
568	0:10 X vs. 11:0 X	0.0060	-0.1380 to 0.1500	No
569	0:10 X vs. 11:1 X	0.004333	-0.1396 to 0.1483	No
570	0:10 X vs. 11:10 X	0.004000	-0.1400 to 0.1480	No
571	0:10 X vs. 11:40 X	0.01233	-0.1316 to 0.1563	No
572	0:10 X vs. 11:50 X	0.02433	-0.1196 to 0.1683	No
573	0:10 X vs. 12:0 X	0.005333	-0.1386 to 0.1493	No
574	0:10 X vs. 12:1 X	0.0230	-0.1210 to 0.1670	No
575	0:10 X vs. 12:10 X	0.005333	-0.1386 to 0.1493	No
576	0:10 X vs. 12:40 X	0.0190	-0.1250 to 0.1630	No
577	0:10 X vs. 12:50 X	0.01567	-0.1283 to 0.1596	No
578	0:10 X vs. 13:0 X	0.01133	-0.1326 to 0.1553	No
579	0:10 X vs. 13:1 X	0.0240	-0.1200 to 0.1680	No
580	0:10 X vs. 13:10 X	0.0020	-0.1420 to 0.1460	No
581	0:10 X vs. 13:40 X	0.005667	-0.1383 to 0.1496	No
582	0:10 X vs. 13:50 X	0.004667	-0.1393 to 0.1486	No
583	0:10 X vs. 14:0 X	0.002667	-0.1413 to 0.1466	No
584	0:10 X vs. 14:1 X	0.0110	-0.1330 to 0.1550	No
585	0:10 X vs. 14:10 X	-0.0030	-0.1470 to 0.1410	No

	2way ANOVA<br>Multiple comparisons	A<br>Data Set-A<br>Y	B<br>Data Set-B<br>Y	C<br>Data Set-C<br>Y
586	0:**10 X** vs. 14:**40 X**	0.0003333	-0.1436 to 0.1443	No
587	0:**10 X** vs. 14:**50 X**	-0.003667	-0.1476 to 0.1403	No
588	0:**10 X** vs. 15:**0 X**	-0.009667	-0.1536 to 0.1343	No
589	0:**10 X** vs. 15:**1 X**	0.01033	-0.1336 to 0.1543	No
590	0:**10 X** vs. 15:**10 X**	-0.0170	-0.1610 to 0.1270	No
591	0:**10 X** vs. 15:**40 X**	0.0006667	-0.1433 to 0.1446	No
592	0:**10 X** vs. 15:**50 X**	-0.0170	-0.1610 to 0.1270	No
593	0:**10 X** vs. 16:**0 X**	-0.1020	-0.2460 to 0.04196	No
594	0:**10 X** vs. 16:**1 X**	0.007000	-0.1370 to 0.1510	No
595	0:**10 X** vs. 16:**10 X**	-0.01867	-0.1626 to 0.1253	No
596	0:**10 X** vs. 16:**40 X**	-0.0380	-0.1820 to 0.1060	No
597	0:**10 X** vs. 16:**50 X**	-0.08033	-0.2243 to 0.06363	No
598	0:**10 X** vs. 17:**0 X**	-0.1547	-0.2986 to -0.01071	Yes
599	0:**10 X** vs. 17:**1 X**	-0.06900	-0.2130 to 0.07496	No
600	0:**10 X** vs. 17:**10 X**	-0.1257	-0.2696 to 0.01829	No
601	0:**10 X** vs. 17:**40 X**	-0.0490	-0.1930 to 0.09496	No
602	0:**10 X** vs. 17:**50 X**	-0.09433	-0.2383 to 0.04963	No
603	0:**10 X** vs. 18:**0 X**	-0.2033	-0.3473 to -0.05937	Yes
604	0:**10 X** vs. 18:**1 X**	-0.1117	-0.2556 to 0.03229	No
605	0:**10 X** vs. 18:**10 X**	-0.2040	-0.3480 to -0.06004	Yes
606	0:**10 X** vs. 18:**40 X**	-0.05333	-0.1973 to 0.09063	No
607	0:**10 X** vs. 18:**50 X**	-0.09833	-0.2423 to 0.04563	No
608	0:**10 X** vs. 19:**0 X**	-0.2383	-0.3823 to -0.09437	Yes
609	0:**10 X** vs. 19:**1 X**	-0.2263	-0.3703 to -0.08237	Yes
610	0:**10 X** vs. 19:**10 X**	-0.2240	-0.3680 to -0.08004	Yes
611	0:**10 X** vs. 19:**40 X**	-0.0820	-0.2260 to 0.06196	No
612	0:**10 X** vs. 19:**50 X**	-0.0930	-0.2370 to 0.05096	No
613	0:**10 X** vs. 20:**0 X**	-0.3180	-0.4620 to -0.1740	Yes
614	0:**10 X** vs. 20:**1 X**	-0.1373	-0.2813 to 0.006626	No
615	0:**10 X** vs. 20:**10 X**	-0.2263	-0.3703 to -0.08237	Yes
616	0:**10 X** vs. 20:**40 X**	-0.09367	-0.2376 to 0.05029	No
617	0:**10 X** vs. 20:**50 X**	-0.1377	-0.2816 to 0.006293	No
618	0:**10 X** vs. 21:**0 X**	-0.3413	-0.4853 to -0.1974	Yes
619	0:**10 X** vs. 21:**1 X**	-0.2943	-0.4383 to -0.1504	Yes
620	0:**10 X** vs. 21:**10 X**	-0.2913	-0.4353 to -0.1474	Yes
621	0:**10 X** vs. 21:**40 X**	-0.1667	-0.3106 to -0.02271	Yes
622	0:**10 X** vs. 21:**50 X**	-0.2570	-0.4010 to -0.1130	Yes
623	0:**10 X** vs. 22:**0 X**	-0.3830	-0.5270 to -0.2390	Yes
624	0:**10 X** vs. 22:**1 X**	-0.3777	-0.5216 to -0.2337	Yes
625	0:**10 X** vs. 22:**10 X**	-0.3730	-0.5170 to -0.2290	Yes
626	0:**10 X** vs. 22:**40 X**	-0.2733	-0.4173 to -0.1294	Yes
627	0:**10 X** vs. 22:**50 X**	-0.3360	-0.4800 to -0.1920	Yes
628	0:**10 X** vs. 23:**0 X**	-0.4483	-0.5923 to -0.3044	Yes
629	0:**10 X** vs. 23:**1 X**	-0.6380	-0.7990 to -0.4770	Yes
630	0:**10 X** vs. 23:**10 X**	-0.3877	-0.5316 to -0.2437	Yes

	2way ANOVA<br>Multiple comparisons	A<br>Data Set-A<br>Y	B<br>Data Set-B<br>Y	C<br>Data Set-C<br>Y
631	0:**10 X** vs. 23:**40 X**	-0.2680	-0.4290 to -0.1070	Yes
632	0:**10 X** vs. 23:**50 X**	-0.3020	-0.4460 to -0.1580	Yes
633	0:**10 X** vs. 24:**0 X**	-0.5657	-0.7096 to -0.4217	Yes
634	0:**10 X** vs. 24:**1 X**	-0.7220	-0.8830 to -0.5610	Yes
635	0:**10 X** vs. 24:**10 X**	-0.5495	-0.7105 to -0.3885	Yes
636	0:**10 X** vs. 24:**40 X**	-0.2843	-0.4283 to -0.1404	Yes
637	0:**10 X** vs. 24:**50 X**	-0.2060	-0.3500 to -0.06204	Yes
638	0:**10 X** vs. 25:**0 X**	-0.9030	-1.064 to -0.7420	Yes
639	0:**10 X** vs. 25:**1 X**	-1.093	-1.253 to -0.9315	Yes
640	0:**10 X** vs. 25:**10 X**	-0.4540	-0.6576 to -0.2504	Yes
641	0:**10 X** vs. 25:**40 X**	-0.2863	-0.4303 to -0.1424	Yes
642	0:**10 X** vs. 25:**50 X**	-0.2607	-0.4046 to -0.1167	Yes
643	0:**10 X** vs. 26:**0 X**	-1.116	-1.276 to -0.9545	Yes
644	0:**10 X** vs. 26:**1 X**	-1.229	-1.389 to -1.068	Yes
645	0:**10 X** vs. 26:**10 X**	-0.9150	-1.076 to -0.7540	Yes
646	0:**10 X** vs. 26:**40 X**	-0.3607	-0.5046 to -0.2167	Yes
647	0:**10 X** vs. 26:**50 X**	-0.2210	-0.3650 to -0.07704	Yes
648	0:**10 X** vs. 27:**0 X**	-1.303	-1.447 to -1.159	Yes
649	0:**10 X** vs. 27:**1 X**	-1.394	-1.555 to -1.233	Yes
650	0:**10 X** vs. 27:**10 X**	-1.206	-1.367 to -1.045	Yes
651	0:**10 X** vs. 27:**40 X**	-0.3607	-0.5046 to -0.2167	Yes
652	0:**10 X** vs. 27:**50 X**	-0.2203	-0.3643 to -0.07637	Yes
653	0:**10 X** vs. 28:**0 X**	-1.253	-1.413 to -1.092	Yes
654	0:**10 X** vs. 28:**1 X**	-1.293	-1.437 to -1.149	Yes
655	0:**10 X** vs. 28:**10 X**	-1.095	-1.256 to -0.9340	Yes
656	0:**10 X** vs. 28:**40 X**	-0.3730	-0.5170 to -0.2290	Yes
657	0:**10 X** vs. 28:**50 X**	-0.2683	-0.4123 to -0.1244	Yes
658	0:**10 X** vs. 29:**0 X**	-1.734	-1.895 to -1.573	Yes
659	0:**10 X** vs. 29:**1 X**	-1.819	-1.980 to -1.658	Yes
660	0:**10 X** vs. 29:**10 X**	-1.034	-1.194 to -0.8725	Yes
661	0:**10 X** vs. 29:**40 X**	-0.4810	-0.6250 to -0.3370	Yes
662	0:**10 X** vs. 29:**50 X**	-0.2697	-0.4136 to -0.1257	Yes
663	0:**10 X** vs. 30:**0 X**	-1.449	-1.609 to -1.288	Yes
664	0:**10 X** vs. 30:**1 X**	-1.770	-1.931 to -1.609	Yes
665	0:**10 X** vs. 30:**10 X**	-1.108	-1.269 to -0.9470	Yes
666	0:**10 X** vs. 30:**40 X**	-0.4080	-0.5520 to -0.2640	Yes
667	0:**10 X** vs. 30:**50 X**	-0.3220	-0.4660 to -0.1780	Yes
668	0:**10 X** vs. 31:**0 X**	-1.767	-1.927 to -1.606	Yes
669	0:**10 X** vs. 31:**1 X**	-1.757	-1.918 to -1.596	Yes
670	0:**10 X** vs. 31:**10 X**	-1.240	-1.400 to -1.079	Yes
671	0:**10 X** vs. 31:**40 X**	-0.4997	-0.6436 to -0.3557	Yes
672	0:**10 X** vs. 31:**50 X**	-0.3010	-0.4450 to -0.1570	Yes
673	0:**10 X** vs. 32:**0 X**	-2.358	-2.502 to -2.214	Yes
674	0:**10 X** vs. 32:**1 X**	-1.814	-1.975 to -1.653	Yes
675	0:**10 X** vs. 32:**10 X**	-1.670	-1.814 to -1.526	Yes

	2way ANOVA<br>Multiple comparisons	A<br>Data Set-A<br>Y	B<br>Data Set-B<br>Y	C<br>Data Set-C<br>Y
676	0:**10 X** vs. 32:**40 X**	-0.4960	-0.6400 to -0.3520	Yes
677	0:**10 X** vs. 32:**50 X**	-0.3007	-0.4446 to -0.1567	Yes
678	0:**10 X** vs. 33:**0 X**	-2.362	-2.506 to -2.218	Yes
679	0:**10 X** vs. 33:**1 X**	-2.176	-2.320 to -2.032	Yes
680	0:**10 X** vs. 33:**10 X**	-1.837	-1.981 to -1.693	Yes
681	0:**10 X** vs. 33:**40 X**	-0.5147	-0.6586 to -0.3707	Yes
682	0:**10 X** vs. 33:**50 X**	-0.3157	-0.4596 to -0.1717	Yes
683	0:**10 X** vs. 34:**0 X**	-2.286	-2.430 to -2.142	Yes
684	0:**10 X** vs. 34:**1 X**	-2.294	-2.438 to -2.150	Yes
685	0:**10 X** vs. 34:**10 X**	-1.927	-2.071 to -1.783	Yes
686	0:**10 X** vs. 34:**40 X**	-0.5267	-0.6706 to -0.3827	Yes
687	0:**10 X** vs. 34:**50 X**	-0.4647	-0.6086 to -0.3207	Yes
688	0:**10 X** vs. 35:**0 X**	-2.635	-2.779 to -2.491	Yes
689	0:**10 X** vs. 35:**1 X**	-2.401	-2.545 to -2.257	Yes
690	0:**10 X** vs. 35:**10 X**	-1.889	-2.033 to -1.745	Yes
691	0:**10 X** vs. 35:**40 X**	-0.5357	-0.6796 to -0.3917	Yes
692	0:**10 X** vs. 35:**50 X**	-0.4773	-0.6213 to -0.3334	Yes
693	0:**10 X** vs. 36:**0 X**	-2.842	-3.002 to -2.681	Yes
694	0:**10 X** vs. 36:**1 X**	-2.537	-2.681 to -2.393	Yes
695	0:**10 X** vs. 36:**10 X**	-1.894	-2.038 to -1.750	Yes
696	0:**10 X** vs. 36:**40 X**	-0.5910	-0.7350 to -0.4470	Yes
697	0:**10 X** vs. 36:**50 X**	-0.4407	-0.5846 to -0.2967	Yes
698	0:**10 X** vs. 37:**0 X**	-3.225	-3.369 to -3.081	Yes
699	0:**10 X** vs. 37:**1 X**	-2.607	-2.751 to -2.463	Yes
700	0:**10 X** vs. 37:**10 X**	-1.896	-2.040 to -1.752	Yes
701	0:**10 X** vs. 37:**40 X**	-0.6283	-0.7723 to -0.4844	Yes
702	0:**10 X** vs. 37:**50 X**	-0.4493	-0.5933 to -0.3054	Yes
703	0:**10 X** vs. 38:**0 X**	-3.170	-3.314 to -3.026	Yes
704	0:**10 X** vs. 38:**1 X**	-2.726	-2.870 to -2.582	Yes
705	0:**10 X** vs. 38:**10 X**	-2.171	-2.315 to -2.027	Yes
706	0:**10 X** vs. 38:**40 X**	-0.6327	-0.7766 to -0.4887	Yes
707	0:**10 X** vs. 38:**50 X**	-0.4533	-0.5973 to -0.3094	Yes
708	0:**10 X** vs. 39:**0 X**	-3.401	-3.545 to -3.257	Yes
709	0:**10 X** vs. 39:**1 X**	-3.293	-3.437 to -3.149	Yes
710	0:**10 X** vs. 39:**10 X**	-2.316	-2.460 to -2.172	Yes
711	0:**10 X** vs. 39:**40 X**	-0.6387	-0.7826 to -0.4947	Yes
712	0:**10 X** vs. 39:**50 X**	-0.4843	-0.6283 to -0.3404	Yes
713	0:**10 X** vs. 40:**0 X**	-3.442	-3.586 to -3.298	Yes
714	0:**10 X** vs. 40:**1 X**	-3.368	-3.512 to -3.224	Yes
715	0:**10 X** vs. 40:**10 X**	-2.573	-2.717 to -2.429	Yes
716	0:**10 X** vs. 40:**40 X**	-0.6433	-0.7873 to -0.4994	Yes
717	0:**10 X** vs. 40:**50 X**	-0.4907	-0.6346 to -0.3467	Yes
718	0:**10 X** vs. 41:**0 X**	-3.483	-3.627 to -3.339	Yes
719	0:**10 X** vs. 41:**1 X**	-3.482	-3.626 to -3.338	Yes
720	0:**10 X** vs. 41:**10 X**	-2.626	-2.770 to -2.482	Yes

	2way ANOVA<br>Multiple comparisons	A<br>Data Set-A<br>Y	B<br>Data Set-B<br>Y	C<br>Data Set-C<br>Y
721	0:10 X vs. 41:40 X	-0.5907	-0.7346 to -0.4467	Yes
722	0:10 X vs. 41:50 X	-0.5033	-0.6473 to -0.3594	Yes
723	0:10 X vs. 42:0 X	-3.625	-3.769 to -3.481	Yes
724	0:10 X vs. 42:1 X	-3.644	-3.788 to -3.500	Yes
725	0:10 X vs. 42:10 X	-2.627	-2.771 to -2.483	Yes
726	0:10 X vs. 42:40 X	-0.6033	-0.7473 to -0.4594	Yes
727	0:10 X vs. 42:50 X	-0.5067	-0.6506 to -0.3627	Yes
728	0:10 X vs. 43:0 X	-3.715	-3.859 to -3.571	Yes
729	0:10 X vs. 43:1 X	-3.629	-3.773 to -3.485	Yes
730	0:10 X vs. 43:10 X	-3.345	-3.489 to -3.201	Yes
731	0:10 X vs. 43:40 X	-0.6017	-0.7456 to -0.4577	Yes
732	0:10 X vs. 43:50 X	-0.5070	-0.6510 to -0.3630	Yes
733	0:10 X vs. 44:0 X	-3.739	-3.883 to -3.595	Yes
734	0:10 X vs. 44:1 X	-3.628	-3.772 to -3.484	Yes
735	0:10 X vs. 44:10 X	-3.408	-3.552 to -3.264	Yes
736	0:10 X vs. 44:40 X	-0.6037	-0.7476 to -0.4597	Yes
737	0:10 X vs. 44:50 X	-0.4393	-0.5833 to -0.2954	Yes
738	0:10 X vs. 45:0 X	-3.603	-3.747 to -3.459	Yes
739	0:10 X vs. 45:1 X	-3.405	-3.549 to -3.261	Yes
740	0:10 X vs. 45:10 X	-3.288	-3.432 to -3.144	Yes
741	0:10 X vs. 45:40 X	-0.6050	-0.7490 to -0.4610	Yes
742	0:10 X vs. 45:50 X	-0.4363	-0.5803 to -0.2924	Yes
743	0:10 X vs. 46:0 X	-3.408	-3.552 to -3.264	Yes
744	0:10 X vs. 46:1 X	-3.375	-3.519 to -3.231	Yes
745	0:10 X vs. 46:10 X	-3.283	-3.427 to -3.139	Yes
746	0:10 X vs. 46:40 X	-0.6187	-0.7626 to -0.4747	Yes
747	0:10 X vs. 46:50 X	-0.4293	-0.5733 to -0.2854	Yes
748	0:10 X vs. 47:0 X	-3.383	-3.527 to -3.239	Yes
749	0:10 X vs. 47:1 X	-3.287	-3.431 to -3.143	Yes
750	0:10 X vs. 47:10 X	-3.269	-3.413 to -3.125	Yes
751	0:10 X vs. 47:40 X	-0.6287	-0.7726 to -0.4847	Yes
752	0:10 X vs. 47:50 X	-0.3873	-0.5313 to -0.2434	Yes
753	0:10 X vs. 48:0 X	-3.371	-3.531 to -3.210	Yes
754	0:10 X vs. 48:1 X	-3.274	-3.418 to -3.130	Yes
755	0:10 X vs. 48:10 X	-3.269	-3.413 to -3.125	Yes
756	0:10 X vs. 48:40 X	-0.6350	-0.7790 to -0.4910	Yes
757	0:10 X vs. 48:50 X	-0.3947	-0.5386 to -0.2507	Yes
758	0:10 X vs. 49:0 X	-3.371	-3.515 to -3.227	Yes
759	0:10 X vs. 49:1 X	-3.274	-3.418 to -3.130	Yes
760	0:10 X vs. 49:10 X	-3.269	-3.413 to -3.125	Yes
761	0:10 X vs. 49:40 X	-0.6393	-0.7833 to -0.4954	Yes
762	0:10 X vs. 49:50 X	-0.3940	-0.5380 to -0.2500	Yes
763	0:10 X vs. 50:0 X	-3.371	-3.515 to -3.227	Yes
764	0:10 X vs. 50:1 X	-3.274	-3.418 to -3.130	Yes
765	0:10 X vs. 50:10 X	-3.269	-3.413 to -3.125	Yes

	2way ANOVA Multiple comparisons	A Data Set-A Y	B Data Set-B Y	C Data Set-C Y
766	0:**10 X** vs. 50:**40 X**	-0.6470	-0.7910 to -0.5030	Yes
767	0:**10 X** vs. 50:**50 X**	-0.3937	-0.5376 to -0.2497	Yes
768	0:**40 X** vs. 0:**50 X**	0.0	-0.1440 to 0.1440	No
769	0:**40 X** vs. 1:**0 X**	0.04233	-0.1016 to 0.1863	No
770	0:**40 X** vs. 1:**1 X**	0.03467	-0.1093 to 0.1786	No
771	0:**40 X** vs. 1:**10 X**	0.0440	-0.09996 to 0.1880	No
772	0:**40 X** vs. 1:**40 X**	0.03833	-0.1056 to 0.1823	No
773	0:**40 X** vs. 1:**50 X**	0.0410	-0.1030 to 0.1850	No
774	0:**40 X** vs. 2:**0 X**	0.03767	-0.1063 to 0.1816	No
775	0:**40 X** vs. 2:**1 X**	0.03267	-0.1113 to 0.1766	No
776	0:**40 X** vs. 2:**10 X**	0.03867	-0.1053 to 0.1826	No
777	0:**40 X** vs. 2:**40 X**	0.0380	-0.1060 to 0.1820	No
778	0:**40 X** vs. 2:**50 X**	0.0410	-0.1030 to 0.1850	No
779	0:**40 X** vs. 3:**0 X**	0.03667	-0.1073 to 0.1806	No
780	0:**40 X** vs. 3:**1 X**	0.03433	-0.1096 to 0.1783	No
781	0:**40 X** vs. 3:**10 X**	0.0390	-0.1050 to 0.1830	No
782	0:**40 X** vs. 3:**40 X**	0.04133	-0.1026 to 0.1853	No
783	0:**40 X** vs. 3:**50 X**	0.04233	-0.1016 to 0.1863	No
784	0:**40 X** vs. 4:**0 X**	0.03233	-0.1116 to 0.1763	No
785	0:**40 X** vs. 4:**1 X**	0.03367	-0.1103 to 0.1776	No
786	0:**40 X** vs. 4:**10 X**	0.0190	-0.1250 to 0.1630	No
787	0:**40 X** vs. 4:**40 X**	0.03933	-0.1046 to 0.1833	No
788	0:**40 X** vs. 4:**50 X**	0.04133	-0.1026 to 0.1853	No
789	0:**40 X** vs. 5:**0 X**	0.03133	-0.1126 to 0.1753	No
790	0:**40 X** vs. 5:**1 X**	0.03333	-0.1106 to 0.1773	No
791	0:**40 X** vs. 5:**10 X**	0.01833	-0.1256 to 0.1623	No
792	0:**40 X** vs. 5:**40 X**	0.03933	-0.1046 to 0.1833	No
793	0:**40 X** vs. 5:**50 X**	0.04033	-0.1036 to 0.1843	No
794	0:**40 X** vs. 6:**0 X**	0.0320	-0.1120 to 0.1760	No
795	0:**40 X** vs. 6:**1 X**	0.0330	-0.1110 to 0.1770	No
796	0:**40 X** vs. 6:**10 X**	0.01833	-0.1256 to 0.1623	No
797	0:**40 X** vs. 6:**40 X**	0.0370	-0.1070 to 0.1810	No
798	0:**40 X** vs. 6:**50 X**	0.03867	-0.1053 to 0.1826	No
799	0:**40 X** vs. 7:**0 X**	0.0300	-0.1140 to 0.1740	No
800	0:**40 X** vs. 7:**1 X**	0.03867	-0.1053 to 0.1826	No
801	0:**40 X** vs. 7:**10 X**	0.02667	-0.1173 to 0.1706	No
802	0:**40 X** vs. 7:**40 X**	0.03533	-0.1086 to 0.1793	No
803	0:**40 X** vs. 7:**50 X**	0.03733	-0.1066 to 0.1813	No
804	0:**40 X** vs. 8:**0 X**	0.02533	-0.1186 to 0.1693	No
805	0:**40 X** vs. 8:**1 X**	0.03867	-0.1053 to 0.1826	No
806	0:**40 X** vs. 8:**10 X**	0.03133	-0.1126 to 0.1753	No
807	0:**40 X** vs. 8:**40 X**	0.03067	-0.1133 to 0.1746	No
808	0:**40 X** vs. 8:**50 X**	0.03367	-0.1103 to 0.1776	No
809	0:**40 X** vs. 9:**0 X**	0.01733	-0.1266 to 0.1613	No
810	0:**40 X** vs. 9:**1 X**	0.009333	-0.1346 to 0.1533	No

	2way ANOVA<br>Multiple comparisons	A<br>Data Set-A<br>Y	B<br>Data Set-B<br>Y	C<br>Data Set-C<br>Y
811	0:**40 X** vs. 9:**10 X**	0.008333	-0.1356 to 0.1523	No
812	0:**40 X** vs. 9:**40 X**	0.0260	-0.1180 to 0.1700	No
813	0:**40 X** vs. 9:**50 X**	0.03267	-0.1113 to 0.1766	No
814	0:**40 X** vs. 10:**0 X**	0.01567	-0.1283 to 0.1596	No
815	0:**40 X** vs. 10:**1 X**	0.008667	-0.1353 to 0.1526	No
816	0:**40 X** vs. 10:**10 X**	0.0060	-0.1380 to 0.1500	No
817	0:**40 X** vs. 10:**40 X**	0.01633	-0.1276 to 0.1603	No
818	0:**40 X** vs. 10:**50 X**	0.02967	-0.1143 to 0.1736	No
819	0:**40 X** vs. 11:**0 X**	0.0060	-0.1380 to 0.1500	No
820	0:**40 X** vs. 11:**1 X**	0.004333	-0.1396 to 0.1483	No
821	0:**40 X** vs. 11:**10 X**	0.004000	-0.1400 to 0.1480	No
822	0:**40 X** vs. 11:**40 X**	0.01233	-0.1316 to 0.1563	No
823	0:**40 X** vs. 11:**50 X**	0.02433	-0.1196 to 0.1683	No
824	0:**40 X** vs. 12:**0 X**	0.005333	-0.1386 to 0.1493	No
825	0:**40 X** vs. 12:**1 X**	0.0230	-0.1210 to 0.1670	No
826	0:**40 X** vs. 12:**10 X**	0.005333	-0.1386 to 0.1493	No
827	0:**40 X** vs. 12:**40 X**	0.0190	-0.1250 to 0.1630	No
828	0:**40 X** vs. 12:**50 X**	0.01567	-0.1283 to 0.1596	No
829	0:**40 X** vs. 13:**0 X**	0.01133	-0.1326 to 0.1553	No
830	0:**40 X** vs. 13:**1 X**	0.0240	-0.1200 to 0.1680	No
831	0:**40 X** vs. 13:**10 X**	0.0020	-0.1420 to 0.1460	No
832	0:**40 X** vs. 13:**40 X**	0.005667	-0.1383 to 0.1496	No
833	0:**40 X** vs. 13:**50 X**	0.004667	-0.1393 to 0.1486	No
834	0:**40 X** vs. 14:**0 X**	0.002667	-0.1413 to 0.1466	No
835	0:**40 X** vs. 14:**1 X**	0.0110	-0.1330 to 0.1550	No
836	0:**40 X** vs. 14:**10 X**	-0.0030	-0.1470 to 0.1410	No
837	0:**40 X** vs. 14:**40 X**	0.0003333	-0.1436 to 0.1443	No
838	0:**40 X** vs. 14:**50 X**	-0.003667	-0.1476 to 0.1403	No
839	0:**40 X** vs. 15:**0 X**	-0.009667	-0.1536 to 0.1343	No
840	0:**40 X** vs. 15:**1 X**	0.01033	-0.1336 to 0.1543	No
841	0:**40 X** vs. 15:**10 X**	-0.0170	-0.1610 to 0.1270	No
842	0:**40 X** vs. 15:**40 X**	0.0006667	-0.1433 to 0.1446	No
843	0:**40 X** vs. 15:**50 X**	-0.0170	-0.1610 to 0.1270	No
844	0:**40 X** vs. 16:**0 X**	-0.1020	-0.2460 to 0.04196	No
845	0:**40 X** vs. 16:**1 X**	0.007000	-0.1370 to 0.1510	No
846	0:**40 X** vs. 16:**10 X**	-0.01867	-0.1626 to 0.1253	No
847	0:**40 X** vs. 16:**40 X**	-0.0380	-0.1820 to 0.1060	No
848	0:**40 X** vs. 16:**50 X**	-0.08033	-0.2243 to 0.06363	No
849	0:**40 X** vs. 17:**0 X**	-0.1547	-0.2986 to -0.01071	Yes
850	0:**40 X** vs. 17:**1 X**	-0.06900	-0.2130 to 0.07496	No
851	0:**40 X** vs. 17:**10 X**	-0.1257	-0.2696 to 0.01829	No
852	0:**40 X** vs. 17:**40 X**	-0.0490	-0.1930 to 0.09496	No
853	0:**40 X** vs. 17:**50 X**	-0.09433	-0.2383 to 0.04963	No
854	0:**40 X** vs. 18:**0 X**	-0.2033	-0.3473 to -0.05937	Yes
855	0:**40 X** vs. 18:**1 X**	-0.1117	-0.2556 to 0.03229	No

	2way ANOVA<br>Multiple comparisons	A<br>Data Set-A<br>Y	B<br>Data Set-B<br>Y	C<br>Data Set-C<br>Y
856	0:**40 X** vs. 18:**10 X**	-0.2040	-0.3480 to -0.06004	Yes
857	0:**40 X** vs. 18:**40 X**	-0.05333	-0.1973 to 0.09063	No
858	0:**40 X** vs. 18:**50 X**	-0.09833	-0.2423 to 0.04563	No
859	0:**40 X** vs. 19:**0 X**	-0.2383	-0.3823 to -0.09437	Yes
860	0:**40 X** vs. 19:**1 X**	-0.2263	-0.3703 to -0.08237	Yes
861	0:**40 X** vs. 19:**10 X**	-0.2240	-0.3680 to -0.08004	Yes
862	0:**40 X** vs. 19:**40 X**	-0.0820	-0.2260 to 0.06196	No
863	0:**40 X** vs. 19:**50 X**	-0.0930	-0.2370 to 0.05096	No
864	0:**40 X** vs. 20:**0 X**	-0.3180	-0.4620 to -0.1740	Yes
865	0:**40 X** vs. 20:**1 X**	-0.1373	-0.2813 to 0.006626	No
866	0:**40 X** vs. 20:**10 X**	-0.2263	-0.3703 to -0.08237	Yes
867	0:**40 X** vs. 20:**40 X**	-0.09367	-0.2376 to 0.05029	No
868	0:**40 X** vs. 20:**50 X**	-0.1377	-0.2816 to 0.006293	No
869	0:**40 X** vs. 21:**0 X**	-0.3413	-0.4853 to -0.1974	Yes
870	0:**40 X** vs. 21:**1 X**	-0.2943	-0.4383 to -0.1504	Yes
871	0:**40 X** vs. 21:**10 X**	-0.2913	-0.4353 to -0.1474	Yes
872	0:**40 X** vs. 21:**40 X**	-0.1667	-0.3106 to -0.02271	Yes
873	0:**40 X** vs. 21:**50 X**	-0.2570	-0.4010 to -0.1130	Yes
874	0:**40 X** vs. 22:**0 X**	-0.3830	-0.5270 to -0.2390	Yes
875	0:**40 X** vs. 22:**1 X**	-0.3777	-0.5216 to -0.2337	Yes
876	0:**40 X** vs. 22:**10 X**	-0.3730	-0.5170 to -0.2290	Yes
877	0:**40 X** vs. 22:**40 X**	-0.2733	-0.4173 to -0.1294	Yes
878	0:**40 X** vs. 22:**50 X**	-0.3360	-0.4800 to -0.1920	Yes
879	0:**40 X** vs. 23:**0 X**	-0.4483	-0.5923 to -0.3044	Yes
880	0:**40 X** vs. 23:**1 X**	-0.6380	-0.7990 to -0.4770	Yes
881	0:**40 X** vs. 23:**10 X**	-0.3877	-0.5316 to -0.2437	Yes
882	0:**40 X** vs. 23:**40 X**	-0.2680	-0.4290 to -0.1070	Yes
883	0:**40 X** vs. 23:**50 X**	-0.3020	-0.4460 to -0.1580	Yes
884	0:**40 X** vs. 24:**0 X**	-0.5657	-0.7096 to -0.4217	Yes
885	0:**40 X** vs. 24:**1 X**	-0.7220	-0.8830 to -0.5610	Yes
886	0:**40 X** vs. 24:**10 X**	-0.5495	-0.7105 to -0.3885	Yes
887	0:**40 X** vs. 24:**40 X**	-0.2843	-0.4283 to -0.1404	Yes
888	0:**40 X** vs. 24:**50 X**	-0.2060	-0.3500 to -0.06204	Yes
889	0:**40 X** vs. 25:**0 X**	-0.9030	-1.064 to -0.7420	Yes
890	0:**40 X** vs. 25:**1 X**	-1.093	-1.253 to -0.9315	Yes
891	0:**40 X** vs. 25:**10 X**	-0.4540	-0.6576 to -0.2504	Yes
892	0:**40 X** vs. 25:**40 X**	-0.2863	-0.4303 to -0.1424	Yes
893	0:**40 X** vs. 25:**50 X**	-0.2607	-0.4046 to -0.1167	Yes
894	0:**40 X** vs. 26:**0 X**	-1.116	-1.276 to -0.9545	Yes
895	0:**40 X** vs. 26:**1 X**	-1.229	-1.389 to -1.068	Yes
896	0:**40 X** vs. 26:**10 X**	-0.9150	-1.076 to -0.7540	Yes
897	0:**40 X** vs. 26:**40 X**	-0.3607	-0.5046 to -0.2167	Yes
898	0:**40 X** vs. 26:**50 X**	-0.2210	-0.3650 to -0.07704	Yes
899	0:**40 X** vs. 27:**0 X**	-1.303	-1.447 to -1.159	Yes
900	0:**40 X** vs. 27:**1 X**	-1.394	-1.555 to -1.233	Yes

	2way ANOVA<br>Multiple comparisons	A<br>Data Set-A<br>Y	B<br>Data Set-B<br>Y	C<br>Data Set-C<br>Y
901	0:40 X vs. 27:10 X	-1.206	-1.367 to -1.045	Yes
902	0:40 X vs. 27:40 X	-0.3607	-0.5046 to -0.2167	Yes
903	0:40 X vs. 27:50 X	-0.2203	-0.3643 to -0.07637	Yes
904	0:40 X vs. 28:0 X	-1.253	-1.413 to -1.092	Yes
905	0:40 X vs. 28:1 X	-1.293	-1.437 to -1.149	Yes
906	0:40 X vs. 28:10 X	-1.095	-1.256 to -0.9340	Yes
907	0:40 X vs. 28:40 X	-0.3730	-0.5170 to -0.2290	Yes
908	0:40 X vs. 28:50 X	-0.2683	-0.4123 to -0.1244	Yes
909	0:40 X vs. 29:0 X	-1.734	-1.895 to -1.573	Yes
910	0:40 X vs. 29:1 X	-1.819	-1.980 to -1.658	Yes
911	0:40 X vs. 29:10 X	-1.034	-1.194 to -0.8725	Yes
912	0:40 X vs. 29:40 X	-0.4810	-0.6250 to -0.3370	Yes
913	0:40 X vs. 29:50 X	-0.2697	-0.4136 to -0.1257	Yes
914	0:40 X vs. 30:0 X	-1.449	-1.609 to -1.288	Yes
915	0:40 X vs. 30:1 X	-1.770	-1.931 to -1.609	Yes
916	0:40 X vs. 30:10 X	-1.108	-1.269 to -0.9470	Yes
917	0:40 X vs. 30:40 X	-0.4080	-0.5520 to -0.2640	Yes
918	0:40 X vs. 30:50 X	-0.3220	-0.4660 to -0.1780	Yes
919	0:40 X vs. 31:0 X	-1.767	-1.927 to -1.606	Yes
920	0:40 X vs. 31:1 X	-1.757	-1.918 to -1.596	Yes
921	0:40 X vs. 31:10 X	-1.240	-1.400 to -1.079	Yes
922	0:40 X vs. 31:40 X	-0.4997	-0.6436 to -0.3557	Yes
923	0:40 X vs. 31:50 X	-0.3010	-0.4450 to -0.1570	Yes
924	0:40 X vs. 32:0 X	-2.358	-2.502 to -2.214	Yes
925	0:40 X vs. 32:1 X	-1.814	-1.975 to -1.653	Yes
926	0:40 X vs. 32:10 X	-1.670	-1.814 to -1.526	Yes
927	0:40 X vs. 32:40 X	-0.4960	-0.6400 to -0.3520	Yes
928	0:40 X vs. 32:50 X	-0.3007	-0.4446 to -0.1567	Yes
929	0:40 X vs. 33:0 X	-2.362	-2.506 to -2.218	Yes
930	0:40 X vs. 33:1 X	-2.176	-2.320 to -2.032	Yes
931	0:40 X vs. 33:10 X	-1.837	-1.981 to -1.693	Yes
932	0:40 X vs. 33:40 X	-0.5147	-0.6586 to -0.3707	Yes
933	0:40 X vs. 33:50 X	-0.3157	-0.4596 to -0.1717	Yes
934	0:40 X vs. 34:0 X	-2.286	-2.430 to -2.142	Yes
935	0:40 X vs. 34:1 X	-2.294	-2.438 to -2.150	Yes
936	0:40 X vs. 34:10 X	-1.927	-2.071 to -1.783	Yes
937	0:40 X vs. 34:40 X	-0.5267	-0.6706 to -0.3827	Yes
938	0:40 X vs. 34:50 X	-0.4647	-0.6086 to -0.3207	Yes
939	0:40 X vs. 35:0 X	-2.635	-2.779 to -2.491	Yes
940	0:40 X vs. 35:1 X	-2.401	-2.545 to -2.257	Yes
941	0:40 X vs. 35:10 X	-1.889	-2.033 to -1.745	Yes
942	0:40 X vs. 35:40 X	-0.5357	-0.6796 to -0.3917	Yes
943	0:40 X vs. 35:50 X	-0.4773	-0.6213 to -0.3334	Yes
944	0:40 X vs. 36:0 X	-2.842	-3.002 to -2.681	Yes
945	0:40 X vs. 36:1 X	-2.537	-2.681 to -2.393	Yes

	2way ANOVA<br>Multiple comparisons	A<br>Data Set-A<br>Y	B<br>Data Set-B<br>Y	C<br>Data Set-C<br>Y
946	0:40 X vs. 36:10 X	-1.894	-2.038 to -1.750	Yes
947	0:40 X vs. 36:40 X	-0.5910	-0.7350 to -0.4470	Yes
948	0:40 X vs. 36:50 X	-0.4407	-0.5846 to -0.2967	Yes
949	0:40 X vs. 37:0 X	-3.225	-3.369 to -3.081	Yes
950	0:40 X vs. 37:1 X	-2.607	-2.751 to -2.463	Yes
951	0:40 X vs. 37:10 X	-1.896	-2.040 to -1.752	Yes
952	0:40 X vs. 37:40 X	-0.6283	-0.7723 to -0.4844	Yes
953	0:40 X vs. 37:50 X	-0.4493	-0.5933 to -0.3054	Yes
954	0:40 X vs. 38:0 X	-3.170	-3.314 to -3.026	Yes
955	0:40 X vs. 38:1 X	-2.726	-2.870 to -2.582	Yes
956	0:40 X vs. 38:10 X	-2.171	-2.315 to -2.027	Yes
957	0:40 X vs. 38:40 X	-0.6327	-0.7766 to -0.4887	Yes
958	0:40 X vs. 38:50 X	-0.4533	-0.5973 to -0.3094	Yes
959	0:40 X vs. 39:0 X	-3.401	-3.545 to -3.257	Yes
960	0:40 X vs. 39:1 X	-3.293	-3.437 to -3.149	Yes
961	0:40 X vs. 39:10 X	-2.316	-2.460 to -2.172	Yes
962	0:40 X vs. 39:40 X	-0.6387	-0.7826 to -0.4947	Yes
963	0:40 X vs. 39:50 X	-0.4843	-0.6283 to -0.3404	Yes
964	0:40 X vs. 40:0 X	-3.442	-3.586 to -3.298	Yes
965	0:40 X vs. 40:1 X	-3.368	-3.512 to -3.224	Yes
966	0:40 X vs. 40:10 X	-2.573	-2.717 to -2.429	Yes
967	0:40 X vs. 40:40 X	-0.6433	-0.7873 to -0.4994	Yes
968	0:40 X vs. 40:50 X	-0.4907	-0.6346 to -0.3467	Yes
969	0:40 X vs. 41:0 X	-3.483	-3.627 to -3.339	Yes
970	0:40 X vs. 41:1 X	-3.482	-3.626 to -3.338	Yes
971	0:40 X vs. 41:10 X	-2.626	-2.770 to -2.482	Yes
972	0:40 X vs. 41:40 X	-0.5907	-0.7346 to -0.4467	Yes
973	0:40 X vs. 41:50 X	-0.5033	-0.6473 to -0.3594	Yes
974	0:40 X vs. 42:0 X	-3.625	-3.769 to -3.481	Yes
975	0:40 X vs. 42:1 X	-3.644	-3.788 to -3.500	Yes
976	0:40 X vs. 42:10 X	-2.627	-2.771 to -2.483	Yes
977	0:40 X vs. 42:40 X	-0.6033	-0.7473 to -0.4594	Yes
978	0:40 X vs. 42:50 X	-0.5067	-0.6506 to -0.3627	Yes
979	0:40 X vs. 43:0 X	-3.715	-3.859 to -3.571	Yes
980	0:40 X vs. 43:1 X	-3.629	-3.773 to -3.485	Yes
981	0:40 X vs. 43:10 X	-3.345	-3.489 to -3.201	Yes
982	0:40 X vs. 43:40 X	-0.6017	-0.7456 to -0.4577	Yes
983	0:40 X vs. 43:50 X	-0.5070	-0.6510 to -0.3630	Yes
984	0:40 X vs. 44:0 X	-3.739	-3.883 to -3.595	Yes
985	0:40 X vs. 44:1 X	-3.628	-3.772 to -3.484	Yes
986	0:40 X vs. 44:10 X	-3.408	-3.552 to -3.264	Yes
987	0:40 X vs. 44:40 X	-0.6037	-0.7476 to -0.4597	Yes
988	0:40 X vs. 44:50 X	-0.4393	-0.5833 to -0.2954	Yes
989	0:40 X vs. 45:0 X	-3.603	-3.747 to -3.459	Yes
990	0:40 X vs. 45:1 X	-3.405	-3.549 to -3.261	Yes

	2way ANOVA<br>Multiple comparisons	A<br>Data Set-A<br>Y	B<br>Data Set-B<br>Y	C<br>Data Set-C<br>Y
991	0:40 X vs. 45:10 X	-3.288	-3.432 to -3.144	Yes
992	0:40 X vs. 45:40 X	-0.6050	-0.7490 to -0.4610	Yes
993	0:40 X vs. 45:50 X	-0.4363	-0.5803 to -0.2924	Yes
994	0:40 X vs. 46:0 X	-3.408	-3.552 to -3.264	Yes
995	0:40 X vs. 46:1 X	-3.375	-3.519 to -3.231	Yes
996	0:40 X vs. 46:10 X	-3.283	-3.427 to -3.139	Yes
997	0:40 X vs. 46:40 X	-0.6187	-0.7626 to -0.4747	Yes
998	0:40 X vs. 46:50 X	-0.4293	-0.5733 to -0.2854	Yes
999	0:40 X vs. 47:0 X	-3.383	-3.527 to -3.239	Yes
1000	0:40 X vs. 47:1 X	-3.287	-3.431 to -3.143	Yes
1001	0:40 X vs. 47:10 X	-3.269	-3.413 to -3.125	Yes
1002	0:40 X vs. 47:40 X	-0.6287	-0.7726 to -0.4847	Yes
1003	0:40 X vs. 47:50 X	-0.3873	-0.5313 to -0.2434	Yes
1004	0:40 X vs. 48:0 X	-3.371	-3.531 to -3.210	Yes
1005	0:40 X vs. 48:1 X	-3.274	-3.418 to -3.130	Yes
1006	0:40 X vs. 48:10 X	-3.269	-3.413 to -3.125	Yes
1007	0:40 X vs. 48:40 X	-0.6350	-0.7790 to -0.4910	Yes
1008	0:40 X vs. 48:50 X	-0.3947	-0.5386 to -0.2507	Yes
1009	0:40 X vs. 49:0 X	-3.371	-3.515 to -3.227	Yes
1010	0:40 X vs. 49:1 X	-3.274	-3.418 to -3.130	Yes
1011	0:40 X vs. 49:10 X	-3.269	-3.413 to -3.125	Yes
1012	0:40 X vs. 49:40 X	-0.6393	-0.7833 to -0.4954	Yes
1013	0:40 X vs. 49:50 X	-0.3940	-0.5380 to -0.2500	Yes
1014	0:40 X vs. 50:0 X	-3.371	-3.515 to -3.227	Yes
1015	0:40 X vs. 50:1 X	-3.274	-3.418 to -3.130	Yes
1016	0:40 X vs. 50:10 X	-3.269	-3.413 to -3.125	Yes
1017	0:40 X vs. 50:40 X	-0.6470	-0.7910 to -0.5030	Yes
1018	0:40 X vs. 50:50 X	-0.3937	-0.5376 to -0.2497	Yes
1019	0:50 X vs. 1:0 X	0.04233	-0.1016 to 0.1863	No
1020	0:50 X vs. 1:1 X	0.03467	-0.1093 to 0.1786	No
1021	0:50 X vs. 1:10 X	0.0440	-0.09996 to 0.1880	No
1022	0:50 X vs. 1:40 X	0.03833	-0.1056 to 0.1823	No
1023	0:50 X vs. 1:50 X	0.0410	-0.1030 to 0.1850	No
1024	0:50 X vs. 2:0 X	0.03767	-0.1063 to 0.1816	No
1025	0:50 X vs. 2:1 X	0.03267	-0.1113 to 0.1766	No
1026	0:50 X vs. 2:10 X	0.03867	-0.1053 to 0.1826	No
1027	0:50 X vs. 2:40 X	0.0380	-0.1060 to 0.1820	No
1028	0:50 X vs. 2:50 X	0.0410	-0.1030 to 0.1850	No
1029	0:50 X vs. 3:0 X	0.03667	-0.1073 to 0.1806	No
1030	0:50 X vs. 3:1 X	0.03433	-0.1096 to 0.1783	No
1031	0:50 X vs. 3:10 X	0.0390	-0.1050 to 0.1830	No
1032	0:50 X vs. 3:40 X	0.04133	-0.1026 to 0.1853	No
1033	0:50 X vs. 3:50 X	0.04233	-0.1016 to 0.1863	No
1034	0:50 X vs. 4:0 X	0.03233	-0.1116 to 0.1763	No
1035	0:50 X vs. 4:1 X	0.03367	-0.1103 to 0.1776	No

	2way ANOVA<br>Multiple comparisons	A<br>Data Set-A<br>Y	B<br>Data Set-B<br>Y	C<br>Data Set-C<br>Y
1036	0:**50 X** vs. 4:**10 X**	0.0190	-0.1250 to 0.1630	No
1037	0:**50 X** vs. 4:**40 X**	0.03933	-0.1046 to 0.1833	No
1038	0:**50 X** vs. 4:**50 X**	0.04133	-0.1026 to 0.1853	No
1039	0:**50 X** vs. 5:**0 X**	0.03133	-0.1126 to 0.1753	No
1040	0:**50 X** vs. 5:**1 X**	0.03333	-0.1106 to 0.1773	No
1041	0:**50 X** vs. 5:**10 X**	0.01833	-0.1256 to 0.1623	No
1042	0:**50 X** vs. 5:**40 X**	0.03933	-0.1046 to 0.1833	No
1043	0:**50 X** vs. 5:**50 X**	0.04033	-0.1036 to 0.1843	No
1044	0:**50 X** vs. 6:**0 X**	0.0320	-0.1120 to 0.1760	No
1045	0:**50 X** vs. 6:**1 X**	0.0330	-0.1110 to 0.1770	No
1046	0:**50 X** vs. 6:**10 X**	0.01833	-0.1256 to 0.1623	No
1047	0:**50 X** vs. 6:**40 X**	0.0370	-0.1070 to 0.1810	No
1048	0:**50 X** vs. 6:**50 X**	0.03867	-0.1053 to 0.1826	No
1049	0:**50 X** vs. 7:**0 X**	0.0300	-0.1140 to 0.1740	No
1050	0:**50 X** vs. 7:**1 X**	0.03867	-0.1053 to 0.1826	No
1051	0:**50 X** vs. 7:**10 X**	0.02667	-0.1173 to 0.1706	No
1052	0:**50 X** vs. 7:**40 X**	0.03533	-0.1086 to 0.1793	No
1053	0:**50 X** vs. 7:**50 X**	0.03733	-0.1066 to 0.1813	No
1054	0:**50 X** vs. 8:**0 X**	0.02533	-0.1186 to 0.1693	No
1055	0:**50 X** vs. 8:**1 X**	0.03867	-0.1053 to 0.1826	No
1056	0:**50 X** vs. 8:**10 X**	0.03133	-0.1126 to 0.1753	No
1057	0:**50 X** vs. 8:**40 X**	0.03067	-0.1133 to 0.1746	No
1058	0:**50 X** vs. 8:**50 X**	0.03367	-0.1103 to 0.1776	No
1059	0:**50 X** vs. 9:**0 X**	0.01733	-0.1266 to 0.1613	No
1060	0:**50 X** vs. 9:**1 X**	0.009333	-0.1346 to 0.1533	No
1061	0:**50 X** vs. 9:**10 X**	0.008333	-0.1356 to 0.1523	No
1062	0:**50 X** vs. 9:**40 X**	0.0260	-0.1180 to 0.1700	No
1063	0:**50 X** vs. 9:**50 X**	0.03267	-0.1113 to 0.1766	No
1064	0:**50 X** vs. 10:**0 X**	0.01567	-0.1283 to 0.1596	No
1065	0:**50 X** vs. 10:**1 X**	0.008667	-0.1353 to 0.1526	No
1066	0:**50 X** vs. 10:**10 X**	0.0060	-0.1380 to 0.1500	No
1067	0:**50 X** vs. 10:**40 X**	0.01633	-0.1276 to 0.1603	No
1068	0:**50 X** vs. 10:**50 X**	0.02967	-0.1143 to 0.1736	No
1069	0:**50 X** vs. 11:**0 X**	0.0060	-0.1380 to 0.1500	No
1070	0:**50 X** vs. 11:**1 X**	0.004333	-0.1396 to 0.1483	No
1071	0:**50 X** vs. 11:**10 X**	0.004000	-0.1400 to 0.1480	No
1072	0:**50 X** vs. 11:**40 X**	0.01233	-0.1316 to 0.1563	No
1073	0:**50 X** vs. 11:**50 X**	0.02433	-0.1196 to 0.1683	No
1074	0:**50 X** vs. 12:**0 X**	0.005333	-0.1386 to 0.1493	No
1075	0:**50 X** vs. 12:**1 X**	0.0230	-0.1210 to 0.1670	No
1076	0:**50 X** vs. 12:**10 X**	0.005333	-0.1386 to 0.1493	No
1077	0:**50 X** vs. 12:**40 X**	0.0190	-0.1250 to 0.1630	No
1078	0:**50 X** vs. 12:**50 X**	0.01567	-0.1283 to 0.1596	No
1079	0:**50 X** vs. 13:**0 X**	0.01133	-0.1326 to 0.1553	No
1080	0:**50 X** vs. 13:**1 X**	0.0240	-0.1200 to 0.1680	No

	2way ANOVA<br>Multiple comparisons	A<br>Data Set-A<br>Y	B<br>Data Set-B<br>Y	C<br>Data Set-C<br>Y
1081	0:50 X vs. 13:10 X	0.0020	-0.1420 to 0.1460	No
1082	0:50 X vs. 13:40 X	0.005667	-0.1383 to 0.1496	No
1083	0:50 X vs. 13:50 X	0.004667	-0.1393 to 0.1486	No
1084	0:50 X vs. 14:0 X	0.002667	-0.1413 to 0.1466	No
1085	0:50 X vs. 14:1 X	0.0110	-0.1330 to 0.1550	No
1086	0:50 X vs. 14:10 X	-0.0030	-0.1470 to 0.1410	No
1087	0:50 X vs. 14:40 X	0.0003333	-0.1436 to 0.1443	No
1088	0:50 X vs. 14:50 X	-0.003667	-0.1476 to 0.1403	No
1089	0:50 X vs. 15:0 X	-0.009667	-0.1536 to 0.1343	No
1090	0:50 X vs. 15:1 X	0.01033	-0.1336 to 0.1543	No
1091	0:50 X vs. 15:10 X	-0.0170	-0.1610 to 0.1270	No
1092	0:50 X vs. 15:40 X	0.0006667	-0.1433 to 0.1446	No
1093	0:50 X vs. 15:50 X	-0.0170	-0.1610 to 0.1270	No
1094	0:50 X vs. 16:0 X	-0.1020	-0.2460 to 0.04196	No
1095	0:50 X vs. 16:1 X	0.007000	-0.1370 to 0.1510	No
1096	0:50 X vs. 16:10 X	-0.01867	-0.1626 to 0.1253	No
1097	0:50 X vs. 16:40 X	-0.0380	-0.1820 to 0.1060	No
1098	0:50 X vs. 16:50 X	-0.08033	-0.2243 to 0.06363	No
1099	0:50 X vs. 17:0 X	-0.1547	-0.2986 to -0.01071	Yes
1100	0:50 X vs. 17:1 X	-0.06900	-0.2130 to 0.07496	No
1101	0:50 X vs. 17:10 X	-0.1257	-0.2696 to 0.01829	No
1102	0:50 X vs. 17:40 X	-0.0490	-0.1930 to 0.09496	No
1103	0:50 X vs. 17:50 X	-0.09433	-0.2383 to 0.04963	No
1104	0:50 X vs. 18:0 X	-0.2033	-0.3473 to -0.05937	Yes
1105	0:50 X vs. 18:1 X	-0.1117	-0.2556 to 0.03229	No
1106	0:50 X vs. 18:10 X	-0.2040	-0.3480 to -0.06004	Yes
1107	0:50 X vs. 18:40 X	-0.05333	-0.1973 to 0.09063	No
1108	0:50 X vs. 18:50 X	-0.09833	-0.2423 to 0.04563	No
1109	0:50 X vs. 19:0 X	-0.2383	-0.3823 to -0.09437	Yes
1110	0:50 X vs. 19:1 X	-0.2263	-0.3703 to -0.08237	Yes
1111	0:50 X vs. 19:10 X	-0.2240	-0.3680 to -0.08004	Yes
1112	0:50 X vs. 19:40 X	-0.0820	-0.2260 to 0.06196	No
1113	0:50 X vs. 19:50 X	-0.0930	-0.2370 to 0.05096	No
1114	0:50 X vs. 20:0 X	-0.3180	-0.4620 to -0.1740	Yes
1115	0:50 X vs. 20:1 X	-0.1373	-0.2813 to 0.006626	No
1116	0:50 X vs. 20:10 X	-0.2263	-0.3703 to -0.08237	Yes
1117	0:50 X vs. 20:40 X	-0.09367	-0.2376 to 0.05029	No
1118	0:50 X vs. 20:50 X	-0.1377	-0.2816 to 0.006293	No
1119	0:50 X vs. 21:0 X	-0.3413	-0.4853 to -0.1974	Yes
1120	0:50 X vs. 21:1 X	-0.2943	-0.4383 to -0.1504	Yes
1121	0:50 X vs. 21:10 X	-0.2913	-0.4353 to -0.1474	Yes
1122	0:50 X vs. 21:40 X	-0.1667	-0.3106 to -0.02271	Yes
1123	0:50 X vs. 21:50 X	-0.2570	-0.4010 to -0.1130	Yes
1124	0:50 X vs. 22:0 X	-0.3830	-0.5270 to -0.2390	Yes
1125	0:50 X vs. 22:1 X	-0.3777	-0.5216 to -0.2337	Yes

	2way ANOVA<br>Multiple comparisons	A<br>Data Set-A<br>Y	B<br>Data Set-B<br>Y	C<br>Data Set-C<br>Y
**1126**	0:**50 X** vs. 22:**10 X**	-0.3730	-0.5170 to -0.2290	Yes
**1127**	0:**50 X** vs. 22:**40 X**	-0.2733	-0.4173 to -0.1294	Yes
**1128**	0:**50 X** vs. 22:**50 X**	-0.3360	-0.4800 to -0.1920	Yes
**1129**	0:**50 X** vs. 23:**0 X**	-0.4483	-0.5923 to -0.3044	Yes
**1130**	0:**50 X** vs. 23:**1 X**	-0.6380	-0.7990 to -0.4770	Yes
**1131**	0:**50 X** vs. 23:**10 X**	-0.3877	-0.5316 to -0.2437	Yes
**1132**	0:**50 X** vs. 23:**40 X**	-0.2680	-0.4290 to -0.1070	Yes
**1133**	0:**50 X** vs. 23:**50 X**	-0.3020	-0.4460 to -0.1580	Yes
**1134**	0:**50 X** vs. 24:**0 X**	-0.5657	-0.7096 to -0.4217	Yes
**1135**	0:**50 X** vs. 24:**1 X**	-0.7220	-0.8830 to -0.5610	Yes
**1136**	0:**50 X** vs. 24:**10 X**	-0.5495	-0.7105 to -0.3885	Yes
**1137**	0:**50 X** vs. 24:**40 X**	-0.2843	-0.4283 to -0.1404	Yes
**1138**	0:**50 X** vs. 24:**50 X**	-0.2060	-0.3500 to -0.06204	Yes
**1139**	0:**50 X** vs. 25:**0 X**	-0.9030	-1.064 to -0.7420	Yes
**1140**	0:**50 X** vs. 25:**1 X**	-1.093	-1.253 to -0.9315	Yes
**1141**	0:**50 X** vs. 25:**10 X**	-0.4540	-0.6576 to -0.2504	Yes
**1142**	0:**50 X** vs. 25:**40 X**	-0.2863	-0.4303 to -0.1424	Yes
**1143**	0:**50 X** vs. 25:**50 X**	-0.2607	-0.4046 to -0.1167	Yes
**1144**	0:**50 X** vs. 26:**0 X**	-1.116	-1.276 to -0.9545	Yes
**1145**	0:**50 X** vs. 26:**1 X**	-1.229	-1.389 to -1.068	Yes
**1146**	0:**50 X** vs. 26:**10 X**	-0.9150	-1.076 to -0.7540	Yes
**1147**	0:**50 X** vs. 26:**40 X**	-0.3607	-0.5046 to -0.2167	Yes
**1148**	0:**50 X** vs. 26:**50 X**	-0.2210	-0.3650 to -0.07704	Yes
**1149**	0:**50 X** vs. 27:**0 X**	-1.303	-1.447 to -1.159	Yes
**1150**	0:**50 X** vs. 27:**1 X**	-1.394	-1.555 to -1.233	Yes
**1151**	0:**50 X** vs. 27:**10 X**	-1.206	-1.367 to -1.045	Yes
**1152**	0:**50 X** vs. 27:**40 X**	-0.3607	-0.5046 to -0.2167	Yes
**1153**	0:**50 X** vs. 27:**50 X**	-0.2203	-0.3643 to -0.07637	Yes
**1154**	0:**50 X** vs. 28:**0 X**	-1.253	-1.413 to -1.092	Yes
**1155**	0:**50 X** vs. 28:**1 X**	-1.293	-1.437 to -1.149	Yes
**1156**	0:**50 X** vs. 28:**10 X**	-1.095	-1.256 to -0.9340	Yes
**1157**	0:**50 X** vs. 28:**40 X**	-0.3730	-0.5170 to -0.2290	Yes
**1158**	0:**50 X** vs. 28:**50 X**	-0.2683	-0.4123 to -0.1244	Yes
**1159**	0:**50 X** vs. 29:**0 X**	-1.734	-1.895 to -1.573	Yes
**1160**	0:**50 X** vs. 29:**1 X**	-1.819	-1.980 to -1.658	Yes
**1161**	0:**50 X** vs. 29:**10 X**	-1.034	-1.194 to -0.8725	Yes
**1162**	0:**50 X** vs. 29:**40 X**	-0.4810	-0.6250 to -0.3370	Yes
**1163**	0:**50 X** vs. 29:**50 X**	-0.2697	-0.4136 to -0.1257	Yes
**1164**	0:**50 X** vs. 30:**0 X**	-1.449	-1.609 to -1.288	Yes
**1165**	0:**50 X** vs. 30:**1 X**	-1.770	-1.931 to -1.609	Yes
**1166**	0:**50 X** vs. 30:**10 X**	-1.108	-1.269 to -0.9470	Yes
**1167**	0:**50 X** vs. 30:**40 X**	-0.4080	-0.5520 to -0.2640	Yes
**1168**	0:**50 X** vs. 30:**50 X**	-0.3220	-0.4660 to -0.1780	Yes
**1169**	0:**50 X** vs. 31:**0 X**	-1.767	-1.927 to -1.606	Yes
**1170**	0:**50 X** vs. 31:**1 X**	-1.757	-1.918 to -1.596	Yes

	2way ANOVA<br>Multiple comparisons	A<br>Data Set-A<br>Y	B<br>Data Set-B<br>Y	C<br>Data Set-C<br>Y
1171	0:50 X vs. 31:10 X	-1.240	-1.400 to -1.079	Yes
1172	0:50 X vs. 31:40 X	-0.4997	-0.6436 to -0.3557	Yes
1173	0:50 X vs. 31:50 X	-0.3010	-0.4450 to -0.1570	Yes
1174	0:50 X vs. 32:0 X	-2.358	-2.502 to -2.214	Yes
1175	0:50 X vs. 32:1 X	-1.814	-1.975 to -1.653	Yes
1176	0:50 X vs. 32:10 X	-1.670	-1.814 to -1.526	Yes
1177	0:50 X vs. 32:40 X	-0.4960	-0.6400 to -0.3520	Yes
1178	0:50 X vs. 32:50 X	-0.3007	-0.4446 to -0.1567	Yes
1179	0:50 X vs. 33:0 X	-2.362	-2.506 to -2.218	Yes
1180	0:50 X vs. 33:1 X	-2.176	-2.320 to -2.032	Yes
1181	0:50 X vs. 33:10 X	-1.837	-1.981 to -1.693	Yes
1182	0:50 X vs. 33:40 X	-0.5147	-0.6586 to -0.3707	Yes
1183	0:50 X vs. 33:50 X	-0.3157	-0.4596 to -0.1717	Yes
1184	0:50 X vs. 34:0 X	-2.286	-2.430 to -2.142	Yes
1185	0:50 X vs. 34:1 X	-2.294	-2.438 to -2.150	Yes
1186	0:50 X vs. 34:10 X	-1.927	-2.071 to -1.783	Yes
1187	0:50 X vs. 34:40 X	-0.5267	-0.6706 to -0.3827	Yes
1188	0:50 X vs. 34:50 X	-0.4647	-0.6086 to -0.3207	Yes
1189	0:50 X vs. 35:0 X	-2.635	-2.779 to -2.491	Yes
1190	0:50 X vs. 35:1 X	-2.401	-2.545 to -2.257	Yes
1191	0:50 X vs. 35:10 X	-1.889	-2.033 to -1.745	Yes
1192	0:50 X vs. 35:40 X	-0.5357	-0.6796 to -0.3917	Yes
1193	0:50 X vs. 35:50 X	-0.4773	-0.6213 to -0.3334	Yes
1194	0:50 X vs. 36:0 X	-2.842	-3.002 to -2.681	Yes
1195	0:50 X vs. 36:1 X	-2.537	-2.681 to -2.393	Yes
1196	0:50 X vs. 36:10 X	-1.894	-2.038 to -1.750	Yes
1197	0:50 X vs. 36:40 X	-0.5910	-0.7350 to -0.4470	Yes
1198	0:50 X vs. 36:50 X	-0.4407	-0.5846 to -0.2967	Yes
1199	0:50 X vs. 37:0 X	-3.225	-3.369 to -3.081	Yes
1200	0:50 X vs. 37:1 X	-2.607	-2.751 to -2.463	Yes
1201	0:50 X vs. 37:10 X	-1.896	-2.040 to -1.752	Yes
1202	0:50 X vs. 37:40 X	-0.6283	-0.7723 to -0.4844	Yes
1203	0:50 X vs. 37:50 X	-0.4493	-0.5933 to -0.3054	Yes
1204	0:50 X vs. 38:0 X	-3.170	-3.314 to -3.026	Yes
1205	0:50 X vs. 38:1 X	-2.726	-2.870 to -2.582	Yes
1206	0:50 X vs. 38:10 X	-2.171	-2.315 to -2.027	Yes
1207	0:50 X vs. 38:40 X	-0.6327	-0.7766 to -0.4887	Yes
1208	0:50 X vs. 38:50 X	-0.4533	-0.5973 to -0.3094	Yes
1209	0:50 X vs. 39:0 X	-3.401	-3.545 to -3.257	Yes
1210	0:50 X vs. 39:1 X	-3.293	-3.437 to -3.149	Yes
1211	0:50 X vs. 39:10 X	-2.316	-2.460 to -2.172	Yes
1212	0:50 X vs. 39:40 X	-0.6387	-0.7826 to -0.4947	Yes
1213	0:50 X vs. 39:50 X	-0.4843	-0.6283 to -0.3404	Yes
1214	0:50 X vs. 40:0 X	-3.442	-3.586 to -3.298	Yes
1215	0:50 X vs. 40:1 X	-3.368	-3.512 to -3.224	Yes

	2way ANOVA Multiple comparisons	A Data Set-A Y	B Data Set-B Y	C Data Set-C Y
1216	0:**50 X** vs. 40:**10 X**	-2.573	-2.717 to -2.429	Yes
1217	0:**50 X** vs. 40:**40 X**	-0.6433	-0.7873 to -0.4994	Yes
1218	0:**50 X** vs. 40:**50 X**	-0.4907	-0.6346 to -0.3467	Yes
1219	0:**50 X** vs. 41:**0 X**	-3.483	-3.627 to -3.339	Yes
1220	0:**50 X** vs. 41:**1 X**	-3.482	-3.626 to -3.338	Yes
1221	0:**50 X** vs. 41:**10 X**	-2.626	-2.770 to -2.482	Yes
1222	0:**50 X** vs. 41:**40 X**	-0.5907	-0.7346 to -0.4467	Yes
1223	0:**50 X** vs. 41:**50 X**	-0.5033	-0.6473 to -0.3594	Yes
1224	0:**50 X** vs. 42:**0 X**	-3.625	-3.769 to -3.481	Yes
1225	0:**50 X** vs. 42:**1 X**	-3.644	-3.788 to -3.500	Yes
1226	0:**50 X** vs. 42:**10 X**	-2.627	-2.771 to -2.483	Yes
1227	0:**50 X** vs. 42:**40 X**	-0.6033	-0.7473 to -0.4594	Yes
1228	0:**50 X** vs. 42:**50 X**	-0.5067	-0.6506 to -0.3627	Yes
1229	0:**50 X** vs. 43:**0 X**	-3.715	-3.859 to -3.571	Yes
1230	0:**50 X** vs. 43:**1 X**	-3.629	-3.773 to -3.485	Yes
1231	0:**50 X** vs. 43:**10 X**	-3.345	-3.489 to -3.201	Yes
1232	0:**50 X** vs. 43:**40 X**	-0.6017	-0.7456 to -0.4577	Yes
1233	0:**50 X** vs. 43:**50 X**	-0.5070	-0.6510 to -0.3630	Yes
1234	0:**50 X** vs. 44:**0 X**	-3.739	-3.883 to -3.595	Yes
1235	0:**50 X** vs. 44:**1 X**	-3.628	-3.772 to -3.484	Yes
1236	0:**50 X** vs. 44:**10 X**	-3.408	-3.552 to -3.264	Yes
1237	0:**50 X** vs. 44:**40 X**	-0.6037	-0.7476 to -0.4597	Yes
1238	0:**50 X** vs. 44:**50 X**	-0.4393	-0.5833 to -0.2954	Yes
1239	0:**50 X** vs. 45:**0 X**	-3.603	-3.747 to -3.459	Yes
1240	0:**50 X** vs. 45:**1 X**	-3.405	-3.549 to -3.261	Yes
1241	0:**50 X** vs. 45:**10 X**	-3.288	-3.432 to -3.144	Yes
1242	0:**50 X** vs. 45:**40 X**	-0.6050	-0.7490 to -0.4610	Yes
1243	0:**50 X** vs. 45:**50 X**	-0.4363	-0.5803 to -0.2924	Yes
1244	0:**50 X** vs. 46:**0 X**	-3.408	-3.552 to -3.264	Yes
1245	0:**50 X** vs. 46:**1 X**	-3.375	-3.519 to -3.231	Yes
1246	0:**50 X** vs. 46:**10 X**	-3.283	-3.427 to -3.139	Yes
1247	0:**50 X** vs. 46:**40 X**	-0.6187	-0.7626 to -0.4747	Yes
1248	0:**50 X** vs. 46:**50 X**	-0.4293	-0.5733 to -0.2854	Yes
1249	0:**50 X** vs. 47:**0 X**	-3.383	-3.527 to -3.239	Yes
1250	0:**50 X** vs. 47:**1 X**	-3.287	-3.431 to -3.143	Yes
1251	0:**50 X** vs. 47:**10 X**	-3.269	-3.413 to -3.125	Yes
1252	0:**50 X** vs. 47:**40 X**	-0.6287	-0.7726 to -0.4847	Yes
1253	0:**50 X** vs. 47:**50 X**	-0.3873	-0.5313 to -0.2434	Yes
1254	0:**50 X** vs. 48:**0 X**	-3.371	-3.531 to -3.210	Yes
1255	0:**50 X** vs. 48:**1 X**	-3.274	-3.418 to -3.130	Yes
1256	0:**50 X** vs. 48:**10 X**	-3.269	-3.413 to -3.125	Yes
1257	0:**50 X** vs. 48:**40 X**	-0.6350	-0.7790 to -0.4910	Yes
1258	0:**50 X** vs. 48:**50 X**	-0.3947	-0.5386 to -0.2507	Yes
1259	0:**50 X** vs. 49:**0 X**	-3.371	-3.515 to -3.227	Yes
1260	0:**50 X** vs. 49:**1 X**	-3.274	-3.418 to -3.130	Yes

	2way ANOVA Multiple comparisons	A Data Set-A Y	B Data Set-B Y	C Data Set-C Y
1261	0:**50 X** vs. 49:**10 X**	-3.269	-3.413 to -3.125	Yes
1262	0:**50 X** vs. 49:**40 X**	-0.6393	-0.7833 to -0.4954	Yes
1263	0:**50 X** vs. 49:**50 X**	-0.3940	-0.5380 to -0.2500	Yes
1264	0:**50 X** vs. 50:**0 X**	-3.371	-3.515 to -3.227	Yes
1265	0:**50 X** vs. 50:**1 X**	-3.274	-3.418 to -3.130	Yes
1266	0:**50 X** vs. 50:**10 X**	-3.269	-3.413 to -3.125	Yes
1267	0:**50 X** vs. 50:**40 X**	-0.6470	-0.7910 to -0.5030	Yes
1268	0:**50 X** vs. 50:**50 X**	-0.3937	-0.5376 to -0.2497	Yes
1269	1:**0 X** vs. 1:**1 X**	-0.007667	-0.1516 to 0.1363	No
1270	1:**0 X** vs. 1:**10 X**	0.001667	-0.1423 to 0.1456	No
1271	1:**0 X** vs. 1:**40 X**	-0.0040	-0.1480 to 0.1400	No
1272	1:**0 X** vs. 1:**50 X**	-0.001333	-0.1453 to 0.1426	No
1273	1:**0 X** vs. 2:**0 X**	-0.004667	-0.1486 to 0.1393	No
1274	1:**0 X** vs. 2:**1 X**	-0.009667	-0.1536 to 0.1343	No
1275	1:**0 X** vs. 2:**10 X**	-0.003667	-0.1476 to 0.1403	No
1276	1:**0 X** vs. 2:**40 X**	-0.004333	-0.1483 to 0.1396	No
1277	1:**0 X** vs. 2:**50 X**	-0.001333	-0.1453 to 0.1426	No
1278	1:**0 X** vs. 3:**0 X**	-0.005667	-0.1496 to 0.1383	No
1279	1:**0 X** vs. 3:**1 X**	-0.008000	-0.1520 to 0.1360	No
1280	1:**0 X** vs. 3:**10 X**	-0.003333	-0.1473 to 0.1406	No
1281	1:**0 X** vs. 3:**40 X**	-0.0010	-0.1450 to 0.1430	No
1282	1:**0 X** vs. 3:**50 X**	-3.104e-010	-0.1440 to 0.1440	No
1283	1:**0 X** vs. 4:**0 X**	-0.0100	-0.1540 to 0.1340	No
1284	1:**0 X** vs. 4:**1 X**	-0.008667	-0.1526 to 0.1353	No
1285	1:**0 X** vs. 4:**10 X**	-0.02333	-0.1673 to 0.1206	No
1286	1:**0 X** vs. 4:**40 X**	-0.0030	-0.1470 to 0.1410	No
1287	1:**0 X** vs. 4:**50 X**	-0.0010	-0.1450 to 0.1430	No
1288	1:**0 X** vs. 5:**0 X**	-0.0110	-0.1550 to 0.1330	No
1289	1:**0 X** vs. 5:**1 X**	-0.0090	-0.1530 to 0.1350	No
1290	1:**0 X** vs. 5:**10 X**	-0.0240	-0.1680 to 0.1200	No
1291	1:**0 X** vs. 5:**40 X**	-0.0030	-0.1470 to 0.1410	No
1292	1:**0 X** vs. 5:**50 X**	-0.0020	-0.1460 to 0.1420	No
1293	1:**0 X** vs. 6:**0 X**	-0.01033	-0.1543 to 0.1336	No
1294	1:**0 X** vs. 6:**1 X**	-0.009333	-0.1533 to 0.1346	No
1295	1:**0 X** vs. 6:**10 X**	-0.0240	-0.1680 to 0.1200	No
1296	1:**0 X** vs. 6:**40 X**	-0.005333	-0.1493 to 0.1386	No
1297	1:**0 X** vs. 6:**50 X**	-0.003667	-0.1476 to 0.1403	No
1298	1:**0 X** vs. 7:**0 X**	-0.01233	-0.1563 to 0.1316	No
1299	1:**0 X** vs. 7:**1 X**	-0.003667	-0.1476 to 0.1403	No
1300	1:**0 X** vs. 7:**10 X**	-0.01567	-0.1596 to 0.1283	No
1301	1:**0 X** vs. 7:**40 X**	-0.0070	-0.1510 to 0.1370	No
1302	1:**0 X** vs. 7:**50 X**	-0.0050	-0.1490 to 0.1390	No
1303	1:**0 X** vs. 8:**0 X**	-0.0170	-0.1610 to 0.1270	No
1304	1:**0 X** vs. 8:**1 X**	-0.003667	-0.1476 to 0.1403	No
1305	1:**0 X** vs. 8:**10 X**	-0.0110	-0.1550 to 0.1330	No

	2way ANOVA<br>Multiple comparisons	A<br>Data Set-A<br>Y	B<br>Data Set-B<br>Y	C<br>Data Set-C<br>Y
1306	1:0 X vs. 8:40 X	-0.01167	-0.1556 to 0.1323	No
1307	1:0 X vs. 8:50 X	-0.008667	-0.1526 to 0.1353	No
1308	1:0 X vs. 9:0 X	-0.0250	-0.1690 to 0.1190	No
1309	1:0 X vs. 9:1 X	-0.0330	-0.1770 to 0.1110	No
1310	1:0 X vs. 9:10 X	-0.0340	-0.1780 to 0.1100	No
1311	1:0 X vs. 9:40 X	-0.01633	-0.1603 to 0.1276	No
1312	1:0 X vs. 9:50 X	-0.009667	-0.1536 to 0.1343	No
1313	1:0 X vs. 10:0 X	-0.02667	-0.1706 to 0.1173	No
1314	1:0 X vs. 10:1 X	-0.03367	-0.1776 to 0.1103	No
1315	1:0 X vs. 10:10 X	-0.03633	-0.1803 to 0.1076	No
1316	1:0 X vs. 10:40 X	-0.0260	-0.1700 to 0.1180	No
1317	1:0 X vs. 10:50 X	-0.01267	-0.1566 to 0.1313	No
1318	1:0 X vs. 11:0 X	-0.03633	-0.1803 to 0.1076	No
1319	1:0 X vs. 11:1 X	-0.0380	-0.1820 to 0.1060	No
1320	1:0 X vs. 11:10 X	-0.03833	-0.1823 to 0.1056	No
1321	1:0 X vs. 11:40 X	-0.0300	-0.1740 to 0.1140	No
1322	1:0 X vs. 11:50 X	-0.0180	-0.1620 to 0.1260	No
1323	1:0 X vs. 12:0 X	-0.0370	-0.1810 to 0.1070	No
1324	1:0 X vs. 12:1 X	-0.01933	-0.1633 to 0.1246	No
1325	1:0 X vs. 12:10 X	-0.0370	-0.1810 to 0.1070	No
1326	1:0 X vs. 12:40 X	-0.02333	-0.1673 to 0.1206	No
1327	1:0 X vs. 12:50 X	-0.02667	-0.1706 to 0.1173	No
1328	1:0 X vs. 13:0 X	-0.0310	-0.1750 to 0.1130	No
1329	1:0 X vs. 13:1 X	-0.01833	-0.1623 to 0.1256	No
1330	1:0 X vs. 13:10 X	-0.04033	-0.1843 to 0.1036	No
1331	1:0 X vs. 13:40 X	-0.03667	-0.1806 to 0.1073	No
1332	1:0 X vs. 13:50 X	-0.03767	-0.1816 to 0.1063	No
1333	1:0 X vs. 14:0 X	-0.03967	-0.1836 to 0.1043	No
1334	1:0 X vs. 14:1 X	-0.03133	-0.1753 to 0.1126	No
1335	1:0 X vs. 14:10 X	-0.04533	-0.1893 to 0.09863	No
1336	1:0 X vs. 14:40 X	-0.0420	-0.1860 to 0.1020	No
1337	1:0 X vs. 14:50 X	-0.0460	-0.1900 to 0.09796	No
1338	1:0 X vs. 15:0 X	-0.0520	-0.1960 to 0.09196	No
1339	1:0 X vs. 15:1 X	-0.0320	-0.1760 to 0.1120	No
1340	1:0 X vs. 15:10 X	-0.05933	-0.2033 to 0.08463	No
1341	1:0 X vs. 15:40 X	-0.04167	-0.1856 to 0.1023	No
1342	1:0 X vs. 15:50 X	-0.05933	-0.2033 to 0.08463	No
1343	1:0 X vs. 16:0 X	-0.1443	-0.2883 to -0.0003735	Yes
1344	1:0 X vs. 16:1 X	-0.03533	-0.1793 to 0.1086	No
1345	1:0 X vs. 16:10 X	-0.0610	-0.2050 to 0.08296	No
1346	1:0 X vs. 16:40 X	-0.08033	-0.2243 to 0.06363	No
1347	1:0 X vs. 16:50 X	-0.1227	-0.2666 to 0.02129	No
1348	1:0 X vs. 17:0 X	-0.1970	-0.3410 to -0.05304	Yes
1349	1:0 X vs. 17:1 X	-0.1113	-0.2553 to 0.03263	No
1350	1:0 X vs. 17:10 X	-0.1680	-0.3120 to -0.02404	Yes

	2way ANOVA<br>Multiple comparisons	A<br>Data Set-A<br>Y	B<br>Data Set-B<br>Y	C<br>Data Set-C<br>Y
1351	1:0 X vs. 17:40 X	-0.09133	-0.2353 to 0.05263	No
1352	1:0 X vs. 17:50 X	-0.1367	-0.2806 to 0.007293	No
1353	1:0 X vs. 18:0 X	-0.2457	-0.3896 to -0.1017	Yes
1354	1:0 X vs. 18:1 X	-0.1540	-0.2980 to -0.01004	Yes
1355	1:0 X vs. 18:10 X	-0.2463	-0.3903 to -0.1024	Yes
1356	1:0 X vs. 18:40 X	-0.09567	-0.2396 to 0.04829	No
1357	1:0 X vs. 18:50 X	-0.1407	-0.2846 to 0.003293	No
1358	1:0 X vs. 19:0 X	-0.2807	-0.4246 to -0.1367	Yes
1359	1:0 X vs. 19:1 X	-0.2687	-0.4126 to -0.1247	Yes
1360	1:0 X vs. 19:10 X	-0.2663	-0.4103 to -0.1224	Yes
1361	1:0 X vs. 19:40 X	-0.1243	-0.2683 to 0.01963	No
1362	1:0 X vs. 19:50 X	-0.1353	-0.2793 to 0.008626	No
1363	1:0 X vs. 20:0 X	-0.3603	-0.5043 to -0.2164	Yes
1364	1:0 X vs. 20:1 X	-0.1797	-0.3236 to -0.03571	Yes
1365	1:0 X vs. 20:10 X	-0.2687	-0.4126 to -0.1247	Yes
1366	1:0 X vs. 20:40 X	-0.1360	-0.2800 to 0.007960	No
1367	1:0 X vs. 20:50 X	-0.1800	-0.3240 to -0.03604	Yes
1368	1:0 X vs. 21:0 X	-0.3837	-0.5276 to -0.2397	Yes
1369	1:0 X vs. 21:1 X	-0.3367	-0.4806 to -0.1927	Yes
1370	1:0 X vs. 21:10 X	-0.3337	-0.4776 to -0.1897	Yes
1371	1:0 X vs. 21:40 X	-0.2090	-0.3530 to -0.06504	Yes
1372	1:0 X vs. 21:50 X	-0.2993	-0.4433 to -0.1554	Yes
1373	1:0 X vs. 22:0 X	-0.4253	-0.5693 to -0.2814	Yes
1374	1:0 X vs. 22:1 X	-0.4200	-0.5640 to -0.2760	Yes
1375	1:0 X vs. 22:10 X	-0.4153	-0.5593 to -0.2714	Yes
1376	1:0 X vs. 22:40 X	-0.3157	-0.4596 to -0.1717	Yes
1377	1:0 X vs. 22:50 X	-0.3783	-0.5223 to -0.2344	Yes
1378	1:0 X vs. 23:0 X	-0.4907	-0.6346 to -0.3467	Yes
1379	1:0 X vs. 23:1 X	-0.6803	-0.8413 to -0.5194	Yes
1380	1:0 X vs. 23:10 X	-0.4300	-0.5740 to -0.2860	Yes
1381	1:0 X vs. 23:40 X	-0.3103	-0.4713 to -0.1494	Yes
1382	1:0 X vs. 23:50 X	-0.3443	-0.4883 to -0.2004	Yes
1383	1:0 X vs. 24:0 X	-0.6080	-0.7520 to -0.4640	Yes
1384	1:0 X vs. 24:1 X	-0.7643	-0.9253 to -0.6034	Yes
1385	1:0 X vs. 24:10 X	-0.5918	-0.7528 to -0.4309	Yes
1386	1:0 X vs. 24:40 X	-0.3267	-0.4706 to -0.1827	Yes
1387	1:0 X vs. 24:50 X	-0.2483	-0.3923 to -0.1044	Yes
1388	1:0 X vs. 25:0 X	-0.9453	-1.106 to -0.7844	Yes
1389	1:0 X vs. 25:1 X	-1.135	-1.296 to -0.9739	Yes
1390	1:0 X vs. 25:10 X	-0.4963	-0.6999 to -0.2927	Yes
1391	1:0 X vs. 25:40 X	-0.3287	-0.4726 to -0.1847	Yes
1392	1:0 X vs. 25:50 X	-0.3030	-0.4470 to -0.1590	Yes
1393	1:0 X vs. 26:0 X	-1.158	-1.319 to -0.9969	Yes
1394	1:0 X vs. 26:1 X	-1.271	-1.432 to -1.110	Yes
1395	1:0 X vs. 26:10 X	-0.9573	-1.118 to -0.7964	Yes

	2way ANOVA<br>Multiple comparisons	A<br>Data Set-A<br>Y	B<br>Data Set-B<br>Y	C<br>Data Set-C<br>Y
**1396**	1:**0 X** vs. 26:**40 X**	-0.4030	-0.5470 to -0.2590	Yes
**1397**	1:**0 X** vs. 26:**50 X**	-0.2633	-0.4073 to -0.1194	Yes
**1398**	1:**0 X** vs. 27:**0 X**	-1.346	-1.490 to -1.202	Yes
**1399**	1:**0 X** vs. 27:**1 X**	-1.436	-1.597 to -1.275	Yes
**1400**	1:**0 X** vs. 27:**10 X**	-1.248	-1.409 to -1.087	Yes
**1401**	1:**0 X** vs. 27:**40 X**	-0.4030	-0.5470 to -0.2590	Yes
**1402**	1:**0 X** vs. 27:**50 X**	-0.2627	-0.4066 to -0.1187	Yes
**1403**	1:**0 X** vs. 28:**0 X**	-1.295	-1.456 to -1.134	Yes
**1404**	1:**0 X** vs. 28:**1 X**	-1.336	-1.480 to -1.192	Yes
**1405**	1:**0 X** vs. 28:**10 X**	-1.137	-1.298 to -0.9764	Yes
**1406**	1:**0 X** vs. 28:**40 X**	-0.4153	-0.5593 to -0.2714	Yes
**1407**	1:**0 X** vs. 28:**50 X**	-0.3107	-0.4546 to -0.1667	Yes
**1408**	1:**0 X** vs. 29:**0 X**	-1.776	-1.937 to -1.615	Yes
**1409**	1:**0 X** vs. 29:**1 X**	-1.861	-2.022 to -1.700	Yes
**1410**	1:**0 X** vs. 29:**10 X**	-1.076	-1.237 to -0.9149	Yes
**1411**	1:**0 X** vs. 29:**40 X**	-0.5233	-0.6673 to -0.3794	Yes
**1412**	1:**0 X** vs. 29:**50 X**	-0.3120	-0.4560 to -0.1680	Yes
**1413**	1:**0 X** vs. 30:**0 X**	-1.491	-1.652 to -1.330	Yes
**1414**	1:**0 X** vs. 30:**1 X**	-1.812	-1.973 to -1.651	Yes
**1415**	1:**0 X** vs. 30:**10 X**	-1.150	-1.311 to -0.9894	Yes
**1416**	1:**0 X** vs. 30:**40 X**	-0.4503	-0.5943 to -0.3064	Yes
**1417**	1:**0 X** vs. 30:**50 X**	-0.3643	-0.5083 to -0.2204	Yes
**1418**	1:**0 X** vs. 31:**0 X**	-1.809	-1.970 to -1.648	Yes
**1419**	1:**0 X** vs. 31:**1 X**	-1.799	-1.960 to -1.638	Yes
**1420**	1:**0 X** vs. 31:**10 X**	-1.282	-1.443 to -1.121	Yes
**1421**	1:**0 X** vs. 31:**40 X**	-0.5420	-0.6860 to -0.3980	Yes
**1422**	1:**0 X** vs. 31:**50 X**	-0.3433	-0.4873 to -0.1994	Yes
**1423**	1:**0 X** vs. 32:**0 X**	-2.400	-2.544 to -2.256	Yes
**1424**	1:**0 X** vs. 32:**1 X**	-1.856	-2.017 to -1.695	Yes
**1425**	1:**0 X** vs. 32:**10 X**	-1.712	-1.856 to -1.568	Yes
**1426**	1:**0 X** vs. 32:**40 X**	-0.5383	-0.6823 to -0.3944	Yes
**1427**	1:**0 X** vs. 32:**50 X**	-0.3430	-0.4870 to -0.1990	Yes
**1428**	1:**0 X** vs. 33:**0 X**	-2.404	-2.548 to -2.260	Yes
**1429**	1:**0 X** vs. 33:**1 X**	-2.219	-2.363 to -2.075	Yes
**1430**	1:**0 X** vs. 33:**10 X**	-1.879	-2.023 to -1.735	Yes
**1431**	1:**0 X** vs. 33:**40 X**	-0.5570	-0.7010 to -0.4130	Yes
**1432**	1:**0 X** vs. 33:**50 X**	-0.3580	-0.5020 to -0.2140	Yes
**1433**	1:**0 X** vs. 34:**0 X**	-2.329	-2.473 to -2.185	Yes
**1434**	1:**0 X** vs. 34:**1 X**	-2.336	-2.480 to -2.192	Yes
**1435**	1:**0 X** vs. 34:**10 X**	-1.969	-2.113 to -1.825	Yes
**1436**	1:**0 X** vs. 34:**40 X**	-0.5690	-0.7130 to -0.4250	Yes
**1437**	1:**0 X** vs. 34:**50 X**	-0.5070	-0.6510 to -0.3630	Yes
**1438**	1:**0 X** vs. 35:**0 X**	-2.677	-2.821 to -2.533	Yes
**1439**	1:**0 X** vs. 35:**1 X**	-2.443	-2.587 to -2.299	Yes
**1440**	1:**0 X** vs. 35:**10 X**	-1.931	-2.075 to -1.787	Yes

	2way ANOVA<br>Multiple comparisons	A<br>Data Set-A<br>Y	B<br>Data Set-B<br>Y	C<br>Data Set-C<br>Y
1441	1:0 X vs. 35:40 X	-0.5780	-0.7220 to -0.4340	Yes
1442	1:0 X vs. 35:50 X	-0.5197	-0.6636 to -0.3757	Yes
1443	1:0 X vs. 36:0 X	-2.884	-3.045 to -2.723	Yes
1444	1:0 X vs. 36:1 X	-2.580	-2.724 to -2.436	Yes
1445	1:0 X vs. 36:10 X	-1.937	-2.081 to -1.793	Yes
1446	1:0 X vs. 36:40 X	-0.6333	-0.7773 to -0.4894	Yes
1447	1:0 X vs. 36:50 X	-0.4830	-0.6270 to -0.3390	Yes
1448	1:0 X vs. 37:0 X	-3.268	-3.412 to -3.124	Yes
1449	1:0 X vs. 37:1 X	-2.650	-2.794 to -2.506	Yes
1450	1:0 X vs. 37:10 X	-1.938	-2.082 to -1.794	Yes
1451	1:0 X vs. 37:40 X	-0.6707	-0.8146 to -0.5267	Yes
1452	1:0 X vs. 37:50 X	-0.4917	-0.6356 to -0.3477	Yes
1453	1:0 X vs. 38:0 X	-3.213	-3.357 to -3.069	Yes
1454	1:0 X vs. 38:1 X	-2.768	-2.912 to -2.624	Yes
1455	1:0 X vs. 38:10 X	-2.214	-2.358 to -2.070	Yes
1456	1:0 X vs. 38:40 X	-0.6750	-0.8190 to -0.5310	Yes
1457	1:0 X vs. 38:50 X	-0.4957	-0.6396 to -0.3517	Yes
1458	1:0 X vs. 39:0 X	-3.443	-3.587 to -3.299	Yes
1459	1:0 X vs. 39:1 X	-3.335	-3.479 to -3.191	Yes
1460	1:0 X vs. 39:10 X	-2.359	-2.503 to -2.215	Yes
1461	1:0 X vs. 39:40 X	-0.6810	-0.8250 to -0.5370	Yes
1462	1:0 X vs. 39:50 X	-0.5267	-0.6706 to -0.3827	Yes
1463	1:0 X vs. 40:0 X	-3.484	-3.628 to -3.340	Yes
1464	1:0 X vs. 40:1 X	-3.410	-3.554 to -3.266	Yes
1465	1:0 X vs. 40:10 X	-2.615	-2.759 to -2.471	Yes
1466	1:0 X vs. 40:40 X	-0.6857	-0.8296 to -0.5417	Yes
1467	1:0 X vs. 40:50 X	-0.5330	-0.6770 to -0.3890	Yes
1468	1:0 X vs. 41:0 X	-3.525	-3.669 to -3.381	Yes
1469	1:0 X vs. 41:1 X	-3.524	-3.668 to -3.380	Yes
1470	1:0 X vs. 41:10 X	-2.669	-2.813 to -2.525	Yes
1471	1:0 X vs. 41:40 X	-0.6330	-0.7770 to -0.4890	Yes
1472	1:0 X vs. 41:50 X	-0.5457	-0.6896 to -0.4017	Yes
1473	1:0 X vs. 42:0 X	-3.667	-3.811 to -3.523	Yes
1474	1:0 X vs. 42:1 X	-3.687	-3.831 to -3.543	Yes
1475	1:0 X vs. 42:10 X	-2.669	-2.813 to -2.525	Yes
1476	1:0 X vs. 42:40 X	-0.6457	-0.7896 to -0.5017	Yes
1477	1:0 X vs. 42:50 X	-0.5490	-0.6930 to -0.4050	Yes
1478	1:0 X vs. 43:0 X	-3.757	-3.901 to -3.613	Yes
1479	1:0 X vs. 43:1 X	-3.671	-3.815 to -3.527	Yes
1480	1:0 X vs. 43:10 X	-3.387	-3.531 to -3.243	Yes
1481	1:0 X vs. 43:40 X	-0.6440	-0.7880 to -0.5000	Yes
1482	1:0 X vs. 43:50 X	-0.5493	-0.6933 to -0.4054	Yes
1483	1:0 X vs. 44:0 X	-3.781	-3.925 to -3.637	Yes
1484	1:0 X vs. 44:1 X	-3.671	-3.815 to -3.527	Yes
1485	1:0 X vs. 44:10 X	-3.450	-3.594 to -3.306	Yes

	2way ANOVA<br>Multiple comparisons	A<br>Data Set-A<br>Y	B<br>Data Set-B<br>Y	C<br>Data Set-C<br>Y
1486	1:**0 X** vs. 44:**40 X**	-0.6460	-0.7900 to -0.5020	Yes
1487	1:**0 X** vs. 44:**50 X**	-0.4817	-0.6256 to -0.3377	Yes
1488	1:**0 X** vs. 45:**0 X**	-3.646	-3.790 to -3.502	Yes
1489	1:**0 X** vs. 45:**1 X**	-3.448	-3.592 to -3.304	Yes
1490	1:**0 X** vs. 45:**10 X**	-3.330	-3.474 to -3.186	Yes
1491	1:**0 X** vs. 45:**40 X**	-0.6473	-0.7913 to -0.5034	Yes
1492	1:**0 X** vs. 45:**50 X**	-0.4787	-0.6226 to -0.3347	Yes
1493	1:**0 X** vs. 46:**0 X**	-3.450	-3.594 to -3.306	Yes
1494	1:**0 X** vs. 46:**1 X**	-3.417	-3.561 to -3.273	Yes
1495	1:**0 X** vs. 46:**10 X**	-3.325	-3.469 to -3.181	Yes
1496	1:**0 X** vs. 46:**40 X**	-0.6610	-0.8050 to -0.5170	Yes
1497	1:**0 X** vs. 46:**50 X**	-0.4717	-0.6156 to -0.3277	Yes
1498	1:**0 X** vs. 47:**0 X**	-3.426	-3.570 to -3.282	Yes
1499	1:**0 X** vs. 47:**1 X**	-3.330	-3.474 to -3.186	Yes
1500	1:**0 X** vs. 47:**10 X**	-3.311	-3.455 to -3.167	Yes
1501	1:**0 X** vs. 47:**40 X**	-0.6710	-0.8150 to -0.5270	Yes
1502	1:**0 X** vs. 47:**50 X**	-0.4297	-0.5736 to -0.2857	Yes
1503	1:**0 X** vs. 48:**0 X**	-3.413	-3.574 to -3.252	Yes
1504	1:**0 X** vs. 48:**1 X**	-3.317	-3.461 to -3.173	Yes
1505	1:**0 X** vs. 48:**10 X**	-3.311	-3.455 to -3.167	Yes
1506	1:**0 X** vs. 48:**40 X**	-0.6773	-0.8213 to -0.5334	Yes
1507	1:**0 X** vs. 48:**50 X**	-0.4370	-0.5810 to -0.2930	Yes
1508	1:**0 X** vs. 49:**0 X**	-3.413	-3.557 to -3.269	Yes
1509	1:**0 X** vs. 49:**1 X**	-3.317	-3.461 to -3.173	Yes
1510	1:**0 X** vs. 49:**10 X**	-3.311	-3.455 to -3.167	Yes
1511	1:**0 X** vs. 49:**40 X**	-0.6817	-0.8256 to -0.5377	Yes
1512	1:**0 X** vs. 49:**50 X**	-0.4363	-0.5803 to -0.2924	Yes
1513	1:**0 X** vs. 50:**0 X**	-3.413	-3.557 to -3.269	Yes
1514	1:**0 X** vs. 50:**1 X**	-3.317	-3.461 to -3.173	Yes
1515	1:**0 X** vs. 50:**10 X**	-3.311	-3.455 to -3.167	Yes
1516	1:**0 X** vs. 50:**40 X**	-0.6893	-0.8333 to -0.5454	Yes
1517	1:**0 X** vs. 50:**50 X**	-0.4360	-0.5800 to -0.2920	Yes
1518	1:**1 X** vs. 1:**10 X**	0.009333	-0.1346 to 0.1533	No
1519	1:**1 X** vs. 1:**40 X**	0.003667	-0.1403 to 0.1476	No
1520	1:**1 X** vs. 1:**50 X**	0.006333	-0.1376 to 0.1503	No
1521	1:**1 X** vs. 2:**0 X**	0.0030	-0.1410 to 0.1470	No
1522	1:**1 X** vs. 2:**1 X**	-0.002000	-0.1460 to 0.1420	No
1523	1:**1 X** vs. 2:**10 X**	0.0040	-0.1400 to 0.1480	No
1524	1:**1 X** vs. 2:**40 X**	0.003333	-0.1406 to 0.1473	No
1525	1:**1 X** vs. 2:**50 X**	0.006333	-0.1376 to 0.1503	No
1526	1:**1 X** vs. 3:**0 X**	0.0020	-0.1420 to 0.1460	No
1527	1:**1 X** vs. 3:**1 X**	-0.0003333	-0.1443 to 0.1436	No
1528	1:**1 X** vs. 3:**10 X**	0.004333	-0.1396 to 0.1483	No
1529	1:**1 X** vs. 3:**40 X**	0.006667	-0.1373 to 0.1506	No
1530	1:**1 X** vs. 3:**50 X**	0.007667	-0.1363 to 0.1516	No

	2way ANOVA<br>Multiple comparisons	A<br>Data Set-A<br>Y	B<br>Data Set-B<br>Y	C<br>Data Set-C<br>Y
1531	1:1 X vs. 4:0 X	-0.002333	-0.1463 to 0.1416	No
1532	1:1 X vs. 4:1 X	-0.001000	-0.1450 to 0.1430	No
1533	1:1 X vs. 4:10 X	-0.01567	-0.1596 to 0.1283	No
1534	1:1 X vs. 4:40 X	0.004667	-0.1393 to 0.1486	No
1535	1:1 X vs. 4:50 X	0.006667	-0.1373 to 0.1506	No
1536	1:1 X vs. 5:0 X	-0.003333	-0.1473 to 0.1406	No
1537	1:1 X vs. 5:1 X	-0.001333	-0.1453 to 0.1426	No
1538	1:1 X vs. 5:10 X	-0.01633	-0.1603 to 0.1276	No
1539	1:1 X vs. 5:40 X	0.004667	-0.1393 to 0.1486	No
1540	1:1 X vs. 5:50 X	0.005667	-0.1383 to 0.1496	No
1541	1:1 X vs. 6:0 X	-0.002667	-0.1466 to 0.1413	No
1542	1:1 X vs. 6:1 X	-0.001667	-0.1456 to 0.1423	No
1543	1:1 X vs. 6:10 X	-0.01633	-0.1603 to 0.1276	No
1544	1:1 X vs. 6:40 X	0.002333	-0.1416 to 0.1463	No
1545	1:1 X vs. 6:50 X	0.0040	-0.1400 to 0.1480	No
1546	1:1 X vs. 7:0 X	-0.004667	-0.1486 to 0.1393	No
1547	1:1 X vs. 7:1 X	0.0040	-0.1400 to 0.1480	No
1548	1:1 X vs. 7:10 X	-0.008000	-0.1520 to 0.1360	No
1549	1:1 X vs. 7:40 X	0.0006667	-0.1433 to 0.1446	No
1550	1:1 X vs. 7:50 X	0.002667	-0.1413 to 0.1466	No
1551	1:1 X vs. 8:0 X	-0.009333	-0.1533 to 0.1346	No
1552	1:1 X vs. 8:1 X	0.0040	-0.1400 to 0.1480	No
1553	1:1 X vs. 8:10 X	-0.003333	-0.1473 to 0.1406	No
1554	1:1 X vs. 8:40 X	-0.004000	-0.1480 to 0.1400	No
1555	1:1 X vs. 8:50 X	-0.001000	-0.1450 to 0.1430	No
1556	1:1 X vs. 9:0 X	-0.01733	-0.1613 to 0.1266	No
1557	1:1 X vs. 9:1 X	-0.02533	-0.1693 to 0.1186	No
1558	1:1 X vs. 9:10 X	-0.02633	-0.1703 to 0.1176	No
1559	1:1 X vs. 9:40 X	-0.008667	-0.1526 to 0.1353	No
1560	1:1 X vs. 9:50 X	-0.0020	-0.1460 to 0.1420	No
1561	1:1 X vs. 10:0 X	-0.0190	-0.1630 to 0.1250	No
1562	1:1 X vs. 10:1 X	-0.0260	-0.1700 to 0.1180	No
1563	1:1 X vs. 10:10 X	-0.02867	-0.1726 to 0.1153	No
1564	1:1 X vs. 10:40 X	-0.01833	-0.1623 to 0.1256	No
1565	1:1 X vs. 10:50 X	-0.005000	-0.1490 to 0.1390	No
1566	1:1 X vs. 11:0 X	-0.02867	-0.1726 to 0.1153	No
1567	1:1 X vs. 11:1 X	-0.03033	-0.1743 to 0.1136	No
1568	1:1 X vs. 11:10 X	-0.03067	-0.1746 to 0.1133	No
1569	1:1 X vs. 11:40 X	-0.02233	-0.1663 to 0.1216	No
1570	1:1 X vs. 11:50 X	-0.01033	-0.1543 to 0.1336	No
1571	1:1 X vs. 12:0 X	-0.02933	-0.1733 to 0.1146	No
1572	1:1 X vs. 12:1 X	-0.01167	-0.1556 to 0.1323	No
1573	1:1 X vs. 12:10 X	-0.02933	-0.1733 to 0.1146	No
1574	1:1 X vs. 12:40 X	-0.01567	-0.1596 to 0.1283	No
1575	1:1 X vs. 12:50 X	-0.0190	-0.1630 to 0.1250	No

	2way ANOVA<br>Multiple comparisons	A<br>Data Set-A<br>Y	B<br>Data Set-B<br>Y	C<br>Data Set-C<br>Y
1576	1:**1 X** vs. 13:**0 X**	-0.02333	-0.1673 to 0.1206	No
1577	1:**1 X** vs. 13:**1 X**	-0.01067	-0.1546 to 0.1333	No
1578	1:**1 X** vs. 13:**10 X**	-0.03267	-0.1766 to 0.1113	No
1579	1:**1 X** vs. 13:**40 X**	-0.0290	-0.1730 to 0.1150	No
1580	1:**1 X** vs. 13:**50 X**	-0.0300	-0.1740 to 0.1140	No
1581	1:**1 X** vs. 14:**0 X**	-0.0320	-0.1760 to 0.1120	No
1582	1:**1 X** vs. 14:**1 X**	-0.02367	-0.1676 to 0.1203	No
1583	1:**1 X** vs. 14:**10 X**	-0.03767	-0.1816 to 0.1063	No
1584	1:**1 X** vs. 14:**40 X**	-0.03433	-0.1783 to 0.1096	No
1585	1:**1 X** vs. 14:**50 X**	-0.03833	-0.1823 to 0.1056	No
1586	1:**1 X** vs. 15:**0 X**	-0.04433	-0.1883 to 0.09963	No
1587	1:**1 X** vs. 15:**1 X**	-0.02433	-0.1683 to 0.1196	No
1588	1:**1 X** vs. 15:**10 X**	-0.05167	-0.1956 to 0.09229	No
1589	1:**1 X** vs. 15:**40 X**	-0.0340	-0.1780 to 0.1100	No
1590	1:**1 X** vs. 15:**50 X**	-0.05167	-0.1956 to 0.09229	No
1591	1:**1 X** vs. 16:**0 X**	-0.1367	-0.2806 to 0.007293	No
1592	1:**1 X** vs. 16:**1 X**	-0.02767	-0.1716 to 0.1163	No
1593	1:**1 X** vs. 16:**10 X**	-0.05333	-0.1973 to 0.09063	No
1594	1:**1 X** vs. 16:**40 X**	-0.07267	-0.2166 to 0.07129	No
1595	1:**1 X** vs. 16:**50 X**	-0.1150	-0.2590 to 0.02896	No
1596	1:**1 X** vs. 17:**0 X**	-0.1893	-0.3333 to -0.04537	Yes
1597	1:**1 X** vs. 17:**1 X**	-0.1037	-0.2476 to 0.04029	No
1598	1:**1 X** vs. 17:**10 X**	-0.1603	-0.3043 to -0.01637	Yes
1599	1:**1 X** vs. 17:**40 X**	-0.08367	-0.2276 to 0.06029	No
1600	1:**1 X** vs. 17:**50 X**	-0.1290	-0.2730 to 0.01496	No
1601	1:**1 X** vs. 18:**0 X**	-0.2380	-0.3820 to -0.09404	Yes
1602	1:**1 X** vs. 18:**1 X**	-0.1463	-0.2903 to -0.002374	Yes
1603	1:**1 X** vs. 18:**10 X**	-0.2387	-0.3826 to -0.09471	Yes
1604	1:**1 X** vs. 18:**40 X**	-0.0880	-0.2320 to 0.05596	No
1605	1:**1 X** vs. 18:**50 X**	-0.1330	-0.2770 to 0.01096	No
1606	1:**1 X** vs. 19:**0 X**	-0.2730	-0.4170 to -0.1290	Yes
1607	1:**1 X** vs. 19:**1 X**	-0.2610	-0.4050 to -0.1170	Yes
1608	1:**1 X** vs. 19:**10 X**	-0.2587	-0.4026 to -0.1147	Yes
1609	1:**1 X** vs. 19:**40 X**	-0.1167	-0.2606 to 0.02729	No
1610	1:**1 X** vs. 19:**50 X**	-0.1277	-0.2716 to 0.01629	No
1611	1:**1 X** vs. 20:**0 X**	-0.3527	-0.4966 to -0.2087	Yes
1612	1:**1 X** vs. 20:**1 X**	-0.1720	-0.3160 to -0.02804	Yes
1613	1:**1 X** vs. 20:**10 X**	-0.2610	-0.4050 to -0.1170	Yes
1614	1:**1 X** vs. 20:**40 X**	-0.1283	-0.2723 to 0.01563	No
1615	1:**1 X** vs. 20:**50 X**	-0.1723	-0.3163 to -0.02837	Yes
1616	1:**1 X** vs. 21:**0 X**	-0.3760	-0.5200 to -0.2320	Yes
1617	1:**1 X** vs. 21:**1 X**	-0.3290	-0.4730 to -0.1850	Yes
1618	1:**1 X** vs. 21:**10 X**	-0.3260	-0.4700 to -0.1820	Yes
1619	1:**1 X** vs. 21:**40 X**	-0.2013	-0.3453 to -0.05737	Yes
1620	1:**1 X** vs. 21:**50 X**	-0.2917	-0.4356 to -0.1477	Yes

	2way ANOVA<br>Multiple comparisons	A<br>Data Set-A<br>Y	B<br>Data Set-B<br>Y	C<br>Data Set-C<br>Y
1621	1:**1 X** vs. 22:**0 X**	-0.4177	-0.5616 to -0.2737	Yes
1622	1:**1 X** vs. 22:**1 X**	-0.4123	-0.5563 to -0.2684	Yes
1623	1:**1 X** vs. 22:**10 X**	-0.4077	-0.5516 to -0.2637	Yes
1624	1:**1 X** vs. 22:**40 X**	-0.3080	-0.4520 to -0.1640	Yes
1625	1:**1 X** vs. 22:**50 X**	-0.3707	-0.5146 to -0.2267	Yes
1626	1:**1 X** vs. 23:**0 X**	-0.4830	-0.6270 to -0.3390	Yes
1627	1:**1 X** vs. 23:**1 X**	-0.6727	-0.8336 to -0.5117	Yes
1628	1:**1 X** vs. 23:**10 X**	-0.4223	-0.5663 to -0.2784	Yes
1629	1:**1 X** vs. 23:**40 X**	-0.3027	-0.4636 to -0.1417	Yes
1630	1:**1 X** vs. 23:**50 X**	-0.3367	-0.4806 to -0.1927	Yes
1631	1:**1 X** vs. 24:**0 X**	-0.6003	-0.7443 to -0.4564	Yes
1632	1:**1 X** vs. 24:**1 X**	-0.7567	-0.9176 to -0.5957	Yes
1633	1:**1 X** vs. 24:**10 X**	-0.5842	-0.7451 to -0.4232	Yes
1634	1:**1 X** vs. 24:**40 X**	-0.3190	-0.4630 to -0.1750	Yes
1635	1:**1 X** vs. 24:**50 X**	-0.2407	-0.3846 to -0.09671	Yes
1636	1:**1 X** vs. 25:**0 X**	-0.9377	-1.099 to -0.7767	Yes
1637	1:**1 X** vs. 25:**1 X**	-1.127	-1.288 to -0.9662	Yes
1638	1:**1 X** vs. 25:**10 X**	-0.4887	-0.6923 to -0.2851	Yes
1639	1:**1 X** vs. 25:**40 X**	-0.3210	-0.4650 to -0.1770	Yes
1640	1:**1 X** vs. 25:**50 X**	-0.2953	-0.4393 to -0.1514	Yes
1641	1:**1 X** vs. 26:**0 X**	-1.150	-1.311 to -0.9892	Yes
1642	1:**1 X** vs. 26:**1 X**	-1.263	-1.424 to -1.102	Yes
1643	1:**1 X** vs. 26:**10 X**	-0.9497	-1.111 to -0.7887	Yes
1644	1:**1 X** vs. 26:**40 X**	-0.3953	-0.5393 to -0.2514	Yes
1645	1:**1 X** vs. 26:**50 X**	-0.2557	-0.3996 to -0.1117	Yes
1646	1:**1 X** vs. 27:**0 X**	-1.338	-1.482 to -1.194	Yes
1647	1:**1 X** vs. 27:**1 X**	-1.429	-1.590 to -1.268	Yes
1648	1:**1 X** vs. 27:**10 X**	-1.241	-1.402 to -1.080	Yes
1649	1:**1 X** vs. 27:**40 X**	-0.3953	-0.5393 to -0.2514	Yes
1650	1:**1 X** vs. 27:**50 X**	-0.2550	-0.3990 to -0.1110	Yes
1651	1:**1 X** vs. 28:**0 X**	-1.287	-1.448 to -1.126	Yes
1652	1:**1 X** vs. 28:**1 X**	-1.328	-1.472 to -1.184	Yes
1653	1:**1 X** vs. 28:**10 X**	-1.130	-1.291 to -0.9687	Yes
1654	1:**1 X** vs. 28:**40 X**	-0.4077	-0.5516 to -0.2637	Yes
1655	1:**1 X** vs. 28:**50 X**	-0.3030	-0.4470 to -0.1590	Yes
1656	1:**1 X** vs. 29:**0 X**	-1.769	-1.930 to -1.608	Yes
1657	1:**1 X** vs. 29:**1 X**	-1.854	-2.015 to -1.693	Yes
1658	1:**1 X** vs. 29:**10 X**	-1.068	-1.229 to -0.9072	Yes
1659	1:**1 X** vs. 29:**40 X**	-0.5157	-0.6596 to -0.3717	Yes
1660	1:**1 X** vs. 29:**50 X**	-0.3043	-0.4483 to -0.1604	Yes
1661	1:**1 X** vs. 30:**0 X**	-1.483	-1.644 to -1.322	Yes
1662	1:**1 X** vs. 30:**1 X**	-1.805	-1.966 to -1.644	Yes
1663	1:**1 X** vs. 30:**10 X**	-1.143	-1.304 to -0.9817	Yes
1664	1:**1 X** vs. 30:**40 X**	-0.4427	-0.5866 to -0.2987	Yes
1665	1:**1 X** vs. 30:**50 X**	-0.3567	-0.5006 to -0.2127	Yes

	2way ANOVA<br>Multiple comparisons	A<br>Data Set-A<br>Y	B<br>Data Set-B<br>Y	C<br>Data Set-C<br>Y
1666	1:1 X vs. 31:0 X	-1.801	-1.962 to -1.640	Yes
1667	1:1 X vs. 31:1 X	-1.792	-1.953 to -1.631	Yes
1668	1:1 X vs. 31:10 X	-1.274	-1.435 to -1.113	Yes
1669	1:1 X vs. 31:40 X	-0.5343	-0.6783 to -0.3904	Yes
1670	1:1 X vs. 31:50 X	-0.3357	-0.4796 to -0.1917	Yes
1671	1:1 X vs. 32:0 X	-2.393	-2.537 to -2.249	Yes
1672	1:1 X vs. 32:1 X	-1.849	-2.010 to -1.688	Yes
1673	1:1 X vs. 32:10 X	-1.705	-1.849 to -1.561	Yes
1674	1:1 X vs. 32:40 X	-0.5307	-0.6746 to -0.3867	Yes
1675	1:1 X vs. 32:50 X	-0.3353	-0.4793 to -0.1914	Yes
1676	1:1 X vs. 33:0 X	-2.396	-2.540 to -2.252	Yes
1677	1:1 X vs. 33:1 X	-2.211	-2.355 to -2.067	Yes
1678	1:1 X vs. 33:10 X	-1.871	-2.015 to -1.727	Yes
1679	1:1 X vs. 33:40 X	-0.5493	-0.6933 to -0.4054	Yes
1680	1:1 X vs. 33:50 X	-0.3503	-0.4943 to -0.2064	Yes
1681	1:1 X vs. 34:0 X	-2.321	-2.465 to -2.177	Yes
1682	1:1 X vs. 34:1 X	-2.328	-2.472 to -2.184	Yes
1683	1:1 X vs. 34:10 X	-1.962	-2.106 to -1.818	Yes
1684	1:1 X vs. 34:40 X	-0.5613	-0.7053 to -0.4174	Yes
1685	1:1 X vs. 34:50 X	-0.4993	-0.6433 to -0.3554	Yes
1686	1:1 X vs. 35:0 X	-2.670	-2.814 to -2.526	Yes
1687	1:1 X vs. 35:1 X	-2.435	-2.579 to -2.291	Yes
1688	1:1 X vs. 35:10 X	-1.923	-2.067 to -1.779	Yes
1689	1:1 X vs. 35:40 X	-0.5703	-0.7143 to -0.4264	Yes
1690	1:1 X vs. 35:50 X	-0.5120	-0.6560 to -0.3680	Yes
1691	1:1 X vs. 36:0 X	-2.876	-3.037 to -2.715	Yes
1692	1:1 X vs. 36:1 X	-2.572	-2.716 to -2.428	Yes
1693	1:1 X vs. 36:10 X	-1.929	-2.073 to -1.785	Yes
1694	1:1 X vs. 36:40 X	-0.6257	-0.7696 to -0.4817	Yes
1695	1:1 X vs. 36:50 X	-0.4753	-0.6193 to -0.3314	Yes
1696	1:1 X vs. 37:0 X	-3.260	-3.404 to -3.116	Yes
1697	1:1 X vs. 37:1 X	-2.642	-2.786 to -2.498	Yes
1698	1:1 X vs. 37:10 X	-1.931	-2.075 to -1.787	Yes
1699	1:1 X vs. 37:40 X	-0.6630	-0.8070 to -0.5190	Yes
1700	1:1 X vs. 37:50 X	-0.4840	-0.6280 to -0.3400	Yes
1701	1:1 X vs. 38:0 X	-3.205	-3.349 to -3.061	Yes
1702	1:1 X vs. 38:1 X	-2.761	-2.905 to -2.617	Yes
1703	1:1 X vs. 38:10 X	-2.206	-2.350 to -2.062	Yes
1704	1:1 X vs. 38:40 X	-0.6673	-0.8113 to -0.5234	Yes
1705	1:1 X vs. 38:50 X	-0.4880	-0.6320 to -0.3440	Yes
1706	1:1 X vs. 39:0 X	-3.436	-3.580 to -3.292	Yes
1707	1:1 X vs. 39:1 X	-3.327	-3.471 to -3.183	Yes
1708	1:1 X vs. 39:10 X	-2.351	-2.495 to -2.207	Yes
1709	1:1 X vs. 39:40 X	-0.6733	-0.8173 to -0.5294	Yes
1710	1:1 X vs. 39:50 X	-0.5190	-0.6630 to -0.3750	Yes

	2way ANOVA<br>Multiple comparisons	A<br>Data Set-A<br>Y	B<br>Data Set-B<br>Y	C<br>Data Set-C<br>Y
**1711**	1:**1 X** vs. 40:**0 X**	-3.477	-3.621 to -3.333	Yes
**1712**	1:**1 X** vs. 40:**1 X**	-3.403	-3.547 to -3.259	Yes
**1713**	1:**1 X** vs. 40:**10 X**	-2.607	-2.751 to -2.463	Yes
**1714**	1:**1 X** vs. 40:**40 X**	-0.6780	-0.8220 to -0.5340	Yes
**1715**	1:**1 X** vs. 40:**50 X**	-0.5253	-0.6693 to -0.3814	Yes
**1716**	1:**1 X** vs. 41:**0 X**	-3.518	-3.662 to -3.374	Yes
**1717**	1:**1 X** vs. 41:**1 X**	-3.517	-3.661 to -3.373	Yes
**1718**	1:**1 X** vs. 41:**10 X**	-2.661	-2.805 to -2.517	Yes
**1719**	1:**1 X** vs. 41:**40 X**	-0.6253	-0.7693 to -0.4814	Yes
**1720**	1:**1 X** vs. 41:**50 X**	-0.5380	-0.6820 to -0.3940	Yes
**1721**	1:**1 X** vs. 42:**0 X**	-3.659	-3.803 to -3.515	Yes
**1722**	1:**1 X** vs. 42:**1 X**	-3.679	-3.823 to -3.535	Yes
**1723**	1:**1 X** vs. 42:**10 X**	-2.661	-2.805 to -2.517	Yes
**1724**	1:**1 X** vs. 42:**40 X**	-0.6380	-0.7820 to -0.4940	Yes
**1725**	1:**1 X** vs. 42:**50 X**	-0.5413	-0.6853 to -0.3974	Yes
**1726**	1:**1 X** vs. 43:**0 X**	-3.749	-3.893 to -3.605	Yes
**1727**	1:**1 X** vs. 43:**1 X**	-3.664	-3.808 to -3.520	Yes
**1728**	1:**1 X** vs. 43:**10 X**	-3.379	-3.523 to -3.235	Yes
**1729**	1:**1 X** vs. 43:**40 X**	-0.6363	-0.7803 to -0.4924	Yes
**1730**	1:**1 X** vs. 43:**50 X**	-0.5417	-0.6856 to -0.3977	Yes
**1731**	1:**1 X** vs. 44:**0 X**	-3.773	-3.917 to -3.629	Yes
**1732**	1:**1 X** vs. 44:**1 X**	-3.663	-3.807 to -3.519	Yes
**1733**	1:**1 X** vs. 44:**10 X**	-3.443	-3.587 to -3.299	Yes
**1734**	1:**1 X** vs. 44:**40 X**	-0.6383	-0.7823 to -0.4944	Yes
**1735**	1:**1 X** vs. 44:**50 X**	-0.4740	-0.6180 to -0.3300	Yes
**1736**	1:**1 X** vs. 45:**0 X**	-3.638	-3.782 to -3.494	Yes
**1737**	1:**1 X** vs. 45:**1 X**	-3.440	-3.584 to -3.296	Yes
**1738**	1:**1 X** vs. 45:**10 X**	-3.322	-3.466 to -3.178	Yes
**1739**	1:**1 X** vs. 45:**40 X**	-0.6397	-0.7836 to -0.4957	Yes
**1740**	1:**1 X** vs. 45:**50 X**	-0.4710	-0.6150 to -0.3270	Yes
**1741**	1:**1 X** vs. 46:**0 X**	-3.442	-3.586 to -3.298	Yes
**1742**	1:**1 X** vs. 46:**1 X**	-3.409	-3.553 to -3.265	Yes
**1743**	1:**1 X** vs. 46:**10 X**	-3.317	-3.461 to -3.173	Yes
**1744**	1:**1 X** vs. 46:**40 X**	-0.6533	-0.7973 to -0.5094	Yes
**1745**	1:**1 X** vs. 46:**50 X**	-0.4640	-0.6080 to -0.3200	Yes
**1746**	1:**1 X** vs. 47:**0 X**	-3.418	-3.562 to -3.274	Yes
**1747**	1:**1 X** vs. 47:**1 X**	-3.322	-3.466 to -3.178	Yes
**1748**	1:**1 X** vs. 47:**10 X**	-3.303	-3.447 to -3.159	Yes
**1749**	1:**1 X** vs. 47:**40 X**	-0.6633	-0.8073 to -0.5194	Yes
**1750**	1:**1 X** vs. 47:**50 X**	-0.4220	-0.5660 to -0.2780	Yes
**1751**	1:**1 X** vs. 48:**0 X**	-3.405	-3.566 to -3.244	Yes
**1752**	1:**1 X** vs. 48:**1 X**	-3.309	-3.453 to -3.165	Yes
**1753**	1:**1 X** vs. 48:**10 X**	-3.303	-3.447 to -3.159	Yes
**1754**	1:**1 X** vs. 48:**40 X**	-0.6697	-0.8136 to -0.5257	Yes
**1755**	1:**1 X** vs. 48:**50 X**	-0.4293	-0.5733 to -0.2854	Yes

	2way ANOVA<br>Multiple comparisons	A<br>Data Set-A<br>Y	B<br>Data Set-B<br>Y	C<br>Data Set-C<br>Y
1756	1:**1 X** vs. 49:**0 X**	-3.405	-3.549 to -3.261	Yes
1757	1:**1 X** vs. 49:**1 X**	-3.309	-3.453 to -3.165	Yes
1758	1:**1 X** vs. 49:**10 X**	-3.303	-3.447 to -3.159	Yes
1759	1:**1 X** vs. 49:**40 X**	-0.6740	-0.8180 to -0.5300	Yes
1760	1:**1 X** vs. 49:**50 X**	-0.4287	-0.5726 to -0.2847	Yes
1761	1:**1 X** vs. 50:**0 X**	-3.405	-3.549 to -3.261	Yes
1762	1:**1 X** vs. 50:**1 X**	-3.309	-3.453 to -3.165	Yes
1763	1:**1 X** vs. 50:**10 X**	-3.303	-3.447 to -3.159	Yes
1764	1:**1 X** vs. 50:**40 X**	-0.6817	-0.8256 to -0.5377	Yes
1765	1:**1 X** vs. 50:**50 X**	-0.4283	-0.5723 to -0.2844	Yes
1766	1:**10 X** vs. 1:**40 X**	-0.005667	-0.1496 to 0.1383	No
1767	1:**10 X** vs. 1:**50 X**	-0.0030	-0.1470 to 0.1410	No
1768	1:**10 X** vs. 2:**0 X**	-0.006333	-0.1503 to 0.1376	No
1769	1:**10 X** vs. 2:**1 X**	-0.01133	-0.1553 to 0.1326	No
1770	1:**10 X** vs. 2:**10 X**	-0.005333	-0.1493 to 0.1386	No
1771	1:**10 X** vs. 2:**40 X**	-0.0060	-0.1500 to 0.1380	No
1772	1:**10 X** vs. 2:**50 X**	-0.0030	-0.1470 to 0.1410	No
1773	1:**10 X** vs. 3:**0 X**	-0.007333	-0.1513 to 0.1366	No
1774	1:**10 X** vs. 3:**1 X**	-0.009667	-0.1536 to 0.1343	No
1775	1:**10 X** vs. 3:**10 X**	-0.0050	-0.1490 to 0.1390	No
1776	1:**10 X** vs. 3:**40 X**	-0.002667	-0.1466 to 0.1413	No
1777	1:**10 X** vs. 3:**50 X**	-0.001667	-0.1456 to 0.1423	No
1778	1:**10 X** vs. 4:**0 X**	-0.01167	-0.1556 to 0.1323	No
1779	1:**10 X** vs. 4:**1 X**	-0.01033	-0.1543 to 0.1336	No
1780	1:**10 X** vs. 4:**10 X**	-0.0250	-0.1690 to 0.1190	No
1781	1:**10 X** vs. 4:**40 X**	-0.004667	-0.1486 to 0.1393	No
1782	1:**10 X** vs. 4:**50 X**	-0.002667	-0.1466 to 0.1413	No
1783	1:**10 X** vs. 5:**0 X**	-0.01267	-0.1566 to 0.1313	No
1784	1:**10 X** vs. 5:**1 X**	-0.01067	-0.1546 to 0.1333	No
1785	1:**10 X** vs. 5:**10 X**	-0.02567	-0.1696 to 0.1183	No
1786	1:**10 X** vs. 5:**40 X**	-0.004667	-0.1486 to 0.1393	No
1787	1:**10 X** vs. 5:**50 X**	-0.003667	-0.1476 to 0.1403	No
1788	1:**10 X** vs. 6:**0 X**	-0.0120	-0.1560 to 0.1320	No
1789	1:**10 X** vs. 6:**1 X**	-0.0110	-0.1550 to 0.1330	No
1790	1:**10 X** vs. 6:**10 X**	-0.02567	-0.1696 to 0.1183	No
1791	1:**10 X** vs. 6:**40 X**	-0.0070	-0.1510 to 0.1370	No
1792	1:**10 X** vs. 6:**50 X**	-0.005333	-0.1493 to 0.1386	No
1793	1:**10 X** vs. 7:**0 X**	-0.0140	-0.1580 to 0.1300	No
1794	1:**10 X** vs. 7:**1 X**	-0.005333	-0.1493 to 0.1386	No
1795	1:**10 X** vs. 7:**10 X**	-0.01733	-0.1613 to 0.1266	No
1796	1:**10 X** vs. 7:**40 X**	-0.008667	-0.1526 to 0.1353	No
1797	1:**10 X** vs. 7:**50 X**	-0.006667	-0.1506 to 0.1373	No
1798	1:**10 X** vs. 8:**0 X**	-0.01867	-0.1626 to 0.1253	No
1799	1:**10 X** vs. 8:**1 X**	-0.005333	-0.1493 to 0.1386	No
1800	1:**10 X** vs. 8:**10 X**	-0.01267	-0.1566 to 0.1313	No

	2way ANOVA<br>Multiple comparisons	A<br>Data Set-A<br>Y	B<br>Data Set-B<br>Y	C<br>Data Set-C<br>Y
1801	1:10 X vs. 8:40 X	-0.01333	-0.1573 to 0.1306	No
1802	1:10 X vs. 8:50 X	-0.01033	-0.1543 to 0.1336	No
1803	1:10 X vs. 9:0 X	-0.02667	-0.1706 to 0.1173	No
1804	1:10 X vs. 9:1 X	-0.03467	-0.1786 to 0.1093	No
1805	1:10 X vs. 9:10 X	-0.03567	-0.1796 to 0.1083	No
1806	1:10 X vs. 9:40 X	-0.0180	-0.1620 to 0.1260	No
1807	1:10 X vs. 9:50 X	-0.01133	-0.1553 to 0.1326	No
1808	1:10 X vs. 10:0 X	-0.02833	-0.1723 to 0.1156	No
1809	1:10 X vs. 10:1 X	-0.03533	-0.1793 to 0.1086	No
1810	1:10 X vs. 10:10 X	-0.0380	-0.1820 to 0.1060	No
1811	1:10 X vs. 10:40 X	-0.02767	-0.1716 to 0.1163	No
1812	1:10 X vs. 10:50 X	-0.01433	-0.1583 to 0.1296	No
1813	1:10 X vs. 11:0 X	-0.0380	-0.1820 to 0.1060	No
1814	1:10 X vs. 11:1 X	-0.03967	-0.1836 to 0.1043	No
1815	1:10 X vs. 11:10 X	-0.0400	-0.1840 to 0.1040	No
1816	1:10 X vs. 11:40 X	-0.03167	-0.1756 to 0.1123	No
1817	1:10 X vs. 11:50 X	-0.01967	-0.1636 to 0.1243	No
1818	1:10 X vs. 12:0 X	-0.03867	-0.1826 to 0.1053	No
1819	1:10 X vs. 12:1 X	-0.0210	-0.1650 to 0.1230	No
1820	1:10 X vs. 12:10 X	-0.03867	-0.1826 to 0.1053	No
1821	1:10 X vs. 12:40 X	-0.0250	-0.1690 to 0.1190	No
1822	1:10 X vs. 12:50 X	-0.02833	-0.1723 to 0.1156	No
1823	1:10 X vs. 13:0 X	-0.03267	-0.1766 to 0.1113	No
1824	1:10 X vs. 13:1 X	-0.0200	-0.1640 to 0.1240	No
1825	1:10 X vs. 13:10 X	-0.0420	-0.1860 to 0.1020	No
1826	1:10 X vs. 13:40 X	-0.03833	-0.1823 to 0.1056	No
1827	1:10 X vs. 13:50 X	-0.03933	-0.1833 to 0.1046	No
1828	1:10 X vs. 14:0 X	-0.04133	-0.1853 to 0.1026	No
1829	1:10 X vs. 14:1 X	-0.0330	-0.1770 to 0.1110	No
1830	1:10 X vs. 14:10 X	-0.0470	-0.1910 to 0.09696	No
1831	1:10 X vs. 14:40 X	-0.04367	-0.1876 to 0.1003	No
1832	1:10 X vs. 14:50 X	-0.04767	-0.1916 to 0.09629	No
1833	1:10 X vs. 15:0 X	-0.05367	-0.1976 to 0.09029	No
1834	1:10 X vs. 15:1 X	-0.03367	-0.1776 to 0.1103	No
1835	1:10 X vs. 15:10 X	-0.0610	-0.2050 to 0.08296	No
1836	1:10 X vs. 15:40 X	-0.04333	-0.1873 to 0.1006	No
1837	1:10 X vs. 15:50 X	-0.0610	-0.2050 to 0.08296	No
1838	1:10 X vs. 16:0 X	-0.1460	-0.2900 to -0.002040	Yes
1839	1:10 X vs. 16:1 X	-0.0370	-0.1810 to 0.1070	No
1840	1:10 X vs. 16:10 X	-0.06267	-0.2066 to 0.08129	No
1841	1:10 X vs. 16:40 X	-0.0820	-0.2260 to 0.06196	No
1842	1:10 X vs. 16:50 X	-0.1243	-0.2683 to 0.01963	No
1843	1:10 X vs. 17:0 X	-0.1987	-0.3426 to -0.05471	Yes
1844	1:10 X vs. 17:1 X	-0.1130	-0.2570 to 0.03096	No
1845	1:10 X vs. 17:10 X	-0.1697	-0.3136 to -0.02571	Yes

	2way ANOVA<br>Multiple comparisons	A<br>Data Set-A<br>Y	B<br>Data Set-B<br>Y	C<br>Data Set-C<br>Y
**1846**	1:**10 X** vs. 17:**40 X**	-0.0930	-0.2370 to 0.05096	No
**1847**	1:**10 X** vs. 17:**50 X**	-0.1383	-0.2823 to 0.005626	No
**1848**	1:**10 X** vs. 18:**0 X**	-0.2473	-0.3913 to -0.1034	Yes
**1849**	1:**10 X** vs. 18:**1 X**	-0.1557	-0.2996 to -0.01171	Yes
**1850**	1:**10 X** vs. 18:**10 X**	-0.2480	-0.3920 to -0.1040	Yes
**1851**	1:**10 X** vs. 18:**40 X**	-0.09733	-0.2413 to 0.04663	No
**1852**	1:**10 X** vs. 18:**50 X**	-0.1423	-0.2863 to 0.001626	No
**1853**	1:**10 X** vs. 19:**0 X**	-0.2823	-0.4263 to -0.1384	Yes
**1854**	1:**10 X** vs. 19:**1 X**	-0.2703	-0.4143 to -0.1264	Yes
**1855**	1:**10 X** vs. 19:**10 X**	-0.2680	-0.4120 to -0.1240	Yes
**1856**	1:**10 X** vs. 19:**40 X**	-0.1260	-0.2700 to 0.01796	No
**1857**	1:**10 X** vs. 19:**50 X**	-0.1370	-0.2810 to 0.006960	No
**1858**	1:**10 X** vs. 20:**0 X**	-0.3620	-0.5060 to -0.2180	Yes
**1859**	1:**10 X** vs. 20:**1 X**	-0.1813	-0.3253 to -0.03737	Yes
**1860**	1:**10 X** vs. 20:**10 X**	-0.2703	-0.4143 to -0.1264	Yes
**1861**	1:**10 X** vs. 20:**40 X**	-0.1377	-0.2816 to 0.006293	No
**1862**	1:**10 X** vs. 20:**50 X**	-0.1817	-0.3256 to -0.03771	Yes
**1863**	1:**10 X** vs. 21:**0 X**	-0.3853	-0.5293 to -0.2414	Yes
**1864**	1:**10 X** vs. 21:**1 X**	-0.3383	-0.4823 to -0.1944	Yes
**1865**	1:**10 X** vs. 21:**10 X**	-0.3353	-0.4793 to -0.1914	Yes
**1866**	1:**10 X** vs. 21:**40 X**	-0.2107	-0.3546 to -0.06671	Yes
**1867**	1:**10 X** vs. 21:**50 X**	-0.3010	-0.4450 to -0.1570	Yes
**1868**	1:**10 X** vs. 22:**0 X**	-0.4270	-0.5710 to -0.2830	Yes
**1869**	1:**10 X** vs. 22:**1 X**	-0.4217	-0.5656 to -0.2777	Yes
**1870**	1:**10 X** vs. 22:**10 X**	-0.4170	-0.5610 to -0.2730	Yes
**1871**	1:**10 X** vs. 22:**40 X**	-0.3173	-0.4613 to -0.1734	Yes
**1872**	1:**10 X** vs. 22:**50 X**	-0.3800	-0.5240 to -0.2360	Yes
**1873**	1:**10 X** vs. 23:**0 X**	-0.4923	-0.6363 to -0.3484	Yes
**1874**	1:**10 X** vs. 23:**1 X**	-0.6820	-0.8430 to -0.5210	Yes
**1875**	1:**10 X** vs. 23:**10 X**	-0.4317	-0.5756 to -0.2877	Yes
**1876**	1:**10 X** vs. 23:**40 X**	-0.3120	-0.4730 to -0.1510	Yes
**1877**	1:**10 X** vs. 23:**50 X**	-0.3460	-0.4900 to -0.2020	Yes
**1878**	1:**10 X** vs. 24:**0 X**	-0.6097	-0.7536 to -0.4657	Yes
**1879**	1:**10 X** vs. 24:**1 X**	-0.7660	-0.9270 to -0.6050	Yes
**1880**	1:**10 X** vs. 24:**10 X**	-0.5935	-0.7545 to -0.4325	Yes
**1881**	1:**10 X** vs. 24:**40 X**	-0.3283	-0.4723 to -0.1844	Yes
**1882**	1:**10 X** vs. 24:**50 X**	-0.2500	-0.3940 to -0.1060	Yes
**1883**	1:**10 X** vs. 25:**0 X**	-0.9470	-1.108 to -0.7860	Yes
**1884**	1:**10 X** vs. 25:**1 X**	-1.137	-1.297 to -0.9755	Yes
**1885**	1:**10 X** vs. 25:**10 X**	-0.4980	-0.7016 to -0.2944	Yes
**1886**	1:**10 X** vs. 25:**40 X**	-0.3303	-0.4743 to -0.1864	Yes
**1887**	1:**10 X** vs. 25:**50 X**	-0.3047	-0.4486 to -0.1607	Yes
**1888**	1:**10 X** vs. 26:**0 X**	-1.160	-1.320 to -0.9985	Yes
**1889**	1:**10 X** vs. 26:**1 X**	-1.273	-1.433 to -1.112	Yes
**1890**	1:**10 X** vs. 26:**10 X**	-0.9590	-1.120 to -0.7980	Yes

2way ANOVA<br>Multiple comparisons	A<br>Data Set-A<br>Y	B<br>Data Set-B<br>Y	C<br>Data Set-C<br>Y
1891 1:10 X vs. 26:40 X	-0.4047	-0.5486 to -0.2607	Yes
1892 1:10 X vs. 26:50 X	-0.2650	-0.4090 to -0.1210	Yes
1893 1:10 X vs. 27:0 X	-1.347	-1.491 to -1.203	Yes
1894 1:10 X vs. 27:1 X	-1.438	-1.599 to -1.277	Yes
1895 1:10 X vs. 27:10 X	-1.250	-1.411 to -1.089	Yes
1896 1:10 X vs. 27:40 X	-0.4047	-0.5486 to -0.2607	Yes
1897 1:10 X vs. 27:50 X	-0.2643	-0.4083 to -0.1204	Yes
1898 1:10 X vs. 28:0 X	-1.297	-1.457 to -1.136	Yes
1899 1:10 X vs. 28:1 X	-1.337	-1.481 to -1.193	Yes
1900 1:10 X vs. 28:10 X	-1.139	-1.300 to -0.9780	Yes
1901 1:10 X vs. 28:40 X	-0.4170	-0.5610 to -0.2730	Yes
1902 1:10 X vs. 28:50 X	-0.3123	-0.4563 to -0.1684	Yes
1903 1:10 X vs. 29:0 X	-1.778	-1.939 to -1.617	Yes
1904 1:10 X vs. 29:1 X	-1.863	-2.024 to -1.702	Yes
1905 1:10 X vs. 29:10 X	-1.078	-1.238 to -0.9165	Yes
1906 1:10 X vs. 29:40 X	-0.5250	-0.6690 to -0.3810	Yes
1907 1:10 X vs. 29:50 X	-0.3137	-0.4576 to -0.1697	Yes
1908 1:10 X vs. 30:0 X	-1.493	-1.653 to -1.332	Yes
1909 1:10 X vs. 30:1 X	-1.814	-1.975 to -1.653	Yes
1910 1:10 X vs. 30:10 X	-1.152	-1.313 to -0.9910	Yes
1911 1:10 X vs. 30:40 X	-0.4520	-0.5960 to -0.3080	Yes
1912 1:10 X vs. 30:50 X	-0.3660	-0.5100 to -0.2220	Yes
1913 1:10 X vs. 31:0 X	-1.811	-1.971 to -1.650	Yes
1914 1:10 X vs. 31:1 X	-1.801	-1.962 to -1.640	Yes
1915 1:10 X vs. 31:10 X	-1.284	-1.444 to -1.123	Yes
1916 1:10 X vs. 31:40 X	-0.5437	-0.6876 to -0.3997	Yes
1917 1:10 X vs. 31:50 X	-0.3450	-0.4890 to -0.2010	Yes
1918 1:10 X vs. 32:0 X	-2.402	-2.546 to -2.258	Yes
1919 1:10 X vs. 32:1 X	-1.858	-2.019 to -1.697	Yes
1920 1:10 X vs. 32:10 X	-1.714	-1.858 to -1.570	Yes
1921 1:10 X vs. 32:40 X	-0.5400	-0.6840 to -0.3960	Yes
1922 1:10 X vs. 32:50 X	-0.3447	-0.4886 to -0.2007	Yes
1923 1:10 X vs. 33:0 X	-2.406	-2.550 to -2.262	Yes
1924 1:10 X vs. 33:1 X	-2.220	-2.364 to -2.076	Yes
1925 1:10 X vs. 33:10 X	-1.881	-2.025 to -1.737	Yes
1926 1:10 X vs. 33:40 X	-0.5587	-0.7026 to -0.4147	Yes
1927 1:10 X vs. 33:50 X	-0.3597	-0.5036 to -0.2157	Yes
1928 1:10 X vs. 34:0 X	-2.330	-2.474 to -2.186	Yes
1929 1:10 X vs. 34:1 X	-2.338	-2.482 to -2.194	Yes
1930 1:10 X vs. 34:10 X	-1.971	-2.115 to -1.827	Yes
1931 1:10 X vs. 34:40 X	-0.5707	-0.7146 to -0.4267	Yes
1932 1:10 X vs. 34:50 X	-0.5087	-0.6526 to -0.3647	Yes
1933 1:10 X vs. 35:0 X	-2.679	-2.823 to -2.535	Yes
1934 1:10 X vs. 35:1 X	-2.445	-2.589 to -2.301	Yes
1935 1:10 X vs. 35:10 X	-1.933	-2.077 to -1.789	Yes

	**2way ANOVA**<br>Multiple comparisons	**A**<br>Data Set-A<br>**Y**	**B**<br>Data Set-B<br>**Y**	**C**<br>Data Set-C<br>**Y**
**1936**	1:**10 X** vs. 35:**40 X**	-0.5797	-0.7236 to -0.4357	Yes
**1937**	1:**10 X** vs. 35:**50 X**	-0.5213	-0.6653 to -0.3774	Yes
**1938**	1:**10 X** vs. 36:**0 X**	-2.886	-3.046 to -2.725	Yes
**1939**	1:**10 X** vs. 36:**1 X**	-2.581	-2.725 to -2.437	Yes
**1940**	1:**10 X** vs. 36:**10 X**	-1.938	-2.082 to -1.794	Yes
**1941**	1:**10 X** vs. 36:**40 X**	-0.6350	-0.7790 to -0.4910	Yes
**1942**	1:**10 X** vs. 36:**50 X**	-0.4847	-0.6286 to -0.3407	Yes
**1943**	1:**10 X** vs. 37:**0 X**	-3.269	-3.413 to -3.125	Yes
**1944**	1:**10 X** vs. 37:**1 X**	-2.651	-2.795 to -2.507	Yes
**1945**	1:**10 X** vs. 37:**10 X**	-1.940	-2.084 to -1.796	Yes
**1946**	1:**10 X** vs. 37:**40 X**	-0.6723	-0.8163 to -0.5284	Yes
**1947**	1:**10 X** vs. 37:**50 X**	-0.4933	-0.6373 to -0.3494	Yes
**1948**	1:**10 X** vs. 38:**0 X**	-3.214	-3.358 to -3.070	Yes
**1949**	1:**10 X** vs. 38:**1 X**	-2.770	-2.914 to -2.626	Yes
**1950**	1:**10 X** vs. 38:**10 X**	-2.215	-2.359 to -2.071	Yes
**1951**	1:**10 X** vs. 38:**40 X**	-0.6767	-0.8206 to -0.5327	Yes
**1952**	1:**10 X** vs. 38:**50 X**	-0.4973	-0.6413 to -0.3534	Yes
**1953**	1:**10 X** vs. 39:**0 X**	-3.445	-3.589 to -3.301	Yes
**1954**	1:**10 X** vs. 39:**1 X**	-3.337	-3.481 to -3.193	Yes
**1955**	1:**10 X** vs. 39:**10 X**	-2.360	-2.504 to -2.216	Yes
**1956**	1:**10 X** vs. 39:**40 X**	-0.6827	-0.8266 to -0.5387	Yes
**1957**	1:**10 X** vs. 39:**50 X**	-0.5283	-0.6723 to -0.3844	Yes
**1958**	1:**10 X** vs. 40:**0 X**	-3.486	-3.630 to -3.342	Yes
**1959**	1:**10 X** vs. 40:**1 X**	-3.412	-3.556 to -3.268	Yes
**1960**	1:**10 X** vs. 40:**10 X**	-2.617	-2.761 to -2.473	Yes
**1961**	1:**10 X** vs. 40:**40 X**	-0.6873	-0.8313 to -0.5434	Yes
**1962**	1:**10 X** vs. 40:**50 X**	-0.5347	-0.6786 to -0.3907	Yes
**1963**	1:**10 X** vs. 41:**0 X**	-3.527	-3.671 to -3.383	Yes
**1964**	1:**10 X** vs. 41:**1 X**	-3.526	-3.670 to -3.382	Yes
**1965**	1:**10 X** vs. 41:**10 X**	-2.670	-2.814 to -2.526	Yes
**1966**	1:**10 X** vs. 41:**40 X**	-0.6347	-0.7786 to -0.4907	Yes
**1967**	1:**10 X** vs. 41:**50 X**	-0.5473	-0.6913 to -0.4034	Yes
**1968**	1:**10 X** vs. 42:**0 X**	-3.669	-3.813 to -3.525	Yes
**1969**	1:**10 X** vs. 42:**1 X**	-3.688	-3.832 to -3.544	Yes
**1970**	1:**10 X** vs. 42:**10 X**	-2.671	-2.815 to -2.527	Yes
**1971**	1:**10 X** vs. 42:**40 X**	-0.6473	-0.7913 to -0.5034	Yes
**1972**	1:**10 X** vs. 42:**50 X**	-0.5507	-0.6946 to -0.4067	Yes
**1973**	1:**10 X** vs. 43:**0 X**	-3.759	-3.903 to -3.615	Yes
**1974**	1:**10 X** vs. 43:**1 X**	-3.673	-3.817 to -3.529	Yes
**1975**	1:**10 X** vs. 43:**10 X**	-3.389	-3.533 to -3.245	Yes
**1976**	1:**10 X** vs. 43:**40 X**	-0.6457	-0.7896 to -0.5017	Yes
**1977**	1:**10 X** vs. 43:**50 X**	-0.5510	-0.6950 to -0.4070	Yes
**1978**	1:**10 X** vs. 44:**0 X**	-3.783	-3.927 to -3.639	Yes
**1979**	1:**10 X** vs. 44:**1 X**	-3.672	-3.816 to -3.528	Yes
**1980**	1:**10 X** vs. 44:**10 X**	-3.452	-3.596 to -3.308	Yes

	2way ANOVA<br>Multiple comparisons	A<br>Data Set-A<br>Y	B<br>Data Set-B<br>Y	C<br>Data Set-C<br>Y
1981	1:10 X vs. 44:40 X	-0.6477	-0.7916 to -0.5037	Yes
1982	1:10 X vs. 44:50 X	-0.4833	-0.6273 to -0.3394	Yes
1983	1:10 X vs. 45:0 X	-3.647	-3.791 to -3.503	Yes
1984	1:10 X vs. 45:1 X	-3.449	-3.593 to -3.305	Yes
1985	1:10 X vs. 45:10 X	-3.332	-3.476 to -3.188	Yes
1986	1:10 X vs. 45:40 X	-0.6490	-0.7930 to -0.5050	Yes
1987	1:10 X vs. 45:50 X	-0.4803	-0.6243 to -0.3364	Yes
1988	1:10 X vs. 46:0 X	-3.452	-3.596 to -3.308	Yes
1989	1:10 X vs. 46:1 X	-3.419	-3.563 to -3.275	Yes
1990	1:10 X vs. 46:10 X	-3.327	-3.471 to -3.183	Yes
1991	1:10 X vs. 46:40 X	-0.6627	-0.8066 to -0.5187	Yes
1992	1:10 X vs. 46:50 X	-0.4733	-0.6173 to -0.3294	Yes
1993	1:10 X vs. 47:0 X	-3.427	-3.571 to -3.283	Yes
1994	1:10 X vs. 47:1 X	-3.331	-3.475 to -3.187	Yes
1995	1:10 X vs. 47:10 X	-3.313	-3.457 to -3.169	Yes
1996	1:10 X vs. 47:40 X	-0.6727	-0.8166 to -0.5287	Yes
1997	1:10 X vs. 47:50 X	-0.4313	-0.5753 to -0.2874	Yes
1998	1:10 X vs. 48:0 X	-3.415	-3.575 to -3.254	Yes
1999	1:10 X vs. 48:1 X	-3.318	-3.462 to -3.174	Yes
2000	1:10 X vs. 48:10 X	-3.313	-3.457 to -3.169	Yes
2001	1:10 X vs. 48:40 X	-0.6790	-0.8230 to -0.5350	Yes
2002	1:10 X vs. 48:50 X	-0.4387	-0.5826 to -0.2947	Yes
2003	1:10 X vs. 49:0 X	-3.415	-3.559 to -3.271	Yes
2004	1:10 X vs. 49:1 X	-3.318	-3.462 to -3.174	Yes
2005	1:10 X vs. 49:10 X	-3.313	-3.457 to -3.169	Yes
2006	1:10 X vs. 49:40 X	-0.6833	-0.8273 to -0.5394	Yes
2007	1:10 X vs. 49:50 X	-0.4380	-0.5820 to -0.2940	Yes
2008	1:10 X vs. 50:0 X	-3.415	-3.559 to -3.271	Yes
2009	1:10 X vs. 50:1 X	-3.318	-3.462 to -3.174	Yes
2010	1:10 X vs. 50:10 X	-3.313	-3.457 to -3.169	Yes
2011	1:10 X vs. 50:40 X	-0.6910	-0.8350 to -0.5470	Yes
2012	1:10 X vs. 50:50 X	-0.4377	-0.5816 to -0.2937	Yes
2013	1:40 X vs. 1:50 X	0.002667	-0.1413 to 0.1466	No
2014	1:40 X vs. 2:0 X	-0.0006667	-0.1446 to 0.1433	No
2015	1:40 X vs. 2:1 X	-0.005667	-0.1496 to 0.1383	No
2016	1:40 X vs. 2:10 X	0.0003333	-0.1436 to 0.1443	No
2017	1:40 X vs. 2:40 X	-0.0003333	-0.1443 to 0.1436	No
2018	1:40 X vs. 2:50 X	0.002667	-0.1413 to 0.1466	No
2019	1:40 X vs. 3:0 X	-0.001667	-0.1456 to 0.1423	No
2020	1:40 X vs. 3:1 X	-0.004000	-0.1480 to 0.1400	No
2021	1:40 X vs. 3:10 X	0.0006667	-0.1433 to 0.1446	No
2022	1:40 X vs. 3:40 X	0.0030	-0.1410 to 0.1470	No
2023	1:40 X vs. 3:50 X	0.0040	-0.1400 to 0.1480	No
2024	1:40 X vs. 4:0 X	-0.0060	-0.1500 to 0.1380	No
2025	1:40 X vs. 4:1 X	-0.004667	-0.1486 to 0.1393	No

2way ANOVA<br>Multiple comparisons		A<br>Data Set-A<br>Y	B<br>Data Set-B<br>Y	C<br>Data Set-C<br>Y
2026	1:**40 X** vs. 4:**10 X**	-0.01933	-0.1633 to 0.1246	No
2027	1:**40 X** vs. 4:**40 X**	0.0010	-0.1430 to 0.1450	No
2028	1:**40 X** vs. 4:**50 X**	0.0030	-0.1410 to 0.1470	No
2029	1:**40 X** vs. 5:**0 X**	-0.0070	-0.1510 to 0.1370	No
2030	1:**40 X** vs. 5:**1 X**	-0.005000	-0.1490 to 0.1390	No
2031	1:**40 X** vs. 5:**10 X**	-0.0200	-0.1640 to 0.1240	No
2032	1:**40 X** vs. 5:**40 X**	0.0010	-0.1430 to 0.1450	No
2033	1:**40 X** vs. 5:**50 X**	0.0020	-0.1420 to 0.1460	No
2034	1:**40 X** vs. 6:**0 X**	-0.006333	-0.1503 to 0.1376	No
2035	1:**40 X** vs. 6:**1 X**	-0.005333	-0.1493 to 0.1386	No
2036	1:**40 X** vs. 6:**10 X**	-0.0200	-0.1640 to 0.1240	No
2037	1:**40 X** vs. 6:**40 X**	-0.001333	-0.1453 to 0.1426	No
2038	1:**40 X** vs. 6:**50 X**	0.0003333	-0.1436 to 0.1443	No
2039	1:**40 X** vs. 7:**0 X**	-0.008333	-0.1523 to 0.1356	No
2040	1:**40 X** vs. 7:**1 X**	0.0003333	-0.1436 to 0.1443	No
2041	1:**40 X** vs. 7:**10 X**	-0.01167	-0.1556 to 0.1323	No
2042	1:**40 X** vs. 7:**40 X**	-0.0030	-0.1470 to 0.1410	No
2043	1:**40 X** vs. 7:**50 X**	-0.0010	-0.1450 to 0.1430	No
2044	1:**40 X** vs. 8:**0 X**	-0.0130	-0.1570 to 0.1310	No
2045	1:**40 X** vs. 8:**1 X**	0.0003333	-0.1436 to 0.1443	No
2046	1:**40 X** vs. 8:**10 X**	-0.007000	-0.1510 to 0.1370	No
2047	1:**40 X** vs. 8:**40 X**	-0.007667	-0.1516 to 0.1363	No
2048	1:**40 X** vs. 8:**50 X**	-0.004667	-0.1486 to 0.1393	No
2049	1:**40 X** vs. 9:**0 X**	-0.0210	-0.1650 to 0.1230	No
2050	1:**40 X** vs. 9:**1 X**	-0.0290	-0.1730 to 0.1150	No
2051	1:**40 X** vs. 9:**10 X**	-0.0300	-0.1740 to 0.1140	No
2052	1:**40 X** vs. 9:**40 X**	-0.01233	-0.1563 to 0.1316	No
2053	1:**40 X** vs. 9:**50 X**	-0.005667	-0.1496 to 0.1383	No
2054	1:**40 X** vs. 10:**0 X**	-0.02267	-0.1666 to 0.1213	No
2055	1:**40 X** vs. 10:**1 X**	-0.02967	-0.1736 to 0.1143	No
2056	1:**40 X** vs. 10:**10 X**	-0.03233	-0.1763 to 0.1116	No
2057	1:**40 X** vs. 10:**40 X**	-0.0220	-0.1660 to 0.1220	No
2058	1:**40 X** vs. 10:**50 X**	-0.008667	-0.1526 to 0.1353	No
2059	1:**40 X** vs. 11:**0 X**	-0.03233	-0.1763 to 0.1116	No
2060	1:**40 X** vs. 11:**1 X**	-0.0340	-0.1780 to 0.1100	No
2061	1:**40 X** vs. 11:**10 X**	-0.03433	-0.1783 to 0.1096	No
2062	1:**40 X** vs. 11:**40 X**	-0.0260	-0.1700 to 0.1180	No
2063	1:**40 X** vs. 11:**50 X**	-0.0140	-0.1580 to 0.1300	No
2064	1:**40 X** vs. 12:**0 X**	-0.0330	-0.1770 to 0.1110	No
2065	1:**40 X** vs. 12:**1 X**	-0.01533	-0.1593 to 0.1286	No
2066	1:**40 X** vs. 12:**10 X**	-0.0330	-0.1770 to 0.1110	No
2067	1:**40 X** vs. 12:**40 X**	-0.01933	-0.1633 to 0.1246	No
2068	1:**40 X** vs. 12:**50 X**	-0.02267	-0.1666 to 0.1213	No
2069	1:**40 X** vs. 13:**0 X**	-0.0270	-0.1710 to 0.1170	No
2070	1:**40 X** vs. 13:**1 X**	-0.01433	-0.1583 to 0.1296	No

	2way ANOVA<br>Multiple comparisons	A<br>Data Set-A<br>Y	B<br>Data Set-B<br>Y	C<br>Data Set-C<br>Y
2071	1:40 X vs. 13:10 X	-0.03633	-0.1803 to 0.1076	No
2072	1:40 X vs. 13:40 X	-0.03267	-0.1766 to 0.1113	No
2073	1:40 X vs. 13:50 X	-0.03367	-0.1776 to 0.1103	No
2074	1:40 X vs. 14:0 X	-0.03567	-0.1796 to 0.1083	No
2075	1:40 X vs. 14:1 X	-0.02733	-0.1713 to 0.1166	No
2076	1:40 X vs. 14:10 X	-0.04133	-0.1853 to 0.1026	No
2077	1:40 X vs. 14:40 X	-0.0380	-0.1820 to 0.1060	No
2078	1:40 X vs. 14:50 X	-0.0420	-0.1860 to 0.1020	No
2079	1:40 X vs. 15:0 X	-0.0480	-0.1920 to 0.09596	No
2080	1:40 X vs. 15:1 X	-0.0280	-0.1720 to 0.1160	No
2081	1:40 X vs. 15:10 X	-0.05533	-0.1993 to 0.08863	No
2082	1:40 X vs. 15:40 X	-0.03767	-0.1816 to 0.1063	No
2083	1:40 X vs. 15:50 X	-0.05533	-0.1993 to 0.08863	No
2084	1:40 X vs. 16:0 X	-0.1403	-0.2843 to 0.003626	No
2085	1:40 X vs. 16:1 X	-0.03133	-0.1753 to 0.1126	No
2086	1:40 X vs. 16:10 X	-0.0570	-0.2010 to 0.08696	No
2087	1:40 X vs. 16:40 X	-0.07633	-0.2203 to 0.06763	No
2088	1:40 X vs. 16:50 X	-0.1187	-0.2626 to 0.02529	No
2089	1:40 X vs. 17:0 X	-0.1930	-0.3370 to -0.04904	Yes
2090	1:40 X vs. 17:1 X	-0.1073	-0.2513 to 0.03663	No
2091	1:40 X vs. 17:10 X	-0.1640	-0.3080 to -0.02004	Yes
2092	1:40 X vs. 17:40 X	-0.08733	-0.2313 to 0.05663	No
2093	1:40 X vs. 17:50 X	-0.1327	-0.2766 to 0.01129	No
2094	1:40 X vs. 18:0 X	-0.2417	-0.3856 to -0.09771	Yes
2095	1:40 X vs. 18:1 X	-0.1500	-0.2940 to -0.006040	Yes
2096	1:40 X vs. 18:10 X	-0.2423	-0.3863 to -0.09837	Yes
2097	1:40 X vs. 18:40 X	-0.09167	-0.2356 to 0.05229	No
2098	1:40 X vs. 18:50 X	-0.1367	-0.2806 to 0.007293	No
2099	1:40 X vs. 19:0 X	-0.2767	-0.4206 to -0.1327	Yes
2100	1:40 X vs. 19:1 X	-0.2647	-0.4086 to -0.1207	Yes
2101	1:40 X vs. 19:10 X	-0.2623	-0.4063 to -0.1184	Yes
2102	1:40 X vs. 19:40 X	-0.1203	-0.2643 to 0.02363	No
2103	1:40 X vs. 19:50 X	-0.1313	-0.2753 to 0.01263	No
2104	1:40 X vs. 20:0 X	-0.3563	-0.5003 to -0.2124	Yes
2105	1:40 X vs. 20:1 X	-0.1757	-0.3196 to -0.03171	Yes
2106	1:40 X vs. 20:10 X	-0.2647	-0.4086 to -0.1207	Yes
2107	1:40 X vs. 20:40 X	-0.1320	-0.2760 to 0.01196	No
2108	1:40 X vs. 20:50 X	-0.1760	-0.3200 to -0.03204	Yes
2109	1:40 X vs. 21:0 X	-0.3797	-0.5236 to -0.2357	Yes
2110	1:40 X vs. 21:1 X	-0.3327	-0.4766 to -0.1887	Yes
2111	1:40 X vs. 21:10 X	-0.3297	-0.4736 to -0.1857	Yes
2112	1:40 X vs. 21:40 X	-0.2050	-0.3490 to -0.06104	Yes
2113	1:40 X vs. 21:50 X	-0.2953	-0.4393 to -0.1514	Yes
2114	1:40 X vs. 22:0 X	-0.4213	-0.5653 to -0.2774	Yes
2115	1:40 X vs. 22:1 X	-0.4160	-0.5600 to -0.2720	Yes

	2way ANOVA<br>Multiple comparisons	A<br>Data Set-A<br>Y	B<br>Data Set-B<br>Y	C<br>Data Set-C<br>Y
2116	1:40 X vs. 22:10 X	-0.4113	-0.5553 to -0.2674	Yes
2117	1:40 X vs. 22:40 X	-0.3117	-0.4556 to -0.1677	Yes
2118	1:40 X vs. 22:50 X	-0.3743	-0.5183 to -0.2304	Yes
2119	1:40 X vs. 23:0 X	-0.4867	-0.6306 to -0.3427	Yes
2120	1:40 X vs. 23:1 X	-0.6763	-0.8373 to -0.5154	Yes
2121	1:40 X vs. 23:10 X	-0.4260	-0.5700 to -0.2820	Yes
2122	1:40 X vs. 23:40 X	-0.3063	-0.4673 to -0.1454	Yes
2123	1:40 X vs. 23:50 X	-0.3403	-0.4843 to -0.1964	Yes
2124	1:40 X vs. 24:0 X	-0.6040	-0.7480 to -0.4600	Yes
2125	1:40 X vs. 24:1 X	-0.7603	-0.9213 to -0.5994	Yes
2126	1:40 X vs. 24:10 X	-0.5878	-0.7488 to -0.4269	Yes
2127	1:40 X vs. 24:40 X	-0.3227	-0.4666 to -0.1787	Yes
2128	1:40 X vs. 24:50 X	-0.2443	-0.3883 to -0.1004	Yes
2129	1:40 X vs. 25:0 X	-0.9413	-1.102 to -0.7804	Yes
2130	1:40 X vs. 25:1 X	-1.131	-1.292 to -0.9699	Yes
2131	1:40 X vs. 25:10 X	-0.4923	-0.6959 to -0.2887	Yes
2132	1:40 X vs. 25:40 X	-0.3247	-0.4686 to -0.1807	Yes
2133	1:40 X vs. 25:50 X	-0.2990	-0.4430 to -0.1550	Yes
2134	1:40 X vs. 26:0 X	-1.154	-1.315 to -0.9929	Yes
2135	1:40 X vs. 26:1 X	-1.267	-1.428 to -1.106	Yes
2136	1:40 X vs. 26:10 X	-0.9533	-1.114 to -0.7924	Yes
2137	1:40 X vs. 26:40 X	-0.3990	-0.5430 to -0.2550	Yes
2138	1:40 X vs. 26:50 X	-0.2593	-0.4033 to -0.1154	Yes
2139	1:40 X vs. 27:0 X	-1.342	-1.486 to -1.198	Yes
2140	1:40 X vs. 27:1 X	-1.432	-1.593 to -1.271	Yes
2141	1:40 X vs. 27:10 X	-1.244	-1.405 to -1.083	Yes
2142	1:40 X vs. 27:40 X	-0.3990	-0.5430 to -0.2550	Yes
2143	1:40 X vs. 27:50 X	-0.2587	-0.4026 to -0.1147	Yes
2144	1:40 X vs. 28:0 X	-1.291	-1.452 to -1.130	Yes
2145	1:40 X vs. 28:1 X	-1.332	-1.476 to -1.188	Yes
2146	1:40 X vs. 28:10 X	-1.133	-1.294 to -0.9724	Yes
2147	1:40 X vs. 28:40 X	-0.4113	-0.5553 to -0.2674	Yes
2148	1:40 X vs. 28:50 X	-0.3067	-0.4506 to -0.1627	Yes
2149	1:40 X vs. 29:0 X	-1.772	-1.933 to -1.611	Yes
2150	1:40 X vs. 29:1 X	-1.857	-2.018 to -1.696	Yes
2151	1:40 X vs. 29:10 X	-1.072	-1.233 to -0.9109	Yes
2152	1:40 X vs. 29:40 X	-0.5193	-0.6633 to -0.3754	Yes
2153	1:40 X vs. 29:50 X	-0.3080	-0.4520 to -0.1640	Yes
2154	1:40 X vs. 30:0 X	-1.487	-1.648 to -1.326	Yes
2155	1:40 X vs. 30:1 X	-1.808	-1.969 to -1.647	Yes
2156	1:40 X vs. 30:10 X	-1.146	-1.307 to -0.9854	Yes
2157	1:40 X vs. 30:40 X	-0.4463	-0.5903 to -0.3024	Yes
2158	1:40 X vs. 30:50 X	-0.3603	-0.5043 to -0.2164	Yes
2159	1:40 X vs. 31:0 X	-1.805	-1.966 to -1.644	Yes
2160	1:40 X vs. 31:1 X	-1.795	-1.956 to -1.634	Yes

	2way ANOVA<br>Multiple comparisons	A<br>Data Set-A<br>Y	B<br>Data Set-B<br>Y	C<br>Data Set-C<br>Y
**2161**	1:**40 X** vs. 31:**10 X**	-1.278	-1.439 to -1.117	Yes
**2162**	1:**40 X** vs. 31:**40 X**	-0.5380	-0.6820 to -0.3940	Yes
**2163**	1:**40 X** vs. 31:**50 X**	-0.3393	-0.4833 to -0.1954	Yes
**2164**	1:**40 X** vs. 32:**0 X**	-2.396	-2.540 to -2.252	Yes
**2165**	1:**40 X** vs. 32:**1 X**	-1.852	-2.013 to -1.691	Yes
**2166**	1:**40 X** vs. 32:**10 X**	-1.708	-1.852 to -1.564	Yes
**2167**	1:**40 X** vs. 32:**40 X**	-0.5343	-0.6783 to -0.3904	Yes
**2168**	1:**40 X** vs. 32:**50 X**	-0.3390	-0.4830 to -0.1950	Yes
**2169**	1:**40 X** vs. 33:**0 X**	-2.400	-2.544 to -2.256	Yes
**2170**	1:**40 X** vs. 33:**1 X**	-2.215	-2.359 to -2.071	Yes
**2171**	1:**40 X** vs. 33:**10 X**	-1.875	-2.019 to -1.731	Yes
**2172**	1:**40 X** vs. 33:**40 X**	-0.5530	-0.6970 to -0.4090	Yes
**2173**	1:**40 X** vs. 33:**50 X**	-0.3540	-0.4980 to -0.2100	Yes
**2174**	1:**40 X** vs. 34:**0 X**	-2.325	-2.469 to -2.181	Yes
**2175**	1:**40 X** vs. 34:**1 X**	-2.332	-2.476 to -2.188	Yes
**2176**	1:**40 X** vs. 34:**10 X**	-1.965	-2.109 to -1.821	Yes
**2177**	1:**40 X** vs. 34:**40 X**	-0.5650	-0.7090 to -0.4210	Yes
**2178**	1:**40 X** vs. 34:**50 X**	-0.5030	-0.6470 to -0.3590	Yes
**2179**	1:**40 X** vs. 35:**0 X**	-2.673	-2.817 to -2.529	Yes
**2180**	1:**40 X** vs. 35:**1 X**	-2.439	-2.583 to -2.295	Yes
**2181**	1:**40 X** vs. 35:**10 X**	-1.927	-2.071 to -1.783	Yes
**2182**	1:**40 X** vs. 35:**40 X**	-0.5740	-0.7180 to -0.4300	Yes
**2183**	1:**40 X** vs. 35:**50 X**	-0.5157	-0.6596 to -0.3717	Yes
**2184**	1:**40 X** vs. 36:**0 X**	-2.880	-3.041 to -2.719	Yes
**2185**	1:**40 X** vs. 36:**1 X**	-2.576	-2.720 to -2.432	Yes
**2186**	1:**40 X** vs. 36:**10 X**	-1.933	-2.077 to -1.789	Yes
**2187**	1:**40 X** vs. 36:**40 X**	-0.6293	-0.7733 to -0.4854	Yes
**2188**	1:**40 X** vs. 36:**50 X**	-0.4790	-0.6230 to -0.3350	Yes
**2189**	1:**40 X** vs. 37:**0 X**	-3.264	-3.408 to -3.120	Yes
**2190**	1:**40 X** vs. 37:**1 X**	-2.646	-2.790 to -2.502	Yes
**2191**	1:**40 X** vs. 37:**10 X**	-1.934	-2.078 to -1.790	Yes
**2192**	1:**40 X** vs. 37:**40 X**	-0.6667	-0.8106 to -0.5227	Yes
**2193**	1:**40 X** vs. 37:**50 X**	-0.4877	-0.6316 to -0.3437	Yes
**2194**	1:**40 X** vs. 38:**0 X**	-3.209	-3.353 to -3.065	Yes
**2195**	1:**40 X** vs. 38:**1 X**	-2.764	-2.908 to -2.620	Yes
**2196**	1:**40 X** vs. 38:**10 X**	-2.210	-2.354 to -2.066	Yes
**2197**	1:**40 X** vs. 38:**40 X**	-0.6710	-0.8150 to -0.5270	Yes
**2198**	1:**40 X** vs. 38:**50 X**	-0.4917	-0.6356 to -0.3477	Yes
**2199**	1:**40 X** vs. 39:**0 X**	-3.439	-3.583 to -3.295	Yes
**2200**	1:**40 X** vs. 39:**1 X**	-3.331	-3.475 to -3.187	Yes
**2201**	1:**40 X** vs. 39:**10 X**	-2.355	-2.499 to -2.211	Yes
**2202**	1:**40 X** vs. 39:**40 X**	-0.6770	-0.8210 to -0.5330	Yes
**2203**	1:**40 X** vs. 39:**50 X**	-0.5227	-0.6666 to -0.3787	Yes
**2204**	1:**40 X** vs. 40:**0 X**	-3.480	-3.624 to -3.336	Yes
**2205**	1:**40 X** vs. 40:**1 X**	-3.406	-3.550 to -3.262	Yes

	2way ANOVA<br>Multiple comparisons	A<br>Data Set-A<br>Y	B<br>Data Set-B<br>Y	C<br>Data Set-C<br>Y
2206	1:**40 X** vs. 40:**10 X**	-2.611	-2.755 to -2.467	Yes
2207	1:**40 X** vs. 40:**40 X**	-0.6817	-0.8256 to -0.5377	Yes
2208	1:**40 X** vs. 40:**50 X**	-0.5290	-0.6730 to -0.3850	Yes
2209	1:**40 X** vs. 41:**0 X**	-3.521	-3.665 to -3.377	Yes
2210	1:**40 X** vs. 41:**1 X**	-3.520	-3.664 to -3.376	Yes
2211	1:**40 X** vs. 41:**10 X**	-2.665	-2.809 to -2.521	Yes
2212	1:**40 X** vs. 41:**40 X**	-0.6290	-0.7730 to -0.4850	Yes
2213	1:**40 X** vs. 41:**50 X**	-0.5417	-0.6856 to -0.3977	Yes
2214	1:**40 X** vs. 42:**0 X**	-3.663	-3.807 to -3.519	Yes
2215	1:**40 X** vs. 42:**1 X**	-3.683	-3.827 to -3.539	Yes
2216	1:**40 X** vs. 42:**10 X**	-2.665	-2.809 to -2.521	Yes
2217	1:**40 X** vs. 42:**40 X**	-0.6417	-0.7856 to -0.4977	Yes
2218	1:**40 X** vs. 42:**50 X**	-0.5450	-0.6890 to -0.4010	Yes
2219	1:**40 X** vs. 43:**0 X**	-3.753	-3.897 to -3.609	Yes
2220	1:**40 X** vs. 43:**1 X**	-3.667	-3.811 to -3.523	Yes
2221	1:**40 X** vs. 43:**10 X**	-3.383	-3.527 to -3.239	Yes
2222	1:**40 X** vs. 43:**40 X**	-0.6400	-0.7840 to -0.4960	Yes
2223	1:**40 X** vs. 43:**50 X**	-0.5453	-0.6893 to -0.4014	Yes
2224	1:**40 X** vs. 44:**0 X**	-3.777	-3.921 to -3.633	Yes
2225	1:**40 X** vs. 44:**1 X**	-3.667	-3.811 to -3.523	Yes
2226	1:**40 X** vs. 44:**10 X**	-3.446	-3.590 to -3.302	Yes
2227	1:**40 X** vs. 44:**40 X**	-0.6420	-0.7860 to -0.4980	Yes
2228	1:**40 X** vs. 44:**50 X**	-0.4777	-0.6216 to -0.3337	Yes
2229	1:**40 X** vs. 45:**0 X**	-3.642	-3.786 to -3.498	Yes
2230	1:**40 X** vs. 45:**1 X**	-3.444	-3.588 to -3.300	Yes
2231	1:**40 X** vs. 45:**10 X**	-3.326	-3.470 to -3.182	Yes
2232	1:**40 X** vs. 45:**40 X**	-0.6433	-0.7873 to -0.4994	Yes
2233	1:**40 X** vs. 45:**50 X**	-0.4747	-0.6186 to -0.3307	Yes
2234	1:**40 X** vs. 46:**0 X**	-3.446	-3.590 to -3.302	Yes
2235	1:**40 X** vs. 46:**1 X**	-3.413	-3.557 to -3.269	Yes
2236	1:**40 X** vs. 46:**10 X**	-3.321	-3.465 to -3.177	Yes
2237	1:**40 X** vs. 46:**40 X**	-0.6570	-0.8010 to -0.5130	Yes
2238	1:**40 X** vs. 46:**50 X**	-0.4677	-0.6116 to -0.3237	Yes
2239	1:**40 X** vs. 47:**0 X**	-3.422	-3.566 to -3.278	Yes
2240	1:**40 X** vs. 47:**1 X**	-3.326	-3.470 to -3.182	Yes
2241	1:**40 X** vs. 47:**10 X**	-3.307	-3.451 to -3.163	Yes
2242	1:**40 X** vs. 47:**40 X**	-0.6670	-0.8110 to -0.5230	Yes
2243	1:**40 X** vs. 47:**50 X**	-0.4257	-0.5696 to -0.2817	Yes
2244	1:**40 X** vs. 48:**0 X**	-3.409	-3.570 to -3.248	Yes
2245	1:**40 X** vs. 48:**1 X**	-3.313	-3.457 to -3.169	Yes
2246	1:**40 X** vs. 48:**10 X**	-3.307	-3.451 to -3.163	Yes
2247	1:**40 X** vs. 48:**40 X**	-0.6733	-0.8173 to -0.5294	Yes
2248	1:**40 X** vs. 48:**50 X**	-0.4330	-0.5770 to -0.2890	Yes
2249	1:**40 X** vs. 49:**0 X**	-3.409	-3.553 to -3.265	Yes
2250	1:**40 X** vs. 49:**1 X**	-3.313	-3.457 to -3.169	Yes

	2way ANOVA Multiple comparisons	A Data Set-A Y	B Data Set-B Y	C Data Set-C Y
2251	1:40 X vs. 49:10 X	-3.307	-3.451 to -3.163	Yes
2252	1:40 X vs. 49:40 X	-0.6777	-0.8216 to -0.5337	Yes
2253	1:40 X vs. 49:50 X	-0.4323	-0.5763 to -0.2884	Yes
2254	1:40 X vs. 50:0 X	-3.409	-3.553 to -3.265	Yes
2255	1:40 X vs. 50:1 X	-3.313	-3.457 to -3.169	Yes
2256	1:40 X vs. 50:10 X	-3.307	-3.451 to -3.163	Yes
2257	1:40 X vs. 50:40 X	-0.6853	-0.8293 to -0.5414	Yes
2258	1:40 X vs. 50:50 X	-0.4320	-0.5760 to -0.2880	Yes
2259	1:50 X vs. 2:0 X	-0.003333	-0.1473 to 0.1406	No
2260	1:50 X vs. 2:1 X	-0.008333	-0.1523 to 0.1356	No
2261	1:50 X vs. 2:10 X	-0.002333	-0.1463 to 0.1416	No
2262	1:50 X vs. 2:40 X	-0.0030	-0.1470 to 0.1410	No
2263	1:50 X vs. 2:50 X	0.0	-0.1440 to 0.1440	No
2264	1:50 X vs. 3:0 X	-0.004333	-0.1483 to 0.1396	No
2265	1:50 X vs. 3:1 X	-0.006667	-0.1506 to 0.1373	No
2266	1:50 X vs. 3:10 X	-0.0020	-0.1460 to 0.1420	No
2267	1:50 X vs. 3:40 X	0.0003333	-0.1436 to 0.1443	No
2268	1:50 X vs. 3:50 X	0.001333	-0.1426 to 0.1453	No
2269	1:50 X vs. 4:0 X	-0.008667	-0.1526 to 0.1353	No
2270	1:50 X vs. 4:1 X	-0.007333	-0.1513 to 0.1366	No
2271	1:50 X vs. 4:10 X	-0.0220	-0.1660 to 0.1220	No
2272	1:50 X vs. 4:40 X	-0.001667	-0.1456 to 0.1423	No
2273	1:50 X vs. 4:50 X	0.0003333	-0.1436 to 0.1443	No
2274	1:50 X vs. 5:0 X	-0.009667	-0.1536 to 0.1343	No
2275	1:50 X vs. 5:1 X	-0.007667	-0.1516 to 0.1363	No
2276	1:50 X vs. 5:10 X	-0.02267	-0.1666 to 0.1213	No
2277	1:50 X vs. 5:40 X	-0.001667	-0.1456 to 0.1423	No
2278	1:50 X vs. 5:50 X	-0.0006667	-0.1446 to 0.1433	No
2279	1:50 X vs. 6:0 X	-0.0090	-0.1530 to 0.1350	No
2280	1:50 X vs. 6:1 X	-0.008000	-0.1520 to 0.1360	No
2281	1:50 X vs. 6:10 X	-0.02267	-0.1666 to 0.1213	No
2282	1:50 X vs. 6:40 X	-0.0040	-0.1480 to 0.1400	No
2283	1:50 X vs. 6:50 X	-0.002333	-0.1463 to 0.1416	No
2284	1:50 X vs. 7:0 X	-0.0110	-0.1550 to 0.1330	No
2285	1:50 X vs. 7:1 X	-0.002333	-0.1463 to 0.1416	No
2286	1:50 X vs. 7:10 X	-0.01433	-0.1583 to 0.1296	No
2287	1:50 X vs. 7:40 X	-0.005667	-0.1496 to 0.1383	No
2288	1:50 X vs. 7:50 X	-0.003667	-0.1476 to 0.1403	No
2289	1:50 X vs. 8:0 X	-0.01567	-0.1596 to 0.1283	No
2290	1:50 X vs. 8:1 X	-0.002333	-0.1463 to 0.1416	No
2291	1:50 X vs. 8:10 X	-0.009667	-0.1536 to 0.1343	No
2292	1:50 X vs. 8:40 X	-0.01033	-0.1543 to 0.1336	No
2293	1:50 X vs. 8:50 X	-0.007333	-0.1513 to 0.1366	No
2294	1:50 X vs. 9:0 X	-0.02367	-0.1676 to 0.1203	No
2295	1:50 X vs. 9:1 X	-0.03167	-0.1756 to 0.1123	No

	2way ANOVA<br>Multiple comparisons	A<br>Data Set-A<br>Y	B<br>Data Set-B<br>Y	C<br>Data Set-C<br>Y
2296	1:50 X vs. 9:10 X	-0.03267	-0.1766 to 0.1113	No
2297	1:50 X vs. 9:40 X	-0.0150	-0.1590 to 0.1290	No
2298	1:50 X vs. 9:50 X	-0.008333	-0.1523 to 0.1356	No
2299	1:50 X vs. 10:0 X	-0.02533	-0.1693 to 0.1186	No
2300	1:50 X vs. 10:1 X	-0.03233	-0.1763 to 0.1116	No
2301	1:50 X vs. 10:10 X	-0.0350	-0.1790 to 0.1090	No
2302	1:50 X vs. 10:40 X	-0.02467	-0.1686 to 0.1193	No
2303	1:50 X vs. 10:50 X	-0.01133	-0.1553 to 0.1326	No
2304	1:50 X vs. 11:0 X	-0.0350	-0.1790 to 0.1090	No
2305	1:50 X vs. 11:1 X	-0.03667	-0.1806 to 0.1073	No
2306	1:50 X vs. 11:10 X	-0.0370	-0.1810 to 0.1070	No
2307	1:50 X vs. 11:40 X	-0.02867	-0.1726 to 0.1153	No
2308	1:50 X vs. 11:50 X	-0.01667	-0.1606 to 0.1273	No
2309	1:50 X vs. 12:0 X	-0.03567	-0.1796 to 0.1083	No
2310	1:50 X vs. 12:1 X	-0.0180	-0.1620 to 0.1260	No
2311	1:50 X vs. 12:10 X	-0.03567	-0.1796 to 0.1083	No
2312	1:50 X vs. 12:40 X	-0.0220	-0.1660 to 0.1220	No
2313	1:50 X vs. 12:50 X	-0.02533	-0.1693 to 0.1186	No
2314	1:50 X vs. 13:0 X	-0.02967	-0.1736 to 0.1143	No
2315	1:50 X vs. 13:1 X	-0.0170	-0.1610 to 0.1270	No
2316	1:50 X vs. 13:10 X	-0.0390	-0.1830 to 0.1050	No
2317	1:50 X vs. 13:40 X	-0.03533	-0.1793 to 0.1086	No
2318	1:50 X vs. 13:50 X	-0.03633	-0.1803 to 0.1076	No
2319	1:50 X vs. 14:0 X	-0.03833	-0.1823 to 0.1056	No
2320	1:50 X vs. 14:1 X	-0.0300	-0.1740 to 0.1140	No
2321	1:50 X vs. 14:10 X	-0.0440	-0.1880 to 0.09996	No
2322	1:50 X vs. 14:40 X	-0.04067	-0.1846 to 0.1033	No
2323	1:50 X vs. 14:50 X	-0.04467	-0.1886 to 0.09929	No
2324	1:50 X vs. 15:0 X	-0.05067	-0.1946 to 0.09329	No
2325	1:50 X vs. 15:1 X	-0.03067	-0.1746 to 0.1133	No
2326	1:50 X vs. 15:10 X	-0.0580	-0.2020 to 0.08596	No
2327	1:50 X vs. 15:40 X	-0.04033	-0.1843 to 0.1036	No
2328	1:50 X vs. 15:50 X	-0.0580	-0.2020 to 0.08596	No
2329	1:50 X vs. 16:0 X	-0.1430	-0.2870 to 0.0009598	No
2330	1:50 X vs. 16:1 X	-0.0340	-0.1780 to 0.1100	No
2331	1:50 X vs. 16:10 X	-0.05967	-0.2036 to 0.08429	No
2332	1:50 X vs. 16:40 X	-0.0790	-0.2230 to 0.06496	No
2333	1:50 X vs. 16:50 X	-0.1213	-0.2653 to 0.02263	No
2334	1:50 X vs. 17:0 X	-0.1957	-0.3396 to -0.05171	Yes
2335	1:50 X vs. 17:1 X	-0.1100	-0.2540 to 0.03396	No
2336	1:50 X vs. 17:10 X	-0.1667	-0.3106 to -0.02271	Yes
2337	1:50 X vs. 17:40 X	-0.0900	-0.2340 to 0.05396	No
2338	1:50 X vs. 17:50 X	-0.1353	-0.2793 to 0.008626	No
2339	1:50 X vs. 18:0 X	-0.2443	-0.3883 to -0.1004	Yes
2340	1:50 X vs. 18:1 X	-0.1527	-0.2966 to -0.008707	Yes

	2way ANOVA<br>Multiple comparisons	A<br>Data Set-A<br>Y	B<br>Data Set-B<br>Y	C<br>Data Set-C<br>Y
**2341**	1:**50 X** vs. 18:**10 X**	-0.2450	-0.3890 to -0.1010	Yes
**2342**	1:**50 X** vs. 18:**40 X**	-0.09433	-0.2383 to 0.04963	No
**2343**	1:**50 X** vs. 18:**50 X**	-0.1393	-0.2833 to 0.004626	No
**2344**	1:**50 X** vs. 19:**0 X**	-0.2793	-0.4233 to -0.1354	Yes
**2345**	1:**50 X** vs. 19:**1 X**	-0.2673	-0.4113 to -0.1234	Yes
**2346**	1:**50 X** vs. 19:**10 X**	-0.2650	-0.4090 to -0.1210	Yes
**2347**	1:**50 X** vs. 19:**40 X**	-0.1230	-0.2670 to 0.02096	No
**2348**	1:**50 X** vs. 19:**50 X**	-0.1340	-0.2780 to 0.009960	No
**2349**	1:**50 X** vs. 20:**0 X**	-0.3590	-0.5030 to -0.2150	Yes
**2350**	1:**50 X** vs. 20:**1 X**	-0.1783	-0.3223 to -0.03437	Yes
**2351**	1:**50 X** vs. 20:**10 X**	-0.2673	-0.4113 to -0.1234	Yes
**2352**	1:**50 X** vs. 20:**40 X**	-0.1347	-0.2786 to 0.009293	No
**2353**	1:**50 X** vs. 20:**50 X**	-0.1787	-0.3226 to -0.03471	Yes
**2354**	1:**50 X** vs. 21:**0 X**	-0.3823	-0.5263 to -0.2384	Yes
**2355**	1:**50 X** vs. 21:**1 X**	-0.3353	-0.4793 to -0.1914	Yes
**2356**	1:**50 X** vs. 21:**10 X**	-0.3323	-0.4763 to -0.1884	Yes
**2357**	1:**50 X** vs. 21:**40 X**	-0.2077	-0.3516 to -0.06371	Yes
**2358**	1:**50 X** vs. 21:**50 X**	-0.2980	-0.4420 to -0.1540	Yes
**2359**	1:**50 X** vs. 22:**0 X**	-0.4240	-0.5680 to -0.2800	Yes
**2360**	1:**50 X** vs. 22:**1 X**	-0.4187	-0.5626 to -0.2747	Yes
**2361**	1:**50 X** vs. 22:**10 X**	-0.4140	-0.5580 to -0.2700	Yes
**2362**	1:**50 X** vs. 22:**40 X**	-0.3143	-0.4583 to -0.1704	Yes
**2363**	1:**50 X** vs. 22:**50 X**	-0.3770	-0.5210 to -0.2330	Yes
**2364**	1:**50 X** vs. 23:**0 X**	-0.4893	-0.6333 to -0.3454	Yes
**2365**	1:**50 X** vs. 23:**1 X**	-0.6790	-0.8400 to -0.5180	Yes
**2366**	1:**50 X** vs. 23:**10 X**	-0.4287	-0.5726 to -0.2847	Yes
**2367**	1:**50 X** vs. 23:**40 X**	-0.3090	-0.4700 to -0.1480	Yes
**2368**	1:**50 X** vs. 23:**50 X**	-0.3430	-0.4870 to -0.1990	Yes
**2369**	1:**50 X** vs. 24:**0 X**	-0.6067	-0.7506 to -0.4627	Yes
**2370**	1:**50 X** vs. 24:**1 X**	-0.7630	-0.9240 to -0.6020	Yes
**2371**	1:**50 X** vs. 24:**10 X**	-0.5905	-0.7515 to -0.4295	Yes
**2372**	1:**50 X** vs. 24:**40 X**	-0.3253	-0.4693 to -0.1814	Yes
**2373**	1:**50 X** vs. 24:**50 X**	-0.2470	-0.3910 to -0.1030	Yes
**2374**	1:**50 X** vs. 25:**0 X**	-0.9440	-1.105 to -0.7830	Yes
**2375**	1:**50 X** vs. 25:**1 X**	-1.134	-1.294 to -0.9725	Yes
**2376**	1:**50 X** vs. 25:**10 X**	-0.4950	-0.6986 to -0.2914	Yes
**2377**	1:**50 X** vs. 25:**40 X**	-0.3273	-0.4713 to -0.1834	Yes
**2378**	1:**50 X** vs. 25:**50 X**	-0.3017	-0.4456 to -0.1577	Yes
**2379**	1:**50 X** vs. 26:**0 X**	-1.157	-1.317 to -0.9955	Yes
**2380**	1:**50 X** vs. 26:**1 X**	-1.270	-1.430 to -1.109	Yes
**2381**	1:**50 X** vs. 26:**10 X**	-0.9560	-1.117 to -0.7950	Yes
**2382**	1:**50 X** vs. 26:**40 X**	-0.4017	-0.5456 to -0.2577	Yes
**2383**	1:**50 X** vs. 26:**50 X**	-0.2620	-0.4060 to -0.1180	Yes
**2384**	1:**50 X** vs. 27:**0 X**	-1.344	-1.488 to -1.200	Yes
**2385**	1:**50 X** vs. 27:**1 X**	-1.435	-1.596 to -1.274	Yes

	2way ANOVA<br>Multiple comparisons	A<br>Data Set-A<br>Y	B<br>Data Set-B<br>Y	C<br>Data Set-C<br>Y
2386	1:50 X vs. 27:10 X	-1.247	-1.408 to -1.086	Yes
2387	1:50 X vs. 27:40 X	-0.4017	-0.5456 to -0.2577	Yes
2388	1:50 X vs. 27:50 X	-0.2613	-0.4053 to -0.1174	Yes
2389	1:50 X vs. 28:0 X	-1.294	-1.454 to -1.133	Yes
2390	1:50 X vs. 28:1 X	-1.334	-1.478 to -1.190	Yes
2391	1:50 X vs. 28:10 X	-1.136	-1.297 to -0.9750	Yes
2392	1:50 X vs. 28:40 X	-0.4140	-0.5580 to -0.2700	Yes
2393	1:50 X vs. 28:50 X	-0.3093	-0.4533 to -0.1654	Yes
2394	1:50 X vs. 29:0 X	-1.775	-1.936 to -1.614	Yes
2395	1:50 X vs. 29:1 X	-1.860	-2.021 to -1.699	Yes
2396	1:50 X vs. 29:10 X	-1.075	-1.235 to -0.9135	Yes
2397	1:50 X vs. 29:40 X	-0.5220	-0.6660 to -0.3780	Yes
2398	1:50 X vs. 29:50 X	-0.3107	-0.4546 to -0.1667	Yes
2399	1:50 X vs. 30:0 X	-1.490	-1.650 to -1.329	Yes
2400	1:50 X vs. 30:1 X	-1.811	-1.972 to -1.650	Yes
2401	1:50 X vs. 30:10 X	-1.149	-1.310 to -0.9880	Yes
2402	1:50 X vs. 30:40 X	-0.4490	-0.5930 to -0.3050	Yes
2403	1:50 X vs. 30:50 X	-0.3630	-0.5070 to -0.2190	Yes
2404	1:50 X vs. 31:0 X	-1.808	-1.968 to -1.647	Yes
2405	1:50 X vs. 31:1 X	-1.798	-1.959 to -1.637	Yes
2406	1:50 X vs. 31:10 X	-1.281	-1.441 to -1.120	Yes
2407	1:50 X vs. 31:40 X	-0.5407	-0.6846 to -0.3967	Yes
2408	1:50 X vs. 31:50 X	-0.3420	-0.4860 to -0.1980	Yes
2409	1:50 X vs. 32:0 X	-2.399	-2.543 to -2.255	Yes
2410	1:50 X vs. 32:1 X	-1.855	-2.016 to -1.694	Yes
2411	1:50 X vs. 32:10 X	-1.711	-1.855 to -1.567	Yes
2412	1:50 X vs. 32:40 X	-0.5370	-0.6810 to -0.3930	Yes
2413	1:50 X vs. 32:50 X	-0.3417	-0.4856 to -0.1977	Yes
2414	1:50 X vs. 33:0 X	-2.403	-2.547 to -2.259	Yes
2415	1:50 X vs. 33:1 X	-2.217	-2.361 to -2.073	Yes
2416	1:50 X vs. 33:10 X	-1.878	-2.022 to -1.734	Yes
2417	1:50 X vs. 33:40 X	-0.5557	-0.6996 to -0.4117	Yes
2418	1:50 X vs. 33:50 X	-0.3567	-0.5006 to -0.2127	Yes
2419	1:50 X vs. 34:0 X	-2.327	-2.471 to -2.183	Yes
2420	1:50 X vs. 34:1 X	-2.335	-2.479 to -2.191	Yes
2421	1:50 X vs. 34:10 X	-1.968	-2.112 to -1.824	Yes
2422	1:50 X vs. 34:40 X	-0.5677	-0.7116 to -0.4237	Yes
2423	1:50 X vs. 34:50 X	-0.5057	-0.6496 to -0.3617	Yes
2424	1:50 X vs. 35:0 X	-2.676	-2.820 to -2.532	Yes
2425	1:50 X vs. 35:1 X	-2.442	-2.586 to -2.298	Yes
2426	1:50 X vs. 35:10 X	-1.930	-2.074 to -1.786	Yes
2427	1:50 X vs. 35:40 X	-0.5767	-0.7206 to -0.4327	Yes
2428	1:50 X vs. 35:50 X	-0.5183	-0.6623 to -0.3744	Yes
2429	1:50 X vs. 36:0 X	-2.883	-3.043 to -2.722	Yes
2430	1:50 X vs. 36:1 X	-2.578	-2.722 to -2.434	Yes

	2way ANOVA<br>Multiple comparisons	A<br>Data Set-A<br>Y	B<br>Data Set-B<br>Y	C<br>Data Set-C<br>Y
2431	1:50 X vs. 36:10 X	-1.935	-2.079 to -1.791	Yes
2432	1:50 X vs. 36:40 X	-0.6320	-0.7760 to -0.4880	Yes
2433	1:50 X vs. 36:50 X	-0.4817	-0.6256 to -0.3377	Yes
2434	1:50 X vs. 37:0 X	-3.266	-3.410 to -3.122	Yes
2435	1:50 X vs. 37:1 X	-2.648	-2.792 to -2.504	Yes
2436	1:50 X vs. 37:10 X	-1.937	-2.081 to -1.793	Yes
2437	1:50 X vs. 37:40 X	-0.6693	-0.8133 to -0.5254	Yes
2438	1:50 X vs. 37:50 X	-0.4903	-0.6343 to -0.3464	Yes
2439	1:50 X vs. 38:0 X	-3.211	-3.355 to -3.067	Yes
2440	1:50 X vs. 38:1 X	-2.767	-2.911 to -2.623	Yes
2441	1:50 X vs. 38:10 X	-2.212	-2.356 to -2.068	Yes
2442	1:50 X vs. 38:40 X	-0.6737	-0.8176 to -0.5297	Yes
2443	1:50 X vs. 38:50 X	-0.4943	-0.6383 to -0.3504	Yes
2444	1:50 X vs. 39:0 X	-3.442	-3.586 to -3.298	Yes
2445	1:50 X vs. 39:1 X	-3.334	-3.478 to -3.190	Yes
2446	1:50 X vs. 39:10 X	-2.357	-2.501 to -2.213	Yes
2447	1:50 X vs. 39:40 X	-0.6797	-0.8236 to -0.5357	Yes
2448	1:50 X vs. 39:50 X	-0.5253	-0.6693 to -0.3814	Yes
2449	1:50 X vs. 40:0 X	-3.483	-3.627 to -3.339	Yes
2450	1:50 X vs. 40:1 X	-3.409	-3.553 to -3.265	Yes
2451	1:50 X vs. 40:10 X	-2.614	-2.758 to -2.470	Yes
2452	1:50 X vs. 40:40 X	-0.6843	-0.8283 to -0.5404	Yes
2453	1:50 X vs. 40:50 X	-0.5317	-0.6756 to -0.3877	Yes
2454	1:50 X vs. 41:0 X	-3.524	-3.668 to -3.380	Yes
2455	1:50 X vs. 41:1 X	-3.523	-3.667 to -3.379	Yes
2456	1:50 X vs. 41:10 X	-2.667	-2.811 to -2.523	Yes
2457	1:50 X vs. 41:40 X	-0.6317	-0.7756 to -0.4877	Yes
2458	1:50 X vs. 41:50 X	-0.5443	-0.6883 to -0.4004	Yes
2459	1:50 X vs. 42:0 X	-3.666	-3.810 to -3.522	Yes
2460	1:50 X vs. 42:1 X	-3.685	-3.829 to -3.541	Yes
2461	1:50 X vs. 42:10 X	-2.668	-2.812 to -2.524	Yes
2462	1:50 X vs. 42:40 X	-0.6443	-0.7883 to -0.5004	Yes
2463	1:50 X vs. 42:50 X	-0.5477	-0.6916 to -0.4037	Yes
2464	1:50 X vs. 43:0 X	-3.756	-3.900 to -3.612	Yes
2465	1:50 X vs. 43:1 X	-3.670	-3.814 to -3.526	Yes
2466	1:50 X vs. 43:10 X	-3.386	-3.530 to -3.242	Yes
2467	1:50 X vs. 43:40 X	-0.6427	-0.7866 to -0.4987	Yes
2468	1:50 X vs. 43:50 X	-0.5480	-0.6920 to -0.4040	Yes
2469	1:50 X vs. 44:0 X	-3.780	-3.924 to -3.636	Yes
2470	1:50 X vs. 44:1 X	-3.669	-3.813 to -3.525	Yes
2471	1:50 X vs. 44:10 X	-3.449	-3.593 to -3.305	Yes
2472	1:50 X vs. 44:40 X	-0.6447	-0.7886 to -0.5007	Yes
2473	1:50 X vs. 44:50 X	-0.4803	-0.6243 to -0.3364	Yes
2474	1:50 X vs. 45:0 X	-3.644	-3.788 to -3.500	Yes
2475	1:50 X vs. 45:1 X	-3.446	-3.590 to -3.302	Yes

	2way ANOVA<br>Multiple comparisons	A<br>Data Set-A<br>Y	B<br>Data Set-B<br>Y	C<br>Data Set-C<br>Y
**2476**	1:**50 X** vs. 45:**10 X**	-3.329	-3.473 to -3.185	Yes
**2477**	1:**50 X** vs. 45:**40 X**	-0.6460	-0.7900 to -0.5020	Yes
**2478**	1:**50 X** vs. 45:**50 X**	-0.4773	-0.6213 to -0.3334	Yes
**2479**	1:**50 X** vs. 46:**0 X**	-3.449	-3.593 to -3.305	Yes
**2480**	1:**50 X** vs. 46:**1 X**	-3.416	-3.560 to -3.272	Yes
**2481**	1:**50 X** vs. 46:**10 X**	-3.324	-3.468 to -3.180	Yes
**2482**	1:**50 X** vs. 46:**40 X**	-0.6597	-0.8036 to -0.5157	Yes
**2483**	1:**50 X** vs. 46:**50 X**	-0.4703	-0.6143 to -0.3264	Yes
**2484**	1:**50 X** vs. 47:**0 X**	-3.424	-3.568 to -3.280	Yes
**2485**	1:**50 X** vs. 47:**1 X**	-3.328	-3.472 to -3.184	Yes
**2486**	1:**50 X** vs. 47:**10 X**	-3.310	-3.454 to -3.166	Yes
**2487**	1:**50 X** vs. 47:**40 X**	-0.6697	-0.8136 to -0.5257	Yes
**2488**	1:**50 X** vs. 47:**50 X**	-0.4283	-0.5723 to -0.2844	Yes
**2489**	1:**50 X** vs. 48:**0 X**	-3.412	-3.572 to -3.251	Yes
**2490**	1:**50 X** vs. 48:**1 X**	-3.315	-3.459 to -3.171	Yes
**2491**	1:**50 X** vs. 48:**10 X**	-3.310	-3.454 to -3.166	Yes
**2492**	1:**50 X** vs. 48:**40 X**	-0.6760	-0.8200 to -0.5320	Yes
**2493**	1:**50 X** vs. 48:**50 X**	-0.4357	-0.5796 to -0.2917	Yes
**2494**	1:**50 X** vs. 49:**0 X**	-3.412	-3.556 to -3.268	Yes
**2495**	1:**50 X** vs. 49:**1 X**	-3.315	-3.459 to -3.171	Yes
**2496**	1:**50 X** vs. 49:**10 X**	-3.310	-3.454 to -3.166	Yes
**2497**	1:**50 X** vs. 49:**40 X**	-0.6803	-0.8243 to -0.5364	Yes
**2498**	1:**50 X** vs. 49:**50 X**	-0.4350	-0.5790 to -0.2910	Yes
**2499**	1:**50 X** vs. 50:**0 X**	-3.412	-3.556 to -3.268	Yes
**2500**	1:**50 X** vs. 50:**1 X**	-3.315	-3.459 to -3.171	Yes
**2501**	1:**50 X** vs. 50:**10 X**	-3.310	-3.454 to -3.166	Yes
**2502**	1:**50 X** vs. 50:**40 X**	-0.6880	-0.8320 to -0.5440	Yes
**2503**	1:**50 X** vs. 50:**50 X**	-0.4347	-0.5786 to -0.2907	Yes
**2504**	2:**0 X** vs. 2:**1 X**	-0.005000	-0.1490 to 0.1390	No
**2505**	2:**0 X** vs. 2:**10 X**	0.001000	-0.1430 to 0.1450	No
**2506**	2:**0 X** vs. 2:**40 X**	0.0003333	-0.1436 to 0.1443	No
**2507**	2:**0 X** vs. 2:**50 X**	0.003333	-0.1406 to 0.1473	No
**2508**	2:**0 X** vs. 3:**0 X**	-0.001000	-0.1450 to 0.1430	No
**2509**	2:**0 X** vs. 3:**1 X**	-0.003333	-0.1473 to 0.1406	No
**2510**	2:**0 X** vs. 3:**10 X**	0.001333	-0.1426 to 0.1453	No
**2511**	2:**0 X** vs. 3:**40 X**	0.003667	-0.1403 to 0.1476	No
**2512**	2:**0 X** vs. 3:**50 X**	0.004667	-0.1393 to 0.1486	No
**2513**	2:**0 X** vs. 4:**0 X**	-0.005333	-0.1493 to 0.1386	No
**2514**	2:**0 X** vs. 4:**1 X**	-0.0040	-0.1480 to 0.1400	No
**2515**	2:**0 X** vs. 4:**10 X**	-0.01867	-0.1626 to 0.1253	No
**2516**	2:**0 X** vs. 4:**40 X**	0.001667	-0.1423 to 0.1456	No
**2517**	2:**0 X** vs. 4:**50 X**	0.003667	-0.1403 to 0.1476	No
**2518**	2:**0 X** vs. 5:**0 X**	-0.006333	-0.1503 to 0.1376	No
**2519**	2:**0 X** vs. 5:**1 X**	-0.004333	-0.1483 to 0.1396	No
**2520**	2:**0 X** vs. 5:**10 X**	-0.01933	-0.1633 to 0.1246	No

	2way ANOVA<br>Multiple comparisons	A<br>Data Set-A<br>Y	B<br>Data Set-B<br>Y	C<br>Data Set-C<br>Y
2521	2:0 X vs. 5:40 X	0.001667	-0.1423 to 0.1456	No
2522	2:0 X vs. 5:50 X	0.002667	-0.1413 to 0.1466	No
2523	2:0 X vs. 6:0 X	-0.005667	-0.1496 to 0.1383	No
2524	2:0 X vs. 6:1 X	-0.004667	-0.1486 to 0.1393	No
2525	2:0 X vs. 6:10 X	-0.01933	-0.1633 to 0.1246	No
2526	2:0 X vs. 6:40 X	-0.0006667	-0.1446 to 0.1433	No
2527	2:0 X vs. 6:50 X	0.001000	-0.1430 to 0.1450	No
2528	2:0 X vs. 7:0 X	-0.007667	-0.1516 to 0.1363	No
2529	2:0 X vs. 7:1 X	0.001000	-0.1430 to 0.1450	No
2530	2:0 X vs. 7:10 X	-0.0110	-0.1550 to 0.1330	No
2531	2:0 X vs. 7:40 X	-0.002333	-0.1463 to 0.1416	No
2532	2:0 X vs. 7:50 X	-0.0003333	-0.1443 to 0.1436	No
2533	2:0 X vs. 8:0 X	-0.01233	-0.1563 to 0.1316	No
2534	2:0 X vs. 8:1 X	0.001000	-0.1430 to 0.1450	No
2535	2:0 X vs. 8:10 X	-0.006333	-0.1503 to 0.1376	No
2536	2:0 X vs. 8:40 X	-0.0070	-0.1510 to 0.1370	No
2537	2:0 X vs. 8:50 X	-0.004000	-0.1480 to 0.1400	No
2538	2:0 X vs. 9:0 X	-0.02033	-0.1643 to 0.1236	No
2539	2:0 X vs. 9:1 X	-0.02833	-0.1723 to 0.1156	No
2540	2:0 X vs. 9:10 X	-0.02933	-0.1733 to 0.1146	No
2541	2:0 X vs. 9:40 X	-0.01167	-0.1556 to 0.1323	No
2542	2:0 X vs. 9:50 X	-0.0050	-0.1490 to 0.1390	No
2543	2:0 X vs. 10:0 X	-0.0220	-0.1660 to 0.1220	No
2544	2:0 X vs. 10:1 X	-0.0290	-0.1730 to 0.1150	No
2545	2:0 X vs. 10:10 X	-0.03167	-0.1756 to 0.1123	No
2546	2:0 X vs. 10:40 X	-0.02133	-0.1653 to 0.1226	No
2547	2:0 X vs. 10:50 X	-0.008000	-0.1520 to 0.1360	No
2548	2:0 X vs. 11:0 X	-0.03167	-0.1756 to 0.1123	No
2549	2:0 X vs. 11:1 X	-0.03333	-0.1773 to 0.1106	No
2550	2:0 X vs. 11:10 X	-0.03367	-0.1776 to 0.1103	No
2551	2:0 X vs. 11:40 X	-0.02533	-0.1693 to 0.1186	No
2552	2:0 X vs. 11:50 X	-0.01333	-0.1573 to 0.1306	No
2553	2:0 X vs. 12:0 X	-0.03233	-0.1763 to 0.1116	No
2554	2:0 X vs. 12:1 X	-0.01467	-0.1586 to 0.1293	No
2555	2:0 X vs. 12:10 X	-0.03233	-0.1763 to 0.1116	No
2556	2:0 X vs. 12:40 X	-0.01867	-0.1626 to 0.1253	No
2557	2:0 X vs. 12:50 X	-0.0220	-0.1660 to 0.1220	No
2558	2:0 X vs. 13:0 X	-0.02633	-0.1703 to 0.1176	No
2559	2:0 X vs. 13:1 X	-0.01367	-0.1576 to 0.1303	No
2560	2:0 X vs. 13:10 X	-0.03567	-0.1796 to 0.1083	No
2561	2:0 X vs. 13:40 X	-0.0320	-0.1760 to 0.1120	No
2562	2:0 X vs. 13:50 X	-0.0330	-0.1770 to 0.1110	No
2563	2:0 X vs. 14:0 X	-0.0350	-0.1790 to 0.1090	No
2564	2:0 X vs. 14:1 X	-0.02667	-0.1706 to 0.1173	No
2565	2:0 X vs. 14:10 X	-0.04067	-0.1846 to 0.1033	No

	2way ANOVA<br>Multiple comparisons	A<br>Data Set-A<br>Y	B<br>Data Set-B<br>Y	C<br>Data Set-C<br>Y
2566	2:0 X vs. 14:40 X	-0.03733	-0.1813 to 0.1066	No
2567	2:0 X vs. 14:50 X	-0.04133	-0.1853 to 0.1026	No
2568	2:0 X vs. 15:0 X	-0.04733	-0.1913 to 0.09663	No
2569	2:0 X vs. 15:1 X	-0.02733	-0.1713 to 0.1166	No
2570	2:0 X vs. 15:10 X	-0.05467	-0.1986 to 0.08929	No
2571	2:0 X vs. 15:40 X	-0.0370	-0.1810 to 0.1070	No
2572	2:0 X vs. 15:50 X	-0.05467	-0.1986 to 0.08929	No
2573	2:0 X vs. 16:0 X	-0.1397	-0.2836 to 0.004293	No
2574	2:0 X vs. 16:1 X	-0.03067	-0.1746 to 0.1133	No
2575	2:0 X vs. 16:10 X	-0.05633	-0.2003 to 0.08763	No
2576	2:0 X vs. 16:40 X	-0.07567	-0.2196 to 0.06829	No
2577	2:0 X vs. 16:50 X	-0.1180	-0.2620 to 0.02596	No
2578	2:0 X vs. 17:0 X	-0.1923	-0.3363 to -0.04837	Yes
2579	2:0 X vs. 17:1 X	-0.1067	-0.2506 to 0.03729	No
2580	2:0 X vs. 17:10 X	-0.1633	-0.3073 to -0.01937	Yes
2581	2:0 X vs. 17:40 X	-0.08667	-0.2306 to 0.05729	No
2582	2:0 X vs. 17:50 X	-0.1320	-0.2760 to 0.01196	No
2583	2:0 X vs. 18:0 X	-0.2410	-0.3850 to -0.09704	Yes
2584	2:0 X vs. 18:1 X	-0.1493	-0.2933 to -0.005374	Yes
2585	2:0 X vs. 18:10 X	-0.2417	-0.3856 to -0.09771	Yes
2586	2:0 X vs. 18:40 X	-0.0910	-0.2350 to 0.05296	No
2587	2:0 X vs. 18:50 X	-0.1360	-0.2800 to 0.007960	No
2588	2:0 X vs. 19:0 X	-0.2760	-0.4200 to -0.1320	Yes
2589	2:0 X vs. 19:1 X	-0.2640	-0.4080 to -0.1200	Yes
2590	2:0 X vs. 19:10 X	-0.2617	-0.4056 to -0.1177	Yes
2591	2:0 X vs. 19:40 X	-0.1197	-0.2636 to 0.02429	No
2592	2:0 X vs. 19:50 X	-0.1307	-0.2746 to 0.01329	No
2593	2:0 X vs. 20:0 X	-0.3557	-0.4996 to -0.2117	Yes
2594	2:0 X vs. 20:1 X	-0.1750	-0.3190 to -0.03104	Yes
2595	2:0 X vs. 20:10 X	-0.2640	-0.4080 to -0.1200	Yes
2596	2:0 X vs. 20:40 X	-0.1313	-0.2753 to 0.01263	No
2597	2:0 X vs. 20:50 X	-0.1753	-0.3193 to -0.03137	Yes
2598	2:0 X vs. 21:0 X	-0.3790	-0.5230 to -0.2350	Yes
2599	2:0 X vs. 21:1 X	-0.3320	-0.4760 to -0.1880	Yes
2600	2:0 X vs. 21:10 X	-0.3290	-0.4730 to -0.1850	Yes
2601	2:0 X vs. 21:40 X	-0.2043	-0.3483 to -0.06037	Yes
2602	2:0 X vs. 21:50 X	-0.2947	-0.4386 to -0.1507	Yes
2603	2:0 X vs. 22:0 X	-0.4207	-0.5646 to -0.2767	Yes
2604	2:0 X vs. 22:1 X	-0.4153	-0.5593 to -0.2714	Yes
2605	2:0 X vs. 22:10 X	-0.4107	-0.5546 to -0.2667	Yes
2606	2:0 X vs. 22:40 X	-0.3110	-0.4550 to -0.1670	Yes
2607	2:0 X vs. 22:50 X	-0.3737	-0.5176 to -0.2297	Yes
2608	2:0 X vs. 23:0 X	-0.4860	-0.6300 to -0.3420	Yes
2609	2:0 X vs. 23:1 X	-0.6757	-0.8366 to -0.5147	Yes
2610	2:0 X vs. 23:10 X	-0.4253	-0.5693 to -0.2814	Yes

	2way ANOVA<br>Multiple comparisons	A<br>Data Set-A<br>Y	B<br>Data Set-B<br>Y	C<br>Data Set-C<br>Y
**2611**	2:**0 X** vs. 23:**40 X**	-0.3057	-0.4666 to -0.1447	Yes
**2612**	2:**0 X** vs. 23:**50 X**	-0.3397	-0.4836 to -0.1957	Yes
**2613**	2:**0 X** vs. 24:**0 X**	-0.6033	-0.7473 to -0.4594	Yes
**2614**	2:**0 X** vs. 24:**1 X**	-0.7597	-0.9206 to -0.5987	Yes
**2615**	2:**0 X** vs. 24:**10 X**	-0.5872	-0.7481 to -0.4262	Yes
**2616**	2:**0 X** vs. 24:**40 X**	-0.3220	-0.4660 to -0.1780	Yes
**2617**	2:**0 X** vs. 24:**50 X**	-0.2437	-0.3876 to -0.09971	Yes
**2618**	2:**0 X** vs. 25:**0 X**	-0.9407	-1.102 to -0.7797	Yes
**2619**	2:**0 X** vs. 25:**1 X**	-1.130	-1.291 to -0.9692	Yes
**2620**	2:**0 X** vs. 25:**10 X**	-0.4917	-0.6953 to -0.2881	Yes
**2621**	2:**0 X** vs. 25:**40 X**	-0.3240	-0.4680 to -0.1800	Yes
**2622**	2:**0 X** vs. 25:**50 X**	-0.2983	-0.4423 to -0.1544	Yes
**2623**	2:**0 X** vs. 26:**0 X**	-1.153	-1.314 to -0.9922	Yes
**2624**	2:**0 X** vs. 26:**1 X**	-1.266	-1.427 to -1.105	Yes
**2625**	2:**0 X** vs. 26:**10 X**	-0.9527	-1.114 to -0.7917	Yes
**2626**	2:**0 X** vs. 26:**40 X**	-0.3983	-0.5423 to -0.2544	Yes
**2627**	2:**0 X** vs. 26:**50 X**	-0.2587	-0.4026 to -0.1147	Yes
**2628**	2:**0 X** vs. 27:**0 X**	-1.341	-1.485 to -1.197	Yes
**2629**	2:**0 X** vs. 27:**1 X**	-1.432	-1.593 to -1.271	Yes
**2630**	2:**0 X** vs. 27:**10 X**	-1.244	-1.405 to -1.083	Yes
**2631**	2:**0 X** vs. 27:**40 X**	-0.3983	-0.5423 to -0.2544	Yes
**2632**	2:**0 X** vs. 27:**50 X**	-0.2580	-0.4020 to -0.1140	Yes
**2633**	2:**0 X** vs. 28:**0 X**	-1.290	-1.451 to -1.129	Yes
**2634**	2:**0 X** vs. 28:**1 X**	-1.331	-1.475 to -1.187	Yes
**2635**	2:**0 X** vs. 28:**10 X**	-1.133	-1.294 to -0.9717	Yes
**2636**	2:**0 X** vs. 28:**40 X**	-0.4107	-0.5546 to -0.2667	Yes
**2637**	2:**0 X** vs. 28:**50 X**	-0.3060	-0.4500 to -0.1620	Yes
**2638**	2:**0 X** vs. 29:**0 X**	-1.772	-1.933 to -1.611	Yes
**2639**	2:**0 X** vs. 29:**1 X**	-1.857	-2.018 to -1.696	Yes
**2640**	2:**0 X** vs. 29:**10 X**	-1.071	-1.232 to -0.9102	Yes
**2641**	2:**0 X** vs. 29:**40 X**	-0.5187	-0.6626 to -0.3747	Yes
**2642**	2:**0 X** vs. 29:**50 X**	-0.3073	-0.4513 to -0.1634	Yes
**2643**	2:**0 X** vs. 30:**0 X**	-1.486	-1.647 to -1.325	Yes
**2644**	2:**0 X** vs. 30:**1 X**	-1.808	-1.969 to -1.647	Yes
**2645**	2:**0 X** vs. 30:**10 X**	-1.146	-1.307 to -0.9847	Yes
**2646**	2:**0 X** vs. 30:**40 X**	-0.4457	-0.5896 to -0.3017	Yes
**2647**	2:**0 X** vs. 30:**50 X**	-0.3597	-0.5036 to -0.2157	Yes
**2648**	2:**0 X** vs. 31:**0 X**	-1.804	-1.965 to -1.643	Yes
**2649**	2:**0 X** vs. 31:**1 X**	-1.795	-1.956 to -1.634	Yes
**2650**	2:**0 X** vs. 31:**10 X**	-1.277	-1.438 to -1.116	Yes
**2651**	2:**0 X** vs. 31:**40 X**	-0.5373	-0.6813 to -0.3934	Yes
**2652**	2:**0 X** vs. 31:**50 X**	-0.3387	-0.4826 to -0.1947	Yes
**2653**	2:**0 X** vs. 32:**0 X**	-2.396	-2.540 to -2.252	Yes
**2654**	2:**0 X** vs. 32:**1 X**	-1.852	-2.013 to -1.691	Yes
**2655**	2:**0 X** vs. 32:**10 X**	-1.708	-1.852 to -1.564	Yes

	2way ANOVA<br>Multiple comparisons	A<br>Data Set-A<br>Y	B<br>Data Set-B<br>Y	C<br>Data Set-C<br>Y
**2656**	2:**0 X** vs. 32:**40 X**	-0.5337	-0.6776 to -0.3897	Yes
**2657**	2:**0 X** vs. 32:**50 X**	-0.3383	-0.4823 to -0.1944	Yes
**2658**	2:**0 X** vs. 33:**0 X**	-2.399	-2.543 to -2.255	Yes
**2659**	2:**0 X** vs. 33:**1 X**	-2.214	-2.358 to -2.070	Yes
**2660**	2:**0 X** vs. 33:**10 X**	-1.874	-2.018 to -1.730	Yes
**2661**	2:**0 X** vs. 33:**40 X**	-0.5523	-0.6963 to -0.4084	Yes
**2662**	2:**0 X** vs. 33:**50 X**	-0.3533	-0.4973 to -0.2094	Yes
**2663**	2:**0 X** vs. 34:**0 X**	-2.324	-2.468 to -2.180	Yes
**2664**	2:**0 X** vs. 34:**1 X**	-2.331	-2.475 to -2.187	Yes
**2665**	2:**0 X** vs. 34:**10 X**	-1.965	-2.109 to -1.821	Yes
**2666**	2:**0 X** vs. 34:**40 X**	-0.5643	-0.7083 to -0.4204	Yes
**2667**	2:**0 X** vs. 34:**50 X**	-0.5023	-0.6463 to -0.3584	Yes
**2668**	2:**0 X** vs. 35:**0 X**	-2.673	-2.817 to -2.529	Yes
**2669**	2:**0 X** vs. 35:**1 X**	-2.438	-2.582 to -2.294	Yes
**2670**	2:**0 X** vs. 35:**10 X**	-1.926	-2.070 to -1.782	Yes
**2671**	2:**0 X** vs. 35:**40 X**	-0.5733	-0.7173 to -0.4294	Yes
**2672**	2:**0 X** vs. 35:**50 X**	-0.5150	-0.6590 to -0.3710	Yes
**2673**	2:**0 X** vs. 36:**0 X**	-2.879	-3.040 to -2.718	Yes
**2674**	2:**0 X** vs. 36:**1 X**	-2.575	-2.719 to -2.431	Yes
**2675**	2:**0 X** vs. 36:**10 X**	-1.932	-2.076 to -1.788	Yes
**2676**	2:**0 X** vs. 36:**40 X**	-0.6287	-0.7726 to -0.4847	Yes
**2677**	2:**0 X** vs. 36:**50 X**	-0.4783	-0.6223 to -0.3344	Yes
**2678**	2:**0 X** vs. 37:**0 X**	-3.263	-3.407 to -3.119	Yes
**2679**	2:**0 X** vs. 37:**1 X**	-2.645	-2.789 to -2.501	Yes
**2680**	2:**0 X** vs. 37:**10 X**	-1.934	-2.078 to -1.790	Yes
**2681**	2:**0 X** vs. 37:**40 X**	-0.6660	-0.8100 to -0.5220	Yes
**2682**	2:**0 X** vs. 37:**50 X**	-0.4870	-0.6310 to -0.3430	Yes
**2683**	2:**0 X** vs. 38:**0 X**	-3.208	-3.352 to -3.064	Yes
**2684**	2:**0 X** vs. 38:**1 X**	-2.764	-2.908 to -2.620	Yes
**2685**	2:**0 X** vs. 38:**10 X**	-2.209	-2.353 to -2.065	Yes
**2686**	2:**0 X** vs. 38:**40 X**	-0.6703	-0.8143 to -0.5264	Yes
**2687**	2:**0 X** vs. 38:**50 X**	-0.4910	-0.6350 to -0.3470	Yes
**2688**	2:**0 X** vs. 39:**0 X**	-3.439	-3.583 to -3.295	Yes
**2689**	2:**0 X** vs. 39:**1 X**	-3.330	-3.474 to -3.186	Yes
**2690**	2:**0 X** vs. 39:**10 X**	-2.354	-2.498 to -2.210	Yes
**2691**	2:**0 X** vs. 39:**40 X**	-0.6763	-0.8203 to -0.5324	Yes
**2692**	2:**0 X** vs. 39:**50 X**	-0.5220	-0.6660 to -0.3780	Yes
**2693**	2:**0 X** vs. 40:**0 X**	-3.480	-3.624 to -3.336	Yes
**2694**	2:**0 X** vs. 40:**1 X**	-3.406	-3.550 to -3.262	Yes
**2695**	2:**0 X** vs. 40:**10 X**	-2.610	-2.754 to -2.466	Yes
**2696**	2:**0 X** vs. 40:**40 X**	-0.6810	-0.8250 to -0.5370	Yes
**2697**	2:**0 X** vs. 40:**50 X**	-0.5283	-0.6723 to -0.3844	Yes
**2698**	2:**0 X** vs. 41:**0 X**	-3.521	-3.665 to -3.377	Yes
**2699**	2:**0 X** vs. 41:**1 X**	-3.520	-3.664 to -3.376	Yes
**2700**	2:**0 X** vs. 41:**10 X**	-2.664	-2.808 to -2.520	Yes

	2way ANOVA<br>Multiple comparisons	A<br>Data Set-A<br>Y	B<br>Data Set-B<br>Y	C<br>Data Set-C<br>Y
2701	2:0 X vs. 41:40 X	-0.6283	-0.7723 to -0.4844	Yes
2702	2:0 X vs. 41:50 X	-0.5410	-0.6850 to -0.3970	Yes
2703	2:0 X vs. 42:0 X	-3.662	-3.806 to -3.518	Yes
2704	2:0 X vs. 42:1 X	-3.682	-3.826 to -3.538	Yes
2705	2:0 X vs. 42:10 X	-2.664	-2.808 to -2.520	Yes
2706	2:0 X vs. 42:40 X	-0.6410	-0.7850 to -0.4970	Yes
2707	2:0 X vs. 42:50 X	-0.5443	-0.6883 to -0.4004	Yes
2708	2:0 X vs. 43:0 X	-3.752	-3.896 to -3.608	Yes
2709	2:0 X vs. 43:1 X	-3.667	-3.811 to -3.523	Yes
2710	2:0 X vs. 43:10 X	-3.382	-3.526 to -3.238	Yes
2711	2:0 X vs. 43:40 X	-0.6393	-0.7833 to -0.4954	Yes
2712	2:0 X vs. 43:50 X	-0.5447	-0.6886 to -0.4007	Yes
2713	2:0 X vs. 44:0 X	-3.776	-3.920 to -3.632	Yes
2714	2:0 X vs. 44:1 X	-3.666	-3.810 to -3.522	Yes
2715	2:0 X vs. 44:10 X	-3.446	-3.590 to -3.302	Yes
2716	2:0 X vs. 44:40 X	-0.6413	-0.7853 to -0.4974	Yes
2717	2:0 X vs. 44:50 X	-0.4770	-0.6210 to -0.3330	Yes
2718	2:0 X vs. 45:0 X	-3.641	-3.785 to -3.497	Yes
2719	2:0 X vs. 45:1 X	-3.443	-3.587 to -3.299	Yes
2720	2:0 X vs. 45:10 X	-3.325	-3.469 to -3.181	Yes
2721	2:0 X vs. 45:40 X	-0.6427	-0.7866 to -0.4987	Yes
2722	2:0 X vs. 45:50 X	-0.4740	-0.6180 to -0.3300	Yes
2723	2:0 X vs. 46:0 X	-3.445	-3.589 to -3.301	Yes
2724	2:0 X vs. 46:1 X	-3.412	-3.556 to -3.268	Yes
2725	2:0 X vs. 46:10 X	-3.320	-3.464 to -3.176	Yes
2726	2:0 X vs. 46:40 X	-0.6563	-0.8003 to -0.5124	Yes
2727	2:0 X vs. 46:50 X	-0.4670	-0.6110 to -0.3230	Yes
2728	2:0 X vs. 47:0 X	-3.421	-3.565 to -3.277	Yes
2729	2:0 X vs. 47:1 X	-3.325	-3.469 to -3.181	Yes
2730	2:0 X vs. 47:10 X	-3.306	-3.450 to -3.162	Yes
2731	2:0 X vs. 47:40 X	-0.6663	-0.8103 to -0.5224	Yes
2732	2:0 X vs. 47:50 X	-0.4250	-0.5690 to -0.2810	Yes
2733	2:0 X vs. 48:0 X	-3.408	-3.569 to -3.247	Yes
2734	2:0 X vs. 48:1 X	-3.312	-3.456 to -3.168	Yes
2735	2:0 X vs. 48:10 X	-3.306	-3.450 to -3.162	Yes
2736	2:0 X vs. 48:40 X	-0.6727	-0.8166 to -0.5287	Yes
2737	2:0 X vs. 48:50 X	-0.4323	-0.5763 to -0.2884	Yes
2738	2:0 X vs. 49:0 X	-3.408	-3.552 to -3.264	Yes
2739	2:0 X vs. 49:1 X	-3.312	-3.456 to -3.168	Yes
2740	2:0 X vs. 49:10 X	-3.306	-3.450 to -3.162	Yes
2741	2:0 X vs. 49:40 X	-0.6770	-0.8210 to -0.5330	Yes
2742	2:0 X vs. 49:50 X	-0.4317	-0.5756 to -0.2877	Yes
2743	2:0 X vs. 50:0 X	-3.408	-3.552 to -3.264	Yes
2744	2:0 X vs. 50:1 X	-3.312	-3.456 to -3.168	Yes
2745	2:0 X vs. 50:10 X	-3.306	-3.450 to -3.162	Yes

	2way ANOVA<br>Multiple comparisons	A<br>Data Set-A<br>Y	B<br>Data Set-B<br>Y	C<br>Data Set-C<br>Y
2746	2:**0 X** vs. 50:**40 X**	-0.6847	-0.8286 to -0.5407	Yes
2747	2:**0 X** vs. 50:**50 X**	-0.4313	-0.5753 to -0.2874	Yes
2748	2:**1 X** vs. 2:**10 X**	0.0060	-0.1380 to 0.1500	No
2749	2:**1 X** vs. 2:**40 X**	0.005333	-0.1386 to 0.1493	No
2750	2:**1 X** vs. 2:**50 X**	0.008333	-0.1356 to 0.1523	No
2751	2:**1 X** vs. 3:**0 X**	0.0040	-0.1400 to 0.1480	No
2752	2:**1 X** vs. 3:**1 X**	0.001667	-0.1423 to 0.1456	No
2753	2:**1 X** vs. 3:**10 X**	0.006333	-0.1376 to 0.1503	No
2754	2:**1 X** vs. 3:**40 X**	0.008667	-0.1353 to 0.1526	No
2755	2:**1 X** vs. 3:**50 X**	0.009667	-0.1343 to 0.1536	No
2756	2:**1 X** vs. 4:**0 X**	-0.0003333	-0.1443 to 0.1436	No
2757	2:**1 X** vs. 4:**1 X**	0.001000	-0.1430 to 0.1450	No
2758	2:**1 X** vs. 4:**10 X**	-0.01367	-0.1576 to 0.1303	No
2759	2:**1 X** vs. 4:**40 X**	0.006667	-0.1373 to 0.1506	No
2760	2:**1 X** vs. 4:**50 X**	0.008667	-0.1353 to 0.1526	No
2761	2:**1 X** vs. 5:**0 X**	-0.001333	-0.1453 to 0.1426	No
2762	2:**1 X** vs. 5:**1 X**	0.0006667	-0.1433 to 0.1446	No
2763	2:**1 X** vs. 5:**10 X**	-0.01433	-0.1583 to 0.1296	No
2764	2:**1 X** vs. 5:**40 X**	0.006667	-0.1373 to 0.1506	No
2765	2:**1 X** vs. 5:**50 X**	0.007667	-0.1363 to 0.1516	No
2766	2:**1 X** vs. 6:**0 X**	-0.0006667	-0.1446 to 0.1433	No
2767	2:**1 X** vs. 6:**1 X**	0.0003333	-0.1436 to 0.1443	No
2768	2:**1 X** vs. 6:**10 X**	-0.01433	-0.1583 to 0.1296	No
2769	2:**1 X** vs. 6:**40 X**	0.004333	-0.1396 to 0.1483	No
2770	2:**1 X** vs. 6:**50 X**	0.0060	-0.1380 to 0.1500	No
2771	2:**1 X** vs. 7:**0 X**	-0.002667	-0.1466 to 0.1413	No
2772	2:**1 X** vs. 7:**1 X**	0.0060	-0.1380 to 0.1500	No
2773	2:**1 X** vs. 7:**10 X**	-0.0060	-0.1500 to 0.1380	No
2774	2:**1 X** vs. 7:**40 X**	0.002667	-0.1413 to 0.1466	No
2775	2:**1 X** vs. 7:**50 X**	0.004667	-0.1393 to 0.1486	No
2776	2:**1 X** vs. 8:**0 X**	-0.007333	-0.1513 to 0.1366	No
2777	2:**1 X** vs. 8:**1 X**	0.0060	-0.1380 to 0.1500	No
2778	2:**1 X** vs. 8:**10 X**	-0.001333	-0.1453 to 0.1426	No
2779	2:**1 X** vs. 8:**40 X**	-0.0020	-0.1460 to 0.1420	No
2780	2:**1 X** vs. 8:**50 X**	0.001000	-0.1430 to 0.1450	No
2781	2:**1 X** vs. 9:**0 X**	-0.01533	-0.1593 to 0.1286	No
2782	2:**1 X** vs. 9:**1 X**	-0.02333	-0.1673 to 0.1206	No
2783	2:**1 X** vs. 9:**10 X**	-0.02433	-0.1683 to 0.1196	No
2784	2:**1 X** vs. 9:**40 X**	-0.006667	-0.1506 to 0.1373	No
2785	2:**1 X** vs. 9:**50 X**	-9.313e-010	-0.1440 to 0.1440	No
2786	2:**1 X** vs. 10:**0 X**	-0.0170	-0.1610 to 0.1270	No
2787	2:**1 X** vs. 10:**1 X**	-0.0240	-0.1680 to 0.1200	No
2788	2:**1 X** vs. 10:**10 X**	-0.02667	-0.1706 to 0.1173	No
2789	2:**1 X** vs. 10:**40 X**	-0.01633	-0.1603 to 0.1276	No
2790	2:**1 X** vs. 10:**50 X**	-0.0030	-0.1470 to 0.1410	No

	2way ANOVA<br>Multiple comparisons	A<br>Data Set-A<br>Y	B<br>Data Set-B<br>Y	C<br>Data Set-C<br>Y
2791	2:1 X vs. 11:0 X	-0.02667	-0.1706 to 0.1173	No
2792	2:1 X vs. 11:1 X	-0.02833	-0.1723 to 0.1156	No
2793	2:1 X vs. 11:10 X	-0.02867	-0.1726 to 0.1153	No
2794	2:1 X vs. 11:40 X	-0.02033	-0.1643 to 0.1236	No
2795	2:1 X vs. 11:50 X	-0.008333	-0.1523 to 0.1356	No
2796	2:1 X vs. 12:0 X	-0.02733	-0.1713 to 0.1166	No
2797	2:1 X vs. 12:1 X	-0.009667	-0.1536 to 0.1343	No
2798	2:1 X vs. 12:10 X	-0.02733	-0.1713 to 0.1166	No
2799	2:1 X vs. 12:40 X	-0.01367	-0.1576 to 0.1303	No
2800	2:1 X vs. 12:50 X	-0.0170	-0.1610 to 0.1270	No
2801	2:1 X vs. 13:0 X	-0.02133	-0.1653 to 0.1226	No
2802	2:1 X vs. 13:1 X	-0.008667	-0.1526 to 0.1353	No
2803	2:1 X vs. 13:10 X	-0.03067	-0.1746 to 0.1133	No
2804	2:1 X vs. 13:40 X	-0.0270	-0.1710 to 0.1170	No
2805	2:1 X vs. 13:50 X	-0.0280	-0.1720 to 0.1160	No
2806	2:1 X vs. 14:0 X	-0.0300	-0.1740 to 0.1140	No
2807	2:1 X vs. 14:1 X	-0.02167	-0.1656 to 0.1223	No
2808	2:1 X vs. 14:10 X	-0.03567	-0.1796 to 0.1083	No
2809	2:1 X vs. 14:40 X	-0.03233	-0.1763 to 0.1116	No
2810	2:1 X vs. 14:50 X	-0.03633	-0.1803 to 0.1076	No
2811	2:1 X vs. 15:0 X	-0.04233	-0.1863 to 0.1016	No
2812	2:1 X vs. 15:1 X	-0.02233	-0.1663 to 0.1216	No
2813	2:1 X vs. 15:10 X	-0.04967	-0.1936 to 0.09429	No
2814	2:1 X vs. 15:40 X	-0.0320	-0.1760 to 0.1120	No
2815	2:1 X vs. 15:50 X	-0.04967	-0.1936 to 0.09429	No
2816	2:1 X vs. 16:0 X	-0.1347	-0.2786 to 0.009293	No
2817	2:1 X vs. 16:1 X	-0.02567	-0.1696 to 0.1183	No
2818	2:1 X vs. 16:10 X	-0.05133	-0.1953 to 0.09263	No
2819	2:1 X vs. 16:40 X	-0.07067	-0.2146 to 0.07329	No
2820	2:1 X vs. 16:50 X	-0.1130	-0.2570 to 0.03096	No
2821	2:1 X vs. 17:0 X	-0.1873	-0.3313 to -0.04337	Yes
2822	2:1 X vs. 17:1 X	-0.1017	-0.2456 to 0.04229	No
2823	2:1 X vs. 17:10 X	-0.1583	-0.3023 to -0.01437	Yes
2824	2:1 X vs. 17:40 X	-0.08167	-0.2256 to 0.06229	No
2825	2:1 X vs. 17:50 X	-0.1270	-0.2710 to 0.01696	No
2826	2:1 X vs. 18:0 X	-0.2360	-0.3800 to -0.09204	Yes
2827	2:1 X vs. 18:1 X	-0.1443	-0.2883 to -0.0003735	Yes
2828	2:1 X vs. 18:10 X	-0.2367	-0.3806 to -0.09271	Yes
2829	2:1 X vs. 18:40 X	-0.0860	-0.2300 to 0.05796	No
2830	2:1 X vs. 18:50 X	-0.1310	-0.2750 to 0.01296	No
2831	2:1 X vs. 19:0 X	-0.2710	-0.4150 to -0.1270	Yes
2832	2:1 X vs. 19:1 X	-0.2590	-0.4030 to -0.1150	Yes
2833	2:1 X vs. 19:10 X	-0.2567	-0.4006 to -0.1127	Yes
2834	2:1 X vs. 19:40 X	-0.1147	-0.2586 to 0.02929	No
2835	2:1 X vs. 19:50 X	-0.1257	-0.2696 to 0.01829	No

2way ANOVA Multiple comparisons		A Data Set-A	B Data Set-B	C Data Set-C
		Y	Y	Y
2836	2:**1 X** vs. 20:**0 X**	-0.3507	-0.4946 to -0.2067	Yes
2837	2:**1 X** vs. 20:**1 X**	-0.1700	-0.3140 to -0.02604	Yes
2838	2:**1 X** vs. 20:**10 X**	-0.2590	-0.4030 to -0.1150	Yes
2839	2:**1 X** vs. 20:**40 X**	-0.1263	-0.2703 to 0.01763	No
2840	2:**1 X** vs. 20:**50 X**	-0.1703	-0.3143 to -0.02637	Yes
2841	2:**1 X** vs. 21:**0 X**	-0.3740	-0.5180 to -0.2300	Yes
2842	2:**1 X** vs. 21:**1 X**	-0.3270	-0.4710 to -0.1830	Yes
2843	2:**1 X** vs. 21:**10 X**	-0.3240	-0.4680 to -0.1800	Yes
2844	2:**1 X** vs. 21:**40 X**	-0.1993	-0.3433 to -0.05537	Yes
2845	2:**1 X** vs. 21:**50 X**	-0.2897	-0.4336 to -0.1457	Yes
2846	2:**1 X** vs. 22:**0 X**	-0.4157	-0.5596 to -0.2717	Yes
2847	2:**1 X** vs. 22:**1 X**	-0.4103	-0.5543 to -0.2664	Yes
2848	2:**1 X** vs. 22:**10 X**	-0.4057	-0.5496 to -0.2617	Yes
2849	2:**1 X** vs. 22:**40 X**	-0.3060	-0.4500 to -0.1620	Yes
2850	2:**1 X** vs. 22:**50 X**	-0.3687	-0.5126 to -0.2247	Yes
2851	2:**1 X** vs. 23:**0 X**	-0.4810	-0.6250 to -0.3370	Yes
2852	2:**1 X** vs. 23:**1 X**	-0.6707	-0.8316 to -0.5097	Yes
2853	2:**1 X** vs. 23:**10 X**	-0.4203	-0.5643 to -0.2764	Yes
2854	2:**1 X** vs. 23:**40 X**	-0.3007	-0.4616 to -0.1397	Yes
2855	2:**1 X** vs. 23:**50 X**	-0.3347	-0.4786 to -0.1907	Yes
2856	2:**1 X** vs. 24:**0 X**	-0.5983	-0.7423 to -0.4544	Yes
2857	2:**1 X** vs. 24:**1 X**	-0.7547	-0.9156 to -0.5937	Yes
2858	2:**1 X** vs. 24:**10 X**	-0.5822	-0.7431 to -0.4212	Yes
2859	2:**1 X** vs. 24:**40 X**	-0.3170	-0.4610 to -0.1730	Yes
2860	2:**1 X** vs. 24:**50 X**	-0.2387	-0.3826 to -0.09471	Yes
2861	2:**1 X** vs. 25:**0 X**	-0.9357	-1.097 to -0.7747	Yes
2862	2:**1 X** vs. 25:**1 X**	-1.125	-1.286 to -0.9642	Yes
2863	2:**1 X** vs. 25:**10 X**	-0.4867	-0.6903 to -0.2831	Yes
2864	2:**1 X** vs. 25:**40 X**	-0.3190	-0.4630 to -0.1750	Yes
2865	2:**1 X** vs. 25:**50 X**	-0.2933	-0.4373 to -0.1494	Yes
2866	2:**1 X** vs. 26:**0 X**	-1.148	-1.309 to -0.9872	Yes
2867	2:**1 X** vs. 26:**1 X**	-1.261	-1.422 to -1.100	Yes
2868	2:**1 X** vs. 26:**10 X**	-0.9477	-1.109 to -0.7867	Yes
2869	2:**1 X** vs. 26:**40 X**	-0.3933	-0.5373 to -0.2494	Yes
2870	2:**1 X** vs. 26:**50 X**	-0.2537	-0.3976 to -0.1097	Yes
2871	2:**1 X** vs. 27:**0 X**	-1.336	-1.480 to -1.192	Yes
2872	2:**1 X** vs. 27:**1 X**	-1.427	-1.588 to -1.266	Yes
2873	2:**1 X** vs. 27:**10 X**	-1.239	-1.400 to -1.078	Yes
2874	2:**1 X** vs. 27:**40 X**	-0.3933	-0.5373 to -0.2494	Yes
2875	2:**1 X** vs. 27:**50 X**	-0.2530	-0.3970 to -0.1090	Yes
2876	2:**1 X** vs. 28:**0 X**	-1.285	-1.446 to -1.124	Yes
2877	2:**1 X** vs. 28:**1 X**	-1.326	-1.470 to -1.182	Yes
2878	2:**1 X** vs. 28:**10 X**	-1.128	-1.289 to -0.9667	Yes
2879	2:**1 X** vs. 28:**40 X**	-0.4057	-0.5496 to -0.2617	Yes
2880	2:**1 X** vs. 28:**50 X**	-0.3010	-0.4450 to -0.1570	Yes

	2way ANOVA<br>Multiple comparisons	A<br>Data Set-A<br>Y	B<br>Data Set-B<br>Y	C<br>Data Set-C<br>Y
2881	2:1 X vs. 29:0 X	-1.767	-1.928 to -1.606	Yes
2882	2:1 X vs. 29:1 X	-1.852	-2.013 to -1.691	Yes
2883	2:1 X vs. 29:10 X	-1.066	-1.227 to -0.9052	Yes
2884	2:1 X vs. 29:40 X	-0.5137	-0.6576 to -0.3697	Yes
2885	2:1 X vs. 29:50 X	-0.3023	-0.4463 to -0.1584	Yes
2886	2:1 X vs. 30:0 X	-1.481	-1.642 to -1.320	Yes
2887	2:1 X vs. 30:1 X	-1.803	-1.964 to -1.642	Yes
2888	2:1 X vs. 30:10 X	-1.141	-1.302 to -0.9797	Yes
2889	2:1 X vs. 30:40 X	-0.4407	-0.5846 to -0.2967	Yes
2890	2:1 X vs. 30:50 X	-0.3547	-0.4986 to -0.2107	Yes
2891	2:1 X vs. 31:0 X	-1.799	-1.960 to -1.638	Yes
2892	2:1 X vs. 31:1 X	-1.790	-1.951 to -1.629	Yes
2893	2:1 X vs. 31:10 X	-1.272	-1.433 to -1.111	Yes
2894	2:1 X vs. 31:40 X	-0.5323	-0.6763 to -0.3884	Yes
2895	2:1 X vs. 31:50 X	-0.3337	-0.4776 to -0.1897	Yes
2896	2:1 X vs. 32:0 X	-2.391	-2.535 to -2.247	Yes
2897	2:1 X vs. 32:1 X	-1.847	-2.008 to -1.686	Yes
2898	2:1 X vs. 32:10 X	-1.703	-1.847 to -1.559	Yes
2899	2:1 X vs. 32:40 X	-0.5287	-0.6726 to -0.3847	Yes
2900	2:1 X vs. 32:50 X	-0.3333	-0.4773 to -0.1894	Yes
2901	2:1 X vs. 33:0 X	-2.394	-2.538 to -2.250	Yes
2902	2:1 X vs. 33:1 X	-2.209	-2.353 to -2.065	Yes
2903	2:1 X vs. 33:10 X	-1.869	-2.013 to -1.725	Yes
2904	2:1 X vs. 33:40 X	-0.5473	-0.6913 to -0.4034	Yes
2905	2:1 X vs. 33:50 X	-0.3483	-0.4923 to -0.2044	Yes
2906	2:1 X vs. 34:0 X	-2.319	-2.463 to -2.175	Yes
2907	2:1 X vs. 34:1 X	-2.326	-2.470 to -2.182	Yes
2908	2:1 X vs. 34:10 X	-1.960	-2.104 to -1.816	Yes
2909	2:1 X vs. 34:40 X	-0.5593	-0.7033 to -0.4154	Yes
2910	2:1 X vs. 34:50 X	-0.4973	-0.6413 to -0.3534	Yes
2911	2:1 X vs. 35:0 X	-2.668	-2.812 to -2.524	Yes
2912	2:1 X vs. 35:1 X	-2.433	-2.577 to -2.289	Yes
2913	2:1 X vs. 35:10 X	-1.921	-2.065 to -1.777	Yes
2914	2:1 X vs. 35:40 X	-0.5683	-0.7123 to -0.4244	Yes
2915	2:1 X vs. 35:50 X	-0.5100	-0.6540 to -0.3660	Yes
2916	2:1 X vs. 36:0 X	-2.874	-3.035 to -2.713	Yes
2917	2:1 X vs. 36:1 X	-2.570	-2.714 to -2.426	Yes
2918	2:1 X vs. 36:10 X	-1.927	-2.071 to -1.783	Yes
2919	2:1 X vs. 36:40 X	-0.6237	-0.7676 to -0.4797	Yes
2920	2:1 X vs. 36:50 X	-0.4733	-0.6173 to -0.3294	Yes
2921	2:1 X vs. 37:0 X	-3.258	-3.402 to -3.114	Yes
2922	2:1 X vs. 37:1 X	-2.640	-2.784 to -2.496	Yes
2923	2:1 X vs. 37:10 X	-1.929	-2.073 to -1.785	Yes
2924	2:1 X vs. 37:40 X	-0.6610	-0.8050 to -0.5170	Yes
2925	2:1 X vs. 37:50 X	-0.4820	-0.6260 to -0.3380	Yes

	2way ANOVA<br>Multiple comparisons	A<br>Data Set-A<br>Y	B<br>Data Set-B<br>Y	C<br>Data Set-C<br>Y
**2926**	2:**1 X** vs. 38:**0 X**	-3.203	-3.347 to -3.059	Yes
**2927**	2:**1 X** vs. 38:**1 X**	-2.759	-2.903 to -2.615	Yes
**2928**	2:**1 X** vs. 38:**10 X**	-2.204	-2.348 to -2.060	Yes
**2929**	2:**1 X** vs. 38:**40 X**	-0.6653	-0.8093 to -0.5214	Yes
**2930**	2:**1 X** vs. 38:**50 X**	-0.4860	-0.6300 to -0.3420	Yes
**2931**	2:**1 X** vs. 39:**0 X**	-3.434	-3.578 to -3.290	Yes
**2932**	2:**1 X** vs. 39:**1 X**	-3.325	-3.469 to -3.181	Yes
**2933**	2:**1 X** vs. 39:**10 X**	-2.349	-2.493 to -2.205	Yes
**2934**	2:**1 X** vs. 39:**40 X**	-0.6713	-0.8153 to -0.5274	Yes
**2935**	2:**1 X** vs. 39:**50 X**	-0.5170	-0.6610 to -0.3730	Yes
**2936**	2:**1 X** vs. 40:**0 X**	-3.475	-3.619 to -3.331	Yes
**2937**	2:**1 X** vs. 40:**1 X**	-3.401	-3.545 to -3.257	Yes
**2938**	2:**1 X** vs. 40:**10 X**	-2.605	-2.749 to -2.461	Yes
**2939**	2:**1 X** vs. 40:**40 X**	-0.6760	-0.8200 to -0.5320	Yes
**2940**	2:**1 X** vs. 40:**50 X**	-0.5233	-0.6673 to -0.3794	Yes
**2941**	2:**1 X** vs. 41:**0 X**	-3.516	-3.660 to -3.372	Yes
**2942**	2:**1 X** vs. 41:**1 X**	-3.515	-3.659 to -3.371	Yes
**2943**	2:**1 X** vs. 41:**10 X**	-2.659	-2.803 to -2.515	Yes
**2944**	2:**1 X** vs. 41:**40 X**	-0.6233	-0.7673 to -0.4794	Yes
**2945**	2:**1 X** vs. 41:**50 X**	-0.5360	-0.6800 to -0.3920	Yes
**2946**	2:**1 X** vs. 42:**0 X**	-3.657	-3.801 to -3.513	Yes
**2947**	2:**1 X** vs. 42:**1 X**	-3.677	-3.821 to -3.533	Yes
**2948**	2:**1 X** vs. 42:**10 X**	-2.659	-2.803 to -2.515	Yes
**2949**	2:**1 X** vs. 42:**40 X**	-0.6360	-0.7800 to -0.4920	Yes
**2950**	2:**1 X** vs. 42:**50 X**	-0.5393	-0.6833 to -0.3954	Yes
**2951**	2:**1 X** vs. 43:**0 X**	-3.747	-3.891 to -3.603	Yes
**2952**	2:**1 X** vs. 43:**1 X**	-3.662	-3.806 to -3.518	Yes
**2953**	2:**1 X** vs. 43:**10 X**	-3.377	-3.521 to -3.233	Yes
**2954**	2:**1 X** vs. 43:**40 X**	-0.6343	-0.7783 to -0.4904	Yes
**2955**	2:**1 X** vs. 43:**50 X**	-0.5397	-0.6836 to -0.3957	Yes
**2956**	2:**1 X** vs. 44:**0 X**	-3.771	-3.915 to -3.627	Yes
**2957**	2:**1 X** vs. 44:**1 X**	-3.661	-3.805 to -3.517	Yes
**2958**	2:**1 X** vs. 44:**10 X**	-3.441	-3.585 to -3.297	Yes
**2959**	2:**1 X** vs. 44:**40 X**	-0.6363	-0.7803 to -0.4924	Yes
**2960**	2:**1 X** vs. 44:**50 X**	-0.4720	-0.6160 to -0.3280	Yes
**2961**	2:**1 X** vs. 45:**0 X**	-3.636	-3.780 to -3.492	Yes
**2962**	2:**1 X** vs. 45:**1 X**	-3.438	-3.582 to -3.294	Yes
**2963**	2:**1 X** vs. 45:**10 X**	-3.320	-3.464 to -3.176	Yes
**2964**	2:**1 X** vs. 45:**40 X**	-0.6377	-0.7816 to -0.4937	Yes
**2965**	2:**1 X** vs. 45:**50 X**	-0.4690	-0.6130 to -0.3250	Yes
**2966**	2:**1 X** vs. 46:**0 X**	-3.440	-3.584 to -3.296	Yes
**2967**	2:**1 X** vs. 46:**1 X**	-3.407	-3.551 to -3.263	Yes
**2968**	2:**1 X** vs. 46:**10 X**	-3.315	-3.459 to -3.171	Yes
**2969**	2:**1 X** vs. 46:**40 X**	-0.6513	-0.7953 to -0.5074	Yes
**2970**	2:**1 X** vs. 46:**50 X**	-0.4620	-0.6060 to -0.3180	Yes

	2way ANOVA<br>Multiple comparisons	A<br>Data Set-A<br>Y	B<br>Data Set-B<br>Y	C<br>Data Set-C<br>Y
2971	2:1 X vs. 47:0 X	-3.416	-3.560 to -3.272	Yes
2972	2:1 X vs. 47:1 X	-3.320	-3.464 to -3.176	Yes
2973	2:1 X vs. 47:10 X	-3.301	-3.445 to -3.157	Yes
2974	2:1 X vs. 47:40 X	-0.6613	-0.8053 to -0.5174	Yes
2975	2:1 X vs. 47:50 X	-0.4200	-0.5640 to -0.2760	Yes
2976	2:1 X vs. 48:0 X	-3.403	-3.564 to -3.242	Yes
2977	2:1 X vs. 48:1 X	-3.307	-3.451 to -3.163	Yes
2978	2:1 X vs. 48:10 X	-3.301	-3.445 to -3.157	Yes
2979	2:1 X vs. 48:40 X	-0.6677	-0.8116 to -0.5237	Yes
2980	2:1 X vs. 48:50 X	-0.4273	-0.5713 to -0.2834	Yes
2981	2:1 X vs. 49:0 X	-3.403	-3.547 to -3.259	Yes
2982	2:1 X vs. 49:1 X	-3.307	-3.451 to -3.163	Yes
2983	2:1 X vs. 49:10 X	-3.301	-3.445 to -3.157	Yes
2984	2:1 X vs. 49:40 X	-0.6720	-0.8160 to -0.5280	Yes
2985	2:1 X vs. 49:50 X	-0.4267	-0.5706 to -0.2827	Yes
2986	2:1 X vs. 50:0 X	-3.403	-3.547 to -3.259	Yes
2987	2:1 X vs. 50:1 X	-3.307	-3.451 to -3.163	Yes
2988	2:1 X vs. 50:10 X	-3.301	-3.445 to -3.157	Yes
2989	2:1 X vs. 50:40 X	-0.6797	-0.8236 to -0.5357	Yes
2990	2:1 X vs. 50:50 X	-0.4263	-0.5703 to -0.2824	Yes
2991	2:10 X vs. 2:40 X	-0.0006667	-0.1446 to 0.1433	No
2992	2:10 X vs. 2:50 X	0.002333	-0.1416 to 0.1463	No
2993	2:10 X vs. 3:0 X	-0.0020	-0.1460 to 0.1420	No
2994	2:10 X vs. 3:1 X	-0.004333	-0.1483 to 0.1396	No
2995	2:10 X vs. 3:10 X	0.0003333	-0.1436 to 0.1443	No
2996	2:10 X vs. 3:40 X	0.002667	-0.1413 to 0.1466	No
2997	2:10 X vs. 3:50 X	0.003667	-0.1403 to 0.1476	No
2998	2:10 X vs. 4:0 X	-0.006333	-0.1503 to 0.1376	No
2999	2:10 X vs. 4:1 X	-0.005000	-0.1490 to 0.1390	No
3000	2:10 X vs. 4:10 X	-0.01967	-0.1636 to 0.1243	No
3001	2:10 X vs. 4:40 X	0.0006667	-0.1433 to 0.1446	No
3002	2:10 X vs. 4:50 X	0.002667	-0.1413 to 0.1466	No
3003	2:10 X vs. 5:0 X	-0.007333	-0.1513 to 0.1366	No
3004	2:10 X vs. 5:1 X	-0.005333	-0.1493 to 0.1386	No
3005	2:10 X vs. 5:10 X	-0.02033	-0.1643 to 0.1236	No
3006	2:10 X vs. 5:40 X	0.0006667	-0.1433 to 0.1446	No
3007	2:10 X vs. 5:50 X	0.001667	-0.1423 to 0.1456	No
3008	2:10 X vs. 6:0 X	-0.006667	-0.1506 to 0.1373	No
3009	2:10 X vs. 6:1 X	-0.005667	-0.1496 to 0.1383	No
3010	2:10 X vs. 6:10 X	-0.02033	-0.1643 to 0.1236	No
3011	2:10 X vs. 6:40 X	-0.001667	-0.1456 to 0.1423	No
3012	2:10 X vs. 6:50 X	0.0	-0.1440 to 0.1440	No
3013	2:10 X vs. 7:0 X	-0.008667	-0.1526 to 0.1353	No
3014	2:10 X vs. 7:1 X	0.0	-0.1440 to 0.1440	No
3015	2:10 X vs. 7:10 X	-0.0120	-0.1560 to 0.1320	No

	2way ANOVA<br>Multiple comparisons	A<br>Data Set-A<br>Y	B<br>Data Set-B<br>Y	C<br>Data Set-C<br>Y
3016	2:10 X vs. 7:40 X	-0.003333	-0.1473 to 0.1406	No
3017	2:10 X vs. 7:50 X	-0.001333	-0.1453 to 0.1426	No
3018	2:10 X vs. 8:0 X	-0.01333	-0.1573 to 0.1306	No
3019	2:10 X vs. 8:1 X	0.0	-0.1440 to 0.1440	No
3020	2:10 X vs. 8:10 X	-0.007333	-0.1513 to 0.1366	No
3021	2:10 X vs. 8:40 X	-0.008000	-0.1520 to 0.1360	No
3022	2:10 X vs. 8:50 X	-0.0050	-0.1490 to 0.1390	No
3023	2:10 X vs. 9:0 X	-0.02133	-0.1653 to 0.1226	No
3024	2:10 X vs. 9:1 X	-0.02933	-0.1733 to 0.1146	No
3025	2:10 X vs. 9:10 X	-0.03033	-0.1743 to 0.1136	No
3026	2:10 X vs. 9:40 X	-0.01267	-0.1566 to 0.1313	No
3027	2:10 X vs. 9:50 X	-0.006000	-0.1500 to 0.1380	No
3028	2:10 X vs. 10:0 X	-0.0230	-0.1670 to 0.1210	No
3029	2:10 X vs. 10:1 X	-0.0300	-0.1740 to 0.1140	No
3030	2:10 X vs. 10:10 X	-0.03267	-0.1766 to 0.1113	No
3031	2:10 X vs. 10:40 X	-0.02233	-0.1663 to 0.1216	No
3032	2:10 X vs. 10:50 X	-0.0090	-0.1530 to 0.1350	No
3033	2:10 X vs. 11:0 X	-0.03267	-0.1766 to 0.1113	No
3034	2:10 X vs. 11:1 X	-0.03433	-0.1783 to 0.1096	No
3035	2:10 X vs. 11:10 X	-0.03467	-0.1786 to 0.1093	No
3036	2:10 X vs. 11:40 X	-0.02633	-0.1703 to 0.1176	No
3037	2:10 X vs. 11:50 X	-0.01433	-0.1583 to 0.1296	No
3038	2:10 X vs. 12:0 X	-0.03333	-0.1773 to 0.1106	No
3039	2:10 X vs. 12:1 X	-0.01567	-0.1596 to 0.1283	No
3040	2:10 X vs. 12:10 X	-0.03333	-0.1773 to 0.1106	No
3041	2:10 X vs. 12:40 X	-0.01967	-0.1636 to 0.1243	No
3042	2:10 X vs. 12:50 X	-0.0230	-0.1670 to 0.1210	No
3043	2:10 X vs. 13:0 X	-0.02733	-0.1713 to 0.1166	No
3044	2:10 X vs. 13:1 X	-0.01467	-0.1586 to 0.1293	No
3045	2:10 X vs. 13:10 X	-0.03667	-0.1806 to 0.1073	No
3046	2:10 X vs. 13:40 X	-0.0330	-0.1770 to 0.1110	No
3047	2:10 X vs. 13:50 X	-0.0340	-0.1780 to 0.1100	No
3048	2:10 X vs. 14:0 X	-0.0360	-0.1800 to 0.1080	No
3049	2:10 X vs. 14:1 X	-0.02767	-0.1716 to 0.1163	No
3050	2:10 X vs. 14:10 X	-0.04167	-0.1856 to 0.1023	No
3051	2:10 X vs. 14:40 X	-0.03833	-0.1823 to 0.1056	No
3052	2:10 X vs. 14:50 X	-0.04233	-0.1863 to 0.1016	No
3053	2:10 X vs. 15:0 X	-0.04833	-0.1923 to 0.09563	No
3054	2:10 X vs. 15:1 X	-0.02833	-0.1723 to 0.1156	No
3055	2:10 X vs. 15:10 X	-0.05567	-0.1996 to 0.08829	No
3056	2:10 X vs. 15:40 X	-0.0380	-0.1820 to 0.1060	No
3057	2:10 X vs. 15:50 X	-0.05567	-0.1996 to 0.08829	No
3058	2:10 X vs. 16:0 X	-0.1407	-0.2846 to 0.003293	No
3059	2:10 X vs. 16:1 X	-0.03167	-0.1756 to 0.1123	No
3060	2:10 X vs. 16:10 X	-0.05733	-0.2013 to 0.08663	No

2way ANOVA<br>Multiple comparisons		A<br>Data Set-A<br>Y	B<br>Data Set-B<br>Y	C<br>Data Set-C<br>Y
3061	2:**10 X** vs. 16:**40 X**	-0.07667	-0.2206 to 0.06729	No
3062	2:**10 X** vs. 16:**50 X**	-0.1190	-0.2630 to 0.02496	No
3063	2:**10 X** vs. 17:**0 X**	-0.1933	-0.3373 to -0.04937	Yes
3064	2:**10 X** vs. 17:**1 X**	-0.1077	-0.2516 to 0.03629	No
3065	2:**10 X** vs. 17:**10 X**	-0.1643	-0.3083 to -0.02037	Yes
3066	2:**10 X** vs. 17:**40 X**	-0.08767	-0.2316 to 0.05629	No
3067	2:**10 X** vs. 17:**50 X**	-0.1330	-0.2770 to 0.01096	No
3068	2:**10 X** vs. 18:**0 X**	-0.2420	-0.3860 to -0.09804	Yes
3069	2:**10 X** vs. 18:**1 X**	-0.1503	-0.2943 to -0.006374	Yes
3070	2:**10 X** vs. 18:**10 X**	-0.2427	-0.3866 to -0.09871	Yes
3071	2:**10 X** vs. 18:**40 X**	-0.0920	-0.2360 to 0.05196	No
3072	2:**10 X** vs. 18:**50 X**	-0.1370	-0.2810 to 0.006960	No
3073	2:**10 X** vs. 19:**0 X**	-0.2770	-0.4210 to -0.1330	Yes
3074	2:**10 X** vs. 19:**1 X**	-0.2650	-0.4090 to -0.1210	Yes
3075	2:**10 X** vs. 19:**10 X**	-0.2627	-0.4066 to -0.1187	Yes
3076	2:**10 X** vs. 19:**40 X**	-0.1207	-0.2646 to 0.02329	No
3077	2:**10 X** vs. 19:**50 X**	-0.1317	-0.2756 to 0.01229	No
3078	2:**10 X** vs. 20:**0 X**	-0.3567	-0.5006 to -0.2127	Yes
3079	2:**10 X** vs. 20:**1 X**	-0.1760	-0.3200 to -0.03204	Yes
3080	2:**10 X** vs. 20:**10 X**	-0.2650	-0.4090 to -0.1210	Yes
3081	2:**10 X** vs. 20:**40 X**	-0.1323	-0.2763 to 0.01163	No
3082	2:**10 X** vs. 20:**50 X**	-0.1763	-0.3203 to -0.03237	Yes
3083	2:**10 X** vs. 21:**0 X**	-0.3800	-0.5240 to -0.2360	Yes
3084	2:**10 X** vs. 21:**1 X**	-0.3330	-0.4770 to -0.1890	Yes
3085	2:**10 X** vs. 21:**10 X**	-0.3300	-0.4740 to -0.1860	Yes
3086	2:**10 X** vs. 21:**40 X**	-0.2053	-0.3493 to -0.06137	Yes
3087	2:**10 X** vs. 21:**50 X**	-0.2957	-0.4396 to -0.1517	Yes
3088	2:**10 X** vs. 22:**0 X**	-0.4217	-0.5656 to -0.2777	Yes
3089	2:**10 X** vs. 22:**1 X**	-0.4163	-0.5603 to -0.2724	Yes
3090	2:**10 X** vs. 22:**10 X**	-0.4117	-0.5556 to -0.2677	Yes
3091	2:**10 X** vs. 22:**40 X**	-0.3120	-0.4560 to -0.1680	Yes
3092	2:**10 X** vs. 22:**50 X**	-0.3747	-0.5186 to -0.2307	Yes
3093	2:**10 X** vs. 23:**0 X**	-0.4870	-0.6310 to -0.3430	Yes
3094	2:**10 X** vs. 23:**1 X**	-0.6767	-0.8376 to -0.5157	Yes
3095	2:**10 X** vs. 23:**10 X**	-0.4263	-0.5703 to -0.2824	Yes
3096	2:**10 X** vs. 23:**40 X**	-0.3067	-0.4676 to -0.1457	Yes
3097	2:**10 X** vs. 23:**50 X**	-0.3407	-0.4846 to -0.1967	Yes
3098	2:**10 X** vs. 24:**0 X**	-0.6043	-0.7483 to -0.4604	Yes
3099	2:**10 X** vs. 24:**1 X**	-0.7607	-0.9216 to -0.5997	Yes
3100	2:**10 X** vs. 24:**10 X**	-0.5882	-0.7491 to -0.4272	Yes
3101	2:**10 X** vs. 24:**40 X**	-0.3230	-0.4670 to -0.1790	Yes
3102	2:**10 X** vs. 24:**50 X**	-0.2447	-0.3886 to -0.1007	Yes
3103	2:**10 X** vs. 25:**0 X**	-0.9417	-1.103 to -0.7807	Yes
3104	2:**10 X** vs. 25:**1 X**	-1.131	-1.292 to -0.9702	Yes
3105	2:**10 X** vs. 25:**10 X**	-0.4927	-0.6963 to -0.2891	Yes

	2way ANOVA<br>Multiple comparisons	A<br>Data Set-A<br>Y	B<br>Data Set-B<br>Y	C<br>Data Set-C<br>Y
3106	2:10 X vs. 25:40 X	-0.3250	-0.4690 to -0.1810	Yes
3107	2:10 X vs. 25:50 X	-0.2993	-0.4433 to -0.1554	Yes
3108	2:10 X vs. 26:0 X	-1.154	-1.315 to -0.9932	Yes
3109	2:10 X vs. 26:1 X	-1.267	-1.428 to -1.106	Yes
3110	2:10 X vs. 26:10 X	-0.9537	-1.115 to -0.7927	Yes
3111	2:10 X vs. 26:40 X	-0.3993	-0.5433 to -0.2554	Yes
3112	2:10 X vs. 26:50 X	-0.2597	-0.4036 to -0.1157	Yes
3113	2:10 X vs. 27:0 X	-1.342	-1.486 to -1.198	Yes
3114	2:10 X vs. 27:1 X	-1.433	-1.594 to -1.272	Yes
3115	2:10 X vs. 27:10 X	-1.245	-1.406 to -1.084	Yes
3116	2:10 X vs. 27:40 X	-0.3993	-0.5433 to -0.2554	Yes
3117	2:10 X vs. 27:50 X	-0.2590	-0.4030 to -0.1150	Yes
3118	2:10 X vs. 28:0 X	-1.291	-1.452 to -1.130	Yes
3119	2:10 X vs. 28:1 X	-1.332	-1.476 to -1.188	Yes
3120	2:10 X vs. 28:10 X	-1.134	-1.295 to -0.9727	Yes
3121	2:10 X vs. 28:40 X	-0.4117	-0.5556 to -0.2677	Yes
3122	2:10 X vs. 28:50 X	-0.3070	-0.4510 to -0.1630	Yes
3123	2:10 X vs. 29:0 X	-1.773	-1.934 to -1.612	Yes
3124	2:10 X vs. 29:1 X	-1.858	-2.019 to -1.697	Yes
3125	2:10 X vs. 29:10 X	-1.072	-1.233 to -0.9112	Yes
3126	2:10 X vs. 29:40 X	-0.5197	-0.6636 to -0.3757	Yes
3127	2:10 X vs. 29:50 X	-0.3083	-0.4523 to -0.1644	Yes
3128	2:10 X vs. 30:0 X	-1.487	-1.648 to -1.326	Yes
3129	2:10 X vs. 30:1 X	-1.809	-1.970 to -1.648	Yes
3130	2:10 X vs. 30:10 X	-1.147	-1.308 to -0.9857	Yes
3131	2:10 X vs. 30:40 X	-0.4467	-0.5906 to -0.3027	Yes
3132	2:10 X vs. 30:50 X	-0.3607	-0.5046 to -0.2167	Yes
3133	2:10 X vs. 31:0 X	-1.805	-1.966 to -1.644	Yes
3134	2:10 X vs. 31:1 X	-1.796	-1.957 to -1.635	Yes
3135	2:10 X vs. 31:10 X	-1.278	-1.439 to -1.117	Yes
3136	2:10 X vs. 31:40 X	-0.5383	-0.6823 to -0.3944	Yes
3137	2:10 X vs. 31:50 X	-0.3397	-0.4836 to -0.1957	Yes
3138	2:10 X vs. 32:0 X	-2.397	-2.541 to -2.253	Yes
3139	2:10 X vs. 32:1 X	-1.853	-2.014 to -1.692	Yes
3140	2:10 X vs. 32:10 X	-1.709	-1.853 to -1.565	Yes
3141	2:10 X vs. 32:40 X	-0.5347	-0.6786 to -0.3907	Yes
3142	2:10 X vs. 32:50 X	-0.3393	-0.4833 to -0.1954	Yes
3143	2:10 X vs. 33:0 X	-2.400	-2.544 to -2.256	Yes
3144	2:10 X vs. 33:1 X	-2.215	-2.359 to -2.071	Yes
3145	2:10 X vs. 33:10 X	-1.875	-2.019 to -1.731	Yes
3146	2:10 X vs. 33:40 X	-0.5533	-0.6973 to -0.4094	Yes
3147	2:10 X vs. 33:50 X	-0.3543	-0.4983 to -0.2104	Yes
3148	2:10 X vs. 34:0 X	-2.325	-2.469 to -2.181	Yes
3149	2:10 X vs. 34:1 X	-2.332	-2.476 to -2.188	Yes
3150	2:10 X vs. 34:10 X	-1.966	-2.110 to -1.822	Yes

	2way ANOVA<br>Multiple comparisons	A<br>Data Set-A<br>Y	B<br>Data Set-B<br>Y	C<br>Data Set-C<br>Y
3151	2:**10 X** vs. 34:**40 X**	-0.5653	-0.7093 to -0.4214	Yes
3152	2:**10 X** vs. 34:**50 X**	-0.5033	-0.6473 to -0.3594	Yes
3153	2:**10 X** vs. 35:**0 X**	-2.674	-2.818 to -2.530	Yes
3154	2:**10 X** vs. 35:**1 X**	-2.439	-2.583 to -2.295	Yes
3155	2:**10 X** vs. 35:**10 X**	-1.927	-2.071 to -1.783	Yes
3156	2:**10 X** vs. 35:**40 X**	-0.5743	-0.7183 to -0.4304	Yes
3157	2:**10 X** vs. 35:**50 X**	-0.5160	-0.6600 to -0.3720	Yes
3158	2:**10 X** vs. 36:**0 X**	-2.880	-3.041 to -2.719	Yes
3159	2:**10 X** vs. 36:**1 X**	-2.576	-2.720 to -2.432	Yes
3160	2:**10 X** vs. 36:**10 X**	-1.933	-2.077 to -1.789	Yes
3161	2:**10 X** vs. 36:**40 X**	-0.6297	-0.7736 to -0.4857	Yes
3162	2:**10 X** vs. 36:**50 X**	-0.4793	-0.6233 to -0.3354	Yes
3163	2:**10 X** vs. 37:**0 X**	-3.264	-3.408 to -3.120	Yes
3164	2:**10 X** vs. 37:**1 X**	-2.646	-2.790 to -2.502	Yes
3165	2:**10 X** vs. 37:**10 X**	-1.935	-2.079 to -1.791	Yes
3166	2:**10 X** vs. 37:**40 X**	-0.6670	-0.8110 to -0.5230	Yes
3167	2:**10 X** vs. 37:**50 X**	-0.4880	-0.6320 to -0.3440	Yes
3168	2:**10 X** vs. 38:**0 X**	-3.209	-3.353 to -3.065	Yes
3169	2:**10 X** vs. 38:**1 X**	-2.765	-2.909 to -2.621	Yes
3170	2:**10 X** vs. 38:**10 X**	-2.210	-2.354 to -2.066	Yes
3171	2:**10 X** vs. 38:**40 X**	-0.6713	-0.8153 to -0.5274	Yes
3172	2:**10 X** vs. 38:**50 X**	-0.4920	-0.6360 to -0.3480	Yes
3173	2:**10 X** vs. 39:**0 X**	-3.440	-3.584 to -3.296	Yes
3174	2:**10 X** vs. 39:**1 X**	-3.331	-3.475 to -3.187	Yes
3175	2:**10 X** vs. 39:**10 X**	-2.355	-2.499 to -2.211	Yes
3176	2:**10 X** vs. 39:**40 X**	-0.6773	-0.8213 to -0.5334	Yes
3177	2:**10 X** vs. 39:**50 X**	-0.5230	-0.6670 to -0.3790	Yes
3178	2:**10 X** vs. 40:**0 X**	-3.481	-3.625 to -3.337	Yes
3179	2:**10 X** vs. 40:**1 X**	-3.407	-3.551 to -3.263	Yes
3180	2:**10 X** vs. 40:**10 X**	-2.611	-2.755 to -2.467	Yes
3181	2:**10 X** vs. 40:**40 X**	-0.6820	-0.8260 to -0.5380	Yes
3182	2:**10 X** vs. 40:**50 X**	-0.5293	-0.6733 to -0.3854	Yes
3183	2:**10 X** vs. 41:**0 X**	-3.522	-3.666 to -3.378	Yes
3184	2:**10 X** vs. 41:**1 X**	-3.521	-3.665 to -3.377	Yes
3185	2:**10 X** vs. 41:**10 X**	-2.665	-2.809 to -2.521	Yes
3186	2:**10 X** vs. 41:**40 X**	-0.6293	-0.7733 to -0.4854	Yes
3187	2:**10 X** vs. 41:**50 X**	-0.5420	-0.6860 to -0.3980	Yes
3188	2:**10 X** vs. 42:**0 X**	-3.663	-3.807 to -3.519	Yes
3189	2:**10 X** vs. 42:**1 X**	-3.683	-3.827 to -3.539	Yes
3190	2:**10 X** vs. 42:**10 X**	-2.665	-2.809 to -2.521	Yes
3191	2:**10 X** vs. 42:**40 X**	-0.6420	-0.7860 to -0.4980	Yes
3192	2:**10 X** vs. 42:**50 X**	-0.5453	-0.6893 to -0.4014	Yes
3193	2:**10 X** vs. 43:**0 X**	-3.753	-3.897 to -3.609	Yes
3194	2:**10 X** vs. 43:**1 X**	-3.668	-3.812 to -3.524	Yes
3195	2:**10 X** vs. 43:**10 X**	-3.383	-3.527 to -3.239	Yes

	2way ANOVA<br>Multiple comparisons	A<br>Data Set-A<br>Y	B<br>Data Set-B<br>Y	C<br>Data Set-C<br>Y
3196	2:10 X vs. 43:40 X	-0.6403	-0.7843 to -0.4964	Yes
3197	2:10 X vs. 43:50 X	-0.5457	-0.6896 to -0.4017	Yes
3198	2:10 X vs. 44:0 X	-3.777	-3.921 to -3.633	Yes
3199	2:10 X vs. 44:1 X	-3.667	-3.811 to -3.523	Yes
3200	2:10 X vs. 44:10 X	-3.447	-3.591 to -3.303	Yes
3201	2:10 X vs. 44:40 X	-0.6423	-0.7863 to -0.4984	Yes
3202	2:10 X vs. 44:50 X	-0.4780	-0.6220 to -0.3340	Yes
3203	2:10 X vs. 45:0 X	-3.642	-3.786 to -3.498	Yes
3204	2:10 X vs. 45:1 X	-3.444	-3.588 to -3.300	Yes
3205	2:10 X vs. 45:10 X	-3.326	-3.470 to -3.182	Yes
3206	2:10 X vs. 45:40 X	-0.6437	-0.7876 to -0.4997	Yes
3207	2:10 X vs. 45:50 X	-0.4750	-0.6190 to -0.3310	Yes
3208	2:10 X vs. 46:0 X	-3.446	-3.590 to -3.302	Yes
3209	2:10 X vs. 46:1 X	-3.413	-3.557 to -3.269	Yes
3210	2:10 X vs. 46:10 X	-3.321	-3.465 to -3.177	Yes
3211	2:10 X vs. 46:40 X	-0.6573	-0.8013 to -0.5134	Yes
3212	2:10 X vs. 46:50 X	-0.4680	-0.6120 to -0.3240	Yes
3213	2:10 X vs. 47:0 X	-3.422	-3.566 to -3.278	Yes
3214	2:10 X vs. 47:1 X	-3.326	-3.470 to -3.182	Yes
3215	2:10 X vs. 47:10 X	-3.307	-3.451 to -3.163	Yes
3216	2:10 X vs. 47:40 X	-0.6673	-0.8113 to -0.5234	Yes
3217	2:10 X vs. 47:50 X	-0.4260	-0.5700 to -0.2820	Yes
3218	2:10 X vs. 48:0 X	-3.409	-3.570 to -3.248	Yes
3219	2:10 X vs. 48:1 X	-3.313	-3.457 to -3.169	Yes
3220	2:10 X vs. 48:10 X	-3.307	-3.451 to -3.163	Yes
3221	2:10 X vs. 48:40 X	-0.6737	-0.8176 to -0.5297	Yes
3222	2:10 X vs. 48:50 X	-0.4333	-0.5773 to -0.2894	Yes
3223	2:10 X vs. 49:0 X	-3.409	-3.553 to -3.265	Yes
3224	2:10 X vs. 49:1 X	-3.313	-3.457 to -3.169	Yes
3225	2:10 X vs. 49:10 X	-3.307	-3.451 to -3.163	Yes
3226	2:10 X vs. 49:40 X	-0.6780	-0.8220 to -0.5340	Yes
3227	2:10 X vs. 49:50 X	-0.4327	-0.5766 to -0.2887	Yes
3228	2:10 X vs. 50:0 X	-3.409	-3.553 to -3.265	Yes
3229	2:10 X vs. 50:1 X	-3.313	-3.457 to -3.169	Yes
3230	2:10 X vs. 50:10 X	-3.307	-3.451 to -3.163	Yes
3231	2:10 X vs. 50:40 X	-0.6857	-0.8296 to -0.5417	Yes
3232	2:10 X vs. 50:50 X	-0.4323	-0.5763 to -0.2884	Yes
3233	2:40 X vs. 2:50 X	0.0030	-0.1410 to 0.1470	No
3234	2:40 X vs. 3:0 X	-0.001333	-0.1453 to 0.1426	No
3235	2:40 X vs. 3:1 X	-0.003667	-0.1476 to 0.1403	No
3236	2:40 X vs. 3:10 X	0.0010	-0.1430 to 0.1450	No
3237	2:40 X vs. 3:40 X	0.003333	-0.1406 to 0.1473	No
3238	2:40 X vs. 3:50 X	0.004333	-0.1396 to 0.1483	No
3239	2:40 X vs. 4:0 X	-0.005667	-0.1496 to 0.1383	No
3240	2:40 X vs. 4:1 X	-0.004333	-0.1483 to 0.1396	No

2way ANOVA<br>Multiple comparisons		A<br>Data Set-A<br>Y	B<br>Data Set-B<br>Y	C<br>Data Set-C<br>Y
3241	2:40 X vs. 4:10 X	-0.0190	-0.1630 to 0.1250	No
3242	2:40 X vs. 4:40 X	0.001333	-0.1426 to 0.1453	No
3243	2:40 X vs. 4:50 X	0.003333	-0.1406 to 0.1473	No
3244	2:40 X vs. 5:0 X	-0.006667	-0.1506 to 0.1373	No
3245	2:40 X vs. 5:1 X	-0.004667	-0.1486 to 0.1393	No
3246	2:40 X vs. 5:10 X	-0.01967	-0.1636 to 0.1243	No
3247	2:40 X vs. 5:40 X	0.001333	-0.1426 to 0.1453	No
3248	2:40 X vs. 5:50 X	0.002333	-0.1416 to 0.1463	No
3249	2:40 X vs. 6:0 X	-0.0060	-0.1500 to 0.1380	No
3250	2:40 X vs. 6:1 X	-0.005000	-0.1490 to 0.1390	No
3251	2:40 X vs. 6:10 X	-0.01967	-0.1636 to 0.1243	No
3252	2:40 X vs. 6:40 X	-0.0010	-0.1450 to 0.1430	No
3253	2:40 X vs. 6:50 X	0.0006667	-0.1433 to 0.1446	No
3254	2:40 X vs. 7:0 X	-0.008000	-0.1520 to 0.1360	No
3255	2:40 X vs. 7:1 X	0.0006667	-0.1433 to 0.1446	No
3256	2:40 X vs. 7:10 X	-0.01133	-0.1553 to 0.1326	No
3257	2:40 X vs. 7:40 X	-0.002667	-0.1466 to 0.1413	No
3258	2:40 X vs. 7:50 X	-0.0006667	-0.1446 to 0.1433	No
3259	2:40 X vs. 8:0 X	-0.01267	-0.1566 to 0.1313	No
3260	2:40 X vs. 8:1 X	0.0006667	-0.1433 to 0.1446	No
3261	2:40 X vs. 8:10 X	-0.006667	-0.1506 to 0.1373	No
3262	2:40 X vs. 8:40 X	-0.007333	-0.1513 to 0.1366	No
3263	2:40 X vs. 8:50 X	-0.004333	-0.1483 to 0.1396	No
3264	2:40 X vs. 9:0 X	-0.02067	-0.1646 to 0.1233	No
3265	2:40 X vs. 9:1 X	-0.02867	-0.1726 to 0.1153	No
3266	2:40 X vs. 9:10 X	-0.02967	-0.1736 to 0.1143	No
3267	2:40 X vs. 9:40 X	-0.0120	-0.1560 to 0.1320	No
3268	2:40 X vs. 9:50 X	-0.005333	-0.1493 to 0.1386	No
3269	2:40 X vs. 10:0 X	-0.02233	-0.1663 to 0.1216	No
3270	2:40 X vs. 10:1 X	-0.02933	-0.1733 to 0.1146	No
3271	2:40 X vs. 10:10 X	-0.0320	-0.1760 to 0.1120	No
3272	2:40 X vs. 10:40 X	-0.02167	-0.1656 to 0.1223	No
3273	2:40 X vs. 10:50 X	-0.008333	-0.1523 to 0.1356	No
3274	2:40 X vs. 11:0 X	-0.0320	-0.1760 to 0.1120	No
3275	2:40 X vs. 11:1 X	-0.03367	-0.1776 to 0.1103	No
3276	2:40 X vs. 11:10 X	-0.0340	-0.1780 to 0.1100	No
3277	2:40 X vs. 11:40 X	-0.02567	-0.1696 to 0.1183	No
3278	2:40 X vs. 11:50 X	-0.01367	-0.1576 to 0.1303	No
3279	2:40 X vs. 12:0 X	-0.03267	-0.1766 to 0.1113	No
3280	2:40 X vs. 12:1 X	-0.0150	-0.1590 to 0.1290	No
3281	2:40 X vs. 12:10 X	-0.03267	-0.1766 to 0.1113	No
3282	2:40 X vs. 12:40 X	-0.0190	-0.1630 to 0.1250	No
3283	2:40 X vs. 12:50 X	-0.02233	-0.1663 to 0.1216	No
3284	2:40 X vs. 13:0 X	-0.02667	-0.1706 to 0.1173	No
3285	2:40 X vs. 13:1 X	-0.0140	-0.1580 to 0.1300	No

	2way ANOVA<br>Multiple comparisons	A<br>Data Set-A<br>Y	B<br>Data Set-B<br>Y	C<br>Data Set-C<br>Y
3286	2:40 X vs. 13:10 X	-0.0360	-0.1800 to 0.1080	No
3287	2:40 X vs. 13:40 X	-0.03233	-0.1763 to 0.1116	No
3288	2:40 X vs. 13:50 X	-0.03333	-0.1773 to 0.1106	No
3289	2:40 X vs. 14:0 X	-0.03533	-0.1793 to 0.1086	No
3290	2:40 X vs. 14:1 X	-0.0270	-0.1710 to 0.1170	No
3291	2:40 X vs. 14:10 X	-0.0410	-0.1850 to 0.1030	No
3292	2:40 X vs. 14:40 X	-0.03767	-0.1816 to 0.1063	No
3293	2:40 X vs. 14:50 X	-0.04167	-0.1856 to 0.1023	No
3294	2:40 X vs. 15:0 X	-0.04767	-0.1916 to 0.09629	No
3295	2:40 X vs. 15:1 X	-0.02767	-0.1716 to 0.1163	No
3296	2:40 X vs. 15:10 X	-0.0550	-0.1990 to 0.08896	No
3297	2:40 X vs. 15:40 X	-0.03733	-0.1813 to 0.1066	No
3298	2:40 X vs. 15:50 X	-0.0550	-0.1990 to 0.08896	No
3299	2:40 X vs. 16:0 X	-0.1400	-0.2840 to 0.003960	No
3300	2:40 X vs. 16:1 X	-0.0310	-0.1750 to 0.1130	No
3301	2:40 X vs. 16:10 X	-0.05667	-0.2006 to 0.08729	No
3302	2:40 X vs. 16:40 X	-0.0760	-0.2200 to 0.06796	No
3303	2:40 X vs. 16:50 X	-0.1183	-0.2623 to 0.02563	No
3304	2:40 X vs. 17:0 X	-0.1927	-0.3366 to -0.04871	Yes
3305	2:40 X vs. 17:1 X	-0.1070	-0.2510 to 0.03696	No
3306	2:40 X vs. 17:10 X	-0.1637	-0.3076 to -0.01971	Yes
3307	2:40 X vs. 17:40 X	-0.0870	-0.2310 to 0.05696	No
3308	2:40 X vs. 17:50 X	-0.1323	-0.2763 to 0.01163	No
3309	2:40 X vs. 18:0 X	-0.2413	-0.3853 to -0.09737	Yes
3310	2:40 X vs. 18:1 X	-0.1497	-0.2936 to -0.005707	Yes
3311	2:40 X vs. 18:10 X	-0.2420	-0.3860 to -0.09804	Yes
3312	2:40 X vs. 18:40 X	-0.09133	-0.2353 to 0.05263	No
3313	2:40 X vs. 18:50 X	-0.1363	-0.2803 to 0.007626	No
3314	2:40 X vs. 19:0 X	-0.2763	-0.4203 to -0.1324	Yes
3315	2:40 X vs. 19:1 X	-0.2643	-0.4083 to -0.1204	Yes
3316	2:40 X vs. 19:10 X	-0.2620	-0.4060 to -0.1180	Yes
3317	2:40 X vs. 19:40 X	-0.1200	-0.2640 to 0.02396	No
3318	2:40 X vs. 19:50 X	-0.1310	-0.2750 to 0.01296	No
3319	2:40 X vs. 20:0 X	-0.3560	-0.5000 to -0.2120	Yes
3320	2:40 X vs. 20:1 X	-0.1753	-0.3193 to -0.03137	Yes
3321	2:40 X vs. 20:10 X	-0.2643	-0.4083 to -0.1204	Yes
3322	2:40 X vs. 20:40 X	-0.1317	-0.2756 to 0.01229	No
3323	2:40 X vs. 20:50 X	-0.1757	-0.3196 to -0.03171	Yes
3324	2:40 X vs. 21:0 X	-0.3793	-0.5233 to -0.2354	Yes
3325	2:40 X vs. 21:1 X	-0.3323	-0.4763 to -0.1884	Yes
3326	2:40 X vs. 21:10 X	-0.3293	-0.4733 to -0.1854	Yes
3327	2:40 X vs. 21:40 X	-0.2047	-0.3486 to -0.06071	Yes
3328	2:40 X vs. 21:50 X	-0.2950	-0.4390 to -0.1510	Yes
3329	2:40 X vs. 22:0 X	-0.4210	-0.5650 to -0.2770	Yes
3330	2:40 X vs. 22:1 X	-0.4157	-0.5596 to -0.2717	Yes

	2way ANOVA<br>Multiple comparisons	A<br>Data Set-A<br>Y	B<br>Data Set-B<br>Y	C<br>Data Set-C<br>Y
3331	2:40 X vs. 22:10 X	-0.4110	-0.5550 to -0.2670	Yes
3332	2:40 X vs. 22:40 X	-0.3113	-0.4553 to -0.1674	Yes
3333	2:40 X vs. 22:50 X	-0.3740	-0.5180 to -0.2300	Yes
3334	2:40 X vs. 23:0 X	-0.4863	-0.6303 to -0.3424	Yes
3335	2:40 X vs. 23:1 X	-0.6760	-0.8370 to -0.5150	Yes
3336	2:40 X vs. 23:10 X	-0.4257	-0.5696 to -0.2817	Yes
3337	2:40 X vs. 23:40 X	-0.3060	-0.4670 to -0.1450	Yes
3338	2:40 X vs. 23:50 X	-0.3400	-0.4840 to -0.1960	Yes
3339	2:40 X vs. 24:0 X	-0.6037	-0.7476 to -0.4597	Yes
3340	2:40 X vs. 24:1 X	-0.7600	-0.9210 to -0.5990	Yes
3341	2:40 X vs. 24:10 X	-0.5875	-0.7485 to -0.4265	Yes
3342	2:40 X vs. 24:40 X	-0.3223	-0.4663 to -0.1784	Yes
3343	2:40 X vs. 24:50 X	-0.2440	-0.3880 to -0.1000	Yes
3344	2:40 X vs. 25:0 X	-0.9410	-1.102 to -0.7800	Yes
3345	2:40 X vs. 25:1 X	-1.131	-1.291 to -0.9695	Yes
3346	2:40 X vs. 25:10 X	-0.4920	-0.6956 to -0.2884	Yes
3347	2:40 X vs. 25:40 X	-0.3243	-0.4683 to -0.1804	Yes
3348	2:40 X vs. 25:50 X	-0.2987	-0.4426 to -0.1547	Yes
3349	2:40 X vs. 26:0 X	-1.154	-1.314 to -0.9925	Yes
3350	2:40 X vs. 26:1 X	-1.267	-1.427 to -1.106	Yes
3351	2:40 X vs. 26:10 X	-0.9530	-1.114 to -0.7920	Yes
3352	2:40 X vs. 26:40 X	-0.3987	-0.5426 to -0.2547	Yes
3353	2:40 X vs. 26:50 X	-0.2590	-0.4030 to -0.1150	Yes
3354	2:40 X vs. 27:0 X	-1.341	-1.485 to -1.197	Yes
3355	2:40 X vs. 27:1 X	-1.432	-1.593 to -1.271	Yes
3356	2:40 X vs. 27:10 X	-1.244	-1.405 to -1.083	Yes
3357	2:40 X vs. 27:40 X	-0.3987	-0.5426 to -0.2547	Yes
3358	2:40 X vs. 27:50 X	-0.2583	-0.4023 to -0.1144	Yes
3359	2:40 X vs. 28:0 X	-1.291	-1.451 to -1.130	Yes
3360	2:40 X vs. 28:1 X	-1.331	-1.475 to -1.187	Yes
3361	2:40 X vs. 28:10 X	-1.133	-1.294 to -0.9720	Yes
3362	2:40 X vs. 28:40 X	-0.4110	-0.5550 to -0.2670	Yes
3363	2:40 X vs. 28:50 X	-0.3063	-0.4503 to -0.1624	Yes
3364	2:40 X vs. 29:0 X	-1.772	-1.933 to -1.611	Yes
3365	2:40 X vs. 29:1 X	-1.857	-2.018 to -1.696	Yes
3366	2:40 X vs. 29:10 X	-1.072	-1.232 to -0.9105	Yes
3367	2:40 X vs. 29:40 X	-0.5190	-0.6630 to -0.3750	Yes
3368	2:40 X vs. 29:50 X	-0.3077	-0.4516 to -0.1637	Yes
3369	2:40 X vs. 30:0 X	-1.487	-1.647 to -1.326	Yes
3370	2:40 X vs. 30:1 X	-1.808	-1.969 to -1.647	Yes
3371	2:40 X vs. 30:10 X	-1.146	-1.307 to -0.9850	Yes
3372	2:40 X vs. 30:40 X	-0.4460	-0.5900 to -0.3020	Yes
3373	2:40 X vs. 30:50 X	-0.3600	-0.5040 to -0.2160	Yes
3374	2:40 X vs. 31:0 X	-1.805	-1.965 to -1.644	Yes
3375	2:40 X vs. 31:1 X	-1.795	-1.956 to -1.634	Yes

	2way ANOVA<br>Multiple comparisons	A<br>Data Set-A<br>Y	B<br>Data Set-B<br>Y	C<br>Data Set-C<br>Y
3376	2:40 X vs. 31:10 X	-1.278	-1.438 to -1.117	Yes
3377	2:40 X vs. 31:40 X	-0.5377	-0.6816 to -0.3937	Yes
3378	2:40 X vs. 31:50 X	-0.3390	-0.4830 to -0.1950	Yes
3379	2:40 X vs. 32:0 X	-2.396	-2.540 to -2.252	Yes
3380	2:40 X vs. 32:1 X	-1.852	-2.013 to -1.691	Yes
3381	2:40 X vs. 32:10 X	-1.708	-1.852 to -1.564	Yes
3382	2:40 X vs. 32:40 X	-0.5340	-0.6780 to -0.3900	Yes
3383	2:40 X vs. 32:50 X	-0.3387	-0.4826 to -0.1947	Yes
3384	2:40 X vs. 33:0 X	-2.400	-2.544 to -2.256	Yes
3385	2:40 X vs. 33:1 X	-2.214	-2.358 to -2.070	Yes
3386	2:40 X vs. 33:10 X	-1.875	-2.019 to -1.731	Yes
3387	2:40 X vs. 33:40 X	-0.5527	-0.6966 to -0.4087	Yes
3388	2:40 X vs. 33:50 X	-0.3537	-0.4976 to -0.2097	Yes
3389	2:40 X vs. 34:0 X	-2.324	-2.468 to -2.180	Yes
3390	2:40 X vs. 34:1 X	-2.332	-2.476 to -2.188	Yes
3391	2:40 X vs. 34:10 X	-1.965	-2.109 to -1.821	Yes
3392	2:40 X vs. 34:40 X	-0.5647	-0.7086 to -0.4207	Yes
3393	2:40 X vs. 34:50 X	-0.5027	-0.6466 to -0.3587	Yes
3394	2:40 X vs. 35:0 X	-2.673	-2.817 to -2.529	Yes
3395	2:40 X vs. 35:1 X	-2.439	-2.583 to -2.295	Yes
3396	2:40 X vs. 35:10 X	-1.927	-2.071 to -1.783	Yes
3397	2:40 X vs. 35:40 X	-0.5737	-0.7176 to -0.4297	Yes
3398	2:40 X vs. 35:50 X	-0.5153	-0.6593 to -0.3714	Yes
3399	2:40 X vs. 36:0 X	-2.880	-3.040 to -2.719	Yes
3400	2:40 X vs. 36:1 X	-2.575	-2.719 to -2.431	Yes
3401	2:40 X vs. 36:10 X	-1.932	-2.076 to -1.788	Yes
3402	2:40 X vs. 36:40 X	-0.6290	-0.7730 to -0.4850	Yes
3403	2:40 X vs. 36:50 X	-0.4787	-0.6226 to -0.3347	Yes
3404	2:40 X vs. 37:0 X	-3.263	-3.407 to -3.119	Yes
3405	2:40 X vs. 37:1 X	-2.645	-2.789 to -2.501	Yes
3406	2:40 X vs. 37:10 X	-1.934	-2.078 to -1.790	Yes
3407	2:40 X vs. 37:40 X	-0.6663	-0.8103 to -0.5224	Yes
3408	2:40 X vs. 37:50 X	-0.4873	-0.6313 to -0.3434	Yes
3409	2:40 X vs. 38:0 X	-3.208	-3.352 to -3.064	Yes
3410	2:40 X vs. 38:1 X	-2.764	-2.908 to -2.620	Yes
3411	2:40 X vs. 38:10 X	-2.209	-2.353 to -2.065	Yes
3412	2:40 X vs. 38:40 X	-0.6707	-0.8146 to -0.5267	Yes
3413	2:40 X vs. 38:50 X	-0.4913	-0.6353 to -0.3474	Yes
3414	2:40 X vs. 39:0 X	-3.439	-3.583 to -3.295	Yes
3415	2:40 X vs. 39:1 X	-3.331	-3.475 to -3.187	Yes
3416	2:40 X vs. 39:10 X	-2.354	-2.498 to -2.210	Yes
3417	2:40 X vs. 39:40 X	-0.6767	-0.8206 to -0.5327	Yes
3418	2:40 X vs. 39:50 X	-0.5223	-0.6663 to -0.3784	Yes
3419	2:40 X vs. 40:0 X	-3.480	-3.624 to -3.336	Yes
3420	2:40 X vs. 40:1 X	-3.406	-3.550 to -3.262	Yes

	2way ANOVA<br>Multiple comparisons	A<br>Data Set-A<br>Y	B<br>Data Set-B<br>Y	C<br>Data Set-C<br>Y
3421	2:40 X vs. 40:10 X	-2.611	-2.755 to -2.467	Yes
3422	2:40 X vs. 40:40 X	-0.6813	-0.8253 to -0.5374	Yes
3423	2:40 X vs. 40:50 X	-0.5287	-0.6726 to -0.3847	Yes
3424	2:40 X vs. 41:0 X	-3.521	-3.665 to -3.377	Yes
3425	2:40 X vs. 41:1 X	-3.520	-3.664 to -3.376	Yes
3426	2:40 X vs. 41:10 X	-2.664	-2.808 to -2.520	Yes
3427	2:40 X vs. 41:40 X	-0.6287	-0.7726 to -0.4847	Yes
3428	2:40 X vs. 41:50 X	-0.5413	-0.6853 to -0.3974	Yes
3429	2:40 X vs. 42:0 X	-3.663	-3.807 to -3.519	Yes
3430	2:40 X vs. 42:1 X	-3.682	-3.826 to -3.538	Yes
3431	2:40 X vs. 42:10 X	-2.665	-2.809 to -2.521	Yes
3432	2:40 X vs. 42:40 X	-0.6413	-0.7853 to -0.4974	Yes
3433	2:40 X vs. 42:50 X	-0.5447	-0.6886 to -0.4007	Yes
3434	2:40 X vs. 43:0 X	-3.753	-3.897 to -3.609	Yes
3435	2:40 X vs. 43:1 X	-3.667	-3.811 to -3.523	Yes
3436	2:40 X vs. 43:10 X	-3.383	-3.527 to -3.239	Yes
3437	2:40 X vs. 43:40 X	-0.6397	-0.7836 to -0.4957	Yes
3438	2:40 X vs. 43:50 X	-0.5450	-0.6890 to -0.4010	Yes
3439	2:40 X vs. 44:0 X	-3.777	-3.921 to -3.633	Yes
3440	2:40 X vs. 44:1 X	-3.666	-3.810 to -3.522	Yes
3441	2:40 X vs. 44:10 X	-3.446	-3.590 to -3.302	Yes
3442	2:40 X vs. 44:40 X	-0.6417	-0.7856 to -0.4977	Yes
3443	2:40 X vs. 44:50 X	-0.4773	-0.6213 to -0.3334	Yes
3444	2:40 X vs. 45:0 X	-3.641	-3.785 to -3.497	Yes
3445	2:40 X vs. 45:1 X	-3.443	-3.587 to -3.299	Yes
3446	2:40 X vs. 45:10 X	-3.326	-3.470 to -3.182	Yes
3447	2:40 X vs. 45:40 X	-0.6430	-0.7870 to -0.4990	Yes
3448	2:40 X vs. 45:50 X	-0.4743	-0.6183 to -0.3304	Yes
3449	2:40 X vs. 46:0 X	-3.446	-3.590 to -3.302	Yes
3450	2:40 X vs. 46:1 X	-3.413	-3.557 to -3.269	Yes
3451	2:40 X vs. 46:10 X	-3.321	-3.465 to -3.177	Yes
3452	2:40 X vs. 46:40 X	-0.6567	-0.8006 to -0.5127	Yes
3453	2:40 X vs. 46:50 X	-0.4673	-0.6113 to -0.3234	Yes
3454	2:40 X vs. 47:0 X	-3.421	-3.565 to -3.277	Yes
3455	2:40 X vs. 47:1 X	-3.325	-3.469 to -3.181	Yes
3456	2:40 X vs. 47:10 X	-3.307	-3.451 to -3.163	Yes
3457	2:40 X vs. 47:40 X	-0.6667	-0.8106 to -0.5227	Yes
3458	2:40 X vs. 47:50 X	-0.4253	-0.5693 to -0.2814	Yes
3459	2:40 X vs. 48:0 X	-3.409	-3.569 to -3.248	Yes
3460	2:40 X vs. 48:1 X	-3.312	-3.456 to -3.168	Yes
3461	2:40 X vs. 48:10 X	-3.307	-3.451 to -3.163	Yes
3462	2:40 X vs. 48:40 X	-0.6730	-0.8170 to -0.5290	Yes
3463	2:40 X vs. 48:50 X	-0.4327	-0.5766 to -0.2887	Yes
3464	2:40 X vs. 49:0 X	-3.409	-3.553 to -3.265	Yes
3465	2:40 X vs. 49:1 X	-3.312	-3.456 to -3.168	Yes

	2way ANOVA<br>Multiple comparisons	A<br>Data Set-A<br>Y	B<br>Data Set-B<br>Y	C<br>Data Set-C<br>Y
3466	2:40 X vs. 49:10 X	-3.307	-3.451 to -3.163	Yes
3467	2:40 X vs. 49:40 X	-0.6773	-0.8213 to -0.5334	Yes
3468	2:40 X vs. 49:50 X	-0.4320	-0.5760 to -0.2880	Yes
3469	2:40 X vs. 50:0 X	-3.409	-3.553 to -3.265	Yes
3470	2:40 X vs. 50:1 X	-3.312	-3.456 to -3.168	Yes
3471	2:40 X vs. 50:10 X	-3.307	-3.451 to -3.163	Yes
3472	2:40 X vs. 50:40 X	-0.6850	-0.8290 to -0.5410	Yes
3473	2:40 X vs. 50:50 X	-0.4317	-0.5756 to -0.2877	Yes
3474	2:50 X vs. 3:0 X	-0.004333	-0.1483 to 0.1396	No
3475	2:50 X vs. 3:1 X	-0.006667	-0.1506 to 0.1373	No
3476	2:50 X vs. 3:10 X	-0.0020	-0.1460 to 0.1420	No
3477	2:50 X vs. 3:40 X	0.0003333	-0.1436 to 0.1443	No
3478	2:50 X vs. 3:50 X	0.001333	-0.1426 to 0.1453	No
3479	2:50 X vs. 4:0 X	-0.008667	-0.1526 to 0.1353	No
3480	2:50 X vs. 4:1 X	-0.007333	-0.1513 to 0.1366	No
3481	2:50 X vs. 4:10 X	-0.0220	-0.1660 to 0.1220	No
3482	2:50 X vs. 4:40 X	-0.001667	-0.1456 to 0.1423	No
3483	2:50 X vs. 4:50 X	0.0003333	-0.1436 to 0.1443	No
3484	2:50 X vs. 5:0 X	-0.009667	-0.1536 to 0.1343	No
3485	2:50 X vs. 5:1 X	-0.007667	-0.1516 to 0.1363	No
3486	2:50 X vs. 5:10 X	-0.02267	-0.1666 to 0.1213	No
3487	2:50 X vs. 5:40 X	-0.001667	-0.1456 to 0.1423	No
3488	2:50 X vs. 5:50 X	-0.0006667	-0.1446 to 0.1433	No
3489	2:50 X vs. 6:0 X	-0.0090	-0.1530 to 0.1350	No
3490	2:50 X vs. 6:1 X	-0.008000	-0.1520 to 0.1360	No
3491	2:50 X vs. 6:10 X	-0.02267	-0.1666 to 0.1213	No
3492	2:50 X vs. 6:40 X	-0.0040	-0.1480 to 0.1400	No
3493	2:50 X vs. 6:50 X	-0.002333	-0.1463 to 0.1416	No
3494	2:50 X vs. 7:0 X	-0.0110	-0.1550 to 0.1330	No
3495	2:50 X vs. 7:1 X	-0.002333	-0.1463 to 0.1416	No
3496	2:50 X vs. 7:10 X	-0.01433	-0.1583 to 0.1296	No
3497	2:50 X vs. 7:40 X	-0.005667	-0.1496 to 0.1383	No
3498	2:50 X vs. 7:50 X	-0.003667	-0.1476 to 0.1403	No
3499	2:50 X vs. 8:0 X	-0.01567	-0.1596 to 0.1283	No
3500	2:50 X vs. 8:1 X	-0.002333	-0.1463 to 0.1416	No
3501	2:50 X vs. 8:10 X	-0.009667	-0.1536 to 0.1343	No
3502	2:50 X vs. 8:40 X	-0.01033	-0.1543 to 0.1336	No
3503	2:50 X vs. 8:50 X	-0.007333	-0.1513 to 0.1366	No
3504	2:50 X vs. 9:0 X	-0.02367	-0.1676 to 0.1203	No
3505	2:50 X vs. 9:1 X	-0.03167	-0.1756 to 0.1123	No
3506	2:50 X vs. 9:10 X	-0.03267	-0.1766 to 0.1113	No
3507	2:50 X vs. 9:40 X	-0.0150	-0.1590 to 0.1290	No
3508	2:50 X vs. 9:50 X	-0.008333	-0.1523 to 0.1356	No
3509	2:50 X vs. 10:0 X	-0.02533	-0.1693 to 0.1186	No
3510	2:50 X vs. 10:1 X	-0.03233	-0.1763 to 0.1116	No

	2way ANOVA<br>Multiple comparisons	A<br>Data Set-A<br>Y	B<br>Data Set-B<br>Y	C<br>Data Set-C<br>Y
3511	2:**50 X** vs. 10:**10 X**	-0.0350	-0.1790 to 0.1090	No
3512	2:**50 X** vs. 10:**40 X**	-0.02467	-0.1686 to 0.1193	No
3513	2:**50 X** vs. 10:**50 X**	-0.01133	-0.1553 to 0.1326	No
3514	2:**50 X** vs. 11:**0 X**	-0.0350	-0.1790 to 0.1090	No
3515	2:**50 X** vs. 11:**1 X**	-0.03667	-0.1806 to 0.1073	No
3516	2:**50 X** vs. 11:**10 X**	-0.0370	-0.1810 to 0.1070	No
3517	2:**50 X** vs. 11:**40 X**	-0.02867	-0.1726 to 0.1153	No
3518	2:**50 X** vs. 11:**50 X**	-0.01667	-0.1606 to 0.1273	No
3519	2:**50 X** vs. 12:**0 X**	-0.03567	-0.1796 to 0.1083	No
3520	2:**50 X** vs. 12:**1 X**	-0.0180	-0.1620 to 0.1260	No
3521	2:**50 X** vs. 12:**10 X**	-0.03567	-0.1796 to 0.1083	No
3522	2:**50 X** vs. 12:**40 X**	-0.0220	-0.1660 to 0.1220	No
3523	2:**50 X** vs. 12:**50 X**	-0.02533	-0.1693 to 0.1186	No
3524	2:**50 X** vs. 13:**0 X**	-0.02967	-0.1736 to 0.1143	No
3525	2:**50 X** vs. 13:**1 X**	-0.0170	-0.1610 to 0.1270	No
3526	2:**50 X** vs. 13:**10 X**	-0.0390	-0.1830 to 0.1050	No
3527	2:**50 X** vs. 13:**40 X**	-0.03533	-0.1793 to 0.1086	No
3528	2:**50 X** vs. 13:**50 X**	-0.03633	-0.1803 to 0.1076	No
3529	2:**50 X** vs. 14:**0 X**	-0.03833	-0.1823 to 0.1056	No
3530	2:**50 X** vs. 14:**1 X**	-0.0300	-0.1740 to 0.1140	No
3531	2:**50 X** vs. 14:**10 X**	-0.0440	-0.1880 to 0.09996	No
3532	2:**50 X** vs. 14:**40 X**	-0.04067	-0.1846 to 0.1033	No
3533	2:**50 X** vs. 14:**50 X**	-0.04467	-0.1886 to 0.09929	No
3534	2:**50 X** vs. 15:**0 X**	-0.05067	-0.1946 to 0.09329	No
3535	2:**50 X** vs. 15:**1 X**	-0.03067	-0.1746 to 0.1133	No
3536	2:**50 X** vs. 15:**10 X**	-0.0580	-0.2020 to 0.08596	No
3537	2:**50 X** vs. 15:**40 X**	-0.04033	-0.1843 to 0.1036	No
3538	2:**50 X** vs. 15:**50 X**	-0.0580	-0.2020 to 0.08596	No
3539	2:**50 X** vs. 16:**0 X**	-0.1430	-0.2870 to 0.0009598	No
3540	2:**50 X** vs. 16:**1 X**	-0.0340	-0.1780 to 0.1100	No
3541	2:**50 X** vs. 16:**10 X**	-0.05967	-0.2036 to 0.08429	No
3542	2:**50 X** vs. 16:**40 X**	-0.0790	-0.2230 to 0.06496	No
3543	2:**50 X** vs. 16:**50 X**	-0.1213	-0.2653 to 0.02263	No
3544	2:**50 X** vs. 17:**0 X**	-0.1957	-0.3396 to -0.05171	Yes
3545	2:**50 X** vs. 17:**1 X**	-0.1100	-0.2540 to 0.03396	No
3546	2:**50 X** vs. 17:**10 X**	-0.1667	-0.3106 to -0.02271	Yes
3547	2:**50 X** vs. 17:**40 X**	-0.0900	-0.2340 to 0.05396	No
3548	2:**50 X** vs. 17:**50 X**	-0.1353	-0.2793 to 0.008626	No
3549	2:**50 X** vs. 18:**0 X**	-0.2443	-0.3883 to -0.1004	Yes
3550	2:**50 X** vs. 18:**1 X**	-0.1527	-0.2966 to -0.008707	Yes
3551	2:**50 X** vs. 18:**10 X**	-0.2450	-0.3890 to -0.1010	Yes
3552	2:**50 X** vs. 18:**40 X**	-0.09433	-0.2383 to 0.04963	No
3553	2:**50 X** vs. 18:**50 X**	-0.1393	-0.2833 to 0.004626	No
3554	2:**50 X** vs. 19:**0 X**	-0.2793	-0.4233 to -0.1354	Yes
3555	2:**50 X** vs. 19:**1 X**	-0.2673	-0.4113 to -0.1234	Yes

	2way ANOVA<br>Multiple comparisons	A<br>Data Set-A<br>Y	B<br>Data Set-B<br>Y	C<br>Data Set-C<br>Y
3556	2:**50 X** vs. 19:**10 X**	-0.2650	-0.4090 to -0.1210	Yes
3557	2:**50 X** vs. 19:**40 X**	-0.1230	-0.2670 to 0.02096	No
3558	2:**50 X** vs. 19:**50 X**	-0.1340	-0.2780 to 0.009960	No
3559	2:**50 X** vs. 20:**0 X**	-0.3590	-0.5030 to -0.2150	Yes
3560	2:**50 X** vs. 20:**1 X**	-0.1783	-0.3223 to -0.03437	Yes
3561	2:**50 X** vs. 20:**10 X**	-0.2673	-0.4113 to -0.1234	Yes
3562	2:**50 X** vs. 20:**40 X**	-0.1347	-0.2786 to 0.009293	No
3563	2:**50 X** vs. 20:**50 X**	-0.1787	-0.3226 to -0.03471	Yes
3564	2:**50 X** vs. 21:**0 X**	-0.3823	-0.5263 to -0.2384	Yes
3565	2:**50 X** vs. 21:**1 X**	-0.3353	-0.4793 to -0.1914	Yes
3566	2:**50 X** vs. 21:**10 X**	-0.3323	-0.4763 to -0.1884	Yes
3567	2:**50 X** vs. 21:**40 X**	-0.2077	-0.3516 to -0.06371	Yes
3568	2:**50 X** vs. 21:**50 X**	-0.2980	-0.4420 to -0.1540	Yes
3569	2:**50 X** vs. 22:**0 X**	-0.4240	-0.5680 to -0.2800	Yes
3570	2:**50 X** vs. 22:**1 X**	-0.4187	-0.5626 to -0.2747	Yes
3571	2:**50 X** vs. 22:**10 X**	-0.4140	-0.5580 to -0.2700	Yes
3572	2:**50 X** vs. 22:**40 X**	-0.3143	-0.4583 to -0.1704	Yes
3573	2:**50 X** vs. 22:**50 X**	-0.3770	-0.5210 to -0.2330	Yes
3574	2:**50 X** vs. 23:**0 X**	-0.4893	-0.6333 to -0.3454	Yes
3575	2:**50 X** vs. 23:**1 X**	-0.6790	-0.8400 to -0.5180	Yes
3576	2:**50 X** vs. 23:**10 X**	-0.4287	-0.5726 to -0.2847	Yes
3577	2:**50 X** vs. 23:**40 X**	-0.3090	-0.4700 to -0.1480	Yes
3578	2:**50 X** vs. 23:**50 X**	-0.3430	-0.4870 to -0.1990	Yes
3579	2:**50 X** vs. 24:**0 X**	-0.6067	-0.7506 to -0.4627	Yes
3580	2:**50 X** vs. 24:**1 X**	-0.7630	-0.9240 to -0.6020	Yes
3581	2:**50 X** vs. 24:**10 X**	-0.5905	-0.7515 to -0.4295	Yes
3582	2:**50 X** vs. 24:**40 X**	-0.3253	-0.4693 to -0.1814	Yes
3583	2:**50 X** vs. 24:**50 X**	-0.2470	-0.3910 to -0.1030	Yes
3584	2:**50 X** vs. 25:**0 X**	-0.9440	-1.105 to -0.7830	Yes
3585	2:**50 X** vs. 25:**1 X**	-1.134	-1.294 to -0.9725	Yes
3586	2:**50 X** vs. 25:**10 X**	-0.4950	-0.6986 to -0.2914	Yes
3587	2:**50 X** vs. 25:**40 X**	-0.3273	-0.4713 to -0.1834	Yes
3588	2:**50 X** vs. 25:**50 X**	-0.3017	-0.4456 to -0.1577	Yes
3589	2:**50 X** vs. 26:**0 X**	-1.157	-1.317 to -0.9955	Yes
3590	2:**50 X** vs. 26:**1 X**	-1.270	-1.430 to -1.109	Yes
3591	2:**50 X** vs. 26:**10 X**	-0.9560	-1.117 to -0.7950	Yes
3592	2:**50 X** vs. 26:**40 X**	-0.4017	-0.5456 to -0.2577	Yes
3593	2:**50 X** vs. 26:**50 X**	-0.2620	-0.4060 to -0.1180	Yes
3594	2:**50 X** vs. 27:**0 X**	-1.344	-1.488 to -1.200	Yes
3595	2:**50 X** vs. 27:**1 X**	-1.435	-1.596 to -1.274	Yes
3596	2:**50 X** vs. 27:**10 X**	-1.247	-1.408 to -1.086	Yes
3597	2:**50 X** vs. 27:**40 X**	-0.4017	-0.5456 to -0.2577	Yes
3598	2:**50 X** vs. 27:**50 X**	-0.2613	-0.4053 to -0.1174	Yes
3599	2:**50 X** vs. 28:**0 X**	-1.294	-1.454 to -1.133	Yes
3600	2:**50 X** vs. 28:**1 X**	-1.334	-1.478 to -1.190	Yes

	2way ANOVA<br>Multiple comparisons	A<br>Data Set-A<br>Y	B<br>Data Set-B<br>Y	C<br>Data Set-C<br>Y
3601	2:**50 X** vs. 28:**10 X**	-1.136	-1.297 to -0.9750	Yes
3602	2:**50 X** vs. 28:**40 X**	-0.4140	-0.5580 to -0.2700	Yes
3603	2:**50 X** vs. 28:**50 X**	-0.3093	-0.4533 to -0.1654	Yes
3604	2:**50 X** vs. 29:**0 X**	-1.775	-1.936 to -1.614	Yes
3605	2:**50 X** vs. 29:**1 X**	-1.860	-2.021 to -1.699	Yes
3606	2:**50 X** vs. 29:**10 X**	-1.075	-1.235 to -0.9135	Yes
3607	2:**50 X** vs. 29:**40 X**	-0.5220	-0.6660 to -0.3780	Yes
3608	2:**50 X** vs. 29:**50 X**	-0.3107	-0.4546 to -0.1667	Yes
3609	2:**50 X** vs. 30:**0 X**	-1.490	-1.650 to -1.329	Yes
3610	2:**50 X** vs. 30:**1 X**	-1.811	-1.972 to -1.650	Yes
3611	2:**50 X** vs. 30:**10 X**	-1.149	-1.310 to -0.9880	Yes
3612	2:**50 X** vs. 30:**40 X**	-0.4490	-0.5930 to -0.3050	Yes
3613	2:**50 X** vs. 30:**50 X**	-0.3630	-0.5070 to -0.2190	Yes
3614	2:**50 X** vs. 31:**0 X**	-1.808	-1.968 to -1.647	Yes
3615	2:**50 X** vs. 31:**1 X**	-1.798	-1.959 to -1.637	Yes
3616	2:**50 X** vs. 31:**10 X**	-1.281	-1.441 to -1.120	Yes
3617	2:**50 X** vs. 31:**40 X**	-0.5407	-0.6846 to -0.3967	Yes
3618	2:**50 X** vs. 31:**50 X**	-0.3420	-0.4860 to -0.1980	Yes
3619	2:**50 X** vs. 32:**0 X**	-2.399	-2.543 to -2.255	Yes
3620	2:**50 X** vs. 32:**1 X**	-1.855	-2.016 to -1.694	Yes
3621	2:**50 X** vs. 32:**10 X**	-1.711	-1.855 to -1.567	Yes
3622	2:**50 X** vs. 32:**40 X**	-0.5370	-0.6810 to -0.3930	Yes
3623	2:**50 X** vs. 32:**50 X**	-0.3417	-0.4856 to -0.1977	Yes
3624	2:**50 X** vs. 33:**0 X**	-2.403	-2.547 to -2.259	Yes
3625	2:**50 X** vs. 33:**1 X**	-2.217	-2.361 to -2.073	Yes
3626	2:**50 X** vs. 33:**10 X**	-1.878	-2.022 to -1.734	Yes
3627	2:**50 X** vs. 33:**40 X**	-0.5557	-0.6996 to -0.4117	Yes
3628	2:**50 X** vs. 33:**50 X**	-0.3567	-0.5006 to -0.2127	Yes
3629	2:**50 X** vs. 34:**0 X**	-2.327	-2.471 to -2.183	Yes
3630	2:**50 X** vs. 34:**1 X**	-2.335	-2.479 to -2.191	Yes
3631	2:**50 X** vs. 34:**10 X**	-1.968	-2.112 to -1.824	Yes
3632	2:**50 X** vs. 34:**40 X**	-0.5677	-0.7116 to -0.4237	Yes
3633	2:**50 X** vs. 34:**50 X**	-0.5057	-0.6496 to -0.3617	Yes
3634	2:**50 X** vs. 35:**0 X**	-2.676	-2.820 to -2.532	Yes
3635	2:**50 X** vs. 35:**1 X**	-2.442	-2.586 to -2.298	Yes
3636	2:**50 X** vs. 35:**10 X**	-1.930	-2.074 to -1.786	Yes
3637	2:**50 X** vs. 35:**40 X**	-0.5767	-0.7206 to -0.4327	Yes
3638	2:**50 X** vs. 35:**50 X**	-0.5183	-0.6623 to -0.3744	Yes
3639	2:**50 X** vs. 36:**0 X**	-2.883	-3.043 to -2.722	Yes
3640	2:**50 X** vs. 36:**1 X**	-2.578	-2.722 to -2.434	Yes
3641	2:**50 X** vs. 36:**10 X**	-1.935	-2.079 to -1.791	Yes
3642	2:**50 X** vs. 36:**40 X**	-0.6320	-0.7760 to -0.4880	Yes
3643	2:**50 X** vs. 36:**50 X**	-0.4817	-0.6256 to -0.3377	Yes
3644	2:**50 X** vs. 37:**0 X**	-3.266	-3.410 to -3.122	Yes
3645	2:**50 X** vs. 37:**1 X**	-2.648	-2.792 to -2.504	Yes

	2way ANOVA<br>Multiple comparisons	A<br>Data Set-A<br>Y	B<br>Data Set-B<br>Y	C<br>Data Set-C<br>Y
3646	2:**50 X** vs. 37:**10 X**	-1.937	-2.081 to -1.793	Yes
3647	2:**50 X** vs. 37:**40 X**	-0.6693	-0.8133 to -0.5254	Yes
3648	2:**50 X** vs. 37:**50 X**	-0.4903	-0.6343 to -0.3464	Yes
3649	2:**50 X** vs. 38:**0 X**	-3.211	-3.355 to -3.067	Yes
3650	2:**50 X** vs. 38:**1 X**	-2.767	-2.911 to -2.623	Yes
3651	2:**50 X** vs. 38:**10 X**	-2.212	-2.356 to -2.068	Yes
3652	2:**50 X** vs. 38:**40 X**	-0.6737	-0.8176 to -0.5297	Yes
3653	2:**50 X** vs. 38:**50 X**	-0.4943	-0.6383 to -0.3504	Yes
3654	2:**50 X** vs. 39:**0 X**	-3.442	-3.586 to -3.298	Yes
3655	2:**50 X** vs. 39:**1 X**	-3.334	-3.478 to -3.190	Yes
3656	2:**50 X** vs. 39:**10 X**	-2.357	-2.501 to -2.213	Yes
3657	2:**50 X** vs. 39:**40 X**	-0.6797	-0.8236 to -0.5357	Yes
3658	2:**50 X** vs. 39:**50 X**	-0.5253	-0.6693 to -0.3814	Yes
3659	2:**50 X** vs. 40:**0 X**	-3.483	-3.627 to -3.339	Yes
3660	2:**50 X** vs. 40:**1 X**	-3.409	-3.553 to -3.265	Yes
3661	2:**50 X** vs. 40:**10 X**	-2.614	-2.758 to -2.470	Yes
3662	2:**50 X** vs. 40:**40 X**	-0.6843	-0.8283 to -0.5404	Yes
3663	2:**50 X** vs. 40:**50 X**	-0.5317	-0.6756 to -0.3877	Yes
3664	2:**50 X** vs. 41:**0 X**	-3.524	-3.668 to -3.380	Yes
3665	2:**50 X** vs. 41:**1 X**	-3.523	-3.667 to -3.379	Yes
3666	2:**50 X** vs. 41:**10 X**	-2.667	-2.811 to -2.523	Yes
3667	2:**50 X** vs. 41:**40 X**	-0.6317	-0.7756 to -0.4877	Yes
3668	2:**50 X** vs. 41:**50 X**	-0.5443	-0.6883 to -0.4004	Yes
3669	2:**50 X** vs. 42:**0 X**	-3.666	-3.810 to -3.522	Yes
3670	2:**50 X** vs. 42:**1 X**	-3.685	-3.829 to -3.541	Yes
3671	2:**50 X** vs. 42:**10 X**	-2.668	-2.812 to -2.524	Yes
3672	2:**50 X** vs. 42:**40 X**	-0.6443	-0.7883 to -0.5004	Yes
3673	2:**50 X** vs. 42:**50 X**	-0.5477	-0.6916 to -0.4037	Yes
3674	2:**50 X** vs. 43:**0 X**	-3.756	-3.900 to -3.612	Yes
3675	2:**50 X** vs. 43:**1 X**	-3.670	-3.814 to -3.526	Yes
3676	2:**50 X** vs. 43:**10 X**	-3.386	-3.530 to -3.242	Yes
3677	2:**50 X** vs. 43:**40 X**	-0.6427	-0.7866 to -0.4987	Yes
3678	2:**50 X** vs. 43:**50 X**	-0.5480	-0.6920 to -0.4040	Yes
3679	2:**50 X** vs. 44:**0 X**	-3.780	-3.924 to -3.636	Yes
3680	2:**50 X** vs. 44:**1 X**	-3.669	-3.813 to -3.525	Yes
3681	2:**50 X** vs. 44:**10 X**	-3.449	-3.593 to -3.305	Yes
3682	2:**50 X** vs. 44:**40 X**	-0.6447	-0.7886 to -0.5007	Yes
3683	2:**50 X** vs. 44:**50 X**	-0.4803	-0.6243 to -0.3364	Yes
3684	2:**50 X** vs. 45:**0 X**	-3.644	-3.788 to -3.500	Yes
3685	2:**50 X** vs. 45:**1 X**	-3.446	-3.590 to -3.302	Yes
3686	2:**50 X** vs. 45:**10 X**	-3.329	-3.473 to -3.185	Yes
3687	2:**50 X** vs. 45:**40 X**	-0.6460	-0.7900 to -0.5020	Yes
3688	2:**50 X** vs. 45:**50 X**	-0.4773	-0.6213 to -0.3334	Yes
3689	2:**50 X** vs. 46:**0 X**	-3.449	-3.593 to -3.305	Yes
3690	2:**50 X** vs. 46:**1 X**	-3.416	-3.560 to -3.272	Yes

2way ANOVA<br>Multiple comparisons	A<br>Data Set-A<br>Y	B<br>Data Set-B<br>Y	C<br>Data Set-C<br>Y
3691 2:50 X vs. 46:10 X	-3.324	-3.468 to -3.180	Yes
3692 2:50 X vs. 46:40 X	-0.6597	-0.8036 to -0.5157	Yes
3693 2:50 X vs. 46:50 X	-0.4703	-0.6143 to -0.3264	Yes
3694 2:50 X vs. 47:0 X	-3.424	-3.568 to -3.280	Yes
3695 2:50 X vs. 47:1 X	-3.328	-3.472 to -3.184	Yes
3696 2:50 X vs. 47:10 X	-3.310	-3.454 to -3.166	Yes
3697 2:50 X vs. 47:40 X	-0.6697	-0.8136 to -0.5257	Yes
3698 2:50 X vs. 47:50 X	-0.4283	-0.5723 to -0.2844	Yes
3699 2:50 X vs. 48:0 X	-3.412	-3.572 to -3.251	Yes
3700 2:50 X vs. 48:1 X	-3.315	-3.459 to -3.171	Yes
3701 2:50 X vs. 48:10 X	-3.310	-3.454 to -3.166	Yes
3702 2:50 X vs. 48:40 X	-0.6760	-0.8200 to -0.5320	Yes
3703 2:50 X vs. 48:50 X	-0.4357	-0.5796 to -0.2917	Yes
3704 2:50 X vs. 49:0 X	-3.412	-3.556 to -3.268	Yes
3705 2:50 X vs. 49:1 X	-3.315	-3.459 to -3.171	Yes
3706 2:50 X vs. 49:10 X	-3.310	-3.454 to -3.166	Yes
3707 2:50 X vs. 49:40 X	-0.6803	-0.8243 to -0.5364	Yes
3708 2:50 X vs. 49:50 X	-0.4350	-0.5790 to -0.2910	Yes
3709 2:50 X vs. 50:0 X	-3.412	-3.556 to -3.268	Yes
3710 2:50 X vs. 50:1 X	-3.315	-3.459 to -3.171	Yes
3711 2:50 X vs. 50:10 X	-3.310	-3.454 to -3.166	Yes
3712 2:50 X vs. 50:40 X	-0.6880	-0.8320 to -0.5440	Yes
3713 2:50 X vs. 50:50 X	-0.4347	-0.5786 to -0.2907	Yes
3714 3:0 X vs. 3:1 X	-0.002333	-0.1463 to 0.1416	No
3715 3:0 X vs. 3:10 X	0.002333	-0.1416 to 0.1463	No
3716 3:0 X vs. 3:40 X	0.004667	-0.1393 to 0.1486	No
3717 3:0 X vs. 3:50 X	0.005667	-0.1383 to 0.1496	No
3718 3:0 X vs. 4:0 X	-0.004333	-0.1483 to 0.1396	No
3719 3:0 X vs. 4:1 X	-0.0030	-0.1470 to 0.1410	No
3720 3:0 X vs. 4:10 X	-0.01767	-0.1616 to 0.1263	No
3721 3:0 X vs. 4:40 X	0.002667	-0.1413 to 0.1466	No
3722 3:0 X vs. 4:50 X	0.004667	-0.1393 to 0.1486	No
3723 3:0 X vs. 5:0 X	-0.005333	-0.1493 to 0.1386	No
3724 3:0 X vs. 5:1 X	-0.003333	-0.1473 to 0.1406	No
3725 3:0 X vs. 5:10 X	-0.01833	-0.1623 to 0.1256	No
3726 3:0 X vs. 5:40 X	0.002667	-0.1413 to 0.1466	No
3727 3:0 X vs. 5:50 X	0.003667	-0.1403 to 0.1476	No
3728 3:0 X vs. 6:0 X	-0.004667	-0.1486 to 0.1393	No
3729 3:0 X vs. 6:1 X	-0.003667	-0.1476 to 0.1403	No
3730 3:0 X vs. 6:10 X	-0.01833	-0.1623 to 0.1256	No
3731 3:0 X vs. 6:40 X	0.0003333	-0.1436 to 0.1443	No
3732 3:0 X vs. 6:50 X	0.0020	-0.1420 to 0.1460	No
3733 3:0 X vs. 7:0 X	-0.006667	-0.1506 to 0.1373	No
3734 3:0 X vs. 7:1 X	0.0020	-0.1420 to 0.1460	No
3735 3:0 X vs. 7:10 X	-0.0100	-0.1540 to 0.1340	No

	2way ANOVA<br>Multiple comparisons	A<br>Data Set-A<br>Y	B<br>Data Set-B<br>Y	C<br>Data Set-C<br>Y
3736	3:**0 X** vs. 7:**40 X**	-0.001333	-0.1453 to 0.1426	No
3737	3:**0 X** vs. 7:**50 X**	0.0006667	-0.1433 to 0.1446	No
3738	3:**0 X** vs. 8:**0 X**	-0.01133	-0.1553 to 0.1326	No
3739	3:**0 X** vs. 8:**1 X**	0.0020	-0.1420 to 0.1460	No
3740	3:**0 X** vs. 8:**10 X**	-0.005333	-0.1493 to 0.1386	No
3741	3:**0 X** vs. 8:**40 X**	-0.0060	-0.1500 to 0.1380	No
3742	3:**0 X** vs. 8:**50 X**	-0.003000	-0.1470 to 0.1410	No
3743	3:**0 X** vs. 9:**0 X**	-0.01933	-0.1633 to 0.1246	No
3744	3:**0 X** vs. 9:**1 X**	-0.02733	-0.1713 to 0.1166	No
3745	3:**0 X** vs. 9:**10 X**	-0.02833	-0.1723 to 0.1156	No
3746	3:**0 X** vs. 9:**40 X**	-0.01067	-0.1546 to 0.1333	No
3747	3:**0 X** vs. 9:**50 X**	-0.004000	-0.1480 to 0.1400	No
3748	3:**0 X** vs. 10:**0 X**	-0.0210	-0.1650 to 0.1230	No
3749	3:**0 X** vs. 10:**1 X**	-0.0280	-0.1720 to 0.1160	No
3750	3:**0 X** vs. 10:**10 X**	-0.03067	-0.1746 to 0.1133	No
3751	3:**0 X** vs. 10:**40 X**	-0.02033	-0.1643 to 0.1236	No
3752	3:**0 X** vs. 10:**50 X**	-0.0070	-0.1510 to 0.1370	No
3753	3:**0 X** vs. 11:**0 X**	-0.03067	-0.1746 to 0.1133	No
3754	3:**0 X** vs. 11:**1 X**	-0.03233	-0.1763 to 0.1116	No
3755	3:**0 X** vs. 11:**10 X**	-0.03267	-0.1766 to 0.1113	No
3756	3:**0 X** vs. 11:**40 X**	-0.02433	-0.1683 to 0.1196	No
3757	3:**0 X** vs. 11:**50 X**	-0.01233	-0.1563 to 0.1316	No
3758	3:**0 X** vs. 12:**0 X**	-0.03133	-0.1753 to 0.1126	No
3759	3:**0 X** vs. 12:**1 X**	-0.01367	-0.1576 to 0.1303	No
3760	3:**0 X** vs. 12:**10 X**	-0.03133	-0.1753 to 0.1126	No
3761	3:**0 X** vs. 12:**40 X**	-0.01767	-0.1616 to 0.1263	No
3762	3:**0 X** vs. 12:**50 X**	-0.0210	-0.1650 to 0.1230	No
3763	3:**0 X** vs. 13:**0 X**	-0.02533	-0.1693 to 0.1186	No
3764	3:**0 X** vs. 13:**1 X**	-0.01267	-0.1566 to 0.1313	No
3765	3:**0 X** vs. 13:**10 X**	-0.03467	-0.1786 to 0.1093	No
3766	3:**0 X** vs. 13:**40 X**	-0.0310	-0.1750 to 0.1130	No
3767	3:**0 X** vs. 13:**50 X**	-0.0320	-0.1760 to 0.1120	No
3768	3:**0 X** vs. 14:**0 X**	-0.0340	-0.1780 to 0.1100	No
3769	3:**0 X** vs. 14:**1 X**	-0.02567	-0.1696 to 0.1183	No
3770	3:**0 X** vs. 14:**10 X**	-0.03967	-0.1836 to 0.1043	No
3771	3:**0 X** vs. 14:**40 X**	-0.03633	-0.1803 to 0.1076	No
3772	3:**0 X** vs. 14:**50 X**	-0.04033	-0.1843 to 0.1036	No
3773	3:**0 X** vs. 15:**0 X**	-0.04633	-0.1903 to 0.09763	No
3774	3:**0 X** vs. 15:**1 X**	-0.02633	-0.1703 to 0.1176	No
3775	3:**0 X** vs. 15:**10 X**	-0.05367	-0.1976 to 0.09029	No
3776	3:**0 X** vs. 15:**40 X**	-0.0360	-0.1800 to 0.1080	No
3777	3:**0 X** vs. 15:**50 X**	-0.05367	-0.1976 to 0.09029	No
3778	3:**0 X** vs. 16:**0 X**	-0.1387	-0.2826 to 0.005293	No
3779	3:**0 X** vs. 16:**1 X**	-0.02967	-0.1736 to 0.1143	No
3780	3:**0 X** vs. 16:**10 X**	-0.05533	-0.1993 to 0.08863	No

	2way ANOVA<br>Multiple comparisons	A<br>Data Set-A<br>Y	B<br>Data Set-B<br>Y	C<br>Data Set-C<br>Y
3781	3:0 X vs. 16:40 X	-0.07467	-0.2186 to 0.06929	No
3782	3:0 X vs. 16:50 X	-0.1170	-0.2610 to 0.02696	No
3783	3:0 X vs. 17:0 X	-0.1913	-0.3353 to -0.04737	Yes
3784	3:0 X vs. 17:1 X	-0.1057	-0.2496 to 0.03829	No
3785	3:0 X vs. 17:10 X	-0.1623	-0.3063 to -0.01837	Yes
3786	3:0 X vs. 17:40 X	-0.08567	-0.2296 to 0.05829	No
3787	3:0 X vs. 17:50 X	-0.1310	-0.2750 to 0.01296	No
3788	3:0 X vs. 18:0 X	-0.2400	-0.3840 to -0.09604	Yes
3789	3:0 X vs. 18:1 X	-0.1483	-0.2923 to -0.004374	Yes
3790	3:0 X vs. 18:10 X	-0.2407	-0.3846 to -0.09671	Yes
3791	3:0 X vs. 18:40 X	-0.0900	-0.2340 to 0.05396	No
3792	3:0 X vs. 18:50 X	-0.1350	-0.2790 to 0.008960	No
3793	3:0 X vs. 19:0 X	-0.2750	-0.4190 to -0.1310	Yes
3794	3:0 X vs. 19:1 X	-0.2630	-0.4070 to -0.1190	Yes
3795	3:0 X vs. 19:10 X	-0.2607	-0.4046 to -0.1167	Yes
3796	3:0 X vs. 19:40 X	-0.1187	-0.2626 to 0.02529	No
3797	3:0 X vs. 19:50 X	-0.1297	-0.2736 to 0.01429	No
3798	3:0 X vs. 20:0 X	-0.3547	-0.4986 to -0.2107	Yes
3799	3:0 X vs. 20:1 X	-0.1740	-0.3180 to -0.03004	Yes
3800	3:0 X vs. 20:10 X	-0.2630	-0.4070 to -0.1190	Yes
3801	3:0 X vs. 20:40 X	-0.1303	-0.2743 to 0.01363	No
3802	3:0 X vs. 20:50 X	-0.1743	-0.3183 to -0.03037	Yes
3803	3:0 X vs. 21:0 X	-0.3780	-0.5220 to -0.2340	Yes
3804	3:0 X vs. 21:1 X	-0.3310	-0.4750 to -0.1870	Yes
3805	3:0 X vs. 21:10 X	-0.3280	-0.4720 to -0.1840	Yes
3806	3:0 X vs. 21:40 X	-0.2033	-0.3473 to -0.05937	Yes
3807	3:0 X vs. 21:50 X	-0.2937	-0.4376 to -0.1497	Yes
3808	3:0 X vs. 22:0 X	-0.4197	-0.5636 to -0.2757	Yes
3809	3:0 X vs. 22:1 X	-0.4143	-0.5583 to -0.2704	Yes
3810	3:0 X vs. 22:10 X	-0.4097	-0.5536 to -0.2657	Yes
3811	3:0 X vs. 22:40 X	-0.3100	-0.4540 to -0.1660	Yes
3812	3:0 X vs. 22:50 X	-0.3727	-0.5166 to -0.2287	Yes
3813	3:0 X vs. 23:0 X	-0.4850	-0.6290 to -0.3410	Yes
3814	3:0 X vs. 23:1 X	-0.6747	-0.8356 to -0.5137	Yes
3815	3:0 X vs. 23:10 X	-0.4243	-0.5683 to -0.2804	Yes
3816	3:0 X vs. 23:40 X	-0.3047	-0.4656 to -0.1437	Yes
3817	3:0 X vs. 23:50 X	-0.3387	-0.4826 to -0.1947	Yes
3818	3:0 X vs. 24:0 X	-0.6023	-0.7463 to -0.4584	Yes
3819	3:0 X vs. 24:1 X	-0.7587	-0.9196 to -0.5977	Yes
3820	3:0 X vs. 24:10 X	-0.5862	-0.7471 to -0.4252	Yes
3821	3:0 X vs. 24:40 X	-0.3210	-0.4650 to -0.1770	Yes
3822	3:0 X vs. 24:50 X	-0.2427	-0.3866 to -0.09871	Yes
3823	3:0 X vs. 25:0 X	-0.9397	-1.101 to -0.7787	Yes
3824	3:0 X vs. 25:1 X	-1.129	-1.290 to -0.9682	Yes
3825	3:0 X vs. 25:10 X	-0.4907	-0.6943 to -0.2871	Yes

	2way ANOVA<br>Multiple comparisons	A<br>Data Set-A<br>Y	B<br>Data Set-B<br>Y	C<br>Data Set-C<br>Y
3826	3:0 X vs. 25:40 X	-0.3230	-0.4670 to -0.1790	Yes
3827	3:0 X vs. 25:50 X	-0.2973	-0.4413 to -0.1534	Yes
3828	3:0 X vs. 26:0 X	-1.152	-1.313 to -0.9912	Yes
3829	3:0 X vs. 26:1 X	-1.265	-1.426 to -1.104	Yes
3830	3:0 X vs. 26:10 X	-0.9517	-1.113 to -0.7907	Yes
3831	3:0 X vs. 26:40 X	-0.3973	-0.5413 to -0.2534	Yes
3832	3:0 X vs. 26:50 X	-0.2577	-0.4016 to -0.1137	Yes
3833	3:0 X vs. 27:0 X	-1.340	-1.484 to -1.196	Yes
3834	3:0 X vs. 27:1 X	-1.431	-1.592 to -1.270	Yes
3835	3:0 X vs. 27:10 X	-1.243	-1.404 to -1.082	Yes
3836	3:0 X vs. 27:40 X	-0.3973	-0.5413 to -0.2534	Yes
3837	3:0 X vs. 27:50 X	-0.2570	-0.4010 to -0.1130	Yes
3838	3:0 X vs. 28:0 X	-1.289	-1.450 to -1.128	Yes
3839	3:0 X vs. 28:1 X	-1.330	-1.474 to -1.186	Yes
3840	3:0 X vs. 28:10 X	-1.132	-1.293 to -0.9707	Yes
3841	3:0 X vs. 28:40 X	-0.4097	-0.5536 to -0.2657	Yes
3842	3:0 X vs. 28:50 X	-0.3050	-0.4490 to -0.1610	Yes
3843	3:0 X vs. 29:0 X	-1.771	-1.932 to -1.610	Yes
3844	3:0 X vs. 29:1 X	-1.856	-2.017 to -1.695	Yes
3845	3:0 X vs. 29:10 X	-1.070	-1.231 to -0.9092	Yes
3846	3:0 X vs. 29:40 X	-0.5177	-0.6616 to -0.3737	Yes
3847	3:0 X vs. 29:50 X	-0.3063	-0.4503 to -0.1624	Yes
3848	3:0 X vs. 30:0 X	-1.485	-1.646 to -1.324	Yes
3849	3:0 X vs. 30:1 X	-1.807	-1.968 to -1.646	Yes
3850	3:0 X vs. 30:10 X	-1.145	-1.306 to -0.9837	Yes
3851	3:0 X vs. 30:40 X	-0.4447	-0.5886 to -0.3007	Yes
3852	3:0 X vs. 30:50 X	-0.3587	-0.5026 to -0.2147	Yes
3853	3:0 X vs. 31:0 X	-1.803	-1.964 to -1.642	Yes
3854	3:0 X vs. 31:1 X	-1.794	-1.955 to -1.633	Yes
3855	3:0 X vs. 31:10 X	-1.276	-1.437 to -1.115	Yes
3856	3:0 X vs. 31:40 X	-0.5363	-0.6803 to -0.3924	Yes
3857	3:0 X vs. 31:50 X	-0.3377	-0.4816 to -0.1937	Yes
3858	3:0 X vs. 32:0 X	-2.395	-2.539 to -2.251	Yes
3859	3:0 X vs. 32:1 X	-1.851	-2.012 to -1.690	Yes
3860	3:0 X vs. 32:10 X	-1.707	-1.851 to -1.563	Yes
3861	3:0 X vs. 32:40 X	-0.5327	-0.6766 to -0.3887	Yes
3862	3:0 X vs. 32:50 X	-0.3373	-0.4813 to -0.1934	Yes
3863	3:0 X vs. 33:0 X	-2.398	-2.542 to -2.254	Yes
3864	3:0 X vs. 33:1 X	-2.213	-2.357 to -2.069	Yes
3865	3:0 X vs. 33:10 X	-1.873	-2.017 to -1.729	Yes
3866	3:0 X vs. 33:40 X	-0.5513	-0.6953 to -0.4074	Yes
3867	3:0 X vs. 33:50 X	-0.3523	-0.4963 to -0.2084	Yes
3868	3:0 X vs. 34:0 X	-2.323	-2.467 to -2.179	Yes
3869	3:0 X vs. 34:1 X	-2.330	-2.474 to -2.186	Yes
3870	3:0 X vs. 34:10 X	-1.964	-2.108 to -1.820	Yes

2way ANOVA<br>Multiple comparisons		A<br>Data Set-A<br>Y	B<br>Data Set-B<br>Y	C<br>Data Set-C<br>Y
3871	3:**0 X** vs. 34:**40 X**	-0.5633	-0.7073 to -0.4194	Yes
3872	3:**0 X** vs. 34:**50 X**	-0.5013	-0.6453 to -0.3574	Yes
3873	3:**0 X** vs. 35:**0 X**	-2.672	-2.816 to -2.528	Yes
3874	3:**0 X** vs. 35:**1 X**	-2.437	-2.581 to -2.293	Yes
3875	3:**0 X** vs. 35:**10 X**	-1.925	-2.069 to -1.781	Yes
3876	3:**0 X** vs. 35:**40 X**	-0.5723	-0.7163 to -0.4284	Yes
3877	3:**0 X** vs. 35:**50 X**	-0.5140	-0.6580 to -0.3700	Yes
3878	3:**0 X** vs. 36:**0 X**	-2.878	-3.039 to -2.717	Yes
3879	3:**0 X** vs. 36:**1 X**	-2.574	-2.718 to -2.430	Yes
3880	3:**0 X** vs. 36:**10 X**	-1.931	-2.075 to -1.787	Yes
3881	3:**0 X** vs. 36:**40 X**	-0.6277	-0.7716 to -0.4837	Yes
3882	3:**0 X** vs. 36:**50 X**	-0.4773	-0.6213 to -0.3334	Yes
3883	3:**0 X** vs. 37:**0 X**	-3.262	-3.406 to -3.118	Yes
3884	3:**0 X** vs. 37:**1 X**	-2.644	-2.788 to -2.500	Yes
3885	3:**0 X** vs. 37:**10 X**	-1.933	-2.077 to -1.789	Yes
3886	3:**0 X** vs. 37:**40 X**	-0.6650	-0.8090 to -0.5210	Yes
3887	3:**0 X** vs. 37:**50 X**	-0.4860	-0.6300 to -0.3420	Yes
3888	3:**0 X** vs. 38:**0 X**	-3.207	-3.351 to -3.063	Yes
3889	3:**0 X** vs. 38:**1 X**	-2.763	-2.907 to -2.619	Yes
3890	3:**0 X** vs. 38:**10 X**	-2.208	-2.352 to -2.064	Yes
3891	3:**0 X** vs. 38:**40 X**	-0.6693	-0.8133 to -0.5254	Yes
3892	3:**0 X** vs. 38:**50 X**	-0.4900	-0.6340 to -0.3460	Yes
3893	3:**0 X** vs. 39:**0 X**	-3.438	-3.582 to -3.294	Yes
3894	3:**0 X** vs. 39:**1 X**	-3.329	-3.473 to -3.185	Yes
3895	3:**0 X** vs. 39:**10 X**	-2.353	-2.497 to -2.209	Yes
3896	3:**0 X** vs. 39:**40 X**	-0.6753	-0.8193 to -0.5314	Yes
3897	3:**0 X** vs. 39:**50 X**	-0.5210	-0.6650 to -0.3770	Yes
3898	3:**0 X** vs. 40:**0 X**	-3.479	-3.623 to -3.335	Yes
3899	3:**0 X** vs. 40:**1 X**	-3.405	-3.549 to -3.261	Yes
3900	3:**0 X** vs. 40:**10 X**	-2.609	-2.753 to -2.465	Yes
3901	3:**0 X** vs. 40:**40 X**	-0.6800	-0.8240 to -0.5360	Yes
3902	3:**0 X** vs. 40:**50 X**	-0.5273	-0.6713 to -0.3834	Yes
3903	3:**0 X** vs. 41:**0 X**	-3.520	-3.664 to -3.376	Yes
3904	3:**0 X** vs. 41:**1 X**	-3.519	-3.663 to -3.375	Yes
3905	3:**0 X** vs. 41:**10 X**	-2.663	-2.807 to -2.519	Yes
3906	3:**0 X** vs. 41:**40 X**	-0.6273	-0.7713 to -0.4834	Yes
3907	3:**0 X** vs. 41:**50 X**	-0.5400	-0.6840 to -0.3960	Yes
3908	3:**0 X** vs. 42:**0 X**	-3.661	-3.805 to -3.517	Yes
3909	3:**0 X** vs. 42:**1 X**	-3.681	-3.825 to -3.537	Yes
3910	3:**0 X** vs. 42:**10 X**	-2.663	-2.807 to -2.519	Yes
3911	3:**0 X** vs. 42:**40 X**	-0.6400	-0.7840 to -0.4960	Yes
3912	3:**0 X** vs. 42:**50 X**	-0.5433	-0.6873 to -0.3994	Yes
3913	3:**0 X** vs. 43:**0 X**	-3.751	-3.895 to -3.607	Yes
3914	3:**0 X** vs. 43:**1 X**	-3.666	-3.810 to -3.522	Yes
3915	3:**0 X** vs. 43:**10 X**	-3.381	-3.525 to -3.237	Yes

	2way ANOVA<br>Multiple comparisons	A<br>Data Set-A<br>Y	B<br>Data Set-B<br>Y	C<br>Data Set-C<br>Y
3916	3:0 X vs. 43:40 X	-0.6383	-0.7823 to -0.4944	Yes
3917	3:0 X vs. 43:50 X	-0.5437	-0.6876 to -0.3997	Yes
3918	3:0 X vs. 44:0 X	-3.775	-3.919 to -3.631	Yes
3919	3:0 X vs. 44:1 X	-3.665	-3.809 to -3.521	Yes
3920	3:0 X vs. 44:10 X	-3.445	-3.589 to -3.301	Yes
3921	3:0 X vs. 44:40 X	-0.6403	-0.7843 to -0.4964	Yes
3922	3:0 X vs. 44:50 X	-0.4760	-0.6200 to -0.3320	Yes
3923	3:0 X vs. 45:0 X	-3.640	-3.784 to -3.496	Yes
3924	3:0 X vs. 45:1 X	-3.442	-3.586 to -3.298	Yes
3925	3:0 X vs. 45:10 X	-3.324	-3.468 to -3.180	Yes
3926	3:0 X vs. 45:40 X	-0.6417	-0.7856 to -0.4977	Yes
3927	3:0 X vs. 45:50 X	-0.4730	-0.6170 to -0.3290	Yes
3928	3:0 X vs. 46:0 X	-3.444	-3.588 to -3.300	Yes
3929	3:0 X vs. 46:1 X	-3.411	-3.555 to -3.267	Yes
3930	3:0 X vs. 46:10 X	-3.319	-3.463 to -3.175	Yes
3931	3:0 X vs. 46:40 X	-0.6553	-0.7993 to -0.5114	Yes
3932	3:0 X vs. 46:50 X	-0.4660	-0.6100 to -0.3220	Yes
3933	3:0 X vs. 47:0 X	-3.420	-3.564 to -3.276	Yes
3934	3:0 X vs. 47:1 X	-3.324	-3.468 to -3.180	Yes
3935	3:0 X vs. 47:10 X	-3.305	-3.449 to -3.161	Yes
3936	3:0 X vs. 47:40 X	-0.6653	-0.8093 to -0.5214	Yes
3937	3:0 X vs. 47:50 X	-0.4240	-0.5680 to -0.2800	Yes
3938	3:0 X vs. 48:0 X	-3.407	-3.568 to -3.246	Yes
3939	3:0 X vs. 48:1 X	-3.311	-3.455 to -3.167	Yes
3940	3:0 X vs. 48:10 X	-3.305	-3.449 to -3.161	Yes
3941	3:0 X vs. 48:40 X	-0.6717	-0.8156 to -0.5277	Yes
3942	3:0 X vs. 48:50 X	-0.4313	-0.5753 to -0.2874	Yes
3943	3:0 X vs. 49:0 X	-3.407	-3.551 to -3.263	Yes
3944	3:0 X vs. 49:1 X	-3.311	-3.455 to -3.167	Yes
3945	3:0 X vs. 49:10 X	-3.305	-3.449 to -3.161	Yes
3946	3:0 X vs. 49:40 X	-0.6760	-0.8200 to -0.5320	Yes
3947	3:0 X vs. 49:50 X	-0.4307	-0.5746 to -0.2867	Yes
3948	3:0 X vs. 50:0 X	-3.407	-3.551 to -3.263	Yes
3949	3:0 X vs. 50:1 X	-3.311	-3.455 to -3.167	Yes
3950	3:0 X vs. 50:10 X	-3.305	-3.449 to -3.161	Yes
3951	3:0 X vs. 50:40 X	-0.6837	-0.8276 to -0.5397	Yes
3952	3:0 X vs. 50:50 X	-0.4303	-0.5743 to -0.2864	Yes
3953	3:1 X vs. 3:10 X	0.004667	-0.1393 to 0.1486	No
3954	3:1 X vs. 3:40 X	0.007000	-0.1370 to 0.1510	No
3955	3:1 X vs. 3:50 X	0.008000	-0.1360 to 0.1520	No
3956	3:1 X vs. 4:0 X	-0.0020	-0.1460 to 0.1420	No
3957	3:1 X vs. 4:1 X	-0.0006667	-0.1446 to 0.1433	No
3958	3:1 X vs. 4:10 X	-0.01533	-0.1593 to 0.1286	No
3959	3:1 X vs. 4:40 X	0.005000	-0.1390 to 0.1490	No
3960	3:1 X vs. 4:50 X	0.007000	-0.1370 to 0.1510	No

	2way ANOVA<br>Multiple comparisons	A<br>Data Set-A<br>Y	B<br>Data Set-B<br>Y	C<br>Data Set-C<br>Y
**3961**	3:**1 X** vs. 5:**0 X**	-0.0030	-0.1470 to 0.1410	No
**3962**	3:**1 X** vs. 5:**1 X**	-0.0010	-0.1450 to 0.1430	No
**3963**	3:**1 X** vs. 5:**10 X**	-0.0160	-0.1600 to 0.1280	No
**3964**	3:**1 X** vs. 5:**40 X**	0.005000	-0.1390 to 0.1490	No
**3965**	3:**1 X** vs. 5:**50 X**	0.0060	-0.1380 to 0.1500	No
**3966**	3:**1 X** vs. 6:**0 X**	-0.002333	-0.1463 to 0.1416	No
**3967**	3:**1 X** vs. 6:**1 X**	-0.001333	-0.1453 to 0.1426	No
**3968**	3:**1 X** vs. 6:**10 X**	-0.0160	-0.1600 to 0.1280	No
**3969**	3:**1 X** vs. 6:**40 X**	0.002667	-0.1413 to 0.1466	No
**3970**	3:**1 X** vs. 6:**50 X**	0.004333	-0.1396 to 0.1483	No
**3971**	3:**1 X** vs. 7:**0 X**	-0.004333	-0.1483 to 0.1396	No
**3972**	3:**1 X** vs. 7:**1 X**	0.004333	-0.1396 to 0.1483	No
**3973**	3:**1 X** vs. 7:**10 X**	-0.007667	-0.1516 to 0.1363	No
**3974**	3:**1 X** vs. 7:**40 X**	0.001000	-0.1430 to 0.1450	No
**3975**	3:**1 X** vs. 7:**50 X**	0.003000	-0.1410 to 0.1470	No
**3976**	3:**1 X** vs. 8:**0 X**	-0.009000	-0.1530 to 0.1350	No
**3977**	3:**1 X** vs. 8:**1 X**	0.004333	-0.1396 to 0.1483	No
**3978**	3:**1 X** vs. 8:**10 X**	-0.0030	-0.1470 to 0.1410	No
**3979**	3:**1 X** vs. 8:**40 X**	-0.003667	-0.1476 to 0.1403	No
**3980**	3:**1 X** vs. 8:**50 X**	-0.0006667	-0.1446 to 0.1433	No
**3981**	3:**1 X** vs. 9:**0 X**	-0.0170	-0.1610 to 0.1270	No
**3982**	3:**1 X** vs. 9:**1 X**	-0.0250	-0.1690 to 0.1190	No
**3983**	3:**1 X** vs. 9:**10 X**	-0.0260	-0.1700 to 0.1180	No
**3984**	3:**1 X** vs. 9:**40 X**	-0.008333	-0.1523 to 0.1356	No
**3985**	3:**1 X** vs. 9:**50 X**	-0.001667	-0.1456 to 0.1423	No
**3986**	3:**1 X** vs. 10:**0 X**	-0.01867	-0.1626 to 0.1253	No
**3987**	3:**1 X** vs. 10:**1 X**	-0.02567	-0.1696 to 0.1183	No
**3988**	3:**1 X** vs. 10:**10 X**	-0.02833	-0.1723 to 0.1156	No
**3989**	3:**1 X** vs. 10:**40 X**	-0.0180	-0.1620 to 0.1260	No
**3990**	3:**1 X** vs. 10:**50 X**	-0.004667	-0.1486 to 0.1393	No
**3991**	3:**1 X** vs. 11:**0 X**	-0.02833	-0.1723 to 0.1156	No
**3992**	3:**1 X** vs. 11:**1 X**	-0.0300	-0.1740 to 0.1140	No
**3993**	3:**1 X** vs. 11:**10 X**	-0.03033	-0.1743 to 0.1136	No
**3994**	3:**1 X** vs. 11:**40 X**	-0.0220	-0.1660 to 0.1220	No
**3995**	3:**1 X** vs. 11:**50 X**	-0.0100	-0.1540 to 0.1340	No
**3996**	3:**1 X** vs. 12:**0 X**	-0.0290	-0.1730 to 0.1150	No
**3997**	3:**1 X** vs. 12:**1 X**	-0.01133	-0.1553 to 0.1326	No
**3998**	3:**1 X** vs. 12:**10 X**	-0.0290	-0.1730 to 0.1150	No
**3999**	3:**1 X** vs. 12:**40 X**	-0.01533	-0.1593 to 0.1286	No
**4000**	3:**1 X** vs. 12:**50 X**	-0.01867	-0.1626 to 0.1253	No
**4001**	3:**1 X** vs. 13:**0 X**	-0.0230	-0.1670 to 0.1210	No
**4002**	3:**1 X** vs. 13:**1 X**	-0.01033	-0.1543 to 0.1336	No
**4003**	3:**1 X** vs. 13:**10 X**	-0.03233	-0.1763 to 0.1116	No
**4004**	3:**1 X** vs. 13:**40 X**	-0.02867	-0.1726 to 0.1153	No
**4005**	3:**1 X** vs. 13:**50 X**	-0.02967	-0.1736 to 0.1143	No

	2way ANOVA<br>Multiple comparisons	A<br>Data Set-A<br>Y	B<br>Data Set-B<br>Y	C<br>Data Set-C<br>Y
4006	3:1 X vs. 14:0 X	-0.03167	-0.1756 to 0.1123	No
4007	3:1 X vs. 14:1 X	-0.02333	-0.1673 to 0.1206	No
4008	3:1 X vs. 14:10 X	-0.03733	-0.1813 to 0.1066	No
4009	3:1 X vs. 14:40 X	-0.0340	-0.1780 to 0.1100	No
4010	3:1 X vs. 14:50 X	-0.0380	-0.1820 to 0.1060	No
4011	3:1 X vs. 15:0 X	-0.0440	-0.1880 to 0.09996	No
4012	3:1 X vs. 15:1 X	-0.0240	-0.1680 to 0.1200	No
4013	3:1 X vs. 15:10 X	-0.05133	-0.1953 to 0.09263	No
4014	3:1 X vs. 15:40 X	-0.03367	-0.1776 to 0.1103	No
4015	3:1 X vs. 15:50 X	-0.05133	-0.1953 to 0.09263	No
4016	3:1 X vs. 16:0 X	-0.1363	-0.2803 to 0.007626	No
4017	3:1 X vs. 16:1 X	-0.02733	-0.1713 to 0.1166	No
4018	3:1 X vs. 16:10 X	-0.0530	-0.1970 to 0.09096	No
4019	3:1 X vs. 16:40 X	-0.07233	-0.2163 to 0.07163	No
4020	3:1 X vs. 16:50 X	-0.1147	-0.2586 to 0.02929	No
4021	3:1 X vs. 17:0 X	-0.1890	-0.3330 to -0.04504	Yes
4022	3:1 X vs. 17:1 X	-0.1033	-0.2473 to 0.04063	No
4023	3:1 X vs. 17:10 X	-0.1600	-0.3040 to -0.01604	Yes
4024	3:1 X vs. 17:40 X	-0.08333	-0.2273 to 0.06063	No
4025	3:1 X vs. 17:50 X	-0.1287	-0.2726 to 0.01529	No
4026	3:1 X vs. 18:0 X	-0.2377	-0.3816 to -0.09371	Yes
4027	3:1 X vs. 18:1 X	-0.1460	-0.2900 to -0.002040	Yes
4028	3:1 X vs. 18:10 X	-0.2383	-0.3823 to -0.09437	Yes
4029	3:1 X vs. 18:40 X	-0.08767	-0.2316 to 0.05629	No
4030	3:1 X vs. 18:50 X	-0.1327	-0.2766 to 0.01129	No
4031	3:1 X vs. 19:0 X	-0.2727	-0.4166 to -0.1287	Yes
4032	3:1 X vs. 19:1 X	-0.2607	-0.4046 to -0.1167	Yes
4033	3:1 X vs. 19:10 X	-0.2583	-0.4023 to -0.1144	Yes
4034	3:1 X vs. 19:40 X	-0.1163	-0.2603 to 0.02763	No
4035	3:1 X vs. 19:50 X	-0.1273	-0.2713 to 0.01663	No
4036	3:1 X vs. 20:0 X	-0.3523	-0.4963 to -0.2084	Yes
4037	3:1 X vs. 20:1 X	-0.1717	-0.3156 to -0.02771	Yes
4038	3:1 X vs. 20:10 X	-0.2607	-0.4046 to -0.1167	Yes
4039	3:1 X vs. 20:40 X	-0.1280	-0.2720 to 0.01596	No
4040	3:1 X vs. 20:50 X	-0.1720	-0.3160 to -0.02804	Yes
4041	3:1 X vs. 21:0 X	-0.3757	-0.5196 to -0.2317	Yes
4042	3:1 X vs. 21:1 X	-0.3287	-0.4726 to -0.1847	Yes
4043	3:1 X vs. 21:10 X	-0.3257	-0.4696 to -0.1817	Yes
4044	3:1 X vs. 21:40 X	-0.2010	-0.3450 to -0.05704	Yes
4045	3:1 X vs. 21:50 X	-0.2913	-0.4353 to -0.1474	Yes
4046	3:1 X vs. 22:0 X	-0.4173	-0.5613 to -0.2734	Yes
4047	3:1 X vs. 22:1 X	-0.4120	-0.5560 to -0.2680	Yes
4048	3:1 X vs. 22:10 X	-0.4073	-0.5513 to -0.2634	Yes
4049	3:1 X vs. 22:40 X	-0.3077	-0.4516 to -0.1637	Yes
4050	3:1 X vs. 22:50 X	-0.3703	-0.5143 to -0.2264	Yes

	2way ANOVA<br>Multiple comparisons	A<br>Data Set-A<br>Y	B<br>Data Set-B<br>Y	C<br>Data Set-C<br>Y
**4051**	3:**1 X** vs. 23:**0 X**	-0.4827	-0.6266 to -0.3387	Yes
**4052**	3:**1 X** vs. 23:**1 X**	-0.6723	-0.8333 to -0.5114	Yes
**4053**	3:**1 X** vs. 23:**10 X**	-0.4220	-0.5660 to -0.2780	Yes
**4054**	3:**1 X** vs. 23:**40 X**	-0.3023	-0.4633 to -0.1414	Yes
**4055**	3:**1 X** vs. 23:**50 X**	-0.3363	-0.4803 to -0.1924	Yes
**4056**	3:**1 X** vs. 24:**0 X**	-0.6000	-0.7440 to -0.4560	Yes
**4057**	3:**1 X** vs. 24:**1 X**	-0.7563	-0.9173 to -0.5954	Yes
**4058**	3:**1 X** vs. 24:**10 X**	-0.5838	-0.7448 to -0.4229	Yes
**4059**	3:**1 X** vs. 24:**40 X**	-0.3187	-0.4626 to -0.1747	Yes
**4060**	3:**1 X** vs. 24:**50 X**	-0.2403	-0.3843 to -0.09637	Yes
**4061**	3:**1 X** vs. 25:**0 X**	-0.9373	-1.098 to -0.7764	Yes
**4062**	3:**1 X** vs. 25:**1 X**	-1.127	-1.288 to -0.9659	Yes
**4063**	3:**1 X** vs. 25:**10 X**	-0.4883	-0.6919 to -0.2847	Yes
**4064**	3:**1 X** vs. 25:**40 X**	-0.3207	-0.4646 to -0.1767	Yes
**4065**	3:**1 X** vs. 25:**50 X**	-0.2950	-0.4390 to -0.1510	Yes
**4066**	3:**1 X** vs. 26:**0 X**	-1.150	-1.311 to -0.9889	Yes
**4067**	3:**1 X** vs. 26:**1 X**	-1.263	-1.424 to -1.102	Yes
**4068**	3:**1 X** vs. 26:**10 X**	-0.9493	-1.110 to -0.7884	Yes
**4069**	3:**1 X** vs. 26:**40 X**	-0.3950	-0.5390 to -0.2510	Yes
**4070**	3:**1 X** vs. 26:**50 X**	-0.2553	-0.3993 to -0.1114	Yes
**4071**	3:**1 X** vs. 27:**0 X**	-1.338	-1.482 to -1.194	Yes
**4072**	3:**1 X** vs. 27:**1 X**	-1.428	-1.589 to -1.267	Yes
**4073**	3:**1 X** vs. 27:**10 X**	-1.240	-1.401 to -1.079	Yes
**4074**	3:**1 X** vs. 27:**40 X**	-0.3950	-0.5390 to -0.2510	Yes
**4075**	3:**1 X** vs. 27:**50 X**	-0.2547	-0.3986 to -0.1107	Yes
**4076**	3:**1 X** vs. 28:**0 X**	-1.287	-1.448 to -1.126	Yes
**4077**	3:**1 X** vs. 28:**1 X**	-1.328	-1.472 to -1.184	Yes
**4078**	3:**1 X** vs. 28:**10 X**	-1.129	-1.290 to -0.9684	Yes
**4079**	3:**1 X** vs. 28:**40 X**	-0.4073	-0.5513 to -0.2634	Yes
**4080**	3:**1 X** vs. 28:**50 X**	-0.3027	-0.4466 to -0.1587	Yes
**4081**	3:**1 X** vs. 29:**0 X**	-1.768	-1.929 to -1.607	Yes
**4082**	3:**1 X** vs. 29:**1 X**	-1.853	-2.014 to -1.692	Yes
**4083**	3:**1 X** vs. 29:**10 X**	-1.068	-1.229 to -0.9069	Yes
**4084**	3:**1 X** vs. 29:**40 X**	-0.5153	-0.6593 to -0.3714	Yes
**4085**	3:**1 X** vs. 29:**50 X**	-0.3040	-0.4480 to -0.1600	Yes
**4086**	3:**1 X** vs. 30:**0 X**	-1.483	-1.644 to -1.322	Yes
**4087**	3:**1 X** vs. 30:**1 X**	-1.804	-1.965 to -1.643	Yes
**4088**	3:**1 X** vs. 30:**10 X**	-1.142	-1.303 to -0.9814	Yes
**4089**	3:**1 X** vs. 30:**40 X**	-0.4423	-0.5863 to -0.2984	Yes
**4090**	3:**1 X** vs. 30:**50 X**	-0.3563	-0.5003 to -0.2124	Yes
**4091**	3:**1 X** vs. 31:**0 X**	-1.801	-1.962 to -1.640	Yes
**4092**	3:**1 X** vs. 31:**1 X**	-1.791	-1.952 to -1.630	Yes
**4093**	3:**1 X** vs. 31:**10 X**	-1.274	-1.435 to -1.113	Yes
**4094**	3:**1 X** vs. 31:**40 X**	-0.5340	-0.6780 to -0.3900	Yes
**4095**	3:**1 X** vs. 31:**50 X**	-0.3353	-0.4793 to -0.1914	Yes

	2way ANOVA Multiple comparisons	A Data Set-A Y	B Data Set-B Y	C Data Set-C Y
4096	3:1 X vs. 32:0 X	-2.392	-2.536 to -2.248	Yes
4097	3:1 X vs. 32:1 X	-1.848	-2.009 to -1.687	Yes
4098	3:1 X vs. 32:10 X	-1.704	-1.848 to -1.560	Yes
4099	3:1 X vs. 32:40 X	-0.5303	-0.6743 to -0.3864	Yes
4100	3:1 X vs. 32:50 X	-0.3350	-0.4790 to -0.1910	Yes
4101	3:1 X vs. 33:0 X	-2.396	-2.540 to -2.252	Yes
4102	3:1 X vs. 33:1 X	-2.211	-2.355 to -2.067	Yes
4103	3:1 X vs. 33:10 X	-1.871	-2.015 to -1.727	Yes
4104	3:1 X vs. 33:40 X	-0.5490	-0.6930 to -0.4050	Yes
4105	3:1 X vs. 33:50 X	-0.3500	-0.4940 to -0.2060	Yes
4106	3:1 X vs. 34:0 X	-2.321	-2.465 to -2.177	Yes
4107	3:1 X vs. 34:1 X	-2.328	-2.472 to -2.184	Yes
4108	3:1 X vs. 34:10 X	-1.961	-2.105 to -1.817	Yes
4109	3:1 X vs. 34:40 X	-0.5610	-0.7050 to -0.4170	Yes
4110	3:1 X vs. 34:50 X	-0.4990	-0.6430 to -0.3550	Yes
4111	3:1 X vs. 35:0 X	-2.669	-2.813 to -2.525	Yes
4112	3:1 X vs. 35:1 X	-2.435	-2.579 to -2.291	Yes
4113	3:1 X vs. 35:10 X	-1.923	-2.067 to -1.779	Yes
4114	3:1 X vs. 35:40 X	-0.5700	-0.7140 to -0.4260	Yes
4115	3:1 X vs. 35:50 X	-0.5117	-0.6556 to -0.3677	Yes
4116	3:1 X vs. 36:0 X	-2.876	-3.037 to -2.715	Yes
4117	3:1 X vs. 36:1 X	-2.572	-2.716 to -2.428	Yes
4118	3:1 X vs. 36:10 X	-1.929	-2.073 to -1.785	Yes
4119	3:1 X vs. 36:40 X	-0.6253	-0.7693 to -0.4814	Yes
4120	3:1 X vs. 36:50 X	-0.4750	-0.6190 to -0.3310	Yes
4121	3:1 X vs. 37:0 X	-3.260	-3.404 to -3.116	Yes
4122	3:1 X vs. 37:1 X	-2.642	-2.786 to -2.498	Yes
4123	3:1 X vs. 37:10 X	-1.930	-2.074 to -1.786	Yes
4124	3:1 X vs. 37:40 X	-0.6627	-0.8066 to -0.5187	Yes
4125	3:1 X vs. 37:50 X	-0.4837	-0.6276 to -0.3397	Yes
4126	3:1 X vs. 38:0 X	-3.205	-3.349 to -3.061	Yes
4127	3:1 X vs. 38:1 X	-2.760	-2.904 to -2.616	Yes
4128	3:1 X vs. 38:10 X	-2.206	-2.350 to -2.062	Yes
4129	3:1 X vs. 38:40 X	-0.6670	-0.8110 to -0.5230	Yes
4130	3:1 X vs. 38:50 X	-0.4877	-0.6316 to -0.3437	Yes
4131	3:1 X vs. 39:0 X	-3.435	-3.579 to -3.291	Yes
4132	3:1 X vs. 39:1 X	-3.327	-3.471 to -3.183	Yes
4133	3:1 X vs. 39:10 X	-2.351	-2.495 to -2.207	Yes
4134	3:1 X vs. 39:40 X	-0.6730	-0.8170 to -0.5290	Yes
4135	3:1 X vs. 39:50 X	-0.5187	-0.6626 to -0.3747	Yes
4136	3:1 X vs. 40:0 X	-3.476	-3.620 to -3.332	Yes
4137	3:1 X vs. 40:1 X	-3.402	-3.546 to -3.258	Yes
4138	3:1 X vs. 40:10 X	-2.607	-2.751 to -2.463	Yes
4139	3:1 X vs. 40:40 X	-0.6777	-0.8216 to -0.5337	Yes
4140	3:1 X vs. 40:50 X	-0.5250	-0.6690 to -0.3810	Yes

2way ANOVA<br>Multiple comparisons		A<br>Data Set-A<br>Y	B<br>Data Set-B<br>Y	C<br>Data Set-C<br>Y
4141	3:1 X vs. 41:0 X	-3.517	-3.661 to -3.373	Yes
4142	3:1 X vs. 41:1 X	-3.516	-3.660 to -3.372	Yes
4143	3:1 X vs. 41:10 X	-2.661	-2.805 to -2.517	Yes
4144	3:1 X vs. 41:40 X	-0.6250	-0.7690 to -0.4810	Yes
4145	3:1 X vs. 41:50 X	-0.5377	-0.6816 to -0.3937	Yes
4146	3:1 X vs. 42:0 X	-3.659	-3.803 to -3.515	Yes
4147	3:1 X vs. 42:1 X	-3.679	-3.823 to -3.535	Yes
4148	3:1 X vs. 42:10 X	-2.661	-2.805 to -2.517	Yes
4149	3:1 X vs. 42:40 X	-0.6377	-0.7816 to -0.4937	Yes
4150	3:1 X vs. 42:50 X	-0.5410	-0.6850 to -0.3970	Yes
4151	3:1 X vs. 43:0 X	-3.749	-3.893 to -3.605	Yes
4152	3:1 X vs. 43:1 X	-3.663	-3.807 to -3.519	Yes
4153	3:1 X vs. 43:10 X	-3.379	-3.523 to -3.235	Yes
4154	3:1 X vs. 43:40 X	-0.6360	-0.7800 to -0.4920	Yes
4155	3:1 X vs. 43:50 X	-0.5413	-0.6853 to -0.3974	Yes
4156	3:1 X vs. 44:0 X	-3.773	-3.917 to -3.629	Yes
4157	3:1 X vs. 44:1 X	-3.663	-3.807 to -3.519	Yes
4158	3:1 X vs. 44:10 X	-3.442	-3.586 to -3.298	Yes
4159	3:1 X vs. 44:40 X	-0.6380	-0.7820 to -0.4940	Yes
4160	3:1 X vs. 44:50 X	-0.4737	-0.6176 to -0.3297	Yes
4161	3:1 X vs. 45:0 X	-3.638	-3.782 to -3.494	Yes
4162	3:1 X vs. 45:1 X	-3.440	-3.584 to -3.296	Yes
4163	3:1 X vs. 45:10 X	-3.322	-3.466 to -3.178	Yes
4164	3:1 X vs. 45:40 X	-0.6393	-0.7833 to -0.4954	Yes
4165	3:1 X vs. 45:50 X	-0.4707	-0.6146 to -0.3267	Yes
4166	3:1 X vs. 46:0 X	-3.442	-3.586 to -3.298	Yes
4167	3:1 X vs. 46:1 X	-3.409	-3.553 to -3.265	Yes
4168	3:1 X vs. 46:10 X	-3.317	-3.461 to -3.173	Yes
4169	3:1 X vs. 46:40 X	-0.6530	-0.7970 to -0.5090	Yes
4170	3:1 X vs. 46:50 X	-0.4637	-0.6076 to -0.3197	Yes
4171	3:1 X vs. 47:0 X	-3.418	-3.562 to -3.274	Yes
4172	3:1 X vs. 47:1 X	-3.322	-3.466 to -3.178	Yes
4173	3:1 X vs. 47:10 X	-3.303	-3.447 to -3.159	Yes
4174	3:1 X vs. 47:40 X	-0.6630	-0.8070 to -0.5190	Yes
4175	3:1 X vs. 47:50 X	-0.4217	-0.5656 to -0.2777	Yes
4176	3:1 X vs. 48:0 X	-3.405	-3.566 to -3.244	Yes
4177	3:1 X vs. 48:1 X	-3.309	-3.453 to -3.165	Yes
4178	3:1 X vs. 48:10 X	-3.303	-3.447 to -3.159	Yes
4179	3:1 X vs. 48:40 X	-0.6693	-0.8133 to -0.5254	Yes
4180	3:1 X vs. 48:50 X	-0.4290	-0.5730 to -0.2850	Yes
4181	3:1 X vs. 49:0 X	-3.405	-3.549 to -3.261	Yes
4182	3:1 X vs. 49:1 X	-3.309	-3.453 to -3.165	Yes
4183	3:1 X vs. 49:10 X	-3.303	-3.447 to -3.159	Yes
4184	3:1 X vs. 49:40 X	-0.6737	-0.8176 to -0.5297	Yes
4185	3:1 X vs. 49:50 X	-0.4283	-0.5723 to -0.2844	Yes

	2way ANOVA<br>Multiple comparisons	A<br>Data Set-A<br>Y	B<br>Data Set-B<br>Y	C<br>Data Set-C<br>Y
4186	3:**1 X** vs. 50:**0 X**	-3.405	-3.549 to -3.261	Yes
4187	3:**1 X** vs. 50:**1 X**	-3.309	-3.453 to -3.165	Yes
4188	3:**1 X** vs. 50:**10 X**	-3.303	-3.447 to -3.159	Yes
4189	3:**1 X** vs. 50:**40 X**	-0.6813	-0.8253 to -0.5374	Yes
4190	3:**1 X** vs. 50:**50 X**	-0.4280	-0.5720 to -0.2840	Yes
4191	3:**10 X** vs. 3:**40 X**	0.002333	-0.1416 to 0.1463	No
4192	3:**10 X** vs. 3:**50 X**	0.003333	-0.1406 to 0.1473	No
4193	3:**10 X** vs. 4:**0 X**	-0.006667	-0.1506 to 0.1373	No
4194	3:**10 X** vs. 4:**1 X**	-0.005333	-0.1493 to 0.1386	No
4195	3:**10 X** vs. 4:**10 X**	-0.0200	-0.1640 to 0.1240	No
4196	3:**10 X** vs. 4:**40 X**	0.0003333	-0.1436 to 0.1443	No
4197	3:**10 X** vs. 4:**50 X**	0.002333	-0.1416 to 0.1463	No
4198	3:**10 X** vs. 5:**0 X**	-0.007667	-0.1516 to 0.1363	No
4199	3:**10 X** vs. 5:**1 X**	-0.005667	-0.1496 to 0.1383	No
4200	3:**10 X** vs. 5:**10 X**	-0.02067	-0.1646 to 0.1233	No
4201	3:**10 X** vs. 5:**40 X**	0.0003333	-0.1436 to 0.1443	No
4202	3:**10 X** vs. 5:**50 X**	0.001333	-0.1426 to 0.1453	No
4203	3:**10 X** vs. 6:**0 X**	-0.0070	-0.1510 to 0.1370	No
4204	3:**10 X** vs. 6:**1 X**	-0.0060	-0.1500 to 0.1380	No
4205	3:**10 X** vs. 6:**10 X**	-0.02067	-0.1646 to 0.1233	No
4206	3:**10 X** vs. 6:**40 X**	-0.0020	-0.1460 to 0.1420	No
4207	3:**10 X** vs. 6:**50 X**	-0.0003333	-0.1443 to 0.1436	No
4208	3:**10 X** vs. 7:**0 X**	-0.0090	-0.1530 to 0.1350	No
4209	3:**10 X** vs. 7:**1 X**	-0.0003333	-0.1443 to 0.1436	No
4210	3:**10 X** vs. 7:**10 X**	-0.01233	-0.1563 to 0.1316	No
4211	3:**10 X** vs. 7:**40 X**	-0.003667	-0.1476 to 0.1403	No
4212	3:**10 X** vs. 7:**50 X**	-0.001667	-0.1456 to 0.1423	No
4213	3:**10 X** vs. 8:**0 X**	-0.01367	-0.1576 to 0.1303	No
4214	3:**10 X** vs. 8:**1 X**	-0.0003333	-0.1443 to 0.1436	No
4215	3:**10 X** vs. 8:**10 X**	-0.007667	-0.1516 to 0.1363	No
4216	3:**10 X** vs. 8:**40 X**	-0.008333	-0.1523 to 0.1356	No
4217	3:**10 X** vs. 8:**50 X**	-0.005333	-0.1493 to 0.1386	No
4218	3:**10 X** vs. 9:**0 X**	-0.02167	-0.1656 to 0.1223	No
4219	3:**10 X** vs. 9:**1 X**	-0.02967	-0.1736 to 0.1143	No
4220	3:**10 X** vs. 9:**10 X**	-0.03067	-0.1746 to 0.1133	No
4221	3:**10 X** vs. 9:**40 X**	-0.0130	-0.1570 to 0.1310	No
4222	3:**10 X** vs. 9:**50 X**	-0.006333	-0.1503 to 0.1376	No
4223	3:**10 X** vs. 10:**0 X**	-0.02333	-0.1673 to 0.1206	No
4224	3:**10 X** vs. 10:**1 X**	-0.03033	-0.1743 to 0.1136	No
4225	3:**10 X** vs. 10:**10 X**	-0.0330	-0.1770 to 0.1110	No
4226	3:**10 X** vs. 10:**40 X**	-0.02267	-0.1666 to 0.1213	No
4227	3:**10 X** vs. 10:**50 X**	-0.009333	-0.1533 to 0.1346	No
4228	3:**10 X** vs. 11:**0 X**	-0.0330	-0.1770 to 0.1110	No
4229	3:**10 X** vs. 11:**1 X**	-0.03467	-0.1786 to 0.1093	No
4230	3:**10 X** vs. 11:**10 X**	-0.0350	-0.1790 to 0.1090	No

	2way ANOVA<br>Multiple comparisons	A<br>Data Set-A<br>Y	B<br>Data Set-B<br>Y	C<br>Data Set-C<br>Y
**4231**	3:**10 X** vs. 11:**40 X**	-0.02667	-0.1706 to 0.1173	No
**4232**	3:**10 X** vs. 11:**50 X**	-0.01467	-0.1586 to 0.1293	No
**4233**	3:**10 X** vs. 12:**0 X**	-0.03367	-0.1776 to 0.1103	No
**4234**	3:**10 X** vs. 12:**1 X**	-0.0160	-0.1600 to 0.1280	No
**4235**	3:**10 X** vs. 12:**10 X**	-0.03367	-0.1776 to 0.1103	No
**4236**	3:**10 X** vs. 12:**40 X**	-0.0200	-0.1640 to 0.1240	No
**4237**	3:**10 X** vs. 12:**50 X**	-0.02333	-0.1673 to 0.1206	No
**4238**	3:**10 X** vs. 13:**0 X**	-0.02767	-0.1716 to 0.1163	No
**4239**	3:**10 X** vs. 13:**1 X**	-0.0150	-0.1590 to 0.1290	No
**4240**	3:**10 X** vs. 13:**10 X**	-0.0370	-0.1810 to 0.1070	No
**4241**	3:**10 X** vs. 13:**40 X**	-0.03333	-0.1773 to 0.1106	No
**4242**	3:**10 X** vs. 13:**50 X**	-0.03433	-0.1783 to 0.1096	No
**4243**	3:**10 X** vs. 14:**0 X**	-0.03633	-0.1803 to 0.1076	No
**4244**	3:**10 X** vs. 14:**1 X**	-0.0280	-0.1720 to 0.1160	No
**4245**	3:**10 X** vs. 14:**10 X**	-0.0420	-0.1860 to 0.1020	No
**4246**	3:**10 X** vs. 14:**40 X**	-0.03867	-0.1826 to 0.1053	No
**4247**	3:**10 X** vs. 14:**50 X**	-0.04267	-0.1866 to 0.1013	No
**4248**	3:**10 X** vs. 15:**0 X**	-0.04867	-0.1926 to 0.09529	No
**4249**	3:**10 X** vs. 15:**1 X**	-0.02867	-0.1726 to 0.1153	No
**4250**	3:**10 X** vs. 15:**10 X**	-0.0560	-0.2000 to 0.08796	No
**4251**	3:**10 X** vs. 15:**40 X**	-0.03833	-0.1823 to 0.1056	No
**4252**	3:**10 X** vs. 15:**50 X**	-0.0560	-0.2000 to 0.08796	No
**4253**	3:**10 X** vs. 16:**0 X**	-0.1410	-0.2850 to 0.002960	No
**4254**	3:**10 X** vs. 16:**1 X**	-0.0320	-0.1760 to 0.1120	No
**4255**	3:**10 X** vs. 16:**10 X**	-0.05767	-0.2016 to 0.08629	No
**4256**	3:**10 X** vs. 16:**40 X**	-0.0770	-0.2210 to 0.06696	No
**4257**	3:**10 X** vs. 16:**50 X**	-0.1193	-0.2633 to 0.02463	No
**4258**	3:**10 X** vs. 17:**0 X**	-0.1937	-0.3376 to -0.04971	Yes
**4259**	3:**10 X** vs. 17:**1 X**	-0.1080	-0.2520 to 0.03596	No
**4260**	3:**10 X** vs. 17:**10 X**	-0.1647	-0.3086 to -0.02071	Yes
**4261**	3:**10 X** vs. 17:**40 X**	-0.0880	-0.2320 to 0.05596	No
**4262**	3:**10 X** vs. 17:**50 X**	-0.1333	-0.2773 to 0.01063	No
**4263**	3:**10 X** vs. 18:**0 X**	-0.2423	-0.3863 to -0.09837	Yes
**4264**	3:**10 X** vs. 18:**1 X**	-0.1507	-0.2946 to -0.006707	Yes
**4265**	3:**10 X** vs. 18:**10 X**	-0.2430	-0.3870 to -0.09904	Yes
**4266**	3:**10 X** vs. 18:**40 X**	-0.09233	-0.2363 to 0.05163	No
**4267**	3:**10 X** vs. 18:**50 X**	-0.1373	-0.2813 to 0.006626	No
**4268**	3:**10 X** vs. 19:**0 X**	-0.2773	-0.4213 to -0.1334	Yes
**4269**	3:**10 X** vs. 19:**1 X**	-0.2653	-0.4093 to -0.1214	Yes
**4270**	3:**10 X** vs. 19:**10 X**	-0.2630	-0.4070 to -0.1190	Yes
**4271**	3:**10 X** vs. 19:**40 X**	-0.1210	-0.2650 to 0.02296	No
**4272**	3:**10 X** vs. 19:**50 X**	-0.1320	-0.2760 to 0.01196	No
**4273**	3:**10 X** vs. 20:**0 X**	-0.3570	-0.5010 to -0.2130	Yes
**4274**	3:**10 X** vs. 20:**1 X**	-0.1763	-0.3203 to -0.03237	Yes
**4275**	3:**10 X** vs. 20:**10 X**	-0.2653	-0.4093 to -0.1214	Yes

	2way ANOVA<br>Multiple comparisons	A<br>Data Set-A<br>Y	B<br>Data Set-B<br>Y	C<br>Data Set-C<br>Y
4276	3:**10 X** vs. 20:**40 X**	-0.1327	-0.2766 to 0.01129	No
4277	3:**10 X** vs. 20:**50 X**	-0.1767	-0.3206 to -0.03271	Yes
4278	3:**10 X** vs. 21:**0 X**	-0.3803	-0.5243 to -0.2364	Yes
4279	3:**10 X** vs. 21:**1 X**	-0.3333	-0.4773 to -0.1894	Yes
4280	3:**10 X** vs. 21:**10 X**	-0.3303	-0.4743 to -0.1864	Yes
4281	3:**10 X** vs. 21:**40 X**	-0.2057	-0.3496 to -0.06171	Yes
4282	3:**10 X** vs. 21:**50 X**	-0.2960	-0.4400 to -0.1520	Yes
4283	3:**10 X** vs. 22:**0 X**	-0.4220	-0.5660 to -0.2780	Yes
4284	3:**10 X** vs. 22:**1 X**	-0.4167	-0.5606 to -0.2727	Yes
4285	3:**10 X** vs. 22:**10 X**	-0.4120	-0.5560 to -0.2680	Yes
4286	3:**10 X** vs. 22:**40 X**	-0.3123	-0.4563 to -0.1684	Yes
4287	3:**10 X** vs. 22:**50 X**	-0.3750	-0.5190 to -0.2310	Yes
4288	3:**10 X** vs. 23:**0 X**	-0.4873	-0.6313 to -0.3434	Yes
4289	3:**10 X** vs. 23:**1 X**	-0.6770	-0.8380 to -0.5160	Yes
4290	3:**10 X** vs. 23:**10 X**	-0.4267	-0.5706 to -0.2827	Yes
4291	3:**10 X** vs. 23:**40 X**	-0.3070	-0.4680 to -0.1460	Yes
4292	3:**10 X** vs. 23:**50 X**	-0.3410	-0.4850 to -0.1970	Yes
4293	3:**10 X** vs. 24:**0 X**	-0.6047	-0.7486 to -0.4607	Yes
4294	3:**10 X** vs. 24:**1 X**	-0.7610	-0.9220 to -0.6000	Yes
4295	3:**10 X** vs. 24:**10 X**	-0.5885	-0.7495 to -0.4275	Yes
4296	3:**10 X** vs. 24:**40 X**	-0.3233	-0.4673 to -0.1794	Yes
4297	3:**10 X** vs. 24:**50 X**	-0.2450	-0.3890 to -0.1010	Yes
4298	3:**10 X** vs. 25:**0 X**	-0.9420	-1.103 to -0.7810	Yes
4299	3:**10 X** vs. 25:**1 X**	-1.132	-1.292 to -0.9705	Yes
4300	3:**10 X** vs. 25:**10 X**	-0.4930	-0.6966 to -0.2894	Yes
4301	3:**10 X** vs. 25:**40 X**	-0.3253	-0.4693 to -0.1814	Yes
4302	3:**10 X** vs. 25:**50 X**	-0.2997	-0.4436 to -0.1557	Yes
4303	3:**10 X** vs. 26:**0 X**	-1.155	-1.315 to -0.9935	Yes
4304	3:**10 X** vs. 26:**1 X**	-1.268	-1.428 to -1.107	Yes
4305	3:**10 X** vs. 26:**10 X**	-0.9540	-1.115 to -0.7930	Yes
4306	3:**10 X** vs. 26:**40 X**	-0.3997	-0.5436 to -0.2557	Yes
4307	3:**10 X** vs. 26:**50 X**	-0.2600	-0.4040 to -0.1160	Yes
4308	3:**10 X** vs. 27:**0 X**	-1.342	-1.486 to -1.198	Yes
4309	3:**10 X** vs. 27:**1 X**	-1.433	-1.594 to -1.272	Yes
4310	3:**10 X** vs. 27:**10 X**	-1.245	-1.406 to -1.084	Yes
4311	3:**10 X** vs. 27:**40 X**	-0.3997	-0.5436 to -0.2557	Yes
4312	3:**10 X** vs. 27:**50 X**	-0.2593	-0.4033 to -0.1154	Yes
4313	3:**10 X** vs. 28:**0 X**	-1.292	-1.452 to -1.131	Yes
4314	3:**10 X** vs. 28:**1 X**	-1.332	-1.476 to -1.188	Yes
4315	3:**10 X** vs. 28:**10 X**	-1.134	-1.295 to -0.9730	Yes
4316	3:**10 X** vs. 28:**40 X**	-0.4120	-0.5560 to -0.2680	Yes
4317	3:**10 X** vs. 28:**50 X**	-0.3073	-0.4513 to -0.1634	Yes
4318	3:**10 X** vs. 29:**0 X**	-1.773	-1.934 to -1.612	Yes
4319	3:**10 X** vs. 29:**1 X**	-1.858	-2.019 to -1.697	Yes
4320	3:**10 X** vs. 29:**10 X**	-1.073	-1.233 to -0.9115	Yes

	2way ANOVA<br>Multiple comparisons	A<br>Data Set-A<br>Y	B<br>Data Set-B<br>Y	C<br>Data Set-C<br>Y
4321	3:10 X vs. 29:40 X	-0.5200	-0.6640 to -0.3760	Yes
4322	3:10 X vs. 29:50 X	-0.3087	-0.4526 to -0.1647	Yes
4323	3:10 X vs. 30:0 X	-1.488	-1.648 to -1.327	Yes
4324	3:10 X vs. 30:1 X	-1.809	-1.970 to -1.648	Yes
4325	3:10 X vs. 30:10 X	-1.147	-1.308 to -0.9860	Yes
4326	3:10 X vs. 30:40 X	-0.4470	-0.5910 to -0.3030	Yes
4327	3:10 X vs. 30:50 X	-0.3610	-0.5050 to -0.2170	Yes
4328	3:10 X vs. 31:0 X	-1.806	-1.966 to -1.645	Yes
4329	3:10 X vs. 31:1 X	-1.796	-1.957 to -1.635	Yes
4330	3:10 X vs. 31:10 X	-1.279	-1.439 to -1.118	Yes
4331	3:10 X vs. 31:40 X	-0.5387	-0.6826 to -0.3947	Yes
4332	3:10 X vs. 31:50 X	-0.3400	-0.4840 to -0.1960	Yes
4333	3:10 X vs. 32:0 X	-2.397	-2.541 to -2.253	Yes
4334	3:10 X vs. 32:1 X	-1.853	-2.014 to -1.692	Yes
4335	3:10 X vs. 32:10 X	-1.709	-1.853 to -1.565	Yes
4336	3:10 X vs. 32:40 X	-0.5350	-0.6790 to -0.3910	Yes
4337	3:10 X vs. 32:50 X	-0.3397	-0.4836 to -0.1957	Yes
4338	3:10 X vs. 33:0 X	-2.401	-2.545 to -2.257	Yes
4339	3:10 X vs. 33:1 X	-2.215	-2.359 to -2.071	Yes
4340	3:10 X vs. 33:10 X	-1.876	-2.020 to -1.732	Yes
4341	3:10 X vs. 33:40 X	-0.5537	-0.6976 to -0.4097	Yes
4342	3:10 X vs. 33:50 X	-0.3547	-0.4986 to -0.2107	Yes
4343	3:10 X vs. 34:0 X	-2.325	-2.469 to -2.181	Yes
4344	3:10 X vs. 34:1 X	-2.333	-2.477 to -2.189	Yes
4345	3:10 X vs. 34:10 X	-1.966	-2.110 to -1.822	Yes
4346	3:10 X vs. 34:40 X	-0.5657	-0.7096 to -0.4217	Yes
4347	3:10 X vs. 34:50 X	-0.5037	-0.6476 to -0.3597	Yes
4348	3:10 X vs. 35:0 X	-2.674	-2.818 to -2.530	Yes
4349	3:10 X vs. 35:1 X	-2.440	-2.584 to -2.296	Yes
4350	3:10 X vs. 35:10 X	-1.928	-2.072 to -1.784	Yes
4351	3:10 X vs. 35:40 X	-0.5747	-0.7186 to -0.4307	Yes
4352	3:10 X vs. 35:50 X	-0.5163	-0.6603 to -0.3724	Yes
4353	3:10 X vs. 36:0 X	-2.881	-3.041 to -2.720	Yes
4354	3:10 X vs. 36:1 X	-2.576	-2.720 to -2.432	Yes
4355	3:10 X vs. 36:10 X	-1.933	-2.077 to -1.789	Yes
4356	3:10 X vs. 36:40 X	-0.6300	-0.7740 to -0.4860	Yes
4357	3:10 X vs. 36:50 X	-0.4797	-0.6236 to -0.3357	Yes
4358	3:10 X vs. 37:0 X	-3.264	-3.408 to -3.120	Yes
4359	3:10 X vs. 37:1 X	-2.646	-2.790 to -2.502	Yes
4360	3:10 X vs. 37:10 X	-1.935	-2.079 to -1.791	Yes
4361	3:10 X vs. 37:40 X	-0.6673	-0.8113 to -0.5234	Yes
4362	3:10 X vs. 37:50 X	-0.4883	-0.6323 to -0.3444	Yes
4363	3:10 X vs. 38:0 X	-3.209	-3.353 to -3.065	Yes
4364	3:10 X vs. 38:1 X	-2.765	-2.909 to -2.621	Yes
4365	3:10 X vs. 38:10 X	-2.210	-2.354 to -2.066	Yes

	2way ANOVA<br>Multiple comparisons	A<br>Data Set-A<br>Y	B<br>Data Set-B<br>Y	C<br>Data Set-C<br>Y
4366	3:**10 X** vs. 38:**40 X**	-0.6717	-0.8156 to -0.5277	Yes
4367	3:**10 X** vs. 38:**50 X**	-0.4923	-0.6363 to -0.3484	Yes
4368	3:**10 X** vs. 39:**0 X**	-3.440	-3.584 to -3.296	Yes
4369	3:**10 X** vs. 39:**1 X**	-3.332	-3.476 to -3.188	Yes
4370	3:**10 X** vs. 39:**10 X**	-2.355	-2.499 to -2.211	Yes
4371	3:**10 X** vs. 39:**40 X**	-0.6777	-0.8216 to -0.5337	Yes
4372	3:**10 X** vs. 39:**50 X**	-0.5233	-0.6673 to -0.3794	Yes
4373	3:**10 X** vs. 40:**0 X**	-3.481	-3.625 to -3.337	Yes
4374	3:**10 X** vs. 40:**1 X**	-3.407	-3.551 to -3.263	Yes
4375	3:**10 X** vs. 40:**10 X**	-2.612	-2.756 to -2.468	Yes
4376	3:**10 X** vs. 40:**40 X**	-0.6823	-0.8263 to -0.5384	Yes
4377	3:**10 X** vs. 40:**50 X**	-0.5297	-0.6736 to -0.3857	Yes
4378	3:**10 X** vs. 41:**0 X**	-3.522	-3.666 to -3.378	Yes
4379	3:**10 X** vs. 41:**1 X**	-3.521	-3.665 to -3.377	Yes
4380	3:**10 X** vs. 41:**10 X**	-2.665	-2.809 to -2.521	Yes
4381	3:**10 X** vs. 41:**40 X**	-0.6297	-0.7736 to -0.4857	Yes
4382	3:**10 X** vs. 41:**50 X**	-0.5423	-0.6863 to -0.3984	Yes
4383	3:**10 X** vs. 42:**0 X**	-3.664	-3.808 to -3.520	Yes
4384	3:**10 X** vs. 42:**1 X**	-3.683	-3.827 to -3.539	Yes
4385	3:**10 X** vs. 42:**10 X**	-2.666	-2.810 to -2.522	Yes
4386	3:**10 X** vs. 42:**40 X**	-0.6423	-0.7863 to -0.4984	Yes
4387	3:**10 X** vs. 42:**50 X**	-0.5457	-0.6896 to -0.4017	Yes
4388	3:**10 X** vs. 43:**0 X**	-3.754	-3.898 to -3.610	Yes
4389	3:**10 X** vs. 43:**1 X**	-3.668	-3.812 to -3.524	Yes
4390	3:**10 X** vs. 43:**10 X**	-3.384	-3.528 to -3.240	Yes
4391	3:**10 X** vs. 43:**40 X**	-0.6407	-0.7846 to -0.4967	Yes
4392	3:**10 X** vs. 43:**50 X**	-0.5460	-0.6900 to -0.4020	Yes
4393	3:**10 X** vs. 44:**0 X**	-3.778	-3.922 to -3.634	Yes
4394	3:**10 X** vs. 44:**1 X**	-3.667	-3.811 to -3.523	Yes
4395	3:**10 X** vs. 44:**10 X**	-3.447	-3.591 to -3.303	Yes
4396	3:**10 X** vs. 44:**40 X**	-0.6427	-0.7866 to -0.4987	Yes
4397	3:**10 X** vs. 44:**50 X**	-0.4783	-0.6223 to -0.3344	Yes
4398	3:**10 X** vs. 45:**0 X**	-3.642	-3.786 to -3.498	Yes
4399	3:**10 X** vs. 45:**1 X**	-3.444	-3.588 to -3.300	Yes
4400	3:**10 X** vs. 45:**10 X**	-3.327	-3.471 to -3.183	Yes
4401	3:**10 X** vs. 45:**40 X**	-0.6440	-0.7880 to -0.5000	Yes
4402	3:**10 X** vs. 45:**50 X**	-0.4753	-0.6193 to -0.3314	Yes
4403	3:**10 X** vs. 46:**0 X**	-3.447	-3.591 to -3.303	Yes
4404	3:**10 X** vs. 46:**1 X**	-3.414	-3.558 to -3.270	Yes
4405	3:**10 X** vs. 46:**10 X**	-3.322	-3.466 to -3.178	Yes
4406	3:**10 X** vs. 46:**40 X**	-0.6577	-0.8016 to -0.5137	Yes
4407	3:**10 X** vs. 46:**50 X**	-0.4683	-0.6123 to -0.3244	Yes
4408	3:**10 X** vs. 47:**0 X**	-3.422	-3.566 to -3.278	Yes
4409	3:**10 X** vs. 47:**1 X**	-3.326	-3.470 to -3.182	Yes
4410	3:**10 X** vs. 47:**10 X**	-3.308	-3.452 to -3.164	Yes

	2way ANOVA<br>Multiple comparisons	A<br>Data Set-A<br>Y	B<br>Data Set-B<br>Y	C<br>Data Set-C<br>Y
4411	3:**10 X** vs. 47:**40 X**	-0.6677	-0.8116 to -0.5237	Yes
4412	3:**10 X** vs. 47:**50 X**	-0.4263	-0.5703 to -0.2824	Yes
4413	3:**10 X** vs. 48:**0 X**	-3.410	-3.570 to -3.249	Yes
4414	3:**10 X** vs. 48:**1 X**	-3.313	-3.457 to -3.169	Yes
4415	3:**10 X** vs. 48:**10 X**	-3.308	-3.452 to -3.164	Yes
4416	3:**10 X** vs. 48:**40 X**	-0.6740	-0.8180 to -0.5300	Yes
4417	3:**10 X** vs. 48:**50 X**	-0.4337	-0.5776 to -0.2897	Yes
4418	3:**10 X** vs. 49:**0 X**	-3.410	-3.554 to -3.266	Yes
4419	3:**10 X** vs. 49:**1 X**	-3.313	-3.457 to -3.169	Yes
4420	3:**10 X** vs. 49:**10 X**	-3.308	-3.452 to -3.164	Yes
4421	3:**10 X** vs. 49:**40 X**	-0.6783	-0.8223 to -0.5344	Yes
4422	3:**10 X** vs. 49:**50 X**	-0.4330	-0.5770 to -0.2890	Yes
4423	3:**10 X** vs. 50:**0 X**	-3.410	-3.554 to -3.266	Yes
4424	3:**10 X** vs. 50:**1 X**	-3.313	-3.457 to -3.169	Yes
4425	3:**10 X** vs. 50:**10 X**	-3.308	-3.452 to -3.164	Yes
4426	3:**10 X** vs. 50:**40 X**	-0.6860	-0.8300 to -0.5420	Yes
4427	3:**10 X** vs. 50:**50 X**	-0.4327	-0.5766 to -0.2887	Yes
4428	3:**40 X** vs. 3:**50 X**	0.001000	-0.1430 to 0.1450	No
4429	3:**40 X** vs. 4:**0 X**	-0.0090	-0.1530 to 0.1350	No
4430	3:**40 X** vs. 4:**1 X**	-0.007667	-0.1516 to 0.1363	No
4431	3:**40 X** vs. 4:**10 X**	-0.02233	-0.1663 to 0.1216	No
4432	3:**40 X** vs. 4:**40 X**	-0.0020	-0.1460 to 0.1420	No
4433	3:**40 X** vs. 4:**50 X**	0.0	-0.1440 to 0.1440	No
4434	3:**40 X** vs. 5:**0 X**	-0.0100	-0.1540 to 0.1340	No
4435	3:**40 X** vs. 5:**1 X**	-0.008000	-0.1520 to 0.1360	No
4436	3:**40 X** vs. 5:**10 X**	-0.0230	-0.1670 to 0.1210	No
4437	3:**40 X** vs. 5:**40 X**	-0.0020	-0.1460 to 0.1420	No
4438	3:**40 X** vs. 5:**50 X**	-0.001000	-0.1450 to 0.1430	No
4439	3:**40 X** vs. 6:**0 X**	-0.009333	-0.1533 to 0.1346	No
4440	3:**40 X** vs. 6:**1 X**	-0.008333	-0.1523 to 0.1356	No
4441	3:**40 X** vs. 6:**10 X**	-0.0230	-0.1670 to 0.1210	No
4442	3:**40 X** vs. 6:**40 X**	-0.004333	-0.1483 to 0.1396	No
4443	3:**40 X** vs. 6:**50 X**	-0.002667	-0.1466 to 0.1413	No
4444	3:**40 X** vs. 7:**0 X**	-0.01133	-0.1553 to 0.1326	No
4445	3:**40 X** vs. 7:**1 X**	-0.002667	-0.1466 to 0.1413	No
4446	3:**40 X** vs. 7:**10 X**	-0.01467	-0.1586 to 0.1293	No
4447	3:**40 X** vs. 7:**40 X**	-0.0060	-0.1500 to 0.1380	No
4448	3:**40 X** vs. 7:**50 X**	-0.0040	-0.1480 to 0.1400	No
4449	3:**40 X** vs. 8:**0 X**	-0.0160	-0.1600 to 0.1280	No
4450	3:**40 X** vs. 8:**1 X**	-0.002667	-0.1466 to 0.1413	No
4451	3:**40 X** vs. 8:**10 X**	-0.01000	-0.1540 to 0.1340	No
4452	3:**40 X** vs. 8:**40 X**	-0.01067	-0.1546 to 0.1333	No
4453	3:**40 X** vs. 8:**50 X**	-0.007667	-0.1516 to 0.1363	No
4454	3:**40 X** vs. 9:**0 X**	-0.0240	-0.1680 to 0.1200	No
4455	3:**40 X** vs. 9:**1 X**	-0.0320	-0.1760 to 0.1120	No

	2way ANOVA<br>Multiple comparisons	A<br>Data Set-A<br>Y	B<br>Data Set-B<br>Y	C<br>Data Set-C<br>Y
4456	3:**40 X** vs. 9:**10 X**	-0.0330	-0.1770 to 0.1110	No
4457	3:**40 X** vs. 9:**40 X**	-0.01533	-0.1593 to 0.1286	No
4458	3:**40 X** vs. 9:**50 X**	-0.008667	-0.1526 to 0.1353	No
4459	3:**40 X** vs. 10:**0 X**	-0.02567	-0.1696 to 0.1183	No
4460	3:**40 X** vs. 10:**1 X**	-0.03267	-0.1766 to 0.1113	No
4461	3:**40 X** vs. 10:**10 X**	-0.03533	-0.1793 to 0.1086	No
4462	3:**40 X** vs. 10:**40 X**	-0.0250	-0.1690 to 0.1190	No
4463	3:**40 X** vs. 10:**50 X**	-0.01167	-0.1556 to 0.1323	No
4464	3:**40 X** vs. 11:**0 X**	-0.03533	-0.1793 to 0.1086	No
4465	3:**40 X** vs. 11:**1 X**	-0.0370	-0.1810 to 0.1070	No
4466	3:**40 X** vs. 11:**10 X**	-0.03733	-0.1813 to 0.1066	No
4467	3:**40 X** vs. 11:**40 X**	-0.0290	-0.1730 to 0.1150	No
4468	3:**40 X** vs. 11:**50 X**	-0.0170	-0.1610 to 0.1270	No
4469	3:**40 X** vs. 12:**0 X**	-0.0360	-0.1800 to 0.1080	No
4470	3:**40 X** vs. 12:**1 X**	-0.01833	-0.1623 to 0.1256	No
4471	3:**40 X** vs. 12:**10 X**	-0.0360	-0.1800 to 0.1080	No
4472	3:**40 X** vs. 12:**40 X**	-0.02233	-0.1663 to 0.1216	No
4473	3:**40 X** vs. 12:**50 X**	-0.02567	-0.1696 to 0.1183	No
4474	3:**40 X** vs. 13:**0 X**	-0.0300	-0.1740 to 0.1140	No
4475	3:**40 X** vs. 13:**1 X**	-0.01733	-0.1613 to 0.1266	No
4476	3:**40 X** vs. 13:**10 X**	-0.03933	-0.1833 to 0.1046	No
4477	3:**40 X** vs. 13:**40 X**	-0.03567	-0.1796 to 0.1083	No
4478	3:**40 X** vs. 13:**50 X**	-0.03667	-0.1806 to 0.1073	No
4479	3:**40 X** vs. 14:**0 X**	-0.03867	-0.1826 to 0.1053	No
4480	3:**40 X** vs. 14:**1 X**	-0.03033	-0.1743 to 0.1136	No
4481	3:**40 X** vs. 14:**10 X**	-0.04433	-0.1883 to 0.09963	No
4482	3:**40 X** vs. 14:**40 X**	-0.0410	-0.1850 to 0.1030	No
4483	3:**40 X** vs. 14:**50 X**	-0.0450	-0.1890 to 0.09896	No
4484	3:**40 X** vs. 15:**0 X**	-0.0510	-0.1950 to 0.09296	No
4485	3:**40 X** vs. 15:**1 X**	-0.0310	-0.1750 to 0.1130	No
4486	3:**40 X** vs. 15:**10 X**	-0.05833	-0.2023 to 0.08563	No
4487	3:**40 X** vs. 15:**40 X**	-0.04067	-0.1846 to 0.1033	No
4488	3:**40 X** vs. 15:**50 X**	-0.05833	-0.2023 to 0.08563	No
4489	3:**40 X** vs. 16:**0 X**	-0.1433	-0.2873 to 0.0006265	No
4490	3:**40 X** vs. 16:**1 X**	-0.03433	-0.1783 to 0.1096	No
4491	3:**40 X** vs. 16:**10 X**	-0.0600	-0.2040 to 0.08396	No
4492	3:**40 X** vs. 16:**40 X**	-0.07933	-0.2233 to 0.06463	No
4493	3:**40 X** vs. 16:**50 X**	-0.1217	-0.2656 to 0.02229	No
4494	3:**40 X** vs. 17:**0 X**	-0.1960	-0.3400 to -0.05204	Yes
4495	3:**40 X** vs. 17:**1 X**	-0.1103	-0.2543 to 0.03363	No
4496	3:**40 X** vs. 17:**10 X**	-0.1670	-0.3110 to -0.02304	Yes
4497	3:**40 X** vs. 17:**40 X**	-0.09033	-0.2343 to 0.05363	No
4498	3:**40 X** vs. 17:**50 X**	-0.1357	-0.2796 to 0.008293	No
4499	3:**40 X** vs. 18:**0 X**	-0.2447	-0.3886 to -0.1007	Yes
4500	3:**40 X** vs. 18:**1 X**	-0.1530	-0.2970 to -0.009040	Yes

	2way ANOVA<br>Multiple comparisons	A<br>Data Set-A<br>Y	B<br>Data Set-B<br>Y	C<br>Data Set-C<br>Y
4501	3:**40 X** vs. 18:**10 X**	-0.2453	-0.3893 to -0.1014	Yes
4502	3:**40 X** vs. 18:**40 X**	-0.09467	-0.2386 to 0.04929	No
4503	3:**40 X** vs. 18:**50 X**	-0.1397	-0.2836 to 0.004293	No
4504	3:**40 X** vs. 19:**0 X**	-0.2797	-0.4236 to -0.1357	Yes
4505	3:**40 X** vs. 19:**1 X**	-0.2677	-0.4116 to -0.1237	Yes
4506	3:**40 X** vs. 19:**10 X**	-0.2653	-0.4093 to -0.1214	Yes
4507	3:**40 X** vs. 19:**40 X**	-0.1233	-0.2673 to 0.02063	No
4508	3:**40 X** vs. 19:**50 X**	-0.1343	-0.2783 to 0.009626	No
4509	3:**40 X** vs. 20:**0 X**	-0.3593	-0.5033 to -0.2154	Yes
4510	3:**40 X** vs. 20:**1 X**	-0.1787	-0.3226 to -0.03471	Yes
4511	3:**40 X** vs. 20:**10 X**	-0.2677	-0.4116 to -0.1237	Yes
4512	3:**40 X** vs. 20:**40 X**	-0.1350	-0.2790 to 0.008960	No
4513	3:**40 X** vs. 20:**50 X**	-0.1790	-0.3230 to -0.03504	Yes
4514	3:**40 X** vs. 21:**0 X**	-0.3827	-0.5266 to -0.2387	Yes
4515	3:**40 X** vs. 21:**1 X**	-0.3357	-0.4796 to -0.1917	Yes
4516	3:**40 X** vs. 21:**10 X**	-0.3327	-0.4766 to -0.1887	Yes
4517	3:**40 X** vs. 21:**40 X**	-0.2080	-0.3520 to -0.06404	Yes
4518	3:**40 X** vs. 21:**50 X**	-0.2983	-0.4423 to -0.1544	Yes
4519	3:**40 X** vs. 22:**0 X**	-0.4243	-0.5683 to -0.2804	Yes
4520	3:**40 X** vs. 22:**1 X**	-0.4190	-0.5630 to -0.2750	Yes
4521	3:**40 X** vs. 22:**10 X**	-0.4143	-0.5583 to -0.2704	Yes
4522	3:**40 X** vs. 22:**40 X**	-0.3147	-0.4586 to -0.1707	Yes
4523	3:**40 X** vs. 22:**50 X**	-0.3773	-0.5213 to -0.2334	Yes
4524	3:**40 X** vs. 23:**0 X**	-0.4897	-0.6336 to -0.3457	Yes
4525	3:**40 X** vs. 23:**1 X**	-0.6793	-0.8403 to -0.5184	Yes
4526	3:**40 X** vs. 23:**10 X**	-0.4290	-0.5730 to -0.2850	Yes
4527	3:**40 X** vs. 23:**40 X**	-0.3093	-0.4703 to -0.1484	Yes
4528	3:**40 X** vs. 23:**50 X**	-0.3433	-0.4873 to -0.1994	Yes
4529	3:**40 X** vs. 24:**0 X**	-0.6070	-0.7510 to -0.4630	Yes
4530	3:**40 X** vs. 24:**1 X**	-0.7633	-0.9243 to -0.6024	Yes
4531	3:**40 X** vs. 24:**10 X**	-0.5908	-0.7518 to -0.4299	Yes
4532	3:**40 X** vs. 24:**40 X**	-0.3257	-0.4696 to -0.1817	Yes
4533	3:**40 X** vs. 24:**50 X**	-0.2473	-0.3913 to -0.1034	Yes
4534	3:**40 X** vs. 25:**0 X**	-0.9443	-1.105 to -0.7834	Yes
4535	3:**40 X** vs. 25:**1 X**	-1.134	-1.295 to -0.9729	Yes
4536	3:**40 X** vs. 25:**10 X**	-0.4953	-0.6989 to -0.2917	Yes
4537	3:**40 X** vs. 25:**40 X**	-0.3277	-0.4716 to -0.1837	Yes
4538	3:**40 X** vs. 25:**50 X**	-0.3020	-0.4460 to -0.1580	Yes
4539	3:**40 X** vs. 26:**0 X**	-1.157	-1.318 to -0.9959	Yes
4540	3:**40 X** vs. 26:**1 X**	-1.270	-1.431 to -1.109	Yes
4541	3:**40 X** vs. 26:**10 X**	-0.9563	-1.117 to -0.7954	Yes
4542	3:**40 X** vs. 26:**40 X**	-0.4020	-0.5460 to -0.2580	Yes
4543	3:**40 X** vs. 26:**50 X**	-0.2623	-0.4063 to -0.1184	Yes
4544	3:**40 X** vs. 27:**0 X**	-1.345	-1.489 to -1.201	Yes
4545	3:**40 X** vs. 27:**1 X**	-1.435	-1.596 to -1.274	Yes

	2way ANOVA<br>Multiple comparisons	A<br>Data Set-A<br>Y	B<br>Data Set-B<br>Y	C<br>Data Set-C<br>Y
4546	3:40 X vs. 27:10 X	-1.247	-1.408 to -1.086	Yes
4547	3:40 X vs. 27:40 X	-0.4020	-0.5460 to -0.2580	Yes
4548	3:40 X vs. 27:50 X	-0.2617	-0.4056 to -0.1177	Yes
4549	3:40 X vs. 28:0 X	-1.294	-1.455 to -1.133	Yes
4550	3:40 X vs. 28:1 X	-1.335	-1.479 to -1.191	Yes
4551	3:40 X vs. 28:10 X	-1.136	-1.297 to -0.9754	Yes
4552	3:40 X vs. 28:40 X	-0.4143	-0.5583 to -0.2704	Yes
4553	3:40 X vs. 28:50 X	-0.3097	-0.4536 to -0.1657	Yes
4554	3:40 X vs. 29:0 X	-1.775	-1.936 to -1.614	Yes
4555	3:40 X vs. 29:1 X	-1.860	-2.021 to -1.699	Yes
4556	3:40 X vs. 29:10 X	-1.075	-1.236 to -0.9139	Yes
4557	3:40 X vs. 29:40 X	-0.5223	-0.6663 to -0.3784	Yes
4558	3:40 X vs. 29:50 X	-0.3110	-0.4550 to -0.1670	Yes
4559	3:40 X vs. 30:0 X	-1.490	-1.651 to -1.329	Yes
4560	3:40 X vs. 30:1 X	-1.811	-1.972 to -1.650	Yes
4561	3:40 X vs. 30:10 X	-1.149	-1.310 to -0.9884	Yes
4562	3:40 X vs. 30:40 X	-0.4493	-0.5933 to -0.3054	Yes
4563	3:40 X vs. 30:50 X	-0.3633	-0.5073 to -0.2194	Yes
4564	3:40 X vs. 31:0 X	-1.808	-1.969 to -1.647	Yes
4565	3:40 X vs. 31:1 X	-1.798	-1.959 to -1.637	Yes
4566	3:40 X vs. 31:10 X	-1.281	-1.442 to -1.120	Yes
4567	3:40 X vs. 31:40 X	-0.5410	-0.6850 to -0.3970	Yes
4568	3:40 X vs. 31:50 X	-0.3423	-0.4863 to -0.1984	Yes
4569	3:40 X vs. 32:0 X	-2.399	-2.543 to -2.255	Yes
4570	3:40 X vs. 32:1 X	-1.855	-2.016 to -1.694	Yes
4571	3:40 X vs. 32:10 X	-1.711	-1.855 to -1.567	Yes
4572	3:40 X vs. 32:40 X	-0.5373	-0.6813 to -0.3934	Yes
4573	3:40 X vs. 32:50 X	-0.3420	-0.4860 to -0.1980	Yes
4574	3:40 X vs. 33:0 X	-2.403	-2.547 to -2.259	Yes
4575	3:40 X vs. 33:1 X	-2.218	-2.362 to -2.074	Yes
4576	3:40 X vs. 33:10 X	-1.878	-2.022 to -1.734	Yes
4577	3:40 X vs. 33:40 X	-0.5560	-0.7000 to -0.4120	Yes
4578	3:40 X vs. 33:50 X	-0.3570	-0.5010 to -0.2130	Yes
4579	3:40 X vs. 34:0 X	-2.328	-2.472 to -2.184	Yes
4580	3:40 X vs. 34:1 X	-2.335	-2.479 to -2.191	Yes
4581	3:40 X vs. 34:10 X	-1.968	-2.112 to -1.824	Yes
4582	3:40 X vs. 34:40 X	-0.5680	-0.7120 to -0.4240	Yes
4583	3:40 X vs. 34:50 X	-0.5060	-0.6500 to -0.3620	Yes
4584	3:40 X vs. 35:0 X	-2.676	-2.820 to -2.532	Yes
4585	3:40 X vs. 35:1 X	-2.442	-2.586 to -2.298	Yes
4586	3:40 X vs. 35:10 X	-1.930	-2.074 to -1.786	Yes
4587	3:40 X vs. 35:40 X	-0.5770	-0.7210 to -0.4330	Yes
4588	3:40 X vs. 35:50 X	-0.5187	-0.6626 to -0.3747	Yes
4589	3:40 X vs. 36:0 X	-2.883	-3.044 to -2.722	Yes
4590	3:40 X vs. 36:1 X	-2.579	-2.723 to -2.435	Yes

	2way ANOVA<br>Multiple comparisons	A<br>Data Set-A<br>Y	B<br>Data Set-B<br>Y	C<br>Data Set-C<br>Y
**4591**	3:**40 X** vs. 36:**10 X**	-1.936	-2.080 to -1.792	Yes
**4592**	3:**40 X** vs. 36:**40 X**	-0.6323	-0.7763 to -0.4884	Yes
**4593**	3:**40 X** vs. 36:**50 X**	-0.4820	-0.6260 to -0.3380	Yes
**4594**	3:**40 X** vs. 37:**0 X**	-3.267	-3.411 to -3.123	Yes
**4595**	3:**40 X** vs. 37:**1 X**	-2.649	-2.793 to -2.505	Yes
**4596**	3:**40 X** vs. 37:**10 X**	-1.937	-2.081 to -1.793	Yes
**4597**	3:**40 X** vs. 37:**40 X**	-0.6697	-0.8136 to -0.5257	Yes
**4598**	3:**40 X** vs. 37:**50 X**	-0.4907	-0.6346 to -0.3467	Yes
**4599**	3:**40 X** vs. 38:**0 X**	-3.212	-3.356 to -3.068	Yes
**4600**	3:**40 X** vs. 38:**1 X**	-2.767	-2.911 to -2.623	Yes
**4601**	3:**40 X** vs. 38:**10 X**	-2.213	-2.357 to -2.069	Yes
**4602**	3:**40 X** vs. 38:**40 X**	-0.6740	-0.8180 to -0.5300	Yes
**4603**	3:**40 X** vs. 38:**50 X**	-0.4947	-0.6386 to -0.3507	Yes
**4604**	3:**40 X** vs. 39:**0 X**	-3.442	-3.586 to -3.298	Yes
**4605**	3:**40 X** vs. 39:**1 X**	-3.334	-3.478 to -3.190	Yes
**4606**	3:**40 X** vs. 39:**10 X**	-2.358	-2.502 to -2.214	Yes
**4607**	3:**40 X** vs. 39:**40 X**	-0.6800	-0.8240 to -0.5360	Yes
**4608**	3:**40 X** vs. 39:**50 X**	-0.5257	-0.6696 to -0.3817	Yes
**4609**	3:**40 X** vs. 40:**0 X**	-3.483	-3.627 to -3.339	Yes
**4610**	3:**40 X** vs. 40:**1 X**	-3.409	-3.553 to -3.265	Yes
**4611**	3:**40 X** vs. 40:**10 X**	-2.614	-2.758 to -2.470	Yes
**4612**	3:**40 X** vs. 40:**40 X**	-0.6847	-0.8286 to -0.5407	Yes
**4613**	3:**40 X** vs. 40:**50 X**	-0.5320	-0.6760 to -0.3880	Yes
**4614**	3:**40 X** vs. 41:**0 X**	-3.524	-3.668 to -3.380	Yes
**4615**	3:**40 X** vs. 41:**1 X**	-3.523	-3.667 to -3.379	Yes
**4616**	3:**40 X** vs. 41:**10 X**	-2.668	-2.812 to -2.524	Yes
**4617**	3:**40 X** vs. 41:**40 X**	-0.6320	-0.7760 to -0.4880	Yes
**4618**	3:**40 X** vs. 41:**50 X**	-0.5447	-0.6886 to -0.4007	Yes
**4619**	3:**40 X** vs. 42:**0 X**	-3.666	-3.810 to -3.522	Yes
**4620**	3:**40 X** vs. 42:**1 X**	-3.686	-3.830 to -3.542	Yes
**4621**	3:**40 X** vs. 42:**10 X**	-2.668	-2.812 to -2.524	Yes
**4622**	3:**40 X** vs. 42:**40 X**	-0.6447	-0.7886 to -0.5007	Yes
**4623**	3:**40 X** vs. 42:**50 X**	-0.5480	-0.6920 to -0.4040	Yes
**4624**	3:**40 X** vs. 43:**0 X**	-3.756	-3.900 to -3.612	Yes
**4625**	3:**40 X** vs. 43:**1 X**	-3.670	-3.814 to -3.526	Yes
**4626**	3:**40 X** vs. 43:**10 X**	-3.386	-3.530 to -3.242	Yes
**4627**	3:**40 X** vs. 43:**40 X**	-0.6430	-0.7870 to -0.4990	Yes
**4628**	3:**40 X** vs. 43:**50 X**	-0.5483	-0.6923 to -0.4044	Yes
**4629**	3:**40 X** vs. 44:**0 X**	-3.780	-3.924 to -3.636	Yes
**4630**	3:**40 X** vs. 44:**1 X**	-3.670	-3.814 to -3.526	Yes
**4631**	3:**40 X** vs. 44:**10 X**	-3.449	-3.593 to -3.305	Yes
**4632**	3:**40 X** vs. 44:**40 X**	-0.6450	-0.7890 to -0.5010	Yes
**4633**	3:**40 X** vs. 44:**50 X**	-0.4807	-0.6246 to -0.3367	Yes
**4634**	3:**40 X** vs. 45:**0 X**	-3.645	-3.789 to -3.501	Yes
**4635**	3:**40 X** vs. 45:**1 X**	-3.447	-3.591 to -3.303	Yes

2way ANOVA Multiple comparisons		A Data Set-A Y	B Data Set-B Y	C Data Set-C Y
4636	3:40 X vs. 45:10 X	-3.329	-3.473 to -3.185	Yes
4637	3:40 X vs. 45:40 X	-0.6463	-0.7903 to -0.5024	Yes
4638	3:40 X vs. 45:50 X	-0.4777	-0.6216 to -0.3337	Yes
4639	3:40 X vs. 46:0 X	-3.449	-3.593 to -3.305	Yes
4640	3:40 X vs. 46:1 X	-3.416	-3.560 to -3.272	Yes
4641	3:40 X vs. 46:10 X	-3.324	-3.468 to -3.180	Yes
4642	3:40 X vs. 46:40 X	-0.6600	-0.8040 to -0.5160	Yes
4643	3:40 X vs. 46:50 X	-0.4707	-0.6146 to -0.3267	Yes
4644	3:40 X vs. 47:0 X	-3.425	-3.569 to -3.281	Yes
4645	3:40 X vs. 47:1 X	-3.329	-3.473 to -3.185	Yes
4646	3:40 X vs. 47:10 X	-3.310	-3.454 to -3.166	Yes
4647	3:40 X vs. 47:40 X	-0.6700	-0.8140 to -0.5260	Yes
4648	3:40 X vs. 47:50 X	-0.4287	-0.5726 to -0.2847	Yes
4649	3:40 X vs. 48:0 X	-3.412	-3.573 to -3.251	Yes
4650	3:40 X vs. 48:1 X	-3.316	-3.460 to -3.172	Yes
4651	3:40 X vs. 48:10 X	-3.310	-3.454 to -3.166	Yes
4652	3:40 X vs. 48:40 X	-0.6763	-0.8203 to -0.5324	Yes
4653	3:40 X vs. 48:50 X	-0.4360	-0.5800 to -0.2920	Yes
4654	3:40 X vs. 49:0 X	-3.412	-3.556 to -3.268	Yes
4655	3:40 X vs. 49:1 X	-3.316	-3.460 to -3.172	Yes
4656	3:40 X vs. 49:10 X	-3.310	-3.454 to -3.166	Yes
4657	3:40 X vs. 49:40 X	-0.6807	-0.8246 to -0.5367	Yes
4658	3:40 X vs. 49:50 X	-0.4353	-0.5793 to -0.2914	Yes
4659	3:40 X vs. 50:0 X	-3.412	-3.556 to -3.268	Yes
4660	3:40 X vs. 50:1 X	-3.316	-3.460 to -3.172	Yes
4661	3:40 X vs. 50:10 X	-3.310	-3.454 to -3.166	Yes
4662	3:40 X vs. 50:40 X	-0.6883	-0.8323 to -0.5444	Yes
4663	3:40 X vs. 50:50 X	-0.4350	-0.5790 to -0.2910	Yes
4664	3:50 X vs. 4:0 X	-0.0100	-0.1540 to 0.1340	No
4665	3:50 X vs. 4:1 X	-0.008667	-0.1526 to 0.1353	No
4666	3:50 X vs. 4:10 X	-0.02333	-0.1673 to 0.1206	No
4667	3:50 X vs. 4:40 X	-0.0030	-0.1470 to 0.1410	No
4668	3:50 X vs. 4:50 X	-0.001000	-0.1450 to 0.1430	No
4669	3:50 X vs. 5:0 X	-0.0110	-0.1550 to 0.1330	No
4670	3:50 X vs. 5:1 X	-0.0090	-0.1530 to 0.1350	No
4671	3:50 X vs. 5:10 X	-0.0240	-0.1680 to 0.1200	No
4672	3:50 X vs. 5:40 X	-0.0030	-0.1470 to 0.1410	No
4673	3:50 X vs. 5:50 X	-0.0020	-0.1460 to 0.1420	No
4674	3:50 X vs. 6:0 X	-0.01033	-0.1543 to 0.1336	No
4675	3:50 X vs. 6:1 X	-0.009333	-0.1533 to 0.1346	No
4676	3:50 X vs. 6:10 X	-0.0240	-0.1680 to 0.1200	No
4677	3:50 X vs. 6:40 X	-0.005333	-0.1493 to 0.1386	No
4678	3:50 X vs. 6:50 X	-0.003667	-0.1476 to 0.1403	No
4679	3:50 X vs. 7:0 X	-0.01233	-0.1563 to 0.1316	No
4680	3:50 X vs. 7:1 X	-0.003667	-0.1476 to 0.1403	No

	2way ANOVA<br>Multiple comparisons	A<br>Data Set-A<br>Y	B<br>Data Set-B<br>Y	C<br>Data Set-C<br>Y
4681	3:50 X vs. 7:10 X	-0.01567	-0.1596 to 0.1283	No
4682	3:50 X vs. 7:40 X	-0.0070	-0.1510 to 0.1370	No
4683	3:50 X vs. 7:50 X	-0.0050	-0.1490 to 0.1390	No
4684	3:50 X vs. 8:0 X	-0.0170	-0.1610 to 0.1270	No
4685	3:50 X vs. 8:1 X	-0.003667	-0.1476 to 0.1403	No
4686	3:50 X vs. 8:10 X	-0.0110	-0.1550 to 0.1330	No
4687	3:50 X vs. 8:40 X	-0.01167	-0.1556 to 0.1323	No
4688	3:50 X vs. 8:50 X	-0.008667	-0.1526 to 0.1353	No
4689	3:50 X vs. 9:0 X	-0.0250	-0.1690 to 0.1190	No
4690	3:50 X vs. 9:1 X	-0.0330	-0.1770 to 0.1110	No
4691	3:50 X vs. 9:10 X	-0.0340	-0.1780 to 0.1100	No
4692	3:50 X vs. 9:40 X	-0.01633	-0.1603 to 0.1276	No
4693	3:50 X vs. 9:50 X	-0.009667	-0.1536 to 0.1343	No
4694	3:50 X vs. 10:0 X	-0.02667	-0.1706 to 0.1173	No
4695	3:50 X vs. 10:1 X	-0.03367	-0.1776 to 0.1103	No
4696	3:50 X vs. 10:10 X	-0.03633	-0.1803 to 0.1076	No
4697	3:50 X vs. 10:40 X	-0.0260	-0.1700 to 0.1180	No
4698	3:50 X vs. 10:50 X	-0.01267	-0.1566 to 0.1313	No
4699	3:50 X vs. 11:0 X	-0.03633	-0.1803 to 0.1076	No
4700	3:50 X vs. 11:1 X	-0.0380	-0.1820 to 0.1060	No
4701	3:50 X vs. 11:10 X	-0.03833	-0.1823 to 0.1056	No
4702	3:50 X vs. 11:40 X	-0.0300	-0.1740 to 0.1140	No
4703	3:50 X vs. 11:50 X	-0.0180	-0.1620 to 0.1260	No
4704	3:50 X vs. 12:0 X	-0.0370	-0.1810 to 0.1070	No
4705	3:50 X vs. 12:1 X	-0.01933	-0.1633 to 0.1246	No
4706	3:50 X vs. 12:10 X	-0.0370	-0.1810 to 0.1070	No
4707	3:50 X vs. 12:40 X	-0.02333	-0.1673 to 0.1206	No
4708	3:50 X vs. 12:50 X	-0.02667	-0.1706 to 0.1173	No
4709	3:50 X vs. 13:0 X	-0.0310	-0.1750 to 0.1130	No
4710	3:50 X vs. 13:1 X	-0.01833	-0.1623 to 0.1256	No
4711	3:50 X vs. 13:10 X	-0.04033	-0.1843 to 0.1036	No
4712	3:50 X vs. 13:40 X	-0.03667	-0.1806 to 0.1073	No
4713	3:50 X vs. 13:50 X	-0.03767	-0.1816 to 0.1063	No
4714	3:50 X vs. 14:0 X	-0.03967	-0.1836 to 0.1043	No
4715	3:50 X vs. 14:1 X	-0.03133	-0.1753 to 0.1126	No
4716	3:50 X vs. 14:10 X	-0.04533	-0.1893 to 0.09863	No
4717	3:50 X vs. 14:40 X	-0.0420	-0.1860 to 0.1020	No
4718	3:50 X vs. 14:50 X	-0.0460	-0.1900 to 0.09796	No
4719	3:50 X vs. 15:0 X	-0.0520	-0.1960 to 0.09196	No
4720	3:50 X vs. 15:1 X	-0.0320	-0.1760 to 0.1120	No
4721	3:50 X vs. 15:10 X	-0.05933	-0.2033 to 0.08463	No
4722	3:50 X vs. 15:40 X	-0.04167	-0.1856 to 0.1023	No
4723	3:50 X vs. 15:50 X	-0.05933	-0.2033 to 0.08463	No
4724	3:50 X vs. 16:0 X	-0.1443	-0.2883 to -0.0003735	Yes
4725	3:50 X vs. 16:1 X	-0.03533	-0.1793 to 0.1086	No

	2way ANOVA<br>Multiple comparisons	A<br>Data Set-A<br>Y	B<br>Data Set-B<br>Y	C<br>Data Set-C<br>Y
4726	3:50 X vs. 16:10 X	-0.0610	-0.2050 to 0.08296	No
4727	3:50 X vs. 16:40 X	-0.08033	-0.2243 to 0.06363	No
4728	3:50 X vs. 16:50 X	-0.1227	-0.2666 to 0.02129	No
4729	3:50 X vs. 17:0 X	-0.1970	-0.3410 to -0.05304	Yes
4730	3:50 X vs. 17:1 X	-0.1113	-0.2553 to 0.03263	No
4731	3:50 X vs. 17:10 X	-0.1680	-0.3120 to -0.02404	Yes
4732	3:50 X vs. 17:40 X	-0.09133	-0.2353 to 0.05263	No
4733	3:50 X vs. 17:50 X	-0.1367	-0.2806 to 0.007293	No
4734	3:50 X vs. 18:0 X	-0.2457	-0.3896 to -0.1017	Yes
4735	3:50 X vs. 18:1 X	-0.1540	-0.2980 to -0.01004	Yes
4736	3:50 X vs. 18:10 X	-0.2463	-0.3903 to -0.1024	Yes
4737	3:50 X vs. 18:40 X	-0.09567	-0.2396 to 0.04829	No
4738	3:50 X vs. 18:50 X	-0.1407	-0.2846 to 0.003293	No
4739	3:50 X vs. 19:0 X	-0.2807	-0.4246 to -0.1367	Yes
4740	3:50 X vs. 19:1 X	-0.2687	-0.4126 to -0.1247	Yes
4741	3:50 X vs. 19:10 X	-0.2663	-0.4103 to -0.1224	Yes
4742	3:50 X vs. 19:40 X	-0.1243	-0.2683 to 0.01963	No
4743	3:50 X vs. 19:50 X	-0.1353	-0.2793 to 0.008626	No
4744	3:50 X vs. 20:0 X	-0.3603	-0.5043 to -0.2164	Yes
4745	3:50 X vs. 20:1 X	-0.1797	-0.3236 to -0.03571	Yes
4746	3:50 X vs. 20:10 X	-0.2687	-0.4126 to -0.1247	Yes
4747	3:50 X vs. 20:40 X	-0.1360	-0.2800 to 0.007960	No
4748	3:50 X vs. 20:50 X	-0.1800	-0.3240 to -0.03604	Yes
4749	3:50 X vs. 21:0 X	-0.3837	-0.5276 to -0.2397	Yes
4750	3:50 X vs. 21:1 X	-0.3367	-0.4806 to -0.1927	Yes
4751	3:50 X vs. 21:10 X	-0.3337	-0.4776 to -0.1897	Yes
4752	3:50 X vs. 21:40 X	-0.2090	-0.3530 to -0.06504	Yes
4753	3:50 X vs. 21:50 X	-0.2993	-0.4433 to -0.1554	Yes
4754	3:50 X vs. 22:0 X	-0.4253	-0.5693 to -0.2814	Yes
4755	3:50 X vs. 22:1 X	-0.4200	-0.5640 to -0.2760	Yes
4756	3:50 X vs. 22:10 X	-0.4153	-0.5593 to -0.2714	Yes
4757	3:50 X vs. 22:40 X	-0.3157	-0.4596 to -0.1717	Yes
4758	3:50 X vs. 22:50 X	-0.3783	-0.5223 to -0.2344	Yes
4759	3:50 X vs. 23:0 X	-0.4907	-0.6346 to -0.3467	Yes
4760	3:50 X vs. 23:1 X	-0.6803	-0.8413 to -0.5194	Yes
4761	3:50 X vs. 23:10 X	-0.4300	-0.5740 to -0.2860	Yes
4762	3:50 X vs. 23:40 X	-0.3103	-0.4713 to -0.1494	Yes
4763	3:50 X vs. 23:50 X	-0.3443	-0.4883 to -0.2004	Yes
4764	3:50 X vs. 24:0 X	-0.6080	-0.7520 to -0.4640	Yes
4765	3:50 X vs. 24:1 X	-0.7643	-0.9253 to -0.6034	Yes
4766	3:50 X vs. 24:10 X	-0.5918	-0.7528 to -0.4309	Yes
4767	3:50 X vs. 24:40 X	-0.3267	-0.4706 to -0.1827	Yes
4768	3:50 X vs. 24:50 X	-0.2483	-0.3923 to -0.1044	Yes
4769	3:50 X vs. 25:0 X	-0.9453	-1.106 to -0.7844	Yes
4770	3:50 X vs. 25:1 X	-1.135	-1.296 to -0.9739	Yes

	2way ANOVA<br>Multiple comparisons	A<br>Data Set-A<br>Y	B<br>Data Set-B<br>Y	C<br>Data Set-C<br>Y
**4771**	3:**50 X** vs. 25:**10 X**	-0.4963	-0.6999 to -0.2927	Yes
**4772**	3:**50 X** vs. 25:**40 X**	-0.3287	-0.4726 to -0.1847	Yes
**4773**	3:**50 X** vs. 25:**50 X**	-0.3030	-0.4470 to -0.1590	Yes
**4774**	3:**50 X** vs. 26:**0 X**	-1.158	-1.319 to -0.9969	Yes
**4775**	3:**50 X** vs. 26:**1 X**	-1.271	-1.432 to -1.110	Yes
**4776**	3:**50 X** vs. 26:**10 X**	-0.9573	-1.118 to -0.7964	Yes
**4777**	3:**50 X** vs. 26:**40 X**	-0.4030	-0.5470 to -0.2590	Yes
**4778**	3:**50 X** vs. 26:**50 X**	-0.2633	-0.4073 to -0.1194	Yes
**4779**	3:**50 X** vs. 27:**0 X**	-1.346	-1.490 to -1.202	Yes
**4780**	3:**50 X** vs. 27:**1 X**	-1.436	-1.597 to -1.275	Yes
**4781**	3:**50 X** vs. 27:**10 X**	-1.248	-1.409 to -1.087	Yes
**4782**	3:**50 X** vs. 27:**40 X**	-0.4030	-0.5470 to -0.2590	Yes
**4783**	3:**50 X** vs. 27:**50 X**	-0.2627	-0.4066 to -0.1187	Yes
**4784**	3:**50 X** vs. 28:**0 X**	-1.295	-1.456 to -1.134	Yes
**4785**	3:**50 X** vs. 28:**1 X**	-1.336	-1.480 to -1.192	Yes
**4786**	3:**50 X** vs. 28:**10 X**	-1.137	-1.298 to -0.9764	Yes
**4787**	3:**50 X** vs. 28:**40 X**	-0.4153	-0.5593 to -0.2714	Yes
**4788**	3:**50 X** vs. 28:**50 X**	-0.3107	-0.4546 to -0.1667	Yes
**4789**	3:**50 X** vs. 29:**0 X**	-1.776	-1.937 to -1.615	Yes
**4790**	3:**50 X** vs. 29:**1 X**	-1.861	-2.022 to -1.700	Yes
**4791**	3:**50 X** vs. 29:**10 X**	-1.076	-1.237 to -0.9149	Yes
**4792**	3:**50 X** vs. 29:**40 X**	-0.5233	-0.6673 to -0.3794	Yes
**4793**	3:**50 X** vs. 29:**50 X**	-0.3120	-0.4560 to -0.1680	Yes
**4794**	3:**50 X** vs. 30:**0 X**	-1.491	-1.652 to -1.330	Yes
**4795**	3:**50 X** vs. 30:**1 X**	-1.812	-1.973 to -1.651	Yes
**4796**	3:**50 X** vs. 30:**10 X**	-1.150	-1.311 to -0.9894	Yes
**4797**	3:**50 X** vs. 30:**40 X**	-0.4503	-0.5943 to -0.3064	Yes
**4798**	3:**50 X** vs. 30:**50 X**	-0.3643	-0.5083 to -0.2204	Yes
**4799**	3:**50 X** vs. 31:**0 X**	-1.809	-1.970 to -1.648	Yes
**4800**	3:**50 X** vs. 31:**1 X**	-1.799	-1.960 to -1.638	Yes
**4801**	3:**50 X** vs. 31:**10 X**	-1.282	-1.443 to -1.121	Yes
**4802**	3:**50 X** vs. 31:**40 X**	-0.5420	-0.6860 to -0.3980	Yes
**4803**	3:**50 X** vs. 31:**50 X**	-0.3433	-0.4873 to -0.1994	Yes
**4804**	3:**50 X** vs. 32:**0 X**	-2.400	-2.544 to -2.256	Yes
**4805**	3:**50 X** vs. 32:**1 X**	-1.856	-2.017 to -1.695	Yes
**4806**	3:**50 X** vs. 32:**10 X**	-1.712	-1.856 to -1.568	Yes
**4807**	3:**50 X** vs. 32:**40 X**	-0.5383	-0.6823 to -0.3944	Yes
**4808**	3:**50 X** vs. 32:**50 X**	-0.3430	-0.4870 to -0.1990	Yes
**4809**	3:**50 X** vs. 33:**0 X**	-2.404	-2.548 to -2.260	Yes
**4810**	3:**50 X** vs. 33:**1 X**	-2.219	-2.363 to -2.075	Yes
**4811**	3:**50 X** vs. 33:**10 X**	-1.879	-2.023 to -1.735	Yes
**4812**	3:**50 X** vs. 33:**40 X**	-0.5570	-0.7010 to -0.4130	Yes
**4813**	3:**50 X** vs. 33:**50 X**	-0.3580	-0.5020 to -0.2140	Yes
**4814**	3:**50 X** vs. 34:**0 X**	-2.329	-2.473 to -2.185	Yes
**4815**	3:**50 X** vs. 34:**1 X**	-2.336	-2.480 to -2.192	Yes

	2way ANOVA<br>Multiple comparisons	A<br>Data Set-A<br>Y	B<br>Data Set-B<br>Y	C<br>Data Set-C<br>Y
4816	3:50 X vs. 34:10 X	-1.969	-2.113 to -1.825	Yes
4817	3:50 X vs. 34:40 X	-0.5690	-0.7130 to -0.4250	Yes
4818	3:50 X vs. 34:50 X	-0.5070	-0.6510 to -0.3630	Yes
4819	3:50 X vs. 35:0 X	-2.677	-2.821 to -2.533	Yes
4820	3:50 X vs. 35:1 X	-2.443	-2.587 to -2.299	Yes
4821	3:50 X vs. 35:10 X	-1.931	-2.075 to -1.787	Yes
4822	3:50 X vs. 35:40 X	-0.5780	-0.7220 to -0.4340	Yes
4823	3:50 X vs. 35:50 X	-0.5197	-0.6636 to -0.3757	Yes
4824	3:50 X vs. 36:0 X	-2.884	-3.045 to -2.723	Yes
4825	3:50 X vs. 36:1 X	-2.580	-2.724 to -2.436	Yes
4826	3:50 X vs. 36:10 X	-1.937	-2.081 to -1.793	Yes
4827	3:50 X vs. 36:40 X	-0.6333	-0.7773 to -0.4894	Yes
4828	3:50 X vs. 36:50 X	-0.4830	-0.6270 to -0.3390	Yes
4829	3:50 X vs. 37:0 X	-3.268	-3.412 to -3.124	Yes
4830	3:50 X vs. 37:1 X	-2.650	-2.794 to -2.506	Yes
4831	3:50 X vs. 37:10 X	-1.938	-2.082 to -1.794	Yes
4832	3:50 X vs. 37:40 X	-0.6707	-0.8146 to -0.5267	Yes
4833	3:50 X vs. 37:50 X	-0.4917	-0.6356 to -0.3477	Yes
4834	3:50 X vs. 38:0 X	-3.213	-3.357 to -3.069	Yes
4835	3:50 X vs. 38:1 X	-2.768	-2.912 to -2.624	Yes
4836	3:50 X vs. 38:10 X	-2.214	-2.358 to -2.070	Yes
4837	3:50 X vs. 38:40 X	-0.6750	-0.8190 to -0.5310	Yes
4838	3:50 X vs. 38:50 X	-0.4957	-0.6396 to -0.3517	Yes
4839	3:50 X vs. 39:0 X	-3.443	-3.587 to -3.299	Yes
4840	3:50 X vs. 39:1 X	-3.335	-3.479 to -3.191	Yes
4841	3:50 X vs. 39:10 X	-2.359	-2.503 to -2.215	Yes
4842	3:50 X vs. 39:40 X	-0.6810	-0.8250 to -0.5370	Yes
4843	3:50 X vs. 39:50 X	-0.5267	-0.6706 to -0.3827	Yes
4844	3:50 X vs. 40:0 X	-3.484	-3.628 to -3.340	Yes
4845	3:50 X vs. 40:1 X	-3.410	-3.554 to -3.266	Yes
4846	3:50 X vs. 40:10 X	-2.615	-2.759 to -2.471	Yes
4847	3:50 X vs. 40:40 X	-0.6857	-0.8296 to -0.5417	Yes
4848	3:50 X vs. 40:50 X	-0.5330	-0.6770 to -0.3890	Yes
4849	3:50 X vs. 41:0 X	-3.525	-3.669 to -3.381	Yes
4850	3:50 X vs. 41:1 X	-3.524	-3.668 to -3.380	Yes
4851	3:50 X vs. 41:10 X	-2.669	-2.813 to -2.525	Yes
4852	3:50 X vs. 41:40 X	-0.6330	-0.7770 to -0.4890	Yes
4853	3:50 X vs. 41:50 X	-0.5457	-0.6896 to -0.4017	Yes
4854	3:50 X vs. 42:0 X	-3.667	-3.811 to -3.523	Yes
4855	3:50 X vs. 42:1 X	-3.687	-3.831 to -3.543	Yes
4856	3:50 X vs. 42:10 X	-2.669	-2.813 to -2.525	Yes
4857	3:50 X vs. 42:40 X	-0.6457	-0.7896 to -0.5017	Yes
4858	3:50 X vs. 42:50 X	-0.5490	-0.6930 to -0.4050	Yes
4859	3:50 X vs. 43:0 X	-3.757	-3.901 to -3.613	Yes
4860	3:50 X vs. 43:1 X	-3.671	-3.815 to -3.527	Yes

	2way ANOVA<br>Multiple comparisons	A<br>Data Set-A<br>Y	B<br>Data Set-B<br>Y	C<br>Data Set-C<br>Y
**4861**	3:**50 X** vs. 43:**10 X**	-3.387	-3.531 to -3.243	Yes
**4862**	3:**50 X** vs. 43:**40 X**	-0.6440	-0.7880 to -0.5000	Yes
**4863**	3:**50 X** vs. 43:**50 X**	-0.5493	-0.6933 to -0.4054	Yes
**4864**	3:**50 X** vs. 44:**0 X**	-3.781	-3.925 to -3.637	Yes
**4865**	3:**50 X** vs. 44:**1 X**	-3.671	-3.815 to -3.527	Yes
**4866**	3:**50 X** vs. 44:**10 X**	-3.450	-3.594 to -3.306	Yes
**4867**	3:**50 X** vs. 44:**40 X**	-0.6460	-0.7900 to -0.5020	Yes
**4868**	3:**50 X** vs. 44:**50 X**	-0.4817	-0.6256 to -0.3377	Yes
**4869**	3:**50 X** vs. 45:**0 X**	-3.646	-3.790 to -3.502	Yes
**4870**	3:**50 X** vs. 45:**1 X**	-3.448	-3.592 to -3.304	Yes
**4871**	3:**50 X** vs. 45:**10 X**	-3.330	-3.474 to -3.186	Yes
**4872**	3:**50 X** vs. 45:**40 X**	-0.6473	-0.7913 to -0.5034	Yes
**4873**	3:**50 X** vs. 45:**50 X**	-0.4787	-0.6226 to -0.3347	Yes
**4874**	3:**50 X** vs. 46:**0 X**	-3.450	-3.594 to -3.306	Yes
**4875**	3:**50 X** vs. 46:**1 X**	-3.417	-3.561 to -3.273	Yes
**4876**	3:**50 X** vs. 46:**10 X**	-3.325	-3.469 to -3.181	Yes
**4877**	3:**50 X** vs. 46:**40 X**	-0.6610	-0.8050 to -0.5170	Yes
**4878**	3:**50 X** vs. 46:**50 X**	-0.4717	-0.6156 to -0.3277	Yes
**4879**	3:**50 X** vs. 47:**0 X**	-3.426	-3.570 to -3.282	Yes
**4880**	3:**50 X** vs. 47:**1 X**	-3.330	-3.474 to -3.186	Yes
**4881**	3:**50 X** vs. 47:**10 X**	-3.311	-3.455 to -3.167	Yes
**4882**	3:**50 X** vs. 47:**40 X**	-0.6710	-0.8150 to -0.5270	Yes
**4883**	3:**50 X** vs. 47:**50 X**	-0.4297	-0.5736 to -0.2857	Yes
**4884**	3:**50 X** vs. 48:**0 X**	-3.413	-3.574 to -3.252	Yes
**4885**	3:**50 X** vs. 48:**1 X**	-3.317	-3.461 to -3.173	Yes
**4886**	3:**50 X** vs. 48:**10 X**	-3.311	-3.455 to -3.167	Yes
**4887**	3:**50 X** vs. 48:**40 X**	-0.6773	-0.8213 to -0.5334	Yes
**4888**	3:**50 X** vs. 48:**50 X**	-0.4370	-0.5810 to -0.2930	Yes
**4889**	3:**50 X** vs. 49:**0 X**	-3.413	-3.557 to -3.269	Yes
**4890**	3:**50 X** vs. 49:**1 X**	-3.317	-3.461 to -3.173	Yes
**4891**	3:**50 X** vs. 49:**10 X**	-3.311	-3.455 to -3.167	Yes
**4892**	3:**50 X** vs. 49:**40 X**	-0.6817	-0.8256 to -0.5377	Yes
**4893**	3:**50 X** vs. 49:**50 X**	-0.4363	-0.5803 to -0.2924	Yes
**4894**	3:**50 X** vs. 50:**0 X**	-3.413	-3.557 to -3.269	Yes
**4895**	3:**50 X** vs. 50:**1 X**	-3.317	-3.461 to -3.173	Yes
**4896**	3:**50 X** vs. 50:**10 X**	-3.311	-3.455 to -3.167	Yes
**4897**	3:**50 X** vs. 50:**40 X**	-0.6893	-0.8333 to -0.5454	Yes
**4898**	3:**50 X** vs. 50:**50 X**	-0.4360	-0.5800 to -0.2920	Yes
**4899**	4:**0 X** vs. 4:**1 X**	0.001333	-0.1426 to 0.1453	No
**4900**	4:**0 X** vs. 4:**10 X**	-0.01333	-0.1573 to 0.1306	No
**4901**	4:**0 X** vs. 4:**40 X**	0.0070	-0.1370 to 0.1510	No
**4902**	4:**0 X** vs. 4:**50 X**	0.0090	-0.1350 to 0.1530	No
**4903**	4:**0 X** vs. 5:**0 X**	-0.0010	-0.1450 to 0.1430	No
**4904**	4:**0 X** vs. 5:**1 X**	0.0010	-0.1430 to 0.1450	No
**4905**	4:**0 X** vs. 5:**10 X**	-0.0140	-0.1580 to 0.1300	No

	2way ANOVA<br>Multiple comparisons	A<br>Data Set-A<br>Y	B<br>Data Set-B<br>Y	C<br>Data Set-C<br>Y
4906	4:0 X vs. 5:40 X	0.0070	-0.1370 to 0.1510	No
4907	4:0 X vs. 5:50 X	0.008000	-0.1360 to 0.1520	No
4908	4:0 X vs. 6:0 X	-0.0003333	-0.1443 to 0.1436	No
4909	4:0 X vs. 6:1 X	0.0006667	-0.1433 to 0.1446	No
4910	4:0 X vs. 6:10 X	-0.0140	-0.1580 to 0.1300	No
4911	4:0 X vs. 6:40 X	0.004667	-0.1393 to 0.1486	No
4912	4:0 X vs. 6:50 X	0.006333	-0.1376 to 0.1503	No
4913	4:0 X vs. 7:0 X	-0.002333	-0.1463 to 0.1416	No
4914	4:0 X vs. 7:1 X	0.006333	-0.1376 to 0.1503	No
4915	4:0 X vs. 7:10 X	-0.005667	-0.1496 to 0.1383	No
4916	4:0 X vs. 7:40 X	0.0030	-0.1410 to 0.1470	No
4917	4:0 X vs. 7:50 X	0.005000	-0.1390 to 0.1490	No
4918	4:0 X vs. 8:0 X	-0.007000	-0.1510 to 0.1370	No
4919	4:0 X vs. 8:1 X	0.006333	-0.1376 to 0.1503	No
4920	4:0 X vs. 8:10 X	-0.001000	-0.1450 to 0.1430	No
4921	4:0 X vs. 8:40 X	-0.001667	-0.1456 to 0.1423	No
4922	4:0 X vs. 8:50 X	0.001333	-0.1426 to 0.1453	No
4923	4:0 X vs. 9:0 X	-0.0150	-0.1590 to 0.1290	No
4924	4:0 X vs. 9:1 X	-0.0230	-0.1670 to 0.1210	No
4925	4:0 X vs. 9:10 X	-0.0240	-0.1680 to 0.1200	No
4926	4:0 X vs. 9:40 X	-0.006333	-0.1503 to 0.1376	No
4927	4:0 X vs. 9:50 X	0.0003333	-0.1436 to 0.1443	No
4928	4:0 X vs. 10:0 X	-0.01667	-0.1606 to 0.1273	No
4929	4:0 X vs. 10:1 X	-0.02367	-0.1676 to 0.1203	No
4930	4:0 X vs. 10:10 X	-0.02633	-0.1703 to 0.1176	No
4931	4:0 X vs. 10:40 X	-0.0160	-0.1600 to 0.1280	No
4932	4:0 X vs. 10:50 X	-0.002667	-0.1466 to 0.1413	No
4933	4:0 X vs. 11:0 X	-0.02633	-0.1703 to 0.1176	No
4934	4:0 X vs. 11:1 X	-0.0280	-0.1720 to 0.1160	No
4935	4:0 X vs. 11:10 X	-0.02833	-0.1723 to 0.1156	No
4936	4:0 X vs. 11:40 X	-0.0200	-0.1640 to 0.1240	No
4937	4:0 X vs. 11:50 X	-0.0080	-0.1520 to 0.1360	No
4938	4:0 X vs. 12:0 X	-0.0270	-0.1710 to 0.1170	No
4939	4:0 X vs. 12:1 X	-0.009333	-0.1533 to 0.1346	No
4940	4:0 X vs. 12:10 X	-0.0270	-0.1710 to 0.1170	No
4941	4:0 X vs. 12:40 X	-0.01333	-0.1573 to 0.1306	No
4942	4:0 X vs. 12:50 X	-0.01667	-0.1606 to 0.1273	No
4943	4:0 X vs. 13:0 X	-0.0210	-0.1650 to 0.1230	No
4944	4:0 X vs. 13:1 X	-0.008333	-0.1523 to 0.1356	No
4945	4:0 X vs. 13:10 X	-0.03033	-0.1743 to 0.1136	No
4946	4:0 X vs. 13:40 X	-0.02667	-0.1706 to 0.1173	No
4947	4:0 X vs. 13:50 X	-0.02767	-0.1716 to 0.1163	No
4948	4:0 X vs. 14:0 X	-0.02967	-0.1736 to 0.1143	No
4949	4:0 X vs. 14:1 X	-0.02133	-0.1653 to 0.1226	No
4950	4:0 X vs. 14:10 X	-0.03533	-0.1793 to 0.1086	No

	2way ANOVA<br>Multiple comparisons	A<br>Data Set-A<br>Y	B<br>Data Set-B<br>Y	C<br>Data Set-C<br>Y
4951	4:0 X vs. 14:40 X	-0.0320	-0.1760 to 0.1120	No
4952	4:0 X vs. 14:50 X	-0.0360	-0.1800 to 0.1080	No
4953	4:0 X vs. 15:0 X	-0.0420	-0.1860 to 0.1020	No
4954	4:0 X vs. 15:1 X	-0.0220	-0.1660 to 0.1220	No
4955	4:0 X vs. 15:10 X	-0.04933	-0.1933 to 0.09463	No
4956	4:0 X vs. 15:40 X	-0.03167	-0.1756 to 0.1123	No
4957	4:0 X vs. 15:50 X	-0.04933	-0.1933 to 0.09463	No
4958	4:0 X vs. 16:0 X	-0.1343	-0.2783 to 0.009626	No
4959	4:0 X vs. 16:1 X	-0.02533	-0.1693 to 0.1186	No
4960	4:0 X vs. 16:10 X	-0.0510	-0.1950 to 0.09296	No
4961	4:0 X vs. 16:40 X	-0.07033	-0.2143 to 0.07363	No
4962	4:0 X vs. 16:50 X	-0.1127	-0.2566 to 0.03129	No
4963	4:0 X vs. 17:0 X	-0.1870	-0.3310 to -0.04304	Yes
4964	4:0 X vs. 17:1 X	-0.1013	-0.2453 to 0.04263	No
4965	4:0 X vs. 17:10 X	-0.1580	-0.3020 to -0.01404	Yes
4966	4:0 X vs. 17:40 X	-0.08133	-0.2253 to 0.06263	No
4967	4:0 X vs. 17:50 X	-0.1267	-0.2706 to 0.01729	No
4968	4:0 X vs. 18:0 X	-0.2357	-0.3796 to -0.09171	Yes
4969	4:0 X vs. 18:1 X	-0.1440	-0.2880 to -4.018e-005	Yes
4970	4:0 X vs. 18:10 X	-0.2363	-0.3803 to -0.09237	Yes
4971	4:0 X vs. 18:40 X	-0.08567	-0.2296 to 0.05829	No
4972	4:0 X vs. 18:50 X	-0.1307	-0.2746 to 0.01329	No
4973	4:0 X vs. 19:0 X	-0.2707	-0.4146 to -0.1267	Yes
4974	4:0 X vs. 19:1 X	-0.2587	-0.4026 to -0.1147	Yes
4975	4:0 X vs. 19:10 X	-0.2563	-0.4003 to -0.1124	Yes
4976	4:0 X vs. 19:40 X	-0.1143	-0.2583 to 0.02963	No
4977	4:0 X vs. 19:50 X	-0.1253	-0.2693 to 0.01863	No
4978	4:0 X vs. 20:0 X	-0.3503	-0.4943 to -0.2064	Yes
4979	4:0 X vs. 20:1 X	-0.1697	-0.3136 to -0.02571	Yes
4980	4:0 X vs. 20:10 X	-0.2587	-0.4026 to -0.1147	Yes
4981	4:0 X vs. 20:40 X	-0.1260	-0.2700 to 0.01796	No
4982	4:0 X vs. 20:50 X	-0.1700	-0.3140 to -0.02604	Yes
4983	4:0 X vs. 21:0 X	-0.3737	-0.5176 to -0.2297	Yes
4984	4:0 X vs. 21:1 X	-0.3267	-0.4706 to -0.1827	Yes
4985	4:0 X vs. 21:10 X	-0.3237	-0.4676 to -0.1797	Yes
4986	4:0 X vs. 21:40 X	-0.1990	-0.3430 to -0.05504	Yes
4987	4:0 X vs. 21:50 X	-0.2893	-0.4333 to -0.1454	Yes
4988	4:0 X vs. 22:0 X	-0.4153	-0.5593 to -0.2714	Yes
4989	4:0 X vs. 22:1 X	-0.4100	-0.5540 to -0.2660	Yes
4990	4:0 X vs. 22:10 X	-0.4053	-0.5493 to -0.2614	Yes
4991	4:0 X vs. 22:40 X	-0.3057	-0.4496 to -0.1617	Yes
4992	4:0 X vs. 22:50 X	-0.3683	-0.5123 to -0.2244	Yes
4993	4:0 X vs. 23:0 X	-0.4807	-0.6246 to -0.3367	Yes
4994	4:0 X vs. 23:1 X	-0.6703	-0.8313 to -0.5094	Yes
4995	4:0 X vs. 23:10 X	-0.4200	-0.5640 to -0.2760	Yes

	2way ANOVA<br>Multiple comparisons	A<br>Data Set-A<br>Y	B<br>Data Set-B<br>Y	C<br>Data Set-C<br>Y
4996	4:0 X vs. 23:40 X	-0.3003	-0.4613 to -0.1394	Yes
4997	4:0 X vs. 23:50 X	-0.3343	-0.4783 to -0.1904	Yes
4998	4:0 X vs. 24:0 X	-0.5980	-0.7420 to -0.4540	Yes
4999	4:0 X vs. 24:1 X	-0.7543	-0.9153 to -0.5934	Yes
5000	4:0 X vs. 24:10 X	-0.5818	-0.7428 to -0.4209	Yes
5001	4:0 X vs. 24:40 X	-0.3167	-0.4606 to -0.1727	Yes
5002	4:0 X vs. 24:50 X	-0.2383	-0.3823 to -0.09437	Yes
5003	4:0 X vs. 25:0 X	-0.9353	-1.096 to -0.7744	Yes
5004	4:0 X vs. 25:1 X	-1.125	-1.286 to -0.9639	Yes
5005	4:0 X vs. 25:10 X	-0.4863	-0.6899 to -0.2827	Yes
5006	4:0 X vs. 25:40 X	-0.3187	-0.4626 to -0.1747	Yes
5007	4:0 X vs. 25:50 X	-0.2930	-0.4370 to -0.1490	Yes
5008	4:0 X vs. 26:0 X	-1.148	-1.309 to -0.9869	Yes
5009	4:0 X vs. 26:1 X	-1.261	-1.422 to -1.100	Yes
5010	4:0 X vs. 26:10 X	-0.9473	-1.108 to -0.7864	Yes
5011	4:0 X vs. 26:40 X	-0.3930	-0.5370 to -0.2490	Yes
5012	4:0 X vs. 26:50 X	-0.2533	-0.3973 to -0.1094	Yes
5013	4:0 X vs. 27:0 X	-1.336	-1.480 to -1.192	Yes
5014	4:0 X vs. 27:1 X	-1.426	-1.587 to -1.265	Yes
5015	4:0 X vs. 27:10 X	-1.238	-1.399 to -1.077	Yes
5016	4:0 X vs. 27:40 X	-0.3930	-0.5370 to -0.2490	Yes
5017	4:0 X vs. 27:50 X	-0.2527	-0.3966 to -0.1087	Yes
5018	4:0 X vs. 28:0 X	-1.285	-1.446 to -1.124	Yes
5019	4:0 X vs. 28:1 X	-1.326	-1.470 to -1.182	Yes
5020	4:0 X vs. 28:10 X	-1.127	-1.288 to -0.9664	Yes
5021	4:0 X vs. 28:40 X	-0.4053	-0.5493 to -0.2614	Yes
5022	4:0 X vs. 28:50 X	-0.3007	-0.4446 to -0.1567	Yes
5023	4:0 X vs. 29:0 X	-1.766	-1.927 to -1.605	Yes
5024	4:0 X vs. 29:1 X	-1.851	-2.012 to -1.690	Yes
5025	4:0 X vs. 29:10 X	-1.066	-1.227 to -0.9049	Yes
5026	4:0 X vs. 29:40 X	-0.5133	-0.6573 to -0.3694	Yes
5027	4:0 X vs. 29:50 X	-0.3020	-0.4460 to -0.1580	Yes
5028	4:0 X vs. 30:0 X	-1.481	-1.642 to -1.320	Yes
5029	4:0 X vs. 30:1 X	-1.802	-1.963 to -1.641	Yes
5030	4:0 X vs. 30:10 X	-1.140	-1.301 to -0.9794	Yes
5031	4:0 X vs. 30:40 X	-0.4403	-0.5843 to -0.2964	Yes
5032	4:0 X vs. 30:50 X	-0.3543	-0.4983 to -0.2104	Yes
5033	4:0 X vs. 31:0 X	-1.799	-1.960 to -1.638	Yes
5034	4:0 X vs. 31:1 X	-1.789	-1.950 to -1.628	Yes
5035	4:0 X vs. 31:10 X	-1.272	-1.433 to -1.111	Yes
5036	4:0 X vs. 31:40 X	-0.5320	-0.6760 to -0.3880	Yes
5037	4:0 X vs. 31:50 X	-0.3333	-0.4773 to -0.1894	Yes
5038	4:0 X vs. 32:0 X	-2.390	-2.534 to -2.246	Yes
5039	4:0 X vs. 32:1 X	-1.846	-2.007 to -1.685	Yes
5040	4:0 X vs. 32:10 X	-1.702	-1.846 to -1.558	Yes

	2way ANOVA Multiple comparisons	A<br>Data Set-A<br>Y	B<br>Data Set-B<br>Y	C<br>Data Set-C<br>Y
5041	4:0 X vs. 32:40 X	-0.5283	-0.6723 to -0.3844	Yes
5042	4:0 X vs. 32:50 X	-0.3330	-0.4770 to -0.1890	Yes
5043	4:0 X vs. 33:0 X	-2.394	-2.538 to -2.250	Yes
5044	4:0 X vs. 33:1 X	-2.209	-2.353 to -2.065	Yes
5045	4:0 X vs. 33:10 X	-1.869	-2.013 to -1.725	Yes
5046	4:0 X vs. 33:40 X	-0.5470	-0.6910 to -0.4030	Yes
5047	4:0 X vs. 33:50 X	-0.3480	-0.4920 to -0.2040	Yes
5048	4:0 X vs. 34:0 X	-2.319	-2.463 to -2.175	Yes
5049	4:0 X vs. 34:1 X	-2.326	-2.470 to -2.182	Yes
5050	4:0 X vs. 34:10 X	-1.959	-2.103 to -1.815	Yes
5051	4:0 X vs. 34:40 X	-0.5590	-0.7030 to -0.4150	Yes
5052	4:0 X vs. 34:50 X	-0.4970	-0.6410 to -0.3530	Yes
5053	4:0 X vs. 35:0 X	-2.667	-2.811 to -2.523	Yes
5054	4:0 X vs. 35:1 X	-2.433	-2.577 to -2.289	Yes
5055	4:0 X vs. 35:10 X	-1.921	-2.065 to -1.777	Yes
5056	4:0 X vs. 35:40 X	-0.5680	-0.7120 to -0.4240	Yes
5057	4:0 X vs. 35:50 X	-0.5097	-0.6536 to -0.3657	Yes
5058	4:0 X vs. 36:0 X	-2.874	-3.035 to -2.713	Yes
5059	4:0 X vs. 36:1 X	-2.570	-2.714 to -2.426	Yes
5060	4:0 X vs. 36:10 X	-1.927	-2.071 to -1.783	Yes
5061	4:0 X vs. 36:40 X	-0.6233	-0.7673 to -0.4794	Yes
5062	4:0 X vs. 36:50 X	-0.4730	-0.6170 to -0.3290	Yes
5063	4:0 X vs. 37:0 X	-3.258	-3.402 to -3.114	Yes
5064	4:0 X vs. 37:1 X	-2.640	-2.784 to -2.496	Yes
5065	4:0 X vs. 37:10 X	-1.928	-2.072 to -1.784	Yes
5066	4:0 X vs. 37:40 X	-0.6607	-0.8046 to -0.5167	Yes
5067	4:0 X vs. 37:50 X	-0.4817	-0.6256 to -0.3377	Yes
5068	4:0 X vs. 38:0 X	-3.203	-3.347 to -3.059	Yes
5069	4:0 X vs. 38:1 X	-2.758	-2.902 to -2.614	Yes
5070	4:0 X vs. 38:10 X	-2.204	-2.348 to -2.060	Yes
5071	4:0 X vs. 38:40 X	-0.6650	-0.8090 to -0.5210	Yes
5072	4:0 X vs. 38:50 X	-0.4857	-0.6296 to -0.3417	Yes
5073	4:0 X vs. 39:0 X	-3.433	-3.577 to -3.289	Yes
5074	4:0 X vs. 39:1 X	-3.325	-3.469 to -3.181	Yes
5075	4:0 X vs. 39:10 X	-2.349	-2.493 to -2.205	Yes
5076	4:0 X vs. 39:40 X	-0.6710	-0.8150 to -0.5270	Yes
5077	4:0 X vs. 39:50 X	-0.5167	-0.6606 to -0.3727	Yes
5078	4:0 X vs. 40:0 X	-3.474	-3.618 to -3.330	Yes
5079	4:0 X vs. 40:1 X	-3.400	-3.544 to -3.256	Yes
5080	4:0 X vs. 40:10 X	-2.605	-2.749 to -2.461	Yes
5081	4:0 X vs. 40:40 X	-0.6757	-0.8196 to -0.5317	Yes
5082	4:0 X vs. 40:50 X	-0.5230	-0.6670 to -0.3790	Yes
5083	4:0 X vs. 41:0 X	-3.515	-3.659 to -3.371	Yes
5084	4:0 X vs. 41:1 X	-3.514	-3.658 to -3.370	Yes
5085	4:0 X vs. 41:10 X	-2.659	-2.803 to -2.515	Yes

2way ANOVA<br>Multiple comparisons	A<br>Data Set-A<br>Y	B<br>Data Set-B<br>Y	C<br>Data Set-C<br>Y
5086 4:0 X vs. 41:40 X	-0.6230	-0.7670 to -0.4790	Yes
5087 4:0 X vs. 41:50 X	-0.5357	-0.6796 to -0.3917	Yes
5088 4:0 X vs. 42:0 X	-3.657	-3.801 to -3.513	Yes
5089 4:0 X vs. 42:1 X	-3.677	-3.821 to -3.533	Yes
5090 4:0 X vs. 42:10 X	-2.659	-2.803 to -2.515	Yes
5091 4:0 X vs. 42:40 X	-0.6357	-0.7796 to -0.4917	Yes
5092 4:0 X vs. 42:50 X	-0.5390	-0.6830 to -0.3950	Yes
5093 4:0 X vs. 43:0 X	-3.747	-3.891 to -3.603	Yes
5094 4:0 X vs. 43:1 X	-3.661	-3.805 to -3.517	Yes
5095 4:0 X vs. 43:10 X	-3.377	-3.521 to -3.233	Yes
5096 4:0 X vs. 43:40 X	-0.6340	-0.7780 to -0.4900	Yes
5097 4:0 X vs. 43:50 X	-0.5393	-0.6833 to -0.3954	Yes
5098 4:0 X vs. 44:0 X	-3.771	-3.915 to -3.627	Yes
5099 4:0 X vs. 44:1 X	-3.661	-3.805 to -3.517	Yes
5100 4:0 X vs. 44:10 X	-3.440	-3.584 to -3.296	Yes
5101 4:0 X vs. 44:40 X	-0.6360	-0.7800 to -0.4920	Yes
5102 4:0 X vs. 44:50 X	-0.4717	-0.6156 to -0.3277	Yes
5103 4:0 X vs. 45:0 X	-3.636	-3.780 to -3.492	Yes
5104 4:0 X vs. 45:1 X	-3.438	-3.582 to -3.294	Yes
5105 4:0 X vs. 45:10 X	-3.320	-3.464 to -3.176	Yes
5106 4:0 X vs. 45:40 X	-0.6373	-0.7813 to -0.4934	Yes
5107 4:0 X vs. 45:50 X	-0.4687	-0.6126 to -0.3247	Yes
5108 4:0 X vs. 46:0 X	-3.440	-3.584 to -3.296	Yes
5109 4:0 X vs. 46:1 X	-3.407	-3.551 to -3.263	Yes
5110 4:0 X vs. 46:10 X	-3.315	-3.459 to -3.171	Yes
5111 4:0 X vs. 46:40 X	-0.6510	-0.7950 to -0.5070	Yes
5112 4:0 X vs. 46:50 X	-0.4617	-0.6056 to -0.3177	Yes
5113 4:0 X vs. 47:0 X	-3.416	-3.560 to -3.272	Yes
5114 4:0 X vs. 47:1 X	-3.320	-3.464 to -3.176	Yes
5115 4:0 X vs. 47:10 X	-3.301	-3.445 to -3.157	Yes
5116 4:0 X vs. 47:40 X	-0.6610	-0.8050 to -0.5170	Yes
5117 4:0 X vs. 47:50 X	-0.4197	-0.5636 to -0.2757	Yes
5118 4:0 X vs. 48:0 X	-3.403	-3.564 to -3.242	Yes
5119 4:0 X vs. 48:1 X	-3.307	-3.451 to -3.163	Yes
5120 4:0 X vs. 48:10 X	-3.301	-3.445 to -3.157	Yes
5121 4:0 X vs. 48:40 X	-0.6673	-0.8113 to -0.5234	Yes
5122 4:0 X vs. 48:50 X	-0.4270	-0.5710 to -0.2830	Yes
5123 4:0 X vs. 49:0 X	-3.403	-3.547 to -3.259	Yes
5124 4:0 X vs. 49:1 X	-3.307	-3.451 to -3.163	Yes
5125 4:0 X vs. 49:10 X	-3.301	-3.445 to -3.157	Yes
5126 4:0 X vs. 49:40 X	-0.6717	-0.8156 to -0.5277	Yes
5127 4:0 X vs. 49:50 X	-0.4263	-0.5703 to -0.2824	Yes
5128 4:0 X vs. 50:0 X	-3.403	-3.547 to -3.259	Yes
5129 4:0 X vs. 50:1 X	-3.307	-3.451 to -3.163	Yes
5130 4:0 X vs. 50:10 X	-3.301	-3.445 to -3.157	Yes

2way ANOVA<br>Multiple comparisons		A<br>Data Set-A<br>Y	B<br>Data Set-B<br>Y	C<br>Data Set-C<br>Y
5131	4:0 X vs. 50:40 X	-0.6793	-0.8233 to -0.5354	Yes
5132	4:0 X vs. 50:50 X	-0.4260	-0.5700 to -0.2820	Yes
5133	4:1 X vs. 4:10 X	-0.01467	-0.1586 to 0.1293	No
5134	4:1 X vs. 4:40 X	0.005667	-0.1383 to 0.1496	No
5135	4:1 X vs. 4:50 X	0.007667	-0.1363 to 0.1516	No
5136	4:1 X vs. 5:0 X	-0.002333	-0.1463 to 0.1416	No
5137	4:1 X vs. 5:1 X	-0.0003333	-0.1443 to 0.1436	No
5138	4:1 X vs. 5:10 X	-0.01533	-0.1593 to 0.1286	No
5139	4:1 X vs. 5:40 X	0.005667	-0.1383 to 0.1496	No
5140	4:1 X vs. 5:50 X	0.006667	-0.1373 to 0.1506	No
5141	4:1 X vs. 6:0 X	-0.001667	-0.1456 to 0.1423	No
5142	4:1 X vs. 6:1 X	-0.0006667	-0.1446 to 0.1433	No
5143	4:1 X vs. 6:10 X	-0.01533	-0.1593 to 0.1286	No
5144	4:1 X vs. 6:40 X	0.003333	-0.1406 to 0.1473	No
5145	4:1 X vs. 6:50 X	0.005000	-0.1390 to 0.1490	No
5146	4:1 X vs. 7:0 X	-0.003667	-0.1476 to 0.1403	No
5147	4:1 X vs. 7:1 X	0.005000	-0.1390 to 0.1490	No
5148	4:1 X vs. 7:10 X	-0.0070	-0.1510 to 0.1370	No
5149	4:1 X vs. 7:40 X	0.001667	-0.1423 to 0.1456	No
5150	4:1 X vs. 7:50 X	0.003667	-0.1403 to 0.1476	No
5151	4:1 X vs. 8:0 X	-0.008333	-0.1523 to 0.1356	No
5152	4:1 X vs. 8:1 X	0.005000	-0.1390 to 0.1490	No
5153	4:1 X vs. 8:10 X	-0.002333	-0.1463 to 0.1416	No
5154	4:1 X vs. 8:40 X	-0.0030	-0.1470 to 0.1410	No
5155	4:1 X vs. 8:50 X	-9.313e-010	-0.1440 to 0.1440	No
5156	4:1 X vs. 9:0 X	-0.01633	-0.1603 to 0.1276	No
5157	4:1 X vs. 9:1 X	-0.02433	-0.1683 to 0.1196	No
5158	4:1 X vs. 9:10 X	-0.02533	-0.1693 to 0.1186	No
5159	4:1 X vs. 9:40 X	-0.007667	-0.1516 to 0.1363	No
5160	4:1 X vs. 9:50 X	-0.001000	-0.1450 to 0.1430	No
5161	4:1 X vs. 10:0 X	-0.0180	-0.1620 to 0.1260	No
5162	4:1 X vs. 10:1 X	-0.0250	-0.1690 to 0.1190	No
5163	4:1 X vs. 10:10 X	-0.02767	-0.1716 to 0.1163	No
5164	4:1 X vs. 10:40 X	-0.01733	-0.1613 to 0.1266	No
5165	4:1 X vs. 10:50 X	-0.0040	-0.1480 to 0.1400	No
5166	4:1 X vs. 11:0 X	-0.02767	-0.1716 to 0.1163	No
5167	4:1 X vs. 11:1 X	-0.02933	-0.1733 to 0.1146	No
5168	4:1 X vs. 11:10 X	-0.02967	-0.1736 to 0.1143	No
5169	4:1 X vs. 11:40 X	-0.02133	-0.1653 to 0.1226	No
5170	4:1 X vs. 11:50 X	-0.009333	-0.1533 to 0.1346	No
5171	4:1 X vs. 12:0 X	-0.02833	-0.1723 to 0.1156	No
5172	4:1 X vs. 12:1 X	-0.01067	-0.1546 to 0.1333	No
5173	4:1 X vs. 12:10 X	-0.02833	-0.1723 to 0.1156	No
5174	4:1 X vs. 12:40 X	-0.01467	-0.1586 to 0.1293	No
5175	4:1 X vs. 12:50 X	-0.0180	-0.1620 to 0.1260	No

	2way ANOVA<br>Multiple comparisons	A<br>Data Set-A<br>Y	B<br>Data Set-B<br>Y	C<br>Data Set-C<br>Y
5176	4:1 X vs. 13:0 X	-0.02233	-0.1663 to 0.1216	No
5177	4:1 X vs. 13:1 X	-0.009667	-0.1536 to 0.1343	No
5178	4:1 X vs. 13:10 X	-0.03167	-0.1756 to 0.1123	No
5179	4:1 X vs. 13:40 X	-0.0280	-0.1720 to 0.1160	No
5180	4:1 X vs. 13:50 X	-0.0290	-0.1730 to 0.1150	No
5181	4:1 X vs. 14:0 X	-0.0310	-0.1750 to 0.1130	No
5182	4:1 X vs. 14:1 X	-0.02267	-0.1666 to 0.1213	No
5183	4:1 X vs. 14:10 X	-0.03667	-0.1806 to 0.1073	No
5184	4:1 X vs. 14:40 X	-0.03333	-0.1773 to 0.1106	No
5185	4:1 X vs. 14:50 X	-0.03733	-0.1813 to 0.1066	No
5186	4:1 X vs. 15:0 X	-0.04333	-0.1873 to 0.1006	No
5187	4:1 X vs. 15:1 X	-0.02333	-0.1673 to 0.1206	No
5188	4:1 X vs. 15:10 X	-0.05067	-0.1946 to 0.09329	No
5189	4:1 X vs. 15:40 X	-0.0330	-0.1770 to 0.1110	No
5190	4:1 X vs. 15:50 X	-0.05067	-0.1946 to 0.09329	No
5191	4:1 X vs. 16:0 X	-0.1357	-0.2796 to 0.008293	No
5192	4:1 X vs. 16:1 X	-0.02667	-0.1706 to 0.1173	No
5193	4:1 X vs. 16:10 X	-0.05233	-0.1963 to 0.09163	No
5194	4:1 X vs. 16:40 X	-0.07167	-0.2156 to 0.07229	No
5195	4:1 X vs. 16:50 X	-0.1140	-0.2580 to 0.02996	No
5196	4:1 X vs. 17:0 X	-0.1883	-0.3323 to -0.04437	Yes
5197	4:1 X vs. 17:1 X	-0.1027	-0.2466 to 0.04129	No
5198	4:1 X vs. 17:10 X	-0.1593	-0.3033 to -0.01537	Yes
5199	4:1 X vs. 17:40 X	-0.08267	-0.2266 to 0.06129	No
5200	4:1 X vs. 17:50 X	-0.1280	-0.2720 to 0.01596	No
5201	4:1 X vs. 18:0 X	-0.2370	-0.3810 to -0.09304	Yes
5202	4:1 X vs. 18:1 X	-0.1453	-0.2893 to -0.001374	Yes
5203	4:1 X vs. 18:10 X	-0.2377	-0.3816 to -0.09371	Yes
5204	4:1 X vs. 18:40 X	-0.0870	-0.2310 to 0.05696	No
5205	4:1 X vs. 18:50 X	-0.1320	-0.2760 to 0.01196	No
5206	4:1 X vs. 19:0 X	-0.2720	-0.4160 to -0.1280	Yes
5207	4:1 X vs. 19:1 X	-0.2600	-0.4040 to -0.1160	Yes
5208	4:1 X vs. 19:10 X	-0.2577	-0.4016 to -0.1137	Yes
5209	4:1 X vs. 19:40 X	-0.1157	-0.2596 to 0.02829	No
5210	4:1 X vs. 19:50 X	-0.1267	-0.2706 to 0.01729	No
5211	4:1 X vs. 20:0 X	-0.3517	-0.4956 to -0.2077	Yes
5212	4:1 X vs. 20:1 X	-0.1710	-0.3150 to -0.02704	Yes
5213	4:1 X vs. 20:10 X	-0.2600	-0.4040 to -0.1160	Yes
5214	4:1 X vs. 20:40 X	-0.1273	-0.2713 to 0.01663	No
5215	4:1 X vs. 20:50 X	-0.1713	-0.3153 to -0.02737	Yes
5216	4:1 X vs. 21:0 X	-0.3750	-0.5190 to -0.2310	Yes
5217	4:1 X vs. 21:1 X	-0.3280	-0.4720 to -0.1840	Yes
5218	4:1 X vs. 21:10 X	-0.3250	-0.4690 to -0.1810	Yes
5219	4:1 X vs. 21:40 X	-0.2003	-0.3443 to -0.05637	Yes
5220	4:1 X vs. 21:50 X	-0.2907	-0.4346 to -0.1467	Yes

	2way ANOVA<br>Multiple comparisons	A<br>Data Set-A<br>Y	B<br>Data Set-B<br>Y	C<br>Data Set-C<br>Y
5221	4:1 X vs. 22:0 X	-0.4167	-0.5606 to -0.2727	Yes
5222	4:1 X vs. 22:1 X	-0.4113	-0.5553 to -0.2674	Yes
5223	4:1 X vs. 22:10 X	-0.4067	-0.5506 to -0.2627	Yes
5224	4:1 X vs. 22:40 X	-0.3070	-0.4510 to -0.1630	Yes
5225	4:1 X vs. 22:50 X	-0.3697	-0.5136 to -0.2257	Yes
5226	4:1 X vs. 23:0 X	-0.4820	-0.6260 to -0.3380	Yes
5227	4:1 X vs. 23:1 X	-0.6717	-0.8326 to -0.5107	Yes
5228	4:1 X vs. 23:10 X	-0.4213	-0.5653 to -0.2774	Yes
5229	4:1 X vs. 23:40 X	-0.3017	-0.4626 to -0.1407	Yes
5230	4:1 X vs. 23:50 X	-0.3357	-0.4796 to -0.1917	Yes
5231	4:1 X vs. 24:0 X	-0.5993	-0.7433 to -0.4554	Yes
5232	4:1 X vs. 24:1 X	-0.7557	-0.9166 to -0.5947	Yes
5233	4:1 X vs. 24:10 X	-0.5832	-0.7441 to -0.4222	Yes
5234	4:1 X vs. 24:40 X	-0.3180	-0.4620 to -0.1740	Yes
5235	4:1 X vs. 24:50 X	-0.2397	-0.3836 to -0.09571	Yes
5236	4:1 X vs. 25:0 X	-0.9367	-1.098 to -0.7757	Yes
5237	4:1 X vs. 25:1 X	-1.126	-1.287 to -0.9652	Yes
5238	4:1 X vs. 25:10 X	-0.4877	-0.6913 to -0.2841	Yes
5239	4:1 X vs. 25:40 X	-0.3200	-0.4640 to -0.1760	Yes
5240	4:1 X vs. 25:50 X	-0.2943	-0.4383 to -0.1504	Yes
5241	4:1 X vs. 26:0 X	-1.149	-1.310 to -0.9882	Yes
5242	4:1 X vs. 26:1 X	-1.262	-1.423 to -1.101	Yes
5243	4:1 X vs. 26:10 X	-0.9487	-1.110 to -0.7877	Yes
5244	4:1 X vs. 26:40 X	-0.3943	-0.5383 to -0.2504	Yes
5245	4:1 X vs. 26:50 X	-0.2547	-0.3986 to -0.1107	Yes
5246	4:1 X vs. 27:0 X	-1.337	-1.481 to -1.193	Yes
5247	4:1 X vs. 27:1 X	-1.428	-1.589 to -1.267	Yes
5248	4:1 X vs. 27:10 X	-1.240	-1.401 to -1.079	Yes
5249	4:1 X vs. 27:40 X	-0.3943	-0.5383 to -0.2504	Yes
5250	4:1 X vs. 27:50 X	-0.2540	-0.3980 to -0.1100	Yes
5251	4:1 X vs. 28:0 X	-1.286	-1.447 to -1.125	Yes
5252	4:1 X vs. 28:1 X	-1.327	-1.471 to -1.183	Yes
5253	4:1 X vs. 28:10 X	-1.129	-1.290 to -0.9677	Yes
5254	4:1 X vs. 28:40 X	-0.4067	-0.5506 to -0.2627	Yes
5255	4:1 X vs. 28:50 X	-0.3020	-0.4460 to -0.1580	Yes
5256	4:1 X vs. 29:0 X	-1.768	-1.929 to -1.607	Yes
5257	4:1 X vs. 29:1 X	-1.853	-2.014 to -1.692	Yes
5258	4:1 X vs. 29:10 X	-1.067	-1.228 to -0.9062	Yes
5259	4:1 X vs. 29:40 X	-0.5147	-0.6586 to -0.3707	Yes
5260	4:1 X vs. 29:50 X	-0.3033	-0.4473 to -0.1594	Yes
5261	4:1 X vs. 30:0 X	-1.482	-1.643 to -1.321	Yes
5262	4:1 X vs. 30:1 X	-1.804	-1.965 to -1.643	Yes
5263	4:1 X vs. 30:10 X	-1.142	-1.303 to -0.9807	Yes
5264	4:1 X vs. 30:40 X	-0.4417	-0.5856 to -0.2977	Yes
5265	4:1 X vs. 30:50 X	-0.3557	-0.4996 to -0.2117	Yes

	2way ANOVA<br>Multiple comparisons	A<br>Data Set-A<br>Y	B<br>Data Set-B<br>Y	C<br>Data Set-C<br>Y
5266	4:1 X vs. 31:0 X	-1.800	-1.961 to -1.639	Yes
5267	4:1 X vs. 31:1 X	-1.791	-1.952 to -1.630	Yes
5268	4:1 X vs. 31:10 X	-1.273	-1.434 to -1.112	Yes
5269	4:1 X vs. 31:40 X	-0.5333	-0.6773 to -0.3894	Yes
5270	4:1 X vs. 31:50 X	-0.3347	-0.4786 to -0.1907	Yes
5271	4:1 X vs. 32:0 X	-2.392	-2.536 to -2.248	Yes
5272	4:1 X vs. 32:1 X	-1.848	-2.009 to -1.687	Yes
5273	4:1 X vs. 32:10 X	-1.704	-1.848 to -1.560	Yes
5274	4:1 X vs. 32:40 X	-0.5297	-0.6736 to -0.3857	Yes
5275	4:1 X vs. 32:50 X	-0.3343	-0.4783 to -0.1904	Yes
5276	4:1 X vs. 33:0 X	-2.395	-2.539 to -2.251	Yes
5277	4:1 X vs. 33:1 X	-2.210	-2.354 to -2.066	Yes
5278	4:1 X vs. 33:10 X	-1.870	-2.014 to -1.726	Yes
5279	4:1 X vs. 33:40 X	-0.5483	-0.6923 to -0.4044	Yes
5280	4:1 X vs. 33:50 X	-0.3493	-0.4933 to -0.2054	Yes
5281	4:1 X vs. 34:0 X	-2.320	-2.464 to -2.176	Yes
5282	4:1 X vs. 34:1 X	-2.327	-2.471 to -2.183	Yes
5283	4:1 X vs. 34:10 X	-1.961	-2.105 to -1.817	Yes
5284	4:1 X vs. 34:40 X	-0.5603	-0.7043 to -0.4164	Yes
5285	4:1 X vs. 34:50 X	-0.4983	-0.6423 to -0.3544	Yes
5286	4:1 X vs. 35:0 X	-2.669	-2.813 to -2.525	Yes
5287	4:1 X vs. 35:1 X	-2.434	-2.578 to -2.290	Yes
5288	4:1 X vs. 35:10 X	-1.922	-2.066 to -1.778	Yes
5289	4:1 X vs. 35:40 X	-0.5693	-0.7133 to -0.4254	Yes
5290	4:1 X vs. 35:50 X	-0.5110	-0.6550 to -0.3670	Yes
5291	4:1 X vs. 36:0 X	-2.875	-3.036 to -2.714	Yes
5292	4:1 X vs. 36:1 X	-2.571	-2.715 to -2.427	Yes
5293	4:1 X vs. 36:10 X	-1.928	-2.072 to -1.784	Yes
5294	4:1 X vs. 36:40 X	-0.6247	-0.7686 to -0.4807	Yes
5295	4:1 X vs. 36:50 X	-0.4743	-0.6183 to -0.3304	Yes
5296	4:1 X vs. 37:0 X	-3.259	-3.403 to -3.115	Yes
5297	4:1 X vs. 37:1 X	-2.641	-2.785 to -2.497	Yes
5298	4:1 X vs. 37:10 X	-1.930	-2.074 to -1.786	Yes
5299	4:1 X vs. 37:40 X	-0.6620	-0.8060 to -0.5180	Yes
5300	4:1 X vs. 37:50 X	-0.4830	-0.6270 to -0.3390	Yes
5301	4:1 X vs. 38:0 X	-3.204	-3.348 to -3.060	Yes
5302	4:1 X vs. 38:1 X	-2.760	-2.904 to -2.616	Yes
5303	4:1 X vs. 38:10 X	-2.205	-2.349 to -2.061	Yes
5304	4:1 X vs. 38:40 X	-0.6663	-0.8103 to -0.5224	Yes
5305	4:1 X vs. 38:50 X	-0.4870	-0.6310 to -0.3430	Yes
5306	4:1 X vs. 39:0 X	-3.435	-3.579 to -3.291	Yes
5307	4:1 X vs. 39:1 X	-3.326	-3.470 to -3.182	Yes
5308	4:1 X vs. 39:10 X	-2.350	-2.494 to -2.206	Yes
5309	4:1 X vs. 39:40 X	-0.6723	-0.8163 to -0.5284	Yes
5310	4:1 X vs. 39:50 X	-0.5180	-0.6620 to -0.3740	Yes

	2way ANOVA<br>Multiple comparisons	A<br>Data Set-A<br>Y	B<br>Data Set-B<br>Y	C<br>Data Set-C<br>Y
5311	4:1 X vs. 40:0 X	-3.476	-3.620 to -3.332	Yes
5312	4:1 X vs. 40:1 X	-3.402	-3.546 to -3.258	Yes
5313	4:1 X vs. 40:10 X	-2.606	-2.750 to -2.462	Yes
5314	4:1 X vs. 40:40 X	-0.6770	-0.8210 to -0.5330	Yes
5315	4:1 X vs. 40:50 X	-0.5243	-0.6683 to -0.3804	Yes
5316	4:1 X vs. 41:0 X	-3.517	-3.661 to -3.373	Yes
5317	4:1 X vs. 41:1 X	-3.516	-3.660 to -3.372	Yes
5318	4:1 X vs. 41:10 X	-2.660	-2.804 to -2.516	Yes
5319	4:1 X vs. 41:40 X	-0.6243	-0.7683 to -0.4804	Yes
5320	4:1 X vs. 41:50 X	-0.5370	-0.6810 to -0.3930	Yes
5321	4:1 X vs. 42:0 X	-3.658	-3.802 to -3.514	Yes
5322	4:1 X vs. 42:1 X	-3.678	-3.822 to -3.534	Yes
5323	4:1 X vs. 42:10 X	-2.660	-2.804 to -2.516	Yes
5324	4:1 X vs. 42:40 X	-0.6370	-0.7810 to -0.4930	Yes
5325	4:1 X vs. 42:50 X	-0.5403	-0.6843 to -0.3964	Yes
5326	4:1 X vs. 43:0 X	-3.748	-3.892 to -3.604	Yes
5327	4:1 X vs. 43:1 X	-3.663	-3.807 to -3.519	Yes
5328	4:1 X vs. 43:10 X	-3.378	-3.522 to -3.234	Yes
5329	4:1 X vs. 43:40 X	-0.6353	-0.7793 to -0.4914	Yes
5330	4:1 X vs. 43:50 X	-0.5407	-0.6846 to -0.3967	Yes
5331	4:1 X vs. 44:0 X	-3.772	-3.916 to -3.628	Yes
5332	4:1 X vs. 44:1 X	-3.662	-3.806 to -3.518	Yes
5333	4:1 X vs. 44:10 X	-3.442	-3.586 to -3.298	Yes
5334	4:1 X vs. 44:40 X	-0.6373	-0.7813 to -0.4934	Yes
5335	4:1 X vs. 44:50 X	-0.4730	-0.6170 to -0.3290	Yes
5336	4:1 X vs. 45:0 X	-3.637	-3.781 to -3.493	Yes
5337	4:1 X vs. 45:1 X	-3.439	-3.583 to -3.295	Yes
5338	4:1 X vs. 45:10 X	-3.321	-3.465 to -3.177	Yes
5339	4:1 X vs. 45:40 X	-0.6387	-0.7826 to -0.4947	Yes
5340	4:1 X vs. 45:50 X	-0.4700	-0.6140 to -0.3260	Yes
5341	4:1 X vs. 46:0 X	-3.441	-3.585 to -3.297	Yes
5342	4:1 X vs. 46:1 X	-3.408	-3.552 to -3.264	Yes
5343	4:1 X vs. 46:10 X	-3.316	-3.460 to -3.172	Yes
5344	4:1 X vs. 46:40 X	-0.6523	-0.7963 to -0.5084	Yes
5345	4:1 X vs. 46:50 X	-0.4630	-0.6070 to -0.3190	Yes
5346	4:1 X vs. 47:0 X	-3.417	-3.561 to -3.273	Yes
5347	4:1 X vs. 47:1 X	-3.321	-3.465 to -3.177	Yes
5348	4:1 X vs. 47:10 X	-3.302	-3.446 to -3.158	Yes
5349	4:1 X vs. 47:40 X	-0.6623	-0.8063 to -0.5184	Yes
5350	4:1 X vs. 47:50 X	-0.4210	-0.5650 to -0.2770	Yes
5351	4:1 X vs. 48:0 X	-3.404	-3.565 to -3.243	Yes
5352	4:1 X vs. 48:1 X	-3.308	-3.452 to -3.164	Yes
5353	4:1 X vs. 48:10 X	-3.302	-3.446 to -3.158	Yes
5354	4:1 X vs. 48:40 X	-0.6687	-0.8126 to -0.5247	Yes
5355	4:1 X vs. 48:50 X	-0.4283	-0.5723 to -0.2844	Yes

	2way ANOVA<br>Multiple comparisons	A<br>Data Set-A<br>Y	B<br>Data Set-B<br>Y	C<br>Data Set-C<br>Y
5356	4:**1 X** vs. 49:**0 X**	-3.404	-3.548 to -3.260	Yes
5357	4:**1 X** vs. 49:**1 X**	-3.308	-3.452 to -3.164	Yes
5358	4:**1 X** vs. 49:**10 X**	-3.302	-3.446 to -3.158	Yes
5359	4:**1 X** vs. 49:**40 X**	-0.6730	-0.8170 to -0.5290	Yes
5360	4:**1 X** vs. 49:**50 X**	-0.4277	-0.5716 to -0.2837	Yes
5361	4:**1 X** vs. 50:**0 X**	-3.404	-3.548 to -3.260	Yes
5362	4:**1 X** vs. 50:**1 X**	-3.308	-3.452 to -3.164	Yes
5363	4:**1 X** vs. 50:**10 X**	-3.302	-3.446 to -3.158	Yes
5364	4:**1 X** vs. 50:**40 X**	-0.6807	-0.8246 to -0.5367	Yes
5365	4:**1 X** vs. 50:**50 X**	-0.4273	-0.5713 to -0.2834	Yes
5366	4:**10 X** vs. 4:**40 X**	0.02033	-0.1236 to 0.1643	No
5367	4:**10 X** vs. 4:**50 X**	0.02233	-0.1216 to 0.1663	No
5368	4:**10 X** vs. 5:**0 X**	0.01233	-0.1316 to 0.1563	No
5369	4:**10 X** vs. 5:**1 X**	0.01433	-0.1296 to 0.1583	No
5370	4:**10 X** vs. 5:**10 X**	-0.0006667	-0.1446 to 0.1433	No
5371	4:**10 X** vs. 5:**40 X**	0.02033	-0.1236 to 0.1643	No
5372	4:**10 X** vs. 5:**50 X**	0.02133	-0.1226 to 0.1653	No
5373	4:**10 X** vs. 6:**0 X**	0.0130	-0.1310 to 0.1570	No
5374	4:**10 X** vs. 6:**1 X**	0.0140	-0.1300 to 0.1580	No
5375	4:**10 X** vs. 6:**10 X**	-0.0006667	-0.1446 to 0.1433	No
5376	4:**10 X** vs. 6:**40 X**	0.0180	-0.1260 to 0.1620	No
5377	4:**10 X** vs. 6:**50 X**	0.01967	-0.1243 to 0.1636	No
5378	4:**10 X** vs. 7:**0 X**	0.0110	-0.1330 to 0.1550	No
5379	4:**10 X** vs. 7:**1 X**	0.01967	-0.1243 to 0.1636	No
5380	4:**10 X** vs. 7:**10 X**	0.007667	-0.1363 to 0.1516	No
5381	4:**10 X** vs. 7:**40 X**	0.01633	-0.1276 to 0.1603	No
5382	4:**10 X** vs. 7:**50 X**	0.01833	-0.1256 to 0.1623	No
5383	4:**10 X** vs. 8:**0 X**	0.006333	-0.1376 to 0.1503	No
5384	4:**10 X** vs. 8:**1 X**	0.01967	-0.1243 to 0.1636	No
5385	4:**10 X** vs. 8:**10 X**	0.01233	-0.1316 to 0.1563	No
5386	4:**10 X** vs. 8:**40 X**	0.01167	-0.1323 to 0.1556	No
5387	4:**10 X** vs. 8:**50 X**	0.01467	-0.1293 to 0.1586	No
5388	4:**10 X** vs. 9:**0 X**	-0.001667	-0.1456 to 0.1423	No
5389	4:**10 X** vs. 9:**1 X**	-0.009667	-0.1536 to 0.1343	No
5390	4:**10 X** vs. 9:**10 X**	-0.01067	-0.1546 to 0.1333	No
5391	4:**10 X** vs. 9:**40 X**	0.007000	-0.1370 to 0.1510	No
5392	4:**10 X** vs. 9:**50 X**	0.01367	-0.1303 to 0.1576	No
5393	4:**10 X** vs. 10:**0 X**	-0.003333	-0.1473 to 0.1406	No
5394	4:**10 X** vs. 10:**1 X**	-0.01033	-0.1543 to 0.1336	No
5395	4:**10 X** vs. 10:**10 X**	-0.0130	-0.1570 to 0.1310	No
5396	4:**10 X** vs. 10:**40 X**	-0.002667	-0.1466 to 0.1413	No
5397	4:**10 X** vs. 10:**50 X**	0.01067	-0.1333 to 0.1546	No
5398	4:**10 X** vs. 11:**0 X**	-0.0130	-0.1570 to 0.1310	No
5399	4:**10 X** vs. 11:**1 X**	-0.01467	-0.1586 to 0.1293	No
5400	4:**10 X** vs. 11:**10 X**	-0.0150	-0.1590 to 0.1290	No

	2way ANOVA<br>Multiple comparisons	A<br>Data Set-A<br>Y	B<br>Data Set-B<br>Y	C<br>Data Set-C<br>Y
5401	4:**10 X** vs. 11:**40 X**	-0.006667	-0.1506 to 0.1373	No
5402	4:**10 X** vs. 11:**50 X**	0.005333	-0.1386 to 0.1493	No
5403	4:**10 X** vs. 12:**0 X**	-0.01367	-0.1576 to 0.1303	No
5404	4:**10 X** vs. 12:**1 X**	0.004000	-0.1400 to 0.1480	No
5405	4:**10 X** vs. 12:**10 X**	-0.01367	-0.1576 to 0.1303	No
5406	4:**10 X** vs. 12:**40 X**	0.0	-0.1440 to 0.1440	No
5407	4:**10 X** vs. 12:**50 X**	-0.003333	-0.1473 to 0.1406	No
5408	4:**10 X** vs. 13:**0 X**	-0.007667	-0.1516 to 0.1363	No
5409	4:**10 X** vs. 13:**1 X**	0.0050	-0.1390 to 0.1490	No
5410	4:**10 X** vs. 13:**10 X**	-0.0170	-0.1610 to 0.1270	No
5411	4:**10 X** vs. 13:**40 X**	-0.01333	-0.1573 to 0.1306	No
5412	4:**10 X** vs. 13:**50 X**	-0.01433	-0.1583 to 0.1296	No
5413	4:**10 X** vs. 14:**0 X**	-0.01633	-0.1603 to 0.1276	No
5414	4:**10 X** vs. 14:**1 X**	-0.0080	-0.1520 to 0.1360	No
5415	4:**10 X** vs. 14:**10 X**	-0.0220	-0.1660 to 0.1220	No
5416	4:**10 X** vs. 14:**40 X**	-0.01867	-0.1626 to 0.1253	No
5417	4:**10 X** vs. 14:**50 X**	-0.02267	-0.1666 to 0.1213	No
5418	4:**10 X** vs. 15:**0 X**	-0.02867	-0.1726 to 0.1153	No
5419	4:**10 X** vs. 15:**1 X**	-0.008667	-0.1526 to 0.1353	No
5420	4:**10 X** vs. 15:**10 X**	-0.0360	-0.1800 to 0.1080	No
5421	4:**10 X** vs. 15:**40 X**	-0.01833	-0.1623 to 0.1256	No
5422	4:**10 X** vs. 15:**50 X**	-0.0360	-0.1800 to 0.1080	No
5423	4:**10 X** vs. 16:**0 X**	-0.1210	-0.2650 to 0.02296	No
5424	4:**10 X** vs. 16:**1 X**	-0.0120	-0.1560 to 0.1320	No
5425	4:**10 X** vs. 16:**10 X**	-0.03767	-0.1816 to 0.1063	No
5426	4:**10 X** vs. 16:**40 X**	-0.0570	-0.2010 to 0.08696	No
5427	4:**10 X** vs. 16:**50 X**	-0.09933	-0.2433 to 0.04463	No
5428	4:**10 X** vs. 17:**0 X**	-0.1737	-0.3176 to -0.02971	Yes
5429	4:**10 X** vs. 17:**1 X**	-0.0880	-0.2320 to 0.05596	No
5430	4:**10 X** vs. 17:**10 X**	-0.1447	-0.2886 to -0.0007068	Yes
5431	4:**10 X** vs. 17:**40 X**	-0.0680	-0.2120 to 0.07596	No
5432	4:**10 X** vs. 17:**50 X**	-0.1133	-0.2573 to 0.03063	No
5433	4:**10 X** vs. 18:**0 X**	-0.2223	-0.3663 to -0.07837	Yes
5434	4:**10 X** vs. 18:**1 X**	-0.1307	-0.2746 to 0.01329	No
5435	4:**10 X** vs. 18:**10 X**	-0.2230	-0.3670 to -0.07904	Yes
5436	4:**10 X** vs. 18:**40 X**	-0.07233	-0.2163 to 0.07163	No
5437	4:**10 X** vs. 18:**50 X**	-0.1173	-0.2613 to 0.02663	No
5438	4:**10 X** vs. 19:**0 X**	-0.2573	-0.4013 to -0.1134	Yes
5439	4:**10 X** vs. 19:**1 X**	-0.2453	-0.3893 to -0.1014	Yes
5440	4:**10 X** vs. 19:**10 X**	-0.2430	-0.3870 to -0.09904	Yes
5441	4:**10 X** vs. 19:**40 X**	-0.1010	-0.2450 to 0.04296	No
5442	4:**10 X** vs. 19:**50 X**	-0.1120	-0.2560 to 0.03196	No
5443	4:**10 X** vs. 20:**0 X**	-0.3370	-0.4810 to -0.1930	Yes
5444	4:**10 X** vs. 20:**1 X**	-0.1563	-0.3003 to -0.01237	Yes
5445	4:**10 X** vs. 20:**10 X**	-0.2453	-0.3893 to -0.1014	Yes

	2way ANOVA<br>Multiple comparisons	A<br>Data Set-A<br>Y	B<br>Data Set-B<br>Y	C<br>Data Set-C<br>Y
5446	4:**10 X** vs. 20:**40 X**	-0.1127	-0.2566 to 0.03129	No
5447	4:**10 X** vs. 20:**50 X**	-0.1567	-0.3006 to -0.01271	Yes
5448	4:**10 X** vs. 21:**0 X**	-0.3603	-0.5043 to -0.2164	Yes
5449	4:**10 X** vs. 21:**1 X**	-0.3133	-0.4573 to -0.1694	Yes
5450	4:**10 X** vs. 21:**10 X**	-0.3103	-0.4543 to -0.1664	Yes
5451	4:**10 X** vs. 21:**40 X**	-0.1857	-0.3296 to -0.04171	Yes
5452	4:**10 X** vs. 21:**50 X**	-0.2760	-0.4200 to -0.1320	Yes
5453	4:**10 X** vs. 22:**0 X**	-0.4020	-0.5460 to -0.2580	Yes
5454	4:**10 X** vs. 22:**1 X**	-0.3967	-0.5406 to -0.2527	Yes
5455	4:**10 X** vs. 22:**10 X**	-0.3920	-0.5360 to -0.2480	Yes
5456	4:**10 X** vs. 22:**40 X**	-0.2923	-0.4363 to -0.1484	Yes
5457	4:**10 X** vs. 22:**50 X**	-0.3550	-0.4990 to -0.2110	Yes
5458	4:**10 X** vs. 23:**0 X**	-0.4673	-0.6113 to -0.3234	Yes
5459	4:**10 X** vs. 23:**1 X**	-0.6570	-0.8180 to -0.4960	Yes
5460	4:**10 X** vs. 23:**10 X**	-0.4067	-0.5506 to -0.2627	Yes
5461	4:**10 X** vs. 23:**40 X**	-0.2870	-0.4480 to -0.1260	Yes
5462	4:**10 X** vs. 23:**50 X**	-0.3210	-0.4650 to -0.1770	Yes
5463	4:**10 X** vs. 24:**0 X**	-0.5847	-0.7286 to -0.4407	Yes
5464	4:**10 X** vs. 24:**1 X**	-0.7410	-0.9020 to -0.5800	Yes
5465	4:**10 X** vs. 24:**10 X**	-0.5685	-0.7295 to -0.4075	Yes
5466	4:**10 X** vs. 24:**40 X**	-0.3033	-0.4473 to -0.1594	Yes
5467	4:**10 X** vs. 24:**50 X**	-0.2250	-0.3690 to -0.08104	Yes
5468	4:**10 X** vs. 25:**0 X**	-0.9220	-1.083 to -0.7610	Yes
5469	4:**10 X** vs. 25:**1 X**	-1.112	-1.272 to -0.9505	Yes
5470	4:**10 X** vs. 25:**10 X**	-0.4730	-0.6766 to -0.2694	Yes
5471	4:**10 X** vs. 25:**40 X**	-0.3053	-0.4493 to -0.1614	Yes
5472	4:**10 X** vs. 25:**50 X**	-0.2797	-0.4236 to -0.1357	Yes
5473	4:**10 X** vs. 26:**0 X**	-1.135	-1.295 to -0.9735	Yes
5474	4:**10 X** vs. 26:**1 X**	-1.248	-1.408 to -1.087	Yes
5475	4:**10 X** vs. 26:**10 X**	-0.9340	-1.095 to -0.7730	Yes
5476	4:**10 X** vs. 26:**40 X**	-0.3797	-0.5236 to -0.2357	Yes
5477	4:**10 X** vs. 26:**50 X**	-0.2400	-0.3840 to -0.09604	Yes
5478	4:**10 X** vs. 27:**0 X**	-1.322	-1.466 to -1.178	Yes
5479	4:**10 X** vs. 27:**1 X**	-1.413	-1.574 to -1.252	Yes
5480	4:**10 X** vs. 27:**10 X**	-1.225	-1.386 to -1.064	Yes
5481	4:**10 X** vs. 27:**40 X**	-0.3797	-0.5236 to -0.2357	Yes
5482	4:**10 X** vs. 27:**50 X**	-0.2393	-0.3833 to -0.09537	Yes
5483	4:**10 X** vs. 28:**0 X**	-1.272	-1.432 to -1.111	Yes
5484	4:**10 X** vs. 28:**1 X**	-1.312	-1.456 to -1.168	Yes
5485	4:**10 X** vs. 28:**10 X**	-1.114	-1.275 to -0.9530	Yes
5486	4:**10 X** vs. 28:**40 X**	-0.3920	-0.5360 to -0.2480	Yes
5487	4:**10 X** vs. 28:**50 X**	-0.2873	-0.4313 to -0.1434	Yes
5488	4:**10 X** vs. 29:**0 X**	-1.753	-1.914 to -1.592	Yes
5489	4:**10 X** vs. 29:**1 X**	-1.838	-1.999 to -1.677	Yes
5490	4:**10 X** vs. 29:**10 X**	-1.053	-1.213 to -0.8915	Yes

	2way ANOVA<br>Multiple comparisons	A<br>Data Set-A<br>Y	B<br>Data Set-B<br>Y	C<br>Data Set-C<br>Y
5491	4:10 X vs. 29:40 X	-0.5000	-0.6440 to -0.3560	Yes
5492	4:10 X vs. 29:50 X	-0.2887	-0.4326 to -0.1447	Yes
5493	4:10 X vs. 30:0 X	-1.468	-1.628 to -1.307	Yes
5494	4:10 X vs. 30:1 X	-1.789	-1.950 to -1.628	Yes
5495	4:10 X vs. 30:10 X	-1.127	-1.288 to -0.9660	Yes
5496	4:10 X vs. 30:40 X	-0.4270	-0.5710 to -0.2830	Yes
5497	4:10 X vs. 30:50 X	-0.3410	-0.4850 to -0.1970	Yes
5498	4:10 X vs. 31:0 X	-1.786	-1.946 to -1.625	Yes
5499	4:10 X vs. 31:1 X	-1.776	-1.937 to -1.615	Yes
5500	4:10 X vs. 31:10 X	-1.259	-1.419 to -1.098	Yes
5501	4:10 X vs. 31:40 X	-0.5187	-0.6626 to -0.3747	Yes
5502	4:10 X vs. 31:50 X	-0.3200	-0.4640 to -0.1760	Yes
5503	4:10 X vs. 32:0 X	-2.377	-2.521 to -2.233	Yes
5504	4:10 X vs. 32:1 X	-1.833	-1.994 to -1.672	Yes
5505	4:10 X vs. 32:10 X	-1.689	-1.833 to -1.545	Yes
5506	4:10 X vs. 32:40 X	-0.5150	-0.6590 to -0.3710	Yes
5507	4:10 X vs. 32:50 X	-0.3197	-0.4636 to -0.1757	Yes
5508	4:10 X vs. 33:0 X	-2.381	-2.525 to -2.237	Yes
5509	4:10 X vs. 33:1 X	-2.195	-2.339 to -2.051	Yes
5510	4:10 X vs. 33:10 X	-1.856	-2.000 to -1.712	Yes
5511	4:10 X vs. 33:40 X	-0.5337	-0.6776 to -0.3897	Yes
5512	4:10 X vs. 33:50 X	-0.3347	-0.4786 to -0.1907	Yes
5513	4:10 X vs. 34:0 X	-2.305	-2.449 to -2.161	Yes
5514	4:10 X vs. 34:1 X	-2.313	-2.457 to -2.169	Yes
5515	4:10 X vs. 34:10 X	-1.946	-2.090 to -1.802	Yes
5516	4:10 X vs. 34:40 X	-0.5457	-0.6896 to -0.4017	Yes
5517	4:10 X vs. 34:50 X	-0.4837	-0.6276 to -0.3397	Yes
5518	4:10 X vs. 35:0 X	-2.654	-2.798 to -2.510	Yes
5519	4:10 X vs. 35:1 X	-2.420	-2.564 to -2.276	Yes
5520	4:10 X vs. 35:10 X	-1.908	-2.052 to -1.764	Yes
5521	4:10 X vs. 35:40 X	-0.5547	-0.6986 to -0.4107	Yes
5522	4:10 X vs. 35:50 X	-0.4963	-0.6403 to -0.3524	Yes
5523	4:10 X vs. 36:0 X	-2.861	-3.021 to -2.700	Yes
5524	4:10 X vs. 36:1 X	-2.556	-2.700 to -2.412	Yes
5525	4:10 X vs. 36:10 X	-1.913	-2.057 to -1.769	Yes
5526	4:10 X vs. 36:40 X	-0.6100	-0.7540 to -0.4660	Yes
5527	4:10 X vs. 36:50 X	-0.4597	-0.6036 to -0.3157	Yes
5528	4:10 X vs. 37:0 X	-3.244	-3.388 to -3.100	Yes
5529	4:10 X vs. 37:1 X	-2.626	-2.770 to -2.482	Yes
5530	4:10 X vs. 37:10 X	-1.915	-2.059 to -1.771	Yes
5531	4:10 X vs. 37:40 X	-0.6473	-0.7913 to -0.5034	Yes
5532	4:10 X vs. 37:50 X	-0.4683	-0.6123 to -0.3244	Yes
5533	4:10 X vs. 38:0 X	-3.189	-3.333 to -3.045	Yes
5534	4:10 X vs. 38:1 X	-2.745	-2.889 to -2.601	Yes
5535	4:10 X vs. 38:10 X	-2.190	-2.334 to -2.046	Yes

	2way ANOVA<br>Multiple comparisons	A<br>Data Set-A<br>Y	B<br>Data Set-B<br>Y	C<br>Data Set-C<br>Y
5536	4:10 X vs. 38:40 X	-0.6517	-0.7956 to -0.5077	Yes
5537	4:10 X vs. 38:50 X	-0.4723	-0.6163 to -0.3284	Yes
5538	4:10 X vs. 39:0 X	-3.420	-3.564 to -3.276	Yes
5539	4:10 X vs. 39:1 X	-3.312	-3.456 to -3.168	Yes
5540	4:10 X vs. 39:10 X	-2.335	-2.479 to -2.191	Yes
5541	4:10 X vs. 39:40 X	-0.6577	-0.8016 to -0.5137	Yes
5542	4:10 X vs. 39:50 X	-0.5033	-0.6473 to -0.3594	Yes
5543	4:10 X vs. 40:0 X	-3.461	-3.605 to -3.317	Yes
5544	4:10 X vs. 40:1 X	-3.387	-3.531 to -3.243	Yes
5545	4:10 X vs. 40:10 X	-2.592	-2.736 to -2.448	Yes
5546	4:10 X vs. 40:40 X	-0.6623	-0.8063 to -0.5184	Yes
5547	4:10 X vs. 40:50 X	-0.5097	-0.6536 to -0.3657	Yes
5548	4:10 X vs. 41:0 X	-3.502	-3.646 to -3.358	Yes
5549	4:10 X vs. 41:1 X	-3.501	-3.645 to -3.357	Yes
5550	4:10 X vs. 41:10 X	-2.645	-2.789 to -2.501	Yes
5551	4:10 X vs. 41:40 X	-0.6097	-0.7536 to -0.4657	Yes
5552	4:10 X vs. 41:50 X	-0.5223	-0.6663 to -0.3784	Yes
5553	4:10 X vs. 42:0 X	-3.644	-3.788 to -3.500	Yes
5554	4:10 X vs. 42:1 X	-3.663	-3.807 to -3.519	Yes
5555	4:10 X vs. 42:10 X	-2.646	-2.790 to -2.502	Yes
5556	4:10 X vs. 42:40 X	-0.6223	-0.7663 to -0.4784	Yes
5557	4:10 X vs. 42:50 X	-0.5257	-0.6696 to -0.3817	Yes
5558	4:10 X vs. 43:0 X	-3.734	-3.878 to -3.590	Yes
5559	4:10 X vs. 43:1 X	-3.648	-3.792 to -3.504	Yes
5560	4:10 X vs. 43:10 X	-3.364	-3.508 to -3.220	Yes
5561	4:10 X vs. 43:40 X	-0.6207	-0.7646 to -0.4767	Yes
5562	4:10 X vs. 43:50 X	-0.5260	-0.6700 to -0.3820	Yes
5563	4:10 X vs. 44:0 X	-3.758	-3.902 to -3.614	Yes
5564	4:10 X vs. 44:1 X	-3.647	-3.791 to -3.503	Yes
5565	4:10 X vs. 44:10 X	-3.427	-3.571 to -3.283	Yes
5566	4:10 X vs. 44:40 X	-0.6227	-0.7666 to -0.4787	Yes
5567	4:10 X vs. 44:50 X	-0.4583	-0.6023 to -0.3144	Yes
5568	4:10 X vs. 45:0 X	-3.622	-3.766 to -3.478	Yes
5569	4:10 X vs. 45:1 X	-3.424	-3.568 to -3.280	Yes
5570	4:10 X vs. 45:10 X	-3.307	-3.451 to -3.163	Yes
5571	4:10 X vs. 45:40 X	-0.6240	-0.7680 to -0.4800	Yes
5572	4:10 X vs. 45:50 X	-0.4553	-0.5993 to -0.3114	Yes
5573	4:10 X vs. 46:0 X	-3.427	-3.571 to -3.283	Yes
5574	4:10 X vs. 46:1 X	-3.394	-3.538 to -3.250	Yes
5575	4:10 X vs. 46:10 X	-3.302	-3.446 to -3.158	Yes
5576	4:10 X vs. 46:40 X	-0.6377	-0.7816 to -0.4937	Yes
5577	4:10 X vs. 46:50 X	-0.4483	-0.5923 to -0.3044	Yes
5578	4:10 X vs. 47:0 X	-3.402	-3.546 to -3.258	Yes
5579	4:10 X vs. 47:1 X	-3.306	-3.450 to -3.162	Yes
5580	4:10 X vs. 47:10 X	-3.288	-3.432 to -3.144	Yes

2way ANOVA<br>Multiple comparisons		A<br>Data Set-A<br>Y	B<br>Data Set-B<br>Y	C<br>Data Set-C<br>Y
5581	4:**10 X** vs. 47:**40 X**	-0.6477	-0.7916 to -0.5037	Yes
5582	4:**10 X** vs. 47:**50 X**	-0.4063	-0.5503 to -0.2624	Yes
5583	4:**10 X** vs. 48:**0 X**	-3.390	-3.550 to -3.229	Yes
5584	4:**10 X** vs. 48:**1 X**	-3.293	-3.437 to -3.149	Yes
5585	4:**10 X** vs. 48:**10 X**	-3.288	-3.432 to -3.144	Yes
5586	4:**10 X** vs. 48:**40 X**	-0.6540	-0.7980 to -0.5100	Yes
5587	4:**10 X** vs. 48:**50 X**	-0.4137	-0.5576 to -0.2697	Yes
5588	4:**10 X** vs. 49:**0 X**	-3.390	-3.534 to -3.246	Yes
5589	4:**10 X** vs. 49:**1 X**	-3.293	-3.437 to -3.149	Yes
5590	4:**10 X** vs. 49:**10 X**	-3.288	-3.432 to -3.144	Yes
5591	4:**10 X** vs. 49:**40 X**	-0.6583	-0.8023 to -0.5144	Yes
5592	4:**10 X** vs. 49:**50 X**	-0.4130	-0.5570 to -0.2690	Yes
5593	4:**10 X** vs. 50:**0 X**	-3.390	-3.534 to -3.246	Yes
5594	4:**10 X** vs. 50:**1 X**	-3.293	-3.437 to -3.149	Yes
5595	4:**10 X** vs. 50:**10 X**	-3.288	-3.432 to -3.144	Yes
5596	4:**10 X** vs. 50:**40 X**	-0.6660	-0.8100 to -0.5220	Yes
5597	4:**10 X** vs. 50:**50 X**	-0.4127	-0.5566 to -0.2687	Yes
5598	4:**40 X** vs. 4:**50 X**	0.0020	-0.1420 to 0.1460	No
5599	4:**40 X** vs. 5:**0 X**	-0.0080	-0.1520 to 0.1360	No
5600	4:**40 X** vs. 5:**1 X**	-0.0060	-0.1500 to 0.1380	No
5601	4:**40 X** vs. 5:**10 X**	-0.0210	-0.1650 to 0.1230	No
5602	4:**40 X** vs. 5:**40 X**	0.0	-0.1440 to 0.1440	No
5603	4:**40 X** vs. 5:**50 X**	0.001000	-0.1430 to 0.1450	No
5604	4:**40 X** vs. 6:**0 X**	-0.007333	-0.1513 to 0.1366	No
5605	4:**40 X** vs. 6:**1 X**	-0.006333	-0.1503 to 0.1376	No
5606	4:**40 X** vs. 6:**10 X**	-0.0210	-0.1650 to 0.1230	No
5607	4:**40 X** vs. 6:**40 X**	-0.002333	-0.1463 to 0.1416	No
5608	4:**40 X** vs. 6:**50 X**	-0.0006667	-0.1446 to 0.1433	No
5609	4:**40 X** vs. 7:**0 X**	-0.009333	-0.1533 to 0.1346	No
5610	4:**40 X** vs. 7:**1 X**	-0.0006667	-0.1446 to 0.1433	No
5611	4:**40 X** vs. 7:**10 X**	-0.01267	-0.1566 to 0.1313	No
5612	4:**40 X** vs. 7:**40 X**	-0.0040	-0.1480 to 0.1400	No
5613	4:**40 X** vs. 7:**50 X**	-0.0020	-0.1460 to 0.1420	No
5614	4:**40 X** vs. 8:**0 X**	-0.0140	-0.1580 to 0.1300	No
5615	4:**40 X** vs. 8:**1 X**	-0.0006667	-0.1446 to 0.1433	No
5616	4:**40 X** vs. 8:**10 X**	-0.008000	-0.1520 to 0.1360	No
5617	4:**40 X** vs. 8:**40 X**	-0.008667	-0.1526 to 0.1353	No
5618	4:**40 X** vs. 8:**50 X**	-0.005667	-0.1496 to 0.1383	No
5619	4:**40 X** vs. 9:**0 X**	-0.0220	-0.1660 to 0.1220	No
5620	4:**40 X** vs. 9:**1 X**	-0.0300	-0.1740 to 0.1140	No
5621	4:**40 X** vs. 9:**10 X**	-0.0310	-0.1750 to 0.1130	No
5622	4:**40 X** vs. 9:**40 X**	-0.01333	-0.1573 to 0.1306	No
5623	4:**40 X** vs. 9:**50 X**	-0.006667	-0.1506 to 0.1373	No
5624	4:**40 X** vs. 10:**0 X**	-0.02367	-0.1676 to 0.1203	No
5625	4:**40 X** vs. 10:**1 X**	-0.03067	-0.1746 to 0.1133	No

2way ANOVA<br>Multiple comparisons	A<br>Data Set-A<br>Y	B<br>Data Set-B<br>Y	C<br>Data Set-C<br>Y
5626 4:**40 X** vs. 10:**10 X**	-0.03333	-0.1773 to 0.1106	No
5627 4:**40 X** vs. 10:**40 X**	-0.0230	-0.1670 to 0.1210	No
5628 4:**40 X** vs. 10:**50 X**	-0.009667	-0.1536 to 0.1343	No
5629 4:**40 X** vs. 11:**0 X**	-0.03333	-0.1773 to 0.1106	No
5630 4:**40 X** vs. 11:**1 X**	-0.0350	-0.1790 to 0.1090	No
5631 4:**40 X** vs. 11:**10 X**	-0.03533	-0.1793 to 0.1086	No
5632 4:**40 X** vs. 11:**40 X**	-0.0270	-0.1710 to 0.1170	No
5633 4:**40 X** vs. 11:**50 X**	-0.0150	-0.1590 to 0.1290	No
5634 4:**40 X** vs. 12:**0 X**	-0.0340	-0.1780 to 0.1100	No
5635 4:**40 X** vs. 12:**1 X**	-0.01633	-0.1603 to 0.1276	No
5636 4:**40 X** vs. 12:**10 X**	-0.0340	-0.1780 to 0.1100	No
5637 4:**40 X** vs. 12:**40 X**	-0.02033	-0.1643 to 0.1236	No
5638 4:**40 X** vs. 12:**50 X**	-0.02367	-0.1676 to 0.1203	No
5639 4:**40 X** vs. 13:**0 X**	-0.0280	-0.1720 to 0.1160	No
5640 4:**40 X** vs. 13:**1 X**	-0.01533	-0.1593 to 0.1286	No
5641 4:**40 X** vs. 13:**10 X**	-0.03733	-0.1813 to 0.1066	No
5642 4:**40 X** vs. 13:**40 X**	-0.03367	-0.1776 to 0.1103	No
5643 4:**40 X** vs. 13:**50 X**	-0.03467	-0.1786 to 0.1093	No
5644 4:**40 X** vs. 14:**0 X**	-0.03667	-0.1806 to 0.1073	No
5645 4:**40 X** vs. 14:**1 X**	-0.02833	-0.1723 to 0.1156	No
5646 4:**40 X** vs. 14:**10 X**	-0.04233	-0.1863 to 0.1016	No
5647 4:**40 X** vs. 14:**40 X**	-0.0390	-0.1830 to 0.1050	No
5648 4:**40 X** vs. 14:**50 X**	-0.0430	-0.1870 to 0.1010	No
5649 4:**40 X** vs. 15:**0 X**	-0.0490	-0.1930 to 0.09496	No
5650 4:**40 X** vs. 15:**1 X**	-0.0290	-0.1730 to 0.1150	No
5651 4:**40 X** vs. 15:**10 X**	-0.05633	-0.2003 to 0.08763	No
5652 4:**40 X** vs. 15:**40 X**	-0.03867	-0.1826 to 0.1053	No
5653 4:**40 X** vs. 15:**50 X**	-0.05633	-0.2003 to 0.08763	No
5654 4:**40 X** vs. 16:**0 X**	-0.1413	-0.2853 to 0.002626	No
5655 4:**40 X** vs. 16:**1 X**	-0.03233	-0.1763 to 0.1116	No
5656 4:**40 X** vs. 16:**10 X**	-0.0580	-0.2020 to 0.08596	No
5657 4:**40 X** vs. 16:**40 X**	-0.07733	-0.2213 to 0.06663	No
5658 4:**40 X** vs. 16:**50 X**	-0.1197	-0.2636 to 0.02429	No
5659 4:**40 X** vs. 17:**0 X**	-0.1940	-0.3380 to -0.05004	Yes
5660 4:**40 X** vs. 17:**1 X**	-0.1083	-0.2523 to 0.03563	No
5661 4:**40 X** vs. 17:**10 X**	-0.1650	-0.3090 to -0.02104	Yes
5662 4:**40 X** vs. 17:**40 X**	-0.08833	-0.2323 to 0.05563	No
5663 4:**40 X** vs. 17:**50 X**	-0.1337	-0.2776 to 0.01029	No
5664 4:**40 X** vs. 18:**0 X**	-0.2427	-0.3866 to -0.09871	Yes
5665 4:**40 X** vs. 18:**1 X**	-0.1510	-0.2950 to -0.007040	Yes
5666 4:**40 X** vs. 18:**10 X**	-0.2433	-0.3873 to -0.09937	Yes
5667 4:**40 X** vs. 18:**40 X**	-0.09267	-0.2366 to 0.05129	No
5668 4:**40 X** vs. 18:**50 X**	-0.1377	-0.2816 to 0.006293	No
5669 4:**40 X** vs. 19:**0 X**	-0.2777	-0.4216 to -0.1337	Yes
5670 4:**40 X** vs. 19:**1 X**	-0.2657	-0.4096 to -0.1217	Yes

	2way ANOVA<br>Multiple comparisons	A<br>Data Set-A<br>Y	B<br>Data Set-B<br>Y	C<br>Data Set-C<br>Y
5671	4:**40 X** vs. 19:**10 X**	-0.2633	-0.4073 to -0.1194	Yes
5672	4:**40 X** vs. 19:**40 X**	-0.1213	-0.2653 to 0.02263	No
5673	4:**40 X** vs. 19:**50 X**	-0.1323	-0.2763 to 0.01163	No
5674	4:**40 X** vs. 20:**0 X**	-0.3573	-0.5013 to -0.2134	Yes
5675	4:**40 X** vs. 20:**1 X**	-0.1767	-0.3206 to -0.03271	Yes
5676	4:**40 X** vs. 20:**10 X**	-0.2657	-0.4096 to -0.1217	Yes
5677	4:**40 X** vs. 20:**40 X**	-0.1330	-0.2770 to 0.01096	No
5678	4:**40 X** vs. 20:**50 X**	-0.1770	-0.3210 to -0.03304	Yes
5679	4:**40 X** vs. 21:**0 X**	-0.3807	-0.5246 to -0.2367	Yes
5680	4:**40 X** vs. 21:**1 X**	-0.3337	-0.4776 to -0.1897	Yes
5681	4:**40 X** vs. 21:**10 X**	-0.3307	-0.4746 to -0.1867	Yes
5682	4:**40 X** vs. 21:**40 X**	-0.2060	-0.3500 to -0.06204	Yes
5683	4:**40 X** vs. 21:**50 X**	-0.2963	-0.4403 to -0.1524	Yes
5684	4:**40 X** vs. 22:**0 X**	-0.4223	-0.5663 to -0.2784	Yes
5685	4:**40 X** vs. 22:**1 X**	-0.4170	-0.5610 to -0.2730	Yes
5686	4:**40 X** vs. 22:**10 X**	-0.4123	-0.5563 to -0.2684	Yes
5687	4:**40 X** vs. 22:**40 X**	-0.3127	-0.4566 to -0.1687	Yes
5688	4:**40 X** vs. 22:**50 X**	-0.3753	-0.5193 to -0.2314	Yes
5689	4:**40 X** vs. 23:**0 X**	-0.4877	-0.6316 to -0.3437	Yes
5690	4:**40 X** vs. 23:**1 X**	-0.6773	-0.8383 to -0.5164	Yes
5691	4:**40 X** vs. 23:**10 X**	-0.4270	-0.5710 to -0.2830	Yes
5692	4:**40 X** vs. 23:**40 X**	-0.3073	-0.4683 to -0.1464	Yes
5693	4:**40 X** vs. 23:**50 X**	-0.3413	-0.4853 to -0.1974	Yes
5694	4:**40 X** vs. 24:**0 X**	-0.6050	-0.7490 to -0.4610	Yes
5695	4:**40 X** vs. 24:**1 X**	-0.7613	-0.9223 to -0.6004	Yes
5696	4:**40 X** vs. 24:**10 X**	-0.5888	-0.7498 to -0.4279	Yes
5697	4:**40 X** vs. 24:**40 X**	-0.3237	-0.4676 to -0.1797	Yes
5698	4:**40 X** vs. 24:**50 X**	-0.2453	-0.3893 to -0.1014	Yes
5699	4:**40 X** vs. 25:**0 X**	-0.9423	-1.103 to -0.7814	Yes
5700	4:**40 X** vs. 25:**1 X**	-1.132	-1.293 to -0.9709	Yes
5701	4:**40 X** vs. 25:**10 X**	-0.4933	-0.6969 to -0.2897	Yes
5702	4:**40 X** vs. 25:**40 X**	-0.3257	-0.4696 to -0.1817	Yes
5703	4:**40 X** vs. 25:**50 X**	-0.3000	-0.4440 to -0.1560	Yes
5704	4:**40 X** vs. 26:**0 X**	-1.155	-1.316 to -0.9939	Yes
5705	4:**40 X** vs. 26:**1 X**	-1.268	-1.429 to -1.107	Yes
5706	4:**40 X** vs. 26:**10 X**	-0.9543	-1.115 to -0.7934	Yes
5707	4:**40 X** vs. 26:**40 X**	-0.4000	-0.5440 to -0.2560	Yes
5708	4:**40 X** vs. 26:**50 X**	-0.2603	-0.4043 to -0.1164	Yes
5709	4:**40 X** vs. 27:**0 X**	-1.343	-1.487 to -1.199	Yes
5710	4:**40 X** vs. 27:**1 X**	-1.433	-1.594 to -1.272	Yes
5711	4:**40 X** vs. 27:**10 X**	-1.245	-1.406 to -1.084	Yes
5712	4:**40 X** vs. 27:**40 X**	-0.4000	-0.5440 to -0.2560	Yes
5713	4:**40 X** vs. 27:**50 X**	-0.2597	-0.4036 to -0.1157	Yes
5714	4:**40 X** vs. 28:**0 X**	-1.292	-1.453 to -1.131	Yes
5715	4:**40 X** vs. 28:**1 X**	-1.333	-1.477 to -1.189	Yes

	2way ANOVA Multiple comparisons	A Data Set-A Y	B Data Set-B Y	C Data Set-C Y
5716	4:**40 X** vs. 28:**10 X**	-1.134	-1.295 to -0.9734	Yes
5717	4:**40 X** vs. 28:**40 X**	-0.4123	-0.5563 to -0.2684	Yes
5718	4:**40 X** vs. 28:**50 X**	-0.3077	-0.4516 to -0.1637	Yes
5719	4:**40 X** vs. 29:**0 X**	-1.773	-1.934 to -1.612	Yes
5720	4:**40 X** vs. 29:**1 X**	-1.858	-2.019 to -1.697	Yes
5721	4:**40 X** vs. 29:**10 X**	-1.073	-1.234 to -0.9119	Yes
5722	4:**40 X** vs. 29:**40 X**	-0.5203	-0.6643 to -0.3764	Yes
5723	4:**40 X** vs. 29:**50 X**	-0.3090	-0.4530 to -0.1650	Yes
5724	4:**40 X** vs. 30:**0 X**	-1.488	-1.649 to -1.327	Yes
5725	4:**40 X** vs. 30:**1 X**	-1.809	-1.970 to -1.648	Yes
5726	4:**40 X** vs. 30:**10 X**	-1.147	-1.308 to -0.9864	Yes
5727	4:**40 X** vs. 30:**40 X**	-0.4473	-0.5913 to -0.3034	Yes
5728	4:**40 X** vs. 30:**50 X**	-0.3613	-0.5053 to -0.2174	Yes
5729	4:**40 X** vs. 31:**0 X**	-1.806	-1.967 to -1.645	Yes
5730	4:**40 X** vs. 31:**1 X**	-1.796	-1.957 to -1.635	Yes
5731	4:**40 X** vs. 31:**10 X**	-1.279	-1.440 to -1.118	Yes
5732	4:**40 X** vs. 31:**40 X**	-0.5390	-0.6830 to -0.3950	Yes
5733	4:**40 X** vs. 31:**50 X**	-0.3403	-0.4843 to -0.1964	Yes
5734	4:**40 X** vs. 32:**0 X**	-2.397	-2.541 to -2.253	Yes
5735	4:**40 X** vs. 32:**1 X**	-1.853	-2.014 to -1.692	Yes
5736	4:**40 X** vs. 32:**10 X**	-1.709	-1.853 to -1.565	Yes
5737	4:**40 X** vs. 32:**40 X**	-0.5353	-0.6793 to -0.3914	Yes
5738	4:**40 X** vs. 32:**50 X**	-0.3400	-0.4840 to -0.1960	Yes
5739	4:**40 X** vs. 33:**0 X**	-2.401	-2.545 to -2.257	Yes
5740	4:**40 X** vs. 33:**1 X**	-2.216	-2.360 to -2.072	Yes
5741	4:**40 X** vs. 33:**10 X**	-1.876	-2.020 to -1.732	Yes
5742	4:**40 X** vs. 33:**40 X**	-0.5540	-0.6980 to -0.4100	Yes
5743	4:**40 X** vs. 33:**50 X**	-0.3550	-0.4990 to -0.2110	Yes
5744	4:**40 X** vs. 34:**0 X**	-2.326	-2.470 to -2.182	Yes
5745	4:**40 X** vs. 34:**1 X**	-2.333	-2.477 to -2.189	Yes
5746	4:**40 X** vs. 34:**10 X**	-1.966	-2.110 to -1.822	Yes
5747	4:**40 X** vs. 34:**40 X**	-0.5660	-0.7100 to -0.4220	Yes
5748	4:**40 X** vs. 34:**50 X**	-0.5040	-0.6480 to -0.3600	Yes
5749	4:**40 X** vs. 35:**0 X**	-2.674	-2.818 to -2.530	Yes
5750	4:**40 X** vs. 35:**1 X**	-2.440	-2.584 to -2.296	Yes
5751	4:**40 X** vs. 35:**10 X**	-1.928	-2.072 to -1.784	Yes
5752	4:**40 X** vs. 35:**40 X**	-0.5750	-0.7190 to -0.4310	Yes
5753	4:**40 X** vs. 35:**50 X**	-0.5167	-0.6606 to -0.3727	Yes
5754	4:**40 X** vs. 36:**0 X**	-2.881	-3.042 to -2.720	Yes
5755	4:**40 X** vs. 36:**1 X**	-2.577	-2.721 to -2.433	Yes
5756	4:**40 X** vs. 36:**10 X**	-1.934	-2.078 to -1.790	Yes
5757	4:**40 X** vs. 36:**40 X**	-0.6303	-0.7743 to -0.4864	Yes
5758	4:**40 X** vs. 36:**50 X**	-0.4800	-0.6240 to -0.3360	Yes
5759	4:**40 X** vs. 37:**0 X**	-3.265	-3.409 to -3.121	Yes
5760	4:**40 X** vs. 37:**1 X**	-2.647	-2.791 to -2.503	Yes

2way ANOVA Multiple comparisons		A Data Set-A Y	B Data Set-B Y	C Data Set-C Y
5761	4:**40 X** vs. 37:**10 X**	-1.935	-2.079 to -1.791	Yes
5762	4:**40 X** vs. 37:**40 X**	-0.6677	-0.8116 to -0.5237	Yes
5763	4:**40 X** vs. 37:**50 X**	-0.4887	-0.6326 to -0.3447	Yes
5764	4:**40 X** vs. 38:**0 X**	-3.210	-3.354 to -3.066	Yes
5765	4:**40 X** vs. 38:**1 X**	-2.765	-2.909 to -2.621	Yes
5766	4:**40 X** vs. 38:**10 X**	-2.211	-2.355 to -2.067	Yes
5767	4:**40 X** vs. 38:**40 X**	-0.6720	-0.8160 to -0.5280	Yes
5768	4:**40 X** vs. 38:**50 X**	-0.4927	-0.6366 to -0.3487	Yes
5769	4:**40 X** vs. 39:**0 X**	-3.440	-3.584 to -3.296	Yes
5770	4:**40 X** vs. 39:**1 X**	-3.332	-3.476 to -3.188	Yes
5771	4:**40 X** vs. 39:**10 X**	-2.356	-2.500 to -2.212	Yes
5772	4:**40 X** vs. 39:**40 X**	-0.6780	-0.8220 to -0.5340	Yes
5773	4:**40 X** vs. 39:**50 X**	-0.5237	-0.6676 to -0.3797	Yes
5774	4:**40 X** vs. 40:**0 X**	-3.481	-3.625 to -3.337	Yes
5775	4:**40 X** vs. 40:**1 X**	-3.407	-3.551 to -3.263	Yes
5776	4:**40 X** vs. 40:**10 X**	-2.612	-2.756 to -2.468	Yes
5777	4:**40 X** vs. 40:**40 X**	-0.6827	-0.8266 to -0.5387	Yes
5778	4:**40 X** vs. 40:**50 X**	-0.5300	-0.6740 to -0.3860	Yes
5779	4:**40 X** vs. 41:**0 X**	-3.522	-3.666 to -3.378	Yes
5780	4:**40 X** vs. 41:**1 X**	-3.521	-3.665 to -3.377	Yes
5781	4:**40 X** vs. 41:**10 X**	-2.666	-2.810 to -2.522	Yes
5782	4:**40 X** vs. 41:**40 X**	-0.6300	-0.7740 to -0.4860	Yes
5783	4:**40 X** vs. 41:**50 X**	-0.5427	-0.6866 to -0.3987	Yes
5784	4:**40 X** vs. 42:**0 X**	-3.664	-3.808 to -3.520	Yes
5785	4:**40 X** vs. 42:**1 X**	-3.684	-3.828 to -3.540	Yes
5786	4:**40 X** vs. 42:**10 X**	-2.666	-2.810 to -2.522	Yes
5787	4:**40 X** vs. 42:**40 X**	-0.6427	-0.7866 to -0.4987	Yes
5788	4:**40 X** vs. 42:**50 X**	-0.5460	-0.6900 to -0.4020	Yes
5789	4:**40 X** vs. 43:**0 X**	-3.754	-3.898 to -3.610	Yes
5790	4:**40 X** vs. 43:**1 X**	-3.668	-3.812 to -3.524	Yes
5791	4:**40 X** vs. 43:**10 X**	-3.384	-3.528 to -3.240	Yes
5792	4:**40 X** vs. 43:**40 X**	-0.6410	-0.7850 to -0.4970	Yes
5793	4:**40 X** vs. 43:**50 X**	-0.5463	-0.6903 to -0.4024	Yes
5794	4:**40 X** vs. 44:**0 X**	-3.778	-3.922 to -3.634	Yes
5795	4:**40 X** vs. 44:**1 X**	-3.668	-3.812 to -3.524	Yes
5796	4:**40 X** vs. 44:**10 X**	-3.447	-3.591 to -3.303	Yes
5797	4:**40 X** vs. 44:**40 X**	-0.6430	-0.7870 to -0.4990	Yes
5798	4:**40 X** vs. 44:**50 X**	-0.4787	-0.6226 to -0.3347	Yes
5799	4:**40 X** vs. 45:**0 X**	-3.643	-3.787 to -3.499	Yes
5800	4:**40 X** vs. 45:**1 X**	-3.445	-3.589 to -3.301	Yes
5801	4:**40 X** vs. 45:**10 X**	-3.327	-3.471 to -3.183	Yes
5802	4:**40 X** vs. 45:**40 X**	-0.6443	-0.7883 to -0.5004	Yes
5803	4:**40 X** vs. 45:**50 X**	-0.4757	-0.6196 to -0.3317	Yes
5804	4:**40 X** vs. 46:**0 X**	-3.447	-3.591 to -3.303	Yes
5805	4:**40 X** vs. 46:**1 X**	-3.414	-3.558 to -3.270	Yes

2way ANOVA<br>Multiple comparisons		A<br>Data Set-A<br>Y	B<br>Data Set-B<br>Y	C<br>Data Set-C<br>Y
5806	4:**40 X** vs. 46:**10 X**	-3.322	-3.466 to -3.178	Yes
5807	4:**40 X** vs. 46:**40 X**	-0.6580	-0.8020 to -0.5140	Yes
5808	4:**40 X** vs. 46:**50 X**	-0.4687	-0.6126 to -0.3247	Yes
5809	4:**40 X** vs. 47:**0 X**	-3.423	-3.567 to -3.279	Yes
5810	4:**40 X** vs. 47:**1 X**	-3.327	-3.471 to -3.183	Yes
5811	4:**40 X** vs. 47:**10 X**	-3.308	-3.452 to -3.164	Yes
5812	4:**40 X** vs. 47:**40 X**	-0.6680	-0.8120 to -0.5240	Yes
5813	4:**40 X** vs. 47:**50 X**	-0.4267	-0.5706 to -0.2827	Yes
5814	4:**40 X** vs. 48:**0 X**	-3.410	-3.571 to -3.249	Yes
5815	4:**40 X** vs. 48:**1 X**	-3.314	-3.458 to -3.170	Yes
5816	4:**40 X** vs. 48:**10 X**	-3.308	-3.452 to -3.164	Yes
5817	4:**40 X** vs. 48:**40 X**	-0.6743	-0.8183 to -0.5304	Yes
5818	4:**40 X** vs. 48:**50 X**	-0.4340	-0.5780 to -0.2900	Yes
5819	4:**40 X** vs. 49:**0 X**	-3.410	-3.554 to -3.266	Yes
5820	4:**40 X** vs. 49:**1 X**	-3.314	-3.458 to -3.170	Yes
5821	4:**40 X** vs. 49:**10 X**	-3.308	-3.452 to -3.164	Yes
5822	4:**40 X** vs. 49:**40 X**	-0.6787	-0.8226 to -0.5347	Yes
5823	4:**40 X** vs. 49:**50 X**	-0.4333	-0.5773 to -0.2894	Yes
5824	4:**40 X** vs. 50:**0 X**	-3.410	-3.554 to -3.266	Yes
5825	4:**40 X** vs. 50:**1 X**	-3.314	-3.458 to -3.170	Yes
5826	4:**40 X** vs. 50:**10 X**	-3.308	-3.452 to -3.164	Yes
5827	4:**40 X** vs. 50:**40 X**	-0.6863	-0.8303 to -0.5424	Yes
5828	4:**40 X** vs. 50:**50 X**	-0.4330	-0.5770 to -0.2890	Yes
5829	4:**50 X** vs. 5:**0 X**	-0.0100	-0.1540 to 0.1340	No
5830	4:**50 X** vs. 5:**1 X**	-0.008000	-0.1520 to 0.1360	No
5831	4:**50 X** vs. 5:**10 X**	-0.0230	-0.1670 to 0.1210	No
5832	4:**50 X** vs. 5:**40 X**	-0.0020	-0.1460 to 0.1420	No
5833	4:**50 X** vs. 5:**50 X**	-0.001000	-0.1450 to 0.1430	No
5834	4:**50 X** vs. 6:**0 X**	-0.009333	-0.1533 to 0.1346	No
5835	4:**50 X** vs. 6:**1 X**	-0.008333	-0.1523 to 0.1356	No
5836	4:**50 X** vs. 6:**10 X**	-0.0230	-0.1670 to 0.1210	No
5837	4:**50 X** vs. 6:**40 X**	-0.004333	-0.1483 to 0.1396	No
5838	4:**50 X** vs. 6:**50 X**	-0.002667	-0.1466 to 0.1413	No
5839	4:**50 X** vs. 7:**0 X**	-0.01133	-0.1553 to 0.1326	No
5840	4:**50 X** vs. 7:**1 X**	-0.002667	-0.1466 to 0.1413	No
5841	4:**50 X** vs. 7:**10 X**	-0.01467	-0.1586 to 0.1293	No
5842	4:**50 X** vs. 7:**40 X**	-0.0060	-0.1500 to 0.1380	No
5843	4:**50 X** vs. 7:**50 X**	-0.0040	-0.1480 to 0.1400	No
5844	4:**50 X** vs. 8:**0 X**	-0.0160	-0.1600 to 0.1280	No
5845	4:**50 X** vs. 8:**1 X**	-0.002667	-0.1466 to 0.1413	No
5846	4:**50 X** vs. 8:**10 X**	-0.01000	-0.1540 to 0.1340	No
5847	4:**50 X** vs. 8:**40 X**	-0.01067	-0.1546 to 0.1333	No
5848	4:**50 X** vs. 8:**50 X**	-0.007667	-0.1516 to 0.1363	No
5849	4:**50 X** vs. 9:**0 X**	-0.0240	-0.1680 to 0.1200	No
5850	4:**50 X** vs. 9:**1 X**	-0.0320	-0.1760 to 0.1120	No

2way ANOVA<br>Multiple comparisons	A<br>Data Set-A<br>Y	B<br>Data Set-B<br>Y	C<br>Data Set-C<br>Y
5851 4:**50 X** vs. 9:**10 X**	-0.0330	-0.1770 to 0.1110	No
5852 4:**50 X** vs. 9:**40 X**	-0.01533	-0.1593 to 0.1286	No
5853 4:**50 X** vs. 9:**50 X**	-0.008667	-0.1526 to 0.1353	No
5854 4:**50 X** vs. 10:**0 X**	-0.02567	-0.1696 to 0.1183	No
5855 4:**50 X** vs. 10:**1 X**	-0.03267	-0.1766 to 0.1113	No
5856 4:**50 X** vs. 10:**10 X**	-0.03533	-0.1793 to 0.1086	No
5857 4:**50 X** vs. 10:**40 X**	-0.0250	-0.1690 to 0.1190	No
5858 4:**50 X** vs. 10:**50 X**	-0.01167	-0.1556 to 0.1323	No
5859 4:**50 X** vs. 11:**0 X**	-0.03533	-0.1793 to 0.1086	No
5860 4:**50 X** vs. 11:**1 X**	-0.0370	-0.1810 to 0.1070	No
5861 4:**50 X** vs. 11:**10 X**	-0.03733	-0.1813 to 0.1066	No
5862 4:**50 X** vs. 11:**40 X**	-0.0290	-0.1730 to 0.1150	No
5863 4:**50 X** vs. 11:**50 X**	-0.0170	-0.1610 to 0.1270	No
5864 4:**50 X** vs. 12:**0 X**	-0.0360	-0.1800 to 0.1080	No
5865 4:**50 X** vs. 12:**1 X**	-0.01833	-0.1623 to 0.1256	No
5866 4:**50 X** vs. 12:**10 X**	-0.0360	-0.1800 to 0.1080	No
5867 4:**50 X** vs. 12:**40 X**	-0.02233	-0.1663 to 0.1216	No
5868 4:**50 X** vs. 12:**50 X**	-0.02567	-0.1696 to 0.1183	No
5869 4:**50 X** vs. 13:**0 X**	-0.0300	-0.1740 to 0.1140	No
5870 4:**50 X** vs. 13:**1 X**	-0.01733	-0.1613 to 0.1266	No
5871 4:**50 X** vs. 13:**10 X**	-0.03933	-0.1833 to 0.1046	No
5872 4:**50 X** vs. 13:**40 X**	-0.03567	-0.1796 to 0.1083	No
5873 4:**50 X** vs. 13:**50 X**	-0.03667	-0.1806 to 0.1073	No
5874 4:**50 X** vs. 14:**0 X**	-0.03867	-0.1826 to 0.1053	No
5875 4:**50 X** vs. 14:**1 X**	-0.03033	-0.1743 to 0.1136	No
5876 4:**50 X** vs. 14:**10 X**	-0.04433	-0.1883 to 0.09963	No
5877 4:**50 X** vs. 14:**40 X**	-0.0410	-0.1850 to 0.1030	No
5878 4:**50 X** vs. 14:**50 X**	-0.0450	-0.1890 to 0.09896	No
5879 4:**50 X** vs. 15:**0 X**	-0.0510	-0.1950 to 0.09296	No
5880 4:**50 X** vs. 15:**1 X**	-0.0310	-0.1750 to 0.1130	No
5881 4:**50 X** vs. 15:**10 X**	-0.05833	-0.2023 to 0.08563	No
5882 4:**50 X** vs. 15:**40 X**	-0.04067	-0.1846 to 0.1033	No
5883 4:**50 X** vs. 15:**50 X**	-0.05833	-0.2023 to 0.08563	No
5884 4:**50 X** vs. 16:**0 X**	-0.1433	-0.2873 to 0.0006265	No
5885 4:**50 X** vs. 16:**1 X**	-0.03433	-0.1783 to 0.1096	No
5886 4:**50 X** vs. 16:**10 X**	-0.0600	-0.2040 to 0.08396	No
5887 4:**50 X** vs. 16:**40 X**	-0.07933	-0.2233 to 0.06463	No
5888 4:**50 X** vs. 16:**50 X**	-0.1217	-0.2656 to 0.02229	No
5889 4:**50 X** vs. 17:**0 X**	-0.1960	-0.3400 to -0.05204	Yes
5890 4:**50 X** vs. 17:**1 X**	-0.1103	-0.2543 to 0.03363	No
5891 4:**50 X** vs. 17:**10 X**	-0.1670	-0.3110 to -0.02304	Yes
5892 4:**50 X** vs. 17:**40 X**	-0.09033	-0.2343 to 0.05363	No
5893 4:**50 X** vs. 17:**50 X**	-0.1357	-0.2796 to 0.008293	No
5894 4:**50 X** vs. 18:**0 X**	-0.2447	-0.3886 to -0.1007	Yes
5895 4:**50 X** vs. 18:**1 X**	-0.1530	-0.2970 to -0.009040	Yes

	2way ANOVA Multiple comparisons	A Data Set-A Y	B Data Set-B Y	C Data Set-C Y
5896	4:**50 X** vs. 18:**10 X**	-0.2453	-0.3893 to -0.1014	Yes
5897	4:**50 X** vs. 18:**40 X**	-0.09467	-0.2386 to 0.04929	No
5898	4:**50 X** vs. 18:**50 X**	-0.1397	-0.2836 to 0.004293	No
5899	4:**50 X** vs. 19:**0 X**	-0.2797	-0.4236 to -0.1357	Yes
5900	4:**50 X** vs. 19:**1 X**	-0.2677	-0.4116 to -0.1237	Yes
5901	4:**50 X** vs. 19:**10 X**	-0.2653	-0.4093 to -0.1214	Yes
5902	4:**50 X** vs. 19:**40 X**	-0.1233	-0.2673 to 0.02063	No
5903	4:**50 X** vs. 19:**50 X**	-0.1343	-0.2783 to 0.009626	No
5904	4:**50 X** vs. 20:**0 X**	-0.3593	-0.5033 to -0.2154	Yes
5905	4:**50 X** vs. 20:**1 X**	-0.1787	-0.3226 to -0.03471	Yes
5906	4:**50 X** vs. 20:**10 X**	-0.2677	-0.4116 to -0.1237	Yes
5907	4:**50 X** vs. 20:**40 X**	-0.1350	-0.2790 to 0.008960	No
5908	4:**50 X** vs. 20:**50 X**	-0.1790	-0.3230 to -0.03504	Yes
5909	4:**50 X** vs. 21:**0 X**	-0.3827	-0.5266 to -0.2387	Yes
5910	4:**50 X** vs. 21:**1 X**	-0.3357	-0.4796 to -0.1917	Yes
5911	4:**50 X** vs. 21:**10 X**	-0.3327	-0.4766 to -0.1887	Yes
5912	4:**50 X** vs. 21:**40 X**	-0.2080	-0.3520 to -0.06404	Yes
5913	4:**50 X** vs. 21:**50 X**	-0.2983	-0.4423 to -0.1544	Yes
5914	4:**50 X** vs. 22:**0 X**	-0.4243	-0.5683 to -0.2804	Yes
5915	4:**50 X** vs. 22:**1 X**	-0.4190	-0.5630 to -0.2750	Yes
5916	4:**50 X** vs. 22:**10 X**	-0.4143	-0.5583 to -0.2704	Yes
5917	4:**50 X** vs. 22:**40 X**	-0.3147	-0.4586 to -0.1707	Yes
5918	4:**50 X** vs. 22:**50 X**	-0.3773	-0.5213 to -0.2334	Yes
5919	4:**50 X** vs. 23:**0 X**	-0.4897	-0.6336 to -0.3457	Yes
5920	4:**50 X** vs. 23:**1 X**	-0.6793	-0.8403 to -0.5184	Yes
5921	4:**50 X** vs. 23:**10 X**	-0.4290	-0.5730 to -0.2850	Yes
5922	4:**50 X** vs. 23:**40 X**	-0.3093	-0.4703 to -0.1484	Yes
5923	4:**50 X** vs. 23:**50 X**	-0.3433	-0.4873 to -0.1994	Yes
5924	4:**50 X** vs. 24:**0 X**	-0.6070	-0.7510 to -0.4630	Yes
5925	4:**50 X** vs. 24:**1 X**	-0.7633	-0.9243 to -0.6024	Yes
5926	4:**50 X** vs. 24:**10 X**	-0.5908	-0.7518 to -0.4299	Yes
5927	4:**50 X** vs. 24:**40 X**	-0.3257	-0.4696 to -0.1817	Yes
5928	4:**50 X** vs. 24:**50 X**	-0.2473	-0.3913 to -0.1034	Yes
5929	4:**50 X** vs. 25:**0 X**	-0.9443	-1.105 to -0.7834	Yes
5930	4:**50 X** vs. 25:**1 X**	-1.134	-1.295 to -0.9729	Yes
5931	4:**50 X** vs. 25:**10 X**	-0.4953	-0.6989 to -0.2917	Yes
5932	4:**50 X** vs. 25:**40 X**	-0.3277	-0.4716 to -0.1837	Yes
5933	4:**50 X** vs. 25:**50 X**	-0.3020	-0.4460 to -0.1580	Yes
5934	4:**50 X** vs. 26:**0 X**	-1.157	-1.318 to -0.9959	Yes
5935	4:**50 X** vs. 26:**1 X**	-1.270	-1.431 to -1.109	Yes
5936	4:**50 X** vs. 26:**10 X**	-0.9563	-1.117 to -0.7954	Yes
5937	4:**50 X** vs. 26:**40 X**	-0.4020	-0.5460 to -0.2580	Yes
5938	4:**50 X** vs. 26:**50 X**	-0.2623	-0.4063 to -0.1184	Yes
5939	4:**50 X** vs. 27:**0 X**	-1.345	-1.489 to -1.201	Yes
5940	4:**50 X** vs. 27:**1 X**	-1.435	-1.596 to -1.274	Yes

2way ANOVA<br>Multiple comparisons	A<br>Data Set-A<br>Y	B<br>Data Set-B<br>Y	C<br>Data Set-C<br>Y
5941 4:**50 X** vs. 27:**10 X**	-1.247	-1.408 to -1.086	Yes
5942 4:**50 X** vs. 27:**40 X**	-0.4020	-0.5460 to -0.2580	Yes
5943 4:**50 X** vs. 27:**50 X**	-0.2617	-0.4056 to -0.1177	Yes
5944 4:**50 X** vs. 28:**0 X**	-1.294	-1.455 to -1.133	Yes
5945 4:**50 X** vs. 28:**1 X**	-1.335	-1.479 to -1.191	Yes
5946 4:**50 X** vs. 28:**10 X**	-1.136	-1.297 to -0.9754	Yes
5947 4:**50 X** vs. 28:**40 X**	-0.4143	-0.5583 to -0.2704	Yes
5948 4:**50 X** vs. 28:**50 X**	-0.3097	-0.4536 to -0.1657	Yes
5949 4:**50 X** vs. 29:**0 X**	-1.775	-1.936 to -1.614	Yes
5950 4:**50 X** vs. 29:**1 X**	-1.860	-2.021 to -1.699	Yes
5951 4:**50 X** vs. 29:**10 X**	-1.075	-1.236 to -0.9139	Yes
5952 4:**50 X** vs. 29:**40 X**	-0.5223	-0.6663 to -0.3784	Yes
5953 4:**50 X** vs. 29:**50 X**	-0.3110	-0.4550 to -0.1670	Yes
5954 4:**50 X** vs. 30:**0 X**	-1.490	-1.651 to -1.329	Yes
5955 4:**50 X** vs. 30:**1 X**	-1.811	-1.972 to -1.650	Yes
5956 4:**50 X** vs. 30:**10 X**	-1.149	-1.310 to -0.9884	Yes
5957 4:**50 X** vs. 30:**40 X**	-0.4493	-0.5933 to -0.3054	Yes
5958 4:**50 X** vs. 30:**50 X**	-0.3633	-0.5073 to -0.2194	Yes
5959 4:**50 X** vs. 31:**0 X**	-1.808	-1.969 to -1.647	Yes
5960 4:**50 X** vs. 31:**1 X**	-1.798	-1.959 to -1.637	Yes
5961 4:**50 X** vs. 31:**10 X**	-1.281	-1.442 to -1.120	Yes
5962 4:**50 X** vs. 31:**40 X**	-0.5410	-0.6850 to -0.3970	Yes
5963 4:**50 X** vs. 31:**50 X**	-0.3423	-0.4863 to -0.1984	Yes
5964 4:**50 X** vs. 32:**0 X**	-2.399	-2.543 to -2.255	Yes
5965 4:**50 X** vs. 32:**1 X**	-1.855	-2.016 to -1.694	Yes
5966 4:**50 X** vs. 32:**10 X**	-1.711	-1.855 to -1.567	Yes
5967 4:**50 X** vs. 32:**40 X**	-0.5373	-0.6813 to -0.3934	Yes
5968 4:**50 X** vs. 32:**50 X**	-0.3420	-0.4860 to -0.1980	Yes
5969 4:**50 X** vs. 33:**0 X**	-2.403	-2.547 to -2.259	Yes
5970 4:**50 X** vs. 33:**1 X**	-2.218	-2.362 to -2.074	Yes
5971 4:**50 X** vs. 33:**10 X**	-1.878	-2.022 to -1.734	Yes
5972 4:**50 X** vs. 33:**40 X**	-0.5560	-0.7000 to -0.4120	Yes
5973 4:**50 X** vs. 33:**50 X**	-0.3570	-0.5010 to -0.2130	Yes
5974 4:**50 X** vs. 34:**0 X**	-2.328	-2.472 to -2.184	Yes
5975 4:**50 X** vs. 34:**1 X**	-2.335	-2.479 to -2.191	Yes
5976 4:**50 X** vs. 34:**10 X**	-1.968	-2.112 to -1.824	Yes
5977 4:**50 X** vs. 34:**40 X**	-0.5680	-0.7120 to -0.4240	Yes
5978 4:**50 X** vs. 34:**50 X**	-0.5060	-0.6500 to -0.3620	Yes
5979 4:**50 X** vs. 35:**0 X**	-2.676	-2.820 to -2.532	Yes
5980 4:**50 X** vs. 35:**1 X**	-2.442	-2.586 to -2.298	Yes
5981 4:**50 X** vs. 35:**10 X**	-1.930	-2.074 to -1.786	Yes
5982 4:**50 X** vs. 35:**40 X**	-0.5770	-0.7210 to -0.4330	Yes
5983 4:**50 X** vs. 35:**50 X**	-0.5187	-0.6626 to -0.3747	Yes
5984 4:**50 X** vs. 36:**0 X**	-2.883	-3.044 to -2.722	Yes
5985 4:**50 X** vs. 36:**1 X**	-2.579	-2.723 to -2.435	Yes

	2way ANOVA Multiple comparisons	A Data Set-A Y	B Data Set-B Y	C Data Set-C Y
5986	4:50 X vs. 36:10 X	-1.936	-2.080 to -1.792	Yes
5987	4:50 X vs. 36:40 X	-0.6323	-0.7763 to -0.4884	Yes
5988	4:50 X vs. 36:50 X	-0.4820	-0.6260 to -0.3380	Yes
5989	4:50 X vs. 37:0 X	-3.267	-3.411 to -3.123	Yes
5990	4:50 X vs. 37:1 X	-2.649	-2.793 to -2.505	Yes
5991	4:50 X vs. 37:10 X	-1.937	-2.081 to -1.793	Yes
5992	4:50 X vs. 37:40 X	-0.6697	-0.8136 to -0.5257	Yes
5993	4:50 X vs. 37:50 X	-0.4907	-0.6346 to -0.3467	Yes
5994	4:50 X vs. 38:0 X	-3.212	-3.356 to -3.068	Yes
5995	4:50 X vs. 38:1 X	-2.767	-2.911 to -2.623	Yes
5996	4:50 X vs. 38:10 X	-2.213	-2.357 to -2.069	Yes
5997	4:50 X vs. 38:40 X	-0.6740	-0.8180 to -0.5300	Yes
5998	4:50 X vs. 38:50 X	-0.4947	-0.6386 to -0.3507	Yes
5999	4:50 X vs. 39:0 X	-3.442	-3.586 to -3.298	Yes
6000	4:50 X vs. 39:1 X	-3.334	-3.478 to -3.190	Yes
6001	4:50 X vs. 39:10 X	-2.358	-2.502 to -2.214	Yes
6002	4:50 X vs. 39:40 X	-0.6800	-0.8240 to -0.5360	Yes
6003	4:50 X vs. 39:50 X	-0.5257	-0.6696 to -0.3817	Yes
6004	4:50 X vs. 40:0 X	-3.483	-3.627 to -3.339	Yes
6005	4:50 X vs. 40:1 X	-3.409	-3.553 to -3.265	Yes
6006	4:50 X vs. 40:10 X	-2.614	-2.758 to -2.470	Yes
6007	4:50 X vs. 40:40 X	-0.6847	-0.8286 to -0.5407	Yes
6008	4:50 X vs. 40:50 X	-0.5320	-0.6760 to -0.3880	Yes
6009	4:50 X vs. 41:0 X	-3.524	-3.668 to -3.380	Yes
6010	4:50 X vs. 41:1 X	-3.523	-3.667 to -3.379	Yes
6011	4:50 X vs. 41:10 X	-2.668	-2.812 to -2.524	Yes
6012	4:50 X vs. 41:40 X	-0.6320	-0.7760 to -0.4880	Yes
6013	4:50 X vs. 41:50 X	-0.5447	-0.6886 to -0.4007	Yes
6014	4:50 X vs. 42:0 X	-3.666	-3.810 to -3.522	Yes
6015	4:50 X vs. 42:1 X	-3.686	-3.830 to -3.542	Yes
6016	4:50 X vs. 42:10 X	-2.668	-2.812 to -2.524	Yes
6017	4:50 X vs. 42:40 X	-0.6447	-0.7886 to -0.5007	Yes
6018	4:50 X vs. 42:50 X	-0.5480	-0.6920 to -0.4040	Yes
6019	4:50 X vs. 43:0 X	-3.756	-3.900 to -3.612	Yes
6020	4:50 X vs. 43:1 X	-3.670	-3.814 to -3.526	Yes
6021	4:50 X vs. 43:10 X	-3.386	-3.530 to -3.242	Yes
6022	4:50 X vs. 43:40 X	-0.6430	-0.7870 to -0.4990	Yes
6023	4:50 X vs. 43:50 X	-0.5483	-0.6923 to -0.4044	Yes
6024	4:50 X vs. 44:0 X	-3.780	-3.924 to -3.636	Yes
6025	4:50 X vs. 44:1 X	-3.670	-3.814 to -3.526	Yes
6026	4:50 X vs. 44:10 X	-3.449	-3.593 to -3.305	Yes
6027	4:50 X vs. 44:40 X	-0.6450	-0.7890 to -0.5010	Yes
6028	4:50 X vs. 44:50 X	-0.4807	-0.6246 to -0.3367	Yes
6029	4:50 X vs. 45:0 X	-3.645	-3.789 to -3.501	Yes
6030	4:50 X vs. 45:1 X	-3.447	-3.591 to -3.303	Yes

	2way ANOVA Multiple comparisons	A Data Set-A Y	B Data Set-B Y	C Data Set-C Y
6031	4:50 X vs. 45:10 X	-3.329	-3.473 to -3.185	Yes
6032	4:50 X vs. 45:40 X	-0.6463	-0.7903 to -0.5024	Yes
6033	4:50 X vs. 45:50 X	-0.4777	-0.6216 to -0.3337	Yes
6034	4:50 X vs. 46:0 X	-3.449	-3.593 to -3.305	Yes
6035	4:50 X vs. 46:1 X	-3.416	-3.560 to -3.272	Yes
6036	4:50 X vs. 46:10 X	-3.324	-3.468 to -3.180	Yes
6037	4:50 X vs. 46:40 X	-0.6600	-0.8040 to -0.5160	Yes
6038	4:50 X vs. 46:50 X	-0.4707	-0.6146 to -0.3267	Yes
6039	4:50 X vs. 47:0 X	-3.425	-3.569 to -3.281	Yes
6040	4:50 X vs. 47:1 X	-3.329	-3.473 to -3.185	Yes
6041	4:50 X vs. 47:10 X	-3.310	-3.454 to -3.166	Yes
6042	4:50 X vs. 47:40 X	-0.6700	-0.8140 to -0.5260	Yes
6043	4:50 X vs. 47:50 X	-0.4287	-0.5726 to -0.2847	Yes
6044	4:50 X vs. 48:0 X	-3.412	-3.573 to -3.251	Yes
6045	4:50 X vs. 48:1 X	-3.316	-3.460 to -3.172	Yes
6046	4:50 X vs. 48:10 X	-3.310	-3.454 to -3.166	Yes
6047	4:50 X vs. 48:40 X	-0.6763	-0.8203 to -0.5324	Yes
6048	4:50 X vs. 48:50 X	-0.4360	-0.5800 to -0.2920	Yes
6049	4:50 X vs. 49:0 X	-3.412	-3.556 to -3.268	Yes
6050	4:50 X vs. 49:1 X	-3.316	-3.460 to -3.172	Yes
6051	4:50 X vs. 49:10 X	-3.310	-3.454 to -3.166	Yes
6052	4:50 X vs. 49:40 X	-0.6807	-0.8246 to -0.5367	Yes
6053	4:50 X vs. 49:50 X	-0.4353	-0.5793 to -0.2914	Yes
6054	4:50 X vs. 50:0 X	-3.412	-3.556 to -3.268	Yes
6055	4:50 X vs. 50:1 X	-3.316	-3.460 to -3.172	Yes
6056	4:50 X vs. 50:10 X	-3.310	-3.454 to -3.166	Yes
6057	4:50 X vs. 50:40 X	-0.6883	-0.8323 to -0.5444	Yes
6058	4:50 X vs. 50:50 X	-0.4350	-0.5790 to -0.2910	Yes
6059	5:0 X vs. 5:1 X	0.0020	-0.1420 to 0.1460	No
6060	5:0 X vs. 5:10 X	-0.0130	-0.1570 to 0.1310	No
6061	5:0 X vs. 5:40 X	0.0080	-0.1360 to 0.1520	No
6062	5:0 X vs. 5:50 X	0.0090	-0.1350 to 0.1530	No
6063	5:0 X vs. 6:0 X	0.0006667	-0.1433 to 0.1446	No
6064	5:0 X vs. 6:1 X	0.001667	-0.1423 to 0.1456	No
6065	5:0 X vs. 6:10 X	-0.0130	-0.1570 to 0.1310	No
6066	5:0 X vs. 6:40 X	0.005667	-0.1383 to 0.1496	No
6067	5:0 X vs. 6:50 X	0.007333	-0.1366 to 0.1513	No
6068	5:0 X vs. 7:0 X	-0.001333	-0.1453 to 0.1426	No
6069	5:0 X vs. 7:1 X	0.007333	-0.1366 to 0.1513	No
6070	5:0 X vs. 7:10 X	-0.004667	-0.1486 to 0.1393	No
6071	5:0 X vs. 7:40 X	0.0040	-0.1400 to 0.1480	No
6072	5:0 X vs. 7:50 X	0.0060	-0.1380 to 0.1500	No
6073	5:0 X vs. 8:0 X	-0.006000	-0.1500 to 0.1380	No
6074	5:0 X vs. 8:1 X	0.007333	-0.1366 to 0.1513	No
6075	5:0 X vs. 8:10 X	6.209e-010	-0.1440 to 0.1440	No

	2way ANOVA<br>Multiple comparisons	A<br>Data Set-A<br>Y	B<br>Data Set-B<br>Y	C<br>Data Set-C<br>Y
6076	5:0 X vs. 8:40 X	-0.0006667	-0.1446 to 0.1433	No
6077	5:0 X vs. 8:50 X	0.002333	-0.1416 to 0.1463	No
6078	5:0 X vs. 9:0 X	-0.0140	-0.1580 to 0.1300	No
6079	5:0 X vs. 9:1 X	-0.0220	-0.1660 to 0.1220	No
6080	5:0 X vs. 9:10 X	-0.0230	-0.1670 to 0.1210	No
6081	5:0 X vs. 9:40 X	-0.005333	-0.1493 to 0.1386	No
6082	5:0 X vs. 9:50 X	0.001333	-0.1426 to 0.1453	No
6083	5:0 X vs. 10:0 X	-0.01567	-0.1596 to 0.1283	No
6084	5:0 X vs. 10:1 X	-0.02267	-0.1666 to 0.1213	No
6085	5:0 X vs. 10:10 X	-0.02533	-0.1693 to 0.1186	No
6086	5:0 X vs. 10:40 X	-0.0150	-0.1590 to 0.1290	No
6087	5:0 X vs. 10:50 X	-0.001667	-0.1456 to 0.1423	No
6088	5:0 X vs. 11:0 X	-0.02533	-0.1693 to 0.1186	No
6089	5:0 X vs. 11:1 X	-0.0270	-0.1710 to 0.1170	No
6090	5:0 X vs. 11:10 X	-0.02733	-0.1713 to 0.1166	No
6091	5:0 X vs. 11:40 X	-0.0190	-0.1630 to 0.1250	No
6092	5:0 X vs. 11:50 X	-0.007000	-0.1510 to 0.1370	No
6093	5:0 X vs. 12:0 X	-0.0260	-0.1700 to 0.1180	No
6094	5:0 X vs. 12:1 X	-0.008333	-0.1523 to 0.1356	No
6095	5:0 X vs. 12:10 X	-0.0260	-0.1700 to 0.1180	No
6096	5:0 X vs. 12:40 X	-0.01233	-0.1563 to 0.1316	No
6097	5:0 X vs. 12:50 X	-0.01567	-0.1596 to 0.1283	No
6098	5:0 X vs. 13:0 X	-0.0200	-0.1640 to 0.1240	No
6099	5:0 X vs. 13:1 X	-0.007333	-0.1513 to 0.1366	No
6100	5:0 X vs. 13:10 X	-0.02933	-0.1733 to 0.1146	No
6101	5:0 X vs. 13:40 X	-0.02567	-0.1696 to 0.1183	No
6102	5:0 X vs. 13:50 X	-0.02667	-0.1706 to 0.1173	No
6103	5:0 X vs. 14:0 X	-0.02867	-0.1726 to 0.1153	No
6104	5:0 X vs. 14:1 X	-0.02033	-0.1643 to 0.1236	No
6105	5:0 X vs. 14:10 X	-0.03433	-0.1783 to 0.1096	No
6106	5:0 X vs. 14:40 X	-0.0310	-0.1750 to 0.1130	No
6107	5:0 X vs. 14:50 X	-0.0350	-0.1790 to 0.1090	No
6108	5:0 X vs. 15:0 X	-0.0410	-0.1850 to 0.1030	No
6109	5:0 X vs. 15:1 X	-0.0210	-0.1650 to 0.1230	No
6110	5:0 X vs. 15:10 X	-0.04833	-0.1923 to 0.09563	No
6111	5:0 X vs. 15:40 X	-0.03067	-0.1746 to 0.1133	No
6112	5:0 X vs. 15:50 X	-0.04833	-0.1923 to 0.09563	No
6113	5:0 X vs. 16:0 X	-0.1333	-0.2773 to 0.01063	No
6114	5:0 X vs. 16:1 X	-0.02433	-0.1683 to 0.1196	No
6115	5:0 X vs. 16:10 X	-0.0500	-0.1940 to 0.09396	No
6116	5:0 X vs. 16:40 X	-0.06933	-0.2133 to 0.07463	No
6117	5:0 X vs. 16:50 X	-0.1117	-0.2556 to 0.03229	No
6118	5:0 X vs. 17:0 X	-0.1860	-0.3300 to -0.04204	Yes
6119	5:0 X vs. 17:1 X	-0.1003	-0.2443 to 0.04363	No
6120	5:0 X vs. 17:10 X	-0.1570	-0.3010 to -0.01304	Yes

	2way ANOVA<br>Multiple comparisons	A<br>Data Set-A<br>Y	B<br>Data Set-B<br>Y	C<br>Data Set-C<br>Y
6121	5:0 X vs. 17:40 X	-0.08033	-0.2243 to 0.06363	No
6122	5:0 X vs. 17:50 X	-0.1257	-0.2696 to 0.01829	No
6123	5:0 X vs. 18:0 X	-0.2347	-0.3786 to -0.09071	Yes
6124	5:0 X vs. 18:1 X	-0.1430	-0.2870 to 0.0009598	No
6125	5:0 X vs. 18:10 X	-0.2353	-0.3793 to -0.09137	Yes
6126	5:0 X vs. 18:40 X	-0.08467	-0.2286 to 0.05929	No
6127	5:0 X vs. 18:50 X	-0.1297	-0.2736 to 0.01429	No
6128	5:0 X vs. 19:0 X	-0.2697	-0.4136 to -0.1257	Yes
6129	5:0 X vs. 19:1 X	-0.2577	-0.4016 to -0.1137	Yes
6130	5:0 X vs. 19:10 X	-0.2553	-0.3993 to -0.1114	Yes
6131	5:0 X vs. 19:40 X	-0.1133	-0.2573 to 0.03063	No
6132	5:0 X vs. 19:50 X	-0.1243	-0.2683 to 0.01963	No
6133	5:0 X vs. 20:0 X	-0.3493	-0.4933 to -0.2054	Yes
6134	5:0 X vs. 20:1 X	-0.1687	-0.3126 to -0.02471	Yes
6135	5:0 X vs. 20:10 X	-0.2577	-0.4016 to -0.1137	Yes
6136	5:0 X vs. 20:40 X	-0.1250	-0.2690 to 0.01896	No
6137	5:0 X vs. 20:50 X	-0.1690	-0.3130 to -0.02504	Yes
6138	5:0 X vs. 21:0 X	-0.3727	-0.5166 to -0.2287	Yes
6139	5:0 X vs. 21:1 X	-0.3257	-0.4696 to -0.1817	Yes
6140	5:0 X vs. 21:10 X	-0.3227	-0.4666 to -0.1787	Yes
6141	5:0 X vs. 21:40 X	-0.1980	-0.3420 to -0.05404	Yes
6142	5:0 X vs. 21:50 X	-0.2883	-0.4323 to -0.1444	Yes
6143	5:0 X vs. 22:0 X	-0.4143	-0.5583 to -0.2704	Yes
6144	5:0 X vs. 22:1 X	-0.4090	-0.5530 to -0.2650	Yes
6145	5:0 X vs. 22:10 X	-0.4043	-0.5483 to -0.2604	Yes
6146	5:0 X vs. 22:40 X	-0.3047	-0.4486 to -0.1607	Yes
6147	5:0 X vs. 22:50 X	-0.3673	-0.5113 to -0.2234	Yes
6148	5:0 X vs. 23:0 X	-0.4797	-0.6236 to -0.3357	Yes
6149	5:0 X vs. 23:1 X	-0.6693	-0.8303 to -0.5084	Yes
6150	5:0 X vs. 23:10 X	-0.4190	-0.5630 to -0.2750	Yes
6151	5:0 X vs. 23:40 X	-0.2993	-0.4603 to -0.1384	Yes
6152	5:0 X vs. 23:50 X	-0.3333	-0.4773 to -0.1894	Yes
6153	5:0 X vs. 24:0 X	-0.5970	-0.7410 to -0.4530	Yes
6154	5:0 X vs. 24:1 X	-0.7533	-0.9143 to -0.5924	Yes
6155	5:0 X vs. 24:10 X	-0.5808	-0.7418 to -0.4199	Yes
6156	5:0 X vs. 24:40 X	-0.3157	-0.4596 to -0.1717	Yes
6157	5:0 X vs. 24:50 X	-0.2373	-0.3813 to -0.09337	Yes
6158	5:0 X vs. 25:0 X	-0.9343	-1.095 to -0.7734	Yes
6159	5:0 X vs. 25:1 X	-1.124	-1.285 to -0.9629	Yes
6160	5:0 X vs. 25:10 X	-0.4853	-0.6889 to -0.2817	Yes
6161	5:0 X vs. 25:40 X	-0.3177	-0.4616 to -0.1737	Yes
6162	5:0 X vs. 25:50 X	-0.2920	-0.4360 to -0.1480	Yes
6163	5:0 X vs. 26:0 X	-1.147	-1.308 to -0.9859	Yes
6164	5:0 X vs. 26:1 X	-1.260	-1.421 to -1.099	Yes
6165	5:0 X vs. 26:10 X	-0.9463	-1.107 to -0.7854	Yes

	2way ANOVA<br>Multiple comparisons	A<br>Data Set-A<br>Y	B<br>Data Set-B<br>Y	C<br>Data Set-C<br>Y
6166	5:0 X vs. 26:40 X	-0.3920	-0.5360 to -0.2480	Yes
6167	5:0 X vs. 26:50 X	-0.2523	-0.3963 to -0.1084	Yes
6168	5:0 X vs. 27:0 X	-1.335	-1.479 to -1.191	Yes
6169	5:0 X vs. 27:1 X	-1.425	-1.586 to -1.264	Yes
6170	5:0 X vs. 27:10 X	-1.237	-1.398 to -1.076	Yes
6171	5:0 X vs. 27:40 X	-0.3920	-0.5360 to -0.2480	Yes
6172	5:0 X vs. 27:50 X	-0.2517	-0.3956 to -0.1077	Yes
6173	5:0 X vs. 28:0 X	-1.284	-1.445 to -1.123	Yes
6174	5:0 X vs. 28:1 X	-1.325	-1.469 to -1.181	Yes
6175	5:0 X vs. 28:10 X	-1.126	-1.287 to -0.9654	Yes
6176	5:0 X vs. 28:40 X	-0.4043	-0.5483 to -0.2604	Yes
6177	5:0 X vs. 28:50 X	-0.2997	-0.4436 to -0.1557	Yes
6178	5:0 X vs. 29:0 X	-1.765	-1.926 to -1.604	Yes
6179	5:0 X vs. 29:1 X	-1.850	-2.011 to -1.689	Yes
6180	5:0 X vs. 29:10 X	-1.065	-1.226 to -0.9039	Yes
6181	5:0 X vs. 29:40 X	-0.5123	-0.6563 to -0.3684	Yes
6182	5:0 X vs. 29:50 X	-0.3010	-0.4450 to -0.1570	Yes
6183	5:0 X vs. 30:0 X	-1.480	-1.641 to -1.319	Yes
6184	5:0 X vs. 30:1 X	-1.801	-1.962 to -1.640	Yes
6185	5:0 X vs. 30:10 X	-1.139	-1.300 to -0.9784	Yes
6186	5:0 X vs. 30:40 X	-0.4393	-0.5833 to -0.2954	Yes
6187	5:0 X vs. 30:50 X	-0.3533	-0.4973 to -0.2094	Yes
6188	5:0 X vs. 31:0 X	-1.798	-1.959 to -1.637	Yes
6189	5:0 X vs. 31:1 X	-1.788	-1.949 to -1.627	Yes
6190	5:0 X vs. 31:10 X	-1.271	-1.432 to -1.110	Yes
6191	5:0 X vs. 31:40 X	-0.5310	-0.6750 to -0.3870	Yes
6192	5:0 X vs. 31:50 X	-0.3323	-0.4763 to -0.1884	Yes
6193	5:0 X vs. 32:0 X	-2.389	-2.533 to -2.245	Yes
6194	5:0 X vs. 32:1 X	-1.845	-2.006 to -1.684	Yes
6195	5:0 X vs. 32:10 X	-1.701	-1.845 to -1.557	Yes
6196	5:0 X vs. 32:40 X	-0.5273	-0.6713 to -0.3834	Yes
6197	5:0 X vs. 32:50 X	-0.3320	-0.4760 to -0.1880	Yes
6198	5:0 X vs. 33:0 X	-2.393	-2.537 to -2.249	Yes
6199	5:0 X vs. 33:1 X	-2.208	-2.352 to -2.064	Yes
6200	5:0 X vs. 33:10 X	-1.868	-2.012 to -1.724	Yes
6201	5:0 X vs. 33:40 X	-0.5460	-0.6900 to -0.4020	Yes
6202	5:0 X vs. 33:50 X	-0.3470	-0.4910 to -0.2030	Yes
6203	5:0 X vs. 34:0 X	-2.318	-2.462 to -2.174	Yes
6204	5:0 X vs. 34:1 X	-2.325	-2.469 to -2.181	Yes
6205	5:0 X vs. 34:10 X	-1.958	-2.102 to -1.814	Yes
6206	5:0 X vs. 34:40 X	-0.5580	-0.7020 to -0.4140	Yes
6207	5:0 X vs. 34:50 X	-0.4960	-0.6400 to -0.3520	Yes
6208	5:0 X vs. 35:0 X	-2.666	-2.810 to -2.522	Yes
6209	5:0 X vs. 35:1 X	-2.432	-2.576 to -2.288	Yes
6210	5:0 X vs. 35:10 X	-1.920	-2.064 to -1.776	Yes

	2way ANOVA Multiple comparisons	A Data Set-A Y	B Data Set-B Y	C Data Set-C Y
**6211**	5:**0 X** vs. 35:**40 X**	-0.5670	-0.7110 to -0.4230	Yes
**6212**	5:**0 X** vs. 35:**50 X**	-0.5087	-0.6526 to -0.3647	Yes
**6213**	5:**0 X** vs. 36:**0 X**	-2.873	-3.034 to -2.712	Yes
**6214**	5:**0 X** vs. 36:**1 X**	-2.569	-2.713 to -2.425	Yes
**6215**	5:**0 X** vs. 36:**10 X**	-1.926	-2.070 to -1.782	Yes
**6216**	5:**0 X** vs. 36:**40 X**	-0.6223	-0.7663 to -0.4784	Yes
**6217**	5:**0 X** vs. 36:**50 X**	-0.4720	-0.6160 to -0.3280	Yes
**6218**	5:**0 X** vs. 37:**0 X**	-3.257	-3.401 to -3.113	Yes
**6219**	5:**0 X** vs. 37:**1 X**	-2.639	-2.783 to -2.495	Yes
**6220**	5:**0 X** vs. 37:**10 X**	-1.927	-2.071 to -1.783	Yes
**6221**	5:**0 X** vs. 37:**40 X**	-0.6597	-0.8036 to -0.5157	Yes
**6222**	5:**0 X** vs. 37:**50 X**	-0.4807	-0.6246 to -0.3367	Yes
**6223**	5:**0 X** vs. 38:**0 X**	-3.202	-3.346 to -3.058	Yes
**6224**	5:**0 X** vs. 38:**1 X**	-2.757	-2.901 to -2.613	Yes
**6225**	5:**0 X** vs. 38:**10 X**	-2.203	-2.347 to -2.059	Yes
**6226**	5:**0 X** vs. 38:**40 X**	-0.6640	-0.8080 to -0.5200	Yes
**6227**	5:**0 X** vs. 38:**50 X**	-0.4847	-0.6286 to -0.3407	Yes
**6228**	5:**0 X** vs. 39:**0 X**	-3.432	-3.576 to -3.288	Yes
**6229**	5:**0 X** vs. 39:**1 X**	-3.324	-3.468 to -3.180	Yes
**6230**	5:**0 X** vs. 39:**10 X**	-2.348	-2.492 to -2.204	Yes
**6231**	5:**0 X** vs. 39:**40 X**	-0.6700	-0.8140 to -0.5260	Yes
**6232**	5:**0 X** vs. 39:**50 X**	-0.5157	-0.6596 to -0.3717	Yes
**6233**	5:**0 X** vs. 40:**0 X**	-3.473	-3.617 to -3.329	Yes
**6234**	5:**0 X** vs. 40:**1 X**	-3.399	-3.543 to -3.255	Yes
**6235**	5:**0 X** vs. 40:**10 X**	-2.604	-2.748 to -2.460	Yes
**6236**	5:**0 X** vs. 40:**40 X**	-0.6747	-0.8186 to -0.5307	Yes
**6237**	5:**0 X** vs. 40:**50 X**	-0.5220	-0.6660 to -0.3780	Yes
**6238**	5:**0 X** vs. 41:**0 X**	-3.514	-3.658 to -3.370	Yes
**6239**	5:**0 X** vs. 41:**1 X**	-3.513	-3.657 to -3.369	Yes
**6240**	5:**0 X** vs. 41:**10 X**	-2.658	-2.802 to -2.514	Yes
**6241**	5:**0 X** vs. 41:**40 X**	-0.6220	-0.7660 to -0.4780	Yes
**6242**	5:**0 X** vs. 41:**50 X**	-0.5347	-0.6786 to -0.3907	Yes
**6243**	5:**0 X** vs. 42:**0 X**	-3.656	-3.800 to -3.512	Yes
**6244**	5:**0 X** vs. 42:**1 X**	-3.676	-3.820 to -3.532	Yes
**6245**	5:**0 X** vs. 42:**10 X**	-2.658	-2.802 to -2.514	Yes
**6246**	5:**0 X** vs. 42:**40 X**	-0.6347	-0.7786 to -0.4907	Yes
**6247**	5:**0 X** vs. 42:**50 X**	-0.5380	-0.6820 to -0.3940	Yes
**6248**	5:**0 X** vs. 43:**0 X**	-3.746	-3.890 to -3.602	Yes
**6249**	5:**0 X** vs. 43:**1 X**	-3.660	-3.804 to -3.516	Yes
**6250**	5:**0 X** vs. 43:**10 X**	-3.376	-3.520 to -3.232	Yes
**6251**	5:**0 X** vs. 43:**40 X**	-0.6330	-0.7770 to -0.4890	Yes
**6252**	5:**0 X** vs. 43:**50 X**	-0.5383	-0.6823 to -0.3944	Yes
**6253**	5:**0 X** vs. 44:**0 X**	-3.770	-3.914 to -3.626	Yes
**6254**	5:**0 X** vs. 44:**1 X**	-3.660	-3.804 to -3.516	Yes
**6255**	5:**0 X** vs. 44:**10 X**	-3.439	-3.583 to -3.295	Yes

	2way ANOVA<br>Multiple comparisons	A<br>Data Set-A<br>Y	B<br>Data Set-B<br>Y	C<br>Data Set-C<br>Y
6256	5:0 X vs. 44:40 X	-0.6350	-0.7790 to -0.4910	Yes
6257	5:0 X vs. 44:50 X	-0.4707	-0.6146 to -0.3267	Yes
6258	5:0 X vs. 45:0 X	-3.635	-3.779 to -3.491	Yes
6259	5:0 X vs. 45:1 X	-3.437	-3.581 to -3.293	Yes
6260	5:0 X vs. 45:10 X	-3.319	-3.463 to -3.175	Yes
6261	5:0 X vs. 45:40 X	-0.6363	-0.7803 to -0.4924	Yes
6262	5:0 X vs. 45:50 X	-0.4677	-0.6116 to -0.3237	Yes
6263	5:0 X vs. 46:0 X	-3.439	-3.583 to -3.295	Yes
6264	5:0 X vs. 46:1 X	-3.406	-3.550 to -3.262	Yes
6265	5:0 X vs. 46:10 X	-3.314	-3.458 to -3.170	Yes
6266	5:0 X vs. 46:40 X	-0.6500	-0.7940 to -0.5060	Yes
6267	5:0 X vs. 46:50 X	-0.4607	-0.6046 to -0.3167	Yes
6268	5:0 X vs. 47:0 X	-3.415	-3.559 to -3.271	Yes
6269	5:0 X vs. 47:1 X	-3.319	-3.463 to -3.175	Yes
6270	5:0 X vs. 47:10 X	-3.300	-3.444 to -3.156	Yes
6271	5:0 X vs. 47:40 X	-0.6600	-0.8040 to -0.5160	Yes
6272	5:0 X vs. 47:50 X	-0.4187	-0.5626 to -0.2747	Yes
6273	5:0 X vs. 48:0 X	-3.402	-3.563 to -3.241	Yes
6274	5:0 X vs. 48:1 X	-3.306	-3.450 to -3.162	Yes
6275	5:0 X vs. 48:10 X	-3.300	-3.444 to -3.156	Yes
6276	5:0 X vs. 48:40 X	-0.6663	-0.8103 to -0.5224	Yes
6277	5:0 X vs. 48:50 X	-0.4260	-0.5700 to -0.2820	Yes
6278	5:0 X vs. 49:0 X	-3.402	-3.546 to -3.258	Yes
6279	5:0 X vs. 49:1 X	-3.306	-3.450 to -3.162	Yes
6280	5:0 X vs. 49:10 X	-3.300	-3.444 to -3.156	Yes
6281	5:0 X vs. 49:40 X	-0.6707	-0.8146 to -0.5267	Yes
6282	5:0 X vs. 49:50 X	-0.4253	-0.5693 to -0.2814	Yes
6283	5:0 X vs. 50:0 X	-3.402	-3.546 to -3.258	Yes
6284	5:0 X vs. 50:1 X	-3.306	-3.450 to -3.162	Yes
6285	5:0 X vs. 50:10 X	-3.300	-3.444 to -3.156	Yes
6286	5:0 X vs. 50:40 X	-0.6783	-0.8223 to -0.5344	Yes
6287	5:0 X vs. 50:50 X	-0.4250	-0.5690 to -0.2810	Yes
6288	5:1 X vs. 5:10 X	-0.0150	-0.1590 to 0.1290	No
6289	5:1 X vs. 5:40 X	0.0060	-0.1380 to 0.1500	No
6290	5:1 X vs. 5:50 X	0.0070	-0.1370 to 0.1510	No
6291	5:1 X vs. 6:0 X	-0.001333	-0.1453 to 0.1426	No
6292	5:1 X vs. 6:1 X	-0.0003333	-0.1443 to 0.1436	No
6293	5:1 X vs. 6:10 X	-0.0150	-0.1590 to 0.1290	No
6294	5:1 X vs. 6:40 X	0.003667	-0.1403 to 0.1476	No
6295	5:1 X vs. 6:50 X	0.005333	-0.1386 to 0.1493	No
6296	5:1 X vs. 7:0 X	-0.003333	-0.1473 to 0.1406	No
6297	5:1 X vs. 7:1 X	0.005333	-0.1386 to 0.1493	No
6298	5:1 X vs. 7:10 X	-0.006667	-0.1506 to 0.1373	No
6299	5:1 X vs. 7:40 X	0.002000	-0.1420 to 0.1460	No
6300	5:1 X vs. 7:50 X	0.004000	-0.1400 to 0.1480	No

	2way ANOVA<br>Multiple comparisons	A<br>Data Set-A<br>Y	B<br>Data Set-B<br>Y	C<br>Data Set-C<br>Y
**6301**	5:**1 X** vs. 8:**0 X**	-0.0080	-0.1520 to 0.1360	No
**6302**	5:**1 X** vs. 8:**1 X**	0.005333	-0.1386 to 0.1493	No
**6303**	5:**1 X** vs. 8:**10 X**	-0.0020	-0.1460 to 0.1420	No
**6304**	5:**1 X** vs. 8:**40 X**	-0.002667	-0.1466 to 0.1413	No
**6305**	5:**1 X** vs. 8:**50 X**	0.0003333	-0.1436 to 0.1443	No
**6306**	5:**1 X** vs. 9:**0 X**	-0.0160	-0.1600 to 0.1280	No
**6307**	5:**1 X** vs. 9:**1 X**	-0.0240	-0.1680 to 0.1200	No
**6308**	5:**1 X** vs. 9:**10 X**	-0.0250	-0.1690 to 0.1190	No
**6309**	5:**1 X** vs. 9:**40 X**	-0.007333	-0.1513 to 0.1366	No
**6310**	5:**1 X** vs. 9:**50 X**	-0.0006667	-0.1446 to 0.1433	No
**6311**	5:**1 X** vs. 10:**0 X**	-0.01767	-0.1616 to 0.1263	No
**6312**	5:**1 X** vs. 10:**1 X**	-0.02467	-0.1686 to 0.1193	No
**6313**	5:**1 X** vs. 10:**10 X**	-0.02733	-0.1713 to 0.1166	No
**6314**	5:**1 X** vs. 10:**40 X**	-0.0170	-0.1610 to 0.1270	No
**6315**	5:**1 X** vs. 10:**50 X**	-0.003667	-0.1476 to 0.1403	No
**6316**	5:**1 X** vs. 11:**0 X**	-0.02733	-0.1713 to 0.1166	No
**6317**	5:**1 X** vs. 11:**1 X**	-0.0290	-0.1730 to 0.1150	No
**6318**	5:**1 X** vs. 11:**10 X**	-0.02933	-0.1733 to 0.1146	No
**6319**	5:**1 X** vs. 11:**40 X**	-0.0210	-0.1650 to 0.1230	No
**6320**	5:**1 X** vs. 11:**50 X**	-0.009000	-0.1530 to 0.1350	No
**6321**	5:**1 X** vs. 12:**0 X**	-0.0280	-0.1720 to 0.1160	No
**6322**	5:**1 X** vs. 12:**1 X**	-0.01033	-0.1543 to 0.1336	No
**6323**	5:**1 X** vs. 12:**10 X**	-0.0280	-0.1720 to 0.1160	No
**6324**	5:**1 X** vs. 12:**40 X**	-0.01433	-0.1583 to 0.1296	No
**6325**	5:**1 X** vs. 12:**50 X**	-0.01767	-0.1616 to 0.1263	No
**6326**	5:**1 X** vs. 13:**0 X**	-0.0220	-0.1660 to 0.1220	No
**6327**	5:**1 X** vs. 13:**1 X**	-0.009333	-0.1533 to 0.1346	No
**6328**	5:**1 X** vs. 13:**10 X**	-0.03133	-0.1753 to 0.1126	No
**6329**	5:**1 X** vs. 13:**40 X**	-0.02767	-0.1716 to 0.1163	No
**6330**	5:**1 X** vs. 13:**50 X**	-0.02867	-0.1726 to 0.1153	No
**6331**	5:**1 X** vs. 14:**0 X**	-0.03067	-0.1746 to 0.1133	No
**6332**	5:**1 X** vs. 14:**1 X**	-0.02233	-0.1663 to 0.1216	No
**6333**	5:**1 X** vs. 14:**10 X**	-0.03633	-0.1803 to 0.1076	No
**6334**	5:**1 X** vs. 14:**40 X**	-0.0330	-0.1770 to 0.1110	No
**6335**	5:**1 X** vs. 14:**50 X**	-0.0370	-0.1810 to 0.1070	No
**6336**	5:**1 X** vs. 15:**0 X**	-0.0430	-0.1870 to 0.1010	No
**6337**	5:**1 X** vs. 15:**1 X**	-0.0230	-0.1670 to 0.1210	No
**6338**	5:**1 X** vs. 15:**10 X**	-0.05033	-0.1943 to 0.09363	No
**6339**	5:**1 X** vs. 15:**40 X**	-0.03267	-0.1766 to 0.1113	No
**6340**	5:**1 X** vs. 15:**50 X**	-0.05033	-0.1943 to 0.09363	No
**6341**	5:**1 X** vs. 16:**0 X**	-0.1353	-0.2793 to 0.008626	No
**6342**	5:**1 X** vs. 16:**1 X**	-0.02633	-0.1703 to 0.1176	No
**6343**	5:**1 X** vs. 16:**10 X**	-0.0520	-0.1960 to 0.09196	No
**6344**	5:**1 X** vs. 16:**40 X**	-0.07133	-0.2153 to 0.07263	No
**6345**	5:**1 X** vs. 16:**50 X**	-0.1137	-0.2576 to 0.03029	No

	2way ANOVA<br>Multiple comparisons	A<br>Data Set-A<br>Y	B<br>Data Set-B<br>Y	C<br>Data Set-C<br>Y
6346	5:**1 X** vs. 17:**0 X**	-0.1880	-0.3320 to -0.04404	Yes
6347	5:**1 X** vs. 17:**1 X**	-0.1023	-0.2463 to 0.04163	No
6348	5:**1 X** vs. 17:**10 X**	-0.1590	-0.3030 to -0.01504	Yes
6349	5:**1 X** vs. 17:**40 X**	-0.08233	-0.2263 to 0.06163	No
6350	5:**1 X** vs. 17:**50 X**	-0.1277	-0.2716 to 0.01629	No
6351	5:**1 X** vs. 18:**0 X**	-0.2367	-0.3806 to -0.09271	Yes
6352	5:**1 X** vs. 18:**1 X**	-0.1450	-0.2890 to -0.001040	Yes
6353	5:**1 X** vs. 18:**10 X**	-0.2373	-0.3813 to -0.09337	Yes
6354	5:**1 X** vs. 18:**40 X**	-0.08667	-0.2306 to 0.05729	No
6355	5:**1 X** vs. 18:**50 X**	-0.1317	-0.2756 to 0.01229	No
6356	5:**1 X** vs. 19:**0 X**	-0.2717	-0.4156 to -0.1277	Yes
6357	5:**1 X** vs. 19:**1 X**	-0.2597	-0.4036 to -0.1157	Yes
6358	5:**1 X** vs. 19:**10 X**	-0.2573	-0.4013 to -0.1134	Yes
6359	5:**1 X** vs. 19:**40 X**	-0.1153	-0.2593 to 0.02863	No
6360	5:**1 X** vs. 19:**50 X**	-0.1263	-0.2703 to 0.01763	No
6361	5:**1 X** vs. 20:**0 X**	-0.3513	-0.4953 to -0.2074	Yes
6362	5:**1 X** vs. 20:**1 X**	-0.1707	-0.3146 to -0.02671	Yes
6363	5:**1 X** vs. 20:**10 X**	-0.2597	-0.4036 to -0.1157	Yes
6364	5:**1 X** vs. 20:**40 X**	-0.1270	-0.2710 to 0.01696	No
6365	5:**1 X** vs. 20:**50 X**	-0.1710	-0.3150 to -0.02704	Yes
6366	5:**1 X** vs. 21:**0 X**	-0.3747	-0.5186 to -0.2307	Yes
6367	5:**1 X** vs. 21:**1 X**	-0.3277	-0.4716 to -0.1837	Yes
6368	5:**1 X** vs. 21:**10 X**	-0.3247	-0.4686 to -0.1807	Yes
6369	5:**1 X** vs. 21:**40 X**	-0.2000	-0.3440 to -0.05604	Yes
6370	5:**1 X** vs. 21:**50 X**	-0.2903	-0.4343 to -0.1464	Yes
6371	5:**1 X** vs. 22:**0 X**	-0.4163	-0.5603 to -0.2724	Yes
6372	5:**1 X** vs. 22:**1 X**	-0.4110	-0.5550 to -0.2670	Yes
6373	5:**1 X** vs. 22:**10 X**	-0.4063	-0.5503 to -0.2624	Yes
6374	5:**1 X** vs. 22:**40 X**	-0.3067	-0.4506 to -0.1627	Yes
6375	5:**1 X** vs. 22:**50 X**	-0.3693	-0.5133 to -0.2254	Yes
6376	5:**1 X** vs. 23:**0 X**	-0.4817	-0.6256 to -0.3377	Yes
6377	5:**1 X** vs. 23:**1 X**	-0.6713	-0.8323 to -0.5104	Yes
6378	5:**1 X** vs. 23:**10 X**	-0.4210	-0.5650 to -0.2770	Yes
6379	5:**1 X** vs. 23:**40 X**	-0.3013	-0.4623 to -0.1404	Yes
6380	5:**1 X** vs. 23:**50 X**	-0.3353	-0.4793 to -0.1914	Yes
6381	5:**1 X** vs. 24:**0 X**	-0.5990	-0.7430 to -0.4550	Yes
6382	5:**1 X** vs. 24:**1 X**	-0.7553	-0.9163 to -0.5944	Yes
6383	5:**1 X** vs. 24:**10 X**	-0.5828	-0.7438 to -0.4219	Yes
6384	5:**1 X** vs. 24:**40 X**	-0.3177	-0.4616 to -0.1737	Yes
6385	5:**1 X** vs. 24:**50 X**	-0.2393	-0.3833 to -0.09537	Yes
6386	5:**1 X** vs. 25:**0 X**	-0.9363	-1.097 to -0.7754	Yes
6387	5:**1 X** vs. 25:**1 X**	-1.126	-1.287 to -0.9649	Yes
6388	5:**1 X** vs. 25:**10 X**	-0.4873	-0.6909 to -0.2837	Yes
6389	5:**1 X** vs. 25:**40 X**	-0.3197	-0.4636 to -0.1757	Yes
6390	5:**1 X** vs. 25:**50 X**	-0.2940	-0.4380 to -0.1500	Yes

	2way ANOVA<br>Multiple comparisons	A<br>Data Set-A<br>Y	B<br>Data Set-B<br>Y	C<br>Data Set-C<br>Y
6391	5:1 X vs. 26:0 X	-1.149	-1.310 to -0.9879	Yes
6392	5:1 X vs. 26:1 X	-1.262	-1.423 to -1.101	Yes
6393	5:1 X vs. 26:10 X	-0.9483	-1.109 to -0.7874	Yes
6394	5:1 X vs. 26:40 X	-0.3940	-0.5380 to -0.2500	Yes
6395	5:1 X vs. 26:50 X	-0.2543	-0.3983 to -0.1104	Yes
6396	5:1 X vs. 27:0 X	-1.337	-1.481 to -1.193	Yes
6397	5:1 X vs. 27:1 X	-1.427	-1.588 to -1.266	Yes
6398	5:1 X vs. 27:10 X	-1.239	-1.400 to -1.078	Yes
6399	5:1 X vs. 27:40 X	-0.3940	-0.5380 to -0.2500	Yes
6400	5:1 X vs. 27:50 X	-0.2537	-0.3976 to -0.1097	Yes
6401	5:1 X vs. 28:0 X	-1.286	-1.447 to -1.125	Yes
6402	5:1 X vs. 28:1 X	-1.327	-1.471 to -1.183	Yes
6403	5:1 X vs. 28:10 X	-1.128	-1.289 to -0.9674	Yes
6404	5:1 X vs. 28:40 X	-0.4063	-0.5503 to -0.2624	Yes
6405	5:1 X vs. 28:50 X	-0.3017	-0.4456 to -0.1577	Yes
6406	5:1 X vs. 29:0 X	-1.767	-1.928 to -1.606	Yes
6407	5:1 X vs. 29:1 X	-1.852	-2.013 to -1.691	Yes
6408	5:1 X vs. 29:10 X	-1.067	-1.228 to -0.9059	Yes
6409	5:1 X vs. 29:40 X	-0.5143	-0.6583 to -0.3704	Yes
6410	5:1 X vs. 29:50 X	-0.3030	-0.4470 to -0.1590	Yes
6411	5:1 X vs. 30:0 X	-1.482	-1.643 to -1.321	Yes
6412	5:1 X vs. 30:1 X	-1.803	-1.964 to -1.642	Yes
6413	5:1 X vs. 30:10 X	-1.141	-1.302 to -0.9804	Yes
6414	5:1 X vs. 30:40 X	-0.4413	-0.5853 to -0.2974	Yes
6415	5:1 X vs. 30:50 X	-0.3553	-0.4993 to -0.2114	Yes
6416	5:1 X vs. 31:0 X	-1.800	-1.961 to -1.639	Yes
6417	5:1 X vs. 31:1 X	-1.790	-1.951 to -1.629	Yes
6418	5:1 X vs. 31:10 X	-1.273	-1.434 to -1.112	Yes
6419	5:1 X vs. 31:40 X	-0.5330	-0.6770 to -0.3890	Yes
6420	5:1 X vs. 31:50 X	-0.3343	-0.4783 to -0.1904	Yes
6421	5:1 X vs. 32:0 X	-2.391	-2.535 to -2.247	Yes
6422	5:1 X vs. 32:1 X	-1.847	-2.008 to -1.686	Yes
6423	5:1 X vs. 32:10 X	-1.703	-1.847 to -1.559	Yes
6424	5:1 X vs. 32:40 X	-0.5293	-0.6733 to -0.3854	Yes
6425	5:1 X vs. 32:50 X	-0.3340	-0.4780 to -0.1900	Yes
6426	5:1 X vs. 33:0 X	-2.395	-2.539 to -2.251	Yes
6427	5:1 X vs. 33:1 X	-2.210	-2.354 to -2.066	Yes
6428	5:1 X vs. 33:10 X	-1.870	-2.014 to -1.726	Yes
6429	5:1 X vs. 33:40 X	-0.5480	-0.6920 to -0.4040	Yes
6430	5:1 X vs. 33:50 X	-0.3490	-0.4930 to -0.2050	Yes
6431	5:1 X vs. 34:0 X	-2.320	-2.464 to -2.176	Yes
6432	5:1 X vs. 34:1 X	-2.327	-2.471 to -2.183	Yes
6433	5:1 X vs. 34:10 X	-1.960	-2.104 to -1.816	Yes
6434	5:1 X vs. 34:40 X	-0.5600	-0.7040 to -0.4160	Yes
6435	5:1 X vs. 34:50 X	-0.4980	-0.6420 to -0.3540	Yes

	2way ANOVA<br>Multiple comparisons	A<br>Data Set-A<br>Y	B<br>Data Set-B<br>Y	C<br>Data Set-C<br>Y
**6436**	5:**1 X** vs. 35:**0 X**	-2.668	-2.812 to -2.524	Yes
**6437**	5:**1 X** vs. 35:**1 X**	-2.434	-2.578 to -2.290	Yes
**6438**	5:**1 X** vs. 35:**10 X**	-1.922	-2.066 to -1.778	Yes
**6439**	5:**1 X** vs. 35:**40 X**	-0.5690	-0.7130 to -0.4250	Yes
**6440**	5:**1 X** vs. 35:**50 X**	-0.5107	-0.6546 to -0.3667	Yes
**6441**	5:**1 X** vs. 36:**0 X**	-2.875	-3.036 to -2.714	Yes
**6442**	5:**1 X** vs. 36:**1 X**	-2.571	-2.715 to -2.427	Yes
**6443**	5:**1 X** vs. 36:**10 X**	-1.928	-2.072 to -1.784	Yes
**6444**	5:**1 X** vs. 36:**40 X**	-0.6243	-0.7683 to -0.4804	Yes
**6445**	5:**1 X** vs. 36:**50 X**	-0.4740	-0.6180 to -0.3300	Yes
**6446**	5:**1 X** vs. 37:**0 X**	-3.259	-3.403 to -3.115	Yes
**6447**	5:**1 X** vs. 37:**1 X**	-2.641	-2.785 to -2.497	Yes
**6448**	5:**1 X** vs. 37:**10 X**	-1.929	-2.073 to -1.785	Yes
**6449**	5:**1 X** vs. 37:**40 X**	-0.6617	-0.8056 to -0.5177	Yes
**6450**	5:**1 X** vs. 37:**50 X**	-0.4827	-0.6266 to -0.3387	Yes
**6451**	5:**1 X** vs. 38:**0 X**	-3.204	-3.348 to -3.060	Yes
**6452**	5:**1 X** vs. 38:**1 X**	-2.759	-2.903 to -2.615	Yes
**6453**	5:**1 X** vs. 38:**10 X**	-2.205	-2.349 to -2.061	Yes
**6454**	5:**1 X** vs. 38:**40 X**	-0.6660	-0.8100 to -0.5220	Yes
**6455**	5:**1 X** vs. 38:**50 X**	-0.4867	-0.6306 to -0.3427	Yes
**6456**	5:**1 X** vs. 39:**0 X**	-3.434	-3.578 to -3.290	Yes
**6457**	5:**1 X** vs. 39:**1 X**	-3.326	-3.470 to -3.182	Yes
**6458**	5:**1 X** vs. 39:**10 X**	-2.350	-2.494 to -2.206	Yes
**6459**	5:**1 X** vs. 39:**40 X**	-0.6720	-0.8160 to -0.5280	Yes
**6460**	5:**1 X** vs. 39:**50 X**	-0.5177	-0.6616 to -0.3737	Yes
**6461**	5:**1 X** vs. 40:**0 X**	-3.475	-3.619 to -3.331	Yes
**6462**	5:**1 X** vs. 40:**1 X**	-3.401	-3.545 to -3.257	Yes
**6463**	5:**1 X** vs. 40:**10 X**	-2.606	-2.750 to -2.462	Yes
**6464**	5:**1 X** vs. 40:**40 X**	-0.6767	-0.8206 to -0.5327	Yes
**6465**	5:**1 X** vs. 40:**50 X**	-0.5240	-0.6680 to -0.3800	Yes
**6466**	5:**1 X** vs. 41:**0 X**	-3.516	-3.660 to -3.372	Yes
**6467**	5:**1 X** vs. 41:**1 X**	-3.515	-3.659 to -3.371	Yes
**6468**	5:**1 X** vs. 41:**10 X**	-2.660	-2.804 to -2.516	Yes
**6469**	5:**1 X** vs. 41:**40 X**	-0.6240	-0.7680 to -0.4800	Yes
**6470**	5:**1 X** vs. 41:**50 X**	-0.5367	-0.6806 to -0.3927	Yes
**6471**	5:**1 X** vs. 42:**0 X**	-3.658	-3.802 to -3.514	Yes
**6472**	5:**1 X** vs. 42:**1 X**	-3.678	-3.822 to -3.534	Yes
**6473**	5:**1 X** vs. 42:**10 X**	-2.660	-2.804 to -2.516	Yes
**6474**	5:**1 X** vs. 42:**40 X**	-0.6367	-0.7806 to -0.4927	Yes
**6475**	5:**1 X** vs. 42:**50 X**	-0.5400	-0.6840 to -0.3960	Yes
**6476**	5:**1 X** vs. 43:**0 X**	-3.748	-3.892 to -3.604	Yes
**6477**	5:**1 X** vs. 43:**1 X**	-3.662	-3.806 to -3.518	Yes
**6478**	5:**1 X** vs. 43:**10 X**	-3.378	-3.522 to -3.234	Yes
**6479**	5:**1 X** vs. 43:**40 X**	-0.6350	-0.7790 to -0.4910	Yes
**6480**	5:**1 X** vs. 43:**50 X**	-0.5403	-0.6843 to -0.3964	Yes

	2way ANOVA<br>Multiple comparisons	A<br>Data Set-A<br>Y	B<br>Data Set-B<br>Y	C<br>Data Set-C<br>Y
**6481**	5:**1 X** vs. 44:**0 X**	-3.772	-3.916 to -3.628	Yes
**6482**	5:**1 X** vs. 44:**1 X**	-3.662	-3.806 to -3.518	Yes
**6483**	5:**1 X** vs. 44:**10 X**	-3.441	-3.585 to -3.297	Yes
**6484**	5:**1 X** vs. 44:**40 X**	-0.6370	-0.7810 to -0.4930	Yes
**6485**	5:**1 X** vs. 44:**50 X**	-0.4727	-0.6166 to -0.3287	Yes
**6486**	5:**1 X** vs. 45:**0 X**	-3.637	-3.781 to -3.493	Yes
**6487**	5:**1 X** vs. 45:**1 X**	-3.439	-3.583 to -3.295	Yes
**6488**	5:**1 X** vs. 45:**10 X**	-3.321	-3.465 to -3.177	Yes
**6489**	5:**1 X** vs. 45:**40 X**	-0.6383	-0.7823 to -0.4944	Yes
**6490**	5:**1 X** vs. 45:**50 X**	-0.4697	-0.6136 to -0.3257	Yes
**6491**	5:**1 X** vs. 46:**0 X**	-3.441	-3.585 to -3.297	Yes
**6492**	5:**1 X** vs. 46:**1 X**	-3.408	-3.552 to -3.264	Yes
**6493**	5:**1 X** vs. 46:**10 X**	-3.316	-3.460 to -3.172	Yes
**6494**	5:**1 X** vs. 46:**40 X**	-0.6520	-0.7960 to -0.5080	Yes
**6495**	5:**1 X** vs. 46:**50 X**	-0.4627	-0.6066 to -0.3187	Yes
**6496**	5:**1 X** vs. 47:**0 X**	-3.417	-3.561 to -3.273	Yes
**6497**	5:**1 X** vs. 47:**1 X**	-3.321	-3.465 to -3.177	Yes
**6498**	5:**1 X** vs. 47:**10 X**	-3.302	-3.446 to -3.158	Yes
**6499**	5:**1 X** vs. 47:**40 X**	-0.6620	-0.8060 to -0.5180	Yes
**6500**	5:**1 X** vs. 47:**50 X**	-0.4207	-0.5646 to -0.2767	Yes
**6501**	5:**1 X** vs. 48:**0 X**	-3.404	-3.565 to -3.243	Yes
**6502**	5:**1 X** vs. 48:**1 X**	-3.308	-3.452 to -3.164	Yes
**6503**	5:**1 X** vs. 48:**10 X**	-3.302	-3.446 to -3.158	Yes
**6504**	5:**1 X** vs. 48:**40 X**	-0.6683	-0.8123 to -0.5244	Yes
**6505**	5:**1 X** vs. 48:**50 X**	-0.4280	-0.5720 to -0.2840	Yes
**6506**	5:**1 X** vs. 49:**0 X**	-3.404	-3.548 to -3.260	Yes
**6507**	5:**1 X** vs. 49:**1 X**	-3.308	-3.452 to -3.164	Yes
**6508**	5:**1 X** vs. 49:**10 X**	-3.302	-3.446 to -3.158	Yes
**6509**	5:**1 X** vs. 49:**40 X**	-0.6727	-0.8166 to -0.5287	Yes
**6510**	5:**1 X** vs. 49:**50 X**	-0.4273	-0.5713 to -0.2834	Yes
**6511**	5:**1 X** vs. 50:**0 X**	-3.404	-3.548 to -3.260	Yes
**6512**	5:**1 X** vs. 50:**1 X**	-3.308	-3.452 to -3.164	Yes
**6513**	5:**1 X** vs. 50:**10 X**	-3.302	-3.446 to -3.158	Yes
**6514**	5:**1 X** vs. 50:**40 X**	-0.6803	-0.8243 to -0.5364	Yes
**6515**	5:**1 X** vs. 50:**50 X**	-0.4270	-0.5710 to -0.2830	Yes
**6516**	5:**10 X** vs. 5:**40 X**	0.0210	-0.1230 to 0.1650	No
**6517**	5:**10 X** vs. 5:**50 X**	0.0220	-0.1220 to 0.1660	No
**6518**	5:**10 X** vs. 6:**0 X**	0.01367	-0.1303 to 0.1576	No
**6519**	5:**10 X** vs. 6:**1 X**	0.01467	-0.1293 to 0.1586	No
**6520**	5:**10 X** vs. 6:**10 X**	0.0	-0.1440 to 0.1440	No
**6521**	5:**10 X** vs. 6:**40 X**	0.01867	-0.1253 to 0.1626	No
**6522**	5:**10 X** vs. 6:**50 X**	0.02033	-0.1236 to 0.1643	No
**6523**	5:**10 X** vs. 7:**0 X**	0.01167	-0.1323 to 0.1556	No
**6524**	5:**10 X** vs. 7:**1 X**	0.02033	-0.1236 to 0.1643	No
**6525**	5:**10 X** vs. 7:**10 X**	0.008333	-0.1356 to 0.1523	No

	2way ANOVA<br>Multiple comparisons	A<br>Data Set-A<br>Y	B<br>Data Set-B<br>Y	C<br>Data Set-C<br>Y
6526	5:10 X vs. 7:40 X	0.0170	-0.1270 to 0.1610	No
6527	5:10 X vs. 7:50 X	0.0190	-0.1250 to 0.1630	No
6528	5:10 X vs. 8:0 X	0.007000	-0.1370 to 0.1510	No
6529	5:10 X vs. 8:1 X	0.02033	-0.1236 to 0.1643	No
6530	5:10 X vs. 8:10 X	0.0130	-0.1310 to 0.1570	No
6531	5:10 X vs. 8:40 X	0.01233	-0.1316 to 0.1563	No
6532	5:10 X vs. 8:50 X	0.01533	-0.1286 to 0.1593	No
6533	5:10 X vs. 9:0 X	-0.001000	-0.1450 to 0.1430	No
6534	5:10 X vs. 9:1 X	-0.009000	-0.1530 to 0.1350	No
6535	5:10 X vs. 9:10 X	-0.0100	-0.1540 to 0.1340	No
6536	5:10 X vs. 9:40 X	0.007667	-0.1363 to 0.1516	No
6537	5:10 X vs. 9:50 X	0.01433	-0.1296 to 0.1583	No
6538	5:10 X vs. 10:0 X	-0.002667	-0.1466 to 0.1413	No
6539	5:10 X vs. 10:1 X	-0.009667	-0.1536 to 0.1343	No
6540	5:10 X vs. 10:10 X	-0.01233	-0.1563 to 0.1316	No
6541	5:10 X vs. 10:40 X	-0.002000	-0.1460 to 0.1420	No
6542	5:10 X vs. 10:50 X	0.01133	-0.1326 to 0.1553	No
6543	5:10 X vs. 11:0 X	-0.01233	-0.1563 to 0.1316	No
6544	5:10 X vs. 11:1 X	-0.0140	-0.1580 to 0.1300	No
6545	5:10 X vs. 11:10 X	-0.01433	-0.1583 to 0.1296	No
6546	5:10 X vs. 11:40 X	-0.0060	-0.1500 to 0.1380	No
6547	5:10 X vs. 11:50 X	0.006000	-0.1380 to 0.1500	No
6548	5:10 X vs. 12:0 X	-0.0130	-0.1570 to 0.1310	No
6549	5:10 X vs. 12:1 X	0.004667	-0.1393 to 0.1486	No
6550	5:10 X vs. 12:10 X	-0.0130	-0.1570 to 0.1310	No
6551	5:10 X vs. 12:40 X	0.0006667	-0.1433 to 0.1446	No
6552	5:10 X vs. 12:50 X	-0.002667	-0.1466 to 0.1413	No
6553	5:10 X vs. 13:0 X	-0.007000	-0.1510 to 0.1370	No
6554	5:10 X vs. 13:1 X	0.005667	-0.1383 to 0.1496	No
6555	5:10 X vs. 13:10 X	-0.01633	-0.1603 to 0.1276	No
6556	5:10 X vs. 13:40 X	-0.01267	-0.1566 to 0.1313	No
6557	5:10 X vs. 13:50 X	-0.01367	-0.1576 to 0.1303	No
6558	5:10 X vs. 14:0 X	-0.01567	-0.1596 to 0.1283	No
6559	5:10 X vs. 14:1 X	-0.007333	-0.1513 to 0.1366	No
6560	5:10 X vs. 14:10 X	-0.02133	-0.1653 to 0.1226	No
6561	5:10 X vs. 14:40 X	-0.0180	-0.1620 to 0.1260	No
6562	5:10 X vs. 14:50 X	-0.0220	-0.1660 to 0.1220	No
6563	5:10 X vs. 15:0 X	-0.0280	-0.1720 to 0.1160	No
6564	5:10 X vs. 15:1 X	-0.0080	-0.1520 to 0.1360	No
6565	5:10 X vs. 15:10 X	-0.03533	-0.1793 to 0.1086	No
6566	5:10 X vs. 15:40 X	-0.01767	-0.1616 to 0.1263	No
6567	5:10 X vs. 15:50 X	-0.03533	-0.1793 to 0.1086	No
6568	5:10 X vs. 16:0 X	-0.1203	-0.2643 to 0.02363	No
6569	5:10 X vs. 16:1 X	-0.01133	-0.1553 to 0.1326	No
6570	5:10 X vs. 16:10 X	-0.0370	-0.1810 to 0.1070	No

	2way ANOVA Multiple comparisons	A Data Set-A Y	B Data Set-B Y	C Data Set-C Y
6571	5:**10 X** vs. 16:**40 X**	-0.05633	-0.2003 to 0.08763	No
6572	5:**10 X** vs. 16:**50 X**	-0.09867	-0.2426 to 0.04529	No
6573	5:**10 X** vs. 17:**0 X**	-0.1730	-0.3170 to -0.02904	Yes
6574	5:**10 X** vs. 17:**1 X**	-0.08733	-0.2313 to 0.05663	No
6575	5:**10 X** vs. 17:**10 X**	-0.1440	-0.2880 to -4.018e-005	Yes
6576	5:**10 X** vs. 17:**40 X**	-0.06733	-0.2113 to 0.07663	No
6577	5:**10 X** vs. 17:**50 X**	-0.1127	-0.2566 to 0.03129	No
6578	5:**10 X** vs. 18:**0 X**	-0.2217	-0.3656 to -0.07771	Yes
6579	5:**10 X** vs. 18:**1 X**	-0.1300	-0.2740 to 0.01396	No
6580	5:**10 X** vs. 18:**10 X**	-0.2223	-0.3663 to -0.07837	Yes
6581	5:**10 X** vs. 18:**40 X**	-0.07167	-0.2156 to 0.07229	No
6582	5:**10 X** vs. 18:**50 X**	-0.1167	-0.2606 to 0.02729	No
6583	5:**10 X** vs. 19:**0 X**	-0.2567	-0.4006 to -0.1127	Yes
6584	5:**10 X** vs. 19:**1 X**	-0.2447	-0.3886 to -0.1007	Yes
6585	5:**10 X** vs. 19:**10 X**	-0.2423	-0.3863 to -0.09837	Yes
6586	5:**10 X** vs. 19:**40 X**	-0.1003	-0.2443 to 0.04363	No
6587	5:**10 X** vs. 19:**50 X**	-0.1113	-0.2553 to 0.03263	No
6588	5:**10 X** vs. 20:**0 X**	-0.3363	-0.4803 to -0.1924	Yes
6589	5:**10 X** vs. 20:**1 X**	-0.1557	-0.2996 to -0.01171	Yes
6590	5:**10 X** vs. 20:**10 X**	-0.2447	-0.3886 to -0.1007	Yes
6591	5:**10 X** vs. 20:**40 X**	-0.1120	-0.2560 to 0.03196	No
6592	5:**10 X** vs. 20:**50 X**	-0.1560	-0.3000 to -0.01204	Yes
6593	5:**10 X** vs. 21:**0 X**	-0.3597	-0.5036 to -0.2157	Yes
6594	5:**10 X** vs. 21:**1 X**	-0.3127	-0.4566 to -0.1687	Yes
6595	5:**10 X** vs. 21:**10 X**	-0.3097	-0.4536 to -0.1657	Yes
6596	5:**10 X** vs. 21:**40 X**	-0.1850	-0.3290 to -0.04104	Yes
6597	5:**10 X** vs. 21:**50 X**	-0.2753	-0.4193 to -0.1314	Yes
6598	5:**10 X** vs. 22:**0 X**	-0.4013	-0.5453 to -0.2574	Yes
6599	5:**10 X** vs. 22:**1 X**	-0.3960	-0.5400 to -0.2520	Yes
6600	5:**10 X** vs. 22:**10 X**	-0.3913	-0.5353 to -0.2474	Yes
6601	5:**10 X** vs. 22:**40 X**	-0.2917	-0.4356 to -0.1477	Yes
6602	5:**10 X** vs. 22:**50 X**	-0.3543	-0.4983 to -0.2104	Yes
6603	5:**10 X** vs. 23:**0 X**	-0.4667	-0.6106 to -0.3227	Yes
6604	5:**10 X** vs. 23:**1 X**	-0.6563	-0.8173 to -0.4954	Yes
6605	5:**10 X** vs. 23:**10 X**	-0.4060	-0.5500 to -0.2620	Yes
6606	5:**10 X** vs. 23:**40 X**	-0.2863	-0.4473 to -0.1254	Yes
6607	5:**10 X** vs. 23:**50 X**	-0.3203	-0.4643 to -0.1764	Yes
6608	5:**10 X** vs. 24:**0 X**	-0.5840	-0.7280 to -0.4400	Yes
6609	5:**10 X** vs. 24:**1 X**	-0.7403	-0.9013 to -0.5794	Yes
6610	5:**10 X** vs. 24:**10 X**	-0.5678	-0.7288 to -0.4069	Yes
6611	5:**10 X** vs. 24:**40 X**	-0.3027	-0.4466 to -0.1587	Yes
6612	5:**10 X** vs. 24:**50 X**	-0.2243	-0.3683 to -0.08037	Yes
6613	5:**10 X** vs. 25:**0 X**	-0.9213	-1.082 to -0.7604	Yes
6614	5:**10 X** vs. 25:**1 X**	-1.111	-1.272 to -0.9499	Yes
6615	5:**10 X** vs. 25:**10 X**	-0.4723	-0.6759 to -0.2687	Yes

	2way ANOVA<br>Multiple comparisons	A<br>Data Set-A<br>Y	B<br>Data Set-B<br>Y	C<br>Data Set-C<br>Y
6616	5:**10 X** vs. 25:**40 X**	-0.3047	-0.4486 to -0.1607	Yes
6617	5:**10 X** vs. 25:**50 X**	-0.2790	-0.4230 to -0.1350	Yes
6618	5:**10 X** vs. 26:**0 X**	-1.134	-1.295 to -0.9729	Yes
6619	5:**10 X** vs. 26:**1 X**	-1.247	-1.408 to -1.086	Yes
6620	5:**10 X** vs. 26:**10 X**	-0.9333	-1.094 to -0.7724	Yes
6621	5:**10 X** vs. 26:**40 X**	-0.3790	-0.5230 to -0.2350	Yes
6622	5:**10 X** vs. 26:**50 X**	-0.2393	-0.3833 to -0.09537	Yes
6623	5:**10 X** vs. 27:**0 X**	-1.322	-1.466 to -1.178	Yes
6624	5:**10 X** vs. 27:**1 X**	-1.412	-1.573 to -1.251	Yes
6625	5:**10 X** vs. 27:**10 X**	-1.224	-1.385 to -1.063	Yes
6626	5:**10 X** vs. 27:**40 X**	-0.3790	-0.5230 to -0.2350	Yes
6627	5:**10 X** vs. 27:**50 X**	-0.2387	-0.3826 to -0.09471	Yes
6628	5:**10 X** vs. 28:**0 X**	-1.271	-1.432 to -1.110	Yes
6629	5:**10 X** vs. 28:**1 X**	-1.312	-1.456 to -1.168	Yes
6630	5:**10 X** vs. 28:**10 X**	-1.113	-1.274 to -0.9524	Yes
6631	5:**10 X** vs. 28:**40 X**	-0.3913	-0.5353 to -0.2474	Yes
6632	5:**10 X** vs. 28:**50 X**	-0.2867	-0.4306 to -0.1427	Yes
6633	5:**10 X** vs. 29:**0 X**	-1.752	-1.913 to -1.591	Yes
6634	5:**10 X** vs. 29:**1 X**	-1.837	-1.998 to -1.676	Yes
6635	5:**10 X** vs. 29:**10 X**	-1.052	-1.213 to -0.8909	Yes
6636	5:**10 X** vs. 29:**40 X**	-0.4993	-0.6433 to -0.3554	Yes
6637	5:**10 X** vs. 29:**50 X**	-0.2880	-0.4320 to -0.1440	Yes
6638	5:**10 X** vs. 30:**0 X**	-1.467	-1.628 to -1.306	Yes
6639	5:**10 X** vs. 30:**1 X**	-1.788	-1.949 to -1.627	Yes
6640	5:**10 X** vs. 30:**10 X**	-1.126	-1.287 to -0.9654	Yes
6641	5:**10 X** vs. 30:**40 X**	-0.4263	-0.5703 to -0.2824	Yes
6642	5:**10 X** vs. 30:**50 X**	-0.3403	-0.4843 to -0.1964	Yes
6643	5:**10 X** vs. 31:**0 X**	-1.785	-1.946 to -1.624	Yes
6644	5:**10 X** vs. 31:**1 X**	-1.775	-1.936 to -1.614	Yes
6645	5:**10 X** vs. 31:**10 X**	-1.258	-1.419 to -1.097	Yes
6646	5:**10 X** vs. 31:**40 X**	-0.5180	-0.6620 to -0.3740	Yes
6647	5:**10 X** vs. 31:**50 X**	-0.3193	-0.4633 to -0.1754	Yes
6648	5:**10 X** vs. 32:**0 X**	-2.376	-2.520 to -2.232	Yes
6649	5:**10 X** vs. 32:**1 X**	-1.832	-1.993 to -1.671	Yes
6650	5:**10 X** vs. 32:**10 X**	-1.688	-1.832 to -1.544	Yes
6651	5:**10 X** vs. 32:**40 X**	-0.5143	-0.6583 to -0.3704	Yes
6652	5:**10 X** vs. 32:**50 X**	-0.3190	-0.4630 to -0.1750	Yes
6653	5:**10 X** vs. 33:**0 X**	-2.380	-2.524 to -2.236	Yes
6654	5:**10 X** vs. 33:**1 X**	-2.195	-2.339 to -2.051	Yes
6655	5:**10 X** vs. 33:**10 X**	-1.855	-1.999 to -1.711	Yes
6656	5:**10 X** vs. 33:**40 X**	-0.5330	-0.6770 to -0.3890	Yes
6657	5:**10 X** vs. 33:**50 X**	-0.3340	-0.4780 to -0.1900	Yes
6658	5:**10 X** vs. 34:**0 X**	-2.305	-2.449 to -2.161	Yes
6659	5:**10 X** vs. 34:**1 X**	-2.312	-2.456 to -2.168	Yes
6660	5:**10 X** vs. 34:**10 X**	-1.945	-2.089 to -1.801	Yes

	2way ANOVA<br>Multiple comparisons	A<br>Data Set-A<br>Y	B<br>Data Set-B<br>Y	C<br>Data Set-C<br>Y
6661	5:**10 X** vs. 34:**40 X**	-0.5450	-0.6890 to -0.4010	Yes
6662	5:**10 X** vs. 34:**50 X**	-0.4830	-0.6270 to -0.3390	Yes
6663	5:**10 X** vs. 35:**0 X**	-2.653	-2.797 to -2.509	Yes
6664	5:**10 X** vs. 35:**1 X**	-2.419	-2.563 to -2.275	Yes
6665	5:**10 X** vs. 35:**10 X**	-1.907	-2.051 to -1.763	Yes
6666	5:**10 X** vs. 35:**40 X**	-0.5540	-0.6980 to -0.4100	Yes
6667	5:**10 X** vs. 35:**50 X**	-0.4957	-0.6396 to -0.3517	Yes
6668	5:**10 X** vs. 36:**0 X**	-2.860	-3.021 to -2.699	Yes
6669	5:**10 X** vs. 36:**1 X**	-2.556	-2.700 to -2.412	Yes
6670	5:**10 X** vs. 36:**10 X**	-1.913	-2.057 to -1.769	Yes
6671	5:**10 X** vs. 36:**40 X**	-0.6093	-0.7533 to -0.4654	Yes
6672	5:**10 X** vs. 36:**50 X**	-0.4590	-0.6030 to -0.3150	Yes
6673	5:**10 X** vs. 37:**0 X**	-3.244	-3.388 to -3.100	Yes
6674	5:**10 X** vs. 37:**1 X**	-2.626	-2.770 to -2.482	Yes
6675	5:**10 X** vs. 37:**10 X**	-1.914	-2.058 to -1.770	Yes
6676	5:**10 X** vs. 37:**40 X**	-0.6467	-0.7906 to -0.5027	Yes
6677	5:**10 X** vs. 37:**50 X**	-0.4677	-0.6116 to -0.3237	Yes
6678	5:**10 X** vs. 38:**0 X**	-3.189	-3.333 to -3.045	Yes
6679	5:**10 X** vs. 38:**1 X**	-2.744	-2.888 to -2.600	Yes
6680	5:**10 X** vs. 38:**10 X**	-2.190	-2.334 to -2.046	Yes
6681	5:**10 X** vs. 38:**40 X**	-0.6510	-0.7950 to -0.5070	Yes
6682	5:**10 X** vs. 38:**50 X**	-0.4717	-0.6156 to -0.3277	Yes
6683	5:**10 X** vs. 39:**0 X**	-3.419	-3.563 to -3.275	Yes
6684	5:**10 X** vs. 39:**1 X**	-3.311	-3.455 to -3.167	Yes
6685	5:**10 X** vs. 39:**10 X**	-2.335	-2.479 to -2.191	Yes
6686	5:**10 X** vs. 39:**40 X**	-0.6570	-0.8010 to -0.5130	Yes
6687	5:**10 X** vs. 39:**50 X**	-0.5027	-0.6466 to -0.3587	Yes
6688	5:**10 X** vs. 40:**0 X**	-3.460	-3.604 to -3.316	Yes
6689	5:**10 X** vs. 40:**1 X**	-3.386	-3.530 to -3.242	Yes
6690	5:**10 X** vs. 40:**10 X**	-2.591	-2.735 to -2.447	Yes
6691	5:**10 X** vs. 40:**40 X**	-0.6617	-0.8056 to -0.5177	Yes
6692	5:**10 X** vs. 40:**50 X**	-0.5090	-0.6530 to -0.3650	Yes
6693	5:**10 X** vs. 41:**0 X**	-3.501	-3.645 to -3.357	Yes
6694	5:**10 X** vs. 41:**1 X**	-3.500	-3.644 to -3.356	Yes
6695	5:**10 X** vs. 41:**10 X**	-2.645	-2.789 to -2.501	Yes
6696	5:**10 X** vs. 41:**40 X**	-0.6090	-0.7530 to -0.4650	Yes
6697	5:**10 X** vs. 41:**50 X**	-0.5217	-0.6656 to -0.3777	Yes
6698	5:**10 X** vs. 42:**0 X**	-3.643	-3.787 to -3.499	Yes
6699	5:**10 X** vs. 42:**1 X**	-3.663	-3.807 to -3.519	Yes
6700	5:**10 X** vs. 42:**10 X**	-2.645	-2.789 to -2.501	Yes
6701	5:**10 X** vs. 42:**40 X**	-0.6217	-0.7656 to -0.4777	Yes
6702	5:**10 X** vs. 42:**50 X**	-0.5250	-0.6690 to -0.3810	Yes
6703	5:**10 X** vs. 43:**0 X**	-3.733	-3.877 to -3.589	Yes
6704	5:**10 X** vs. 43:**1 X**	-3.647	-3.791 to -3.503	Yes
6705	5:**10 X** vs. 43:**10 X**	-3.363	-3.507 to -3.219	Yes

	2way ANOVA<br>Multiple comparisons	A<br>Data Set-A<br>Y	B<br>Data Set-B<br>Y	C<br>Data Set-C<br>Y
6706	5:10 X vs. 43:40 X	-0.6200	-0.7640 to -0.4760	Yes
6707	5:10 X vs. 43:50 X	-0.5253	-0.6693 to -0.3814	Yes
6708	5:10 X vs. 44:0 X	-3.757	-3.901 to -3.613	Yes
6709	5:10 X vs. 44:1 X	-3.647	-3.791 to -3.503	Yes
6710	5:10 X vs. 44:10 X	-3.426	-3.570 to -3.282	Yes
6711	5:10 X vs. 44:40 X	-0.6220	-0.7660 to -0.4780	Yes
6712	5:10 X vs. 44:50 X	-0.4577	-0.6016 to -0.3137	Yes
6713	5:10 X vs. 45:0 X	-3.622	-3.766 to -3.478	Yes
6714	5:10 X vs. 45:1 X	-3.424	-3.568 to -3.280	Yes
6715	5:10 X vs. 45:10 X	-3.306	-3.450 to -3.162	Yes
6716	5:10 X vs. 45:40 X	-0.6233	-0.7673 to -0.4794	Yes
6717	5:10 X vs. 45:50 X	-0.4547	-0.5986 to -0.3107	Yes
6718	5:10 X vs. 46:0 X	-3.426	-3.570 to -3.282	Yes
6719	5:10 X vs. 46:1 X	-3.393	-3.537 to -3.249	Yes
6720	5:10 X vs. 46:10 X	-3.301	-3.445 to -3.157	Yes
6721	5:10 X vs. 46:40 X	-0.6370	-0.7810 to -0.4930	Yes
6722	5:10 X vs. 46:50 X	-0.4477	-0.5916 to -0.3037	Yes
6723	5:10 X vs. 47:0 X	-3.402	-3.546 to -3.258	Yes
6724	5:10 X vs. 47:1 X	-3.306	-3.450 to -3.162	Yes
6725	5:10 X vs. 47:10 X	-3.287	-3.431 to -3.143	Yes
6726	5:10 X vs. 47:40 X	-0.6470	-0.7910 to -0.5030	Yes
6727	5:10 X vs. 47:50 X	-0.4057	-0.5496 to -0.2617	Yes
6728	5:10 X vs. 48:0 X	-3.389	-3.550 to -3.228	Yes
6729	5:10 X vs. 48:1 X	-3.293	-3.437 to -3.149	Yes
6730	5:10 X vs. 48:10 X	-3.287	-3.431 to -3.143	Yes
6731	5:10 X vs. 48:40 X	-0.6533	-0.7973 to -0.5094	Yes
6732	5:10 X vs. 48:50 X	-0.4130	-0.5570 to -0.2690	Yes
6733	5:10 X vs. 49:0 X	-3.389	-3.533 to -3.245	Yes
6734	5:10 X vs. 49:1 X	-3.293	-3.437 to -3.149	Yes
6735	5:10 X vs. 49:10 X	-3.287	-3.431 to -3.143	Yes
6736	5:10 X vs. 49:40 X	-0.6577	-0.8016 to -0.5137	Yes
6737	5:10 X vs. 49:50 X	-0.4123	-0.5563 to -0.2684	Yes
6738	5:10 X vs. 50:0 X	-3.389	-3.533 to -3.245	Yes
6739	5:10 X vs. 50:1 X	-3.293	-3.437 to -3.149	Yes
6740	5:10 X vs. 50:10 X	-3.287	-3.431 to -3.143	Yes
6741	5:10 X vs. 50:40 X	-0.6653	-0.8093 to -0.5214	Yes
6742	5:10 X vs. 50:50 X	-0.4120	-0.5560 to -0.2680	Yes
6743	5:40 X vs. 5:50 X	0.001000	-0.1430 to 0.1450	No
6744	5:40 X vs. 6:0 X	-0.007333	-0.1513 to 0.1366	No
6745	5:40 X vs. 6:1 X	-0.006333	-0.1503 to 0.1376	No
6746	5:40 X vs. 6:10 X	-0.0210	-0.1650 to 0.1230	No
6747	5:40 X vs. 6:40 X	-0.002333	-0.1463 to 0.1416	No
6748	5:40 X vs. 6:50 X	-0.0006667	-0.1446 to 0.1433	No
6749	5:40 X vs. 7:0 X	-0.009333	-0.1533 to 0.1346	No
6750	5:40 X vs. 7:1 X	-0.0006667	-0.1446 to 0.1433	No

	2way ANOVA<br>Multiple comparisons	A<br>Data Set-A<br>Y	B<br>Data Set-B<br>Y	C<br>Data Set-C<br>Y
6751	5:40 X vs. 7:10 X	-0.01267	-0.1566 to 0.1313	No
6752	5:40 X vs. 7:40 X	-0.0040	-0.1480 to 0.1400	No
6753	5:40 X vs. 7:50 X	-0.0020	-0.1460 to 0.1420	No
6754	5:40 X vs. 8:0 X	-0.0140	-0.1580 to 0.1300	No
6755	5:40 X vs. 8:1 X	-0.0006667	-0.1446 to 0.1433	No
6756	5:40 X vs. 8:10 X	-0.008000	-0.1520 to 0.1360	No
6757	5:40 X vs. 8:40 X	-0.008667	-0.1526 to 0.1353	No
6758	5:40 X vs. 8:50 X	-0.005667	-0.1496 to 0.1383	No
6759	5:40 X vs. 9:0 X	-0.0220	-0.1660 to 0.1220	No
6760	5:40 X vs. 9:1 X	-0.0300	-0.1740 to 0.1140	No
6761	5:40 X vs. 9:10 X	-0.0310	-0.1750 to 0.1130	No
6762	5:40 X vs. 9:40 X	-0.01333	-0.1573 to 0.1306	No
6763	5:40 X vs. 9:50 X	-0.006667	-0.1506 to 0.1373	No
6764	5:40 X vs. 10:0 X	-0.02367	-0.1676 to 0.1203	No
6765	5:40 X vs. 10:1 X	-0.03067	-0.1746 to 0.1133	No
6766	5:40 X vs. 10:10 X	-0.03333	-0.1773 to 0.1106	No
6767	5:40 X vs. 10:40 X	-0.0230	-0.1670 to 0.1210	No
6768	5:40 X vs. 10:50 X	-0.009667	-0.1536 to 0.1343	No
6769	5:40 X vs. 11:0 X	-0.03333	-0.1773 to 0.1106	No
6770	5:40 X vs. 11:1 X	-0.0350	-0.1790 to 0.1090	No
6771	5:40 X vs. 11:10 X	-0.03533	-0.1793 to 0.1086	No
6772	5:40 X vs. 11:40 X	-0.0270	-0.1710 to 0.1170	No
6773	5:40 X vs. 11:50 X	-0.0150	-0.1590 to 0.1290	No
6774	5:40 X vs. 12:0 X	-0.0340	-0.1780 to 0.1100	No
6775	5:40 X vs. 12:1 X	-0.01633	-0.1603 to 0.1276	No
6776	5:40 X vs. 12:10 X	-0.0340	-0.1780 to 0.1100	No
6777	5:40 X vs. 12:40 X	-0.02033	-0.1643 to 0.1236	No
6778	5:40 X vs. 12:50 X	-0.02367	-0.1676 to 0.1203	No
6779	5:40 X vs. 13:0 X	-0.0280	-0.1720 to 0.1160	No
6780	5:40 X vs. 13:1 X	-0.01533	-0.1593 to 0.1286	No
6781	5:40 X vs. 13:10 X	-0.03733	-0.1813 to 0.1066	No
6782	5:40 X vs. 13:40 X	-0.03367	-0.1776 to 0.1103	No
6783	5:40 X vs. 13:50 X	-0.03467	-0.1786 to 0.1093	No
6784	5:40 X vs. 14:0 X	-0.03667	-0.1806 to 0.1073	No
6785	5:40 X vs. 14:1 X	-0.02833	-0.1723 to 0.1156	No
6786	5:40 X vs. 14:10 X	-0.04233	-0.1863 to 0.1016	No
6787	5:40 X vs. 14:40 X	-0.0390	-0.1830 to 0.1050	No
6788	5:40 X vs. 14:50 X	-0.0430	-0.1870 to 0.1010	No
6789	5:40 X vs. 15:0 X	-0.0490	-0.1930 to 0.09496	No
6790	5:40 X vs. 15:1 X	-0.0290	-0.1730 to 0.1150	No
6791	5:40 X vs. 15:10 X	-0.05633	-0.2003 to 0.08763	No
6792	5:40 X vs. 15:40 X	-0.03867	-0.1826 to 0.1053	No
6793	5:40 X vs. 15:50 X	-0.05633	-0.2003 to 0.08763	No
6794	5:40 X vs. 16:0 X	-0.1413	-0.2853 to 0.002626	No
6795	5:40 X vs. 16:1 X	-0.03233	-0.1763 to 0.1116	No

2way ANOVA<br>Multiple comparisons		A<br>Data Set-A<br>Y	B<br>Data Set-B<br>Y	C<br>Data Set-C<br>Y
6796	5:40 X vs. 16:10 X	-0.0580	-0.2020 to 0.08596	No
6797	5:40 X vs. 16:40 X	-0.07733	-0.2213 to 0.06663	No
6798	5:40 X vs. 16:50 X	-0.1197	-0.2636 to 0.02429	No
6799	5:40 X vs. 17:0 X	-0.1940	-0.3380 to -0.05004	Yes
6800	5:40 X vs. 17:1 X	-0.1083	-0.2523 to 0.03563	No
6801	5:40 X vs. 17:10 X	-0.1650	-0.3090 to -0.02104	Yes
6802	5:40 X vs. 17:40 X	-0.08833	-0.2323 to 0.05563	No
6803	5:40 X vs. 17:50 X	-0.1337	-0.2776 to 0.01029	No
6804	5:40 X vs. 18:0 X	-0.2427	-0.3866 to -0.09871	Yes
6805	5:40 X vs. 18:1 X	-0.1510	-0.2950 to -0.007040	Yes
6806	5:40 X vs. 18:10 X	-0.2433	-0.3873 to -0.09937	Yes
6807	5:40 X vs. 18:40 X	-0.09267	-0.2366 to 0.05129	No
6808	5:40 X vs. 18:50 X	-0.1377	-0.2816 to 0.006293	No
6809	5:40 X vs. 19:0 X	-0.2777	-0.4216 to -0.1337	Yes
6810	5:40 X vs. 19:1 X	-0.2657	-0.4096 to -0.1217	Yes
6811	5:40 X vs. 19:10 X	-0.2633	-0.4073 to -0.1194	Yes
6812	5:40 X vs. 19:40 X	-0.1213	-0.2653 to 0.02263	No
6813	5:40 X vs. 19:50 X	-0.1323	-0.2763 to 0.01163	No
6814	5:40 X vs. 20:0 X	-0.3573	-0.5013 to -0.2134	Yes
6815	5:40 X vs. 20:1 X	-0.1767	-0.3206 to -0.03271	Yes
6816	5:40 X vs. 20:10 X	-0.2657	-0.4096 to -0.1217	Yes
6817	5:40 X vs. 20:40 X	-0.1330	-0.2770 to 0.01096	No
6818	5:40 X vs. 20:50 X	-0.1770	-0.3210 to -0.03304	Yes
6819	5:40 X vs. 21:0 X	-0.3807	-0.5246 to -0.2367	Yes
6820	5:40 X vs. 21:1 X	-0.3337	-0.4776 to -0.1897	Yes
6821	5:40 X vs. 21:10 X	-0.3307	-0.4746 to -0.1867	Yes
6822	5:40 X vs. 21:40 X	-0.2060	-0.3500 to -0.06204	Yes
6823	5:40 X vs. 21:50 X	-0.2963	-0.4403 to -0.1524	Yes
6824	5:40 X vs. 22:0 X	-0.4223	-0.5663 to -0.2784	Yes
6825	5:40 X vs. 22:1 X	-0.4170	-0.5610 to -0.2730	Yes
6826	5:40 X vs. 22:10 X	-0.4123	-0.5563 to -0.2684	Yes
6827	5:40 X vs. 22:40 X	-0.3127	-0.4566 to -0.1687	Yes
6828	5:40 X vs. 22:50 X	-0.3753	-0.5193 to -0.2314	Yes
6829	5:40 X vs. 23:0 X	-0.4877	-0.6316 to -0.3437	Yes
6830	5:40 X vs. 23:1 X	-0.6773	-0.8383 to -0.5164	Yes
6831	5:40 X vs. 23:10 X	-0.4270	-0.5710 to -0.2830	Yes
6832	5:40 X vs. 23:40 X	-0.3073	-0.4683 to -0.1464	Yes
6833	5:40 X vs. 23:50 X	-0.3413	-0.4853 to -0.1974	Yes
6834	5:40 X vs. 24:0 X	-0.6050	-0.7490 to -0.4610	Yes
6835	5:40 X vs. 24:1 X	-0.7613	-0.9223 to -0.6004	Yes
6836	5:40 X vs. 24:10 X	-0.5888	-0.7498 to -0.4279	Yes
6837	5:40 X vs. 24:40 X	-0.3237	-0.4676 to -0.1797	Yes
6838	5:40 X vs. 24:50 X	-0.2453	-0.3893 to -0.1014	Yes
6839	5:40 X vs. 25:0 X	-0.9423	-1.103 to -0.7814	Yes
6840	5:40 X vs. 25:1 X	-1.132	-1.293 to -0.9709	Yes

	2way ANOVA<br>Multiple comparisons	A<br>Data Set-A<br>Y	B<br>Data Set-B<br>Y	C<br>Data Set-C<br>Y
**6841**	5:**40 X** vs. 25:**10 X**	-0.4933	-0.6969 to -0.2897	Yes
**6842**	5:**40 X** vs. 25:**40 X**	-0.3257	-0.4696 to -0.1817	Yes
**6843**	5:**40 X** vs. 25:**50 X**	-0.3000	-0.4440 to -0.1560	Yes
**6844**	5:**40 X** vs. 26:**0 X**	-1.155	-1.316 to -0.9939	Yes
**6845**	5:**40 X** vs. 26:**1 X**	-1.268	-1.429 to -1.107	Yes
**6846**	5:**40 X** vs. 26:**10 X**	-0.9543	-1.115 to -0.7934	Yes
**6847**	5:**40 X** vs. 26:**40 X**	-0.4000	-0.5440 to -0.2560	Yes
**6848**	5:**40 X** vs. 26:**50 X**	-0.2603	-0.4043 to -0.1164	Yes
**6849**	5:**40 X** vs. 27:**0 X**	-1.343	-1.487 to -1.199	Yes
**6850**	5:**40 X** vs. 27:**1 X**	-1.433	-1.594 to -1.272	Yes
**6851**	5:**40 X** vs. 27:**10 X**	-1.245	-1.406 to -1.084	Yes
**6852**	5:**40 X** vs. 27:**40 X**	-0.4000	-0.5440 to -0.2560	Yes
**6853**	5:**40 X** vs. 27:**50 X**	-0.2597	-0.4036 to -0.1157	Yes
**6854**	5:**40 X** vs. 28:**0 X**	-1.292	-1.453 to -1.131	Yes
**6855**	5:**40 X** vs. 28:**1 X**	-1.333	-1.477 to -1.189	Yes
**6856**	5:**40 X** vs. 28:**10 X**	-1.134	-1.295 to -0.9734	Yes
**6857**	5:**40 X** vs. 28:**40 X**	-0.4123	-0.5563 to -0.2684	Yes
**6858**	5:**40 X** vs. 28:**50 X**	-0.3077	-0.4516 to -0.1637	Yes
**6859**	5:**40 X** vs. 29:**0 X**	-1.773	-1.934 to -1.612	Yes
**6860**	5:**40 X** vs. 29:**1 X**	-1.858	-2.019 to -1.697	Yes
**6861**	5:**40 X** vs. 29:**10 X**	-1.073	-1.234 to -0.9119	Yes
**6862**	5:**40 X** vs. 29:**40 X**	-0.5203	-0.6643 to -0.3764	Yes
**6863**	5:**40 X** vs. 29:**50 X**	-0.3090	-0.4530 to -0.1650	Yes
**6864**	5:**40 X** vs. 30:**0 X**	-1.488	-1.649 to -1.327	Yes
**6865**	5:**40 X** vs. 30:**1 X**	-1.809	-1.970 to -1.648	Yes
**6866**	5:**40 X** vs. 30:**10 X**	-1.147	-1.308 to -0.9864	Yes
**6867**	5:**40 X** vs. 30:**40 X**	-0.4473	-0.5913 to -0.3034	Yes
**6868**	5:**40 X** vs. 30:**50 X**	-0.3613	-0.5053 to -0.2174	Yes
**6869**	5:**40 X** vs. 31:**0 X**	-1.806	-1.967 to -1.645	Yes
**6870**	5:**40 X** vs. 31:**1 X**	-1.796	-1.957 to -1.635	Yes
**6871**	5:**40 X** vs. 31:**10 X**	-1.279	-1.440 to -1.118	Yes
**6872**	5:**40 X** vs. 31:**40 X**	-0.5390	-0.6830 to -0.3950	Yes
**6873**	5:**40 X** vs. 31:**50 X**	-0.3403	-0.4843 to -0.1964	Yes
**6874**	5:**40 X** vs. 32:**0 X**	-2.397	-2.541 to -2.253	Yes
**6875**	5:**40 X** vs. 32:**1 X**	-1.853	-2.014 to -1.692	Yes
**6876**	5:**40 X** vs. 32:**10 X**	-1.709	-1.853 to -1.565	Yes
**6877**	5:**40 X** vs. 32:**40 X**	-0.5353	-0.6793 to -0.3914	Yes
**6878**	5:**40 X** vs. 32:**50 X**	-0.3400	-0.4840 to -0.1960	Yes
**6879**	5:**40 X** vs. 33:**0 X**	-2.401	-2.545 to -2.257	Yes
**6880**	5:**40 X** vs. 33:**1 X**	-2.216	-2.360 to -2.072	Yes
**6881**	5:**40 X** vs. 33:**10 X**	-1.876	-2.020 to -1.732	Yes
**6882**	5:**40 X** vs. 33:**40 X**	-0.5540	-0.6980 to -0.4100	Yes
**6883**	5:**40 X** vs. 33:**50 X**	-0.3550	-0.4990 to -0.2110	Yes
**6884**	5:**40 X** vs. 34:**0 X**	-2.326	-2.470 to -2.182	Yes
**6885**	5:**40 X** vs. 34:**1 X**	-2.333	-2.477 to -2.189	Yes

	2way ANOVA<br>Multiple comparisons	A<br>Data Set-A<br>Y	B<br>Data Set-B<br>Y	C<br>Data Set-C<br>Y
6886	5:40 X vs. 34:10 X	-1.966	-2.110 to -1.822	Yes
6887	5:40 X vs. 34:40 X	-0.5660	-0.7100 to -0.4220	Yes
6888	5:40 X vs. 34:50 X	-0.5040	-0.6480 to -0.3600	Yes
6889	5:40 X vs. 35:0 X	-2.674	-2.818 to -2.530	Yes
6890	5:40 X vs. 35:1 X	-2.440	-2.584 to -2.296	Yes
6891	5:40 X vs. 35:10 X	-1.928	-2.072 to -1.784	Yes
6892	5:40 X vs. 35:40 X	-0.5750	-0.7190 to -0.4310	Yes
6893	5:40 X vs. 35:50 X	-0.5167	-0.6606 to -0.3727	Yes
6894	5:40 X vs. 36:0 X	-2.881	-3.042 to -2.720	Yes
6895	5:40 X vs. 36:1 X	-2.577	-2.721 to -2.433	Yes
6896	5:40 X vs. 36:10 X	-1.934	-2.078 to -1.790	Yes
6897	5:40 X vs. 36:40 X	-0.6303	-0.7743 to -0.4864	Yes
6898	5:40 X vs. 36:50 X	-0.4800	-0.6240 to -0.3360	Yes
6899	5:40 X vs. 37:0 X	-3.265	-3.409 to -3.121	Yes
6900	5:40 X vs. 37:1 X	-2.647	-2.791 to -2.503	Yes
6901	5:40 X vs. 37:10 X	-1.935	-2.079 to -1.791	Yes
6902	5:40 X vs. 37:40 X	-0.6677	-0.8116 to -0.5237	Yes
6903	5:40 X vs. 37:50 X	-0.4887	-0.6326 to -0.3447	Yes
6904	5:40 X vs. 38:0 X	-3.210	-3.354 to -3.066	Yes
6905	5:40 X vs. 38:1 X	-2.765	-2.909 to -2.621	Yes
6906	5:40 X vs. 38:10 X	-2.211	-2.355 to -2.067	Yes
6907	5:40 X vs. 38:40 X	-0.6720	-0.8160 to -0.5280	Yes
6908	5:40 X vs. 38:50 X	-0.4927	-0.6366 to -0.3487	Yes
6909	5:40 X vs. 39:0 X	-3.440	-3.584 to -3.296	Yes
6910	5:40 X vs. 39:1 X	-3.332	-3.476 to -3.188	Yes
6911	5:40 X vs. 39:10 X	-2.356	-2.500 to -2.212	Yes
6912	5:40 X vs. 39:40 X	-0.6780	-0.8220 to -0.5340	Yes
6913	5:40 X vs. 39:50 X	-0.5237	-0.6676 to -0.3797	Yes
6914	5:40 X vs. 40:0 X	-3.481	-3.625 to -3.337	Yes
6915	5:40 X vs. 40:1 X	-3.407	-3.551 to -3.263	Yes
6916	5:40 X vs. 40:10 X	-2.612	-2.756 to -2.468	Yes
6917	5:40 X vs. 40:40 X	-0.6827	-0.8266 to -0.5387	Yes
6918	5:40 X vs. 40:50 X	-0.5300	-0.6740 to -0.3860	Yes
6919	5:40 X vs. 41:0 X	-3.522	-3.666 to -3.378	Yes
6920	5:40 X vs. 41:1 X	-3.521	-3.665 to -3.377	Yes
6921	5:40 X vs. 41:10 X	-2.666	-2.810 to -2.522	Yes
6922	5:40 X vs. 41:40 X	-0.6300	-0.7740 to -0.4860	Yes
6923	5:40 X vs. 41:50 X	-0.5427	-0.6866 to -0.3987	Yes
6924	5:40 X vs. 42:0 X	-3.664	-3.808 to -3.520	Yes
6925	5:40 X vs. 42:1 X	-3.684	-3.828 to -3.540	Yes
6926	5:40 X vs. 42:10 X	-2.666	-2.810 to -2.522	Yes
6927	5:40 X vs. 42:40 X	-0.6427	-0.7866 to -0.4987	Yes
6928	5:40 X vs. 42:50 X	-0.5460	-0.6900 to -0.4020	Yes
6929	5:40 X vs. 43:0 X	-3.754	-3.898 to -3.610	Yes
6930	5:40 X vs. 43:1 X	-3.668	-3.812 to -3.524	Yes

	2way ANOVA<br>Multiple comparisons	A<br>Data Set-A<br>Y	B<br>Data Set-B<br>Y	C<br>Data Set-C<br>Y
6931	5:40 X vs. 43:10 X	-3.384	-3.528 to -3.240	Yes
6932	5:40 X vs. 43:40 X	-0.6410	-0.7850 to -0.4970	Yes
6933	5:40 X vs. 43:50 X	-0.5463	-0.6903 to -0.4024	Yes
6934	5:40 X vs. 44:0 X	-3.778	-3.922 to -3.634	Yes
6935	5:40 X vs. 44:1 X	-3.668	-3.812 to -3.524	Yes
6936	5:40 X vs. 44:10 X	-3.447	-3.591 to -3.303	Yes
6937	5:40 X vs. 44:40 X	-0.6430	-0.7870 to -0.4990	Yes
6938	5:40 X vs. 44:50 X	-0.4787	-0.6226 to -0.3347	Yes
6939	5:40 X vs. 45:0 X	-3.643	-3.787 to -3.499	Yes
6940	5:40 X vs. 45:1 X	-3.445	-3.589 to -3.301	Yes
6941	5:40 X vs. 45:10 X	-3.327	-3.471 to -3.183	Yes
6942	5:40 X vs. 45:40 X	-0.6443	-0.7883 to -0.5004	Yes
6943	5:40 X vs. 45:50 X	-0.4757	-0.6196 to -0.3317	Yes
6944	5:40 X vs. 46:0 X	-3.447	-3.591 to -3.303	Yes
6945	5:40 X vs. 46:1 X	-3.414	-3.558 to -3.270	Yes
6946	5:40 X vs. 46:10 X	-3.322	-3.466 to -3.178	Yes
6947	5:40 X vs. 46:40 X	-0.6580	-0.8020 to -0.5140	Yes
6948	5:40 X vs. 46:50 X	-0.4687	-0.6126 to -0.3247	Yes
6949	5:40 X vs. 47:0 X	-3.423	-3.567 to -3.279	Yes
6950	5:40 X vs. 47:1 X	-3.327	-3.471 to -3.183	Yes
6951	5:40 X vs. 47:10 X	-3.308	-3.452 to -3.164	Yes
6952	5:40 X vs. 47:40 X	-0.6680	-0.8120 to -0.5240	Yes
6953	5:40 X vs. 47:50 X	-0.4267	-0.5706 to -0.2827	Yes
6954	5:40 X vs. 48:0 X	-3.410	-3.571 to -3.249	Yes
6955	5:40 X vs. 48:1 X	-3.314	-3.458 to -3.170	Yes
6956	5:40 X vs. 48:10 X	-3.308	-3.452 to -3.164	Yes
6957	5:40 X vs. 48:40 X	-0.6743	-0.8183 to -0.5304	Yes
6958	5:40 X vs. 48:50 X	-0.4340	-0.5780 to -0.2900	Yes
6959	5:40 X vs. 49:0 X	-3.410	-3.554 to -3.266	Yes
6960	5:40 X vs. 49:1 X	-3.314	-3.458 to -3.170	Yes
6961	5:40 X vs. 49:10 X	-3.308	-3.452 to -3.164	Yes
6962	5:40 X vs. 49:40 X	-0.6787	-0.8226 to -0.5347	Yes
6963	5:40 X vs. 49:50 X	-0.4333	-0.5773 to -0.2894	Yes
6964	5:40 X vs. 50:0 X	-3.410	-3.554 to -3.266	Yes
6965	5:40 X vs. 50:1 X	-3.314	-3.458 to -3.170	Yes
6966	5:40 X vs. 50:10 X	-3.308	-3.452 to -3.164	Yes
6967	5:40 X vs. 50:40 X	-0.6863	-0.8303 to -0.5424	Yes
6968	5:40 X vs. 50:50 X	-0.4330	-0.5770 to -0.2890	Yes
6969	5:50 X vs. 6:0 X	-0.008333	-0.1523 to 0.1356	No
6970	5:50 X vs. 6:1 X	-0.007333	-0.1513 to 0.1366	No
6971	5:50 X vs. 6:10 X	-0.0220	-0.1660 to 0.1220	No
6972	5:50 X vs. 6:40 X	-0.003333	-0.1473 to 0.1406	No
6973	5:50 X vs. 6:50 X	-0.001667	-0.1456 to 0.1423	No
6974	5:50 X vs. 7:0 X	-0.01033	-0.1543 to 0.1336	No
6975	5:50 X vs. 7:1 X	-0.001667	-0.1456 to 0.1423	No

	2way ANOVA<br>Multiple comparisons	A<br>Data Set-A<br>Y	B<br>Data Set-B<br>Y	C<br>Data Set-C<br>Y
6976	5:50 X vs. 7:10 X	-0.01367	-0.1576 to 0.1303	No
6977	5:50 X vs. 7:40 X	-0.0050	-0.1490 to 0.1390	No
6978	5:50 X vs. 7:50 X	-0.0030	-0.1470 to 0.1410	No
6979	5:50 X vs. 8:0 X	-0.0150	-0.1590 to 0.1290	No
6980	5:50 X vs. 8:1 X	-0.001667	-0.1456 to 0.1423	No
6981	5:50 X vs. 8:10 X	-0.0090	-0.1530 to 0.1350	No
6982	5:50 X vs. 8:40 X	-0.009667	-0.1536 to 0.1343	No
6983	5:50 X vs. 8:50 X	-0.006667	-0.1506 to 0.1373	No
6984	5:50 X vs. 9:0 X	-0.0230	-0.1670 to 0.1210	No
6985	5:50 X vs. 9:1 X	-0.0310	-0.1750 to 0.1130	No
6986	5:50 X vs. 9:10 X	-0.0320	-0.1760 to 0.1120	No
6987	5:50 X vs. 9:40 X	-0.01433	-0.1583 to 0.1296	No
6988	5:50 X vs. 9:50 X	-0.007667	-0.1516 to 0.1363	No
6989	5:50 X vs. 10:0 X	-0.02467	-0.1686 to 0.1193	No
6990	5:50 X vs. 10:1 X	-0.03167	-0.1756 to 0.1123	No
6991	5:50 X vs. 10:10 X	-0.03433	-0.1783 to 0.1096	No
6992	5:50 X vs. 10:40 X	-0.0240	-0.1680 to 0.1200	No
6993	5:50 X vs. 10:50 X	-0.01067	-0.1546 to 0.1333	No
6994	5:50 X vs. 11:0 X	-0.03433	-0.1783 to 0.1096	No
6995	5:50 X vs. 11:1 X	-0.0360	-0.1800 to 0.1080	No
6996	5:50 X vs. 11:10 X	-0.03633	-0.1803 to 0.1076	No
6997	5:50 X vs. 11:40 X	-0.0280	-0.1720 to 0.1160	No
6998	5:50 X vs. 11:50 X	-0.0160	-0.1600 to 0.1280	No
6999	5:50 X vs. 12:0 X	-0.0350	-0.1790 to 0.1090	No
7000	5:50 X vs. 12:1 X	-0.01733	-0.1613 to 0.1266	No
7001	5:50 X vs. 12:10 X	-0.0350	-0.1790 to 0.1090	No
7002	5:50 X vs. 12:40 X	-0.02133	-0.1653 to 0.1226	No
7003	5:50 X vs. 12:50 X	-0.02467	-0.1686 to 0.1193	No
7004	5:50 X vs. 13:0 X	-0.0290	-0.1730 to 0.1150	No
7005	5:50 X vs. 13:1 X	-0.01633	-0.1603 to 0.1276	No
7006	5:50 X vs. 13:10 X	-0.03833	-0.1823 to 0.1056	No
7007	5:50 X vs. 13:40 X	-0.03467	-0.1786 to 0.1093	No
7008	5:50 X vs. 13:50 X	-0.03567	-0.1796 to 0.1083	No
7009	5:50 X vs. 14:0 X	-0.03767	-0.1816 to 0.1063	No
7010	5:50 X vs. 14:1 X	-0.02933	-0.1733 to 0.1146	No
7011	5:50 X vs. 14:10 X	-0.04333	-0.1873 to 0.1006	No
7012	5:50 X vs. 14:40 X	-0.0400	-0.1840 to 0.1040	No
7013	5:50 X vs. 14:50 X	-0.0440	-0.1880 to 0.09996	No
7014	5:50 X vs. 15:0 X	-0.0500	-0.1940 to 0.09396	No
7015	5:50 X vs. 15:1 X	-0.0300	-0.1740 to 0.1140	No
7016	5:50 X vs. 15:10 X	-0.05733	-0.2013 to 0.08663	No
7017	5:50 X vs. 15:40 X	-0.03967	-0.1836 to 0.1043	No
7018	5:50 X vs. 15:50 X	-0.05733	-0.2013 to 0.08663	No
7019	5:50 X vs. 16:0 X	-0.1423	-0.2863 to 0.001626	No
7020	5:50 X vs. 16:1 X	-0.03333	-0.1773 to 0.1106	No

	2way ANOVA<br>Multiple comparisons	A<br>Data Set-A<br>Y	B<br>Data Set-B<br>Y	C<br>Data Set-C<br>Y
7021	5:50 X vs. 16:10 X	-0.0590	-0.2030 to 0.08496	No
7022	5:50 X vs. 16:40 X	-0.07833	-0.2223 to 0.06563	No
7023	5:50 X vs. 16:50 X	-0.1207	-0.2646 to 0.02329	No
7024	5:50 X vs. 17:0 X	-0.1950	-0.3390 to -0.05104	Yes
7025	5:50 X vs. 17:1 X	-0.1093	-0.2533 to 0.03463	No
7026	5:50 X vs. 17:10 X	-0.1660	-0.3100 to -0.02204	Yes
7027	5:50 X vs. 17:40 X	-0.08933	-0.2333 to 0.05463	No
7028	5:50 X vs. 17:50 X	-0.1347	-0.2786 to 0.009293	No
7029	5:50 X vs. 18:0 X	-0.2437	-0.3876 to -0.09971	Yes
7030	5:50 X vs. 18:1 X	-0.1520	-0.2960 to -0.008040	Yes
7031	5:50 X vs. 18:10 X	-0.2443	-0.3883 to -0.1004	Yes
7032	5:50 X vs. 18:40 X	-0.09367	-0.2376 to 0.05029	No
7033	5:50 X vs. 18:50 X	-0.1387	-0.2826 to 0.005293	No
7034	5:50 X vs. 19:0 X	-0.2787	-0.4226 to -0.1347	Yes
7035	5:50 X vs. 19:1 X	-0.2667	-0.4106 to -0.1227	Yes
7036	5:50 X vs. 19:10 X	-0.2643	-0.4083 to -0.1204	Yes
7037	5:50 X vs. 19:40 X	-0.1223	-0.2663 to 0.02163	No
7038	5:50 X vs. 19:50 X	-0.1333	-0.2773 to 0.01063	No
7039	5:50 X vs. 20:0 X	-0.3583	-0.5023 to -0.2144	Yes
7040	5:50 X vs. 20:1 X	-0.1777	-0.3216 to -0.03371	Yes
7041	5:50 X vs. 20:10 X	-0.2667	-0.4106 to -0.1227	Yes
7042	5:50 X vs. 20:40 X	-0.1340	-0.2780 to 0.009960	No
7043	5:50 X vs. 20:50 X	-0.1780	-0.3220 to -0.03404	Yes
7044	5:50 X vs. 21:0 X	-0.3817	-0.5256 to -0.2377	Yes
7045	5:50 X vs. 21:1 X	-0.3347	-0.4786 to -0.1907	Yes
7046	5:50 X vs. 21:10 X	-0.3317	-0.4756 to -0.1877	Yes
7047	5:50 X vs. 21:40 X	-0.2070	-0.3510 to -0.06304	Yes
7048	5:50 X vs. 21:50 X	-0.2973	-0.4413 to -0.1534	Yes
7049	5:50 X vs. 22:0 X	-0.4233	-0.5673 to -0.2794	Yes
7050	5:50 X vs. 22:1 X	-0.4180	-0.5620 to -0.2740	Yes
7051	5:50 X vs. 22:10 X	-0.4133	-0.5573 to -0.2694	Yes
7052	5:50 X vs. 22:40 X	-0.3137	-0.4576 to -0.1697	Yes
7053	5:50 X vs. 22:50 X	-0.3763	-0.5203 to -0.2324	Yes
7054	5:50 X vs. 23:0 X	-0.4887	-0.6326 to -0.3447	Yes
7055	5:50 X vs. 23:1 X	-0.6783	-0.8393 to -0.5174	Yes
7056	5:50 X vs. 23:10 X	-0.4280	-0.5720 to -0.2840	Yes
7057	5:50 X vs. 23:40 X	-0.3083	-0.4693 to -0.1474	Yes
7058	5:50 X vs. 23:50 X	-0.3423	-0.4863 to -0.1984	Yes
7059	5:50 X vs. 24:0 X	-0.6060	-0.7500 to -0.4620	Yes
7060	5:50 X vs. 24:1 X	-0.7623	-0.9233 to -0.6014	Yes
7061	5:50 X vs. 24:10 X	-0.5898	-0.7508 to -0.4289	Yes
7062	5:50 X vs. 24:40 X	-0.3247	-0.4686 to -0.1807	Yes
7063	5:50 X vs. 24:50 X	-0.2463	-0.3903 to -0.1024	Yes
7064	5:50 X vs. 25:0 X	-0.9433	-1.104 to -0.7824	Yes
7065	5:50 X vs. 25:1 X	-1.133	-1.294 to -0.9719	Yes

	2way ANOVA<br>Multiple comparisons	A<br>Data Set-A<br>Y	B<br>Data Set-B<br>Y	C<br>Data Set-C<br>Y
7066	5:**50 X** vs. 25:**10 X**	-0.4943	-0.6979 to -0.2907	Yes
7067	5:**50 X** vs. 25:**40 X**	-0.3267	-0.4706 to -0.1827	Yes
7068	5:**50 X** vs. 25:**50 X**	-0.3010	-0.4450 to -0.1570	Yes
7069	5:**50 X** vs. 26:**0 X**	-1.156	-1.317 to -0.9949	Yes
7070	5:**50 X** vs. 26:**1 X**	-1.269	-1.430 to -1.108	Yes
7071	5:**50 X** vs. 26:**10 X**	-0.9553	-1.116 to -0.7944	Yes
7072	5:**50 X** vs. 26:**40 X**	-0.4010	-0.5450 to -0.2570	Yes
7073	5:**50 X** vs. 26:**50 X**	-0.2613	-0.4053 to -0.1174	Yes
7074	5:**50 X** vs. 27:**0 X**	-1.344	-1.488 to -1.200	Yes
7075	5:**50 X** vs. 27:**1 X**	-1.434	-1.595 to -1.273	Yes
7076	5:**50 X** vs. 27:**10 X**	-1.246	-1.407 to -1.085	Yes
7077	5:**50 X** vs. 27:**40 X**	-0.4010	-0.5450 to -0.2570	Yes
7078	5:**50 X** vs. 27:**50 X**	-0.2607	-0.4046 to -0.1167	Yes
7079	5:**50 X** vs. 28:**0 X**	-1.293	-1.454 to -1.132	Yes
7080	5:**50 X** vs. 28:**1 X**	-1.334	-1.478 to -1.190	Yes
7081	5:**50 X** vs. 28:**10 X**	-1.135	-1.296 to -0.9744	Yes
7082	5:**50 X** vs. 28:**40 X**	-0.4133	-0.5573 to -0.2694	Yes
7083	5:**50 X** vs. 28:**50 X**	-0.3087	-0.4526 to -0.1647	Yes
7084	5:**50 X** vs. 29:**0 X**	-1.774	-1.935 to -1.613	Yes
7085	5:**50 X** vs. 29:**1 X**	-1.859	-2.020 to -1.698	Yes
7086	5:**50 X** vs. 29:**10 X**	-1.074	-1.235 to -0.9129	Yes
7087	5:**50 X** vs. 29:**40 X**	-0.5213	-0.6653 to -0.3774	Yes
7088	5:**50 X** vs. 29:**50 X**	-0.3100	-0.4540 to -0.1660	Yes
7089	5:**50 X** vs. 30:**0 X**	-1.489	-1.650 to -1.328	Yes
7090	5:**50 X** vs. 30:**1 X**	-1.810	-1.971 to -1.649	Yes
7091	5:**50 X** vs. 30:**10 X**	-1.148	-1.309 to -0.9874	Yes
7092	5:**50 X** vs. 30:**40 X**	-0.4483	-0.5923 to -0.3044	Yes
7093	5:**50 X** vs. 30:**50 X**	-0.3623	-0.5063 to -0.2184	Yes
7094	5:**50 X** vs. 31:**0 X**	-1.807	-1.968 to -1.646	Yes
7095	5:**50 X** vs. 31:**1 X**	-1.797	-1.958 to -1.636	Yes
7096	5:**50 X** vs. 31:**10 X**	-1.280	-1.441 to -1.119	Yes
7097	5:**50 X** vs. 31:**40 X**	-0.5400	-0.6840 to -0.3960	Yes
7098	5:**50 X** vs. 31:**50 X**	-0.3413	-0.4853 to -0.1974	Yes
7099	5:**50 X** vs. 32:**0 X**	-2.398	-2.542 to -2.254	Yes
7100	5:**50 X** vs. 32:**1 X**	-1.854	-2.015 to -1.693	Yes
7101	5:**50 X** vs. 32:**10 X**	-1.710	-1.854 to -1.566	Yes
7102	5:**50 X** vs. 32:**40 X**	-0.5363	-0.6803 to -0.3924	Yes
7103	5:**50 X** vs. 32:**50 X**	-0.3410	-0.4850 to -0.1970	Yes
7104	5:**50 X** vs. 33:**0 X**	-2.402	-2.546 to -2.258	Yes
7105	5:**50 X** vs. 33:**1 X**	-2.217	-2.361 to -2.073	Yes
7106	5:**50 X** vs. 33:**10 X**	-1.877	-2.021 to -1.733	Yes
7107	5:**50 X** vs. 33:**40 X**	-0.5550	-0.6990 to -0.4110	Yes
7108	5:**50 X** vs. 33:**50 X**	-0.3560	-0.5000 to -0.2120	Yes
7109	5:**50 X** vs. 34:**0 X**	-2.327	-2.471 to -2.183	Yes
7110	5:**50 X** vs. 34:**1 X**	-2.334	-2.478 to -2.190	Yes

	2way ANOVA<br>Multiple comparisons	A<br>Data Set-A<br>Y	B<br>Data Set-B<br>Y	C<br>Data Set-C<br>Y
7111	5:**50 X** vs. 34:**10 X**	-1.967	-2.111 to -1.823	Yes
7112	5:**50 X** vs. 34:**40 X**	-0.5670	-0.7110 to -0.4230	Yes
7113	5:**50 X** vs. 34:**50 X**	-0.5050	-0.6490 to -0.3610	Yes
7114	5:**50 X** vs. 35:**0 X**	-2.675	-2.819 to -2.531	Yes
7115	5:**50 X** vs. 35:**1 X**	-2.441	-2.585 to -2.297	Yes
7116	5:**50 X** vs. 35:**10 X**	-1.929	-2.073 to -1.785	Yes
7117	5:**50 X** vs. 35:**40 X**	-0.5760	-0.7200 to -0.4320	Yes
7118	5:**50 X** vs. 35:**50 X**	-0.5177	-0.6616 to -0.3737	Yes
7119	5:**50 X** vs. 36:**0 X**	-2.882	-3.043 to -2.721	Yes
7120	5:**50 X** vs. 36:**1 X**	-2.578	-2.722 to -2.434	Yes
7121	5:**50 X** vs. 36:**10 X**	-1.935	-2.079 to -1.791	Yes
7122	5:**50 X** vs. 36:**40 X**	-0.6313	-0.7753 to -0.4874	Yes
7123	5:**50 X** vs. 36:**50 X**	-0.4810	-0.6250 to -0.3370	Yes
7124	5:**50 X** vs. 37:**0 X**	-3.266	-3.410 to -3.122	Yes
7125	5:**50 X** vs. 37:**1 X**	-2.648	-2.792 to -2.504	Yes
7126	5:**50 X** vs. 37:**10 X**	-1.936	-2.080 to -1.792	Yes
7127	5:**50 X** vs. 37:**40 X**	-0.6687	-0.8126 to -0.5247	Yes
7128	5:**50 X** vs. 37:**50 X**	-0.4897	-0.6336 to -0.3457	Yes
7129	5:**50 X** vs. 38:**0 X**	-3.211	-3.355 to -3.067	Yes
7130	5:**50 X** vs. 38:**1 X**	-2.766	-2.910 to -2.622	Yes
7131	5:**50 X** vs. 38:**10 X**	-2.212	-2.356 to -2.068	Yes
7132	5:**50 X** vs. 38:**40 X**	-0.6730	-0.8170 to -0.5290	Yes
7133	5:**50 X** vs. 38:**50 X**	-0.4937	-0.6376 to -0.3497	Yes
7134	5:**50 X** vs. 39:**0 X**	-3.441	-3.585 to -3.297	Yes
7135	5:**50 X** vs. 39:**1 X**	-3.333	-3.477 to -3.189	Yes
7136	5:**50 X** vs. 39:**10 X**	-2.357	-2.501 to -2.213	Yes
7137	5:**50 X** vs. 39:**40 X**	-0.6790	-0.8230 to -0.5350	Yes
7138	5:**50 X** vs. 39:**50 X**	-0.5247	-0.6686 to -0.3807	Yes
7139	5:**50 X** vs. 40:**0 X**	-3.482	-3.626 to -3.338	Yes
7140	5:**50 X** vs. 40:**1 X**	-3.408	-3.552 to -3.264	Yes
7141	5:**50 X** vs. 40:**10 X**	-2.613	-2.757 to -2.469	Yes
7142	5:**50 X** vs. 40:**40 X**	-0.6837	-0.8276 to -0.5397	Yes
7143	5:**50 X** vs. 40:**50 X**	-0.5310	-0.6750 to -0.3870	Yes
7144	5:**50 X** vs. 41:**0 X**	-3.523	-3.667 to -3.379	Yes
7145	5:**50 X** vs. 41:**1 X**	-3.522	-3.666 to -3.378	Yes
7146	5:**50 X** vs. 41:**10 X**	-2.667	-2.811 to -2.523	Yes
7147	5:**50 X** vs. 41:**40 X**	-0.6310	-0.7750 to -0.4870	Yes
7148	5:**50 X** vs. 41:**50 X**	-0.5437	-0.6876 to -0.3997	Yes
7149	5:**50 X** vs. 42:**0 X**	-3.665	-3.809 to -3.521	Yes
7150	5:**50 X** vs. 42:**1 X**	-3.685	-3.829 to -3.541	Yes
7151	5:**50 X** vs. 42:**10 X**	-2.667	-2.811 to -2.523	Yes
7152	5:**50 X** vs. 42:**40 X**	-0.6437	-0.7876 to -0.4997	Yes
7153	5:**50 X** vs. 42:**50 X**	-0.5470	-0.6910 to -0.4030	Yes
7154	5:**50 X** vs. 43:**0 X**	-3.755	-3.899 to -3.611	Yes
7155	5:**50 X** vs. 43:**1 X**	-3.669	-3.813 to -3.525	Yes

	2way ANOVA<br>Multiple comparisons	A<br>Data Set-A<br>Y	B<br>Data Set-B<br>Y	C<br>Data Set-C<br>Y
7156	5:**50 X** vs. 43:**10 X**	-3.385	-3.529 to -3.241	Yes
7157	5:**50 X** vs. 43:**40 X**	-0.6420	-0.7860 to -0.4980	Yes
7158	5:**50 X** vs. 43:**50 X**	-0.5473	-0.6913 to -0.4034	Yes
7159	5:**50 X** vs. 44:**0 X**	-3.779	-3.923 to -3.635	Yes
7160	5:**50 X** vs. 44:**1 X**	-3.669	-3.813 to -3.525	Yes
7161	5:**50 X** vs. 44:**10 X**	-3.448	-3.592 to -3.304	Yes
7162	5:**50 X** vs. 44:**40 X**	-0.6440	-0.7880 to -0.5000	Yes
7163	5:**50 X** vs. 44:**50 X**	-0.4797	-0.6236 to -0.3357	Yes
7164	5:**50 X** vs. 45:**0 X**	-3.644	-3.788 to -3.500	Yes
7165	5:**50 X** vs. 45:**1 X**	-3.446	-3.590 to -3.302	Yes
7166	5:**50 X** vs. 45:**10 X**	-3.328	-3.472 to -3.184	Yes
7167	5:**50 X** vs. 45:**40 X**	-0.6453	-0.7893 to -0.5014	Yes
7168	5:**50 X** vs. 45:**50 X**	-0.4767	-0.6206 to -0.3327	Yes
7169	5:**50 X** vs. 46:**0 X**	-3.448	-3.592 to -3.304	Yes
7170	5:**50 X** vs. 46:**1 X**	-3.415	-3.559 to -3.271	Yes
7171	5:**50 X** vs. 46:**10 X**	-3.323	-3.467 to -3.179	Yes
7172	5:**50 X** vs. 46:**40 X**	-0.6590	-0.8030 to -0.5150	Yes
7173	5:**50 X** vs. 46:**50 X**	-0.4697	-0.6136 to -0.3257	Yes
7174	5:**50 X** vs. 47:**0 X**	-3.424	-3.568 to -3.280	Yes
7175	5:**50 X** vs. 47:**1 X**	-3.328	-3.472 to -3.184	Yes
7176	5:**50 X** vs. 47:**10 X**	-3.309	-3.453 to -3.165	Yes
7177	5:**50 X** vs. 47:**40 X**	-0.6690	-0.8130 to -0.5250	Yes
7178	5:**50 X** vs. 47:**50 X**	-0.4277	-0.5716 to -0.2837	Yes
7179	5:**50 X** vs. 48:**0 X**	-3.411	-3.572 to -3.250	Yes
7180	5:**50 X** vs. 48:**1 X**	-3.315	-3.459 to -3.171	Yes
7181	5:**50 X** vs. 48:**10 X**	-3.309	-3.453 to -3.165	Yes
7182	5:**50 X** vs. 48:**40 X**	-0.6753	-0.8193 to -0.5314	Yes
7183	5:**50 X** vs. 48:**50 X**	-0.4350	-0.5790 to -0.2910	Yes
7184	5:**50 X** vs. 49:**0 X**	-3.411	-3.555 to -3.267	Yes
7185	5:**50 X** vs. 49:**1 X**	-3.315	-3.459 to -3.171	Yes
7186	5:**50 X** vs. 49:**10 X**	-3.309	-3.453 to -3.165	Yes
7187	5:**50 X** vs. 49:**40 X**	-0.6797	-0.8236 to -0.5357	Yes
7188	5:**50 X** vs. 49:**50 X**	-0.4343	-0.5783 to -0.2904	Yes
7189	5:**50 X** vs. 50:**0 X**	-3.411	-3.555 to -3.267	Yes
7190	5:**50 X** vs. 50:**1 X**	-3.315	-3.459 to -3.171	Yes
7191	5:**50 X** vs. 50:**10 X**	-3.309	-3.453 to -3.165	Yes
7192	5:**50 X** vs. 50:**40 X**	-0.6873	-0.8313 to -0.5434	Yes
7193	5:**50 X** vs. 50:**50 X**	-0.4340	-0.5780 to -0.2900	Yes
7194	6:**0 X** vs. 6:**1 X**	0.001000	-0.1430 to 0.1450	No
7195	6:**0 X** vs. 6:**10 X**	-0.01367	-0.1576 to 0.1303	No
7196	6:**0 X** vs. 6:**40 X**	0.005000	-0.1390 to 0.1490	No
7197	6:**0 X** vs. 6:**50 X**	0.006667	-0.1373 to 0.1506	No
7198	6:**0 X** vs. 7:**0 X**	-0.0020	-0.1460 to 0.1420	No
7199	6:**0 X** vs. 7:**1 X**	0.006667	-0.1373 to 0.1506	No
7200	6:**0 X** vs. 7:**10 X**	-0.005333	-0.1493 to 0.1386	No

	2way ANOVA<br>Multiple comparisons	A<br>Data Set-A<br>Y	B<br>Data Set-B<br>Y	C<br>Data Set-C<br>Y
7201	6:**0 X** vs. 7:**40 X**	0.003333	-0.1406 to 0.1473	No
7202	6:**0 X** vs. 7:**50 X**	0.005333	-0.1386 to 0.1493	No
7203	6:**0 X** vs. 8:**0 X**	-0.006667	-0.1506 to 0.1373	No
7204	6:**0 X** vs. 8:**1 X**	0.006667	-0.1373 to 0.1506	No
7205	6:**0 X** vs. 8:**10 X**	-0.0006667	-0.1446 to 0.1433	No
7206	6:**0 X** vs. 8:**40 X**	-0.001333	-0.1453 to 0.1426	No
7207	6:**0 X** vs. 8:**50 X**	0.001667	-0.1423 to 0.1456	No
7208	6:**0 X** vs. 9:**0 X**	-0.01467	-0.1586 to 0.1293	No
7209	6:**0 X** vs. 9:**1 X**	-0.02267	-0.1666 to 0.1213	No
7210	6:**0 X** vs. 9:**10 X**	-0.02367	-0.1676 to 0.1203	No
7211	6:**0 X** vs. 9:**40 X**	-0.006000	-0.1500 to 0.1380	No
7212	6:**0 X** vs. 9:**50 X**	0.0006667	-0.1433 to 0.1446	No
7213	6:**0 X** vs. 10:**0 X**	-0.01633	-0.1603 to 0.1276	No
7214	6:**0 X** vs. 10:**1 X**	-0.02333	-0.1673 to 0.1206	No
7215	6:**0 X** vs. 10:**10 X**	-0.0260	-0.1700 to 0.1180	No
7216	6:**0 X** vs. 10:**40 X**	-0.01567	-0.1596 to 0.1283	No
7217	6:**0 X** vs. 10:**50 X**	-0.002333	-0.1463 to 0.1416	No
7218	6:**0 X** vs. 11:**0 X**	-0.0260	-0.1700 to 0.1180	No
7219	6:**0 X** vs. 11:**1 X**	-0.02767	-0.1716 to 0.1163	No
7220	6:**0 X** vs. 11:**10 X**	-0.0280	-0.1720 to 0.1160	No
7221	6:**0 X** vs. 11:**40 X**	-0.01967	-0.1636 to 0.1243	No
7222	6:**0 X** vs. 11:**50 X**	-0.007667	-0.1516 to 0.1363	No
7223	6:**0 X** vs. 12:**0 X**	-0.02667	-0.1706 to 0.1173	No
7224	6:**0 X** vs. 12:**1 X**	-0.009000	-0.1530 to 0.1350	No
7225	6:**0 X** vs. 12:**10 X**	-0.02667	-0.1706 to 0.1173	No
7226	6:**0 X** vs. 12:**40 X**	-0.0130	-0.1570 to 0.1310	No
7227	6:**0 X** vs. 12:**50 X**	-0.01633	-0.1603 to 0.1276	No
7228	6:**0 X** vs. 13:**0 X**	-0.02067	-0.1646 to 0.1233	No
7229	6:**0 X** vs. 13:**1 X**	-0.008000	-0.1520 to 0.1360	No
7230	6:**0 X** vs. 13:**10 X**	-0.0300	-0.1740 to 0.1140	No
7231	6:**0 X** vs. 13:**40 X**	-0.02633	-0.1703 to 0.1176	No
7232	6:**0 X** vs. 13:**50 X**	-0.02733	-0.1713 to 0.1166	No
7233	6:**0 X** vs. 14:**0 X**	-0.02933	-0.1733 to 0.1146	No
7234	6:**0 X** vs. 14:**1 X**	-0.0210	-0.1650 to 0.1230	No
7235	6:**0 X** vs. 14:**10 X**	-0.0350	-0.1790 to 0.1090	No
7236	6:**0 X** vs. 14:**40 X**	-0.03167	-0.1756 to 0.1123	No
7237	6:**0 X** vs. 14:**50 X**	-0.03567	-0.1796 to 0.1083	No
7238	6:**0 X** vs. 15:**0 X**	-0.04167	-0.1856 to 0.1023	No
7239	6:**0 X** vs. 15:**1 X**	-0.02167	-0.1656 to 0.1223	No
7240	6:**0 X** vs. 15:**10 X**	-0.0490	-0.1930 to 0.09496	No
7241	6:**0 X** vs. 15:**40 X**	-0.03133	-0.1753 to 0.1126	No
7242	6:**0 X** vs. 15:**50 X**	-0.0490	-0.1930 to 0.09496	No
7243	6:**0 X** vs. 16:**0 X**	-0.1340	-0.2780 to 0.009960	No
7244	6:**0 X** vs. 16:**1 X**	-0.0250	-0.1690 to 0.1190	No
7245	6:**0 X** vs. 16:**10 X**	-0.05067	-0.1946 to 0.09329	No

	2way ANOVA<br>Multiple comparisons	A<br>Data Set-A<br>Y	B<br>Data Set-B<br>Y	C<br>Data Set-C<br>Y
7246	6:**0 X** vs. 16:**40 X**	-0.0700	-0.2140 to 0.07396	No
7247	6:**0 X** vs. 16:**50 X**	-0.1123	-0.2563 to 0.03163	No
7248	6:**0 X** vs. 17:**0 X**	-0.1867	-0.3306 to -0.04271	Yes
7249	6:**0 X** vs. 17:**1 X**	-0.1010	-0.2450 to 0.04296	No
7250	6:**0 X** vs. 17:**10 X**	-0.1577	-0.3016 to -0.01371	Yes
7251	6:**0 X** vs. 17:**40 X**	-0.0810	-0.2250 to 0.06296	No
7252	6:**0 X** vs. 17:**50 X**	-0.1263	-0.2703 to 0.01763	No
7253	6:**0 X** vs. 18:**0 X**	-0.2353	-0.3793 to -0.09137	Yes
7254	6:**0 X** vs. 18:**1 X**	-0.1437	-0.2876 to 0.0002932	No
7255	6:**0 X** vs. 18:**10 X**	-0.2360	-0.3800 to -0.09204	Yes
7256	6:**0 X** vs. 18:**40 X**	-0.08533	-0.2293 to 0.05863	No
7257	6:**0 X** vs. 18:**50 X**	-0.1303	-0.2743 to 0.01363	No
7258	6:**0 X** vs. 19:**0 X**	-0.2703	-0.4143 to -0.1264	Yes
7259	6:**0 X** vs. 19:**1 X**	-0.2583	-0.4023 to -0.1144	Yes
7260	6:**0 X** vs. 19:**10 X**	-0.2560	-0.4000 to -0.1120	Yes
7261	6:**0 X** vs. 19:**40 X**	-0.1140	-0.2580 to 0.02996	No
7262	6:**0 X** vs. 19:**50 X**	-0.1250	-0.2690 to 0.01896	No
7263	6:**0 X** vs. 20:**0 X**	-0.3500	-0.4940 to -0.2060	Yes
7264	6:**0 X** vs. 20:**1 X**	-0.1693	-0.3133 to -0.02537	Yes
7265	6:**0 X** vs. 20:**10 X**	-0.2583	-0.4023 to -0.1144	Yes
7266	6:**0 X** vs. 20:**40 X**	-0.1257	-0.2696 to 0.01829	No
7267	6:**0 X** vs. 20:**50 X**	-0.1697	-0.3136 to -0.02571	Yes
7268	6:**0 X** vs. 21:**0 X**	-0.3733	-0.5173 to -0.2294	Yes
7269	6:**0 X** vs. 21:**1 X**	-0.3263	-0.4703 to -0.1824	Yes
7270	6:**0 X** vs. 21:**10 X**	-0.3233	-0.4673 to -0.1794	Yes
7271	6:**0 X** vs. 21:**40 X**	-0.1987	-0.3426 to -0.05471	Yes
7272	6:**0 X** vs. 21:**50 X**	-0.2890	-0.4330 to -0.1450	Yes
7273	6:**0 X** vs. 22:**0 X**	-0.4150	-0.5590 to -0.2710	Yes
7274	6:**0 X** vs. 22:**1 X**	-0.4097	-0.5536 to -0.2657	Yes
7275	6:**0 X** vs. 22:**10 X**	-0.4050	-0.5490 to -0.2610	Yes
7276	6:**0 X** vs. 22:**40 X**	-0.3053	-0.4493 to -0.1614	Yes
7277	6:**0 X** vs. 22:**50 X**	-0.3680	-0.5120 to -0.2240	Yes
7278	6:**0 X** vs. 23:**0 X**	-0.4803	-0.6243 to -0.3364	Yes
7279	6:**0 X** vs. 23:**1 X**	-0.6700	-0.8310 to -0.5090	Yes
7280	6:**0 X** vs. 23:**10 X**	-0.4197	-0.5636 to -0.2757	Yes
7281	6:**0 X** vs. 23:**40 X**	-0.3000	-0.4610 to -0.1390	Yes
7282	6:**0 X** vs. 23:**50 X**	-0.3340	-0.4780 to -0.1900	Yes
7283	6:**0 X** vs. 24:**0 X**	-0.5977	-0.7416 to -0.4537	Yes
7284	6:**0 X** vs. 24:**1 X**	-0.7540	-0.9150 to -0.5930	Yes
7285	6:**0 X** vs. 24:**10 X**	-0.5815	-0.7425 to -0.4205	Yes
7286	6:**0 X** vs. 24:**40 X**	-0.3163	-0.4603 to -0.1724	Yes
7287	6:**0 X** vs. 24:**50 X**	-0.2380	-0.3820 to -0.09404	Yes
7288	6:**0 X** vs. 25:**0 X**	-0.9350	-1.096 to -0.7740	Yes
7289	6:**0 X** vs. 25:**1 X**	-1.125	-1.285 to -0.9635	Yes
7290	6:**0 X** vs. 25:**10 X**	-0.4860	-0.6896 to -0.2824	Yes

	2way ANOVA<br>Multiple comparisons	A<br>Data Set-A<br>Y	B<br>Data Set-B<br>Y	C<br>Data Set-C<br>Y
7291	6:0 X vs. 25:40 X	-0.3183	-0.4623 to -0.1744	Yes
7292	6:0 X vs. 25:50 X	-0.2927	-0.4366 to -0.1487	Yes
7293	6:0 X vs. 26:0 X	-1.148	-1.308 to -0.9865	Yes
7294	6:0 X vs. 26:1 X	-1.261	-1.421 to -1.100	Yes
7295	6:0 X vs. 26:10 X	-0.9470	-1.108 to -0.7860	Yes
7296	6:0 X vs. 26:40 X	-0.3927	-0.5366 to -0.2487	Yes
7297	6:0 X vs. 26:50 X	-0.2530	-0.3970 to -0.1090	Yes
7298	6:0 X vs. 27:0 X	-1.335	-1.479 to -1.191	Yes
7299	6:0 X vs. 27:1 X	-1.426	-1.587 to -1.265	Yes
7300	6:0 X vs. 27:10 X	-1.238	-1.399 to -1.077	Yes
7301	6:0 X vs. 27:40 X	-0.3927	-0.5366 to -0.2487	Yes
7302	6:0 X vs. 27:50 X	-0.2523	-0.3963 to -0.1084	Yes
7303	6:0 X vs. 28:0 X	-1.285	-1.445 to -1.124	Yes
7304	6:0 X vs. 28:1 X	-1.325	-1.469 to -1.181	Yes
7305	6:0 X vs. 28:10 X	-1.127	-1.288 to -0.9660	Yes
7306	6:0 X vs. 28:40 X	-0.4050	-0.5490 to -0.2610	Yes
7307	6:0 X vs. 28:50 X	-0.3003	-0.4443 to -0.1564	Yes
7308	6:0 X vs. 29:0 X	-1.766	-1.927 to -1.605	Yes
7309	6:0 X vs. 29:1 X	-1.851	-2.012 to -1.690	Yes
7310	6:0 X vs. 29:10 X	-1.066	-1.226 to -0.9045	Yes
7311	6:0 X vs. 29:40 X	-0.5130	-0.6570 to -0.3690	Yes
7312	6:0 X vs. 29:50 X	-0.3017	-0.4456 to -0.1577	Yes
7313	6:0 X vs. 30:0 X	-1.481	-1.641 to -1.320	Yes
7314	6:0 X vs. 30:1 X	-1.802	-1.963 to -1.641	Yes
7315	6:0 X vs. 30:10 X	-1.140	-1.301 to -0.9790	Yes
7316	6:0 X vs. 30:40 X	-0.4400	-0.5840 to -0.2960	Yes
7317	6:0 X vs. 30:50 X	-0.3540	-0.4980 to -0.2100	Yes
7318	6:0 X vs. 31:0 X	-1.799	-1.959 to -1.638	Yes
7319	6:0 X vs. 31:1 X	-1.789	-1.950 to -1.628	Yes
7320	6:0 X vs. 31:10 X	-1.272	-1.432 to -1.111	Yes
7321	6:0 X vs. 31:40 X	-0.5317	-0.6756 to -0.3877	Yes
7322	6:0 X vs. 31:50 X	-0.3330	-0.4770 to -0.1890	Yes
7323	6:0 X vs. 32:0 X	-2.390	-2.534 to -2.246	Yes
7324	6:0 X vs. 32:1 X	-1.846	-2.007 to -1.685	Yes
7325	6:0 X vs. 32:10 X	-1.702	-1.846 to -1.558	Yes
7326	6:0 X vs. 32:40 X	-0.5280	-0.6720 to -0.3840	Yes
7327	6:0 X vs. 32:50 X	-0.3327	-0.4766 to -0.1887	Yes
7328	6:0 X vs. 33:0 X	-2.394	-2.538 to -2.250	Yes
7329	6:0 X vs. 33:1 X	-2.208	-2.352 to -2.064	Yes
7330	6:0 X vs. 33:10 X	-1.869	-2.013 to -1.725	Yes
7331	6:0 X vs. 33:40 X	-0.5467	-0.6906 to -0.4027	Yes
7332	6:0 X vs. 33:50 X	-0.3477	-0.4916 to -0.2037	Yes
7333	6:0 X vs. 34:0 X	-2.318	-2.462 to -2.174	Yes
7334	6:0 X vs. 34:1 X	-2.326	-2.470 to -2.182	Yes
7335	6:0 X vs. 34:10 X	-1.959	-2.103 to -1.815	Yes

	2way ANOVA<br>Multiple comparisons	A<br>Data Set-A<br>Y	B<br>Data Set-B<br>Y	C<br>Data Set-C<br>Y
7336	6:0 X vs. 34:40 X	-0.5587	-0.7026 to -0.4147	Yes
7337	6:0 X vs. 34:50 X	-0.4967	-0.6406 to -0.3527	Yes
7338	6:0 X vs. 35:0 X	-2.667	-2.811 to -2.523	Yes
7339	6:0 X vs. 35:1 X	-2.433	-2.577 to -2.289	Yes
7340	6:0 X vs. 35:10 X	-1.921	-2.065 to -1.777	Yes
7341	6:0 X vs. 35:40 X	-0.5677	-0.7116 to -0.4237	Yes
7342	6:0 X vs. 35:50 X	-0.5093	-0.6533 to -0.3654	Yes
7343	6:0 X vs. 36:0 X	-2.874	-3.034 to -2.713	Yes
7344	6:0 X vs. 36:1 X	-2.569	-2.713 to -2.425	Yes
7345	6:0 X vs. 36:10 X	-1.926	-2.070 to -1.782	Yes
7346	6:0 X vs. 36:40 X	-0.6230	-0.7670 to -0.4790	Yes
7347	6:0 X vs. 36:50 X	-0.4727	-0.6166 to -0.3287	Yes
7348	6:0 X vs. 37:0 X	-3.257	-3.401 to -3.113	Yes
7349	6:0 X vs. 37:1 X	-2.639	-2.783 to -2.495	Yes
7350	6:0 X vs. 37:10 X	-1.928	-2.072 to -1.784	Yes
7351	6:0 X vs. 37:40 X	-0.6603	-0.8043 to -0.5164	Yes
7352	6:0 X vs. 37:50 X	-0.4813	-0.6253 to -0.3374	Yes
7353	6:0 X vs. 38:0 X	-3.202	-3.346 to -3.058	Yes
7354	6:0 X vs. 38:1 X	-2.758	-2.902 to -2.614	Yes
7355	6:0 X vs. 38:10 X	-2.203	-2.347 to -2.059	Yes
7356	6:0 X vs. 38:40 X	-0.6647	-0.8086 to -0.5207	Yes
7357	6:0 X vs. 38:50 X	-0.4853	-0.6293 to -0.3414	Yes
7358	6:0 X vs. 39:0 X	-3.433	-3.577 to -3.289	Yes
7359	6:0 X vs. 39:1 X	-3.325	-3.469 to -3.181	Yes
7360	6:0 X vs. 39:10 X	-2.348	-2.492 to -2.204	Yes
7361	6:0 X vs. 39:40 X	-0.6707	-0.8146 to -0.5267	Yes
7362	6:0 X vs. 39:50 X	-0.5163	-0.6603 to -0.3724	Yes
7363	6:0 X vs. 40:0 X	-3.474	-3.618 to -3.330	Yes
7364	6:0 X vs. 40:1 X	-3.400	-3.544 to -3.256	Yes
7365	6:0 X vs. 40:10 X	-2.605	-2.749 to -2.461	Yes
7366	6:0 X vs. 40:40 X	-0.6753	-0.8193 to -0.5314	Yes
7367	6:0 X vs. 40:50 X	-0.5227	-0.6666 to -0.3787	Yes
7368	6:0 X vs. 41:0 X	-3.515	-3.659 to -3.371	Yes
7369	6:0 X vs. 41:1 X	-3.514	-3.658 to -3.370	Yes
7370	6:0 X vs. 41:10 X	-2.658	-2.802 to -2.514	Yes
7371	6:0 X vs. 41:40 X	-0.6227	-0.7666 to -0.4787	Yes
7372	6:0 X vs. 41:50 X	-0.5353	-0.6793 to -0.3914	Yes
7373	6:0 X vs. 42:0 X	-3.657	-3.801 to -3.513	Yes
7374	6:0 X vs. 42:1 X	-3.676	-3.820 to -3.532	Yes
7375	6:0 X vs. 42:10 X	-2.659	-2.803 to -2.515	Yes
7376	6:0 X vs. 42:40 X	-0.6353	-0.7793 to -0.4914	Yes
7377	6:0 X vs. 42:50 X	-0.5387	-0.6826 to -0.3947	Yes
7378	6:0 X vs. 43:0 X	-3.747	-3.891 to -3.603	Yes
7379	6:0 X vs. 43:1 X	-3.661	-3.805 to -3.517	Yes
7380	6:0 X vs. 43:10 X	-3.377	-3.521 to -3.233	Yes

	2way ANOVA<br>Multiple comparisons	A<br>Data Set-A<br>Y	B<br>Data Set-B<br>Y	C<br>Data Set-C<br>Y
7381	6:**0 X** vs. 43:**40 X**	-0.6337	-0.7776 to -0.4897	Yes
7382	6:**0 X** vs. 43:**50 X**	-0.5390	-0.6830 to -0.3950	Yes
7383	6:**0 X** vs. 44:**0 X**	-3.771	-3.915 to -3.627	Yes
7384	6:**0 X** vs. 44:**1 X**	-3.660	-3.804 to -3.516	Yes
7385	6:**0 X** vs. 44:**10 X**	-3.440	-3.584 to -3.296	Yes
7386	6:**0 X** vs. 44:**40 X**	-0.6357	-0.7796 to -0.4917	Yes
7387	6:**0 X** vs. 44:**50 X**	-0.4713	-0.6153 to -0.3274	Yes
7388	6:**0 X** vs. 45:**0 X**	-3.635	-3.779 to -3.491	Yes
7389	6:**0 X** vs. 45:**1 X**	-3.437	-3.581 to -3.293	Yes
7390	6:**0 X** vs. 45:**10 X**	-3.320	-3.464 to -3.176	Yes
7391	6:**0 X** vs. 45:**40 X**	-0.6370	-0.7810 to -0.4930	Yes
7392	6:**0 X** vs. 45:**50 X**	-0.4683	-0.6123 to -0.3244	Yes
7393	6:**0 X** vs. 46:**0 X**	-3.440	-3.584 to -3.296	Yes
7394	6:**0 X** vs. 46:**1 X**	-3.407	-3.551 to -3.263	Yes
7395	6:**0 X** vs. 46:**10 X**	-3.315	-3.459 to -3.171	Yes
7396	6:**0 X** vs. 46:**40 X**	-0.6507	-0.7946 to -0.5067	Yes
7397	6:**0 X** vs. 46:**50 X**	-0.4613	-0.6053 to -0.3174	Yes
7398	6:**0 X** vs. 47:**0 X**	-3.415	-3.559 to -3.271	Yes
7399	6:**0 X** vs. 47:**1 X**	-3.319	-3.463 to -3.175	Yes
7400	6:**0 X** vs. 47:**10 X**	-3.301	-3.445 to -3.157	Yes
7401	6:**0 X** vs. 47:**40 X**	-0.6607	-0.8046 to -0.5167	Yes
7402	6:**0 X** vs. 47:**50 X**	-0.4193	-0.5633 to -0.2754	Yes
7403	6:**0 X** vs. 48:**0 X**	-3.403	-3.563 to -3.242	Yes
7404	6:**0 X** vs. 48:**1 X**	-3.306	-3.450 to -3.162	Yes
7405	6:**0 X** vs. 48:**10 X**	-3.301	-3.445 to -3.157	Yes
7406	6:**0 X** vs. 48:**40 X**	-0.6670	-0.8110 to -0.5230	Yes
7407	6:**0 X** vs. 48:**50 X**	-0.4267	-0.5706 to -0.2827	Yes
7408	6:**0 X** vs. 49:**0 X**	-3.403	-3.547 to -3.259	Yes
7409	6:**0 X** vs. 49:**1 X**	-3.306	-3.450 to -3.162	Yes
7410	6:**0 X** vs. 49:**10 X**	-3.301	-3.445 to -3.157	Yes
7411	6:**0 X** vs. 49:**40 X**	-0.6713	-0.8153 to -0.5274	Yes
7412	6:**0 X** vs. 49:**50 X**	-0.4260	-0.5700 to -0.2820	Yes
7413	6:**0 X** vs. 50:**0 X**	-3.403	-3.547 to -3.259	Yes
7414	6:**0 X** vs. 50:**1 X**	-3.306	-3.450 to -3.162	Yes
7415	6:**0 X** vs. 50:**10 X**	-3.301	-3.445 to -3.157	Yes
7416	6:**0 X** vs. 50:**40 X**	-0.6790	-0.8230 to -0.5350	Yes
7417	6:**0 X** vs. 50:**50 X**	-0.4257	-0.5696 to -0.2817	Yes
7418	6:**1 X** vs. 6:**10 X**	-0.01467	-0.1586 to 0.1293	No
7419	6:**1 X** vs. 6:**40 X**	0.004000	-0.1400 to 0.1480	No
7420	6:**1 X** vs. 6:**50 X**	0.005667	-0.1383 to 0.1496	No
7421	6:**1 X** vs. 7:**0 X**	-0.0030	-0.1470 to 0.1410	No
7422	6:**1 X** vs. 7:**1 X**	0.005667	-0.1383 to 0.1496	No
7423	6:**1 X** vs. 7:**10 X**	-0.006333	-0.1503 to 0.1376	No
7424	6:**1 X** vs. 7:**40 X**	0.002333	-0.1416 to 0.1463	No
7425	6:**1 X** vs. 7:**50 X**	0.004333	-0.1396 to 0.1483	No

	2way ANOVA<br>Multiple comparisons	A<br>Data Set-A<br>Y	B<br>Data Set-B<br>Y	C<br>Data Set-C<br>Y
7426	6:1 X vs. 8:0 X	-0.007667	-0.1516 to 0.1363	No
7427	6:1 X vs. 8:1 X	0.005667	-0.1383 to 0.1496	No
7428	6:1 X vs. 8:10 X	-0.001667	-0.1456 to 0.1423	No
7429	6:1 X vs. 8:40 X	-0.002333	-0.1463 to 0.1416	No
7430	6:1 X vs. 8:50 X	0.0006667	-0.1433 to 0.1446	No
7431	6:1 X vs. 9:0 X	-0.01567	-0.1596 to 0.1283	No
7432	6:1 X vs. 9:1 X	-0.02367	-0.1676 to 0.1203	No
7433	6:1 X vs. 9:10 X	-0.02467	-0.1686 to 0.1193	No
7434	6:1 X vs. 9:40 X	-0.007000	-0.1510 to 0.1370	No
7435	6:1 X vs. 9:50 X	-0.0003333	-0.1443 to 0.1436	No
7436	6:1 X vs. 10:0 X	-0.01733	-0.1613 to 0.1266	No
7437	6:1 X vs. 10:1 X	-0.02433	-0.1683 to 0.1196	No
7438	6:1 X vs. 10:10 X	-0.0270	-0.1710 to 0.1170	No
7439	6:1 X vs. 10:40 X	-0.01667	-0.1606 to 0.1273	No
7440	6:1 X vs. 10:50 X	-0.003333	-0.1473 to 0.1406	No
7441	6:1 X vs. 11:0 X	-0.0270	-0.1710 to 0.1170	No
7442	6:1 X vs. 11:1 X	-0.02867	-0.1726 to 0.1153	No
7443	6:1 X vs. 11:10 X	-0.0290	-0.1730 to 0.1150	No
7444	6:1 X vs. 11:40 X	-0.02067	-0.1646 to 0.1233	No
7445	6:1 X vs. 11:50 X	-0.008667	-0.1526 to 0.1353	No
7446	6:1 X vs. 12:0 X	-0.02767	-0.1716 to 0.1163	No
7447	6:1 X vs. 12:1 X	-0.0100	-0.1540 to 0.1340	No
7448	6:1 X vs. 12:10 X	-0.02767	-0.1716 to 0.1163	No
7449	6:1 X vs. 12:40 X	-0.0140	-0.1580 to 0.1300	No
7450	6:1 X vs. 12:50 X	-0.01733	-0.1613 to 0.1266	No
7451	6:1 X vs. 13:0 X	-0.02167	-0.1656 to 0.1223	No
7452	6:1 X vs. 13:1 X	-0.0090	-0.1530 to 0.1350	No
7453	6:1 X vs. 13:10 X	-0.0310	-0.1750 to 0.1130	No
7454	6:1 X vs. 13:40 X	-0.02733	-0.1713 to 0.1166	No
7455	6:1 X vs. 13:50 X	-0.02833	-0.1723 to 0.1156	No
7456	6:1 X vs. 14:0 X	-0.03033	-0.1743 to 0.1136	No
7457	6:1 X vs. 14:1 X	-0.0220	-0.1660 to 0.1220	No
7458	6:1 X vs. 14:10 X	-0.0360	-0.1800 to 0.1080	No
7459	6:1 X vs. 14:40 X	-0.03267	-0.1766 to 0.1113	No
7460	6:1 X vs. 14:50 X	-0.03667	-0.1806 to 0.1073	No
7461	6:1 X vs. 15:0 X	-0.04267	-0.1866 to 0.1013	No
7462	6:1 X vs. 15:1 X	-0.02267	-0.1666 to 0.1213	No
7463	6:1 X vs. 15:10 X	-0.0500	-0.1940 to 0.09396	No
7464	6:1 X vs. 15:40 X	-0.03233	-0.1763 to 0.1116	No
7465	6:1 X vs. 15:50 X	-0.0500	-0.1940 to 0.09396	No
7466	6:1 X vs. 16:0 X	-0.1350	-0.2790 to 0.008960	No
7467	6:1 X vs. 16:1 X	-0.0260	-0.1700 to 0.1180	No
7468	6:1 X vs. 16:10 X	-0.05167	-0.1956 to 0.09229	No
7469	6:1 X vs. 16:40 X	-0.0710	-0.2150 to 0.07296	No
7470	6:1 X vs. 16:50 X	-0.1133	-0.2573 to 0.03063	No

	2way ANOVA<br>Multiple comparisons	A<br>Data Set-A<br>Y	B<br>Data Set-B<br>Y	C<br>Data Set-C<br>Y
7471	6:**1 X** vs. 17:**0 X**	-0.1877	-0.3316 to -0.04371	Yes
7472	6:**1 X** vs. 17:**1 X**	-0.1020	-0.2460 to 0.04196	No
7473	6:**1 X** vs. 17:**10 X**	-0.1587	-0.3026 to -0.01471	Yes
7474	6:**1 X** vs. 17:**40 X**	-0.0820	-0.2260 to 0.06196	No
7475	6:**1 X** vs. 17:**50 X**	-0.1273	-0.2713 to 0.01663	No
7476	6:**1 X** vs. 18:**0 X**	-0.2363	-0.3803 to -0.09237	Yes
7477	6:**1 X** vs. 18:**1 X**	-0.1447	-0.2886 to -0.0007068	Yes
7478	6:**1 X** vs. 18:**10 X**	-0.2370	-0.3810 to -0.09304	Yes
7479	6:**1 X** vs. 18:**40 X**	-0.08633	-0.2303 to 0.05763	No
7480	6:**1 X** vs. 18:**50 X**	-0.1313	-0.2753 to 0.01263	No
7481	6:**1 X** vs. 19:**0 X**	-0.2713	-0.4153 to -0.1274	Yes
7482	6:**1 X** vs. 19:**1 X**	-0.2593	-0.4033 to -0.1154	Yes
7483	6:**1 X** vs. 19:**10 X**	-0.2570	-0.4010 to -0.1130	Yes
7484	6:**1 X** vs. 19:**40 X**	-0.1150	-0.2590 to 0.02896	No
7485	6:**1 X** vs. 19:**50 X**	-0.1260	-0.2700 to 0.01796	No
7486	6:**1 X** vs. 20:**0 X**	-0.3510	-0.4950 to -0.2070	Yes
7487	6:**1 X** vs. 20:**1 X**	-0.1703	-0.3143 to -0.02637	Yes
7488	6:**1 X** vs. 20:**10 X**	-0.2593	-0.4033 to -0.1154	Yes
7489	6:**1 X** vs. 20:**40 X**	-0.1267	-0.2706 to 0.01729	No
7490	6:**1 X** vs. 20:**50 X**	-0.1707	-0.3146 to -0.02671	Yes
7491	6:**1 X** vs. 21:**0 X**	-0.3743	-0.5183 to -0.2304	Yes
7492	6:**1 X** vs. 21:**1 X**	-0.3273	-0.4713 to -0.1834	Yes
7493	6:**1 X** vs. 21:**10 X**	-0.3243	-0.4683 to -0.1804	Yes
7494	6:**1 X** vs. 21:**40 X**	-0.1997	-0.3436 to -0.05571	Yes
7495	6:**1 X** vs. 21:**50 X**	-0.2900	-0.4340 to -0.1460	Yes
7496	6:**1 X** vs. 22:**0 X**	-0.4160	-0.5600 to -0.2720	Yes
7497	6:**1 X** vs. 22:**1 X**	-0.4107	-0.5546 to -0.2667	Yes
7498	6:**1 X** vs. 22:**10 X**	-0.4060	-0.5500 to -0.2620	Yes
7499	6:**1 X** vs. 22:**40 X**	-0.3063	-0.4503 to -0.1624	Yes
7500	6:**1 X** vs. 22:**50 X**	-0.3690	-0.5130 to -0.2250	Yes
7501	6:**1 X** vs. 23:**0 X**	-0.4813	-0.6253 to -0.3374	Yes
7502	6:**1 X** vs. 23:**1 X**	-0.6710	-0.8320 to -0.5100	Yes
7503	6:**1 X** vs. 23:**10 X**	-0.4207	-0.5646 to -0.2767	Yes
7504	6:**1 X** vs. 23:**40 X**	-0.3010	-0.4620 to -0.1400	Yes
7505	6:**1 X** vs. 23:**50 X**	-0.3350	-0.4790 to -0.1910	Yes
7506	6:**1 X** vs. 24:**0 X**	-0.5987	-0.7426 to -0.4547	Yes
7507	6:**1 X** vs. 24:**1 X**	-0.7550	-0.9160 to -0.5940	Yes
7508	6:**1 X** vs. 24:**10 X**	-0.5825	-0.7435 to -0.4215	Yes
7509	6:**1 X** vs. 24:**40 X**	-0.3173	-0.4613 to -0.1734	Yes
7510	6:**1 X** vs. 24:**50 X**	-0.2390	-0.3830 to -0.09504	Yes
7511	6:**1 X** vs. 25:**0 X**	-0.9360	-1.097 to -0.7750	Yes
7512	6:**1 X** vs. 25:**1 X**	-1.126	-1.286 to -0.9645	Yes
7513	6:**1 X** vs. 25:**10 X**	-0.4870	-0.6906 to -0.2834	Yes
7514	6:**1 X** vs. 25:**40 X**	-0.3193	-0.4633 to -0.1754	Yes
7515	6:**1 X** vs. 25:**50 X**	-0.2937	-0.4376 to -0.1497	Yes

	2way ANOVA<br>Multiple comparisons	A<br>Data Set-A<br>Y	B<br>Data Set-B<br>Y	C<br>Data Set-C<br>Y
7516	6:1 X vs. 26:0 X	-1.149	-1.309 to -0.9875	Yes
7517	6:1 X vs. 26:1 X	-1.262	-1.422 to -1.101	Yes
7518	6:1 X vs. 26:10 X	-0.9480	-1.109 to -0.7870	Yes
7519	6:1 X vs. 26:40 X	-0.3937	-0.5376 to -0.2497	Yes
7520	6:1 X vs. 26:50 X	-0.2540	-0.3980 to -0.1100	Yes
7521	6:1 X vs. 27:0 X	-1.336	-1.480 to -1.192	Yes
7522	6:1 X vs. 27:1 X	-1.427	-1.588 to -1.266	Yes
7523	6:1 X vs. 27:10 X	-1.239	-1.400 to -1.078	Yes
7524	6:1 X vs. 27:40 X	-0.3937	-0.5376 to -0.2497	Yes
7525	6:1 X vs. 27:50 X	-0.2533	-0.3973 to -0.1094	Yes
7526	6:1 X vs. 28:0 X	-1.286	-1.446 to -1.125	Yes
7527	6:1 X vs. 28:1 X	-1.326	-1.470 to -1.182	Yes
7528	6:1 X vs. 28:10 X	-1.128	-1.289 to -0.9670	Yes
7529	6:1 X vs. 28:40 X	-0.4060	-0.5500 to -0.2620	Yes
7530	6:1 X vs. 28:50 X	-0.3013	-0.4453 to -0.1574	Yes
7531	6:1 X vs. 29:0 X	-1.767	-1.928 to -1.606	Yes
7532	6:1 X vs. 29:1 X	-1.852	-2.013 to -1.691	Yes
7533	6:1 X vs. 29:10 X	-1.067	-1.227 to -0.9055	Yes
7534	6:1 X vs. 29:40 X	-0.5140	-0.6580 to -0.3700	Yes
7535	6:1 X vs. 29:50 X	-0.3027	-0.4466 to -0.1587	Yes
7536	6:1 X vs. 30:0 X	-1.482	-1.642 to -1.321	Yes
7537	6:1 X vs. 30:1 X	-1.803	-1.964 to -1.642	Yes
7538	6:1 X vs. 30:10 X	-1.141	-1.302 to -0.9800	Yes
7539	6:1 X vs. 30:40 X	-0.4410	-0.5850 to -0.2970	Yes
7540	6:1 X vs. 30:50 X	-0.3550	-0.4990 to -0.2110	Yes
7541	6:1 X vs. 31:0 X	-1.800	-1.960 to -1.639	Yes
7542	6:1 X vs. 31:1 X	-1.790	-1.951 to -1.629	Yes
7543	6:1 X vs. 31:10 X	-1.273	-1.433 to -1.112	Yes
7544	6:1 X vs. 31:40 X	-0.5327	-0.6766 to -0.3887	Yes
7545	6:1 X vs. 31:50 X	-0.3340	-0.4780 to -0.1900	Yes
7546	6:1 X vs. 32:0 X	-2.391	-2.535 to -2.247	Yes
7547	6:1 X vs. 32:1 X	-1.847	-2.008 to -1.686	Yes
7548	6:1 X vs. 32:10 X	-1.703	-1.847 to -1.559	Yes
7549	6:1 X vs. 32:40 X	-0.5290	-0.6730 to -0.3850	Yes
7550	6:1 X vs. 32:50 X	-0.3337	-0.4776 to -0.1897	Yes
7551	6:1 X vs. 33:0 X	-2.395	-2.539 to -2.251	Yes
7552	6:1 X vs. 33:1 X	-2.209	-2.353 to -2.065	Yes
7553	6:1 X vs. 33:10 X	-1.870	-2.014 to -1.726	Yes
7554	6:1 X vs. 33:40 X	-0.5477	-0.6916 to -0.4037	Yes
7555	6:1 X vs. 33:50 X	-0.3487	-0.4926 to -0.2047	Yes
7556	6:1 X vs. 34:0 X	-2.319	-2.463 to -2.175	Yes
7557	6:1 X vs. 34:1 X	-2.327	-2.471 to -2.183	Yes
7558	6:1 X vs. 34:10 X	-1.960	-2.104 to -1.816	Yes
7559	6:1 X vs. 34:40 X	-0.5597	-0.7036 to -0.4157	Yes
7560	6:1 X vs. 34:50 X	-0.4977	-0.6416 to -0.3537	Yes

2way ANOVA<br>Multiple comparisons		A<br>Data Set-A<br>Y	B<br>Data Set-B<br>Y	C<br>Data Set-C<br>Y
7561	6:1 X vs. 35:0 X	-2.668	-2.812 to -2.524	Yes
7562	6:1 X vs. 35:1 X	-2.434	-2.578 to -2.290	Yes
7563	6:1 X vs. 35:10 X	-1.922	-2.066 to -1.778	Yes
7564	6:1 X vs. 35:40 X	-0.5687	-0.7126 to -0.4247	Yes
7565	6:1 X vs. 35:50 X	-0.5103	-0.6543 to -0.3664	Yes
7566	6:1 X vs. 36:0 X	-2.875	-3.035 to -2.714	Yes
7567	6:1 X vs. 36:1 X	-2.570	-2.714 to -2.426	Yes
7568	6:1 X vs. 36:10 X	-1.927	-2.071 to -1.783	Yes
7569	6:1 X vs. 36:40 X	-0.6240	-0.7680 to -0.4800	Yes
7570	6:1 X vs. 36:50 X	-0.4737	-0.6176 to -0.3297	Yes
7571	6:1 X vs. 37:0 X	-3.258	-3.402 to -3.114	Yes
7572	6:1 X vs. 37:1 X	-2.640	-2.784 to -2.496	Yes
7573	6:1 X vs. 37:10 X	-1.929	-2.073 to -1.785	Yes
7574	6:1 X vs. 37:40 X	-0.6613	-0.8053 to -0.5174	Yes
7575	6:1 X vs. 37:50 X	-0.4823	-0.6263 to -0.3384	Yes
7576	6:1 X vs. 38:0 X	-3.203	-3.347 to -3.059	Yes
7577	6:1 X vs. 38:1 X	-2.759	-2.903 to -2.615	Yes
7578	6:1 X vs. 38:10 X	-2.204	-2.348 to -2.060	Yes
7579	6:1 X vs. 38:40 X	-0.6657	-0.8096 to -0.5217	Yes
7580	6:1 X vs. 38:50 X	-0.4863	-0.6303 to -0.3424	Yes
7581	6:1 X vs. 39:0 X	-3.434	-3.578 to -3.290	Yes
7582	6:1 X vs. 39:1 X	-3.326	-3.470 to -3.182	Yes
7583	6:1 X vs. 39:10 X	-2.349	-2.493 to -2.205	Yes
7584	6:1 X vs. 39:40 X	-0.6717	-0.8156 to -0.5277	Yes
7585	6:1 X vs. 39:50 X	-0.5173	-0.6613 to -0.3734	Yes
7586	6:1 X vs. 40:0 X	-3.475	-3.619 to -3.331	Yes
7587	6:1 X vs. 40:1 X	-3.401	-3.545 to -3.257	Yes
7588	6:1 X vs. 40:10 X	-2.606	-2.750 to -2.462	Yes
7589	6:1 X vs. 40:40 X	-0.6763	-0.8203 to -0.5324	Yes
7590	6:1 X vs. 40:50 X	-0.5237	-0.6676 to -0.3797	Yes
7591	6:1 X vs. 41:0 X	-3.516	-3.660 to -3.372	Yes
7592	6:1 X vs. 41:1 X	-3.515	-3.659 to -3.371	Yes
7593	6:1 X vs. 41:10 X	-2.659	-2.803 to -2.515	Yes
7594	6:1 X vs. 41:40 X	-0.6237	-0.7676 to -0.4797	Yes
7595	6:1 X vs. 41:50 X	-0.5363	-0.6803 to -0.3924	Yes
7596	6:1 X vs. 42:0 X	-3.658	-3.802 to -3.514	Yes
7597	6:1 X vs. 42:1 X	-3.677	-3.821 to -3.533	Yes
7598	6:1 X vs. 42:10 X	-2.660	-2.804 to -2.516	Yes
7599	6:1 X vs. 42:40 X	-0.6363	-0.7803 to -0.4924	Yes
7600	6:1 X vs. 42:50 X	-0.5397	-0.6836 to -0.3957	Yes
7601	6:1 X vs. 43:0 X	-3.748	-3.892 to -3.604	Yes
7602	6:1 X vs. 43:1 X	-3.662	-3.806 to -3.518	Yes
7603	6:1 X vs. 43:10 X	-3.378	-3.522 to -3.234	Yes
7604	6:1 X vs. 43:40 X	-0.6347	-0.7786 to -0.4907	Yes
7605	6:1 X vs. 43:50 X	-0.5400	-0.6840 to -0.3960	Yes

2way ANOVA<br>Multiple comparisons		A<br>Data Set-A<br>Y	B<br>Data Set-B<br>Y	C<br>Data Set-C<br>Y
7606	6:**1 X** vs. 44:**0 X**	-3.772	-3.916 to -3.628	Yes
7607	6:**1 X** vs. 44:**1 X**	-3.661	-3.805 to -3.517	Yes
7608	6:**1 X** vs. 44:**10 X**	-3.441	-3.585 to -3.297	Yes
7609	6:**1 X** vs. 44:**40 X**	-0.6367	-0.7806 to -0.4927	Yes
7610	6:**1 X** vs. 44:**50 X**	-0.4723	-0.6163 to -0.3284	Yes
7611	6:**1 X** vs. 45:**0 X**	-3.636	-3.780 to -3.492	Yes
7612	6:**1 X** vs. 45:**1 X**	-3.438	-3.582 to -3.294	Yes
7613	6:**1 X** vs. 45:**10 X**	-3.321	-3.465 to -3.177	Yes
7614	6:**1 X** vs. 45:**40 X**	-0.6380	-0.7820 to -0.4940	Yes
7615	6:**1 X** vs. 45:**50 X**	-0.4693	-0.6133 to -0.3254	Yes
7616	6:**1 X** vs. 46:**0 X**	-3.441	-3.585 to -3.297	Yes
7617	6:**1 X** vs. 46:**1 X**	-3.408	-3.552 to -3.264	Yes
7618	6:**1 X** vs. 46:**10 X**	-3.316	-3.460 to -3.172	Yes
7619	6:**1 X** vs. 46:**40 X**	-0.6517	-0.7956 to -0.5077	Yes
7620	6:**1 X** vs. 46:**50 X**	-0.4623	-0.6063 to -0.3184	Yes
7621	6:**1 X** vs. 47:**0 X**	-3.416	-3.560 to -3.272	Yes
7622	6:**1 X** vs. 47:**1 X**	-3.320	-3.464 to -3.176	Yes
7623	6:**1 X** vs. 47:**10 X**	-3.302	-3.446 to -3.158	Yes
7624	6:**1 X** vs. 47:**40 X**	-0.6617	-0.8056 to -0.5177	Yes
7625	6:**1 X** vs. 47:**50 X**	-0.4203	-0.5643 to -0.2764	Yes
7626	6:**1 X** vs. 48:**0 X**	-3.404	-3.564 to -3.243	Yes
7627	6:**1 X** vs. 48:**1 X**	-3.307	-3.451 to -3.163	Yes
7628	6:**1 X** vs. 48:**10 X**	-3.302	-3.446 to -3.158	Yes
7629	6:**1 X** vs. 48:**40 X**	-0.6680	-0.8120 to -0.5240	Yes
7630	6:**1 X** vs. 48:**50 X**	-0.4277	-0.5716 to -0.2837	Yes
7631	6:**1 X** vs. 49:**0 X**	-3.404	-3.548 to -3.260	Yes
7632	6:**1 X** vs. 49:**1 X**	-3.307	-3.451 to -3.163	Yes
7633	6:**1 X** vs. 49:**10 X**	-3.302	-3.446 to -3.158	Yes
7634	6:**1 X** vs. 49:**40 X**	-0.6723	-0.8163 to -0.5284	Yes
7635	6:**1 X** vs. 49:**50 X**	-0.4270	-0.5710 to -0.2830	Yes
7636	6:**1 X** vs. 50:**0 X**	-3.404	-3.548 to -3.260	Yes
7637	6:**1 X** vs. 50:**1 X**	-3.307	-3.451 to -3.163	Yes
7638	6:**1 X** vs. 50:**10 X**	-3.302	-3.446 to -3.158	Yes
7639	6:**1 X** vs. 50:**40 X**	-0.6800	-0.8240 to -0.5360	Yes
7640	6:**1 X** vs. 50:**50 X**	-0.4267	-0.5706 to -0.2827	Yes
7641	6:**10 X** vs. 6:**40 X**	0.01867	-0.1253 to 0.1626	No
7642	6:**10 X** vs. 6:**50 X**	0.02033	-0.1236 to 0.1643	No
7643	6:**10 X** vs. 7:**0 X**	0.01167	-0.1323 to 0.1556	No
7644	6:**10 X** vs. 7:**1 X**	0.02033	-0.1236 to 0.1643	No
7645	6:**10 X** vs. 7:**10 X**	0.008333	-0.1356 to 0.1523	No
7646	6:**10 X** vs. 7:**40 X**	0.0170	-0.1270 to 0.1610	No
7647	6:**10 X** vs. 7:**50 X**	0.0190	-0.1250 to 0.1630	No
7648	6:**10 X** vs. 8:**0 X**	0.007000	-0.1370 to 0.1510	No
7649	6:**10 X** vs. 8:**1 X**	0.02033	-0.1236 to 0.1643	No
7650	6:**10 X** vs. 8:**10 X**	0.0130	-0.1310 to 0.1570	No

2way ANOVA<br>Multiple comparisons	A<br>Data Set-A<br>Y	B<br>Data Set-B<br>Y	C<br>Data Set-C<br>Y
**7651** 6:**10 X** vs. 8:**40 X**	0.01233	-0.1316 to 0.1563	No
**7652** 6:**10 X** vs. 8:**50 X**	0.01533	-0.1286 to 0.1593	No
**7653** 6:**10 X** vs. 9:**0 X**	-0.001000	-0.1450 to 0.1430	No
**7654** 6:**10 X** vs. 9:**1 X**	-0.009000	-0.1530 to 0.1350	No
**7655** 6:**10 X** vs. 9:**10 X**	-0.0100	-0.1540 to 0.1340	No
**7656** 6:**10 X** vs. 9:**40 X**	0.007667	-0.1363 to 0.1516	No
**7657** 6:**10 X** vs. 9:**50 X**	0.01433	-0.1296 to 0.1583	No
**7658** 6:**10 X** vs. 10:**0 X**	-0.002667	-0.1466 to 0.1413	No
**7659** 6:**10 X** vs. 10:**1 X**	-0.009667	-0.1536 to 0.1343	No
**7660** 6:**10 X** vs. 10:**10 X**	-0.01233	-0.1563 to 0.1316	No
**7661** 6:**10 X** vs. 10:**40 X**	-0.002000	-0.1460 to 0.1420	No
**7662** 6:**10 X** vs. 10:**50 X**	0.01133	-0.1326 to 0.1553	No
**7663** 6:**10 X** vs. 11:**0 X**	-0.01233	-0.1563 to 0.1316	No
**7664** 6:**10 X** vs. 11:**1 X**	-0.0140	-0.1580 to 0.1300	No
**7665** 6:**10 X** vs. 11:**10 X**	-0.01433	-0.1583 to 0.1296	No
**7666** 6:**10 X** vs. 11:**40 X**	-0.0060	-0.1500 to 0.1380	No
**7667** 6:**10 X** vs. 11:**50 X**	0.006000	-0.1380 to 0.1500	No
**7668** 6:**10 X** vs. 12:**0 X**	-0.0130	-0.1570 to 0.1310	No
**7669** 6:**10 X** vs. 12:**1 X**	0.004667	-0.1393 to 0.1486	No
**7670** 6:**10 X** vs. 12:**10 X**	-0.0130	-0.1570 to 0.1310	No
**7671** 6:**10 X** vs. 12:**40 X**	0.0006667	-0.1433 to 0.1446	No
**7672** 6:**10 X** vs. 12:**50 X**	-0.002667	-0.1466 to 0.1413	No
**7673** 6:**10 X** vs. 13:**0 X**	-0.007000	-0.1510 to 0.1370	No
**7674** 6:**10 X** vs. 13:**1 X**	0.005667	-0.1383 to 0.1496	No
**7675** 6:**10 X** vs. 13:**10 X**	-0.01633	-0.1603 to 0.1276	No
**7676** 6:**10 X** vs. 13:**40 X**	-0.01267	-0.1566 to 0.1313	No
**7677** 6:**10 X** vs. 13:**50 X**	-0.01367	-0.1576 to 0.1303	No
**7678** 6:**10 X** vs. 14:**0 X**	-0.01567	-0.1596 to 0.1283	No
**7679** 6:**10 X** vs. 14:**1 X**	-0.007333	-0.1513 to 0.1366	No
**7680** 6:**10 X** vs. 14:**10 X**	-0.02133	-0.1653 to 0.1226	No
**7681** 6:**10 X** vs. 14:**40 X**	-0.0180	-0.1620 to 0.1260	No
**7682** 6:**10 X** vs. 14:**50 X**	-0.0220	-0.1660 to 0.1220	No
**7683** 6:**10 X** vs. 15:**0 X**	-0.0280	-0.1720 to 0.1160	No
**7684** 6:**10 X** vs. 15:**1 X**	-0.0080	-0.1520 to 0.1360	No
**7685** 6:**10 X** vs. 15:**10 X**	-0.03533	-0.1793 to 0.1086	No
**7686** 6:**10 X** vs. 15:**40 X**	-0.01767	-0.1616 to 0.1263	No
**7687** 6:**10 X** vs. 15:**50 X**	-0.03533	-0.1793 to 0.1086	No
**7688** 6:**10 X** vs. 16:**0 X**	-0.1203	-0.2643 to 0.02363	No
**7689** 6:**10 X** vs. 16:**1 X**	-0.01133	-0.1553 to 0.1326	No
**7690** 6:**10 X** vs. 16:**10 X**	-0.0370	-0.1810 to 0.1070	No
**7691** 6:**10 X** vs. 16:**40 X**	-0.05633	-0.2003 to 0.08763	No
**7692** 6:**10 X** vs. 16:**50 X**	-0.09867	-0.2426 to 0.04529	No
**7693** 6:**10 X** vs. 17:**0 X**	-0.1730	-0.3170 to -0.02904	Yes
**7694** 6:**10 X** vs. 17:**1 X**	-0.08733	-0.2313 to 0.05663	No
**7695** 6:**10 X** vs. 17:**10 X**	-0.1440	-0.2880 to -4.018e-005	Yes

	2way ANOVA<br>Multiple comparisons	A<br>Data Set-A<br>Y	B<br>Data Set-B<br>Y	C<br>Data Set-C<br>Y
7696	6:**10 X** vs. 17:**40 X**	-0.06733	-0.2113 to 0.07663	No
7697	6:**10 X** vs. 17:**50 X**	-0.1127	-0.2566 to 0.03129	No
7698	6:**10 X** vs. 18:**0 X**	-0.2217	-0.3656 to -0.07771	Yes
7699	6:**10 X** vs. 18:**1 X**	-0.1300	-0.2740 to 0.01396	No
7700	6:**10 X** vs. 18:**10 X**	-0.2223	-0.3663 to -0.07837	Yes
7701	6:**10 X** vs. 18:**40 X**	-0.07167	-0.2156 to 0.07229	No
7702	6:**10 X** vs. 18:**50 X**	-0.1167	-0.2606 to 0.02729	No
7703	6:**10 X** vs. 19:**0 X**	-0.2567	-0.4006 to -0.1127	Yes
7704	6:**10 X** vs. 19:**1 X**	-0.2447	-0.3886 to -0.1007	Yes
7705	6:**10 X** vs. 19:**10 X**	-0.2423	-0.3863 to -0.09837	Yes
7706	6:**10 X** vs. 19:**40 X**	-0.1003	-0.2443 to 0.04363	No
7707	6:**10 X** vs. 19:**50 X**	-0.1113	-0.2553 to 0.03263	No
7708	6:**10 X** vs. 20:**0 X**	-0.3363	-0.4803 to -0.1924	Yes
7709	6:**10 X** vs. 20:**1 X**	-0.1557	-0.2996 to -0.01171	Yes
7710	6:**10 X** vs. 20:**10 X**	-0.2447	-0.3886 to -0.1007	Yes
7711	6:**10 X** vs. 20:**40 X**	-0.1120	-0.2560 to 0.03196	No
7712	6:**10 X** vs. 20:**50 X**	-0.1560	-0.3000 to -0.01204	Yes
7713	6:**10 X** vs. 21:**0 X**	-0.3597	-0.5036 to -0.2157	Yes
7714	6:**10 X** vs. 21:**1 X**	-0.3127	-0.4566 to -0.1687	Yes
7715	6:**10 X** vs. 21:**10 X**	-0.3097	-0.4536 to -0.1657	Yes
7716	6:**10 X** vs. 21:**40 X**	-0.1850	-0.3290 to -0.04104	Yes
7717	6:**10 X** vs. 21:**50 X**	-0.2753	-0.4193 to -0.1314	Yes
7718	6:**10 X** vs. 22:**0 X**	-0.4013	-0.5453 to -0.2574	Yes
7719	6:**10 X** vs. 22:**1 X**	-0.3960	-0.5400 to -0.2520	Yes
7720	6:**10 X** vs. 22:**10 X**	-0.3913	-0.5353 to -0.2474	Yes
7721	6:**10 X** vs. 22:**40 X**	-0.2917	-0.4356 to -0.1477	Yes
7722	6:**10 X** vs. 22:**50 X**	-0.3543	-0.4983 to -0.2104	Yes
7723	6:**10 X** vs. 23:**0 X**	-0.4667	-0.6106 to -0.3227	Yes
7724	6:**10 X** vs. 23:**1 X**	-0.6563	-0.8173 to -0.4954	Yes
7725	6:**10 X** vs. 23:**10 X**	-0.4060	-0.5500 to -0.2620	Yes
7726	6:**10 X** vs. 23:**40 X**	-0.2863	-0.4473 to -0.1254	Yes
7727	6:**10 X** vs. 23:**50 X**	-0.3203	-0.4643 to -0.1764	Yes
7728	6:**10 X** vs. 24:**0 X**	-0.5840	-0.7280 to -0.4400	Yes
7729	6:**10 X** vs. 24:**1 X**	-0.7403	-0.9013 to -0.5794	Yes
7730	6:**10 X** vs. 24:**10 X**	-0.5678	-0.7288 to -0.4069	Yes
7731	6:**10 X** vs. 24:**40 X**	-0.3027	-0.4466 to -0.1587	Yes
7732	6:**10 X** vs. 24:**50 X**	-0.2243	-0.3683 to -0.08037	Yes
7733	6:**10 X** vs. 25:**0 X**	-0.9213	-1.082 to -0.7604	Yes
7734	6:**10 X** vs. 25:**1 X**	-1.111	-1.272 to -0.9499	Yes
7735	6:**10 X** vs. 25:**10 X**	-0.4723	-0.6759 to -0.2687	Yes
7736	6:**10 X** vs. 25:**40 X**	-0.3047	-0.4486 to -0.1607	Yes
7737	6:**10 X** vs. 25:**50 X**	-0.2790	-0.4230 to -0.1350	Yes
7738	6:**10 X** vs. 26:**0 X**	-1.134	-1.295 to -0.9729	Yes
7739	6:**10 X** vs. 26:**1 X**	-1.247	-1.408 to -1.086	Yes
7740	6:**10 X** vs. 26:**10 X**	-0.9333	-1.094 to -0.7724	Yes

	2way ANOVA<br>Multiple comparisons	A<br>Data Set-A<br>Y	B<br>Data Set-B<br>Y	C<br>Data Set-C<br>Y
7741	6:**10 X** vs. 26:**40 X**	-0.3790	-0.5230 to -0.2350	Yes
7742	6:**10 X** vs. 26:**50 X**	-0.2393	-0.3833 to -0.09537	Yes
7743	6:**10 X** vs. 27:**0 X**	-1.322	-1.466 to -1.178	Yes
7744	6:**10 X** vs. 27:**1 X**	-1.412	-1.573 to -1.251	Yes
7745	6:**10 X** vs. 27:**10 X**	-1.224	-1.385 to -1.063	Yes
7746	6:**10 X** vs. 27:**40 X**	-0.3790	-0.5230 to -0.2350	Yes
7747	6:**10 X** vs. 27:**50 X**	-0.2387	-0.3826 to -0.09471	Yes
7748	6:**10 X** vs. 28:**0 X**	-1.271	-1.432 to -1.110	Yes
7749	6:**10 X** vs. 28:**1 X**	-1.312	-1.456 to -1.168	Yes
7750	6:**10 X** vs. 28:**10 X**	-1.113	-1.274 to -0.9524	Yes
7751	6:**10 X** vs. 28:**40 X**	-0.3913	-0.5353 to -0.2474	Yes
7752	6:**10 X** vs. 28:**50 X**	-0.2867	-0.4306 to -0.1427	Yes
7753	6:**10 X** vs. 29:**0 X**	-1.752	-1.913 to -1.591	Yes
7754	6:**10 X** vs. 29:**1 X**	-1.837	-1.998 to -1.676	Yes
7755	6:**10 X** vs. 29:**10 X**	-1.052	-1.213 to -0.8909	Yes
7756	6:**10 X** vs. 29:**40 X**	-0.4993	-0.6433 to -0.3554	Yes
7757	6:**10 X** vs. 29:**50 X**	-0.2880	-0.4320 to -0.1440	Yes
7758	6:**10 X** vs. 30:**0 X**	-1.467	-1.628 to -1.306	Yes
7759	6:**10 X** vs. 30:**1 X**	-1.788	-1.949 to -1.627	Yes
7760	6:**10 X** vs. 30:**10 X**	-1.126	-1.287 to -0.9654	Yes
7761	6:**10 X** vs. 30:**40 X**	-0.4263	-0.5703 to -0.2824	Yes
7762	6:**10 X** vs. 30:**50 X**	-0.3403	-0.4843 to -0.1964	Yes
7763	6:**10 X** vs. 31:**0 X**	-1.785	-1.946 to -1.624	Yes
7764	6:**10 X** vs. 31:**1 X**	-1.775	-1.936 to -1.614	Yes
7765	6:**10 X** vs. 31:**10 X**	-1.258	-1.419 to -1.097	Yes
7766	6:**10 X** vs. 31:**40 X**	-0.5180	-0.6620 to -0.3740	Yes
7767	6:**10 X** vs. 31:**50 X**	-0.3193	-0.4633 to -0.1754	Yes
7768	6:**10 X** vs. 32:**0 X**	-2.376	-2.520 to -2.232	Yes
7769	6:**10 X** vs. 32:**1 X**	-1.832	-1.993 to -1.671	Yes
7770	6:**10 X** vs. 32:**10 X**	-1.688	-1.832 to -1.544	Yes
7771	6:**10 X** vs. 32:**40 X**	-0.5143	-0.6583 to -0.3704	Yes
7772	6:**10 X** vs. 32:**50 X**	-0.3190	-0.4630 to -0.1750	Yes
7773	6:**10 X** vs. 33:**0 X**	-2.380	-2.524 to -2.236	Yes
7774	6:**10 X** vs. 33:**1 X**	-2.195	-2.339 to -2.051	Yes
7775	6:**10 X** vs. 33:**10 X**	-1.855	-1.999 to -1.711	Yes
7776	6:**10 X** vs. 33:**40 X**	-0.5330	-0.6770 to -0.3890	Yes
7777	6:**10 X** vs. 33:**50 X**	-0.3340	-0.4780 to -0.1900	Yes
7778	6:**10 X** vs. 34:**0 X**	-2.305	-2.449 to -2.161	Yes
7779	6:**10 X** vs. 34:**1 X**	-2.312	-2.456 to -2.168	Yes
7780	6:**10 X** vs. 34:**10 X**	-1.945	-2.089 to -1.801	Yes
7781	6:**10 X** vs. 34:**40 X**	-0.5450	-0.6890 to -0.4010	Yes
7782	6:**10 X** vs. 34:**50 X**	-0.4830	-0.6270 to -0.3390	Yes
7783	6:**10 X** vs. 35:**0 X**	-2.653	-2.797 to -2.509	Yes
7784	6:**10 X** vs. 35:**1 X**	-2.419	-2.563 to -2.275	Yes
7785	6:**10 X** vs. 35:**10 X**	-1.907	-2.051 to -1.763	Yes

	2way ANOVA<br>Multiple comparisons	A<br>Data Set-A<br>Y	B<br>Data Set-B<br>Y	C<br>Data Set-C<br>Y
**7786**	6:**10 X** vs. 35:**40 X**	-0.5540	-0.6980 to -0.4100	Yes
**7787**	6:**10 X** vs. 35:**50 X**	-0.4957	-0.6396 to -0.3517	Yes
**7788**	6:**10 X** vs. 36:**0 X**	-2.860	-3.021 to -2.699	Yes
**7789**	6:**10 X** vs. 36:**1 X**	-2.556	-2.700 to -2.412	Yes
**7790**	6:**10 X** vs. 36:**10 X**	-1.913	-2.057 to -1.769	Yes
**7791**	6:**10 X** vs. 36:**40 X**	-0.6093	-0.7533 to -0.4654	Yes
**7792**	6:**10 X** vs. 36:**50 X**	-0.4590	-0.6030 to -0.3150	Yes
**7793**	6:**10 X** vs. 37:**0 X**	-3.244	-3.388 to -3.100	Yes
**7794**	6:**10 X** vs. 37:**1 X**	-2.626	-2.770 to -2.482	Yes
**7795**	6:**10 X** vs. 37:**10 X**	-1.914	-2.058 to -1.770	Yes
**7796**	6:**10 X** vs. 37:**40 X**	-0.6467	-0.7906 to -0.5027	Yes
**7797**	6:**10 X** vs. 37:**50 X**	-0.4677	-0.6116 to -0.3237	Yes
**7798**	6:**10 X** vs. 38:**0 X**	-3.189	-3.333 to -3.045	Yes
**7799**	6:**10 X** vs. 38:**1 X**	-2.744	-2.888 to -2.600	Yes
**7800**	6:**10 X** vs. 38:**10 X**	-2.190	-2.334 to -2.046	Yes
**7801**	6:**10 X** vs. 38:**40 X**	-0.6510	-0.7950 to -0.5070	Yes
**7802**	6:**10 X** vs. 38:**50 X**	-0.4717	-0.6156 to -0.3277	Yes
**7803**	6:**10 X** vs. 39:**0 X**	-3.419	-3.563 to -3.275	Yes
**7804**	6:**10 X** vs. 39:**1 X**	-3.311	-3.455 to -3.167	Yes
**7805**	6:**10 X** vs. 39:**10 X**	-2.335	-2.479 to -2.191	Yes
**7806**	6:**10 X** vs. 39:**40 X**	-0.6570	-0.8010 to -0.5130	Yes
**7807**	6:**10 X** vs. 39:**50 X**	-0.5027	-0.6466 to -0.3587	Yes
**7808**	6:**10 X** vs. 40:**0 X**	-3.460	-3.604 to -3.316	Yes
**7809**	6:**10 X** vs. 40:**1 X**	-3.386	-3.530 to -3.242	Yes
**7810**	6:**10 X** vs. 40:**10 X**	-2.591	-2.735 to -2.447	Yes
**7811**	6:**10 X** vs. 40:**40 X**	-0.6617	-0.8056 to -0.5177	Yes
**7812**	6:**10 X** vs. 40:**50 X**	-0.5090	-0.6530 to -0.3650	Yes
**7813**	6:**10 X** vs. 41:**0 X**	-3.501	-3.645 to -3.357	Yes
**7814**	6:**10 X** vs. 41:**1 X**	-3.500	-3.644 to -3.356	Yes
**7815**	6:**10 X** vs. 41:**10 X**	-2.645	-2.789 to -2.501	Yes
**7816**	6:**10 X** vs. 41:**40 X**	-0.6090	-0.7530 to -0.4650	Yes
**7817**	6:**10 X** vs. 41:**50 X**	-0.5217	-0.6656 to -0.3777	Yes
**7818**	6:**10 X** vs. 42:**0 X**	-3.643	-3.787 to -3.499	Yes
**7819**	6:**10 X** vs. 42:**1 X**	-3.663	-3.807 to -3.519	Yes
**7820**	6:**10 X** vs. 42:**10 X**	-2.645	-2.789 to -2.501	Yes
**7821**	6:**10 X** vs. 42:**40 X**	-0.6217	-0.7656 to -0.4777	Yes
**7822**	6:**10 X** vs. 42:**50 X**	-0.5250	-0.6690 to -0.3810	Yes
**7823**	6:**10 X** vs. 43:**0 X**	-3.733	-3.877 to -3.589	Yes
**7824**	6:**10 X** vs. 43:**1 X**	-3.647	-3.791 to -3.503	Yes
**7825**	6:**10 X** vs. 43:**10 X**	-3.363	-3.507 to -3.219	Yes
**7826**	6:**10 X** vs. 43:**40 X**	-0.6200	-0.7640 to -0.4760	Yes
**7827**	6:**10 X** vs. 43:**50 X**	-0.5253	-0.6693 to -0.3814	Yes
**7828**	6:**10 X** vs. 44:**0 X**	-3.757	-3.901 to -3.613	Yes
**7829**	6:**10 X** vs. 44:**1 X**	-3.647	-3.791 to -3.503	Yes
**7830**	6:**10 X** vs. 44:**10 X**	-3.426	-3.570 to -3.282	Yes

	**2way ANOVA** Multiple comparisons	**A** Data Set-A **Y**	**B** Data Set-B **Y**	**C** Data Set-C **Y**
**7831**	6:**10 X** vs. 44:**40 X**	-0.6220	-0.7660 to -0.4780	Yes
**7832**	6:**10 X** vs. 44:**50 X**	-0.4577	-0.6016 to -0.3137	Yes
**7833**	6:**10 X** vs. 45:**0 X**	-3.622	-3.766 to -3.478	Yes
**7834**	6:**10 X** vs. 45:**1 X**	-3.424	-3.568 to -3.280	Yes
**7835**	6:**10 X** vs. 45:**10 X**	-3.306	-3.450 to -3.162	Yes
**7836**	6:**10 X** vs. 45:**40 X**	-0.6233	-0.7673 to -0.4794	Yes
**7837**	6:**10 X** vs. 45:**50 X**	-0.4547	-0.5986 to -0.3107	Yes
**7838**	6:**10 X** vs. 46:**0 X**	-3.426	-3.570 to -3.282	Yes
**7839**	6:**10 X** vs. 46:**1 X**	-3.393	-3.537 to -3.249	Yes
**7840**	6:**10 X** vs. 46:**10 X**	-3.301	-3.445 to -3.157	Yes
**7841**	6:**10 X** vs. 46:**40 X**	-0.6370	-0.7810 to -0.4930	Yes
**7842**	6:**10 X** vs. 46:**50 X**	-0.4477	-0.5916 to -0.3037	Yes
**7843**	6:**10 X** vs. 47:**0 X**	-3.402	-3.546 to -3.258	Yes
**7844**	6:**10 X** vs. 47:**1 X**	-3.306	-3.450 to -3.162	Yes
**7845**	6:**10 X** vs. 47:**10 X**	-3.287	-3.431 to -3.143	Yes
**7846**	6:**10 X** vs. 47:**40 X**	-0.6470	-0.7910 to -0.5030	Yes
**7847**	6:**10 X** vs. 47:**50 X**	-0.4057	-0.5496 to -0.2617	Yes
**7848**	6:**10 X** vs. 48:**0 X**	-3.389	-3.550 to -3.228	Yes
**7849**	6:**10 X** vs. 48:**1 X**	-3.293	-3.437 to -3.149	Yes
**7850**	6:**10 X** vs. 48:**10 X**	-3.287	-3.431 to -3.143	Yes
**7851**	6:**10 X** vs. 48:**40 X**	-0.6533	-0.7973 to -0.5094	Yes
**7852**	6:**10 X** vs. 48:**50 X**	-0.4130	-0.5570 to -0.2690	Yes
**7853**	6:**10 X** vs. 49:**0 X**	-3.389	-3.533 to -3.245	Yes
**7854**	6:**10 X** vs. 49:**1 X**	-3.293	-3.437 to -3.149	Yes
**7855**	6:**10 X** vs. 49:**10 X**	-3.287	-3.431 to -3.143	Yes
**7856**	6:**10 X** vs. 49:**40 X**	-0.6577	-0.8016 to -0.5137	Yes
**7857**	6:**10 X** vs. 49:**50 X**	-0.4123	-0.5563 to -0.2684	Yes
**7858**	6:**10 X** vs. 50:**0 X**	-3.389	-3.533 to -3.245	Yes
**7859**	6:**10 X** vs. 50:**1 X**	-3.293	-3.437 to -3.149	Yes
**7860**	6:**10 X** vs. 50:**10 X**	-3.287	-3.431 to -3.143	Yes
**7861**	6:**10 X** vs. 50:**40 X**	-0.6653	-0.8093 to -0.5214	Yes
**7862**	6:**10 X** vs. 50:**50 X**	-0.4120	-0.5560 to -0.2680	Yes
**7863**	6:**40 X** vs. 6:**50 X**	0.001667	-0.1423 to 0.1456	No
**7864**	6:**40 X** vs. 7:**0 X**	-0.007000	-0.1510 to 0.1370	No
**7865**	6:**40 X** vs. 7:**1 X**	0.001667	-0.1423 to 0.1456	No
**7866**	6:**40 X** vs. 7:**10 X**	-0.01033	-0.1543 to 0.1336	No
**7867**	6:**40 X** vs. 7:**40 X**	-0.001667	-0.1456 to 0.1423	No
**7868**	6:**40 X** vs. 7:**50 X**	0.0003333	-0.1436 to 0.1443	No
**7869**	6:**40 X** vs. 8:**0 X**	-0.01167	-0.1556 to 0.1323	No
**7870**	6:**40 X** vs. 8:**1 X**	0.001667	-0.1423 to 0.1456	No
**7871**	6:**40 X** vs. 8:**10 X**	-0.005667	-0.1496 to 0.1383	No
**7872**	6:**40 X** vs. 8:**40 X**	-0.006333	-0.1503 to 0.1376	No
**7873**	6:**40 X** vs. 8:**50 X**	-0.003333	-0.1473 to 0.1406	No
**7874**	6:**40 X** vs. 9:**0 X**	-0.01967	-0.1636 to 0.1243	No
**7875**	6:**40 X** vs. 9:**1 X**	-0.02767	-0.1716 to 0.1163	No

	2way ANOVA<br>Multiple comparisons	A<br>Data Set-A<br>Y	B<br>Data Set-B<br>Y	C<br>Data Set-C<br>Y
7876	6:40 X vs. 9:10 X	-0.02867	-0.1726 to 0.1153	No
7877	6:40 X vs. 9:40 X	-0.0110	-0.1550 to 0.1330	No
7878	6:40 X vs. 9:50 X	-0.004333	-0.1483 to 0.1396	No
7879	6:40 X vs. 10:0 X	-0.02133	-0.1653 to 0.1226	No
7880	6:40 X vs. 10:1 X	-0.02833	-0.1723 to 0.1156	No
7881	6:40 X vs. 10:10 X	-0.0310	-0.1750 to 0.1130	No
7882	6:40 X vs. 10:40 X	-0.02067	-0.1646 to 0.1233	No
7883	6:40 X vs. 10:50 X	-0.007333	-0.1513 to 0.1366	No
7884	6:40 X vs. 11:0 X	-0.0310	-0.1750 to 0.1130	No
7885	6:40 X vs. 11:1 X	-0.03267	-0.1766 to 0.1113	No
7886	6:40 X vs. 11:10 X	-0.0330	-0.1770 to 0.1110	No
7887	6:40 X vs. 11:40 X	-0.02467	-0.1686 to 0.1193	No
7888	6:40 X vs. 11:50 X	-0.01267	-0.1566 to 0.1313	No
7889	6:40 X vs. 12:0 X	-0.03167	-0.1756 to 0.1123	No
7890	6:40 X vs. 12:1 X	-0.0140	-0.1580 to 0.1300	No
7891	6:40 X vs. 12:10 X	-0.03167	-0.1756 to 0.1123	No
7892	6:40 X vs. 12:40 X	-0.0180	-0.1620 to 0.1260	No
7893	6:40 X vs. 12:50 X	-0.02133	-0.1653 to 0.1226	No
7894	6:40 X vs. 13:0 X	-0.02567	-0.1696 to 0.1183	No
7895	6:40 X vs. 13:1 X	-0.0130	-0.1570 to 0.1310	No
7896	6:40 X vs. 13:10 X	-0.0350	-0.1790 to 0.1090	No
7897	6:40 X vs. 13:40 X	-0.03133	-0.1753 to 0.1126	No
7898	6:40 X vs. 13:50 X	-0.03233	-0.1763 to 0.1116	No
7899	6:40 X vs. 14:0 X	-0.03433	-0.1783 to 0.1096	No
7900	6:40 X vs. 14:1 X	-0.0260	-0.1700 to 0.1180	No
7901	6:40 X vs. 14:10 X	-0.0400	-0.1840 to 0.1040	No
7902	6:40 X vs. 14:40 X	-0.03667	-0.1806 to 0.1073	No
7903	6:40 X vs. 14:50 X	-0.04067	-0.1846 to 0.1033	No
7904	6:40 X vs. 15:0 X	-0.04667	-0.1906 to 0.09729	No
7905	6:40 X vs. 15:1 X	-0.02667	-0.1706 to 0.1173	No
7906	6:40 X vs. 15:10 X	-0.0540	-0.1980 to 0.08996	No
7907	6:40 X vs. 15:40 X	-0.03633	-0.1803 to 0.1076	No
7908	6:40 X vs. 15:50 X	-0.0540	-0.1980 to 0.08996	No
7909	6:40 X vs. 16:0 X	-0.1390	-0.2830 to 0.004960	No
7910	6:40 X vs. 16:1 X	-0.0300	-0.1740 to 0.1140	No
7911	6:40 X vs. 16:10 X	-0.05567	-0.1996 to 0.08829	No
7912	6:40 X vs. 16:40 X	-0.0750	-0.2190 to 0.06896	No
7913	6:40 X vs. 16:50 X	-0.1173	-0.2613 to 0.02663	No
7914	6:40 X vs. 17:0 X	-0.1917	-0.3356 to -0.04771	Yes
7915	6:40 X vs. 17:1 X	-0.1060	-0.2500 to 0.03796	No
7916	6:40 X vs. 17:10 X	-0.1627	-0.3066 to -0.01871	Yes
7917	6:40 X vs. 17:40 X	-0.0860	-0.2300 to 0.05796	No
7918	6:40 X vs. 17:50 X	-0.1313	-0.2753 to 0.01263	No
7919	6:40 X vs. 18:0 X	-0.2403	-0.3843 to -0.09637	Yes
7920	6:40 X vs. 18:1 X	-0.1487	-0.2926 to -0.004707	Yes

	2way ANOVA<br>Multiple comparisons	A<br>Data Set-A<br>Y	B<br>Data Set-B<br>Y	C<br>Data Set-C<br>Y
7921	6:40 X vs. 18:10 X	-0.2410	-0.3850 to -0.09704	Yes
7922	6:40 X vs. 18:40 X	-0.09033	-0.2343 to 0.05363	No
7923	6:40 X vs. 18:50 X	-0.1353	-0.2793 to 0.008626	No
7924	6:40 X vs. 19:0 X	-0.2753	-0.4193 to -0.1314	Yes
7925	6:40 X vs. 19:1 X	-0.2633	-0.4073 to -0.1194	Yes
7926	6:40 X vs. 19:10 X	-0.2610	-0.4050 to -0.1170	Yes
7927	6:40 X vs. 19:40 X	-0.1190	-0.2630 to 0.02496	No
7928	6:40 X vs. 19:50 X	-0.1300	-0.2740 to 0.01396	No
7929	6:40 X vs. 20:0 X	-0.3550	-0.4990 to -0.2110	Yes
7930	6:40 X vs. 20:1 X	-0.1743	-0.3183 to -0.03037	Yes
7931	6:40 X vs. 20:10 X	-0.2633	-0.4073 to -0.1194	Yes
7932	6:40 X vs. 20:40 X	-0.1307	-0.2746 to 0.01329	No
7933	6:40 X vs. 20:50 X	-0.1747	-0.3186 to -0.03071	Yes
7934	6:40 X vs. 21:0 X	-0.3783	-0.5223 to -0.2344	Yes
7935	6:40 X vs. 21:1 X	-0.3313	-0.4753 to -0.1874	Yes
7936	6:40 X vs. 21:10 X	-0.3283	-0.4723 to -0.1844	Yes
7937	6:40 X vs. 21:40 X	-0.2037	-0.3476 to -0.05971	Yes
7938	6:40 X vs. 21:50 X	-0.2940	-0.4380 to -0.1500	Yes
7939	6:40 X vs. 22:0 X	-0.4200	-0.5640 to -0.2760	Yes
7940	6:40 X vs. 22:1 X	-0.4147	-0.5586 to -0.2707	Yes
7941	6:40 X vs. 22:10 X	-0.4100	-0.5540 to -0.2660	Yes
7942	6:40 X vs. 22:40 X	-0.3103	-0.4543 to -0.1664	Yes
7943	6:40 X vs. 22:50 X	-0.3730	-0.5170 to -0.2290	Yes
7944	6:40 X vs. 23:0 X	-0.4853	-0.6293 to -0.3414	Yes
7945	6:40 X vs. 23:1 X	-0.6750	-0.8360 to -0.5140	Yes
7946	6:40 X vs. 23:10 X	-0.4247	-0.5686 to -0.2807	Yes
7947	6:40 X vs. 23:40 X	-0.3050	-0.4660 to -0.1440	Yes
7948	6:40 X vs. 23:50 X	-0.3390	-0.4830 to -0.1950	Yes
7949	6:40 X vs. 24:0 X	-0.6027	-0.7466 to -0.4587	Yes
7950	6:40 X vs. 24:1 X	-0.7590	-0.9200 to -0.5980	Yes
7951	6:40 X vs. 24:10 X	-0.5865	-0.7475 to -0.4255	Yes
7952	6:40 X vs. 24:40 X	-0.3213	-0.4653 to -0.1774	Yes
7953	6:40 X vs. 24:50 X	-0.2430	-0.3870 to -0.09904	Yes
7954	6:40 X vs. 25:0 X	-0.9400	-1.101 to -0.7790	Yes
7955	6:40 X vs. 25:1 X	-1.130	-1.290 to -0.9685	Yes
7956	6:40 X vs. 25:10 X	-0.4910	-0.6946 to -0.2874	Yes
7957	6:40 X vs. 25:40 X	-0.3233	-0.4673 to -0.1794	Yes
7958	6:40 X vs. 25:50 X	-0.2977	-0.4416 to -0.1537	Yes
7959	6:40 X vs. 26:0 X	-1.153	-1.313 to -0.9915	Yes
7960	6:40 X vs. 26:1 X	-1.266	-1.426 to -1.105	Yes
7961	6:40 X vs. 26:10 X	-0.9520	-1.113 to -0.7910	Yes
7962	6:40 X vs. 26:40 X	-0.3977	-0.5416 to -0.2537	Yes
7963	6:40 X vs. 26:50 X	-0.2580	-0.4020 to -0.1140	Yes
7964	6:40 X vs. 27:0 X	-1.340	-1.484 to -1.196	Yes
7965	6:40 X vs. 27:1 X	-1.431	-1.592 to -1.270	Yes

	2way ANOVA<br>Multiple comparisons	A<br>Data Set-A<br>Y	B<br>Data Set-B<br>Y	C<br>Data Set-C<br>Y
7966	6:40 X vs. 27:10 X	-1.243	-1.404 to -1.082	Yes
7967	6:40 X vs. 27:40 X	-0.3977	-0.5416 to -0.2537	Yes
7968	6:40 X vs. 27:50 X	-0.2573	-0.4013 to -0.1134	Yes
7969	6:40 X vs. 28:0 X	-1.290	-1.450 to -1.129	Yes
7970	6:40 X vs. 28:1 X	-1.330	-1.474 to -1.186	Yes
7971	6:40 X vs. 28:10 X	-1.132	-1.293 to -0.9710	Yes
7972	6:40 X vs. 28:40 X	-0.4100	-0.5540 to -0.2660	Yes
7973	6:40 X vs. 28:50 X	-0.3053	-0.4493 to -0.1614	Yes
7974	6:40 X vs. 29:0 X	-1.771	-1.932 to -1.610	Yes
7975	6:40 X vs. 29:1 X	-1.856	-2.017 to -1.695	Yes
7976	6:40 X vs. 29:10 X	-1.071	-1.231 to -0.9095	Yes
7977	6:40 X vs. 29:40 X	-0.5180	-0.6620 to -0.3740	Yes
7978	6:40 X vs. 29:50 X	-0.3067	-0.4506 to -0.1627	Yes
7979	6:40 X vs. 30:0 X	-1.486	-1.646 to -1.325	Yes
7980	6:40 X vs. 30:1 X	-1.807	-1.968 to -1.646	Yes
7981	6:40 X vs. 30:10 X	-1.145	-1.306 to -0.9840	Yes
7982	6:40 X vs. 30:40 X	-0.4450	-0.5890 to -0.3010	Yes
7983	6:40 X vs. 30:50 X	-0.3590	-0.5030 to -0.2150	Yes
7984	6:40 X vs. 31:0 X	-1.804	-1.964 to -1.643	Yes
7985	6:40 X vs. 31:1 X	-1.794	-1.955 to -1.633	Yes
7986	6:40 X vs. 31:10 X	-1.277	-1.437 to -1.116	Yes
7987	6:40 X vs. 31:40 X	-0.5367	-0.6806 to -0.3927	Yes
7988	6:40 X vs. 31:50 X	-0.3380	-0.4820 to -0.1940	Yes
7989	6:40 X vs. 32:0 X	-2.395	-2.539 to -2.251	Yes
7990	6:40 X vs. 32:1 X	-1.851	-2.012 to -1.690	Yes
7991	6:40 X vs. 32:10 X	-1.707	-1.851 to -1.563	Yes
7992	6:40 X vs. 32:40 X	-0.5330	-0.6770 to -0.3890	Yes
7993	6:40 X vs. 32:50 X	-0.3377	-0.4816 to -0.1937	Yes
7994	6:40 X vs. 33:0 X	-2.399	-2.543 to -2.255	Yes
7995	6:40 X vs. 33:1 X	-2.213	-2.357 to -2.069	Yes
7996	6:40 X vs. 33:10 X	-1.874	-2.018 to -1.730	Yes
7997	6:40 X vs. 33:40 X	-0.5517	-0.6956 to -0.4077	Yes
7998	6:40 X vs. 33:50 X	-0.3527	-0.4966 to -0.2087	Yes
7999	6:40 X vs. 34:0 X	-2.323	-2.467 to -2.179	Yes
8000	6:40 X vs. 34:1 X	-2.331	-2.475 to -2.187	Yes
8001	6:40 X vs. 34:10 X	-1.964	-2.108 to -1.820	Yes
8002	6:40 X vs. 34:40 X	-0.5637	-0.7076 to -0.4197	Yes
8003	6:40 X vs. 34:50 X	-0.5017	-0.6456 to -0.3577	Yes
8004	6:40 X vs. 35:0 X	-2.672	-2.816 to -2.528	Yes
8005	6:40 X vs. 35:1 X	-2.438	-2.582 to -2.294	Yes
8006	6:40 X vs. 35:10 X	-1.926	-2.070 to -1.782	Yes
8007	6:40 X vs. 35:40 X	-0.5727	-0.7166 to -0.4287	Yes
8008	6:40 X vs. 35:50 X	-0.5143	-0.6583 to -0.3704	Yes
8009	6:40 X vs. 36:0 X	-2.879	-3.039 to -2.718	Yes
8010	6:40 X vs. 36:1 X	-2.574	-2.718 to -2.430	Yes

	2way ANOVA<br>Multiple comparisons	A<br>Data Set-A<br>Y	B<br>Data Set-B<br>Y	C<br>Data Set-C<br>Y
**8011**	6:**40 X** vs. 36:**10 X**	-1.931	-2.075 to -1.787	Yes
**8012**	6:**40 X** vs. 36:**40 X**	-0.6280	-0.7720 to -0.4840	Yes
**8013**	6:**40 X** vs. 36:**50 X**	-0.4777	-0.6216 to -0.3337	Yes
**8014**	6:**40 X** vs. 37:**0 X**	-3.262	-3.406 to -3.118	Yes
**8015**	6:**40 X** vs. 37:**1 X**	-2.644	-2.788 to -2.500	Yes
**8016**	6:**40 X** vs. 37:**10 X**	-1.933	-2.077 to -1.789	Yes
**8017**	6:**40 X** vs. 37:**40 X**	-0.6653	-0.8093 to -0.5214	Yes
**8018**	6:**40 X** vs. 37:**50 X**	-0.4863	-0.6303 to -0.3424	Yes
**8019**	6:**40 X** vs. 38:**0 X**	-3.207	-3.351 to -3.063	Yes
**8020**	6:**40 X** vs. 38:**1 X**	-2.763	-2.907 to -2.619	Yes
**8021**	6:**40 X** vs. 38:**10 X**	-2.208	-2.352 to -2.064	Yes
**8022**	6:**40 X** vs. 38:**40 X**	-0.6697	-0.8136 to -0.5257	Yes
**8023**	6:**40 X** vs. 38:**50 X**	-0.4903	-0.6343 to -0.3464	Yes
**8024**	6:**40 X** vs. 39:**0 X**	-3.438	-3.582 to -3.294	Yes
**8025**	6:**40 X** vs. 39:**1 X**	-3.330	-3.474 to -3.186	Yes
**8026**	6:**40 X** vs. 39:**10 X**	-2.353	-2.497 to -2.209	Yes
**8027**	6:**40 X** vs. 39:**40 X**	-0.6757	-0.8196 to -0.5317	Yes
**8028**	6:**40 X** vs. 39:**50 X**	-0.5213	-0.6653 to -0.3774	Yes
**8029**	6:**40 X** vs. 40:**0 X**	-3.479	-3.623 to -3.335	Yes
**8030**	6:**40 X** vs. 40:**1 X**	-3.405	-3.549 to -3.261	Yes
**8031**	6:**40 X** vs. 40:**10 X**	-2.610	-2.754 to -2.466	Yes
**8032**	6:**40 X** vs. 40:**40 X**	-0.6803	-0.8243 to -0.5364	Yes
**8033**	6:**40 X** vs. 40:**50 X**	-0.5277	-0.6716 to -0.3837	Yes
**8034**	6:**40 X** vs. 41:**0 X**	-3.520	-3.664 to -3.376	Yes
**8035**	6:**40 X** vs. 41:**1 X**	-3.519	-3.663 to -3.375	Yes
**8036**	6:**40 X** vs. 41:**10 X**	-2.663	-2.807 to -2.519	Yes
**8037**	6:**40 X** vs. 41:**40 X**	-0.6277	-0.7716 to -0.4837	Yes
**8038**	6:**40 X** vs. 41:**50 X**	-0.5403	-0.6843 to -0.3964	Yes
**8039**	6:**40 X** vs. 42:**0 X**	-3.662	-3.806 to -3.518	Yes
**8040**	6:**40 X** vs. 42:**1 X**	-3.681	-3.825 to -3.537	Yes
**8041**	6:**40 X** vs. 42:**10 X**	-2.664	-2.808 to -2.520	Yes
**8042**	6:**40 X** vs. 42:**40 X**	-0.6403	-0.7843 to -0.4964	Yes
**8043**	6:**40 X** vs. 42:**50 X**	-0.5437	-0.6876 to -0.3997	Yes
**8044**	6:**40 X** vs. 43:**0 X**	-3.752	-3.896 to -3.608	Yes
**8045**	6:**40 X** vs. 43:**1 X**	-3.666	-3.810 to -3.522	Yes
**8046**	6:**40 X** vs. 43:**10 X**	-3.382	-3.526 to -3.238	Yes
**8047**	6:**40 X** vs. 43:**40 X**	-0.6387	-0.7826 to -0.4947	Yes
**8048**	6:**40 X** vs. 43:**50 X**	-0.5440	-0.6880 to -0.4000	Yes
**8049**	6:**40 X** vs. 44:**0 X**	-3.776	-3.920 to -3.632	Yes
**8050**	6:**40 X** vs. 44:**1 X**	-3.665	-3.809 to -3.521	Yes
**8051**	6:**40 X** vs. 44:**10 X**	-3.445	-3.589 to -3.301	Yes
**8052**	6:**40 X** vs. 44:**40 X**	-0.6407	-0.7846 to -0.4967	Yes
**8053**	6:**40 X** vs. 44:**50 X**	-0.4763	-0.6203 to -0.3324	Yes
**8054**	6:**40 X** vs. 45:**0 X**	-3.640	-3.784 to -3.496	Yes
**8055**	6:**40 X** vs. 45:**1 X**	-3.442	-3.586 to -3.298	Yes

	2way ANOVA<br>Multiple comparisons	A<br>Data Set-A<br>Y	B<br>Data Set-B<br>Y	C<br>Data Set-C<br>Y
**8056**	6:**40 X** vs. 45:**10 X**	-3.325	-3.469 to -3.181	Yes
**8057**	6:**40 X** vs. 45:**40 X**	-0.6420	-0.7860 to -0.4980	Yes
**8058**	6:**40 X** vs. 45:**50 X**	-0.4733	-0.6173 to -0.3294	Yes
**8059**	6:**40 X** vs. 46:**0 X**	-3.445	-3.589 to -3.301	Yes
**8060**	6:**40 X** vs. 46:**1 X**	-3.412	-3.556 to -3.268	Yes
**8061**	6:**40 X** vs. 46:**10 X**	-3.320	-3.464 to -3.176	Yes
**8062**	6:**40 X** vs. 46:**40 X**	-0.6557	-0.7996 to -0.5117	Yes
**8063**	6:**40 X** vs. 46:**50 X**	-0.4663	-0.6103 to -0.3224	Yes
**8064**	6:**40 X** vs. 47:**0 X**	-3.420	-3.564 to -3.276	Yes
**8065**	6:**40 X** vs. 47:**1 X**	-3.324	-3.468 to -3.180	Yes
**8066**	6:**40 X** vs. 47:**10 X**	-3.306	-3.450 to -3.162	Yes
**8067**	6:**40 X** vs. 47:**40 X**	-0.6657	-0.8096 to -0.5217	Yes
**8068**	6:**40 X** vs. 47:**50 X**	-0.4243	-0.5683 to -0.2804	Yes
**8069**	6:**40 X** vs. 48:**0 X**	-3.408	-3.568 to -3.247	Yes
**8070**	6:**40 X** vs. 48:**1 X**	-3.311	-3.455 to -3.167	Yes
**8071**	6:**40 X** vs. 48:**10 X**	-3.306	-3.450 to -3.162	Yes
**8072**	6:**40 X** vs. 48:**40 X**	-0.6720	-0.8160 to -0.5280	Yes
**8073**	6:**40 X** vs. 48:**50 X**	-0.4317	-0.5756 to -0.2877	Yes
**8074**	6:**40 X** vs. 49:**0 X**	-3.408	-3.552 to -3.264	Yes
**8075**	6:**40 X** vs. 49:**1 X**	-3.311	-3.455 to -3.167	Yes
**8076**	6:**40 X** vs. 49:**10 X**	-3.306	-3.450 to -3.162	Yes
**8077**	6:**40 X** vs. 49:**40 X**	-0.6763	-0.8203 to -0.5324	Yes
**8078**	6:**40 X** vs. 49:**50 X**	-0.4310	-0.5750 to -0.2870	Yes
**8079**	6:**40 X** vs. 50:**0 X**	-3.408	-3.552 to -3.264	Yes
**8080**	6:**40 X** vs. 50:**1 X**	-3.311	-3.455 to -3.167	Yes
**8081**	6:**40 X** vs. 50:**10 X**	-3.306	-3.450 to -3.162	Yes
**8082**	6:**40 X** vs. 50:**40 X**	-0.6840	-0.8280 to -0.5400	Yes
**8083**	6:**40 X** vs. 50:**50 X**	-0.4307	-0.5746 to -0.2867	Yes
**8084**	6:**50 X** vs. 7:**0 X**	-0.008667	-0.1526 to 0.1353	No
**8085**	6:**50 X** vs. 7:**1 X**	0.0	-0.1440 to 0.1440	No
**8086**	6:**50 X** vs. 7:**10 X**	-0.0120	-0.1560 to 0.1320	No
**8087**	6:**50 X** vs. 7:**40 X**	-0.003333	-0.1473 to 0.1406	No
**8088**	6:**50 X** vs. 7:**50 X**	-0.001333	-0.1453 to 0.1426	No
**8089**	6:**50 X** vs. 8:**0 X**	-0.01333	-0.1573 to 0.1306	No
**8090**	6:**50 X** vs. 8:**1 X**	0.0	-0.1440 to 0.1440	No
**8091**	6:**50 X** vs. 8:**10 X**	-0.007333	-0.1513 to 0.1366	No
**8092**	6:**50 X** vs. 8:**40 X**	-0.008000	-0.1520 to 0.1360	No
**8093**	6:**50 X** vs. 8:**50 X**	-0.0050	-0.1490 to 0.1390	No
**8094**	6:**50 X** vs. 9:**0 X**	-0.02133	-0.1653 to 0.1226	No
**8095**	6:**50 X** vs. 9:**1 X**	-0.02933	-0.1733 to 0.1146	No
**8096**	6:**50 X** vs. 9:**10 X**	-0.03033	-0.1743 to 0.1136	No
**8097**	6:**50 X** vs. 9:**40 X**	-0.01267	-0.1566 to 0.1313	No
**8098**	6:**50 X** vs. 9:**50 X**	-0.006000	-0.1500 to 0.1380	No
**8099**	6:**50 X** vs. 10:**0 X**	-0.0230	-0.1670 to 0.1210	No
**8100**	6:**50 X** vs. 10:**1 X**	-0.0300	-0.1740 to 0.1140	No

	2way ANOVA<br>Multiple comparisons	A<br>Data Set-A<br>Y	B<br>Data Set-B<br>Y	C<br>Data Set-C<br>Y
8101	6:**50 X** vs. 10:**10 X**	-0.03267	-0.1766 to 0.1113	No
8102	6:**50 X** vs. 10:**40 X**	-0.02233	-0.1663 to 0.1216	No
8103	6:**50 X** vs. 10:**50 X**	-0.0090	-0.1530 to 0.1350	No
8104	6:**50 X** vs. 11:**0 X**	-0.03267	-0.1766 to 0.1113	No
8105	6:**50 X** vs. 11:**1 X**	-0.03433	-0.1783 to 0.1096	No
8106	6:**50 X** vs. 11:**10 X**	-0.03467	-0.1786 to 0.1093	No
8107	6:**50 X** vs. 11:**40 X**	-0.02633	-0.1703 to 0.1176	No
8108	6:**50 X** vs. 11:**50 X**	-0.01433	-0.1583 to 0.1296	No
8109	6:**50 X** vs. 12:**0 X**	-0.03333	-0.1773 to 0.1106	No
8110	6:**50 X** vs. 12:**1 X**	-0.01567	-0.1596 to 0.1283	No
8111	6:**50 X** vs. 12:**10 X**	-0.03333	-0.1773 to 0.1106	No
8112	6:**50 X** vs. 12:**40 X**	-0.01967	-0.1636 to 0.1243	No
8113	6:**50 X** vs. 12:**50 X**	-0.0230	-0.1670 to 0.1210	No
8114	6:**50 X** vs. 13:**0 X**	-0.02733	-0.1713 to 0.1166	No
8115	6:**50 X** vs. 13:**1 X**	-0.01467	-0.1586 to 0.1293	No
8116	6:**50 X** vs. 13:**10 X**	-0.03667	-0.1806 to 0.1073	No
8117	6:**50 X** vs. 13:**40 X**	-0.0330	-0.1770 to 0.1110	No
8118	6:**50 X** vs. 13:**50 X**	-0.0340	-0.1780 to 0.1100	No
8119	6:**50 X** vs. 14:**0 X**	-0.0360	-0.1800 to 0.1080	No
8120	6:**50 X** vs. 14:**1 X**	-0.02767	-0.1716 to 0.1163	No
8121	6:**50 X** vs. 14:**10 X**	-0.04167	-0.1856 to 0.1023	No
8122	6:**50 X** vs. 14:**40 X**	-0.03833	-0.1823 to 0.1056	No
8123	6:**50 X** vs. 14:**50 X**	-0.04233	-0.1863 to 0.1016	No
8124	6:**50 X** vs. 15:**0 X**	-0.04833	-0.1923 to 0.09563	No
8125	6:**50 X** vs. 15:**1 X**	-0.02833	-0.1723 to 0.1156	No
8126	6:**50 X** vs. 15:**10 X**	-0.05567	-0.1996 to 0.08829	No
8127	6:**50 X** vs. 15:**40 X**	-0.0380	-0.1820 to 0.1060	No
8128	6:**50 X** vs. 15:**50 X**	-0.05567	-0.1996 to 0.08829	No
8129	6:**50 X** vs. 16:**0 X**	-0.1407	-0.2846 to 0.003293	No
8130	6:**50 X** vs. 16:**1 X**	-0.03167	-0.1756 to 0.1123	No
8131	6:**50 X** vs. 16:**10 X**	-0.05733	-0.2013 to 0.08663	No
8132	6:**50 X** vs. 16:**40 X**	-0.07667	-0.2206 to 0.06729	No
8133	6:**50 X** vs. 16:**50 X**	-0.1190	-0.2630 to 0.02496	No
8134	6:**50 X** vs. 17:**0 X**	-0.1933	-0.3373 to -0.04937	Yes
8135	6:**50 X** vs. 17:**1 X**	-0.1077	-0.2516 to 0.03629	No
8136	6:**50 X** vs. 17:**10 X**	-0.1643	-0.3083 to -0.02037	Yes
8137	6:**50 X** vs. 17:**40 X**	-0.08767	-0.2316 to 0.05629	No
8138	6:**50 X** vs. 17:**50 X**	-0.1330	-0.2770 to 0.01096	No
8139	6:**50 X** vs. 18:**0 X**	-0.2420	-0.3860 to -0.09804	Yes
8140	6:**50 X** vs. 18:**1 X**	-0.1503	-0.2943 to -0.006374	Yes
8141	6:**50 X** vs. 18:**10 X**	-0.2427	-0.3866 to -0.09871	Yes
8142	6:**50 X** vs. 18:**40 X**	-0.0920	-0.2360 to 0.05196	No
8143	6:**50 X** vs. 18:**50 X**	-0.1370	-0.2810 to 0.006960	No
8144	6:**50 X** vs. 19:**0 X**	-0.2770	-0.4210 to -0.1330	Yes
8145	6:**50 X** vs. 19:**1 X**	-0.2650	-0.4090 to -0.1210	Yes

	2way ANOVA<br>Multiple comparisons	A<br>Data Set-A<br>Y	B<br>Data Set-B<br>Y	C<br>Data Set-C<br>Y
8146	6:50 X vs. 19:10 X	-0.2627	-0.4066 to -0.1187	Yes
8147	6:50 X vs. 19:40 X	-0.1207	-0.2646 to 0.02329	No
8148	6:50 X vs. 19:50 X	-0.1317	-0.2756 to 0.01229	No
8149	6:50 X vs. 20:0 X	-0.3567	-0.5006 to -0.2127	Yes
8150	6:50 X vs. 20:1 X	-0.1760	-0.3200 to -0.03204	Yes
8151	6:50 X vs. 20:10 X	-0.2650	-0.4090 to -0.1210	Yes
8152	6:50 X vs. 20:40 X	-0.1323	-0.2763 to 0.01163	No
8153	6:50 X vs. 20:50 X	-0.1763	-0.3203 to -0.03237	Yes
8154	6:50 X vs. 21:0 X	-0.3800	-0.5240 to -0.2360	Yes
8155	6:50 X vs. 21:1 X	-0.3330	-0.4770 to -0.1890	Yes
8156	6:50 X vs. 21:10 X	-0.3300	-0.4740 to -0.1860	Yes
8157	6:50 X vs. 21:40 X	-0.2053	-0.3493 to -0.06137	Yes
8158	6:50 X vs. 21:50 X	-0.2957	-0.4396 to -0.1517	Yes
8159	6:50 X vs. 22:0 X	-0.4217	-0.5656 to -0.2777	Yes
8160	6:50 X vs. 22:1 X	-0.4163	-0.5603 to -0.2724	Yes
8161	6:50 X vs. 22:10 X	-0.4117	-0.5556 to -0.2677	Yes
8162	6:50 X vs. 22:40 X	-0.3120	-0.4560 to -0.1680	Yes
8163	6:50 X vs. 22:50 X	-0.3747	-0.5186 to -0.2307	Yes
8164	6:50 X vs. 23:0 X	-0.4870	-0.6310 to -0.3430	Yes
8165	6:50 X vs. 23:1 X	-0.6767	-0.8376 to -0.5157	Yes
8166	6:50 X vs. 23:10 X	-0.4263	-0.5703 to -0.2824	Yes
8167	6:50 X vs. 23:40 X	-0.3067	-0.4676 to -0.1457	Yes
8168	6:50 X vs. 23:50 X	-0.3407	-0.4846 to -0.1967	Yes
8169	6:50 X vs. 24:0 X	-0.6043	-0.7483 to -0.4604	Yes
8170	6:50 X vs. 24:1 X	-0.7607	-0.9216 to -0.5997	Yes
8171	6:50 X vs. 24:10 X	-0.5882	-0.7491 to -0.4272	Yes
8172	6:50 X vs. 24:40 X	-0.3230	-0.4670 to -0.1790	Yes
8173	6:50 X vs. 24:50 X	-0.2447	-0.3886 to -0.1007	Yes
8174	6:50 X vs. 25:0 X	-0.9417	-1.103 to -0.7807	Yes
8175	6:50 X vs. 25:1 X	-1.131	-1.292 to -0.9702	Yes
8176	6:50 X vs. 25:10 X	-0.4927	-0.6963 to -0.2891	Yes
8177	6:50 X vs. 25:40 X	-0.3250	-0.4690 to -0.1810	Yes
8178	6:50 X vs. 25:50 X	-0.2993	-0.4433 to -0.1554	Yes
8179	6:50 X vs. 26:0 X	-1.154	-1.315 to -0.9932	Yes
8180	6:50 X vs. 26:1 X	-1.267	-1.428 to -1.106	Yes
8181	6:50 X vs. 26:10 X	-0.9537	-1.115 to -0.7927	Yes
8182	6:50 X vs. 26:40 X	-0.3993	-0.5433 to -0.2554	Yes
8183	6:50 X vs. 26:50 X	-0.2597	-0.4036 to -0.1157	Yes
8184	6:50 X vs. 27:0 X	-1.342	-1.486 to -1.198	Yes
8185	6:50 X vs. 27:1 X	-1.433	-1.594 to -1.272	Yes
8186	6:50 X vs. 27:10 X	-1.245	-1.406 to -1.084	Yes
8187	6:50 X vs. 27:40 X	-0.3993	-0.5433 to -0.2554	Yes
8188	6:50 X vs. 27:50 X	-0.2590	-0.4030 to -0.1150	Yes
8189	6:50 X vs. 28:0 X	-1.291	-1.452 to -1.130	Yes
8190	6:50 X vs. 28:1 X	-1.332	-1.476 to -1.188	Yes

	2way ANOVA<br>Multiple comparisons	A<br>Data Set-A<br>Y	B<br>Data Set-B<br>Y	C<br>Data Set-C<br>Y
8191	6:50 X vs. 28:10 X	-1.134	-1.295 to -0.9727	Yes
8192	6:50 X vs. 28:40 X	-0.4117	-0.5556 to -0.2677	Yes
8193	6:50 X vs. 28:50 X	-0.3070	-0.4510 to -0.1630	Yes
8194	6:50 X vs. 29:0 X	-1.773	-1.934 to -1.612	Yes
8195	6:50 X vs. 29:1 X	-1.858	-2.019 to -1.697	Yes
8196	6:50 X vs. 29:10 X	-1.072	-1.233 to -0.9112	Yes
8197	6:50 X vs. 29:40 X	-0.5197	-0.6636 to -0.3757	Yes
8198	6:50 X vs. 29:50 X	-0.3083	-0.4523 to -0.1644	Yes
8199	6:50 X vs. 30:0 X	-1.487	-1.648 to -1.326	Yes
8200	6:50 X vs. 30:1 X	-1.809	-1.970 to -1.648	Yes
8201	6:50 X vs. 30:10 X	-1.147	-1.308 to -0.9857	Yes
8202	6:50 X vs. 30:40 X	-0.4467	-0.5906 to -0.3027	Yes
8203	6:50 X vs. 30:50 X	-0.3607	-0.5046 to -0.2167	Yes
8204	6:50 X vs. 31:0 X	-1.805	-1.966 to -1.644	Yes
8205	6:50 X vs. 31:1 X	-1.796	-1.957 to -1.635	Yes
8206	6:50 X vs. 31:10 X	-1.278	-1.439 to -1.117	Yes
8207	6:50 X vs. 31:40 X	-0.5383	-0.6823 to -0.3944	Yes
8208	6:50 X vs. 31:50 X	-0.3397	-0.4836 to -0.1957	Yes
8209	6:50 X vs. 32:0 X	-2.397	-2.541 to -2.253	Yes
8210	6:50 X vs. 32:1 X	-1.853	-2.014 to -1.692	Yes
8211	6:50 X vs. 32:10 X	-1.709	-1.853 to -1.565	Yes
8212	6:50 X vs. 32:40 X	-0.5347	-0.6786 to -0.3907	Yes
8213	6:50 X vs. 32:50 X	-0.3393	-0.4833 to -0.1954	Yes
8214	6:50 X vs. 33:0 X	-2.400	-2.544 to -2.256	Yes
8215	6:50 X vs. 33:1 X	-2.215	-2.359 to -2.071	Yes
8216	6:50 X vs. 33:10 X	-1.875	-2.019 to -1.731	Yes
8217	6:50 X vs. 33:40 X	-0.5533	-0.6973 to -0.4094	Yes
8218	6:50 X vs. 33:50 X	-0.3543	-0.4983 to -0.2104	Yes
8219	6:50 X vs. 34:0 X	-2.325	-2.469 to -2.181	Yes
8220	6:50 X vs. 34:1 X	-2.332	-2.476 to -2.188	Yes
8221	6:50 X vs. 34:10 X	-1.966	-2.110 to -1.822	Yes
8222	6:50 X vs. 34:40 X	-0.5653	-0.7093 to -0.4214	Yes
8223	6:50 X vs. 34:50 X	-0.5033	-0.6473 to -0.3594	Yes
8224	6:50 X vs. 35:0 X	-2.674	-2.818 to -2.530	Yes
8225	6:50 X vs. 35:1 X	-2.439	-2.583 to -2.295	Yes
8226	6:50 X vs. 35:10 X	-1.927	-2.071 to -1.783	Yes
8227	6:50 X vs. 35:40 X	-0.5743	-0.7183 to -0.4304	Yes
8228	6:50 X vs. 35:50 X	-0.5160	-0.6600 to -0.3720	Yes
8229	6:50 X vs. 36:0 X	-2.880	-3.041 to -2.719	Yes
8230	6:50 X vs. 36:1 X	-2.576	-2.720 to -2.432	Yes
8231	6:50 X vs. 36:10 X	-1.933	-2.077 to -1.789	Yes
8232	6:50 X vs. 36:40 X	-0.6297	-0.7736 to -0.4857	Yes
8233	6:50 X vs. 36:50 X	-0.4793	-0.6233 to -0.3354	Yes
8234	6:50 X vs. 37:0 X	-3.264	-3.408 to -3.120	Yes
8235	6:50 X vs. 37:1 X	-2.646	-2.790 to -2.502	Yes

	2way ANOVA<br>Multiple comparisons	A<br>Data Set-A<br>Y	B<br>Data Set-B<br>Y	C<br>Data Set-C<br>Y
8236	6:50 X vs. 37:10 X	-1.935	-2.079 to -1.791	Yes
8237	6:50 X vs. 37:40 X	-0.6670	-0.8110 to -0.5230	Yes
8238	6:50 X vs. 37:50 X	-0.4880	-0.6320 to -0.3440	Yes
8239	6:50 X vs. 38:0 X	-3.209	-3.353 to -3.065	Yes
8240	6:50 X vs. 38:1 X	-2.765	-2.909 to -2.621	Yes
8241	6:50 X vs. 38:10 X	-2.210	-2.354 to -2.066	Yes
8242	6:50 X vs. 38:40 X	-0.6713	-0.8153 to -0.5274	Yes
8243	6:50 X vs. 38:50 X	-0.4920	-0.6360 to -0.3480	Yes
8244	6:50 X vs. 39:0 X	-3.440	-3.584 to -3.296	Yes
8245	6:50 X vs. 39:1 X	-3.331	-3.475 to -3.187	Yes
8246	6:50 X vs. 39:10 X	-2.355	-2.499 to -2.211	Yes
8247	6:50 X vs. 39:40 X	-0.6773	-0.8213 to -0.5334	Yes
8248	6:50 X vs. 39:50 X	-0.5230	-0.6670 to -0.3790	Yes
8249	6:50 X vs. 40:0 X	-3.481	-3.625 to -3.337	Yes
8250	6:50 X vs. 40:1 X	-3.407	-3.551 to -3.263	Yes
8251	6:50 X vs. 40:10 X	-2.611	-2.755 to -2.467	Yes
8252	6:50 X vs. 40:40 X	-0.6820	-0.8260 to -0.5380	Yes
8253	6:50 X vs. 40:50 X	-0.5293	-0.6733 to -0.3854	Yes
8254	6:50 X vs. 41:0 X	-3.522	-3.666 to -3.378	Yes
8255	6:50 X vs. 41:1 X	-3.521	-3.665 to -3.377	Yes
8256	6:50 X vs. 41:10 X	-2.665	-2.809 to -2.521	Yes
8257	6:50 X vs. 41:40 X	-0.6293	-0.7733 to -0.4854	Yes
8258	6:50 X vs. 41:50 X	-0.5420	-0.6860 to -0.3980	Yes
8259	6:50 X vs. 42:0 X	-3.663	-3.807 to -3.519	Yes
8260	6:50 X vs. 42:1 X	-3.683	-3.827 to -3.539	Yes
8261	6:50 X vs. 42:10 X	-2.665	-2.809 to -2.521	Yes
8262	6:50 X vs. 42:40 X	-0.6420	-0.7860 to -0.4980	Yes
8263	6:50 X vs. 42:50 X	-0.5453	-0.6893 to -0.4014	Yes
8264	6:50 X vs. 43:0 X	-3.753	-3.897 to -3.609	Yes
8265	6:50 X vs. 43:1 X	-3.668	-3.812 to -3.524	Yes
8266	6:50 X vs. 43:10 X	-3.383	-3.527 to -3.239	Yes
8267	6:50 X vs. 43:40 X	-0.6403	-0.7843 to -0.4964	Yes
8268	6:50 X vs. 43:50 X	-0.5457	-0.6896 to -0.4017	Yes
8269	6:50 X vs. 44:0 X	-3.777	-3.921 to -3.633	Yes
8270	6:50 X vs. 44:1 X	-3.667	-3.811 to -3.523	Yes
8271	6:50 X vs. 44:10 X	-3.447	-3.591 to -3.303	Yes
8272	6:50 X vs. 44:40 X	-0.6423	-0.7863 to -0.4984	Yes
8273	6:50 X vs. 44:50 X	-0.4780	-0.6220 to -0.3340	Yes
8274	6:50 X vs. 45:0 X	-3.642	-3.786 to -3.498	Yes
8275	6:50 X vs. 45:1 X	-3.444	-3.588 to -3.300	Yes
8276	6:50 X vs. 45:10 X	-3.326	-3.470 to -3.182	Yes
8277	6:50 X vs. 45:40 X	-0.6437	-0.7876 to -0.4997	Yes
8278	6:50 X vs. 45:50 X	-0.4750	-0.6190 to -0.3310	Yes
8279	6:50 X vs. 46:0 X	-3.446	-3.590 to -3.302	Yes
8280	6:50 X vs. 46:1 X	-3.413	-3.557 to -3.269	Yes

	2way ANOVA<br>Multiple comparisons	A<br>Data Set-A<br>Y	B<br>Data Set-B<br>Y	C<br>Data Set-C<br>Y
8281	6:**50 X** vs. 46:**10 X**	-3.321	-3.465 to -3.177	Yes
8282	6:**50 X** vs. 46:**40 X**	-0.6573	-0.8013 to -0.5134	Yes
8283	6:**50 X** vs. 46:**50 X**	-0.4680	-0.6120 to -0.3240	Yes
8284	6:**50 X** vs. 47:**0 X**	-3.422	-3.566 to -3.278	Yes
8285	6:**50 X** vs. 47:**1 X**	-3.326	-3.470 to -3.182	Yes
8286	6:**50 X** vs. 47:**10 X**	-3.307	-3.451 to -3.163	Yes
8287	6:**50 X** vs. 47:**40 X**	-0.6673	-0.8113 to -0.5234	Yes
8288	6:**50 X** vs. 47:**50 X**	-0.4260	-0.5700 to -0.2820	Yes
8289	6:**50 X** vs. 48:**0 X**	-3.409	-3.570 to -3.248	Yes
8290	6:**50 X** vs. 48:**1 X**	-3.313	-3.457 to -3.169	Yes
8291	6:**50 X** vs. 48:**10 X**	-3.307	-3.451 to -3.163	Yes
8292	6:**50 X** vs. 48:**40 X**	-0.6737	-0.8176 to -0.5297	Yes
8293	6:**50 X** vs. 48:**50 X**	-0.4333	-0.5773 to -0.2894	Yes
8294	6:**50 X** vs. 49:**0 X**	-3.409	-3.553 to -3.265	Yes
8295	6:**50 X** vs. 49:**1 X**	-3.313	-3.457 to -3.169	Yes
8296	6:**50 X** vs. 49:**10 X**	-3.307	-3.451 to -3.163	Yes
8297	6:**50 X** vs. 49:**40 X**	-0.6780	-0.8220 to -0.5340	Yes
8298	6:**50 X** vs. 49:**50 X**	-0.4327	-0.5766 to -0.2887	Yes
8299	6:**50 X** vs. 50:**0 X**	-3.409	-3.553 to -3.265	Yes
8300	6:**50 X** vs. 50:**1 X**	-3.313	-3.457 to -3.169	Yes
8301	6:**50 X** vs. 50:**10 X**	-3.307	-3.451 to -3.163	Yes
8302	6:**50 X** vs. 50:**40 X**	-0.6857	-0.8296 to -0.5417	Yes
8303	6:**50 X** vs. 50:**50 X**	-0.4323	-0.5763 to -0.2884	Yes
8304	7:**0 X** vs. 7:**1 X**	0.008667	-0.1353 to 0.1526	No
8305	7:**0 X** vs. 7:**10 X**	-0.003333	-0.1473 to 0.1406	No
8306	7:**0 X** vs. 7:**40 X**	0.005333	-0.1386 to 0.1493	No
8307	7:**0 X** vs. 7:**50 X**	0.007333	-0.1366 to 0.1513	No
8308	7:**0 X** vs. 8:**0 X**	-0.004667	-0.1486 to 0.1393	No
8309	7:**0 X** vs. 8:**1 X**	0.008667	-0.1353 to 0.1526	No
8310	7:**0 X** vs. 8:**10 X**	0.001333	-0.1426 to 0.1453	No
8311	7:**0 X** vs. 8:**40 X**	0.0006667	-0.1433 to 0.1446	No
8312	7:**0 X** vs. 8:**50 X**	0.003667	-0.1403 to 0.1476	No
8313	7:**0 X** vs. 9:**0 X**	-0.01267	-0.1566 to 0.1313	No
8314	7:**0 X** vs. 9:**1 X**	-0.02067	-0.1646 to 0.1233	No
8315	7:**0 X** vs. 9:**10 X**	-0.02167	-0.1656 to 0.1223	No
8316	7:**0 X** vs. 9:**40 X**	-0.004000	-0.1480 to 0.1400	No
8317	7:**0 X** vs. 9:**50 X**	0.002667	-0.1413 to 0.1466	No
8318	7:**0 X** vs. 10:**0 X**	-0.01433	-0.1583 to 0.1296	No
8319	7:**0 X** vs. 10:**1 X**	-0.02133	-0.1653 to 0.1226	No
8320	7:**0 X** vs. 10:**10 X**	-0.0240	-0.1680 to 0.1200	No
8321	7:**0 X** vs. 10:**40 X**	-0.01367	-0.1576 to 0.1303	No
8322	7:**0 X** vs. 10:**50 X**	-0.0003333	-0.1443 to 0.1436	No
8323	7:**0 X** vs. 11:**0 X**	-0.0240	-0.1680 to 0.1200	No
8324	7:**0 X** vs. 11:**1 X**	-0.02567	-0.1696 to 0.1183	No
8325	7:**0 X** vs. 11:**10 X**	-0.0260	-0.1700 to 0.1180	No

	2way ANOVA<br>Multiple comparisons	A<br>Data Set-A<br>Y	B<br>Data Set-B<br>Y	C<br>Data Set-C<br>Y
8326	7:**0 X** vs. 11:**40 X**	-0.01767	-0.1616 to 0.1263	No
8327	7:**0 X** vs. 11:**50 X**	-0.005667	-0.1496 to 0.1383	No
8328	7:**0 X** vs. 12:**0 X**	-0.02467	-0.1686 to 0.1193	No
8329	7:**0 X** vs. 12:**1 X**	-0.007000	-0.1510 to 0.1370	No
8330	7:**0 X** vs. 12:**10 X**	-0.02467	-0.1686 to 0.1193	No
8331	7:**0 X** vs. 12:**40 X**	-0.0110	-0.1550 to 0.1330	No
8332	7:**0 X** vs. 12:**50 X**	-0.01433	-0.1583 to 0.1296	No
8333	7:**0 X** vs. 13:**0 X**	-0.01867	-0.1626 to 0.1253	No
8334	7:**0 X** vs. 13:**1 X**	-0.0060	-0.1500 to 0.1380	No
8335	7:**0 X** vs. 13:**10 X**	-0.0280	-0.1720 to 0.1160	No
8336	7:**0 X** vs. 13:**40 X**	-0.02433	-0.1683 to 0.1196	No
8337	7:**0 X** vs. 13:**50 X**	-0.02533	-0.1693 to 0.1186	No
8338	7:**0 X** vs. 14:**0 X**	-0.02733	-0.1713 to 0.1166	No
8339	7:**0 X** vs. 14:**1 X**	-0.0190	-0.1630 to 0.1250	No
8340	7:**0 X** vs. 14:**10 X**	-0.0330	-0.1770 to 0.1110	No
8341	7:**0 X** vs. 14:**40 X**	-0.02967	-0.1736 to 0.1143	No
8342	7:**0 X** vs. 14:**50 X**	-0.03367	-0.1776 to 0.1103	No
8343	7:**0 X** vs. 15:**0 X**	-0.03967	-0.1836 to 0.1043	No
8344	7:**0 X** vs. 15:**1 X**	-0.01967	-0.1636 to 0.1243	No
8345	7:**0 X** vs. 15:**10 X**	-0.0470	-0.1910 to 0.09696	No
8346	7:**0 X** vs. 15:**40 X**	-0.02933	-0.1733 to 0.1146	No
8347	7:**0 X** vs. 15:**50 X**	-0.0470	-0.1910 to 0.09696	No
8348	7:**0 X** vs. 16:**0 X**	-0.1320	-0.2760 to 0.01196	No
8349	7:**0 X** vs. 16:**1 X**	-0.0230	-0.1670 to 0.1210	No
8350	7:**0 X** vs. 16:**10 X**	-0.04867	-0.1926 to 0.09529	No
8351	7:**0 X** vs. 16:**40 X**	-0.0680	-0.2120 to 0.07596	No
8352	7:**0 X** vs. 16:**50 X**	-0.1103	-0.2543 to 0.03363	No
8353	7:**0 X** vs. 17:**0 X**	-0.1847	-0.3286 to -0.04071	Yes
8354	7:**0 X** vs. 17:**1 X**	-0.09900	-0.2430 to 0.04496	No
8355	7:**0 X** vs. 17:**10 X**	-0.1557	-0.2996 to -0.01171	Yes
8356	7:**0 X** vs. 17:**40 X**	-0.0790	-0.2230 to 0.06496	No
8357	7:**0 X** vs. 17:**50 X**	-0.1243	-0.2683 to 0.01963	No
8358	7:**0 X** vs. 18:**0 X**	-0.2333	-0.3773 to -0.08937	Yes
8359	7:**0 X** vs. 18:**1 X**	-0.1417	-0.2856 to 0.002293	No
8360	7:**0 X** vs. 18:**10 X**	-0.2340	-0.3780 to -0.09004	Yes
8361	7:**0 X** vs. 18:**40 X**	-0.08333	-0.2273 to 0.06063	No
8362	7:**0 X** vs. 18:**50 X**	-0.1283	-0.2723 to 0.01563	No
8363	7:**0 X** vs. 19:**0 X**	-0.2683	-0.4123 to -0.1244	Yes
8364	7:**0 X** vs. 19:**1 X**	-0.2563	-0.4003 to -0.1124	Yes
8365	7:**0 X** vs. 19:**10 X**	-0.2540	-0.3980 to -0.1100	Yes
8366	7:**0 X** vs. 19:**40 X**	-0.1120	-0.2560 to 0.03196	No
8367	7:**0 X** vs. 19:**50 X**	-0.1230	-0.2670 to 0.02096	No
8368	7:**0 X** vs. 20:**0 X**	-0.3480	-0.4920 to -0.2040	Yes
8369	7:**0 X** vs. 20:**1 X**	-0.1673	-0.3113 to -0.02337	Yes
8370	7:**0 X** vs. 20:**10 X**	-0.2563	-0.4003 to -0.1124	Yes

	2way ANOVA<br>Multiple comparisons	A<br>Data Set-A<br>Y	B<br>Data Set-B<br>Y	C<br>Data Set-C<br>Y
8371	7:0 X vs. 20:40 X	-0.1237	-0.2676 to 0.02029	No
8372	7:0 X vs. 20:50 X	-0.1677	-0.3116 to -0.02371	Yes
8373	7:0 X vs. 21:0 X	-0.3713	-0.5153 to -0.2274	Yes
8374	7:0 X vs. 21:1 X	-0.3243	-0.4683 to -0.1804	Yes
8375	7:0 X vs. 21:10 X	-0.3213	-0.4653 to -0.1774	Yes
8376	7:0 X vs. 21:40 X	-0.1967	-0.3406 to -0.05271	Yes
8377	7:0 X vs. 21:50 X	-0.2870	-0.4310 to -0.1430	Yes
8378	7:0 X vs. 22:0 X	-0.4130	-0.5570 to -0.2690	Yes
8379	7:0 X vs. 22:1 X	-0.4077	-0.5516 to -0.2637	Yes
8380	7:0 X vs. 22:10 X	-0.4030	-0.5470 to -0.2590	Yes
8381	7:0 X vs. 22:40 X	-0.3033	-0.4473 to -0.1594	Yes
8382	7:0 X vs. 22:50 X	-0.3660	-0.5100 to -0.2220	Yes
8383	7:0 X vs. 23:0 X	-0.4783	-0.6223 to -0.3344	Yes
8384	7:0 X vs. 23:1 X	-0.6680	-0.8290 to -0.5070	Yes
8385	7:0 X vs. 23:10 X	-0.4177	-0.5616 to -0.2737	Yes
8386	7:0 X vs. 23:40 X	-0.2980	-0.4590 to -0.1370	Yes
8387	7:0 X vs. 23:50 X	-0.3320	-0.4760 to -0.1880	Yes
8388	7:0 X vs. 24:0 X	-0.5957	-0.7396 to -0.4517	Yes
8389	7:0 X vs. 24:1 X	-0.7520	-0.9130 to -0.5910	Yes
8390	7:0 X vs. 24:10 X	-0.5795	-0.7405 to -0.4185	Yes
8391	7:0 X vs. 24:40 X	-0.3143	-0.4583 to -0.1704	Yes
8392	7:0 X vs. 24:50 X	-0.2360	-0.3800 to -0.09204	Yes
8393	7:0 X vs. 25:0 X	-0.9330	-1.094 to -0.7720	Yes
8394	7:0 X vs. 25:1 X	-1.123	-1.283 to -0.9615	Yes
8395	7:0 X vs. 25:10 X	-0.4840	-0.6876 to -0.2804	Yes
8396	7:0 X vs. 25:40 X	-0.3163	-0.4603 to -0.1724	Yes
8397	7:0 X vs. 25:50 X	-0.2907	-0.4346 to -0.1467	Yes
8398	7:0 X vs. 26:0 X	-1.146	-1.306 to -0.9845	Yes
8399	7:0 X vs. 26:1 X	-1.259	-1.419 to -1.098	Yes
8400	7:0 X vs. 26:10 X	-0.9450	-1.106 to -0.7840	Yes
8401	7:0 X vs. 26:40 X	-0.3907	-0.5346 to -0.2467	Yes
8402	7:0 X vs. 26:50 X	-0.2510	-0.3950 to -0.1070	Yes
8403	7:0 X vs. 27:0 X	-1.333	-1.477 to -1.189	Yes
8404	7:0 X vs. 27:1 X	-1.424	-1.585 to -1.263	Yes
8405	7:0 X vs. 27:10 X	-1.236	-1.397 to -1.075	Yes
8406	7:0 X vs. 27:40 X	-0.3907	-0.5346 to -0.2467	Yes
8407	7:0 X vs. 27:50 X	-0.2503	-0.3943 to -0.1064	Yes
8408	7:0 X vs. 28:0 X	-1.283	-1.443 to -1.122	Yes
8409	7:0 X vs. 28:1 X	-1.323	-1.467 to -1.179	Yes
8410	7:0 X vs. 28:10 X	-1.125	-1.286 to -0.9640	Yes
8411	7:0 X vs. 28:40 X	-0.4030	-0.5470 to -0.2590	Yes
8412	7:0 X vs. 28:50 X	-0.2983	-0.4423 to -0.1544	Yes
8413	7:0 X vs. 29:0 X	-1.764	-1.925 to -1.603	Yes
8414	7:0 X vs. 29:1 X	-1.849	-2.010 to -1.688	Yes
8415	7:0 X vs. 29:10 X	-1.064	-1.224 to -0.9025	Yes

	2way ANOVA<br>Multiple comparisons	A<br>Data Set-A<br>Y	B<br>Data Set-B<br>Y	C<br>Data Set-C<br>Y
8416	7:0 X vs. 29:40 X	-0.5110	-0.6550 to -0.3670	Yes
8417	7:0 X vs. 29:50 X	-0.2997	-0.4436 to -0.1557	Yes
8418	7:0 X vs. 30:0 X	-1.479	-1.639 to -1.318	Yes
8419	7:0 X vs. 30:1 X	-1.800	-1.961 to -1.639	Yes
8420	7:0 X vs. 30:10 X	-1.138	-1.299 to -0.9770	Yes
8421	7:0 X vs. 30:40 X	-0.4380	-0.5820 to -0.2940	Yes
8422	7:0 X vs. 30:50 X	-0.3520	-0.4960 to -0.2080	Yes
8423	7:0 X vs. 31:0 X	-1.797	-1.957 to -1.636	Yes
8424	7:0 X vs. 31:1 X	-1.787	-1.948 to -1.626	Yes
8425	7:0 X vs. 31:10 X	-1.270	-1.430 to -1.109	Yes
8426	7:0 X vs. 31:40 X	-0.5297	-0.6736 to -0.3857	Yes
8427	7:0 X vs. 31:50 X	-0.3310	-0.4750 to -0.1870	Yes
8428	7:0 X vs. 32:0 X	-2.388	-2.532 to -2.244	Yes
8429	7:0 X vs. 32:1 X	-1.844	-2.005 to -1.683	Yes
8430	7:0 X vs. 32:10 X	-1.700	-1.844 to -1.556	Yes
8431	7:0 X vs. 32:40 X	-0.5260	-0.6700 to -0.3820	Yes
8432	7:0 X vs. 32:50 X	-0.3307	-0.4746 to -0.1867	Yes
8433	7:0 X vs. 33:0 X	-2.392	-2.536 to -2.248	Yes
8434	7:0 X vs. 33:1 X	-2.206	-2.350 to -2.062	Yes
8435	7:0 X vs. 33:10 X	-1.867	-2.011 to -1.723	Yes
8436	7:0 X vs. 33:40 X	-0.5447	-0.6886 to -0.4007	Yes
8437	7:0 X vs. 33:50 X	-0.3457	-0.4896 to -0.2017	Yes
8438	7:0 X vs. 34:0 X	-2.316	-2.460 to -2.172	Yes
8439	7:0 X vs. 34:1 X	-2.324	-2.468 to -2.180	Yes
8440	7:0 X vs. 34:10 X	-1.957	-2.101 to -1.813	Yes
8441	7:0 X vs. 34:40 X	-0.5567	-0.7006 to -0.4127	Yes
8442	7:0 X vs. 34:50 X	-0.4947	-0.6386 to -0.3507	Yes
8443	7:0 X vs. 35:0 X	-2.665	-2.809 to -2.521	Yes
8444	7:0 X vs. 35:1 X	-2.431	-2.575 to -2.287	Yes
8445	7:0 X vs. 35:10 X	-1.919	-2.063 to -1.775	Yes
8446	7:0 X vs. 35:40 X	-0.5657	-0.7096 to -0.4217	Yes
8447	7:0 X vs. 35:50 X	-0.5073	-0.6513 to -0.3634	Yes
8448	7:0 X vs. 36:0 X	-2.872	-3.032 to -2.711	Yes
8449	7:0 X vs. 36:1 X	-2.567	-2.711 to -2.423	Yes
8450	7:0 X vs. 36:10 X	-1.924	-2.068 to -1.780	Yes
8451	7:0 X vs. 36:40 X	-0.6210	-0.7650 to -0.4770	Yes
8452	7:0 X vs. 36:50 X	-0.4707	-0.6146 to -0.3267	Yes
8453	7:0 X vs. 37:0 X	-3.255	-3.399 to -3.111	Yes
8454	7:0 X vs. 37:1 X	-2.637	-2.781 to -2.493	Yes
8455	7:0 X vs. 37:10 X	-1.926	-2.070 to -1.782	Yes
8456	7:0 X vs. 37:40 X	-0.6583	-0.8023 to -0.5144	Yes
8457	7:0 X vs. 37:50 X	-0.4793	-0.6233 to -0.3354	Yes
8458	7:0 X vs. 38:0 X	-3.200	-3.344 to -3.056	Yes
8459	7:0 X vs. 38:1 X	-2.756	-2.900 to -2.612	Yes
8460	7:0 X vs. 38:10 X	-2.201	-2.345 to -2.057	Yes

	2way ANOVA<br>Multiple comparisons	A<br>Data Set-A<br>Y	B<br>Data Set-B<br>Y	C<br>Data Set-C<br>Y
8461	7:**0 X** vs. 38:**40 X**	-0.6627	-0.8066 to -0.5187	Yes
8462	7:**0 X** vs. 38:**50 X**	-0.4833	-0.6273 to -0.3394	Yes
8463	7:**0 X** vs. 39:**0 X**	-3.431	-3.575 to -3.287	Yes
8464	7:**0 X** vs. 39:**1 X**	-3.323	-3.467 to -3.179	Yes
8465	7:**0 X** vs. 39:**10 X**	-2.346	-2.490 to -2.202	Yes
8466	7:**0 X** vs. 39:**40 X**	-0.6687	-0.8126 to -0.5247	Yes
8467	7:**0 X** vs. 39:**50 X**	-0.5143	-0.6583 to -0.3704	Yes
8468	7:**0 X** vs. 40:**0 X**	-3.472	-3.616 to -3.328	Yes
8469	7:**0 X** vs. 40:**1 X**	-3.398	-3.542 to -3.254	Yes
8470	7:**0 X** vs. 40:**10 X**	-2.603	-2.747 to -2.459	Yes
8471	7:**0 X** vs. 40:**40 X**	-0.6733	-0.8173 to -0.5294	Yes
8472	7:**0 X** vs. 40:**50 X**	-0.5207	-0.6646 to -0.3767	Yes
8473	7:**0 X** vs. 41:**0 X**	-3.513	-3.657 to -3.369	Yes
8474	7:**0 X** vs. 41:**1 X**	-3.512	-3.656 to -3.368	Yes
8475	7:**0 X** vs. 41:**10 X**	-2.656	-2.800 to -2.512	Yes
8476	7:**0 X** vs. 41:**40 X**	-0.6207	-0.7646 to -0.4767	Yes
8477	7:**0 X** vs. 41:**50 X**	-0.5333	-0.6773 to -0.3894	Yes
8478	7:**0 X** vs. 42:**0 X**	-3.655	-3.799 to -3.511	Yes
8479	7:**0 X** vs. 42:**1 X**	-3.674	-3.818 to -3.530	Yes
8480	7:**0 X** vs. 42:**10 X**	-2.657	-2.801 to -2.513	Yes
8481	7:**0 X** vs. 42:**40 X**	-0.6333	-0.7773 to -0.4894	Yes
8482	7:**0 X** vs. 42:**50 X**	-0.5367	-0.6806 to -0.3927	Yes
8483	7:**0 X** vs. 43:**0 X**	-3.745	-3.889 to -3.601	Yes
8484	7:**0 X** vs. 43:**1 X**	-3.659	-3.803 to -3.515	Yes
8485	7:**0 X** vs. 43:**10 X**	-3.375	-3.519 to -3.231	Yes
8486	7:**0 X** vs. 43:**40 X**	-0.6317	-0.7756 to -0.4877	Yes
8487	7:**0 X** vs. 43:**50 X**	-0.5370	-0.6810 to -0.3930	Yes
8488	7:**0 X** vs. 44:**0 X**	-3.769	-3.913 to -3.625	Yes
8489	7:**0 X** vs. 44:**1 X**	-3.658	-3.802 to -3.514	Yes
8490	7:**0 X** vs. 44:**10 X**	-3.438	-3.582 to -3.294	Yes
8491	7:**0 X** vs. 44:**40 X**	-0.6337	-0.7776 to -0.4897	Yes
8492	7:**0 X** vs. 44:**50 X**	-0.4693	-0.6133 to -0.3254	Yes
8493	7:**0 X** vs. 45:**0 X**	-3.633	-3.777 to -3.489	Yes
8494	7:**0 X** vs. 45:**1 X**	-3.435	-3.579 to -3.291	Yes
8495	7:**0 X** vs. 45:**10 X**	-3.318	-3.462 to -3.174	Yes
8496	7:**0 X** vs. 45:**40 X**	-0.6350	-0.7790 to -0.4910	Yes
8497	7:**0 X** vs. 45:**50 X**	-0.4663	-0.6103 to -0.3224	Yes
8498	7:**0 X** vs. 46:**0 X**	-3.438	-3.582 to -3.294	Yes
8499	7:**0 X** vs. 46:**1 X**	-3.405	-3.549 to -3.261	Yes
8500	7:**0 X** vs. 46:**10 X**	-3.313	-3.457 to -3.169	Yes
8501	7:**0 X** vs. 46:**40 X**	-0.6487	-0.7926 to -0.5047	Yes
8502	7:**0 X** vs. 46:**50 X**	-0.4593	-0.6033 to -0.3154	Yes
8503	7:**0 X** vs. 47:**0 X**	-3.413	-3.557 to -3.269	Yes
8504	7:**0 X** vs. 47:**1 X**	-3.317	-3.461 to -3.173	Yes
8505	7:**0 X** vs. 47:**10 X**	-3.299	-3.443 to -3.155	Yes

	2way ANOVA Multiple comparisons	A Data Set-A Y	B Data Set-B Y	C Data Set-C Y
8506	7:**0 X** vs. 47:**40 X**	-0.6587	-0.8026 to -0.5147	Yes
8507	7:**0 X** vs. 47:**50 X**	-0.4173	-0.5613 to -0.2734	Yes
8508	7:**0 X** vs. 48:**0 X**	-3.401	-3.561 to -3.240	Yes
8509	7:**0 X** vs. 48:**1 X**	-3.304	-3.448 to -3.160	Yes
8510	7:**0 X** vs. 48:**10 X**	-3.299	-3.443 to -3.155	Yes
8511	7:**0 X** vs. 48:**40 X**	-0.6650	-0.8090 to -0.5210	Yes
8512	7:**0 X** vs. 48:**50 X**	-0.4247	-0.5686 to -0.2807	Yes
8513	7:**0 X** vs. 49:**0 X**	-3.401	-3.545 to -3.257	Yes
8514	7:**0 X** vs. 49:**1 X**	-3.304	-3.448 to -3.160	Yes
8515	7:**0 X** vs. 49:**10 X**	-3.299	-3.443 to -3.155	Yes
8516	7:**0 X** vs. 49:**40 X**	-0.6693	-0.8133 to -0.5254	Yes
8517	7:**0 X** vs. 49:**50 X**	-0.4240	-0.5680 to -0.2800	Yes
8518	7:**0 X** vs. 50:**0 X**	-3.401	-3.545 to -3.257	Yes
8519	7:**0 X** vs. 50:**1 X**	-3.304	-3.448 to -3.160	Yes
8520	7:**0 X** vs. 50:**10 X**	-3.299	-3.443 to -3.155	Yes
8521	7:**0 X** vs. 50:**40 X**	-0.6770	-0.8210 to -0.5330	Yes
8522	7:**0 X** vs. 50:**50 X**	-0.4237	-0.5676 to -0.2797	Yes
8523	7:**1 X** vs. 7:**10 X**	-0.0120	-0.1560 to 0.1320	No
8524	7:**1 X** vs. 7:**40 X**	-0.003333	-0.1473 to 0.1406	No
8525	7:**1 X** vs. 7:**50 X**	-0.001333	-0.1453 to 0.1426	No
8526	7:**1 X** vs. 8:**0 X**	-0.01333	-0.1573 to 0.1306	No
8527	7:**1 X** vs. 8:**1 X**	0.0	-0.1440 to 0.1440	No
8528	7:**1 X** vs. 8:**10 X**	-0.007333	-0.1513 to 0.1366	No
8529	7:**1 X** vs. 8:**40 X**	-0.008000	-0.1520 to 0.1360	No
8530	7:**1 X** vs. 8:**50 X**	-0.0050	-0.1490 to 0.1390	No
8531	7:**1 X** vs. 9:**0 X**	-0.02133	-0.1653 to 0.1226	No
8532	7:**1 X** vs. 9:**1 X**	-0.02933	-0.1733 to 0.1146	No
8533	7:**1 X** vs. 9:**10 X**	-0.03033	-0.1743 to 0.1136	No
8534	7:**1 X** vs. 9:**40 X**	-0.01267	-0.1566 to 0.1313	No
8535	7:**1 X** vs. 9:**50 X**	-0.006000	-0.1500 to 0.1380	No
8536	7:**1 X** vs. 10:**0 X**	-0.0230	-0.1670 to 0.1210	No
8537	7:**1 X** vs. 10:**1 X**	-0.0300	-0.1740 to 0.1140	No
8538	7:**1 X** vs. 10:**10 X**	-0.03267	-0.1766 to 0.1113	No
8539	7:**1 X** vs. 10:**40 X**	-0.02233	-0.1663 to 0.1216	No
8540	7:**1 X** vs. 10:**50 X**	-0.0090	-0.1530 to 0.1350	No
8541	7:**1 X** vs. 11:**0 X**	-0.03267	-0.1766 to 0.1113	No
8542	7:**1 X** vs. 11:**1 X**	-0.03433	-0.1783 to 0.1096	No
8543	7:**1 X** vs. 11:**10 X**	-0.03467	-0.1786 to 0.1093	No
8544	7:**1 X** vs. 11:**40 X**	-0.02633	-0.1703 to 0.1176	No
8545	7:**1 X** vs. 11:**50 X**	-0.01433	-0.1583 to 0.1296	No
8546	7:**1 X** vs. 12:**0 X**	-0.03333	-0.1773 to 0.1106	No
8547	7:**1 X** vs. 12:**1 X**	-0.01567	-0.1596 to 0.1283	No
8548	7:**1 X** vs. 12:**10 X**	-0.03333	-0.1773 to 0.1106	No
8549	7:**1 X** vs. 12:**40 X**	-0.01967	-0.1636 to 0.1243	No
8550	7:**1 X** vs. 12:**50 X**	-0.0230	-0.1670 to 0.1210	No

	2way ANOVA<br>Multiple comparisons	A<br>Data Set-A<br>Y	B<br>Data Set-B<br>Y	C<br>Data Set-C<br>Y
8551	7:**1 X** vs. 13:**0 X**	-0.02733	-0.1713 to 0.1166	No
8552	7:**1 X** vs. 13:**1 X**	-0.01467	-0.1586 to 0.1293	No
8553	7:**1 X** vs. 13:**10 X**	-0.03667	-0.1806 to 0.1073	No
8554	7:**1 X** vs. 13:**40 X**	-0.0330	-0.1770 to 0.1110	No
8555	7:**1 X** vs. 13:**50 X**	-0.0340	-0.1780 to 0.1100	No
8556	7:**1 X** vs. 14:**0 X**	-0.0360	-0.1800 to 0.1080	No
8557	7:**1 X** vs. 14:**1 X**	-0.02767	-0.1716 to 0.1163	No
8558	7:**1 X** vs. 14:**10 X**	-0.04167	-0.1856 to 0.1023	No
8559	7:**1 X** vs. 14:**40 X**	-0.03833	-0.1823 to 0.1056	No
8560	7:**1 X** vs. 14:**50 X**	-0.04233	-0.1863 to 0.1016	No
8561	7:**1 X** vs. 15:**0 X**	-0.04833	-0.1923 to 0.09563	No
8562	7:**1 X** vs. 15:**1 X**	-0.02833	-0.1723 to 0.1156	No
8563	7:**1 X** vs. 15:**10 X**	-0.05567	-0.1996 to 0.08829	No
8564	7:**1 X** vs. 15:**40 X**	-0.0380	-0.1820 to 0.1060	No
8565	7:**1 X** vs. 15:**50 X**	-0.05567	-0.1996 to 0.08829	No
8566	7:**1 X** vs. 16:**0 X**	-0.1407	-0.2846 to 0.003293	No
8567	7:**1 X** vs. 16:**1 X**	-0.03167	-0.1756 to 0.1123	No
8568	7:**1 X** vs. 16:**10 X**	-0.05733	-0.2013 to 0.08663	No
8569	7:**1 X** vs. 16:**40 X**	-0.07667	-0.2206 to 0.06729	No
8570	7:**1 X** vs. 16:**50 X**	-0.1190	-0.2630 to 0.02496	No
8571	7:**1 X** vs. 17:**0 X**	-0.1933	-0.3373 to -0.04937	Yes
8572	7:**1 X** vs. 17:**1 X**	-0.1077	-0.2516 to 0.03629	No
8573	7:**1 X** vs. 17:**10 X**	-0.1643	-0.3083 to -0.02037	Yes
8574	7:**1 X** vs. 17:**40 X**	-0.08767	-0.2316 to 0.05629	No
8575	7:**1 X** vs. 17:**50 X**	-0.1330	-0.2770 to 0.01096	No
8576	7:**1 X** vs. 18:**0 X**	-0.2420	-0.3860 to -0.09804	Yes
8577	7:**1 X** vs. 18:**1 X**	-0.1503	-0.2943 to -0.006374	Yes
8578	7:**1 X** vs. 18:**10 X**	-0.2427	-0.3866 to -0.09871	Yes
8579	7:**1 X** vs. 18:**40 X**	-0.0920	-0.2360 to 0.05196	No
8580	7:**1 X** vs. 18:**50 X**	-0.1370	-0.2810 to 0.006960	No
8581	7:**1 X** vs. 19:**0 X**	-0.2770	-0.4210 to -0.1330	Yes
8582	7:**1 X** vs. 19:**1 X**	-0.2650	-0.4090 to -0.1210	Yes
8583	7:**1 X** vs. 19:**10 X**	-0.2627	-0.4066 to -0.1187	Yes
8584	7:**1 X** vs. 19:**40 X**	-0.1207	-0.2646 to 0.02329	No
8585	7:**1 X** vs. 19:**50 X**	-0.1317	-0.2756 to 0.01229	No
8586	7:**1 X** vs. 20:**0 X**	-0.3567	-0.5006 to -0.2127	Yes
8587	7:**1 X** vs. 20:**1 X**	-0.1760	-0.3200 to -0.03204	Yes
8588	7:**1 X** vs. 20:**10 X**	-0.2650	-0.4090 to -0.1210	Yes
8589	7:**1 X** vs. 20:**40 X**	-0.1323	-0.2763 to 0.01163	No
8590	7:**1 X** vs. 20:**50 X**	-0.1763	-0.3203 to -0.03237	Yes
8591	7:**1 X** vs. 21:**0 X**	-0.3800	-0.5240 to -0.2360	Yes
8592	7:**1 X** vs. 21:**1 X**	-0.3330	-0.4770 to -0.1890	Yes
8593	7:**1 X** vs. 21:**10 X**	-0.3300	-0.4740 to -0.1860	Yes
8594	7:**1 X** vs. 21:**40 X**	-0.2053	-0.3493 to -0.06137	Yes
8595	7:**1 X** vs. 21:**50 X**	-0.2957	-0.4396 to -0.1517	Yes

2way ANOVA<br>Multiple comparisons		A<br>Data Set-A<br>Y	B<br>Data Set-B<br>Y	C<br>Data Set-C<br>Y
8596	7:**1 X** vs. 22:**0 X**	-0.4217	-0.5656 to -0.2777	Yes
8597	7:**1 X** vs. 22:**1 X**	-0.4163	-0.5603 to -0.2724	Yes
8598	7:**1 X** vs. 22:**10 X**	-0.4117	-0.5556 to -0.2677	Yes
8599	7:**1 X** vs. 22:**40 X**	-0.3120	-0.4560 to -0.1680	Yes
8600	7:**1 X** vs. 22:**50 X**	-0.3747	-0.5186 to -0.2307	Yes
8601	7:**1 X** vs. 23:**0 X**	-0.4870	-0.6310 to -0.3430	Yes
8602	7:**1 X** vs. 23:**1 X**	-0.6767	-0.8376 to -0.5157	Yes
8603	7:**1 X** vs. 23:**10 X**	-0.4263	-0.5703 to -0.2824	Yes
8604	7:**1 X** vs. 23:**40 X**	-0.3067	-0.4676 to -0.1457	Yes
8605	7:**1 X** vs. 23:**50 X**	-0.3407	-0.4846 to -0.1967	Yes
8606	7:**1 X** vs. 24:**0 X**	-0.6043	-0.7483 to -0.4604	Yes
8607	7:**1 X** vs. 24:**1 X**	-0.7607	-0.9216 to -0.5997	Yes
8608	7:**1 X** vs. 24:**10 X**	-0.5882	-0.7491 to -0.4272	Yes
8609	7:**1 X** vs. 24:**40 X**	-0.3230	-0.4670 to -0.1790	Yes
8610	7:**1 X** vs. 24:**50 X**	-0.2447	-0.3886 to -0.1007	Yes
8611	7:**1 X** vs. 25:**0 X**	-0.9417	-1.103 to -0.7807	Yes
8612	7:**1 X** vs. 25:**1 X**	-1.131	-1.292 to -0.9702	Yes
8613	7:**1 X** vs. 25:**10 X**	-0.4927	-0.6963 to -0.2891	Yes
8614	7:**1 X** vs. 25:**40 X**	-0.3250	-0.4690 to -0.1810	Yes
8615	7:**1 X** vs. 25:**50 X**	-0.2993	-0.4433 to -0.1554	Yes
8616	7:**1 X** vs. 26:**0 X**	-1.154	-1.315 to -0.9932	Yes
8617	7:**1 X** vs. 26:**1 X**	-1.267	-1.428 to -1.106	Yes
8618	7:**1 X** vs. 26:**10 X**	-0.9537	-1.115 to -0.7927	Yes
8619	7:**1 X** vs. 26:**40 X**	-0.3993	-0.5433 to -0.2554	Yes
8620	7:**1 X** vs. 26:**50 X**	-0.2597	-0.4036 to -0.1157	Yes
8621	7:**1 X** vs. 27:**0 X**	-1.342	-1.486 to -1.198	Yes
8622	7:**1 X** vs. 27:**1 X**	-1.433	-1.594 to -1.272	Yes
8623	7:**1 X** vs. 27:**10 X**	-1.245	-1.406 to -1.084	Yes
8624	7:**1 X** vs. 27:**40 X**	-0.3993	-0.5433 to -0.2554	Yes
8625	7:**1 X** vs. 27:**50 X**	-0.2590	-0.4030 to -0.1150	Yes
8626	7:**1 X** vs. 28:**0 X**	-1.291	-1.452 to -1.130	Yes
8627	7:**1 X** vs. 28:**1 X**	-1.332	-1.476 to -1.188	Yes
8628	7:**1 X** vs. 28:**10 X**	-1.134	-1.295 to -0.9727	Yes
8629	7:**1 X** vs. 28:**40 X**	-0.4117	-0.5556 to -0.2677	Yes
8630	7:**1 X** vs. 28:**50 X**	-0.3070	-0.4510 to -0.1630	Yes
8631	7:**1 X** vs. 29:**0 X**	-1.773	-1.934 to -1.612	Yes
8632	7:**1 X** vs. 29:**1 X**	-1.858	-2.019 to -1.697	Yes
8633	7:**1 X** vs. 29:**10 X**	-1.072	-1.233 to -0.9112	Yes
8634	7:**1 X** vs. 29:**40 X**	-0.5197	-0.6636 to -0.3757	Yes
8635	7:**1 X** vs. 29:**50 X**	-0.3083	-0.4523 to -0.1644	Yes
8636	7:**1 X** vs. 30:**0 X**	-1.487	-1.648 to -1.326	Yes
8637	7:**1 X** vs. 30:**1 X**	-1.809	-1.970 to -1.648	Yes
8638	7:**1 X** vs. 30:**10 X**	-1.147	-1.308 to -0.9857	Yes
8639	7:**1 X** vs. 30:**40 X**	-0.4467	-0.5906 to -0.3027	Yes
8640	7:**1 X** vs. 30:**50 X**	-0.3607	-0.5046 to -0.2167	Yes

	2way ANOVA Multiple comparisons	A Data Set-A Y	B Data Set-B Y	C Data Set-C Y
8641	7:1 X vs. 31:0 X	-1.805	-1.966 to -1.644	Yes
8642	7:1 X vs. 31:1 X	-1.796	-1.957 to -1.635	Yes
8643	7:1 X vs. 31:10 X	-1.278	-1.439 to -1.117	Yes
8644	7:1 X vs. 31:40 X	-0.5383	-0.6823 to -0.3944	Yes
8645	7:1 X vs. 31:50 X	-0.3397	-0.4836 to -0.1957	Yes
8646	7:1 X vs. 32:0 X	-2.397	-2.541 to -2.253	Yes
8647	7:1 X vs. 32:1 X	-1.853	-2.014 to -1.692	Yes
8648	7:1 X vs. 32:10 X	-1.709	-1.853 to -1.565	Yes
8649	7:1 X vs. 32:40 X	-0.5347	-0.6786 to -0.3907	Yes
8650	7:1 X vs. 32:50 X	-0.3393	-0.4833 to -0.1954	Yes
8651	7:1 X vs. 33:0 X	-2.400	-2.544 to -2.256	Yes
8652	7:1 X vs. 33:1 X	-2.215	-2.359 to -2.071	Yes
8653	7:1 X vs. 33:10 X	-1.875	-2.019 to -1.731	Yes
8654	7:1 X vs. 33:40 X	-0.5533	-0.6973 to -0.4094	Yes
8655	7:1 X vs. 33:50 X	-0.3543	-0.4983 to -0.2104	Yes
8656	7:1 X vs. 34:0 X	-2.325	-2.469 to -2.181	Yes
8657	7:1 X vs. 34:1 X	-2.332	-2.476 to -2.188	Yes
8658	7:1 X vs. 34:10 X	-1.966	-2.110 to -1.822	Yes
8659	7:1 X vs. 34:40 X	-0.5653	-0.7093 to -0.4214	Yes
8660	7:1 X vs. 34:50 X	-0.5033	-0.6473 to -0.3594	Yes
8661	7:1 X vs. 35:0 X	-2.674	-2.818 to -2.530	Yes
8662	7:1 X vs. 35:1 X	-2.439	-2.583 to -2.295	Yes
8663	7:1 X vs. 35:10 X	-1.927	-2.071 to -1.783	Yes
8664	7:1 X vs. 35:40 X	-0.5743	-0.7183 to -0.4304	Yes
8665	7:1 X vs. 35:50 X	-0.5160	-0.6600 to -0.3720	Yes
8666	7:1 X vs. 36:0 X	-2.880	-3.041 to -2.719	Yes
8667	7:1 X vs. 36:1 X	-2.576	-2.720 to -2.432	Yes
8668	7:1 X vs. 36:10 X	-1.933	-2.077 to -1.789	Yes
8669	7:1 X vs. 36:40 X	-0.6297	-0.7736 to -0.4857	Yes
8670	7:1 X vs. 36:50 X	-0.4793	-0.6233 to -0.3354	Yes
8671	7:1 X vs. 37:0 X	-3.264	-3.408 to -3.120	Yes
8672	7:1 X vs. 37:1 X	-2.646	-2.790 to -2.502	Yes
8673	7:1 X vs. 37:10 X	-1.935	-2.079 to -1.791	Yes
8674	7:1 X vs. 37:40 X	-0.6670	-0.8110 to -0.5230	Yes
8675	7:1 X vs. 37:50 X	-0.4880	-0.6320 to -0.3440	Yes
8676	7:1 X vs. 38:0 X	-3.209	-3.353 to -3.065	Yes
8677	7:1 X vs. 38:1 X	-2.765	-2.909 to -2.621	Yes
8678	7:1 X vs. 38:10 X	-2.210	-2.354 to -2.066	Yes
8679	7:1 X vs. 38:40 X	-0.6713	-0.8153 to -0.5274	Yes
8680	7:1 X vs. 38:50 X	-0.4920	-0.6360 to -0.3480	Yes
8681	7:1 X vs. 39:0 X	-3.440	-3.584 to -3.296	Yes
8682	7:1 X vs. 39:1 X	-3.331	-3.475 to -3.187	Yes
8683	7:1 X vs. 39:10 X	-2.355	-2.499 to -2.211	Yes
8684	7:1 X vs. 39:40 X	-0.6773	-0.8213 to -0.5334	Yes
8685	7:1 X vs. 39:50 X	-0.5230	-0.6670 to -0.3790	Yes

	2way ANOVA<br>Multiple comparisons	A<br>Data Set-A<br>Y	B<br>Data Set-B<br>Y	C<br>Data Set-C<br>Y
8686	7:1 X vs. 40:0 X	-3.481	-3.625 to -3.337	Yes
8687	7:1 X vs. 40:1 X	-3.407	-3.551 to -3.263	Yes
8688	7:1 X vs. 40:10 X	-2.611	-2.755 to -2.467	Yes
8689	7:1 X vs. 40:40 X	-0.6820	-0.8260 to -0.5380	Yes
8690	7:1 X vs. 40:50 X	-0.5293	-0.6733 to -0.3854	Yes
8691	7:1 X vs. 41:0 X	-3.522	-3.666 to -3.378	Yes
8692	7:1 X vs. 41:1 X	-3.521	-3.665 to -3.377	Yes
8693	7:1 X vs. 41:10 X	-2.665	-2.809 to -2.521	Yes
8694	7:1 X vs. 41:40 X	-0.6293	-0.7733 to -0.4854	Yes
8695	7:1 X vs. 41:50 X	-0.5420	-0.6860 to -0.3980	Yes
8696	7:1 X vs. 42:0 X	-3.663	-3.807 to -3.519	Yes
8697	7:1 X vs. 42:1 X	-3.683	-3.827 to -3.539	Yes
8698	7:1 X vs. 42:10 X	-2.665	-2.809 to -2.521	Yes
8699	7:1 X vs. 42:40 X	-0.6420	-0.7860 to -0.4980	Yes
8700	7:1 X vs. 42:50 X	-0.5453	-0.6893 to -0.4014	Yes
8701	7:1 X vs. 43:0 X	-3.753	-3.897 to -3.609	Yes
8702	7:1 X vs. 43:1 X	-3.668	-3.812 to -3.524	Yes
8703	7:1 X vs. 43:10 X	-3.383	-3.527 to -3.239	Yes
8704	7:1 X vs. 43:40 X	-0.6403	-0.7843 to -0.4964	Yes
8705	7:1 X vs. 43:50 X	-0.5457	-0.6896 to -0.4017	Yes
8706	7:1 X vs. 44:0 X	-3.777	-3.921 to -3.633	Yes
8707	7:1 X vs. 44:1 X	-3.667	-3.811 to -3.523	Yes
8708	7:1 X vs. 44:10 X	-3.447	-3.591 to -3.303	Yes
8709	7:1 X vs. 44:40 X	-0.6423	-0.7863 to -0.4984	Yes
8710	7:1 X vs. 44:50 X	-0.4780	-0.6220 to -0.3340	Yes
8711	7:1 X vs. 45:0 X	-3.642	-3.786 to -3.498	Yes
8712	7:1 X vs. 45:1 X	-3.444	-3.588 to -3.300	Yes
8713	7:1 X vs. 45:10 X	-3.326	-3.470 to -3.182	Yes
8714	7:1 X vs. 45:40 X	-0.6437	-0.7876 to -0.4997	Yes
8715	7:1 X vs. 45:50 X	-0.4750	-0.6190 to -0.3310	Yes
8716	7:1 X vs. 46:0 X	-3.446	-3.590 to -3.302	Yes
8717	7:1 X vs. 46:1 X	-3.413	-3.557 to -3.269	Yes
8718	7:1 X vs. 46:10 X	-3.321	-3.465 to -3.177	Yes
8719	7:1 X vs. 46:40 X	-0.6573	-0.8013 to -0.5134	Yes
8720	7:1 X vs. 46:50 X	-0.4680	-0.6120 to -0.3240	Yes
8721	7:1 X vs. 47:0 X	-3.422	-3.566 to -3.278	Yes
8722	7:1 X vs. 47:1 X	-3.326	-3.470 to -3.182	Yes
8723	7:1 X vs. 47:10 X	-3.307	-3.451 to -3.163	Yes
8724	7:1 X vs. 47:40 X	-0.6673	-0.8113 to -0.5234	Yes
8725	7:1 X vs. 47:50 X	-0.4260	-0.5700 to -0.2820	Yes
8726	7:1 X vs. 48:0 X	-3.409	-3.570 to -3.248	Yes
8727	7:1 X vs. 48:1 X	-3.313	-3.457 to -3.169	Yes
8728	7:1 X vs. 48:10 X	-3.307	-3.451 to -3.163	Yes
8729	7:1 X vs. 48:40 X	-0.6737	-0.8176 to -0.5297	Yes
8730	7:1 X vs. 48:50 X	-0.4333	-0.5773 to -0.2894	Yes

	2way ANOVA<br>Multiple comparisons	A<br>Data Set-A<br>Y	B<br>Data Set-B<br>Y	C<br>Data Set-C<br>Y
8731	7:1 X vs. 49:0 X	-3.409	-3.553 to -3.265	Yes
8732	7:1 X vs. 49:1 X	-3.313	-3.457 to -3.169	Yes
8733	7:1 X vs. 49:10 X	-3.307	-3.451 to -3.163	Yes
8734	7:1 X vs. 49:40 X	-0.6780	-0.8220 to -0.5340	Yes
8735	7:1 X vs. 49:50 X	-0.4327	-0.5766 to -0.2887	Yes
8736	7:1 X vs. 50:0 X	-3.409	-3.553 to -3.265	Yes
8737	7:1 X vs. 50:1 X	-3.313	-3.457 to -3.169	Yes
8738	7:1 X vs. 50:10 X	-3.307	-3.451 to -3.163	Yes
8739	7:1 X vs. 50:40 X	-0.6857	-0.8296 to -0.5417	Yes
8740	7:1 X vs. 50:50 X	-0.4323	-0.5763 to -0.2884	Yes
8741	7:10 X vs. 7:40 X	0.008667	-0.1353 to 0.1526	No
8742	7:10 X vs. 7:50 X	0.01067	-0.1333 to 0.1546	No
8743	7:10 X vs. 8:0 X	-0.001333	-0.1453 to 0.1426	No
8744	7:10 X vs. 8:1 X	0.0120	-0.1320 to 0.1560	No
8745	7:10 X vs. 8:10 X	0.004667	-0.1393 to 0.1486	No
8746	7:10 X vs. 8:40 X	0.0040	-0.1400 to 0.1480	No
8747	7:10 X vs. 8:50 X	0.007000	-0.1370 to 0.1510	No
8748	7:10 X vs. 9:0 X	-0.009333	-0.1533 to 0.1346	No
8749	7:10 X vs. 9:1 X	-0.01733	-0.1613 to 0.1266	No
8750	7:10 X vs. 9:10 X	-0.01833	-0.1623 to 0.1256	No
8751	7:10 X vs. 9:40 X	-0.0006667	-0.1446 to 0.1433	No
8752	7:10 X vs. 9:50 X	0.006000	-0.1380 to 0.1500	No
8753	7:10 X vs. 10:0 X	-0.0110	-0.1550 to 0.1330	No
8754	7:10 X vs. 10:1 X	-0.0180	-0.1620 to 0.1260	No
8755	7:10 X vs. 10:10 X	-0.02067	-0.1646 to 0.1233	No
8756	7:10 X vs. 10:40 X	-0.01033	-0.1543 to 0.1336	No
8757	7:10 X vs. 10:50 X	0.0030	-0.1410 to 0.1470	No
8758	7:10 X vs. 11:0 X	-0.02067	-0.1646 to 0.1233	No
8759	7:10 X vs. 11:1 X	-0.02233	-0.1663 to 0.1216	No
8760	7:10 X vs. 11:10 X	-0.02267	-0.1666 to 0.1213	No
8761	7:10 X vs. 11:40 X	-0.01433	-0.1583 to 0.1296	No
8762	7:10 X vs. 11:50 X	-0.002333	-0.1463 to 0.1416	No
8763	7:10 X vs. 12:0 X	-0.02133	-0.1653 to 0.1226	No
8764	7:10 X vs. 12:1 X	-0.003667	-0.1476 to 0.1403	No
8765	7:10 X vs. 12:10 X	-0.02133	-0.1653 to 0.1226	No
8766	7:10 X vs. 12:40 X	-0.007667	-0.1516 to 0.1363	No
8767	7:10 X vs. 12:50 X	-0.0110	-0.1550 to 0.1330	No
8768	7:10 X vs. 13:0 X	-0.01533	-0.1593 to 0.1286	No
8769	7:10 X vs. 13:1 X	-0.002667	-0.1466 to 0.1413	No
8770	7:10 X vs. 13:10 X	-0.02467	-0.1686 to 0.1193	No
8771	7:10 X vs. 13:40 X	-0.0210	-0.1650 to 0.1230	No
8772	7:10 X vs. 13:50 X	-0.0220	-0.1660 to 0.1220	No
8773	7:10 X vs. 14:0 X	-0.0240	-0.1680 to 0.1200	No
8774	7:10 X vs. 14:1 X	-0.01567	-0.1596 to 0.1283	No
8775	7:10 X vs. 14:10 X	-0.02967	-0.1736 to 0.1143	No

	2way ANOVA<br>Multiple comparisons	A<br>Data Set-A<br>Y	B<br>Data Set-B<br>Y	C<br>Data Set-C<br>Y
8776	7:**10 X** vs. 14:**40 X**	-0.02633	-0.1703 to 0.1176	No
8777	7:**10 X** vs. 14:**50 X**	-0.03033	-0.1743 to 0.1136	No
8778	7:**10 X** vs. 15:**0 X**	-0.03633	-0.1803 to 0.1076	No
8779	7:**10 X** vs. 15:**1 X**	-0.01633	-0.1603 to 0.1276	No
8780	7:**10 X** vs. 15:**10 X**	-0.04367	-0.1876 to 0.1003	No
8781	7:**10 X** vs. 15:**40 X**	-0.0260	-0.1700 to 0.1180	No
8782	7:**10 X** vs. 15:**50 X**	-0.04367	-0.1876 to 0.1003	No
8783	7:**10 X** vs. 16:**0 X**	-0.1287	-0.2726 to 0.01529	No
8784	7:**10 X** vs. 16:**1 X**	-0.01967	-0.1636 to 0.1243	No
8785	7:**10 X** vs. 16:**10 X**	-0.04533	-0.1893 to 0.09863	No
8786	7:**10 X** vs. 16:**40 X**	-0.06467	-0.2086 to 0.07929	No
8787	7:**10 X** vs. 16:**50 X**	-0.1070	-0.2510 to 0.03696	No
8788	7:**10 X** vs. 17:**0 X**	-0.1813	-0.3253 to -0.03737	Yes
8789	7:**10 X** vs. 17:**1 X**	-0.09567	-0.2396 to 0.04829	No
8790	7:**10 X** vs. 17:**10 X**	-0.1523	-0.2963 to -0.008374	Yes
8791	7:**10 X** vs. 17:**40 X**	-0.07567	-0.2196 to 0.06829	No
8792	7:**10 X** vs. 17:**50 X**	-0.1210	-0.2650 to 0.02296	No
8793	7:**10 X** vs. 18:**0 X**	-0.2300	-0.3740 to -0.08604	Yes
8794	7:**10 X** vs. 18:**1 X**	-0.1383	-0.2823 to 0.005626	No
8795	7:**10 X** vs. 18:**10 X**	-0.2307	-0.3746 to -0.08671	Yes
8796	7:**10 X** vs. 18:**40 X**	-0.0800	-0.2240 to 0.06396	No
8797	7:**10 X** vs. 18:**50 X**	-0.1250	-0.2690 to 0.01896	No
8798	7:**10 X** vs. 19:**0 X**	-0.2650	-0.4090 to -0.1210	Yes
8799	7:**10 X** vs. 19:**1 X**	-0.2530	-0.3970 to -0.1090	Yes
8800	7:**10 X** vs. 19:**10 X**	-0.2507	-0.3946 to -0.1067	Yes
8801	7:**10 X** vs. 19:**40 X**	-0.1087	-0.2526 to 0.03529	No
8802	7:**10 X** vs. 19:**50 X**	-0.1197	-0.2636 to 0.02429	No
8803	7:**10 X** vs. 20:**0 X**	-0.3447	-0.4886 to -0.2007	Yes
8804	7:**10 X** vs. 20:**1 X**	-0.1640	-0.3080 to -0.02004	Yes
8805	7:**10 X** vs. 20:**10 X**	-0.2530	-0.3970 to -0.1090	Yes
8806	7:**10 X** vs. 20:**40 X**	-0.1203	-0.2643 to 0.02363	No
8807	7:**10 X** vs. 20:**50 X**	-0.1643	-0.3083 to -0.02037	Yes
8808	7:**10 X** vs. 21:**0 X**	-0.3680	-0.5120 to -0.2240	Yes
8809	7:**10 X** vs. 21:**1 X**	-0.3210	-0.4650 to -0.1770	Yes
8810	7:**10 X** vs. 21:**10 X**	-0.3180	-0.4620 to -0.1740	Yes
8811	7:**10 X** vs. 21:**40 X**	-0.1933	-0.3373 to -0.04937	Yes
8812	7:**10 X** vs. 21:**50 X**	-0.2837	-0.4276 to -0.1397	Yes
8813	7:**10 X** vs. 22:**0 X**	-0.4097	-0.5536 to -0.2657	Yes
8814	7:**10 X** vs. 22:**1 X**	-0.4043	-0.5483 to -0.2604	Yes
8815	7:**10 X** vs. 22:**10 X**	-0.3997	-0.5436 to -0.2557	Yes
8816	7:**10 X** vs. 22:**40 X**	-0.3000	-0.4440 to -0.1560	Yes
8817	7:**10 X** vs. 22:**50 X**	-0.3627	-0.5066 to -0.2187	Yes
8818	7:**10 X** vs. 23:**0 X**	-0.4750	-0.6190 to -0.3310	Yes
8819	7:**10 X** vs. 23:**1 X**	-0.6647	-0.8256 to -0.5037	Yes
8820	7:**10 X** vs. 23:**10 X**	-0.4143	-0.5583 to -0.2704	Yes

	2way ANOVA<br>Multiple comparisons	A<br>Data Set-A<br>Y	B<br>Data Set-B<br>Y	C<br>Data Set-C<br>Y
8821	7:10 X vs. 23:40 X	-0.2947	-0.4556 to -0.1337	Yes
8822	7:10 X vs. 23:50 X	-0.3287	-0.4726 to -0.1847	Yes
8823	7:10 X vs. 24:0 X	-0.5923	-0.7363 to -0.4484	Yes
8824	7:10 X vs. 24:1 X	-0.7487	-0.9096 to -0.5877	Yes
8825	7:10 X vs. 24:10 X	-0.5762	-0.7371 to -0.4152	Yes
8826	7:10 X vs. 24:40 X	-0.3110	-0.4550 to -0.1670	Yes
8827	7:10 X vs. 24:50 X	-0.2327	-0.3766 to -0.08871	Yes
8828	7:10 X vs. 25:0 X	-0.9297	-1.091 to -0.7687	Yes
8829	7:10 X vs. 25:1 X	-1.119	-1.280 to -0.9582	Yes
8830	7:10 X vs. 25:10 X	-0.4807	-0.6843 to -0.2771	Yes
8831	7:10 X vs. 25:40 X	-0.3130	-0.4570 to -0.1690	Yes
8832	7:10 X vs. 25:50 X	-0.2873	-0.4313 to -0.1434	Yes
8833	7:10 X vs. 26:0 X	-1.142	-1.303 to -0.9812	Yes
8834	7:10 X vs. 26:1 X	-1.255	-1.416 to -1.094	Yes
8835	7:10 X vs. 26:10 X	-0.9417	-1.103 to -0.7807	Yes
8836	7:10 X vs. 26:40 X	-0.3873	-0.5313 to -0.2434	Yes
8837	7:10 X vs. 26:50 X	-0.2477	-0.3916 to -0.1037	Yes
8838	7:10 X vs. 27:0 X	-1.330	-1.474 to -1.186	Yes
8839	7:10 X vs. 27:1 X	-1.421	-1.582 to -1.260	Yes
8840	7:10 X vs. 27:10 X	-1.233	-1.394 to -1.072	Yes
8841	7:10 X vs. 27:40 X	-0.3873	-0.5313 to -0.2434	Yes
8842	7:10 X vs. 27:50 X	-0.2470	-0.3910 to -0.1030	Yes
8843	7:10 X vs. 28:0 X	-1.279	-1.440 to -1.118	Yes
8844	7:10 X vs. 28:1 X	-1.320	-1.464 to -1.176	Yes
8845	7:10 X vs. 28:10 X	-1.122	-1.283 to -0.9607	Yes
8846	7:10 X vs. 28:40 X	-0.3997	-0.5436 to -0.2557	Yes
8847	7:10 X vs. 28:50 X	-0.2950	-0.4390 to -0.1510	Yes
8848	7:10 X vs. 29:0 X	-1.761	-1.922 to -1.600	Yes
8849	7:10 X vs. 29:1 X	-1.846	-2.007 to -1.685	Yes
8850	7:10 X vs. 29:10 X	-1.060	-1.221 to -0.8992	Yes
8851	7:10 X vs. 29:40 X	-0.5077	-0.6516 to -0.3637	Yes
8852	7:10 X vs. 29:50 X	-0.2963	-0.4403 to -0.1524	Yes
8853	7:10 X vs. 30:0 X	-1.475	-1.636 to -1.314	Yes
8854	7:10 X vs. 30:1 X	-1.797	-1.958 to -1.636	Yes
8855	7:10 X vs. 30:10 X	-1.135	-1.296 to -0.9737	Yes
8856	7:10 X vs. 30:40 X	-0.4347	-0.5786 to -0.2907	Yes
8857	7:10 X vs. 30:50 X	-0.3487	-0.4926 to -0.2047	Yes
8858	7:10 X vs. 31:0 X	-1.793	-1.954 to -1.632	Yes
8859	7:10 X vs. 31:1 X	-1.784	-1.945 to -1.623	Yes
8860	7:10 X vs. 31:10 X	-1.266	-1.427 to -1.105	Yes
8861	7:10 X vs. 31:40 X	-0.5263	-0.6703 to -0.3824	Yes
8862	7:10 X vs. 31:50 X	-0.3277	-0.4716 to -0.1837	Yes
8863	7:10 X vs. 32:0 X	-2.385	-2.529 to -2.241	Yes
8864	7:10 X vs. 32:1 X	-1.841	-2.002 to -1.680	Yes
8865	7:10 X vs. 32:10 X	-1.697	-1.841 to -1.553	Yes

	2way ANOVA<br>Multiple comparisons	A<br>Data Set-A<br>Y	B<br>Data Set-B<br>Y	C<br>Data Set-C<br>Y
8866	7:**10 X** vs. 32:**40 X**	-0.5227	-0.6666 to -0.3787	Yes
8867	7:**10 X** vs. 32:**50 X**	-0.3273	-0.4713 to -0.1834	Yes
8868	7:**10 X** vs. 33:**0 X**	-2.388	-2.532 to -2.244	Yes
8869	7:**10 X** vs. 33:**1 X**	-2.203	-2.347 to -2.059	Yes
8870	7:**10 X** vs. 33:**10 X**	-1.863	-2.007 to -1.719	Yes
8871	7:**10 X** vs. 33:**40 X**	-0.5413	-0.6853 to -0.3974	Yes
8872	7:**10 X** vs. 33:**50 X**	-0.3423	-0.4863 to -0.1984	Yes
8873	7:**10 X** vs. 34:**0 X**	-2.313	-2.457 to -2.169	Yes
8874	7:**10 X** vs. 34:**1 X**	-2.320	-2.464 to -2.176	Yes
8875	7:**10 X** vs. 34:**10 X**	-1.954	-2.098 to -1.810	Yes
8876	7:**10 X** vs. 34:**40 X**	-0.5533	-0.6973 to -0.4094	Yes
8877	7:**10 X** vs. 34:**50 X**	-0.4913	-0.6353 to -0.3474	Yes
8878	7:**10 X** vs. 35:**0 X**	-2.662	-2.806 to -2.518	Yes
8879	7:**10 X** vs. 35:**1 X**	-2.427	-2.571 to -2.283	Yes
8880	7:**10 X** vs. 35:**10 X**	-1.915	-2.059 to -1.771	Yes
8881	7:**10 X** vs. 35:**40 X**	-0.5623	-0.7063 to -0.4184	Yes
8882	7:**10 X** vs. 35:**50 X**	-0.5040	-0.6480 to -0.3600	Yes
8883	7:**10 X** vs. 36:**0 X**	-2.868	-3.029 to -2.707	Yes
8884	7:**10 X** vs. 36:**1 X**	-2.564	-2.708 to -2.420	Yes
8885	7:**10 X** vs. 36:**10 X**	-1.921	-2.065 to -1.777	Yes
8886	7:**10 X** vs. 36:**40 X**	-0.6177	-0.7616 to -0.4737	Yes
8887	7:**10 X** vs. 36:**50 X**	-0.4673	-0.6113 to -0.3234	Yes
8888	7:**10 X** vs. 37:**0 X**	-3.252	-3.396 to -3.108	Yes
8889	7:**10 X** vs. 37:**1 X**	-2.634	-2.778 to -2.490	Yes
8890	7:**10 X** vs. 37:**10 X**	-1.923	-2.067 to -1.779	Yes
8891	7:**10 X** vs. 37:**40 X**	-0.6550	-0.7990 to -0.5110	Yes
8892	7:**10 X** vs. 37:**50 X**	-0.4760	-0.6200 to -0.3320	Yes
8893	7:**10 X** vs. 38:**0 X**	-3.197	-3.341 to -3.053	Yes
8894	7:**10 X** vs. 38:**1 X**	-2.753	-2.897 to -2.609	Yes
8895	7:**10 X** vs. 38:**10 X**	-2.198	-2.342 to -2.054	Yes
8896	7:**10 X** vs. 38:**40 X**	-0.6593	-0.8033 to -0.5154	Yes
8897	7:**10 X** vs. 38:**50 X**	-0.4800	-0.6240 to -0.3360	Yes
8898	7:**10 X** vs. 39:**0 X**	-3.428	-3.572 to -3.284	Yes
8899	7:**10 X** vs. 39:**1 X**	-3.319	-3.463 to -3.175	Yes
8900	7:**10 X** vs. 39:**10 X**	-2.343	-2.487 to -2.199	Yes
8901	7:**10 X** vs. 39:**40 X**	-0.6653	-0.8093 to -0.5214	Yes
8902	7:**10 X** vs. 39:**50 X**	-0.5110	-0.6550 to -0.3670	Yes
8903	7:**10 X** vs. 40:**0 X**	-3.469	-3.613 to -3.325	Yes
8904	7:**10 X** vs. 40:**1 X**	-3.395	-3.539 to -3.251	Yes
8905	7:**10 X** vs. 40:**10 X**	-2.599	-2.743 to -2.455	Yes
8906	7:**10 X** vs. 40:**40 X**	-0.6700	-0.8140 to -0.5260	Yes
8907	7:**10 X** vs. 40:**50 X**	-0.5173	-0.6613 to -0.3734	Yes
8908	7:**10 X** vs. 41:**0 X**	-3.510	-3.654 to -3.366	Yes
8909	7:**10 X** vs. 41:**1 X**	-3.509	-3.653 to -3.365	Yes
8910	7:**10 X** vs. 41:**10 X**	-2.653	-2.797 to -2.509	Yes

2way ANOVA<br>Multiple comparisons	A<br>Data Set-A<br>Y	B<br>Data Set-B<br>Y	C<br>Data Set-C<br>Y
8911 7:**10 X** vs. 41:**40 X**	-0.6173	-0.7613 to -0.4734	Yes
8912 7:**10 X** vs. 41:**50 X**	-0.5300	-0.6740 to -0.3860	Yes
8913 7:**10 X** vs. 42:**0 X**	-3.651	-3.795 to -3.507	Yes
8914 7:**10 X** vs. 42:**1 X**	-3.671	-3.815 to -3.527	Yes
8915 7:**10 X** vs. 42:**10 X**	-2.653	-2.797 to -2.509	Yes
8916 7:**10 X** vs. 42:**40 X**	-0.6300	-0.7740 to -0.4860	Yes
8917 7:**10 X** vs. 42:**50 X**	-0.5333	-0.6773 to -0.3894	Yes
8918 7:**10 X** vs. 43:**0 X**	-3.741	-3.885 to -3.597	Yes
8919 7:**10 X** vs. 43:**1 X**	-3.656	-3.800 to -3.512	Yes
8920 7:**10 X** vs. 43:**10 X**	-3.371	-3.515 to -3.227	Yes
8921 7:**10 X** vs. 43:**40 X**	-0.6283	-0.7723 to -0.4844	Yes
8922 7:**10 X** vs. 43:**50 X**	-0.5337	-0.6776 to -0.3897	Yes
8923 7:**10 X** vs. 44:**0 X**	-3.765	-3.909 to -3.621	Yes
8924 7:**10 X** vs. 44:**1 X**	-3.655	-3.799 to -3.511	Yes
8925 7:**10 X** vs. 44:**10 X**	-3.435	-3.579 to -3.291	Yes
8926 7:**10 X** vs. 44:**40 X**	-0.6303	-0.7743 to -0.4864	Yes
8927 7:**10 X** vs. 44:**50 X**	-0.4660	-0.6100 to -0.3220	Yes
8928 7:**10 X** vs. 45:**0 X**	-3.630	-3.774 to -3.486	Yes
8929 7:**10 X** vs. 45:**1 X**	-3.432	-3.576 to -3.288	Yes
8930 7:**10 X** vs. 45:**10 X**	-3.314	-3.458 to -3.170	Yes
8931 7:**10 X** vs. 45:**40 X**	-0.6317	-0.7756 to -0.4877	Yes
8932 7:**10 X** vs. 45:**50 X**	-0.4630	-0.6070 to -0.3190	Yes
8933 7:**10 X** vs. 46:**0 X**	-3.434	-3.578 to -3.290	Yes
8934 7:**10 X** vs. 46:**1 X**	-3.401	-3.545 to -3.257	Yes
8935 7:**10 X** vs. 46:**10 X**	-3.309	-3.453 to -3.165	Yes
8936 7:**10 X** vs. 46:**40 X**	-0.6453	-0.7893 to -0.5014	Yes
8937 7:**10 X** vs. 46:**50 X**	-0.4560	-0.6000 to -0.3120	Yes
8938 7:**10 X** vs. 47:**0 X**	-3.410	-3.554 to -3.266	Yes
8939 7:**10 X** vs. 47:**1 X**	-3.314	-3.458 to -3.170	Yes
8940 7:**10 X** vs. 47:**10 X**	-3.295	-3.439 to -3.151	Yes
8941 7:**10 X** vs. 47:**40 X**	-0.6553	-0.7993 to -0.5114	Yes
8942 7:**10 X** vs. 47:**50 X**	-0.4140	-0.5580 to -0.2700	Yes
8943 7:**10 X** vs. 48:**0 X**	-3.397	-3.558 to -3.236	Yes
8944 7:**10 X** vs. 48:**1 X**	-3.301	-3.445 to -3.157	Yes
8945 7:**10 X** vs. 48:**10 X**	-3.295	-3.439 to -3.151	Yes
8946 7:**10 X** vs. 48:**40 X**	-0.6617	-0.8056 to -0.5177	Yes
8947 7:**10 X** vs. 48:**50 X**	-0.4213	-0.5653 to -0.2774	Yes
8948 7:**10 X** vs. 49:**0 X**	-3.397	-3.541 to -3.253	Yes
8949 7:**10 X** vs. 49:**1 X**	-3.301	-3.445 to -3.157	Yes
8950 7:**10 X** vs. 49:**10 X**	-3.295	-3.439 to -3.151	Yes
8951 7:**10 X** vs. 49:**40 X**	-0.6660	-0.8100 to -0.5220	Yes
8952 7:**10 X** vs. 49:**50 X**	-0.4207	-0.5646 to -0.2767	Yes
8953 7:**10 X** vs. 50:**0 X**	-3.397	-3.541 to -3.253	Yes
8954 7:**10 X** vs. 50:**1 X**	-3.301	-3.445 to -3.157	Yes
8955 7:**10 X** vs. 50:**10 X**	-3.295	-3.439 to -3.151	Yes

	2way ANOVA Multiple comparisons	A Data Set-A Y	B Data Set-B Y	C Data Set-C Y
8956	7:**10 X** vs. 50:**40 X**	-0.6737	-0.8176 to -0.5297	Yes
8957	7:**10 X** vs. 50:**50 X**	-0.4203	-0.5643 to -0.2764	Yes
8958	7:**40 X** vs. 7:**50 X**	0.0020	-0.1420 to 0.1460	No
8959	7:**40 X** vs. 8:**0 X**	-0.0100	-0.1540 to 0.1340	No
8960	7:**40 X** vs. 8:**1 X**	0.003333	-0.1406 to 0.1473	No
8961	7:**40 X** vs. 8:**10 X**	-0.004000	-0.1480 to 0.1400	No
8962	7:**40 X** vs. 8:**40 X**	-0.004667	-0.1486 to 0.1393	No
8963	7:**40 X** vs. 8:**50 X**	-0.001667	-0.1456 to 0.1423	No
8964	7:**40 X** vs. 9:**0 X**	-0.0180	-0.1620 to 0.1260	No
8965	7:**40 X** vs. 9:**1 X**	-0.0260	-0.1700 to 0.1180	No
8966	7:**40 X** vs. 9:**10 X**	-0.0270	-0.1710 to 0.1170	No
8967	7:**40 X** vs. 9:**40 X**	-0.009333	-0.1533 to 0.1346	No
8968	7:**40 X** vs. 9:**50 X**	-0.002667	-0.1466 to 0.1413	No
8969	7:**40 X** vs. 10:**0 X**	-0.01967	-0.1636 to 0.1243	No
8970	7:**40 X** vs. 10:**1 X**	-0.02667	-0.1706 to 0.1173	No
8971	7:**40 X** vs. 10:**10 X**	-0.02933	-0.1733 to 0.1146	No
8972	7:**40 X** vs. 10:**40 X**	-0.0190	-0.1630 to 0.1250	No
8973	7:**40 X** vs. 10:**50 X**	-0.005667	-0.1496 to 0.1383	No
8974	7:**40 X** vs. 11:**0 X**	-0.02933	-0.1733 to 0.1146	No
8975	7:**40 X** vs. 11:**1 X**	-0.0310	-0.1750 to 0.1130	No
8976	7:**40 X** vs. 11:**10 X**	-0.03133	-0.1753 to 0.1126	No
8977	7:**40 X** vs. 11:**40 X**	-0.0230	-0.1670 to 0.1210	No
8978	7:**40 X** vs. 11:**50 X**	-0.0110	-0.1550 to 0.1330	No
8979	7:**40 X** vs. 12:**0 X**	-0.0300	-0.1740 to 0.1140	No
8980	7:**40 X** vs. 12:**1 X**	-0.01233	-0.1563 to 0.1316	No
8981	7:**40 X** vs. 12:**10 X**	-0.0300	-0.1740 to 0.1140	No
8982	7:**40 X** vs. 12:**40 X**	-0.01633	-0.1603 to 0.1276	No
8983	7:**40 X** vs. 12:**50 X**	-0.01967	-0.1636 to 0.1243	No
8984	7:**40 X** vs. 13:**0 X**	-0.0240	-0.1680 to 0.1200	No
8985	7:**40 X** vs. 13:**1 X**	-0.01133	-0.1553 to 0.1326	No
8986	7:**40 X** vs. 13:**10 X**	-0.03333	-0.1773 to 0.1106	No
8987	7:**40 X** vs. 13:**40 X**	-0.02967	-0.1736 to 0.1143	No
8988	7:**40 X** vs. 13:**50 X**	-0.03067	-0.1746 to 0.1133	No
8989	7:**40 X** vs. 14:**0 X**	-0.03267	-0.1766 to 0.1113	No
8990	7:**40 X** vs. 14:**1 X**	-0.02433	-0.1683 to 0.1196	No
8991	7:**40 X** vs. 14:**10 X**	-0.03833	-0.1823 to 0.1056	No
8992	7:**40 X** vs. 14:**40 X**	-0.0350	-0.1790 to 0.1090	No
8993	7:**40 X** vs. 14:**50 X**	-0.0390	-0.1830 to 0.1050	No
8994	7:**40 X** vs. 15:**0 X**	-0.0450	-0.1890 to 0.09896	No
8995	7:**40 X** vs. 15:**1 X**	-0.0250	-0.1690 to 0.1190	No
8996	7:**40 X** vs. 15:**10 X**	-0.05233	-0.1963 to 0.09163	No
8997	7:**40 X** vs. 15:**40 X**	-0.03467	-0.1786 to 0.1093	No
8998	7:**40 X** vs. 15:**50 X**	-0.05233	-0.1963 to 0.09163	No
8999	7:**40 X** vs. 16:**0 X**	-0.1373	-0.2813 to 0.006626	No
9000	7:**40 X** vs. 16:**1 X**	-0.02833	-0.1723 to 0.1156	No

2way ANOVA<br>Multiple comparisons		A<br>Data Set-A<br>Y	B<br>Data Set-B<br>Y	C<br>Data Set-C<br>Y
9001	7:40 X vs. 16:10 X	-0.0540	-0.1980 to 0.08996	No
9002	7:40 X vs. 16:40 X	-0.07333	-0.2173 to 0.07063	No
9003	7:40 X vs. 16:50 X	-0.1157	-0.2596 to 0.02829	No
9004	7:40 X vs. 17:0 X	-0.1900	-0.3340 to -0.04604	Yes
9005	7:40 X vs. 17:1 X	-0.1043	-0.2483 to 0.03963	No
9006	7:40 X vs. 17:10 X	-0.1610	-0.3050 to -0.01704	Yes
9007	7:40 X vs. 17:40 X	-0.08433	-0.2283 to 0.05963	No
9008	7:40 X vs. 17:50 X	-0.1297	-0.2736 to 0.01429	No
9009	7:40 X vs. 18:0 X	-0.2387	-0.3826 to -0.09471	Yes
9010	7:40 X vs. 18:1 X	-0.1470	-0.2910 to -0.003040	Yes
9011	7:40 X vs. 18:10 X	-0.2393	-0.3833 to -0.09537	Yes
9012	7:40 X vs. 18:40 X	-0.08867	-0.2326 to 0.05529	No
9013	7:40 X vs. 18:50 X	-0.1337	-0.2776 to 0.01029	No
9014	7:40 X vs. 19:0 X	-0.2737	-0.4176 to -0.1297	Yes
9015	7:40 X vs. 19:1 X	-0.2617	-0.4056 to -0.1177	Yes
9016	7:40 X vs. 19:10 X	-0.2593	-0.4033 to -0.1154	Yes
9017	7:40 X vs. 19:40 X	-0.1173	-0.2613 to 0.02663	No
9018	7:40 X vs. 19:50 X	-0.1283	-0.2723 to 0.01563	No
9019	7:40 X vs. 20:0 X	-0.3533	-0.4973 to -0.2094	Yes
9020	7:40 X vs. 20:1 X	-0.1727	-0.3166 to -0.02871	Yes
9021	7:40 X vs. 20:10 X	-0.2617	-0.4056 to -0.1177	Yes
9022	7:40 X vs. 20:40 X	-0.1290	-0.2730 to 0.01496	No
9023	7:40 X vs. 20:50 X	-0.1730	-0.3170 to -0.02904	Yes
9024	7:40 X vs. 21:0 X	-0.3767	-0.5206 to -0.2327	Yes
9025	7:40 X vs. 21:1 X	-0.3297	-0.4736 to -0.1857	Yes
9026	7:40 X vs. 21:10 X	-0.3267	-0.4706 to -0.1827	Yes
9027	7:40 X vs. 21:40 X	-0.2020	-0.3460 to -0.05804	Yes
9028	7:40 X vs. 21:50 X	-0.2923	-0.4363 to -0.1484	Yes
9029	7:40 X vs. 22:0 X	-0.4183	-0.5623 to -0.2744	Yes
9030	7:40 X vs. 22:1 X	-0.4130	-0.5570 to -0.2690	Yes
9031	7:40 X vs. 22:10 X	-0.4083	-0.5523 to -0.2644	Yes
9032	7:40 X vs. 22:40 X	-0.3087	-0.4526 to -0.1647	Yes
9033	7:40 X vs. 22:50 X	-0.3713	-0.5153 to -0.2274	Yes
9034	7:40 X vs. 23:0 X	-0.4837	-0.6276 to -0.3397	Yes
9035	7:40 X vs. 23:1 X	-0.6733	-0.8343 to -0.5124	Yes
9036	7:40 X vs. 23:10 X	-0.4230	-0.5670 to -0.2790	Yes
9037	7:40 X vs. 23:40 X	-0.3033	-0.4643 to -0.1424	Yes
9038	7:40 X vs. 23:50 X	-0.3373	-0.4813 to -0.1934	Yes
9039	7:40 X vs. 24:0 X	-0.6010	-0.7450 to -0.4570	Yes
9040	7:40 X vs. 24:1 X	-0.7573	-0.9183 to -0.5964	Yes
9041	7:40 X vs. 24:10 X	-0.5848	-0.7458 to -0.4239	Yes
9042	7:40 X vs. 24:40 X	-0.3197	-0.4636 to -0.1757	Yes
9043	7:40 X vs. 24:50 X	-0.2413	-0.3853 to -0.09737	Yes
9044	7:40 X vs. 25:0 X	-0.9383	-1.099 to -0.7774	Yes
9045	7:40 X vs. 25:1 X	-1.128	-1.289 to -0.9669	Yes

	2way ANOVA<br>Multiple comparisons	A<br>Data Set-A<br>Y	B<br>Data Set-B<br>Y	C<br>Data Set-C<br>Y
9046	7:**40 X** vs. 25:**10 X**	-0.4893	-0.6929 to -0.2857	Yes
9047	7:**40 X** vs. 25:**40 X**	-0.3217	-0.4656 to -0.1777	Yes
9048	7:**40 X** vs. 25:**50 X**	-0.2960	-0.4400 to -0.1520	Yes
9049	7:**40 X** vs. 26:**0 X**	-1.151	-1.312 to -0.9899	Yes
9050	7:**40 X** vs. 26:**1 X**	-1.264	-1.425 to -1.103	Yes
9051	7:**40 X** vs. 26:**10 X**	-0.9503	-1.111 to -0.7894	Yes
9052	7:**40 X** vs. 26:**40 X**	-0.3960	-0.5400 to -0.2520	Yes
9053	7:**40 X** vs. 26:**50 X**	-0.2563	-0.4003 to -0.1124	Yes
9054	7:**40 X** vs. 27:**0 X**	-1.339	-1.483 to -1.195	Yes
9055	7:**40 X** vs. 27:**1 X**	-1.429	-1.590 to -1.268	Yes
9056	7:**40 X** vs. 27:**10 X**	-1.241	-1.402 to -1.080	Yes
9057	7:**40 X** vs. 27:**40 X**	-0.3960	-0.5400 to -0.2520	Yes
9058	7:**40 X** vs. 27:**50 X**	-0.2557	-0.3996 to -0.1117	Yes
9059	7:**40 X** vs. 28:**0 X**	-1.288	-1.449 to -1.127	Yes
9060	7:**40 X** vs. 28:**1 X**	-1.329	-1.473 to -1.185	Yes
9061	7:**40 X** vs. 28:**10 X**	-1.130	-1.291 to -0.9694	Yes
9062	7:**40 X** vs. 28:**40 X**	-0.4083	-0.5523 to -0.2644	Yes
9063	7:**40 X** vs. 28:**50 X**	-0.3037	-0.4476 to -0.1597	Yes
9064	7:**40 X** vs. 29:**0 X**	-1.769	-1.930 to -1.608	Yes
9065	7:**40 X** vs. 29:**1 X**	-1.854	-2.015 to -1.693	Yes
9066	7:**40 X** vs. 29:**10 X**	-1.069	-1.230 to -0.9079	Yes
9067	7:**40 X** vs. 29:**40 X**	-0.5163	-0.6603 to -0.3724	Yes
9068	7:**40 X** vs. 29:**50 X**	-0.3050	-0.4490 to -0.1610	Yes
9069	7:**40 X** vs. 30:**0 X**	-1.484	-1.645 to -1.323	Yes
9070	7:**40 X** vs. 30:**1 X**	-1.805	-1.966 to -1.644	Yes
9071	7:**40 X** vs. 30:**10 X**	-1.143	-1.304 to -0.9824	Yes
9072	7:**40 X** vs. 30:**40 X**	-0.4433	-0.5873 to -0.2994	Yes
9073	7:**40 X** vs. 30:**50 X**	-0.3573	-0.5013 to -0.2134	Yes
9074	7:**40 X** vs. 31:**0 X**	-1.802	-1.963 to -1.641	Yes
9075	7:**40 X** vs. 31:**1 X**	-1.792	-1.953 to -1.631	Yes
9076	7:**40 X** vs. 31:**10 X**	-1.275	-1.436 to -1.114	Yes
9077	7:**40 X** vs. 31:**40 X**	-0.5350	-0.6790 to -0.3910	Yes
9078	7:**40 X** vs. 31:**50 X**	-0.3363	-0.4803 to -0.1924	Yes
9079	7:**40 X** vs. 32:**0 X**	-2.393	-2.537 to -2.249	Yes
9080	7:**40 X** vs. 32:**1 X**	-1.849	-2.010 to -1.688	Yes
9081	7:**40 X** vs. 32:**10 X**	-1.705	-1.849 to -1.561	Yes
9082	7:**40 X** vs. 32:**40 X**	-0.5313	-0.6753 to -0.3874	Yes
9083	7:**40 X** vs. 32:**50 X**	-0.3360	-0.4800 to -0.1920	Yes
9084	7:**40 X** vs. 33:**0 X**	-2.397	-2.541 to -2.253	Yes
9085	7:**40 X** vs. 33:**1 X**	-2.212	-2.356 to -2.068	Yes
9086	7:**40 X** vs. 33:**10 X**	-1.872	-2.016 to -1.728	Yes
9087	7:**40 X** vs. 33:**40 X**	-0.5500	-0.6940 to -0.4060	Yes
9088	7:**40 X** vs. 33:**50 X**	-0.3510	-0.4950 to -0.2070	Yes
9089	7:**40 X** vs. 34:**0 X**	-2.322	-2.466 to -2.178	Yes
9090	7:**40 X** vs. 34:**1 X**	-2.329	-2.473 to -2.185	Yes

	2way ANOVA<br>Multiple comparisons	A<br>Data Set-A<br>Y	B<br>Data Set-B<br>Y	C<br>Data Set-C<br>Y
9091	7:40 X vs. 34:10 X	-1.962	-2.106 to -1.818	Yes
9092	7:40 X vs. 34:40 X	-0.5620	-0.7060 to -0.4180	Yes
9093	7:40 X vs. 34:50 X	-0.5000	-0.6440 to -0.3560	Yes
9094	7:40 X vs. 35:0 X	-2.670	-2.814 to -2.526	Yes
9095	7:40 X vs. 35:1 X	-2.436	-2.580 to -2.292	Yes
9096	7:40 X vs. 35:10 X	-1.924	-2.068 to -1.780	Yes
9097	7:40 X vs. 35:40 X	-0.5710	-0.7150 to -0.4270	Yes
9098	7:40 X vs. 35:50 X	-0.5127	-0.6566 to -0.3687	Yes
9099	7:40 X vs. 36:0 X	-2.877	-3.038 to -2.716	Yes
9100	7:40 X vs. 36:1 X	-2.573	-2.717 to -2.429	Yes
9101	7:40 X vs. 36:10 X	-1.930	-2.074 to -1.786	Yes
9102	7:40 X vs. 36:40 X	-0.6263	-0.7703 to -0.4824	Yes
9103	7:40 X vs. 36:50 X	-0.4760	-0.6200 to -0.3320	Yes
9104	7:40 X vs. 37:0 X	-3.261	-3.405 to -3.117	Yes
9105	7:40 X vs. 37:1 X	-2.643	-2.787 to -2.499	Yes
9106	7:40 X vs. 37:10 X	-1.931	-2.075 to -1.787	Yes
9107	7:40 X vs. 37:40 X	-0.6637	-0.8076 to -0.5197	Yes
9108	7:40 X vs. 37:50 X	-0.4847	-0.6286 to -0.3407	Yes
9109	7:40 X vs. 38:0 X	-3.206	-3.350 to -3.062	Yes
9110	7:40 X vs. 38:1 X	-2.761	-2.905 to -2.617	Yes
9111	7:40 X vs. 38:10 X	-2.207	-2.351 to -2.063	Yes
9112	7:40 X vs. 38:40 X	-0.6680	-0.8120 to -0.5240	Yes
9113	7:40 X vs. 38:50 X	-0.4887	-0.6326 to -0.3447	Yes
9114	7:40 X vs. 39:0 X	-3.436	-3.580 to -3.292	Yes
9115	7:40 X vs. 39:1 X	-3.328	-3.472 to -3.184	Yes
9116	7:40 X vs. 39:10 X	-2.352	-2.496 to -2.208	Yes
9117	7:40 X vs. 39:40 X	-0.6740	-0.8180 to -0.5300	Yes
9118	7:40 X vs. 39:50 X	-0.5197	-0.6636 to -0.3757	Yes
9119	7:40 X vs. 40:0 X	-3.477	-3.621 to -3.333	Yes
9120	7:40 X vs. 40:1 X	-3.403	-3.547 to -3.259	Yes
9121	7:40 X vs. 40:10 X	-2.608	-2.752 to -2.464	Yes
9122	7:40 X vs. 40:40 X	-0.6787	-0.8226 to -0.5347	Yes
9123	7:40 X vs. 40:50 X	-0.5260	-0.6700 to -0.3820	Yes
9124	7:40 X vs. 41:0 X	-3.518	-3.662 to -3.374	Yes
9125	7:40 X vs. 41:1 X	-3.517	-3.661 to -3.373	Yes
9126	7:40 X vs. 41:10 X	-2.662	-2.806 to -2.518	Yes
9127	7:40 X vs. 41:40 X	-0.6260	-0.7700 to -0.4820	Yes
9128	7:40 X vs. 41:50 X	-0.5387	-0.6826 to -0.3947	Yes
9129	7:40 X vs. 42:0 X	-3.660	-3.804 to -3.516	Yes
9130	7:40 X vs. 42:1 X	-3.680	-3.824 to -3.536	Yes
9131	7:40 X vs. 42:10 X	-2.662	-2.806 to -2.518	Yes
9132	7:40 X vs. 42:40 X	-0.6387	-0.7826 to -0.4947	Yes
9133	7:40 X vs. 42:50 X	-0.5420	-0.6860 to -0.3980	Yes
9134	7:40 X vs. 43:0 X	-3.750	-3.894 to -3.606	Yes
9135	7:40 X vs. 43:1 X	-3.664	-3.808 to -3.520	Yes

	2way ANOVA Multiple comparisons	A Data Set-A Y	B Data Set-B Y	C Data Set-C Y
9136	7:**40 X** vs. 43:**10 X**	-3.380	-3.524 to -3.236	Yes
9137	7:**40 X** vs. 43:**40 X**	-0.6370	-0.7810 to -0.4930	Yes
9138	7:**40 X** vs. 43:**50 X**	-0.5423	-0.6863 to -0.3984	Yes
9139	7:**40 X** vs. 44:**0 X**	-3.774	-3.918 to -3.630	Yes
9140	7:**40 X** vs. 44:**1 X**	-3.664	-3.808 to -3.520	Yes
9141	7:**40 X** vs. 44:**10 X**	-3.443	-3.587 to -3.299	Yes
9142	7:**40 X** vs. 44:**40 X**	-0.6390	-0.7830 to -0.4950	Yes
9143	7:**40 X** vs. 44:**50 X**	-0.4747	-0.6186 to -0.3307	Yes
9144	7:**40 X** vs. 45:**0 X**	-3.639	-3.783 to -3.495	Yes
9145	7:**40 X** vs. 45:**1 X**	-3.441	-3.585 to -3.297	Yes
9146	7:**40 X** vs. 45:**10 X**	-3.323	-3.467 to -3.179	Yes
9147	7:**40 X** vs. 45:**40 X**	-0.6403	-0.7843 to -0.4964	Yes
9148	7:**40 X** vs. 45:**50 X**	-0.4717	-0.6156 to -0.3277	Yes
9149	7:**40 X** vs. 46:**0 X**	-3.443	-3.587 to -3.299	Yes
9150	7:**40 X** vs. 46:**1 X**	-3.410	-3.554 to -3.266	Yes
9151	7:**40 X** vs. 46:**10 X**	-3.318	-3.462 to -3.174	Yes
9152	7:**40 X** vs. 46:**40 X**	-0.6540	-0.7980 to -0.5100	Yes
9153	7:**40 X** vs. 46:**50 X**	-0.4647	-0.6086 to -0.3207	Yes
9154	7:**40 X** vs. 47:**0 X**	-3.419	-3.563 to -3.275	Yes
9155	7:**40 X** vs. 47:**1 X**	-3.323	-3.467 to -3.179	Yes
9156	7:**40 X** vs. 47:**10 X**	-3.304	-3.448 to -3.160	Yes
9157	7:**40 X** vs. 47:**40 X**	-0.6640	-0.8080 to -0.5200	Yes
9158	7:**40 X** vs. 47:**50 X**	-0.4227	-0.5666 to -0.2787	Yes
9159	7:**40 X** vs. 48:**0 X**	-3.406	-3.567 to -3.245	Yes
9160	7:**40 X** vs. 48:**1 X**	-3.310	-3.454 to -3.166	Yes
9161	7:**40 X** vs. 48:**10 X**	-3.304	-3.448 to -3.160	Yes
9162	7:**40 X** vs. 48:**40 X**	-0.6703	-0.8143 to -0.5264	Yes
9163	7:**40 X** vs. 48:**50 X**	-0.4300	-0.5740 to -0.2860	Yes
9164	7:**40 X** vs. 49:**0 X**	-3.406	-3.550 to -3.262	Yes
9165	7:**40 X** vs. 49:**1 X**	-3.310	-3.454 to -3.166	Yes
9166	7:**40 X** vs. 49:**10 X**	-3.304	-3.448 to -3.160	Yes
9167	7:**40 X** vs. 49:**40 X**	-0.6747	-0.8186 to -0.5307	Yes
9168	7:**40 X** vs. 49:**50 X**	-0.4293	-0.5733 to -0.2854	Yes
9169	7:**40 X** vs. 50:**0 X**	-3.406	-3.550 to -3.262	Yes
9170	7:**40 X** vs. 50:**1 X**	-3.310	-3.454 to -3.166	Yes
9171	7:**40 X** vs. 50:**10 X**	-3.304	-3.448 to -3.160	Yes
9172	7:**40 X** vs. 50:**40 X**	-0.6823	-0.8263 to -0.5384	Yes
9173	7:**40 X** vs. 50:**50 X**	-0.4290	-0.5730 to -0.2850	Yes
9174	7:**50 X** vs. 8:**0 X**	-0.0120	-0.1560 to 0.1320	No
9175	7:**50 X** vs. 8:**1 X**	0.001333	-0.1426 to 0.1453	No
9176	7:**50 X** vs. 8:**10 X**	-0.006000	-0.1500 to 0.1380	No
9177	7:**50 X** vs. 8:**40 X**	-0.006667	-0.1506 to 0.1373	No
9178	7:**50 X** vs. 8:**50 X**	-0.003667	-0.1476 to 0.1403	No
9179	7:**50 X** vs. 9:**0 X**	-0.0200	-0.1640 to 0.1240	No
9180	7:**50 X** vs. 9:**1 X**	-0.0280	-0.1720 to 0.1160	No

2way ANOVA<br>Multiple comparisons		A<br>Data Set-A<br>Y	B<br>Data Set-B<br>Y	C<br>Data Set-C<br>Y
9181	7:50 X vs. 9:10 X	-0.0290	-0.1730 to 0.1150	No
9182	7:50 X vs. 9:40 X	-0.01133	-0.1553 to 0.1326	No
9183	7:50 X vs. 9:50 X	-0.004667	-0.1486 to 0.1393	No
9184	7:50 X vs. 10:0 X	-0.02167	-0.1656 to 0.1223	No
9185	7:50 X vs. 10:1 X	-0.02867	-0.1726 to 0.1153	No
9186	7:50 X vs. 10:10 X	-0.03133	-0.1753 to 0.1126	No
9187	7:50 X vs. 10:40 X	-0.0210	-0.1650 to 0.1230	No
9188	7:50 X vs. 10:50 X	-0.007667	-0.1516 to 0.1363	No
9189	7:50 X vs. 11:0 X	-0.03133	-0.1753 to 0.1126	No
9190	7:50 X vs. 11:1 X	-0.0330	-0.1770 to 0.1110	No
9191	7:50 X vs. 11:10 X	-0.03333	-0.1773 to 0.1106	No
9192	7:50 X vs. 11:40 X	-0.0250	-0.1690 to 0.1190	No
9193	7:50 X vs. 11:50 X	-0.0130	-0.1570 to 0.1310	No
9194	7:50 X vs. 12:0 X	-0.0320	-0.1760 to 0.1120	No
9195	7:50 X vs. 12:1 X	-0.01433	-0.1583 to 0.1296	No
9196	7:50 X vs. 12:10 X	-0.0320	-0.1760 to 0.1120	No
9197	7:50 X vs. 12:40 X	-0.01833	-0.1623 to 0.1256	No
9198	7:50 X vs. 12:50 X	-0.02167	-0.1656 to 0.1223	No
9199	7:50 X vs. 13:0 X	-0.0260	-0.1700 to 0.1180	No
9200	7:50 X vs. 13:1 X	-0.01333	-0.1573 to 0.1306	No
9201	7:50 X vs. 13:10 X	-0.03533	-0.1793 to 0.1086	No
9202	7:50 X vs. 13:40 X	-0.03167	-0.1756 to 0.1123	No
9203	7:50 X vs. 13:50 X	-0.03267	-0.1766 to 0.1113	No
9204	7:50 X vs. 14:0 X	-0.03467	-0.1786 to 0.1093	No
9205	7:50 X vs. 14:1 X	-0.02633	-0.1703 to 0.1176	No
9206	7:50 X vs. 14:10 X	-0.04033	-0.1843 to 0.1036	No
9207	7:50 X vs. 14:40 X	-0.0370	-0.1810 to 0.1070	No
9208	7:50 X vs. 14:50 X	-0.0410	-0.1850 to 0.1030	No
9209	7:50 X vs. 15:0 X	-0.0470	-0.1910 to 0.09696	No
9210	7:50 X vs. 15:1 X	-0.0270	-0.1710 to 0.1170	No
9211	7:50 X vs. 15:10 X	-0.05433	-0.1983 to 0.08963	No
9212	7:50 X vs. 15:40 X	-0.03667	-0.1806 to 0.1073	No
9213	7:50 X vs. 15:50 X	-0.05433	-0.1983 to 0.08963	No
9214	7:50 X vs. 16:0 X	-0.1393	-0.2833 to 0.004626	No
9215	7:50 X vs. 16:1 X	-0.03033	-0.1743 to 0.1136	No
9216	7:50 X vs. 16:10 X	-0.0560	-0.2000 to 0.08796	No
9217	7:50 X vs. 16:40 X	-0.07533	-0.2193 to 0.06863	No
9218	7:50 X vs. 16:50 X	-0.1177	-0.2616 to 0.02629	No
9219	7:50 X vs. 17:0 X	-0.1920	-0.3360 to -0.04804	Yes
9220	7:50 X vs. 17:1 X	-0.1063	-0.2503 to 0.03763	No
9221	7:50 X vs. 17:10 X	-0.1630	-0.3070 to -0.01904	Yes
9222	7:50 X vs. 17:40 X	-0.08633	-0.2303 to 0.05763	No
9223	7:50 X vs. 17:50 X	-0.1317	-0.2756 to 0.01229	No
9224	7:50 X vs. 18:0 X	-0.2407	-0.3846 to -0.09671	Yes
9225	7:50 X vs. 18:1 X	-0.1490	-0.2930 to -0.005040	Yes

	2way ANOVA<br>Multiple comparisons	A<br>Data Set-A<br>Y	B<br>Data Set-B<br>Y	C<br>Data Set-C<br>Y
9226	7:50 X vs. 18:10 X	-0.2413	-0.3853 to -0.09737	Yes
9227	7:50 X vs. 18:40 X	-0.09067	-0.2346 to 0.05329	No
9228	7:50 X vs. 18:50 X	-0.1357	-0.2796 to 0.008293	No
9229	7:50 X vs. 19:0 X	-0.2757	-0.4196 to -0.1317	Yes
9230	7:50 X vs. 19:1 X	-0.2637	-0.4076 to -0.1197	Yes
9231	7:50 X vs. 19:10 X	-0.2613	-0.4053 to -0.1174	Yes
9232	7:50 X vs. 19:40 X	-0.1193	-0.2633 to 0.02463	No
9233	7:50 X vs. 19:50 X	-0.1303	-0.2743 to 0.01363	No
9234	7:50 X vs. 20:0 X	-0.3553	-0.4993 to -0.2114	Yes
9235	7:50 X vs. 20:1 X	-0.1747	-0.3186 to -0.03071	Yes
9236	7:50 X vs. 20:10 X	-0.2637	-0.4076 to -0.1197	Yes
9237	7:50 X vs. 20:40 X	-0.1310	-0.2750 to 0.01296	No
9238	7:50 X vs. 20:50 X	-0.1750	-0.3190 to -0.03104	Yes
9239	7:50 X vs. 21:0 X	-0.3787	-0.5226 to -0.2347	Yes
9240	7:50 X vs. 21:1 X	-0.3317	-0.4756 to -0.1877	Yes
9241	7:50 X vs. 21:10 X	-0.3287	-0.4726 to -0.1847	Yes
9242	7:50 X vs. 21:40 X	-0.2040	-0.3480 to -0.06004	Yes
9243	7:50 X vs. 21:50 X	-0.2943	-0.4383 to -0.1504	Yes
9244	7:50 X vs. 22:0 X	-0.4203	-0.5643 to -0.2764	Yes
9245	7:50 X vs. 22:1 X	-0.4150	-0.5590 to -0.2710	Yes
9246	7:50 X vs. 22:10 X	-0.4103	-0.5543 to -0.2664	Yes
9247	7:50 X vs. 22:40 X	-0.3107	-0.4546 to -0.1667	Yes
9248	7:50 X vs. 22:50 X	-0.3733	-0.5173 to -0.2294	Yes
9249	7:50 X vs. 23:0 X	-0.4857	-0.6296 to -0.3417	Yes
9250	7:50 X vs. 23:1 X	-0.6753	-0.8363 to -0.5144	Yes
9251	7:50 X vs. 23:10 X	-0.4250	-0.5690 to -0.2810	Yes
9252	7:50 X vs. 23:40 X	-0.3053	-0.4663 to -0.1444	Yes
9253	7:50 X vs. 23:50 X	-0.3393	-0.4833 to -0.1954	Yes
9254	7:50 X vs. 24:0 X	-0.6030	-0.7470 to -0.4590	Yes
9255	7:50 X vs. 24:1 X	-0.7593	-0.9203 to -0.5984	Yes
9256	7:50 X vs. 24:10 X	-0.5868	-0.7478 to -0.4259	Yes
9257	7:50 X vs. 24:40 X	-0.3217	-0.4656 to -0.1777	Yes
9258	7:50 X vs. 24:50 X	-0.2433	-0.3873 to -0.09937	Yes
9259	7:50 X vs. 25:0 X	-0.9403	-1.101 to -0.7794	Yes
9260	7:50 X vs. 25:1 X	-1.130	-1.291 to -0.9689	Yes
9261	7:50 X vs. 25:10 X	-0.4913	-0.6949 to -0.2877	Yes
9262	7:50 X vs. 25:40 X	-0.3237	-0.4676 to -0.1797	Yes
9263	7:50 X vs. 25:50 X	-0.2980	-0.4420 to -0.1540	Yes
9264	7:50 X vs. 26:0 X	-1.153	-1.314 to -0.9919	Yes
9265	7:50 X vs. 26:1 X	-1.266	-1.427 to -1.105	Yes
9266	7:50 X vs. 26:10 X	-0.9523	-1.113 to -0.7914	Yes
9267	7:50 X vs. 26:40 X	-0.3980	-0.5420 to -0.2540	Yes
9268	7:50 X vs. 26:50 X	-0.2583	-0.4023 to -0.1144	Yes
9269	7:50 X vs. 27:0 X	-1.341	-1.485 to -1.197	Yes
9270	7:50 X vs. 27:1 X	-1.431	-1.592 to -1.270	Yes

2way ANOVA Multiple comparisons	A Data Set-A Y	B Data Set-B Y	C Data Set-C Y
9271 7:50 X vs. 27:10 X	-1.243	-1.404 to -1.082	Yes
9272 7:50 X vs. 27:40 X	-0.3980	-0.5420 to -0.2540	Yes
9273 7:50 X vs. 27:50 X	-0.2577	-0.4016 to -0.1137	Yes
9274 7:50 X vs. 28:0 X	-1.290	-1.451 to -1.129	Yes
9275 7:50 X vs. 28:1 X	-1.331	-1.475 to -1.187	Yes
9276 7:50 X vs. 28:10 X	-1.132	-1.293 to -0.9714	Yes
9277 7:50 X vs. 28:40 X	-0.4103	-0.5543 to -0.2664	Yes
9278 7:50 X vs. 28:50 X	-0.3057	-0.4496 to -0.1617	Yes
9279 7:50 X vs. 29:0 X	-1.771	-1.932 to -1.610	Yes
9280 7:50 X vs. 29:1 X	-1.856	-2.017 to -1.695	Yes
9281 7:50 X vs. 29:10 X	-1.071	-1.232 to -0.9099	Yes
9282 7:50 X vs. 29:40 X	-0.5183	-0.6623 to -0.3744	Yes
9283 7:50 X vs. 29:50 X	-0.3070	-0.4510 to -0.1630	Yes
9284 7:50 X vs. 30:0 X	-1.486	-1.647 to -1.325	Yes
9285 7:50 X vs. 30:1 X	-1.807	-1.968 to -1.646	Yes
9286 7:50 X vs. 30:10 X	-1.145	-1.306 to -0.9844	Yes
9287 7:50 X vs. 30:40 X	-0.4453	-0.5893 to -0.3014	Yes
9288 7:50 X vs. 30:50 X	-0.3593	-0.5033 to -0.2154	Yes
9289 7:50 X vs. 31:0 X	-1.804	-1.965 to -1.643	Yes
9290 7:50 X vs. 31:1 X	-1.794	-1.955 to -1.633	Yes
9291 7:50 X vs. 31:10 X	-1.277	-1.438 to -1.116	Yes
9292 7:50 X vs. 31:40 X	-0.5370	-0.6810 to -0.3930	Yes
9293 7:50 X vs. 31:50 X	-0.3383	-0.4823 to -0.1944	Yes
9294 7:50 X vs. 32:0 X	-2.395	-2.539 to -2.251	Yes
9295 7:50 X vs. 32:1 X	-1.851	-2.012 to -1.690	Yes
9296 7:50 X vs. 32:10 X	-1.707	-1.851 to -1.563	Yes
9297 7:50 X vs. 32:40 X	-0.5333	-0.6773 to -0.3894	Yes
9298 7:50 X vs. 32:50 X	-0.3380	-0.4820 to -0.1940	Yes
9299 7:50 X vs. 33:0 X	-2.399	-2.543 to -2.255	Yes
9300 7:50 X vs. 33:1 X	-2.214	-2.358 to -2.070	Yes
9301 7:50 X vs. 33:10 X	-1.874	-2.018 to -1.730	Yes
9302 7:50 X vs. 33:40 X	-0.5520	-0.6960 to -0.4080	Yes
9303 7:50 X vs. 33:50 X	-0.3530	-0.4970 to -0.2090	Yes
9304 7:50 X vs. 34:0 X	-2.324	-2.468 to -2.180	Yes
9305 7:50 X vs. 34:1 X	-2.331	-2.475 to -2.187	Yes
9306 7:50 X vs. 34:10 X	-1.964	-2.108 to -1.820	Yes
9307 7:50 X vs. 34:40 X	-0.5640	-0.7080 to -0.4200	Yes
9308 7:50 X vs. 34:50 X	-0.5020	-0.6460 to -0.3580	Yes
9309 7:50 X vs. 35:0 X	-2.672	-2.816 to -2.528	Yes
9310 7:50 X vs. 35:1 X	-2.438	-2.582 to -2.294	Yes
9311 7:50 X vs. 35:10 X	-1.926	-2.070 to -1.782	Yes
9312 7:50 X vs. 35:40 X	-0.5730	-0.7170 to -0.4290	Yes
9313 7:50 X vs. 35:50 X	-0.5147	-0.6586 to -0.3707	Yes
9314 7:50 X vs. 36:0 X	-2.879	-3.040 to -2.718	Yes
9315 7:50 X vs. 36:1 X	-2.575	-2.719 to -2.431	Yes

	2way ANOVA Multiple comparisons	A Data Set-A Y	B Data Set-B Y	C Data Set-C Y
9316	7:**50 X** vs. 36:**10 X**	-1.932	-2.076 to -1.788	Yes
9317	7:**50 X** vs. 36:**40 X**	-0.6283	-0.7723 to -0.4844	Yes
9318	7:**50 X** vs. 36:**50 X**	-0.4780	-0.6220 to -0.3340	Yes
9319	7:**50 X** vs. 37:**0 X**	-3.263	-3.407 to -3.119	Yes
9320	7:**50 X** vs. 37:**1 X**	-2.645	-2.789 to -2.501	Yes
9321	7:**50 X** vs. 37:**10 X**	-1.933	-2.077 to -1.789	Yes
9322	7:**50 X** vs. 37:**40 X**	-0.6657	-0.8096 to -0.5217	Yes
9323	7:**50 X** vs. 37:**50 X**	-0.4867	-0.6306 to -0.3427	Yes
9324	7:**50 X** vs. 38:**0 X**	-3.208	-3.352 to -3.064	Yes
9325	7:**50 X** vs. 38:**1 X**	-2.763	-2.907 to -2.619	Yes
9326	7:**50 X** vs. 38:**10 X**	-2.209	-2.353 to -2.065	Yes
9327	7:**50 X** vs. 38:**40 X**	-0.6700	-0.8140 to -0.5260	Yes
9328	7:**50 X** vs. 38:**50 X**	-0.4907	-0.6346 to -0.3467	Yes
9329	7:**50 X** vs. 39:**0 X**	-3.438	-3.582 to -3.294	Yes
9330	7:**50 X** vs. 39:**1 X**	-3.330	-3.474 to -3.186	Yes
9331	7:**50 X** vs. 39:**10 X**	-2.354	-2.498 to -2.210	Yes
9332	7:**50 X** vs. 39:**40 X**	-0.6760	-0.8200 to -0.5320	Yes
9333	7:**50 X** vs. 39:**50 X**	-0.5217	-0.6656 to -0.3777	Yes
9334	7:**50 X** vs. 40:**0 X**	-3.479	-3.623 to -3.335	Yes
9335	7:**50 X** vs. 40:**1 X**	-3.405	-3.549 to -3.261	Yes
9336	7:**50 X** vs. 40:**10 X**	-2.610	-2.754 to -2.466	Yes
9337	7:**50 X** vs. 40:**40 X**	-0.6807	-0.8246 to -0.5367	Yes
9338	7:**50 X** vs. 40:**50 X**	-0.5280	-0.6720 to -0.3840	Yes
9339	7:**50 X** vs. 41:**0 X**	-3.520	-3.664 to -3.376	Yes
9340	7:**50 X** vs. 41:**1 X**	-3.519	-3.663 to -3.375	Yes
9341	7:**50 X** vs. 41:**10 X**	-2.664	-2.808 to -2.520	Yes
9342	7:**50 X** vs. 41:**40 X**	-0.6280	-0.7720 to -0.4840	Yes
9343	7:**50 X** vs. 41:**50 X**	-0.5407	-0.6846 to -0.3967	Yes
9344	7:**50 X** vs. 42:**0 X**	-3.662	-3.806 to -3.518	Yes
9345	7:**50 X** vs. 42:**1 X**	-3.682	-3.826 to -3.538	Yes
9346	7:**50 X** vs. 42:**10 X**	-2.664	-2.808 to -2.520	Yes
9347	7:**50 X** vs. 42:**40 X**	-0.6407	-0.7846 to -0.4967	Yes
9348	7:**50 X** vs. 42:**50 X**	-0.5440	-0.6880 to -0.4000	Yes
9349	7:**50 X** vs. 43:**0 X**	-3.752	-3.896 to -3.608	Yes
9350	7:**50 X** vs. 43:**1 X**	-3.666	-3.810 to -3.522	Yes
9351	7:**50 X** vs. 43:**10 X**	-3.382	-3.526 to -3.238	Yes
9352	7:**50 X** vs. 43:**40 X**	-0.6390	-0.7830 to -0.4950	Yes
9353	7:**50 X** vs. 43:**50 X**	-0.5443	-0.6883 to -0.4004	Yes
9354	7:**50 X** vs. 44:**0 X**	-3.776	-3.920 to -3.632	Yes
9355	7:**50 X** vs. 44:**1 X**	-3.666	-3.810 to -3.522	Yes
9356	7:**50 X** vs. 44:**10 X**	-3.445	-3.589 to -3.301	Yes
9357	7:**50 X** vs. 44:**40 X**	-0.6410	-0.7850 to -0.4970	Yes
9358	7:**50 X** vs. 44:**50 X**	-0.4767	-0.6206 to -0.3327	Yes
9359	7:**50 X** vs. 45:**0 X**	-3.641	-3.785 to -3.497	Yes
9360	7:**50 X** vs. 45:**1 X**	-3.443	-3.587 to -3.299	Yes

	2way ANOVA Multiple comparisons	A Data Set-A Y	B Data Set-B Y	C Data Set-C Y
9361	7:**50 X** vs. 45:**10 X**	-3.325	-3.469 to -3.181	Yes
9362	7:**50 X** vs. 45:**40 X**	-0.6423	-0.7863 to -0.4984	Yes
9363	7:**50 X** vs. 45:**50 X**	-0.4737	-0.6176 to -0.3297	Yes
9364	7:**50 X** vs. 46:**0 X**	-3.445	-3.589 to -3.301	Yes
9365	7:**50 X** vs. 46:**1 X**	-3.412	-3.556 to -3.268	Yes
9366	7:**50 X** vs. 46:**10 X**	-3.320	-3.464 to -3.176	Yes
9367	7:**50 X** vs. 46:**40 X**	-0.6560	-0.8000 to -0.5120	Yes
9368	7:**50 X** vs. 46:**50 X**	-0.4667	-0.6106 to -0.3227	Yes
9369	7:**50 X** vs. 47:**0 X**	-3.421	-3.565 to -3.277	Yes
9370	7:**50 X** vs. 47:**1 X**	-3.325	-3.469 to -3.181	Yes
9371	7:**50 X** vs. 47:**10 X**	-3.306	-3.450 to -3.162	Yes
9372	7:**50 X** vs. 47:**40 X**	-0.6660	-0.8100 to -0.5220	Yes
9373	7:**50 X** vs. 47:**50 X**	-0.4247	-0.5686 to -0.2807	Yes
9374	7:**50 X** vs. 48:**0 X**	-3.408	-3.569 to -3.247	Yes
9375	7:**50 X** vs. 48:**1 X**	-3.312	-3.456 to -3.168	Yes
9376	7:**50 X** vs. 48:**10 X**	-3.306	-3.450 to -3.162	Yes
9377	7:**50 X** vs. 48:**40 X**	-0.6723	-0.8163 to -0.5284	Yes
9378	7:**50 X** vs. 48:**50 X**	-0.4320	-0.5760 to -0.2880	Yes
9379	7:**50 X** vs. 49:**0 X**	-3.408	-3.552 to -3.264	Yes
9380	7:**50 X** vs. 49:**1 X**	-3.312	-3.456 to -3.168	Yes
9381	7:**50 X** vs. 49:**10 X**	-3.306	-3.450 to -3.162	Yes
9382	7:**50 X** vs. 49:**40 X**	-0.6767	-0.8206 to -0.5327	Yes
9383	7:**50 X** vs. 49:**50 X**	-0.4313	-0.5753 to -0.2874	Yes
9384	7:**50 X** vs. 50:**0 X**	-3.408	-3.552 to -3.264	Yes
9385	7:**50 X** vs. 50:**1 X**	-3.312	-3.456 to -3.168	Yes
9386	7:**50 X** vs. 50:**10 X**	-3.306	-3.450 to -3.162	Yes
9387	7:**50 X** vs. 50:**40 X**	-0.6843	-0.8283 to -0.5404	Yes
9388	7:**50 X** vs. 50:**50 X**	-0.4310	-0.5750 to -0.2870	Yes
9389	8:**0 X** vs. 8:**1 X**	0.01333	-0.1306 to 0.1573	No
9390	8:**0 X** vs. 8:**10 X**	0.006000	-0.1380 to 0.1500	No
9391	8:**0 X** vs. 8:**40 X**	0.005333	-0.1386 to 0.1493	No
9392	8:**0 X** vs. 8:**50 X**	0.008333	-0.1356 to 0.1523	No
9393	8:**0 X** vs. 9:**0 X**	-0.008000	-0.1520 to 0.1360	No
9394	8:**0 X** vs. 9:**1 X**	-0.0160	-0.1600 to 0.1280	No
9395	8:**0 X** vs. 9:**10 X**	-0.0170	-0.1610 to 0.1270	No
9396	8:**0 X** vs. 9:**40 X**	0.0006667	-0.1433 to 0.1446	No
9397	8:**0 X** vs. 9:**50 X**	0.007333	-0.1366 to 0.1513	No
9398	8:**0 X** vs. 10:**0 X**	-0.009667	-0.1536 to 0.1343	No
9399	8:**0 X** vs. 10:**1 X**	-0.01667	-0.1606 to 0.1273	No
9400	8:**0 X** vs. 10:**10 X**	-0.01933	-0.1633 to 0.1246	No
9401	8:**0 X** vs. 10:**40 X**	-0.009000	-0.1530 to 0.1350	No
9402	8:**0 X** vs. 10:**50 X**	0.004333	-0.1396 to 0.1483	No
9403	8:**0 X** vs. 11:**0 X**	-0.01933	-0.1633 to 0.1246	No
9404	8:**0 X** vs. 11:**1 X**	-0.0210	-0.1650 to 0.1230	No
9405	8:**0 X** vs. 11:**10 X**	-0.02133	-0.1653 to 0.1226	No

	2way ANOVA<br>Multiple comparisons	A<br>Data Set-A<br>Y	B<br>Data Set-B<br>Y	C<br>Data Set-C<br>Y
**9406**	8:**0 X** vs. 11:**40 X**	-0.0130	-0.1570 to 0.1310	No
**9407**	8:**0 X** vs. 11:**50 X**	-0.0010	-0.1450 to 0.1430	No
**9408**	8:**0 X** vs. 12:**0 X**	-0.0200	-0.1640 to 0.1240	No
**9409**	8:**0 X** vs. 12:**1 X**	-0.002333	-0.1463 to 0.1416	No
**9410**	8:**0 X** vs. 12:**10 X**	-0.0200	-0.1640 to 0.1240	No
**9411**	8:**0 X** vs. 12:**40 X**	-0.006333	-0.1503 to 0.1376	No
**9412**	8:**0 X** vs. 12:**50 X**	-0.009667	-0.1536 to 0.1343	No
**9413**	8:**0 X** vs. 13:**0 X**	-0.0140	-0.1580 to 0.1300	No
**9414**	8:**0 X** vs. 13:**1 X**	-0.001333	-0.1453 to 0.1426	No
**9415**	8:**0 X** vs. 13:**10 X**	-0.02333	-0.1673 to 0.1206	No
**9416**	8:**0 X** vs. 13:**40 X**	-0.01967	-0.1636 to 0.1243	No
**9417**	8:**0 X** vs. 13:**50 X**	-0.02067	-0.1646 to 0.1233	No
**9418**	8:**0 X** vs. 14:**0 X**	-0.02267	-0.1666 to 0.1213	No
**9419**	8:**0 X** vs. 14:**1 X**	-0.01433	-0.1583 to 0.1296	No
**9420**	8:**0 X** vs. 14:**10 X**	-0.02833	-0.1723 to 0.1156	No
**9421**	8:**0 X** vs. 14:**40 X**	-0.0250	-0.1690 to 0.1190	No
**9422**	8:**0 X** vs. 14:**50 X**	-0.0290	-0.1730 to 0.1150	No
**9423**	8:**0 X** vs. 15:**0 X**	-0.0350	-0.1790 to 0.1090	No
**9424**	8:**0 X** vs. 15:**1 X**	-0.0150	-0.1590 to 0.1290	No
**9425**	8:**0 X** vs. 15:**10 X**	-0.04233	-0.1863 to 0.1016	No
**9426**	8:**0 X** vs. 15:**40 X**	-0.02467	-0.1686 to 0.1193	No
**9427**	8:**0 X** vs. 15:**50 X**	-0.04233	-0.1863 to 0.1016	No
**9428**	8:**0 X** vs. 16:**0 X**	-0.1273	-0.2713 to 0.01663	No
**9429**	8:**0 X** vs. 16:**1 X**	-0.01833	-0.1623 to 0.1256	No
**9430**	8:**0 X** vs. 16:**10 X**	-0.0440	-0.1880 to 0.09996	No
**9431**	8:**0 X** vs. 16:**40 X**	-0.06333	-0.2073 to 0.08063	No
**9432**	8:**0 X** vs. 16:**50 X**	-0.1057	-0.2496 to 0.03829	No
**9433**	8:**0 X** vs. 17:**0 X**	-0.1800	-0.3240 to -0.03604	Yes
**9434**	8:**0 X** vs. 17:**1 X**	-0.09433	-0.2383 to 0.04963	No
**9435**	8:**0 X** vs. 17:**10 X**	-0.1510	-0.2950 to -0.007040	Yes
**9436**	8:**0 X** vs. 17:**40 X**	-0.07433	-0.2183 to 0.06963	No
**9437**	8:**0 X** vs. 17:**50 X**	-0.1197	-0.2636 to 0.02429	No
**9438**	8:**0 X** vs. 18:**0 X**	-0.2287	-0.3726 to -0.08471	Yes
**9439**	8:**0 X** vs. 18:**1 X**	-0.1370	-0.2810 to 0.006960	No
**9440**	8:**0 X** vs. 18:**10 X**	-0.2293	-0.3733 to -0.08537	Yes
**9441**	8:**0 X** vs. 18:**40 X**	-0.07867	-0.2226 to 0.06529	No
**9442**	8:**0 X** vs. 18:**50 X**	-0.1237	-0.2676 to 0.02029	No
**9443**	8:**0 X** vs. 19:**0 X**	-0.2637	-0.4076 to -0.1197	Yes
**9444**	8:**0 X** vs. 19:**1 X**	-0.2517	-0.3956 to -0.1077	Yes
**9445**	8:**0 X** vs. 19:**10 X**	-0.2493	-0.3933 to -0.1054	Yes
**9446**	8:**0 X** vs. 19:**40 X**	-0.1073	-0.2513 to 0.03663	No
**9447**	8:**0 X** vs. 19:**50 X**	-0.1183	-0.2623 to 0.02563	No
**9448**	8:**0 X** vs. 20:**0 X**	-0.3433	-0.4873 to -0.1994	Yes
**9449**	8:**0 X** vs. 20:**1 X**	-0.1627	-0.3066 to -0.01871	Yes
**9450**	8:**0 X** vs. 20:**10 X**	-0.2517	-0.3956 to -0.1077	Yes

2way ANOVA<br>Multiple comparisons		A<br>Data Set-A<br>Y	B<br>Data Set-B<br>Y	C<br>Data Set-C<br>Y
9451	8:0 X vs. 20:40 X	-0.1190	-0.2630 to 0.02496	No
9452	8:0 X vs. 20:50 X	-0.1630	-0.3070 to -0.01904	Yes
9453	8:0 X vs. 21:0 X	-0.3667	-0.5106 to -0.2227	Yes
9454	8:0 X vs. 21:1 X	-0.3197	-0.4636 to -0.1757	Yes
9455	8:0 X vs. 21:10 X	-0.3167	-0.4606 to -0.1727	Yes
9456	8:0 X vs. 21:40 X	-0.1920	-0.3360 to -0.04804	Yes
9457	8:0 X vs. 21:50 X	-0.2823	-0.4263 to -0.1384	Yes
9458	8:0 X vs. 22:0 X	-0.4083	-0.5523 to -0.2644	Yes
9459	8:0 X vs. 22:1 X	-0.4030	-0.5470 to -0.2590	Yes
9460	8:0 X vs. 22:10 X	-0.3983	-0.5423 to -0.2544	Yes
9461	8:0 X vs. 22:40 X	-0.2987	-0.4426 to -0.1547	Yes
9462	8:0 X vs. 22:50 X	-0.3613	-0.5053 to -0.2174	Yes
9463	8:0 X vs. 23:0 X	-0.4737	-0.6176 to -0.3297	Yes
9464	8:0 X vs. 23:1 X	-0.6633	-0.8243 to -0.5024	Yes
9465	8:0 X vs. 23:10 X	-0.4130	-0.5570 to -0.2690	Yes
9466	8:0 X vs. 23:40 X	-0.2933	-0.4543 to -0.1324	Yes
9467	8:0 X vs. 23:50 X	-0.3273	-0.4713 to -0.1834	Yes
9468	8:0 X vs. 24:0 X	-0.5910	-0.7350 to -0.4470	Yes
9469	8:0 X vs. 24:1 X	-0.7473	-0.9083 to -0.5864	Yes
9470	8:0 X vs. 24:10 X	-0.5748	-0.7358 to -0.4139	Yes
9471	8:0 X vs. 24:40 X	-0.3097	-0.4536 to -0.1657	Yes
9472	8:0 X vs. 24:50 X	-0.2313	-0.3753 to -0.08737	Yes
9473	8:0 X vs. 25:0 X	-0.9283	-1.089 to -0.7674	Yes
9474	8:0 X vs. 25:1 X	-1.118	-1.279 to -0.9569	Yes
9475	8:0 X vs. 25:10 X	-0.4793	-0.6829 to -0.2757	Yes
9476	8:0 X vs. 25:40 X	-0.3117	-0.4556 to -0.1677	Yes
9477	8:0 X vs. 25:50 X	-0.2860	-0.4300 to -0.1420	Yes
9478	8:0 X vs. 26:0 X	-1.141	-1.302 to -0.9799	Yes
9479	8:0 X vs. 26:1 X	-1.254	-1.415 to -1.093	Yes
9480	8:0 X vs. 26:10 X	-0.9403	-1.101 to -0.7794	Yes
9481	8:0 X vs. 26:40 X	-0.3860	-0.5300 to -0.2420	Yes
9482	8:0 X vs. 26:50 X	-0.2463	-0.3903 to -0.1024	Yes
9483	8:0 X vs. 27:0 X	-1.329	-1.473 to -1.185	Yes
9484	8:0 X vs. 27:1 X	-1.419	-1.580 to -1.258	Yes
9485	8:0 X vs. 27:10 X	-1.231	-1.392 to -1.070	Yes
9486	8:0 X vs. 27:40 X	-0.3860	-0.5300 to -0.2420	Yes
9487	8:0 X vs. 27:50 X	-0.2457	-0.3896 to -0.1017	Yes
9488	8:0 X vs. 28:0 X	-1.278	-1.439 to -1.117	Yes
9489	8:0 X vs. 28:1 X	-1.319	-1.463 to -1.175	Yes
9490	8:0 X vs. 28:10 X	-1.120	-1.281 to -0.9594	Yes
9491	8:0 X vs. 28:40 X	-0.3983	-0.5423 to -0.2544	Yes
9492	8:0 X vs. 28:50 X	-0.2937	-0.4376 to -0.1497	Yes
9493	8:0 X vs. 29:0 X	-1.759	-1.920 to -1.598	Yes
9494	8:0 X vs. 29:1 X	-1.844	-2.005 to -1.683	Yes
9495	8:0 X vs. 29:10 X	-1.059	-1.220 to -0.8979	Yes

	2way ANOVA<br>Multiple comparisons	A<br>Data Set-A<br>Y	B<br>Data Set-B<br>Y	C<br>Data Set-C<br>Y
9496	8:0 X vs. 29:40 X	-0.5063	-0.6503 to -0.3624	Yes
9497	8:0 X vs. 29:50 X	-0.2950	-0.4390 to -0.1510	Yes
9498	8:0 X vs. 30:0 X	-1.474	-1.635 to -1.313	Yes
9499	8:0 X vs. 30:1 X	-1.795	-1.956 to -1.634	Yes
9500	8:0 X vs. 30:10 X	-1.133	-1.294 to -0.9724	Yes
9501	8:0 X vs. 30:40 X	-0.4333	-0.5773 to -0.2894	Yes
9502	8:0 X vs. 30:50 X	-0.3473	-0.4913 to -0.2034	Yes
9503	8:0 X vs. 31:0 X	-1.792	-1.953 to -1.631	Yes
9504	8:0 X vs. 31:1 X	-1.782	-1.943 to -1.621	Yes
9505	8:0 X vs. 31:10 X	-1.265	-1.426 to -1.104	Yes
9506	8:0 X vs. 31:40 X	-0.5250	-0.6690 to -0.3810	Yes
9507	8:0 X vs. 31:50 X	-0.3263	-0.4703 to -0.1824	Yes
9508	8:0 X vs. 32:0 X	-2.383	-2.527 to -2.239	Yes
9509	8:0 X vs. 32:1 X	-1.839	-2.000 to -1.678	Yes
9510	8:0 X vs. 32:10 X	-1.695	-1.839 to -1.551	Yes
9511	8:0 X vs. 32:40 X	-0.5213	-0.6653 to -0.3774	Yes
9512	8:0 X vs. 32:50 X	-0.3260	-0.4700 to -0.1820	Yes
9513	8:0 X vs. 33:0 X	-2.387	-2.531 to -2.243	Yes
9514	8:0 X vs. 33:1 X	-2.202	-2.346 to -2.058	Yes
9515	8:0 X vs. 33:10 X	-1.862	-2.006 to -1.718	Yes
9516	8:0 X vs. 33:40 X	-0.5400	-0.6840 to -0.3960	Yes
9517	8:0 X vs. 33:50 X	-0.3410	-0.4850 to -0.1970	Yes
9518	8:0 X vs. 34:0 X	-2.312	-2.456 to -2.168	Yes
9519	8:0 X vs. 34:1 X	-2.319	-2.463 to -2.175	Yes
9520	8:0 X vs. 34:10 X	-1.952	-2.096 to -1.808	Yes
9521	8:0 X vs. 34:40 X	-0.5520	-0.6960 to -0.4080	Yes
9522	8:0 X vs. 34:50 X	-0.4900	-0.6340 to -0.3460	Yes
9523	8:0 X vs. 35:0 X	-2.660	-2.804 to -2.516	Yes
9524	8:0 X vs. 35:1 X	-2.426	-2.570 to -2.282	Yes
9525	8:0 X vs. 35:10 X	-1.914	-2.058 to -1.770	Yes
9526	8:0 X vs. 35:40 X	-0.5610	-0.7050 to -0.4170	Yes
9527	8:0 X vs. 35:50 X	-0.5027	-0.6466 to -0.3587	Yes
9528	8:0 X vs. 36:0 X	-2.867	-3.028 to -2.706	Yes
9529	8:0 X vs. 36:1 X	-2.563	-2.707 to -2.419	Yes
9530	8:0 X vs. 36:10 X	-1.920	-2.064 to -1.776	Yes
9531	8:0 X vs. 36:40 X	-0.6163	-0.7603 to -0.4724	Yes
9532	8:0 X vs. 36:50 X	-0.4660	-0.6100 to -0.3220	Yes
9533	8:0 X vs. 37:0 X	-3.251	-3.395 to -3.107	Yes
9534	8:0 X vs. 37:1 X	-2.633	-2.777 to -2.489	Yes
9535	8:0 X vs. 37:10 X	-1.921	-2.065 to -1.777	Yes
9536	8:0 X vs. 37:40 X	-0.6537	-0.7976 to -0.5097	Yes
9537	8:0 X vs. 37:50 X	-0.4747	-0.6186 to -0.3307	Yes
9538	8:0 X vs. 38:0 X	-3.196	-3.340 to -3.052	Yes
9539	8:0 X vs. 38:1 X	-2.751	-2.895 to -2.607	Yes
9540	8:0 X vs. 38:10 X	-2.197	-2.341 to -2.053	Yes

	2way ANOVA<br>Multiple comparisons	A<br>Data Set-A<br>Y	B<br>Data Set-B<br>Y	C<br>Data Set-C<br>Y
9541	8:**0 X** vs. 38:**40 X**	-0.6580	-0.8020 to -0.5140	Yes
9542	8:**0 X** vs. 38:**50 X**	-0.4787	-0.6226 to -0.3347	Yes
9543	8:**0 X** vs. 39:**0 X**	-3.426	-3.570 to -3.282	Yes
9544	8:**0 X** vs. 39:**1 X**	-3.318	-3.462 to -3.174	Yes
9545	8:**0 X** vs. 39:**10 X**	-2.342	-2.486 to -2.198	Yes
9546	8:**0 X** vs. 39:**40 X**	-0.6640	-0.8080 to -0.5200	Yes
9547	8:**0 X** vs. 39:**50 X**	-0.5097	-0.6536 to -0.3657	Yes
9548	8:**0 X** vs. 40:**0 X**	-3.467	-3.611 to -3.323	Yes
9549	8:**0 X** vs. 40:**1 X**	-3.393	-3.537 to -3.249	Yes
9550	8:**0 X** vs. 40:**10 X**	-2.598	-2.742 to -2.454	Yes
9551	8:**0 X** vs. 40:**40 X**	-0.6687	-0.8126 to -0.5247	Yes
9552	8:**0 X** vs. 40:**50 X**	-0.5160	-0.6600 to -0.3720	Yes
9553	8:**0 X** vs. 41:**0 X**	-3.508	-3.652 to -3.364	Yes
9554	8:**0 X** vs. 41:**1 X**	-3.507	-3.651 to -3.363	Yes
9555	8:**0 X** vs. 41:**10 X**	-2.652	-2.796 to -2.508	Yes
9556	8:**0 X** vs. 41:**40 X**	-0.6160	-0.7600 to -0.4720	Yes
9557	8:**0 X** vs. 41:**50 X**	-0.5287	-0.6726 to -0.3847	Yes
9558	8:**0 X** vs. 42:**0 X**	-3.650	-3.794 to -3.506	Yes
9559	8:**0 X** vs. 42:**1 X**	-3.670	-3.814 to -3.526	Yes
9560	8:**0 X** vs. 42:**10 X**	-2.652	-2.796 to -2.508	Yes
9561	8:**0 X** vs. 42:**40 X**	-0.6287	-0.7726 to -0.4847	Yes
9562	8:**0 X** vs. 42:**50 X**	-0.5320	-0.6760 to -0.3880	Yes
9563	8:**0 X** vs. 43:**0 X**	-3.740	-3.884 to -3.596	Yes
9564	8:**0 X** vs. 43:**1 X**	-3.654	-3.798 to -3.510	Yes
9565	8:**0 X** vs. 43:**10 X**	-3.370	-3.514 to -3.226	Yes
9566	8:**0 X** vs. 43:**40 X**	-0.6270	-0.7710 to -0.4830	Yes
9567	8:**0 X** vs. 43:**50 X**	-0.5323	-0.6763 to -0.3884	Yes
9568	8:**0 X** vs. 44:**0 X**	-3.764	-3.908 to -3.620	Yes
9569	8:**0 X** vs. 44:**1 X**	-3.654	-3.798 to -3.510	Yes
9570	8:**0 X** vs. 44:**10 X**	-3.433	-3.577 to -3.289	Yes
9571	8:**0 X** vs. 44:**40 X**	-0.6290	-0.7730 to -0.4850	Yes
9572	8:**0 X** vs. 44:**50 X**	-0.4647	-0.6086 to -0.3207	Yes
9573	8:**0 X** vs. 45:**0 X**	-3.629	-3.773 to -3.485	Yes
9574	8:**0 X** vs. 45:**1 X**	-3.431	-3.575 to -3.287	Yes
9575	8:**0 X** vs. 45:**10 X**	-3.313	-3.457 to -3.169	Yes
9576	8:**0 X** vs. 45:**40 X**	-0.6303	-0.7743 to -0.4864	Yes
9577	8:**0 X** vs. 45:**50 X**	-0.4617	-0.6056 to -0.3177	Yes
9578	8:**0 X** vs. 46:**0 X**	-3.433	-3.577 to -3.289	Yes
9579	8:**0 X** vs. 46:**1 X**	-3.400	-3.544 to -3.256	Yes
9580	8:**0 X** vs. 46:**10 X**	-3.308	-3.452 to -3.164	Yes
9581	8:**0 X** vs. 46:**40 X**	-0.6440	-0.7880 to -0.5000	Yes
9582	8:**0 X** vs. 46:**50 X**	-0.4547	-0.5986 to -0.3107	Yes
9583	8:**0 X** vs. 47:**0 X**	-3.409	-3.553 to -3.265	Yes
9584	8:**0 X** vs. 47:**1 X**	-3.313	-3.457 to -3.169	Yes
9585	8:**0 X** vs. 47:**10 X**	-3.294	-3.438 to -3.150	Yes

2way ANOVA<br>Multiple comparisons		A<br>Data Set-A<br>Y	B<br>Data Set-B<br>Y	C<br>Data Set-C<br>Y
9586	8:**0 X** vs. 47:**40 X**	-0.6540	-0.7980 to -0.5100	Yes
9587	8:**0 X** vs. 47:**50 X**	-0.4127	-0.5566 to -0.2687	Yes
9588	8:**0 X** vs. 48:**0 X**	-3.396	-3.557 to -3.235	Yes
9589	8:**0 X** vs. 48:**1 X**	-3.300	-3.444 to -3.156	Yes
9590	8:**0 X** vs. 48:**10 X**	-3.294	-3.438 to -3.150	Yes
9591	8:**0 X** vs. 48:**40 X**	-0.6603	-0.8043 to -0.5164	Yes
9592	8:**0 X** vs. 48:**50 X**	-0.4200	-0.5640 to -0.2760	Yes
9593	8:**0 X** vs. 49:**0 X**	-3.396	-3.540 to -3.252	Yes
9594	8:**0 X** vs. 49:**1 X**	-3.300	-3.444 to -3.156	Yes
9595	8:**0 X** vs. 49:**10 X**	-3.294	-3.438 to -3.150	Yes
9596	8:**0 X** vs. 49:**40 X**	-0.6647	-0.8086 to -0.5207	Yes
9597	8:**0 X** vs. 49:**50 X**	-0.4193	-0.5633 to -0.2754	Yes
9598	8:**0 X** vs. 50:**0 X**	-3.396	-3.540 to -3.252	Yes
9599	8:**0 X** vs. 50:**1 X**	-3.300	-3.444 to -3.156	Yes
9600	8:**0 X** vs. 50:**10 X**	-3.294	-3.438 to -3.150	Yes
9601	8:**0 X** vs. 50:**40 X**	-0.6723	-0.8163 to -0.5284	Yes
9602	8:**0 X** vs. 50:**50 X**	-0.4190	-0.5630 to -0.2750	Yes
9603	8:**1 X** vs. 8:**10 X**	-0.007333	-0.1513 to 0.1366	No
9604	8:**1 X** vs. 8:**40 X**	-0.008000	-0.1520 to 0.1360	No
9605	8:**1 X** vs. 8:**50 X**	-0.0050	-0.1490 to 0.1390	No
9606	8:**1 X** vs. 9:**0 X**	-0.02133	-0.1653 to 0.1226	No
9607	8:**1 X** vs. 9:**1 X**	-0.02933	-0.1733 to 0.1146	No
9608	8:**1 X** vs. 9:**10 X**	-0.03033	-0.1743 to 0.1136	No
9609	8:**1 X** vs. 9:**40 X**	-0.01267	-0.1566 to 0.1313	No
9610	8:**1 X** vs. 9:**50 X**	-0.006000	-0.1500 to 0.1380	No
9611	8:**1 X** vs. 10:**0 X**	-0.0230	-0.1670 to 0.1210	No
9612	8:**1 X** vs. 10:**1 X**	-0.0300	-0.1740 to 0.1140	No
9613	8:**1 X** vs. 10:**10 X**	-0.03267	-0.1766 to 0.1113	No
9614	8:**1 X** vs. 10:**40 X**	-0.02233	-0.1663 to 0.1216	No
9615	8:**1 X** vs. 10:**50 X**	-0.0090	-0.1530 to 0.1350	No
9616	8:**1 X** vs. 11:**0 X**	-0.03267	-0.1766 to 0.1113	No
9617	8:**1 X** vs. 11:**1 X**	-0.03433	-0.1783 to 0.1096	No
9618	8:**1 X** vs. 11:**10 X**	-0.03467	-0.1786 to 0.1093	No
9619	8:**1 X** vs. 11:**40 X**	-0.02633	-0.1703 to 0.1176	No
9620	8:**1 X** vs. 11:**50 X**	-0.01433	-0.1583 to 0.1296	No
9621	8:**1 X** vs. 12:**0 X**	-0.03333	-0.1773 to 0.1106	No
9622	8:**1 X** vs. 12:**1 X**	-0.01567	-0.1596 to 0.1283	No
9623	8:**1 X** vs. 12:**10 X**	-0.03333	-0.1773 to 0.1106	No
9624	8:**1 X** vs. 12:**40 X**	-0.01967	-0.1636 to 0.1243	No
9625	8:**1 X** vs. 12:**50 X**	-0.0230	-0.1670 to 0.1210	No
9626	8:**1 X** vs. 13:**0 X**	-0.02733	-0.1713 to 0.1166	No
9627	8:**1 X** vs. 13:**1 X**	-0.01467	-0.1586 to 0.1293	No
9628	8:**1 X** vs. 13:**10 X**	-0.03667	-0.1806 to 0.1073	No
9629	8:**1 X** vs. 13:**40 X**	-0.0330	-0.1770 to 0.1110	No
9630	8:**1 X** vs. 13:**50 X**	-0.0340	-0.1780 to 0.1100	No

	2way ANOVA<br>Multiple comparisons	A<br>Data Set-A<br>Y	B<br>Data Set-B<br>Y	C<br>Data Set-C<br>Y
9631	8:1 X vs. 14:0 X	-0.0360	-0.1800 to 0.1080	No
9632	8:1 X vs. 14:1 X	-0.02767	-0.1716 to 0.1163	No
9633	8:1 X vs. 14:10 X	-0.04167	-0.1856 to 0.1023	No
9634	8:1 X vs. 14:40 X	-0.03833	-0.1823 to 0.1056	No
9635	8:1 X vs. 14:50 X	-0.04233	-0.1863 to 0.1016	No
9636	8:1 X vs. 15:0 X	-0.04833	-0.1923 to 0.09563	No
9637	8:1 X vs. 15:1 X	-0.02833	-0.1723 to 0.1156	No
9638	8:1 X vs. 15:10 X	-0.05567	-0.1996 to 0.08829	No
9639	8:1 X vs. 15:40 X	-0.0380	-0.1820 to 0.1060	No
9640	8:1 X vs. 15:50 X	-0.05567	-0.1996 to 0.08829	No
9641	8:1 X vs. 16:0 X	-0.1407	-0.2846 to 0.003293	No
9642	8:1 X vs. 16:1 X	-0.03167	-0.1756 to 0.1123	No
9643	8:1 X vs. 16:10 X	-0.05733	-0.2013 to 0.08663	No
9644	8:1 X vs. 16:40 X	-0.07667	-0.2206 to 0.06729	No
9645	8:1 X vs. 16:50 X	-0.1190	-0.2630 to 0.02496	No
9646	8:1 X vs. 17:0 X	-0.1933	-0.3373 to -0.04937	Yes
9647	8:1 X vs. 17:1 X	-0.1077	-0.2516 to 0.03629	No
9648	8:1 X vs. 17:10 X	-0.1643	-0.3083 to -0.02037	Yes
9649	8:1 X vs. 17:40 X	-0.08767	-0.2316 to 0.05629	No
9650	8:1 X vs. 17:50 X	-0.1330	-0.2770 to 0.01096	No
9651	8:1 X vs. 18:0 X	-0.2420	-0.3860 to -0.09804	Yes
9652	8:1 X vs. 18:1 X	-0.1503	-0.2943 to -0.006374	Yes
9653	8:1 X vs. 18:10 X	-0.2427	-0.3866 to -0.09871	Yes
9654	8:1 X vs. 18:40 X	-0.0920	-0.2360 to 0.05196	No
9655	8:1 X vs. 18:50 X	-0.1370	-0.2810 to 0.006960	No
9656	8:1 X vs. 19:0 X	-0.2770	-0.4210 to -0.1330	Yes
9657	8:1 X vs. 19:1 X	-0.2650	-0.4090 to -0.1210	Yes
9658	8:1 X vs. 19:10 X	-0.2627	-0.4066 to -0.1187	Yes
9659	8:1 X vs. 19:40 X	-0.1207	-0.2646 to 0.02329	No
9660	8:1 X vs. 19:50 X	-0.1317	-0.2756 to 0.01229	No
9661	8:1 X vs. 20:0 X	-0.3567	-0.5006 to -0.2127	Yes
9662	8:1 X vs. 20:1 X	-0.1760	-0.3200 to -0.03204	Yes
9663	8:1 X vs. 20:10 X	-0.2650	-0.4090 to -0.1210	Yes
9664	8:1 X vs. 20:40 X	-0.1323	-0.2763 to 0.01163	No
9665	8:1 X vs. 20:50 X	-0.1763	-0.3203 to -0.03237	Yes
9666	8:1 X vs. 21:0 X	-0.3800	-0.5240 to -0.2360	Yes
9667	8:1 X vs. 21:1 X	-0.3330	-0.4770 to -0.1890	Yes
9668	8:1 X vs. 21:10 X	-0.3300	-0.4740 to -0.1860	Yes
9669	8:1 X vs. 21:40 X	-0.2053	-0.3493 to -0.06137	Yes
9670	8:1 X vs. 21:50 X	-0.2957	-0.4396 to -0.1517	Yes
9671	8:1 X vs. 22:0 X	-0.4217	-0.5656 to -0.2777	Yes
9672	8:1 X vs. 22:1 X	-0.4163	-0.5603 to -0.2724	Yes
9673	8:1 X vs. 22:10 X	-0.4117	-0.5556 to -0.2677	Yes
9674	8:1 X vs. 22:40 X	-0.3120	-0.4560 to -0.1680	Yes
9675	8:1 X vs. 22:50 X	-0.3747	-0.5186 to -0.2307	Yes

	2way ANOVA Multiple comparisons	A Data Set-A Y	B Data Set-B Y	C Data Set-C Y
9676	8:1 X vs. 23:0 X	-0.4870	-0.6310 to -0.3430	Yes
9677	8:1 X vs. 23:1 X	-0.6767	-0.8376 to -0.5157	Yes
9678	8:1 X vs. 23:10 X	-0.4263	-0.5703 to -0.2824	Yes
9679	8:1 X vs. 23:40 X	-0.3067	-0.4676 to -0.1457	Yes
9680	8:1 X vs. 23:50 X	-0.3407	-0.4846 to -0.1967	Yes
9681	8:1 X vs. 24:0 X	-0.6043	-0.7483 to -0.4604	Yes
9682	8:1 X vs. 24:1 X	-0.7607	-0.9216 to -0.5997	Yes
9683	8:1 X vs. 24:10 X	-0.5882	-0.7491 to -0.4272	Yes
9684	8:1 X vs. 24:40 X	-0.3230	-0.4670 to -0.1790	Yes
9685	8:1 X vs. 24:50 X	-0.2447	-0.3886 to -0.1007	Yes
9686	8:1 X vs. 25:0 X	-0.9417	-1.103 to -0.7807	Yes
9687	8:1 X vs. 25:1 X	-1.131	-1.292 to -0.9702	Yes
9688	8:1 X vs. 25:10 X	-0.4927	-0.6963 to -0.2891	Yes
9689	8:1 X vs. 25:40 X	-0.3250	-0.4690 to -0.1810	Yes
9690	8:1 X vs. 25:50 X	-0.2993	-0.4433 to -0.1554	Yes
9691	8:1 X vs. 26:0 X	-1.154	-1.315 to -0.9932	Yes
9692	8:1 X vs. 26:1 X	-1.267	-1.428 to -1.106	Yes
9693	8:1 X vs. 26:10 X	-0.9537	-1.115 to -0.7927	Yes
9694	8:1 X vs. 26:40 X	-0.3993	-0.5433 to -0.2554	Yes
9695	8:1 X vs. 26:50 X	-0.2597	-0.4036 to -0.1157	Yes
9696	8:1 X vs. 27:0 X	-1.342	-1.486 to -1.198	Yes
9697	8:1 X vs. 27:1 X	-1.433	-1.594 to -1.272	Yes
9698	8:1 X vs. 27:10 X	-1.245	-1.406 to -1.084	Yes
9699	8:1 X vs. 27:40 X	-0.3993	-0.5433 to -0.2554	Yes
9700	8:1 X vs. 27:50 X	-0.2590	-0.4030 to -0.1150	Yes
9701	8:1 X vs. 28:0 X	-1.291	-1.452 to -1.130	Yes
9702	8:1 X vs. 28:1 X	-1.332	-1.476 to -1.188	Yes
9703	8:1 X vs. 28:10 X	-1.134	-1.295 to -0.9727	Yes
9704	8:1 X vs. 28:40 X	-0.4117	-0.5556 to -0.2677	Yes
9705	8:1 X vs. 28:50 X	-0.3070	-0.4510 to -0.1630	Yes
9706	8:1 X vs. 29:0 X	-1.773	-1.934 to -1.612	Yes
9707	8:1 X vs. 29:1 X	-1.858	-2.019 to -1.697	Yes
9708	8:1 X vs. 29:10 X	-1.072	-1.233 to -0.9112	Yes
9709	8:1 X vs. 29:40 X	-0.5197	-0.6636 to -0.3757	Yes
9710	8:1 X vs. 29:50 X	-0.3083	-0.4523 to -0.1644	Yes
9711	8:1 X vs. 30:0 X	-1.487	-1.648 to -1.326	Yes
9712	8:1 X vs. 30:1 X	-1.809	-1.970 to -1.648	Yes
9713	8:1 X vs. 30:10 X	-1.147	-1.308 to -0.9857	Yes
9714	8:1 X vs. 30:40 X	-0.4467	-0.5906 to -0.3027	Yes
9715	8:1 X vs. 30:50 X	-0.3607	-0.5046 to -0.2167	Yes
9716	8:1 X vs. 31:0 X	-1.805	-1.966 to -1.644	Yes
9717	8:1 X vs. 31:1 X	-1.796	-1.957 to -1.635	Yes
9718	8:1 X vs. 31:10 X	-1.278	-1.439 to -1.117	Yes
9719	8:1 X vs. 31:40 X	-0.5383	-0.6823 to -0.3944	Yes
9720	8:1 X vs. 31:50 X	-0.3397	-0.4836 to -0.1957	Yes

2way ANOVA<br>Multiple comparisons	A<br>Data Set-A<br>Y	B<br>Data Set-B<br>Y	C<br>Data Set-C<br>Y
9721 8:1 X vs. 32:0 X	-2.397	-2.541 to -2.253	Yes
9722 8:1 X vs. 32:1 X	-1.853	-2.014 to -1.692	Yes
9723 8:1 X vs. 32:10 X	-1.709	-1.853 to -1.565	Yes
9724 8:1 X vs. 32:40 X	-0.5347	-0.6786 to -0.3907	Yes
9725 8:1 X vs. 32:50 X	-0.3393	-0.4833 to -0.1954	Yes
9726 8:1 X vs. 33:0 X	-2.400	-2.544 to -2.256	Yes
9727 8:1 X vs. 33:1 X	-2.215	-2.359 to -2.071	Yes
9728 8:1 X vs. 33:10 X	-1.875	-2.019 to -1.731	Yes
9729 8:1 X vs. 33:40 X	-0.5533	-0.6973 to -0.4094	Yes
9730 8:1 X vs. 33:50 X	-0.3543	-0.4983 to -0.2104	Yes
9731 8:1 X vs. 34:0 X	-2.325	-2.469 to -2.181	Yes
9732 8:1 X vs. 34:1 X	-2.332	-2.476 to -2.188	Yes
9733 8:1 X vs. 34:10 X	-1.966	-2.110 to -1.822	Yes
9734 8:1 X vs. 34:40 X	-0.5653	-0.7093 to -0.4214	Yes
9735 8:1 X vs. 34:50 X	-0.5033	-0.6473 to -0.3594	Yes
9736 8:1 X vs. 35:0 X	-2.674	-2.818 to -2.530	Yes
9737 8:1 X vs. 35:1 X	-2.439	-2.583 to -2.295	Yes
9738 8:1 X vs. 35:10 X	-1.927	-2.071 to -1.783	Yes
9739 8:1 X vs. 35:40 X	-0.5743	-0.7183 to -0.4304	Yes
9740 8:1 X vs. 35:50 X	-0.5160	-0.6600 to -0.3720	Yes
9741 8:1 X vs. 36:0 X	-2.880	-3.041 to -2.719	Yes
9742 8:1 X vs. 36:1 X	-2.576	-2.720 to -2.432	Yes
9743 8:1 X vs. 36:10 X	-1.933	-2.077 to -1.789	Yes
9744 8:1 X vs. 36:40 X	-0.6297	-0.7736 to -0.4857	Yes
9745 8:1 X vs. 36:50 X	-0.4793	-0.6233 to -0.3354	Yes
9746 8:1 X vs. 37:0 X	-3.264	-3.408 to -3.120	Yes
9747 8:1 X vs. 37:1 X	-2.646	-2.790 to -2.502	Yes
9748 8:1 X vs. 37:10 X	-1.935	-2.079 to -1.791	Yes
9749 8:1 X vs. 37:40 X	-0.6670	-0.8110 to -0.5230	Yes
9750 8:1 X vs. 37:50 X	-0.4880	-0.6320 to -0.3440	Yes
9751 8:1 X vs. 38:0 X	-3.209	-3.353 to -3.065	Yes
9752 8:1 X vs. 38:1 X	-2.765	-2.909 to -2.621	Yes
9753 8:1 X vs. 38:10 X	-2.210	-2.354 to -2.066	Yes
9754 8:1 X vs. 38:40 X	-0.6713	-0.8153 to -0.5274	Yes
9755 8:1 X vs. 38:50 X	-0.4920	-0.6360 to -0.3480	Yes
9756 8:1 X vs. 39:0 X	-3.440	-3.584 to -3.296	Yes
9757 8:1 X vs. 39:1 X	-3.331	-3.475 to -3.187	Yes
9758 8:1 X vs. 39:10 X	-2.355	-2.499 to -2.211	Yes
9759 8:1 X vs. 39:40 X	-0.6773	-0.8213 to -0.5334	Yes
9760 8:1 X vs. 39:50 X	-0.5230	-0.6670 to -0.3790	Yes
9761 8:1 X vs. 40:0 X	-3.481	-3.625 to -3.337	Yes
9762 8:1 X vs. 40:1 X	-3.407	-3.551 to -3.263	Yes
9763 8:1 X vs. 40:10 X	-2.611	-2.755 to -2.467	Yes
9764 8:1 X vs. 40:40 X	-0.6820	-0.8260 to -0.5380	Yes
9765 8:1 X vs. 40:50 X	-0.5293	-0.6733 to -0.3854	Yes

	2way ANOVA<br>Multiple comparisons	A<br>Data Set-A<br>Y	B<br>Data Set-B<br>Y	C<br>Data Set-C<br>Y
9766	8:1 X vs. 41:0 X	-3.522	-3.666 to -3.378	Yes
9767	8:1 X vs. 41:1 X	-3.521	-3.665 to -3.377	Yes
9768	8:1 X vs. 41:10 X	-2.665	-2.809 to -2.521	Yes
9769	8:1 X vs. 41:40 X	-0.6293	-0.7733 to -0.4854	Yes
9770	8:1 X vs. 41:50 X	-0.5420	-0.6860 to -0.3980	Yes
9771	8:1 X vs. 42:0 X	-3.663	-3.807 to -3.519	Yes
9772	8:1 X vs. 42:1 X	-3.683	-3.827 to -3.539	Yes
9773	8:1 X vs. 42:10 X	-2.665	-2.809 to -2.521	Yes
9774	8:1 X vs. 42:40 X	-0.6420	-0.7860 to -0.4980	Yes
9775	8:1 X vs. 42:50 X	-0.5453	-0.6893 to -0.4014	Yes
9776	8:1 X vs. 43:0 X	-3.753	-3.897 to -3.609	Yes
9777	8:1 X vs. 43:1 X	-3.668	-3.812 to -3.524	Yes
9778	8:1 X vs. 43:10 X	-3.383	-3.527 to -3.239	Yes
9779	8:1 X vs. 43:40 X	-0.6403	-0.7843 to -0.4964	Yes
9780	8:1 X vs. 43:50 X	-0.5457	-0.6896 to -0.4017	Yes
9781	8:1 X vs. 44:0 X	-3.777	-3.921 to -3.633	Yes
9782	8:1 X vs. 44:1 X	-3.667	-3.811 to -3.523	Yes
9783	8:1 X vs. 44:10 X	-3.447	-3.591 to -3.303	Yes
9784	8:1 X vs. 44:40 X	-0.6423	-0.7863 to -0.4984	Yes
9785	8:1 X vs. 44:50 X	-0.4780	-0.6220 to -0.3340	Yes
9786	8:1 X vs. 45:0 X	-3.642	-3.786 to -3.498	Yes
9787	8:1 X vs. 45:1 X	-3.444	-3.588 to -3.300	Yes
9788	8:1 X vs. 45:10 X	-3.326	-3.470 to -3.182	Yes
9789	8:1 X vs. 45:40 X	-0.6437	-0.7876 to -0.4997	Yes
9790	8:1 X vs. 45:50 X	-0.4750	-0.6190 to -0.3310	Yes
9791	8:1 X vs. 46:0 X	-3.446	-3.590 to -3.302	Yes
9792	8:1 X vs. 46:1 X	-3.413	-3.557 to -3.269	Yes
9793	8:1 X vs. 46:10 X	-3.321	-3.465 to -3.177	Yes
9794	8:1 X vs. 46:40 X	-0.6573	-0.8013 to -0.5134	Yes
9795	8:1 X vs. 46:50 X	-0.4680	-0.6120 to -0.3240	Yes
9796	8:1 X vs. 47:0 X	-3.422	-3.566 to -3.278	Yes
9797	8:1 X vs. 47:1 X	-3.326	-3.470 to -3.182	Yes
9798	8:1 X vs. 47:10 X	-3.307	-3.451 to -3.163	Yes
9799	8:1 X vs. 47:40 X	-0.6673	-0.8113 to -0.5234	Yes
9800	8:1 X vs. 47:50 X	-0.4260	-0.5700 to -0.2820	Yes
9801	8:1 X vs. 48:0 X	-3.409	-3.570 to -3.248	Yes
9802	8:1 X vs. 48:1 X	-3.313	-3.457 to -3.169	Yes
9803	8:1 X vs. 48:10 X	-3.307	-3.451 to -3.163	Yes
9804	8:1 X vs. 48:40 X	-0.6737	-0.8176 to -0.5297	Yes
9805	8:1 X vs. 48:50 X	-0.4333	-0.5773 to -0.2894	Yes
9806	8:1 X vs. 49:0 X	-3.409	-3.553 to -3.265	Yes
9807	8:1 X vs. 49:1 X	-3.313	-3.457 to -3.169	Yes
9808	8:1 X vs. 49:10 X	-3.307	-3.451 to -3.163	Yes
9809	8:1 X vs. 49:40 X	-0.6780	-0.8220 to -0.5340	Yes
9810	8:1 X vs. 49:50 X	-0.4327	-0.5766 to -0.2887	Yes

	2way ANOVA<br>Multiple comparisons	A<br>Data Set-A<br>Y	B<br>Data Set-B<br>Y	C<br>Data Set-C<br>Y
9811	8:**1 X** vs. 50:**0 X**	-3.409	-3.553 to -3.265	Yes
9812	8:**1 X** vs. 50:**1 X**	-3.313	-3.457 to -3.169	Yes
9813	8:**1 X** vs. 50:**10 X**	-3.307	-3.451 to -3.163	Yes
9814	8:**1 X** vs. 50:**40 X**	-0.6857	-0.8296 to -0.5417	Yes
9815	8:**1 X** vs. 50:**50 X**	-0.4323	-0.5763 to -0.2884	Yes
9816	8:**10 X** vs. 8:**40 X**	-0.0006667	-0.1446 to 0.1433	No
9817	8:**10 X** vs. 8:**50 X**	0.002333	-0.1416 to 0.1463	No
9818	8:**10 X** vs. 9:**0 X**	-0.0140	-0.1580 to 0.1300	No
9819	8:**10 X** vs. 9:**1 X**	-0.0220	-0.1660 to 0.1220	No
9820	8:**10 X** vs. 9:**10 X**	-0.0230	-0.1670 to 0.1210	No
9821	8:**10 X** vs. 9:**40 X**	-0.005333	-0.1493 to 0.1386	No
9822	8:**10 X** vs. 9:**50 X**	0.001333	-0.1426 to 0.1453	No
9823	8:**10 X** vs. 10:**0 X**	-0.01567	-0.1596 to 0.1283	No
9824	8:**10 X** vs. 10:**1 X**	-0.02267	-0.1666 to 0.1213	No
9825	8:**10 X** vs. 10:**10 X**	-0.02533	-0.1693 to 0.1186	No
9826	8:**10 X** vs. 10:**40 X**	-0.0150	-0.1590 to 0.1290	No
9827	8:**10 X** vs. 10:**50 X**	-0.001667	-0.1456 to 0.1423	No
9828	8:**10 X** vs. 11:**0 X**	-0.02533	-0.1693 to 0.1186	No
9829	8:**10 X** vs. 11:**1 X**	-0.0270	-0.1710 to 0.1170	No
9830	8:**10 X** vs. 11:**10 X**	-0.02733	-0.1713 to 0.1166	No
9831	8:**10 X** vs. 11:**40 X**	-0.0190	-0.1630 to 0.1250	No
9832	8:**10 X** vs. 11:**50 X**	-0.007000	-0.1510 to 0.1370	No
9833	8:**10 X** vs. 12:**0 X**	-0.0260	-0.1700 to 0.1180	No
9834	8:**10 X** vs. 12:**1 X**	-0.008333	-0.1523 to 0.1356	No
9835	8:**10 X** vs. 12:**10 X**	-0.0260	-0.1700 to 0.1180	No
9836	8:**10 X** vs. 12:**40 X**	-0.01233	-0.1563 to 0.1316	No
9837	8:**10 X** vs. 12:**50 X**	-0.01567	-0.1596 to 0.1283	No
9838	8:**10 X** vs. 13:**0 X**	-0.0200	-0.1640 to 0.1240	No
9839	8:**10 X** vs. 13:**1 X**	-0.007333	-0.1513 to 0.1366	No
9840	8:**10 X** vs. 13:**10 X**	-0.02933	-0.1733 to 0.1146	No
9841	8:**10 X** vs. 13:**40 X**	-0.02567	-0.1696 to 0.1183	No
9842	8:**10 X** vs. 13:**50 X**	-0.02667	-0.1706 to 0.1173	No
9843	8:**10 X** vs. 14:**0 X**	-0.02867	-0.1726 to 0.1153	No
9844	8:**10 X** vs. 14:**1 X**	-0.02033	-0.1643 to 0.1236	No
9845	8:**10 X** vs. 14:**10 X**	-0.03433	-0.1783 to 0.1096	No
9846	8:**10 X** vs. 14:**40 X**	-0.0310	-0.1750 to 0.1130	No
9847	8:**10 X** vs. 14:**50 X**	-0.0350	-0.1790 to 0.1090	No
9848	8:**10 X** vs. 15:**0 X**	-0.0410	-0.1850 to 0.1030	No
9849	8:**10 X** vs. 15:**1 X**	-0.0210	-0.1650 to 0.1230	No
9850	8:**10 X** vs. 15:**10 X**	-0.04833	-0.1923 to 0.09563	No
9851	8:**10 X** vs. 15:**40 X**	-0.03067	-0.1746 to 0.1133	No
9852	8:**10 X** vs. 15:**50 X**	-0.04833	-0.1923 to 0.09563	No
9853	8:**10 X** vs. 16:**0 X**	-0.1333	-0.2773 to 0.01063	No
9854	8:**10 X** vs. 16:**1 X**	-0.02433	-0.1683 to 0.1196	No
9855	8:**10 X** vs. 16:**10 X**	-0.0500	-0.1940 to 0.09396	No

	2way ANOVA<br>Multiple comparisons	A<br>Data Set-A<br>Y	B<br>Data Set-B<br>Y	C<br>Data Set-C<br>Y
**9856**	8:**10 X** vs. 16:**40 X**	-0.06933	-0.2133 to 0.07463	No
**9857**	8:**10 X** vs. 16:**50 X**	-0.1117	-0.2556 to 0.03229	No
**9858**	8:**10 X** vs. 17:**0 X**	-0.1860	-0.3300 to -0.04204	Yes
**9859**	8:**10 X** vs. 17:**1 X**	-0.1003	-0.2443 to 0.04363	No
**9860**	8:**10 X** vs. 17:**10 X**	-0.1570	-0.3010 to -0.01304	Yes
**9861**	8:**10 X** vs. 17:**40 X**	-0.08033	-0.2243 to 0.06363	No
**9862**	8:**10 X** vs. 17:**50 X**	-0.1257	-0.2696 to 0.01829	No
**9863**	8:**10 X** vs. 18:**0 X**	-0.2347	-0.3786 to -0.09071	Yes
**9864**	8:**10 X** vs. 18:**1 X**	-0.1430	-0.2870 to 0.0009598	No
**9865**	8:**10 X** vs. 18:**10 X**	-0.2353	-0.3793 to -0.09137	Yes
**9866**	8:**10 X** vs. 18:**40 X**	-0.08467	-0.2286 to 0.05929	No
**9867**	8:**10 X** vs. 18:**50 X**	-0.1297	-0.2736 to 0.01429	No
**9868**	8:**10 X** vs. 19:**0 X**	-0.2697	-0.4136 to -0.1257	Yes
**9869**	8:**10 X** vs. 19:**1 X**	-0.2577	-0.4016 to -0.1137	Yes
**9870**	8:**10 X** vs. 19:**10 X**	-0.2553	-0.3993 to -0.1114	Yes
**9871**	8:**10 X** vs. 19:**40 X**	-0.1133	-0.2573 to 0.03063	No
**9872**	8:**10 X** vs. 19:**50 X**	-0.1243	-0.2683 to 0.01963	No
**9873**	8:**10 X** vs. 20:**0 X**	-0.3493	-0.4933 to -0.2054	Yes
**9874**	8:**10 X** vs. 20:**1 X**	-0.1687	-0.3126 to -0.02471	Yes
**9875**	8:**10 X** vs. 20:**10 X**	-0.2577	-0.4016 to -0.1137	Yes
**9876**	8:**10 X** vs. 20:**40 X**	-0.1250	-0.2690 to 0.01896	No
**9877**	8:**10 X** vs. 20:**50 X**	-0.1690	-0.3130 to -0.02504	Yes
**9878**	8:**10 X** vs. 21:**0 X**	-0.3727	-0.5166 to -0.2287	Yes
**9879**	8:**10 X** vs. 21:**1 X**	-0.3257	-0.4696 to -0.1817	Yes
**9880**	8:**10 X** vs. 21:**10 X**	-0.3227	-0.4666 to -0.1787	Yes
**9881**	8:**10 X** vs. 21:**40 X**	-0.1980	-0.3420 to -0.05404	Yes
**9882**	8:**10 X** vs. 21:**50 X**	-0.2883	-0.4323 to -0.1444	Yes
**9883**	8:**10 X** vs. 22:**0 X**	-0.4143	-0.5583 to -0.2704	Yes
**9884**	8:**10 X** vs. 22:**1 X**	-0.4090	-0.5530 to -0.2650	Yes
**9885**	8:**10 X** vs. 22:**10 X**	-0.4043	-0.5483 to -0.2604	Yes
**9886**	8:**10 X** vs. 22:**40 X**	-0.3047	-0.4486 to -0.1607	Yes
**9887**	8:**10 X** vs. 22:**50 X**	-0.3673	-0.5113 to -0.2234	Yes
**9888**	8:**10 X** vs. 23:**0 X**	-0.4797	-0.6236 to -0.3357	Yes
**9889**	8:**10 X** vs. 23:**1 X**	-0.6693	-0.8303 to -0.5084	Yes
**9890**	8:**10 X** vs. 23:**10 X**	-0.4190	-0.5630 to -0.2750	Yes
**9891**	8:**10 X** vs. 23:**40 X**	-0.2993	-0.4603 to -0.1384	Yes
**9892**	8:**10 X** vs. 23:**50 X**	-0.3333	-0.4773 to -0.1894	Yes
**9893**	8:**10 X** vs. 24:**0 X**	-0.5970	-0.7410 to -0.4530	Yes
**9894**	8:**10 X** vs. 24:**1 X**	-0.7533	-0.9143 to -0.5924	Yes
**9895**	8:**10 X** vs. 24:**10 X**	-0.5808	-0.7418 to -0.4199	Yes
**9896**	8:**10 X** vs. 24:**40 X**	-0.3157	-0.4596 to -0.1717	Yes
**9897**	8:**10 X** vs. 24:**50 X**	-0.2373	-0.3813 to -0.09337	Yes
**9898**	8:**10 X** vs. 25:**0 X**	-0.9343	-1.095 to -0.7734	Yes
**9899**	8:**10 X** vs. 25:**1 X**	-1.124	-1.285 to -0.9629	Yes
**9900**	8:**10 X** vs. 25:**10 X**	-0.4853	-0.6889 to -0.2817	Yes

	2way ANOVA<br>Multiple comparisons	A<br>Data Set-A<br>Y	B<br>Data Set-B<br>Y	C<br>Data Set-C<br>Y
9901	8:**10 X** vs. 25:**40 X**	-0.3177	-0.4616 to -0.1737	Yes
9902	8:**10 X** vs. 25:**50 X**	-0.2920	-0.4360 to -0.1480	Yes
9903	8:**10 X** vs. 26:**0 X**	-1.147	-1.308 to -0.9859	Yes
9904	8:**10 X** vs. 26:**1 X**	-1.260	-1.421 to -1.099	Yes
9905	8:**10 X** vs. 26:**10 X**	-0.9463	-1.107 to -0.7854	Yes
9906	8:**10 X** vs. 26:**40 X**	-0.3920	-0.5360 to -0.2480	Yes
9907	8:**10 X** vs. 26:**50 X**	-0.2523	-0.3963 to -0.1084	Yes
9908	8:**10 X** vs. 27:**0 X**	-1.335	-1.479 to -1.191	Yes
9909	8:**10 X** vs. 27:**1 X**	-1.425	-1.586 to -1.264	Yes
9910	8:**10 X** vs. 27:**10 X**	-1.237	-1.398 to -1.076	Yes
9911	8:**10 X** vs. 27:**40 X**	-0.3920	-0.5360 to -0.2480	Yes
9912	8:**10 X** vs. 27:**50 X**	-0.2517	-0.3956 to -0.1077	Yes
9913	8:**10 X** vs. 28:**0 X**	-1.284	-1.445 to -1.123	Yes
9914	8:**10 X** vs. 28:**1 X**	-1.325	-1.469 to -1.181	Yes
9915	8:**10 X** vs. 28:**10 X**	-1.126	-1.287 to -0.9654	Yes
9916	8:**10 X** vs. 28:**40 X**	-0.4043	-0.5483 to -0.2604	Yes
9917	8:**10 X** vs. 28:**50 X**	-0.2997	-0.4436 to -0.1557	Yes
9918	8:**10 X** vs. 29:**0 X**	-1.765	-1.926 to -1.604	Yes
9919	8:**10 X** vs. 29:**1 X**	-1.850	-2.011 to -1.689	Yes
9920	8:**10 X** vs. 29:**10 X**	-1.065	-1.226 to -0.9039	Yes
9921	8:**10 X** vs. 29:**40 X**	-0.5123	-0.6563 to -0.3684	Yes
9922	8:**10 X** vs. 29:**50 X**	-0.3010	-0.4450 to -0.1570	Yes
9923	8:**10 X** vs. 30:**0 X**	-1.480	-1.641 to -1.319	Yes
9924	8:**10 X** vs. 30:**1 X**	-1.801	-1.962 to -1.640	Yes
9925	8:**10 X** vs. 30:**10 X**	-1.139	-1.300 to -0.9784	Yes
9926	8:**10 X** vs. 30:**40 X**	-0.4393	-0.5833 to -0.2954	Yes
9927	8:**10 X** vs. 30:**50 X**	-0.3533	-0.4973 to -0.2094	Yes
9928	8:**10 X** vs. 31:**0 X**	-1.798	-1.959 to -1.637	Yes
9929	8:**10 X** vs. 31:**1 X**	-1.788	-1.949 to -1.627	Yes
9930	8:**10 X** vs. 31:**10 X**	-1.271	-1.432 to -1.110	Yes
9931	8:**10 X** vs. 31:**40 X**	-0.5310	-0.6750 to -0.3870	Yes
9932	8:**10 X** vs. 31:**50 X**	-0.3323	-0.4763 to -0.1884	Yes
9933	8:**10 X** vs. 32:**0 X**	-2.389	-2.533 to -2.245	Yes
9934	8:**10 X** vs. 32:**1 X**	-1.845	-2.006 to -1.684	Yes
9935	8:**10 X** vs. 32:**10 X**	-1.701	-1.845 to -1.557	Yes
9936	8:**10 X** vs. 32:**40 X**	-0.5273	-0.6713 to -0.3834	Yes
9937	8:**10 X** vs. 32:**50 X**	-0.3320	-0.4760 to -0.1880	Yes
9938	8:**10 X** vs. 33:**0 X**	-2.393	-2.537 to -2.249	Yes
9939	8:**10 X** vs. 33:**1 X**	-2.208	-2.352 to -2.064	Yes
9940	8:**10 X** vs. 33:**10 X**	-1.868	-2.012 to -1.724	Yes
9941	8:**10 X** vs. 33:**40 X**	-0.5460	-0.6900 to -0.4020	Yes
9942	8:**10 X** vs. 33:**50 X**	-0.3470	-0.4910 to -0.2030	Yes
9943	8:**10 X** vs. 34:**0 X**	-2.318	-2.462 to -2.174	Yes
9944	8:**10 X** vs. 34:**1 X**	-2.325	-2.469 to -2.181	Yes
9945	8:**10 X** vs. 34:**10 X**	-1.958	-2.102 to -1.814	Yes

	2way ANOVA<br>Multiple comparisons	A<br>Data Set-A<br>Y	B<br>Data Set-B<br>Y	C<br>Data Set-C<br>Y
9946	8:10 X vs. 34:40 X	-0.5580	-0.7020 to -0.4140	Yes
9947	8:10 X vs. 34:50 X	-0.4960	-0.6400 to -0.3520	Yes
9948	8:10 X vs. 35:0 X	-2.666	-2.810 to -2.522	Yes
9949	8:10 X vs. 35:1 X	-2.432	-2.576 to -2.288	Yes
9950	8:10 X vs. 35:10 X	-1.920	-2.064 to -1.776	Yes
9951	8:10 X vs. 35:40 X	-0.5670	-0.7110 to -0.4230	Yes
9952	8:10 X vs. 35:50 X	-0.5087	-0.6526 to -0.3647	Yes
9953	8:10 X vs. 36:0 X	-2.873	-3.034 to -2.712	Yes
9954	8:10 X vs. 36:1 X	-2.569	-2.713 to -2.425	Yes
9955	8:10 X vs. 36:10 X	-1.926	-2.070 to -1.782	Yes
9956	8:10 X vs. 36:40 X	-0.6223	-0.7663 to -0.4784	Yes
9957	8:10 X vs. 36:50 X	-0.4720	-0.6160 to -0.3280	Yes
9958	8:10 X vs. 37:0 X	-3.257	-3.401 to -3.113	Yes
9959	8:10 X vs. 37:1 X	-2.639	-2.783 to -2.495	Yes
9960	8:10 X vs. 37:10 X	-1.927	-2.071 to -1.783	Yes
9961	8:10 X vs. 37:40 X	-0.6597	-0.8036 to -0.5157	Yes
9962	8:10 X vs. 37:50 X	-0.4807	-0.6246 to -0.3367	Yes
9963	8:10 X vs. 38:0 X	-3.202	-3.346 to -3.058	Yes
9964	8:10 X vs. 38:1 X	-2.757	-2.901 to -2.613	Yes
9965	8:10 X vs. 38:10 X	-2.203	-2.347 to -2.059	Yes
9966	8:10 X vs. 38:40 X	-0.6640	-0.8080 to -0.5200	Yes
9967	8:10 X vs. 38:50 X	-0.4847	-0.6286 to -0.3407	Yes
9968	8:10 X vs. 39:0 X	-3.432	-3.576 to -3.288	Yes
9969	8:10 X vs. 39:1 X	-3.324	-3.468 to -3.180	Yes
9970	8:10 X vs. 39:10 X	-2.348	-2.492 to -2.204	Yes
9971	8:10 X vs. 39:40 X	-0.6700	-0.8140 to -0.5260	Yes
9972	8:10 X vs. 39:50 X	-0.5157	-0.6596 to -0.3717	Yes
9973	8:10 X vs. 40:0 X	-3.473	-3.617 to -3.329	Yes
9974	8:10 X vs. 40:1 X	-3.399	-3.543 to -3.255	Yes
9975	8:10 X vs. 40:10 X	-2.604	-2.748 to -2.460	Yes
9976	8:10 X vs. 40:40 X	-0.6747	-0.8186 to -0.5307	Yes
9977	8:10 X vs. 40:50 X	-0.5220	-0.6660 to -0.3780	Yes
9978	8:10 X vs. 41:0 X	-3.514	-3.658 to -3.370	Yes
9979	8:10 X vs. 41:1 X	-3.513	-3.657 to -3.369	Yes
9980	8:10 X vs. 41:10 X	-2.658	-2.802 to -2.514	Yes
9981	8:10 X vs. 41:40 X	-0.6220	-0.7660 to -0.4780	Yes
9982	8:10 X vs. 41:50 X	-0.5347	-0.6786 to -0.3907	Yes
9983	8:10 X vs. 42:0 X	-3.656	-3.800 to -3.512	Yes
9984	8:10 X vs. 42:1 X	-3.676	-3.820 to -3.532	Yes
9985	8:10 X vs. 42:10 X	-2.658	-2.802 to -2.514	Yes
9986	8:10 X vs. 42:40 X	-0.6347	-0.7786 to -0.4907	Yes
9987	8:10 X vs. 42:50 X	-0.5380	-0.6820 to -0.3940	Yes
9988	8:10 X vs. 43:0 X	-3.746	-3.890 to -3.602	Yes
9989	8:10 X vs. 43:1 X	-3.660	-3.804 to -3.516	Yes
9990	8:10 X vs. 43:10 X	-3.376	-3.520 to -3.232	Yes

	2way ANOVA<br>Multiple comparisons	A<br>Data Set-A<br>Y	B<br>Data Set-B<br>Y	C<br>Data Set-C<br>Y
9991	8:10 X vs. 43:40 X	-0.6330	-0.7770 to -0.4890	Yes
9992	8:10 X vs. 43:50 X	-0.5383	-0.6823 to -0.3944	Yes
9993	8:10 X vs. 44:0 X	-3.770	-3.914 to -3.626	Yes
9994	8:10 X vs. 44:1 X	-3.660	-3.804 to -3.516	Yes
9995	8:10 X vs. 44:10 X	-3.439	-3.583 to -3.295	Yes
9996	8:10 X vs. 44:40 X	-0.6350	-0.7790 to -0.4910	Yes
9997	8:10 X vs. 44:50 X	-0.4707	-0.6146 to -0.3267	Yes
9998	8:10 X vs. 45:0 X	-3.635	-3.779 to -3.491	Yes
9999	8:10 X vs. 45:1 X	-3.437	-3.581 to -3.293	Yes
10000	8:10 X vs. 45:10 X	-3.319	-3.463 to -3.175	Yes
10001	8:10 X vs. 45:40 X	-0.6363	-0.7803 to -0.4924	Yes
10002	8:10 X vs. 45:50 X	-0.4677	-0.6116 to -0.3237	Yes
10003	8:10 X vs. 46:0 X	-3.439	-3.583 to -3.295	Yes
10004	8:10 X vs. 46:1 X	-3.406	-3.550 to -3.262	Yes
10005	8:10 X vs. 46:10 X	-3.314	-3.458 to -3.170	Yes
10006	8:10 X vs. 46:40 X	-0.6500	-0.7940 to -0.5060	Yes
10007	8:10 X vs. 46:50 X	-0.4607	-0.6046 to -0.3167	Yes
10008	8:10 X vs. 47:0 X	-3.415	-3.559 to -3.271	Yes
10009	8:10 X vs. 47:1 X	-3.319	-3.463 to -3.175	Yes
10010	8:10 X vs. 47:10 X	-3.300	-3.444 to -3.156	Yes
10011	8:10 X vs. 47:40 X	-0.6600	-0.8040 to -0.5160	Yes
10012	8:10 X vs. 47:50 X	-0.4187	-0.5626 to -0.2747	Yes
10013	8:10 X vs. 48:0 X	-3.402	-3.563 to -3.241	Yes
10014	8:10 X vs. 48:1 X	-3.306	-3.450 to -3.162	Yes
10015	8:10 X vs. 48:10 X	-3.300	-3.444 to -3.156	Yes
10016	8:10 X vs. 48:40 X	-0.6663	-0.8103 to -0.5224	Yes
10017	8:10 X vs. 48:50 X	-0.4260	-0.5700 to -0.2820	Yes
10018	8:10 X vs. 49:0 X	-3.402	-3.546 to -3.258	Yes
10019	8:10 X vs. 49:1 X	-3.306	-3.450 to -3.162	Yes
10020	8:10 X vs. 49:10 X	-3.300	-3.444 to -3.156	Yes
10021	8:10 X vs. 49:40 X	-0.6707	-0.8146 to -0.5267	Yes
10022	8:10 X vs. 49:50 X	-0.4253	-0.5693 to -0.2814	Yes
10023	8:10 X vs. 50:0 X	-3.402	-3.546 to -3.258	Yes
10024	8:10 X vs. 50:1 X	-3.306	-3.450 to -3.162	Yes
10025	8:10 X vs. 50:10 X	-3.300	-3.444 to -3.156	Yes
10026	8:10 X vs. 50:40 X	-0.6783	-0.8223 to -0.5344	Yes
10027	8:10 X vs. 50:50 X	-0.4250	-0.5690 to -0.2810	Yes
10028	8:40 X vs. 8:50 X	0.003000	-0.1410 to 0.1470	No
10029	8:40 X vs. 9:0 X	-0.01333	-0.1573 to 0.1306	No
10030	8:40 X vs. 9:1 X	-0.02133	-0.1653 to 0.1226	No
10031	8:40 X vs. 9:10 X	-0.02233	-0.1663 to 0.1216	No
10032	8:40 X vs. 9:40 X	-0.004667	-0.1486 to 0.1393	No
10033	8:40 X vs. 9:50 X	0.002000	-0.1420 to 0.1460	No
10034	8:40 X vs. 10:0 X	-0.0150	-0.1590 to 0.1290	No
10035	8:40 X vs. 10:1 X	-0.0220	-0.1660 to 0.1220	No

2way ANOVA<br>Multiple comparisons	A<br>Data Set-A<br>Y	B<br>Data Set-B<br>Y	C<br>Data Set-C<br>Y
10036 8:40 X vs. 10:10 X	-0.02467	-0.1686 to 0.1193	No
10037 8:40 X vs. 10:40 X	-0.01433	-0.1583 to 0.1296	No
10038 8:40 X vs. 10:50 X	-0.0010	-0.1450 to 0.1430	No
10039 8:40 X vs. 11:0 X	-0.02467	-0.1686 to 0.1193	No
10040 8:40 X vs. 11:1 X	-0.02633	-0.1703 to 0.1176	No
10041 8:40 X vs. 11:10 X	-0.02667	-0.1706 to 0.1173	No
10042 8:40 X vs. 11:40 X	-0.01833	-0.1623 to 0.1256	No
10043 8:40 X vs. 11:50 X	-0.006333	-0.1503 to 0.1376	No
10044 8:40 X vs. 12:0 X	-0.02533	-0.1693 to 0.1186	No
10045 8:40 X vs. 12:1 X	-0.007667	-0.1516 to 0.1363	No
10046 8:40 X vs. 12:10 X	-0.02533	-0.1693 to 0.1186	No
10047 8:40 X vs. 12:40 X	-0.01167	-0.1556 to 0.1323	No
10048 8:40 X vs. 12:50 X	-0.0150	-0.1590 to 0.1290	No
10049 8:40 X vs. 13:0 X	-0.01933	-0.1633 to 0.1246	No
10050 8:40 X vs. 13:1 X	-0.006667	-0.1506 to 0.1373	No
10051 8:40 X vs. 13:10 X	-0.02867	-0.1726 to 0.1153	No
10052 8:40 X vs. 13:40 X	-0.0250	-0.1690 to 0.1190	No
10053 8:40 X vs. 13:50 X	-0.0260	-0.1700 to 0.1180	No
10054 8:40 X vs. 14:0 X	-0.0280	-0.1720 to 0.1160	No
10055 8:40 X vs. 14:1 X	-0.01967	-0.1636 to 0.1243	No
10056 8:40 X vs. 14:10 X	-0.03367	-0.1776 to 0.1103	No
10057 8:40 X vs. 14:40 X	-0.03033	-0.1743 to 0.1136	No
10058 8:40 X vs. 14:50 X	-0.03433	-0.1783 to 0.1096	No
10059 8:40 X vs. 15:0 X	-0.04033	-0.1843 to 0.1036	No
10060 8:40 X vs. 15:1 X	-0.02033	-0.1643 to 0.1236	No
10061 8:40 X vs. 15:10 X	-0.04767	-0.1916 to 0.09629	No
10062 8:40 X vs. 15:40 X	-0.0300	-0.1740 to 0.1140	No
10063 8:40 X vs. 15:50 X	-0.04767	-0.1916 to 0.09629	No
10064 8:40 X vs. 16:0 X	-0.1327	-0.2766 to 0.01129	No
10065 8:40 X vs. 16:1 X	-0.02367	-0.1676 to 0.1203	No
10066 8:40 X vs. 16:10 X	-0.04933	-0.1933 to 0.09463	No
10067 8:40 X vs. 16:40 X	-0.06867	-0.2126 to 0.07529	No
10068 8:40 X vs. 16:50 X	-0.1110	-0.2550 to 0.03296	No
10069 8:40 X vs. 17:0 X	-0.1853	-0.3293 to -0.04137	Yes
10070 8:40 X vs. 17:1 X	-0.09967	-0.2436 to 0.04429	No
10071 8:40 X vs. 17:10 X	-0.1563	-0.3003 to -0.01237	Yes
10072 8:40 X vs. 17:40 X	-0.07967	-0.2236 to 0.06429	No
10073 8:40 X vs. 17:50 X	-0.1250	-0.2690 to 0.01896	No
10074 8:40 X vs. 18:0 X	-0.2340	-0.3780 to -0.09004	Yes
10075 8:40 X vs. 18:1 X	-0.1423	-0.2863 to 0.001626	No
10076 8:40 X vs. 18:10 X	-0.2347	-0.3786 to -0.09071	Yes
10077 8:40 X vs. 18:40 X	-0.0840	-0.2280 to 0.05996	No
10078 8:40 X vs. 18:50 X	-0.1290	-0.2730 to 0.01496	No
10079 8:40 X vs. 19:0 X	-0.2690	-0.4130 to -0.1250	Yes
10080 8:40 X vs. 19:1 X	-0.2570	-0.4010 to -0.1130	Yes

	2way ANOVA Multiple comparisons	A Data Set-A Y	B Data Set-B Y	C Data Set-C Y
10081	8:**40 X** vs. 19:**10 X**	-0.2547	-0.3986 to -0.1107	Yes
10082	8:**40 X** vs. 19:**40 X**	-0.1127	-0.2566 to 0.03129	No
10083	8:**40 X** vs. 19:**50 X**	-0.1237	-0.2676 to 0.02029	No
10084	8:**40 X** vs. 20:**0 X**	-0.3487	-0.4926 to -0.2047	Yes
10085	8:**40 X** vs. 20:**1 X**	-0.1680	-0.3120 to -0.02404	Yes
10086	8:**40 X** vs. 20:**10 X**	-0.2570	-0.4010 to -0.1130	Yes
10087	8:**40 X** vs. 20:**40 X**	-0.1243	-0.2683 to 0.01963	No
10088	8:**40 X** vs. 20:**50 X**	-0.1683	-0.3123 to -0.02437	Yes
10089	8:**40 X** vs. 21:**0 X**	-0.3720	-0.5160 to -0.2280	Yes
10090	8:**40 X** vs. 21:**1 X**	-0.3250	-0.4690 to -0.1810	Yes
10091	8:**40 X** vs. 21:**10 X**	-0.3220	-0.4660 to -0.1780	Yes
10092	8:**40 X** vs. 21:**40 X**	-0.1973	-0.3413 to -0.05337	Yes
10093	8:**40 X** vs. 21:**50 X**	-0.2877	-0.4316 to -0.1437	Yes
10094	8:**40 X** vs. 22:**0 X**	-0.4137	-0.5576 to -0.2697	Yes
10095	8:**40 X** vs. 22:**1 X**	-0.4083	-0.5523 to -0.2644	Yes
10096	8:**40 X** vs. 22:**10 X**	-0.4037	-0.5476 to -0.2597	Yes
10097	8:**40 X** vs. 22:**40 X**	-0.3040	-0.4480 to -0.1600	Yes
10098	8:**40 X** vs. 22:**50 X**	-0.3667	-0.5106 to -0.2227	Yes
10099	8:**40 X** vs. 23:**0 X**	-0.4790	-0.6230 to -0.3350	Yes
10100	8:**40 X** vs. 23:**1 X**	-0.6687	-0.8296 to -0.5077	Yes
10101	8:**40 X** vs. 23:**10 X**	-0.4183	-0.5623 to -0.2744	Yes
10102	8:**40 X** vs. 23:**40 X**	-0.2987	-0.4596 to -0.1377	Yes
10103	8:**40 X** vs. 23:**50 X**	-0.3327	-0.4766 to -0.1887	Yes
10104	8:**40 X** vs. 24:**0 X**	-0.5963	-0.7403 to -0.4524	Yes
10105	8:**40 X** vs. 24:**1 X**	-0.7527	-0.9136 to -0.5917	Yes
10106	8:**40 X** vs. 24:**10 X**	-0.5802	-0.7411 to -0.4192	Yes
10107	8:**40 X** vs. 24:**40 X**	-0.3150	-0.4590 to -0.1710	Yes
10108	8:**40 X** vs. 24:**50 X**	-0.2367	-0.3806 to -0.09271	Yes
10109	8:**40 X** vs. 25:**0 X**	-0.9337	-1.095 to -0.7727	Yes
10110	8:**40 X** vs. 25:**1 X**	-1.123	-1.284 to -0.9622	Yes
10111	8:**40 X** vs. 25:**10 X**	-0.4847	-0.6883 to -0.2811	Yes
10112	8:**40 X** vs. 25:**40 X**	-0.3170	-0.4610 to -0.1730	Yes
10113	8:**40 X** vs. 25:**50 X**	-0.2913	-0.4353 to -0.1474	Yes
10114	8:**40 X** vs. 26:**0 X**	-1.146	-1.307 to -0.9852	Yes
10115	8:**40 X** vs. 26:**1 X**	-1.259	-1.420 to -1.098	Yes
10116	8:**40 X** vs. 26:**10 X**	-0.9457	-1.107 to -0.7847	Yes
10117	8:**40 X** vs. 26:**40 X**	-0.3913	-0.5353 to -0.2474	Yes
10118	8:**40 X** vs. 26:**50 X**	-0.2517	-0.3956 to -0.1077	Yes
10119	8:**40 X** vs. 27:**0 X**	-1.334	-1.478 to -1.190	Yes
10120	8:**40 X** vs. 27:**1 X**	-1.425	-1.586 to -1.264	Yes
10121	8:**40 X** vs. 27:**10 X**	-1.237	-1.398 to -1.076	Yes
10122	8:**40 X** vs. 27:**40 X**	-0.3913	-0.5353 to -0.2474	Yes
10123	8:**40 X** vs. 27:**50 X**	-0.2510	-0.3950 to -0.1070	Yes
10124	8:**40 X** vs. 28:**0 X**	-1.283	-1.444 to -1.122	Yes
10125	8:**40 X** vs. 28:**1 X**	-1.324	-1.468 to -1.180	Yes

	2way ANOVA<br>Multiple comparisons	A<br>Data Set-A<br>Y	B<br>Data Set-B<br>Y	C<br>Data Set-C<br>Y
10126	8:**40 X** vs. 28:**10 X**	-1.126	-1.287 to -0.9647	Yes
10127	8:**40 X** vs. 28:**40 X**	-0.4037	-0.5476 to -0.2597	Yes
10128	8:**40 X** vs. 28:**50 X**	-0.2990	-0.4430 to -0.1550	Yes
10129	8:**40 X** vs. 29:**0 X**	-1.765	-1.926 to -1.604	Yes
10130	8:**40 X** vs. 29:**1 X**	-1.850	-2.011 to -1.689	Yes
10131	8:**40 X** vs. 29:**10 X**	-1.064	-1.225 to -0.9032	Yes
10132	8:**40 X** vs. 29:**40 X**	-0.5117	-0.6556 to -0.3677	Yes
10133	8:**40 X** vs. 29:**50 X**	-0.3003	-0.4443 to -0.1564	Yes
10134	8:**40 X** vs. 30:**0 X**	-1.479	-1.640 to -1.318	Yes
10135	8:**40 X** vs. 30:**1 X**	-1.801	-1.962 to -1.640	Yes
10136	8:**40 X** vs. 30:**10 X**	-1.139	-1.300 to -0.9777	Yes
10137	8:**40 X** vs. 30:**40 X**	-0.4387	-0.5826 to -0.2947	Yes
10138	8:**40 X** vs. 30:**50 X**	-0.3527	-0.4966 to -0.2087	Yes
10139	8:**40 X** vs. 31:**0 X**	-1.797	-1.958 to -1.636	Yes
10140	8:**40 X** vs. 31:**1 X**	-1.788	-1.949 to -1.627	Yes
10141	8:**40 X** vs. 31:**10 X**	-1.270	-1.431 to -1.109	Yes
10142	8:**40 X** vs. 31:**40 X**	-0.5303	-0.6743 to -0.3864	Yes
10143	8:**40 X** vs. 31:**50 X**	-0.3317	-0.4756 to -0.1877	Yes
10144	8:**40 X** vs. 32:**0 X**	-2.389	-2.533 to -2.245	Yes
10145	8:**40 X** vs. 32:**1 X**	-1.845	-2.006 to -1.684	Yes
10146	8:**40 X** vs. 32:**10 X**	-1.701	-1.845 to -1.557	Yes
10147	8:**40 X** vs. 32:**40 X**	-0.5267	-0.6706 to -0.3827	Yes
10148	8:**40 X** vs. 32:**50 X**	-0.3313	-0.4753 to -0.1874	Yes
10149	8:**40 X** vs. 33:**0 X**	-2.392	-2.536 to -2.248	Yes
10150	8:**40 X** vs. 33:**1 X**	-2.207	-2.351 to -2.063	Yes
10151	8:**40 X** vs. 33:**10 X**	-1.867	-2.011 to -1.723	Yes
10152	8:**40 X** vs. 33:**40 X**	-0.5453	-0.6893 to -0.4014	Yes
10153	8:**40 X** vs. 33:**50 X**	-0.3463	-0.4903 to -0.2024	Yes
10154	8:**40 X** vs. 34:**0 X**	-2.317	-2.461 to -2.173	Yes
10155	8:**40 X** vs. 34:**1 X**	-2.324	-2.468 to -2.180	Yes
10156	8:**40 X** vs. 34:**10 X**	-1.958	-2.102 to -1.814	Yes
10157	8:**40 X** vs. 34:**40 X**	-0.5573	-0.7013 to -0.4134	Yes
10158	8:**40 X** vs. 34:**50 X**	-0.4953	-0.6393 to -0.3514	Yes
10159	8:**40 X** vs. 35:**0 X**	-2.666	-2.810 to -2.522	Yes
10160	8:**40 X** vs. 35:**1 X**	-2.431	-2.575 to -2.287	Yes
10161	8:**40 X** vs. 35:**10 X**	-1.919	-2.063 to -1.775	Yes
10162	8:**40 X** vs. 35:**40 X**	-0.5663	-0.7103 to -0.4224	Yes
10163	8:**40 X** vs. 35:**50 X**	-0.5080	-0.6520 to -0.3640	Yes
10164	8:**40 X** vs. 36:**0 X**	-2.872	-3.033 to -2.711	Yes
10165	8:**40 X** vs. 36:**1 X**	-2.568	-2.712 to -2.424	Yes
10166	8:**40 X** vs. 36:**10 X**	-1.925	-2.069 to -1.781	Yes
10167	8:**40 X** vs. 36:**40 X**	-0.6217	-0.7656 to -0.4777	Yes
10168	8:**40 X** vs. 36:**50 X**	-0.4713	-0.6153 to -0.3274	Yes
10169	8:**40 X** vs. 37:**0 X**	-3.256	-3.400 to -3.112	Yes
10170	8:**40 X** vs. 37:**1 X**	-2.638	-2.782 to -2.494	Yes

	2way ANOVA<br>Multiple comparisons	A<br>Data Set-A<br>Y	B<br>Data Set-B<br>Y	C<br>Data Set-C<br>Y
10171	8:**40 X** vs. 37:**10 X**	-1.927	-2.071 to -1.783	Yes
10172	8:**40 X** vs. 37:**40 X**	-0.6590	-0.8030 to -0.5150	Yes
10173	8:**40 X** vs. 37:**50 X**	-0.4800	-0.6240 to -0.3360	Yes
10174	8:**40 X** vs. 38:**0 X**	-3.201	-3.345 to -3.057	Yes
10175	8:**40 X** vs. 38:**1 X**	-2.757	-2.901 to -2.613	Yes
10176	8:**40 X** vs. 38:**10 X**	-2.202	-2.346 to -2.058	Yes
10177	8:**40 X** vs. 38:**40 X**	-0.6633	-0.8073 to -0.5194	Yes
10178	8:**40 X** vs. 38:**50 X**	-0.4840	-0.6280 to -0.3400	Yes
10179	8:**40 X** vs. 39:**0 X**	-3.432	-3.576 to -3.288	Yes
10180	8:**40 X** vs. 39:**1 X**	-3.323	-3.467 to -3.179	Yes
10181	8:**40 X** vs. 39:**10 X**	-2.347	-2.491 to -2.203	Yes
10182	8:**40 X** vs. 39:**40 X**	-0.6693	-0.8133 to -0.5254	Yes
10183	8:**40 X** vs. 39:**50 X**	-0.5150	-0.6590 to -0.3710	Yes
10184	8:**40 X** vs. 40:**0 X**	-3.473	-3.617 to -3.329	Yes
10185	8:**40 X** vs. 40:**1 X**	-3.399	-3.543 to -3.255	Yes
10186	8:**40 X** vs. 40:**10 X**	-2.603	-2.747 to -2.459	Yes
10187	8:**40 X** vs. 40:**40 X**	-0.6740	-0.8180 to -0.5300	Yes
10188	8:**40 X** vs. 40:**50 X**	-0.5213	-0.6653 to -0.3774	Yes
10189	8:**40 X** vs. 41:**0 X**	-3.514	-3.658 to -3.370	Yes
10190	8:**40 X** vs. 41:**1 X**	-3.513	-3.657 to -3.369	Yes
10191	8:**40 X** vs. 41:**10 X**	-2.657	-2.801 to -2.513	Yes
10192	8:**40 X** vs. 41:**40 X**	-0.6213	-0.7653 to -0.4774	Yes
10193	8:**40 X** vs. 41:**50 X**	-0.5340	-0.6780 to -0.3900	Yes
10194	8:**40 X** vs. 42:**0 X**	-3.655	-3.799 to -3.511	Yes
10195	8:**40 X** vs. 42:**1 X**	-3.675	-3.819 to -3.531	Yes
10196	8:**40 X** vs. 42:**10 X**	-2.657	-2.801 to -2.513	Yes
10197	8:**40 X** vs. 42:**40 X**	-0.6340	-0.7780 to -0.4900	Yes
10198	8:**40 X** vs. 42:**50 X**	-0.5373	-0.6813 to -0.3934	Yes
10199	8:**40 X** vs. 43:**0 X**	-3.745	-3.889 to -3.601	Yes
10200	8:**40 X** vs. 43:**1 X**	-3.660	-3.804 to -3.516	Yes
10201	8:**40 X** vs. 43:**10 X**	-3.375	-3.519 to -3.231	Yes
10202	8:**40 X** vs. 43:**40 X**	-0.6323	-0.7763 to -0.4884	Yes
10203	8:**40 X** vs. 43:**50 X**	-0.5377	-0.6816 to -0.3937	Yes
10204	8:**40 X** vs. 44:**0 X**	-3.769	-3.913 to -3.625	Yes
10205	8:**40 X** vs. 44:**1 X**	-3.659	-3.803 to -3.515	Yes
10206	8:**40 X** vs. 44:**10 X**	-3.439	-3.583 to -3.295	Yes
10207	8:**40 X** vs. 44:**40 X**	-0.6343	-0.7783 to -0.4904	Yes
10208	8:**40 X** vs. 44:**50 X**	-0.4700	-0.6140 to -0.3260	Yes
10209	8:**40 X** vs. 45:**0 X**	-3.634	-3.778 to -3.490	Yes
10210	8:**40 X** vs. 45:**1 X**	-3.436	-3.580 to -3.292	Yes
10211	8:**40 X** vs. 45:**10 X**	-3.318	-3.462 to -3.174	Yes
10212	8:**40 X** vs. 45:**40 X**	-0.6357	-0.7796 to -0.4917	Yes
10213	8:**40 X** vs. 45:**50 X**	-0.4670	-0.6110 to -0.3230	Yes
10214	8:**40 X** vs. 46:**0 X**	-3.438	-3.582 to -3.294	Yes
10215	8:**40 X** vs. 46:**1 X**	-3.405	-3.549 to -3.261	Yes

2way ANOVA<br>Multiple comparisons	A<br>Data Set-A<br>Y	B<br>Data Set-B<br>Y	C<br>Data Set-C<br>Y
10216 8:**40 X** vs. 46:**10 X**	-3.313	-3.457 to -3.169	Yes
10217 8:**40 X** vs. 46:**40 X**	-0.6493	-0.7933 to -0.5054	Yes
10218 8:**40 X** vs. 46:**50 X**	-0.4600	-0.6040 to -0.3160	Yes
10219 8:**40 X** vs. 47:**0 X**	-3.414	-3.558 to -3.270	Yes
10220 8:**40 X** vs. 47:**1 X**	-3.318	-3.462 to -3.174	Yes
10221 8:**40 X** vs. 47:**10 X**	-3.299	-3.443 to -3.155	Yes
10222 8:**40 X** vs. 47:**40 X**	-0.6593	-0.8033 to -0.5154	Yes
10223 8:**40 X** vs. 47:**50 X**	-0.4180	-0.5620 to -0.2740	Yes
10224 8:**40 X** vs. 48:**0 X**	-3.401	-3.562 to -3.240	Yes
10225 8:**40 X** vs. 48:**1 X**	-3.305	-3.449 to -3.161	Yes
10226 8:**40 X** vs. 48:**10 X**	-3.299	-3.443 to -3.155	Yes
10227 8:**40 X** vs. 48:**40 X**	-0.6657	-0.8096 to -0.5217	Yes
10228 8:**40 X** vs. 48:**50 X**	-0.4253	-0.5693 to -0.2814	Yes
10229 8:**40 X** vs. 49:**0 X**	-3.401	-3.545 to -3.257	Yes
10230 8:**40 X** vs. 49:**1 X**	-3.305	-3.449 to -3.161	Yes
10231 8:**40 X** vs. 49:**10 X**	-3.299	-3.443 to -3.155	Yes
10232 8:**40 X** vs. 49:**40 X**	-0.6700	-0.8140 to -0.5260	Yes
10233 8:**40 X** vs. 49:**50 X**	-0.4247	-0.5686 to -0.2807	Yes
10234 8:**40 X** vs. 50:**0 X**	-3.401	-3.545 to -3.257	Yes
10235 8:**40 X** vs. 50:**1 X**	-3.305	-3.449 to -3.161	Yes
10236 8:**40 X** vs. 50:**10 X**	-3.299	-3.443 to -3.155	Yes
10237 8:**40 X** vs. 50:**40 X**	-0.6777	-0.8216 to -0.5337	Yes
10238 8:**40 X** vs. 50:**50 X**	-0.4243	-0.5683 to -0.2804	Yes
10239 8:**50 X** vs. 9:**0 X**	-0.01633	-0.1603 to 0.1276	No
10240 8:**50 X** vs. 9:**1 X**	-0.02433	-0.1683 to 0.1196	No
10241 8:**50 X** vs. 9:**10 X**	-0.02533	-0.1693 to 0.1186	No
10242 8:**50 X** vs. 9:**40 X**	-0.007667	-0.1516 to 0.1363	No
10243 8:**50 X** vs. 9:**50 X**	-0.001000	-0.1450 to 0.1430	No
10244 8:**50 X** vs. 10:**0 X**	-0.0180	-0.1620 to 0.1260	No
10245 8:**50 X** vs. 10:**1 X**	-0.0250	-0.1690 to 0.1190	No
10246 8:**50 X** vs. 10:**10 X**	-0.02767	-0.1716 to 0.1163	No
10247 8:**50 X** vs. 10:**40 X**	-0.01733	-0.1613 to 0.1266	No
10248 8:**50 X** vs. 10:**50 X**	-0.004000	-0.1480 to 0.1400	No
10249 8:**50 X** vs. 11:**0 X**	-0.02767	-0.1716 to 0.1163	No
10250 8:**50 X** vs. 11:**1 X**	-0.02933	-0.1733 to 0.1146	No
10251 8:**50 X** vs. 11:**10 X**	-0.02967	-0.1736 to 0.1143	No
10252 8:**50 X** vs. 11:**40 X**	-0.02133	-0.1653 to 0.1226	No
10253 8:**50 X** vs. 11:**50 X**	-0.009333	-0.1533 to 0.1346	No
10254 8:**50 X** vs. 12:**0 X**	-0.02833	-0.1723 to 0.1156	No
10255 8:**50 X** vs. 12:**1 X**	-0.01067	-0.1546 to 0.1333	No
10256 8:**50 X** vs. 12:**10 X**	-0.02833	-0.1723 to 0.1156	No
10257 8:**50 X** vs. 12:**40 X**	-0.01467	-0.1586 to 0.1293	No
10258 8:**50 X** vs. 12:**50 X**	-0.0180	-0.1620 to 0.1260	No
10259 8:**50 X** vs. 13:**0 X**	-0.02233	-0.1663 to 0.1216	No
10260 8:**50 X** vs. 13:**1 X**	-0.009667	-0.1536 to 0.1343	No

2way ANOVA<br>Multiple comparisons	A<br>Data Set-A<br>Y	B<br>Data Set-B<br>Y	C<br>Data Set-C<br>Y
10261 8:50 X vs. 13:10 X	-0.03167	-0.1756 to 0.1123	No
10262 8:50 X vs. 13:40 X	-0.0280	-0.1720 to 0.1160	No
10263 8:50 X vs. 13:50 X	-0.0290	-0.1730 to 0.1150	No
10264 8:50 X vs. 14:0 X	-0.0310	-0.1750 to 0.1130	No
10265 8:50 X vs. 14:1 X	-0.02267	-0.1666 to 0.1213	No
10266 8:50 X vs. 14:10 X	-0.03667	-0.1806 to 0.1073	No
10267 8:50 X vs. 14:40 X	-0.03333	-0.1773 to 0.1106	No
10268 8:50 X vs. 14:50 X	-0.03733	-0.1813 to 0.1066	No
10269 8:50 X vs. 15:0 X	-0.04333	-0.1873 to 0.1006	No
10270 8:50 X vs. 15:1 X	-0.02333	-0.1673 to 0.1206	No
10271 8:50 X vs. 15:10 X	-0.05067	-0.1946 to 0.09329	No
10272 8:50 X vs. 15:40 X	-0.0330	-0.1770 to 0.1110	No
10273 8:50 X vs. 15:50 X	-0.05067	-0.1946 to 0.09329	No
10274 8:50 X vs. 16:0 X	-0.1357	-0.2796 to 0.008293	No
10275 8:50 X vs. 16:1 X	-0.02667	-0.1706 to 0.1173	No
10276 8:50 X vs. 16:10 X	-0.05233	-0.1963 to 0.09163	No
10277 8:50 X vs. 16:40 X	-0.07167	-0.2156 to 0.07229	No
10278 8:50 X vs. 16:50 X	-0.1140	-0.2580 to 0.02996	No
10279 8:50 X vs. 17:0 X	-0.1883	-0.3323 to -0.04437	Yes
10280 8:50 X vs. 17:1 X	-0.1027	-0.2466 to 0.04129	No
10281 8:50 X vs. 17:10 X	-0.1593	-0.3033 to -0.01537	Yes
10282 8:50 X vs. 17:40 X	-0.08267	-0.2266 to 0.06129	No
10283 8:50 X vs. 17:50 X	-0.1280	-0.2720 to 0.01596	No
10284 8:50 X vs. 18:0 X	-0.2370	-0.3810 to -0.09304	Yes
10285 8:50 X vs. 18:1 X	-0.1453	-0.2893 to -0.001374	Yes
10286 8:50 X vs. 18:10 X	-0.2377	-0.3816 to -0.09371	Yes
10287 8:50 X vs. 18:40 X	-0.0870	-0.2310 to 0.05696	No
10288 8:50 X vs. 18:50 X	-0.1320	-0.2760 to 0.01196	No
10289 8:50 X vs. 19:0 X	-0.2720	-0.4160 to -0.1280	Yes
10290 8:50 X vs. 19:1 X	-0.2600	-0.4040 to -0.1160	Yes
10291 8:50 X vs. 19:10 X	-0.2577	-0.4016 to -0.1137	Yes
10292 8:50 X vs. 19:40 X	-0.1157	-0.2596 to 0.02829	No
10293 8:50 X vs. 19:50 X	-0.1267	-0.2706 to 0.01729	No
10294 8:50 X vs. 20:0 X	-0.3517	-0.4956 to -0.2077	Yes
10295 8:50 X vs. 20:1 X	-0.1710	-0.3150 to -0.02704	Yes
10296 8:50 X vs. 20:10 X	-0.2600	-0.4040 to -0.1160	Yes
10297 8:50 X vs. 20:40 X	-0.1273	-0.2713 to 0.01663	No
10298 8:50 X vs. 20:50 X	-0.1713	-0.3153 to -0.02737	Yes
10299 8:50 X vs. 21:0 X	-0.3750	-0.5190 to -0.2310	Yes
10300 8:50 X vs. 21:1 X	-0.3280	-0.4720 to -0.1840	Yes
10301 8:50 X vs. 21:10 X	-0.3250	-0.4690 to -0.1810	Yes
10302 8:50 X vs. 21:40 X	-0.2003	-0.3443 to -0.05637	Yes
10303 8:50 X vs. 21:50 X	-0.2907	-0.4346 to -0.1467	Yes
10304 8:50 X vs. 22:0 X	-0.4167	-0.5606 to -0.2727	Yes
10305 8:50 X vs. 22:1 X	-0.4113	-0.5553 to -0.2674	Yes

	2way ANOVA Multiple comparisons	A Data Set-A Y	B Data Set-B Y	C Data Set-C Y
10306	8:**50 X** vs. 22:**10 X**	-0.4067	-0.5506 to -0.2627	Yes
10307	8:**50 X** vs. 22:**40 X**	-0.3070	-0.4510 to -0.1630	Yes
10308	8:**50 X** vs. 22:**50 X**	-0.3697	-0.5136 to -0.2257	Yes
10309	8:**50 X** vs. 23:**0 X**	-0.4820	-0.6260 to -0.3380	Yes
10310	8:**50 X** vs. 23:**1 X**	-0.6717	-0.8326 to -0.5107	Yes
10311	8:**50 X** vs. 23:**10 X**	-0.4213	-0.5653 to -0.2774	Yes
10312	8:**50 X** vs. 23:**40 X**	-0.3017	-0.4626 to -0.1407	Yes
10313	8:**50 X** vs. 23:**50 X**	-0.3357	-0.4796 to -0.1917	Yes
10314	8:**50 X** vs. 24:**0 X**	-0.5993	-0.7433 to -0.4554	Yes
10315	8:**50 X** vs. 24:**1 X**	-0.7557	-0.9166 to -0.5947	Yes
10316	8:**50 X** vs. 24:**10 X**	-0.5832	-0.7441 to -0.4222	Yes
10317	8:**50 X** vs. 24:**40 X**	-0.3180	-0.4620 to -0.1740	Yes
10318	8:**50 X** vs. 24:**50 X**	-0.2397	-0.3836 to -0.09571	Yes
10319	8:**50 X** vs. 25:**0 X**	-0.9367	-1.098 to -0.7757	Yes
10320	8:**50 X** vs. 25:**1 X**	-1.126	-1.287 to -0.9652	Yes
10321	8:**50 X** vs. 25:**10 X**	-0.4877	-0.6913 to -0.2841	Yes
10322	8:**50 X** vs. 25:**40 X**	-0.3200	-0.4640 to -0.1760	Yes
10323	8:**50 X** vs. 25:**50 X**	-0.2943	-0.4383 to -0.1504	Yes
10324	8:**50 X** vs. 26:**0 X**	-1.149	-1.310 to -0.9882	Yes
10325	8:**50 X** vs. 26:**1 X**	-1.262	-1.423 to -1.101	Yes
10326	8:**50 X** vs. 26:**10 X**	-0.9487	-1.110 to -0.7877	Yes
10327	8:**50 X** vs. 26:**40 X**	-0.3943	-0.5383 to -0.2504	Yes
10328	8:**50 X** vs. 26:**50 X**	-0.2547	-0.3986 to -0.1107	Yes
10329	8:**50 X** vs. 27:**0 X**	-1.337	-1.481 to -1.193	Yes
10330	8:**50 X** vs. 27:**1 X**	-1.428	-1.589 to -1.267	Yes
10331	8:**50 X** vs. 27:**10 X**	-1.240	-1.401 to -1.079	Yes
10332	8:**50 X** vs. 27:**40 X**	-0.3943	-0.5383 to -0.2504	Yes
10333	8:**50 X** vs. 27:**50 X**	-0.2540	-0.3980 to -0.1100	Yes
10334	8:**50 X** vs. 28:**0 X**	-1.286	-1.447 to -1.125	Yes
10335	8:**50 X** vs. 28:**1 X**	-1.327	-1.471 to -1.183	Yes
10336	8:**50 X** vs. 28:**10 X**	-1.129	-1.290 to -0.9677	Yes
10337	8:**50 X** vs. 28:**40 X**	-0.4067	-0.5506 to -0.2627	Yes
10338	8:**50 X** vs. 28:**50 X**	-0.3020	-0.4460 to -0.1580	Yes
10339	8:**50 X** vs. 29:**0 X**	-1.768	-1.929 to -1.607	Yes
10340	8:**50 X** vs. 29:**1 X**	-1.853	-2.014 to -1.692	Yes
10341	8:**50 X** vs. 29:**10 X**	-1.067	-1.228 to -0.9062	Yes
10342	8:**50 X** vs. 29:**40 X**	-0.5147	-0.6586 to -0.3707	Yes
10343	8:**50 X** vs. 29:**50 X**	-0.3033	-0.4473 to -0.1594	Yes
10344	8:**50 X** vs. 30:**0 X**	-1.482	-1.643 to -1.321	Yes
10345	8:**50 X** vs. 30:**1 X**	-1.804	-1.965 to -1.643	Yes
10346	8:**50 X** vs. 30:**10 X**	-1.142	-1.303 to -0.9807	Yes
10347	8:**50 X** vs. 30:**40 X**	-0.4417	-0.5856 to -0.2977	Yes
10348	8:**50 X** vs. 30:**50 X**	-0.3557	-0.4996 to -0.2117	Yes
10349	8:**50 X** vs. 31:**0 X**	-1.800	-1.961 to -1.639	Yes
10350	8:**50 X** vs. 31:**1 X**	-1.791	-1.952 to -1.630	Yes

2way ANOVA<br>Multiple comparisons	A<br>Data Set-A<br>Y	B<br>Data Set-B<br>Y	C<br>Data Set-C<br>Y
10351 8:50 X vs. 31:10 X	-1.273	-1.434 to -1.112	Yes
10352 8:50 X vs. 31:40 X	-0.5333	-0.6773 to -0.3894	Yes
10353 8:50 X vs. 31:50 X	-0.3347	-0.4786 to -0.1907	Yes
10354 8:50 X vs. 32:0 X	-2.392	-2.536 to -2.248	Yes
10355 8:50 X vs. 32:1 X	-1.848	-2.009 to -1.687	Yes
10356 8:50 X vs. 32:10 X	-1.704	-1.848 to -1.560	Yes
10357 8:50 X vs. 32:40 X	-0.5297	-0.6736 to -0.3857	Yes
10358 8:50 X vs. 32:50 X	-0.3343	-0.4783 to -0.1904	Yes
10359 8:50 X vs. 33:0 X	-2.395	-2.539 to -2.251	Yes
10360 8:50 X vs. 33:1 X	-2.210	-2.354 to -2.066	Yes
10361 8:50 X vs. 33:10 X	-1.870	-2.014 to -1.726	Yes
10362 8:50 X vs. 33:40 X	-0.5483	-0.6923 to -0.4044	Yes
10363 8:50 X vs. 33:50 X	-0.3493	-0.4933 to -0.2054	Yes
10364 8:50 X vs. 34:0 X	-2.320	-2.464 to -2.176	Yes
10365 8:50 X vs. 34:1 X	-2.327	-2.471 to -2.183	Yes
10366 8:50 X vs. 34:10 X	-1.961	-2.105 to -1.817	Yes
10367 8:50 X vs. 34:40 X	-0.5603	-0.7043 to -0.4164	Yes
10368 8:50 X vs. 34:50 X	-0.4983	-0.6423 to -0.3544	Yes
10369 8:50 X vs. 35:0 X	-2.669	-2.813 to -2.525	Yes
10370 8:50 X vs. 35:1 X	-2.434	-2.578 to -2.290	Yes
10371 8:50 X vs. 35:10 X	-1.922	-2.066 to -1.778	Yes
10372 8:50 X vs. 35:40 X	-0.5693	-0.7133 to -0.4254	Yes
10373 8:50 X vs. 35:50 X	-0.5110	-0.6550 to -0.3670	Yes
10374 8:50 X vs. 36:0 X	-2.875	-3.036 to -2.714	Yes
10375 8:50 X vs. 36:1 X	-2.571	-2.715 to -2.427	Yes
10376 8:50 X vs. 36:10 X	-1.928	-2.072 to -1.784	Yes
10377 8:50 X vs. 36:40 X	-0.6247	-0.7686 to -0.4807	Yes
10378 8:50 X vs. 36:50 X	-0.4743	-0.6183 to -0.3304	Yes
10379 8:50 X vs. 37:0 X	-3.259	-3.403 to -3.115	Yes
10380 8:50 X vs. 37:1 X	-2.641	-2.785 to -2.497	Yes
10381 8:50 X vs. 37:10 X	-1.930	-2.074 to -1.786	Yes
10382 8:50 X vs. 37:40 X	-0.6620	-0.8060 to -0.5180	Yes
10383 8:50 X vs. 37:50 X	-0.4830	-0.6270 to -0.3390	Yes
10384 8:50 X vs. 38:0 X	-3.204	-3.348 to -3.060	Yes
10385 8:50 X vs. 38:1 X	-2.760	-2.904 to -2.616	Yes
10386 8:50 X vs. 38:10 X	-2.205	-2.349 to -2.061	Yes
10387 8:50 X vs. 38:40 X	-0.6663	-0.8103 to -0.5224	Yes
10388 8:50 X vs. 38:50 X	-0.4870	-0.6310 to -0.3430	Yes
10389 8:50 X vs. 39:0 X	-3.435	-3.579 to -3.291	Yes
10390 8:50 X vs. 39:1 X	-3.326	-3.470 to -3.182	Yes
10391 8:50 X vs. 39:10 X	-2.350	-2.494 to -2.206	Yes
10392 8:50 X vs. 39:40 X	-0.6723	-0.8163 to -0.5284	Yes
10393 8:50 X vs. 39:50 X	-0.5180	-0.6620 to -0.3740	Yes
10394 8:50 X vs. 40:0 X	-3.476	-3.620 to -3.332	Yes
10395 8:50 X vs. 40:1 X	-3.402	-3.546 to -3.258	Yes

	2way ANOVA<br>Multiple comparisons	A<br>Data Set-A<br>Y	B<br>Data Set-B<br>Y	C<br>Data Set-C<br>Y
10396	8:50 X vs. 40:10 X	-2.606	-2.750 to -2.462	Yes
10397	8:50 X vs. 40:40 X	-0.6770	-0.8210 to -0.5330	Yes
10398	8:50 X vs. 40:50 X	-0.5243	-0.6683 to -0.3804	Yes
10399	8:50 X vs. 41:0 X	-3.517	-3.661 to -3.373	Yes
10400	8:50 X vs. 41:1 X	-3.516	-3.660 to -3.372	Yes
10401	8:50 X vs. 41:10 X	-2.660	-2.804 to -2.516	Yes
10402	8:50 X vs. 41:40 X	-0.6243	-0.7683 to -0.4804	Yes
10403	8:50 X vs. 41:50 X	-0.5370	-0.6810 to -0.3930	Yes
10404	8:50 X vs. 42:0 X	-3.658	-3.802 to -3.514	Yes
10405	8:50 X vs. 42:1 X	-3.678	-3.822 to -3.534	Yes
10406	8:50 X vs. 42:10 X	-2.660	-2.804 to -2.516	Yes
10407	8:50 X vs. 42:40 X	-0.6370	-0.7810 to -0.4930	Yes
10408	8:50 X vs. 42:50 X	-0.5403	-0.6843 to -0.3964	Yes
10409	8:50 X vs. 43:0 X	-3.748	-3.892 to -3.604	Yes
10410	8:50 X vs. 43:1 X	-3.663	-3.807 to -3.519	Yes
10411	8:50 X vs. 43:10 X	-3.378	-3.522 to -3.234	Yes
10412	8:50 X vs. 43:40 X	-0.6353	-0.7793 to -0.4914	Yes
10413	8:50 X vs. 43:50 X	-0.5407	-0.6846 to -0.3967	Yes
10414	8:50 X vs. 44:0 X	-3.772	-3.916 to -3.628	Yes
10415	8:50 X vs. 44:1 X	-3.662	-3.806 to -3.518	Yes
10416	8:50 X vs. 44:10 X	-3.442	-3.586 to -3.298	Yes
10417	8:50 X vs. 44:40 X	-0.6373	-0.7813 to -0.4934	Yes
10418	8:50 X vs. 44:50 X	-0.4730	-0.6170 to -0.3290	Yes
10419	8:50 X vs. 45:0 X	-3.637	-3.781 to -3.493	Yes
10420	8:50 X vs. 45:1 X	-3.439	-3.583 to -3.295	Yes
10421	8:50 X vs. 45:10 X	-3.321	-3.465 to -3.177	Yes
10422	8:50 X vs. 45:40 X	-0.6387	-0.7826 to -0.4947	Yes
10423	8:50 X vs. 45:50 X	-0.4700	-0.6140 to -0.3260	Yes
10424	8:50 X vs. 46:0 X	-3.441	-3.585 to -3.297	Yes
10425	8:50 X vs. 46:1 X	-3.408	-3.552 to -3.264	Yes
10426	8:50 X vs. 46:10 X	-3.316	-3.460 to -3.172	Yes
10427	8:50 X vs. 46:40 X	-0.6523	-0.7963 to -0.5084	Yes
10428	8:50 X vs. 46:50 X	-0.4630	-0.6070 to -0.3190	Yes
10429	8:50 X vs. 47:0 X	-3.417	-3.561 to -3.273	Yes
10430	8:50 X vs. 47:1 X	-3.321	-3.465 to -3.177	Yes
10431	8:50 X vs. 47:10 X	-3.302	-3.446 to -3.158	Yes
10432	8:50 X vs. 47:40 X	-0.6623	-0.8063 to -0.5184	Yes
10433	8:50 X vs. 47:50 X	-0.4210	-0.5650 to -0.2770	Yes
10434	8:50 X vs. 48:0 X	-3.404	-3.565 to -3.243	Yes
10435	8:50 X vs. 48:1 X	-3.308	-3.452 to -3.164	Yes
10436	8:50 X vs. 48:10 X	-3.302	-3.446 to -3.158	Yes
10437	8:50 X vs. 48:40 X	-0.6687	-0.8126 to -0.5247	Yes
10438	8:50 X vs. 48:50 X	-0.4283	-0.5723 to -0.2844	Yes
10439	8:50 X vs. 49:0 X	-3.404	-3.548 to -3.260	Yes
10440	8:50 X vs. 49:1 X	-3.308	-3.452 to -3.164	Yes

	2way ANOVA<br>Multiple comparisons	A<br>Data Set-A<br>Y	B<br>Data Set-B<br>Y	C<br>Data Set-C<br>Y
10441	8:**50 X** vs. 49:**10 X**	-3.302	-3.446 to -3.158	Yes
10442	8:**50 X** vs. 49:**40 X**	-0.6730	-0.8170 to -0.5290	Yes
10443	8:**50 X** vs. 49:**50 X**	-0.4277	-0.5716 to -0.2837	Yes
10444	8:**50 X** vs. 50:**0 X**	-3.404	-3.548 to -3.260	Yes
10445	8:**50 X** vs. 50:**1 X**	-3.308	-3.452 to -3.164	Yes
10446	8:**50 X** vs. 50:**10 X**	-3.302	-3.446 to -3.158	Yes
10447	8:**50 X** vs. 50:**40 X**	-0.6807	-0.8246 to -0.5367	Yes
10448	8:**50 X** vs. 50:**50 X**	-0.4273	-0.5713 to -0.2834	Yes
10449	9:**0 X** vs. 9:**1 X**	-0.008000	-0.1520 to 0.1360	No
10450	9:**0 X** vs. 9:**10 X**	-0.009000	-0.1530 to 0.1350	No
10451	9:**0 X** vs. 9:**40 X**	0.008667	-0.1353 to 0.1526	No
10452	9:**0 X** vs. 9:**50 X**	0.01533	-0.1286 to 0.1593	No
10453	9:**0 X** vs. 10:**0 X**	-0.001667	-0.1456 to 0.1423	No
10454	9:**0 X** vs. 10:**1 X**	-0.008667	-0.1526 to 0.1353	No
10455	9:**0 X** vs. 10:**10 X**	-0.01133	-0.1553 to 0.1326	No
10456	9:**0 X** vs. 10:**40 X**	-0.001000	-0.1450 to 0.1430	No
10457	9:**0 X** vs. 10:**50 X**	0.01233	-0.1316 to 0.1563	No
10458	9:**0 X** vs. 11:**0 X**	-0.01133	-0.1553 to 0.1326	No
10459	9:**0 X** vs. 11:**1 X**	-0.0130	-0.1570 to 0.1310	No
10460	9:**0 X** vs. 11:**10 X**	-0.01333	-0.1573 to 0.1306	No
10461	9:**0 X** vs. 11:**40 X**	-0.005000	-0.1490 to 0.1390	No
10462	9:**0 X** vs. 11:**50 X**	0.007000	-0.1370 to 0.1510	No
10463	9:**0 X** vs. 12:**0 X**	-0.0120	-0.1560 to 0.1320	No
10464	9:**0 X** vs. 12:**1 X**	0.005667	-0.1383 to 0.1496	No
10465	9:**0 X** vs. 12:**10 X**	-0.0120	-0.1560 to 0.1320	No
10466	9:**0 X** vs. 12:**40 X**	0.001667	-0.1423 to 0.1456	No
10467	9:**0 X** vs. 12:**50 X**	-0.001667	-0.1456 to 0.1423	No
10468	9:**0 X** vs. 13:**0 X**	-0.006000	-0.1500 to 0.1380	No
10469	9:**0 X** vs. 13:**1 X**	0.006667	-0.1373 to 0.1506	No
10470	9:**0 X** vs. 13:**10 X**	-0.01533	-0.1593 to 0.1286	No
10471	9:**0 X** vs. 13:**40 X**	-0.01167	-0.1556 to 0.1323	No
10472	9:**0 X** vs. 13:**50 X**	-0.01267	-0.1566 to 0.1313	No
10473	9:**0 X** vs. 14:**0 X**	-0.01467	-0.1586 to 0.1293	No
10474	9:**0 X** vs. 14:**1 X**	-0.006333	-0.1503 to 0.1376	No
10475	9:**0 X** vs. 14:**10 X**	-0.02033	-0.1643 to 0.1236	No
10476	9:**0 X** vs. 14:**40 X**	-0.0170	-0.1610 to 0.1270	No
10477	9:**0 X** vs. 14:**50 X**	-0.0210	-0.1650 to 0.1230	No
10478	9:**0 X** vs. 15:**0 X**	-0.0270	-0.1710 to 0.1170	No
10479	9:**0 X** vs. 15:**1 X**	-0.007000	-0.1510 to 0.1370	No
10480	9:**0 X** vs. 15:**10 X**	-0.03433	-0.1783 to 0.1096	No
10481	9:**0 X** vs. 15:**40 X**	-0.01667	-0.1606 to 0.1273	No
10482	9:**0 X** vs. 15:**50 X**	-0.03433	-0.1783 to 0.1096	No
10483	9:**0 X** vs. 16:**0 X**	-0.1193	-0.2633 to 0.02463	No
10484	9:**0 X** vs. 16:**1 X**	-0.01033	-0.1543 to 0.1336	No
10485	9:**0 X** vs. 16:**10 X**	-0.0360	-0.1800 to 0.1080	No

	2way ANOVA<br>Multiple comparisons	A<br>Data Set-A<br>Y	B<br>Data Set-B<br>Y	C<br>Data Set-C<br>Y
10486	9:0 X vs. 16:40 X	-0.05533	-0.1993 to 0.08863	No
10487	9:0 X vs. 16:50 X	-0.09767	-0.2416 to 0.04629	No
10488	9:0 X vs. 17:0 X	-0.1720	-0.3160 to -0.02804	Yes
10489	9:0 X vs. 17:1 X	-0.08633	-0.2303 to 0.05763	No
10490	9:0 X vs. 17:10 X	-0.1430	-0.2870 to 0.0009598	No
10491	9:0 X vs. 17:40 X	-0.06633	-0.2103 to 0.07763	No
10492	9:0 X vs. 17:50 X	-0.1117	-0.2556 to 0.03229	No
10493	9:0 X vs. 18:0 X	-0.2207	-0.3646 to -0.07671	Yes
10494	9:0 X vs. 18:1 X	-0.1290	-0.2730 to 0.01496	No
10495	9:0 X vs. 18:10 X	-0.2213	-0.3653 to -0.07737	Yes
10496	9:0 X vs. 18:40 X	-0.07067	-0.2146 to 0.07329	No
10497	9:0 X vs. 18:50 X	-0.1157	-0.2596 to 0.02829	No
10498	9:0 X vs. 19:0 X	-0.2557	-0.3996 to -0.1117	Yes
10499	9:0 X vs. 19:1 X	-0.2437	-0.3876 to -0.09971	Yes
10500	9:0 X vs. 19:10 X	-0.2413	-0.3853 to -0.09737	Yes
10501	9:0 X vs. 19:40 X	-0.09933	-0.2433 to 0.04463	No
10502	9:0 X vs. 19:50 X	-0.1103	-0.2543 to 0.03363	No
10503	9:0 X vs. 20:0 X	-0.3353	-0.4793 to -0.1914	Yes
10504	9:0 X vs. 20:1 X	-0.1547	-0.2986 to -0.01071	Yes
10505	9:0 X vs. 20:10 X	-0.2437	-0.3876 to -0.09971	Yes
10506	9:0 X vs. 20:40 X	-0.1110	-0.2550 to 0.03296	No
10507	9:0 X vs. 20:50 X	-0.1550	-0.2990 to -0.01104	Yes
10508	9:0 X vs. 21:0 X	-0.3587	-0.5026 to -0.2147	Yes
10509	9:0 X vs. 21:1 X	-0.3117	-0.4556 to -0.1677	Yes
10510	9:0 X vs. 21:10 X	-0.3087	-0.4526 to -0.1647	Yes
10511	9:0 X vs. 21:40 X	-0.1840	-0.3280 to -0.04004	Yes
10512	9:0 X vs. 21:50 X	-0.2743	-0.4183 to -0.1304	Yes
10513	9:0 X vs. 22:0 X	-0.4003	-0.5443 to -0.2564	Yes
10514	9:0 X vs. 22:1 X	-0.3950	-0.5390 to -0.2510	Yes
10515	9:0 X vs. 22:10 X	-0.3903	-0.5343 to -0.2464	Yes
10516	9:0 X vs. 22:40 X	-0.2907	-0.4346 to -0.1467	Yes
10517	9:0 X vs. 22:50 X	-0.3533	-0.4973 to -0.2094	Yes
10518	9:0 X vs. 23:0 X	-0.4657	-0.6096 to -0.3217	Yes
10519	9:0 X vs. 23:1 X	-0.6553	-0.8163 to -0.4944	Yes
10520	9:0 X vs. 23:10 X	-0.4050	-0.5490 to -0.2610	Yes
10521	9:0 X vs. 23:40 X	-0.2853	-0.4463 to -0.1244	Yes
10522	9:0 X vs. 23:50 X	-0.3193	-0.4633 to -0.1754	Yes
10523	9:0 X vs. 24:0 X	-0.5830	-0.7270 to -0.4390	Yes
10524	9:0 X vs. 24:1 X	-0.7393	-0.9003 to -0.5784	Yes
10525	9:0 X vs. 24:10 X	-0.5668	-0.7278 to -0.4059	Yes
10526	9:0 X vs. 24:40 X	-0.3017	-0.4456 to -0.1577	Yes
10527	9:0 X vs. 24:50 X	-0.2233	-0.3673 to -0.07937	Yes
10528	9:0 X vs. 25:0 X	-0.9203	-1.081 to -0.7594	Yes
10529	9:0 X vs. 25:1 X	-1.110	-1.271 to -0.9489	Yes
10530	9:0 X vs. 25:10 X	-0.4713	-0.6749 to -0.2677	Yes

	2way ANOVA<br>Multiple comparisons	A<br>Data Set-A<br>Y	B<br>Data Set-B<br>Y	C<br>Data Set-C<br>Y
10531	9:0 X vs. 25:40 X	-0.3037	-0.4476 to -0.1597	Yes
10532	9:0 X vs. 25:50 X	-0.2780	-0.4220 to -0.1340	Yes
10533	9:0 X vs. 26:0 X	-1.133	-1.294 to -0.9719	Yes
10534	9:0 X vs. 26:1 X	-1.246	-1.407 to -1.085	Yes
10535	9:0 X vs. 26:10 X	-0.9323	-1.093 to -0.7714	Yes
10536	9:0 X vs. 26:40 X	-0.3780	-0.5220 to -0.2340	Yes
10537	9:0 X vs. 26:50 X	-0.2383	-0.3823 to -0.09437	Yes
10538	9:0 X vs. 27:0 X	-1.321	-1.465 to -1.177	Yes
10539	9:0 X vs. 27:1 X	-1.411	-1.572 to -1.250	Yes
10540	9:0 X vs. 27:10 X	-1.223	-1.384 to -1.062	Yes
10541	9:0 X vs. 27:40 X	-0.3780	-0.5220 to -0.2340	Yes
10542	9:0 X vs. 27:50 X	-0.2377	-0.3816 to -0.09371	Yes
10543	9:0 X vs. 28:0 X	-1.270	-1.431 to -1.109	Yes
10544	9:0 X vs. 28:1 X	-1.311	-1.455 to -1.167	Yes
10545	9:0 X vs. 28:10 X	-1.112	-1.273 to -0.9514	Yes
10546	9:0 X vs. 28:40 X	-0.3903	-0.5343 to -0.2464	Yes
10547	9:0 X vs. 28:50 X	-0.2857	-0.4296 to -0.1417	Yes
10548	9:0 X vs. 29:0 X	-1.751	-1.912 to -1.590	Yes
10549	9:0 X vs. 29:1 X	-1.836	-1.997 to -1.675	Yes
10550	9:0 X vs. 29:10 X	-1.051	-1.212 to -0.8899	Yes
10551	9:0 X vs. 29:40 X	-0.4983	-0.6423 to -0.3544	Yes
10552	9:0 X vs. 29:50 X	-0.2870	-0.4310 to -0.1430	Yes
10553	9:0 X vs. 30:0 X	-1.466	-1.627 to -1.305	Yes
10554	9:0 X vs. 30:1 X	-1.787	-1.948 to -1.626	Yes
10555	9:0 X vs. 30:10 X	-1.125	-1.286 to -0.9644	Yes
10556	9:0 X vs. 30:40 X	-0.4253	-0.5693 to -0.2814	Yes
10557	9:0 X vs. 30:50 X	-0.3393	-0.4833 to -0.1954	Yes
10558	9:0 X vs. 31:0 X	-1.784	-1.945 to -1.623	Yes
10559	9:0 X vs. 31:1 X	-1.774	-1.935 to -1.613	Yes
10560	9:0 X vs. 31:10 X	-1.257	-1.418 to -1.096	Yes
10561	9:0 X vs. 31:40 X	-0.5170	-0.6610 to -0.3730	Yes
10562	9:0 X vs. 31:50 X	-0.3183	-0.4623 to -0.1744	Yes
10563	9:0 X vs. 32:0 X	-2.375	-2.519 to -2.231	Yes
10564	9:0 X vs. 32:1 X	-1.831	-1.992 to -1.670	Yes
10565	9:0 X vs. 32:10 X	-1.687	-1.831 to -1.543	Yes
10566	9:0 X vs. 32:40 X	-0.5133	-0.6573 to -0.3694	Yes
10567	9:0 X vs. 32:50 X	-0.3180	-0.4620 to -0.1740	Yes
10568	9:0 X vs. 33:0 X	-2.379	-2.523 to -2.235	Yes
10569	9:0 X vs. 33:1 X	-2.194	-2.338 to -2.050	Yes
10570	9:0 X vs. 33:10 X	-1.854	-1.998 to -1.710	Yes
10571	9:0 X vs. 33:40 X	-0.5320	-0.6760 to -0.3880	Yes
10572	9:0 X vs. 33:50 X	-0.3330	-0.4770 to -0.1890	Yes
10573	9:0 X vs. 34:0 X	-2.304	-2.448 to -2.160	Yes
10574	9:0 X vs. 34:1 X	-2.311	-2.455 to -2.167	Yes
10575	9:0 X vs. 34:10 X	-1.944	-2.088 to -1.800	Yes

	2way ANOVA<br>Multiple comparisons	A<br>Data Set-A<br>Y	B<br>Data Set-B<br>Y	C<br>Data Set-C<br>Y
10576	9:0 X vs. 34:40 X	-0.5440	-0.6880 to -0.4000	Yes
10577	9:0 X vs. 34:50 X	-0.4820	-0.6260 to -0.3380	Yes
10578	9:0 X vs. 35:0 X	-2.652	-2.796 to -2.508	Yes
10579	9:0 X vs. 35:1 X	-2.418	-2.562 to -2.274	Yes
10580	9:0 X vs. 35:10 X	-1.906	-2.050 to -1.762	Yes
10581	9:0 X vs. 35:40 X	-0.5530	-0.6970 to -0.4090	Yes
10582	9:0 X vs. 35:50 X	-0.4947	-0.6386 to -0.3507	Yes
10583	9:0 X vs. 36:0 X	-2.859	-3.020 to -2.698	Yes
10584	9:0 X vs. 36:1 X	-2.555	-2.699 to -2.411	Yes
10585	9:0 X vs. 36:10 X	-1.912	-2.056 to -1.768	Yes
10586	9:0 X vs. 36:40 X	-0.6083	-0.7523 to -0.4644	Yes
10587	9:0 X vs. 36:50 X	-0.4580	-0.6020 to -0.3140	Yes
10588	9:0 X vs. 37:0 X	-3.243	-3.387 to -3.099	Yes
10589	9:0 X vs. 37:1 X	-2.625	-2.769 to -2.481	Yes
10590	9:0 X vs. 37:10 X	-1.913	-2.057 to -1.769	Yes
10591	9:0 X vs. 37:40 X	-0.6457	-0.7896 to -0.5017	Yes
10592	9:0 X vs. 37:50 X	-0.4667	-0.6106 to -0.3227	Yes
10593	9:0 X vs. 38:0 X	-3.188	-3.332 to -3.044	Yes
10594	9:0 X vs. 38:1 X	-2.743	-2.887 to -2.599	Yes
10595	9:0 X vs. 38:10 X	-2.189	-2.333 to -2.045	Yes
10596	9:0 X vs. 38:40 X	-0.6500	-0.7940 to -0.5060	Yes
10597	9:0 X vs. 38:50 X	-0.4707	-0.6146 to -0.3267	Yes
10598	9:0 X vs. 39:0 X	-3.418	-3.562 to -3.274	Yes
10599	9:0 X vs. 39:1 X	-3.310	-3.454 to -3.166	Yes
10600	9:0 X vs. 39:10 X	-2.334	-2.478 to -2.190	Yes
10601	9:0 X vs. 39:40 X	-0.6560	-0.8000 to -0.5120	Yes
10602	9:0 X vs. 39:50 X	-0.5017	-0.6456 to -0.3577	Yes
10603	9:0 X vs. 40:0 X	-3.459	-3.603 to -3.315	Yes
10604	9:0 X vs. 40:1 X	-3.385	-3.529 to -3.241	Yes
10605	9:0 X vs. 40:10 X	-2.590	-2.734 to -2.446	Yes
10606	9:0 X vs. 40:40 X	-0.6607	-0.8046 to -0.5167	Yes
10607	9:0 X vs. 40:50 X	-0.5080	-0.6520 to -0.3640	Yes
10608	9:0 X vs. 41:0 X	-3.500	-3.644 to -3.356	Yes
10609	9:0 X vs. 41:1 X	-3.499	-3.643 to -3.355	Yes
10610	9:0 X vs. 41:10 X	-2.644	-2.788 to -2.500	Yes
10611	9:0 X vs. 41:40 X	-0.6080	-0.7520 to -0.4640	Yes
10612	9:0 X vs. 41:50 X	-0.5207	-0.6646 to -0.3767	Yes
10613	9:0 X vs. 42:0 X	-3.642	-3.786 to -3.498	Yes
10614	9:0 X vs. 42:1 X	-3.662	-3.806 to -3.518	Yes
10615	9:0 X vs. 42:10 X	-2.644	-2.788 to -2.500	Yes
10616	9:0 X vs. 42:40 X	-0.6207	-0.7646 to -0.4767	Yes
10617	9:0 X vs. 42:50 X	-0.5240	-0.6680 to -0.3800	Yes
10618	9:0 X vs. 43:0 X	-3.732	-3.876 to -3.588	Yes
10619	9:0 X vs. 43:1 X	-3.646	-3.790 to -3.502	Yes
10620	9:0 X vs. 43:10 X	-3.362	-3.506 to -3.218	Yes

	2way ANOVA<br>Multiple comparisons	A<br>Data Set-A<br>Y	B<br>Data Set-B<br>Y	C<br>Data Set-C<br>Y
10621	9:0 X vs. 43:40 X	-0.6190	-0.7630 to -0.4750	Yes
10622	9:0 X vs. 43:50 X	-0.5243	-0.6683 to -0.3804	Yes
10623	9:0 X vs. 44:0 X	-3.756	-3.900 to -3.612	Yes
10624	9:0 X vs. 44:1 X	-3.646	-3.790 to -3.502	Yes
10625	9:0 X vs. 44:10 X	-3.425	-3.569 to -3.281	Yes
10626	9:0 X vs. 44:40 X	-0.6210	-0.7650 to -0.4770	Yes
10627	9:0 X vs. 44:50 X	-0.4567	-0.6006 to -0.3127	Yes
10628	9:0 X vs. 45:0 X	-3.621	-3.765 to -3.477	Yes
10629	9:0 X vs. 45:1 X	-3.423	-3.567 to -3.279	Yes
10630	9:0 X vs. 45:10 X	-3.305	-3.449 to -3.161	Yes
10631	9:0 X vs. 45:40 X	-0.6223	-0.7663 to -0.4784	Yes
10632	9:0 X vs. 45:50 X	-0.4537	-0.5976 to -0.3097	Yes
10633	9:0 X vs. 46:0 X	-3.425	-3.569 to -3.281	Yes
10634	9:0 X vs. 46:1 X	-3.392	-3.536 to -3.248	Yes
10635	9:0 X vs. 46:10 X	-3.300	-3.444 to -3.156	Yes
10636	9:0 X vs. 46:40 X	-0.6360	-0.7800 to -0.4920	Yes
10637	9:0 X vs. 46:50 X	-0.4467	-0.5906 to -0.3027	Yes
10638	9:0 X vs. 47:0 X	-3.401	-3.545 to -3.257	Yes
10639	9:0 X vs. 47:1 X	-3.305	-3.449 to -3.161	Yes
10640	9:0 X vs. 47:10 X	-3.286	-3.430 to -3.142	Yes
10641	9:0 X vs. 47:40 X	-0.6460	-0.7900 to -0.5020	Yes
10642	9:0 X vs. 47:50 X	-0.4047	-0.5486 to -0.2607	Yes
10643	9:0 X vs. 48:0 X	-3.388	-3.549 to -3.227	Yes
10644	9:0 X vs. 48:1 X	-3.292	-3.436 to -3.148	Yes
10645	9:0 X vs. 48:10 X	-3.286	-3.430 to -3.142	Yes
10646	9:0 X vs. 48:40 X	-0.6523	-0.7963 to -0.5084	Yes
10647	9:0 X vs. 48:50 X	-0.4120	-0.5560 to -0.2680	Yes
10648	9:0 X vs. 49:0 X	-3.388	-3.532 to -3.244	Yes
10649	9:0 X vs. 49:1 X	-3.292	-3.436 to -3.148	Yes
10650	9:0 X vs. 49:10 X	-3.286	-3.430 to -3.142	Yes
10651	9:0 X vs. 49:40 X	-0.6567	-0.8006 to -0.5127	Yes
10652	9:0 X vs. 49:50 X	-0.4113	-0.5553 to -0.2674	Yes
10653	9:0 X vs. 50:0 X	-3.388	-3.532 to -3.244	Yes
10654	9:0 X vs. 50:1 X	-3.292	-3.436 to -3.148	Yes
10655	9:0 X vs. 50:10 X	-3.286	-3.430 to -3.142	Yes
10656	9:0 X vs. 50:40 X	-0.6643	-0.8083 to -0.5204	Yes
10657	9:0 X vs. 50:50 X	-0.4110	-0.5550 to -0.2670	Yes
10658	9:1 X vs. 9:10 X	-0.001000	-0.1450 to 0.1430	No
10659	9:1 X vs. 9:40 X	0.01667	-0.1273 to 0.1606	No
10660	9:1 X vs. 9:50 X	0.02333	-0.1206 to 0.1673	No
10661	9:1 X vs. 10:0 X	0.006333	-0.1376 to 0.1503	No
10662	9:1 X vs. 10:1 X	-0.0006667	-0.1446 to 0.1433	No
10663	9:1 X vs. 10:10 X	-0.003333	-0.1473 to 0.1406	No
10664	9:1 X vs. 10:40 X	0.007000	-0.1370 to 0.1510	No
10665	9:1 X vs. 10:50 X	0.02033	-0.1236 to 0.1643	No

2way ANOVA Multiple comparisons		A Data Set-A Y	B Data Set-B Y	C Data Set-C Y
10666	9:1 X vs. 11:0 X	-0.003333	-0.1473 to 0.1406	No
10667	9:1 X vs. 11:1 X	-0.005000	-0.1490 to 0.1390	No
10668	9:1 X vs. 11:10 X	-0.005333	-0.1493 to 0.1386	No
10669	9:1 X vs. 11:40 X	0.003000	-0.1410 to 0.1470	No
10670	9:1 X vs. 11:50 X	0.0150	-0.1290 to 0.1590	No
10671	9:1 X vs. 12:0 X	-0.004000	-0.1480 to 0.1400	No
10672	9:1 X vs. 12:1 X	0.01367	-0.1303 to 0.1576	No
10673	9:1 X vs. 12:10 X	-0.004000	-0.1480 to 0.1400	No
10674	9:1 X vs. 12:40 X	0.009667	-0.1343 to 0.1536	No
10675	9:1 X vs. 12:50 X	0.006333	-0.1376 to 0.1503	No
10676	9:1 X vs. 13:0 X	0.0020	-0.1420 to 0.1460	No
10677	9:1 X vs. 13:1 X	0.01467	-0.1293 to 0.1586	No
10678	9:1 X vs. 13:10 X	-0.007333	-0.1513 to 0.1366	No
10679	9:1 X vs. 13:40 X	-0.003667	-0.1476 to 0.1403	No
10680	9:1 X vs. 13:50 X	-0.004667	-0.1486 to 0.1393	No
10681	9:1 X vs. 14:0 X	-0.006667	-0.1506 to 0.1373	No
10682	9:1 X vs. 14:1 X	0.001667	-0.1423 to 0.1456	No
10683	9:1 X vs. 14:10 X	-0.01233	-0.1563 to 0.1316	No
10684	9:1 X vs. 14:40 X	-0.009000	-0.1530 to 0.1350	No
10685	9:1 X vs. 14:50 X	-0.0130	-0.1570 to 0.1310	No
10686	9:1 X vs. 15:0 X	-0.0190	-0.1630 to 0.1250	No
10687	9:1 X vs. 15:1 X	0.001000	-0.1430 to 0.1450	No
10688	9:1 X vs. 15:10 X	-0.02633	-0.1703 to 0.1176	No
10689	9:1 X vs. 15:40 X	-0.008667	-0.1526 to 0.1353	No
10690	9:1 X vs. 15:50 X	-0.02633	-0.1703 to 0.1176	No
10691	9:1 X vs. 16:0 X	-0.1113	-0.2553 to 0.03263	No
10692	9:1 X vs. 16:1 X	-0.002333	-0.1463 to 0.1416	No
10693	9:1 X vs. 16:10 X	-0.0280	-0.1720 to 0.1160	No
10694	9:1 X vs. 16:40 X	-0.04733	-0.1913 to 0.09663	No
10695	9:1 X vs. 16:50 X	-0.08967	-0.2336 to 0.05429	No
10696	9:1 X vs. 17:0 X	-0.1640	-0.3080 to -0.02004	Yes
10697	9:1 X vs. 17:1 X	-0.07833	-0.2223 to 0.06563	No
10698	9:1 X vs. 17:10 X	-0.1350	-0.2790 to 0.008960	No
10699	9:1 X vs. 17:40 X	-0.05833	-0.2023 to 0.08563	No
10700	9:1 X vs. 17:50 X	-0.1037	-0.2476 to 0.04029	No
10701	9:1 X vs. 18:0 X	-0.2127	-0.3566 to -0.06871	Yes
10702	9:1 X vs. 18:1 X	-0.1210	-0.2650 to 0.02296	No
10703	9:1 X vs. 18:10 X	-0.2133	-0.3573 to -0.06937	Yes
10704	9:1 X vs. 18:40 X	-0.06267	-0.2066 to 0.08129	No
10705	9:1 X vs. 18:50 X	-0.1077	-0.2516 to 0.03629	No
10706	9:1 X vs. 19:0 X	-0.2477	-0.3916 to -0.1037	Yes
10707	9:1 X vs. 19:1 X	-0.2357	-0.3796 to -0.09171	Yes
10708	9:1 X vs. 19:10 X	-0.2333	-0.3773 to -0.08937	Yes
10709	9:1 X vs. 19:40 X	-0.09133	-0.2353 to 0.05263	No
10710	9:1 X vs. 19:50 X	-0.1023	-0.2463 to 0.04163	No

2way ANOVA<br>Multiple comparisons	A<br>Data Set-A<br>Y	B<br>Data Set-B<br>Y	C<br>Data Set-C<br>Y
10711 9:**1 X** vs. 20:**0 X**	-0.3273	-0.4713 to -0.1834	Yes
10712 9:**1 X** vs. 20:**1 X**	-0.1467	-0.2906 to -0.002707	Yes
10713 9:**1 X** vs. 20:**10 X**	-0.2357	-0.3796 to -0.09171	Yes
10714 9:**1 X** vs. 20:**40 X**	-0.1030	-0.2470 to 0.04096	No
10715 9:**1 X** vs. 20:**50 X**	-0.1470	-0.2910 to -0.003040	Yes
10716 9:**1 X** vs. 21:**0 X**	-0.3507	-0.4946 to -0.2067	Yes
10717 9:**1 X** vs. 21:**1 X**	-0.3037	-0.4476 to -0.1597	Yes
10718 9:**1 X** vs. 21:**10 X**	-0.3007	-0.4446 to -0.1567	Yes
10719 9:**1 X** vs. 21:**40 X**	-0.1760	-0.3200 to -0.03204	Yes
10720 9:**1 X** vs. 21:**50 X**	-0.2663	-0.4103 to -0.1224	Yes
10721 9:**1 X** vs. 22:**0 X**	-0.3923	-0.5363 to -0.2484	Yes
10722 9:**1 X** vs. 22:**1 X**	-0.3870	-0.5310 to -0.2430	Yes
10723 9:**1 X** vs. 22:**10 X**	-0.3823	-0.5263 to -0.2384	Yes
10724 9:**1 X** vs. 22:**40 X**	-0.2827	-0.4266 to -0.1387	Yes
10725 9:**1 X** vs. 22:**50 X**	-0.3453	-0.4893 to -0.2014	Yes
10726 9:**1 X** vs. 23:**0 X**	-0.4577	-0.6016 to -0.3137	Yes
10727 9:**1 X** vs. 23:**1 X**	-0.6473	-0.8083 to -0.4864	Yes
10728 9:**1 X** vs. 23:**10 X**	-0.3970	-0.5410 to -0.2530	Yes
10729 9:**1 X** vs. 23:**40 X**	-0.2773	-0.4383 to -0.1164	Yes
10730 9:**1 X** vs. 23:**50 X**	-0.3113	-0.4553 to -0.1674	Yes
10731 9:**1 X** vs. 24:**0 X**	-0.5750	-0.7190 to -0.4310	Yes
10732 9:**1 X** vs. 24:**1 X**	-0.7313	-0.8923 to -0.5704	Yes
10733 9:**1 X** vs. 24:**10 X**	-0.5588	-0.7198 to -0.3979	Yes
10734 9:**1 X** vs. 24:**40 X**	-0.2937	-0.4376 to -0.1497	Yes
10735 9:**1 X** vs. 24:**50 X**	-0.2153	-0.3593 to -0.07137	Yes
10736 9:**1 X** vs. 25:**0 X**	-0.9123	-1.073 to -0.7514	Yes
10737 9:**1 X** vs. 25:**1 X**	-1.102	-1.263 to -0.9409	Yes
10738 9:**1 X** vs. 25:**10 X**	-0.4633	-0.6669 to -0.2597	Yes
10739 9:**1 X** vs. 25:**40 X**	-0.2957	-0.4396 to -0.1517	Yes
10740 9:**1 X** vs. 25:**50 X**	-0.2700	-0.4140 to -0.1260	Yes
10741 9:**1 X** vs. 26:**0 X**	-1.125	-1.286 to -0.9639	Yes
10742 9:**1 X** vs. 26:**1 X**	-1.238	-1.399 to -1.077	Yes
10743 9:**1 X** vs. 26:**10 X**	-0.9243	-1.085 to -0.7634	Yes
10744 9:**1 X** vs. 26:**40 X**	-0.3700	-0.5140 to -0.2260	Yes
10745 9:**1 X** vs. 26:**50 X**	-0.2303	-0.3743 to -0.08637	Yes
10746 9:**1 X** vs. 27:**0 X**	-1.313	-1.457 to -1.169	Yes
10747 9:**1 X** vs. 27:**1 X**	-1.403	-1.564 to -1.242	Yes
10748 9:**1 X** vs. 27:**10 X**	-1.215	-1.376 to -1.054	Yes
10749 9:**1 X** vs. 27:**40 X**	-0.3700	-0.5140 to -0.2260	Yes
10750 9:**1 X** vs. 27:**50 X**	-0.2297	-0.3736 to -0.08571	Yes
10751 9:**1 X** vs. 28:**0 X**	-1.262	-1.423 to -1.101	Yes
10752 9:**1 X** vs. 28:**1 X**	-1.303	-1.447 to -1.159	Yes
10753 9:**1 X** vs. 28:**10 X**	-1.104	-1.265 to -0.9434	Yes
10754 9:**1 X** vs. 28:**40 X**	-0.3823	-0.5263 to -0.2384	Yes
10755 9:**1 X** vs. 28:**50 X**	-0.2777	-0.4216 to -0.1337	Yes

2way ANOVA<br>Multiple comparisons		A<br>Data Set-A<br>Y	B<br>Data Set-B<br>Y	C<br>Data Set-C<br>Y
10756	9:1 X vs. 29:0 X	-1.743	-1.904 to -1.582	Yes
10757	9:1 X vs. 29:1 X	-1.828	-1.989 to -1.667	Yes
10758	9:1 X vs. 29:10 X	-1.043	-1.204 to -0.8819	Yes
10759	9:1 X vs. 29:40 X	-0.4903	-0.6343 to -0.3464	Yes
10760	9:1 X vs. 29:50 X	-0.2790	-0.4230 to -0.1350	Yes
10761	9:1 X vs. 30:0 X	-1.458	-1.619 to -1.297	Yes
10762	9:1 X vs. 30:1 X	-1.779	-1.940 to -1.618	Yes
10763	9:1 X vs. 30:10 X	-1.117	-1.278 to -0.9564	Yes
10764	9:1 X vs. 30:40 X	-0.4173	-0.5613 to -0.2734	Yes
10765	9:1 X vs. 30:50 X	-0.3313	-0.4753 to -0.1874	Yes
10766	9:1 X vs. 31:0 X	-1.776	-1.937 to -1.615	Yes
10767	9:1 X vs. 31:1 X	-1.766	-1.927 to -1.605	Yes
10768	9:1 X vs. 31:10 X	-1.249	-1.410 to -1.088	Yes
10769	9:1 X vs. 31:40 X	-0.5090	-0.6530 to -0.3650	Yes
10770	9:1 X vs. 31:50 X	-0.3103	-0.4543 to -0.1664	Yes
10771	9:1 X vs. 32:0 X	-2.367	-2.511 to -2.223	Yes
10772	9:1 X vs. 32:1 X	-1.823	-1.984 to -1.662	Yes
10773	9:1 X vs. 32:10 X	-1.679	-1.823 to -1.535	Yes
10774	9:1 X vs. 32:40 X	-0.5053	-0.6493 to -0.3614	Yes
10775	9:1 X vs. 32:50 X	-0.3100	-0.4540 to -0.1660	Yes
10776	9:1 X vs. 33:0 X	-2.371	-2.515 to -2.227	Yes
10777	9:1 X vs. 33:1 X	-2.186	-2.330 to -2.042	Yes
10778	9:1 X vs. 33:10 X	-1.846	-1.990 to -1.702	Yes
10779	9:1 X vs. 33:40 X	-0.5240	-0.6680 to -0.3800	Yes
10780	9:1 X vs. 33:50 X	-0.3250	-0.4690 to -0.1810	Yes
10781	9:1 X vs. 34:0 X	-2.296	-2.440 to -2.152	Yes
10782	9:1 X vs. 34:1 X	-2.303	-2.447 to -2.159	Yes
10783	9:1 X vs. 34:10 X	-1.936	-2.080 to -1.792	Yes
10784	9:1 X vs. 34:40 X	-0.5360	-0.6800 to -0.3920	Yes
10785	9:1 X vs. 34:50 X	-0.4740	-0.6180 to -0.3300	Yes
10786	9:1 X vs. 35:0 X	-2.644	-2.788 to -2.500	Yes
10787	9:1 X vs. 35:1 X	-2.410	-2.554 to -2.266	Yes
10788	9:1 X vs. 35:10 X	-1.898	-2.042 to -1.754	Yes
10789	9:1 X vs. 35:40 X	-0.5450	-0.6890 to -0.4010	Yes
10790	9:1 X vs. 35:50 X	-0.4867	-0.6306 to -0.3427	Yes
10791	9:1 X vs. 36:0 X	-2.851	-3.012 to -2.690	Yes
10792	9:1 X vs. 36:1 X	-2.547	-2.691 to -2.403	Yes
10793	9:1 X vs. 36:10 X	-1.904	-2.048 to -1.760	Yes
10794	9:1 X vs. 36:40 X	-0.6003	-0.7443 to -0.4564	Yes
10795	9:1 X vs. 36:50 X	-0.4500	-0.5940 to -0.3060	Yes
10796	9:1 X vs. 37:0 X	-3.235	-3.379 to -3.091	Yes
10797	9:1 X vs. 37:1 X	-2.617	-2.761 to -2.473	Yes
10798	9:1 X vs. 37:10 X	-1.905	-2.049 to -1.761	Yes
10799	9:1 X vs. 37:40 X	-0.6377	-0.7816 to -0.4937	Yes
10800	9:1 X vs. 37:50 X	-0.4587	-0.6026 to -0.3147	Yes

	2way ANOVA<br>Multiple comparisons	A<br>Data Set-A<br>Y	B<br>Data Set-B<br>Y	C<br>Data Set-C<br>Y
10801	9:1 X vs. 38:0 X	-3.180	-3.324 to -3.036	Yes
10802	9:1 X vs. 38:1 X	-2.735	-2.879 to -2.591	Yes
10803	9:1 X vs. 38:10 X	-2.181	-2.325 to -2.037	Yes
10804	9:1 X vs. 38:40 X	-0.6420	-0.7860 to -0.4980	Yes
10805	9:1 X vs. 38:50 X	-0.4627	-0.6066 to -0.3187	Yes
10806	9:1 X vs. 39:0 X	-3.410	-3.554 to -3.266	Yes
10807	9:1 X vs. 39:1 X	-3.302	-3.446 to -3.158	Yes
10808	9:1 X vs. 39:10 X	-2.326	-2.470 to -2.182	Yes
10809	9:1 X vs. 39:40 X	-0.6480	-0.7920 to -0.5040	Yes
10810	9:1 X vs. 39:50 X	-0.4937	-0.6376 to -0.3497	Yes
10811	9:1 X vs. 40:0 X	-3.451	-3.595 to -3.307	Yes
10812	9:1 X vs. 40:1 X	-3.377	-3.521 to -3.233	Yes
10813	9:1 X vs. 40:10 X	-2.582	-2.726 to -2.438	Yes
10814	9:1 X vs. 40:40 X	-0.6527	-0.7966 to -0.5087	Yes
10815	9:1 X vs. 40:50 X	-0.5000	-0.6440 to -0.3560	Yes
10816	9:1 X vs. 41:0 X	-3.492	-3.636 to -3.348	Yes
10817	9:1 X vs. 41:1 X	-3.491	-3.635 to -3.347	Yes
10818	9:1 X vs. 41:10 X	-2.636	-2.780 to -2.492	Yes
10819	9:1 X vs. 41:40 X	-0.6000	-0.7440 to -0.4560	Yes
10820	9:1 X vs. 41:50 X	-0.5127	-0.6566 to -0.3687	Yes
10821	9:1 X vs. 42:0 X	-3.634	-3.778 to -3.490	Yes
10822	9:1 X vs. 42:1 X	-3.654	-3.798 to -3.510	Yes
10823	9:1 X vs. 42:10 X	-2.636	-2.780 to -2.492	Yes
10824	9:1 X vs. 42:40 X	-0.6127	-0.7566 to -0.4687	Yes
10825	9:1 X vs. 42:50 X	-0.5160	-0.6600 to -0.3720	Yes
10826	9:1 X vs. 43:0 X	-3.724	-3.868 to -3.580	Yes
10827	9:1 X vs. 43:1 X	-3.638	-3.782 to -3.494	Yes
10828	9:1 X vs. 43:10 X	-3.354	-3.498 to -3.210	Yes
10829	9:1 X vs. 43:40 X	-0.6110	-0.7550 to -0.4670	Yes
10830	9:1 X vs. 43:50 X	-0.5163	-0.6603 to -0.3724	Yes
10831	9:1 X vs. 44:0 X	-3.748	-3.892 to -3.604	Yes
10832	9:1 X vs. 44:1 X	-3.638	-3.782 to -3.494	Yes
10833	9:1 X vs. 44:10 X	-3.417	-3.561 to -3.273	Yes
10834	9:1 X vs. 44:40 X	-0.6130	-0.7570 to -0.4690	Yes
10835	9:1 X vs. 44:50 X	-0.4487	-0.5926 to -0.3047	Yes
10836	9:1 X vs. 45:0 X	-3.613	-3.757 to -3.469	Yes
10837	9:1 X vs. 45:1 X	-3.415	-3.559 to -3.271	Yes
10838	9:1 X vs. 45:10 X	-3.297	-3.441 to -3.153	Yes
10839	9:1 X vs. 45:40 X	-0.6143	-0.7583 to -0.4704	Yes
10840	9:1 X vs. 45:50 X	-0.4457	-0.5896 to -0.3017	Yes
10841	9:1 X vs. 46:0 X	-3.417	-3.561 to -3.273	Yes
10842	9:1 X vs. 46:1 X	-3.384	-3.528 to -3.240	Yes
10843	9:1 X vs. 46:10 X	-3.292	-3.436 to -3.148	Yes
10844	9:1 X vs. 46:40 X	-0.6280	-0.7720 to -0.4840	Yes
10845	9:1 X vs. 46:50 X	-0.4387	-0.5826 to -0.2947	Yes

2way ANOVA Multiple comparisons	A Data Set-A Y	B Data Set-B Y	C Data Set-C Y
10846 9:1 X vs. 47:0 X	-3.393	-3.537 to -3.249	Yes
10847 9:1 X vs. 47:1 X	-3.297	-3.441 to -3.153	Yes
10848 9:1 X vs. 47:10 X	-3.278	-3.422 to -3.134	Yes
10849 9:1 X vs. 47:40 X	-0.6380	-0.7820 to -0.4940	Yes
10850 9:1 X vs. 47:50 X	-0.3967	-0.5406 to -0.2527	Yes
10851 9:1 X vs. 48:0 X	-3.380	-3.541 to -3.219	Yes
10852 9:1 X vs. 48:1 X	-3.284	-3.428 to -3.140	Yes
10853 9:1 X vs. 48:10 X	-3.278	-3.422 to -3.134	Yes
10854 9:1 X vs. 48:40 X	-0.6443	-0.7883 to -0.5004	Yes
10855 9:1 X vs. 48:50 X	-0.4040	-0.5480 to -0.2600	Yes
10856 9:1 X vs. 49:0 X	-3.380	-3.524 to -3.236	Yes
10857 9:1 X vs. 49:1 X	-3.284	-3.428 to -3.140	Yes
10858 9:1 X vs. 49:10 X	-3.278	-3.422 to -3.134	Yes
10859 9:1 X vs. 49:40 X	-0.6487	-0.7926 to -0.5047	Yes
10860 9:1 X vs. 49:50 X	-0.4033	-0.5473 to -0.2594	Yes
10861 9:1 X vs. 50:0 X	-3.380	-3.524 to -3.236	Yes
10862 9:1 X vs. 50:1 X	-3.284	-3.428 to -3.140	Yes
10863 9:1 X vs. 50:10 X	-3.278	-3.422 to -3.134	Yes
10864 9:1 X vs. 50:40 X	-0.6563	-0.8003 to -0.5124	Yes
10865 9:1 X vs. 50:50 X	-0.4030	-0.5470 to -0.2590	Yes
10866 9:10 X vs. 9:40 X	0.01767	-0.1263 to 0.1616	No
10867 9:10 X vs. 9:50 X	0.02433	-0.1196 to 0.1683	No
10868 9:10 X vs. 10:0 X	0.007333	-0.1366 to 0.1513	No
10869 9:10 X vs. 10:1 X	0.0003333	-0.1436 to 0.1443	No
10870 9:10 X vs. 10:10 X	-0.002333	-0.1463 to 0.1416	No
10871 9:10 X vs. 10:40 X	0.008000	-0.1360 to 0.1520	No
10872 9:10 X vs. 10:50 X	0.02133	-0.1226 to 0.1653	No
10873 9:10 X vs. 11:0 X	-0.002333	-0.1463 to 0.1416	No
10874 9:10 X vs. 11:1 X	-0.004000	-0.1480 to 0.1400	No
10875 9:10 X vs. 11:10 X	-0.004333	-0.1483 to 0.1396	No
10876 9:10 X vs. 11:40 X	0.004000	-0.1400 to 0.1480	No
10877 9:10 X vs. 11:50 X	0.0160	-0.1280 to 0.1600	No
10878 9:10 X vs. 12:0 X	-0.003000	-0.1470 to 0.1410	No
10879 9:10 X vs. 12:1 X	0.01467	-0.1293 to 0.1586	No
10880 9:10 X vs. 12:10 X	-0.0030	-0.1470 to 0.1410	No
10881 9:10 X vs. 12:40 X	0.01067	-0.1333 to 0.1546	No
10882 9:10 X vs. 12:50 X	0.007333	-0.1366 to 0.1513	No
10883 9:10 X vs. 13:0 X	0.0030	-0.1410 to 0.1470	No
10884 9:10 X vs. 13:1 X	0.01567	-0.1283 to 0.1596	No
10885 9:10 X vs. 13:10 X	-0.006333	-0.1503 to 0.1376	No
10886 9:10 X vs. 13:40 X	-0.002667	-0.1466 to 0.1413	No
10887 9:10 X vs. 13:50 X	-0.003667	-0.1476 to 0.1403	No
10888 9:10 X vs. 14:0 X	-0.005667	-0.1496 to 0.1383	No
10889 9:10 X vs. 14:1 X	0.002667	-0.1413 to 0.1466	No
10890 9:10 X vs. 14:10 X	-0.01133	-0.1553 to 0.1326	No

	2way ANOVA<br>Multiple comparisons	A<br>Data Set-A<br>Y	B<br>Data Set-B<br>Y	C<br>Data Set-C<br>Y
10891	9:**10 X** vs. 14:**40 X**	-0.008000	-0.1520 to 0.1360	No
10892	9:**10 X** vs. 14:**50 X**	-0.0120	-0.1560 to 0.1320	No
10893	9:**10 X** vs. 15:**0 X**	-0.0180	-0.1620 to 0.1260	No
10894	9:**10 X** vs. 15:**1 X**	0.0020	-0.1420 to 0.1460	No
10895	9:**10 X** vs. 15:**10 X**	-0.02533	-0.1693 to 0.1186	No
10896	9:**10 X** vs. 15:**40 X**	-0.007667	-0.1516 to 0.1363	No
10897	9:**10 X** vs. 15:**50 X**	-0.02533	-0.1693 to 0.1186	No
10898	9:**10 X** vs. 16:**0 X**	-0.1103	-0.2543 to 0.03363	No
10899	9:**10 X** vs. 16:**1 X**	-0.001333	-0.1453 to 0.1426	No
10900	9:**10 X** vs. 16:**10 X**	-0.0270	-0.1710 to 0.1170	No
10901	9:**10 X** vs. 16:**40 X**	-0.04633	-0.1903 to 0.09763	No
10902	9:**10 X** vs. 16:**50 X**	-0.08867	-0.2326 to 0.05529	No
10903	9:**10 X** vs. 17:**0 X**	-0.1630	-0.3070 to -0.01904	Yes
10904	9:**10 X** vs. 17:**1 X**	-0.07733	-0.2213 to 0.06663	No
10905	9:**10 X** vs. 17:**10 X**	-0.1340	-0.2780 to 0.009960	No
10906	9:**10 X** vs. 17:**40 X**	-0.05733	-0.2013 to 0.08663	No
10907	9:**10 X** vs. 17:**50 X**	-0.1027	-0.2466 to 0.04129	No
10908	9:**10 X** vs. 18:**0 X**	-0.2117	-0.3556 to -0.06771	Yes
10909	9:**10 X** vs. 18:**1 X**	-0.1200	-0.2640 to 0.02396	No
10910	9:**10 X** vs. 18:**10 X**	-0.2123	-0.3563 to -0.06837	Yes
10911	9:**10 X** vs. 18:**40 X**	-0.06167	-0.2056 to 0.08229	No
10912	9:**10 X** vs. 18:**50 X**	-0.1067	-0.2506 to 0.03729	No
10913	9:**10 X** vs. 19:**0 X**	-0.2467	-0.3906 to -0.1027	Yes
10914	9:**10 X** vs. 19:**1 X**	-0.2347	-0.3786 to -0.09071	Yes
10915	9:**10 X** vs. 19:**10 X**	-0.2323	-0.3763 to -0.08837	Yes
10916	9:**10 X** vs. 19:**40 X**	-0.09033	-0.2343 to 0.05363	No
10917	9:**10 X** vs. 19:**50 X**	-0.1013	-0.2453 to 0.04263	No
10918	9:**10 X** vs. 20:**0 X**	-0.3263	-0.4703 to -0.1824	Yes
10919	9:**10 X** vs. 20:**1 X**	-0.1457	-0.2896 to -0.001707	Yes
10920	9:**10 X** vs. 20:**10 X**	-0.2347	-0.3786 to -0.09071	Yes
10921	9:**10 X** vs. 20:**40 X**	-0.1020	-0.2460 to 0.04196	No
10922	9:**10 X** vs. 20:**50 X**	-0.1460	-0.2900 to -0.002040	Yes
10923	9:**10 X** vs. 21:**0 X**	-0.3497	-0.4936 to -0.2057	Yes
10924	9:**10 X** vs. 21:**1 X**	-0.3027	-0.4466 to -0.1587	Yes
10925	9:**10 X** vs. 21:**10 X**	-0.2997	-0.4436 to -0.1557	Yes
10926	9:**10 X** vs. 21:**40 X**	-0.1750	-0.3190 to -0.03104	Yes
10927	9:**10 X** vs. 21:**50 X**	-0.2653	-0.4093 to -0.1214	Yes
10928	9:**10 X** vs. 22:**0 X**	-0.3913	-0.5353 to -0.2474	Yes
10929	9:**10 X** vs. 22:**1 X**	-0.3860	-0.5300 to -0.2420	Yes
10930	9:**10 X** vs. 22:**10 X**	-0.3813	-0.5253 to -0.2374	Yes
10931	9:**10 X** vs. 22:**40 X**	-0.2817	-0.4256 to -0.1377	Yes
10932	9:**10 X** vs. 22:**50 X**	-0.3443	-0.4883 to -0.2004	Yes
10933	9:**10 X** vs. 23:**0 X**	-0.4567	-0.6006 to -0.3127	Yes
10934	9:**10 X** vs. 23:**1 X**	-0.6463	-0.8073 to -0.4854	Yes
10935	9:**10 X** vs. 23:**10 X**	-0.3960	-0.5400 to -0.2520	Yes

	2way ANOVA Multiple comparisons	A Data Set-A Y	B Data Set-B Y	C Data Set-C Y
10936	9:**10 X** vs. 23:**40 X**	-0.2763	-0.4373 to -0.1154	Yes
10937	9:**10 X** vs. 23:**50 X**	-0.3103	-0.4543 to -0.1664	Yes
10938	9:**10 X** vs. 24:**0 X**	-0.5740	-0.7180 to -0.4300	Yes
10939	9:**10 X** vs. 24:**1 X**	-0.7303	-0.8913 to -0.5694	Yes
10940	9:**10 X** vs. 24:**10 X**	-0.5578	-0.7188 to -0.3969	Yes
10941	9:**10 X** vs. 24:**40 X**	-0.2927	-0.4366 to -0.1487	Yes
10942	9:**10 X** vs. 24:**50 X**	-0.2143	-0.3583 to -0.07037	Yes
10943	9:**10 X** vs. 25:**0 X**	-0.9113	-1.072 to -0.7504	Yes
10944	9:**10 X** vs. 25:**1 X**	-1.101	-1.262 to -0.9399	Yes
10945	9:**10 X** vs. 25:**10 X**	-0.4623	-0.6659 to -0.2587	Yes
10946	9:**10 X** vs. 25:**40 X**	-0.2947	-0.4386 to -0.1507	Yes
10947	9:**10 X** vs. 25:**50 X**	-0.2690	-0.4130 to -0.1250	Yes
10948	9:**10 X** vs. 26:**0 X**	-1.124	-1.285 to -0.9629	Yes
10949	9:**10 X** vs. 26:**1 X**	-1.237	-1.398 to -1.076	Yes
10950	9:**10 X** vs. 26:**10 X**	-0.9233	-1.084 to -0.7624	Yes
10951	9:**10 X** vs. 26:**40 X**	-0.3690	-0.5130 to -0.2250	Yes
10952	9:**10 X** vs. 26:**50 X**	-0.2293	-0.3733 to -0.08537	Yes
10953	9:**10 X** vs. 27:**0 X**	-1.312	-1.456 to -1.168	Yes
10954	9:**10 X** vs. 27:**1 X**	-1.402	-1.563 to -1.241	Yes
10955	9:**10 X** vs. 27:**10 X**	-1.214	-1.375 to -1.053	Yes
10956	9:**10 X** vs. 27:**40 X**	-0.3690	-0.5130 to -0.2250	Yes
10957	9:**10 X** vs. 27:**50 X**	-0.2287	-0.3726 to -0.08471	Yes
10958	9:**10 X** vs. 28:**0 X**	-1.261	-1.422 to -1.100	Yes
10959	9:**10 X** vs. 28:**1 X**	-1.302	-1.446 to -1.158	Yes
10960	9:**10 X** vs. 28:**10 X**	-1.103	-1.264 to -0.9424	Yes
10961	9:**10 X** vs. 28:**40 X**	-0.3813	-0.5253 to -0.2374	Yes
10962	9:**10 X** vs. 28:**50 X**	-0.2767	-0.4206 to -0.1327	Yes
10963	9:**10 X** vs. 29:**0 X**	-1.742	-1.903 to -1.581	Yes
10964	9:**10 X** vs. 29:**1 X**	-1.827	-1.988 to -1.666	Yes
10965	9:**10 X** vs. 29:**10 X**	-1.042	-1.203 to -0.8809	Yes
10966	9:**10 X** vs. 29:**40 X**	-0.4893	-0.6333 to -0.3454	Yes
10967	9:**10 X** vs. 29:**50 X**	-0.2780	-0.4220 to -0.1340	Yes
10968	9:**10 X** vs. 30:**0 X**	-1.457	-1.618 to -1.296	Yes
10969	9:**10 X** vs. 30:**1 X**	-1.778	-1.939 to -1.617	Yes
10970	9:**10 X** vs. 30:**10 X**	-1.116	-1.277 to -0.9554	Yes
10971	9:**10 X** vs. 30:**40 X**	-0.4163	-0.5603 to -0.2724	Yes
10972	9:**10 X** vs. 30:**50 X**	-0.3303	-0.4743 to -0.1864	Yes
10973	9:**10 X** vs. 31:**0 X**	-1.775	-1.936 to -1.614	Yes
10974	9:**10 X** vs. 31:**1 X**	-1.765	-1.926 to -1.604	Yes
10975	9:**10 X** vs. 31:**10 X**	-1.248	-1.409 to -1.087	Yes
10976	9:**10 X** vs. 31:**40 X**	-0.5080	-0.6520 to -0.3640	Yes
10977	9:**10 X** vs. 31:**50 X**	-0.3093	-0.4533 to -0.1654	Yes
10978	9:**10 X** vs. 32:**0 X**	-2.366	-2.510 to -2.222	Yes
10979	9:**10 X** vs. 32:**1 X**	-1.822	-1.983 to -1.661	Yes
10980	9:**10 X** vs. 32:**10 X**	-1.678	-1.822 to -1.534	Yes

2way ANOVA<br>Multiple comparisons	A<br>Data Set-A<br>Y	B<br>Data Set-B<br>Y	C<br>Data Set-C<br>Y
10981 9:**10 X** vs. 32:**40 X**	-0.5043	-0.6483 to -0.3604	Yes
10982 9:**10 X** vs. 32:**50 X**	-0.3090	-0.4530 to -0.1650	Yes
10983 9:**10 X** vs. 33:**0 X**	-2.370	-2.514 to -2.226	Yes
10984 9:**10 X** vs. 33:**1 X**	-2.185	-2.329 to -2.041	Yes
10985 9:**10 X** vs. 33:**10 X**	-1.845	-1.989 to -1.701	Yes
10986 9:**10 X** vs. 33:**40 X**	-0.5230	-0.6670 to -0.3790	Yes
10987 9:**10 X** vs. 33:**50 X**	-0.3240	-0.4680 to -0.1800	Yes
10988 9:**10 X** vs. 34:**0 X**	-2.295	-2.439 to -2.151	Yes
10989 9:**10 X** vs. 34:**1 X**	-2.302	-2.446 to -2.158	Yes
10990 9:**10 X** vs. 34:**10 X**	-1.935	-2.079 to -1.791	Yes
10991 9:**10 X** vs. 34:**40 X**	-0.5350	-0.6790 to -0.3910	Yes
10992 9:**10 X** vs. 34:**50 X**	-0.4730	-0.6170 to -0.3290	Yes
10993 9:**10 X** vs. 35:**0 X**	-2.643	-2.787 to -2.499	Yes
10994 9:**10 X** vs. 35:**1 X**	-2.409	-2.553 to -2.265	Yes
10995 9:**10 X** vs. 35:**10 X**	-1.897	-2.041 to -1.753	Yes
10996 9:**10 X** vs. 35:**40 X**	-0.5440	-0.6880 to -0.4000	Yes
10997 9:**10 X** vs. 35:**50 X**	-0.4857	-0.6296 to -0.3417	Yes
10998 9:**10 X** vs. 36:**0 X**	-2.850	-3.011 to -2.689	Yes
10999 9:**10 X** vs. 36:**1 X**	-2.546	-2.690 to -2.402	Yes
11000 9:**10 X** vs. 36:**10 X**	-1.903	-2.047 to -1.759	Yes
11001 9:**10 X** vs. 36:**40 X**	-0.5993	-0.7433 to -0.4554	Yes
11002 9:**10 X** vs. 36:**50 X**	-0.4490	-0.5930 to -0.3050	Yes
11003 9:**10 X** vs. 37:**0 X**	-3.234	-3.378 to -3.090	Yes
11004 9:**10 X** vs. 37:**1 X**	-2.616	-2.760 to -2.472	Yes
11005 9:**10 X** vs. 37:**10 X**	-1.904	-2.048 to -1.760	Yes
11006 9:**10 X** vs. 37:**40 X**	-0.6367	-0.7806 to -0.4927	Yes
11007 9:**10 X** vs. 37:**50 X**	-0.4577	-0.6016 to -0.3137	Yes
11008 9:**10 X** vs. 38:**0 X**	-3.179	-3.323 to -3.035	Yes
11009 9:**10 X** vs. 38:**1 X**	-2.734	-2.878 to -2.590	Yes
11010 9:**10 X** vs. 38:**10 X**	-2.180	-2.324 to -2.036	Yes
11011 9:**10 X** vs. 38:**40 X**	-0.6410	-0.7850 to -0.4970	Yes
11012 9:**10 X** vs. 38:**50 X**	-0.4617	-0.6056 to -0.3177	Yes
11013 9:**10 X** vs. 39:**0 X**	-3.409	-3.553 to -3.265	Yes
11014 9:**10 X** vs. 39:**1 X**	-3.301	-3.445 to -3.157	Yes
11015 9:**10 X** vs. 39:**10 X**	-2.325	-2.469 to -2.181	Yes
11016 9:**10 X** vs. 39:**40 X**	-0.6470	-0.7910 to -0.5030	Yes
11017 9:**10 X** vs. 39:**50 X**	-0.4927	-0.6366 to -0.3487	Yes
11018 9:**10 X** vs. 40:**0 X**	-3.450	-3.594 to -3.306	Yes
11019 9:**10 X** vs. 40:**1 X**	-3.376	-3.520 to -3.232	Yes
11020 9:**10 X** vs. 40:**10 X**	-2.581	-2.725 to -2.437	Yes
11021 9:**10 X** vs. 40:**40 X**	-0.6517	-0.7956 to -0.5077	Yes
11022 9:**10 X** vs. 40:**50 X**	-0.4990	-0.6430 to -0.3550	Yes
11023 9:**10 X** vs. 41:**0 X**	-3.491	-3.635 to -3.347	Yes
11024 9:**10 X** vs. 41:**1 X**	-3.490	-3.634 to -3.346	Yes
11025 9:**10 X** vs. 41:**10 X**	-2.635	-2.779 to -2.491	Yes

2way ANOVA<br>Multiple comparisons	A<br>Data Set-A<br>Y	B<br>Data Set-B<br>Y	C<br>Data Set-C<br>Y
11026 9:**10 X** vs. 41:**40 X**	-0.5990	-0.7430 to -0.4550	Yes
11027 9:**10 X** vs. 41:**50 X**	-0.5117	-0.6556 to -0.3677	Yes
11028 9:**10 X** vs. 42:**0 X**	-3.633	-3.777 to -3.489	Yes
11029 9:**10 X** vs. 42:**1 X**	-3.653	-3.797 to -3.509	Yes
11030 9:**10 X** vs. 42:**10 X**	-2.635	-2.779 to -2.491	Yes
11031 9:**10 X** vs. 42:**40 X**	-0.6117	-0.7556 to -0.4677	Yes
11032 9:**10 X** vs. 42:**50 X**	-0.5150	-0.6590 to -0.3710	Yes
11033 9:**10 X** vs. 43:**0 X**	-3.723	-3.867 to -3.579	Yes
11034 9:**10 X** vs. 43:**1 X**	-3.637	-3.781 to -3.493	Yes
11035 9:**10 X** vs. 43:**10 X**	-3.353	-3.497 to -3.209	Yes
11036 9:**10 X** vs. 43:**40 X**	-0.6100	-0.7540 to -0.4660	Yes
11037 9:**10 X** vs. 43:**50 X**	-0.5153	-0.6593 to -0.3714	Yes
11038 9:**10 X** vs. 44:**0 X**	-3.747	-3.891 to -3.603	Yes
11039 9:**10 X** vs. 44:**1 X**	-3.637	-3.781 to -3.493	Yes
11040 9:**10 X** vs. 44:**10 X**	-3.416	-3.560 to -3.272	Yes
11041 9:**10 X** vs. 44:**40 X**	-0.6120	-0.7560 to -0.4680	Yes
11042 9:**10 X** vs. 44:**50 X**	-0.4477	-0.5916 to -0.3037	Yes
11043 9:**10 X** vs. 45:**0 X**	-3.612	-3.756 to -3.468	Yes
11044 9:**10 X** vs. 45:**1 X**	-3.414	-3.558 to -3.270	Yes
11045 9:**10 X** vs. 45:**10 X**	-3.296	-3.440 to -3.152	Yes
11046 9:**10 X** vs. 45:**40 X**	-0.6133	-0.7573 to -0.4694	Yes
11047 9:**10 X** vs. 45:**50 X**	-0.4447	-0.5886 to -0.3007	Yes
11048 9:**10 X** vs. 46:**0 X**	-3.416	-3.560 to -3.272	Yes
11049 9:**10 X** vs. 46:**1 X**	-3.383	-3.527 to -3.239	Yes
11050 9:**10 X** vs. 46:**10 X**	-3.291	-3.435 to -3.147	Yes
11051 9:**10 X** vs. 46:**40 X**	-0.6270	-0.7710 to -0.4830	Yes
11052 9:**10 X** vs. 46:**50 X**	-0.4377	-0.5816 to -0.2937	Yes
11053 9:**10 X** vs. 47:**0 X**	-3.392	-3.536 to -3.248	Yes
11054 9:**10 X** vs. 47:**1 X**	-3.296	-3.440 to -3.152	Yes
11055 9:**10 X** vs. 47:**10 X**	-3.277	-3.421 to -3.133	Yes
11056 9:**10 X** vs. 47:**40 X**	-0.6370	-0.7810 to -0.4930	Yes
11057 9:**10 X** vs. 47:**50 X**	-0.3957	-0.5396 to -0.2517	Yes
11058 9:**10 X** vs. 48:**0 X**	-3.379	-3.540 to -3.218	Yes
11059 9:**10 X** vs. 48:**1 X**	-3.283	-3.427 to -3.139	Yes
11060 9:**10 X** vs. 48:**10 X**	-3.277	-3.421 to -3.133	Yes
11061 9:**10 X** vs. 48:**40 X**	-0.6433	-0.7873 to -0.4994	Yes
11062 9:**10 X** vs. 48:**50 X**	-0.4030	-0.5470 to -0.2590	Yes
11063 9:**10 X** vs. 49:**0 X**	-3.379	-3.523 to -3.235	Yes
11064 9:**10 X** vs. 49:**1 X**	-3.283	-3.427 to -3.139	Yes
11065 9:**10 X** vs. 49:**10 X**	-3.277	-3.421 to -3.133	Yes
11066 9:**10 X** vs. 49:**40 X**	-0.6477	-0.7916 to -0.5037	Yes
11067 9:**10 X** vs. 49:**50 X**	-0.4023	-0.5463 to -0.2584	Yes
11068 9:**10 X** vs. 50:**0 X**	-3.379	-3.523 to -3.235	Yes
11069 9:**10 X** vs. 50:**1 X**	-3.283	-3.427 to -3.139	Yes
11070 9:**10 X** vs. 50:**10 X**	-3.277	-3.421 to -3.133	Yes

	2way ANOVA<br>Multiple comparisons	A<br>Data Set-A<br>Y	B<br>Data Set-B<br>Y	C<br>Data Set-C<br>Y
11071	9:**10 X** vs. 50:**40 X**	-0.6553	-0.7993 to -0.5114	Yes
11072	9:**10 X** vs. 50:**50 X**	-0.4020	-0.5460 to -0.2580	Yes
11073	9:**40 X** vs. 9:**50 X**	0.006667	-0.1373 to 0.1506	No
11074	9:**40 X** vs. 10:**0 X**	-0.01033	-0.1543 to 0.1336	No
11075	9:**40 X** vs. 10:**1 X**	-0.01733	-0.1613 to 0.1266	No
11076	9:**40 X** vs. 10:**10 X**	-0.0200	-0.1640 to 0.1240	No
11077	9:**40 X** vs. 10:**40 X**	-0.009667	-0.1536 to 0.1343	No
11078	9:**40 X** vs. 10:**50 X**	0.003667	-0.1403 to 0.1476	No
11079	9:**40 X** vs. 11:**0 X**	-0.0200	-0.1640 to 0.1240	No
11080	9:**40 X** vs. 11:**1 X**	-0.02167	-0.1656 to 0.1223	No
11081	9:**40 X** vs. 11:**10 X**	-0.0220	-0.1660 to 0.1220	No
11082	9:**40 X** vs. 11:**40 X**	-0.01367	-0.1576 to 0.1303	No
11083	9:**40 X** vs. 11:**50 X**	-0.001667	-0.1456 to 0.1423	No
11084	9:**40 X** vs. 12:**0 X**	-0.02067	-0.1646 to 0.1233	No
11085	9:**40 X** vs. 12:**1 X**	-0.0030	-0.1470 to 0.1410	No
11086	9:**40 X** vs. 12:**10 X**	-0.02067	-0.1646 to 0.1233	No
11087	9:**40 X** vs. 12:**40 X**	-0.007000	-0.1510 to 0.1370	No
11088	9:**40 X** vs. 12:**50 X**	-0.01033	-0.1543 to 0.1336	No
11089	9:**40 X** vs. 13:**0 X**	-0.01467	-0.1586 to 0.1293	No
11090	9:**40 X** vs. 13:**1 X**	-0.002000	-0.1460 to 0.1420	No
11091	9:**40 X** vs. 13:**10 X**	-0.0240	-0.1680 to 0.1200	No
11092	9:**40 X** vs. 13:**40 X**	-0.02033	-0.1643 to 0.1236	No
11093	9:**40 X** vs. 13:**50 X**	-0.02133	-0.1653 to 0.1226	No
11094	9:**40 X** vs. 14:**0 X**	-0.02333	-0.1673 to 0.1206	No
11095	9:**40 X** vs. 14:**1 X**	-0.0150	-0.1590 to 0.1290	No
11096	9:**40 X** vs. 14:**10 X**	-0.0290	-0.1730 to 0.1150	No
11097	9:**40 X** vs. 14:**40 X**	-0.02567	-0.1696 to 0.1183	No
11098	9:**40 X** vs. 14:**50 X**	-0.02967	-0.1736 to 0.1143	No
11099	9:**40 X** vs. 15:**0 X**	-0.03567	-0.1796 to 0.1083	No
11100	9:**40 X** vs. 15:**1 X**	-0.01567	-0.1596 to 0.1283	No
11101	9:**40 X** vs. 15:**10 X**	-0.0430	-0.1870 to 0.1010	No
11102	9:**40 X** vs. 15:**40 X**	-0.02533	-0.1693 to 0.1186	No
11103	9:**40 X** vs. 15:**50 X**	-0.0430	-0.1870 to 0.1010	No
11104	9:**40 X** vs. 16:**0 X**	-0.1280	-0.2720 to 0.01596	No
11105	9:**40 X** vs. 16:**1 X**	-0.0190	-0.1630 to 0.1250	No
11106	9:**40 X** vs. 16:**10 X**	-0.04467	-0.1886 to 0.09929	No
11107	9:**40 X** vs. 16:**40 X**	-0.0640	-0.2080 to 0.07996	No
11108	9:**40 X** vs. 16:**50 X**	-0.1063	-0.2503 to 0.03763	No
11109	9:**40 X** vs. 17:**0 X**	-0.1807	-0.3246 to -0.03671	Yes
11110	9:**40 X** vs. 17:**1 X**	-0.0950	-0.2390 to 0.04896	No
11111	9:**40 X** vs. 17:**10 X**	-0.1517	-0.2956 to -0.007707	Yes
11112	9:**40 X** vs. 17:**40 X**	-0.0750	-0.2190 to 0.06896	No
11113	9:**40 X** vs. 17:**50 X**	-0.1203	-0.2643 to 0.02363	No
11114	9:**40 X** vs. 18:**0 X**	-0.2293	-0.3733 to -0.08537	Yes
11115	9:**40 X** vs. 18:**1 X**	-0.1377	-0.2816 to 0.006293	No

2way ANOVA<br>Multiple comparisons		A<br>Data Set-A<br>Y	B<br>Data Set-B<br>Y	C<br>Data Set-C<br>Y
11116	9:**40 X** vs. 18:**10 X**	-0.2300	-0.3740 to -0.08604	Yes
11117	9:**40 X** vs. 18:**40 X**	-0.07933	-0.2233 to 0.06463	No
11118	9:**40 X** vs. 18:**50 X**	-0.1243	-0.2683 to 0.01963	No
11119	9:**40 X** vs. 19:**0 X**	-0.2643	-0.4083 to -0.1204	Yes
11120	9:**40 X** vs. 19:**1 X**	-0.2523	-0.3963 to -0.1084	Yes
11121	9:**40 X** vs. 19:**10 X**	-0.2500	-0.3940 to -0.1060	Yes
11122	9:**40 X** vs. 19:**40 X**	-0.1080	-0.2520 to 0.03596	No
11123	9:**40 X** vs. 19:**50 X**	-0.1190	-0.2630 to 0.02496	No
11124	9:**40 X** vs. 20:**0 X**	-0.3440	-0.4880 to -0.2000	Yes
11125	9:**40 X** vs. 20:**1 X**	-0.1633	-0.3073 to -0.01937	Yes
11126	9:**40 X** vs. 20:**10 X**	-0.2523	-0.3963 to -0.1084	Yes
11127	9:**40 X** vs. 20:**40 X**	-0.1197	-0.2636 to 0.02429	No
11128	9:**40 X** vs. 20:**50 X**	-0.1637	-0.3076 to -0.01971	Yes
11129	9:**40 X** vs. 21:**0 X**	-0.3673	-0.5113 to -0.2234	Yes
11130	9:**40 X** vs. 21:**1 X**	-0.3203	-0.4643 to -0.1764	Yes
11131	9:**40 X** vs. 21:**10 X**	-0.3173	-0.4613 to -0.1734	Yes
11132	9:**40 X** vs. 21:**40 X**	-0.1927	-0.3366 to -0.04871	Yes
11133	9:**40 X** vs. 21:**50 X**	-0.2830	-0.4270 to -0.1390	Yes
11134	9:**40 X** vs. 22:**0 X**	-0.4090	-0.5530 to -0.2650	Yes
11135	9:**40 X** vs. 22:**1 X**	-0.4037	-0.5476 to -0.2597	Yes
11136	9:**40 X** vs. 22:**10 X**	-0.3990	-0.5430 to -0.2550	Yes
11137	9:**40 X** vs. 22:**40 X**	-0.2993	-0.4433 to -0.1554	Yes
11138	9:**40 X** vs. 22:**50 X**	-0.3620	-0.5060 to -0.2180	Yes
11139	9:**40 X** vs. 23:**0 X**	-0.4743	-0.6183 to -0.3304	Yes
11140	9:**40 X** vs. 23:**1 X**	-0.6640	-0.8250 to -0.5030	Yes
11141	9:**40 X** vs. 23:**10 X**	-0.4137	-0.5576 to -0.2697	Yes
11142	9:**40 X** vs. 23:**40 X**	-0.2940	-0.4550 to -0.1330	Yes
11143	9:**40 X** vs. 23:**50 X**	-0.3280	-0.4720 to -0.1840	Yes
11144	9:**40 X** vs. 24:**0 X**	-0.5917	-0.7356 to -0.4477	Yes
11145	9:**40 X** vs. 24:**1 X**	-0.7480	-0.9090 to -0.5870	Yes
11146	9:**40 X** vs. 24:**10 X**	-0.5755	-0.7365 to -0.4145	Yes
11147	9:**40 X** vs. 24:**40 X**	-0.3103	-0.4543 to -0.1664	Yes
11148	9:**40 X** vs. 24:**50 X**	-0.2320	-0.3760 to -0.08804	Yes
11149	9:**40 X** vs. 25:**0 X**	-0.9290	-1.090 to -0.7680	Yes
11150	9:**40 X** vs. 25:**1 X**	-1.119	-1.279 to -0.9575	Yes
11151	9:**40 X** vs. 25:**10 X**	-0.4800	-0.6836 to -0.2764	Yes
11152	9:**40 X** vs. 25:**40 X**	-0.3123	-0.4563 to -0.1684	Yes
11153	9:**40 X** vs. 25:**50 X**	-0.2867	-0.4306 to -0.1427	Yes
11154	9:**40 X** vs. 26:**0 X**	-1.142	-1.302 to -0.9805	Yes
11155	9:**40 X** vs. 26:**1 X**	-1.255	-1.415 to -1.094	Yes
11156	9:**40 X** vs. 26:**10 X**	-0.9410	-1.102 to -0.7800	Yes
11157	9:**40 X** vs. 26:**40 X**	-0.3867	-0.5306 to -0.2427	Yes
11158	9:**40 X** vs. 26:**50 X**	-0.2470	-0.3910 to -0.1030	Yes
11159	9:**40 X** vs. 27:**0 X**	-1.329	-1.473 to -1.185	Yes
11160	9:**40 X** vs. 27:**1 X**	-1.420	-1.581 to -1.259	Yes

2way ANOVA<br>Multiple comparisons	A<br>Data Set-A<br>Y	B<br>Data Set-B<br>Y	C<br>Data Set-C<br>Y
11161 9:40 X vs. 27:10 X	-1.232	-1.393 to -1.071	Yes
11162 9:40 X vs. 27:40 X	-0.3867	-0.5306 to -0.2427	Yes
11163 9:40 X vs. 27:50 X	-0.2463	-0.3903 to -0.1024	Yes
11164 9:40 X vs. 28:0 X	-1.279	-1.439 to -1.118	Yes
11165 9:40 X vs. 28:1 X	-1.319	-1.463 to -1.175	Yes
11166 9:40 X vs. 28:10 X	-1.121	-1.282 to -0.9600	Yes
11167 9:40 X vs. 28:40 X	-0.3990	-0.5430 to -0.2550	Yes
11168 9:40 X vs. 28:50 X	-0.2943	-0.4383 to -0.1504	Yes
11169 9:40 X vs. 29:0 X	-1.760	-1.921 to -1.599	Yes
11170 9:40 X vs. 29:1 X	-1.845	-2.006 to -1.684	Yes
11171 9:40 X vs. 29:10 X	-1.060	-1.220 to -0.8985	Yes
11172 9:40 X vs. 29:40 X	-0.5070	-0.6510 to -0.3630	Yes
11173 9:40 X vs. 29:50 X	-0.2957	-0.4396 to -0.1517	Yes
11174 9:40 X vs. 30:0 X	-1.475	-1.635 to -1.314	Yes
11175 9:40 X vs. 30:1 X	-1.796	-1.957 to -1.635	Yes
11176 9:40 X vs. 30:10 X	-1.134	-1.295 to -0.9730	Yes
11177 9:40 X vs. 30:40 X	-0.4340	-0.5780 to -0.2900	Yes
11178 9:40 X vs. 30:50 X	-0.3480	-0.4920 to -0.2040	Yes
11179 9:40 X vs. 31:0 X	-1.793	-1.953 to -1.632	Yes
11180 9:40 X vs. 31:1 X	-1.783	-1.944 to -1.622	Yes
11181 9:40 X vs. 31:10 X	-1.266	-1.426 to -1.105	Yes
11182 9:40 X vs. 31:40 X	-0.5257	-0.6696 to -0.3817	Yes
11183 9:40 X vs. 31:50 X	-0.3270	-0.4710 to -0.1830	Yes
11184 9:40 X vs. 32:0 X	-2.384	-2.528 to -2.240	Yes
11185 9:40 X vs. 32:1 X	-1.840	-2.001 to -1.679	Yes
11186 9:40 X vs. 32:10 X	-1.696	-1.840 to -1.552	Yes
11187 9:40 X vs. 32:40 X	-0.5220	-0.6660 to -0.3780	Yes
11188 9:40 X vs. 32:50 X	-0.3267	-0.4706 to -0.1827	Yes
11189 9:40 X vs. 33:0 X	-2.388	-2.532 to -2.244	Yes
11190 9:40 X vs. 33:1 X	-2.202	-2.346 to -2.058	Yes
11191 9:40 X vs. 33:10 X	-1.863	-2.007 to -1.719	Yes
11192 9:40 X vs. 33:40 X	-0.5407	-0.6846 to -0.3967	Yes
11193 9:40 X vs. 33:50 X	-0.3417	-0.4856 to -0.1977	Yes
11194 9:40 X vs. 34:0 X	-2.312	-2.456 to -2.168	Yes
11195 9:40 X vs. 34:1 X	-2.320	-2.464 to -2.176	Yes
11196 9:40 X vs. 34:10 X	-1.953	-2.097 to -1.809	Yes
11197 9:40 X vs. 34:40 X	-0.5527	-0.6966 to -0.4087	Yes
11198 9:40 X vs. 34:50 X	-0.4907	-0.6346 to -0.3467	Yes
11199 9:40 X vs. 35:0 X	-2.661	-2.805 to -2.517	Yes
11200 9:40 X vs. 35:1 X	-2.427	-2.571 to -2.283	Yes
11201 9:40 X vs. 35:10 X	-1.915	-2.059 to -1.771	Yes
11202 9:40 X vs. 35:40 X	-0.5617	-0.7056 to -0.4177	Yes
11203 9:40 X vs. 35:50 X	-0.5033	-0.6473 to -0.3594	Yes
11204 9:40 X vs. 36:0 X	-2.868	-3.028 to -2.707	Yes
11205 9:40 X vs. 36:1 X	-2.563	-2.707 to -2.419	Yes

	2way ANOVA<br>Multiple comparisons	A<br>Data Set-A<br>Y	B<br>Data Set-B<br>Y	C<br>Data Set-C<br>Y
11206	9:**40 X** vs. 36:**10 X**	-1.920	-2.064 to -1.776	Yes
11207	9:**40 X** vs. 36:**40 X**	-0.6170	-0.7610 to -0.4730	Yes
11208	9:**40 X** vs. 36:**50 X**	-0.4667	-0.6106 to -0.3227	Yes
11209	9:**40 X** vs. 37:**0 X**	-3.251	-3.395 to -3.107	Yes
11210	9:**40 X** vs. 37:**1 X**	-2.633	-2.777 to -2.489	Yes
11211	9:**40 X** vs. 37:**10 X**	-1.922	-2.066 to -1.778	Yes
11212	9:**40 X** vs. 37:**40 X**	-0.6543	-0.7983 to -0.5104	Yes
11213	9:**40 X** vs. 37:**50 X**	-0.4753	-0.6193 to -0.3314	Yes
11214	9:**40 X** vs. 38:**0 X**	-3.196	-3.340 to -3.052	Yes
11215	9:**40 X** vs. 38:**1 X**	-2.752	-2.896 to -2.608	Yes
11216	9:**40 X** vs. 38:**10 X**	-2.197	-2.341 to -2.053	Yes
11217	9:**40 X** vs. 38:**40 X**	-0.6587	-0.8026 to -0.5147	Yes
11218	9:**40 X** vs. 38:**50 X**	-0.4793	-0.6233 to -0.3354	Yes
11219	9:**40 X** vs. 39:**0 X**	-3.427	-3.571 to -3.283	Yes
11220	9:**40 X** vs. 39:**1 X**	-3.319	-3.463 to -3.175	Yes
11221	9:**40 X** vs. 39:**10 X**	-2.342	-2.486 to -2.198	Yes
11222	9:**40 X** vs. 39:**40 X**	-0.6647	-0.8086 to -0.5207	Yes
11223	9:**40 X** vs. 39:**50 X**	-0.5103	-0.6543 to -0.3664	Yes
11224	9:**40 X** vs. 40:**0 X**	-3.468	-3.612 to -3.324	Yes
11225	9:**40 X** vs. 40:**1 X**	-3.394	-3.538 to -3.250	Yes
11226	9:**40 X** vs. 40:**10 X**	-2.599	-2.743 to -2.455	Yes
11227	9:**40 X** vs. 40:**40 X**	-0.6693	-0.8133 to -0.5254	Yes
11228	9:**40 X** vs. 40:**50 X**	-0.5167	-0.6606 to -0.3727	Yes
11229	9:**40 X** vs. 41:**0 X**	-3.509	-3.653 to -3.365	Yes
11230	9:**40 X** vs. 41:**1 X**	-3.508	-3.652 to -3.364	Yes
11231	9:**40 X** vs. 41:**10 X**	-2.652	-2.796 to -2.508	Yes
11232	9:**40 X** vs. 41:**40 X**	-0.6167	-0.7606 to -0.4727	Yes
11233	9:**40 X** vs. 41:**50 X**	-0.5293	-0.6733 to -0.3854	Yes
11234	9:**40 X** vs. 42:**0 X**	-3.651	-3.795 to -3.507	Yes
11235	9:**40 X** vs. 42:**1 X**	-3.670	-3.814 to -3.526	Yes
11236	9:**40 X** vs. 42:**10 X**	-2.653	-2.797 to -2.509	Yes
11237	9:**40 X** vs. 42:**40 X**	-0.6293	-0.7733 to -0.4854	Yes
11238	9:**40 X** vs. 42:**50 X**	-0.5327	-0.6766 to -0.3887	Yes
11239	9:**40 X** vs. 43:**0 X**	-3.741	-3.885 to -3.597	Yes
11240	9:**40 X** vs. 43:**1 X**	-3.655	-3.799 to -3.511	Yes
11241	9:**40 X** vs. 43:**10 X**	-3.371	-3.515 to -3.227	Yes
11242	9:**40 X** vs. 43:**40 X**	-0.6277	-0.7716 to -0.4837	Yes
11243	9:**40 X** vs. 43:**50 X**	-0.5330	-0.6770 to -0.3890	Yes
11244	9:**40 X** vs. 44:**0 X**	-3.765	-3.909 to -3.621	Yes
11245	9:**40 X** vs. 44:**1 X**	-3.654	-3.798 to -3.510	Yes
11246	9:**40 X** vs. 44:**10 X**	-3.434	-3.578 to -3.290	Yes
11247	9:**40 X** vs. 44:**40 X**	-0.6297	-0.7736 to -0.4857	Yes
11248	9:**40 X** vs. 44:**50 X**	-0.4653	-0.6093 to -0.3214	Yes
11249	9:**40 X** vs. 45:**0 X**	-3.629	-3.773 to -3.485	Yes
11250	9:**40 X** vs. 45:**1 X**	-3.431	-3.575 to -3.287	Yes

	2way ANOVA<br>Multiple comparisons	A<br>Data Set-A<br>Y	B<br>Data Set-B<br>Y	C<br>Data Set-C<br>Y
11251	9:40 X vs. 45:10 X	-3.314	-3.458 to -3.170	Yes
11252	9:40 X vs. 45:40 X	-0.6310	-0.7750 to -0.4870	Yes
11253	9:40 X vs. 45:50 X	-0.4623	-0.6063 to -0.3184	Yes
11254	9:40 X vs. 46:0 X	-3.434	-3.578 to -3.290	Yes
11255	9:40 X vs. 46:1 X	-3.401	-3.545 to -3.257	Yes
11256	9:40 X vs. 46:10 X	-3.309	-3.453 to -3.165	Yes
11257	9:40 X vs. 46:40 X	-0.6447	-0.7886 to -0.5007	Yes
11258	9:40 X vs. 46:50 X	-0.4553	-0.5993 to -0.3114	Yes
11259	9:40 X vs. 47:0 X	-3.409	-3.553 to -3.265	Yes
11260	9:40 X vs. 47:1 X	-3.313	-3.457 to -3.169	Yes
11261	9:40 X vs. 47:10 X	-3.295	-3.439 to -3.151	Yes
11262	9:40 X vs. 47:40 X	-0.6547	-0.7986 to -0.5107	Yes
11263	9:40 X vs. 47:50 X	-0.4133	-0.5573 to -0.2694	Yes
11264	9:40 X vs. 48:0 X	-3.397	-3.557 to -3.236	Yes
11265	9:40 X vs. 48:1 X	-3.300	-3.444 to -3.156	Yes
11266	9:40 X vs. 48:10 X	-3.295	-3.439 to -3.151	Yes
11267	9:40 X vs. 48:40 X	-0.6610	-0.8050 to -0.5170	Yes
11268	9:40 X vs. 48:50 X	-0.4207	-0.5646 to -0.2767	Yes
11269	9:40 X vs. 49:0 X	-3.397	-3.541 to -3.253	Yes
11270	9:40 X vs. 49:1 X	-3.300	-3.444 to -3.156	Yes
11271	9:40 X vs. 49:10 X	-3.295	-3.439 to -3.151	Yes
11272	9:40 X vs. 49:40 X	-0.6653	-0.8093 to -0.5214	Yes
11273	9:40 X vs. 49:50 X	-0.4200	-0.5640 to -0.2760	Yes
11274	9:40 X vs. 50:0 X	-3.397	-3.541 to -3.253	Yes
11275	9:40 X vs. 50:1 X	-3.300	-3.444 to -3.156	Yes
11276	9:40 X vs. 50:10 X	-3.295	-3.439 to -3.151	Yes
11277	9:40 X vs. 50:40 X	-0.6730	-0.8170 to -0.5290	Yes
11278	9:40 X vs. 50:50 X	-0.4197	-0.5636 to -0.2757	Yes
11279	9:50 X vs. 10:0 X	-0.0170	-0.1610 to 0.1270	No
11280	9:50 X vs. 10:1 X	-0.0240	-0.1680 to 0.1200	No
11281	9:50 X vs. 10:10 X	-0.02667	-0.1706 to 0.1173	No
11282	9:50 X vs. 10:40 X	-0.01633	-0.1603 to 0.1276	No
11283	9:50 X vs. 10:50 X	-0.003000	-0.1470 to 0.1410	No
11284	9:50 X vs. 11:0 X	-0.02667	-0.1706 to 0.1173	No
11285	9:50 X vs. 11:1 X	-0.02833	-0.1723 to 0.1156	No
11286	9:50 X vs. 11:10 X	-0.02867	-0.1726 to 0.1153	No
11287	9:50 X vs. 11:40 X	-0.02033	-0.1643 to 0.1236	No
11288	9:50 X vs. 11:50 X	-0.008333	-0.1523 to 0.1356	No
11289	9:50 X vs. 12:0 X	-0.02733	-0.1713 to 0.1166	No
11290	9:50 X vs. 12:1 X	-0.009667	-0.1536 to 0.1343	No
11291	9:50 X vs. 12:10 X	-0.02733	-0.1713 to 0.1166	No
11292	9:50 X vs. 12:40 X	-0.01367	-0.1576 to 0.1303	No
11293	9:50 X vs. 12:50 X	-0.0170	-0.1610 to 0.1270	No
11294	9:50 X vs. 13:0 X	-0.02133	-0.1653 to 0.1226	No
11295	9:50 X vs. 13:1 X	-0.008667	-0.1526 to 0.1353	No

	2way ANOVA<br>Multiple comparisons	A<br>Data Set-A<br>Y	B<br>Data Set-B<br>Y	C<br>Data Set-C<br>Y
11296	9:50 X vs. 13:10 X	-0.03067	-0.1746 to 0.1133	No
11297	9:50 X vs. 13:40 X	-0.0270	-0.1710 to 0.1170	No
11298	9:50 X vs. 13:50 X	-0.0280	-0.1720 to 0.1160	No
11299	9:50 X vs. 14:0 X	-0.0300	-0.1740 to 0.1140	No
11300	9:50 X vs. 14:1 X	-0.02167	-0.1656 to 0.1223	No
11301	9:50 X vs. 14:10 X	-0.03567	-0.1796 to 0.1083	No
11302	9:50 X vs. 14:40 X	-0.03233	-0.1763 to 0.1116	No
11303	9:50 X vs. 14:50 X	-0.03633	-0.1803 to 0.1076	No
11304	9:50 X vs. 15:0 X	-0.04233	-0.1863 to 0.1016	No
11305	9:50 X vs. 15:1 X	-0.02233	-0.1663 to 0.1216	No
11306	9:50 X vs. 15:10 X	-0.04967	-0.1936 to 0.09429	No
11307	9:50 X vs. 15:40 X	-0.0320	-0.1760 to 0.1120	No
11308	9:50 X vs. 15:50 X	-0.04967	-0.1936 to 0.09429	No
11309	9:50 X vs. 16:0 X	-0.1347	-0.2786 to 0.009293	No
11310	9:50 X vs. 16:1 X	-0.02567	-0.1696 to 0.1183	No
11311	9:50 X vs. 16:10 X	-0.05133	-0.1953 to 0.09263	No
11312	9:50 X vs. 16:40 X	-0.07067	-0.2146 to 0.07329	No
11313	9:50 X vs. 16:50 X	-0.1130	-0.2570 to 0.03096	No
11314	9:50 X vs. 17:0 X	-0.1873	-0.3313 to -0.04337	Yes
11315	9:50 X vs. 17:1 X	-0.1017	-0.2456 to 0.04229	No
11316	9:50 X vs. 17:10 X	-0.1583	-0.3023 to -0.01437	Yes
11317	9:50 X vs. 17:40 X	-0.08167	-0.2256 to 0.06229	No
11318	9:50 X vs. 17:50 X	-0.1270	-0.2710 to 0.01696	No
11319	9:50 X vs. 18:0 X	-0.2360	-0.3800 to -0.09204	Yes
11320	9:50 X vs. 18:1 X	-0.1443	-0.2883 to -0.0003735	Yes
11321	9:50 X vs. 18:10 X	-0.2367	-0.3806 to -0.09271	Yes
11322	9:50 X vs. 18:40 X	-0.0860	-0.2300 to 0.05796	No
11323	9:50 X vs. 18:50 X	-0.1310	-0.2750 to 0.01296	No
11324	9:50 X vs. 19:0 X	-0.2710	-0.4150 to -0.1270	Yes
11325	9:50 X vs. 19:1 X	-0.2590	-0.4030 to -0.1150	Yes
11326	9:50 X vs. 19:10 X	-0.2567	-0.4006 to -0.1127	Yes
11327	9:50 X vs. 19:40 X	-0.1147	-0.2586 to 0.02929	No
11328	9:50 X vs. 19:50 X	-0.1257	-0.2696 to 0.01829	No
11329	9:50 X vs. 20:0 X	-0.3507	-0.4946 to -0.2067	Yes
11330	9:50 X vs. 20:1 X	-0.1700	-0.3140 to -0.02604	Yes
11331	9:50 X vs. 20:10 X	-0.2590	-0.4030 to -0.1150	Yes
11332	9:50 X vs. 20:40 X	-0.1263	-0.2703 to 0.01763	No
11333	9:50 X vs. 20:50 X	-0.1703	-0.3143 to -0.02637	Yes
11334	9:50 X vs. 21:0 X	-0.3740	-0.5180 to -0.2300	Yes
11335	9:50 X vs. 21:1 X	-0.3270	-0.4710 to -0.1830	Yes
11336	9:50 X vs. 21:10 X	-0.3240	-0.4680 to -0.1800	Yes
11337	9:50 X vs. 21:40 X	-0.1993	-0.3433 to -0.05537	Yes
11338	9:50 X vs. 21:50 X	-0.2897	-0.4336 to -0.1457	Yes
11339	9:50 X vs. 22:0 X	-0.4157	-0.5596 to -0.2717	Yes
11340	9:50 X vs. 22:1 X	-0.4103	-0.5543 to -0.2664	Yes

2way ANOVA<br>Multiple comparisons	A<br>Data Set-A<br>Y	B<br>Data Set-B<br>Y	C<br>Data Set-C<br>Y
11341 9:50 X vs. 22:10 X	-0.4057	-0.5496 to -0.2617	Yes
11342 9:50 X vs. 22:40 X	-0.3060	-0.4500 to -0.1620	Yes
11343 9:50 X vs. 22:50 X	-0.3687	-0.5126 to -0.2247	Yes
11344 9:50 X vs. 23:0 X	-0.4810	-0.6250 to -0.3370	Yes
11345 9:50 X vs. 23:1 X	-0.6707	-0.8316 to -0.5097	Yes
11346 9:50 X vs. 23:10 X	-0.4203	-0.5643 to -0.2764	Yes
11347 9:50 X vs. 23:40 X	-0.3007	-0.4616 to -0.1397	Yes
11348 9:50 X vs. 23:50 X	-0.3347	-0.4786 to -0.1907	Yes
11349 9:50 X vs. 24:0 X	-0.5983	-0.7423 to -0.4544	Yes
11350 9:50 X vs. 24:1 X	-0.7547	-0.9156 to -0.5937	Yes
11351 9:50 X vs. 24:10 X	-0.5822	-0.7431 to -0.4212	Yes
11352 9:50 X vs. 24:40 X	-0.3170	-0.4610 to -0.1730	Yes
11353 9:50 X vs. 24:50 X	-0.2387	-0.3826 to -0.09471	Yes
11354 9:50 X vs. 25:0 X	-0.9357	-1.097 to -0.7747	Yes
11355 9:50 X vs. 25:1 X	-1.125	-1.286 to -0.9642	Yes
11356 9:50 X vs. 25:10 X	-0.4867	-0.6903 to -0.2831	Yes
11357 9:50 X vs. 25:40 X	-0.3190	-0.4630 to -0.1750	Yes
11358 9:50 X vs. 25:50 X	-0.2933	-0.4373 to -0.1494	Yes
11359 9:50 X vs. 26:0 X	-1.148	-1.309 to -0.9872	Yes
11360 9:50 X vs. 26:1 X	-1.261	-1.422 to -1.100	Yes
11361 9:50 X vs. 26:10 X	-0.9477	-1.109 to -0.7867	Yes
11362 9:50 X vs. 26:40 X	-0.3933	-0.5373 to -0.2494	Yes
11363 9:50 X vs. 26:50 X	-0.2537	-0.3976 to -0.1097	Yes
11364 9:50 X vs. 27:0 X	-1.336	-1.480 to -1.192	Yes
11365 9:50 X vs. 27:1 X	-1.427	-1.588 to -1.266	Yes
11366 9:50 X vs. 27:10 X	-1.239	-1.400 to -1.078	Yes
11367 9:50 X vs. 27:40 X	-0.3933	-0.5373 to -0.2494	Yes
11368 9:50 X vs. 27:50 X	-0.2530	-0.3970 to -0.1090	Yes
11369 9:50 X vs. 28:0 X	-1.285	-1.446 to -1.124	Yes
11370 9:50 X vs. 28:1 X	-1.326	-1.470 to -1.182	Yes
11371 9:50 X vs. 28:10 X	-1.128	-1.289 to -0.9667	Yes
11372 9:50 X vs. 28:40 X	-0.4057	-0.5496 to -0.2617	Yes
11373 9:50 X vs. 28:50 X	-0.3010	-0.4450 to -0.1570	Yes
11374 9:50 X vs. 29:0 X	-1.767	-1.928 to -1.606	Yes
11375 9:50 X vs. 29:1 X	-1.852	-2.013 to -1.691	Yes
11376 9:50 X vs. 29:10 X	-1.066	-1.227 to -0.9052	Yes
11377 9:50 X vs. 29:40 X	-0.5137	-0.6576 to -0.3697	Yes
11378 9:50 X vs. 29:50 X	-0.3023	-0.4463 to -0.1584	Yes
11379 9:50 X vs. 30:0 X	-1.481	-1.642 to -1.320	Yes
11380 9:50 X vs. 30:1 X	-1.803	-1.964 to -1.642	Yes
11381 9:50 X vs. 30:10 X	-1.141	-1.302 to -0.9797	Yes
11382 9:50 X vs. 30:40 X	-0.4407	-0.5846 to -0.2967	Yes
11383 9:50 X vs. 30:50 X	-0.3547	-0.4986 to -0.2107	Yes
11384 9:50 X vs. 31:0 X	-1.799	-1.960 to -1.638	Yes
11385 9:50 X vs. 31:1 X	-1.790	-1.951 to -1.629	Yes

	2way ANOVA<br>Multiple comparisons	A<br>Data Set-A<br>Y	B<br>Data Set-B<br>Y	C<br>Data Set-C<br>Y
11386	9:**50 X** vs. 31:**10 X**	-1.272	-1.433 to -1.111	Yes
11387	9:**50 X** vs. 31:**40 X**	-0.5323	-0.6763 to -0.3884	Yes
11388	9:**50 X** vs. 31:**50 X**	-0.3337	-0.4776 to -0.1897	Yes
11389	9:**50 X** vs. 32:**0 X**	-2.391	-2.535 to -2.247	Yes
11390	9:**50 X** vs. 32:**1 X**	-1.847	-2.008 to -1.686	Yes
11391	9:**50 X** vs. 32:**10 X**	-1.703	-1.847 to -1.559	Yes
11392	9:**50 X** vs. 32:**40 X**	-0.5287	-0.6726 to -0.3847	Yes
11393	9:**50 X** vs. 32:**50 X**	-0.3333	-0.4773 to -0.1894	Yes
11394	9:**50 X** vs. 33:**0 X**	-2.394	-2.538 to -2.250	Yes
11395	9:**50 X** vs. 33:**1 X**	-2.209	-2.353 to -2.065	Yes
11396	9:**50 X** vs. 33:**10 X**	-1.869	-2.013 to -1.725	Yes
11397	9:**50 X** vs. 33:**40 X**	-0.5473	-0.6913 to -0.4034	Yes
11398	9:**50 X** vs. 33:**50 X**	-0.3483	-0.4923 to -0.2044	Yes
11399	9:**50 X** vs. 34:**0 X**	-2.319	-2.463 to -2.175	Yes
11400	9:**50 X** vs. 34:**1 X**	-2.326	-2.470 to -2.182	Yes
11401	9:**50 X** vs. 34:**10 X**	-1.960	-2.104 to -1.816	Yes
11402	9:**50 X** vs. 34:**40 X**	-0.5593	-0.7033 to -0.4154	Yes
11403	9:**50 X** vs. 34:**50 X**	-0.4973	-0.6413 to -0.3534	Yes
11404	9:**50 X** vs. 35:**0 X**	-2.668	-2.812 to -2.524	Yes
11405	9:**50 X** vs. 35:**1 X**	-2.433	-2.577 to -2.289	Yes
11406	9:**50 X** vs. 35:**10 X**	-1.921	-2.065 to -1.777	Yes
11407	9:**50 X** vs. 35:**40 X**	-0.5683	-0.7123 to -0.4244	Yes
11408	9:**50 X** vs. 35:**50 X**	-0.5100	-0.6540 to -0.3660	Yes
11409	9:**50 X** vs. 36:**0 X**	-2.874	-3.035 to -2.713	Yes
11410	9:**50 X** vs. 36:**1 X**	-2.570	-2.714 to -2.426	Yes
11411	9:**50 X** vs. 36:**10 X**	-1.927	-2.071 to -1.783	Yes
11412	9:**50 X** vs. 36:**40 X**	-0.6237	-0.7676 to -0.4797	Yes
11413	9:**50 X** vs. 36:**50 X**	-0.4733	-0.6173 to -0.3294	Yes
11414	9:**50 X** vs. 37:**0 X**	-3.258	-3.402 to -3.114	Yes
11415	9:**50 X** vs. 37:**1 X**	-2.640	-2.784 to -2.496	Yes
11416	9:**50 X** vs. 37:**10 X**	-1.929	-2.073 to -1.785	Yes
11417	9:**50 X** vs. 37:**40 X**	-0.6610	-0.8050 to -0.5170	Yes
11418	9:**50 X** vs. 37:**50 X**	-0.4820	-0.6260 to -0.3380	Yes
11419	9:**50 X** vs. 38:**0 X**	-3.203	-3.347 to -3.059	Yes
11420	9:**50 X** vs. 38:**1 X**	-2.759	-2.903 to -2.615	Yes
11421	9:**50 X** vs. 38:**10 X**	-2.204	-2.348 to -2.060	Yes
11422	9:**50 X** vs. 38:**40 X**	-0.6653	-0.8093 to -0.5214	Yes
11423	9:**50 X** vs. 38:**50 X**	-0.4860	-0.6300 to -0.3420	Yes
11424	9:**50 X** vs. 39:**0 X**	-3.434	-3.578 to -3.290	Yes
11425	9:**50 X** vs. 39:**1 X**	-3.325	-3.469 to -3.181	Yes
11426	9:**50 X** vs. 39:**10 X**	-2.349	-2.493 to -2.205	Yes
11427	9:**50 X** vs. 39:**40 X**	-0.6713	-0.8153 to -0.5274	Yes
11428	9:**50 X** vs. 39:**50 X**	-0.5170	-0.6610 to -0.3730	Yes
11429	9:**50 X** vs. 40:**0 X**	-3.475	-3.619 to -3.331	Yes
11430	9:**50 X** vs. 40:**1 X**	-3.401	-3.545 to -3.257	Yes

2way ANOVA<br>Multiple comparisons	A<br>Data Set-A<br>Y	B<br>Data Set-B<br>Y	C<br>Data Set-C<br>Y
11431 9:50 X vs. 40:10 X	-2.605	-2.749 to -2.461	Yes
11432 9:50 X vs. 40:40 X	-0.6760	-0.8200 to -0.5320	Yes
11433 9:50 X vs. 40:50 X	-0.5233	-0.6673 to -0.3794	Yes
11434 9:50 X vs. 41:0 X	-3.516	-3.660 to -3.372	Yes
11435 9:50 X vs. 41:1 X	-3.515	-3.659 to -3.371	Yes
11436 9:50 X vs. 41:10 X	-2.659	-2.803 to -2.515	Yes
11437 9:50 X vs. 41:40 X	-0.6233	-0.7673 to -0.4794	Yes
11438 9:50 X vs. 41:50 X	-0.5360	-0.6800 to -0.3920	Yes
11439 9:50 X vs. 42:0 X	-3.657	-3.801 to -3.513	Yes
11440 9:50 X vs. 42:1 X	-3.677	-3.821 to -3.533	Yes
11441 9:50 X vs. 42:10 X	-2.659	-2.803 to -2.515	Yes
11442 9:50 X vs. 42:40 X	-0.6360	-0.7800 to -0.4920	Yes
11443 9:50 X vs. 42:50 X	-0.5393	-0.6833 to -0.3954	Yes
11444 9:50 X vs. 43:0 X	-3.747	-3.891 to -3.603	Yes
11445 9:50 X vs. 43:1 X	-3.662	-3.806 to -3.518	Yes
11446 9:50 X vs. 43:10 X	-3.377	-3.521 to -3.233	Yes
11447 9:50 X vs. 43:40 X	-0.6343	-0.7783 to -0.4904	Yes
11448 9:50 X vs. 43:50 X	-0.5397	-0.6836 to -0.3957	Yes
11449 9:50 X vs. 44:0 X	-3.771	-3.915 to -3.627	Yes
11450 9:50 X vs. 44:1 X	-3.661	-3.805 to -3.517	Yes
11451 9:50 X vs. 44:10 X	-3.441	-3.585 to -3.297	Yes
11452 9:50 X vs. 44:40 X	-0.6363	-0.7803 to -0.4924	Yes
11453 9:50 X vs. 44:50 X	-0.4720	-0.6160 to -0.3280	Yes
11454 9:50 X vs. 45:0 X	-3.636	-3.780 to -3.492	Yes
11455 9:50 X vs. 45:1 X	-3.438	-3.582 to -3.294	Yes
11456 9:50 X vs. 45:10 X	-3.320	-3.464 to -3.176	Yes
11457 9:50 X vs. 45:40 X	-0.6377	-0.7816 to -0.4937	Yes
11458 9:50 X vs. 45:50 X	-0.4690	-0.6130 to -0.3250	Yes
11459 9:50 X vs. 46:0 X	-3.440	-3.584 to -3.296	Yes
11460 9:50 X vs. 46:1 X	-3.407	-3.551 to -3.263	Yes
11461 9:50 X vs. 46:10 X	-3.315	-3.459 to -3.171	Yes
11462 9:50 X vs. 46:40 X	-0.6513	-0.7953 to -0.5074	Yes
11463 9:50 X vs. 46:50 X	-0.4620	-0.6060 to -0.3180	Yes
11464 9:50 X vs. 47:0 X	-3.416	-3.560 to -3.272	Yes
11465 9:50 X vs. 47:1 X	-3.320	-3.464 to -3.176	Yes
11466 9:50 X vs. 47:10 X	-3.301	-3.445 to -3.157	Yes
11467 9:50 X vs. 47:40 X	-0.6613	-0.8053 to -0.5174	Yes
11468 9:50 X vs. 47:50 X	-0.4200	-0.5640 to -0.2760	Yes
11469 9:50 X vs. 48:0 X	-3.403	-3.564 to -3.242	Yes
11470 9:50 X vs. 48:1 X	-3.307	-3.451 to -3.163	Yes
11471 9:50 X vs. 48:10 X	-3.301	-3.445 to -3.157	Yes
11472 9:50 X vs. 48:40 X	-0.6677	-0.8116 to -0.5237	Yes
11473 9:50 X vs. 48:50 X	-0.4273	-0.5713 to -0.2834	Yes
11474 9:50 X vs. 49:0 X	-3.403	-3.547 to -3.259	Yes
11475 9:50 X vs. 49:1 X	-3.307	-3.451 to -3.163	Yes

2way ANOVA<br>Multiple comparisons	A<br>Data Set-A<br>Y	B<br>Data Set-B<br>Y	C<br>Data Set-C<br>Y
11476 9:**50 X** vs. 49:**10 X**	-3.301	-3.445 to -3.157	Yes
11477 9:**50 X** vs. 49:**40 X**	-0.6720	-0.8160 to -0.5280	Yes
11478 9:**50 X** vs. 49:**50 X**	-0.4267	-0.5706 to -0.2827	Yes
11479 9:**50 X** vs. 50:**0 X**	-3.403	-3.547 to -3.259	Yes
11480 9:**50 X** vs. 50:**1 X**	-3.307	-3.451 to -3.163	Yes
11481 9:**50 X** vs. 50:**10 X**	-3.301	-3.445 to -3.157	Yes
11482 9:**50 X** vs. 50:**40 X**	-0.6797	-0.8236 to -0.5357	Yes
11483 9:**50 X** vs. 50:**50 X**	-0.4263	-0.5703 to -0.2824	Yes
11484 10:**0 X** vs. 10:**1 X**	-0.007000	-0.1510 to 0.1370	No
11485 10:**0 X** vs. 10:**10 X**	-0.009667	-0.1536 to 0.1343	No
11486 10:**0 X** vs. 10:**40 X**	0.0006667	-0.1433 to 0.1446	No
11487 10:**0 X** vs. 10:**50 X**	0.0140	-0.1300 to 0.1580	No
11488 10:**0 X** vs. 11:**0 X**	-0.009667	-0.1536 to 0.1343	No
11489 10:**0 X** vs. 11:**1 X**	-0.01133	-0.1553 to 0.1326	No
11490 10:**0 X** vs. 11:**10 X**	-0.01167	-0.1556 to 0.1323	No
11491 10:**0 X** vs. 11:**40 X**	-0.003333	-0.1473 to 0.1406	No
11492 10:**0 X** vs. 11:**50 X**	0.008667	-0.1353 to 0.1526	No
11493 10:**0 X** vs. 12:**0 X**	-0.01033	-0.1543 to 0.1336	No
11494 10:**0 X** vs. 12:**1 X**	0.007333	-0.1366 to 0.1513	No
11495 10:**0 X** vs. 12:**10 X**	-0.01033	-0.1543 to 0.1336	No
11496 10:**0 X** vs. 12:**40 X**	0.003333	-0.1406 to 0.1473	No
11497 10:**0 X** vs. 12:**50 X**	0.0	-0.1440 to 0.1440	No
11498 10:**0 X** vs. 13:**0 X**	-0.004333	-0.1483 to 0.1396	No
11499 10:**0 X** vs. 13:**1 X**	0.008333	-0.1356 to 0.1523	No
11500 10:**0 X** vs. 13:**10 X**	-0.01367	-0.1576 to 0.1303	No
11501 10:**0 X** vs. 13:**40 X**	-0.01000	-0.1540 to 0.1340	No
11502 10:**0 X** vs. 13:**50 X**	-0.0110	-0.1550 to 0.1330	No
11503 10:**0 X** vs. 14:**0 X**	-0.0130	-0.1570 to 0.1310	No
11504 10:**0 X** vs. 14:**1 X**	-0.004667	-0.1486 to 0.1393	No
11505 10:**0 X** vs. 14:**10 X**	-0.01867	-0.1626 to 0.1253	No
11506 10:**0 X** vs. 14:**40 X**	-0.01533	-0.1593 to 0.1286	No
11507 10:**0 X** vs. 14:**50 X**	-0.01933	-0.1633 to 0.1246	No
11508 10:**0 X** vs. 15:**0 X**	-0.02533	-0.1693 to 0.1186	No
11509 10:**0 X** vs. 15:**1 X**	-0.005333	-0.1493 to 0.1386	No
11510 10:**0 X** vs. 15:**10 X**	-0.03267	-0.1766 to 0.1113	No
11511 10:**0 X** vs. 15:**40 X**	-0.0150	-0.1590 to 0.1290	No
11512 10:**0 X** vs. 15:**50 X**	-0.03267	-0.1766 to 0.1113	No
11513 10:**0 X** vs. 16:**0 X**	-0.1177	-0.2616 to 0.02629	No
11514 10:**0 X** vs. 16:**1 X**	-0.008667	-0.1526 to 0.1353	No
11515 10:**0 X** vs. 16:**10 X**	-0.03433	-0.1783 to 0.1096	No
11516 10:**0 X** vs. 16:**40 X**	-0.05367	-0.1976 to 0.09029	No
11517 10:**0 X** vs. 16:**50 X**	-0.09600	-0.2400 to 0.04796	No
11518 10:**0 X** vs. 17:**0 X**	-0.1703	-0.3143 to -0.02637	Yes
11519 10:**0 X** vs. 17:**1 X**	-0.08467	-0.2286 to 0.05929	No
11520 10:**0 X** vs. 17:**10 X**	-0.1413	-0.2853 to 0.002626	No

2way ANOVA<br>Multiple comparisons	A<br>Data Set-A<br>Y	B<br>Data Set-B<br>Y	C<br>Data Set-C<br>Y
11521 10:**0 X** vs. 17:**40 X**	-0.06467	-0.2086 to 0.07929	No
11522 10:**0 X** vs. 17:**50 X**	-0.1100	-0.2540 to 0.03396	No
11523 10:**0 X** vs. 18:**0 X**	-0.2190	-0.3630 to -0.07504	Yes
11524 10:**0 X** vs. 18:**1 X**	-0.1273	-0.2713 to 0.01663	No
11525 10:**0 X** vs. 18:**10 X**	-0.2197	-0.3636 to -0.07571	Yes
11526 10:**0 X** vs. 18:**40 X**	-0.0690	-0.2130 to 0.07496	No
11527 10:**0 X** vs. 18:**50 X**	-0.1140	-0.2580 to 0.02996	No
11528 10:**0 X** vs. 19:**0 X**	-0.2540	-0.3980 to -0.1100	Yes
11529 10:**0 X** vs. 19:**1 X**	-0.2420	-0.3860 to -0.09804	Yes
11530 10:**0 X** vs. 19:**10 X**	-0.2397	-0.3836 to -0.09571	Yes
11531 10:**0 X** vs. 19:**40 X**	-0.09767	-0.2416 to 0.04629	No
11532 10:**0 X** vs. 19:**50 X**	-0.1087	-0.2526 to 0.03529	No
11533 10:**0 X** vs. 20:**0 X**	-0.3337	-0.4776 to -0.1897	Yes
11534 10:**0 X** vs. 20:**1 X**	-0.1530	-0.2970 to -0.009040	Yes
11535 10:**0 X** vs. 20:**10 X**	-0.2420	-0.3860 to -0.09804	Yes
11536 10:**0 X** vs. 20:**40 X**	-0.1093	-0.2533 to 0.03463	No
11537 10:**0 X** vs. 20:**50 X**	-0.1533	-0.2973 to -0.009374	Yes
11538 10:**0 X** vs. 21:**0 X**	-0.3570	-0.5010 to -0.2130	Yes
11539 10:**0 X** vs. 21:**1 X**	-0.3100	-0.4540 to -0.1660	Yes
11540 10:**0 X** vs. 21:**10 X**	-0.3070	-0.4510 to -0.1630	Yes
11541 10:**0 X** vs. 21:**40 X**	-0.1823	-0.3263 to -0.03837	Yes
11542 10:**0 X** vs. 21:**50 X**	-0.2727	-0.4166 to -0.1287	Yes
11543 10:**0 X** vs. 22:**0 X**	-0.3987	-0.5426 to -0.2547	Yes
11544 10:**0 X** vs. 22:**1 X**	-0.3933	-0.5373 to -0.2494	Yes
11545 10:**0 X** vs. 22:**10 X**	-0.3887	-0.5326 to -0.2447	Yes
11546 10:**0 X** vs. 22:**40 X**	-0.2890	-0.4330 to -0.1450	Yes
11547 10:**0 X** vs. 22:**50 X**	-0.3517	-0.4956 to -0.2077	Yes
11548 10:**0 X** vs. 23:**0 X**	-0.4640	-0.6080 to -0.3200	Yes
11549 10:**0 X** vs. 23:**1 X**	-0.6537	-0.8146 to -0.4927	Yes
11550 10:**0 X** vs. 23:**10 X**	-0.4033	-0.5473 to -0.2594	Yes
11551 10:**0 X** vs. 23:**40 X**	-0.2837	-0.4446 to -0.1227	Yes
11552 10:**0 X** vs. 23:**50 X**	-0.3177	-0.4616 to -0.1737	Yes
11553 10:**0 X** vs. 24:**0 X**	-0.5813	-0.7253 to -0.4374	Yes
11554 10:**0 X** vs. 24:**1 X**	-0.7377	-0.8986 to -0.5767	Yes
11555 10:**0 X** vs. 24:**10 X**	-0.5652	-0.7261 to -0.4042	Yes
11556 10:**0 X** vs. 24:**40 X**	-0.3000	-0.4440 to -0.1560	Yes
11557 10:**0 X** vs. 24:**50 X**	-0.2217	-0.3656 to -0.07771	Yes
11558 10:**0 X** vs. 25:**0 X**	-0.9187	-1.080 to -0.7577	Yes
11559 10:**0 X** vs. 25:**1 X**	-1.108	-1.269 to -0.9472	Yes
11560 10:**0 X** vs. 25:**10 X**	-0.4697	-0.6733 to -0.2661	Yes
11561 10:**0 X** vs. 25:**40 X**	-0.3020	-0.4460 to -0.1580	Yes
11562 10:**0 X** vs. 25:**50 X**	-0.2763	-0.4203 to -0.1324	Yes
11563 10:**0 X** vs. 26:**0 X**	-1.131	-1.292 to -0.9702	Yes
11564 10:**0 X** vs. 26:**1 X**	-1.244	-1.405 to -1.083	Yes
11565 10:**0 X** vs. 26:**10 X**	-0.9307	-1.092 to -0.7697	Yes

	2way ANOVA<br>Multiple comparisons	A<br>Data Set-A<br>Y	B<br>Data Set-B<br>Y	C<br>Data Set-C<br>Y
11566	10:**0 X** vs. 26:**40 X**	-0.3763	-0.5203 to -0.2324	Yes
11567	10:**0 X** vs. 26:**50 X**	-0.2367	-0.3806 to -0.09271	Yes
11568	10:**0 X** vs. 27:**0 X**	-1.319	-1.463 to -1.175	Yes
11569	10:**0 X** vs. 27:**1 X**	-1.410	-1.571 to -1.249	Yes
11570	10:**0 X** vs. 27:**10 X**	-1.222	-1.383 to -1.061	Yes
11571	10:**0 X** vs. 27:**40 X**	-0.3763	-0.5203 to -0.2324	Yes
11572	10:**0 X** vs. 27:**50 X**	-0.2360	-0.3800 to -0.09204	Yes
11573	10:**0 X** vs. 28:**0 X**	-1.268	-1.429 to -1.107	Yes
11574	10:**0 X** vs. 28:**1 X**	-1.309	-1.453 to -1.165	Yes
11575	10:**0 X** vs. 28:**10 X**	-1.111	-1.272 to -0.9497	Yes
11576	10:**0 X** vs. 28:**40 X**	-0.3887	-0.5326 to -0.2447	Yes
11577	10:**0 X** vs. 28:**50 X**	-0.2840	-0.4280 to -0.1400	Yes
11578	10:**0 X** vs. 29:**0 X**	-1.750	-1.911 to -1.589	Yes
11579	10:**0 X** vs. 29:**1 X**	-1.835	-1.996 to -1.674	Yes
11580	10:**0 X** vs. 29:**10 X**	-1.049	-1.210 to -0.8882	Yes
11581	10:**0 X** vs. 29:**40 X**	-0.4967	-0.6406 to -0.3527	Yes
11582	10:**0 X** vs. 29:**50 X**	-0.2853	-0.4293 to -0.1414	Yes
11583	10:**0 X** vs. 30:**0 X**	-1.464	-1.625 to -1.303	Yes
11584	10:**0 X** vs. 30:**1 X**	-1.786	-1.947 to -1.625	Yes
11585	10:**0 X** vs. 30:**10 X**	-1.124	-1.285 to -0.9627	Yes
11586	10:**0 X** vs. 30:**40 X**	-0.4237	-0.5676 to -0.2797	Yes
11587	10:**0 X** vs. 30:**50 X**	-0.3377	-0.4816 to -0.1937	Yes
11588	10:**0 X** vs. 31:**0 X**	-1.782	-1.943 to -1.621	Yes
11589	10:**0 X** vs. 31:**1 X**	-1.773	-1.934 to -1.612	Yes
11590	10:**0 X** vs. 31:**10 X**	-1.255	-1.416 to -1.094	Yes
11591	10:**0 X** vs. 31:**40 X**	-0.5153	-0.6593 to -0.3714	Yes
11592	10:**0 X** vs. 31:**50 X**	-0.3167	-0.4606 to -0.1727	Yes
11593	10:**0 X** vs. 32:**0 X**	-2.374	-2.518 to -2.230	Yes
11594	10:**0 X** vs. 32:**1 X**	-1.830	-1.991 to -1.669	Yes
11595	10:**0 X** vs. 32:**10 X**	-1.686	-1.830 to -1.542	Yes
11596	10:**0 X** vs. 32:**40 X**	-0.5117	-0.6556 to -0.3677	Yes
11597	10:**0 X** vs. 32:**50 X**	-0.3163	-0.4603 to -0.1724	Yes
11598	10:**0 X** vs. 33:**0 X**	-2.377	-2.521 to -2.233	Yes
11599	10:**0 X** vs. 33:**1 X**	-2.192	-2.336 to -2.048	Yes
11600	10:**0 X** vs. 33:**10 X**	-1.852	-1.996 to -1.708	Yes
11601	10:**0 X** vs. 33:**40 X**	-0.5303	-0.6743 to -0.3864	Yes
11602	10:**0 X** vs. 33:**50 X**	-0.3313	-0.4753 to -0.1874	Yes
11603	10:**0 X** vs. 34:**0 X**	-2.302	-2.446 to -2.158	Yes
11604	10:**0 X** vs. 34:**1 X**	-2.309	-2.453 to -2.165	Yes
11605	10:**0 X** vs. 34:**10 X**	-1.943	-2.087 to -1.799	Yes
11606	10:**0 X** vs. 34:**40 X**	-0.5423	-0.6863 to -0.3984	Yes
11607	10:**0 X** vs. 34:**50 X**	-0.4803	-0.6243 to -0.3364	Yes
11608	10:**0 X** vs. 35:**0 X**	-2.651	-2.795 to -2.507	Yes
11609	10:**0 X** vs. 35:**1 X**	-2.416	-2.560 to -2.272	Yes
11610	10:**0 X** vs. 35:**10 X**	-1.904	-2.048 to -1.760	Yes

2way ANOVA Multiple comparisons	A Data Set-A Y	B Data Set-B Y	C Data Set-C Y
11611 10:**0 X** vs. 35:**40 X**	-0.5513	-0.6953 to -0.4074	Yes
11612 10:**0 X** vs. 35:**50 X**	-0.4930	-0.6370 to -0.3490	Yes
11613 10:**0 X** vs. 36:**0 X**	-2.857	-3.018 to -2.696	Yes
11614 10:**0 X** vs. 36:**1 X**	-2.553	-2.697 to -2.409	Yes
11615 10:**0 X** vs. 36:**10 X**	-1.910	-2.054 to -1.766	Yes
11616 10:**0 X** vs. 36:**40 X**	-0.6067	-0.7506 to -0.4627	Yes
11617 10:**0 X** vs. 36:**50 X**	-0.4563	-0.6003 to -0.3124	Yes
11618 10:**0 X** vs. 37:**0 X**	-3.241	-3.385 to -3.097	Yes
11619 10:**0 X** vs. 37:**1 X**	-2.623	-2.767 to -2.479	Yes
11620 10:**0 X** vs. 37:**10 X**	-1.912	-2.056 to -1.768	Yes
11621 10:**0 X** vs. 37:**40 X**	-0.6440	-0.7880 to -0.5000	Yes
11622 10:**0 X** vs. 37:**50 X**	-0.4650	-0.6090 to -0.3210	Yes
11623 10:**0 X** vs. 38:**0 X**	-3.186	-3.330 to -3.042	Yes
11624 10:**0 X** vs. 38:**1 X**	-2.742	-2.886 to -2.598	Yes
11625 10:**0 X** vs. 38:**10 X**	-2.187	-2.331 to -2.043	Yes
11626 10:**0 X** vs. 38:**40 X**	-0.6483	-0.7923 to -0.5044	Yes
11627 10:**0 X** vs. 38:**50 X**	-0.4690	-0.6130 to -0.3250	Yes
11628 10:**0 X** vs. 39:**0 X**	-3.417	-3.561 to -3.273	Yes
11629 10:**0 X** vs. 39:**1 X**	-3.308	-3.452 to -3.164	Yes
11630 10:**0 X** vs. 39:**10 X**	-2.332	-2.476 to -2.188	Yes
11631 10:**0 X** vs. 39:**40 X**	-0.6543	-0.7983 to -0.5104	Yes
11632 10:**0 X** vs. 39:**50 X**	-0.5000	-0.6440 to -0.3560	Yes
11633 10:**0 X** vs. 40:**0 X**	-3.458	-3.602 to -3.314	Yes
11634 10:**0 X** vs. 40:**1 X**	-3.384	-3.528 to -3.240	Yes
11635 10:**0 X** vs. 40:**10 X**	-2.588	-2.732 to -2.444	Yes
11636 10:**0 X** vs. 40:**40 X**	-0.6590	-0.8030 to -0.5150	Yes
11637 10:**0 X** vs. 40:**50 X**	-0.5063	-0.6503 to -0.3624	Yes
11638 10:**0 X** vs. 41:**0 X**	-3.499	-3.643 to -3.355	Yes
11639 10:**0 X** vs. 41:**1 X**	-3.498	-3.642 to -3.354	Yes
11640 10:**0 X** vs. 41:**10 X**	-2.642	-2.786 to -2.498	Yes
11641 10:**0 X** vs. 41:**40 X**	-0.6063	-0.7503 to -0.4624	Yes
11642 10:**0 X** vs. 41:**50 X**	-0.5190	-0.6630 to -0.3750	Yes
11643 10:**0 X** vs. 42:**0 X**	-3.640	-3.784 to -3.496	Yes
11644 10:**0 X** vs. 42:**1 X**	-3.660	-3.804 to -3.516	Yes
11645 10:**0 X** vs. 42:**10 X**	-2.642	-2.786 to -2.498	Yes
11646 10:**0 X** vs. 42:**40 X**	-0.6190	-0.7630 to -0.4750	Yes
11647 10:**0 X** vs. 42:**50 X**	-0.5223	-0.6663 to -0.3784	Yes
11648 10:**0 X** vs. 43:**0 X**	-3.730	-3.874 to -3.586	Yes
11649 10:**0 X** vs. 43:**1 X**	-3.645	-3.789 to -3.501	Yes
11650 10:**0 X** vs. 43:**10 X**	-3.360	-3.504 to -3.216	Yes
11651 10:**0 X** vs. 43:**40 X**	-0.6173	-0.7613 to -0.4734	Yes
11652 10:**0 X** vs. 43:**50 X**	-0.5227	-0.6666 to -0.3787	Yes
11653 10:**0 X** vs. 44:**0 X**	-3.754	-3.898 to -3.610	Yes
11654 10:**0 X** vs. 44:**1 X**	-3.644	-3.788 to -3.500	Yes
11655 10:**0 X** vs. 44:**10 X**	-3.424	-3.568 to -3.280	Yes

2way ANOVA<br>Multiple comparisons	A<br>Data Set-A<br>Y	B<br>Data Set-B<br>Y	C<br>Data Set-C<br>Y
11656 10:0 X vs. 44:40 X	-0.6193	-0.7633 to -0.4754	Yes
11657 10:0 X vs. 44:50 X	-0.4550	-0.5990 to -0.3110	Yes
11658 10:0 X vs. 45:0 X	-3.619	-3.763 to -3.475	Yes
11659 10:0 X vs. 45:1 X	-3.421	-3.565 to -3.277	Yes
11660 10:0 X vs. 45:10 X	-3.303	-3.447 to -3.159	Yes
11661 10:0 X vs. 45:40 X	-0.6207	-0.7646 to -0.4767	Yes
11662 10:0 X vs. 45:50 X	-0.4520	-0.5960 to -0.3080	Yes
11663 10:0 X vs. 46:0 X	-3.423	-3.567 to -3.279	Yes
11664 10:0 X vs. 46:1 X	-3.390	-3.534 to -3.246	Yes
11665 10:0 X vs. 46:10 X	-3.298	-3.442 to -3.154	Yes
11666 10:0 X vs. 46:40 X	-0.6343	-0.7783 to -0.4904	Yes
11667 10:0 X vs. 46:50 X	-0.4450	-0.5890 to -0.3010	Yes
11668 10:0 X vs. 47:0 X	-3.399	-3.543 to -3.255	Yes
11669 10:0 X vs. 47:1 X	-3.303	-3.447 to -3.159	Yes
11670 10:0 X vs. 47:10 X	-3.284	-3.428 to -3.140	Yes
11671 10:0 X vs. 47:40 X	-0.6443	-0.7883 to -0.5004	Yes
11672 10:0 X vs. 47:50 X	-0.4030	-0.5470 to -0.2590	Yes
11673 10:0 X vs. 48:0 X	-3.386	-3.547 to -3.225	Yes
11674 10:0 X vs. 48:1 X	-3.290	-3.434 to -3.146	Yes
11675 10:0 X vs. 48:10 X	-3.284	-3.428 to -3.140	Yes
11676 10:0 X vs. 48:40 X	-0.6507	-0.7946 to -0.5067	Yes
11677 10:0 X vs. 48:50 X	-0.4103	-0.5543 to -0.2664	Yes
11678 10:0 X vs. 49:0 X	-3.386	-3.530 to -3.242	Yes
11679 10:0 X vs. 49:1 X	-3.290	-3.434 to -3.146	Yes
11680 10:0 X vs. 49:10 X	-3.284	-3.428 to -3.140	Yes
11681 10:0 X vs. 49:40 X	-0.6550	-0.7990 to -0.5110	Yes
11682 10:0 X vs. 49:50 X	-0.4097	-0.5536 to -0.2657	Yes
11683 10:0 X vs. 50:0 X	-3.386	-3.530 to -3.242	Yes
11684 10:0 X vs. 50:1 X	-3.290	-3.434 to -3.146	Yes
11685 10:0 X vs. 50:10 X	-3.284	-3.428 to -3.140	Yes
11686 10:0 X vs. 50:40 X	-0.6627	-0.8066 to -0.5187	Yes
11687 10:0 X vs. 50:50 X	-0.4093	-0.5533 to -0.2654	Yes
11688 10:1 X vs. 10:10 X	-0.002667	-0.1466 to 0.1413	No
11689 10:1 X vs. 10:40 X	0.007667	-0.1363 to 0.1516	No
11690 10:1 X vs. 10:50 X	0.0210	-0.1230 to 0.1650	No
11691 10:1 X vs. 11:0 X	-0.002667	-0.1466 to 0.1413	No
11692 10:1 X vs. 11:1 X	-0.004333	-0.1483 to 0.1396	No
11693 10:1 X vs. 11:10 X	-0.004667	-0.1486 to 0.1393	No
11694 10:1 X vs. 11:40 X	0.003667	-0.1403 to 0.1476	No
11695 10:1 X vs. 11:50 X	0.01567	-0.1283 to 0.1596	No
11696 10:1 X vs. 12:0 X	-0.003333	-0.1473 to 0.1406	No
11697 10:1 X vs. 12:1 X	0.01433	-0.1296 to 0.1583	No
11698 10:1 X vs. 12:10 X	-0.003333	-0.1473 to 0.1406	No
11699 10:1 X vs. 12:40 X	0.01033	-0.1336 to 0.1543	No
11700 10:1 X vs. 12:50 X	0.007000	-0.1370 to 0.1510	No

		A	B	C
	**2way ANOVA** Multiple comparisons	Data Set-A	Data Set-B	Data Set-C
		**Y**	**Y**	**Y**
11701	10:**1 X** vs. 13:**0 X**	0.002667	-0.1413 to 0.1466	No
11702	10:**1 X** vs. 13:**1 X**	0.01533	-0.1286 to 0.1593	No
11703	10:**1 X** vs. 13:**10 X**	-0.006667	-0.1506 to 0.1373	No
11704	10:**1 X** vs. 13:**40 X**	-0.003000	-0.1470 to 0.1410	No
11705	10:**1 X** vs. 13:**50 X**	-0.004000	-0.1480 to 0.1400	No
11706	10:**1 X** vs. 14:**0 X**	-0.006000	-0.1500 to 0.1380	No
11707	10:**1 X** vs. 14:**1 X**	0.002333	-0.1416 to 0.1463	No
11708	10:**1 X** vs. 14:**10 X**	-0.01167	-0.1556 to 0.1323	No
11709	10:**1 X** vs. 14:**40 X**	-0.008333	-0.1523 to 0.1356	No
11710	10:**1 X** vs. 14:**50 X**	-0.01233	-0.1563 to 0.1316	No
11711	10:**1 X** vs. 15:**0 X**	-0.01833	-0.1623 to 0.1256	No
11712	10:**1 X** vs. 15:**1 X**	0.001667	-0.1423 to 0.1456	No
11713	10:**1 X** vs. 15:**10 X**	-0.02567	-0.1696 to 0.1183	No
11714	10:**1 X** vs. 15:**40 X**	-0.008000	-0.1520 to 0.1360	No
11715	10:**1 X** vs. 15:**50 X**	-0.02567	-0.1696 to 0.1183	No
11716	10:**1 X** vs. 16:**0 X**	-0.1107	-0.2546 to 0.03329	No
11717	10:**1 X** vs. 16:**1 X**	-0.001667	-0.1456 to 0.1423	No
11718	10:**1 X** vs. 16:**10 X**	-0.02733	-0.1713 to 0.1166	No
11719	10:**1 X** vs. 16:**40 X**	-0.04667	-0.1906 to 0.09729	No
11720	10:**1 X** vs. 16:**50 X**	-0.08900	-0.2330 to 0.05496	No
11721	10:**1 X** vs. 17:**0 X**	-0.1633	-0.3073 to -0.01937	Yes
11722	10:**1 X** vs. 17:**1 X**	-0.07767	-0.2216 to 0.06629	No
11723	10:**1 X** vs. 17:**10 X**	-0.1343	-0.2783 to 0.009626	No
11724	10:**1 X** vs. 17:**40 X**	-0.05767	-0.2016 to 0.08629	No
11725	10:**1 X** vs. 17:**50 X**	-0.1030	-0.2470 to 0.04096	No
11726	10:**1 X** vs. 18:**0 X**	-0.2120	-0.3560 to -0.06804	Yes
11727	10:**1 X** vs. 18:**1 X**	-0.1203	-0.2643 to 0.02363	No
11728	10:**1 X** vs. 18:**10 X**	-0.2127	-0.3566 to -0.06871	Yes
11729	10:**1 X** vs. 18:**40 X**	-0.0620	-0.2060 to 0.08196	No
11730	10:**1 X** vs. 18:**50 X**	-0.1070	-0.2510 to 0.03696	No
11731	10:**1 X** vs. 19:**0 X**	-0.2470	-0.3910 to -0.1030	Yes
11732	10:**1 X** vs. 19:**1 X**	-0.2350	-0.3790 to -0.09104	Yes
11733	10:**1 X** vs. 19:**10 X**	-0.2327	-0.3766 to -0.08871	Yes
11734	10:**1 X** vs. 19:**40 X**	-0.09067	-0.2346 to 0.05329	No
11735	10:**1 X** vs. 19:**50 X**	-0.1017	-0.2456 to 0.04229	No
11736	10:**1 X** vs. 20:**0 X**	-0.3267	-0.4706 to -0.1827	Yes
11737	10:**1 X** vs. 20:**1 X**	-0.1460	-0.2900 to -0.002040	Yes
11738	10:**1 X** vs. 20:**10 X**	-0.2350	-0.3790 to -0.09104	Yes
11739	10:**1 X** vs. 20:**40 X**	-0.1023	-0.2463 to 0.04163	No
11740	10:**1 X** vs. 20:**50 X**	-0.1463	-0.2903 to -0.002374	Yes
11741	10:**1 X** vs. 21:**0 X**	-0.3500	-0.4940 to -0.2060	Yes
11742	10:**1 X** vs. 21:**1 X**	-0.3030	-0.4470 to -0.1590	Yes
11743	10:**1 X** vs. 21:**10 X**	-0.3000	-0.4440 to -0.1560	Yes
11744	10:**1 X** vs. 21:**40 X**	-0.1753	-0.3193 to -0.03137	Yes
11745	10:**1 X** vs. 21:**50 X**	-0.2657	-0.4096 to -0.1217	Yes

	2way ANOVA<br>Multiple comparisons	A<br>Data Set-A<br>Y	B<br>Data Set-B<br>Y	C<br>Data Set-C<br>Y
11746	10:**1 X** vs. 22:**0 X**	-0.3917	-0.5356 to -0.2477	Yes
11747	10:**1 X** vs. 22:**1 X**	-0.3863	-0.5303 to -0.2424	Yes
11748	10:**1 X** vs. 22:**10 X**	-0.3817	-0.5256 to -0.2377	Yes
11749	10:**1 X** vs. 22:**40 X**	-0.2820	-0.4260 to -0.1380	Yes
11750	10:**1 X** vs. 22:**50 X**	-0.3447	-0.4886 to -0.2007	Yes
11751	10:**1 X** vs. 23:**0 X**	-0.4570	-0.6010 to -0.3130	Yes
11752	10:**1 X** vs. 23:**1 X**	-0.6467	-0.8076 to -0.4857	Yes
11753	10:**1 X** vs. 23:**10 X**	-0.3963	-0.5403 to -0.2524	Yes
11754	10:**1 X** vs. 23:**40 X**	-0.2767	-0.4376 to -0.1157	Yes
11755	10:**1 X** vs. 23:**50 X**	-0.3107	-0.4546 to -0.1667	Yes
11756	10:**1 X** vs. 24:**0 X**	-0.5743	-0.7183 to -0.4304	Yes
11757	10:**1 X** vs. 24:**1 X**	-0.7307	-0.8916 to -0.5697	Yes
11758	10:**1 X** vs. 24:**10 X**	-0.5582	-0.7191 to -0.3972	Yes
11759	10:**1 X** vs. 24:**40 X**	-0.2930	-0.4370 to -0.1490	Yes
11760	10:**1 X** vs. 24:**50 X**	-0.2147	-0.3586 to -0.07071	Yes
11761	10:**1 X** vs. 25:**0 X**	-0.9117	-1.073 to -0.7507	Yes
11762	10:**1 X** vs. 25:**1 X**	-1.101	-1.262 to -0.9402	Yes
11763	10:**1 X** vs. 25:**10 X**	-0.4627	-0.6663 to -0.2591	Yes
11764	10:**1 X** vs. 25:**40 X**	-0.2950	-0.4390 to -0.1510	Yes
11765	10:**1 X** vs. 25:**50 X**	-0.2693	-0.4133 to -0.1254	Yes
11766	10:**1 X** vs. 26:**0 X**	-1.124	-1.285 to -0.9632	Yes
11767	10:**1 X** vs. 26:**1 X**	-1.237	-1.398 to -1.076	Yes
11768	10:**1 X** vs. 26:**10 X**	-0.9237	-1.085 to -0.7627	Yes
11769	10:**1 X** vs. 26:**40 X**	-0.3693	-0.5133 to -0.2254	Yes
11770	10:**1 X** vs. 26:**50 X**	-0.2297	-0.3736 to -0.08571	Yes
11771	10:**1 X** vs. 27:**0 X**	-1.312	-1.456 to -1.168	Yes
11772	10:**1 X** vs. 27:**1 X**	-1.403	-1.564 to -1.242	Yes
11773	10:**1 X** vs. 27:**10 X**	-1.215	-1.376 to -1.054	Yes
11774	10:**1 X** vs. 27:**40 X**	-0.3693	-0.5133 to -0.2254	Yes
11775	10:**1 X** vs. 27:**50 X**	-0.2290	-0.3730 to -0.08504	Yes
11776	10:**1 X** vs. 28:**0 X**	-1.261	-1.422 to -1.100	Yes
11777	10:**1 X** vs. 28:**1 X**	-1.302	-1.446 to -1.158	Yes
11778	10:**1 X** vs. 28:**10 X**	-1.104	-1.265 to -0.9427	Yes
11779	10:**1 X** vs. 28:**40 X**	-0.3817	-0.5256 to -0.2377	Yes
11780	10:**1 X** vs. 28:**50 X**	-0.2770	-0.4210 to -0.1330	Yes
11781	10:**1 X** vs. 29:**0 X**	-1.743	-1.904 to -1.582	Yes
11782	10:**1 X** vs. 29:**1 X**	-1.828	-1.989 to -1.667	Yes
11783	10:**1 X** vs. 29:**10 X**	-1.042	-1.203 to -0.8812	Yes
11784	10:**1 X** vs. 29:**40 X**	-0.4897	-0.6336 to -0.3457	Yes
11785	10:**1 X** vs. 29:**50 X**	-0.2783	-0.4223 to -0.1344	Yes
11786	10:**1 X** vs. 30:**0 X**	-1.457	-1.618 to -1.296	Yes
11787	10:**1 X** vs. 30:**1 X**	-1.779	-1.940 to -1.618	Yes
11788	10:**1 X** vs. 30:**10 X**	-1.117	-1.278 to -0.9557	Yes
11789	10:**1 X** vs. 30:**40 X**	-0.4167	-0.5606 to -0.2727	Yes
11790	10:**1 X** vs. 30:**50 X**	-0.3307	-0.4746 to -0.1867	Yes

2way ANOVA<br>Multiple comparisons	A<br>Data Set-A<br>Y	B<br>Data Set-B<br>Y	C<br>Data Set-C<br>Y
11791 10:1 X vs. 31:0 X	-1.775	-1.936 to -1.614	Yes
11792 10:1 X vs. 31:1 X	-1.766	-1.927 to -1.605	Yes
11793 10:1 X vs. 31:10 X	-1.248	-1.409 to -1.087	Yes
11794 10:1 X vs. 31:40 X	-0.5083	-0.6523 to -0.3644	Yes
11795 10:1 X vs. 31:50 X	-0.3097	-0.4536 to -0.1657	Yes
11796 10:1 X vs. 32:0 X	-2.367	-2.511 to -2.223	Yes
11797 10:1 X vs. 32:1 X	-1.823	-1.984 to -1.662	Yes
11798 10:1 X vs. 32:10 X	-1.679	-1.823 to -1.535	Yes
11799 10:1 X vs. 32:40 X	-0.5047	-0.6486 to -0.3607	Yes
11800 10:1 X vs. 32:50 X	-0.3093	-0.4533 to -0.1654	Yes
11801 10:1 X vs. 33:0 X	-2.370	-2.514 to -2.226	Yes
11802 10:1 X vs. 33:1 X	-2.185	-2.329 to -2.041	Yes
11803 10:1 X vs. 33:10 X	-1.845	-1.989 to -1.701	Yes
11804 10:1 X vs. 33:40 X	-0.5233	-0.6673 to -0.3794	Yes
11805 10:1 X vs. 33:50 X	-0.3243	-0.4683 to -0.1804	Yes
11806 10:1 X vs. 34:0 X	-2.295	-2.439 to -2.151	Yes
11807 10:1 X vs. 34:1 X	-2.302	-2.446 to -2.158	Yes
11808 10:1 X vs. 34:10 X	-1.936	-2.080 to -1.792	Yes
11809 10:1 X vs. 34:40 X	-0.5353	-0.6793 to -0.3914	Yes
11810 10:1 X vs. 34:50 X	-0.4733	-0.6173 to -0.3294	Yes
11811 10:1 X vs. 35:0 X	-2.644	-2.788 to -2.500	Yes
11812 10:1 X vs. 35:1 X	-2.409	-2.553 to -2.265	Yes
11813 10:1 X vs. 35:10 X	-1.897	-2.041 to -1.753	Yes
11814 10:1 X vs. 35:40 X	-0.5443	-0.6883 to -0.4004	Yes
11815 10:1 X vs. 35:50 X	-0.4860	-0.6300 to -0.3420	Yes
11816 10:1 X vs. 36:0 X	-2.850	-3.011 to -2.689	Yes
11817 10:1 X vs. 36:1 X	-2.546	-2.690 to -2.402	Yes
11818 10:1 X vs. 36:10 X	-1.903	-2.047 to -1.759	Yes
11819 10:1 X vs. 36:40 X	-0.5997	-0.7436 to -0.4557	Yes
11820 10:1 X vs. 36:50 X	-0.4493	-0.5933 to -0.3054	Yes
11821 10:1 X vs. 37:0 X	-3.234	-3.378 to -3.090	Yes
11822 10:1 X vs. 37:1 X	-2.616	-2.760 to -2.472	Yes
11823 10:1 X vs. 37:10 X	-1.905	-2.049 to -1.761	Yes
11824 10:1 X vs. 37:40 X	-0.6370	-0.7810 to -0.4930	Yes
11825 10:1 X vs. 37:50 X	-0.4580	-0.6020 to -0.3140	Yes
11826 10:1 X vs. 38:0 X	-3.179	-3.323 to -3.035	Yes
11827 10:1 X vs. 38:1 X	-2.735	-2.879 to -2.591	Yes
11828 10:1 X vs. 38:10 X	-2.180	-2.324 to -2.036	Yes
11829 10:1 X vs. 38:40 X	-0.6413	-0.7853 to -0.4974	Yes
11830 10:1 X vs. 38:50 X	-0.4620	-0.6060 to -0.3180	Yes
11831 10:1 X vs. 39:0 X	-3.410	-3.554 to -3.266	Yes
11832 10:1 X vs. 39:1 X	-3.301	-3.445 to -3.157	Yes
11833 10:1 X vs. 39:10 X	-2.325	-2.469 to -2.181	Yes
11834 10:1 X vs. 39:40 X	-0.6473	-0.7913 to -0.5034	Yes
11835 10:1 X vs. 39:50 X	-0.4930	-0.6370 to -0.3490	Yes

	2way ANOVA<br>Multiple comparisons	A<br>Data Set-A<br>Y	B<br>Data Set-B<br>Y	C<br>Data Set-C<br>Y
11836	10:**1 X** vs. 40:**0 X**	-3.451	-3.595 to -3.307	Yes
11837	10:**1 X** vs. 40:**1 X**	-3.377	-3.521 to -3.233	Yes
11838	10:**1 X** vs. 40:**10 X**	-2.581	-2.725 to -2.437	Yes
11839	10:**1 X** vs. 40:**40 X**	-0.6520	-0.7960 to -0.5080	Yes
11840	10:**1 X** vs. 40:**50 X**	-0.4993	-0.6433 to -0.3554	Yes
11841	10:**1 X** vs. 41:**0 X**	-3.492	-3.636 to -3.348	Yes
11842	10:**1 X** vs. 41:**1 X**	-3.491	-3.635 to -3.347	Yes
11843	10:**1 X** vs. 41:**10 X**	-2.635	-2.779 to -2.491	Yes
11844	10:**1 X** vs. 41:**40 X**	-0.5993	-0.7433 to -0.4554	Yes
11845	10:**1 X** vs. 41:**50 X**	-0.5120	-0.6560 to -0.3680	Yes
11846	10:**1 X** vs. 42:**0 X**	-3.633	-3.777 to -3.489	Yes
11847	10:**1 X** vs. 42:**1 X**	-3.653	-3.797 to -3.509	Yes
11848	10:**1 X** vs. 42:**10 X**	-2.635	-2.779 to -2.491	Yes
11849	10:**1 X** vs. 42:**40 X**	-0.6120	-0.7560 to -0.4680	Yes
11850	10:**1 X** vs. 42:**50 X**	-0.5153	-0.6593 to -0.3714	Yes
11851	10:**1 X** vs. 43:**0 X**	-3.723	-3.867 to -3.579	Yes
11852	10:**1 X** vs. 43:**1 X**	-3.638	-3.782 to -3.494	Yes
11853	10:**1 X** vs. 43:**10 X**	-3.353	-3.497 to -3.209	Yes
11854	10:**1 X** vs. 43:**40 X**	-0.6103	-0.7543 to -0.4664	Yes
11855	10:**1 X** vs. 43:**50 X**	-0.5157	-0.6596 to -0.3717	Yes
11856	10:**1 X** vs. 44:**0 X**	-3.747	-3.891 to -3.603	Yes
11857	10:**1 X** vs. 44:**1 X**	-3.637	-3.781 to -3.493	Yes
11858	10:**1 X** vs. 44:**10 X**	-3.417	-3.561 to -3.273	Yes
11859	10:**1 X** vs. 44:**40 X**	-0.6123	-0.7563 to -0.4684	Yes
11860	10:**1 X** vs. 44:**50 X**	-0.4480	-0.5920 to -0.3040	Yes
11861	10:**1 X** vs. 45:**0 X**	-3.612	-3.756 to -3.468	Yes
11862	10:**1 X** vs. 45:**1 X**	-3.414	-3.558 to -3.270	Yes
11863	10:**1 X** vs. 45:**10 X**	-3.296	-3.440 to -3.152	Yes
11864	10:**1 X** vs. 45:**40 X**	-0.6137	-0.7576 to -0.4697	Yes
11865	10:**1 X** vs. 45:**50 X**	-0.4450	-0.5890 to -0.3010	Yes
11866	10:**1 X** vs. 46:**0 X**	-3.416	-3.560 to -3.272	Yes
11867	10:**1 X** vs. 46:**1 X**	-3.383	-3.527 to -3.239	Yes
11868	10:**1 X** vs. 46:**10 X**	-3.291	-3.435 to -3.147	Yes
11869	10:**1 X** vs. 46:**40 X**	-0.6273	-0.7713 to -0.4834	Yes
11870	10:**1 X** vs. 46:**50 X**	-0.4380	-0.5820 to -0.2940	Yes
11871	10:**1 X** vs. 47:**0 X**	-3.392	-3.536 to -3.248	Yes
11872	10:**1 X** vs. 47:**1 X**	-3.296	-3.440 to -3.152	Yes
11873	10:**1 X** vs. 47:**10 X**	-3.277	-3.421 to -3.133	Yes
11874	10:**1 X** vs. 47:**40 X**	-0.6373	-0.7813 to -0.4934	Yes
11875	10:**1 X** vs. 47:**50 X**	-0.3960	-0.5400 to -0.2520	Yes
11876	10:**1 X** vs. 48:**0 X**	-3.379	-3.540 to -3.218	Yes
11877	10:**1 X** vs. 48:**1 X**	-3.283	-3.427 to -3.139	Yes
11878	10:**1 X** vs. 48:**10 X**	-3.277	-3.421 to -3.133	Yes
11879	10:**1 X** vs. 48:**40 X**	-0.6437	-0.7876 to -0.4997	Yes
11880	10:**1 X** vs. 48:**50 X**	-0.4033	-0.5473 to -0.2594	Yes

2way ANOVA<br>Multiple comparisons	A<br>Data Set-A<br>Y	B<br>Data Set-B<br>Y	C<br>Data Set-C<br>Y
11881 10:1 X vs. 49:0 X	-3.379	-3.523 to -3.235	Yes
11882 10:1 X vs. 49:1 X	-3.283	-3.427 to -3.139	Yes
11883 10:1 X vs. 49:10 X	-3.277	-3.421 to -3.133	Yes
11884 10:1 X vs. 49:40 X	-0.6480	-0.7920 to -0.5040	Yes
11885 10:1 X vs. 49:50 X	-0.4027	-0.5466 to -0.2587	Yes
11886 10:1 X vs. 50:0 X	-3.379	-3.523 to -3.235	Yes
11887 10:1 X vs. 50:1 X	-3.283	-3.427 to -3.139	Yes
11888 10:1 X vs. 50:10 X	-3.277	-3.421 to -3.133	Yes
11889 10:1 X vs. 50:40 X	-0.6557	-0.7996 to -0.5117	Yes
11890 10:1 X vs. 50:50 X	-0.4023	-0.5463 to -0.2584	Yes
11891 10:10 X vs. 10:40 X	0.01033	-0.1336 to 0.1543	No
11892 10:10 X vs. 10:50 X	0.02367	-0.1203 to 0.1676	No
11893 10:10 X vs. 11:0 X	0.0	-0.1440 to 0.1440	No
11894 10:10 X vs. 11:1 X	-0.001667	-0.1456 to 0.1423	No
11895 10:10 X vs. 11:10 X	-0.002000	-0.1460 to 0.1420	No
11896 10:10 X vs. 11:40 X	0.006333	-0.1376 to 0.1503	No
11897 10:10 X vs. 11:50 X	0.01833	-0.1256 to 0.1623	No
11898 10:10 X vs. 12:0 X	-0.0006667	-0.1446 to 0.1433	No
11899 10:10 X vs. 12:1 X	0.0170	-0.1270 to 0.1610	No
11900 10:10 X vs. 12:10 X	-0.0006667	-0.1446 to 0.1433	No
11901 10:10 X vs. 12:40 X	0.0130	-0.1310 to 0.1570	No
11902 10:10 X vs. 12:50 X	0.009667	-0.1343 to 0.1536	No
11903 10:10 X vs. 13:0 X	0.005333	-0.1386 to 0.1493	No
11904 10:10 X vs. 13:1 X	0.0180	-0.1260 to 0.1620	No
11905 10:10 X vs. 13:10 X	-0.004000	-0.1480 to 0.1400	No
11906 10:10 X vs. 13:40 X	-0.0003333	-0.1443 to 0.1436	No
11907 10:10 X vs. 13:50 X	-0.001333	-0.1453 to 0.1426	No
11908 10:10 X vs. 14:0 X	-0.003333	-0.1473 to 0.1406	No
11909 10:10 X vs. 14:1 X	0.005000	-0.1390 to 0.1490	No
11910 10:10 X vs. 14:10 X	-0.0090	-0.1530 to 0.1350	No
11911 10:10 X vs. 14:40 X	-0.005667	-0.1496 to 0.1383	No
11912 10:10 X vs. 14:50 X	-0.009667	-0.1536 to 0.1343	No
11913 10:10 X vs. 15:0 X	-0.01567	-0.1596 to 0.1283	No
11914 10:10 X vs. 15:1 X	0.004333	-0.1396 to 0.1483	No
11915 10:10 X vs. 15:10 X	-0.0230	-0.1670 to 0.1210	No
11916 10:10 X vs. 15:40 X	-0.005333	-0.1493 to 0.1386	No
11917 10:10 X vs. 15:50 X	-0.0230	-0.1670 to 0.1210	No
11918 10:10 X vs. 16:0 X	-0.1080	-0.2520 to 0.03596	No
11919 10:10 X vs. 16:1 X	0.001000	-0.1430 to 0.1450	No
11920 10:10 X vs. 16:10 X	-0.02467	-0.1686 to 0.1193	No
11921 10:10 X vs. 16:40 X	-0.0440	-0.1880 to 0.09996	No
11922 10:10 X vs. 16:50 X	-0.08633	-0.2303 to 0.05763	No
11923 10:10 X vs. 17:0 X	-0.1607	-0.3046 to -0.01671	Yes
11924 10:10 X vs. 17:1 X	-0.0750	-0.2190 to 0.06896	No
11925 10:10 X vs. 17:10 X	-0.1317	-0.2756 to 0.01229	No

2way ANOVA Multiple comparisons		A Data Set-A Y	B Data Set-B Y	C Data Set-C Y
11926	10:**10 X** vs. 17:**40 X**	-0.0550	-0.1990 to 0.08896	No
11927	10:**10 X** vs. 17:**50 X**	-0.1003	-0.2443 to 0.04363	No
11928	10:**10 X** vs. 18:**0 X**	-0.2093	-0.3533 to -0.06537	Yes
11929	10:**10 X** vs. 18:**1 X**	-0.1177	-0.2616 to 0.02629	No
11930	10:**10 X** vs. 18:**10 X**	-0.2100	-0.3540 to -0.06604	Yes
11931	10:**10 X** vs. 18:**40 X**	-0.05933	-0.2033 to 0.08463	No
11932	10:**10 X** vs. 18:**50 X**	-0.1043	-0.2483 to 0.03963	No
11933	10:**10 X** vs. 19:**0 X**	-0.2443	-0.3883 to -0.1004	Yes
11934	10:**10 X** vs. 19:**1 X**	-0.2323	-0.3763 to -0.08837	Yes
11935	10:**10 X** vs. 19:**10 X**	-0.2300	-0.3740 to -0.08604	Yes
11936	10:**10 X** vs. 19:**40 X**	-0.0880	-0.2320 to 0.05596	No
11937	10:**10 X** vs. 19:**50 X**	-0.0990	-0.2430 to 0.04496	No
11938	10:**10 X** vs. 20:**0 X**	-0.3240	-0.4680 to -0.1800	Yes
11939	10:**10 X** vs. 20:**1 X**	-0.1433	-0.2873 to 0.0006265	No
11940	10:**10 X** vs. 20:**10 X**	-0.2323	-0.3763 to -0.08837	Yes
11941	10:**10 X** vs. 20:**40 X**	-0.09967	-0.2436 to 0.04429	No
11942	10:**10 X** vs. 20:**50 X**	-0.1437	-0.2876 to 0.0002932	No
11943	10:**10 X** vs. 21:**0 X**	-0.3473	-0.4913 to -0.2034	Yes
11944	10:**10 X** vs. 21:**1 X**	-0.3003	-0.4443 to -0.1564	Yes
11945	10:**10 X** vs. 21:**10 X**	-0.2973	-0.4413 to -0.1534	Yes
11946	10:**10 X** vs. 21:**40 X**	-0.1727	-0.3166 to -0.02871	Yes
11947	10:**10 X** vs. 21:**50 X**	-0.2630	-0.4070 to -0.1190	Yes
11948	10:**10 X** vs. 22:**0 X**	-0.3890	-0.5330 to -0.2450	Yes
11949	10:**10 X** vs. 22:**1 X**	-0.3837	-0.5276 to -0.2397	Yes
11950	10:**10 X** vs. 22:**10 X**	-0.3790	-0.5230 to -0.2350	Yes
11951	10:**10 X** vs. 22:**40 X**	-0.2793	-0.4233 to -0.1354	Yes
11952	10:**10 X** vs. 22:**50 X**	-0.3420	-0.4860 to -0.1980	Yes
11953	10:**10 X** vs. 23:**0 X**	-0.4543	-0.5983 to -0.3104	Yes
11954	10:**10 X** vs. 23:**1 X**	-0.6440	-0.8050 to -0.4830	Yes
11955	10:**10 X** vs. 23:**10 X**	-0.3937	-0.5376 to -0.2497	Yes
11956	10:**10 X** vs. 23:**40 X**	-0.2740	-0.4350 to -0.1130	Yes
11957	10:**10 X** vs. 23:**50 X**	-0.3080	-0.4520 to -0.1640	Yes
11958	10:**10 X** vs. 24:**0 X**	-0.5717	-0.7156 to -0.4277	Yes
11959	10:**10 X** vs. 24:**1 X**	-0.7280	-0.8890 to -0.5670	Yes
11960	10:**10 X** vs. 24:**10 X**	-0.5555	-0.7165 to -0.3945	Yes
11961	10:**10 X** vs. 24:**40 X**	-0.2903	-0.4343 to -0.1464	Yes
11962	10:**10 X** vs. 24:**50 X**	-0.2120	-0.3560 to -0.06804	Yes
11963	10:**10 X** vs. 25:**0 X**	-0.9090	-1.070 to -0.7480	Yes
11964	10:**10 X** vs. 25:**1 X**	-1.099	-1.259 to -0.9375	Yes
11965	10:**10 X** vs. 25:**10 X**	-0.4600	-0.6636 to -0.2564	Yes
11966	10:**10 X** vs. 25:**40 X**	-0.2923	-0.4363 to -0.1484	Yes
11967	10:**10 X** vs. 25:**50 X**	-0.2667	-0.4106 to -0.1227	Yes
11968	10:**10 X** vs. 26:**0 X**	-1.122	-1.282 to -0.9605	Yes
11969	10:**10 X** vs. 26:**1 X**	-1.235	-1.395 to -1.074	Yes
11970	10:**10 X** vs. 26:**10 X**	-0.9210	-1.082 to -0.7600	Yes

2way ANOVA<br>Multiple comparisons	A<br>Data Set-A<br>Y	B<br>Data Set-B<br>Y	C<br>Data Set-C<br>Y
11971 10:**10 X** vs. 26:**40 X**	-0.3667	-0.5106 to -0.2227	Yes
11972 10:**10 X** vs. 26:**50 X**	-0.2270	-0.3710 to -0.08304	Yes
11973 10:**10 X** vs. 27:**0 X**	-1.309	-1.453 to -1.165	Yes
11974 10:**10 X** vs. 27:**1 X**	-1.400	-1.561 to -1.239	Yes
11975 10:**10 X** vs. 27:**10 X**	-1.212	-1.373 to -1.051	Yes
11976 10:**10 X** vs. 27:**40 X**	-0.3667	-0.5106 to -0.2227	Yes
11977 10:**10 X** vs. 27:**50 X**	-0.2263	-0.3703 to -0.08237	Yes
11978 10:**10 X** vs. 28:**0 X**	-1.259	-1.419 to -1.098	Yes
11979 10:**10 X** vs. 28:**1 X**	-1.299	-1.443 to -1.155	Yes
11980 10:**10 X** vs. 28:**10 X**	-1.101	-1.262 to -0.9400	Yes
11981 10:**10 X** vs. 28:**40 X**	-0.3790	-0.5230 to -0.2350	Yes
11982 10:**10 X** vs. 28:**50 X**	-0.2743	-0.4183 to -0.1304	Yes
11983 10:**10 X** vs. 29:**0 X**	-1.740	-1.901 to -1.579	Yes
11984 10:**10 X** vs. 29:**1 X**	-1.825	-1.986 to -1.664	Yes
11985 10:**10 X** vs. 29:**10 X**	-1.040	-1.200 to -0.8785	Yes
11986 10:**10 X** vs. 29:**40 X**	-0.4870	-0.6310 to -0.3430	Yes
11987 10:**10 X** vs. 29:**50 X**	-0.2757	-0.4196 to -0.1317	Yes
11988 10:**10 X** vs. 30:**0 X**	-1.455	-1.615 to -1.294	Yes
11989 10:**10 X** vs. 30:**1 X**	-1.776	-1.937 to -1.615	Yes
11990 10:**10 X** vs. 30:**10 X**	-1.114	-1.275 to -0.9530	Yes
11991 10:**10 X** vs. 30:**40 X**	-0.4140	-0.5580 to -0.2700	Yes
11992 10:**10 X** vs. 30:**50 X**	-0.3280	-0.4720 to -0.1840	Yes
11993 10:**10 X** vs. 31:**0 X**	-1.773	-1.933 to -1.612	Yes
11994 10:**10 X** vs. 31:**1 X**	-1.763	-1.924 to -1.602	Yes
11995 10:**10 X** vs. 31:**10 X**	-1.246	-1.406 to -1.085	Yes
11996 10:**10 X** vs. 31:**40 X**	-0.5057	-0.6496 to -0.3617	Yes
11997 10:**10 X** vs. 31:**50 X**	-0.3070	-0.4510 to -0.1630	Yes
11998 10:**10 X** vs. 32:**0 X**	-2.364	-2.508 to -2.220	Yes
11999 10:**10 X** vs. 32:**1 X**	-1.820	-1.981 to -1.659	Yes
12000 10:**10 X** vs. 32:**10 X**	-1.676	-1.820 to -1.532	Yes
12001 10:**10 X** vs. 32:**40 X**	-0.5020	-0.6460 to -0.3580	Yes
12002 10:**10 X** vs. 32:**50 X**	-0.3067	-0.4506 to -0.1627	Yes
12003 10:**10 X** vs. 33:**0 X**	-2.368	-2.512 to -2.224	Yes
12004 10:**10 X** vs. 33:**1 X**	-2.182	-2.326 to -2.038	Yes
12005 10:**10 X** vs. 33:**10 X**	-1.843	-1.987 to -1.699	Yes
12006 10:**10 X** vs. 33:**40 X**	-0.5207	-0.6646 to -0.3767	Yes
12007 10:**10 X** vs. 33:**50 X**	-0.3217	-0.4656 to -0.1777	Yes
12008 10:**10 X** vs. 34:**0 X**	-2.292	-2.436 to -2.148	Yes
12009 10:**10 X** vs. 34:**1 X**	-2.300	-2.444 to -2.156	Yes
12010 10:**10 X** vs. 34:**10 X**	-1.933	-2.077 to -1.789	Yes
12011 10:**10 X** vs. 34:**40 X**	-0.5327	-0.6766 to -0.3887	Yes
12012 10:**10 X** vs. 34:**50 X**	-0.4707	-0.6146 to -0.3267	Yes
12013 10:**10 X** vs. 35:**0 X**	-2.641	-2.785 to -2.497	Yes
12014 10:**10 X** vs. 35:**1 X**	-2.407	-2.551 to -2.263	Yes
12015 10:**10 X** vs. 35:**10 X**	-1.895	-2.039 to -1.751	Yes

	2way ANOVA Multiple comparisons	A Data Set-A Y	B Data Set-B Y	C Data Set-C Y
12016	10:**10 X** vs. 35:**40 X**	-0.5417	-0.6856 to -0.3977	Yes
12017	10:**10 X** vs. 35:**50 X**	-0.4833	-0.6273 to -0.3394	Yes
12018	10:**10 X** vs. 36:**0 X**	-2.848	-3.008 to -2.687	Yes
12019	10:**10 X** vs. 36:**1 X**	-2.543	-2.687 to -2.399	Yes
12020	10:**10 X** vs. 36:**10 X**	-1.900	-2.044 to -1.756	Yes
12021	10:**10 X** vs. 36:**40 X**	-0.5970	-0.7410 to -0.4530	Yes
12022	10:**10 X** vs. 36:**50 X**	-0.4467	-0.5906 to -0.3027	Yes
12023	10:**10 X** vs. 37:**0 X**	-3.231	-3.375 to -3.087	Yes
12024	10:**10 X** vs. 37:**1 X**	-2.613	-2.757 to -2.469	Yes
12025	10:**10 X** vs. 37:**10 X**	-1.902	-2.046 to -1.758	Yes
12026	10:**10 X** vs. 37:**40 X**	-0.6343	-0.7783 to -0.4904	Yes
12027	10:**10 X** vs. 37:**50 X**	-0.4553	-0.5993 to -0.3114	Yes
12028	10:**10 X** vs. 38:**0 X**	-3.176	-3.320 to -3.032	Yes
12029	10:**10 X** vs. 38:**1 X**	-2.732	-2.876 to -2.588	Yes
12030	10:**10 X** vs. 38:**10 X**	-2.177	-2.321 to -2.033	Yes
12031	10:**10 X** vs. 38:**40 X**	-0.6387	-0.7826 to -0.4947	Yes
12032	10:**10 X** vs. 38:**50 X**	-0.4593	-0.6033 to -0.3154	Yes
12033	10:**10 X** vs. 39:**0 X**	-3.407	-3.551 to -3.263	Yes
12034	10:**10 X** vs. 39:**1 X**	-3.299	-3.443 to -3.155	Yes
12035	10:**10 X** vs. 39:**10 X**	-2.322	-2.466 to -2.178	Yes
12036	10:**10 X** vs. 39:**40 X**	-0.6447	-0.7886 to -0.5007	Yes
12037	10:**10 X** vs. 39:**50 X**	-0.4903	-0.6343 to -0.3464	Yes
12038	10:**10 X** vs. 40:**0 X**	-3.448	-3.592 to -3.304	Yes
12039	10:**10 X** vs. 40:**1 X**	-3.374	-3.518 to -3.230	Yes
12040	10:**10 X** vs. 40:**10 X**	-2.579	-2.723 to -2.435	Yes
12041	10:**10 X** vs. 40:**40 X**	-0.6493	-0.7933 to -0.5054	Yes
12042	10:**10 X** vs. 40:**50 X**	-0.4967	-0.6406 to -0.3527	Yes
12043	10:**10 X** vs. 41:**0 X**	-3.489	-3.633 to -3.345	Yes
12044	10:**10 X** vs. 41:**1 X**	-3.488	-3.632 to -3.344	Yes
12045	10:**10 X** vs. 41:**10 X**	-2.632	-2.776 to -2.488	Yes
12046	10:**10 X** vs. 41:**40 X**	-0.5967	-0.7406 to -0.4527	Yes
12047	10:**10 X** vs. 41:**50 X**	-0.5093	-0.6533 to -0.3654	Yes
12048	10:**10 X** vs. 42:**0 X**	-3.631	-3.775 to -3.487	Yes
12049	10:**10 X** vs. 42:**1 X**	-3.650	-3.794 to -3.506	Yes
12050	10:**10 X** vs. 42:**10 X**	-2.633	-2.777 to -2.489	Yes
12051	10:**10 X** vs. 42:**40 X**	-0.6093	-0.7533 to -0.4654	Yes
12052	10:**10 X** vs. 42:**50 X**	-0.5127	-0.6566 to -0.3687	Yes
12053	10:**10 X** vs. 43:**0 X**	-3.721	-3.865 to -3.577	Yes
12054	10:**10 X** vs. 43:**1 X**	-3.635	-3.779 to -3.491	Yes
12055	10:**10 X** vs. 43:**10 X**	-3.351	-3.495 to -3.207	Yes
12056	10:**10 X** vs. 43:**40 X**	-0.6077	-0.7516 to -0.4637	Yes
12057	10:**10 X** vs. 43:**50 X**	-0.5130	-0.6570 to -0.3690	Yes
12058	10:**10 X** vs. 44:**0 X**	-3.745	-3.889 to -3.601	Yes
12059	10:**10 X** vs. 44:**1 X**	-3.634	-3.778 to -3.490	Yes
12060	10:**10 X** vs. 44:**10 X**	-3.414	-3.558 to -3.270	Yes

	2way ANOVA<br>Multiple comparisons	A<br>Data Set-A<br>Y	B<br>Data Set-B<br>Y	C<br>Data Set-C<br>Y
12061	10:10 X vs. 44:40 X	-0.6097	-0.7536 to -0.4657	Yes
12062	10:10 X vs. 44:50 X	-0.4453	-0.5893 to -0.3014	Yes
12063	10:10 X vs. 45:0 X	-3.609	-3.753 to -3.465	Yes
12064	10:10 X vs. 45:1 X	-3.411	-3.555 to -3.267	Yes
12065	10:10 X vs. 45:10 X	-3.294	-3.438 to -3.150	Yes
12066	10:10 X vs. 45:40 X	-0.6110	-0.7550 to -0.4670	Yes
12067	10:10 X vs. 45:50 X	-0.4423	-0.5863 to -0.2984	Yes
12068	10:10 X vs. 46:0 X	-3.414	-3.558 to -3.270	Yes
12069	10:10 X vs. 46:1 X	-3.381	-3.525 to -3.237	Yes
12070	10:10 X vs. 46:10 X	-3.289	-3.433 to -3.145	Yes
12071	10:10 X vs. 46:40 X	-0.6247	-0.7686 to -0.4807	Yes
12072	10:10 X vs. 46:50 X	-0.4353	-0.5793 to -0.2914	Yes
12073	10:10 X vs. 47:0 X	-3.389	-3.533 to -3.245	Yes
12074	10:10 X vs. 47:1 X	-3.293	-3.437 to -3.149	Yes
12075	10:10 X vs. 47:10 X	-3.275	-3.419 to -3.131	Yes
12076	10:10 X vs. 47:40 X	-0.6347	-0.7786 to -0.4907	Yes
12077	10:10 X vs. 47:50 X	-0.3933	-0.5373 to -0.2494	Yes
12078	10:10 X vs. 48:0 X	-3.377	-3.537 to -3.216	Yes
12079	10:10 X vs. 48:1 X	-3.280	-3.424 to -3.136	Yes
12080	10:10 X vs. 48:10 X	-3.275	-3.419 to -3.131	Yes
12081	10:10 X vs. 48:40 X	-0.6410	-0.7850 to -0.4970	Yes
12082	10:10 X vs. 48:50 X	-0.4007	-0.5446 to -0.2567	Yes
12083	10:10 X vs. 49:0 X	-3.377	-3.521 to -3.233	Yes
12084	10:10 X vs. 49:1 X	-3.280	-3.424 to -3.136	Yes
12085	10:10 X vs. 49:10 X	-3.275	-3.419 to -3.131	Yes
12086	10:10 X vs. 49:40 X	-0.6453	-0.7893 to -0.5014	Yes
12087	10:10 X vs. 49:50 X	-0.4000	-0.5440 to -0.2560	Yes
12088	10:10 X vs. 50:0 X	-3.377	-3.521 to -3.233	Yes
12089	10:10 X vs. 50:1 X	-3.280	-3.424 to -3.136	Yes
12090	10:10 X vs. 50:10 X	-3.275	-3.419 to -3.131	Yes
12091	10:10 X vs. 50:40 X	-0.6530	-0.7970 to -0.5090	Yes
12092	10:10 X vs. 50:50 X	-0.3997	-0.5436 to -0.2557	Yes
12093	10:40 X vs. 10:50 X	0.01333	-0.1306 to 0.1573	No
12094	10:40 X vs. 11:0 X	-0.01033	-0.1543 to 0.1336	No
12095	10:40 X vs. 11:1 X	-0.0120	-0.1560 to 0.1320	No
12096	10:40 X vs. 11:10 X	-0.01233	-0.1563 to 0.1316	No
12097	10:40 X vs. 11:40 X	-0.004000	-0.1480 to 0.1400	No
12098	10:40 X vs. 11:50 X	0.0080	-0.1360 to 0.1520	No
12099	10:40 X vs. 12:0 X	-0.0110	-0.1550 to 0.1330	No
12100	10:40 X vs. 12:1 X	0.006667	-0.1373 to 0.1506	No
12101	10:40 X vs. 12:10 X	-0.0110	-0.1550 to 0.1330	No
12102	10:40 X vs. 12:40 X	0.002667	-0.1413 to 0.1466	No
12103	10:40 X vs. 12:50 X	-0.0006667	-0.1446 to 0.1433	No
12104	10:40 X vs. 13:0 X	-0.005000	-0.1490 to 0.1390	No
12105	10:40 X vs. 13:1 X	0.007667	-0.1363 to 0.1516	No

	2way ANOVA<br>Multiple comparisons	A<br>Data Set-A<br>Y	B<br>Data Set-B<br>Y	C<br>Data Set-C<br>Y
12106	10:**40 X** vs. 13:**10 X**	-0.01433	-0.1583 to 0.1296	No
12107	10:**40 X** vs. 13:**40 X**	-0.01067	-0.1546 to 0.1333	No
12108	10:**40 X** vs. 13:**50 X**	-0.01167	-0.1556 to 0.1323	No
12109	10:**40 X** vs. 14:**0 X**	-0.01367	-0.1576 to 0.1303	No
12110	10:**40 X** vs. 14:**1 X**	-0.005333	-0.1493 to 0.1386	No
12111	10:**40 X** vs. 14:**10 X**	-0.01933	-0.1633 to 0.1246	No
12112	10:**40 X** vs. 14:**40 X**	-0.0160	-0.1600 to 0.1280	No
12113	10:**40 X** vs. 14:**50 X**	-0.0200	-0.1640 to 0.1240	No
12114	10:**40 X** vs. 15:**0 X**	-0.0260	-0.1700 to 0.1180	No
12115	10:**40 X** vs. 15:**1 X**	-0.006000	-0.1500 to 0.1380	No
12116	10:**40 X** vs. 15:**10 X**	-0.03333	-0.1773 to 0.1106	No
12117	10:**40 X** vs. 15:**40 X**	-0.01567	-0.1596 to 0.1283	No
12118	10:**40 X** vs. 15:**50 X**	-0.03333	-0.1773 to 0.1106	No
12119	10:**40 X** vs. 16:**0 X**	-0.1183	-0.2623 to 0.02563	No
12120	10:**40 X** vs. 16:**1 X**	-0.009333	-0.1533 to 0.1346	No
12121	10:**40 X** vs. 16:**10 X**	-0.0350	-0.1790 to 0.1090	No
12122	10:**40 X** vs. 16:**40 X**	-0.05433	-0.1983 to 0.08963	No
12123	10:**40 X** vs. 16:**50 X**	-0.09667	-0.2406 to 0.04729	No
12124	10:**40 X** vs. 17:**0 X**	-0.1710	-0.3150 to -0.02704	Yes
12125	10:**40 X** vs. 17:**1 X**	-0.08533	-0.2293 to 0.05863	No
12126	10:**40 X** vs. 17:**10 X**	-0.1420	-0.2860 to 0.001960	No
12127	10:**40 X** vs. 17:**40 X**	-0.06533	-0.2093 to 0.07863	No
12128	10:**40 X** vs. 17:**50 X**	-0.1107	-0.2546 to 0.03329	No
12129	10:**40 X** vs. 18:**0 X**	-0.2197	-0.3636 to -0.07571	Yes
12130	10:**40 X** vs. 18:**1 X**	-0.1280	-0.2720 to 0.01596	No
12131	10:**40 X** vs. 18:**10 X**	-0.2203	-0.3643 to -0.07637	Yes
12132	10:**40 X** vs. 18:**40 X**	-0.06967	-0.2136 to 0.07429	No
12133	10:**40 X** vs. 18:**50 X**	-0.1147	-0.2586 to 0.02929	No
12134	10:**40 X** vs. 19:**0 X**	-0.2547	-0.3986 to -0.1107	Yes
12135	10:**40 X** vs. 19:**1 X**	-0.2427	-0.3866 to -0.09871	Yes
12136	10:**40 X** vs. 19:**10 X**	-0.2403	-0.3843 to -0.09637	Yes
12137	10:**40 X** vs. 19:**40 X**	-0.09833	-0.2423 to 0.04563	No
12138	10:**40 X** vs. 19:**50 X**	-0.1093	-0.2533 to 0.03463	No
12139	10:**40 X** vs. 20:**0 X**	-0.3343	-0.4783 to -0.1904	Yes
12140	10:**40 X** vs. 20:**1 X**	-0.1537	-0.2976 to -0.009707	Yes
12141	10:**40 X** vs. 20:**10 X**	-0.2427	-0.3866 to -0.09871	Yes
12142	10:**40 X** vs. 20:**40 X**	-0.1100	-0.2540 to 0.03396	No
12143	10:**40 X** vs. 20:**50 X**	-0.1540	-0.2980 to -0.01004	Yes
12144	10:**40 X** vs. 21:**0 X**	-0.3577	-0.5016 to -0.2137	Yes
12145	10:**40 X** vs. 21:**1 X**	-0.3107	-0.4546 to -0.1667	Yes
12146	10:**40 X** vs. 21:**10 X**	-0.3077	-0.4516 to -0.1637	Yes
12147	10:**40 X** vs. 21:**40 X**	-0.1830	-0.3270 to -0.03904	Yes
12148	10:**40 X** vs. 21:**50 X**	-0.2733	-0.4173 to -0.1294	Yes
12149	10:**40 X** vs. 22:**0 X**	-0.3993	-0.5433 to -0.2554	Yes
12150	10:**40 X** vs. 22:**1 X**	-0.3940	-0.5380 to -0.2500	Yes

	2way ANOVA<br>Multiple comparisons	A<br>Data Set-A<br>Y	B<br>Data Set-B<br>Y	C<br>Data Set-C<br>Y
12151	10:40 X vs. 22:10 X	-0.3893	-0.5333 to -0.2454	Yes
12152	10:40 X vs. 22:40 X	-0.2897	-0.4336 to -0.1457	Yes
12153	10:40 X vs. 22:50 X	-0.3523	-0.4963 to -0.2084	Yes
12154	10:40 X vs. 23:0 X	-0.4647	-0.6086 to -0.3207	Yes
12155	10:40 X vs. 23:1 X	-0.6543	-0.8153 to -0.4934	Yes
12156	10:40 X vs. 23:10 X	-0.4040	-0.5480 to -0.2600	Yes
12157	10:40 X vs. 23:40 X	-0.2843	-0.4453 to -0.1234	Yes
12158	10:40 X vs. 23:50 X	-0.3183	-0.4623 to -0.1744	Yes
12159	10:40 X vs. 24:0 X	-0.5820	-0.7260 to -0.4380	Yes
12160	10:40 X vs. 24:1 X	-0.7383	-0.8993 to -0.5774	Yes
12161	10:40 X vs. 24:10 X	-0.5658	-0.7268 to -0.4049	Yes
12162	10:40 X vs. 24:40 X	-0.3007	-0.4446 to -0.1567	Yes
12163	10:40 X vs. 24:50 X	-0.2223	-0.3663 to -0.07837	Yes
12164	10:40 X vs. 25:0 X	-0.9193	-1.080 to -0.7584	Yes
12165	10:40 X vs. 25:1 X	-1.109	-1.270 to -0.9479	Yes
12166	10:40 X vs. 25:10 X	-0.4703	-0.6739 to -0.2667	Yes
12167	10:40 X vs. 25:40 X	-0.3027	-0.4466 to -0.1587	Yes
12168	10:40 X vs. 25:50 X	-0.2770	-0.4210 to -0.1330	Yes
12169	10:40 X vs. 26:0 X	-1.132	-1.293 to -0.9709	Yes
12170	10:40 X vs. 26:1 X	-1.245	-1.406 to -1.084	Yes
12171	10:40 X vs. 26:10 X	-0.9313	-1.092 to -0.7704	Yes
12172	10:40 X vs. 26:40 X	-0.3770	-0.5210 to -0.2330	Yes
12173	10:40 X vs. 26:50 X	-0.2373	-0.3813 to -0.09337	Yes
12174	10:40 X vs. 27:0 X	-1.320	-1.464 to -1.176	Yes
12175	10:40 X vs. 27:1 X	-1.410	-1.571 to -1.249	Yes
12176	10:40 X vs. 27:10 X	-1.222	-1.383 to -1.061	Yes
12177	10:40 X vs. 27:40 X	-0.3770	-0.5210 to -0.2330	Yes
12178	10:40 X vs. 27:50 X	-0.2367	-0.3806 to -0.09271	Yes
12179	10:40 X vs. 28:0 X	-1.269	-1.430 to -1.108	Yes
12180	10:40 X vs. 28:1 X	-1.310	-1.454 to -1.166	Yes
12181	10:40 X vs. 28:10 X	-1.111	-1.272 to -0.9504	Yes
12182	10:40 X vs. 28:40 X	-0.3893	-0.5333 to -0.2454	Yes
12183	10:40 X vs. 28:50 X	-0.2847	-0.4286 to -0.1407	Yes
12184	10:40 X vs. 29:0 X	-1.750	-1.911 to -1.589	Yes
12185	10:40 X vs. 29:1 X	-1.835	-1.996 to -1.674	Yes
12186	10:40 X vs. 29:10 X	-1.050	-1.211 to -0.8889	Yes
12187	10:40 X vs. 29:40 X	-0.4973	-0.6413 to -0.3534	Yes
12188	10:40 X vs. 29:50 X	-0.2860	-0.4300 to -0.1420	Yes
12189	10:40 X vs. 30:0 X	-1.465	-1.626 to -1.304	Yes
12190	10:40 X vs. 30:1 X	-1.786	-1.947 to -1.625	Yes
12191	10:40 X vs. 30:10 X	-1.124	-1.285 to -0.9634	Yes
12192	10:40 X vs. 30:40 X	-0.4243	-0.5683 to -0.2804	Yes
12193	10:40 X vs. 30:50 X	-0.3383	-0.4823 to -0.1944	Yes
12194	10:40 X vs. 31:0 X	-1.783	-1.944 to -1.622	Yes
12195	10:40 X vs. 31:1 X	-1.773	-1.934 to -1.612	Yes

2way ANOVA<br>Multiple comparisons		A<br>Data Set-A<br>Y	B<br>Data Set-B<br>Y	C<br>Data Set-C<br>Y
12196	10:**40 X** vs. 31:**10 X**	-1.256	-1.417 to -1.095	Yes
12197	10:**40 X** vs. 31:**40 X**	-0.5160	-0.6600 to -0.3720	Yes
12198	10:**40 X** vs. 31:**50 X**	-0.3173	-0.4613 to -0.1734	Yes
12199	10:**40 X** vs. 32:**0 X**	-2.374	-2.518 to -2.230	Yes
12200	10:**40 X** vs. 32:**1 X**	-1.830	-1.991 to -1.669	Yes
12201	10:**40 X** vs. 32:**10 X**	-1.686	-1.830 to -1.542	Yes
12202	10:**40 X** vs. 32:**40 X**	-0.5123	-0.6563 to -0.3684	Yes
12203	10:**40 X** vs. 32:**50 X**	-0.3170	-0.4610 to -0.1730	Yes
12204	10:**40 X** vs. 33:**0 X**	-2.378	-2.522 to -2.234	Yes
12205	10:**40 X** vs. 33:**1 X**	-2.193	-2.337 to -2.049	Yes
12206	10:**40 X** vs. 33:**10 X**	-1.853	-1.997 to -1.709	Yes
12207	10:**40 X** vs. 33:**40 X**	-0.5310	-0.6750 to -0.3870	Yes
12208	10:**40 X** vs. 33:**50 X**	-0.3320	-0.4760 to -0.1880	Yes
12209	10:**40 X** vs. 34:**0 X**	-2.303	-2.447 to -2.159	Yes
12210	10:**40 X** vs. 34:**1 X**	-2.310	-2.454 to -2.166	Yes
12211	10:**40 X** vs. 34:**10 X**	-1.943	-2.087 to -1.799	Yes
12212	10:**40 X** vs. 34:**40 X**	-0.5430	-0.6870 to -0.3990	Yes
12213	10:**40 X** vs. 34:**50 X**	-0.4810	-0.6250 to -0.3370	Yes
12214	10:**40 X** vs. 35:**0 X**	-2.651	-2.795 to -2.507	Yes
12215	10:**40 X** vs. 35:**1 X**	-2.417	-2.561 to -2.273	Yes
12216	10:**40 X** vs. 35:**10 X**	-1.905	-2.049 to -1.761	Yes
12217	10:**40 X** vs. 35:**40 X**	-0.5520	-0.6960 to -0.4080	Yes
12218	10:**40 X** vs. 35:**50 X**	-0.4937	-0.6376 to -0.3497	Yes
12219	10:**40 X** vs. 36:**0 X**	-2.858	-3.019 to -2.697	Yes
12220	10:**40 X** vs. 36:**1 X**	-2.554	-2.698 to -2.410	Yes
12221	10:**40 X** vs. 36:**10 X**	-1.911	-2.055 to -1.767	Yes
12222	10:**40 X** vs. 36:**40 X**	-0.6073	-0.7513 to -0.4634	Yes
12223	10:**40 X** vs. 36:**50 X**	-0.4570	-0.6010 to -0.3130	Yes
12224	10:**40 X** vs. 37:**0 X**	-3.242	-3.386 to -3.098	Yes
12225	10:**40 X** vs. 37:**1 X**	-2.624	-2.768 to -2.480	Yes
12226	10:**40 X** vs. 37:**10 X**	-1.912	-2.056 to -1.768	Yes
12227	10:**40 X** vs. 37:**40 X**	-0.6447	-0.7886 to -0.5007	Yes
12228	10:**40 X** vs. 37:**50 X**	-0.4657	-0.6096 to -0.3217	Yes
12229	10:**40 X** vs. 38:**0 X**	-3.187	-3.331 to -3.043	Yes
12230	10:**40 X** vs. 38:**1 X**	-2.742	-2.886 to -2.598	Yes
12231	10:**40 X** vs. 38:**10 X**	-2.188	-2.332 to -2.044	Yes
12232	10:**40 X** vs. 38:**40 X**	-0.6490	-0.7930 to -0.5050	Yes
12233	10:**40 X** vs. 38:**50 X**	-0.4697	-0.6136 to -0.3257	Yes
12234	10:**40 X** vs. 39:**0 X**	-3.417	-3.561 to -3.273	Yes
12235	10:**40 X** vs. 39:**1 X**	-3.309	-3.453 to -3.165	Yes
12236	10:**40 X** vs. 39:**10 X**	-2.333	-2.477 to -2.189	Yes
12237	10:**40 X** vs. 39:**40 X**	-0.6550	-0.7990 to -0.5110	Yes
12238	10:**40 X** vs. 39:**50 X**	-0.5007	-0.6446 to -0.3567	Yes
12239	10:**40 X** vs. 40:**0 X**	-3.458	-3.602 to -3.314	Yes
12240	10:**40 X** vs. 40:**1 X**	-3.384	-3.528 to -3.240	Yes

	2way ANOVA<br>Multiple comparisons	A<br>Data Set-A<br>Y	B<br>Data Set-B<br>Y	C<br>Data Set-C<br>Y
12241	10:40 X vs. 40:10 X	-2.589	-2.733 to -2.445	Yes
12242	10:40 X vs. 40:40 X	-0.6597	-0.8036 to -0.5157	Yes
12243	10:40 X vs. 40:50 X	-0.5070	-0.6510 to -0.3630	Yes
12244	10:40 X vs. 41:0 X	-3.499	-3.643 to -3.355	Yes
12245	10:40 X vs. 41:1 X	-3.498	-3.642 to -3.354	Yes
12246	10:40 X vs. 41:10 X	-2.643	-2.787 to -2.499	Yes
12247	10:40 X vs. 41:40 X	-0.6070	-0.7510 to -0.4630	Yes
12248	10:40 X vs. 41:50 X	-0.5197	-0.6636 to -0.3757	Yes
12249	10:40 X vs. 42:0 X	-3.641	-3.785 to -3.497	Yes
12250	10:40 X vs. 42:1 X	-3.661	-3.805 to -3.517	Yes
12251	10:40 X vs. 42:10 X	-2.643	-2.787 to -2.499	Yes
12252	10:40 X vs. 42:40 X	-0.6197	-0.7636 to -0.4757	Yes
12253	10:40 X vs. 42:50 X	-0.5230	-0.6670 to -0.3790	Yes
12254	10:40 X vs. 43:0 X	-3.731	-3.875 to -3.587	Yes
12255	10:40 X vs. 43:1 X	-3.645	-3.789 to -3.501	Yes
12256	10:40 X vs. 43:10 X	-3.361	-3.505 to -3.217	Yes
12257	10:40 X vs. 43:40 X	-0.6180	-0.7620 to -0.4740	Yes
12258	10:40 X vs. 43:50 X	-0.5233	-0.6673 to -0.3794	Yes
12259	10:40 X vs. 44:0 X	-3.755	-3.899 to -3.611	Yes
12260	10:40 X vs. 44:1 X	-3.645	-3.789 to -3.501	Yes
12261	10:40 X vs. 44:10 X	-3.424	-3.568 to -3.280	Yes
12262	10:40 X vs. 44:40 X	-0.6200	-0.7640 to -0.4760	Yes
12263	10:40 X vs. 44:50 X	-0.4557	-0.5996 to -0.3117	Yes
12264	10:40 X vs. 45:0 X	-3.620	-3.764 to -3.476	Yes
12265	10:40 X vs. 45:1 X	-3.422	-3.566 to -3.278	Yes
12266	10:40 X vs. 45:10 X	-3.304	-3.448 to -3.160	Yes
12267	10:40 X vs. 45:40 X	-0.6213	-0.7653 to -0.4774	Yes
12268	10:40 X vs. 45:50 X	-0.4527	-0.5966 to -0.3087	Yes
12269	10:40 X vs. 46:0 X	-3.424	-3.568 to -3.280	Yes
12270	10:40 X vs. 46:1 X	-3.391	-3.535 to -3.247	Yes
12271	10:40 X vs. 46:10 X	-3.299	-3.443 to -3.155	Yes
12272	10:40 X vs. 46:40 X	-0.6350	-0.7790 to -0.4910	Yes
12273	10:40 X vs. 46:50 X	-0.4457	-0.5896 to -0.3017	Yes
12274	10:40 X vs. 47:0 X	-3.400	-3.544 to -3.256	Yes
12275	10:40 X vs. 47:1 X	-3.304	-3.448 to -3.160	Yes
12276	10:40 X vs. 47:10 X	-3.285	-3.429 to -3.141	Yes
12277	10:40 X vs. 47:40 X	-0.6450	-0.7890 to -0.5010	Yes
12278	10:40 X vs. 47:50 X	-0.4037	-0.5476 to -0.2597	Yes
12279	10:40 X vs. 48:0 X	-3.387	-3.548 to -3.226	Yes
12280	10:40 X vs. 48:1 X	-3.291	-3.435 to -3.147	Yes
12281	10:40 X vs. 48:10 X	-3.285	-3.429 to -3.141	Yes
12282	10:40 X vs. 48:40 X	-0.6513	-0.7953 to -0.5074	Yes
12283	10:40 X vs. 48:50 X	-0.4110	-0.5550 to -0.2670	Yes
12284	10:40 X vs. 49:0 X	-3.387	-3.531 to -3.243	Yes
12285	10:40 X vs. 49:1 X	-3.291	-3.435 to -3.147	Yes

2way ANOVA<br>Multiple comparisons		A<br>Data Set-A<br>Y	B<br>Data Set-B<br>Y	C<br>Data Set-C<br>Y
12286	10:40 X vs. 49:10 X	-3.285	-3.429 to -3.141	Yes
12287	10:40 X vs. 49:40 X	-0.6557	-0.7996 to -0.5117	Yes
12288	10:40 X vs. 49:50 X	-0.4103	-0.5543 to -0.2664	Yes
12289	10:40 X vs. 50:0 X	-3.387	-3.531 to -3.243	Yes
12290	10:40 X vs. 50:1 X	-3.291	-3.435 to -3.147	Yes
12291	10:40 X vs. 50:10 X	-3.285	-3.429 to -3.141	Yes
12292	10:40 X vs. 50:40 X	-0.6633	-0.8073 to -0.5194	Yes
12293	10:40 X vs. 50:50 X	-0.4100	-0.5540 to -0.2660	Yes
12294	10:50 X vs. 11:0 X	-0.02367	-0.1676 to 0.1203	No
12295	10:50 X vs. 11:1 X	-0.02533	-0.1693 to 0.1186	No
12296	10:50 X vs. 11:10 X	-0.02567	-0.1696 to 0.1183	No
12297	10:50 X vs. 11:40 X	-0.01733	-0.1613 to 0.1266	No
12298	10:50 X vs. 11:50 X	-0.005333	-0.1493 to 0.1386	No
12299	10:50 X vs. 12:0 X	-0.02433	-0.1683 to 0.1196	No
12300	10:50 X vs. 12:1 X	-0.006667	-0.1506 to 0.1373	No
12301	10:50 X vs. 12:10 X	-0.02433	-0.1683 to 0.1196	No
12302	10:50 X vs. 12:40 X	-0.01067	-0.1546 to 0.1333	No
12303	10:50 X vs. 12:50 X	-0.0140	-0.1580 to 0.1300	No
12304	10:50 X vs. 13:0 X	-0.01833	-0.1623 to 0.1256	No
12305	10:50 X vs. 13:1 X	-0.005667	-0.1496 to 0.1383	No
12306	10:50 X vs. 13:10 X	-0.02767	-0.1716 to 0.1163	No
12307	10:50 X vs. 13:40 X	-0.0240	-0.1680 to 0.1200	No
12308	10:50 X vs. 13:50 X	-0.0250	-0.1690 to 0.1190	No
12309	10:50 X vs. 14:0 X	-0.0270	-0.1710 to 0.1170	No
12310	10:50 X vs. 14:1 X	-0.01867	-0.1626 to 0.1253	No
12311	10:50 X vs. 14:10 X	-0.03267	-0.1766 to 0.1113	No
12312	10:50 X vs. 14:40 X	-0.02933	-0.1733 to 0.1146	No
12313	10:50 X vs. 14:50 X	-0.03333	-0.1773 to 0.1106	No
12314	10:50 X vs. 15:0 X	-0.03933	-0.1833 to 0.1046	No
12315	10:50 X vs. 15:1 X	-0.01933	-0.1633 to 0.1246	No
12316	10:50 X vs. 15:10 X	-0.04667	-0.1906 to 0.09729	No
12317	10:50 X vs. 15:40 X	-0.0290	-0.1730 to 0.1150	No
12318	10:50 X vs. 15:50 X	-0.04667	-0.1906 to 0.09729	No
12319	10:50 X vs. 16:0 X	-0.1317	-0.2756 to 0.01229	No
12320	10:50 X vs. 16:1 X	-0.02267	-0.1666 to 0.1213	No
12321	10:50 X vs. 16:10 X	-0.04833	-0.1923 to 0.09563	No
12322	10:50 X vs. 16:40 X	-0.06767	-0.2116 to 0.07629	No
12323	10:50 X vs. 16:50 X	-0.1100	-0.2540 to 0.03396	No
12324	10:50 X vs. 17:0 X	-0.1843	-0.3283 to -0.04037	Yes
12325	10:50 X vs. 17:1 X	-0.09867	-0.2426 to 0.04529	No
12326	10:50 X vs. 17:10 X	-0.1553	-0.2993 to -0.01137	Yes
12327	10:50 X vs. 17:40 X	-0.07867	-0.2226 to 0.06529	No
12328	10:50 X vs. 17:50 X	-0.1240	-0.2680 to 0.01996	No
12329	10:50 X vs. 18:0 X	-0.2330	-0.3770 to -0.08904	Yes
12330	10:50 X vs. 18:1 X	-0.1413	-0.2853 to 0.002626	No

	2way ANOVA<br>Multiple comparisons	A<br>Data Set-A<br>Y	B<br>Data Set-B<br>Y	C<br>Data Set-C<br>Y
12331	10:50 X vs. 18:10 X	-0.2337	-0.3776 to -0.08971	Yes
12332	10:50 X vs. 18:40 X	-0.0830	-0.2270 to 0.06096	No
12333	10:50 X vs. 18:50 X	-0.1280	-0.2720 to 0.01596	No
12334	10:50 X vs. 19:0 X	-0.2680	-0.4120 to -0.1240	Yes
12335	10:50 X vs. 19:1 X	-0.2560	-0.4000 to -0.1120	Yes
12336	10:50 X vs. 19:10 X	-0.2537	-0.3976 to -0.1097	Yes
12337	10:50 X vs. 19:40 X	-0.1117	-0.2556 to 0.03229	No
12338	10:50 X vs. 19:50 X	-0.1227	-0.2666 to 0.02129	No
12339	10:50 X vs. 20:0 X	-0.3477	-0.4916 to -0.2037	Yes
12340	10:50 X vs. 20:1 X	-0.1670	-0.3110 to -0.02304	Yes
12341	10:50 X vs. 20:10 X	-0.2560	-0.4000 to -0.1120	Yes
12342	10:50 X vs. 20:40 X	-0.1233	-0.2673 to 0.02063	No
12343	10:50 X vs. 20:50 X	-0.1673	-0.3113 to -0.02337	Yes
12344	10:50 X vs. 21:0 X	-0.3710	-0.5150 to -0.2270	Yes
12345	10:50 X vs. 21:1 X	-0.3240	-0.4680 to -0.1800	Yes
12346	10:50 X vs. 21:10 X	-0.3210	-0.4650 to -0.1770	Yes
12347	10:50 X vs. 21:40 X	-0.1963	-0.3403 to -0.05237	Yes
12348	10:50 X vs. 21:50 X	-0.2867	-0.4306 to -0.1427	Yes
12349	10:50 X vs. 22:0 X	-0.4127	-0.5566 to -0.2687	Yes
12350	10:50 X vs. 22:1 X	-0.4073	-0.5513 to -0.2634	Yes
12351	10:50 X vs. 22:10 X	-0.4027	-0.5466 to -0.2587	Yes
12352	10:50 X vs. 22:40 X	-0.3030	-0.4470 to -0.1590	Yes
12353	10:50 X vs. 22:50 X	-0.3657	-0.5096 to -0.2217	Yes
12354	10:50 X vs. 23:0 X	-0.4780	-0.6220 to -0.3340	Yes
12355	10:50 X vs. 23:1 X	-0.6677	-0.8286 to -0.5067	Yes
12356	10:50 X vs. 23:10 X	-0.4173	-0.5613 to -0.2734	Yes
12357	10:50 X vs. 23:40 X	-0.2977	-0.4586 to -0.1367	Yes
12358	10:50 X vs. 23:50 X	-0.3317	-0.4756 to -0.1877	Yes
12359	10:50 X vs. 24:0 X	-0.5953	-0.7393 to -0.4514	Yes
12360	10:50 X vs. 24:1 X	-0.7517	-0.9126 to -0.5907	Yes
12361	10:50 X vs. 24:10 X	-0.5792	-0.7401 to -0.4182	Yes
12362	10:50 X vs. 24:40 X	-0.3140	-0.4580 to -0.1700	Yes
12363	10:50 X vs. 24:50 X	-0.2357	-0.3796 to -0.09171	Yes
12364	10:50 X vs. 25:0 X	-0.9327	-1.094 to -0.7717	Yes
12365	10:50 X vs. 25:1 X	-1.122	-1.283 to -0.9612	Yes
12366	10:50 X vs. 25:10 X	-0.4837	-0.6873 to -0.2801	Yes
12367	10:50 X vs. 25:40 X	-0.3160	-0.4600 to -0.1720	Yes
12368	10:50 X vs. 25:50 X	-0.2903	-0.4343 to -0.1464	Yes
12369	10:50 X vs. 26:0 X	-1.145	-1.306 to -0.9842	Yes
12370	10:50 X vs. 26:1 X	-1.258	-1.419 to -1.097	Yes
12371	10:50 X vs. 26:10 X	-0.9447	-1.106 to -0.7837	Yes
12372	10:50 X vs. 26:40 X	-0.3903	-0.5343 to -0.2464	Yes
12373	10:50 X vs. 26:50 X	-0.2507	-0.3946 to -0.1067	Yes
12374	10:50 X vs. 27:0 X	-1.333	-1.477 to -1.189	Yes
12375	10:50 X vs. 27:1 X	-1.424	-1.585 to -1.263	Yes

	2way ANOVA<br>Multiple comparisons	A<br>Data Set-A<br>Y	B<br>Data Set-B<br>Y	C<br>Data Set-C<br>Y
12376	10:**50 X** vs. 27:**10 X**	-1.236	-1.397 to -1.075	Yes
12377	10:**50 X** vs. 27:**40 X**	-0.3903	-0.5343 to -0.2464	Yes
12378	10:**50 X** vs. 27:**50 X**	-0.2500	-0.3940 to -0.1060	Yes
12379	10:**50 X** vs. 28:**0 X**	-1.282	-1.443 to -1.121	Yes
12380	10:**50 X** vs. 28:**1 X**	-1.323	-1.467 to -1.179	Yes
12381	10:**50 X** vs. 28:**10 X**	-1.125	-1.286 to -0.9637	Yes
12382	10:**50 X** vs. 28:**40 X**	-0.4027	-0.5466 to -0.2587	Yes
12383	10:**50 X** vs. 28:**50 X**	-0.2980	-0.4420 to -0.1540	Yes
12384	10:**50 X** vs. 29:**0 X**	-1.764	-1.925 to -1.603	Yes
12385	10:**50 X** vs. 29:**1 X**	-1.849	-2.010 to -1.688	Yes
12386	10:**50 X** vs. 29:**10 X**	-1.063	-1.224 to -0.9022	Yes
12387	10:**50 X** vs. 29:**40 X**	-0.5107	-0.6546 to -0.3667	Yes
12388	10:**50 X** vs. 29:**50 X**	-0.2993	-0.4433 to -0.1554	Yes
12389	10:**50 X** vs. 30:**0 X**	-1.478	-1.639 to -1.317	Yes
12390	10:**50 X** vs. 30:**1 X**	-1.800	-1.961 to -1.639	Yes
12391	10:**50 X** vs. 30:**10 X**	-1.138	-1.299 to -0.9767	Yes
12392	10:**50 X** vs. 30:**40 X**	-0.4377	-0.5816 to -0.2937	Yes
12393	10:**50 X** vs. 30:**50 X**	-0.3517	-0.4956 to -0.2077	Yes
12394	10:**50 X** vs. 31:**0 X**	-1.796	-1.957 to -1.635	Yes
12395	10:**50 X** vs. 31:**1 X**	-1.787	-1.948 to -1.626	Yes
12396	10:**50 X** vs. 31:**10 X**	-1.269	-1.430 to -1.108	Yes
12397	10:**50 X** vs. 31:**40 X**	-0.5293	-0.6733 to -0.3854	Yes
12398	10:**50 X** vs. 31:**50 X**	-0.3307	-0.4746 to -0.1867	Yes
12399	10:**50 X** vs. 32:**0 X**	-2.388	-2.532 to -2.244	Yes
12400	10:**50 X** vs. 32:**1 X**	-1.844	-2.005 to -1.683	Yes
12401	10:**50 X** vs. 32:**10 X**	-1.700	-1.844 to -1.556	Yes
12402	10:**50 X** vs. 32:**40 X**	-0.5257	-0.6696 to -0.3817	Yes
12403	10:**50 X** vs. 32:**50 X**	-0.3303	-0.4743 to -0.1864	Yes
12404	10:**50 X** vs. 33:**0 X**	-2.391	-2.535 to -2.247	Yes
12405	10:**50 X** vs. 33:**1 X**	-2.206	-2.350 to -2.062	Yes
12406	10:**50 X** vs. 33:**10 X**	-1.866	-2.010 to -1.722	Yes
12407	10:**50 X** vs. 33:**40 X**	-0.5443	-0.6883 to -0.4004	Yes
12408	10:**50 X** vs. 33:**50 X**	-0.3453	-0.4893 to -0.2014	Yes
12409	10:**50 X** vs. 34:**0 X**	-2.316	-2.460 to -2.172	Yes
12410	10:**50 X** vs. 34:**1 X**	-2.323	-2.467 to -2.179	Yes
12411	10:**50 X** vs. 34:**10 X**	-1.957	-2.101 to -1.813	Yes
12412	10:**50 X** vs. 34:**40 X**	-0.5563	-0.7003 to -0.4124	Yes
12413	10:**50 X** vs. 34:**50 X**	-0.4943	-0.6383 to -0.3504	Yes
12414	10:**50 X** vs. 35:**0 X**	-2.665	-2.809 to -2.521	Yes
12415	10:**50 X** vs. 35:**1 X**	-2.430	-2.574 to -2.286	Yes
12416	10:**50 X** vs. 35:**10 X**	-1.918	-2.062 to -1.774	Yes
12417	10:**50 X** vs. 35:**40 X**	-0.5653	-0.7093 to -0.4214	Yes
12418	10:**50 X** vs. 35:**50 X**	-0.5070	-0.6510 to -0.3630	Yes
12419	10:**50 X** vs. 36:**0 X**	-2.871	-3.032 to -2.710	Yes
12420	10:**50 X** vs. 36:**1 X**	-2.567	-2.711 to -2.423	Yes

	2way ANOVA<br>Multiple comparisons	A<br>Data Set-A<br>Y	B<br>Data Set-B<br>Y	C<br>Data Set-C<br>Y
12421	10:50 X vs. 36:10 X	-1.924	-2.068 to -1.780	Yes
12422	10:50 X vs. 36:40 X	-0.6207	-0.7646 to -0.4767	Yes
12423	10:50 X vs. 36:50 X	-0.4703	-0.6143 to -0.3264	Yes
12424	10:50 X vs. 37:0 X	-3.255	-3.399 to -3.111	Yes
12425	10:50 X vs. 37:1 X	-2.637	-2.781 to -2.493	Yes
12426	10:50 X vs. 37:10 X	-1.926	-2.070 to -1.782	Yes
12427	10:50 X vs. 37:40 X	-0.6580	-0.8020 to -0.5140	Yes
12428	10:50 X vs. 37:50 X	-0.4790	-0.6230 to -0.3350	Yes
12429	10:50 X vs. 38:0 X	-3.200	-3.344 to -3.056	Yes
12430	10:50 X vs. 38:1 X	-2.756	-2.900 to -2.612	Yes
12431	10:50 X vs. 38:10 X	-2.201	-2.345 to -2.057	Yes
12432	10:50 X vs. 38:40 X	-0.6623	-0.8063 to -0.5184	Yes
12433	10:50 X vs. 38:50 X	-0.4830	-0.6270 to -0.3390	Yes
12434	10:50 X vs. 39:0 X	-3.431	-3.575 to -3.287	Yes
12435	10:50 X vs. 39:1 X	-3.322	-3.466 to -3.178	Yes
12436	10:50 X vs. 39:10 X	-2.346	-2.490 to -2.202	Yes
12437	10:50 X vs. 39:40 X	-0.6683	-0.8123 to -0.5244	Yes
12438	10:50 X vs. 39:50 X	-0.5140	-0.6580 to -0.3700	Yes
12439	10:50 X vs. 40:0 X	-3.472	-3.616 to -3.328	Yes
12440	10:50 X vs. 40:1 X	-3.398	-3.542 to -3.254	Yes
12441	10:50 X vs. 40:10 X	-2.602	-2.746 to -2.458	Yes
12442	10:50 X vs. 40:40 X	-0.6730	-0.8170 to -0.5290	Yes
12443	10:50 X vs. 40:50 X	-0.5203	-0.6643 to -0.3764	Yes
12444	10:50 X vs. 41:0 X	-3.513	-3.657 to -3.369	Yes
12445	10:50 X vs. 41:1 X	-3.512	-3.656 to -3.368	Yes
12446	10:50 X vs. 41:10 X	-2.656	-2.800 to -2.512	Yes
12447	10:50 X vs. 41:40 X	-0.6203	-0.7643 to -0.4764	Yes
12448	10:50 X vs. 41:50 X	-0.5330	-0.6770 to -0.3890	Yes
12449	10:50 X vs. 42:0 X	-3.654	-3.798 to -3.510	Yes
12450	10:50 X vs. 42:1 X	-3.674	-3.818 to -3.530	Yes
12451	10:50 X vs. 42:10 X	-2.656	-2.800 to -2.512	Yes
12452	10:50 X vs. 42:40 X	-0.6330	-0.7770 to -0.4890	Yes
12453	10:50 X vs. 42:50 X	-0.5363	-0.6803 to -0.3924	Yes
12454	10:50 X vs. 43:0 X	-3.744	-3.888 to -3.600	Yes
12455	10:50 X vs. 43:1 X	-3.659	-3.803 to -3.515	Yes
12456	10:50 X vs. 43:10 X	-3.374	-3.518 to -3.230	Yes
12457	10:50 X vs. 43:40 X	-0.6313	-0.7753 to -0.4874	Yes
12458	10:50 X vs. 43:50 X	-0.5367	-0.6806 to -0.3927	Yes
12459	10:50 X vs. 44:0 X	-3.768	-3.912 to -3.624	Yes
12460	10:50 X vs. 44:1 X	-3.658	-3.802 to -3.514	Yes
12461	10:50 X vs. 44:10 X	-3.438	-3.582 to -3.294	Yes
12462	10:50 X vs. 44:40 X	-0.6333	-0.7773 to -0.4894	Yes
12463	10:50 X vs. 44:50 X	-0.4690	-0.6130 to -0.3250	Yes
12464	10:50 X vs. 45:0 X	-3.633	-3.777 to -3.489	Yes
12465	10:50 X vs. 45:1 X	-3.435	-3.579 to -3.291	Yes

	2way ANOVA<br>Multiple comparisons	A<br>Data Set-A<br>Y	B<br>Data Set-B<br>Y	C<br>Data Set-C<br>Y
12466	10:50 X vs. 45:10 X	-3.317	-3.461 to -3.173	Yes
12467	10:50 X vs. 45:40 X	-0.6347	-0.7786 to -0.4907	Yes
12468	10:50 X vs. 45:50 X	-0.4660	-0.6100 to -0.3220	Yes
12469	10:50 X vs. 46:0 X	-3.437	-3.581 to -3.293	Yes
12470	10:50 X vs. 46:1 X	-3.404	-3.548 to -3.260	Yes
12471	10:50 X vs. 46:10 X	-3.312	-3.456 to -3.168	Yes
12472	10:50 X vs. 46:40 X	-0.6483	-0.7923 to -0.5044	Yes
12473	10:50 X vs. 46:50 X	-0.4590	-0.6030 to -0.3150	Yes
12474	10:50 X vs. 47:0 X	-3.413	-3.557 to -3.269	Yes
12475	10:50 X vs. 47:1 X	-3.317	-3.461 to -3.173	Yes
12476	10:50 X vs. 47:10 X	-3.298	-3.442 to -3.154	Yes
12477	10:50 X vs. 47:40 X	-0.6583	-0.8023 to -0.5144	Yes
12478	10:50 X vs. 47:50 X	-0.4170	-0.5610 to -0.2730	Yes
12479	10:50 X vs. 48:0 X	-3.400	-3.561 to -3.239	Yes
12480	10:50 X vs. 48:1 X	-3.304	-3.448 to -3.160	Yes
12481	10:50 X vs. 48:10 X	-3.298	-3.442 to -3.154	Yes
12482	10:50 X vs. 48:40 X	-0.6647	-0.8086 to -0.5207	Yes
12483	10:50 X vs. 48:50 X	-0.4243	-0.5683 to -0.2804	Yes
12484	10:50 X vs. 49:0 X	-3.400	-3.544 to -3.256	Yes
12485	10:50 X vs. 49:1 X	-3.304	-3.448 to -3.160	Yes
12486	10:50 X vs. 49:10 X	-3.298	-3.442 to -3.154	Yes
12487	10:50 X vs. 49:40 X	-0.6690	-0.8130 to -0.5250	Yes
12488	10:50 X vs. 49:50 X	-0.4237	-0.5676 to -0.2797	Yes
12489	10:50 X vs. 50:0 X	-3.400	-3.544 to -3.256	Yes
12490	10:50 X vs. 50:1 X	-3.304	-3.448 to -3.160	Yes
12491	10:50 X vs. 50:10 X	-3.298	-3.442 to -3.154	Yes
12492	10:50 X vs. 50:40 X	-0.6767	-0.8206 to -0.5327	Yes
12493	10:50 X vs. 50:50 X	-0.4233	-0.5673 to -0.2794	Yes
12494	11:0 X vs. 11:1 X	-0.001667	-0.1456 to 0.1423	No
12495	11:0 X vs. 11:10 X	-0.002000	-0.1460 to 0.1420	No
12496	11:0 X vs. 11:40 X	0.006333	-0.1376 to 0.1503	No
12497	11:0 X vs. 11:50 X	0.01833	-0.1256 to 0.1623	No
12498	11:0 X vs. 12:0 X	-0.0006667	-0.1446 to 0.1433	No
12499	11:0 X vs. 12:1 X	0.0170	-0.1270 to 0.1610	No
12500	11:0 X vs. 12:10 X	-0.0006667	-0.1446 to 0.1433	No
12501	11:0 X vs. 12:40 X	0.0130	-0.1310 to 0.1570	No
12502	11:0 X vs. 12:50 X	0.009667	-0.1343 to 0.1536	No
12503	11:0 X vs. 13:0 X	0.005333	-0.1386 to 0.1493	No
12504	11:0 X vs. 13:1 X	0.0180	-0.1260 to 0.1620	No
12505	11:0 X vs. 13:10 X	-0.004000	-0.1480 to 0.1400	No
12506	11:0 X vs. 13:40 X	-0.0003333	-0.1443 to 0.1436	No
12507	11:0 X vs. 13:50 X	-0.001333	-0.1453 to 0.1426	No
12508	11:0 X vs. 14:0 X	-0.003333	-0.1473 to 0.1406	No
12509	11:0 X vs. 14:1 X	0.005000	-0.1390 to 0.1490	No
12510	11:0 X vs. 14:10 X	-0.0090	-0.1530 to 0.1350	No

	2way ANOVA<br>Multiple comparisons	A<br>Data Set-A<br>Y	B<br>Data Set-B<br>Y	C<br>Data Set-C<br>Y
12511	11:0 X vs. 14:40 X	-0.005667	-0.1496 to 0.1383	No
12512	11:0 X vs. 14:50 X	-0.009667	-0.1536 to 0.1343	No
12513	11:0 X vs. 15:0 X	-0.01567	-0.1596 to 0.1283	No
12514	11:0 X vs. 15:1 X	0.004333	-0.1396 to 0.1483	No
12515	11:0 X vs. 15:10 X	-0.0230	-0.1670 to 0.1210	No
12516	11:0 X vs. 15:40 X	-0.005333	-0.1493 to 0.1386	No
12517	11:0 X vs. 15:50 X	-0.0230	-0.1670 to 0.1210	No
12518	11:0 X vs. 16:0 X	-0.1080	-0.2520 to 0.03596	No
12519	11:0 X vs. 16:1 X	0.001000	-0.1430 to 0.1450	No
12520	11:0 X vs. 16:10 X	-0.02467	-0.1686 to 0.1193	No
12521	11:0 X vs. 16:40 X	-0.0440	-0.1880 to 0.09996	No
12522	11:0 X vs. 16:50 X	-0.08633	-0.2303 to 0.05763	No
12523	11:0 X vs. 17:0 X	-0.1607	-0.3046 to -0.01671	Yes
12524	11:0 X vs. 17:1 X	-0.0750	-0.2190 to 0.06896	No
12525	11:0 X vs. 17:10 X	-0.1317	-0.2756 to 0.01229	No
12526	11:0 X vs. 17:40 X	-0.0550	-0.1990 to 0.08896	No
12527	11:0 X vs. 17:50 X	-0.1003	-0.2443 to 0.04363	No
12528	11:0 X vs. 18:0 X	-0.2093	-0.3533 to -0.06537	Yes
12529	11:0 X vs. 18:1 X	-0.1177	-0.2616 to 0.02629	No
12530	11:0 X vs. 18:10 X	-0.2100	-0.3540 to -0.06604	Yes
12531	11:0 X vs. 18:40 X	-0.05933	-0.2033 to 0.08463	No
12532	11:0 X vs. 18:50 X	-0.1043	-0.2483 to 0.03963	No
12533	11:0 X vs. 19:0 X	-0.2443	-0.3883 to -0.1004	Yes
12534	11:0 X vs. 19:1 X	-0.2323	-0.3763 to -0.08837	Yes
12535	11:0 X vs. 19:10 X	-0.2300	-0.3740 to -0.08604	Yes
12536	11:0 X vs. 19:40 X	-0.0880	-0.2320 to 0.05596	No
12537	11:0 X vs. 19:50 X	-0.0990	-0.2430 to 0.04496	No
12538	11:0 X vs. 20:0 X	-0.3240	-0.4680 to -0.1800	Yes
12539	11:0 X vs. 20:1 X	-0.1433	-0.2873 to 0.0006265	No
12540	11:0 X vs. 20:10 X	-0.2323	-0.3763 to -0.08837	Yes
12541	11:0 X vs. 20:40 X	-0.09967	-0.2436 to 0.04429	No
12542	11:0 X vs. 20:50 X	-0.1437	-0.2876 to 0.0002932	No
12543	11:0 X vs. 21:0 X	-0.3473	-0.4913 to -0.2034	Yes
12544	11:0 X vs. 21:1 X	-0.3003	-0.4443 to -0.1564	Yes
12545	11:0 X vs. 21:10 X	-0.2973	-0.4413 to -0.1534	Yes
12546	11:0 X vs. 21:40 X	-0.1727	-0.3166 to -0.02871	Yes
12547	11:0 X vs. 21:50 X	-0.2630	-0.4070 to -0.1190	Yes
12548	11:0 X vs. 22:0 X	-0.3890	-0.5330 to -0.2450	Yes
12549	11:0 X vs. 22:1 X	-0.3837	-0.5276 to -0.2397	Yes
12550	11:0 X vs. 22:10 X	-0.3790	-0.5230 to -0.2350	Yes
12551	11:0 X vs. 22:40 X	-0.2793	-0.4233 to -0.1354	Yes
12552	11:0 X vs. 22:50 X	-0.3420	-0.4860 to -0.1980	Yes
12553	11:0 X vs. 23:0 X	-0.4543	-0.5983 to -0.3104	Yes
12554	11:0 X vs. 23:1 X	-0.6440	-0.8050 to -0.4830	Yes
12555	11:0 X vs. 23:10 X	-0.3937	-0.5376 to -0.2497	Yes

2way ANOVA<br>Multiple comparisons	A<br>Data Set-A<br>Y	B<br>Data Set-B<br>Y	C<br>Data Set-C<br>Y
12556 11:0 X vs. 23:40 X	-0.2740	-0.4350 to -0.1130	Yes
12557 11:0 X vs. 23:50 X	-0.3080	-0.4520 to -0.1640	Yes
12558 11:0 X vs. 24:0 X	-0.5717	-0.7156 to -0.4277	Yes
12559 11:0 X vs. 24:1 X	-0.7280	-0.8890 to -0.5670	Yes
12560 11:0 X vs. 24:10 X	-0.5555	-0.7165 to -0.3945	Yes
12561 11:0 X vs. 24:40 X	-0.2903	-0.4343 to -0.1464	Yes
12562 11:0 X vs. 24:50 X	-0.2120	-0.3560 to -0.06804	Yes
12563 11:0 X vs. 25:0 X	-0.9090	-1.070 to -0.7480	Yes
12564 11:0 X vs. 25:1 X	-1.099	-1.259 to -0.9375	Yes
12565 11:0 X vs. 25:10 X	-0.4600	-0.6636 to -0.2564	Yes
12566 11:0 X vs. 25:40 X	-0.2923	-0.4363 to -0.1484	Yes
12567 11:0 X vs. 25:50 X	-0.2667	-0.4106 to -0.1227	Yes
12568 11:0 X vs. 26:0 X	-1.122	-1.282 to -0.9605	Yes
12569 11:0 X vs. 26:1 X	-1.235	-1.395 to -1.074	Yes
12570 11:0 X vs. 26:10 X	-0.9210	-1.082 to -0.7600	Yes
12571 11:0 X vs. 26:40 X	-0.3667	-0.5106 to -0.2227	Yes
12572 11:0 X vs. 26:50 X	-0.2270	-0.3710 to -0.08304	Yes
12573 11:0 X vs. 27:0 X	-1.309	-1.453 to -1.165	Yes
12574 11:0 X vs. 27:1 X	-1.400	-1.561 to -1.239	Yes
12575 11:0 X vs. 27:10 X	-1.212	-1.373 to -1.051	Yes
12576 11:0 X vs. 27:40 X	-0.3667	-0.5106 to -0.2227	Yes
12577 11:0 X vs. 27:50 X	-0.2263	-0.3703 to -0.08237	Yes
12578 11:0 X vs. 28:0 X	-1.259	-1.419 to -1.098	Yes
12579 11:0 X vs. 28:1 X	-1.299	-1.443 to -1.155	Yes
12580 11:0 X vs. 28:10 X	-1.101	-1.262 to -0.9400	Yes
12581 11:0 X vs. 28:40 X	-0.3790	-0.5230 to -0.2350	Yes
12582 11:0 X vs. 28:50 X	-0.2743	-0.4183 to -0.1304	Yes
12583 11:0 X vs. 29:0 X	-1.740	-1.901 to -1.579	Yes
12584 11:0 X vs. 29:1 X	-1.825	-1.986 to -1.664	Yes
12585 11:0 X vs. 29:10 X	-1.040	-1.200 to -0.8785	Yes
12586 11:0 X vs. 29:40 X	-0.4870	-0.6310 to -0.3430	Yes
12587 11:0 X vs. 29:50 X	-0.2757	-0.4196 to -0.1317	Yes
12588 11:0 X vs. 30:0 X	-1.455	-1.615 to -1.294	Yes
12589 11:0 X vs. 30:1 X	-1.776	-1.937 to -1.615	Yes
12590 11:0 X vs. 30:10 X	-1.114	-1.275 to -0.9530	Yes
12591 11:0 X vs. 30:40 X	-0.4140	-0.5580 to -0.2700	Yes
12592 11:0 X vs. 30:50 X	-0.3280	-0.4720 to -0.1840	Yes
12593 11:0 X vs. 31:0 X	-1.773	-1.933 to -1.612	Yes
12594 11:0 X vs. 31:1 X	-1.763	-1.924 to -1.602	Yes
12595 11:0 X vs. 31:10 X	-1.246	-1.406 to -1.085	Yes
12596 11:0 X vs. 31:40 X	-0.5057	-0.6496 to -0.3617	Yes
12597 11:0 X vs. 31:50 X	-0.3070	-0.4510 to -0.1630	Yes
12598 11:0 X vs. 32:0 X	-2.364	-2.508 to -2.220	Yes
12599 11:0 X vs. 32:1 X	-1.820	-1.981 to -1.659	Yes
12600 11:0 X vs. 32:10 X	-1.676	-1.820 to -1.532	Yes

2way ANOVA<br>Multiple comparisons		A<br>Data Set-A<br>Y	B<br>Data Set-B<br>Y	C<br>Data Set-C<br>Y
12601	11:0 X vs. 32:40 X	-0.5020	-0.6460 to -0.3580	Yes
12602	11:0 X vs. 32:50 X	-0.3067	-0.4506 to -0.1627	Yes
12603	11:0 X vs. 33:0 X	-2.368	-2.512 to -2.224	Yes
12604	11:0 X vs. 33:1 X	-2.182	-2.326 to -2.038	Yes
12605	11:0 X vs. 33:10 X	-1.843	-1.987 to -1.699	Yes
12606	11:0 X vs. 33:40 X	-0.5207	-0.6646 to -0.3767	Yes
12607	11:0 X vs. 33:50 X	-0.3217	-0.4656 to -0.1777	Yes
12608	11:0 X vs. 34:0 X	-2.292	-2.436 to -2.148	Yes
12609	11:0 X vs. 34:1 X	-2.300	-2.444 to -2.156	Yes
12610	11:0 X vs. 34:10 X	-1.933	-2.077 to -1.789	Yes
12611	11:0 X vs. 34:40 X	-0.5327	-0.6766 to -0.3887	Yes
12612	11:0 X vs. 34:50 X	-0.4707	-0.6146 to -0.3267	Yes
12613	11:0 X vs. 35:0 X	-2.641	-2.785 to -2.497	Yes
12614	11:0 X vs. 35:1 X	-2.407	-2.551 to -2.263	Yes
12615	11:0 X vs. 35:10 X	-1.895	-2.039 to -1.751	Yes
12616	11:0 X vs. 35:40 X	-0.5417	-0.6856 to -0.3977	Yes
12617	11:0 X vs. 35:50 X	-0.4833	-0.6273 to -0.3394	Yes
12618	11:0 X vs. 36:0 X	-2.848	-3.008 to -2.687	Yes
12619	11:0 X vs. 36:1 X	-2.543	-2.687 to -2.399	Yes
12620	11:0 X vs. 36:10 X	-1.900	-2.044 to -1.756	Yes
12621	11:0 X vs. 36:40 X	-0.5970	-0.7410 to -0.4530	Yes
12622	11:0 X vs. 36:50 X	-0.4467	-0.5906 to -0.3027	Yes
12623	11:0 X vs. 37:0 X	-3.231	-3.375 to -3.087	Yes
12624	11:0 X vs. 37:1 X	-2.613	-2.757 to -2.469	Yes
12625	11:0 X vs. 37:10 X	-1.902	-2.046 to -1.758	Yes
12626	11:0 X vs. 37:40 X	-0.6343	-0.7783 to -0.4904	Yes
12627	11:0 X vs. 37:50 X	-0.4553	-0.5993 to -0.3114	Yes
12628	11:0 X vs. 38:0 X	-3.176	-3.320 to -3.032	Yes
12629	11:0 X vs. 38:1 X	-2.732	-2.876 to -2.588	Yes
12630	11:0 X vs. 38:10 X	-2.177	-2.321 to -2.033	Yes
12631	11:0 X vs. 38:40 X	-0.6387	-0.7826 to -0.4947	Yes
12632	11:0 X vs. 38:50 X	-0.4593	-0.6033 to -0.3154	Yes
12633	11:0 X vs. 39:0 X	-3.407	-3.551 to -3.263	Yes
12634	11:0 X vs. 39:1 X	-3.299	-3.443 to -3.155	Yes
12635	11:0 X vs. 39:10 X	-2.322	-2.466 to -2.178	Yes
12636	11:0 X vs. 39:40 X	-0.6447	-0.7886 to -0.5007	Yes
12637	11:0 X vs. 39:50 X	-0.4903	-0.6343 to -0.3464	Yes
12638	11:0 X vs. 40:0 X	-3.448	-3.592 to -3.304	Yes
12639	11:0 X vs. 40:1 X	-3.374	-3.518 to -3.230	Yes
12640	11:0 X vs. 40:10 X	-2.579	-2.723 to -2.435	Yes
12641	11:0 X vs. 40:40 X	-0.6493	-0.7933 to -0.5054	Yes
12642	11:0 X vs. 40:50 X	-0.4967	-0.6406 to -0.3527	Yes
12643	11:0 X vs. 41:0 X	-3.489	-3.633 to -3.345	Yes
12644	11:0 X vs. 41:1 X	-3.488	-3.632 to -3.344	Yes
12645	11:0 X vs. 41:10 X	-2.632	-2.776 to -2.488	Yes

	2way ANOVA<br>Multiple comparisons	A<br>Data Set-A<br>Y	B<br>Data Set-B<br>Y	C<br>Data Set-C<br>Y
12646	11:**0 X** vs. 41:**40 X**	-0.5967	-0.7406 to -0.4527	Yes
12647	11:**0 X** vs. 41:**50 X**	-0.5093	-0.6533 to -0.3654	Yes
12648	11:**0 X** vs. 42:**0 X**	-3.631	-3.775 to -3.487	Yes
12649	11:**0 X** vs. 42:**1 X**	-3.650	-3.794 to -3.506	Yes
12650	11:**0 X** vs. 42:**10 X**	-2.633	-2.777 to -2.489	Yes
12651	11:**0 X** vs. 42:**40 X**	-0.6093	-0.7533 to -0.4654	Yes
12652	11:**0 X** vs. 42:**50 X**	-0.5127	-0.6566 to -0.3687	Yes
12653	11:**0 X** vs. 43:**0 X**	-3.721	-3.865 to -3.577	Yes
12654	11:**0 X** vs. 43:**1 X**	-3.635	-3.779 to -3.491	Yes
12655	11:**0 X** vs. 43:**10 X**	-3.351	-3.495 to -3.207	Yes
12656	11:**0 X** vs. 43:**40 X**	-0.6077	-0.7516 to -0.4637	Yes
12657	11:**0 X** vs. 43:**50 X**	-0.5130	-0.6570 to -0.3690	Yes
12658	11:**0 X** vs. 44:**0 X**	-3.745	-3.889 to -3.601	Yes
12659	11:**0 X** vs. 44:**1 X**	-3.634	-3.778 to -3.490	Yes
12660	11:**0 X** vs. 44:**10 X**	-3.414	-3.558 to -3.270	Yes
12661	11:**0 X** vs. 44:**40 X**	-0.6097	-0.7536 to -0.4657	Yes
12662	11:**0 X** vs. 44:**50 X**	-0.4453	-0.5893 to -0.3014	Yes
12663	11:**0 X** vs. 45:**0 X**	-3.609	-3.753 to -3.465	Yes
12664	11:**0 X** vs. 45:**1 X**	-3.411	-3.555 to -3.267	Yes
12665	11:**0 X** vs. 45:**10 X**	-3.294	-3.438 to -3.150	Yes
12666	11:**0 X** vs. 45:**40 X**	-0.6110	-0.7550 to -0.4670	Yes
12667	11:**0 X** vs. 45:**50 X**	-0.4423	-0.5863 to -0.2984	Yes
12668	11:**0 X** vs. 46:**0 X**	-3.414	-3.558 to -3.270	Yes
12669	11:**0 X** vs. 46:**1 X**	-3.381	-3.525 to -3.237	Yes
12670	11:**0 X** vs. 46:**10 X**	-3.289	-3.433 to -3.145	Yes
12671	11:**0 X** vs. 46:**40 X**	-0.6247	-0.7686 to -0.4807	Yes
12672	11:**0 X** vs. 46:**50 X**	-0.4353	-0.5793 to -0.2914	Yes
12673	11:**0 X** vs. 47:**0 X**	-3.389	-3.533 to -3.245	Yes
12674	11:**0 X** vs. 47:**1 X**	-3.293	-3.437 to -3.149	Yes
12675	11:**0 X** vs. 47:**10 X**	-3.275	-3.419 to -3.131	Yes
12676	11:**0 X** vs. 47:**40 X**	-0.6347	-0.7786 to -0.4907	Yes
12677	11:**0 X** vs. 47:**50 X**	-0.3933	-0.5373 to -0.2494	Yes
12678	11:**0 X** vs. 48:**0 X**	-3.377	-3.537 to -3.216	Yes
12679	11:**0 X** vs. 48:**1 X**	-3.280	-3.424 to -3.136	Yes
12680	11:**0 X** vs. 48:**10 X**	-3.275	-3.419 to -3.131	Yes
12681	11:**0 X** vs. 48:**40 X**	-0.6410	-0.7850 to -0.4970	Yes
12682	11:**0 X** vs. 48:**50 X**	-0.4007	-0.5446 to -0.2567	Yes
12683	11:**0 X** vs. 49:**0 X**	-3.377	-3.521 to -3.233	Yes
12684	11:**0 X** vs. 49:**1 X**	-3.280	-3.424 to -3.136	Yes
12685	11:**0 X** vs. 49:**10 X**	-3.275	-3.419 to -3.131	Yes
12686	11:**0 X** vs. 49:**40 X**	-0.6453	-0.7893 to -0.5014	Yes
12687	11:**0 X** vs. 49:**50 X**	-0.4000	-0.5440 to -0.2560	Yes
12688	11:**0 X** vs. 50:**0 X**	-3.377	-3.521 to -3.233	Yes
12689	11:**0 X** vs. 50:**1 X**	-3.280	-3.424 to -3.136	Yes
12690	11:**0 X** vs. 50:**10 X**	-3.275	-3.419 to -3.131	Yes

2way ANOVA<br>Multiple comparisons	A<br>Data Set-A<br>Y	B<br>Data Set-B<br>Y	C<br>Data Set-C<br>Y
12691 11:**0 X** vs. 50:**40 X**	-0.6530	-0.7970 to -0.5090	Yes
12692 11:**0 X** vs. 50:**50 X**	-0.3997	-0.5436 to -0.2557	Yes
12693 11:**1 X** vs. 11:**10 X**	-0.0003333	-0.1443 to 0.1436	No
12694 11:**1 X** vs. 11:**40 X**	0.0080	-0.1360 to 0.1520	No
12695 11:**1 X** vs. 11:**50 X**	0.0200	-0.1240 to 0.1640	No
12696 11:**1 X** vs. 12:**0 X**	0.001000	-0.1430 to 0.1450	No
12697 11:**1 X** vs. 12:**1 X**	0.01867	-0.1253 to 0.1626	No
12698 11:**1 X** vs. 12:**10 X**	0.001000	-0.1430 to 0.1450	No
12699 11:**1 X** vs. 12:**40 X**	0.01467	-0.1293 to 0.1586	No
12700 11:**1 X** vs. 12:**50 X**	0.01133	-0.1326 to 0.1553	No
12701 11:**1 X** vs. 13:**0 X**	0.007000	-0.1370 to 0.1510	No
12702 11:**1 X** vs. 13:**1 X**	0.01967	-0.1243 to 0.1636	No
12703 11:**1 X** vs. 13:**10 X**	-0.002333	-0.1463 to 0.1416	No
12704 11:**1 X** vs. 13:**40 X**	0.001333	-0.1426 to 0.1453	No
12705 11:**1 X** vs. 13:**50 X**	0.0003333	-0.1436 to 0.1443	No
12706 11:**1 X** vs. 14:**0 X**	-0.001667	-0.1456 to 0.1423	No
12707 11:**1 X** vs. 14:**1 X**	0.006667	-0.1373 to 0.1506	No
12708 11:**1 X** vs. 14:**10 X**	-0.007333	-0.1513 to 0.1366	No
12709 11:**1 X** vs. 14:**40 X**	-0.004000	-0.1480 to 0.1400	No
12710 11:**1 X** vs. 14:**50 X**	-0.008000	-0.1520 to 0.1360	No
12711 11:**1 X** vs. 15:**0 X**	-0.0140	-0.1580 to 0.1300	No
12712 11:**1 X** vs. 15:**1 X**	0.0060	-0.1380 to 0.1500	No
12713 11:**1 X** vs. 15:**10 X**	-0.02133	-0.1653 to 0.1226	No
12714 11:**1 X** vs. 15:**40 X**	-0.003667	-0.1476 to 0.1403	No
12715 11:**1 X** vs. 15:**50 X**	-0.02133	-0.1653 to 0.1226	No
12716 11:**1 X** vs. 16:**0 X**	-0.1063	-0.2503 to 0.03763	No
12717 11:**1 X** vs. 16:**1 X**	0.002667	-0.1413 to 0.1466	No
12718 11:**1 X** vs. 16:**10 X**	-0.0230	-0.1670 to 0.1210	No
12719 11:**1 X** vs. 16:**40 X**	-0.04233	-0.1863 to 0.1016	No
12720 11:**1 X** vs. 16:**50 X**	-0.08467	-0.2286 to 0.05929	No
12721 11:**1 X** vs. 17:**0 X**	-0.1590	-0.3030 to -0.01504	Yes
12722 11:**1 X** vs. 17:**1 X**	-0.07333	-0.2173 to 0.07063	No
12723 11:**1 X** vs. 17:**10 X**	-0.1300	-0.2740 to 0.01396	No
12724 11:**1 X** vs. 17:**40 X**	-0.05333	-0.1973 to 0.09063	No
12725 11:**1 X** vs. 17:**50 X**	-0.09867	-0.2426 to 0.04529	No
12726 11:**1 X** vs. 18:**0 X**	-0.2077	-0.3516 to -0.06371	Yes
12727 11:**1 X** vs. 18:**1 X**	-0.1160	-0.2600 to 0.02796	No
12728 11:**1 X** vs. 18:**10 X**	-0.2083	-0.3523 to -0.06437	Yes
12729 11:**1 X** vs. 18:**40 X**	-0.05767	-0.2016 to 0.08629	No
12730 11:**1 X** vs. 18:**50 X**	-0.1027	-0.2466 to 0.04129	No
12731 11:**1 X** vs. 19:**0 X**	-0.2427	-0.3866 to -0.09871	Yes
12732 11:**1 X** vs. 19:**1 X**	-0.2307	-0.3746 to -0.08671	Yes
12733 11:**1 X** vs. 19:**10 X**	-0.2283	-0.3723 to -0.08437	Yes
12734 11:**1 X** vs. 19:**40 X**	-0.08633	-0.2303 to 0.05763	No
12735 11:**1 X** vs. 19:**50 X**	-0.09733	-0.2413 to 0.04663	No

	2way ANOVA<br>Multiple comparisons	A<br>Data Set-A<br>Y	B<br>Data Set-B<br>Y	C<br>Data Set-C<br>Y
12736	11:1 X vs. 20:0 X	-0.3223	-0.4663 to -0.1784	Yes
12737	11:1 X vs. 20:1 X	-0.1417	-0.2856 to 0.002293	No
12738	11:1 X vs. 20:10 X	-0.2307	-0.3746 to -0.08671	Yes
12739	11:1 X vs. 20:40 X	-0.0980	-0.2420 to 0.04596	No
12740	11:1 X vs. 20:50 X	-0.1420	-0.2860 to 0.001960	No
12741	11:1 X vs. 21:0 X	-0.3457	-0.4896 to -0.2017	Yes
12742	11:1 X vs. 21:1 X	-0.2987	-0.4426 to -0.1547	Yes
12743	11:1 X vs. 21:10 X	-0.2957	-0.4396 to -0.1517	Yes
12744	11:1 X vs. 21:40 X	-0.1710	-0.3150 to -0.02704	Yes
12745	11:1 X vs. 21:50 X	-0.2613	-0.4053 to -0.1174	Yes
12746	11:1 X vs. 22:0 X	-0.3873	-0.5313 to -0.2434	Yes
12747	11:1 X vs. 22:1 X	-0.3820	-0.5260 to -0.2380	Yes
12748	11:1 X vs. 22:10 X	-0.3773	-0.5213 to -0.2334	Yes
12749	11:1 X vs. 22:40 X	-0.2777	-0.4216 to -0.1337	Yes
12750	11:1 X vs. 22:50 X	-0.3403	-0.4843 to -0.1964	Yes
12751	11:1 X vs. 23:0 X	-0.4527	-0.5966 to -0.3087	Yes
12752	11:1 X vs. 23:1 X	-0.6423	-0.8033 to -0.4814	Yes
12753	11:1 X vs. 23:10 X	-0.3920	-0.5360 to -0.2480	Yes
12754	11:1 X vs. 23:40 X	-0.2723	-0.4333 to -0.1114	Yes
12755	11:1 X vs. 23:50 X	-0.3063	-0.4503 to -0.1624	Yes
12756	11:1 X vs. 24:0 X	-0.5700	-0.7140 to -0.4260	Yes
12757	11:1 X vs. 24:1 X	-0.7263	-0.8873 to -0.5654	Yes
12758	11:1 X vs. 24:10 X	-0.5538	-0.7148 to -0.3929	Yes
12759	11:1 X vs. 24:40 X	-0.2887	-0.4326 to -0.1447	Yes
12760	11:1 X vs. 24:50 X	-0.2103	-0.3543 to -0.06637	Yes
12761	11:1 X vs. 25:0 X	-0.9073	-1.068 to -0.7464	Yes
12762	11:1 X vs. 25:1 X	-1.097	-1.258 to -0.9359	Yes
12763	11:1 X vs. 25:10 X	-0.4583	-0.6619 to -0.2547	Yes
12764	11:1 X vs. 25:40 X	-0.2907	-0.4346 to -0.1467	Yes
12765	11:1 X vs. 25:50 X	-0.2650	-0.4090 to -0.1210	Yes
12766	11:1 X vs. 26:0 X	-1.120	-1.281 to -0.9589	Yes
12767	11:1 X vs. 26:1 X	-1.233	-1.394 to -1.072	Yes
12768	11:1 X vs. 26:10 X	-0.9193	-1.080 to -0.7584	Yes
12769	11:1 X vs. 26:40 X	-0.3650	-0.5090 to -0.2210	Yes
12770	11:1 X vs. 26:50 X	-0.2253	-0.3693 to -0.08137	Yes
12771	11:1 X vs. 27:0 X	-1.308	-1.452 to -1.164	Yes
12772	11:1 X vs. 27:1 X	-1.398	-1.559 to -1.237	Yes
12773	11:1 X vs. 27:10 X	-1.210	-1.371 to -1.049	Yes
12774	11:1 X vs. 27:40 X	-0.3650	-0.5090 to -0.2210	Yes
12775	11:1 X vs. 27:50 X	-0.2247	-0.3686 to -0.08071	Yes
12776	11:1 X vs. 28:0 X	-1.257	-1.418 to -1.096	Yes
12777	11:1 X vs. 28:1 X	-1.298	-1.442 to -1.154	Yes
12778	11:1 X vs. 28:10 X	-1.099	-1.260 to -0.9384	Yes
12779	11:1 X vs. 28:40 X	-0.3773	-0.5213 to -0.2334	Yes
12780	11:1 X vs. 28:50 X	-0.2727	-0.4166 to -0.1287	Yes

2way ANOVA<br>Multiple comparisons	A<br>Data Set-A<br>Y	B<br>Data Set-B<br>Y	C<br>Data Set-C<br>Y
12781 11:1 X vs. 29:0 X	-1.738	-1.899 to -1.577	Yes
12782 11:1 X vs. 29:1 X	-1.823	-1.984 to -1.662	Yes
12783 11:1 X vs. 29:10 X	-1.038	-1.199 to -0.8769	Yes
12784 11:1 X vs. 29:40 X	-0.4853	-0.6293 to -0.3414	Yes
12785 11:1 X vs. 29:50 X	-0.2740	-0.4180 to -0.1300	Yes
12786 11:1 X vs. 30:0 X	-1.453	-1.614 to -1.292	Yes
12787 11:1 X vs. 30:1 X	-1.774	-1.935 to -1.613	Yes
12788 11:1 X vs. 30:10 X	-1.112	-1.273 to -0.9514	Yes
12789 11:1 X vs. 30:40 X	-0.4123	-0.5563 to -0.2684	Yes
12790 11:1 X vs. 30:50 X	-0.3263	-0.4703 to -0.1824	Yes
12791 11:1 X vs. 31:0 X	-1.771	-1.932 to -1.610	Yes
12792 11:1 X vs. 31:1 X	-1.761	-1.922 to -1.600	Yes
12793 11:1 X vs. 31:10 X	-1.244	-1.405 to -1.083	Yes
12794 11:1 X vs. 31:40 X	-0.5040	-0.6480 to -0.3600	Yes
12795 11:1 X vs. 31:50 X	-0.3053	-0.4493 to -0.1614	Yes
12796 11:1 X vs. 32:0 X	-2.362	-2.506 to -2.218	Yes
12797 11:1 X vs. 32:1 X	-1.818	-1.979 to -1.657	Yes
12798 11:1 X vs. 32:10 X	-1.674	-1.818 to -1.530	Yes
12799 11:1 X vs. 32:40 X	-0.5003	-0.6443 to -0.3564	Yes
12800 11:1 X vs. 32:50 X	-0.3050	-0.4490 to -0.1610	Yes
12801 11:1 X vs. 33:0 X	-2.366	-2.510 to -2.222	Yes
12802 11:1 X vs. 33:1 X	-2.181	-2.325 to -2.037	Yes
12803 11:1 X vs. 33:10 X	-1.841	-1.985 to -1.697	Yes
12804 11:1 X vs. 33:40 X	-0.5190	-0.6630 to -0.3750	Yes
12805 11:1 X vs. 33:50 X	-0.3200	-0.4640 to -0.1760	Yes
12806 11:1 X vs. 34:0 X	-2.291	-2.435 to -2.147	Yes
12807 11:1 X vs. 34:1 X	-2.298	-2.442 to -2.154	Yes
12808 11:1 X vs. 34:10 X	-1.931	-2.075 to -1.787	Yes
12809 11:1 X vs. 34:40 X	-0.5310	-0.6750 to -0.3870	Yes
12810 11:1 X vs. 34:50 X	-0.4690	-0.6130 to -0.3250	Yes
12811 11:1 X vs. 35:0 X	-2.639	-2.783 to -2.495	Yes
12812 11:1 X vs. 35:1 X	-2.405	-2.549 to -2.261	Yes
12813 11:1 X vs. 35:10 X	-1.893	-2.037 to -1.749	Yes
12814 11:1 X vs. 35:40 X	-0.5400	-0.6840 to -0.3960	Yes
12815 11:1 X vs. 35:50 X	-0.4817	-0.6256 to -0.3377	Yes
12816 11:1 X vs. 36:0 X	-2.846	-3.007 to -2.685	Yes
12817 11:1 X vs. 36:1 X	-2.542	-2.686 to -2.398	Yes
12818 11:1 X vs. 36:10 X	-1.899	-2.043 to -1.755	Yes
12819 11:1 X vs. 36:40 X	-0.5953	-0.7393 to -0.4514	Yes
12820 11:1 X vs. 36:50 X	-0.4450	-0.5890 to -0.3010	Yes
12821 11:1 X vs. 37:0 X	-3.230	-3.374 to -3.086	Yes
12822 11:1 X vs. 37:1 X	-2.612	-2.756 to -2.468	Yes
12823 11:1 X vs. 37:10 X	-1.900	-2.044 to -1.756	Yes
12824 11:1 X vs. 37:40 X	-0.6327	-0.7766 to -0.4887	Yes
12825 11:1 X vs. 37:50 X	-0.4537	-0.5976 to -0.3097	Yes

	2way ANOVA<br>Multiple comparisons	A<br>Data Set-A<br>Y	B<br>Data Set-B<br>Y	C<br>Data Set-C<br>Y
12826	11:**1 X** vs. 38:**0 X**	-3.175	-3.319 to -3.031	Yes
12827	11:**1 X** vs. 38:**1 X**	-2.730	-2.874 to -2.586	Yes
12828	11:**1 X** vs. 38:**10 X**	-2.176	-2.320 to -2.032	Yes
12829	11:**1 X** vs. 38:**40 X**	-0.6370	-0.7810 to -0.4930	Yes
12830	11:**1 X** vs. 38:**50 X**	-0.4577	-0.6016 to -0.3137	Yes
12831	11:**1 X** vs. 39:**0 X**	-3.405	-3.549 to -3.261	Yes
12832	11:**1 X** vs. 39:**1 X**	-3.297	-3.441 to -3.153	Yes
12833	11:**1 X** vs. 39:**10 X**	-2.321	-2.465 to -2.177	Yes
12834	11:**1 X** vs. 39:**40 X**	-0.6430	-0.7870 to -0.4990	Yes
12835	11:**1 X** vs. 39:**50 X**	-0.4887	-0.6326 to -0.3447	Yes
12836	11:**1 X** vs. 40:**0 X**	-3.446	-3.590 to -3.302	Yes
12837	11:**1 X** vs. 40:**1 X**	-3.372	-3.516 to -3.228	Yes
12838	11:**1 X** vs. 40:**10 X**	-2.577	-2.721 to -2.433	Yes
12839	11:**1 X** vs. 40:**40 X**	-0.6477	-0.7916 to -0.5037	Yes
12840	11:**1 X** vs. 40:**50 X**	-0.4950	-0.6390 to -0.3510	Yes
12841	11:**1 X** vs. 41:**0 X**	-3.487	-3.631 to -3.343	Yes
12842	11:**1 X** vs. 41:**1 X**	-3.486	-3.630 to -3.342	Yes
12843	11:**1 X** vs. 41:**10 X**	-2.631	-2.775 to -2.487	Yes
12844	11:**1 X** vs. 41:**40 X**	-0.5950	-0.7390 to -0.4510	Yes
12845	11:**1 X** vs. 41:**50 X**	-0.5077	-0.6516 to -0.3637	Yes
12846	11:**1 X** vs. 42:**0 X**	-3.629	-3.773 to -3.485	Yes
12847	11:**1 X** vs. 42:**1 X**	-3.649	-3.793 to -3.505	Yes
12848	11:**1 X** vs. 42:**10 X**	-2.631	-2.775 to -2.487	Yes
12849	11:**1 X** vs. 42:**40 X**	-0.6077	-0.7516 to -0.4637	Yes
12850	11:**1 X** vs. 42:**50 X**	-0.5110	-0.6550 to -0.3670	Yes
12851	11:**1 X** vs. 43:**0 X**	-3.719	-3.863 to -3.575	Yes
12852	11:**1 X** vs. 43:**1 X**	-3.633	-3.777 to -3.489	Yes
12853	11:**1 X** vs. 43:**10 X**	-3.349	-3.493 to -3.205	Yes
12854	11:**1 X** vs. 43:**40 X**	-0.6060	-0.7500 to -0.4620	Yes
12855	11:**1 X** vs. 43:**50 X**	-0.5113	-0.6553 to -0.3674	Yes
12856	11:**1 X** vs. 44:**0 X**	-3.743	-3.887 to -3.599	Yes
12857	11:**1 X** vs. 44:**1 X**	-3.633	-3.777 to -3.489	Yes
12858	11:**1 X** vs. 44:**10 X**	-3.412	-3.556 to -3.268	Yes
12859	11:**1 X** vs. 44:**40 X**	-0.6080	-0.7520 to -0.4640	Yes
12860	11:**1 X** vs. 44:**50 X**	-0.4437	-0.5876 to -0.2997	Yes
12861	11:**1 X** vs. 45:**0 X**	-3.608	-3.752 to -3.464	Yes
12862	11:**1 X** vs. 45:**1 X**	-3.410	-3.554 to -3.266	Yes
12863	11:**1 X** vs. 45:**10 X**	-3.292	-3.436 to -3.148	Yes
12864	11:**1 X** vs. 45:**40 X**	-0.6093	-0.7533 to -0.4654	Yes
12865	11:**1 X** vs. 45:**50 X**	-0.4407	-0.5846 to -0.2967	Yes
12866	11:**1 X** vs. 46:**0 X**	-3.412	-3.556 to -3.268	Yes
12867	11:**1 X** vs. 46:**1 X**	-3.379	-3.523 to -3.235	Yes
12868	11:**1 X** vs. 46:**10 X**	-3.287	-3.431 to -3.143	Yes
12869	11:**1 X** vs. 46:**40 X**	-0.6230	-0.7670 to -0.4790	Yes
12870	11:**1 X** vs. 46:**50 X**	-0.4337	-0.5776 to -0.2897	Yes

2way ANOVA<br>Multiple comparisons	A<br>Data Set-A<br>Y	B<br>Data Set-B<br>Y	C<br>Data Set-C<br>Y
12871 11:1 X vs. 47:0 X	-3.388	-3.532 to -3.244	Yes
12872 11:1 X vs. 47:1 X	-3.292	-3.436 to -3.148	Yes
12873 11:1 X vs. 47:10 X	-3.273	-3.417 to -3.129	Yes
12874 11:1 X vs. 47:40 X	-0.6330	-0.7770 to -0.4890	Yes
12875 11:1 X vs. 47:50 X	-0.3917	-0.5356 to -0.2477	Yes
12876 11:1 X vs. 48:0 X	-3.375	-3.536 to -3.214	Yes
12877 11:1 X vs. 48:1 X	-3.279	-3.423 to -3.135	Yes
12878 11:1 X vs. 48:10 X	-3.273	-3.417 to -3.129	Yes
12879 11:1 X vs. 48:40 X	-0.6393	-0.7833 to -0.4954	Yes
12880 11:1 X vs. 48:50 X	-0.3990	-0.5430 to -0.2550	Yes
12881 11:1 X vs. 49:0 X	-3.375	-3.519 to -3.231	Yes
12882 11:1 X vs. 49:1 X	-3.279	-3.423 to -3.135	Yes
12883 11:1 X vs. 49:10 X	-3.273	-3.417 to -3.129	Yes
12884 11:1 X vs. 49:40 X	-0.6437	-0.7876 to -0.4997	Yes
12885 11:1 X vs. 49:50 X	-0.3983	-0.5423 to -0.2544	Yes
12886 11:1 X vs. 50:0 X	-3.375	-3.519 to -3.231	Yes
12887 11:1 X vs. 50:1 X	-3.279	-3.423 to -3.135	Yes
12888 11:1 X vs. 50:10 X	-3.273	-3.417 to -3.129	Yes
12889 11:1 X vs. 50:40 X	-0.6513	-0.7953 to -0.5074	Yes
12890 11:1 X vs. 50:50 X	-0.3980	-0.5420 to -0.2540	Yes
12891 11:10 X vs. 11:40 X	0.008333	-0.1356 to 0.1523	No
12892 11:10 X vs. 11:50 X	0.02033	-0.1236 to 0.1643	No
12893 11:10 X vs. 12:0 X	0.001333	-0.1426 to 0.1453	No
12894 11:10 X vs. 12:1 X	0.0190	-0.1250 to 0.1630	No
12895 11:10 X vs. 12:10 X	0.001333	-0.1426 to 0.1453	No
12896 11:10 X vs. 12:40 X	0.0150	-0.1290 to 0.1590	No
12897 11:10 X vs. 12:50 X	0.01167	-0.1323 to 0.1556	No
12898 11:10 X vs. 13:0 X	0.007333	-0.1366 to 0.1513	No
12899 11:10 X vs. 13:1 X	0.0200	-0.1240 to 0.1640	No
12900 11:10 X vs. 13:10 X	-0.0020	-0.1460 to 0.1420	No
12901 11:10 X vs. 13:40 X	0.001667	-0.1423 to 0.1456	No
12902 11:10 X vs. 13:50 X	0.0006667	-0.1433 to 0.1446	No
12903 11:10 X vs. 14:0 X	-0.001333	-0.1453 to 0.1426	No
12904 11:10 X vs. 14:1 X	0.007000	-0.1370 to 0.1510	No
12905 11:10 X vs. 14:10 X	-0.007000	-0.1510 to 0.1370	No
12906 11:10 X vs. 14:40 X	-0.003667	-0.1476 to 0.1403	No
12907 11:10 X vs. 14:50 X	-0.007667	-0.1516 to 0.1363	No
12908 11:10 X vs. 15:0 X	-0.01367	-0.1576 to 0.1303	No
12909 11:10 X vs. 15:1 X	0.006333	-0.1376 to 0.1503	No
12910 11:10 X vs. 15:10 X	-0.0210	-0.1650 to 0.1230	No
12911 11:10 X vs. 15:40 X	-0.003333	-0.1473 to 0.1406	No
12912 11:10 X vs. 15:50 X	-0.0210	-0.1650 to 0.1230	No
12913 11:10 X vs. 16:0 X	-0.1060	-0.2500 to 0.03796	No
12914 11:10 X vs. 16:1 X	0.0030	-0.1410 to 0.1470	No
12915 11:10 X vs. 16:10 X	-0.02267	-0.1666 to 0.1213	No

	2way ANOVA<br>Multiple comparisons	A<br>Data Set-A<br>Y	B<br>Data Set-B<br>Y	C<br>Data Set-C<br>Y
12916	11:10 X vs. 16:40 X	-0.0420	-0.1860 to 0.1020	No
12917	11:10 X vs. 16:50 X	-0.08433	-0.2283 to 0.05963	No
12918	11:10 X vs. 17:0 X	-0.1587	-0.3026 to -0.01471	Yes
12919	11:10 X vs. 17:1 X	-0.0730	-0.2170 to 0.07096	No
12920	11:10 X vs. 17:10 X	-0.1297	-0.2736 to 0.01429	No
12921	11:10 X vs. 17:40 X	-0.0530	-0.1970 to 0.09096	No
12922	11:10 X vs. 17:50 X	-0.09833	-0.2423 to 0.04563	No
12923	11:10 X vs. 18:0 X	-0.2073	-0.3513 to -0.06337	Yes
12924	11:10 X vs. 18:1 X	-0.1157	-0.2596 to 0.02829	No
12925	11:10 X vs. 18:10 X	-0.2080	-0.3520 to -0.06404	Yes
12926	11:10 X vs. 18:40 X	-0.05733	-0.2013 to 0.08663	No
12927	11:10 X vs. 18:50 X	-0.1023	-0.2463 to 0.04163	No
12928	11:10 X vs. 19:0 X	-0.2423	-0.3863 to -0.09837	Yes
12929	11:10 X vs. 19:1 X	-0.2303	-0.3743 to -0.08637	Yes
12930	11:10 X vs. 19:10 X	-0.2280	-0.3720 to -0.08404	Yes
12931	11:10 X vs. 19:40 X	-0.0860	-0.2300 to 0.05796	No
12932	11:10 X vs. 19:50 X	-0.0970	-0.2410 to 0.04696	No
12933	11:10 X vs. 20:0 X	-0.3220	-0.4660 to -0.1780	Yes
12934	11:10 X vs. 20:1 X	-0.1413	-0.2853 to 0.002626	No
12935	11:10 X vs. 20:10 X	-0.2303	-0.3743 to -0.08637	Yes
12936	11:10 X vs. 20:40 X	-0.09767	-0.2416 to 0.04629	No
12937	11:10 X vs. 20:50 X	-0.1417	-0.2856 to 0.002293	No
12938	11:10 X vs. 21:0 X	-0.3453	-0.4893 to -0.2014	Yes
12939	11:10 X vs. 21:1 X	-0.2983	-0.4423 to -0.1544	Yes
12940	11:10 X vs. 21:10 X	-0.2953	-0.4393 to -0.1514	Yes
12941	11:10 X vs. 21:40 X	-0.1707	-0.3146 to -0.02671	Yes
12942	11:10 X vs. 21:50 X	-0.2610	-0.4050 to -0.1170	Yes
12943	11:10 X vs. 22:0 X	-0.3870	-0.5310 to -0.2430	Yes
12944	11:10 X vs. 22:1 X	-0.3817	-0.5256 to -0.2377	Yes
12945	11:10 X vs. 22:10 X	-0.3770	-0.5210 to -0.2330	Yes
12946	11:10 X vs. 22:40 X	-0.2773	-0.4213 to -0.1334	Yes
12947	11:10 X vs. 22:50 X	-0.3400	-0.4840 to -0.1960	Yes
12948	11:10 X vs. 23:0 X	-0.4523	-0.5963 to -0.3084	Yes
12949	11:10 X vs. 23:1 X	-0.6420	-0.8030 to -0.4810	Yes
12950	11:10 X vs. 23:10 X	-0.3917	-0.5356 to -0.2477	Yes
12951	11:10 X vs. 23:40 X	-0.2720	-0.4330 to -0.1110	Yes
12952	11:10 X vs. 23:50 X	-0.3060	-0.4500 to -0.1620	Yes
12953	11:10 X vs. 24:0 X	-0.5697	-0.7136 to -0.4257	Yes
12954	11:10 X vs. 24:1 X	-0.7260	-0.8870 to -0.5650	Yes
12955	11:10 X vs. 24:10 X	-0.5535	-0.7145 to -0.3925	Yes
12956	11:10 X vs. 24:40 X	-0.2883	-0.4323 to -0.1444	Yes
12957	11:10 X vs. 24:50 X	-0.2100	-0.3540 to -0.06604	Yes
12958	11:10 X vs. 25:0 X	-0.9070	-1.068 to -0.7460	Yes
12959	11:10 X vs. 25:1 X	-1.097	-1.257 to -0.9355	Yes
12960	11:10 X vs. 25:10 X	-0.4580	-0.6616 to -0.2544	Yes

	2way ANOVA<br>Multiple comparisons	A<br>Data Set-A<br>Y	B<br>Data Set-B<br>Y	C<br>Data Set-C<br>Y
12961	11:10 X vs. 25:40 X	-0.2903	-0.4343 to -0.1464	Yes
12962	11:10 X vs. 25:50 X	-0.2647	-0.4086 to -0.1207	Yes
12963	11:10 X vs. 26:0 X	-1.120	-1.280 to -0.9585	Yes
12964	11:10 X vs. 26:1 X	-1.233	-1.393 to -1.072	Yes
12965	11:10 X vs. 26:10 X	-0.9190	-1.080 to -0.7580	Yes
12966	11:10 X vs. 26:40 X	-0.3647	-0.5086 to -0.2207	Yes
12967	11:10 X vs. 26:50 X	-0.2250	-0.3690 to -0.08104	Yes
12968	11:10 X vs. 27:0 X	-1.307	-1.451 to -1.163	Yes
12969	11:10 X vs. 27:1 X	-1.398	-1.559 to -1.237	Yes
12970	11:10 X vs. 27:10 X	-1.210	-1.371 to -1.049	Yes
12971	11:10 X vs. 27:40 X	-0.3647	-0.5086 to -0.2207	Yes
12972	11:10 X vs. 27:50 X	-0.2243	-0.3683 to -0.08037	Yes
12973	11:10 X vs. 28:0 X	-1.257	-1.417 to -1.096	Yes
12974	11:10 X vs. 28:1 X	-1.297	-1.441 to -1.153	Yes
12975	11:10 X vs. 28:10 X	-1.099	-1.260 to -0.9380	Yes
12976	11:10 X vs. 28:40 X	-0.3770	-0.5210 to -0.2330	Yes
12977	11:10 X vs. 28:50 X	-0.2723	-0.4163 to -0.1284	Yes
12978	11:10 X vs. 29:0 X	-1.738	-1.899 to -1.577	Yes
12979	11:10 X vs. 29:1 X	-1.823	-1.984 to -1.662	Yes
12980	11:10 X vs. 29:10 X	-1.038	-1.198 to -0.8765	Yes
12981	11:10 X vs. 29:40 X	-0.4850	-0.6290 to -0.3410	Yes
12982	11:10 X vs. 29:50 X	-0.2737	-0.4176 to -0.1297	Yes
12983	11:10 X vs. 30:0 X	-1.453	-1.613 to -1.292	Yes
12984	11:10 X vs. 30:1 X	-1.774	-1.935 to -1.613	Yes
12985	11:10 X vs. 30:10 X	-1.112	-1.273 to -0.9510	Yes
12986	11:10 X vs. 30:40 X	-0.4120	-0.5560 to -0.2680	Yes
12987	11:10 X vs. 30:50 X	-0.3260	-0.4700 to -0.1820	Yes
12988	11:10 X vs. 31:0 X	-1.771	-1.931 to -1.610	Yes
12989	11:10 X vs. 31:1 X	-1.761	-1.922 to -1.600	Yes
12990	11:10 X vs. 31:10 X	-1.244	-1.404 to -1.083	Yes
12991	11:10 X vs. 31:40 X	-0.5037	-0.6476 to -0.3597	Yes
12992	11:10 X vs. 31:50 X	-0.3050	-0.4490 to -0.1610	Yes
12993	11:10 X vs. 32:0 X	-2.362	-2.506 to -2.218	Yes
12994	11:10 X vs. 32:1 X	-1.818	-1.979 to -1.657	Yes
12995	11:10 X vs. 32:10 X	-1.674	-1.818 to -1.530	Yes
12996	11:10 X vs. 32:40 X	-0.5000	-0.6440 to -0.3560	Yes
12997	11:10 X vs. 32:50 X	-0.3047	-0.4486 to -0.1607	Yes
12998	11:10 X vs. 33:0 X	-2.366	-2.510 to -2.222	Yes
12999	11:10 X vs. 33:1 X	-2.180	-2.324 to -2.036	Yes
13000	11:10 X vs. 33:10 X	-1.841	-1.985 to -1.697	Yes
13001	11:10 X vs. 33:40 X	-0.5187	-0.6626 to -0.3747	Yes
13002	11:10 X vs. 33:50 X	-0.3197	-0.4636 to -0.1757	Yes
13003	11:10 X vs. 34:0 X	-2.290	-2.434 to -2.146	Yes
13004	11:10 X vs. 34:1 X	-2.298	-2.442 to -2.154	Yes
13005	11:10 X vs. 34:10 X	-1.931	-2.075 to -1.787	Yes

2way ANOVA<br>Multiple comparisons		A<br>Data Set-A<br>Y	B<br>Data Set-B<br>Y	C<br>Data Set-C<br>Y
13006	11:10 X vs. 34:40 X	-0.5307	-0.6746 to -0.3867	Yes
13007	11:10 X vs. 34:50 X	-0.4687	-0.6126 to -0.3247	Yes
13008	11:10 X vs. 35:0 X	-2.639	-2.783 to -2.495	Yes
13009	11:10 X vs. 35:1 X	-2.405	-2.549 to -2.261	Yes
13010	11:10 X vs. 35:10 X	-1.893	-2.037 to -1.749	Yes
13011	11:10 X vs. 35:40 X	-0.5397	-0.6836 to -0.3957	Yes
13012	11:10 X vs. 35:50 X	-0.4813	-0.6253 to -0.3374	Yes
13013	11:10 X vs. 36:0 X	-2.846	-3.006 to -2.685	Yes
13014	11:10 X vs. 36:1 X	-2.541	-2.685 to -2.397	Yes
13015	11:10 X vs. 36:10 X	-1.898	-2.042 to -1.754	Yes
13016	11:10 X vs. 36:40 X	-0.5950	-0.7390 to -0.4510	Yes
13017	11:10 X vs. 36:50 X	-0.4447	-0.5886 to -0.3007	Yes
13018	11:10 X vs. 37:0 X	-3.229	-3.373 to -3.085	Yes
13019	11:10 X vs. 37:1 X	-2.611	-2.755 to -2.467	Yes
13020	11:10 X vs. 37:10 X	-1.900	-2.044 to -1.756	Yes
13021	11:10 X vs. 37:40 X	-0.6323	-0.7763 to -0.4884	Yes
13022	11:10 X vs. 37:50 X	-0.4533	-0.5973 to -0.3094	Yes
13023	11:10 X vs. 38:0 X	-3.174	-3.318 to -3.030	Yes
13024	11:10 X vs. 38:1 X	-2.730	-2.874 to -2.586	Yes
13025	11:10 X vs. 38:10 X	-2.175	-2.319 to -2.031	Yes
13026	11:10 X vs. 38:40 X	-0.6367	-0.7806 to -0.4927	Yes
13027	11:10 X vs. 38:50 X	-0.4573	-0.6013 to -0.3134	Yes
13028	11:10 X vs. 39:0 X	-3.405	-3.549 to -3.261	Yes
13029	11:10 X vs. 39:1 X	-3.297	-3.441 to -3.153	Yes
13030	11:10 X vs. 39:10 X	-2.320	-2.464 to -2.176	Yes
13031	11:10 X vs. 39:40 X	-0.6427	-0.7866 to -0.4987	Yes
13032	11:10 X vs. 39:50 X	-0.4883	-0.6323 to -0.3444	Yes
13033	11:10 X vs. 40:0 X	-3.446	-3.590 to -3.302	Yes
13034	11:10 X vs. 40:1 X	-3.372	-3.516 to -3.228	Yes
13035	11:10 X vs. 40:10 X	-2.577	-2.721 to -2.433	Yes
13036	11:10 X vs. 40:40 X	-0.6473	-0.7913 to -0.5034	Yes
13037	11:10 X vs. 40:50 X	-0.4947	-0.6386 to -0.3507	Yes
13038	11:10 X vs. 41:0 X	-3.487	-3.631 to -3.343	Yes
13039	11:10 X vs. 41:1 X	-3.486	-3.630 to -3.342	Yes
13040	11:10 X vs. 41:10 X	-2.630	-2.774 to -2.486	Yes
13041	11:10 X vs. 41:40 X	-0.5947	-0.7386 to -0.4507	Yes
13042	11:10 X vs. 41:50 X	-0.5073	-0.6513 to -0.3634	Yes
13043	11:10 X vs. 42:0 X	-3.629	-3.773 to -3.485	Yes
13044	11:10 X vs. 42:1 X	-3.648	-3.792 to -3.504	Yes
13045	11:10 X vs. 42:10 X	-2.631	-2.775 to -2.487	Yes
13046	11:10 X vs. 42:40 X	-0.6073	-0.7513 to -0.4634	Yes
13047	11:10 X vs. 42:50 X	-0.5107	-0.6546 to -0.3667	Yes
13048	11:10 X vs. 43:0 X	-3.719	-3.863 to -3.575	Yes
13049	11:10 X vs. 43:1 X	-3.633	-3.777 to -3.489	Yes
13050	11:10 X vs. 43:10 X	-3.349	-3.493 to -3.205	Yes

	2way ANOVA<br>Multiple comparisons	A<br>Data Set-A<br>Y	B<br>Data Set-B<br>Y	C<br>Data Set-C<br>Y
13051	11:**10 X** vs. 43:**40 X**	-0.6057	-0.7496 to -0.4617	Yes
13052	11:**10 X** vs. 43:**50 X**	-0.5110	-0.6550 to -0.3670	Yes
13053	11:**10 X** vs. 44:**0 X**	-3.743	-3.887 to -3.599	Yes
13054	11:**10 X** vs. 44:**1 X**	-3.632	-3.776 to -3.488	Yes
13055	11:**10 X** vs. 44:**10 X**	-3.412	-3.556 to -3.268	Yes
13056	11:**10 X** vs. 44:**40 X**	-0.6077	-0.7516 to -0.4637	Yes
13057	11:**10 X** vs. 44:**50 X**	-0.4433	-0.5873 to -0.2994	Yes
13058	11:**10 X** vs. 45:**0 X**	-3.607	-3.751 to -3.463	Yes
13059	11:**10 X** vs. 45:**1 X**	-3.409	-3.553 to -3.265	Yes
13060	11:**10 X** vs. 45:**10 X**	-3.292	-3.436 to -3.148	Yes
13061	11:**10 X** vs. 45:**40 X**	-0.6090	-0.7530 to -0.4650	Yes
13062	11:**10 X** vs. 45:**50 X**	-0.4403	-0.5843 to -0.2964	Yes
13063	11:**10 X** vs. 46:**0 X**	-3.412	-3.556 to -3.268	Yes
13064	11:**10 X** vs. 46:**1 X**	-3.379	-3.523 to -3.235	Yes
13065	11:**10 X** vs. 46:**10 X**	-3.287	-3.431 to -3.143	Yes
13066	11:**10 X** vs. 46:**40 X**	-0.6227	-0.7666 to -0.4787	Yes
13067	11:**10 X** vs. 46:**50 X**	-0.4333	-0.5773 to -0.2894	Yes
13068	11:**10 X** vs. 47:**0 X**	-3.387	-3.531 to -3.243	Yes
13069	11:**10 X** vs. 47:**1 X**	-3.291	-3.435 to -3.147	Yes
13070	11:**10 X** vs. 47:**10 X**	-3.273	-3.417 to -3.129	Yes
13071	11:**10 X** vs. 47:**40 X**	-0.6327	-0.7766 to -0.4887	Yes
13072	11:**10 X** vs. 47:**50 X**	-0.3913	-0.5353 to -0.2474	Yes
13073	11:**10 X** vs. 48:**0 X**	-3.375	-3.535 to -3.214	Yes
13074	11:**10 X** vs. 48:**1 X**	-3.278	-3.422 to -3.134	Yes
13075	11:**10 X** vs. 48:**10 X**	-3.273	-3.417 to -3.129	Yes
13076	11:**10 X** vs. 48:**40 X**	-0.6390	-0.7830 to -0.4950	Yes
13077	11:**10 X** vs. 48:**50 X**	-0.3987	-0.5426 to -0.2547	Yes
13078	11:**10 X** vs. 49:**0 X**	-3.375	-3.519 to -3.231	Yes
13079	11:**10 X** vs. 49:**1 X**	-3.278	-3.422 to -3.134	Yes
13080	11:**10 X** vs. 49:**10 X**	-3.273	-3.417 to -3.129	Yes
13081	11:**10 X** vs. 49:**40 X**	-0.6433	-0.7873 to -0.4994	Yes
13082	11:**10 X** vs. 49:**50 X**	-0.3980	-0.5420 to -0.2540	Yes
13083	11:**10 X** vs. 50:**0 X**	-3.375	-3.519 to -3.231	Yes
13084	11:**10 X** vs. 50:**1 X**	-3.278	-3.422 to -3.134	Yes
13085	11:**10 X** vs. 50:**10 X**	-3.273	-3.417 to -3.129	Yes
13086	11:**10 X** vs. 50:**40 X**	-0.6510	-0.7950 to -0.5070	Yes
13087	11:**10 X** vs. 50:**50 X**	-0.3977	-0.5416 to -0.2537	Yes
13088	11:**40 X** vs. 11:**50 X**	0.0120	-0.1320 to 0.1560	No
13089	11:**40 X** vs. 12:**0 X**	-0.007000	-0.1510 to 0.1370	No
13090	11:**40 X** vs. 12:**1 X**	0.01067	-0.1333 to 0.1546	No
13091	11:**40 X** vs. 12:**10 X**	-0.007000	-0.1510 to 0.1370	No
13092	11:**40 X** vs. 12:**40 X**	0.006667	-0.1373 to 0.1506	No
13093	11:**40 X** vs. 12:**50 X**	0.003333	-0.1406 to 0.1473	No
13094	11:**40 X** vs. 13:**0 X**	-0.001000	-0.1450 to 0.1430	No
13095	11:**40 X** vs. 13:**1 X**	0.01167	-0.1323 to 0.1556	No

	2way ANOVA<br>Multiple comparisons	A<br>Data Set-A<br>Y	B<br>Data Set-B<br>Y	C<br>Data Set-C<br>Y
13096	11:40 X vs. 13:10 X	-0.01033	-0.1543 to 0.1336	No
13097	11:40 X vs. 13:40 X	-0.006667	-0.1506 to 0.1373	No
13098	11:40 X vs. 13:50 X	-0.007667	-0.1516 to 0.1363	No
13099	11:40 X vs. 14:0 X	-0.009667	-0.1536 to 0.1343	No
13100	11:40 X vs. 14:1 X	-0.001333	-0.1453 to 0.1426	No
13101	11:40 X vs. 14:10 X	-0.01533	-0.1593 to 0.1286	No
13102	11:40 X vs. 14:40 X	-0.0120	-0.1560 to 0.1320	No
13103	11:40 X vs. 14:50 X	-0.0160	-0.1600 to 0.1280	No
13104	11:40 X vs. 15:0 X	-0.0220	-0.1660 to 0.1220	No
13105	11:40 X vs. 15:1 X	-0.0020	-0.1460 to 0.1420	No
13106	11:40 X vs. 15:10 X	-0.02933	-0.1733 to 0.1146	No
13107	11:40 X vs. 15:40 X	-0.01167	-0.1556 to 0.1323	No
13108	11:40 X vs. 15:50 X	-0.02933	-0.1733 to 0.1146	No
13109	11:40 X vs. 16:0 X	-0.1143	-0.2583 to 0.02963	No
13110	11:40 X vs. 16:1 X	-0.005333	-0.1493 to 0.1386	No
13111	11:40 X vs. 16:10 X	-0.0310	-0.1750 to 0.1130	No
13112	11:40 X vs. 16:40 X	-0.05033	-0.1943 to 0.09363	No
13113	11:40 X vs. 16:50 X	-0.09267	-0.2366 to 0.05129	No
13114	11:40 X vs. 17:0 X	-0.1670	-0.3110 to -0.02304	Yes
13115	11:40 X vs. 17:1 X	-0.08133	-0.2253 to 0.06263	No
13116	11:40 X vs. 17:10 X	-0.1380	-0.2820 to 0.005960	No
13117	11:40 X vs. 17:40 X	-0.06133	-0.2053 to 0.08263	No
13118	11:40 X vs. 17:50 X	-0.1067	-0.2506 to 0.03729	No
13119	11:40 X vs. 18:0 X	-0.2157	-0.3596 to -0.07171	Yes
13120	11:40 X vs. 18:1 X	-0.1240	-0.2680 to 0.01996	No
13121	11:40 X vs. 18:10 X	-0.2163	-0.3603 to -0.07237	Yes
13122	11:40 X vs. 18:40 X	-0.06567	-0.2096 to 0.07829	No
13123	11:40 X vs. 18:50 X	-0.1107	-0.2546 to 0.03329	No
13124	11:40 X vs. 19:0 X	-0.2507	-0.3946 to -0.1067	Yes
13125	11:40 X vs. 19:1 X	-0.2387	-0.3826 to -0.09471	Yes
13126	11:40 X vs. 19:10 X	-0.2363	-0.3803 to -0.09237	Yes
13127	11:40 X vs. 19:40 X	-0.09433	-0.2383 to 0.04963	No
13128	11:40 X vs. 19:50 X	-0.1053	-0.2493 to 0.03863	No
13129	11:40 X vs. 20:0 X	-0.3303	-0.4743 to -0.1864	Yes
13130	11:40 X vs. 20:1 X	-0.1497	-0.2936 to -0.005707	Yes
13131	11:40 X vs. 20:10 X	-0.2387	-0.3826 to -0.09471	Yes
13132	11:40 X vs. 20:40 X	-0.1060	-0.2500 to 0.03796	No
13133	11:40 X vs. 20:50 X	-0.1500	-0.2940 to -0.006040	Yes
13134	11:40 X vs. 21:0 X	-0.3537	-0.4976 to -0.2097	Yes
13135	11:40 X vs. 21:1 X	-0.3067	-0.4506 to -0.1627	Yes
13136	11:40 X vs. 21:10 X	-0.3037	-0.4476 to -0.1597	Yes
13137	11:40 X vs. 21:40 X	-0.1790	-0.3230 to -0.03504	Yes
13138	11:40 X vs. 21:50 X	-0.2693	-0.4133 to -0.1254	Yes
13139	11:40 X vs. 22:0 X	-0.3953	-0.5393 to -0.2514	Yes
13140	11:40 X vs. 22:1 X	-0.3900	-0.5340 to -0.2460	Yes

	2way ANOVA<br>Multiple comparisons	A<br>Data Set-A<br>Y	B<br>Data Set-B<br>Y	C<br>Data Set-C<br>Y
13141	11:40 X vs. 22:10 X	-0.3853	-0.5293 to -0.2414	Yes
13142	11:40 X vs. 22:40 X	-0.2857	-0.4296 to -0.1417	Yes
13143	11:40 X vs. 22:50 X	-0.3483	-0.4923 to -0.2044	Yes
13144	11:40 X vs. 23:0 X	-0.4607	-0.6046 to -0.3167	Yes
13145	11:40 X vs. 23:1 X	-0.6503	-0.8113 to -0.4894	Yes
13146	11:40 X vs. 23:10 X	-0.4000	-0.5440 to -0.2560	Yes
13147	11:40 X vs. 23:40 X	-0.2803	-0.4413 to -0.1194	Yes
13148	11:40 X vs. 23:50 X	-0.3143	-0.4583 to -0.1704	Yes
13149	11:40 X vs. 24:0 X	-0.5780	-0.7220 to -0.4340	Yes
13150	11:40 X vs. 24:1 X	-0.7343	-0.8953 to -0.5734	Yes
13151	11:40 X vs. 24:10 X	-0.5618	-0.7228 to -0.4009	Yes
13152	11:40 X vs. 24:40 X	-0.2967	-0.4406 to -0.1527	Yes
13153	11:40 X vs. 24:50 X	-0.2183	-0.3623 to -0.07437	Yes
13154	11:40 X vs. 25:0 X	-0.9153	-1.076 to -0.7544	Yes
13155	11:40 X vs. 25:1 X	-1.105	-1.266 to -0.9439	Yes
13156	11:40 X vs. 25:10 X	-0.4663	-0.6699 to -0.2627	Yes
13157	11:40 X vs. 25:40 X	-0.2987	-0.4426 to -0.1547	Yes
13158	11:40 X vs. 25:50 X	-0.2730	-0.4170 to -0.1290	Yes
13159	11:40 X vs. 26:0 X	-1.128	-1.289 to -0.9669	Yes
13160	11:40 X vs. 26:1 X	-1.241	-1.402 to -1.080	Yes
13161	11:40 X vs. 26:10 X	-0.9273	-1.088 to -0.7664	Yes
13162	11:40 X vs. 26:40 X	-0.3730	-0.5170 to -0.2290	Yes
13163	11:40 X vs. 26:50 X	-0.2333	-0.3773 to -0.08937	Yes
13164	11:40 X vs. 27:0 X	-1.316	-1.460 to -1.172	Yes
13165	11:40 X vs. 27:1 X	-1.406	-1.567 to -1.245	Yes
13166	11:40 X vs. 27:10 X	-1.218	-1.379 to -1.057	Yes
13167	11:40 X vs. 27:40 X	-0.3730	-0.5170 to -0.2290	Yes
13168	11:40 X vs. 27:50 X	-0.2327	-0.3766 to -0.08871	Yes
13169	11:40 X vs. 28:0 X	-1.265	-1.426 to -1.104	Yes
13170	11:40 X vs. 28:1 X	-1.306	-1.450 to -1.162	Yes
13171	11:40 X vs. 28:10 X	-1.107	-1.268 to -0.9464	Yes
13172	11:40 X vs. 28:40 X	-0.3853	-0.5293 to -0.2414	Yes
13173	11:40 X vs. 28:50 X	-0.2807	-0.4246 to -0.1367	Yes
13174	11:40 X vs. 29:0 X	-1.746	-1.907 to -1.585	Yes
13175	11:40 X vs. 29:1 X	-1.831	-1.992 to -1.670	Yes
13176	11:40 X vs. 29:10 X	-1.046	-1.207 to -0.8849	Yes
13177	11:40 X vs. 29:40 X	-0.4933	-0.6373 to -0.3494	Yes
13178	11:40 X vs. 29:50 X	-0.2820	-0.4260 to -0.1380	Yes
13179	11:40 X vs. 30:0 X	-1.461	-1.622 to -1.300	Yes
13180	11:40 X vs. 30:1 X	-1.782	-1.943 to -1.621	Yes
13181	11:40 X vs. 30:10 X	-1.120	-1.281 to -0.9594	Yes
13182	11:40 X vs. 30:40 X	-0.4203	-0.5643 to -0.2764	Yes
13183	11:40 X vs. 30:50 X	-0.3343	-0.4783 to -0.1904	Yes
13184	11:40 X vs. 31:0 X	-1.779	-1.940 to -1.618	Yes
13185	11:40 X vs. 31:1 X	-1.769	-1.930 to -1.608	Yes

2way ANOVA<br>Multiple comparisons	A<br>Data Set-A<br>Y	B<br>Data Set-B<br>Y	C<br>Data Set-C<br>Y
13186 11:40 X vs. 31:10 X	-1.252	-1.413 to -1.091	Yes
13187 11:40 X vs. 31:40 X	-0.5120	-0.6560 to -0.3680	Yes
13188 11:40 X vs. 31:50 X	-0.3133	-0.4573 to -0.1694	Yes
13189 11:40 X vs. 32:0 X	-2.370	-2.514 to -2.226	Yes
13190 11:40 X vs. 32:1 X	-1.826	-1.987 to -1.665	Yes
13191 11:40 X vs. 32:10 X	-1.682	-1.826 to -1.538	Yes
13192 11:40 X vs. 32:40 X	-0.5083	-0.6523 to -0.3644	Yes
13193 11:40 X vs. 32:50 X	-0.3130	-0.4570 to -0.1690	Yes
13194 11:40 X vs. 33:0 X	-2.374	-2.518 to -2.230	Yes
13195 11:40 X vs. 33:1 X	-2.189	-2.333 to -2.045	Yes
13196 11:40 X vs. 33:10 X	-1.849	-1.993 to -1.705	Yes
13197 11:40 X vs. 33:40 X	-0.5270	-0.6710 to -0.3830	Yes
13198 11:40 X vs. 33:50 X	-0.3280	-0.4720 to -0.1840	Yes
13199 11:40 X vs. 34:0 X	-2.299	-2.443 to -2.155	Yes
13200 11:40 X vs. 34:1 X	-2.306	-2.450 to -2.162	Yes
13201 11:40 X vs. 34:10 X	-1.939	-2.083 to -1.795	Yes
13202 11:40 X vs. 34:40 X	-0.5390	-0.6830 to -0.3950	Yes
13203 11:40 X vs. 34:50 X	-0.4770	-0.6210 to -0.3330	Yes
13204 11:40 X vs. 35:0 X	-2.647	-2.791 to -2.503	Yes
13205 11:40 X vs. 35:1 X	-2.413	-2.557 to -2.269	Yes
13206 11:40 X vs. 35:10 X	-1.901	-2.045 to -1.757	Yes
13207 11:40 X vs. 35:40 X	-0.5480	-0.6920 to -0.4040	Yes
13208 11:40 X vs. 35:50 X	-0.4897	-0.6336 to -0.3457	Yes
13209 11:40 X vs. 36:0 X	-2.854	-3.015 to -2.693	Yes
13210 11:40 X vs. 36:1 X	-2.550	-2.694 to -2.406	Yes
13211 11:40 X vs. 36:10 X	-1.907	-2.051 to -1.763	Yes
13212 11:40 X vs. 36:40 X	-0.6033	-0.7473 to -0.4594	Yes
13213 11:40 X vs. 36:50 X	-0.4530	-0.5970 to -0.3090	Yes
13214 11:40 X vs. 37:0 X	-3.238	-3.382 to -3.094	Yes
13215 11:40 X vs. 37:1 X	-2.620	-2.764 to -2.476	Yes
13216 11:40 X vs. 37:10 X	-1.908	-2.052 to -1.764	Yes
13217 11:40 X vs. 37:40 X	-0.6407	-0.7846 to -0.4967	Yes
13218 11:40 X vs. 37:50 X	-0.4617	-0.6056 to -0.3177	Yes
13219 11:40 X vs. 38:0 X	-3.183	-3.327 to -3.039	Yes
13220 11:40 X vs. 38:1 X	-2.738	-2.882 to -2.594	Yes
13221 11:40 X vs. 38:10 X	-2.184	-2.328 to -2.040	Yes
13222 11:40 X vs. 38:40 X	-0.6450	-0.7890 to -0.5010	Yes
13223 11:40 X vs. 38:50 X	-0.4657	-0.6096 to -0.3217	Yes
13224 11:40 X vs. 39:0 X	-3.413	-3.557 to -3.269	Yes
13225 11:40 X vs. 39:1 X	-3.305	-3.449 to -3.161	Yes
13226 11:40 X vs. 39:10 X	-2.329	-2.473 to -2.185	Yes
13227 11:40 X vs. 39:40 X	-0.6510	-0.7950 to -0.5070	Yes
13228 11:40 X vs. 39:50 X	-0.4967	-0.6406 to -0.3527	Yes
13229 11:40 X vs. 40:0 X	-3.454	-3.598 to -3.310	Yes
13230 11:40 X vs. 40:1 X	-3.380	-3.524 to -3.236	Yes

2way ANOVA<br>Multiple comparisons		A<br>Data Set-A<br>Y	B<br>Data Set-B<br>Y	C<br>Data Set-C<br>Y
13231	11:**40 X** vs. 40:**10 X**	-2.585	-2.729 to -2.441	Yes
13232	11:**40 X** vs. 40:**40 X**	-0.6557	-0.7996 to -0.5117	Yes
13233	11:**40 X** vs. 40:**50 X**	-0.5030	-0.6470 to -0.3590	Yes
13234	11:**40 X** vs. 41:**0 X**	-3.495	-3.639 to -3.351	Yes
13235	11:**40 X** vs. 41:**1 X**	-3.494	-3.638 to -3.350	Yes
13236	11:**40 X** vs. 41:**10 X**	-2.639	-2.783 to -2.495	Yes
13237	11:**40 X** vs. 41:**40 X**	-0.6030	-0.7470 to -0.4590	Yes
13238	11:**40 X** vs. 41:**50 X**	-0.5157	-0.6596 to -0.3717	Yes
13239	11:**40 X** vs. 42:**0 X**	-3.637	-3.781 to -3.493	Yes
13240	11:**40 X** vs. 42:**1 X**	-3.657	-3.801 to -3.513	Yes
13241	11:**40 X** vs. 42:**10 X**	-2.639	-2.783 to -2.495	Yes
13242	11:**40 X** vs. 42:**40 X**	-0.6157	-0.7596 to -0.4717	Yes
13243	11:**40 X** vs. 42:**50 X**	-0.5190	-0.6630 to -0.3750	Yes
13244	11:**40 X** vs. 43:**0 X**	-3.727	-3.871 to -3.583	Yes
13245	11:**40 X** vs. 43:**1 X**	-3.641	-3.785 to -3.497	Yes
13246	11:**40 X** vs. 43:**10 X**	-3.357	-3.501 to -3.213	Yes
13247	11:**40 X** vs. 43:**40 X**	-0.6140	-0.7580 to -0.4700	Yes
13248	11:**40 X** vs. 43:**50 X**	-0.5193	-0.6633 to -0.3754	Yes
13249	11:**40 X** vs. 44:**0 X**	-3.751	-3.895 to -3.607	Yes
13250	11:**40 X** vs. 44:**1 X**	-3.641	-3.785 to -3.497	Yes
13251	11:**40 X** vs. 44:**10 X**	-3.420	-3.564 to -3.276	Yes
13252	11:**40 X** vs. 44:**40 X**	-0.6160	-0.7600 to -0.4720	Yes
13253	11:**40 X** vs. 44:**50 X**	-0.4517	-0.5956 to -0.3077	Yes
13254	11:**40 X** vs. 45:**0 X**	-3.616	-3.760 to -3.472	Yes
13255	11:**40 X** vs. 45:**1 X**	-3.418	-3.562 to -3.274	Yes
13256	11:**40 X** vs. 45:**10 X**	-3.300	-3.444 to -3.156	Yes
13257	11:**40 X** vs. 45:**40 X**	-0.6173	-0.7613 to -0.4734	Yes
13258	11:**40 X** vs. 45:**50 X**	-0.4487	-0.5926 to -0.3047	Yes
13259	11:**40 X** vs. 46:**0 X**	-3.420	-3.564 to -3.276	Yes
13260	11:**40 X** vs. 46:**1 X**	-3.387	-3.531 to -3.243	Yes
13261	11:**40 X** vs. 46:**10 X**	-3.295	-3.439 to -3.151	Yes
13262	11:**40 X** vs. 46:**40 X**	-0.6310	-0.7750 to -0.4870	Yes
13263	11:**40 X** vs. 46:**50 X**	-0.4417	-0.5856 to -0.2977	Yes
13264	11:**40 X** vs. 47:**0 X**	-3.396	-3.540 to -3.252	Yes
13265	11:**40 X** vs. 47:**1 X**	-3.300	-3.444 to -3.156	Yes
13266	11:**40 X** vs. 47:**10 X**	-3.281	-3.425 to -3.137	Yes
13267	11:**40 X** vs. 47:**40 X**	-0.6410	-0.7850 to -0.4970	Yes
13268	11:**40 X** vs. 47:**50 X**	-0.3997	-0.5436 to -0.2557	Yes
13269	11:**40 X** vs. 48:**0 X**	-3.383	-3.544 to -3.222	Yes
13270	11:**40 X** vs. 48:**1 X**	-3.287	-3.431 to -3.143	Yes
13271	11:**40 X** vs. 48:**10 X**	-3.281	-3.425 to -3.137	Yes
13272	11:**40 X** vs. 48:**40 X**	-0.6473	-0.7913 to -0.5034	Yes
13273	11:**40 X** vs. 48:**50 X**	-0.4070	-0.5510 to -0.2630	Yes
13274	11:**40 X** vs. 49:**0 X**	-3.383	-3.527 to -3.239	Yes
13275	11:**40 X** vs. 49:**1 X**	-3.287	-3.431 to -3.143	Yes

	2way ANOVA<br>Multiple comparisons	A<br>Data Set-A<br>Y	B<br>Data Set-B<br>Y	C<br>Data Set-C<br>Y
13276	11:40 X vs. 49:10 X	-3.281	-3.425 to -3.137	Yes
13277	11:40 X vs. 49:40 X	-0.6517	-0.7956 to -0.5077	Yes
13278	11:40 X vs. 49:50 X	-0.4063	-0.5503 to -0.2624	Yes
13279	11:40 X vs. 50:0 X	-3.383	-3.527 to -3.239	Yes
13280	11:40 X vs. 50:1 X	-3.287	-3.431 to -3.143	Yes
13281	11:40 X vs. 50:10 X	-3.281	-3.425 to -3.137	Yes
13282	11:40 X vs. 50:40 X	-0.6593	-0.8033 to -0.5154	Yes
13283	11:40 X vs. 50:50 X	-0.4060	-0.5500 to -0.2620	Yes
13284	11:50 X vs. 12:0 X	-0.0190	-0.1630 to 0.1250	No
13285	11:50 X vs. 12:1 X	-0.001333	-0.1453 to 0.1426	No
13286	11:50 X vs. 12:10 X	-0.0190	-0.1630 to 0.1250	No
13287	11:50 X vs. 12:40 X	-0.005333	-0.1493 to 0.1386	No
13288	11:50 X vs. 12:50 X	-0.008667	-0.1526 to 0.1353	No
13289	11:50 X vs. 13:0 X	-0.0130	-0.1570 to 0.1310	No
13290	11:50 X vs. 13:1 X	-0.0003333	-0.1443 to 0.1436	No
13291	11:50 X vs. 13:10 X	-0.02233	-0.1663 to 0.1216	No
13292	11:50 X vs. 13:40 X	-0.01867	-0.1626 to 0.1253	No
13293	11:50 X vs. 13:50 X	-0.01967	-0.1636 to 0.1243	No
13294	11:50 X vs. 14:0 X	-0.02167	-0.1656 to 0.1223	No
13295	11:50 X vs. 14:1 X	-0.01333	-0.1573 to 0.1306	No
13296	11:50 X vs. 14:10 X	-0.02733	-0.1713 to 0.1166	No
13297	11:50 X vs. 14:40 X	-0.0240	-0.1680 to 0.1200	No
13298	11:50 X vs. 14:50 X	-0.0280	-0.1720 to 0.1160	No
13299	11:50 X vs. 15:0 X	-0.0340	-0.1780 to 0.1100	No
13300	11:50 X vs. 15:1 X	-0.0140	-0.1580 to 0.1300	No
13301	11:50 X vs. 15:10 X	-0.04133	-0.1853 to 0.1026	No
13302	11:50 X vs. 15:40 X	-0.02367	-0.1676 to 0.1203	No
13303	11:50 X vs. 15:50 X	-0.04133	-0.1853 to 0.1026	No
13304	11:50 X vs. 16:0 X	-0.1263	-0.2703 to 0.01763	No
13305	11:50 X vs. 16:1 X	-0.01733	-0.1613 to 0.1266	No
13306	11:50 X vs. 16:10 X	-0.0430	-0.1870 to 0.1010	No
13307	11:50 X vs. 16:40 X	-0.06233	-0.2063 to 0.08163	No
13308	11:50 X vs. 16:50 X	-0.1047	-0.2486 to 0.03929	No
13309	11:50 X vs. 17:0 X	-0.1790	-0.3230 to -0.03504	Yes
13310	11:50 X vs. 17:1 X	-0.09333	-0.2373 to 0.05063	No
13311	11:50 X vs. 17:10 X	-0.1500	-0.2940 to -0.006040	Yes
13312	11:50 X vs. 17:40 X	-0.07333	-0.2173 to 0.07063	No
13313	11:50 X vs. 17:50 X	-0.1187	-0.2626 to 0.02529	No
13314	11:50 X vs. 18:0 X	-0.2277	-0.3716 to -0.08371	Yes
13315	11:50 X vs. 18:1 X	-0.1360	-0.2800 to 0.007960	No
13316	11:50 X vs. 18:10 X	-0.2283	-0.3723 to -0.08437	Yes
13317	11:50 X vs. 18:40 X	-0.07767	-0.2216 to 0.06629	No
13318	11:50 X vs. 18:50 X	-0.1227	-0.2666 to 0.02129	No
13319	11:50 X vs. 19:0 X	-0.2627	-0.4066 to -0.1187	Yes
13320	11:50 X vs. 19:1 X	-0.2507	-0.3946 to -0.1067	Yes

	2way ANOVA<br>Multiple comparisons	A<br>Data Set-A<br>Y	B<br>Data Set-B<br>Y	C<br>Data Set-C<br>Y
13321	11:50 X vs. 19:10 X	-0.2483	-0.3923 to -0.1044	Yes
13322	11:50 X vs. 19:40 X	-0.1063	-0.2503 to 0.03763	No
13323	11:50 X vs. 19:50 X	-0.1173	-0.2613 to 0.02663	No
13324	11:50 X vs. 20:0 X	-0.3423	-0.4863 to -0.1984	Yes
13325	11:50 X vs. 20:1 X	-0.1617	-0.3056 to -0.01771	Yes
13326	11:50 X vs. 20:10 X	-0.2507	-0.3946 to -0.1067	Yes
13327	11:50 X vs. 20:40 X	-0.1180	-0.2620 to 0.02596	No
13328	11:50 X vs. 20:50 X	-0.1620	-0.3060 to -0.01804	Yes
13329	11:50 X vs. 21:0 X	-0.3657	-0.5096 to -0.2217	Yes
13330	11:50 X vs. 21:1 X	-0.3187	-0.4626 to -0.1747	Yes
13331	11:50 X vs. 21:10 X	-0.3157	-0.4596 to -0.1717	Yes
13332	11:50 X vs. 21:40 X	-0.1910	-0.3350 to -0.04704	Yes
13333	11:50 X vs. 21:50 X	-0.2813	-0.4253 to -0.1374	Yes
13334	11:50 X vs. 22:0 X	-0.4073	-0.5513 to -0.2634	Yes
13335	11:50 X vs. 22:1 X	-0.4020	-0.5460 to -0.2580	Yes
13336	11:50 X vs. 22:10 X	-0.3973	-0.5413 to -0.2534	Yes
13337	11:50 X vs. 22:40 X	-0.2977	-0.4416 to -0.1537	Yes
13338	11:50 X vs. 22:50 X	-0.3603	-0.5043 to -0.2164	Yes
13339	11:50 X vs. 23:0 X	-0.4727	-0.6166 to -0.3287	Yes
13340	11:50 X vs. 23:1 X	-0.6623	-0.8233 to -0.5014	Yes
13341	11:50 X vs. 23:10 X	-0.4120	-0.5560 to -0.2680	Yes
13342	11:50 X vs. 23:40 X	-0.2923	-0.4533 to -0.1314	Yes
13343	11:50 X vs. 23:50 X	-0.3263	-0.4703 to -0.1824	Yes
13344	11:50 X vs. 24:0 X	-0.5900	-0.7340 to -0.4460	Yes
13345	11:50 X vs. 24:1 X	-0.7463	-0.9073 to -0.5854	Yes
13346	11:50 X vs. 24:10 X	-0.5738	-0.7348 to -0.4129	Yes
13347	11:50 X vs. 24:40 X	-0.3087	-0.4526 to -0.1647	Yes
13348	11:50 X vs. 24:50 X	-0.2303	-0.3743 to -0.08637	Yes
13349	11:50 X vs. 25:0 X	-0.9273	-1.088 to -0.7664	Yes
13350	11:50 X vs. 25:1 X	-1.117	-1.278 to -0.9559	Yes
13351	11:50 X vs. 25:10 X	-0.4783	-0.6819 to -0.2747	Yes
13352	11:50 X vs. 25:40 X	-0.3107	-0.4546 to -0.1667	Yes
13353	11:50 X vs. 25:50 X	-0.2850	-0.4290 to -0.1410	Yes
13354	11:50 X vs. 26:0 X	-1.140	-1.301 to -0.9789	Yes
13355	11:50 X vs. 26:1 X	-1.253	-1.414 to -1.092	Yes
13356	11:50 X vs. 26:10 X	-0.9393	-1.100 to -0.7784	Yes
13357	11:50 X vs. 26:40 X	-0.3850	-0.5290 to -0.2410	Yes
13358	11:50 X vs. 26:50 X	-0.2453	-0.3893 to -0.1014	Yes
13359	11:50 X vs. 27:0 X	-1.328	-1.472 to -1.184	Yes
13360	11:50 X vs. 27:1 X	-1.418	-1.579 to -1.257	Yes
13361	11:50 X vs. 27:10 X	-1.230	-1.391 to -1.069	Yes
13362	11:50 X vs. 27:40 X	-0.3850	-0.5290 to -0.2410	Yes
13363	11:50 X vs. 27:50 X	-0.2447	-0.3886 to -0.1007	Yes
13364	11:50 X vs. 28:0 X	-1.277	-1.438 to -1.116	Yes
13365	11:50 X vs. 28:1 X	-1.318	-1.462 to -1.174	Yes

	2way ANOVA<br>Multiple comparisons	A<br>Data Set-A<br>Y	B<br>Data Set-B<br>Y	C<br>Data Set-C<br>Y
13366	11:50 X vs. 28:10 X	-1.119	-1.280 to -0.9584	Yes
13367	11:50 X vs. 28:40 X	-0.3973	-0.5413 to -0.2534	Yes
13368	11:50 X vs. 28:50 X	-0.2927	-0.4366 to -0.1487	Yes
13369	11:50 X vs. 29:0 X	-1.758	-1.919 to -1.597	Yes
13370	11:50 X vs. 29:1 X	-1.843	-2.004 to -1.682	Yes
13371	11:50 X vs. 29:10 X	-1.058	-1.219 to -0.8969	Yes
13372	11:50 X vs. 29:40 X	-0.5053	-0.6493 to -0.3614	Yes
13373	11:50 X vs. 29:50 X	-0.2940	-0.4380 to -0.1500	Yes
13374	11:50 X vs. 30:0 X	-1.473	-1.634 to -1.312	Yes
13375	11:50 X vs. 30:1 X	-1.794	-1.955 to -1.633	Yes
13376	11:50 X vs. 30:10 X	-1.132	-1.293 to -0.9714	Yes
13377	11:50 X vs. 30:40 X	-0.4323	-0.5763 to -0.2884	Yes
13378	11:50 X vs. 30:50 X	-0.3463	-0.4903 to -0.2024	Yes
13379	11:50 X vs. 31:0 X	-1.791	-1.952 to -1.630	Yes
13380	11:50 X vs. 31:1 X	-1.781	-1.942 to -1.620	Yes
13381	11:50 X vs. 31:10 X	-1.264	-1.425 to -1.103	Yes
13382	11:50 X vs. 31:40 X	-0.5240	-0.6680 to -0.3800	Yes
13383	11:50 X vs. 31:50 X	-0.3253	-0.4693 to -0.1814	Yes
13384	11:50 X vs. 32:0 X	-2.382	-2.526 to -2.238	Yes
13385	11:50 X vs. 32:1 X	-1.838	-1.999 to -1.677	Yes
13386	11:50 X vs. 32:10 X	-1.694	-1.838 to -1.550	Yes
13387	11:50 X vs. 32:40 X	-0.5203	-0.6643 to -0.3764	Yes
13388	11:50 X vs. 32:50 X	-0.3250	-0.4690 to -0.1810	Yes
13389	11:50 X vs. 33:0 X	-2.386	-2.530 to -2.242	Yes
13390	11:50 X vs. 33:1 X	-2.201	-2.345 to -2.057	Yes
13391	11:50 X vs. 33:10 X	-1.861	-2.005 to -1.717	Yes
13392	11:50 X vs. 33:40 X	-0.5390	-0.6830 to -0.3950	Yes
13393	11:50 X vs. 33:50 X	-0.3400	-0.4840 to -0.1960	Yes
13394	11:50 X vs. 34:0 X	-2.311	-2.455 to -2.167	Yes
13395	11:50 X vs. 34:1 X	-2.318	-2.462 to -2.174	Yes
13396	11:50 X vs. 34:10 X	-1.951	-2.095 to -1.807	Yes
13397	11:50 X vs. 34:40 X	-0.5510	-0.6950 to -0.4070	Yes
13398	11:50 X vs. 34:50 X	-0.4890	-0.6330 to -0.3450	Yes
13399	11:50 X vs. 35:0 X	-2.659	-2.803 to -2.515	Yes
13400	11:50 X vs. 35:1 X	-2.425	-2.569 to -2.281	Yes
13401	11:50 X vs. 35:10 X	-1.913	-2.057 to -1.769	Yes
13402	11:50 X vs. 35:40 X	-0.5600	-0.7040 to -0.4160	Yes
13403	11:50 X vs. 35:50 X	-0.5017	-0.6456 to -0.3577	Yes
13404	11:50 X vs. 36:0 X	-2.866	-3.027 to -2.705	Yes
13405	11:50 X vs. 36:1 X	-2.562	-2.706 to -2.418	Yes
13406	11:50 X vs. 36:10 X	-1.919	-2.063 to -1.775	Yes
13407	11:50 X vs. 36:40 X	-0.6153	-0.7593 to -0.4714	Yes
13408	11:50 X vs. 36:50 X	-0.4650	-0.6090 to -0.3210	Yes
13409	11:50 X vs. 37:0 X	-3.250	-3.394 to -3.106	Yes
13410	11:50 X vs. 37:1 X	-2.632	-2.776 to -2.488	Yes

	2way ANOVA Multiple comparisons	A Data Set-A Y	B Data Set-B Y	C Data Set-C Y
13411	11:**50 X** vs. 37:**10 X**	-1.920	-2.064 to -1.776	Yes
13412	11:**50 X** vs. 37:**40 X**	-0.6527	-0.7966 to -0.5087	Yes
13413	11:**50 X** vs. 37:**50 X**	-0.4737	-0.6176 to -0.3297	Yes
13414	11:**50 X** vs. 38:**0 X**	-3.195	-3.339 to -3.051	Yes
13415	11:**50 X** vs. 38:**1 X**	-2.750	-2.894 to -2.606	Yes
13416	11:**50 X** vs. 38:**10 X**	-2.196	-2.340 to -2.052	Yes
13417	11:**50 X** vs. 38:**40 X**	-0.6570	-0.8010 to -0.5130	Yes
13418	11:**50 X** vs. 38:**50 X**	-0.4777	-0.6216 to -0.3337	Yes
13419	11:**50 X** vs. 39:**0 X**	-3.425	-3.569 to -3.281	Yes
13420	11:**50 X** vs. 39:**1 X**	-3.317	-3.461 to -3.173	Yes
13421	11:**50 X** vs. 39:**10 X**	-2.341	-2.485 to -2.197	Yes
13422	11:**50 X** vs. 39:**40 X**	-0.6630	-0.8070 to -0.5190	Yes
13423	11:**50 X** vs. 39:**50 X**	-0.5087	-0.6526 to -0.3647	Yes
13424	11:**50 X** vs. 40:**0 X**	-3.466	-3.610 to -3.322	Yes
13425	11:**50 X** vs. 40:**1 X**	-3.392	-3.536 to -3.248	Yes
13426	11:**50 X** vs. 40:**10 X**	-2.597	-2.741 to -2.453	Yes
13427	11:**50 X** vs. 40:**40 X**	-0.6677	-0.8116 to -0.5237	Yes
13428	11:**50 X** vs. 40:**50 X**	-0.5150	-0.6590 to -0.3710	Yes
13429	11:**50 X** vs. 41:**0 X**	-3.507	-3.651 to -3.363	Yes
13430	11:**50 X** vs. 41:**1 X**	-3.506	-3.650 to -3.362	Yes
13431	11:**50 X** vs. 41:**10 X**	-2.651	-2.795 to -2.507	Yes
13432	11:**50 X** vs. 41:**40 X**	-0.6150	-0.7590 to -0.4710	Yes
13433	11:**50 X** vs. 41:**50 X**	-0.5277	-0.6716 to -0.3837	Yes
13434	11:**50 X** vs. 42:**0 X**	-3.649	-3.793 to -3.505	Yes
13435	11:**50 X** vs. 42:**1 X**	-3.669	-3.813 to -3.525	Yes
13436	11:**50 X** vs. 42:**10 X**	-2.651	-2.795 to -2.507	Yes
13437	11:**50 X** vs. 42:**40 X**	-0.6277	-0.7716 to -0.4837	Yes
13438	11:**50 X** vs. 42:**50 X**	-0.5310	-0.6750 to -0.3870	Yes
13439	11:**50 X** vs. 43:**0 X**	-3.739	-3.883 to -3.595	Yes
13440	11:**50 X** vs. 43:**1 X**	-3.653	-3.797 to -3.509	Yes
13441	11:**50 X** vs. 43:**10 X**	-3.369	-3.513 to -3.225	Yes
13442	11:**50 X** vs. 43:**40 X**	-0.6260	-0.7700 to -0.4820	Yes
13443	11:**50 X** vs. 43:**50 X**	-0.5313	-0.6753 to -0.3874	Yes
13444	11:**50 X** vs. 44:**0 X**	-3.763	-3.907 to -3.619	Yes
13445	11:**50 X** vs. 44:**1 X**	-3.653	-3.797 to -3.509	Yes
13446	11:**50 X** vs. 44:**10 X**	-3.432	-3.576 to -3.288	Yes
13447	11:**50 X** vs. 44:**40 X**	-0.6280	-0.7720 to -0.4840	Yes
13448	11:**50 X** vs. 44:**50 X**	-0.4637	-0.6076 to -0.3197	Yes
13449	11:**50 X** vs. 45:**0 X**	-3.628	-3.772 to -3.484	Yes
13450	11:**50 X** vs. 45:**1 X**	-3.430	-3.574 to -3.286	Yes
13451	11:**50 X** vs. 45:**10 X**	-3.312	-3.456 to -3.168	Yes
13452	11:**50 X** vs. 45:**40 X**	-0.6293	-0.7733 to -0.4854	Yes
13453	11:**50 X** vs. 45:**50 X**	-0.4607	-0.6046 to -0.3167	Yes
13454	11:**50 X** vs. 46:**0 X**	-3.432	-3.576 to -3.288	Yes
13455	11:**50 X** vs. 46:**1 X**	-3.399	-3.543 to -3.255	Yes

	2way ANOVA<br>Multiple comparisons	A<br>Data Set-A<br>Y	B<br>Data Set-B<br>Y	C<br>Data Set-C<br>Y
13456	11:**50 X** vs. 46:**10 X**	-3.307	-3.451 to -3.163	Yes
13457	11:**50 X** vs. 46:**40 X**	-0.6430	-0.7870 to -0.4990	Yes
13458	11:**50 X** vs. 46:**50 X**	-0.4537	-0.5976 to -0.3097	Yes
13459	11:**50 X** vs. 47:**0 X**	-3.408	-3.552 to -3.264	Yes
13460	11:**50 X** vs. 47:**1 X**	-3.312	-3.456 to -3.168	Yes
13461	11:**50 X** vs. 47:**10 X**	-3.293	-3.437 to -3.149	Yes
13462	11:**50 X** vs. 47:**40 X**	-0.6530	-0.7970 to -0.5090	Yes
13463	11:**50 X** vs. 47:**50 X**	-0.4117	-0.5556 to -0.2677	Yes
13464	11:**50 X** vs. 48:**0 X**	-3.395	-3.556 to -3.234	Yes
13465	11:**50 X** vs. 48:**1 X**	-3.299	-3.443 to -3.155	Yes
13466	11:**50 X** vs. 48:**10 X**	-3.293	-3.437 to -3.149	Yes
13467	11:**50 X** vs. 48:**40 X**	-0.6593	-0.8033 to -0.5154	Yes
13468	11:**50 X** vs. 48:**50 X**	-0.4190	-0.5630 to -0.2750	Yes
13469	11:**50 X** vs. 49:**0 X**	-3.395	-3.539 to -3.251	Yes
13470	11:**50 X** vs. 49:**1 X**	-3.299	-3.443 to -3.155	Yes
13471	11:**50 X** vs. 49:**10 X**	-3.293	-3.437 to -3.149	Yes
13472	11:**50 X** vs. 49:**40 X**	-0.6637	-0.8076 to -0.5197	Yes
13473	11:**50 X** vs. 49:**50 X**	-0.4183	-0.5623 to -0.2744	Yes
13474	11:**50 X** vs. 50:**0 X**	-3.395	-3.539 to -3.251	Yes
13475	11:**50 X** vs. 50:**1 X**	-3.299	-3.443 to -3.155	Yes
13476	11:**50 X** vs. 50:**10 X**	-3.293	-3.437 to -3.149	Yes
13477	11:**50 X** vs. 50:**40 X**	-0.6713	-0.8153 to -0.5274	Yes
13478	11:**50 X** vs. 50:**50 X**	-0.4180	-0.5620 to -0.2740	Yes
13479	12:**0 X** vs. 12:**1 X**	0.01767	-0.1263 to 0.1616	No
13480	12:**0 X** vs. 12:**10 X**	1.242e-009	-0.1440 to 0.1440	No
13481	12:**0 X** vs. 12:**40 X**	0.01367	-0.1303 to 0.1576	No
13482	12:**0 X** vs. 12:**50 X**	0.01033	-0.1336 to 0.1543	No
13483	12:**0 X** vs. 13:**0 X**	0.006000	-0.1380 to 0.1500	No
13484	12:**0 X** vs. 13:**1 X**	0.01867	-0.1253 to 0.1626	No
13485	12:**0 X** vs. 13:**10 X**	-0.003333	-0.1473 to 0.1406	No
13486	12:**0 X** vs. 13:**40 X**	0.0003333	-0.1436 to 0.1443	No
13487	12:**0 X** vs. 13:**50 X**	-0.0006667	-0.1446 to 0.1433	No
13488	12:**0 X** vs. 14:**0 X**	-0.002667	-0.1466 to 0.1413	No
13489	12:**0 X** vs. 14:**1 X**	0.005667	-0.1383 to 0.1496	No
13490	12:**0 X** vs. 14:**10 X**	-0.008333	-0.1523 to 0.1356	No
13491	12:**0 X** vs. 14:**40 X**	-0.0050	-0.1490 to 0.1390	No
13492	12:**0 X** vs. 14:**50 X**	-0.0090	-0.1530 to 0.1350	No
13493	12:**0 X** vs. 15:**0 X**	-0.0150	-0.1590 to 0.1290	No
13494	12:**0 X** vs. 15:**1 X**	0.005000	-0.1390 to 0.1490	No
13495	12:**0 X** vs. 15:**10 X**	-0.02233	-0.1663 to 0.1216	No
13496	12:**0 X** vs. 15:**40 X**	-0.004667	-0.1486 to 0.1393	No
13497	12:**0 X** vs. 15:**50 X**	-0.02233	-0.1663 to 0.1216	No
13498	12:**0 X** vs. 16:**0 X**	-0.1073	-0.2513 to 0.03663	No
13499	12:**0 X** vs. 16:**1 X**	0.001667	-0.1423 to 0.1456	No
13500	12:**0 X** vs. 16:**10 X**	-0.0240	-0.1680 to 0.1200	No

	2way ANOVA<br>Multiple comparisons	A<br>Data Set-A<br>Y	B<br>Data Set-B<br>Y	C<br>Data Set-C<br>Y
13501	12:**0 X** vs. 16:**40 X**	-0.04333	-0.1873 to 0.1006	No
13502	12:**0 X** vs. 16:**50 X**	-0.08567	-0.2296 to 0.05829	No
13503	12:**0 X** vs. 17:**0 X**	-0.1600	-0.3040 to -0.01604	Yes
13504	12:**0 X** vs. 17:**1 X**	-0.07433	-0.2183 to 0.06963	No
13505	12:**0 X** vs. 17:**10 X**	-0.1310	-0.2750 to 0.01296	No
13506	12:**0 X** vs. 17:**40 X**	-0.05433	-0.1983 to 0.08963	No
13507	12:**0 X** vs. 17:**50 X**	-0.09967	-0.2436 to 0.04429	No
13508	12:**0 X** vs. 18:**0 X**	-0.2087	-0.3526 to -0.06471	Yes
13509	12:**0 X** vs. 18:**1 X**	-0.1170	-0.2610 to 0.02696	No
13510	12:**0 X** vs. 18:**10 X**	-0.2093	-0.3533 to -0.06537	Yes
13511	12:**0 X** vs. 18:**40 X**	-0.05867	-0.2026 to 0.08529	No
13512	12:**0 X** vs. 18:**50 X**	-0.1037	-0.2476 to 0.04029	No
13513	12:**0 X** vs. 19:**0 X**	-0.2437	-0.3876 to -0.09971	Yes
13514	12:**0 X** vs. 19:**1 X**	-0.2317	-0.3756 to -0.08771	Yes
13515	12:**0 X** vs. 19:**10 X**	-0.2293	-0.3733 to -0.08537	Yes
13516	12:**0 X** vs. 19:**40 X**	-0.08733	-0.2313 to 0.05663	No
13517	12:**0 X** vs. 19:**50 X**	-0.09833	-0.2423 to 0.04563	No
13518	12:**0 X** vs. 20:**0 X**	-0.3233	-0.4673 to -0.1794	Yes
13519	12:**0 X** vs. 20:**1 X**	-0.1427	-0.2866 to 0.001293	No
13520	12:**0 X** vs. 20:**10 X**	-0.2317	-0.3756 to -0.08771	Yes
13521	12:**0 X** vs. 20:**40 X**	-0.0990	-0.2430 to 0.04496	No
13522	12:**0 X** vs. 20:**50 X**	-0.1430	-0.2870 to 0.0009598	No
13523	12:**0 X** vs. 21:**0 X**	-0.3467	-0.4906 to -0.2027	Yes
13524	12:**0 X** vs. 21:**1 X**	-0.2997	-0.4436 to -0.1557	Yes
13525	12:**0 X** vs. 21:**10 X**	-0.2967	-0.4406 to -0.1527	Yes
13526	12:**0 X** vs. 21:**40 X**	-0.1720	-0.3160 to -0.02804	Yes
13527	12:**0 X** vs. 21:**50 X**	-0.2623	-0.4063 to -0.1184	Yes
13528	12:**0 X** vs. 22:**0 X**	-0.3883	-0.5323 to -0.2444	Yes
13529	12:**0 X** vs. 22:**1 X**	-0.3830	-0.5270 to -0.2390	Yes
13530	12:**0 X** vs. 22:**10 X**	-0.3783	-0.5223 to -0.2344	Yes
13531	12:**0 X** vs. 22:**40 X**	-0.2787	-0.4226 to -0.1347	Yes
13532	12:**0 X** vs. 22:**50 X**	-0.3413	-0.4853 to -0.1974	Yes
13533	12:**0 X** vs. 23:**0 X**	-0.4537	-0.5976 to -0.3097	Yes
13534	12:**0 X** vs. 23:**1 X**	-0.6433	-0.8043 to -0.4824	Yes
13535	12:**0 X** vs. 23:**10 X**	-0.3930	-0.5370 to -0.2490	Yes
13536	12:**0 X** vs. 23:**40 X**	-0.2733	-0.4343 to -0.1124	Yes
13537	12:**0 X** vs. 23:**50 X**	-0.3073	-0.4513 to -0.1634	Yes
13538	12:**0 X** vs. 24:**0 X**	-0.5710	-0.7150 to -0.4270	Yes
13539	12:**0 X** vs. 24:**1 X**	-0.7273	-0.8883 to -0.5664	Yes
13540	12:**0 X** vs. 24:**10 X**	-0.5548	-0.7158 to -0.3939	Yes
13541	12:**0 X** vs. 24:**40 X**	-0.2897	-0.4336 to -0.1457	Yes
13542	12:**0 X** vs. 24:**50 X**	-0.2113	-0.3553 to -0.06737	Yes
13543	12:**0 X** vs. 25:**0 X**	-0.9083	-1.069 to -0.7474	Yes
13544	12:**0 X** vs. 25:**1 X**	-1.098	-1.259 to -0.9369	Yes
13545	12:**0 X** vs. 25:**10 X**	-0.4593	-0.6629 to -0.2557	Yes

2way ANOVA<br>Multiple comparisons	A<br>Data Set-A<br>Y	B<br>Data Set-B<br>Y	C<br>Data Set-C<br>Y
13546 12:0 X vs. 25:40 X	-0.2917	-0.4356 to -0.1477	Yes
13547 12:0 X vs. 25:50 X	-0.2660	-0.4100 to -0.1220	Yes
13548 12:0 X vs. 26:0 X	-1.121	-1.282 to -0.9599	Yes
13549 12:0 X vs. 26:1 X	-1.234	-1.395 to -1.073	Yes
13550 12:0 X vs. 26:10 X	-0.9203	-1.081 to -0.7594	Yes
13551 12:0 X vs. 26:40 X	-0.3660	-0.5100 to -0.2220	Yes
13552 12:0 X vs. 26:50 X	-0.2263	-0.3703 to -0.08237	Yes
13553 12:0 X vs. 27:0 X	-1.309	-1.453 to -1.165	Yes
13554 12:0 X vs. 27:1 X	-1.399	-1.560 to -1.238	Yes
13555 12:0 X vs. 27:10 X	-1.211	-1.372 to -1.050	Yes
13556 12:0 X vs. 27:40 X	-0.3660	-0.5100 to -0.2220	Yes
13557 12:0 X vs. 27:50 X	-0.2257	-0.3696 to -0.08171	Yes
13558 12:0 X vs. 28:0 X	-1.258	-1.419 to -1.097	Yes
13559 12:0 X vs. 28:1 X	-1.299	-1.443 to -1.155	Yes
13560 12:0 X vs. 28:10 X	-1.100	-1.261 to -0.9394	Yes
13561 12:0 X vs. 28:40 X	-0.3783	-0.5223 to -0.2344	Yes
13562 12:0 X vs. 28:50 X	-0.2737	-0.4176 to -0.1297	Yes
13563 12:0 X vs. 29:0 X	-1.739	-1.900 to -1.578	Yes
13564 12:0 X vs. 29:1 X	-1.824	-1.985 to -1.663	Yes
13565 12:0 X vs. 29:10 X	-1.039	-1.200 to -0.8779	Yes
13566 12:0 X vs. 29:40 X	-0.4863	-0.6303 to -0.3424	Yes
13567 12:0 X vs. 29:50 X	-0.2750	-0.4190 to -0.1310	Yes
13568 12:0 X vs. 30:0 X	-1.454	-1.615 to -1.293	Yes
13569 12:0 X vs. 30:1 X	-1.775	-1.936 to -1.614	Yes
13570 12:0 X vs. 30:10 X	-1.113	-1.274 to -0.9524	Yes
13571 12:0 X vs. 30:40 X	-0.4133	-0.5573 to -0.2694	Yes
13572 12:0 X vs. 30:50 X	-0.3273	-0.4713 to -0.1834	Yes
13573 12:0 X vs. 31:0 X	-1.772	-1.933 to -1.611	Yes
13574 12:0 X vs. 31:1 X	-1.762	-1.923 to -1.601	Yes
13575 12:0 X vs. 31:10 X	-1.245	-1.406 to -1.084	Yes
13576 12:0 X vs. 31:40 X	-0.5050	-0.6490 to -0.3610	Yes
13577 12:0 X vs. 31:50 X	-0.3063	-0.4503 to -0.1624	Yes
13578 12:0 X vs. 32:0 X	-2.363	-2.507 to -2.219	Yes
13579 12:0 X vs. 32:1 X	-1.819	-1.980 to -1.658	Yes
13580 12:0 X vs. 32:10 X	-1.675	-1.819 to -1.531	Yes
13581 12:0 X vs. 32:40 X	-0.5013	-0.6453 to -0.3574	Yes
13582 12:0 X vs. 32:50 X	-0.3060	-0.4500 to -0.1620	Yes
13583 12:0 X vs. 33:0 X	-2.367	-2.511 to -2.223	Yes
13584 12:0 X vs. 33:1 X	-2.182	-2.326 to -2.038	Yes
13585 12:0 X vs. 33:10 X	-1.842	-1.986 to -1.698	Yes
13586 12:0 X vs. 33:40 X	-0.5200	-0.6640 to -0.3760	Yes
13587 12:0 X vs. 33:50 X	-0.3210	-0.4650 to -0.1770	Yes
13588 12:0 X vs. 34:0 X	-2.292	-2.436 to -2.148	Yes
13589 12:0 X vs. 34:1 X	-2.299	-2.443 to -2.155	Yes
13590 12:0 X vs. 34:10 X	-1.932	-2.076 to -1.788	Yes

	2way ANOVA<br>Multiple comparisons	A<br>Data Set-A<br>Y	B<br>Data Set-B<br>Y	C<br>Data Set-C<br>Y
13591	12:0 X vs. 34:40 X	-0.5320	-0.6760 to -0.3880	Yes
13592	12:0 X vs. 34:50 X	-0.4700	-0.6140 to -0.3260	Yes
13593	12:0 X vs. 35:0 X	-2.640	-2.784 to -2.496	Yes
13594	12:0 X vs. 35:1 X	-2.406	-2.550 to -2.262	Yes
13595	12:0 X vs. 35:10 X	-1.894	-2.038 to -1.750	Yes
13596	12:0 X vs. 35:40 X	-0.5410	-0.6850 to -0.3970	Yes
13597	12:0 X vs. 35:50 X	-0.4827	-0.6266 to -0.3387	Yes
13598	12:0 X vs. 36:0 X	-2.847	-3.008 to -2.686	Yes
13599	12:0 X vs. 36:1 X	-2.543	-2.687 to -2.399	Yes
13600	12:0 X vs. 36:10 X	-1.900	-2.044 to -1.756	Yes
13601	12:0 X vs. 36:40 X	-0.5963	-0.7403 to -0.4524	Yes
13602	12:0 X vs. 36:50 X	-0.4460	-0.5900 to -0.3020	Yes
13603	12:0 X vs. 37:0 X	-3.231	-3.375 to -3.087	Yes
13604	12:0 X vs. 37:1 X	-2.613	-2.757 to -2.469	Yes
13605	12:0 X vs. 37:10 X	-1.901	-2.045 to -1.757	Yes
13606	12:0 X vs. 37:40 X	-0.6337	-0.7776 to -0.4897	Yes
13607	12:0 X vs. 37:50 X	-0.4547	-0.5986 to -0.3107	Yes
13608	12:0 X vs. 38:0 X	-3.176	-3.320 to -3.032	Yes
13609	12:0 X vs. 38:1 X	-2.731	-2.875 to -2.587	Yes
13610	12:0 X vs. 38:10 X	-2.177	-2.321 to -2.033	Yes
13611	12:0 X vs. 38:40 X	-0.6380	-0.7820 to -0.4940	Yes
13612	12:0 X vs. 38:50 X	-0.4587	-0.6026 to -0.3147	Yes
13613	12:0 X vs. 39:0 X	-3.406	-3.550 to -3.262	Yes
13614	12:0 X vs. 39:1 X	-3.298	-3.442 to -3.154	Yes
13615	12:0 X vs. 39:10 X	-2.322	-2.466 to -2.178	Yes
13616	12:0 X vs. 39:40 X	-0.6440	-0.7880 to -0.5000	Yes
13617	12:0 X vs. 39:50 X	-0.4897	-0.6336 to -0.3457	Yes
13618	12:0 X vs. 40:0 X	-3.447	-3.591 to -3.303	Yes
13619	12:0 X vs. 40:1 X	-3.373	-3.517 to -3.229	Yes
13620	12:0 X vs. 40:10 X	-2.578	-2.722 to -2.434	Yes
13621	12:0 X vs. 40:40 X	-0.6487	-0.7926 to -0.5047	Yes
13622	12:0 X vs. 40:50 X	-0.4960	-0.6400 to -0.3520	Yes
13623	12:0 X vs. 41:0 X	-3.488	-3.632 to -3.344	Yes
13624	12:0 X vs. 41:1 X	-3.487	-3.631 to -3.343	Yes
13625	12:0 X vs. 41:10 X	-2.632	-2.776 to -2.488	Yes
13626	12:0 X vs. 41:40 X	-0.5960	-0.7400 to -0.4520	Yes
13627	12:0 X vs. 41:50 X	-0.5087	-0.6526 to -0.3647	Yes
13628	12:0 X vs. 42:0 X	-3.630	-3.774 to -3.486	Yes
13629	12:0 X vs. 42:1 X	-3.650	-3.794 to -3.506	Yes
13630	12:0 X vs. 42:10 X	-2.632	-2.776 to -2.488	Yes
13631	12:0 X vs. 42:40 X	-0.6087	-0.7526 to -0.4647	Yes
13632	12:0 X vs. 42:50 X	-0.5120	-0.6560 to -0.3680	Yes
13633	12:0 X vs. 43:0 X	-3.720	-3.864 to -3.576	Yes
13634	12:0 X vs. 43:1 X	-3.634	-3.778 to -3.490	Yes
13635	12:0 X vs. 43:10 X	-3.350	-3.494 to -3.206	Yes

	2way ANOVA<br>Multiple comparisons	A<br>Data Set-A<br>Y	B<br>Data Set-B<br>Y	C<br>Data Set-C<br>Y
13636	12:**0 X** vs. 43:**40 X**	-0.6070	-0.7510 to -0.4630	Yes
13637	12:**0 X** vs. 43:**50 X**	-0.5123	-0.6563 to -0.3684	Yes
13638	12:**0 X** vs. 44:**0 X**	-3.744	-3.888 to -3.600	Yes
13639	12:**0 X** vs. 44:**1 X**	-3.634	-3.778 to -3.490	Yes
13640	12:**0 X** vs. 44:**10 X**	-3.413	-3.557 to -3.269	Yes
13641	12:**0 X** vs. 44:**40 X**	-0.6090	-0.7530 to -0.4650	Yes
13642	12:**0 X** vs. 44:**50 X**	-0.4447	-0.5886 to -0.3007	Yes
13643	12:**0 X** vs. 45:**0 X**	-3.609	-3.753 to -3.465	Yes
13644	12:**0 X** vs. 45:**1 X**	-3.411	-3.555 to -3.267	Yes
13645	12:**0 X** vs. 45:**10 X**	-3.293	-3.437 to -3.149	Yes
13646	12:**0 X** vs. 45:**40 X**	-0.6103	-0.7543 to -0.4664	Yes
13647	12:**0 X** vs. 45:**50 X**	-0.4417	-0.5856 to -0.2977	Yes
13648	12:**0 X** vs. 46:**0 X**	-3.413	-3.557 to -3.269	Yes
13649	12:**0 X** vs. 46:**1 X**	-3.380	-3.524 to -3.236	Yes
13650	12:**0 X** vs. 46:**10 X**	-3.288	-3.432 to -3.144	Yes
13651	12:**0 X** vs. 46:**40 X**	-0.6240	-0.7680 to -0.4800	Yes
13652	12:**0 X** vs. 46:**50 X**	-0.4347	-0.5786 to -0.2907	Yes
13653	12:**0 X** vs. 47:**0 X**	-3.389	-3.533 to -3.245	Yes
13654	12:**0 X** vs. 47:**1 X**	-3.293	-3.437 to -3.149	Yes
13655	12:**0 X** vs. 47:**10 X**	-3.274	-3.418 to -3.130	Yes
13656	12:**0 X** vs. 47:**40 X**	-0.6340	-0.7780 to -0.4900	Yes
13657	12:**0 X** vs. 47:**50 X**	-0.3927	-0.5366 to -0.2487	Yes
13658	12:**0 X** vs. 48:**0 X**	-3.376	-3.537 to -3.215	Yes
13659	12:**0 X** vs. 48:**1 X**	-3.280	-3.424 to -3.136	Yes
13660	12:**0 X** vs. 48:**10 X**	-3.274	-3.418 to -3.130	Yes
13661	12:**0 X** vs. 48:**40 X**	-0.6403	-0.7843 to -0.4964	Yes
13662	12:**0 X** vs. 48:**50 X**	-0.4000	-0.5440 to -0.2560	Yes
13663	12:**0 X** vs. 49:**0 X**	-3.376	-3.520 to -3.232	Yes
13664	12:**0 X** vs. 49:**1 X**	-3.280	-3.424 to -3.136	Yes
13665	12:**0 X** vs. 49:**10 X**	-3.274	-3.418 to -3.130	Yes
13666	12:**0 X** vs. 49:**40 X**	-0.6447	-0.7886 to -0.5007	Yes
13667	12:**0 X** vs. 49:**50 X**	-0.3993	-0.5433 to -0.2554	Yes
13668	12:**0 X** vs. 50:**0 X**	-3.376	-3.520 to -3.232	Yes
13669	12:**0 X** vs. 50:**1 X**	-3.280	-3.424 to -3.136	Yes
13670	12:**0 X** vs. 50:**10 X**	-3.274	-3.418 to -3.130	Yes
13671	12:**0 X** vs. 50:**40 X**	-0.6523	-0.7963 to -0.5084	Yes
13672	12:**0 X** vs. 50:**50 X**	-0.3990	-0.5430 to -0.2550	Yes
13673	12:**1 X** vs. 12:**10 X**	-0.01767	-0.1616 to 0.1263	No
13674	12:**1 X** vs. 12:**40 X**	-0.004000	-0.1480 to 0.1400	No
13675	12:**1 X** vs. 12:**50 X**	-0.007333	-0.1513 to 0.1366	No
13676	12:**1 X** vs. 13:**0 X**	-0.01167	-0.1556 to 0.1323	No
13677	12:**1 X** vs. 13:**1 X**	0.001000	-0.1430 to 0.1450	No
13678	12:**1 X** vs. 13:**10 X**	-0.0210	-0.1650 to 0.1230	No
13679	12:**1 X** vs. 13:**40 X**	-0.01733	-0.1613 to 0.1266	No
13680	12:**1 X** vs. 13:**50 X**	-0.01833	-0.1623 to 0.1256	No

	2way ANOVA<br>Multiple comparisons	A<br>Data Set-A<br>Y	B<br>Data Set-B<br>Y	C<br>Data Set-C<br>Y
13681	12:**1 X** vs. 14:**0 X**	-0.02033	-0.1643 to 0.1236	No
13682	12:**1 X** vs. 14:**1 X**	-0.0120	-0.1560 to 0.1320	No
13683	12:**1 X** vs. 14:**10 X**	-0.0260	-0.1700 to 0.1180	No
13684	12:**1 X** vs. 14:**40 X**	-0.02267	-0.1666 to 0.1213	No
13685	12:**1 X** vs. 14:**50 X**	-0.02667	-0.1706 to 0.1173	No
13686	12:**1 X** vs. 15:**0 X**	-0.03267	-0.1766 to 0.1113	No
13687	12:**1 X** vs. 15:**1 X**	-0.01267	-0.1566 to 0.1313	No
13688	12:**1 X** vs. 15:**10 X**	-0.0400	-0.1840 to 0.1040	No
13689	12:**1 X** vs. 15:**40 X**	-0.02233	-0.1663 to 0.1216	No
13690	12:**1 X** vs. 15:**50 X**	-0.0400	-0.1840 to 0.1040	No
13691	12:**1 X** vs. 16:**0 X**	-0.1250	-0.2690 to 0.01896	No
13692	12:**1 X** vs. 16:**1 X**	-0.0160	-0.1600 to 0.1280	No
13693	12:**1 X** vs. 16:**10 X**	-0.04167	-0.1856 to 0.1023	No
13694	12:**1 X** vs. 16:**40 X**	-0.0610	-0.2050 to 0.08296	No
13695	12:**1 X** vs. 16:**50 X**	-0.1033	-0.2473 to 0.04063	No
13696	12:**1 X** vs. 17:**0 X**	-0.1777	-0.3216 to -0.03371	Yes
13697	12:**1 X** vs. 17:**1 X**	-0.0920	-0.2360 to 0.05196	No
13698	12:**1 X** vs. 17:**10 X**	-0.1487	-0.2926 to -0.004707	Yes
13699	12:**1 X** vs. 17:**40 X**	-0.0720	-0.2160 to 0.07196	No
13700	12:**1 X** vs. 17:**50 X**	-0.1173	-0.2613 to 0.02663	No
13701	12:**1 X** vs. 18:**0 X**	-0.2263	-0.3703 to -0.08237	Yes
13702	12:**1 X** vs. 18:**1 X**	-0.1347	-0.2786 to 0.009293	No
13703	12:**1 X** vs. 18:**10 X**	-0.2270	-0.3710 to -0.08304	Yes
13704	12:**1 X** vs. 18:**40 X**	-0.07633	-0.2203 to 0.06763	No
13705	12:**1 X** vs. 18:**50 X**	-0.1213	-0.2653 to 0.02263	No
13706	12:**1 X** vs. 19:**0 X**	-0.2613	-0.4053 to -0.1174	Yes
13707	12:**1 X** vs. 19:**1 X**	-0.2493	-0.3933 to -0.1054	Yes
13708	12:**1 X** vs. 19:**10 X**	-0.2470	-0.3910 to -0.1030	Yes
13709	12:**1 X** vs. 19:**40 X**	-0.1050	-0.2490 to 0.03896	No
13710	12:**1 X** vs. 19:**50 X**	-0.1160	-0.2600 to 0.02796	No
13711	12:**1 X** vs. 20:**0 X**	-0.3410	-0.4850 to -0.1970	Yes
13712	12:**1 X** vs. 20:**1 X**	-0.1603	-0.3043 to -0.01637	Yes
13713	12:**1 X** vs. 20:**10 X**	-0.2493	-0.3933 to -0.1054	Yes
13714	12:**1 X** vs. 20:**40 X**	-0.1167	-0.2606 to 0.02729	No
13715	12:**1 X** vs. 20:**50 X**	-0.1607	-0.3046 to -0.01671	Yes
13716	12:**1 X** vs. 21:**0 X**	-0.3643	-0.5083 to -0.2204	Yes
13717	12:**1 X** vs. 21:**1 X**	-0.3173	-0.4613 to -0.1734	Yes
13718	12:**1 X** vs. 21:**10 X**	-0.3143	-0.4583 to -0.1704	Yes
13719	12:**1 X** vs. 21:**40 X**	-0.1897	-0.3336 to -0.04571	Yes
13720	12:**1 X** vs. 21:**50 X**	-0.2800	-0.4240 to -0.1360	Yes
13721	12:**1 X** vs. 22:**0 X**	-0.4060	-0.5500 to -0.2620	Yes
13722	12:**1 X** vs. 22:**1 X**	-0.4007	-0.5446 to -0.2567	Yes
13723	12:**1 X** vs. 22:**10 X**	-0.3960	-0.5400 to -0.2520	Yes
13724	12:**1 X** vs. 22:**40 X**	-0.2963	-0.4403 to -0.1524	Yes
13725	12:**1 X** vs. 22:**50 X**	-0.3590	-0.5030 to -0.2150	Yes

2way ANOVA<br>Multiple comparisons		A<br>Data Set-A<br>Y	B<br>Data Set-B<br>Y	C<br>Data Set-C<br>Y
13726	12:**1 X** vs. 23:**0 X**	-0.4713	-0.6153 to -0.3274	Yes
13727	12:**1 X** vs. 23:**1 X**	-0.6610	-0.8220 to -0.5000	Yes
13728	12:**1 X** vs. 23:**10 X**	-0.4107	-0.5546 to -0.2667	Yes
13729	12:**1 X** vs. 23:**40 X**	-0.2910	-0.4520 to -0.1300	Yes
13730	12:**1 X** vs. 23:**50 X**	-0.3250	-0.4690 to -0.1810	Yes
13731	12:**1 X** vs. 24:**0 X**	-0.5887	-0.7326 to -0.4447	Yes
13732	12:**1 X** vs. 24:**1 X**	-0.7450	-0.9060 to -0.5840	Yes
13733	12:**1 X** vs. 24:**10 X**	-0.5725	-0.7335 to -0.4115	Yes
13734	12:**1 X** vs. 24:**40 X**	-0.3073	-0.4513 to -0.1634	Yes
13735	12:**1 X** vs. 24:**50 X**	-0.2290	-0.3730 to -0.08504	Yes
13736	12:**1 X** vs. 25:**0 X**	-0.9260	-1.087 to -0.7650	Yes
13737	12:**1 X** vs. 25:**1 X**	-1.116	-1.276 to -0.9545	Yes
13738	12:**1 X** vs. 25:**10 X**	-0.4770	-0.6806 to -0.2734	Yes
13739	12:**1 X** vs. 25:**40 X**	-0.3093	-0.4533 to -0.1654	Yes
13740	12:**1 X** vs. 25:**50 X**	-0.2837	-0.4276 to -0.1397	Yes
13741	12:**1 X** vs. 26:**0 X**	-1.139	-1.299 to -0.9775	Yes
13742	12:**1 X** vs. 26:**1 X**	-1.252	-1.412 to -1.091	Yes
13743	12:**1 X** vs. 26:**10 X**	-0.9380	-1.099 to -0.7770	Yes
13744	12:**1 X** vs. 26:**40 X**	-0.3837	-0.5276 to -0.2397	Yes
13745	12:**1 X** vs. 26:**50 X**	-0.2440	-0.3880 to -0.1000	Yes
13746	12:**1 X** vs. 27:**0 X**	-1.326	-1.470 to -1.182	Yes
13747	12:**1 X** vs. 27:**1 X**	-1.417	-1.578 to -1.256	Yes
13748	12:**1 X** vs. 27:**10 X**	-1.229	-1.390 to -1.068	Yes
13749	12:**1 X** vs. 27:**40 X**	-0.3837	-0.5276 to -0.2397	Yes
13750	12:**1 X** vs. 27:**50 X**	-0.2433	-0.3873 to -0.09937	Yes
13751	12:**1 X** vs. 28:**0 X**	-1.276	-1.436 to -1.115	Yes
13752	12:**1 X** vs. 28:**1 X**	-1.316	-1.460 to -1.172	Yes
13753	12:**1 X** vs. 28:**10 X**	-1.118	-1.279 to -0.9570	Yes
13754	12:**1 X** vs. 28:**40 X**	-0.3960	-0.5400 to -0.2520	Yes
13755	12:**1 X** vs. 28:**50 X**	-0.2913	-0.4353 to -0.1474	Yes
13756	12:**1 X** vs. 29:**0 X**	-1.757	-1.918 to -1.596	Yes
13757	12:**1 X** vs. 29:**1 X**	-1.842	-2.003 to -1.681	Yes
13758	12:**1 X** vs. 29:**10 X**	-1.057	-1.217 to -0.8955	Yes
13759	12:**1 X** vs. 29:**40 X**	-0.5040	-0.6480 to -0.3600	Yes
13760	12:**1 X** vs. 29:**50 X**	-0.2927	-0.4366 to -0.1487	Yes
13761	12:**1 X** vs. 30:**0 X**	-1.472	-1.632 to -1.311	Yes
13762	12:**1 X** vs. 30:**1 X**	-1.793	-1.954 to -1.632	Yes
13763	12:**1 X** vs. 30:**10 X**	-1.131	-1.292 to -0.9700	Yes
13764	12:**1 X** vs. 30:**40 X**	-0.4310	-0.5750 to -0.2870	Yes
13765	12:**1 X** vs. 30:**50 X**	-0.3450	-0.4890 to -0.2010	Yes
13766	12:**1 X** vs. 31:**0 X**	-1.790	-1.950 to -1.629	Yes
13767	12:**1 X** vs. 31:**1 X**	-1.780	-1.941 to -1.619	Yes
13768	12:**1 X** vs. 31:**10 X**	-1.263	-1.423 to -1.102	Yes
13769	12:**1 X** vs. 31:**40 X**	-0.5227	-0.6666 to -0.3787	Yes
13770	12:**1 X** vs. 31:**50 X**	-0.3240	-0.4680 to -0.1800	Yes

	2way ANOVA<br>Multiple comparisons	A<br>Data Set-A<br>Y	B<br>Data Set-B<br>Y	C<br>Data Set-C<br>Y
13771	12:**1 X** vs. 32:**0 X**	-2.381	-2.525 to -2.237	Yes
13772	12:**1 X** vs. 32:**1 X**	-1.837	-1.998 to -1.676	Yes
13773	12:**1 X** vs. 32:**10 X**	-1.693	-1.837 to -1.549	Yes
13774	12:**1 X** vs. 32:**40 X**	-0.5190	-0.6630 to -0.3750	Yes
13775	12:**1 X** vs. 32:**50 X**	-0.3237	-0.4676 to -0.1797	Yes
13776	12:**1 X** vs. 33:**0 X**	-2.385	-2.529 to -2.241	Yes
13777	12:**1 X** vs. 33:**1 X**	-2.199	-2.343 to -2.055	Yes
13778	12:**1 X** vs. 33:**10 X**	-1.860	-2.004 to -1.716	Yes
13779	12:**1 X** vs. 33:**40 X**	-0.5377	-0.6816 to -0.3937	Yes
13780	12:**1 X** vs. 33:**50 X**	-0.3387	-0.4826 to -0.1947	Yes
13781	12:**1 X** vs. 34:**0 X**	-2.309	-2.453 to -2.165	Yes
13782	12:**1 X** vs. 34:**1 X**	-2.317	-2.461 to -2.173	Yes
13783	12:**1 X** vs. 34:**10 X**	-1.950	-2.094 to -1.806	Yes
13784	12:**1 X** vs. 34:**40 X**	-0.5497	-0.6936 to -0.4057	Yes
13785	12:**1 X** vs. 34:**50 X**	-0.4877	-0.6316 to -0.3437	Yes
13786	12:**1 X** vs. 35:**0 X**	-2.658	-2.802 to -2.514	Yes
13787	12:**1 X** vs. 35:**1 X**	-2.424	-2.568 to -2.280	Yes
13788	12:**1 X** vs. 35:**10 X**	-1.912	-2.056 to -1.768	Yes
13789	12:**1 X** vs. 35:**40 X**	-0.5587	-0.7026 to -0.4147	Yes
13790	12:**1 X** vs. 35:**50 X**	-0.5003	-0.6443 to -0.3564	Yes
13791	12:**1 X** vs. 36:**0 X**	-2.865	-3.025 to -2.704	Yes
13792	12:**1 X** vs. 36:**1 X**	-2.560	-2.704 to -2.416	Yes
13793	12:**1 X** vs. 36:**10 X**	-1.917	-2.061 to -1.773	Yes
13794	12:**1 X** vs. 36:**40 X**	-0.6140	-0.7580 to -0.4700	Yes
13795	12:**1 X** vs. 36:**50 X**	-0.4637	-0.6076 to -0.3197	Yes
13796	12:**1 X** vs. 37:**0 X**	-3.248	-3.392 to -3.104	Yes
13797	12:**1 X** vs. 37:**1 X**	-2.630	-2.774 to -2.486	Yes
13798	12:**1 X** vs. 37:**10 X**	-1.919	-2.063 to -1.775	Yes
13799	12:**1 X** vs. 37:**40 X**	-0.6513	-0.7953 to -0.5074	Yes
13800	12:**1 X** vs. 37:**50 X**	-0.4723	-0.6163 to -0.3284	Yes
13801	12:**1 X** vs. 38:**0 X**	-3.193	-3.337 to -3.049	Yes
13802	12:**1 X** vs. 38:**1 X**	-2.749	-2.893 to -2.605	Yes
13803	12:**1 X** vs. 38:**10 X**	-2.194	-2.338 to -2.050	Yes
13804	12:**1 X** vs. 38:**40 X**	-0.6557	-0.7996 to -0.5117	Yes
13805	12:**1 X** vs. 38:**50 X**	-0.4763	-0.6203 to -0.3324	Yes
13806	12:**1 X** vs. 39:**0 X**	-3.424	-3.568 to -3.280	Yes
13807	12:**1 X** vs. 39:**1 X**	-3.316	-3.460 to -3.172	Yes
13808	12:**1 X** vs. 39:**10 X**	-2.339	-2.483 to -2.195	Yes
13809	12:**1 X** vs. 39:**40 X**	-0.6617	-0.8056 to -0.5177	Yes
13810	12:**1 X** vs. 39:**50 X**	-0.5073	-0.6513 to -0.3634	Yes
13811	12:**1 X** vs. 40:**0 X**	-3.465	-3.609 to -3.321	Yes
13812	12:**1 X** vs. 40:**1 X**	-3.391	-3.535 to -3.247	Yes
13813	12:**1 X** vs. 40:**10 X**	-2.596	-2.740 to -2.452	Yes
13814	12:**1 X** vs. 40:**40 X**	-0.6663	-0.8103 to -0.5224	Yes
13815	12:**1 X** vs. 40:**50 X**	-0.5137	-0.6576 to -0.3697	Yes

2way ANOVA<br>Multiple comparisons	A<br>Data Set-A<br>Y	B<br>Data Set-B<br>Y	C<br>Data Set-C<br>Y
13816 12:1 X vs. 41:0 X	-3.506	-3.650 to -3.362	Yes
13817 12:1 X vs. 41:1 X	-3.505	-3.649 to -3.361	Yes
13818 12:1 X vs. 41:10 X	-2.649	-2.793 to -2.505	Yes
13819 12:1 X vs. 41:40 X	-0.6137	-0.7576 to -0.4697	Yes
13820 12:1 X vs. 41:50 X	-0.5263	-0.6703 to -0.3824	Yes
13821 12:1 X vs. 42:0 X	-3.648	-3.792 to -3.504	Yes
13822 12:1 X vs. 42:1 X	-3.667	-3.811 to -3.523	Yes
13823 12:1 X vs. 42:10 X	-2.650	-2.794 to -2.506	Yes
13824 12:1 X vs. 42:40 X	-0.6263	-0.7703 to -0.4824	Yes
13825 12:1 X vs. 42:50 X	-0.5297	-0.6736 to -0.3857	Yes
13826 12:1 X vs. 43:0 X	-3.738	-3.882 to -3.594	Yes
13827 12:1 X vs. 43:1 X	-3.652	-3.796 to -3.508	Yes
13828 12:1 X vs. 43:10 X	-3.368	-3.512 to -3.224	Yes
13829 12:1 X vs. 43:40 X	-0.6247	-0.7686 to -0.4807	Yes
13830 12:1 X vs. 43:50 X	-0.5300	-0.6740 to -0.3860	Yes
13831 12:1 X vs. 44:0 X	-3.762	-3.906 to -3.618	Yes
13832 12:1 X vs. 44:1 X	-3.651	-3.795 to -3.507	Yes
13833 12:1 X vs. 44:10 X	-3.431	-3.575 to -3.287	Yes
13834 12:1 X vs. 44:40 X	-0.6267	-0.7706 to -0.4827	Yes
13835 12:1 X vs. 44:50 X	-0.4623	-0.6063 to -0.3184	Yes
13836 12:1 X vs. 45:0 X	-3.626	-3.770 to -3.482	Yes
13837 12:1 X vs. 45:1 X	-3.428	-3.572 to -3.284	Yes
13838 12:1 X vs. 45:10 X	-3.311	-3.455 to -3.167	Yes
13839 12:1 X vs. 45:40 X	-0.6280	-0.7720 to -0.4840	Yes
13840 12:1 X vs. 45:50 X	-0.4593	-0.6033 to -0.3154	Yes
13841 12:1 X vs. 46:0 X	-3.431	-3.575 to -3.287	Yes
13842 12:1 X vs. 46:1 X	-3.398	-3.542 to -3.254	Yes
13843 12:1 X vs. 46:10 X	-3.306	-3.450 to -3.162	Yes
13844 12:1 X vs. 46:40 X	-0.6417	-0.7856 to -0.4977	Yes
13845 12:1 X vs. 46:50 X	-0.4523	-0.5963 to -0.3084	Yes
13846 12:1 X vs. 47:0 X	-3.406	-3.550 to -3.262	Yes
13847 12:1 X vs. 47:1 X	-3.310	-3.454 to -3.166	Yes
13848 12:1 X vs. 47:10 X	-3.292	-3.436 to -3.148	Yes
13849 12:1 X vs. 47:40 X	-0.6517	-0.7956 to -0.5077	Yes
13850 12:1 X vs. 47:50 X	-0.4103	-0.5543 to -0.2664	Yes
13851 12:1 X vs. 48:0 X	-3.394	-3.554 to -3.233	Yes
13852 12:1 X vs. 48:1 X	-3.297	-3.441 to -3.153	Yes
13853 12:1 X vs. 48:10 X	-3.292	-3.436 to -3.148	Yes
13854 12:1 X vs. 48:40 X	-0.6580	-0.8020 to -0.5140	Yes
13855 12:1 X vs. 48:50 X	-0.4177	-0.5616 to -0.2737	Yes
13856 12:1 X vs. 49:0 X	-3.394	-3.538 to -3.250	Yes
13857 12:1 X vs. 49:1 X	-3.297	-3.441 to -3.153	Yes
13858 12:1 X vs. 49:10 X	-3.292	-3.436 to -3.148	Yes
13859 12:1 X vs. 49:40 X	-0.6623	-0.8063 to -0.5184	Yes
13860 12:1 X vs. 49:50 X	-0.4170	-0.5610 to -0.2730	Yes

2way ANOVA<br>Multiple comparisons	A<br>Data Set-A<br>Y	B<br>Data Set-B<br>Y	C<br>Data Set-C<br>Y
13861 12:1 X vs. 50:0 X	-3.394	-3.538 to -3.250	Yes
13862 12:1 X vs. 50:1 X	-3.297	-3.441 to -3.153	Yes
13863 12:1 X vs. 50:10 X	-3.292	-3.436 to -3.148	Yes
13864 12:1 X vs. 50:40 X	-0.6700	-0.8140 to -0.5260	Yes
13865 12:1 X vs. 50:50 X	-0.4167	-0.5606 to -0.2727	Yes
13866 12:10 X vs. 12:40 X	0.01367	-0.1303 to 0.1576	No
13867 12:10 X vs. 12:50 X	0.01033	-0.1336 to 0.1543	No
13868 12:10 X vs. 13:0 X	0.0060	-0.1380 to 0.1500	No
13869 12:10 X vs. 13:1 X	0.01867	-0.1253 to 0.1626	No
13870 12:10 X vs. 13:10 X	-0.003333	-0.1473 to 0.1406	No
13871 12:10 X vs. 13:40 X	0.0003333	-0.1436 to 0.1443	No
13872 12:10 X vs. 13:50 X	-0.0006667	-0.1446 to 0.1433	No
13873 12:10 X vs. 14:0 X	-0.002667	-0.1466 to 0.1413	No
13874 12:10 X vs. 14:1 X	0.005667	-0.1383 to 0.1496	No
13875 12:10 X vs. 14:10 X	-0.008333	-0.1523 to 0.1356	No
13876 12:10 X vs. 14:40 X	-0.005000	-0.1490 to 0.1390	No
13877 12:10 X vs. 14:50 X	-0.009000	-0.1530 to 0.1350	No
13878 12:10 X vs. 15:0 X	-0.0150	-0.1590 to 0.1290	No
13879 12:10 X vs. 15:1 X	0.0050	-0.1390 to 0.1490	No
13880 12:10 X vs. 15:10 X	-0.02233	-0.1663 to 0.1216	No
13881 12:10 X vs. 15:40 X	-0.004667	-0.1486 to 0.1393	No
13882 12:10 X vs. 15:50 X	-0.02233	-0.1663 to 0.1216	No
13883 12:10 X vs. 16:0 X	-0.1073	-0.2513 to 0.03663	No
13884 12:10 X vs. 16:1 X	0.001667	-0.1423 to 0.1456	No
13885 12:10 X vs. 16:10 X	-0.0240	-0.1680 to 0.1200	No
13886 12:10 X vs. 16:40 X	-0.04333	-0.1873 to 0.1006	No
13887 12:10 X vs. 16:50 X	-0.08567	-0.2296 to 0.05829	No
13888 12:10 X vs. 17:0 X	-0.1600	-0.3040 to -0.01604	Yes
13889 12:10 X vs. 17:1 X	-0.07433	-0.2183 to 0.06963	No
13890 12:10 X vs. 17:10 X	-0.1310	-0.2750 to 0.01296	No
13891 12:10 X vs. 17:40 X	-0.05433	-0.1983 to 0.08963	No
13892 12:10 X vs. 17:50 X	-0.09967	-0.2436 to 0.04429	No
13893 12:10 X vs. 18:0 X	-0.2087	-0.3526 to -0.06471	Yes
13894 12:10 X vs. 18:1 X	-0.1170	-0.2610 to 0.02696	No
13895 12:10 X vs. 18:10 X	-0.2093	-0.3533 to -0.06537	Yes
13896 12:10 X vs. 18:40 X	-0.05867	-0.2026 to 0.08529	No
13897 12:10 X vs. 18:50 X	-0.1037	-0.2476 to 0.04029	No
13898 12:10 X vs. 19:0 X	-0.2437	-0.3876 to -0.09971	Yes
13899 12:10 X vs. 19:1 X	-0.2317	-0.3756 to -0.08771	Yes
13900 12:10 X vs. 19:10 X	-0.2293	-0.3733 to -0.08537	Yes
13901 12:10 X vs. 19:40 X	-0.08733	-0.2313 to 0.05663	No
13902 12:10 X vs. 19:50 X	-0.09833	-0.2423 to 0.04563	No
13903 12:10 X vs. 20:0 X	-0.3233	-0.4673 to -0.1794	Yes
13904 12:10 X vs. 20:1 X	-0.1427	-0.2866 to 0.001293	No
13905 12:10 X vs. 20:10 X	-0.2317	-0.3756 to -0.08771	Yes

2way ANOVA<br>Multiple comparisons		A<br>Data Set-A<br>Y	B<br>Data Set-B<br>Y	C<br>Data Set-C<br>Y
13906	12:10 X vs. 20:40 X	-0.0990	-0.2430 to 0.04496	No
13907	12:10 X vs. 20:50 X	-0.1430	-0.2870 to 0.0009598	No
13908	12:10 X vs. 21:0 X	-0.3467	-0.4906 to -0.2027	Yes
13909	12:10 X vs. 21:1 X	-0.2997	-0.4436 to -0.1557	Yes
13910	12:10 X vs. 21:10 X	-0.2967	-0.4406 to -0.1527	Yes
13911	12:10 X vs. 21:40 X	-0.1720	-0.3160 to -0.02804	Yes
13912	12:10 X vs. 21:50 X	-0.2623	-0.4063 to -0.1184	Yes
13913	12:10 X vs. 22:0 X	-0.3883	-0.5323 to -0.2444	Yes
13914	12:10 X vs. 22:1 X	-0.3830	-0.5270 to -0.2390	Yes
13915	12:10 X vs. 22:10 X	-0.3783	-0.5223 to -0.2344	Yes
13916	12:10 X vs. 22:40 X	-0.2787	-0.4226 to -0.1347	Yes
13917	12:10 X vs. 22:50 X	-0.3413	-0.4853 to -0.1974	Yes
13918	12:10 X vs. 23:0 X	-0.4537	-0.5976 to -0.3097	Yes
13919	12:10 X vs. 23:1 X	-0.6433	-0.8043 to -0.4824	Yes
13920	12:10 X vs. 23:10 X	-0.3930	-0.5370 to -0.2490	Yes
13921	12:10 X vs. 23:40 X	-0.2733	-0.4343 to -0.1124	Yes
13922	12:10 X vs. 23:50 X	-0.3073	-0.4513 to -0.1634	Yes
13923	12:10 X vs. 24:0 X	-0.5710	-0.7150 to -0.4270	Yes
13924	12:10 X vs. 24:1 X	-0.7273	-0.8883 to -0.5664	Yes
13925	12:10 X vs. 24:10 X	-0.5548	-0.7158 to -0.3939	Yes
13926	12:10 X vs. 24:40 X	-0.2897	-0.4336 to -0.1457	Yes
13927	12:10 X vs. 24:50 X	-0.2113	-0.3553 to -0.06737	Yes
13928	12:10 X vs. 25:0 X	-0.9083	-1.069 to -0.7474	Yes
13929	12:10 X vs. 25:1 X	-1.098	-1.259 to -0.9369	Yes
13930	12:10 X vs. 25:10 X	-0.4593	-0.6629 to -0.2557	Yes
13931	12:10 X vs. 25:40 X	-0.2917	-0.4356 to -0.1477	Yes
13932	12:10 X vs. 25:50 X	-0.2660	-0.4100 to -0.1220	Yes
13933	12:10 X vs. 26:0 X	-1.121	-1.282 to -0.9599	Yes
13934	12:10 X vs. 26:1 X	-1.234	-1.395 to -1.073	Yes
13935	12:10 X vs. 26:10 X	-0.9203	-1.081 to -0.7594	Yes
13936	12:10 X vs. 26:40 X	-0.3660	-0.5100 to -0.2220	Yes
13937	12:10 X vs. 26:50 X	-0.2263	-0.3703 to -0.08237	Yes
13938	12:10 X vs. 27:0 X	-1.309	-1.453 to -1.165	Yes
13939	12:10 X vs. 27:1 X	-1.399	-1.560 to -1.238	Yes
13940	12:10 X vs. 27:10 X	-1.211	-1.372 to -1.050	Yes
13941	12:10 X vs. 27:40 X	-0.3660	-0.5100 to -0.2220	Yes
13942	12:10 X vs. 27:50 X	-0.2257	-0.3696 to -0.08171	Yes
13943	12:10 X vs. 28:0 X	-1.258	-1.419 to -1.097	Yes
13944	12:10 X vs. 28:1 X	-1.299	-1.443 to -1.155	Yes
13945	12:10 X vs. 28:10 X	-1.100	-1.261 to -0.9394	Yes
13946	12:10 X vs. 28:40 X	-0.3783	-0.5223 to -0.2344	Yes
13947	12:10 X vs. 28:50 X	-0.2737	-0.4176 to -0.1297	Yes
13948	12:10 X vs. 29:0 X	-1.739	-1.900 to -1.578	Yes
13949	12:10 X vs. 29:1 X	-1.824	-1.985 to -1.663	Yes
13950	12:10 X vs. 29:10 X	-1.039	-1.200 to -0.8779	Yes

	2way ANOVA<br>Multiple comparisons	A<br>Data Set-A<br>Y	B<br>Data Set-B<br>Y	C<br>Data Set-C<br>Y
13951	12:10 X vs. 29:40 X	-0.4863	-0.6303 to -0.3424	Yes
13952	12:10 X vs. 29:50 X	-0.2750	-0.4190 to -0.1310	Yes
13953	12:10 X vs. 30:0 X	-1.454	-1.615 to -1.293	Yes
13954	12:10 X vs. 30:1 X	-1.775	-1.936 to -1.614	Yes
13955	12:10 X vs. 30:10 X	-1.113	-1.274 to -0.9524	Yes
13956	12:10 X vs. 30:40 X	-0.4133	-0.5573 to -0.2694	Yes
13957	12:10 X vs. 30:50 X	-0.3273	-0.4713 to -0.1834	Yes
13958	12:10 X vs. 31:0 X	-1.772	-1.933 to -1.611	Yes
13959	12:10 X vs. 31:1 X	-1.762	-1.923 to -1.601	Yes
13960	12:10 X vs. 31:10 X	-1.245	-1.406 to -1.084	Yes
13961	12:10 X vs. 31:40 X	-0.5050	-0.6490 to -0.3610	Yes
13962	12:10 X vs. 31:50 X	-0.3063	-0.4503 to -0.1624	Yes
13963	12:10 X vs. 32:0 X	-2.363	-2.507 to -2.219	Yes
13964	12:10 X vs. 32:1 X	-1.819	-1.980 to -1.658	Yes
13965	12:10 X vs. 32:10 X	-1.675	-1.819 to -1.531	Yes
13966	12:10 X vs. 32:40 X	-0.5013	-0.6453 to -0.3574	Yes
13967	12:10 X vs. 32:50 X	-0.3060	-0.4500 to -0.1620	Yes
13968	12:10 X vs. 33:0 X	-2.367	-2.511 to -2.223	Yes
13969	12:10 X vs. 33:1 X	-2.182	-2.326 to -2.038	Yes
13970	12:10 X vs. 33:10 X	-1.842	-1.986 to -1.698	Yes
13971	12:10 X vs. 33:40 X	-0.5200	-0.6640 to -0.3760	Yes
13972	12:10 X vs. 33:50 X	-0.3210	-0.4650 to -0.1770	Yes
13973	12:10 X vs. 34:0 X	-2.292	-2.436 to -2.148	Yes
13974	12:10 X vs. 34:1 X	-2.299	-2.443 to -2.155	Yes
13975	12:10 X vs. 34:10 X	-1.932	-2.076 to -1.788	Yes
13976	12:10 X vs. 34:40 X	-0.5320	-0.6760 to -0.3880	Yes
13977	12:10 X vs. 34:50 X	-0.4700	-0.6140 to -0.3260	Yes
13978	12:10 X vs. 35:0 X	-2.640	-2.784 to -2.496	Yes
13979	12:10 X vs. 35:1 X	-2.406	-2.550 to -2.262	Yes
13980	12:10 X vs. 35:10 X	-1.894	-2.038 to -1.750	Yes
13981	12:10 X vs. 35:40 X	-0.5410	-0.6850 to -0.3970	Yes
13982	12:10 X vs. 35:50 X	-0.4827	-0.6266 to -0.3387	Yes
13983	12:10 X vs. 36:0 X	-2.847	-3.008 to -2.686	Yes
13984	12:10 X vs. 36:1 X	-2.543	-2.687 to -2.399	Yes
13985	12:10 X vs. 36:10 X	-1.900	-2.044 to -1.756	Yes
13986	12:10 X vs. 36:40 X	-0.5963	-0.7403 to -0.4524	Yes
13987	12:10 X vs. 36:50 X	-0.4460	-0.5900 to -0.3020	Yes
13988	12:10 X vs. 37:0 X	-3.231	-3.375 to -3.087	Yes
13989	12:10 X vs. 37:1 X	-2.613	-2.757 to -2.469	Yes
13990	12:10 X vs. 37:10 X	-1.901	-2.045 to -1.757	Yes
13991	12:10 X vs. 37:40 X	-0.6337	-0.7776 to -0.4897	Yes
13992	12:10 X vs. 37:50 X	-0.4547	-0.5986 to -0.3107	Yes
13993	12:10 X vs. 38:0 X	-3.176	-3.320 to -3.032	Yes
13994	12:10 X vs. 38:1 X	-2.731	-2.875 to -2.587	Yes
13995	12:10 X vs. 38:10 X	-2.177	-2.321 to -2.033	Yes

	2way ANOVA<br>Multiple comparisons	A<br>Data Set-A<br>Y	B<br>Data Set-B<br>Y	C<br>Data Set-C<br>Y
13996	12:**10 X** vs. 38:**40 X**	-0.6380	-0.7820 to -0.4940	Yes
13997	12:**10 X** vs. 38:**50 X**	-0.4587	-0.6026 to -0.3147	Yes
13998	12:**10 X** vs. 39:**0 X**	-3.406	-3.550 to -3.262	Yes
13999	12:**10 X** vs. 39:**1 X**	-3.298	-3.442 to -3.154	Yes
14000	12:**10 X** vs. 39:**10 X**	-2.322	-2.466 to -2.178	Yes
14001	12:**10 X** vs. 39:**40 X**	-0.6440	-0.7880 to -0.5000	Yes
14002	12:**10 X** vs. 39:**50 X**	-0.4897	-0.6336 to -0.3457	Yes
14003	12:**10 X** vs. 40:**0 X**	-3.447	-3.591 to -3.303	Yes
14004	12:**10 X** vs. 40:**1 X**	-3.373	-3.517 to -3.229	Yes
14005	12:**10 X** vs. 40:**10 X**	-2.578	-2.722 to -2.434	Yes
14006	12:**10 X** vs. 40:**40 X**	-0.6487	-0.7926 to -0.5047	Yes
14007	12:**10 X** vs. 40:**50 X**	-0.4960	-0.6400 to -0.3520	Yes
14008	12:**10 X** vs. 41:**0 X**	-3.488	-3.632 to -3.344	Yes
14009	12:**10 X** vs. 41:**1 X**	-3.487	-3.631 to -3.343	Yes
14010	12:**10 X** vs. 41:**10 X**	-2.632	-2.776 to -2.488	Yes
14011	12:**10 X** vs. 41:**40 X**	-0.5960	-0.7400 to -0.4520	Yes
14012	12:**10 X** vs. 41:**50 X**	-0.5087	-0.6526 to -0.3647	Yes
14013	12:**10 X** vs. 42:**0 X**	-3.630	-3.774 to -3.486	Yes
14014	12:**10 X** vs. 42:**1 X**	-3.650	-3.794 to -3.506	Yes
14015	12:**10 X** vs. 42:**10 X**	-2.632	-2.776 to -2.488	Yes
14016	12:**10 X** vs. 42:**40 X**	-0.6087	-0.7526 to -0.4647	Yes
14017	12:**10 X** vs. 42:**50 X**	-0.5120	-0.6560 to -0.3680	Yes
14018	12:**10 X** vs. 43:**0 X**	-3.720	-3.864 to -3.576	Yes
14019	12:**10 X** vs. 43:**1 X**	-3.634	-3.778 to -3.490	Yes
14020	12:**10 X** vs. 43:**10 X**	-3.350	-3.494 to -3.206	Yes
14021	12:**10 X** vs. 43:**40 X**	-0.6070	-0.7510 to -0.4630	Yes
14022	12:**10 X** vs. 43:**50 X**	-0.5123	-0.6563 to -0.3684	Yes
14023	12:**10 X** vs. 44:**0 X**	-3.744	-3.888 to -3.600	Yes
14024	12:**10 X** vs. 44:**1 X**	-3.634	-3.778 to -3.490	Yes
14025	12:**10 X** vs. 44:**10 X**	-3.413	-3.557 to -3.269	Yes
14026	12:**10 X** vs. 44:**40 X**	-0.6090	-0.7530 to -0.4650	Yes
14027	12:**10 X** vs. 44:**50 X**	-0.4447	-0.5886 to -0.3007	Yes
14028	12:**10 X** vs. 45:**0 X**	-3.609	-3.753 to -3.465	Yes
14029	12:**10 X** vs. 45:**1 X**	-3.411	-3.555 to -3.267	Yes
14030	12:**10 X** vs. 45:**10 X**	-3.293	-3.437 to -3.149	Yes
14031	12:**10 X** vs. 45:**40 X**	-0.6103	-0.7543 to -0.4664	Yes
14032	12:**10 X** vs. 45:**50 X**	-0.4417	-0.5856 to -0.2977	Yes
14033	12:**10 X** vs. 46:**0 X**	-3.413	-3.557 to -3.269	Yes
14034	12:**10 X** vs. 46:**1 X**	-3.380	-3.524 to -3.236	Yes
14035	12:**10 X** vs. 46:**10 X**	-3.288	-3.432 to -3.144	Yes
14036	12:**10 X** vs. 46:**40 X**	-0.6240	-0.7680 to -0.4800	Yes
14037	12:**10 X** vs. 46:**50 X**	-0.4347	-0.5786 to -0.2907	Yes
14038	12:**10 X** vs. 47:**0 X**	-3.389	-3.533 to -3.245	Yes
14039	12:**10 X** vs. 47:**1 X**	-3.293	-3.437 to -3.149	Yes
14040	12:**10 X** vs. 47:**10 X**	-3.274	-3.418 to -3.130	Yes

2way ANOVA<br>Multiple comparisons		A<br>Data Set-A<br>Y	B<br>Data Set-B<br>Y	C<br>Data Set-C<br>Y
14041	12:10 X vs. 47:40 X	-0.6340	-0.7780 to -0.4900	Yes
14042	12:10 X vs. 47:50 X	-0.3927	-0.5366 to -0.2487	Yes
14043	12:10 X vs. 48:0 X	-3.376	-3.537 to -3.215	Yes
14044	12:10 X vs. 48:1 X	-3.280	-3.424 to -3.136	Yes
14045	12:10 X vs. 48:10 X	-3.274	-3.418 to -3.130	Yes
14046	12:10 X vs. 48:40 X	-0.6403	-0.7843 to -0.4964	Yes
14047	12:10 X vs. 48:50 X	-0.4000	-0.5440 to -0.2560	Yes
14048	12:10 X vs. 49:0 X	-3.376	-3.520 to -3.232	Yes
14049	12:10 X vs. 49:1 X	-3.280	-3.424 to -3.136	Yes
14050	12:10 X vs. 49:10 X	-3.274	-3.418 to -3.130	Yes
14051	12:10 X vs. 49:40 X	-0.6447	-0.7886 to -0.5007	Yes
14052	12:10 X vs. 49:50 X	-0.3993	-0.5433 to -0.2554	Yes
14053	12:10 X vs. 50:0 X	-3.376	-3.520 to -3.232	Yes
14054	12:10 X vs. 50:1 X	-3.280	-3.424 to -3.136	Yes
14055	12:10 X vs. 50:10 X	-3.274	-3.418 to -3.130	Yes
14056	12:10 X vs. 50:40 X	-0.6523	-0.7963 to -0.5084	Yes
14057	12:10 X vs. 50:50 X	-0.3990	-0.5430 to -0.2550	Yes
14058	12:40 X vs. 12:50 X	-0.003333	-0.1473 to 0.1406	No
14059	12:40 X vs. 13:0 X	-0.007667	-0.1516 to 0.1363	No
14060	12:40 X vs. 13:1 X	0.0050	-0.1390 to 0.1490	No
14061	12:40 X vs. 13:10 X	-0.0170	-0.1610 to 0.1270	No
14062	12:40 X vs. 13:40 X	-0.01333	-0.1573 to 0.1306	No
14063	12:40 X vs. 13:50 X	-0.01433	-0.1583 to 0.1296	No
14064	12:40 X vs. 14:0 X	-0.01633	-0.1603 to 0.1276	No
14065	12:40 X vs. 14:1 X	-0.0080	-0.1520 to 0.1360	No
14066	12:40 X vs. 14:10 X	-0.0220	-0.1660 to 0.1220	No
14067	12:40 X vs. 14:40 X	-0.01867	-0.1626 to 0.1253	No
14068	12:40 X vs. 14:50 X	-0.02267	-0.1666 to 0.1213	No
14069	12:40 X vs. 15:0 X	-0.02867	-0.1726 to 0.1153	No
14070	12:40 X vs. 15:1 X	-0.008667	-0.1526 to 0.1353	No
14071	12:40 X vs. 15:10 X	-0.0360	-0.1800 to 0.1080	No
14072	12:40 X vs. 15:40 X	-0.01833	-0.1623 to 0.1256	No
14073	12:40 X vs. 15:50 X	-0.0360	-0.1800 to 0.1080	No
14074	12:40 X vs. 16:0 X	-0.1210	-0.2650 to 0.02296	No
14075	12:40 X vs. 16:1 X	-0.0120	-0.1560 to 0.1320	No
14076	12:40 X vs. 16:10 X	-0.03767	-0.1816 to 0.1063	No
14077	12:40 X vs. 16:40 X	-0.0570	-0.2010 to 0.08696	No
14078	12:40 X vs. 16:50 X	-0.09933	-0.2433 to 0.04463	No
14079	12:40 X vs. 17:0 X	-0.1737	-0.3176 to -0.02971	Yes
14080	12:40 X vs. 17:1 X	-0.0880	-0.2320 to 0.05596	No
14081	12:40 X vs. 17:10 X	-0.1447	-0.2886 to -0.0007068	Yes
14082	12:40 X vs. 17:40 X	-0.0680	-0.2120 to 0.07596	No
14083	12:40 X vs. 17:50 X	-0.1133	-0.2573 to 0.03063	No
14084	12:40 X vs. 18:0 X	-0.2223	-0.3663 to -0.07837	Yes
14085	12:40 X vs. 18:1 X	-0.1307	-0.2746 to 0.01329	No

2way ANOVA<br>Multiple comparisons	A<br>Data Set-A<br>Y	B<br>Data Set-B<br>Y	C<br>Data Set-C<br>Y
14086 12:40 X vs. 18:10 X	-0.2230	-0.3670 to -0.07904	Yes
14087 12:40 X vs. 18:40 X	-0.07233	-0.2163 to 0.07163	No
14088 12:40 X vs. 18:50 X	-0.1173	-0.2613 to 0.02663	No
14089 12:40 X vs. 19:0 X	-0.2573	-0.4013 to -0.1134	Yes
14090 12:40 X vs. 19:1 X	-0.2453	-0.3893 to -0.1014	Yes
14091 12:40 X vs. 19:10 X	-0.2430	-0.3870 to -0.09904	Yes
14092 12:40 X vs. 19:40 X	-0.1010	-0.2450 to 0.04296	No
14093 12:40 X vs. 19:50 X	-0.1120	-0.2560 to 0.03196	No
14094 12:40 X vs. 20:0 X	-0.3370	-0.4810 to -0.1930	Yes
14095 12:40 X vs. 20:1 X	-0.1563	-0.3003 to -0.01237	Yes
14096 12:40 X vs. 20:10 X	-0.2453	-0.3893 to -0.1014	Yes
14097 12:40 X vs. 20:40 X	-0.1127	-0.2566 to 0.03129	No
14098 12:40 X vs. 20:50 X	-0.1567	-0.3006 to -0.01271	Yes
14099 12:40 X vs. 21:0 X	-0.3603	-0.5043 to -0.2164	Yes
14100 12:40 X vs. 21:1 X	-0.3133	-0.4573 to -0.1694	Yes
14101 12:40 X vs. 21:10 X	-0.3103	-0.4543 to -0.1664	Yes
14102 12:40 X vs. 21:40 X	-0.1857	-0.3296 to -0.04171	Yes
14103 12:40 X vs. 21:50 X	-0.2760	-0.4200 to -0.1320	Yes
14104 12:40 X vs. 22:0 X	-0.4020	-0.5460 to -0.2580	Yes
14105 12:40 X vs. 22:1 X	-0.3967	-0.5406 to -0.2527	Yes
14106 12:40 X vs. 22:10 X	-0.3920	-0.5360 to -0.2480	Yes
14107 12:40 X vs. 22:40 X	-0.2923	-0.4363 to -0.1484	Yes
14108 12:40 X vs. 22:50 X	-0.3550	-0.4990 to -0.2110	Yes
14109 12:40 X vs. 23:0 X	-0.4673	-0.6113 to -0.3234	Yes
14110 12:40 X vs. 23:1 X	-0.6570	-0.8180 to -0.4960	Yes
14111 12:40 X vs. 23:10 X	-0.4067	-0.5506 to -0.2627	Yes
14112 12:40 X vs. 23:40 X	-0.2870	-0.4480 to -0.1260	Yes
14113 12:40 X vs. 23:50 X	-0.3210	-0.4650 to -0.1770	Yes
14114 12:40 X vs. 24:0 X	-0.5847	-0.7286 to -0.4407	Yes
14115 12:40 X vs. 24:1 X	-0.7410	-0.9020 to -0.5800	Yes
14116 12:40 X vs. 24:10 X	-0.5685	-0.7295 to -0.4075	Yes
14117 12:40 X vs. 24:40 X	-0.3033	-0.4473 to -0.1594	Yes
14118 12:40 X vs. 24:50 X	-0.2250	-0.3690 to -0.08104	Yes
14119 12:40 X vs. 25:0 X	-0.9220	-1.083 to -0.7610	Yes
14120 12:40 X vs. 25:1 X	-1.112	-1.272 to -0.9505	Yes
14121 12:40 X vs. 25:10 X	-0.4730	-0.6766 to -0.2694	Yes
14122 12:40 X vs. 25:40 X	-0.3053	-0.4493 to -0.1614	Yes
14123 12:40 X vs. 25:50 X	-0.2797	-0.4236 to -0.1357	Yes
14124 12:40 X vs. 26:0 X	-1.135	-1.295 to -0.9735	Yes
14125 12:40 X vs. 26:1 X	-1.248	-1.408 to -1.087	Yes
14126 12:40 X vs. 26:10 X	-0.9340	-1.095 to -0.7730	Yes
14127 12:40 X vs. 26:40 X	-0.3797	-0.5236 to -0.2357	Yes
14128 12:40 X vs. 26:50 X	-0.2400	-0.3840 to -0.09604	Yes
14129 12:40 X vs. 27:0 X	-1.322	-1.466 to -1.178	Yes
14130 12:40 X vs. 27:1 X	-1.413	-1.574 to -1.252	Yes

2way ANOVA<br>Multiple comparisons	A<br>Data Set-A<br>Y	B<br>Data Set-B<br>Y	C<br>Data Set-C<br>Y
14131 12:40 X vs. 27:10 X	-1.225	-1.386 to -1.064	Yes
14132 12:40 X vs. 27:40 X	-0.3797	-0.5236 to -0.2357	Yes
14133 12:40 X vs. 27:50 X	-0.2393	-0.3833 to -0.09537	Yes
14134 12:40 X vs. 28:0 X	-1.272	-1.432 to -1.111	Yes
14135 12:40 X vs. 28:1 X	-1.312	-1.456 to -1.168	Yes
14136 12:40 X vs. 28:10 X	-1.114	-1.275 to -0.9530	Yes
14137 12:40 X vs. 28:40 X	-0.3920	-0.5360 to -0.2480	Yes
14138 12:40 X vs. 28:50 X	-0.2873	-0.4313 to -0.1434	Yes
14139 12:40 X vs. 29:0 X	-1.753	-1.914 to -1.592	Yes
14140 12:40 X vs. 29:1 X	-1.838	-1.999 to -1.677	Yes
14141 12:40 X vs. 29:10 X	-1.053	-1.213 to -0.8915	Yes
14142 12:40 X vs. 29:40 X	-0.5000	-0.6440 to -0.3560	Yes
14143 12:40 X vs. 29:50 X	-0.2887	-0.4326 to -0.1447	Yes
14144 12:40 X vs. 30:0 X	-1.468	-1.628 to -1.307	Yes
14145 12:40 X vs. 30:1 X	-1.789	-1.950 to -1.628	Yes
14146 12:40 X vs. 30:10 X	-1.127	-1.288 to -0.9660	Yes
14147 12:40 X vs. 30:40 X	-0.4270	-0.5710 to -0.2830	Yes
14148 12:40 X vs. 30:50 X	-0.3410	-0.4850 to -0.1970	Yes
14149 12:40 X vs. 31:0 X	-1.786	-1.946 to -1.625	Yes
14150 12:40 X vs. 31:1 X	-1.776	-1.937 to -1.615	Yes
14151 12:40 X vs. 31:10 X	-1.259	-1.419 to -1.098	Yes
14152 12:40 X vs. 31:40 X	-0.5187	-0.6626 to -0.3747	Yes
14153 12:40 X vs. 31:50 X	-0.3200	-0.4640 to -0.1760	Yes
14154 12:40 X vs. 32:0 X	-2.377	-2.521 to -2.233	Yes
14155 12:40 X vs. 32:1 X	-1.833	-1.994 to -1.672	Yes
14156 12:40 X vs. 32:10 X	-1.689	-1.833 to -1.545	Yes
14157 12:40 X vs. 32:40 X	-0.5150	-0.6590 to -0.3710	Yes
14158 12:40 X vs. 32:50 X	-0.3197	-0.4636 to -0.1757	Yes
14159 12:40 X vs. 33:0 X	-2.381	-2.525 to -2.237	Yes
14160 12:40 X vs. 33:1 X	-2.195	-2.339 to -2.051	Yes
14161 12:40 X vs. 33:10 X	-1.856	-2.000 to -1.712	Yes
14162 12:40 X vs. 33:40 X	-0.5337	-0.6776 to -0.3897	Yes
14163 12:40 X vs. 33:50 X	-0.3347	-0.4786 to -0.1907	Yes
14164 12:40 X vs. 34:0 X	-2.305	-2.449 to -2.161	Yes
14165 12:40 X vs. 34:1 X	-2.313	-2.457 to -2.169	Yes
14166 12:40 X vs. 34:10 X	-1.946	-2.090 to -1.802	Yes
14167 12:40 X vs. 34:40 X	-0.5457	-0.6896 to -0.4017	Yes
14168 12:40 X vs. 34:50 X	-0.4837	-0.6276 to -0.3397	Yes
14169 12:40 X vs. 35:0 X	-2.654	-2.798 to -2.510	Yes
14170 12:40 X vs. 35:1 X	-2.420	-2.564 to -2.276	Yes
14171 12:40 X vs. 35:10 X	-1.908	-2.052 to -1.764	Yes
14172 12:40 X vs. 35:40 X	-0.5547	-0.6986 to -0.4107	Yes
14173 12:40 X vs. 35:50 X	-0.4963	-0.6403 to -0.3524	Yes
14174 12:40 X vs. 36:0 X	-2.861	-3.021 to -2.700	Yes
14175 12:40 X vs. 36:1 X	-2.556	-2.700 to -2.412	Yes

	2way ANOVA<br>Multiple comparisons	A<br>Data Set-A<br>Y	B<br>Data Set-B<br>Y	C<br>Data Set-C<br>Y
14176	12:**40 X** vs. 36:**10 X**	-1.913	-2.057 to -1.769	Yes
14177	12:**40 X** vs. 36:**40 X**	-0.6100	-0.7540 to -0.4660	Yes
14178	12:**40 X** vs. 36:**50 X**	-0.4597	-0.6036 to -0.3157	Yes
14179	12:**40 X** vs. 37:**0 X**	-3.244	-3.388 to -3.100	Yes
14180	12:**40 X** vs. 37:**1 X**	-2.626	-2.770 to -2.482	Yes
14181	12:**40 X** vs. 37:**10 X**	-1.915	-2.059 to -1.771	Yes
14182	12:**40 X** vs. 37:**40 X**	-0.6473	-0.7913 to -0.5034	Yes
14183	12:**40 X** vs. 37:**50 X**	-0.4683	-0.6123 to -0.3244	Yes
14184	12:**40 X** vs. 38:**0 X**	-3.189	-3.333 to -3.045	Yes
14185	12:**40 X** vs. 38:**1 X**	-2.745	-2.889 to -2.601	Yes
14186	12:**40 X** vs. 38:**10 X**	-2.190	-2.334 to -2.046	Yes
14187	12:**40 X** vs. 38:**40 X**	-0.6517	-0.7956 to -0.5077	Yes
14188	12:**40 X** vs. 38:**50 X**	-0.4723	-0.6163 to -0.3284	Yes
14189	12:**40 X** vs. 39:**0 X**	-3.420	-3.564 to -3.276	Yes
14190	12:**40 X** vs. 39:**1 X**	-3.312	-3.456 to -3.168	Yes
14191	12:**40 X** vs. 39:**10 X**	-2.335	-2.479 to -2.191	Yes
14192	12:**40 X** vs. 39:**40 X**	-0.6577	-0.8016 to -0.5137	Yes
14193	12:**40 X** vs. 39:**50 X**	-0.5033	-0.6473 to -0.3594	Yes
14194	12:**40 X** vs. 40:**0 X**	-3.461	-3.605 to -3.317	Yes
14195	12:**40 X** vs. 40:**1 X**	-3.387	-3.531 to -3.243	Yes
14196	12:**40 X** vs. 40:**10 X**	-2.592	-2.736 to -2.448	Yes
14197	12:**40 X** vs. 40:**40 X**	-0.6623	-0.8063 to -0.5184	Yes
14198	12:**40 X** vs. 40:**50 X**	-0.5097	-0.6536 to -0.3657	Yes
14199	12:**40 X** vs. 41:**0 X**	-3.502	-3.646 to -3.358	Yes
14200	12:**40 X** vs. 41:**1 X**	-3.501	-3.645 to -3.357	Yes
14201	12:**40 X** vs. 41:**10 X**	-2.645	-2.789 to -2.501	Yes
14202	12:**40 X** vs. 41:**40 X**	-0.6097	-0.7536 to -0.4657	Yes
14203	12:**40 X** vs. 41:**50 X**	-0.5223	-0.6663 to -0.3784	Yes
14204	12:**40 X** vs. 42:**0 X**	-3.644	-3.788 to -3.500	Yes
14205	12:**40 X** vs. 42:**1 X**	-3.663	-3.807 to -3.519	Yes
14206	12:**40 X** vs. 42:**10 X**	-2.646	-2.790 to -2.502	Yes
14207	12:**40 X** vs. 42:**40 X**	-0.6223	-0.7663 to -0.4784	Yes
14208	12:**40 X** vs. 42:**50 X**	-0.5257	-0.6696 to -0.3817	Yes
14209	12:**40 X** vs. 43:**0 X**	-3.734	-3.878 to -3.590	Yes
14210	12:**40 X** vs. 43:**1 X**	-3.648	-3.792 to -3.504	Yes
14211	12:**40 X** vs. 43:**10 X**	-3.364	-3.508 to -3.220	Yes
14212	12:**40 X** vs. 43:**40 X**	-0.6207	-0.7646 to -0.4767	Yes
14213	12:**40 X** vs. 43:**50 X**	-0.5260	-0.6700 to -0.3820	Yes
14214	12:**40 X** vs. 44:**0 X**	-3.758	-3.902 to -3.614	Yes
14215	12:**40 X** vs. 44:**1 X**	-3.647	-3.791 to -3.503	Yes
14216	12:**40 X** vs. 44:**10 X**	-3.427	-3.571 to -3.283	Yes
14217	12:**40 X** vs. 44:**40 X**	-0.6227	-0.7666 to -0.4787	Yes
14218	12:**40 X** vs. 44:**50 X**	-0.4583	-0.6023 to -0.3144	Yes
14219	12:**40 X** vs. 45:**0 X**	-3.622	-3.766 to -3.478	Yes
14220	12:**40 X** vs. 45:**1 X**	-3.424	-3.568 to -3.280	Yes

	2way ANOVA<br>Multiple comparisons	A<br>Data Set-A<br>Y	B<br>Data Set-B<br>Y	C<br>Data Set-C<br>Y
14221	12:**40 X** vs. 45:**10 X**	-3.307	-3.451 to -3.163	Yes
14222	12:**40 X** vs. 45:**40 X**	-0.6240	-0.7680 to -0.4800	Yes
14223	12:**40 X** vs. 45:**50 X**	-0.4553	-0.5993 to -0.3114	Yes
14224	12:**40 X** vs. 46:**0 X**	-3.427	-3.571 to -3.283	Yes
14225	12:**40 X** vs. 46:**1 X**	-3.394	-3.538 to -3.250	Yes
14226	12:**40 X** vs. 46:**10 X**	-3.302	-3.446 to -3.158	Yes
14227	12:**40 X** vs. 46:**40 X**	-0.6377	-0.7816 to -0.4937	Yes
14228	12:**40 X** vs. 46:**50 X**	-0.4483	-0.5923 to -0.3044	Yes
14229	12:**40 X** vs. 47:**0 X**	-3.402	-3.546 to -3.258	Yes
14230	12:**40 X** vs. 47:**1 X**	-3.306	-3.450 to -3.162	Yes
14231	12:**40 X** vs. 47:**10 X**	-3.288	-3.432 to -3.144	Yes
14232	12:**40 X** vs. 47:**40 X**	-0.6477	-0.7916 to -0.5037	Yes
14233	12:**40 X** vs. 47:**50 X**	-0.4063	-0.5503 to -0.2624	Yes
14234	12:**40 X** vs. 48:**0 X**	-3.390	-3.550 to -3.229	Yes
14235	12:**40 X** vs. 48:**1 X**	-3.293	-3.437 to -3.149	Yes
14236	12:**40 X** vs. 48:**10 X**	-3.288	-3.432 to -3.144	Yes
14237	12:**40 X** vs. 48:**40 X**	-0.6540	-0.7980 to -0.5100	Yes
14238	12:**40 X** vs. 48:**50 X**	-0.4137	-0.5576 to -0.2697	Yes
14239	12:**40 X** vs. 49:**0 X**	-3.390	-3.534 to -3.246	Yes
14240	12:**40 X** vs. 49:**1 X**	-3.293	-3.437 to -3.149	Yes
14241	12:**40 X** vs. 49:**10 X**	-3.288	-3.432 to -3.144	Yes
14242	12:**40 X** vs. 49:**40 X**	-0.6583	-0.8023 to -0.5144	Yes
14243	12:**40 X** vs. 49:**50 X**	-0.4130	-0.5570 to -0.2690	Yes
14244	12:**40 X** vs. 50:**0 X**	-3.390	-3.534 to -3.246	Yes
14245	12:**40 X** vs. 50:**1 X**	-3.293	-3.437 to -3.149	Yes
14246	12:**40 X** vs. 50:**10 X**	-3.288	-3.432 to -3.144	Yes
14247	12:**40 X** vs. 50:**40 X**	-0.6660	-0.8100 to -0.5220	Yes
14248	12:**40 X** vs. 50:**50 X**	-0.4127	-0.5566 to -0.2687	Yes
14249	12:**50 X** vs. 13:**0 X**	-0.004333	-0.1483 to 0.1396	No
14250	12:**50 X** vs. 13:**1 X**	0.008333	-0.1356 to 0.1523	No
14251	12:**50 X** vs. 13:**10 X**	-0.01367	-0.1576 to 0.1303	No
14252	12:**50 X** vs. 13:**40 X**	-0.01000	-0.1540 to 0.1340	No
14253	12:**50 X** vs. 13:**50 X**	-0.0110	-0.1550 to 0.1330	No
14254	12:**50 X** vs. 14:**0 X**	-0.0130	-0.1570 to 0.1310	No
14255	12:**50 X** vs. 14:**1 X**	-0.004667	-0.1486 to 0.1393	No
14256	12:**50 X** vs. 14:**10 X**	-0.01867	-0.1626 to 0.1253	No
14257	12:**50 X** vs. 14:**40 X**	-0.01533	-0.1593 to 0.1286	No
14258	12:**50 X** vs. 14:**50 X**	-0.01933	-0.1633 to 0.1246	No
14259	12:**50 X** vs. 15:**0 X**	-0.02533	-0.1693 to 0.1186	No
14260	12:**50 X** vs. 15:**1 X**	-0.005333	-0.1493 to 0.1386	No
14261	12:**50 X** vs. 15:**10 X**	-0.03267	-0.1766 to 0.1113	No
14262	12:**50 X** vs. 15:**40 X**	-0.0150	-0.1590 to 0.1290	No
14263	12:**50 X** vs. 15:**50 X**	-0.03267	-0.1766 to 0.1113	No
14264	12:**50 X** vs. 16:**0 X**	-0.1177	-0.2616 to 0.02629	No
14265	12:**50 X** vs. 16:**1 X**	-0.008667	-0.1526 to 0.1353	No

2way ANOVA<br>Multiple comparisons	A<br>Data Set-A<br>Y	B<br>Data Set-B<br>Y	C<br>Data Set-C<br>Y
14266 12:**50 X** vs. 16:**10 X**	-0.03433	-0.1783 to 0.1096	No
14267 12:**50 X** vs. 16:**40 X**	-0.05367	-0.1976 to 0.09029	No
14268 12:**50 X** vs. 16:**50 X**	-0.09600	-0.2400 to 0.04796	No
14269 12:**50 X** vs. 17:**0 X**	-0.1703	-0.3143 to -0.02637	Yes
14270 12:**50 X** vs. 17:**1 X**	-0.08467	-0.2286 to 0.05929	No
14271 12:**50 X** vs. 17:**10 X**	-0.1413	-0.2853 to 0.002626	No
14272 12:**50 X** vs. 17:**40 X**	-0.06467	-0.2086 to 0.07929	No
14273 12:**50 X** vs. 17:**50 X**	-0.1100	-0.2540 to 0.03396	No
14274 12:**50 X** vs. 18:**0 X**	-0.2190	-0.3630 to -0.07504	Yes
14275 12:**50 X** vs. 18:**1 X**	-0.1273	-0.2713 to 0.01663	No
14276 12:**50 X** vs. 18:**10 X**	-0.2197	-0.3636 to -0.07571	Yes
14277 12:**50 X** vs. 18:**40 X**	-0.0690	-0.2130 to 0.07496	No
14278 12:**50 X** vs. 18:**50 X**	-0.1140	-0.2580 to 0.02996	No
14279 12:**50 X** vs. 19:**0 X**	-0.2540	-0.3980 to -0.1100	Yes
14280 12:**50 X** vs. 19:**1 X**	-0.2420	-0.3860 to -0.09804	Yes
14281 12:**50 X** vs. 19:**10 X**	-0.2397	-0.3836 to -0.09571	Yes
14282 12:**50 X** vs. 19:**40 X**	-0.09767	-0.2416 to 0.04629	No
14283 12:**50 X** vs. 19:**50 X**	-0.1087	-0.2526 to 0.03529	No
14284 12:**50 X** vs. 20:**0 X**	-0.3337	-0.4776 to -0.1897	Yes
14285 12:**50 X** vs. 20:**1 X**	-0.1530	-0.2970 to -0.009040	Yes
14286 12:**50 X** vs. 20:**10 X**	-0.2420	-0.3860 to -0.09804	Yes
14287 12:**50 X** vs. 20:**40 X**	-0.1093	-0.2533 to 0.03463	No
14288 12:**50 X** vs. 20:**50 X**	-0.1533	-0.2973 to -0.009374	Yes
14289 12:**50 X** vs. 21:**0 X**	-0.3570	-0.5010 to -0.2130	Yes
14290 12:**50 X** vs. 21:**1 X**	-0.3100	-0.4540 to -0.1660	Yes
14291 12:**50 X** vs. 21:**10 X**	-0.3070	-0.4510 to -0.1630	Yes
14292 12:**50 X** vs. 21:**40 X**	-0.1823	-0.3263 to -0.03837	Yes
14293 12:**50 X** vs. 21:**50 X**	-0.2727	-0.4166 to -0.1287	Yes
14294 12:**50 X** vs. 22:**0 X**	-0.3987	-0.5426 to -0.2547	Yes
14295 12:**50 X** vs. 22:**1 X**	-0.3933	-0.5373 to -0.2494	Yes
14296 12:**50 X** vs. 22:**10 X**	-0.3887	-0.5326 to -0.2447	Yes
14297 12:**50 X** vs. 22:**40 X**	-0.2890	-0.4330 to -0.1450	Yes
14298 12:**50 X** vs. 22:**50 X**	-0.3517	-0.4956 to -0.2077	Yes
14299 12:**50 X** vs. 23:**0 X**	-0.4640	-0.6080 to -0.3200	Yes
14300 12:**50 X** vs. 23:**1 X**	-0.6537	-0.8146 to -0.4927	Yes
14301 12:**50 X** vs. 23:**10 X**	-0.4033	-0.5473 to -0.2594	Yes
14302 12:**50 X** vs. 23:**40 X**	-0.2837	-0.4446 to -0.1227	Yes
14303 12:**50 X** vs. 23:**50 X**	-0.3177	-0.4616 to -0.1737	Yes
14304 12:**50 X** vs. 24:**0 X**	-0.5813	-0.7253 to -0.4374	Yes
14305 12:**50 X** vs. 24:**1 X**	-0.7377	-0.8986 to -0.5767	Yes
14306 12:**50 X** vs. 24:**10 X**	-0.5652	-0.7261 to -0.4042	Yes
14307 12:**50 X** vs. 24:**40 X**	-0.3000	-0.4440 to -0.1560	Yes
14308 12:**50 X** vs. 24:**50 X**	-0.2217	-0.3656 to -0.07771	Yes
14309 12:**50 X** vs. 25:**0 X**	-0.9187	-1.080 to -0.7577	Yes
14310 12:**50 X** vs. 25:**1 X**	-1.108	-1.269 to -0.9472	Yes

	2way ANOVA Multiple comparisons	A Data Set-A Y	B Data Set-B Y	C Data Set-C Y
14311	12:50 X vs. 25:10 X	-0.4697	-0.6733 to -0.2661	Yes
14312	12:50 X vs. 25:40 X	-0.3020	-0.4460 to -0.1580	Yes
14313	12:50 X vs. 25:50 X	-0.2763	-0.4203 to -0.1324	Yes
14314	12:50 X vs. 26:0 X	-1.131	-1.292 to -0.9702	Yes
14315	12:50 X vs. 26:1 X	-1.244	-1.405 to -1.083	Yes
14316	12:50 X vs. 26:10 X	-0.9307	-1.092 to -0.7697	Yes
14317	12:50 X vs. 26:40 X	-0.3763	-0.5203 to -0.2324	Yes
14318	12:50 X vs. 26:50 X	-0.2367	-0.3806 to -0.09271	Yes
14319	12:50 X vs. 27:0 X	-1.319	-1.463 to -1.175	Yes
14320	12:50 X vs. 27:1 X	-1.410	-1.571 to -1.249	Yes
14321	12:50 X vs. 27:10 X	-1.222	-1.383 to -1.061	Yes
14322	12:50 X vs. 27:40 X	-0.3763	-0.5203 to -0.2324	Yes
14323	12:50 X vs. 27:50 X	-0.2360	-0.3800 to -0.09204	Yes
14324	12:50 X vs. 28:0 X	-1.268	-1.429 to -1.107	Yes
14325	12:50 X vs. 28:1 X	-1.309	-1.453 to -1.165	Yes
14326	12:50 X vs. 28:10 X	-1.111	-1.272 to -0.9497	Yes
14327	12:50 X vs. 28:40 X	-0.3887	-0.5326 to -0.2447	Yes
14328	12:50 X vs. 28:50 X	-0.2840	-0.4280 to -0.1400	Yes
14329	12:50 X vs. 29:0 X	-1.750	-1.911 to -1.589	Yes
14330	12:50 X vs. 29:1 X	-1.835	-1.996 to -1.674	Yes
14331	12:50 X vs. 29:10 X	-1.049	-1.210 to -0.8882	Yes
14332	12:50 X vs. 29:40 X	-0.4967	-0.6406 to -0.3527	Yes
14333	12:50 X vs. 29:50 X	-0.2853	-0.4293 to -0.1414	Yes
14334	12:50 X vs. 30:0 X	-1.464	-1.625 to -1.303	Yes
14335	12:50 X vs. 30:1 X	-1.786	-1.947 to -1.625	Yes
14336	12:50 X vs. 30:10 X	-1.124	-1.285 to -0.9627	Yes
14337	12:50 X vs. 30:40 X	-0.4237	-0.5676 to -0.2797	Yes
14338	12:50 X vs. 30:50 X	-0.3377	-0.4816 to -0.1937	Yes
14339	12:50 X vs. 31:0 X	-1.782	-1.943 to -1.621	Yes
14340	12:50 X vs. 31:1 X	-1.773	-1.934 to -1.612	Yes
14341	12:50 X vs. 31:10 X	-1.255	-1.416 to -1.094	Yes
14342	12:50 X vs. 31:40 X	-0.5153	-0.6593 to -0.3714	Yes
14343	12:50 X vs. 31:50 X	-0.3167	-0.4606 to -0.1727	Yes
14344	12:50 X vs. 32:0 X	-2.374	-2.518 to -2.230	Yes
14345	12:50 X vs. 32:1 X	-1.830	-1.991 to -1.669	Yes
14346	12:50 X vs. 32:10 X	-1.686	-1.830 to -1.542	Yes
14347	12:50 X vs. 32:40 X	-0.5117	-0.6556 to -0.3677	Yes
14348	12:50 X vs. 32:50 X	-0.3163	-0.4603 to -0.1724	Yes
14349	12:50 X vs. 33:0 X	-2.377	-2.521 to -2.233	Yes
14350	12:50 X vs. 33:1 X	-2.192	-2.336 to -2.048	Yes
14351	12:50 X vs. 33:10 X	-1.852	-1.996 to -1.708	Yes
14352	12:50 X vs. 33:40 X	-0.5303	-0.6743 to -0.3864	Yes
14353	12:50 X vs. 33:50 X	-0.3313	-0.4753 to -0.1874	Yes
14354	12:50 X vs. 34:0 X	-2.302	-2.446 to -2.158	Yes
14355	12:50 X vs. 34:1 X	-2.309	-2.453 to -2.165	Yes

2way ANOVA<br>Multiple comparisons	A<br>Data Set-A<br>Y	B<br>Data Set-B<br>Y	C<br>Data Set-C<br>Y
14356 12:50 X vs. 34:10 X	-1.943	-2.087 to -1.799	Yes
14357 12:50 X vs. 34:40 X	-0.5423	-0.6863 to -0.3984	Yes
14358 12:50 X vs. 34:50 X	-0.4803	-0.6243 to -0.3364	Yes
14359 12:50 X vs. 35:0 X	-2.651	-2.795 to -2.507	Yes
14360 12:50 X vs. 35:1 X	-2.416	-2.560 to -2.272	Yes
14361 12:50 X vs. 35:10 X	-1.904	-2.048 to -1.760	Yes
14362 12:50 X vs. 35:40 X	-0.5513	-0.6953 to -0.4074	Yes
14363 12:50 X vs. 35:50 X	-0.4930	-0.6370 to -0.3490	Yes
14364 12:50 X vs. 36:0 X	-2.857	-3.018 to -2.696	Yes
14365 12:50 X vs. 36:1 X	-2.553	-2.697 to -2.409	Yes
14366 12:50 X vs. 36:10 X	-1.910	-2.054 to -1.766	Yes
14367 12:50 X vs. 36:40 X	-0.6067	-0.7506 to -0.4627	Yes
14368 12:50 X vs. 36:50 X	-0.4563	-0.6003 to -0.3124	Yes
14369 12:50 X vs. 37:0 X	-3.241	-3.385 to -3.097	Yes
14370 12:50 X vs. 37:1 X	-2.623	-2.767 to -2.479	Yes
14371 12:50 X vs. 37:10 X	-1.912	-2.056 to -1.768	Yes
14372 12:50 X vs. 37:40 X	-0.6440	-0.7880 to -0.5000	Yes
14373 12:50 X vs. 37:50 X	-0.4650	-0.6090 to -0.3210	Yes
14374 12:50 X vs. 38:0 X	-3.186	-3.330 to -3.042	Yes
14375 12:50 X vs. 38:1 X	-2.742	-2.886 to -2.598	Yes
14376 12:50 X vs. 38:10 X	-2.187	-2.331 to -2.043	Yes
14377 12:50 X vs. 38:40 X	-0.6483	-0.7923 to -0.5044	Yes
14378 12:50 X vs. 38:50 X	-0.4690	-0.6130 to -0.3250	Yes
14379 12:50 X vs. 39:0 X	-3.417	-3.561 to -3.273	Yes
14380 12:50 X vs. 39:1 X	-3.308	-3.452 to -3.164	Yes
14381 12:50 X vs. 39:10 X	-2.332	-2.476 to -2.188	Yes
14382 12:50 X vs. 39:40 X	-0.6543	-0.7983 to -0.5104	Yes
14383 12:50 X vs. 39:50 X	-0.5000	-0.6440 to -0.3560	Yes
14384 12:50 X vs. 40:0 X	-3.458	-3.602 to -3.314	Yes
14385 12:50 X vs. 40:1 X	-3.384	-3.528 to -3.240	Yes
14386 12:50 X vs. 40:10 X	-2.588	-2.732 to -2.444	Yes
14387 12:50 X vs. 40:40 X	-0.6590	-0.8030 to -0.5150	Yes
14388 12:50 X vs. 40:50 X	-0.5063	-0.6503 to -0.3624	Yes
14389 12:50 X vs. 41:0 X	-3.499	-3.643 to -3.355	Yes
14390 12:50 X vs. 41:1 X	-3.498	-3.642 to -3.354	Yes
14391 12:50 X vs. 41:10 X	-2.642	-2.786 to -2.498	Yes
14392 12:50 X vs. 41:40 X	-0.6063	-0.7503 to -0.4624	Yes
14393 12:50 X vs. 41:50 X	-0.5190	-0.6630 to -0.3750	Yes
14394 12:50 X vs. 42:0 X	-3.640	-3.784 to -3.496	Yes
14395 12:50 X vs. 42:1 X	-3.660	-3.804 to -3.516	Yes
14396 12:50 X vs. 42:10 X	-2.642	-2.786 to -2.498	Yes
14397 12:50 X vs. 42:40 X	-0.6190	-0.7630 to -0.4750	Yes
14398 12:50 X vs. 42:50 X	-0.5223	-0.6663 to -0.3784	Yes
14399 12:50 X vs. 43:0 X	-3.730	-3.874 to -3.586	Yes
14400 12:50 X vs. 43:1 X	-3.645	-3.789 to -3.501	Yes

	2way ANOVA Multiple comparisons	A Data Set-A Y	B Data Set-B Y	C Data Set-C Y
14401	12:**50 X** vs. 43:**10 X**	-3.360	-3.504 to -3.216	Yes
14402	12:**50 X** vs. 43:**40 X**	-0.6173	-0.7613 to -0.4734	Yes
14403	12:**50 X** vs. 43:**50 X**	-0.5227	-0.6666 to -0.3787	Yes
14404	12:**50 X** vs. 44:**0 X**	-3.754	-3.898 to -3.610	Yes
14405	12:**50 X** vs. 44:**1 X**	-3.644	-3.788 to -3.500	Yes
14406	12:**50 X** vs. 44:**10 X**	-3.424	-3.568 to -3.280	Yes
14407	12:**50 X** vs. 44:**40 X**	-0.6193	-0.7633 to -0.4754	Yes
14408	12:**50 X** vs. 44:**50 X**	-0.4550	-0.5990 to -0.3110	Yes
14409	12:**50 X** vs. 45:**0 X**	-3.619	-3.763 to -3.475	Yes
14410	12:**50 X** vs. 45:**1 X**	-3.421	-3.565 to -3.277	Yes
14411	12:**50 X** vs. 45:**10 X**	-3.303	-3.447 to -3.159	Yes
14412	12:**50 X** vs. 45:**40 X**	-0.6207	-0.7646 to -0.4767	Yes
14413	12:**50 X** vs. 45:**50 X**	-0.4520	-0.5960 to -0.3080	Yes
14414	12:**50 X** vs. 46:**0 X**	-3.423	-3.567 to -3.279	Yes
14415	12:**50 X** vs. 46:**1 X**	-3.390	-3.534 to -3.246	Yes
14416	12:**50 X** vs. 46:**10 X**	-3.298	-3.442 to -3.154	Yes
14417	12:**50 X** vs. 46:**40 X**	-0.6343	-0.7783 to -0.4904	Yes
14418	12:**50 X** vs. 46:**50 X**	-0.4450	-0.5890 to -0.3010	Yes
14419	12:**50 X** vs. 47:**0 X**	-3.399	-3.543 to -3.255	Yes
14420	12:**50 X** vs. 47:**1 X**	-3.303	-3.447 to -3.159	Yes
14421	12:**50 X** vs. 47:**10 X**	-3.284	-3.428 to -3.140	Yes
14422	12:**50 X** vs. 47:**40 X**	-0.6443	-0.7883 to -0.5004	Yes
14423	12:**50 X** vs. 47:**50 X**	-0.4030	-0.5470 to -0.2590	Yes
14424	12:**50 X** vs. 48:**0 X**	-3.386	-3.547 to -3.225	Yes
14425	12:**50 X** vs. 48:**1 X**	-3.290	-3.434 to -3.146	Yes
14426	12:**50 X** vs. 48:**10 X**	-3.284	-3.428 to -3.140	Yes
14427	12:**50 X** vs. 48:**40 X**	-0.6507	-0.7946 to -0.5067	Yes
14428	12:**50 X** vs. 48:**50 X**	-0.4103	-0.5543 to -0.2664	Yes
14429	12:**50 X** vs. 49:**0 X**	-3.386	-3.530 to -3.242	Yes
14430	12:**50 X** vs. 49:**1 X**	-3.290	-3.434 to -3.146	Yes
14431	12:**50 X** vs. 49:**10 X**	-3.284	-3.428 to -3.140	Yes
14432	12:**50 X** vs. 49:**40 X**	-0.6550	-0.7990 to -0.5110	Yes
14433	12:**50 X** vs. 49:**50 X**	-0.4097	-0.5536 to -0.2657	Yes
14434	12:**50 X** vs. 50:**0 X**	-3.386	-3.530 to -3.242	Yes
14435	12:**50 X** vs. 50:**1 X**	-3.290	-3.434 to -3.146	Yes
14436	12:**50 X** vs. 50:**10 X**	-3.284	-3.428 to -3.140	Yes
14437	12:**50 X** vs. 50:**40 X**	-0.6627	-0.8066 to -0.5187	Yes
14438	12:**50 X** vs. 50:**50 X**	-0.4093	-0.5533 to -0.2654	Yes
14439	13:**0 X** vs. 13:**1 X**	0.01267	-0.1313 to 0.1566	No
14440	13:**0 X** vs. 13:**10 X**	-0.009333	-0.1533 to 0.1346	No
14441	13:**0 X** vs. 13:**40 X**	-0.005667	-0.1496 to 0.1383	No
14442	13:**0 X** vs. 13:**50 X**	-0.006667	-0.1506 to 0.1373	No
14443	13:**0 X** vs. 14:**0 X**	-0.008667	-0.1526 to 0.1353	No
14444	13:**0 X** vs. 14:**1 X**	-0.0003333	-0.1443 to 0.1436	No
14445	13:**0 X** vs. 14:**10 X**	-0.01433	-0.1583 to 0.1296	No

2way ANOVA<br>Multiple comparisons	A<br>Data Set-A<br>Y	B<br>Data Set-B<br>Y	C<br>Data Set-C<br>Y
14446 13:0 X vs. 14:40 X	-0.0110	-0.1550 to 0.1330	No
14447 13:0 X vs. 14:50 X	-0.0150	-0.1590 to 0.1290	No
14448 13:0 X vs. 15:0 X	-0.0210	-0.1650 to 0.1230	No
14449 13:0 X vs. 15:1 X	-0.001000	-0.1450 to 0.1430	No
14450 13:0 X vs. 15:10 X	-0.02833	-0.1723 to 0.1156	No
14451 13:0 X vs. 15:40 X	-0.01067	-0.1546 to 0.1333	No
14452 13:0 X vs. 15:50 X	-0.02833	-0.1723 to 0.1156	No
14453 13:0 X vs. 16:0 X	-0.1133	-0.2573 to 0.03063	No
14454 13:0 X vs. 16:1 X	-0.004333	-0.1483 to 0.1396	No
14455 13:0 X vs. 16:10 X	-0.0300	-0.1740 to 0.1140	No
14456 13:0 X vs. 16:40 X	-0.04933	-0.1933 to 0.09463	No
14457 13:0 X vs. 16:50 X	-0.09167	-0.2356 to 0.05229	No
14458 13:0 X vs. 17:0 X	-0.1660	-0.3100 to -0.02204	Yes
14459 13:0 X vs. 17:1 X	-0.08033	-0.2243 to 0.06363	No
14460 13:0 X vs. 17:10 X	-0.1370	-0.2810 to 0.006960	No
14461 13:0 X vs. 17:40 X	-0.06033	-0.2043 to 0.08363	No
14462 13:0 X vs. 17:50 X	-0.1057	-0.2496 to 0.03829	No
14463 13:0 X vs. 18:0 X	-0.2147	-0.3586 to -0.07071	Yes
14464 13:0 X vs. 18:1 X	-0.1230	-0.2670 to 0.02096	No
14465 13:0 X vs. 18:10 X	-0.2153	-0.3593 to -0.07137	Yes
14466 13:0 X vs. 18:40 X	-0.06467	-0.2086 to 0.07929	No
14467 13:0 X vs. 18:50 X	-0.1097	-0.2536 to 0.03429	No
14468 13:0 X vs. 19:0 X	-0.2497	-0.3936 to -0.1057	Yes
14469 13:0 X vs. 19:1 X	-0.2377	-0.3816 to -0.09371	Yes
14470 13:0 X vs. 19:10 X	-0.2353	-0.3793 to -0.09137	Yes
14471 13:0 X vs. 19:40 X	-0.09333	-0.2373 to 0.05063	No
14472 13:0 X vs. 19:50 X	-0.1043	-0.2483 to 0.03963	No
14473 13:0 X vs. 20:0 X	-0.3293	-0.4733 to -0.1854	Yes
14474 13:0 X vs. 20:1 X	-0.1487	-0.2926 to -0.004707	Yes
14475 13:0 X vs. 20:10 X	-0.2377	-0.3816 to -0.09371	Yes
14476 13:0 X vs. 20:40 X	-0.1050	-0.2490 to 0.03896	No
14477 13:0 X vs. 20:50 X	-0.1490	-0.2930 to -0.005040	Yes
14478 13:0 X vs. 21:0 X	-0.3527	-0.4966 to -0.2087	Yes
14479 13:0 X vs. 21:1 X	-0.3057	-0.4496 to -0.1617	Yes
14480 13:0 X vs. 21:10 X	-0.3027	-0.4466 to -0.1587	Yes
14481 13:0 X vs. 21:40 X	-0.1780	-0.3220 to -0.03404	Yes
14482 13:0 X vs. 21:50 X	-0.2683	-0.4123 to -0.1244	Yes
14483 13:0 X vs. 22:0 X	-0.3943	-0.5383 to -0.2504	Yes
14484 13:0 X vs. 22:1 X	-0.3890	-0.5330 to -0.2450	Yes
14485 13:0 X vs. 22:10 X	-0.3843	-0.5283 to -0.2404	Yes
14486 13:0 X vs. 22:40 X	-0.2847	-0.4286 to -0.1407	Yes
14487 13:0 X vs. 22:50 X	-0.3473	-0.4913 to -0.2034	Yes
14488 13:0 X vs. 23:0 X	-0.4597	-0.6036 to -0.3157	Yes
14489 13:0 X vs. 23:1 X	-0.6493	-0.8103 to -0.4884	Yes
14490 13:0 X vs. 23:10 X	-0.3990	-0.5430 to -0.2550	Yes

	2way ANOVA<br>Multiple comparisons	A<br>Data Set-A<br>Y	B<br>Data Set-B<br>Y	C<br>Data Set-C<br>Y
14491	13:**0 X** vs. 23:**40 X**	-0.2793	-0.4403 to -0.1184	Yes
14492	13:**0 X** vs. 23:**50 X**	-0.3133	-0.4573 to -0.1694	Yes
14493	13:**0 X** vs. 24:**0 X**	-0.5770	-0.7210 to -0.4330	Yes
14494	13:**0 X** vs. 24:**1 X**	-0.7333	-0.8943 to -0.5724	Yes
14495	13:**0 X** vs. 24:**10 X**	-0.5608	-0.7218 to -0.3999	Yes
14496	13:**0 X** vs. 24:**40 X**	-0.2957	-0.4396 to -0.1517	Yes
14497	13:**0 X** vs. 24:**50 X**	-0.2173	-0.3613 to -0.07337	Yes
14498	13:**0 X** vs. 25:**0 X**	-0.9143	-1.075 to -0.7534	Yes
14499	13:**0 X** vs. 25:**1 X**	-1.104	-1.265 to -0.9429	Yes
14500	13:**0 X** vs. 25:**10 X**	-0.4653	-0.6689 to -0.2617	Yes
14501	13:**0 X** vs. 25:**40 X**	-0.2977	-0.4416 to -0.1537	Yes
14502	13:**0 X** vs. 25:**50 X**	-0.2720	-0.4160 to -0.1280	Yes
14503	13:**0 X** vs. 26:**0 X**	-1.127	-1.288 to -0.9659	Yes
14504	13:**0 X** vs. 26:**1 X**	-1.240	-1.401 to -1.079	Yes
14505	13:**0 X** vs. 26:**10 X**	-0.9263	-1.087 to -0.7654	Yes
14506	13:**0 X** vs. 26:**40 X**	-0.3720	-0.5160 to -0.2280	Yes
14507	13:**0 X** vs. 26:**50 X**	-0.2323	-0.3763 to -0.08837	Yes
14508	13:**0 X** vs. 27:**0 X**	-1.315	-1.459 to -1.171	Yes
14509	13:**0 X** vs. 27:**1 X**	-1.405	-1.566 to -1.244	Yes
14510	13:**0 X** vs. 27:**10 X**	-1.217	-1.378 to -1.056	Yes
14511	13:**0 X** vs. 27:**40 X**	-0.3720	-0.5160 to -0.2280	Yes
14512	13:**0 X** vs. 27:**50 X**	-0.2317	-0.3756 to -0.08771	Yes
14513	13:**0 X** vs. 28:**0 X**	-1.264	-1.425 to -1.103	Yes
14514	13:**0 X** vs. 28:**1 X**	-1.305	-1.449 to -1.161	Yes
14515	13:**0 X** vs. 28:**10 X**	-1.106	-1.267 to -0.9454	Yes
14516	13:**0 X** vs. 28:**40 X**	-0.3843	-0.5283 to -0.2404	Yes
14517	13:**0 X** vs. 28:**50 X**	-0.2797	-0.4236 to -0.1357	Yes
14518	13:**0 X** vs. 29:**0 X**	-1.745	-1.906 to -1.584	Yes
14519	13:**0 X** vs. 29:**1 X**	-1.830	-1.991 to -1.669	Yes
14520	13:**0 X** vs. 29:**10 X**	-1.045	-1.206 to -0.8839	Yes
14521	13:**0 X** vs. 29:**40 X**	-0.4923	-0.6363 to -0.3484	Yes
14522	13:**0 X** vs. 29:**50 X**	-0.2810	-0.4250 to -0.1370	Yes
14523	13:**0 X** vs. 30:**0 X**	-1.460	-1.621 to -1.299	Yes
14524	13:**0 X** vs. 30:**1 X**	-1.781	-1.942 to -1.620	Yes
14525	13:**0 X** vs. 30:**10 X**	-1.119	-1.280 to -0.9584	Yes
14526	13:**0 X** vs. 30:**40 X**	-0.4193	-0.5633 to -0.2754	Yes
14527	13:**0 X** vs. 30:**50 X**	-0.3333	-0.4773 to -0.1894	Yes
14528	13:**0 X** vs. 31:**0 X**	-1.778	-1.939 to -1.617	Yes
14529	13:**0 X** vs. 31:**1 X**	-1.768	-1.929 to -1.607	Yes
14530	13:**0 X** vs. 31:**10 X**	-1.251	-1.412 to -1.090	Yes
14531	13:**0 X** vs. 31:**40 X**	-0.5110	-0.6550 to -0.3670	Yes
14532	13:**0 X** vs. 31:**50 X**	-0.3123	-0.4563 to -0.1684	Yes
14533	13:**0 X** vs. 32:**0 X**	-2.369	-2.513 to -2.225	Yes
14534	13:**0 X** vs. 32:**1 X**	-1.825	-1.986 to -1.664	Yes
14535	13:**0 X** vs. 32:**10 X**	-1.681	-1.825 to -1.537	Yes

	2way ANOVA<br>Multiple comparisons	A<br>Data Set-A<br>Y	B<br>Data Set-B<br>Y	C<br>Data Set-C<br>Y
14536	13:**0 X** vs. 32:**40 X**	-0.5073	-0.6513 to -0.3634	Yes
14537	13:**0 X** vs. 32:**50 X**	-0.3120	-0.4560 to -0.1680	Yes
14538	13:**0 X** vs. 33:**0 X**	-2.373	-2.517 to -2.229	Yes
14539	13:**0 X** vs. 33:**1 X**	-2.188	-2.332 to -2.044	Yes
14540	13:**0 X** vs. 33:**10 X**	-1.848	-1.992 to -1.704	Yes
14541	13:**0 X** vs. 33:**40 X**	-0.5260	-0.6700 to -0.3820	Yes
14542	13:**0 X** vs. 33:**50 X**	-0.3270	-0.4710 to -0.1830	Yes
14543	13:**0 X** vs. 34:**0 X**	-2.298	-2.442 to -2.154	Yes
14544	13:**0 X** vs. 34:**1 X**	-2.305	-2.449 to -2.161	Yes
14545	13:**0 X** vs. 34:**10 X**	-1.938	-2.082 to -1.794	Yes
14546	13:**0 X** vs. 34:**40 X**	-0.5380	-0.6820 to -0.3940	Yes
14547	13:**0 X** vs. 34:**50 X**	-0.4760	-0.6200 to -0.3320	Yes
14548	13:**0 X** vs. 35:**0 X**	-2.646	-2.790 to -2.502	Yes
14549	13:**0 X** vs. 35:**1 X**	-2.412	-2.556 to -2.268	Yes
14550	13:**0 X** vs. 35:**10 X**	-1.900	-2.044 to -1.756	Yes
14551	13:**0 X** vs. 35:**40 X**	-0.5470	-0.6910 to -0.4030	Yes
14552	13:**0 X** vs. 35:**50 X**	-0.4887	-0.6326 to -0.3447	Yes
14553	13:**0 X** vs. 36:**0 X**	-2.853	-3.014 to -2.692	Yes
14554	13:**0 X** vs. 36:**1 X**	-2.549	-2.693 to -2.405	Yes
14555	13:**0 X** vs. 36:**10 X**	-1.906	-2.050 to -1.762	Yes
14556	13:**0 X** vs. 36:**40 X**	-0.6023	-0.7463 to -0.4584	Yes
14557	13:**0 X** vs. 36:**50 X**	-0.4520	-0.5960 to -0.3080	Yes
14558	13:**0 X** vs. 37:**0 X**	-3.237	-3.381 to -3.093	Yes
14559	13:**0 X** vs. 37:**1 X**	-2.619	-2.763 to -2.475	Yes
14560	13:**0 X** vs. 37:**10 X**	-1.907	-2.051 to -1.763	Yes
14561	13:**0 X** vs. 37:**40 X**	-0.6397	-0.7836 to -0.4957	Yes
14562	13:**0 X** vs. 37:**50 X**	-0.4607	-0.6046 to -0.3167	Yes
14563	13:**0 X** vs. 38:**0 X**	-3.182	-3.326 to -3.038	Yes
14564	13:**0 X** vs. 38:**1 X**	-2.737	-2.881 to -2.593	Yes
14565	13:**0 X** vs. 38:**10 X**	-2.183	-2.327 to -2.039	Yes
14566	13:**0 X** vs. 38:**40 X**	-0.6440	-0.7880 to -0.5000	Yes
14567	13:**0 X** vs. 38:**50 X**	-0.4647	-0.6086 to -0.3207	Yes
14568	13:**0 X** vs. 39:**0 X**	-3.412	-3.556 to -3.268	Yes
14569	13:**0 X** vs. 39:**1 X**	-3.304	-3.448 to -3.160	Yes
14570	13:**0 X** vs. 39:**10 X**	-2.328	-2.472 to -2.184	Yes
14571	13:**0 X** vs. 39:**40 X**	-0.6500	-0.7940 to -0.5060	Yes
14572	13:**0 X** vs. 39:**50 X**	-0.4957	-0.6396 to -0.3517	Yes
14573	13:**0 X** vs. 40:**0 X**	-3.453	-3.597 to -3.309	Yes
14574	13:**0 X** vs. 40:**1 X**	-3.379	-3.523 to -3.235	Yes
14575	13:**0 X** vs. 40:**10 X**	-2.584	-2.728 to -2.440	Yes
14576	13:**0 X** vs. 40:**40 X**	-0.6547	-0.7986 to -0.5107	Yes
14577	13:**0 X** vs. 40:**50 X**	-0.5020	-0.6460 to -0.3580	Yes
14578	13:**0 X** vs. 41:**0 X**	-3.494	-3.638 to -3.350	Yes
14579	13:**0 X** vs. 41:**1 X**	-3.493	-3.637 to -3.349	Yes
14580	13:**0 X** vs. 41:**10 X**	-2.638	-2.782 to -2.494	Yes

2way ANOVA<br>Multiple comparisons	A<br>Data Set-A<br>Y	B<br>Data Set-B<br>Y	C<br>Data Set-C<br>Y
14581 13:0 X vs. 41:40 X	-0.6020	-0.7460 to -0.4580	Yes
14582 13:0 X vs. 41:50 X	-0.5147	-0.6586 to -0.3707	Yes
14583 13:0 X vs. 42:0 X	-3.636	-3.780 to -3.492	Yes
14584 13:0 X vs. 42:1 X	-3.656	-3.800 to -3.512	Yes
14585 13:0 X vs. 42:10 X	-2.638	-2.782 to -2.494	Yes
14586 13:0 X vs. 42:40 X	-0.6147	-0.7586 to -0.4707	Yes
14587 13:0 X vs. 42:50 X	-0.5180	-0.6620 to -0.3740	Yes
14588 13:0 X vs. 43:0 X	-3.726	-3.870 to -3.582	Yes
14589 13:0 X vs. 43:1 X	-3.640	-3.784 to -3.496	Yes
14590 13:0 X vs. 43:10 X	-3.356	-3.500 to -3.212	Yes
14591 13:0 X vs. 43:40 X	-0.6130	-0.7570 to -0.4690	Yes
14592 13:0 X vs. 43:50 X	-0.5183	-0.6623 to -0.3744	Yes
14593 13:0 X vs. 44:0 X	-3.750	-3.894 to -3.606	Yes
14594 13:0 X vs. 44:1 X	-3.640	-3.784 to -3.496	Yes
14595 13:0 X vs. 44:10 X	-3.419	-3.563 to -3.275	Yes
14596 13:0 X vs. 44:40 X	-0.6150	-0.7590 to -0.4710	Yes
14597 13:0 X vs. 44:50 X	-0.4507	-0.5946 to -0.3067	Yes
14598 13:0 X vs. 45:0 X	-3.615	-3.759 to -3.471	Yes
14599 13:0 X vs. 45:1 X	-3.417	-3.561 to -3.273	Yes
14600 13:0 X vs. 45:10 X	-3.299	-3.443 to -3.155	Yes
14601 13:0 X vs. 45:40 X	-0.6163	-0.7603 to -0.4724	Yes
14602 13:0 X vs. 45:50 X	-0.4477	-0.5916 to -0.3037	Yes
14603 13:0 X vs. 46:0 X	-3.419	-3.563 to -3.275	Yes
14604 13:0 X vs. 46:1 X	-3.386	-3.530 to -3.242	Yes
14605 13:0 X vs. 46:10 X	-3.294	-3.438 to -3.150	Yes
14606 13:0 X vs. 46:40 X	-0.6300	-0.7740 to -0.4860	Yes
14607 13:0 X vs. 46:50 X	-0.4407	-0.5846 to -0.2967	Yes
14608 13:0 X vs. 47:0 X	-3.395	-3.539 to -3.251	Yes
14609 13:0 X vs. 47:1 X	-3.299	-3.443 to -3.155	Yes
14610 13:0 X vs. 47:10 X	-3.280	-3.424 to -3.136	Yes
14611 13:0 X vs. 47:40 X	-0.6400	-0.7840 to -0.4960	Yes
14612 13:0 X vs. 47:50 X	-0.3987	-0.5426 to -0.2547	Yes
14613 13:0 X vs. 48:0 X	-3.382	-3.543 to -3.221	Yes
14614 13:0 X vs. 48:1 X	-3.286	-3.430 to -3.142	Yes
14615 13:0 X vs. 48:10 X	-3.280	-3.424 to -3.136	Yes
14616 13:0 X vs. 48:40 X	-0.6463	-0.7903 to -0.5024	Yes
14617 13:0 X vs. 48:50 X	-0.4060	-0.5500 to -0.2620	Yes
14618 13:0 X vs. 49:0 X	-3.382	-3.526 to -3.238	Yes
14619 13:0 X vs. 49:1 X	-3.286	-3.430 to -3.142	Yes
14620 13:0 X vs. 49:10 X	-3.280	-3.424 to -3.136	Yes
14621 13:0 X vs. 49:40 X	-0.6507	-0.7946 to -0.5067	Yes
14622 13:0 X vs. 49:50 X	-0.4053	-0.5493 to -0.2614	Yes
14623 13:0 X vs. 50:0 X	-3.382	-3.526 to -3.238	Yes
14624 13:0 X vs. 50:1 X	-3.286	-3.430 to -3.142	Yes
14625 13:0 X vs. 50:10 X	-3.280	-3.424 to -3.136	Yes

2way ANOVA Multiple comparisons	A Data Set-A Y	B Data Set-B Y	C Data Set-C Y
14626 13:**0 X** vs. 50:**40 X**	-0.6583	-0.8023 to -0.5144	Yes
14627 13:**0 X** vs. 50:**50 X**	-0.4050	-0.5490 to -0.2610	Yes
14628 13:**1 X** vs. 13:**10 X**	-0.0220	-0.1660 to 0.1220	No
14629 13:**1 X** vs. 13:**40 X**	-0.01833	-0.1623 to 0.1256	No
14630 13:**1 X** vs. 13:**50 X**	-0.01933	-0.1633 to 0.1246	No
14631 13:**1 X** vs. 14:**0 X**	-0.02133	-0.1653 to 0.1226	No
14632 13:**1 X** vs. 14:**1 X**	-0.0130	-0.1570 to 0.1310	No
14633 13:**1 X** vs. 14:**10 X**	-0.0270	-0.1710 to 0.1170	No
14634 13:**1 X** vs. 14:**40 X**	-0.02367	-0.1676 to 0.1203	No
14635 13:**1 X** vs. 14:**50 X**	-0.02767	-0.1716 to 0.1163	No
14636 13:**1 X** vs. 15:**0 X**	-0.03367	-0.1776 to 0.1103	No
14637 13:**1 X** vs. 15:**1 X**	-0.01367	-0.1576 to 0.1303	No
14638 13:**1 X** vs. 15:**10 X**	-0.0410	-0.1850 to 0.1030	No
14639 13:**1 X** vs. 15:**40 X**	-0.02333	-0.1673 to 0.1206	No
14640 13:**1 X** vs. 15:**50 X**	-0.0410	-0.1850 to 0.1030	No
14641 13:**1 X** vs. 16:**0 X**	-0.1260	-0.2700 to 0.01796	No
14642 13:**1 X** vs. 16:**1 X**	-0.0170	-0.1610 to 0.1270	No
14643 13:**1 X** vs. 16:**10 X**	-0.04267	-0.1866 to 0.1013	No
14644 13:**1 X** vs. 16:**40 X**	-0.0620	-0.2060 to 0.08196	No
14645 13:**1 X** vs. 16:**50 X**	-0.1043	-0.2483 to 0.03963	No
14646 13:**1 X** vs. 17:**0 X**	-0.1787	-0.3226 to -0.03471	Yes
14647 13:**1 X** vs. 17:**1 X**	-0.0930	-0.2370 to 0.05096	No
14648 13:**1 X** vs. 17:**10 X**	-0.1497	-0.2936 to -0.005707	Yes
14649 13:**1 X** vs. 17:**40 X**	-0.0730	-0.2170 to 0.07096	No
14650 13:**1 X** vs. 17:**50 X**	-0.1183	-0.2623 to 0.02563	No
14651 13:**1 X** vs. 18:**0 X**	-0.2273	-0.3713 to -0.08337	Yes
14652 13:**1 X** vs. 18:**1 X**	-0.1357	-0.2796 to 0.008293	No
14653 13:**1 X** vs. 18:**10 X**	-0.2280	-0.3720 to -0.08404	Yes
14654 13:**1 X** vs. 18:**40 X**	-0.07733	-0.2213 to 0.06663	No
14655 13:**1 X** vs. 18:**50 X**	-0.1223	-0.2663 to 0.02163	No
14656 13:**1 X** vs. 19:**0 X**	-0.2623	-0.4063 to -0.1184	Yes
14657 13:**1 X** vs. 19:**1 X**	-0.2503	-0.3943 to -0.1064	Yes
14658 13:**1 X** vs. 19:**10 X**	-0.2480	-0.3920 to -0.1040	Yes
14659 13:**1 X** vs. 19:**40 X**	-0.1060	-0.2500 to 0.03796	No
14660 13:**1 X** vs. 19:**50 X**	-0.1170	-0.2610 to 0.02696	No
14661 13:**1 X** vs. 20:**0 X**	-0.3420	-0.4860 to -0.1980	Yes
14662 13:**1 X** vs. 20:**1 X**	-0.1613	-0.3053 to -0.01737	Yes
14663 13:**1 X** vs. 20:**10 X**	-0.2503	-0.3943 to -0.1064	Yes
14664 13:**1 X** vs. 20:**40 X**	-0.1177	-0.2616 to 0.02629	No
14665 13:**1 X** vs. 20:**50 X**	-0.1617	-0.3056 to -0.01771	Yes
14666 13:**1 X** vs. 21:**0 X**	-0.3653	-0.5093 to -0.2214	Yes
14667 13:**1 X** vs. 21:**1 X**	-0.3183	-0.4623 to -0.1744	Yes
14668 13:**1 X** vs. 21:**10 X**	-0.3153	-0.4593 to -0.1714	Yes
14669 13:**1 X** vs. 21:**40 X**	-0.1907	-0.3346 to -0.04671	Yes
14670 13:**1 X** vs. 21:**50 X**	-0.2810	-0.4250 to -0.1370	Yes

	2way ANOVA Multiple comparisons	A Data Set-A Y	B Data Set-B Y	C Data Set-C Y
14671	13:1 X vs. 22:0 X	-0.4070	-0.5510 to -0.2630	Yes
14672	13:1 X vs. 22:1 X	-0.4017	-0.5456 to -0.2577	Yes
14673	13:1 X vs. 22:10 X	-0.3970	-0.5410 to -0.2530	Yes
14674	13:1 X vs. 22:40 X	-0.2973	-0.4413 to -0.1534	Yes
14675	13:1 X vs. 22:50 X	-0.3600	-0.5040 to -0.2160	Yes
14676	13:1 X vs. 23:0 X	-0.4723	-0.6163 to -0.3284	Yes
14677	13:1 X vs. 23:1 X	-0.6620	-0.8230 to -0.5010	Yes
14678	13:1 X vs. 23:10 X	-0.4117	-0.5556 to -0.2677	Yes
14679	13:1 X vs. 23:40 X	-0.2920	-0.4530 to -0.1310	Yes
14680	13:1 X vs. 23:50 X	-0.3260	-0.4700 to -0.1820	Yes
14681	13:1 X vs. 24:0 X	-0.5897	-0.7336 to -0.4457	Yes
14682	13:1 X vs. 24:1 X	-0.7460	-0.9070 to -0.5850	Yes
14683	13:1 X vs. 24:10 X	-0.5735	-0.7345 to -0.4125	Yes
14684	13:1 X vs. 24:40 X	-0.3083	-0.4523 to -0.1644	Yes
14685	13:1 X vs. 24:50 X	-0.2300	-0.3740 to -0.08604	Yes
14686	13:1 X vs. 25:0 X	-0.9270	-1.088 to -0.7660	Yes
14687	13:1 X vs. 25:1 X	-1.117	-1.277 to -0.9555	Yes
14688	13:1 X vs. 25:10 X	-0.4780	-0.6816 to -0.2744	Yes
14689	13:1 X vs. 25:40 X	-0.3103	-0.4543 to -0.1664	Yes
14690	13:1 X vs. 25:50 X	-0.2847	-0.4286 to -0.1407	Yes
14691	13:1 X vs. 26:0 X	-1.140	-1.300 to -0.9785	Yes
14692	13:1 X vs. 26:1 X	-1.253	-1.413 to -1.092	Yes
14693	13:1 X vs. 26:10 X	-0.9390	-1.100 to -0.7780	Yes
14694	13:1 X vs. 26:40 X	-0.3847	-0.5286 to -0.2407	Yes
14695	13:1 X vs. 26:50 X	-0.2450	-0.3890 to -0.1010	Yes
14696	13:1 X vs. 27:0 X	-1.327	-1.471 to -1.183	Yes
14697	13:1 X vs. 27:1 X	-1.418	-1.579 to -1.257	Yes
14698	13:1 X vs. 27:10 X	-1.230	-1.391 to -1.069	Yes
14699	13:1 X vs. 27:40 X	-0.3847	-0.5286 to -0.2407	Yes
14700	13:1 X vs. 27:50 X	-0.2443	-0.3883 to -0.1004	Yes
14701	13:1 X vs. 28:0 X	-1.277	-1.437 to -1.116	Yes
14702	13:1 X vs. 28:1 X	-1.317	-1.461 to -1.173	Yes
14703	13:1 X vs. 28:10 X	-1.119	-1.280 to -0.9580	Yes
14704	13:1 X vs. 28:40 X	-0.3970	-0.5410 to -0.2530	Yes
14705	13:1 X vs. 28:50 X	-0.2923	-0.4363 to -0.1484	Yes
14706	13:1 X vs. 29:0 X	-1.758	-1.919 to -1.597	Yes
14707	13:1 X vs. 29:1 X	-1.843	-2.004 to -1.682	Yes
14708	13:1 X vs. 29:10 X	-1.058	-1.218 to -0.8965	Yes
14709	13:1 X vs. 29:40 X	-0.5050	-0.6490 to -0.3610	Yes
14710	13:1 X vs. 29:50 X	-0.2937	-0.4376 to -0.1497	Yes
14711	13:1 X vs. 30:0 X	-1.473	-1.633 to -1.312	Yes
14712	13:1 X vs. 30:1 X	-1.794	-1.955 to -1.633	Yes
14713	13:1 X vs. 30:10 X	-1.132	-1.293 to -0.9710	Yes
14714	13:1 X vs. 30:40 X	-0.4320	-0.5760 to -0.2880	Yes
14715	13:1 X vs. 30:50 X	-0.3460	-0.4900 to -0.2020	Yes

2way ANOVA<br>Multiple comparisons		A<br>Data Set-A<br>Y	B<br>Data Set-B<br>Y	C<br>Data Set-C<br>Y
14716	13:1 X vs. 31:0 X	-1.791	-1.951 to -1.630	Yes
14717	13:1 X vs. 31:1 X	-1.781	-1.942 to -1.620	Yes
14718	13:1 X vs. 31:10 X	-1.264	-1.424 to -1.103	Yes
14719	13:1 X vs. 31:40 X	-0.5237	-0.6676 to -0.3797	Yes
14720	13:1 X vs. 31:50 X	-0.3250	-0.4690 to -0.1810	Yes
14721	13:1 X vs. 32:0 X	-2.382	-2.526 to -2.238	Yes
14722	13:1 X vs. 32:1 X	-1.838	-1.999 to -1.677	Yes
14723	13:1 X vs. 32:10 X	-1.694	-1.838 to -1.550	Yes
14724	13:1 X vs. 32:40 X	-0.5200	-0.6640 to -0.3760	Yes
14725	13:1 X vs. 32:50 X	-0.3247	-0.4686 to -0.1807	Yes
14726	13:1 X vs. 33:0 X	-2.386	-2.530 to -2.242	Yes
14727	13:1 X vs. 33:1 X	-2.200	-2.344 to -2.056	Yes
14728	13:1 X vs. 33:10 X	-1.861	-2.005 to -1.717	Yes
14729	13:1 X vs. 33:40 X	-0.5387	-0.6826 to -0.3947	Yes
14730	13:1 X vs. 33:50 X	-0.3397	-0.4836 to -0.1957	Yes
14731	13:1 X vs. 34:0 X	-2.310	-2.454 to -2.166	Yes
14732	13:1 X vs. 34:1 X	-2.318	-2.462 to -2.174	Yes
14733	13:1 X vs. 34:10 X	-1.951	-2.095 to -1.807	Yes
14734	13:1 X vs. 34:40 X	-0.5507	-0.6946 to -0.4067	Yes
14735	13:1 X vs. 34:50 X	-0.4887	-0.6326 to -0.3447	Yes
14736	13:1 X vs. 35:0 X	-2.659	-2.803 to -2.515	Yes
14737	13:1 X vs. 35:1 X	-2.425	-2.569 to -2.281	Yes
14738	13:1 X vs. 35:10 X	-1.913	-2.057 to -1.769	Yes
14739	13:1 X vs. 35:40 X	-0.5597	-0.7036 to -0.4157	Yes
14740	13:1 X vs. 35:50 X	-0.5013	-0.6453 to -0.3574	Yes
14741	13:1 X vs. 36:0 X	-2.866	-3.026 to -2.705	Yes
14742	13:1 X vs. 36:1 X	-2.561	-2.705 to -2.417	Yes
14743	13:1 X vs. 36:10 X	-1.918	-2.062 to -1.774	Yes
14744	13:1 X vs. 36:40 X	-0.6150	-0.7590 to -0.4710	Yes
14745	13:1 X vs. 36:50 X	-0.4647	-0.6086 to -0.3207	Yes
14746	13:1 X vs. 37:0 X	-3.249	-3.393 to -3.105	Yes
14747	13:1 X vs. 37:1 X	-2.631	-2.775 to -2.487	Yes
14748	13:1 X vs. 37:10 X	-1.920	-2.064 to -1.776	Yes
14749	13:1 X vs. 37:40 X	-0.6523	-0.7963 to -0.5084	Yes
14750	13:1 X vs. 37:50 X	-0.4733	-0.6173 to -0.3294	Yes
14751	13:1 X vs. 38:0 X	-3.194	-3.338 to -3.050	Yes
14752	13:1 X vs. 38:1 X	-2.750	-2.894 to -2.606	Yes
14753	13:1 X vs. 38:10 X	-2.195	-2.339 to -2.051	Yes
14754	13:1 X vs. 38:40 X	-0.6567	-0.8006 to -0.5127	Yes
14755	13:1 X vs. 38:50 X	-0.4773	-0.6213 to -0.3334	Yes
14756	13:1 X vs. 39:0 X	-3.425	-3.569 to -3.281	Yes
14757	13:1 X vs. 39:1 X	-3.317	-3.461 to -3.173	Yes
14758	13:1 X vs. 39:10 X	-2.340	-2.484 to -2.196	Yes
14759	13:1 X vs. 39:40 X	-0.6627	-0.8066 to -0.5187	Yes
14760	13:1 X vs. 39:50 X	-0.5083	-0.6523 to -0.3644	Yes

	2way ANOVA Multiple comparisons	A Data Set-A Y	B Data Set-B Y	C Data Set-C Y
14761	13:**1 X** vs. 40:**0 X**	-3.466	-3.610 to -3.322	Yes
14762	13:**1 X** vs. 40:**1 X**	-3.392	-3.536 to -3.248	Yes
14763	13:**1 X** vs. 40:**10 X**	-2.597	-2.741 to -2.453	Yes
14764	13:**1 X** vs. 40:**40 X**	-0.6673	-0.8113 to -0.5234	Yes
14765	13:**1 X** vs. 40:**50 X**	-0.5147	-0.6586 to -0.3707	Yes
14766	13:**1 X** vs. 41:**0 X**	-3.507	-3.651 to -3.363	Yes
14767	13:**1 X** vs. 41:**1 X**	-3.506	-3.650 to -3.362	Yes
14768	13:**1 X** vs. 41:**10 X**	-2.650	-2.794 to -2.506	Yes
14769	13:**1 X** vs. 41:**40 X**	-0.6147	-0.7586 to -0.4707	Yes
14770	13:**1 X** vs. 41:**50 X**	-0.5273	-0.6713 to -0.3834	Yes
14771	13:**1 X** vs. 42:**0 X**	-3.649	-3.793 to -3.505	Yes
14772	13:**1 X** vs. 42:**1 X**	-3.668	-3.812 to -3.524	Yes
14773	13:**1 X** vs. 42:**10 X**	-2.651	-2.795 to -2.507	Yes
14774	13:**1 X** vs. 42:**40 X**	-0.6273	-0.7713 to -0.4834	Yes
14775	13:**1 X** vs. 42:**50 X**	-0.5307	-0.6746 to -0.3867	Yes
14776	13:**1 X** vs. 43:**0 X**	-3.739	-3.883 to -3.595	Yes
14777	13:**1 X** vs. 43:**1 X**	-3.653	-3.797 to -3.509	Yes
14778	13:**1 X** vs. 43:**10 X**	-3.369	-3.513 to -3.225	Yes
14779	13:**1 X** vs. 43:**40 X**	-0.6257	-0.7696 to -0.4817	Yes
14780	13:**1 X** vs. 43:**50 X**	-0.5310	-0.6750 to -0.3870	Yes
14781	13:**1 X** vs. 44:**0 X**	-3.763	-3.907 to -3.619	Yes
14782	13:**1 X** vs. 44:**1 X**	-3.652	-3.796 to -3.508	Yes
14783	13:**1 X** vs. 44:**10 X**	-3.432	-3.576 to -3.288	Yes
14784	13:**1 X** vs. 44:**40 X**	-0.6277	-0.7716 to -0.4837	Yes
14785	13:**1 X** vs. 44:**50 X**	-0.4633	-0.6073 to -0.3194	Yes
14786	13:**1 X** vs. 45:**0 X**	-3.627	-3.771 to -3.483	Yes
14787	13:**1 X** vs. 45:**1 X**	-3.429	-3.573 to -3.285	Yes
14788	13:**1 X** vs. 45:**10 X**	-3.312	-3.456 to -3.168	Yes
14789	13:**1 X** vs. 45:**40 X**	-0.6290	-0.7730 to -0.4850	Yes
14790	13:**1 X** vs. 45:**50 X**	-0.4603	-0.6043 to -0.3164	Yes
14791	13:**1 X** vs. 46:**0 X**	-3.432	-3.576 to -3.288	Yes
14792	13:**1 X** vs. 46:**1 X**	-3.399	-3.543 to -3.255	Yes
14793	13:**1 X** vs. 46:**10 X**	-3.307	-3.451 to -3.163	Yes
14794	13:**1 X** vs. 46:**40 X**	-0.6427	-0.7866 to -0.4987	Yes
14795	13:**1 X** vs. 46:**50 X**	-0.4533	-0.5973 to -0.3094	Yes
14796	13:**1 X** vs. 47:**0 X**	-3.407	-3.551 to -3.263	Yes
14797	13:**1 X** vs. 47:**1 X**	-3.311	-3.455 to -3.167	Yes
14798	13:**1 X** vs. 47:**10 X**	-3.293	-3.437 to -3.149	Yes
14799	13:**1 X** vs. 47:**40 X**	-0.6527	-0.7966 to -0.5087	Yes
14800	13:**1 X** vs. 47:**50 X**	-0.4113	-0.5553 to -0.2674	Yes
14801	13:**1 X** vs. 48:**0 X**	-3.395	-3.555 to -3.234	Yes
14802	13:**1 X** vs. 48:**1 X**	-3.298	-3.442 to -3.154	Yes
14803	13:**1 X** vs. 48:**10 X**	-3.293	-3.437 to -3.149	Yes
14804	13:**1 X** vs. 48:**40 X**	-0.6590	-0.8030 to -0.5150	Yes
14805	13:**1 X** vs. 48:**50 X**	-0.4187	-0.5626 to -0.2747	Yes

	2way ANOVA<br>Multiple comparisons	A<br>Data Set-A<br>Y	B<br>Data Set-B<br>Y	C<br>Data Set-C<br>Y
14806	13:**1 X** vs. 49:**0 X**	-3.395	-3.539 to -3.251	Yes
14807	13:**1 X** vs. 49:**1 X**	-3.298	-3.442 to -3.154	Yes
14808	13:**1 X** vs. 49:**10 X**	-3.293	-3.437 to -3.149	Yes
14809	13:**1 X** vs. 49:**40 X**	-0.6633	-0.8073 to -0.5194	Yes
14810	13:**1 X** vs. 49:**50 X**	-0.4180	-0.5620 to -0.2740	Yes
14811	13:**1 X** vs. 50:**0 X**	-3.395	-3.539 to -3.251	Yes
14812	13:**1 X** vs. 50:**1 X**	-3.298	-3.442 to -3.154	Yes
14813	13:**1 X** vs. 50:**10 X**	-3.293	-3.437 to -3.149	Yes
14814	13:**1 X** vs. 50:**40 X**	-0.6710	-0.8150 to -0.5270	Yes
14815	13:**1 X** vs. 50:**50 X**	-0.4177	-0.5616 to -0.2737	Yes
14816	13:**10 X** vs. 13:**40 X**	0.003667	-0.1403 to 0.1476	No
14817	13:**10 X** vs. 13:**50 X**	0.002667	-0.1413 to 0.1466	No
14818	13:**10 X** vs. 14:**0 X**	0.0006667	-0.1433 to 0.1446	No
14819	13:**10 X** vs. 14:**1 X**	0.009000	-0.1350 to 0.1530	No
14820	13:**10 X** vs. 14:**10 X**	-0.0050	-0.1490 to 0.1390	No
14821	13:**10 X** vs. 14:**40 X**	-0.001667	-0.1456 to 0.1423	No
14822	13:**10 X** vs. 14:**50 X**	-0.005667	-0.1496 to 0.1383	No
14823	13:**10 X** vs. 15:**0 X**	-0.01167	-0.1556 to 0.1323	No
14824	13:**10 X** vs. 15:**1 X**	0.008333	-0.1356 to 0.1523	No
14825	13:**10 X** vs. 15:**10 X**	-0.0190	-0.1630 to 0.1250	No
14826	13:**10 X** vs. 15:**40 X**	-0.001333	-0.1453 to 0.1426	No
14827	13:**10 X** vs. 15:**50 X**	-0.0190	-0.1630 to 0.1250	No
14828	13:**10 X** vs. 16:**0 X**	-0.1040	-0.2480 to 0.03996	No
14829	13:**10 X** vs. 16:**1 X**	0.0050	-0.1390 to 0.1490	No
14830	13:**10 X** vs. 16:**10 X**	-0.02067	-0.1646 to 0.1233	No
14831	13:**10 X** vs. 16:**40 X**	-0.0400	-0.1840 to 0.1040	No
14832	13:**10 X** vs. 16:**50 X**	-0.08233	-0.2263 to 0.06163	No
14833	13:**10 X** vs. 17:**0 X**	-0.1567	-0.3006 to -0.01271	Yes
14834	13:**10 X** vs. 17:**1 X**	-0.0710	-0.2150 to 0.07296	No
14835	13:**10 X** vs. 17:**10 X**	-0.1277	-0.2716 to 0.01629	No
14836	13:**10 X** vs. 17:**40 X**	-0.0510	-0.1950 to 0.09296	No
14837	13:**10 X** vs. 17:**50 X**	-0.09633	-0.2403 to 0.04763	No
14838	13:**10 X** vs. 18:**0 X**	-0.2053	-0.3493 to -0.06137	Yes
14839	13:**10 X** vs. 18:**1 X**	-0.1137	-0.2576 to 0.03029	No
14840	13:**10 X** vs. 18:**10 X**	-0.2060	-0.3500 to -0.06204	Yes
14841	13:**10 X** vs. 18:**40 X**	-0.05533	-0.1993 to 0.08863	No
14842	13:**10 X** vs. 18:**50 X**	-0.1003	-0.2443 to 0.04363	No
14843	13:**10 X** vs. 19:**0 X**	-0.2403	-0.3843 to -0.09637	Yes
14844	13:**10 X** vs. 19:**1 X**	-0.2283	-0.3723 to -0.08437	Yes
14845	13:**10 X** vs. 19:**10 X**	-0.2260	-0.3700 to -0.08204	Yes
14846	13:**10 X** vs. 19:**40 X**	-0.0840	-0.2280 to 0.05996	No
14847	13:**10 X** vs. 19:**50 X**	-0.0950	-0.2390 to 0.04896	No
14848	13:**10 X** vs. 20:**0 X**	-0.3200	-0.4640 to -0.1760	Yes
14849	13:**10 X** vs. 20:**1 X**	-0.1393	-0.2833 to 0.004626	No
14850	13:**10 X** vs. 20:**10 X**	-0.2283	-0.3723 to -0.08437	Yes

2way ANOVA<br>Multiple comparisons		A<br>Data Set-A<br>Y	B<br>Data Set-B<br>Y	C<br>Data Set-C<br>Y
14851	13:10 X vs. 20:40 X	-0.09567	-0.2396 to 0.04829	No
14852	13:10 X vs. 20:50 X	-0.1397	-0.2836 to 0.004293	No
14853	13:10 X vs. 21:0 X	-0.3433	-0.4873 to -0.1994	Yes
14854	13:10 X vs. 21:1 X	-0.2963	-0.4403 to -0.1524	Yes
14855	13:10 X vs. 21:10 X	-0.2933	-0.4373 to -0.1494	Yes
14856	13:10 X vs. 21:40 X	-0.1687	-0.3126 to -0.02471	Yes
14857	13:10 X vs. 21:50 X	-0.2590	-0.4030 to -0.1150	Yes
14858	13:10 X vs. 22:0 X	-0.3850	-0.5290 to -0.2410	Yes
14859	13:10 X vs. 22:1 X	-0.3797	-0.5236 to -0.2357	Yes
14860	13:10 X vs. 22:10 X	-0.3750	-0.5190 to -0.2310	Yes
14861	13:10 X vs. 22:40 X	-0.2753	-0.4193 to -0.1314	Yes
14862	13:10 X vs. 22:50 X	-0.3380	-0.4820 to -0.1940	Yes
14863	13:10 X vs. 23:0 X	-0.4503	-0.5943 to -0.3064	Yes
14864	13:10 X vs. 23:1 X	-0.6400	-0.8010 to -0.4790	Yes
14865	13:10 X vs. 23:10 X	-0.3897	-0.5336 to -0.2457	Yes
14866	13:10 X vs. 23:40 X	-0.2700	-0.4310 to -0.1090	Yes
14867	13:10 X vs. 23:50 X	-0.3040	-0.4480 to -0.1600	Yes
14868	13:10 X vs. 24:0 X	-0.5677	-0.7116 to -0.4237	Yes
14869	13:10 X vs. 24:1 X	-0.7240	-0.8850 to -0.5630	Yes
14870	13:10 X vs. 24:10 X	-0.5515	-0.7125 to -0.3905	Yes
14871	13:10 X vs. 24:40 X	-0.2863	-0.4303 to -0.1424	Yes
14872	13:10 X vs. 24:50 X	-0.2080	-0.3520 to -0.06404	Yes
14873	13:10 X vs. 25:0 X	-0.9050	-1.066 to -0.7440	Yes
14874	13:10 X vs. 25:1 X	-1.095	-1.255 to -0.9335	Yes
14875	13:10 X vs. 25:10 X	-0.4560	-0.6596 to -0.2524	Yes
14876	13:10 X vs. 25:40 X	-0.2883	-0.4323 to -0.1444	Yes
14877	13:10 X vs. 25:50 X	-0.2627	-0.4066 to -0.1187	Yes
14878	13:10 X vs. 26:0 X	-1.118	-1.278 to -0.9565	Yes
14879	13:10 X vs. 26:1 X	-1.231	-1.391 to -1.070	Yes
14880	13:10 X vs. 26:10 X	-0.9170	-1.078 to -0.7560	Yes
14881	13:10 X vs. 26:40 X	-0.3627	-0.5066 to -0.2187	Yes
14882	13:10 X vs. 26:50 X	-0.2230	-0.3670 to -0.07904	Yes
14883	13:10 X vs. 27:0 X	-1.305	-1.449 to -1.161	Yes
14884	13:10 X vs. 27:1 X	-1.396	-1.557 to -1.235	Yes
14885	13:10 X vs. 27:10 X	-1.208	-1.369 to -1.047	Yes
14886	13:10 X vs. 27:40 X	-0.3627	-0.5066 to -0.2187	Yes
14887	13:10 X vs. 27:50 X	-0.2223	-0.3663 to -0.07837	Yes
14888	13:10 X vs. 28:0 X	-1.255	-1.415 to -1.094	Yes
14889	13:10 X vs. 28:1 X	-1.295	-1.439 to -1.151	Yes
14890	13:10 X vs. 28:10 X	-1.097	-1.258 to -0.9360	Yes
14891	13:10 X vs. 28:40 X	-0.3750	-0.5190 to -0.2310	Yes
14892	13:10 X vs. 28:50 X	-0.2703	-0.4143 to -0.1264	Yes
14893	13:10 X vs. 29:0 X	-1.736	-1.897 to -1.575	Yes
14894	13:10 X vs. 29:1 X	-1.821	-1.982 to -1.660	Yes
14895	13:10 X vs. 29:10 X	-1.036	-1.196 to -0.8745	Yes

	2way ANOVA<br>Multiple comparisons	A<br>Data Set-A<br>Y	B<br>Data Set-B<br>Y	C<br>Data Set-C<br>Y
14896	13:10 X vs. 29:40 X	-0.4830	-0.6270 to -0.3390	Yes
14897	13:10 X vs. 29:50 X	-0.2717	-0.4156 to -0.1277	Yes
14898	13:10 X vs. 30:0 X	-1.451	-1.611 to -1.290	Yes
14899	13:10 X vs. 30:1 X	-1.772	-1.933 to -1.611	Yes
14900	13:10 X vs. 30:10 X	-1.110	-1.271 to -0.9490	Yes
14901	13:10 X vs. 30:40 X	-0.4100	-0.5540 to -0.2660	Yes
14902	13:10 X vs. 30:50 X	-0.3240	-0.4680 to -0.1800	Yes
14903	13:10 X vs. 31:0 X	-1.769	-1.929 to -1.608	Yes
14904	13:10 X vs. 31:1 X	-1.759	-1.920 to -1.598	Yes
14905	13:10 X vs. 31:10 X	-1.242	-1.402 to -1.081	Yes
14906	13:10 X vs. 31:40 X	-0.5017	-0.6456 to -0.3577	Yes
14907	13:10 X vs. 31:50 X	-0.3030	-0.4470 to -0.1590	Yes
14908	13:10 X vs. 32:0 X	-2.360	-2.504 to -2.216	Yes
14909	13:10 X vs. 32:1 X	-1.816	-1.977 to -1.655	Yes
14910	13:10 X vs. 32:10 X	-1.672	-1.816 to -1.528	Yes
14911	13:10 X vs. 32:40 X	-0.4980	-0.6420 to -0.3540	Yes
14912	13:10 X vs. 32:50 X	-0.3027	-0.4466 to -0.1587	Yes
14913	13:10 X vs. 33:0 X	-2.364	-2.508 to -2.220	Yes
14914	13:10 X vs. 33:1 X	-2.178	-2.322 to -2.034	Yes
14915	13:10 X vs. 33:10 X	-1.839	-1.983 to -1.695	Yes
14916	13:10 X vs. 33:40 X	-0.5167	-0.6606 to -0.3727	Yes
14917	13:10 X vs. 33:50 X	-0.3177	-0.4616 to -0.1737	Yes
14918	13:10 X vs. 34:0 X	-2.288	-2.432 to -2.144	Yes
14919	13:10 X vs. 34:1 X	-2.296	-2.440 to -2.152	Yes
14920	13:10 X vs. 34:10 X	-1.929	-2.073 to -1.785	Yes
14921	13:10 X vs. 34:40 X	-0.5287	-0.6726 to -0.3847	Yes
14922	13:10 X vs. 34:50 X	-0.4667	-0.6106 to -0.3227	Yes
14923	13:10 X vs. 35:0 X	-2.637	-2.781 to -2.493	Yes
14924	13:10 X vs. 35:1 X	-2.403	-2.547 to -2.259	Yes
14925	13:10 X vs. 35:10 X	-1.891	-2.035 to -1.747	Yes
14926	13:10 X vs. 35:40 X	-0.5377	-0.6816 to -0.3937	Yes
14927	13:10 X vs. 35:50 X	-0.4793	-0.6233 to -0.3354	Yes
14928	13:10 X vs. 36:0 X	-2.844	-3.004 to -2.683	Yes
14929	13:10 X vs. 36:1 X	-2.539	-2.683 to -2.395	Yes
14930	13:10 X vs. 36:10 X	-1.896	-2.040 to -1.752	Yes
14931	13:10 X vs. 36:40 X	-0.5930	-0.7370 to -0.4490	Yes
14932	13:10 X vs. 36:50 X	-0.4427	-0.5866 to -0.2987	Yes
14933	13:10 X vs. 37:0 X	-3.227	-3.371 to -3.083	Yes
14934	13:10 X vs. 37:1 X	-2.609	-2.753 to -2.465	Yes
14935	13:10 X vs. 37:10 X	-1.898	-2.042 to -1.754	Yes
14936	13:10 X vs. 37:40 X	-0.6303	-0.7743 to -0.4864	Yes
14937	13:10 X vs. 37:50 X	-0.4513	-0.5953 to -0.3074	Yes
14938	13:10 X vs. 38:0 X	-3.172	-3.316 to -3.028	Yes
14939	13:10 X vs. 38:1 X	-2.728	-2.872 to -2.584	Yes
14940	13:10 X vs. 38:10 X	-2.173	-2.317 to -2.029	Yes

	2way ANOVA<br>Multiple comparisons	A<br>Data Set-A<br>Y	B<br>Data Set-B<br>Y	C<br>Data Set-C<br>Y
14941	13:10 X vs. 38:40 X	-0.6347	-0.7786 to -0.4907	Yes
14942	13:10 X vs. 38:50 X	-0.4553	-0.5993 to -0.3114	Yes
14943	13:10 X vs. 39:0 X	-3.403	-3.547 to -3.259	Yes
14944	13:10 X vs. 39:1 X	-3.295	-3.439 to -3.151	Yes
14945	13:10 X vs. 39:10 X	-2.318	-2.462 to -2.174	Yes
14946	13:10 X vs. 39:40 X	-0.6407	-0.7846 to -0.4967	Yes
14947	13:10 X vs. 39:50 X	-0.4863	-0.6303 to -0.3424	Yes
14948	13:10 X vs. 40:0 X	-3.444	-3.588 to -3.300	Yes
14949	13:10 X vs. 40:1 X	-3.370	-3.514 to -3.226	Yes
14950	13:10 X vs. 40:10 X	-2.575	-2.719 to -2.431	Yes
14951	13:10 X vs. 40:40 X	-0.6453	-0.7893 to -0.5014	Yes
14952	13:10 X vs. 40:50 X	-0.4927	-0.6366 to -0.3487	Yes
14953	13:10 X vs. 41:0 X	-3.485	-3.629 to -3.341	Yes
14954	13:10 X vs. 41:1 X	-3.484	-3.628 to -3.340	Yes
14955	13:10 X vs. 41:10 X	-2.628	-2.772 to -2.484	Yes
14956	13:10 X vs. 41:40 X	-0.5927	-0.7366 to -0.4487	Yes
14957	13:10 X vs. 41:50 X	-0.5053	-0.6493 to -0.3614	Yes
14958	13:10 X vs. 42:0 X	-3.627	-3.771 to -3.483	Yes
14959	13:10 X vs. 42:1 X	-3.646	-3.790 to -3.502	Yes
14960	13:10 X vs. 42:10 X	-2.629	-2.773 to -2.485	Yes
14961	13:10 X vs. 42:40 X	-0.6053	-0.7493 to -0.4614	Yes
14962	13:10 X vs. 42:50 X	-0.5087	-0.6526 to -0.3647	Yes
14963	13:10 X vs. 43:0 X	-3.717	-3.861 to -3.573	Yes
14964	13:10 X vs. 43:1 X	-3.631	-3.775 to -3.487	Yes
14965	13:10 X vs. 43:10 X	-3.347	-3.491 to -3.203	Yes
14966	13:10 X vs. 43:40 X	-0.6037	-0.7476 to -0.4597	Yes
14967	13:10 X vs. 43:50 X	-0.5090	-0.6530 to -0.3650	Yes
14968	13:10 X vs. 44:0 X	-3.741	-3.885 to -3.597	Yes
14969	13:10 X vs. 44:1 X	-3.630	-3.774 to -3.486	Yes
14970	13:10 X vs. 44:10 X	-3.410	-3.554 to -3.266	Yes
14971	13:10 X vs. 44:40 X	-0.6057	-0.7496 to -0.4617	Yes
14972	13:10 X vs. 44:50 X	-0.4413	-0.5853 to -0.2974	Yes
14973	13:10 X vs. 45:0 X	-3.605	-3.749 to -3.461	Yes
14974	13:10 X vs. 45:1 X	-3.407	-3.551 to -3.263	Yes
14975	13:10 X vs. 45:10 X	-3.290	-3.434 to -3.146	Yes
14976	13:10 X vs. 45:40 X	-0.6070	-0.7510 to -0.4630	Yes
14977	13:10 X vs. 45:50 X	-0.4383	-0.5823 to -0.2944	Yes
14978	13:10 X vs. 46:0 X	-3.410	-3.554 to -3.266	Yes
14979	13:10 X vs. 46:1 X	-3.377	-3.521 to -3.233	Yes
14980	13:10 X vs. 46:10 X	-3.285	-3.429 to -3.141	Yes
14981	13:10 X vs. 46:40 X	-0.6207	-0.7646 to -0.4767	Yes
14982	13:10 X vs. 46:50 X	-0.4313	-0.5753 to -0.2874	Yes
14983	13:10 X vs. 47:0 X	-3.385	-3.529 to -3.241	Yes
14984	13:10 X vs. 47:1 X	-3.289	-3.433 to -3.145	Yes
14985	13:10 X vs. 47:10 X	-3.271	-3.415 to -3.127	Yes

	2way ANOVA<br>Multiple comparisons	A<br>Data Set-A<br>Y	B<br>Data Set-B<br>Y	C<br>Data Set-C<br>Y
14986	13:10 X vs. 47:40 X	-0.6307	-0.7746 to -0.4867	Yes
14987	13:10 X vs. 47:50 X	-0.3893	-0.5333 to -0.2454	Yes
14988	13:10 X vs. 48:0 X	-3.373	-3.533 to -3.212	Yes
14989	13:10 X vs. 48:1 X	-3.276	-3.420 to -3.132	Yes
14990	13:10 X vs. 48:10 X	-3.271	-3.415 to -3.127	Yes
14991	13:10 X vs. 48:40 X	-0.6370	-0.7810 to -0.4930	Yes
14992	13:10 X vs. 48:50 X	-0.3967	-0.5406 to -0.2527	Yes
14993	13:10 X vs. 49:0 X	-3.373	-3.517 to -3.229	Yes
14994	13:10 X vs. 49:1 X	-3.276	-3.420 to -3.132	Yes
14995	13:10 X vs. 49:10 X	-3.271	-3.415 to -3.127	Yes
14996	13:10 X vs. 49:40 X	-0.6413	-0.7853 to -0.4974	Yes
14997	13:10 X vs. 49:50 X	-0.3960	-0.5400 to -0.2520	Yes
14998	13:10 X vs. 50:0 X	-3.373	-3.517 to -3.229	Yes
14999	13:10 X vs. 50:1 X	-3.276	-3.420 to -3.132	Yes
15000	13:10 X vs. 50:10 X	-3.271	-3.415 to -3.127	Yes
15001	13:10 X vs. 50:40 X	-0.6490	-0.7930 to -0.5050	Yes
15002	13:10 X vs. 50:50 X	-0.3957	-0.5396 to -0.2517	Yes
15003	13:40 X vs. 13:50 X	-0.001000	-0.1450 to 0.1430	No
15004	13:40 X vs. 14:0 X	-0.0030	-0.1470 to 0.1410	No
15005	13:40 X vs. 14:1 X	0.005333	-0.1386 to 0.1493	No
15006	13:40 X vs. 14:10 X	-0.008667	-0.1526 to 0.1353	No
15007	13:40 X vs. 14:40 X	-0.005333	-0.1493 to 0.1386	No
15008	13:40 X vs. 14:50 X	-0.009333	-0.1533 to 0.1346	No
15009	13:40 X vs. 15:0 X	-0.01533	-0.1593 to 0.1286	No
15010	13:40 X vs. 15:1 X	0.004667	-0.1393 to 0.1486	No
15011	13:40 X vs. 15:10 X	-0.02267	-0.1666 to 0.1213	No
15012	13:40 X vs. 15:40 X	-0.0050	-0.1490 to 0.1390	No
15013	13:40 X vs. 15:50 X	-0.02267	-0.1666 to 0.1213	No
15014	13:40 X vs. 16:0 X	-0.1077	-0.2516 to 0.03629	No
15015	13:40 X vs. 16:1 X	0.001333	-0.1426 to 0.1453	No
15016	13:40 X vs. 16:10 X	-0.02433	-0.1683 to 0.1196	No
15017	13:40 X vs. 16:40 X	-0.04367	-0.1876 to 0.1003	No
15018	13:40 X vs. 16:50 X	-0.0860	-0.2300 to 0.05796	No
15019	13:40 X vs. 17:0 X	-0.1603	-0.3043 to -0.01637	Yes
15020	13:40 X vs. 17:1 X	-0.07467	-0.2186 to 0.06929	No
15021	13:40 X vs. 17:10 X	-0.1313	-0.2753 to 0.01263	No
15022	13:40 X vs. 17:40 X	-0.05467	-0.1986 to 0.08929	No
15023	13:40 X vs. 17:50 X	-0.1000	-0.2440 to 0.04396	No
15024	13:40 X vs. 18:0 X	-0.2090	-0.3530 to -0.06504	Yes
15025	13:40 X vs. 18:1 X	-0.1173	-0.2613 to 0.02663	No
15026	13:40 X vs. 18:10 X	-0.2097	-0.3536 to -0.06571	Yes
15027	13:40 X vs. 18:40 X	-0.0590	-0.2030 to 0.08496	No
15028	13:40 X vs. 18:50 X	-0.1040	-0.2480 to 0.03996	No
15029	13:40 X vs. 19:0 X	-0.2440	-0.3880 to -0.1000	Yes
15030	13:40 X vs. 19:1 X	-0.2320	-0.3760 to -0.08804	Yes

2way ANOVA<br>Multiple comparisons		A<br>Data Set-A<br>Y	B<br>Data Set-B<br>Y	C<br>Data Set-C<br>Y
15031	13:40 X vs. 19:10 X	-0.2297	-0.3736 to -0.08571	Yes
15032	13:40 X vs. 19:40 X	-0.08767	-0.2316 to 0.05629	No
15033	13:40 X vs. 19:50 X	-0.09867	-0.2426 to 0.04529	No
15034	13:40 X vs. 20:0 X	-0.3237	-0.4676 to -0.1797	Yes
15035	13:40 X vs. 20:1 X	-0.1430	-0.2870 to 0.0009598	No
15036	13:40 X vs. 20:10 X	-0.2320	-0.3760 to -0.08804	Yes
15037	13:40 X vs. 20:40 X	-0.09933	-0.2433 to 0.04463	No
15038	13:40 X vs. 20:50 X	-0.1433	-0.2873 to 0.0006265	No
15039	13:40 X vs. 21:0 X	-0.3470	-0.4910 to -0.2030	Yes
15040	13:40 X vs. 21:1 X	-0.3000	-0.4440 to -0.1560	Yes
15041	13:40 X vs. 21:10 X	-0.2970	-0.4410 to -0.1530	Yes
15042	13:40 X vs. 21:40 X	-0.1723	-0.3163 to -0.02837	Yes
15043	13:40 X vs. 21:50 X	-0.2627	-0.4066 to -0.1187	Yes
15044	13:40 X vs. 22:0 X	-0.3887	-0.5326 to -0.2447	Yes
15045	13:40 X vs. 22:1 X	-0.3833	-0.5273 to -0.2394	Yes
15046	13:40 X vs. 22:10 X	-0.3787	-0.5226 to -0.2347	Yes
15047	13:40 X vs. 22:40 X	-0.2790	-0.4230 to -0.1350	Yes
15048	13:40 X vs. 22:50 X	-0.3417	-0.4856 to -0.1977	Yes
15049	13:40 X vs. 23:0 X	-0.4540	-0.5980 to -0.3100	Yes
15050	13:40 X vs. 23:1 X	-0.6437	-0.8046 to -0.4827	Yes
15051	13:40 X vs. 23:10 X	-0.3933	-0.5373 to -0.2494	Yes
15052	13:40 X vs. 23:40 X	-0.2737	-0.4346 to -0.1127	Yes
15053	13:40 X vs. 23:50 X	-0.3077	-0.4516 to -0.1637	Yes
15054	13:40 X vs. 24:0 X	-0.5713	-0.7153 to -0.4274	Yes
15055	13:40 X vs. 24:1 X	-0.7277	-0.8886 to -0.5667	Yes
15056	13:40 X vs. 24:10 X	-0.5552	-0.7161 to -0.3942	Yes
15057	13:40 X vs. 24:40 X	-0.2900	-0.4340 to -0.1460	Yes
15058	13:40 X vs. 24:50 X	-0.2117	-0.3556 to -0.06771	Yes
15059	13:40 X vs. 25:0 X	-0.9087	-1.070 to -0.7477	Yes
15060	13:40 X vs. 25:1 X	-1.098	-1.259 to -0.9372	Yes
15061	13:40 X vs. 25:10 X	-0.4597	-0.6633 to -0.2561	Yes
15062	13:40 X vs. 25:40 X	-0.2920	-0.4360 to -0.1480	Yes
15063	13:40 X vs. 25:50 X	-0.2663	-0.4103 to -0.1224	Yes
15064	13:40 X vs. 26:0 X	-1.121	-1.282 to -0.9602	Yes
15065	13:40 X vs. 26:1 X	-1.234	-1.395 to -1.073	Yes
15066	13:40 X vs. 26:10 X	-0.9207	-1.082 to -0.7597	Yes
15067	13:40 X vs. 26:40 X	-0.3663	-0.5103 to -0.2224	Yes
15068	13:40 X vs. 26:50 X	-0.2267	-0.3706 to -0.08271	Yes
15069	13:40 X vs. 27:0 X	-1.309	-1.453 to -1.165	Yes
15070	13:40 X vs. 27:1 X	-1.400	-1.561 to -1.239	Yes
15071	13:40 X vs. 27:10 X	-1.212	-1.373 to -1.051	Yes
15072	13:40 X vs. 27:40 X	-0.3663	-0.5103 to -0.2224	Yes
15073	13:40 X vs. 27:50 X	-0.2260	-0.3700 to -0.08204	Yes
15074	13:40 X vs. 28:0 X	-1.258	-1.419 to -1.097	Yes
15075	13:40 X vs. 28:1 X	-1.299	-1.443 to -1.155	Yes

	2way ANOVA<br>Multiple comparisons	A<br>Data Set-A<br>Y	B<br>Data Set-B<br>Y	C<br>Data Set-C<br>Y
15076	13:40 X vs. 28:10 X	-1.101	-1.262 to -0.9397	Yes
15077	13:40 X vs. 28:40 X	-0.3787	-0.5226 to -0.2347	Yes
15078	13:40 X vs. 28:50 X	-0.2740	-0.4180 to -0.1300	Yes
15079	13:40 X vs. 29:0 X	-1.740	-1.901 to -1.579	Yes
15080	13:40 X vs. 29:1 X	-1.825	-1.986 to -1.664	Yes
15081	13:40 X vs. 29:10 X	-1.039	-1.200 to -0.8782	Yes
15082	13:40 X vs. 29:40 X	-0.4867	-0.6306 to -0.3427	Yes
15083	13:40 X vs. 29:50 X	-0.2753	-0.4193 to -0.1314	Yes
15084	13:40 X vs. 30:0 X	-1.454	-1.615 to -1.293	Yes
15085	13:40 X vs. 30:1 X	-1.776	-1.937 to -1.615	Yes
15086	13:40 X vs. 30:10 X	-1.114	-1.275 to -0.9527	Yes
15087	13:40 X vs. 30:40 X	-0.4137	-0.5576 to -0.2697	Yes
15088	13:40 X vs. 30:50 X	-0.3277	-0.4716 to -0.1837	Yes
15089	13:40 X vs. 31:0 X	-1.772	-1.933 to -1.611	Yes
15090	13:40 X vs. 31:1 X	-1.763	-1.924 to -1.602	Yes
15091	13:40 X vs. 31:10 X	-1.245	-1.406 to -1.084	Yes
15092	13:40 X vs. 31:40 X	-0.5053	-0.6493 to -0.3614	Yes
15093	13:40 X vs. 31:50 X	-0.3067	-0.4506 to -0.1627	Yes
15094	13:40 X vs. 32:0 X	-2.364	-2.508 to -2.220	Yes
15095	13:40 X vs. 32:1 X	-1.820	-1.981 to -1.659	Yes
15096	13:40 X vs. 32:10 X	-1.676	-1.820 to -1.532	Yes
15097	13:40 X vs. 32:40 X	-0.5017	-0.6456 to -0.3577	Yes
15098	13:40 X vs. 32:50 X	-0.3063	-0.4503 to -0.1624	Yes
15099	13:40 X vs. 33:0 X	-2.367	-2.511 to -2.223	Yes
15100	13:40 X vs. 33:1 X	-2.182	-2.326 to -2.038	Yes
15101	13:40 X vs. 33:10 X	-1.842	-1.986 to -1.698	Yes
15102	13:40 X vs. 33:40 X	-0.5203	-0.6643 to -0.3764	Yes
15103	13:40 X vs. 33:50 X	-0.3213	-0.4653 to -0.1774	Yes
15104	13:40 X vs. 34:0 X	-2.292	-2.436 to -2.148	Yes
15105	13:40 X vs. 34:1 X	-2.299	-2.443 to -2.155	Yes
15106	13:40 X vs. 34:10 X	-1.933	-2.077 to -1.789	Yes
15107	13:40 X vs. 34:40 X	-0.5323	-0.6763 to -0.3884	Yes
15108	13:40 X vs. 34:50 X	-0.4703	-0.6143 to -0.3264	Yes
15109	13:40 X vs. 35:0 X	-2.641	-2.785 to -2.497	Yes
15110	13:40 X vs. 35:1 X	-2.406	-2.550 to -2.262	Yes
15111	13:40 X vs. 35:10 X	-1.894	-2.038 to -1.750	Yes
15112	13:40 X vs. 35:40 X	-0.5413	-0.6853 to -0.3974	Yes
15113	13:40 X vs. 35:50 X	-0.4830	-0.6270 to -0.3390	Yes
15114	13:40 X vs. 36:0 X	-2.847	-3.008 to -2.686	Yes
15115	13:40 X vs. 36:1 X	-2.543	-2.687 to -2.399	Yes
15116	13:40 X vs. 36:10 X	-1.900	-2.044 to -1.756	Yes
15117	13:40 X vs. 36:40 X	-0.5967	-0.7406 to -0.4527	Yes
15118	13:40 X vs. 36:50 X	-0.4463	-0.5903 to -0.3024	Yes
15119	13:40 X vs. 37:0 X	-3.231	-3.375 to -3.087	Yes
15120	13:40 X vs. 37:1 X	-2.613	-2.757 to -2.469	Yes

2way ANOVA<br>Multiple comparisons	A<br>Data Set-A<br>Y	B<br>Data Set-B<br>Y	C<br>Data Set-C<br>Y
15121 13:40 X vs. 37:10 X	-1.902	-2.046 to -1.758	Yes
15122 13:40 X vs. 37:40 X	-0.6340	-0.7780 to -0.4900	Yes
15123 13:40 X vs. 37:50 X	-0.4550	-0.5990 to -0.3110	Yes
15124 13:40 X vs. 38:0 X	-3.176	-3.320 to -3.032	Yes
15125 13:40 X vs. 38:1 X	-2.732	-2.876 to -2.588	Yes
15126 13:40 X vs. 38:10 X	-2.177	-2.321 to -2.033	Yes
15127 13:40 X vs. 38:40 X	-0.6383	-0.7823 to -0.4944	Yes
15128 13:40 X vs. 38:50 X	-0.4590	-0.6030 to -0.3150	Yes
15129 13:40 X vs. 39:0 X	-3.407	-3.551 to -3.263	Yes
15130 13:40 X vs. 39:1 X	-3.298	-3.442 to -3.154	Yes
15131 13:40 X vs. 39:10 X	-2.322	-2.466 to -2.178	Yes
15132 13:40 X vs. 39:40 X	-0.6443	-0.7883 to -0.5004	Yes
15133 13:40 X vs. 39:50 X	-0.4900	-0.6340 to -0.3460	Yes
15134 13:40 X vs. 40:0 X	-3.448	-3.592 to -3.304	Yes
15135 13:40 X vs. 40:1 X	-3.374	-3.518 to -3.230	Yes
15136 13:40 X vs. 40:10 X	-2.578	-2.722 to -2.434	Yes
15137 13:40 X vs. 40:40 X	-0.6490	-0.7930 to -0.5050	Yes
15138 13:40 X vs. 40:50 X	-0.4963	-0.6403 to -0.3524	Yes
15139 13:40 X vs. 41:0 X	-3.489	-3.633 to -3.345	Yes
15140 13:40 X vs. 41:1 X	-3.488	-3.632 to -3.344	Yes
15141 13:40 X vs. 41:10 X	-2.632	-2.776 to -2.488	Yes
15142 13:40 X vs. 41:40 X	-0.5963	-0.7403 to -0.4524	Yes
15143 13:40 X vs. 41:50 X	-0.5090	-0.6530 to -0.3650	Yes
15144 13:40 X vs. 42:0 X	-3.630	-3.774 to -3.486	Yes
15145 13:40 X vs. 42:1 X	-3.650	-3.794 to -3.506	Yes
15146 13:40 X vs. 42:10 X	-2.632	-2.776 to -2.488	Yes
15147 13:40 X vs. 42:40 X	-0.6090	-0.7530 to -0.4650	Yes
15148 13:40 X vs. 42:50 X	-0.5123	-0.6563 to -0.3684	Yes
15149 13:40 X vs. 43:0 X	-3.720	-3.864 to -3.576	Yes
15150 13:40 X vs. 43:1 X	-3.635	-3.779 to -3.491	Yes
15151 13:40 X vs. 43:10 X	-3.350	-3.494 to -3.206	Yes
15152 13:40 X vs. 43:40 X	-0.6073	-0.7513 to -0.4634	Yes
15153 13:40 X vs. 43:50 X	-0.5127	-0.6566 to -0.3687	Yes
15154 13:40 X vs. 44:0 X	-3.744	-3.888 to -3.600	Yes
15155 13:40 X vs. 44:1 X	-3.634	-3.778 to -3.490	Yes
15156 13:40 X vs. 44:10 X	-3.414	-3.558 to -3.270	Yes
15157 13:40 X vs. 44:40 X	-0.6093	-0.7533 to -0.4654	Yes
15158 13:40 X vs. 44:50 X	-0.4450	-0.5890 to -0.3010	Yes
15159 13:40 X vs. 45:0 X	-3.609	-3.753 to -3.465	Yes
15160 13:40 X vs. 45:1 X	-3.411	-3.555 to -3.267	Yes
15161 13:40 X vs. 45:10 X	-3.293	-3.437 to -3.149	Yes
15162 13:40 X vs. 45:40 X	-0.6107	-0.7546 to -0.4667	Yes
15163 13:40 X vs. 45:50 X	-0.4420	-0.5860 to -0.2980	Yes
15164 13:40 X vs. 46:0 X	-3.413	-3.557 to -3.269	Yes
15165 13:40 X vs. 46:1 X	-3.380	-3.524 to -3.236	Yes

	2way ANOVA<br>Multiple comparisons	A<br>Data Set-A<br>Y	B<br>Data Set-B<br>Y	C<br>Data Set-C<br>Y
15166	13:40 X vs. 46:10 X	-3.288	-3.432 to -3.144	Yes
15167	13:40 X vs. 46:40 X	-0.6243	-0.7683 to -0.4804	Yes
15168	13:40 X vs. 46:50 X	-0.4350	-0.5790 to -0.2910	Yes
15169	13:40 X vs. 47:0 X	-3.389	-3.533 to -3.245	Yes
15170	13:40 X vs. 47:1 X	-3.293	-3.437 to -3.149	Yes
15171	13:40 X vs. 47:10 X	-3.274	-3.418 to -3.130	Yes
15172	13:40 X vs. 47:40 X	-0.6343	-0.7783 to -0.4904	Yes
15173	13:40 X vs. 47:50 X	-0.3930	-0.5370 to -0.2490	Yes
15174	13:40 X vs. 48:0 X	-3.376	-3.537 to -3.215	Yes
15175	13:40 X vs. 48:1 X	-3.280	-3.424 to -3.136	Yes
15176	13:40 X vs. 48:10 X	-3.274	-3.418 to -3.130	Yes
15177	13:40 X vs. 48:40 X	-0.6407	-0.7846 to -0.4967	Yes
15178	13:40 X vs. 48:50 X	-0.4003	-0.5443 to -0.2564	Yes
15179	13:40 X vs. 49:0 X	-3.376	-3.520 to -3.232	Yes
15180	13:40 X vs. 49:1 X	-3.280	-3.424 to -3.136	Yes
15181	13:40 X vs. 49:10 X	-3.274	-3.418 to -3.130	Yes
15182	13:40 X vs. 49:40 X	-0.6450	-0.7890 to -0.5010	Yes
15183	13:40 X vs. 49:50 X	-0.3997	-0.5436 to -0.2557	Yes
15184	13:40 X vs. 50:0 X	-3.376	-3.520 to -3.232	Yes
15185	13:40 X vs. 50:1 X	-3.280	-3.424 to -3.136	Yes
15186	13:40 X vs. 50:10 X	-3.274	-3.418 to -3.130	Yes
15187	13:40 X vs. 50:40 X	-0.6527	-0.7966 to -0.5087	Yes
15188	13:40 X vs. 50:50 X	-0.3993	-0.5433 to -0.2554	Yes
15189	13:50 X vs. 14:0 X	-0.002000	-0.1460 to 0.1420	No
15190	13:50 X vs. 14:1 X	0.006333	-0.1376 to 0.1503	No
15191	13:50 X vs. 14:10 X	-0.007667	-0.1516 to 0.1363	No
15192	13:50 X vs. 14:40 X	-0.004333	-0.1483 to 0.1396	No
15193	13:50 X vs. 14:50 X	-0.008333	-0.1523 to 0.1356	No
15194	13:50 X vs. 15:0 X	-0.01433	-0.1583 to 0.1296	No
15195	13:50 X vs. 15:1 X	0.005667	-0.1383 to 0.1496	No
15196	13:50 X vs. 15:10 X	-0.02167	-0.1656 to 0.1223	No
15197	13:50 X vs. 15:40 X	-0.004000	-0.1480 to 0.1400	No
15198	13:50 X vs. 15:50 X	-0.02167	-0.1656 to 0.1223	No
15199	13:50 X vs. 16:0 X	-0.1067	-0.2506 to 0.03729	No
15200	13:50 X vs. 16:1 X	0.002333	-0.1416 to 0.1463	No
15201	13:50 X vs. 16:10 X	-0.02333	-0.1673 to 0.1206	No
15202	13:50 X vs. 16:40 X	-0.04267	-0.1866 to 0.1013	No
15203	13:50 X vs. 16:50 X	-0.08500	-0.2290 to 0.05896	No
15204	13:50 X vs. 17:0 X	-0.1593	-0.3033 to -0.01537	Yes
15205	13:50 X vs. 17:1 X	-0.07367	-0.2176 to 0.07029	No
15206	13:50 X vs. 17:10 X	-0.1303	-0.2743 to 0.01363	No
15207	13:50 X vs. 17:40 X	-0.05367	-0.1976 to 0.09029	No
15208	13:50 X vs. 17:50 X	-0.0990	-0.2430 to 0.04496	No
15209	13:50 X vs. 18:0 X	-0.2080	-0.3520 to -0.06404	Yes
15210	13:50 X vs. 18:1 X	-0.1163	-0.2603 to 0.02763	No

2way ANOVA<br>Multiple comparisons	A<br>Data Set-A<br>Y	B<br>Data Set-B<br>Y	C<br>Data Set-C<br>Y
15211 13:50 X vs. 18:10 X	-0.2087	-0.3526 to -0.06471	Yes
15212 13:50 X vs. 18:40 X	-0.0580	-0.2020 to 0.08596	No
15213 13:50 X vs. 18:50 X	-0.1030	-0.2470 to 0.04096	No
15214 13:50 X vs. 19:0 X	-0.2430	-0.3870 to -0.09904	Yes
15215 13:50 X vs. 19:1 X	-0.2310	-0.3750 to -0.08704	Yes
15216 13:50 X vs. 19:10 X	-0.2287	-0.3726 to -0.08471	Yes
15217 13:50 X vs. 19:40 X	-0.08667	-0.2306 to 0.05729	No
15218 13:50 X vs. 19:50 X	-0.09767	-0.2416 to 0.04629	No
15219 13:50 X vs. 20:0 X	-0.3227	-0.4666 to -0.1787	Yes
15220 13:50 X vs. 20:1 X	-0.1420	-0.2860 to 0.001960	No
15221 13:50 X vs. 20:10 X	-0.2310	-0.3750 to -0.08704	Yes
15222 13:50 X vs. 20:40 X	-0.09833	-0.2423 to 0.04563	No
15223 13:50 X vs. 20:50 X	-0.1423	-0.2863 to 0.001626	No
15224 13:50 X vs. 21:0 X	-0.3460	-0.4900 to -0.2020	Yes
15225 13:50 X vs. 21:1 X	-0.2990	-0.4430 to -0.1550	Yes
15226 13:50 X vs. 21:10 X	-0.2960	-0.4400 to -0.1520	Yes
15227 13:50 X vs. 21:40 X	-0.1713	-0.3153 to -0.02737	Yes
15228 13:50 X vs. 21:50 X	-0.2617	-0.4056 to -0.1177	Yes
15229 13:50 X vs. 22:0 X	-0.3877	-0.5316 to -0.2437	Yes
15230 13:50 X vs. 22:1 X	-0.3823	-0.5263 to -0.2384	Yes
15231 13:50 X vs. 22:10 X	-0.3777	-0.5216 to -0.2337	Yes
15232 13:50 X vs. 22:40 X	-0.2780	-0.4220 to -0.1340	Yes
15233 13:50 X vs. 22:50 X	-0.3407	-0.4846 to -0.1967	Yes
15234 13:50 X vs. 23:0 X	-0.4530	-0.5970 to -0.3090	Yes
15235 13:50 X vs. 23:1 X	-0.6427	-0.8036 to -0.4817	Yes
15236 13:50 X vs. 23:10 X	-0.3923	-0.5363 to -0.2484	Yes
15237 13:50 X vs. 23:40 X	-0.2727	-0.4336 to -0.1117	Yes
15238 13:50 X vs. 23:50 X	-0.3067	-0.4506 to -0.1627	Yes
15239 13:50 X vs. 24:0 X	-0.5703	-0.7143 to -0.4264	Yes
15240 13:50 X vs. 24:1 X	-0.7267	-0.8876 to -0.5657	Yes
15241 13:50 X vs. 24:10 X	-0.5542	-0.7151 to -0.3932	Yes
15242 13:50 X vs. 24:40 X	-0.2890	-0.4330 to -0.1450	Yes
15243 13:50 X vs. 24:50 X	-0.2107	-0.3546 to -0.06671	Yes
15244 13:50 X vs. 25:0 X	-0.9077	-1.069 to -0.7467	Yes
15245 13:50 X vs. 25:1 X	-1.097	-1.258 to -0.9362	Yes
15246 13:50 X vs. 25:10 X	-0.4587	-0.6623 to -0.2551	Yes
15247 13:50 X vs. 25:40 X	-0.2910	-0.4350 to -0.1470	Yes
15248 13:50 X vs. 25:50 X	-0.2653	-0.4093 to -0.1214	Yes
15249 13:50 X vs. 26:0 X	-1.120	-1.281 to -0.9592	Yes
15250 13:50 X vs. 26:1 X	-1.233	-1.394 to -1.072	Yes
15251 13:50 X vs. 26:10 X	-0.9197	-1.081 to -0.7587	Yes
15252 13:50 X vs. 26:40 X	-0.3653	-0.5093 to -0.2214	Yes
15253 13:50 X vs. 26:50 X	-0.2257	-0.3696 to -0.08171	Yes
15254 13:50 X vs. 27:0 X	-1.308	-1.452 to -1.164	Yes
15255 13:50 X vs. 27:1 X	-1.399	-1.560 to -1.238	Yes

	2way ANOVA<br>Multiple comparisons	A<br>Data Set-A<br>Y	B<br>Data Set-B<br>Y	C<br>Data Set-C<br>Y
15256	13:50 X vs. 27:10 X	-1.211	-1.372 to -1.050	Yes
15257	13:50 X vs. 27:40 X	-0.3653	-0.5093 to -0.2214	Yes
15258	13:50 X vs. 27:50 X	-0.2250	-0.3690 to -0.08104	Yes
15259	13:50 X vs. 28:0 X	-1.257	-1.418 to -1.096	Yes
15260	13:50 X vs. 28:1 X	-1.298	-1.442 to -1.154	Yes
15261	13:50 X vs. 28:10 X	-1.100	-1.261 to -0.9387	Yes
15262	13:50 X vs. 28:40 X	-0.3777	-0.5216 to -0.2337	Yes
15263	13:50 X vs. 28:50 X	-0.2730	-0.4170 to -0.1290	Yes
15264	13:50 X vs. 29:0 X	-1.739	-1.900 to -1.578	Yes
15265	13:50 X vs. 29:1 X	-1.824	-1.985 to -1.663	Yes
15266	13:50 X vs. 29:10 X	-1.038	-1.199 to -0.8772	Yes
15267	13:50 X vs. 29:40 X	-0.4857	-0.6296 to -0.3417	Yes
15268	13:50 X vs. 29:50 X	-0.2743	-0.4183 to -0.1304	Yes
15269	13:50 X vs. 30:0 X	-1.453	-1.614 to -1.292	Yes
15270	13:50 X vs. 30:1 X	-1.775	-1.936 to -1.614	Yes
15271	13:50 X vs. 30:10 X	-1.113	-1.274 to -0.9517	Yes
15272	13:50 X vs. 30:40 X	-0.4127	-0.5566 to -0.2687	Yes
15273	13:50 X vs. 30:50 X	-0.3267	-0.4706 to -0.1827	Yes
15274	13:50 X vs. 31:0 X	-1.771	-1.932 to -1.610	Yes
15275	13:50 X vs. 31:1 X	-1.762	-1.923 to -1.601	Yes
15276	13:50 X vs. 31:10 X	-1.244	-1.405 to -1.083	Yes
15277	13:50 X vs. 31:40 X	-0.5043	-0.6483 to -0.3604	Yes
15278	13:50 X vs. 31:50 X	-0.3057	-0.4496 to -0.1617	Yes
15279	13:50 X vs. 32:0 X	-2.363	-2.507 to -2.219	Yes
15280	13:50 X vs. 32:1 X	-1.819	-1.980 to -1.658	Yes
15281	13:50 X vs. 32:10 X	-1.675	-1.819 to -1.531	Yes
15282	13:50 X vs. 32:40 X	-0.5007	-0.6446 to -0.3567	Yes
15283	13:50 X vs. 32:50 X	-0.3053	-0.4493 to -0.1614	Yes
15284	13:50 X vs. 33:0 X	-2.366	-2.510 to -2.222	Yes
15285	13:50 X vs. 33:1 X	-2.181	-2.325 to -2.037	Yes
15286	13:50 X vs. 33:10 X	-1.841	-1.985 to -1.697	Yes
15287	13:50 X vs. 33:40 X	-0.5193	-0.6633 to -0.3754	Yes
15288	13:50 X vs. 33:50 X	-0.3203	-0.4643 to -0.1764	Yes
15289	13:50 X vs. 34:0 X	-2.291	-2.435 to -2.147	Yes
15290	13:50 X vs. 34:1 X	-2.298	-2.442 to -2.154	Yes
15291	13:50 X vs. 34:10 X	-1.932	-2.076 to -1.788	Yes
15292	13:50 X vs. 34:40 X	-0.5313	-0.6753 to -0.3874	Yes
15293	13:50 X vs. 34:50 X	-0.4693	-0.6133 to -0.3254	Yes
15294	13:50 X vs. 35:0 X	-2.640	-2.784 to -2.496	Yes
15295	13:50 X vs. 35:1 X	-2.405	-2.549 to -2.261	Yes
15296	13:50 X vs. 35:10 X	-1.893	-2.037 to -1.749	Yes
15297	13:50 X vs. 35:40 X	-0.5403	-0.6843 to -0.3964	Yes
15298	13:50 X vs. 35:50 X	-0.4820	-0.6260 to -0.3380	Yes
15299	13:50 X vs. 36:0 X	-2.846	-3.007 to -2.685	Yes
15300	13:50 X vs. 36:1 X	-2.542	-2.686 to -2.398	Yes

	2way ANOVA<br>Multiple comparisons	A<br>Data Set-A<br>Y	B<br>Data Set-B<br>Y	C<br>Data Set-C<br>Y
15301	13:**50 X** vs. 36:**10 X**	-1.899	-2.043 to -1.755	Yes
15302	13:**50 X** vs. 36:**40 X**	-0.5957	-0.7396 to -0.4517	Yes
15303	13:**50 X** vs. 36:**50 X**	-0.4453	-0.5893 to -0.3014	Yes
15304	13:**50 X** vs. 37:**0 X**	-3.230	-3.374 to -3.086	Yes
15305	13:**50 X** vs. 37:**1 X**	-2.612	-2.756 to -2.468	Yes
15306	13:**50 X** vs. 37:**10 X**	-1.901	-2.045 to -1.757	Yes
15307	13:**50 X** vs. 37:**40 X**	-0.6330	-0.7770 to -0.4890	Yes
15308	13:**50 X** vs. 37:**50 X**	-0.4540	-0.5980 to -0.3100	Yes
15309	13:**50 X** vs. 38:**0 X**	-3.175	-3.319 to -3.031	Yes
15310	13:**50 X** vs. 38:**1 X**	-2.731	-2.875 to -2.587	Yes
15311	13:**50 X** vs. 38:**10 X**	-2.176	-2.320 to -2.032	Yes
15312	13:**50 X** vs. 38:**40 X**	-0.6373	-0.7813 to -0.4934	Yes
15313	13:**50 X** vs. 38:**50 X**	-0.4580	-0.6020 to -0.3140	Yes
15314	13:**50 X** vs. 39:**0 X**	-3.406	-3.550 to -3.262	Yes
15315	13:**50 X** vs. 39:**1 X**	-3.297	-3.441 to -3.153	Yes
15316	13:**50 X** vs. 39:**10 X**	-2.321	-2.465 to -2.177	Yes
15317	13:**50 X** vs. 39:**40 X**	-0.6433	-0.7873 to -0.4994	Yes
15318	13:**50 X** vs. 39:**50 X**	-0.4890	-0.6330 to -0.3450	Yes
15319	13:**50 X** vs. 40:**0 X**	-3.447	-3.591 to -3.303	Yes
15320	13:**50 X** vs. 40:**1 X**	-3.373	-3.517 to -3.229	Yes
15321	13:**50 X** vs. 40:**10 X**	-2.577	-2.721 to -2.433	Yes
15322	13:**50 X** vs. 40:**40 X**	-0.6480	-0.7920 to -0.5040	Yes
15323	13:**50 X** vs. 40:**50 X**	-0.4953	-0.6393 to -0.3514	Yes
15324	13:**50 X** vs. 41:**0 X**	-3.488	-3.632 to -3.344	Yes
15325	13:**50 X** vs. 41:**1 X**	-3.487	-3.631 to -3.343	Yes
15326	13:**50 X** vs. 41:**10 X**	-2.631	-2.775 to -2.487	Yes
15327	13:**50 X** vs. 41:**40 X**	-0.5953	-0.7393 to -0.4514	Yes
15328	13:**50 X** vs. 41:**50 X**	-0.5080	-0.6520 to -0.3640	Yes
15329	13:**50 X** vs. 42:**0 X**	-3.629	-3.773 to -3.485	Yes
15330	13:**50 X** vs. 42:**1 X**	-3.649	-3.793 to -3.505	Yes
15331	13:**50 X** vs. 42:**10 X**	-2.631	-2.775 to -2.487	Yes
15332	13:**50 X** vs. 42:**40 X**	-0.6080	-0.7520 to -0.4640	Yes
15333	13:**50 X** vs. 42:**50 X**	-0.5113	-0.6553 to -0.3674	Yes
15334	13:**50 X** vs. 43:**0 X**	-3.719	-3.863 to -3.575	Yes
15335	13:**50 X** vs. 43:**1 X**	-3.634	-3.778 to -3.490	Yes
15336	13:**50 X** vs. 43:**10 X**	-3.349	-3.493 to -3.205	Yes
15337	13:**50 X** vs. 43:**40 X**	-0.6063	-0.7503 to -0.4624	Yes
15338	13:**50 X** vs. 43:**50 X**	-0.5117	-0.6556 to -0.3677	Yes
15339	13:**50 X** vs. 44:**0 X**	-3.743	-3.887 to -3.599	Yes
15340	13:**50 X** vs. 44:**1 X**	-3.633	-3.777 to -3.489	Yes
15341	13:**50 X** vs. 44:**10 X**	-3.413	-3.557 to -3.269	Yes
15342	13:**50 X** vs. 44:**40 X**	-0.6083	-0.7523 to -0.4644	Yes
15343	13:**50 X** vs. 44:**50 X**	-0.4440	-0.5880 to -0.3000	Yes
15344	13:**50 X** vs. 45:**0 X**	-3.608	-3.752 to -3.464	Yes
15345	13:**50 X** vs. 45:**1 X**	-3.410	-3.554 to -3.266	Yes

	2way ANOVA<br>Multiple comparisons	A<br>Data Set-A<br>Y	B<br>Data Set-B<br>Y	C<br>Data Set-C<br>Y
15346	13:**50 X** vs. 45:**10 X**	-3.292	-3.436 to -3.148	Yes
15347	13:**50 X** vs. 45:**40 X**	-0.6097	-0.7536 to -0.4657	Yes
15348	13:**50 X** vs. 45:**50 X**	-0.4410	-0.5850 to -0.2970	Yes
15349	13:**50 X** vs. 46:**0 X**	-3.412	-3.556 to -3.268	Yes
15350	13:**50 X** vs. 46:**1 X**	-3.379	-3.523 to -3.235	Yes
15351	13:**50 X** vs. 46:**10 X**	-3.287	-3.431 to -3.143	Yes
15352	13:**50 X** vs. 46:**40 X**	-0.6233	-0.7673 to -0.4794	Yes
15353	13:**50 X** vs. 46:**50 X**	-0.4340	-0.5780 to -0.2900	Yes
15354	13:**50 X** vs. 47:**0 X**	-3.388	-3.532 to -3.244	Yes
15355	13:**50 X** vs. 47:**1 X**	-3.292	-3.436 to -3.148	Yes
15356	13:**50 X** vs. 47:**10 X**	-3.273	-3.417 to -3.129	Yes
15357	13:**50 X** vs. 47:**40 X**	-0.6333	-0.7773 to -0.4894	Yes
15358	13:**50 X** vs. 47:**50 X**	-0.3920	-0.5360 to -0.2480	Yes
15359	13:**50 X** vs. 48:**0 X**	-3.375	-3.536 to -3.214	Yes
15360	13:**50 X** vs. 48:**1 X**	-3.279	-3.423 to -3.135	Yes
15361	13:**50 X** vs. 48:**10 X**	-3.273	-3.417 to -3.129	Yes
15362	13:**50 X** vs. 48:**40 X**	-0.6397	-0.7836 to -0.4957	Yes
15363	13:**50 X** vs. 48:**50 X**	-0.3993	-0.5433 to -0.2554	Yes
15364	13:**50 X** vs. 49:**0 X**	-3.375	-3.519 to -3.231	Yes
15365	13:**50 X** vs. 49:**1 X**	-3.279	-3.423 to -3.135	Yes
15366	13:**50 X** vs. 49:**10 X**	-3.273	-3.417 to -3.129	Yes
15367	13:**50 X** vs. 49:**40 X**	-0.6440	-0.7880 to -0.5000	Yes
15368	13:**50 X** vs. 49:**50 X**	-0.3987	-0.5426 to -0.2547	Yes
15369	13:**50 X** vs. 50:**0 X**	-3.375	-3.519 to -3.231	Yes
15370	13:**50 X** vs. 50:**1 X**	-3.279	-3.423 to -3.135	Yes
15371	13:**50 X** vs. 50:**10 X**	-3.273	-3.417 to -3.129	Yes
15372	13:**50 X** vs. 50:**40 X**	-0.6517	-0.7956 to -0.5077	Yes
15373	13:**50 X** vs. 50:**50 X**	-0.3983	-0.5423 to -0.2544	Yes
15374	14:**0 X** vs. 14:**1 X**	0.008333	-0.1356 to 0.1523	No
15375	14:**0 X** vs. 14:**10 X**	-0.005667	-0.1496 to 0.1383	No
15376	14:**0 X** vs. 14:**40 X**	-0.002333	-0.1463 to 0.1416	No
15377	14:**0 X** vs. 14:**50 X**	-0.006333	-0.1503 to 0.1376	No
15378	14:**0 X** vs. 15:**0 X**	-0.01233	-0.1563 to 0.1316	No
15379	14:**0 X** vs. 15:**1 X**	0.007667	-0.1363 to 0.1516	No
15380	14:**0 X** vs. 15:**10 X**	-0.01967	-0.1636 to 0.1243	No
15381	14:**0 X** vs. 15:**40 X**	-0.0020	-0.1460 to 0.1420	No
15382	14:**0 X** vs. 15:**50 X**	-0.01967	-0.1636 to 0.1243	No
15383	14:**0 X** vs. 16:**0 X**	-0.1047	-0.2486 to 0.03929	No
15384	14:**0 X** vs. 16:**1 X**	0.004333	-0.1396 to 0.1483	No
15385	14:**0 X** vs. 16:**10 X**	-0.02133	-0.1653 to 0.1226	No
15386	14:**0 X** vs. 16:**40 X**	-0.04067	-0.1846 to 0.1033	No
15387	14:**0 X** vs. 16:**50 X**	-0.0830	-0.2270 to 0.06096	No
15388	14:**0 X** vs. 17:**0 X**	-0.1573	-0.3013 to -0.01337	Yes
15389	14:**0 X** vs. 17:**1 X**	-0.07167	-0.2156 to 0.07229	No
15390	14:**0 X** vs. 17:**10 X**	-0.1283	-0.2723 to 0.01563	No

	**2way ANOVA**<br>Multiple comparisons	**A**<br>Data Set-A<br>**Y**	**B**<br>Data Set-B<br>**Y**	**C**<br>Data Set-C<br>**Y**
**15391**	14:**0 X** vs. 17:**40 X**	-0.05167	-0.1956 to 0.09229	No
**15392**	14:**0 X** vs. 17:**50 X**	-0.0970	-0.2410 to 0.04696	No
**15393**	14:**0 X** vs. 18:**0 X**	-0.2060	-0.3500 to -0.06204	Yes
**15394**	14:**0 X** vs. 18:**1 X**	-0.1143	-0.2583 to 0.02963	No
**15395**	14:**0 X** vs. 18:**10 X**	-0.2067	-0.3506 to -0.06271	Yes
**15396**	14:**0 X** vs. 18:**40 X**	-0.0560	-0.2000 to 0.08796	No
**15397**	14:**0 X** vs. 18:**50 X**	-0.1010	-0.2450 to 0.04296	No
**15398**	14:**0 X** vs. 19:**0 X**	-0.2410	-0.3850 to -0.09704	Yes
**15399**	14:**0 X** vs. 19:**1 X**	-0.2290	-0.3730 to -0.08504	Yes
**15400**	14:**0 X** vs. 19:**10 X**	-0.2267	-0.3706 to -0.08271	Yes
**15401**	14:**0 X** vs. 19:**40 X**	-0.08467	-0.2286 to 0.05929	No
**15402**	14:**0 X** vs. 19:**50 X**	-0.09567	-0.2396 to 0.04829	No
**15403**	14:**0 X** vs. 20:**0 X**	-0.3207	-0.4646 to -0.1767	Yes
**15404**	14:**0 X** vs. 20:**1 X**	-0.1400	-0.2840 to 0.003960	No
**15405**	14:**0 X** vs. 20:**10 X**	-0.2290	-0.3730 to -0.08504	Yes
**15406**	14:**0 X** vs. 20:**40 X**	-0.09633	-0.2403 to 0.04763	No
**15407**	14:**0 X** vs. 20:**50 X**	-0.1403	-0.2843 to 0.003626	No
**15408**	14:**0 X** vs. 21:**0 X**	-0.3440	-0.4880 to -0.2000	Yes
**15409**	14:**0 X** vs. 21:**1 X**	-0.2970	-0.4410 to -0.1530	Yes
**15410**	14:**0 X** vs. 21:**10 X**	-0.2940	-0.4380 to -0.1500	Yes
**15411**	14:**0 X** vs. 21:**40 X**	-0.1693	-0.3133 to -0.02537	Yes
**15412**	14:**0 X** vs. 21:**50 X**	-0.2597	-0.4036 to -0.1157	Yes
**15413**	14:**0 X** vs. 22:**0 X**	-0.3857	-0.5296 to -0.2417	Yes
**15414**	14:**0 X** vs. 22:**1 X**	-0.3803	-0.5243 to -0.2364	Yes
**15415**	14:**0 X** vs. 22:**10 X**	-0.3757	-0.5196 to -0.2317	Yes
**15416**	14:**0 X** vs. 22:**40 X**	-0.2760	-0.4200 to -0.1320	Yes
**15417**	14:**0 X** vs. 22:**50 X**	-0.3387	-0.4826 to -0.1947	Yes
**15418**	14:**0 X** vs. 23:**0 X**	-0.4510	-0.5950 to -0.3070	Yes
**15419**	14:**0 X** vs. 23:**1 X**	-0.6407	-0.8016 to -0.4797	Yes
**15420**	14:**0 X** vs. 23:**10 X**	-0.3903	-0.5343 to -0.2464	Yes
**15421**	14:**0 X** vs. 23:**40 X**	-0.2707	-0.4316 to -0.1097	Yes
**15422**	14:**0 X** vs. 23:**50 X**	-0.3047	-0.4486 to -0.1607	Yes
**15423**	14:**0 X** vs. 24:**0 X**	-0.5683	-0.7123 to -0.4244	Yes
**15424**	14:**0 X** vs. 24:**1 X**	-0.7247	-0.8856 to -0.5637	Yes
**15425**	14:**0 X** vs. 24:**10 X**	-0.5522	-0.7131 to -0.3912	Yes
**15426**	14:**0 X** vs. 24:**40 X**	-0.2870	-0.4310 to -0.1430	Yes
**15427**	14:**0 X** vs. 24:**50 X**	-0.2087	-0.3526 to -0.06471	Yes
**15428**	14:**0 X** vs. 25:**0 X**	-0.9057	-1.067 to -0.7447	Yes
**15429**	14:**0 X** vs. 25:**1 X**	-1.095	-1.256 to -0.9342	Yes
**15430**	14:**0 X** vs. 25:**10 X**	-0.4567	-0.6603 to -0.2531	Yes
**15431**	14:**0 X** vs. 25:**40 X**	-0.2890	-0.4330 to -0.1450	Yes
**15432**	14:**0 X** vs. 25:**50 X**	-0.2633	-0.4073 to -0.1194	Yes
**15433**	14:**0 X** vs. 26:**0 X**	-1.118	-1.279 to -0.9572	Yes
**15434**	14:**0 X** vs. 26:**1 X**	-1.231	-1.392 to -1.070	Yes
**15435**	14:**0 X** vs. 26:**10 X**	-0.9177	-1.079 to -0.7567	Yes

2way ANOVA<br>Multiple comparisons		A<br>Data Set-A<br>Y	B<br>Data Set-B<br>Y	C<br>Data Set-C<br>Y
15436	14:**0 X** vs. 26:**40 X**	-0.3633	-0.5073 to -0.2194	Yes
15437	14:**0 X** vs. 26:**50 X**	-0.2237	-0.3676 to -0.07971	Yes
15438	14:**0 X** vs. 27:**0 X**	-1.306	-1.450 to -1.162	Yes
15439	14:**0 X** vs. 27:**1 X**	-1.397	-1.558 to -1.236	Yes
15440	14:**0 X** vs. 27:**10 X**	-1.209	-1.370 to -1.048	Yes
15441	14:**0 X** vs. 27:**40 X**	-0.3633	-0.5073 to -0.2194	Yes
15442	14:**0 X** vs. 27:**50 X**	-0.2230	-0.3670 to -0.07904	Yes
15443	14:**0 X** vs. 28:**0 X**	-1.255	-1.416 to -1.094	Yes
15444	14:**0 X** vs. 28:**1 X**	-1.296	-1.440 to -1.152	Yes
15445	14:**0 X** vs. 28:**10 X**	-1.098	-1.259 to -0.9367	Yes
15446	14:**0 X** vs. 28:**40 X**	-0.3757	-0.5196 to -0.2317	Yes
15447	14:**0 X** vs. 28:**50 X**	-0.2710	-0.4150 to -0.1270	Yes
15448	14:**0 X** vs. 29:**0 X**	-1.737	-1.898 to -1.576	Yes
15449	14:**0 X** vs. 29:**1 X**	-1.822	-1.983 to -1.661	Yes
15450	14:**0 X** vs. 29:**10 X**	-1.036	-1.197 to -0.8752	Yes
15451	14:**0 X** vs. 29:**40 X**	-0.4837	-0.6276 to -0.3397	Yes
15452	14:**0 X** vs. 29:**50 X**	-0.2723	-0.4163 to -0.1284	Yes
15453	14:**0 X** vs. 30:**0 X**	-1.451	-1.612 to -1.290	Yes
15454	14:**0 X** vs. 30:**1 X**	-1.773	-1.934 to -1.612	Yes
15455	14:**0 X** vs. 30:**10 X**	-1.111	-1.272 to -0.9497	Yes
15456	14:**0 X** vs. 30:**40 X**	-0.4107	-0.5546 to -0.2667	Yes
15457	14:**0 X** vs. 30:**50 X**	-0.3247	-0.4686 to -0.1807	Yes
15458	14:**0 X** vs. 31:**0 X**	-1.769	-1.930 to -1.608	Yes
15459	14:**0 X** vs. 31:**1 X**	-1.760	-1.921 to -1.599	Yes
15460	14:**0 X** vs. 31:**10 X**	-1.242	-1.403 to -1.081	Yes
15461	14:**0 X** vs. 31:**40 X**	-0.5023	-0.6463 to -0.3584	Yes
15462	14:**0 X** vs. 31:**50 X**	-0.3037	-0.4476 to -0.1597	Yes
15463	14:**0 X** vs. 32:**0 X**	-2.361	-2.505 to -2.217	Yes
15464	14:**0 X** vs. 32:**1 X**	-1.817	-1.978 to -1.656	Yes
15465	14:**0 X** vs. 32:**10 X**	-1.673	-1.817 to -1.529	Yes
15466	14:**0 X** vs. 32:**40 X**	-0.4987	-0.6426 to -0.3547	Yes
15467	14:**0 X** vs. 32:**50 X**	-0.3033	-0.4473 to -0.1594	Yes
15468	14:**0 X** vs. 33:**0 X**	-2.364	-2.508 to -2.220	Yes
15469	14:**0 X** vs. 33:**1 X**	-2.179	-2.323 to -2.035	Yes
15470	14:**0 X** vs. 33:**10 X**	-1.839	-1.983 to -1.695	Yes
15471	14:**0 X** vs. 33:**40 X**	-0.5173	-0.6613 to -0.3734	Yes
15472	14:**0 X** vs. 33:**50 X**	-0.3183	-0.4623 to -0.1744	Yes
15473	14:**0 X** vs. 34:**0 X**	-2.289	-2.433 to -2.145	Yes
15474	14:**0 X** vs. 34:**1 X**	-2.296	-2.440 to -2.152	Yes
15475	14:**0 X** vs. 34:**10 X**	-1.930	-2.074 to -1.786	Yes
15476	14:**0 X** vs. 34:**40 X**	-0.5293	-0.6733 to -0.3854	Yes
15477	14:**0 X** vs. 34:**50 X**	-0.4673	-0.6113 to -0.3234	Yes
15478	14:**0 X** vs. 35:**0 X**	-2.638	-2.782 to -2.494	Yes
15479	14:**0 X** vs. 35:**1 X**	-2.403	-2.547 to -2.259	Yes
15480	14:**0 X** vs. 35:**10 X**	-1.891	-2.035 to -1.747	Yes

	2way ANOVA<br>Multiple comparisons	A<br>Data Set-A<br>Y	B<br>Data Set-B<br>Y	C<br>Data Set-C<br>Y
15481	14:**0 X** vs. 35:**40 X**	-0.5383	-0.6823 to -0.3944	Yes
15482	14:**0 X** vs. 35:**50 X**	-0.4800	-0.6240 to -0.3360	Yes
15483	14:**0 X** vs. 36:**0 X**	-2.844	-3.005 to -2.683	Yes
15484	14:**0 X** vs. 36:**1 X**	-2.540	-2.684 to -2.396	Yes
15485	14:**0 X** vs. 36:**10 X**	-1.897	-2.041 to -1.753	Yes
15486	14:**0 X** vs. 36:**40 X**	-0.5937	-0.7376 to -0.4497	Yes
15487	14:**0 X** vs. 36:**50 X**	-0.4433	-0.5873 to -0.2994	Yes
15488	14:**0 X** vs. 37:**0 X**	-3.228	-3.372 to -3.084	Yes
15489	14:**0 X** vs. 37:**1 X**	-2.610	-2.754 to -2.466	Yes
15490	14:**0 X** vs. 37:**10 X**	-1.899	-2.043 to -1.755	Yes
15491	14:**0 X** vs. 37:**40 X**	-0.6310	-0.7750 to -0.4870	Yes
15492	14:**0 X** vs. 37:**50 X**	-0.4520	-0.5960 to -0.3080	Yes
15493	14:**0 X** vs. 38:**0 X**	-3.173	-3.317 to -3.029	Yes
15494	14:**0 X** vs. 38:**1 X**	-2.729	-2.873 to -2.585	Yes
15495	14:**0 X** vs. 38:**10 X**	-2.174	-2.318 to -2.030	Yes
15496	14:**0 X** vs. 38:**40 X**	-0.6353	-0.7793 to -0.4914	Yes
15497	14:**0 X** vs. 38:**50 X**	-0.4560	-0.6000 to -0.3120	Yes
15498	14:**0 X** vs. 39:**0 X**	-3.404	-3.548 to -3.260	Yes
15499	14:**0 X** vs. 39:**1 X**	-3.295	-3.439 to -3.151	Yes
15500	14:**0 X** vs. 39:**10 X**	-2.319	-2.463 to -2.175	Yes
15501	14:**0 X** vs. 39:**40 X**	-0.6413	-0.7853 to -0.4974	Yes
15502	14:**0 X** vs. 39:**50 X**	-0.4870	-0.6310 to -0.3430	Yes
15503	14:**0 X** vs. 40:**0 X**	-3.445	-3.589 to -3.301	Yes
15504	14:**0 X** vs. 40:**1 X**	-3.371	-3.515 to -3.227	Yes
15505	14:**0 X** vs. 40:**10 X**	-2.575	-2.719 to -2.431	Yes
15506	14:**0 X** vs. 40:**40 X**	-0.6460	-0.7900 to -0.5020	Yes
15507	14:**0 X** vs. 40:**50 X**	-0.4933	-0.6373 to -0.3494	Yes
15508	14:**0 X** vs. 41:**0 X**	-3.486	-3.630 to -3.342	Yes
15509	14:**0 X** vs. 41:**1 X**	-3.485	-3.629 to -3.341	Yes
15510	14:**0 X** vs. 41:**10 X**	-2.629	-2.773 to -2.485	Yes
15511	14:**0 X** vs. 41:**40 X**	-0.5933	-0.7373 to -0.4494	Yes
15512	14:**0 X** vs. 41:**50 X**	-0.5060	-0.6500 to -0.3620	Yes
15513	14:**0 X** vs. 42:**0 X**	-3.627	-3.771 to -3.483	Yes
15514	14:**0 X** vs. 42:**1 X**	-3.647	-3.791 to -3.503	Yes
15515	14:**0 X** vs. 42:**10 X**	-2.629	-2.773 to -2.485	Yes
15516	14:**0 X** vs. 42:**40 X**	-0.6060	-0.7500 to -0.4620	Yes
15517	14:**0 X** vs. 42:**50 X**	-0.5093	-0.6533 to -0.3654	Yes
15518	14:**0 X** vs. 43:**0 X**	-3.717	-3.861 to -3.573	Yes
15519	14:**0 X** vs. 43:**1 X**	-3.632	-3.776 to -3.488	Yes
15520	14:**0 X** vs. 43:**10 X**	-3.347	-3.491 to -3.203	Yes
15521	14:**0 X** vs. 43:**40 X**	-0.6043	-0.7483 to -0.4604	Yes
15522	14:**0 X** vs. 43:**50 X**	-0.5097	-0.6536 to -0.3657	Yes
15523	14:**0 X** vs. 44:**0 X**	-3.741	-3.885 to -3.597	Yes
15524	14:**0 X** vs. 44:**1 X**	-3.631	-3.775 to -3.487	Yes
15525	14:**0 X** vs. 44:**10 X**	-3.411	-3.555 to -3.267	Yes

2way ANOVA<br>Multiple comparisons	A<br>Data Set-A<br>Y	B<br>Data Set-B<br>Y	C<br>Data Set-C<br>Y
15526 14:0 X vs. 44:40 X	-0.6063	-0.7503 to -0.4624	Yes
15527 14:0 X vs. 44:50 X	-0.4420	-0.5860 to -0.2980	Yes
15528 14:0 X vs. 45:0 X	-3.606	-3.750 to -3.462	Yes
15529 14:0 X vs. 45:1 X	-3.408	-3.552 to -3.264	Yes
15530 14:0 X vs. 45:10 X	-3.290	-3.434 to -3.146	Yes
15531 14:0 X vs. 45:40 X	-0.6077	-0.7516 to -0.4637	Yes
15532 14:0 X vs. 45:50 X	-0.4390	-0.5830 to -0.2950	Yes
15533 14:0 X vs. 46:0 X	-3.410	-3.554 to -3.266	Yes
15534 14:0 X vs. 46:1 X	-3.377	-3.521 to -3.233	Yes
15535 14:0 X vs. 46:10 X	-3.285	-3.429 to -3.141	Yes
15536 14:0 X vs. 46:40 X	-0.6213	-0.7653 to -0.4774	Yes
15537 14:0 X vs. 46:50 X	-0.4320	-0.5760 to -0.2880	Yes
15538 14:0 X vs. 47:0 X	-3.386	-3.530 to -3.242	Yes
15539 14:0 X vs. 47:1 X	-3.290	-3.434 to -3.146	Yes
15540 14:0 X vs. 47:10 X	-3.271	-3.415 to -3.127	Yes
15541 14:0 X vs. 47:40 X	-0.6313	-0.7753 to -0.4874	Yes
15542 14:0 X vs. 47:50 X	-0.3900	-0.5340 to -0.2460	Yes
15543 14:0 X vs. 48:0 X	-3.373	-3.534 to -3.212	Yes
15544 14:0 X vs. 48:1 X	-3.277	-3.421 to -3.133	Yes
15545 14:0 X vs. 48:10 X	-3.271	-3.415 to -3.127	Yes
15546 14:0 X vs. 48:40 X	-0.6377	-0.7816 to -0.4937	Yes
15547 14:0 X vs. 48:50 X	-0.3973	-0.5413 to -0.2534	Yes
15548 14:0 X vs. 49:0 X	-3.373	-3.517 to -3.229	Yes
15549 14:0 X vs. 49:1 X	-3.277	-3.421 to -3.133	Yes
15550 14:0 X vs. 49:10 X	-3.271	-3.415 to -3.127	Yes
15551 14:0 X vs. 49:40 X	-0.6420	-0.7860 to -0.4980	Yes
15552 14:0 X vs. 49:50 X	-0.3967	-0.5406 to -0.2527	Yes
15553 14:0 X vs. 50:0 X	-3.373	-3.517 to -3.229	Yes
15554 14:0 X vs. 50:1 X	-3.277	-3.421 to -3.133	Yes
15555 14:0 X vs. 50:10 X	-3.271	-3.415 to -3.127	Yes
15556 14:0 X vs. 50:40 X	-0.6497	-0.7936 to -0.5057	Yes
15557 14:0 X vs. 50:50 X	-0.3963	-0.5403 to -0.2524	Yes
15558 14:1 X vs. 14:10 X	-0.0140	-0.1580 to 0.1300	No
15559 14:1 X vs. 14:40 X	-0.01067	-0.1546 to 0.1333	No
15560 14:1 X vs. 14:50 X	-0.01467	-0.1586 to 0.1293	No
15561 14:1 X vs. 15:0 X	-0.02067	-0.1646 to 0.1233	No
15562 14:1 X vs. 15:1 X	-0.0006667	-0.1446 to 0.1433	No
15563 14:1 X vs. 15:10 X	-0.0280	-0.1720 to 0.1160	No
15564 14:1 X vs. 15:40 X	-0.01033	-0.1543 to 0.1336	No
15565 14:1 X vs. 15:50 X	-0.0280	-0.1720 to 0.1160	No
15566 14:1 X vs. 16:0 X	-0.1130	-0.2570 to 0.03096	No
15567 14:1 X vs. 16:1 X	-0.004000	-0.1480 to 0.1400	No
15568 14:1 X vs. 16:10 X	-0.02967	-0.1736 to 0.1143	No
15569 14:1 X vs. 16:40 X	-0.0490	-0.1930 to 0.09496	No
15570 14:1 X vs. 16:50 X	-0.09133	-0.2353 to 0.05263	No

2way ANOVA Multiple comparisons	A Data Set-A Y	B Data Set-B Y	C Data Set-C Y
15571 14:**1 X** vs. 17:**0 X**	-0.1657	-0.3096 to -0.02171	Yes
15572 14:**1 X** vs. 17:**1 X**	-0.08000	-0.2240 to 0.06396	No
15573 14:**1 X** vs. 17:**10 X**	-0.1367	-0.2806 to 0.007293	No
15574 14:**1 X** vs. 17:**40 X**	-0.0600	-0.2040 to 0.08396	No
15575 14:**1 X** vs. 17:**50 X**	-0.1053	-0.2493 to 0.03863	No
15576 14:**1 X** vs. 18:**0 X**	-0.2143	-0.3583 to -0.07037	Yes
15577 14:**1 X** vs. 18:**1 X**	-0.1227	-0.2666 to 0.02129	No
15578 14:**1 X** vs. 18:**10 X**	-0.2150	-0.3590 to -0.07104	Yes
15579 14:**1 X** vs. 18:**40 X**	-0.06433	-0.2083 to 0.07963	No
15580 14:**1 X** vs. 18:**50 X**	-0.1093	-0.2533 to 0.03463	No
15581 14:**1 X** vs. 19:**0 X**	-0.2493	-0.3933 to -0.1054	Yes
15582 14:**1 X** vs. 19:**1 X**	-0.2373	-0.3813 to -0.09337	Yes
15583 14:**1 X** vs. 19:**10 X**	-0.2350	-0.3790 to -0.09104	Yes
15584 14:**1 X** vs. 19:**40 X**	-0.0930	-0.2370 to 0.05096	No
15585 14:**1 X** vs. 19:**50 X**	-0.1040	-0.2480 to 0.03996	No
15586 14:**1 X** vs. 20:**0 X**	-0.3290	-0.4730 to -0.1850	Yes
15587 14:**1 X** vs. 20:**1 X**	-0.1483	-0.2923 to -0.004374	Yes
15588 14:**1 X** vs. 20:**10 X**	-0.2373	-0.3813 to -0.09337	Yes
15589 14:**1 X** vs. 20:**40 X**	-0.1047	-0.2486 to 0.03929	No
15590 14:**1 X** vs. 20:**50 X**	-0.1487	-0.2926 to -0.004707	Yes
15591 14:**1 X** vs. 21:**0 X**	-0.3523	-0.4963 to -0.2084	Yes
15592 14:**1 X** vs. 21:**1 X**	-0.3053	-0.4493 to -0.1614	Yes
15593 14:**1 X** vs. 21:**10 X**	-0.3023	-0.4463 to -0.1584	Yes
15594 14:**1 X** vs. 21:**40 X**	-0.1777	-0.3216 to -0.03371	Yes
15595 14:**1 X** vs. 21:**50 X**	-0.2680	-0.4120 to -0.1240	Yes
15596 14:**1 X** vs. 22:**0 X**	-0.3940	-0.5380 to -0.2500	Yes
15597 14:**1 X** vs. 22:**1 X**	-0.3887	-0.5326 to -0.2447	Yes
15598 14:**1 X** vs. 22:**10 X**	-0.3840	-0.5280 to -0.2400	Yes
15599 14:**1 X** vs. 22:**40 X**	-0.2843	-0.4283 to -0.1404	Yes
15600 14:**1 X** vs. 22:**50 X**	-0.3470	-0.4910 to -0.2030	Yes
15601 14:**1 X** vs. 23:**0 X**	-0.4593	-0.6033 to -0.3154	Yes
15602 14:**1 X** vs. 23:**1 X**	-0.6490	-0.8100 to -0.4880	Yes
15603 14:**1 X** vs. 23:**10 X**	-0.3987	-0.5426 to -0.2547	Yes
15604 14:**1 X** vs. 23:**40 X**	-0.2790	-0.4400 to -0.1180	Yes
15605 14:**1 X** vs. 23:**50 X**	-0.3130	-0.4570 to -0.1690	Yes
15606 14:**1 X** vs. 24:**0 X**	-0.5767	-0.7206 to -0.4327	Yes
15607 14:**1 X** vs. 24:**1 X**	-0.7330	-0.8940 to -0.5720	Yes
15608 14:**1 X** vs. 24:**10 X**	-0.5605	-0.7215 to -0.3995	Yes
15609 14:**1 X** vs. 24:**40 X**	-0.2953	-0.4393 to -0.1514	Yes
15610 14:**1 X** vs. 24:**50 X**	-0.2170	-0.3610 to -0.07304	Yes
15611 14:**1 X** vs. 25:**0 X**	-0.9140	-1.075 to -0.7530	Yes
15612 14:**1 X** vs. 25:**1 X**	-1.104	-1.264 to -0.9425	Yes
15613 14:**1 X** vs. 25:**10 X**	-0.4650	-0.6686 to -0.2614	Yes
15614 14:**1 X** vs. 25:**40 X**	-0.2973	-0.4413 to -0.1534	Yes
15615 14:**1 X** vs. 25:**50 X**	-0.2717	-0.4156 to -0.1277	Yes

2way ANOVA<br>Multiple comparisons	A<br>Data Set-A<br>Y	B<br>Data Set-B<br>Y	C<br>Data Set-C<br>Y
15616 14:1 X vs. 26:0 X	-1.127	-1.287 to -0.9655	Yes
15617 14:1 X vs. 26:1 X	-1.240	-1.400 to -1.079	Yes
15618 14:1 X vs. 26:10 X	-0.9260	-1.087 to -0.7650	Yes
15619 14:1 X vs. 26:40 X	-0.3717	-0.5156 to -0.2277	Yes
15620 14:1 X vs. 26:50 X	-0.2320	-0.3760 to -0.08804	Yes
15621 14:1 X vs. 27:0 X	-1.314	-1.458 to -1.170	Yes
15622 14:1 X vs. 27:1 X	-1.405	-1.566 to -1.244	Yes
15623 14:1 X vs. 27:10 X	-1.217	-1.378 to -1.056	Yes
15624 14:1 X vs. 27:40 X	-0.3717	-0.5156 to -0.2277	Yes
15625 14:1 X vs. 27:50 X	-0.2313	-0.3753 to -0.08737	Yes
15626 14:1 X vs. 28:0 X	-1.264	-1.424 to -1.103	Yes
15627 14:1 X vs. 28:1 X	-1.304	-1.448 to -1.160	Yes
15628 14:1 X vs. 28:10 X	-1.106	-1.267 to -0.9450	Yes
15629 14:1 X vs. 28:40 X	-0.3840	-0.5280 to -0.2400	Yes
15630 14:1 X vs. 28:50 X	-0.2793	-0.4233 to -0.1354	Yes
15631 14:1 X vs. 29:0 X	-1.745	-1.906 to -1.584	Yes
15632 14:1 X vs. 29:1 X	-1.830	-1.991 to -1.669	Yes
15633 14:1 X vs. 29:10 X	-1.045	-1.205 to -0.8835	Yes
15634 14:1 X vs. 29:40 X	-0.4920	-0.6360 to -0.3480	Yes
15635 14:1 X vs. 29:50 X	-0.2807	-0.4246 to -0.1367	Yes
15636 14:1 X vs. 30:0 X	-1.460	-1.620 to -1.299	Yes
15637 14:1 X vs. 30:1 X	-1.781	-1.942 to -1.620	Yes
15638 14:1 X vs. 30:10 X	-1.119	-1.280 to -0.9580	Yes
15639 14:1 X vs. 30:40 X	-0.4190	-0.5630 to -0.2750	Yes
15640 14:1 X vs. 30:50 X	-0.3330	-0.4770 to -0.1890	Yes
15641 14:1 X vs. 31:0 X	-1.778	-1.938 to -1.617	Yes
15642 14:1 X vs. 31:1 X	-1.768	-1.929 to -1.607	Yes
15643 14:1 X vs. 31:10 X	-1.251	-1.411 to -1.090	Yes
15644 14:1 X vs. 31:40 X	-0.5107	-0.6546 to -0.3667	Yes
15645 14:1 X vs. 31:50 X	-0.3120	-0.4560 to -0.1680	Yes
15646 14:1 X vs. 32:0 X	-2.369	-2.513 to -2.225	Yes
15647 14:1 X vs. 32:1 X	-1.825	-1.986 to -1.664	Yes
15648 14:1 X vs. 32:10 X	-1.681	-1.825 to -1.537	Yes
15649 14:1 X vs. 32:40 X	-0.5070	-0.6510 to -0.3630	Yes
15650 14:1 X vs. 32:50 X	-0.3117	-0.4556 to -0.1677	Yes
15651 14:1 X vs. 33:0 X	-2.373	-2.517 to -2.229	Yes
15652 14:1 X vs. 33:1 X	-2.187	-2.331 to -2.043	Yes
15653 14:1 X vs. 33:10 X	-1.848	-1.992 to -1.704	Yes
15654 14:1 X vs. 33:40 X	-0.5257	-0.6696 to -0.3817	Yes
15655 14:1 X vs. 33:50 X	-0.3267	-0.4706 to -0.1827	Yes
15656 14:1 X vs. 34:0 X	-2.297	-2.441 to -2.153	Yes
15657 14:1 X vs. 34:1 X	-2.305	-2.449 to -2.161	Yes
15658 14:1 X vs. 34:10 X	-1.938	-2.082 to -1.794	Yes
15659 14:1 X vs. 34:40 X	-0.5377	-0.6816 to -0.3937	Yes
15660 14:1 X vs. 34:50 X	-0.4757	-0.6196 to -0.3317	Yes

	2way ANOVA<br>Multiple comparisons	A<br>Data Set-A<br>Y	B<br>Data Set-B<br>Y	C<br>Data Set-C<br>Y
15661	14:1 X vs. 35:0 X	-2.646	-2.790 to -2.502	Yes
15662	14:1 X vs. 35:1 X	-2.412	-2.556 to -2.268	Yes
15663	14:1 X vs. 35:10 X	-1.900	-2.044 to -1.756	Yes
15664	14:1 X vs. 35:40 X	-0.5467	-0.6906 to -0.4027	Yes
15665	14:1 X vs. 35:50 X	-0.4883	-0.6323 to -0.3444	Yes
15666	14:1 X vs. 36:0 X	-2.853	-3.013 to -2.692	Yes
15667	14:1 X vs. 36:1 X	-2.548	-2.692 to -2.404	Yes
15668	14:1 X vs. 36:10 X	-1.905	-2.049 to -1.761	Yes
15669	14:1 X vs. 36:40 X	-0.6020	-0.7460 to -0.4580	Yes
15670	14:1 X vs. 36:50 X	-0.4517	-0.5956 to -0.3077	Yes
15671	14:1 X vs. 37:0 X	-3.236	-3.380 to -3.092	Yes
15672	14:1 X vs. 37:1 X	-2.618	-2.762 to -2.474	Yes
15673	14:1 X vs. 37:10 X	-1.907	-2.051 to -1.763	Yes
15674	14:1 X vs. 37:40 X	-0.6393	-0.7833 to -0.4954	Yes
15675	14:1 X vs. 37:50 X	-0.4603	-0.6043 to -0.3164	Yes
15676	14:1 X vs. 38:0 X	-3.181	-3.325 to -3.037	Yes
15677	14:1 X vs. 38:1 X	-2.737	-2.881 to -2.593	Yes
15678	14:1 X vs. 38:10 X	-2.182	-2.326 to -2.038	Yes
15679	14:1 X vs. 38:40 X	-0.6437	-0.7876 to -0.4997	Yes
15680	14:1 X vs. 38:50 X	-0.4643	-0.6083 to -0.3204	Yes
15681	14:1 X vs. 39:0 X	-3.412	-3.556 to -3.268	Yes
15682	14:1 X vs. 39:1 X	-3.304	-3.448 to -3.160	Yes
15683	14:1 X vs. 39:10 X	-2.327	-2.471 to -2.183	Yes
15684	14:1 X vs. 39:40 X	-0.6497	-0.7936 to -0.5057	Yes
15685	14:1 X vs. 39:50 X	-0.4953	-0.6393 to -0.3514	Yes
15686	14:1 X vs. 40:0 X	-3.453	-3.597 to -3.309	Yes
15687	14:1 X vs. 40:1 X	-3.379	-3.523 to -3.235	Yes
15688	14:1 X vs. 40:10 X	-2.584	-2.728 to -2.440	Yes
15689	14:1 X vs. 40:40 X	-0.6543	-0.7983 to -0.5104	Yes
15690	14:1 X vs. 40:50 X	-0.5017	-0.6456 to -0.3577	Yes
15691	14:1 X vs. 41:0 X	-3.494	-3.638 to -3.350	Yes
15692	14:1 X vs. 41:1 X	-3.493	-3.637 to -3.349	Yes
15693	14:1 X vs. 41:10 X	-2.637	-2.781 to -2.493	Yes
15694	14:1 X vs. 41:40 X	-0.6017	-0.7456 to -0.4577	Yes
15695	14:1 X vs. 41:50 X	-0.5143	-0.6583 to -0.3704	Yes
15696	14:1 X vs. 42:0 X	-3.636	-3.780 to -3.492	Yes
15697	14:1 X vs. 42:1 X	-3.655	-3.799 to -3.511	Yes
15698	14:1 X vs. 42:10 X	-2.638	-2.782 to -2.494	Yes
15699	14:1 X vs. 42:40 X	-0.6143	-0.7583 to -0.4704	Yes
15700	14:1 X vs. 42:50 X	-0.5177	-0.6616 to -0.3737	Yes
15701	14:1 X vs. 43:0 X	-3.726	-3.870 to -3.582	Yes
15702	14:1 X vs. 43:1 X	-3.640	-3.784 to -3.496	Yes
15703	14:1 X vs. 43:10 X	-3.356	-3.500 to -3.212	Yes
15704	14:1 X vs. 43:40 X	-0.6127	-0.7566 to -0.4687	Yes
15705	14:1 X vs. 43:50 X	-0.5180	-0.6620 to -0.3740	Yes

	2way ANOVA Multiple comparisons	A Data Set-A Y	B Data Set-B Y	C Data Set-C Y
15706	14:**1 X** vs. 44:**0 X**	-3.750	-3.894 to -3.606	Yes
15707	14:**1 X** vs. 44:**1 X**	-3.639	-3.783 to -3.495	Yes
15708	14:**1 X** vs. 44:**10 X**	-3.419	-3.563 to -3.275	Yes
15709	14:**1 X** vs. 44:**40 X**	-0.6147	-0.7586 to -0.4707	Yes
15710	14:**1 X** vs. 44:**50 X**	-0.4503	-0.5943 to -0.3064	Yes
15711	14:**1 X** vs. 45:**0 X**	-3.614	-3.758 to -3.470	Yes
15712	14:**1 X** vs. 45:**1 X**	-3.416	-3.560 to -3.272	Yes
15713	14:**1 X** vs. 45:**10 X**	-3.299	-3.443 to -3.155	Yes
15714	14:**1 X** vs. 45:**40 X**	-0.6160	-0.7600 to -0.4720	Yes
15715	14:**1 X** vs. 45:**50 X**	-0.4473	-0.5913 to -0.3034	Yes
15716	14:**1 X** vs. 46:**0 X**	-3.419	-3.563 to -3.275	Yes
15717	14:**1 X** vs. 46:**1 X**	-3.386	-3.530 to -3.242	Yes
15718	14:**1 X** vs. 46:**10 X**	-3.294	-3.438 to -3.150	Yes
15719	14:**1 X** vs. 46:**40 X**	-0.6297	-0.7736 to -0.4857	Yes
15720	14:**1 X** vs. 46:**50 X**	-0.4403	-0.5843 to -0.2964	Yes
15721	14:**1 X** vs. 47:**0 X**	-3.394	-3.538 to -3.250	Yes
15722	14:**1 X** vs. 47:**1 X**	-3.298	-3.442 to -3.154	Yes
15723	14:**1 X** vs. 47:**10 X**	-3.280	-3.424 to -3.136	Yes
15724	14:**1 X** vs. 47:**40 X**	-0.6397	-0.7836 to -0.4957	Yes
15725	14:**1 X** vs. 47:**50 X**	-0.3983	-0.5423 to -0.2544	Yes
15726	14:**1 X** vs. 48:**0 X**	-3.382	-3.542 to -3.221	Yes
15727	14:**1 X** vs. 48:**1 X**	-3.285	-3.429 to -3.141	Yes
15728	14:**1 X** vs. 48:**10 X**	-3.280	-3.424 to -3.136	Yes
15729	14:**1 X** vs. 48:**40 X**	-0.6460	-0.7900 to -0.5020	Yes
15730	14:**1 X** vs. 48:**50 X**	-0.4057	-0.5496 to -0.2617	Yes
15731	14:**1 X** vs. 49:**0 X**	-3.382	-3.526 to -3.238	Yes
15732	14:**1 X** vs. 49:**1 X**	-3.285	-3.429 to -3.141	Yes
15733	14:**1 X** vs. 49:**10 X**	-3.280	-3.424 to -3.136	Yes
15734	14:**1 X** vs. 49:**40 X**	-0.6503	-0.7943 to -0.5064	Yes
15735	14:**1 X** vs. 49:**50 X**	-0.4050	-0.5490 to -0.2610	Yes
15736	14:**1 X** vs. 50:**0 X**	-3.382	-3.526 to -3.238	Yes
15737	14:**1 X** vs. 50:**1 X**	-3.285	-3.429 to -3.141	Yes
15738	14:**1 X** vs. 50:**10 X**	-3.280	-3.424 to -3.136	Yes
15739	14:**1 X** vs. 50:**40 X**	-0.6580	-0.8020 to -0.5140	Yes
15740	14:**1 X** vs. 50:**50 X**	-0.4047	-0.5486 to -0.2607	Yes
15741	14:**10 X** vs. 14:**40 X**	0.003333	-0.1406 to 0.1473	No
15742	14:**10 X** vs. 14:**50 X**	-0.0006667	-0.1446 to 0.1433	No
15743	14:**10 X** vs. 15:**0 X**	-0.006667	-0.1506 to 0.1373	No
15744	14:**10 X** vs. 15:**1 X**	0.01333	-0.1306 to 0.1573	No
15745	14:**10 X** vs. 15:**10 X**	-0.0140	-0.1580 to 0.1300	No
15746	14:**10 X** vs. 15:**40 X**	0.003667	-0.1403 to 0.1476	No
15747	14:**10 X** vs. 15:**50 X**	-0.0140	-0.1580 to 0.1300	No
15748	14:**10 X** vs. 16:**0 X**	-0.0990	-0.2430 to 0.04496	No
15749	14:**10 X** vs. 16:**1 X**	0.0100	-0.1340 to 0.1540	No
15750	14:**10 X** vs. 16:**10 X**	-0.01567	-0.1596 to 0.1283	No

	2way ANOVA Multiple comparisons	A Data Set-A Y	B Data Set-B Y	C Data Set-C Y
15751	14:**10 X** vs. 16:**40 X**	-0.0350	-0.1790 to 0.1090	No
15752	14:**10 X** vs. 16:**50 X**	-0.07733	-0.2213 to 0.06663	No
15753	14:**10 X** vs. 17:**0 X**	-0.1517	-0.2956 to -0.007707	Yes
15754	14:**10 X** vs. 17:**1 X**	-0.0660	-0.2100 to 0.07796	No
15755	14:**10 X** vs. 17:**10 X**	-0.1227	-0.2666 to 0.02129	No
15756	14:**10 X** vs. 17:**40 X**	-0.0460	-0.1900 to 0.09796	No
15757	14:**10 X** vs. 17:**50 X**	-0.09133	-0.2353 to 0.05263	No
15758	14:**10 X** vs. 18:**0 X**	-0.2003	-0.3443 to -0.05637	Yes
15759	14:**10 X** vs. 18:**1 X**	-0.1087	-0.2526 to 0.03529	No
15760	14:**10 X** vs. 18:**10 X**	-0.2010	-0.3450 to -0.05704	Yes
15761	14:**10 X** vs. 18:**40 X**	-0.05033	-0.1943 to 0.09363	No
15762	14:**10 X** vs. 18:**50 X**	-0.09533	-0.2393 to 0.04863	No
15763	14:**10 X** vs. 19:**0 X**	-0.2353	-0.3793 to -0.09137	Yes
15764	14:**10 X** vs. 19:**1 X**	-0.2233	-0.3673 to -0.07937	Yes
15765	14:**10 X** vs. 19:**10 X**	-0.2210	-0.3650 to -0.07704	Yes
15766	14:**10 X** vs. 19:**40 X**	-0.0790	-0.2230 to 0.06496	No
15767	14:**10 X** vs. 19:**50 X**	-0.0900	-0.2340 to 0.05396	No
15768	14:**10 X** vs. 20:**0 X**	-0.3150	-0.4590 to -0.1710	Yes
15769	14:**10 X** vs. 20:**1 X**	-0.1343	-0.2783 to 0.009626	No
15770	14:**10 X** vs. 20:**10 X**	-0.2233	-0.3673 to -0.07937	Yes
15771	14:**10 X** vs. 20:**40 X**	-0.09067	-0.2346 to 0.05329	No
15772	14:**10 X** vs. 20:**50 X**	-0.1347	-0.2786 to 0.009293	No
15773	14:**10 X** vs. 21:**0 X**	-0.3383	-0.4823 to -0.1944	Yes
15774	14:**10 X** vs. 21:**1 X**	-0.2913	-0.4353 to -0.1474	Yes
15775	14:**10 X** vs. 21:**10 X**	-0.2883	-0.4323 to -0.1444	Yes
15776	14:**10 X** vs. 21:**40 X**	-0.1637	-0.3076 to -0.01971	Yes
15777	14:**10 X** vs. 21:**50 X**	-0.2540	-0.3980 to -0.1100	Yes
15778	14:**10 X** vs. 22:**0 X**	-0.3800	-0.5240 to -0.2360	Yes
15779	14:**10 X** vs. 22:**1 X**	-0.3747	-0.5186 to -0.2307	Yes
15780	14:**10 X** vs. 22:**10 X**	-0.3700	-0.5140 to -0.2260	Yes
15781	14:**10 X** vs. 22:**40 X**	-0.2703	-0.4143 to -0.1264	Yes
15782	14:**10 X** vs. 22:**50 X**	-0.3330	-0.4770 to -0.1890	Yes
15783	14:**10 X** vs. 23:**0 X**	-0.4453	-0.5893 to -0.3014	Yes
15784	14:**10 X** vs. 23:**1 X**	-0.6350	-0.7960 to -0.4740	Yes
15785	14:**10 X** vs. 23:**10 X**	-0.3847	-0.5286 to -0.2407	Yes
15786	14:**10 X** vs. 23:**40 X**	-0.2650	-0.4260 to -0.1040	Yes
15787	14:**10 X** vs. 23:**50 X**	-0.2990	-0.4430 to -0.1550	Yes
15788	14:**10 X** vs. 24:**0 X**	-0.5627	-0.7066 to -0.4187	Yes
15789	14:**10 X** vs. 24:**1 X**	-0.7190	-0.8800 to -0.5580	Yes
15790	14:**10 X** vs. 24:**10 X**	-0.5465	-0.7075 to -0.3855	Yes
15791	14:**10 X** vs. 24:**40 X**	-0.2813	-0.4253 to -0.1374	Yes
15792	14:**10 X** vs. 24:**50 X**	-0.2030	-0.3470 to -0.05904	Yes
15793	14:**10 X** vs. 25:**0 X**	-0.9000	-1.061 to -0.7390	Yes
15794	14:**10 X** vs. 25:**1 X**	-1.090	-1.250 to -0.9285	Yes
15795	14:**10 X** vs. 25:**10 X**	-0.4510	-0.6546 to -0.2474	Yes

	2way ANOVA<br>Multiple comparisons	A<br>Data Set-A<br>Y	B<br>Data Set-B<br>Y	C<br>Data Set-C<br>Y
15796	14:10 X vs. 25:40 X	-0.2833	-0.4273 to -0.1394	Yes
15797	14:10 X vs. 25:50 X	-0.2577	-0.4016 to -0.1137	Yes
15798	14:10 X vs. 26:0 X	-1.113	-1.273 to -0.9515	Yes
15799	14:10 X vs. 26:1 X	-1.226	-1.386 to -1.065	Yes
15800	14:10 X vs. 26:10 X	-0.9120	-1.073 to -0.7510	Yes
15801	14:10 X vs. 26:40 X	-0.3577	-0.5016 to -0.2137	Yes
15802	14:10 X vs. 26:50 X	-0.2180	-0.3620 to -0.07404	Yes
15803	14:10 X vs. 27:0 X	-1.300	-1.444 to -1.156	Yes
15804	14:10 X vs. 27:1 X	-1.391	-1.552 to -1.230	Yes
15805	14:10 X vs. 27:10 X	-1.203	-1.364 to -1.042	Yes
15806	14:10 X vs. 27:40 X	-0.3577	-0.5016 to -0.2137	Yes
15807	14:10 X vs. 27:50 X	-0.2173	-0.3613 to -0.07337	Yes
15808	14:10 X vs. 28:0 X	-1.250	-1.410 to -1.089	Yes
15809	14:10 X vs. 28:1 X	-1.290	-1.434 to -1.146	Yes
15810	14:10 X vs. 28:10 X	-1.092	-1.253 to -0.9310	Yes
15811	14:10 X vs. 28:40 X	-0.3700	-0.5140 to -0.2260	Yes
15812	14:10 X vs. 28:50 X	-0.2653	-0.4093 to -0.1214	Yes
15813	14:10 X vs. 29:0 X	-1.731	-1.892 to -1.570	Yes
15814	14:10 X vs. 29:1 X	-1.816	-1.977 to -1.655	Yes
15815	14:10 X vs. 29:10 X	-1.031	-1.191 to -0.8695	Yes
15816	14:10 X vs. 29:40 X	-0.4780	-0.6220 to -0.3340	Yes
15817	14:10 X vs. 29:50 X	-0.2667	-0.4106 to -0.1227	Yes
15818	14:10 X vs. 30:0 X	-1.446	-1.606 to -1.285	Yes
15819	14:10 X vs. 30:1 X	-1.767	-1.928 to -1.606	Yes
15820	14:10 X vs. 30:10 X	-1.105	-1.266 to -0.9440	Yes
15821	14:10 X vs. 30:40 X	-0.4050	-0.5490 to -0.2610	Yes
15822	14:10 X vs. 30:50 X	-0.3190	-0.4630 to -0.1750	Yes
15823	14:10 X vs. 31:0 X	-1.764	-1.924 to -1.603	Yes
15824	14:10 X vs. 31:1 X	-1.754	-1.915 to -1.593	Yes
15825	14:10 X vs. 31:10 X	-1.237	-1.397 to -1.076	Yes
15826	14:10 X vs. 31:40 X	-0.4967	-0.6406 to -0.3527	Yes
15827	14:10 X vs. 31:50 X	-0.2980	-0.4420 to -0.1540	Yes
15828	14:10 X vs. 32:0 X	-2.355	-2.499 to -2.211	Yes
15829	14:10 X vs. 32:1 X	-1.811	-1.972 to -1.650	Yes
15830	14:10 X vs. 32:10 X	-1.667	-1.811 to -1.523	Yes
15831	14:10 X vs. 32:40 X	-0.4930	-0.6370 to -0.3490	Yes
15832	14:10 X vs. 32:50 X	-0.2977	-0.4416 to -0.1537	Yes
15833	14:10 X vs. 33:0 X	-2.359	-2.503 to -2.215	Yes
15834	14:10 X vs. 33:1 X	-2.173	-2.317 to -2.029	Yes
15835	14:10 X vs. 33:10 X	-1.834	-1.978 to -1.690	Yes
15836	14:10 X vs. 33:40 X	-0.5117	-0.6556 to -0.3677	Yes
15837	14:10 X vs. 33:50 X	-0.3127	-0.4566 to -0.1687	Yes
15838	14:10 X vs. 34:0 X	-2.283	-2.427 to -2.139	Yes
15839	14:10 X vs. 34:1 X	-2.291	-2.435 to -2.147	Yes
15840	14:10 X vs. 34:10 X	-1.924	-2.068 to -1.780	Yes

	2way ANOVA<br>Multiple comparisons	A<br>Data Set-A<br>Y	B<br>Data Set-B<br>Y	C<br>Data Set-C<br>Y
15841	14:10 X vs. 34:40 X	-0.5237	-0.6676 to -0.3797	Yes
15842	14:10 X vs. 34:50 X	-0.4617	-0.6056 to -0.3177	Yes
15843	14:10 X vs. 35:0 X	-2.632	-2.776 to -2.488	Yes
15844	14:10 X vs. 35:1 X	-2.398	-2.542 to -2.254	Yes
15845	14:10 X vs. 35:10 X	-1.886	-2.030 to -1.742	Yes
15846	14:10 X vs. 35:40 X	-0.5327	-0.6766 to -0.3887	Yes
15847	14:10 X vs. 35:50 X	-0.4743	-0.6183 to -0.3304	Yes
15848	14:10 X vs. 36:0 X	-2.839	-2.999 to -2.678	Yes
15849	14:10 X vs. 36:1 X	-2.534	-2.678 to -2.390	Yes
15850	14:10 X vs. 36:10 X	-1.891	-2.035 to -1.747	Yes
15851	14:10 X vs. 36:40 X	-0.5880	-0.7320 to -0.4440	Yes
15852	14:10 X vs. 36:50 X	-0.4377	-0.5816 to -0.2937	Yes
15853	14:10 X vs. 37:0 X	-3.222	-3.366 to -3.078	Yes
15854	14:10 X vs. 37:1 X	-2.604	-2.748 to -2.460	Yes
15855	14:10 X vs. 37:10 X	-1.893	-2.037 to -1.749	Yes
15856	14:10 X vs. 37:40 X	-0.6253	-0.7693 to -0.4814	Yes
15857	14:10 X vs. 37:50 X	-0.4463	-0.5903 to -0.3024	Yes
15858	14:10 X vs. 38:0 X	-3.167	-3.311 to -3.023	Yes
15859	14:10 X vs. 38:1 X	-2.723	-2.867 to -2.579	Yes
15860	14:10 X vs. 38:10 X	-2.168	-2.312 to -2.024	Yes
15861	14:10 X vs. 38:40 X	-0.6297	-0.7736 to -0.4857	Yes
15862	14:10 X vs. 38:50 X	-0.4503	-0.5943 to -0.3064	Yes
15863	14:10 X vs. 39:0 X	-3.398	-3.542 to -3.254	Yes
15864	14:10 X vs. 39:1 X	-3.290	-3.434 to -3.146	Yes
15865	14:10 X vs. 39:10 X	-2.313	-2.457 to -2.169	Yes
15866	14:10 X vs. 39:40 X	-0.6357	-0.7796 to -0.4917	Yes
15867	14:10 X vs. 39:50 X	-0.4813	-0.6253 to -0.3374	Yes
15868	14:10 X vs. 40:0 X	-3.439	-3.583 to -3.295	Yes
15869	14:10 X vs. 40:1 X	-3.365	-3.509 to -3.221	Yes
15870	14:10 X vs. 40:10 X	-2.570	-2.714 to -2.426	Yes
15871	14:10 X vs. 40:40 X	-0.6403	-0.7843 to -0.4964	Yes
15872	14:10 X vs. 40:50 X	-0.4877	-0.6316 to -0.3437	Yes
15873	14:10 X vs. 41:0 X	-3.480	-3.624 to -3.336	Yes
15874	14:10 X vs. 41:1 X	-3.479	-3.623 to -3.335	Yes
15875	14:10 X vs. 41:10 X	-2.623	-2.767 to -2.479	Yes
15876	14:10 X vs. 41:40 X	-0.5877	-0.7316 to -0.4437	Yes
15877	14:10 X vs. 41:50 X	-0.5003	-0.6443 to -0.3564	Yes
15878	14:10 X vs. 42:0 X	-3.622	-3.766 to -3.478	Yes
15879	14:10 X vs. 42:1 X	-3.641	-3.785 to -3.497	Yes
15880	14:10 X vs. 42:10 X	-2.624	-2.768 to -2.480	Yes
15881	14:10 X vs. 42:40 X	-0.6003	-0.7443 to -0.4564	Yes
15882	14:10 X vs. 42:50 X	-0.5037	-0.6476 to -0.3597	Yes
15883	14:10 X vs. 43:0 X	-3.712	-3.856 to -3.568	Yes
15884	14:10 X vs. 43:1 X	-3.626	-3.770 to -3.482	Yes
15885	14:10 X vs. 43:10 X	-3.342	-3.486 to -3.198	Yes

2way ANOVA<br>Multiple comparisons		A<br>Data Set-A<br>Y	B<br>Data Set-B<br>Y	C<br>Data Set-C<br>Y
15886	14:10 X vs. 43:40 X	-0.5987	-0.7426 to -0.4547	Yes
15887	14:10 X vs. 43:50 X	-0.5040	-0.6480 to -0.3600	Yes
15888	14:10 X vs. 44:0 X	-3.736	-3.880 to -3.592	Yes
15889	14:10 X vs. 44:1 X	-3.625	-3.769 to -3.481	Yes
15890	14:10 X vs. 44:10 X	-3.405	-3.549 to -3.261	Yes
15891	14:10 X vs. 44:40 X	-0.6007	-0.7446 to -0.4567	Yes
15892	14:10 X vs. 44:50 X	-0.4363	-0.5803 to -0.2924	Yes
15893	14:10 X vs. 45:0 X	-3.600	-3.744 to -3.456	Yes
15894	14:10 X vs. 45:1 X	-3.402	-3.546 to -3.258	Yes
15895	14:10 X vs. 45:10 X	-3.285	-3.429 to -3.141	Yes
15896	14:10 X vs. 45:40 X	-0.6020	-0.7460 to -0.4580	Yes
15897	14:10 X vs. 45:50 X	-0.4333	-0.5773 to -0.2894	Yes
15898	14:10 X vs. 46:0 X	-3.405	-3.549 to -3.261	Yes
15899	14:10 X vs. 46:1 X	-3.372	-3.516 to -3.228	Yes
15900	14:10 X vs. 46:10 X	-3.280	-3.424 to -3.136	Yes
15901	14:10 X vs. 46:40 X	-0.6157	-0.7596 to -0.4717	Yes
15902	14:10 X vs. 46:50 X	-0.4263	-0.5703 to -0.2824	Yes
15903	14:10 X vs. 47:0 X	-3.380	-3.524 to -3.236	Yes
15904	14:10 X vs. 47:1 X	-3.284	-3.428 to -3.140	Yes
15905	14:10 X vs. 47:10 X	-3.266	-3.410 to -3.122	Yes
15906	14:10 X vs. 47:40 X	-0.6257	-0.7696 to -0.4817	Yes
15907	14:10 X vs. 47:50 X	-0.3843	-0.5283 to -0.2404	Yes
15908	14:10 X vs. 48:0 X	-3.368	-3.528 to -3.207	Yes
15909	14:10 X vs. 48:1 X	-3.271	-3.415 to -3.127	Yes
15910	14:10 X vs. 48:10 X	-3.266	-3.410 to -3.122	Yes
15911	14:10 X vs. 48:40 X	-0.6320	-0.7760 to -0.4880	Yes
15912	14:10 X vs. 48:50 X	-0.3917	-0.5356 to -0.2477	Yes
15913	14:10 X vs. 49:0 X	-3.368	-3.512 to -3.224	Yes
15914	14:10 X vs. 49:1 X	-3.271	-3.415 to -3.127	Yes
15915	14:10 X vs. 49:10 X	-3.266	-3.410 to -3.122	Yes
15916	14:10 X vs. 49:40 X	-0.6363	-0.7803 to -0.4924	Yes
15917	14:10 X vs. 49:50 X	-0.3910	-0.5350 to -0.2470	Yes
15918	14:10 X vs. 50:0 X	-3.368	-3.512 to -3.224	Yes
15919	14:10 X vs. 50:1 X	-3.271	-3.415 to -3.127	Yes
15920	14:10 X vs. 50:10 X	-3.266	-3.410 to -3.122	Yes
15921	14:10 X vs. 50:40 X	-0.6440	-0.7880 to -0.5000	Yes
15922	14:10 X vs. 50:50 X	-0.3907	-0.5346 to -0.2467	Yes
15923	14:40 X vs. 14:50 X	-0.004000	-0.1480 to 0.1400	No
15924	14:40 X vs. 15:0 X	-0.0100	-0.1540 to 0.1340	No
15925	14:40 X vs. 15:1 X	0.0100	-0.1340 to 0.1540	No
15926	14:40 X vs. 15:10 X	-0.01733	-0.1613 to 0.1266	No
15927	14:40 X vs. 15:40 X	0.0003333	-0.1436 to 0.1443	No
15928	14:40 X vs. 15:50 X	-0.01733	-0.1613 to 0.1266	No
15929	14:40 X vs. 16:0 X	-0.1023	-0.2463 to 0.04163	No
15930	14:40 X vs. 16:1 X	0.006667	-0.1373 to 0.1506	No

	2way ANOVA<br>Multiple comparisons	A<br>Data Set-A<br>Y	B<br>Data Set-B<br>Y	C<br>Data Set-C<br>Y
15931	14:40 X vs. 16:10 X	-0.0190	-0.1630 to 0.1250	No
15932	14:40 X vs. 16:40 X	-0.03833	-0.1823 to 0.1056	No
15933	14:40 X vs. 16:50 X	-0.08067	-0.2246 to 0.06329	No
15934	14:40 X vs. 17:0 X	-0.1550	-0.2990 to -0.01104	Yes
15935	14:40 X vs. 17:1 X	-0.06933	-0.2133 to 0.07463	No
15936	14:40 X vs. 17:10 X	-0.1260	-0.2700 to 0.01796	No
15937	14:40 X vs. 17:40 X	-0.04933	-0.1933 to 0.09463	No
15938	14:40 X vs. 17:50 X	-0.09467	-0.2386 to 0.04929	No
15939	14:40 X vs. 18:0 X	-0.2037	-0.3476 to -0.05971	Yes
15940	14:40 X vs. 18:1 X	-0.1120	-0.2560 to 0.03196	No
15941	14:40 X vs. 18:10 X	-0.2043	-0.3483 to -0.06037	Yes
15942	14:40 X vs. 18:40 X	-0.05367	-0.1976 to 0.09029	No
15943	14:40 X vs. 18:50 X	-0.09867	-0.2426 to 0.04529	No
15944	14:40 X vs. 19:0 X	-0.2387	-0.3826 to -0.09471	Yes
15945	14:40 X vs. 19:1 X	-0.2267	-0.3706 to -0.08271	Yes
15946	14:40 X vs. 19:10 X	-0.2243	-0.3683 to -0.08037	Yes
15947	14:40 X vs. 19:40 X	-0.08233	-0.2263 to 0.06163	No
15948	14:40 X vs. 19:50 X	-0.09333	-0.2373 to 0.05063	No
15949	14:40 X vs. 20:0 X	-0.3183	-0.4623 to -0.1744	Yes
15950	14:40 X vs. 20:1 X	-0.1377	-0.2816 to 0.006293	No
15951	14:40 X vs. 20:10 X	-0.2267	-0.3706 to -0.08271	Yes
15952	14:40 X vs. 20:40 X	-0.0940	-0.2380 to 0.04996	No
15953	14:40 X vs. 20:50 X	-0.1380	-0.2820 to 0.005960	No
15954	14:40 X vs. 21:0 X	-0.3417	-0.4856 to -0.1977	Yes
15955	14:40 X vs. 21:1 X	-0.2947	-0.4386 to -0.1507	Yes
15956	14:40 X vs. 21:10 X	-0.2917	-0.4356 to -0.1477	Yes
15957	14:40 X vs. 21:40 X	-0.1670	-0.3110 to -0.02304	Yes
15958	14:40 X vs. 21:50 X	-0.2573	-0.4013 to -0.1134	Yes
15959	14:40 X vs. 22:0 X	-0.3833	-0.5273 to -0.2394	Yes
15960	14:40 X vs. 22:1 X	-0.3780	-0.5220 to -0.2340	Yes
15961	14:40 X vs. 22:10 X	-0.3733	-0.5173 to -0.2294	Yes
15962	14:40 X vs. 22:40 X	-0.2737	-0.4176 to -0.1297	Yes
15963	14:40 X vs. 22:50 X	-0.3363	-0.4803 to -0.1924	Yes
15964	14:40 X vs. 23:0 X	-0.4487	-0.5926 to -0.3047	Yes
15965	14:40 X vs. 23:1 X	-0.6383	-0.7993 to -0.4774	Yes
15966	14:40 X vs. 23:10 X	-0.3880	-0.5320 to -0.2440	Yes
15967	14:40 X vs. 23:40 X	-0.2683	-0.4293 to -0.1074	Yes
15968	14:40 X vs. 23:50 X	-0.3023	-0.4463 to -0.1584	Yes
15969	14:40 X vs. 24:0 X	-0.5660	-0.7100 to -0.4220	Yes
15970	14:40 X vs. 24:1 X	-0.7223	-0.8833 to -0.5614	Yes
15971	14:40 X vs. 24:10 X	-0.5498	-0.7108 to -0.3889	Yes
15972	14:40 X vs. 24:40 X	-0.2847	-0.4286 to -0.1407	Yes
15973	14:40 X vs. 24:50 X	-0.2063	-0.3503 to -0.06237	Yes
15974	14:40 X vs. 25:0 X	-0.9033	-1.064 to -0.7424	Yes
15975	14:40 X vs. 25:1 X	-1.093	-1.254 to -0.9319	Yes

	2way ANOVA<br>Multiple comparisons	A<br>Data Set-A<br>Y	B<br>Data Set-B<br>Y	C<br>Data Set-C<br>Y
15976	14:**40 X** vs. 25:**10 X**	-0.4543	-0.6579 to -0.2507	Yes
15977	14:**40 X** vs. 25:**40 X**	-0.2867	-0.4306 to -0.1427	Yes
15978	14:**40 X** vs. 25:**50 X**	-0.2610	-0.4050 to -0.1170	Yes
15979	14:**40 X** vs. 26:**0 X**	-1.116	-1.277 to -0.9549	Yes
15980	14:**40 X** vs. 26:**1 X**	-1.229	-1.390 to -1.068	Yes
15981	14:**40 X** vs. 26:**10 X**	-0.9153	-1.076 to -0.7544	Yes
15982	14:**40 X** vs. 26:**40 X**	-0.3610	-0.5050 to -0.2170	Yes
15983	14:**40 X** vs. 26:**50 X**	-0.2213	-0.3653 to -0.07737	Yes
15984	14:**40 X** vs. 27:**0 X**	-1.304	-1.448 to -1.160	Yes
15985	14:**40 X** vs. 27:**1 X**	-1.394	-1.555 to -1.233	Yes
15986	14:**40 X** vs. 27:**10 X**	-1.206	-1.367 to -1.045	Yes
15987	14:**40 X** vs. 27:**40 X**	-0.3610	-0.5050 to -0.2170	Yes
15988	14:**40 X** vs. 27:**50 X**	-0.2207	-0.3646 to -0.07671	Yes
15989	14:**40 X** vs. 28:**0 X**	-1.253	-1.414 to -1.092	Yes
15990	14:**40 X** vs. 28:**1 X**	-1.294	-1.438 to -1.150	Yes
15991	14:**40 X** vs. 28:**10 X**	-1.095	-1.256 to -0.9344	Yes
15992	14:**40 X** vs. 28:**40 X**	-0.3733	-0.5173 to -0.2294	Yes
15993	14:**40 X** vs. 28:**50 X**	-0.2687	-0.4126 to -0.1247	Yes
15994	14:**40 X** vs. 29:**0 X**	-1.734	-1.895 to -1.573	Yes
15995	14:**40 X** vs. 29:**1 X**	-1.819	-1.980 to -1.658	Yes
15996	14:**40 X** vs. 29:**10 X**	-1.034	-1.195 to -0.8729	Yes
15997	14:**40 X** vs. 29:**40 X**	-0.4813	-0.6253 to -0.3374	Yes
15998	14:**40 X** vs. 29:**50 X**	-0.2700	-0.4140 to -0.1260	Yes
15999	14:**40 X** vs. 30:**0 X**	-1.449	-1.610 to -1.288	Yes
16000	14:**40 X** vs. 30:**1 X**	-1.770	-1.931 to -1.609	Yes
16001	14:**40 X** vs. 30:**10 X**	-1.108	-1.269 to -0.9474	Yes
16002	14:**40 X** vs. 30:**40 X**	-0.4083	-0.5523 to -0.2644	Yes
16003	14:**40 X** vs. 30:**50 X**	-0.3223	-0.4663 to -0.1784	Yes
16004	14:**40 X** vs. 31:**0 X**	-1.767	-1.928 to -1.606	Yes
16005	14:**40 X** vs. 31:**1 X**	-1.757	-1.918 to -1.596	Yes
16006	14:**40 X** vs. 31:**10 X**	-1.240	-1.401 to -1.079	Yes
16007	14:**40 X** vs. 31:**40 X**	-0.5000	-0.6440 to -0.3560	Yes
16008	14:**40 X** vs. 31:**50 X**	-0.3013	-0.4453 to -0.1574	Yes
16009	14:**40 X** vs. 32:**0 X**	-2.358	-2.502 to -2.214	Yes
16010	14:**40 X** vs. 32:**1 X**	-1.814	-1.975 to -1.653	Yes
16011	14:**40 X** vs. 32:**10 X**	-1.670	-1.814 to -1.526	Yes
16012	14:**40 X** vs. 32:**40 X**	-0.4963	-0.6403 to -0.3524	Yes
16013	14:**40 X** vs. 32:**50 X**	-0.3010	-0.4450 to -0.1570	Yes
16014	14:**40 X** vs. 33:**0 X**	-2.362	-2.506 to -2.218	Yes
16015	14:**40 X** vs. 33:**1 X**	-2.177	-2.321 to -2.033	Yes
16016	14:**40 X** vs. 33:**10 X**	-1.837	-1.981 to -1.693	Yes
16017	14:**40 X** vs. 33:**40 X**	-0.5150	-0.6590 to -0.3710	Yes
16018	14:**40 X** vs. 33:**50 X**	-0.3160	-0.4600 to -0.1720	Yes
16019	14:**40 X** vs. 34:**0 X**	-2.287	-2.431 to -2.143	Yes
16020	14:**40 X** vs. 34:**1 X**	-2.294	-2.438 to -2.150	Yes

2way ANOVA<br>Multiple comparisons	A<br>Data Set-A<br>Y	B<br>Data Set-B<br>Y	C<br>Data Set-C<br>Y
16021 14:40 X vs. 34:10 X	-1.927	-2.071 to -1.783	Yes
16022 14:40 X vs. 34:40 X	-0.5270	-0.6710 to -0.3830	Yes
16023 14:40 X vs. 34:50 X	-0.4650	-0.6090 to -0.3210	Yes
16024 14:40 X vs. 35:0 X	-2.635	-2.779 to -2.491	Yes
16025 14:40 X vs. 35:1 X	-2.401	-2.545 to -2.257	Yes
16026 14:40 X vs. 35:10 X	-1.889	-2.033 to -1.745	Yes
16027 14:40 X vs. 35:40 X	-0.5360	-0.6800 to -0.3920	Yes
16028 14:40 X vs. 35:50 X	-0.4777	-0.6216 to -0.3337	Yes
16029 14:40 X vs. 36:0 X	-2.842	-3.003 to -2.681	Yes
16030 14:40 X vs. 36:1 X	-2.538	-2.682 to -2.394	Yes
16031 14:40 X vs. 36:10 X	-1.895	-2.039 to -1.751	Yes
16032 14:40 X vs. 36:40 X	-0.5913	-0.7353 to -0.4474	Yes
16033 14:40 X vs. 36:50 X	-0.4410	-0.5850 to -0.2970	Yes
16034 14:40 X vs. 37:0 X	-3.226	-3.370 to -3.082	Yes
16035 14:40 X vs. 37:1 X	-2.608	-2.752 to -2.464	Yes
16036 14:40 X vs. 37:10 X	-1.896	-2.040 to -1.752	Yes
16037 14:40 X vs. 37:40 X	-0.6287	-0.7726 to -0.4847	Yes
16038 14:40 X vs. 37:50 X	-0.4497	-0.5936 to -0.3057	Yes
16039 14:40 X vs. 38:0 X	-3.171	-3.315 to -3.027	Yes
16040 14:40 X vs. 38:1 X	-2.726	-2.870 to -2.582	Yes
16041 14:40 X vs. 38:10 X	-2.172	-2.316 to -2.028	Yes
16042 14:40 X vs. 38:40 X	-0.6330	-0.7770 to -0.4890	Yes
16043 14:40 X vs. 38:50 X	-0.4537	-0.5976 to -0.3097	Yes
16044 14:40 X vs. 39:0 X	-3.401	-3.545 to -3.257	Yes
16045 14:40 X vs. 39:1 X	-3.293	-3.437 to -3.149	Yes
16046 14:40 X vs. 39:10 X	-2.317	-2.461 to -2.173	Yes
16047 14:40 X vs. 39:40 X	-0.6390	-0.7830 to -0.4950	Yes
16048 14:40 X vs. 39:50 X	-0.4847	-0.6286 to -0.3407	Yes
16049 14:40 X vs. 40:0 X	-3.442	-3.586 to -3.298	Yes
16050 14:40 X vs. 40:1 X	-3.368	-3.512 to -3.224	Yes
16051 14:40 X vs. 40:10 X	-2.573	-2.717 to -2.429	Yes
16052 14:40 X vs. 40:40 X	-0.6437	-0.7876 to -0.4997	Yes
16053 14:40 X vs. 40:50 X	-0.4910	-0.6350 to -0.3470	Yes
16054 14:40 X vs. 41:0 X	-3.483	-3.627 to -3.339	Yes
16055 14:40 X vs. 41:1 X	-3.482	-3.626 to -3.338	Yes
16056 14:40 X vs. 41:10 X	-2.627	-2.771 to -2.483	Yes
16057 14:40 X vs. 41:40 X	-0.5910	-0.7350 to -0.4470	Yes
16058 14:40 X vs. 41:50 X	-0.5037	-0.6476 to -0.3597	Yes
16059 14:40 X vs. 42:0 X	-3.625	-3.769 to -3.481	Yes
16060 14:40 X vs. 42:1 X	-3.645	-3.789 to -3.501	Yes
16061 14:40 X vs. 42:10 X	-2.627	-2.771 to -2.483	Yes
16062 14:40 X vs. 42:40 X	-0.6037	-0.7476 to -0.4597	Yes
16063 14:40 X vs. 42:50 X	-0.5070	-0.6510 to -0.3630	Yes
16064 14:40 X vs. 43:0 X	-3.715	-3.859 to -3.571	Yes
16065 14:40 X vs. 43:1 X	-3.629	-3.773 to -3.485	Yes

	2way ANOVA<br>Multiple comparisons	A<br>Data Set-A<br>Y	B<br>Data Set-B<br>Y	C<br>Data Set-C<br>Y
16066	14:**40 X** vs. 43:**10 X**	-3.345	-3.489 to -3.201	Yes
16067	14:**40 X** vs. 43:**40 X**	-0.6020	-0.7460 to -0.4580	Yes
16068	14:**40 X** vs. 43:**50 X**	-0.5073	-0.6513 to -0.3634	Yes
16069	14:**40 X** vs. 44:**0 X**	-3.739	-3.883 to -3.595	Yes
16070	14:**40 X** vs. 44:**1 X**	-3.629	-3.773 to -3.485	Yes
16071	14:**40 X** vs. 44:**10 X**	-3.408	-3.552 to -3.264	Yes
16072	14:**40 X** vs. 44:**40 X**	-0.6040	-0.7480 to -0.4600	Yes
16073	14:**40 X** vs. 44:**50 X**	-0.4397	-0.5836 to -0.2957	Yes
16074	14:**40 X** vs. 45:**0 X**	-3.604	-3.748 to -3.460	Yes
16075	14:**40 X** vs. 45:**1 X**	-3.406	-3.550 to -3.262	Yes
16076	14:**40 X** vs. 45:**10 X**	-3.288	-3.432 to -3.144	Yes
16077	14:**40 X** vs. 45:**40 X**	-0.6053	-0.7493 to -0.4614	Yes
16078	14:**40 X** vs. 45:**50 X**	-0.4367	-0.5806 to -0.2927	Yes
16079	14:**40 X** vs. 46:**0 X**	-3.408	-3.552 to -3.264	Yes
16080	14:**40 X** vs. 46:**1 X**	-3.375	-3.519 to -3.231	Yes
16081	14:**40 X** vs. 46:**10 X**	-3.283	-3.427 to -3.139	Yes
16082	14:**40 X** vs. 46:**40 X**	-0.6190	-0.7630 to -0.4750	Yes
16083	14:**40 X** vs. 46:**50 X**	-0.4297	-0.5736 to -0.2857	Yes
16084	14:**40 X** vs. 47:**0 X**	-3.384	-3.528 to -3.240	Yes
16085	14:**40 X** vs. 47:**1 X**	-3.288	-3.432 to -3.144	Yes
16086	14:**40 X** vs. 47:**10 X**	-3.269	-3.413 to -3.125	Yes
16087	14:**40 X** vs. 47:**40 X**	-0.6290	-0.7730 to -0.4850	Yes
16088	14:**40 X** vs. 47:**50 X**	-0.3877	-0.5316 to -0.2437	Yes
16089	14:**40 X** vs. 48:**0 X**	-3.371	-3.532 to -3.210	Yes
16090	14:**40 X** vs. 48:**1 X**	-3.275	-3.419 to -3.131	Yes
16091	14:**40 X** vs. 48:**10 X**	-3.269	-3.413 to -3.125	Yes
16092	14:**40 X** vs. 48:**40 X**	-0.6353	-0.7793 to -0.4914	Yes
16093	14:**40 X** vs. 48:**50 X**	-0.3950	-0.5390 to -0.2510	Yes
16094	14:**40 X** vs. 49:**0 X**	-3.371	-3.515 to -3.227	Yes
16095	14:**40 X** vs. 49:**1 X**	-3.275	-3.419 to -3.131	Yes
16096	14:**40 X** vs. 49:**10 X**	-3.269	-3.413 to -3.125	Yes
16097	14:**40 X** vs. 49:**40 X**	-0.6397	-0.7836 to -0.4957	Yes
16098	14:**40 X** vs. 49:**50 X**	-0.3943	-0.5383 to -0.2504	Yes
16099	14:**40 X** vs. 50:**0 X**	-3.371	-3.515 to -3.227	Yes
16100	14:**40 X** vs. 50:**1 X**	-3.275	-3.419 to -3.131	Yes
16101	14:**40 X** vs. 50:**10 X**	-3.269	-3.413 to -3.125	Yes
16102	14:**40 X** vs. 50:**40 X**	-0.6473	-0.7913 to -0.5034	Yes
16103	14:**40 X** vs. 50:**50 X**	-0.3940	-0.5380 to -0.2500	Yes
16104	14:**50 X** vs. 15:**0 X**	-0.006000	-0.1500 to 0.1380	No
16105	14:**50 X** vs. 15:**1 X**	0.0140	-0.1300 to 0.1580	No
16106	14:**50 X** vs. 15:**10 X**	-0.01333	-0.1573 to 0.1306	No
16107	14:**50 X** vs. 15:**40 X**	0.004333	-0.1396 to 0.1483	No
16108	14:**50 X** vs. 15:**50 X**	-0.01333	-0.1573 to 0.1306	No
16109	14:**50 X** vs. 16:**0 X**	-0.09833	-0.2423 to 0.04563	No
16110	14:**50 X** vs. 16:**1 X**	0.01067	-0.1333 to 0.1546	No

	**2way ANOVA**<br>Multiple comparisons	**A**<br>Data Set-A<br>**Y**	**B**<br>Data Set-B<br>**Y**	**C**<br>Data Set-C<br>**Y**
**16111**	14:**50 X** vs. 16:**10 X**	-0.0150	-0.1590 to 0.1290	No
**16112**	14:**50 X** vs. 16:**40 X**	-0.03433	-0.1783 to 0.1096	No
**16113**	14:**50 X** vs. 16:**50 X**	-0.07667	-0.2206 to 0.06729	No
**16114**	14:**50 X** vs. 17:**0 X**	-0.1510	-0.2950 to -0.007040	Yes
**16115**	14:**50 X** vs. 17:**1 X**	-0.06533	-0.2093 to 0.07863	No
**16116**	14:**50 X** vs. 17:**10 X**	-0.1220	-0.2660 to 0.02196	No
**16117**	14:**50 X** vs. 17:**40 X**	-0.04533	-0.1893 to 0.09863	No
**16118**	14:**50 X** vs. 17:**50 X**	-0.09067	-0.2346 to 0.05329	No
**16119**	14:**50 X** vs. 18:**0 X**	-0.1997	-0.3436 to -0.05571	Yes
**16120**	14:**50 X** vs. 18:**1 X**	-0.1080	-0.2520 to 0.03596	No
**16121**	14:**50 X** vs. 18:**10 X**	-0.2003	-0.3443 to -0.05637	Yes
**16122**	14:**50 X** vs. 18:**40 X**	-0.04967	-0.1936 to 0.09429	No
**16123**	14:**50 X** vs. 18:**50 X**	-0.09467	-0.2386 to 0.04929	No
**16124**	14:**50 X** vs. 19:**0 X**	-0.2347	-0.3786 to -0.09071	Yes
**16125**	14:**50 X** vs. 19:**1 X**	-0.2227	-0.3666 to -0.07871	Yes
**16126**	14:**50 X** vs. 19:**10 X**	-0.2203	-0.3643 to -0.07637	Yes
**16127**	14:**50 X** vs. 19:**40 X**	-0.07833	-0.2223 to 0.06563	No
**16128**	14:**50 X** vs. 19:**50 X**	-0.08933	-0.2333 to 0.05463	No
**16129**	14:**50 X** vs. 20:**0 X**	-0.3143	-0.4583 to -0.1704	Yes
**16130**	14:**50 X** vs. 20:**1 X**	-0.1337	-0.2776 to 0.01029	No
**16131**	14:**50 X** vs. 20:**10 X**	-0.2227	-0.3666 to -0.07871	Yes
**16132**	14:**50 X** vs. 20:**40 X**	-0.0900	-0.2340 to 0.05396	No
**16133**	14:**50 X** vs. 20:**50 X**	-0.1340	-0.2780 to 0.009960	No
**16134**	14:**50 X** vs. 21:**0 X**	-0.3377	-0.4816 to -0.1937	Yes
**16135**	14:**50 X** vs. 21:**1 X**	-0.2907	-0.4346 to -0.1467	Yes
**16136**	14:**50 X** vs. 21:**10 X**	-0.2877	-0.4316 to -0.1437	Yes
**16137**	14:**50 X** vs. 21:**40 X**	-0.1630	-0.3070 to -0.01904	Yes
**16138**	14:**50 X** vs. 21:**50 X**	-0.2533	-0.3973 to -0.1094	Yes
**16139**	14:**50 X** vs. 22:**0 X**	-0.3793	-0.5233 to -0.2354	Yes
**16140**	14:**50 X** vs. 22:**1 X**	-0.3740	-0.5180 to -0.2300	Yes
**16141**	14:**50 X** vs. 22:**10 X**	-0.3693	-0.5133 to -0.2254	Yes
**16142**	14:**50 X** vs. 22:**40 X**	-0.2697	-0.4136 to -0.1257	Yes
**16143**	14:**50 X** vs. 22:**50 X**	-0.3323	-0.4763 to -0.1884	Yes
**16144**	14:**50 X** vs. 23:**0 X**	-0.4447	-0.5886 to -0.3007	Yes
**16145**	14:**50 X** vs. 23:**1 X**	-0.6343	-0.7953 to -0.4734	Yes
**16146**	14:**50 X** vs. 23:**10 X**	-0.3840	-0.5280 to -0.2400	Yes
**16147**	14:**50 X** vs. 23:**40 X**	-0.2643	-0.4253 to -0.1034	Yes
**16148**	14:**50 X** vs. 23:**50 X**	-0.2983	-0.4423 to -0.1544	Yes
**16149**	14:**50 X** vs. 24:**0 X**	-0.5620	-0.7060 to -0.4180	Yes
**16150**	14:**50 X** vs. 24:**1 X**	-0.7183	-0.8793 to -0.5574	Yes
**16151**	14:**50 X** vs. 24:**10 X**	-0.5458	-0.7068 to -0.3849	Yes
**16152**	14:**50 X** vs. 24:**40 X**	-0.2807	-0.4246 to -0.1367	Yes
**16153**	14:**50 X** vs. 24:**50 X**	-0.2023	-0.3463 to -0.05837	Yes
**16154**	14:**50 X** vs. 25:**0 X**	-0.8993	-1.060 to -0.7384	Yes
**16155**	14:**50 X** vs. 25:**1 X**	-1.089	-1.250 to -0.9279	Yes

	2way ANOVA<br>Multiple comparisons	A<br>Data Set-A<br>Y	B<br>Data Set-B<br>Y	C<br>Data Set-C<br>Y
16156	14:50 X vs. 25:10 X	-0.4503	-0.6539 to -0.2467	Yes
16157	14:50 X vs. 25:40 X	-0.2827	-0.4266 to -0.1387	Yes
16158	14:50 X vs. 25:50 X	-0.2570	-0.4010 to -0.1130	Yes
16159	14:50 X vs. 26:0 X	-1.112	-1.273 to -0.9509	Yes
16160	14:50 X vs. 26:1 X	-1.225	-1.386 to -1.064	Yes
16161	14:50 X vs. 26:10 X	-0.9113	-1.072 to -0.7504	Yes
16162	14:50 X vs. 26:40 X	-0.3570	-0.5010 to -0.2130	Yes
16163	14:50 X vs. 26:50 X	-0.2173	-0.3613 to -0.07337	Yes
16164	14:50 X vs. 27:0 X	-1.300	-1.444 to -1.156	Yes
16165	14:50 X vs. 27:1 X	-1.390	-1.551 to -1.229	Yes
16166	14:50 X vs. 27:10 X	-1.202	-1.363 to -1.041	Yes
16167	14:50 X vs. 27:40 X	-0.3570	-0.5010 to -0.2130	Yes
16168	14:50 X vs. 27:50 X	-0.2167	-0.3606 to -0.07271	Yes
16169	14:50 X vs. 28:0 X	-1.249	-1.410 to -1.088	Yes
16170	14:50 X vs. 28:1 X	-1.290	-1.434 to -1.146	Yes
16171	14:50 X vs. 28:10 X	-1.091	-1.252 to -0.9304	Yes
16172	14:50 X vs. 28:40 X	-0.3693	-0.5133 to -0.2254	Yes
16173	14:50 X vs. 28:50 X	-0.2647	-0.4086 to -0.1207	Yes
16174	14:50 X vs. 29:0 X	-1.730	-1.891 to -1.569	Yes
16175	14:50 X vs. 29:1 X	-1.815	-1.976 to -1.654	Yes
16176	14:50 X vs. 29:10 X	-1.030	-1.191 to -0.8689	Yes
16177	14:50 X vs. 29:40 X	-0.4773	-0.6213 to -0.3334	Yes
16178	14:50 X vs. 29:50 X	-0.2660	-0.4100 to -0.1220	Yes
16179	14:50 X vs. 30:0 X	-1.445	-1.606 to -1.284	Yes
16180	14:50 X vs. 30:1 X	-1.766	-1.927 to -1.605	Yes
16181	14:50 X vs. 30:10 X	-1.104	-1.265 to -0.9434	Yes
16182	14:50 X vs. 30:40 X	-0.4043	-0.5483 to -0.2604	Yes
16183	14:50 X vs. 30:50 X	-0.3183	-0.4623 to -0.1744	Yes
16184	14:50 X vs. 31:0 X	-1.763	-1.924 to -1.602	Yes
16185	14:50 X vs. 31:1 X	-1.753	-1.914 to -1.592	Yes
16186	14:50 X vs. 31:10 X	-1.236	-1.397 to -1.075	Yes
16187	14:50 X vs. 31:40 X	-0.4960	-0.6400 to -0.3520	Yes
16188	14:50 X vs. 31:50 X	-0.2973	-0.4413 to -0.1534	Yes
16189	14:50 X vs. 32:0 X	-2.354	-2.498 to -2.210	Yes
16190	14:50 X vs. 32:1 X	-1.810	-1.971 to -1.649	Yes
16191	14:50 X vs. 32:10 X	-1.666	-1.810 to -1.522	Yes
16192	14:50 X vs. 32:40 X	-0.4923	-0.6363 to -0.3484	Yes
16193	14:50 X vs. 32:50 X	-0.2970	-0.4410 to -0.1530	Yes
16194	14:50 X vs. 33:0 X	-2.358	-2.502 to -2.214	Yes
16195	14:50 X vs. 33:1 X	-2.173	-2.317 to -2.029	Yes
16196	14:50 X vs. 33:10 X	-1.833	-1.977 to -1.689	Yes
16197	14:50 X vs. 33:40 X	-0.5110	-0.6550 to -0.3670	Yes
16198	14:50 X vs. 33:50 X	-0.3120	-0.4560 to -0.1680	Yes
16199	14:50 X vs. 34:0 X	-2.283	-2.427 to -2.139	Yes
16200	14:50 X vs. 34:1 X	-2.290	-2.434 to -2.146	Yes

	2way ANOVA<br>Multiple comparisons	A<br>Data Set-A<br>Y	B<br>Data Set-B<br>Y	C<br>Data Set-C<br>Y
16201	14:**50 X** vs. 34:**10 X**	-1.923	-2.067 to -1.779	Yes
16202	14:**50 X** vs. 34:**40 X**	-0.5230	-0.6670 to -0.3790	Yes
16203	14:**50 X** vs. 34:**50 X**	-0.4610	-0.6050 to -0.3170	Yes
16204	14:**50 X** vs. 35:**0 X**	-2.631	-2.775 to -2.487	Yes
16205	14:**50 X** vs. 35:**1 X**	-2.397	-2.541 to -2.253	Yes
16206	14:**50 X** vs. 35:**10 X**	-1.885	-2.029 to -1.741	Yes
16207	14:**50 X** vs. 35:**40 X**	-0.5320	-0.6760 to -0.3880	Yes
16208	14:**50 X** vs. 35:**50 X**	-0.4737	-0.6176 to -0.3297	Yes
16209	14:**50 X** vs. 36:**0 X**	-2.838	-2.999 to -2.677	Yes
16210	14:**50 X** vs. 36:**1 X**	-2.534	-2.678 to -2.390	Yes
16211	14:**50 X** vs. 36:**10 X**	-1.891	-2.035 to -1.747	Yes
16212	14:**50 X** vs. 36:**40 X**	-0.5873	-0.7313 to -0.4434	Yes
16213	14:**50 X** vs. 36:**50 X**	-0.4370	-0.5810 to -0.2930	Yes
16214	14:**50 X** vs. 37:**0 X**	-3.222	-3.366 to -3.078	Yes
16215	14:**50 X** vs. 37:**1 X**	-2.604	-2.748 to -2.460	Yes
16216	14:**50 X** vs. 37:**10 X**	-1.892	-2.036 to -1.748	Yes
16217	14:**50 X** vs. 37:**40 X**	-0.6247	-0.7686 to -0.4807	Yes
16218	14:**50 X** vs. 37:**50 X**	-0.4457	-0.5896 to -0.3017	Yes
16219	14:**50 X** vs. 38:**0 X**	-3.167	-3.311 to -3.023	Yes
16220	14:**50 X** vs. 38:**1 X**	-2.722	-2.866 to -2.578	Yes
16221	14:**50 X** vs. 38:**10 X**	-2.168	-2.312 to -2.024	Yes
16222	14:**50 X** vs. 38:**40 X**	-0.6290	-0.7730 to -0.4850	Yes
16223	14:**50 X** vs. 38:**50 X**	-0.4497	-0.5936 to -0.3057	Yes
16224	14:**50 X** vs. 39:**0 X**	-3.397	-3.541 to -3.253	Yes
16225	14:**50 X** vs. 39:**1 X**	-3.289	-3.433 to -3.145	Yes
16226	14:**50 X** vs. 39:**10 X**	-2.313	-2.457 to -2.169	Yes
16227	14:**50 X** vs. 39:**40 X**	-0.6350	-0.7790 to -0.4910	Yes
16228	14:**50 X** vs. 39:**50 X**	-0.4807	-0.6246 to -0.3367	Yes
16229	14:**50 X** vs. 40:**0 X**	-3.438	-3.582 to -3.294	Yes
16230	14:**50 X** vs. 40:**1 X**	-3.364	-3.508 to -3.220	Yes
16231	14:**50 X** vs. 40:**10 X**	-2.569	-2.713 to -2.425	Yes
16232	14:**50 X** vs. 40:**40 X**	-0.6397	-0.7836 to -0.4957	Yes
16233	14:**50 X** vs. 40:**50 X**	-0.4870	-0.6310 to -0.3430	Yes
16234	14:**50 X** vs. 41:**0 X**	-3.479	-3.623 to -3.335	Yes
16235	14:**50 X** vs. 41:**1 X**	-3.478	-3.622 to -3.334	Yes
16236	14:**50 X** vs. 41:**10 X**	-2.623	-2.767 to -2.479	Yes
16237	14:**50 X** vs. 41:**40 X**	-0.5870	-0.7310 to -0.4430	Yes
16238	14:**50 X** vs. 41:**50 X**	-0.4997	-0.6436 to -0.3557	Yes
16239	14:**50 X** vs. 42:**0 X**	-3.621	-3.765 to -3.477	Yes
16240	14:**50 X** vs. 42:**1 X**	-3.641	-3.785 to -3.497	Yes
16241	14:**50 X** vs. 42:**10 X**	-2.623	-2.767 to -2.479	Yes
16242	14:**50 X** vs. 42:**40 X**	-0.5997	-0.7436 to -0.4557	Yes
16243	14:**50 X** vs. 42:**50 X**	-0.5030	-0.6470 to -0.3590	Yes
16244	14:**50 X** vs. 43:**0 X**	-3.711	-3.855 to -3.567	Yes
16245	14:**50 X** vs. 43:**1 X**	-3.625	-3.769 to -3.481	Yes

2way ANOVA<br>Multiple comparisons	A<br>Data Set-A<br>Y	B<br>Data Set-B<br>Y	C<br>Data Set-C<br>Y
16246 14:50 X vs. 43:10 X	-3.341	-3.485 to -3.197	Yes
16247 14:50 X vs. 43:40 X	-0.5980	-0.7420 to -0.4540	Yes
16248 14:50 X vs. 43:50 X	-0.5033	-0.6473 to -0.3594	Yes
16249 14:50 X vs. 44:0 X	-3.735	-3.879 to -3.591	Yes
16250 14:50 X vs. 44:1 X	-3.625	-3.769 to -3.481	Yes
16251 14:50 X vs. 44:10 X	-3.404	-3.548 to -3.260	Yes
16252 14:50 X vs. 44:40 X	-0.6000	-0.7440 to -0.4560	Yes
16253 14:50 X vs. 44:50 X	-0.4357	-0.5796 to -0.2917	Yes
16254 14:50 X vs. 45:0 X	-3.600	-3.744 to -3.456	Yes
16255 14:50 X vs. 45:1 X	-3.402	-3.546 to -3.258	Yes
16256 14:50 X vs. 45:10 X	-3.284	-3.428 to -3.140	Yes
16257 14:50 X vs. 45:40 X	-0.6013	-0.7453 to -0.4574	Yes
16258 14:50 X vs. 45:50 X	-0.4327	-0.5766 to -0.2887	Yes
16259 14:50 X vs. 46:0 X	-3.404	-3.548 to -3.260	Yes
16260 14:50 X vs. 46:1 X	-3.371	-3.515 to -3.227	Yes
16261 14:50 X vs. 46:10 X	-3.279	-3.423 to -3.135	Yes
16262 14:50 X vs. 46:40 X	-0.6150	-0.7590 to -0.4710	Yes
16263 14:50 X vs. 46:50 X	-0.4257	-0.5696 to -0.2817	Yes
16264 14:50 X vs. 47:0 X	-3.380	-3.524 to -3.236	Yes
16265 14:50 X vs. 47:1 X	-3.284	-3.428 to -3.140	Yes
16266 14:50 X vs. 47:10 X	-3.265	-3.409 to -3.121	Yes
16267 14:50 X vs. 47:40 X	-0.6250	-0.7690 to -0.4810	Yes
16268 14:50 X vs. 47:50 X	-0.3837	-0.5276 to -0.2397	Yes
16269 14:50 X vs. 48:0 X	-3.367	-3.528 to -3.206	Yes
16270 14:50 X vs. 48:1 X	-3.271	-3.415 to -3.127	Yes
16271 14:50 X vs. 48:10 X	-3.265	-3.409 to -3.121	Yes
16272 14:50 X vs. 48:40 X	-0.6313	-0.7753 to -0.4874	Yes
16273 14:50 X vs. 48:50 X	-0.3910	-0.5350 to -0.2470	Yes
16274 14:50 X vs. 49:0 X	-3.367	-3.511 to -3.223	Yes
16275 14:50 X vs. 49:1 X	-3.271	-3.415 to -3.127	Yes
16276 14:50 X vs. 49:10 X	-3.265	-3.409 to -3.121	Yes
16277 14:50 X vs. 49:40 X	-0.6357	-0.7796 to -0.4917	Yes
16278 14:50 X vs. 49:50 X	-0.3903	-0.5343 to -0.2464	Yes
16279 14:50 X vs. 50:0 X	-3.367	-3.511 to -3.223	Yes
16280 14:50 X vs. 50:1 X	-3.271	-3.415 to -3.127	Yes
16281 14:50 X vs. 50:10 X	-3.265	-3.409 to -3.121	Yes
16282 14:50 X vs. 50:40 X	-0.6433	-0.7873 to -0.4994	Yes
16283 14:50 X vs. 50:50 X	-0.3900	-0.5340 to -0.2460	Yes
16284 15:0 X vs. 15:1 X	0.0200	-0.1240 to 0.1640	No
16285 15:0 X vs. 15:10 X	-0.007333	-0.1513 to 0.1366	No
16286 15:0 X vs. 15:40 X	0.01033	-0.1336 to 0.1543	No
16287 15:0 X vs. 15:50 X	-0.007333	-0.1513 to 0.1366	No
16288 15:0 X vs. 16:0 X	-0.09233	-0.2363 to 0.05163	No
16289 15:0 X vs. 16:1 X	0.01667	-0.1273 to 0.1606	No
16290 15:0 X vs. 16:10 X	-0.009000	-0.1530 to 0.1350	No

	2way ANOVA<br>Multiple comparisons	A<br>Data Set-A<br>Y	B<br>Data Set-B<br>Y	C<br>Data Set-C<br>Y
16291	15:0 X vs. 16:40 X	-0.02833	-0.1723 to 0.1156	No
16292	15:0 X vs. 16:50 X	-0.07067	-0.2146 to 0.07329	No
16293	15:0 X vs. 17:0 X	-0.1450	-0.2890 to -0.001040	Yes
16294	15:0 X vs. 17:1 X	-0.05933	-0.2033 to 0.08463	No
16295	15:0 X vs. 17:10 X	-0.1160	-0.2600 to 0.02796	No
16296	15:0 X vs. 17:40 X	-0.03933	-0.1833 to 0.1046	No
16297	15:0 X vs. 17:50 X	-0.08467	-0.2286 to 0.05929	No
16298	15:0 X vs. 18:0 X	-0.1937	-0.3376 to -0.04971	Yes
16299	15:0 X vs. 18:1 X	-0.1020	-0.2460 to 0.04196	No
16300	15:0 X vs. 18:10 X	-0.1943	-0.3383 to -0.05037	Yes
16301	15:0 X vs. 18:40 X	-0.04367	-0.1876 to 0.1003	No
16302	15:0 X vs. 18:50 X	-0.08867	-0.2326 to 0.05529	No
16303	15:0 X vs. 19:0 X	-0.2287	-0.3726 to -0.08471	Yes
16304	15:0 X vs. 19:1 X	-0.2167	-0.3606 to -0.07271	Yes
16305	15:0 X vs. 19:10 X	-0.2143	-0.3583 to -0.07037	Yes
16306	15:0 X vs. 19:40 X	-0.07233	-0.2163 to 0.07163	No
16307	15:0 X vs. 19:50 X	-0.08333	-0.2273 to 0.06063	No
16308	15:0 X vs. 20:0 X	-0.3083	-0.4523 to -0.1644	Yes
16309	15:0 X vs. 20:1 X	-0.1277	-0.2716 to 0.01629	No
16310	15:0 X vs. 20:10 X	-0.2167	-0.3606 to -0.07271	Yes
16311	15:0 X vs. 20:40 X	-0.0840	-0.2280 to 0.05996	No
16312	15:0 X vs. 20:50 X	-0.1280	-0.2720 to 0.01596	No
16313	15:0 X vs. 21:0 X	-0.3317	-0.4756 to -0.1877	Yes
16314	15:0 X vs. 21:1 X	-0.2847	-0.4286 to -0.1407	Yes
16315	15:0 X vs. 21:10 X	-0.2817	-0.4256 to -0.1377	Yes
16316	15:0 X vs. 21:40 X	-0.1570	-0.3010 to -0.01304	Yes
16317	15:0 X vs. 21:50 X	-0.2473	-0.3913 to -0.1034	Yes
16318	15:0 X vs. 22:0 X	-0.3733	-0.5173 to -0.2294	Yes
16319	15:0 X vs. 22:1 X	-0.3680	-0.5120 to -0.2240	Yes
16320	15:0 X vs. 22:10 X	-0.3633	-0.5073 to -0.2194	Yes
16321	15:0 X vs. 22:40 X	-0.2637	-0.4076 to -0.1197	Yes
16322	15:0 X vs. 22:50 X	-0.3263	-0.4703 to -0.1824	Yes
16323	15:0 X vs. 23:0 X	-0.4387	-0.5826 to -0.2947	Yes
16324	15:0 X vs. 23:1 X	-0.6283	-0.7893 to -0.4674	Yes
16325	15:0 X vs. 23:10 X	-0.3780	-0.5220 to -0.2340	Yes
16326	15:0 X vs. 23:40 X	-0.2583	-0.4193 to -0.09738	Yes
16327	15:0 X vs. 23:50 X	-0.2923	-0.4363 to -0.1484	Yes
16328	15:0 X vs. 24:0 X	-0.5560	-0.7000 to -0.4120	Yes
16329	15:0 X vs. 24:1 X	-0.7123	-0.8733 to -0.5514	Yes
16330	15:0 X vs. 24:10 X	-0.5398	-0.7008 to -0.3789	Yes
16331	15:0 X vs. 24:40 X	-0.2747	-0.4186 to -0.1307	Yes
16332	15:0 X vs. 24:50 X	-0.1963	-0.3403 to -0.05237	Yes
16333	15:0 X vs. 25:0 X	-0.8933	-1.054 to -0.7324	Yes
16334	15:0 X vs. 25:1 X	-1.083	-1.244 to -0.9219	Yes
16335	15:0 X vs. 25:10 X	-0.4443	-0.6479 to -0.2407	Yes

	2way ANOVA<br>Multiple comparisons	A<br>Data Set-A<br>Y	B<br>Data Set-B<br>Y	C<br>Data Set-C<br>Y
16336	15:**0 X** vs. 25:**40 X**	-0.2767	-0.4206 to -0.1327	Yes
16337	15:**0 X** vs. 25:**50 X**	-0.2510	-0.3950 to -0.1070	Yes
16338	15:**0 X** vs. 26:**0 X**	-1.106	-1.267 to -0.9449	Yes
16339	15:**0 X** vs. 26:**1 X**	-1.219	-1.380 to -1.058	Yes
16340	15:**0 X** vs. 26:**10 X**	-0.9053	-1.066 to -0.7444	Yes
16341	15:**0 X** vs. 26:**40 X**	-0.3510	-0.4950 to -0.2070	Yes
16342	15:**0 X** vs. 26:**50 X**	-0.2113	-0.3553 to -0.06737	Yes
16343	15:**0 X** vs. 27:**0 X**	-1.294	-1.438 to -1.150	Yes
16344	15:**0 X** vs. 27:**1 X**	-1.384	-1.545 to -1.223	Yes
16345	15:**0 X** vs. 27:**10 X**	-1.196	-1.357 to -1.035	Yes
16346	15:**0 X** vs. 27:**40 X**	-0.3510	-0.4950 to -0.2070	Yes
16347	15:**0 X** vs. 27:**50 X**	-0.2107	-0.3546 to -0.06671	Yes
16348	15:**0 X** vs. 28:**0 X**	-1.243	-1.404 to -1.082	Yes
16349	15:**0 X** vs. 28:**1 X**	-1.284	-1.428 to -1.140	Yes
16350	15:**0 X** vs. 28:**10 X**	-1.085	-1.246 to -0.9244	Yes
16351	15:**0 X** vs. 28:**40 X**	-0.3633	-0.5073 to -0.2194	Yes
16352	15:**0 X** vs. 28:**50 X**	-0.2587	-0.4026 to -0.1147	Yes
16353	15:**0 X** vs. 29:**0 X**	-1.724	-1.885 to -1.563	Yes
16354	15:**0 X** vs. 29:**1 X**	-1.809	-1.970 to -1.648	Yes
16355	15:**0 X** vs. 29:**10 X**	-1.024	-1.185 to -0.8629	Yes
16356	15:**0 X** vs. 29:**40 X**	-0.4713	-0.6153 to -0.3274	Yes
16357	15:**0 X** vs. 29:**50 X**	-0.2600	-0.4040 to -0.1160	Yes
16358	15:**0 X** vs. 30:**0 X**	-1.439	-1.600 to -1.278	Yes
16359	15:**0 X** vs. 30:**1 X**	-1.760	-1.921 to -1.599	Yes
16360	15:**0 X** vs. 30:**10 X**	-1.098	-1.259 to -0.9374	Yes
16361	15:**0 X** vs. 30:**40 X**	-0.3983	-0.5423 to -0.2544	Yes
16362	15:**0 X** vs. 30:**50 X**	-0.3123	-0.4563 to -0.1684	Yes
16363	15:**0 X** vs. 31:**0 X**	-1.757	-1.918 to -1.596	Yes
16364	15:**0 X** vs. 31:**1 X**	-1.747	-1.908 to -1.586	Yes
16365	15:**0 X** vs. 31:**10 X**	-1.230	-1.391 to -1.069	Yes
16366	15:**0 X** vs. 31:**40 X**	-0.4900	-0.6340 to -0.3460	Yes
16367	15:**0 X** vs. 31:**50 X**	-0.2913	-0.4353 to -0.1474	Yes
16368	15:**0 X** vs. 32:**0 X**	-2.348	-2.492 to -2.204	Yes
16369	15:**0 X** vs. 32:**1 X**	-1.804	-1.965 to -1.643	Yes
16370	15:**0 X** vs. 32:**10 X**	-1.660	-1.804 to -1.516	Yes
16371	15:**0 X** vs. 32:**40 X**	-0.4863	-0.6303 to -0.3424	Yes
16372	15:**0 X** vs. 32:**50 X**	-0.2910	-0.4350 to -0.1470	Yes
16373	15:**0 X** vs. 33:**0 X**	-2.352	-2.496 to -2.208	Yes
16374	15:**0 X** vs. 33:**1 X**	-2.167	-2.311 to -2.023	Yes
16375	15:**0 X** vs. 33:**10 X**	-1.827	-1.971 to -1.683	Yes
16376	15:**0 X** vs. 33:**40 X**	-0.5050	-0.6490 to -0.3610	Yes
16377	15:**0 X** vs. 33:**50 X**	-0.3060	-0.4500 to -0.1620	Yes
16378	15:**0 X** vs. 34:**0 X**	-2.277	-2.421 to -2.133	Yes
16379	15:**0 X** vs. 34:**1 X**	-2.284	-2.428 to -2.140	Yes
16380	15:**0 X** vs. 34:**10 X**	-1.917	-2.061 to -1.773	Yes

2way ANOVA<br>Multiple comparisons		A<br>Data Set-A<br>Y	B<br>Data Set-B<br>Y	C<br>Data Set-C<br>Y
16381	15:**0 X** vs. 34:**40 X**	-0.5170	-0.6610 to -0.3730	Yes
16382	15:**0 X** vs. 34:**50 X**	-0.4550	-0.5990 to -0.3110	Yes
16383	15:**0 X** vs. 35:**0 X**	-2.625	-2.769 to -2.481	Yes
16384	15:**0 X** vs. 35:**1 X**	-2.391	-2.535 to -2.247	Yes
16385	15:**0 X** vs. 35:**10 X**	-1.879	-2.023 to -1.735	Yes
16386	15:**0 X** vs. 35:**40 X**	-0.5260	-0.6700 to -0.3820	Yes
16387	15:**0 X** vs. 35:**50 X**	-0.4677	-0.6116 to -0.3237	Yes
16388	15:**0 X** vs. 36:**0 X**	-2.832	-2.993 to -2.671	Yes
16389	15:**0 X** vs. 36:**1 X**	-2.528	-2.672 to -2.384	Yes
16390	15:**0 X** vs. 36:**10 X**	-1.885	-2.029 to -1.741	Yes
16391	15:**0 X** vs. 36:**40 X**	-0.5813	-0.7253 to -0.4374	Yes
16392	15:**0 X** vs. 36:**50 X**	-0.4310	-0.5750 to -0.2870	Yes
16393	15:**0 X** vs. 37:**0 X**	-3.216	-3.360 to -3.072	Yes
16394	15:**0 X** vs. 37:**1 X**	-2.598	-2.742 to -2.454	Yes
16395	15:**0 X** vs. 37:**10 X**	-1.886	-2.030 to -1.742	Yes
16396	15:**0 X** vs. 37:**40 X**	-0.6187	-0.7626 to -0.4747	Yes
16397	15:**0 X** vs. 37:**50 X**	-0.4397	-0.5836 to -0.2957	Yes
16398	15:**0 X** vs. 38:**0 X**	-3.161	-3.305 to -3.017	Yes
16399	15:**0 X** vs. 38:**1 X**	-2.716	-2.860 to -2.572	Yes
16400	15:**0 X** vs. 38:**10 X**	-2.162	-2.306 to -2.018	Yes
16401	15:**0 X** vs. 38:**40 X**	-0.6230	-0.7670 to -0.4790	Yes
16402	15:**0 X** vs. 38:**50 X**	-0.4437	-0.5876 to -0.2997	Yes
16403	15:**0 X** vs. 39:**0 X**	-3.391	-3.535 to -3.247	Yes
16404	15:**0 X** vs. 39:**1 X**	-3.283	-3.427 to -3.139	Yes
16405	15:**0 X** vs. 39:**10 X**	-2.307	-2.451 to -2.163	Yes
16406	15:**0 X** vs. 39:**40 X**	-0.6290	-0.7730 to -0.4850	Yes
16407	15:**0 X** vs. 39:**50 X**	-0.4747	-0.6186 to -0.3307	Yes
16408	15:**0 X** vs. 40:**0 X**	-3.432	-3.576 to -3.288	Yes
16409	15:**0 X** vs. 40:**1 X**	-3.358	-3.502 to -3.214	Yes
16410	15:**0 X** vs. 40:**10 X**	-2.563	-2.707 to -2.419	Yes
16411	15:**0 X** vs. 40:**40 X**	-0.6337	-0.7776 to -0.4897	Yes
16412	15:**0 X** vs. 40:**50 X**	-0.4810	-0.6250 to -0.3370	Yes
16413	15:**0 X** vs. 41:**0 X**	-3.473	-3.617 to -3.329	Yes
16414	15:**0 X** vs. 41:**1 X**	-3.472	-3.616 to -3.328	Yes
16415	15:**0 X** vs. 41:**10 X**	-2.617	-2.761 to -2.473	Yes
16416	15:**0 X** vs. 41:**40 X**	-0.5810	-0.7250 to -0.4370	Yes
16417	15:**0 X** vs. 41:**50 X**	-0.4937	-0.6376 to -0.3497	Yes
16418	15:**0 X** vs. 42:**0 X**	-3.615	-3.759 to -3.471	Yes
16419	15:**0 X** vs. 42:**1 X**	-3.635	-3.779 to -3.491	Yes
16420	15:**0 X** vs. 42:**10 X**	-2.617	-2.761 to -2.473	Yes
16421	15:**0 X** vs. 42:**40 X**	-0.5937	-0.7376 to -0.4497	Yes
16422	15:**0 X** vs. 42:**50 X**	-0.4970	-0.6410 to -0.3530	Yes
16423	15:**0 X** vs. 43:**0 X**	-3.705	-3.849 to -3.561	Yes
16424	15:**0 X** vs. 43:**1 X**	-3.619	-3.763 to -3.475	Yes
16425	15:**0 X** vs. 43:**10 X**	-3.335	-3.479 to -3.191	Yes

	2way ANOVA<br>Multiple comparisons	A<br>Data Set-A<br>Y	B<br>Data Set-B<br>Y	C<br>Data Set-C<br>Y
16426	15:**0 X** vs. 43:**40 X**	-0.5920	-0.7360 to -0.4480	Yes
16427	15:**0 X** vs. 43:**50 X**	-0.4973	-0.6413 to -0.3534	Yes
16428	15:**0 X** vs. 44:**0 X**	-3.729	-3.873 to -3.585	Yes
16429	15:**0 X** vs. 44:**1 X**	-3.619	-3.763 to -3.475	Yes
16430	15:**0 X** vs. 44:**10 X**	-3.398	-3.542 to -3.254	Yes
16431	15:**0 X** vs. 44:**40 X**	-0.5940	-0.7380 to -0.4500	Yes
16432	15:**0 X** vs. 44:**50 X**	-0.4297	-0.5736 to -0.2857	Yes
16433	15:**0 X** vs. 45:**0 X**	-3.594	-3.738 to -3.450	Yes
16434	15:**0 X** vs. 45:**1 X**	-3.396	-3.540 to -3.252	Yes
16435	15:**0 X** vs. 45:**10 X**	-3.278	-3.422 to -3.134	Yes
16436	15:**0 X** vs. 45:**40 X**	-0.5953	-0.7393 to -0.4514	Yes
16437	15:**0 X** vs. 45:**50 X**	-0.4267	-0.5706 to -0.2827	Yes
16438	15:**0 X** vs. 46:**0 X**	-3.398	-3.542 to -3.254	Yes
16439	15:**0 X** vs. 46:**1 X**	-3.365	-3.509 to -3.221	Yes
16440	15:**0 X** vs. 46:**10 X**	-3.273	-3.417 to -3.129	Yes
16441	15:**0 X** vs. 46:**40 X**	-0.6090	-0.7530 to -0.4650	Yes
16442	15:**0 X** vs. 46:**50 X**	-0.4197	-0.5636 to -0.2757	Yes
16443	15:**0 X** vs. 47:**0 X**	-3.374	-3.518 to -3.230	Yes
16444	15:**0 X** vs. 47:**1 X**	-3.278	-3.422 to -3.134	Yes
16445	15:**0 X** vs. 47:**10 X**	-3.259	-3.403 to -3.115	Yes
16446	15:**0 X** vs. 47:**40 X**	-0.6190	-0.7630 to -0.4750	Yes
16447	15:**0 X** vs. 47:**50 X**	-0.3777	-0.5216 to -0.2337	Yes
16448	15:**0 X** vs. 48:**0 X**	-3.361	-3.522 to -3.200	Yes
16449	15:**0 X** vs. 48:**1 X**	-3.265	-3.409 to -3.121	Yes
16450	15:**0 X** vs. 48:**10 X**	-3.259	-3.403 to -3.115	Yes
16451	15:**0 X** vs. 48:**40 X**	-0.6253	-0.7693 to -0.4814	Yes
16452	15:**0 X** vs. 48:**50 X**	-0.3850	-0.5290 to -0.2410	Yes
16453	15:**0 X** vs. 49:**0 X**	-3.361	-3.505 to -3.217	Yes
16454	15:**0 X** vs. 49:**1 X**	-3.265	-3.409 to -3.121	Yes
16455	15:**0 X** vs. 49:**10 X**	-3.259	-3.403 to -3.115	Yes
16456	15:**0 X** vs. 49:**40 X**	-0.6297	-0.7736 to -0.4857	Yes
16457	15:**0 X** vs. 49:**50 X**	-0.3843	-0.5283 to -0.2404	Yes
16458	15:**0 X** vs. 50:**0 X**	-3.361	-3.505 to -3.217	Yes
16459	15:**0 X** vs. 50:**1 X**	-3.265	-3.409 to -3.121	Yes
16460	15:**0 X** vs. 50:**10 X**	-3.259	-3.403 to -3.115	Yes
16461	15:**0 X** vs. 50:**40 X**	-0.6373	-0.7813 to -0.4934	Yes
16462	15:**0 X** vs. 50:**50 X**	-0.3840	-0.5280 to -0.2400	Yes
16463	15:**1 X** vs. 15:**10 X**	-0.02733	-0.1713 to 0.1166	No
16464	15:**1 X** vs. 15:**40 X**	-0.009667	-0.1536 to 0.1343	No
16465	15:**1 X** vs. 15:**50 X**	-0.02733	-0.1713 to 0.1166	No
16466	15:**1 X** vs. 16:**0 X**	-0.1123	-0.2563 to 0.03163	No
16467	15:**1 X** vs. 16:**1 X**	-0.003333	-0.1473 to 0.1406	No
16468	15:**1 X** vs. 16:**10 X**	-0.0290	-0.1730 to 0.1150	No
16469	15:**1 X** vs. 16:**40 X**	-0.04833	-0.1923 to 0.09563	No
16470	15:**1 X** vs. 16:**50 X**	-0.09067	-0.2346 to 0.05329	No

	2way ANOVA<br>Multiple comparisons	A<br>Data Set-A<br>Y	B<br>Data Set-B<br>Y	C<br>Data Set-C<br>Y
16471	15:**1 X** vs. 17:**0 X**	-0.1650	-0.3090 to -0.02104	Yes
16472	15:**1 X** vs. 17:**1 X**	-0.07933	-0.2233 to 0.06463	No
16473	15:**1 X** vs. 17:**10 X**	-0.1360	-0.2800 to 0.007960	No
16474	15:**1 X** vs. 17:**40 X**	-0.05933	-0.2033 to 0.08463	No
16475	15:**1 X** vs. 17:**50 X**	-0.1047	-0.2486 to 0.03929	No
16476	15:**1 X** vs. 18:**0 X**	-0.2137	-0.3576 to -0.06971	Yes
16477	15:**1 X** vs. 18:**1 X**	-0.1220	-0.2660 to 0.02196	No
16478	15:**1 X** vs. 18:**10 X**	-0.2143	-0.3583 to -0.07037	Yes
16479	15:**1 X** vs. 18:**40 X**	-0.06367	-0.2076 to 0.08029	No
16480	15:**1 X** vs. 18:**50 X**	-0.1087	-0.2526 to 0.03529	No
16481	15:**1 X** vs. 19:**0 X**	-0.2487	-0.3926 to -0.1047	Yes
16482	15:**1 X** vs. 19:**1 X**	-0.2367	-0.3806 to -0.09271	Yes
16483	15:**1 X** vs. 19:**10 X**	-0.2343	-0.3783 to -0.09037	Yes
16484	15:**1 X** vs. 19:**40 X**	-0.09233	-0.2363 to 0.05163	No
16485	15:**1 X** vs. 19:**50 X**	-0.1033	-0.2473 to 0.04063	No
16486	15:**1 X** vs. 20:**0 X**	-0.3283	-0.4723 to -0.1844	Yes
16487	15:**1 X** vs. 20:**1 X**	-0.1477	-0.2916 to -0.003707	Yes
16488	15:**1 X** vs. 20:**10 X**	-0.2367	-0.3806 to -0.09271	Yes
16489	15:**1 X** vs. 20:**40 X**	-0.1040	-0.2480 to 0.03996	No
16490	15:**1 X** vs. 20:**50 X**	-0.1480	-0.2920 to -0.004040	Yes
16491	15:**1 X** vs. 21:**0 X**	-0.3517	-0.4956 to -0.2077	Yes
16492	15:**1 X** vs. 21:**1 X**	-0.3047	-0.4486 to -0.1607	Yes
16493	15:**1 X** vs. 21:**10 X**	-0.3017	-0.4456 to -0.1577	Yes
16494	15:**1 X** vs. 21:**40 X**	-0.1770	-0.3210 to -0.03304	Yes
16495	15:**1 X** vs. 21:**50 X**	-0.2673	-0.4113 to -0.1234	Yes
16496	15:**1 X** vs. 22:**0 X**	-0.3933	-0.5373 to -0.2494	Yes
16497	15:**1 X** vs. 22:**1 X**	-0.3880	-0.5320 to -0.2440	Yes
16498	15:**1 X** vs. 22:**10 X**	-0.3833	-0.5273 to -0.2394	Yes
16499	15:**1 X** vs. 22:**40 X**	-0.2837	-0.4276 to -0.1397	Yes
16500	15:**1 X** vs. 22:**50 X**	-0.3463	-0.4903 to -0.2024	Yes
16501	15:**1 X** vs. 23:**0 X**	-0.4587	-0.6026 to -0.3147	Yes
16502	15:**1 X** vs. 23:**1 X**	-0.6483	-0.8093 to -0.4874	Yes
16503	15:**1 X** vs. 23:**10 X**	-0.3980	-0.5420 to -0.2540	Yes
16504	15:**1 X** vs. 23:**40 X**	-0.2783	-0.4393 to -0.1174	Yes
16505	15:**1 X** vs. 23:**50 X**	-0.3123	-0.4563 to -0.1684	Yes
16506	15:**1 X** vs. 24:**0 X**	-0.5760	-0.7200 to -0.4320	Yes
16507	15:**1 X** vs. 24:**1 X**	-0.7323	-0.8933 to -0.5714	Yes
16508	15:**1 X** vs. 24:**10 X**	-0.5598	-0.7208 to -0.3989	Yes
16509	15:**1 X** vs. 24:**40 X**	-0.2947	-0.4386 to -0.1507	Yes
16510	15:**1 X** vs. 24:**50 X**	-0.2163	-0.3603 to -0.07237	Yes
16511	15:**1 X** vs. 25:**0 X**	-0.9133	-1.074 to -0.7524	Yes
16512	15:**1 X** vs. 25:**1 X**	-1.103	-1.264 to -0.9419	Yes
16513	15:**1 X** vs. 25:**10 X**	-0.4643	-0.6679 to -0.2607	Yes
16514	15:**1 X** vs. 25:**40 X**	-0.2967	-0.4406 to -0.1527	Yes
16515	15:**1 X** vs. 25:**50 X**	-0.2710	-0.4150 to -0.1270	Yes

2way ANOVA<br>Multiple comparisons	A<br>Data Set-A<br>Y	B<br>Data Set-B<br>Y	C<br>Data Set-C<br>Y
16516 15:1 X vs. 26:0 X	-1.126	-1.287 to -0.9649	Yes
16517 15:1 X vs. 26:1 X	-1.239	-1.400 to -1.078	Yes
16518 15:1 X vs. 26:10 X	-0.9253	-1.086 to -0.7644	Yes
16519 15:1 X vs. 26:40 X	-0.3710	-0.5150 to -0.2270	Yes
16520 15:1 X vs. 26:50 X	-0.2313	-0.3753 to -0.08737	Yes
16521 15:1 X vs. 27:0 X	-1.314	-1.458 to -1.170	Yes
16522 15:1 X vs. 27:1 X	-1.404	-1.565 to -1.243	Yes
16523 15:1 X vs. 27:10 X	-1.216	-1.377 to -1.055	Yes
16524 15:1 X vs. 27:40 X	-0.3710	-0.5150 to -0.2270	Yes
16525 15:1 X vs. 27:50 X	-0.2307	-0.3746 to -0.08671	Yes
16526 15:1 X vs. 28:0 X	-1.263	-1.424 to -1.102	Yes
16527 15:1 X vs. 28:1 X	-1.304	-1.448 to -1.160	Yes
16528 15:1 X vs. 28:10 X	-1.105	-1.266 to -0.9444	Yes
16529 15:1 X vs. 28:40 X	-0.3833	-0.5273 to -0.2394	Yes
16530 15:1 X vs. 28:50 X	-0.2787	-0.4226 to -0.1347	Yes
16531 15:1 X vs. 29:0 X	-1.744	-1.905 to -1.583	Yes
16532 15:1 X vs. 29:1 X	-1.829	-1.990 to -1.668	Yes
16533 15:1 X vs. 29:10 X	-1.044	-1.205 to -0.8829	Yes
16534 15:1 X vs. 29:40 X	-0.4913	-0.6353 to -0.3474	Yes
16535 15:1 X vs. 29:50 X	-0.2800	-0.4240 to -0.1360	Yes
16536 15:1 X vs. 30:0 X	-1.459	-1.620 to -1.298	Yes
16537 15:1 X vs. 30:1 X	-1.780	-1.941 to -1.619	Yes
16538 15:1 X vs. 30:10 X	-1.118	-1.279 to -0.9574	Yes
16539 15:1 X vs. 30:40 X	-0.4183	-0.5623 to -0.2744	Yes
16540 15:1 X vs. 30:50 X	-0.3323	-0.4763 to -0.1884	Yes
16541 15:1 X vs. 31:0 X	-1.777	-1.938 to -1.616	Yes
16542 15:1 X vs. 31:1 X	-1.767	-1.928 to -1.606	Yes
16543 15:1 X vs. 31:10 X	-1.250	-1.411 to -1.089	Yes
16544 15:1 X vs. 31:40 X	-0.5100	-0.6540 to -0.3660	Yes
16545 15:1 X vs. 31:50 X	-0.3113	-0.4553 to -0.1674	Yes
16546 15:1 X vs. 32:0 X	-2.368	-2.512 to -2.224	Yes
16547 15:1 X vs. 32:1 X	-1.824	-1.985 to -1.663	Yes
16548 15:1 X vs. 32:10 X	-1.680	-1.824 to -1.536	Yes
16549 15:1 X vs. 32:40 X	-0.5063	-0.6503 to -0.3624	Yes
16550 15:1 X vs. 32:50 X	-0.3110	-0.4550 to -0.1670	Yes
16551 15:1 X vs. 33:0 X	-2.372	-2.516 to -2.228	Yes
16552 15:1 X vs. 33:1 X	-2.187	-2.331 to -2.043	Yes
16553 15:1 X vs. 33:10 X	-1.847	-1.991 to -1.703	Yes
16554 15:1 X vs. 33:40 X	-0.5250	-0.6690 to -0.3810	Yes
16555 15:1 X vs. 33:50 X	-0.3260	-0.4700 to -0.1820	Yes
16556 15:1 X vs. 34:0 X	-2.297	-2.441 to -2.153	Yes
16557 15:1 X vs. 34:1 X	-2.304	-2.448 to -2.160	Yes
16558 15:1 X vs. 34:10 X	-1.937	-2.081 to -1.793	Yes
16559 15:1 X vs. 34:40 X	-0.5370	-0.6810 to -0.3930	Yes
16560 15:1 X vs. 34:50 X	-0.4750	-0.6190 to -0.3310	Yes

2way ANOVA<br>Multiple comparisons		A<br>Data Set-A<br>Y	B<br>Data Set-B<br>Y	C<br>Data Set-C<br>Y
16561	15:1 X vs. 35:0 X	-2.645	-2.789 to -2.501	Yes
16562	15:1 X vs. 35:1 X	-2.411	-2.555 to -2.267	Yes
16563	15:1 X vs. 35:10 X	-1.899	-2.043 to -1.755	Yes
16564	15:1 X vs. 35:40 X	-0.5460	-0.6900 to -0.4020	Yes
16565	15:1 X vs. 35:50 X	-0.4877	-0.6316 to -0.3437	Yes
16566	15:1 X vs. 36:0 X	-2.852	-3.013 to -2.691	Yes
16567	15:1 X vs. 36:1 X	-2.548	-2.692 to -2.404	Yes
16568	15:1 X vs. 36:10 X	-1.905	-2.049 to -1.761	Yes
16569	15:1 X vs. 36:40 X	-0.6013	-0.7453 to -0.4574	Yes
16570	15:1 X vs. 36:50 X	-0.4510	-0.5950 to -0.3070	Yes
16571	15:1 X vs. 37:0 X	-3.236	-3.380 to -3.092	Yes
16572	15:1 X vs. 37:1 X	-2.618	-2.762 to -2.474	Yes
16573	15:1 X vs. 37:10 X	-1.906	-2.050 to -1.762	Yes
16574	15:1 X vs. 37:40 X	-0.6387	-0.7826 to -0.4947	Yes
16575	15:1 X vs. 37:50 X	-0.4597	-0.6036 to -0.3157	Yes
16576	15:1 X vs. 38:0 X	-3.181	-3.325 to -3.037	Yes
16577	15:1 X vs. 38:1 X	-2.736	-2.880 to -2.592	Yes
16578	15:1 X vs. 38:10 X	-2.182	-2.326 to -2.038	Yes
16579	15:1 X vs. 38:40 X	-0.6430	-0.7870 to -0.4990	Yes
16580	15:1 X vs. 38:50 X	-0.4637	-0.6076 to -0.3197	Yes
16581	15:1 X vs. 39:0 X	-3.411	-3.555 to -3.267	Yes
16582	15:1 X vs. 39:1 X	-3.303	-3.447 to -3.159	Yes
16583	15:1 X vs. 39:10 X	-2.327	-2.471 to -2.183	Yes
16584	15:1 X vs. 39:40 X	-0.6490	-0.7930 to -0.5050	Yes
16585	15:1 X vs. 39:50 X	-0.4947	-0.6386 to -0.3507	Yes
16586	15:1 X vs. 40:0 X	-3.452	-3.596 to -3.308	Yes
16587	15:1 X vs. 40:1 X	-3.378	-3.522 to -3.234	Yes
16588	15:1 X vs. 40:10 X	-2.583	-2.727 to -2.439	Yes
16589	15:1 X vs. 40:40 X	-0.6537	-0.7976 to -0.5097	Yes
16590	15:1 X vs. 40:50 X	-0.5010	-0.6450 to -0.3570	Yes
16591	15:1 X vs. 41:0 X	-3.493	-3.637 to -3.349	Yes
16592	15:1 X vs. 41:1 X	-3.492	-3.636 to -3.348	Yes
16593	15:1 X vs. 41:10 X	-2.637	-2.781 to -2.493	Yes
16594	15:1 X vs. 41:40 X	-0.6010	-0.7450 to -0.4570	Yes
16595	15:1 X vs. 41:50 X	-0.5137	-0.6576 to -0.3697	Yes
16596	15:1 X vs. 42:0 X	-3.635	-3.779 to -3.491	Yes
16597	15:1 X vs. 42:1 X	-3.655	-3.799 to -3.511	Yes
16598	15:1 X vs. 42:10 X	-2.637	-2.781 to -2.493	Yes
16599	15:1 X vs. 42:40 X	-0.6137	-0.7576 to -0.4697	Yes
16600	15:1 X vs. 42:50 X	-0.5170	-0.6610 to -0.3730	Yes
16601	15:1 X vs. 43:0 X	-3.725	-3.869 to -3.581	Yes
16602	15:1 X vs. 43:1 X	-3.639	-3.783 to -3.495	Yes
16603	15:1 X vs. 43:10 X	-3.355	-3.499 to -3.211	Yes
16604	15:1 X vs. 43:40 X	-0.6120	-0.7560 to -0.4680	Yes
16605	15:1 X vs. 43:50 X	-0.5173	-0.6613 to -0.3734	Yes

2way ANOVA<br>Multiple comparisons		A<br>Data Set-A<br>Y	B<br>Data Set-B<br>Y	C<br>Data Set-C<br>Y
16606	15:**1 X** vs. 44:**0 X**	-3.749	-3.893 to -3.605	Yes
16607	15:**1 X** vs. 44:**1 X**	-3.639	-3.783 to -3.495	Yes
16608	15:**1 X** vs. 44:**10 X**	-3.418	-3.562 to -3.274	Yes
16609	15:**1 X** vs. 44:**40 X**	-0.6140	-0.7580 to -0.4700	Yes
16610	15:**1 X** vs. 44:**50 X**	-0.4497	-0.5936 to -0.3057	Yes
16611	15:**1 X** vs. 45:**0 X**	-3.614	-3.758 to -3.470	Yes
16612	15:**1 X** vs. 45:**1 X**	-3.416	-3.560 to -3.272	Yes
16613	15:**1 X** vs. 45:**10 X**	-3.298	-3.442 to -3.154	Yes
16614	15:**1 X** vs. 45:**40 X**	-0.6153	-0.7593 to -0.4714	Yes
16615	15:**1 X** vs. 45:**50 X**	-0.4467	-0.5906 to -0.3027	Yes
16616	15:**1 X** vs. 46:**0 X**	-3.418	-3.562 to -3.274	Yes
16617	15:**1 X** vs. 46:**1 X**	-3.385	-3.529 to -3.241	Yes
16618	15:**1 X** vs. 46:**10 X**	-3.293	-3.437 to -3.149	Yes
16619	15:**1 X** vs. 46:**40 X**	-0.6290	-0.7730 to -0.4850	Yes
16620	15:**1 X** vs. 46:**50 X**	-0.4397	-0.5836 to -0.2957	Yes
16621	15:**1 X** vs. 47:**0 X**	-3.394	-3.538 to -3.250	Yes
16622	15:**1 X** vs. 47:**1 X**	-3.298	-3.442 to -3.154	Yes
16623	15:**1 X** vs. 47:**10 X**	-3.279	-3.423 to -3.135	Yes
16624	15:**1 X** vs. 47:**40 X**	-0.6390	-0.7830 to -0.4950	Yes
16625	15:**1 X** vs. 47:**50 X**	-0.3977	-0.5416 to -0.2537	Yes
16626	15:**1 X** vs. 48:**0 X**	-3.381	-3.542 to -3.220	Yes
16627	15:**1 X** vs. 48:**1 X**	-3.285	-3.429 to -3.141	Yes
16628	15:**1 X** vs. 48:**10 X**	-3.279	-3.423 to -3.135	Yes
16629	15:**1 X** vs. 48:**40 X**	-0.6453	-0.7893 to -0.5014	Yes
16630	15:**1 X** vs. 48:**50 X**	-0.4050	-0.5490 to -0.2610	Yes
16631	15:**1 X** vs. 49:**0 X**	-3.381	-3.525 to -3.237	Yes
16632	15:**1 X** vs. 49:**1 X**	-3.285	-3.429 to -3.141	Yes
16633	15:**1 X** vs. 49:**10 X**	-3.279	-3.423 to -3.135	Yes
16634	15:**1 X** vs. 49:**40 X**	-0.6497	-0.7936 to -0.5057	Yes
16635	15:**1 X** vs. 49:**50 X**	-0.4043	-0.5483 to -0.2604	Yes
16636	15:**1 X** vs. 50:**0 X**	-3.381	-3.525 to -3.237	Yes
16637	15:**1 X** vs. 50:**1 X**	-3.285	-3.429 to -3.141	Yes
16638	15:**1 X** vs. 50:**10 X**	-3.279	-3.423 to -3.135	Yes
16639	15:**1 X** vs. 50:**40 X**	-0.6573	-0.8013 to -0.5134	Yes
16640	15:**1 X** vs. 50:**50 X**	-0.4040	-0.5480 to -0.2600	Yes
16641	15:**10 X** vs. 15:**40 X**	0.01767	-0.1263 to 0.1616	No
16642	15:**10 X** vs. 15:**50 X**	0.0	-0.1440 to 0.1440	No
16643	15:**10 X** vs. 16:**0 X**	-0.0850	-0.2290 to 0.05896	No
16644	15:**10 X** vs. 16:**1 X**	0.0240	-0.1200 to 0.1680	No
16645	15:**10 X** vs. 16:**10 X**	-0.001667	-0.1456 to 0.1423	No
16646	15:**10 X** vs. 16:**40 X**	-0.0210	-0.1650 to 0.1230	No
16647	15:**10 X** vs. 16:**50 X**	-0.06333	-0.2073 to 0.08063	No
16648	15:**10 X** vs. 17:**0 X**	-0.1377	-0.2816 to 0.006293	No
16649	15:**10 X** vs. 17:**1 X**	-0.0520	-0.1960 to 0.09196	No
16650	15:**10 X** vs. 17:**10 X**	-0.1087	-0.2526 to 0.03529	No

2way ANOVA<br>Multiple comparisons		A<br>Data Set-A<br>Y	B<br>Data Set-B<br>Y	C<br>Data Set-C<br>Y
16651	15:**10 X** vs. 17:**40 X**	-0.0320	-0.1760 to 0.1120	No
16652	15:**10 X** vs. 17:**50 X**	-0.07733	-0.2213 to 0.06663	No
16653	15:**10 X** vs. 18:**0 X**	-0.1863	-0.3303 to -0.04237	Yes
16654	15:**10 X** vs. 18:**1 X**	-0.09467	-0.2386 to 0.04929	No
16655	15:**10 X** vs. 18:**10 X**	-0.1870	-0.3310 to -0.04304	Yes
16656	15:**10 X** vs. 18:**40 X**	-0.03633	-0.1803 to 0.1076	No
16657	15:**10 X** vs. 18:**50 X**	-0.08133	-0.2253 to 0.06263	No
16658	15:**10 X** vs. 19:**0 X**	-0.2213	-0.3653 to -0.07737	Yes
16659	15:**10 X** vs. 19:**1 X**	-0.2093	-0.3533 to -0.06537	Yes
16660	15:**10 X** vs. 19:**10 X**	-0.2070	-0.3510 to -0.06304	Yes
16661	15:**10 X** vs. 19:**40 X**	-0.0650	-0.2090 to 0.07896	No
16662	15:**10 X** vs. 19:**50 X**	-0.0760	-0.2200 to 0.06796	No
16663	15:**10 X** vs. 20:**0 X**	-0.3010	-0.4450 to -0.1570	Yes
16664	15:**10 X** vs. 20:**1 X**	-0.1203	-0.2643 to 0.02363	No
16665	15:**10 X** vs. 20:**10 X**	-0.2093	-0.3533 to -0.06537	Yes
16666	15:**10 X** vs. 20:**40 X**	-0.07667	-0.2206 to 0.06729	No
16667	15:**10 X** vs. 20:**50 X**	-0.1207	-0.2646 to 0.02329	No
16668	15:**10 X** vs. 21:**0 X**	-0.3243	-0.4683 to -0.1804	Yes
16669	15:**10 X** vs. 21:**1 X**	-0.2773	-0.4213 to -0.1334	Yes
16670	15:**10 X** vs. 21:**10 X**	-0.2743	-0.4183 to -0.1304	Yes
16671	15:**10 X** vs. 21:**40 X**	-0.1497	-0.2936 to -0.005707	Yes
16672	15:**10 X** vs. 21:**50 X**	-0.2400	-0.3840 to -0.09604	Yes
16673	15:**10 X** vs. 22:**0 X**	-0.3660	-0.5100 to -0.2220	Yes
16674	15:**10 X** vs. 22:**1 X**	-0.3607	-0.5046 to -0.2167	Yes
16675	15:**10 X** vs. 22:**10 X**	-0.3560	-0.5000 to -0.2120	Yes
16676	15:**10 X** vs. 22:**40 X**	-0.2563	-0.4003 to -0.1124	Yes
16677	15:**10 X** vs. 22:**50 X**	-0.3190	-0.4630 to -0.1750	Yes
16678	15:**10 X** vs. 23:**0 X**	-0.4313	-0.5753 to -0.2874	Yes
16679	15:**10 X** vs. 23:**1 X**	-0.6210	-0.7820 to -0.4600	Yes
16680	15:**10 X** vs. 23:**10 X**	-0.3707	-0.5146 to -0.2267	Yes
16681	15:**10 X** vs. 23:**40 X**	-0.2510	-0.4120 to -0.09005	Yes
16682	15:**10 X** vs. 23:**50 X**	-0.2850	-0.4290 to -0.1410	Yes
16683	15:**10 X** vs. 24:**0 X**	-0.5487	-0.6926 to -0.4047	Yes
16684	15:**10 X** vs. 24:**1 X**	-0.7050	-0.8660 to -0.5440	Yes
16685	15:**10 X** vs. 24:**10 X**	-0.5325	-0.6935 to -0.3715	Yes
16686	15:**10 X** vs. 24:**40 X**	-0.2673	-0.4113 to -0.1234	Yes
16687	15:**10 X** vs. 24:**50 X**	-0.1890	-0.3330 to -0.04504	Yes
16688	15:**10 X** vs. 25:**0 X**	-0.8860	-1.047 to -0.7250	Yes
16689	15:**10 X** vs. 25:**1 X**	-1.076	-1.236 to -0.9145	Yes
16690	15:**10 X** vs. 25:**10 X**	-0.4370	-0.6406 to -0.2334	Yes
16691	15:**10 X** vs. 25:**40 X**	-0.2693	-0.4133 to -0.1254	Yes
16692	15:**10 X** vs. 25:**50 X**	-0.2437	-0.3876 to -0.09971	Yes
16693	15:**10 X** vs. 26:**0 X**	-1.099	-1.259 to -0.9375	Yes
16694	15:**10 X** vs. 26:**1 X**	-1.212	-1.372 to -1.051	Yes
16695	15:**10 X** vs. 26:**10 X**	-0.8980	-1.059 to -0.7370	Yes

2way ANOVA Multiple comparisons	A Data Set-A Y	B Data Set-B Y	C Data Set-C Y
16696 15:**10 X** vs. 26:**40 X**	-0.3437	-0.4876 to -0.1997	Yes
16697 15:**10 X** vs. 26:**50 X**	-0.2040	-0.3480 to -0.06004	Yes
16698 15:**10 X** vs. 27:**0 X**	-1.286	-1.430 to -1.142	Yes
16699 15:**10 X** vs. 27:**1 X**	-1.377	-1.538 to -1.216	Yes
16700 15:**10 X** vs. 27:**10 X**	-1.189	-1.350 to -1.028	Yes
16701 15:**10 X** vs. 27:**40 X**	-0.3437	-0.4876 to -0.1997	Yes
16702 15:**10 X** vs. 27:**50 X**	-0.2033	-0.3473 to -0.05937	Yes
16703 15:**10 X** vs. 28:**0 X**	-1.236	-1.396 to -1.075	Yes
16704 15:**10 X** vs. 28:**1 X**	-1.276	-1.420 to -1.132	Yes
16705 15:**10 X** vs. 28:**10 X**	-1.078	-1.239 to -0.9170	Yes
16706 15:**10 X** vs. 28:**40 X**	-0.3560	-0.5000 to -0.2120	Yes
16707 15:**10 X** vs. 28:**50 X**	-0.2513	-0.3953 to -0.1074	Yes
16708 15:**10 X** vs. 29:**0 X**	-1.717	-1.878 to -1.556	Yes
16709 15:**10 X** vs. 29:**1 X**	-1.802	-1.963 to -1.641	Yes
16710 15:**10 X** vs. 29:**10 X**	-1.017	-1.177 to -0.8555	Yes
16711 15:**10 X** vs. 29:**40 X**	-0.4640	-0.6080 to -0.3200	Yes
16712 15:**10 X** vs. 29:**50 X**	-0.2527	-0.3966 to -0.1087	Yes
16713 15:**10 X** vs. 30:**0 X**	-1.432	-1.592 to -1.271	Yes
16714 15:**10 X** vs. 30:**1 X**	-1.753	-1.914 to -1.592	Yes
16715 15:**10 X** vs. 30:**10 X**	-1.091	-1.252 to -0.9300	Yes
16716 15:**10 X** vs. 30:**40 X**	-0.3910	-0.5350 to -0.2470	Yes
16717 15:**10 X** vs. 30:**50 X**	-0.3050	-0.4490 to -0.1610	Yes
16718 15:**10 X** vs. 31:**0 X**	-1.750	-1.910 to -1.589	Yes
16719 15:**10 X** vs. 31:**1 X**	-1.740	-1.901 to -1.579	Yes
16720 15:**10 X** vs. 31:**10 X**	-1.223	-1.383 to -1.062	Yes
16721 15:**10 X** vs. 31:**40 X**	-0.4827	-0.6266 to -0.3387	Yes
16722 15:**10 X** vs. 31:**50 X**	-0.2840	-0.4280 to -0.1400	Yes
16723 15:**10 X** vs. 32:**0 X**	-2.341	-2.485 to -2.197	Yes
16724 15:**10 X** vs. 32:**1 X**	-1.797	-1.958 to -1.636	Yes
16725 15:**10 X** vs. 32:**10 X**	-1.653	-1.797 to -1.509	Yes
16726 15:**10 X** vs. 32:**40 X**	-0.4790	-0.6230 to -0.3350	Yes
16727 15:**10 X** vs. 32:**50 X**	-0.2837	-0.4276 to -0.1397	Yes
16728 15:**10 X** vs. 33:**0 X**	-2.345	-2.489 to -2.201	Yes
16729 15:**10 X** vs. 33:**1 X**	-2.159	-2.303 to -2.015	Yes
16730 15:**10 X** vs. 33:**10 X**	-1.820	-1.964 to -1.676	Yes
16731 15:**10 X** vs. 33:**40 X**	-0.4977	-0.6416 to -0.3537	Yes
16732 15:**10 X** vs. 33:**50 X**	-0.2987	-0.4426 to -0.1547	Yes
16733 15:**10 X** vs. 34:**0 X**	-2.269	-2.413 to -2.125	Yes
16734 15:**10 X** vs. 34:**1 X**	-2.277	-2.421 to -2.133	Yes
16735 15:**10 X** vs. 34:**10 X**	-1.910	-2.054 to -1.766	Yes
16736 15:**10 X** vs. 34:**40 X**	-0.5097	-0.6536 to -0.3657	Yes
16737 15:**10 X** vs. 34:**50 X**	-0.4477	-0.5916 to -0.3037	Yes
16738 15:**10 X** vs. 35:**0 X**	-2.618	-2.762 to -2.474	Yes
16739 15:**10 X** vs. 35:**1 X**	-2.384	-2.528 to -2.240	Yes
16740 15:**10 X** vs. 35:**10 X**	-1.872	-2.016 to -1.728	Yes

	2way ANOVA<br>Multiple comparisons	A<br>Data Set-A<br>Y	B<br>Data Set-B<br>Y	C<br>Data Set-C<br>Y
16741	15:10 X vs. 35:40 X	-0.5187	-0.6626 to -0.3747	Yes
16742	15:10 X vs. 35:50 X	-0.4603	-0.6043 to -0.3164	Yes
16743	15:10 X vs. 36:0 X	-2.825	-2.985 to -2.664	Yes
16744	15:10 X vs. 36:1 X	-2.520	-2.664 to -2.376	Yes
16745	15:10 X vs. 36:10 X	-1.877	-2.021 to -1.733	Yes
16746	15:10 X vs. 36:40 X	-0.5740	-0.7180 to -0.4300	Yes
16747	15:10 X vs. 36:50 X	-0.4237	-0.5676 to -0.2797	Yes
16748	15:10 X vs. 37:0 X	-3.208	-3.352 to -3.064	Yes
16749	15:10 X vs. 37:1 X	-2.590	-2.734 to -2.446	Yes
16750	15:10 X vs. 37:10 X	-1.879	-2.023 to -1.735	Yes
16751	15:10 X vs. 37:40 X	-0.6113	-0.7553 to -0.4674	Yes
16752	15:10 X vs. 37:50 X	-0.4323	-0.5763 to -0.2884	Yes
16753	15:10 X vs. 38:0 X	-3.153	-3.297 to -3.009	Yes
16754	15:10 X vs. 38:1 X	-2.709	-2.853 to -2.565	Yes
16755	15:10 X vs. 38:10 X	-2.154	-2.298 to -2.010	Yes
16756	15:10 X vs. 38:40 X	-0.6157	-0.7596 to -0.4717	Yes
16757	15:10 X vs. 38:50 X	-0.4363	-0.5803 to -0.2924	Yes
16758	15:10 X vs. 39:0 X	-3.384	-3.528 to -3.240	Yes
16759	15:10 X vs. 39:1 X	-3.276	-3.420 to -3.132	Yes
16760	15:10 X vs. 39:10 X	-2.299	-2.443 to -2.155	Yes
16761	15:10 X vs. 39:40 X	-0.6217	-0.7656 to -0.4777	Yes
16762	15:10 X vs. 39:50 X	-0.4673	-0.6113 to -0.3234	Yes
16763	15:10 X vs. 40:0 X	-3.425	-3.569 to -3.281	Yes
16764	15:10 X vs. 40:1 X	-3.351	-3.495 to -3.207	Yes
16765	15:10 X vs. 40:10 X	-2.556	-2.700 to -2.412	Yes
16766	15:10 X vs. 40:40 X	-0.6263	-0.7703 to -0.4824	Yes
16767	15:10 X vs. 40:50 X	-0.4737	-0.6176 to -0.3297	Yes
16768	15:10 X vs. 41:0 X	-3.466	-3.610 to -3.322	Yes
16769	15:10 X vs. 41:1 X	-3.465	-3.609 to -3.321	Yes
16770	15:10 X vs. 41:10 X	-2.609	-2.753 to -2.465	Yes
16771	15:10 X vs. 41:40 X	-0.5737	-0.7176 to -0.4297	Yes
16772	15:10 X vs. 41:50 X	-0.4863	-0.6303 to -0.3424	Yes
16773	15:10 X vs. 42:0 X	-3.608	-3.752 to -3.464	Yes
16774	15:10 X vs. 42:1 X	-3.627	-3.771 to -3.483	Yes
16775	15:10 X vs. 42:10 X	-2.610	-2.754 to -2.466	Yes
16776	15:10 X vs. 42:40 X	-0.5863	-0.7303 to -0.4424	Yes
16777	15:10 X vs. 42:50 X	-0.4897	-0.6336 to -0.3457	Yes
16778	15:10 X vs. 43:0 X	-3.698	-3.842 to -3.554	Yes
16779	15:10 X vs. 43:1 X	-3.612	-3.756 to -3.468	Yes
16780	15:10 X vs. 43:10 X	-3.328	-3.472 to -3.184	Yes
16781	15:10 X vs. 43:40 X	-0.5847	-0.7286 to -0.4407	Yes
16782	15:10 X vs. 43:50 X	-0.4900	-0.6340 to -0.3460	Yes
16783	15:10 X vs. 44:0 X	-3.722	-3.866 to -3.578	Yes
16784	15:10 X vs. 44:1 X	-3.611	-3.755 to -3.467	Yes
16785	15:10 X vs. 44:10 X	-3.391	-3.535 to -3.247	Yes

	2way ANOVA<br>Multiple comparisons	A<br>Data Set-A<br>Y	B<br>Data Set-B<br>Y	C<br>Data Set-C<br>Y
16786	15:**10** **X** vs. 44:**40** **X**	-0.5867	-0.7306 to -0.4427	Yes
16787	15:**10** **X** vs. 44:**50** **X**	-0.4223	-0.5663 to -0.2784	Yes
16788	15:**10** **X** vs. 45:**0** **X**	-3.586	-3.730 to -3.442	Yes
16789	15:**10** **X** vs. 45:**1** **X**	-3.388	-3.532 to -3.244	Yes
16790	15:**10** **X** vs. 45:**10** **X**	-3.271	-3.415 to -3.127	Yes
16791	15:**10** **X** vs. 45:**40** **X**	-0.5880	-0.7320 to -0.4440	Yes
16792	15:**10** **X** vs. 45:**50** **X**	-0.4193	-0.5633 to -0.2754	Yes
16793	15:**10** **X** vs. 46:**0** **X**	-3.391	-3.535 to -3.247	Yes
16794	15:**10** **X** vs. 46:**1** **X**	-3.358	-3.502 to -3.214	Yes
16795	15:**10** **X** vs. 46:**10** **X**	-3.266	-3.410 to -3.122	Yes
16796	15:**10** **X** vs. 46:**40** **X**	-0.6017	-0.7456 to -0.4577	Yes
16797	15:**10** **X** vs. 46:**50** **X**	-0.4123	-0.5563 to -0.2684	Yes
16798	15:**10** **X** vs. 47:**0** **X**	-3.366	-3.510 to -3.222	Yes
16799	15:**10** **X** vs. 47:**1** **X**	-3.270	-3.414 to -3.126	Yes
16800	15:**10** **X** vs. 47:**10** **X**	-3.252	-3.396 to -3.108	Yes
16801	15:**10** **X** vs. 47:**40** **X**	-0.6117	-0.7556 to -0.4677	Yes
16802	15:**10** **X** vs. 47:**50** **X**	-0.3703	-0.5143 to -0.2264	Yes
16803	15:**10** **X** vs. 48:**0** **X**	-3.354	-3.514 to -3.193	Yes
16804	15:**10** **X** vs. 48:**1** **X**	-3.257	-3.401 to -3.113	Yes
16805	15:**10** **X** vs. 48:**10** **X**	-3.252	-3.396 to -3.108	Yes
16806	15:**10** **X** vs. 48:**40** **X**	-0.6180	-0.7620 to -0.4740	Yes
16807	15:**10** **X** vs. 48:**50** **X**	-0.3777	-0.5216 to -0.2337	Yes
16808	15:**10** **X** vs. 49:**0** **X**	-3.354	-3.498 to -3.210	Yes
16809	15:**10** **X** vs. 49:**1** **X**	-3.257	-3.401 to -3.113	Yes
16810	15:**10** **X** vs. 49:**10** **X**	-3.252	-3.396 to -3.108	Yes
16811	15:**10** **X** vs. 49:**40** **X**	-0.6223	-0.7663 to -0.4784	Yes
16812	15:**10** **X** vs. 49:**50** **X**	-0.3770	-0.5210 to -0.2330	Yes
16813	15:**10** **X** vs. 50:**0** **X**	-3.354	-3.498 to -3.210	Yes
16814	15:**10** **X** vs. 50:**1** **X**	-3.257	-3.401 to -3.113	Yes
16815	15:**10** **X** vs. 50:**10** **X**	-3.252	-3.396 to -3.108	Yes
16816	15:**10** **X** vs. 50:**40** **X**	-0.6300	-0.7740 to -0.4860	Yes
16817	15:**10** **X** vs. 50:**50** **X**	-0.3767	-0.5206 to -0.2327	Yes
16818	15:**40** **X** vs. 15:**50** **X**	-0.01767	-0.1616 to 0.1263	No
16819	15:**40** **X** vs. 16:**0** **X**	-0.1027	-0.2466 to 0.04129	No
16820	15:**40** **X** vs. 16:**1** **X**	0.006333	-0.1376 to 0.1503	No
16821	15:**40** **X** vs. 16:**10** **X**	-0.01933	-0.1633 to 0.1246	No
16822	15:**40** **X** vs. 16:**40** **X**	-0.03867	-0.1826 to 0.1053	No
16823	15:**40** **X** vs. 16:**50** **X**	-0.08100	-0.2250 to 0.06296	No
16824	15:**40** **X** vs. 17:**0** **X**	-0.1553	-0.2993 to -0.01137	Yes
16825	15:**40** **X** vs. 17:**1** **X**	-0.06967	-0.2136 to 0.07429	No
16826	15:**40** **X** vs. 17:**10** **X**	-0.1263	-0.2703 to 0.01763	No
16827	15:**40** **X** vs. 17:**40** **X**	-0.04967	-0.1936 to 0.09429	No
16828	15:**40** **X** vs. 17:**50** **X**	-0.0950	-0.2390 to 0.04896	No
16829	15:**40** **X** vs. 18:**0** **X**	-0.2040	-0.3480 to -0.06004	Yes
16830	15:**40** **X** vs. 18:**1** **X**	-0.1123	-0.2563 to 0.03163	No

	2way ANOVA<br>Multiple comparisons	A<br>Data Set-A<br>Y	B<br>Data Set-B<br>Y	C<br>Data Set-C<br>Y
16831	15:40 X vs. 18:10 X	-0.2047	-0.3486 to -0.06071	Yes
16832	15:40 X vs. 18:40 X	-0.0540	-0.1980 to 0.08996	No
16833	15:40 X vs. 18:50 X	-0.0990	-0.2430 to 0.04496	No
16834	15:40 X vs. 19:0 X	-0.2390	-0.3830 to -0.09504	Yes
16835	15:40 X vs. 19:1 X	-0.2270	-0.3710 to -0.08304	Yes
16836	15:40 X vs. 19:10 X	-0.2247	-0.3686 to -0.08071	Yes
16837	15:40 X vs. 19:40 X	-0.08267	-0.2266 to 0.06129	No
16838	15:40 X vs. 19:50 X	-0.09367	-0.2376 to 0.05029	No
16839	15:40 X vs. 20:0 X	-0.3187	-0.4626 to -0.1747	Yes
16840	15:40 X vs. 20:1 X	-0.1380	-0.2820 to 0.005960	No
16841	15:40 X vs. 20:10 X	-0.2270	-0.3710 to -0.08304	Yes
16842	15:40 X vs. 20:40 X	-0.09433	-0.2383 to 0.04963	No
16843	15:40 X vs. 20:50 X	-0.1383	-0.2823 to 0.005626	No
16844	15:40 X vs. 21:0 X	-0.3420	-0.4860 to -0.1980	Yes
16845	15:40 X vs. 21:1 X	-0.2950	-0.4390 to -0.1510	Yes
16846	15:40 X vs. 21:10 X	-0.2920	-0.4360 to -0.1480	Yes
16847	15:40 X vs. 21:40 X	-0.1673	-0.3113 to -0.02337	Yes
16848	15:40 X vs. 21:50 X	-0.2577	-0.4016 to -0.1137	Yes
16849	15:40 X vs. 22:0 X	-0.3837	-0.5276 to -0.2397	Yes
16850	15:40 X vs. 22:1 X	-0.3783	-0.5223 to -0.2344	Yes
16851	15:40 X vs. 22:10 X	-0.3737	-0.5176 to -0.2297	Yes
16852	15:40 X vs. 22:40 X	-0.2740	-0.4180 to -0.1300	Yes
16853	15:40 X vs. 22:50 X	-0.3367	-0.4806 to -0.1927	Yes
16854	15:40 X vs. 23:0 X	-0.4490	-0.5930 to -0.3050	Yes
16855	15:40 X vs. 23:1 X	-0.6387	-0.7996 to -0.4777	Yes
16856	15:40 X vs. 23:10 X	-0.3883	-0.5323 to -0.2444	Yes
16857	15:40 X vs. 23:40 X	-0.2687	-0.4296 to -0.1077	Yes
16858	15:40 X vs. 23:50 X	-0.3027	-0.4466 to -0.1587	Yes
16859	15:40 X vs. 24:0 X	-0.5663	-0.7103 to -0.4224	Yes
16860	15:40 X vs. 24:1 X	-0.7227	-0.8836 to -0.5617	Yes
16861	15:40 X vs. 24:10 X	-0.5502	-0.7111 to -0.3892	Yes
16862	15:40 X vs. 24:40 X	-0.2850	-0.4290 to -0.1410	Yes
16863	15:40 X vs. 24:50 X	-0.2067	-0.3506 to -0.06271	Yes
16864	15:40 X vs. 25:0 X	-0.9037	-1.065 to -0.7427	Yes
16865	15:40 X vs. 25:1 X	-1.093	-1.254 to -0.9322	Yes
16866	15:40 X vs. 25:10 X	-0.4547	-0.6583 to -0.2511	Yes
16867	15:40 X vs. 25:40 X	-0.2870	-0.4310 to -0.1430	Yes
16868	15:40 X vs. 25:50 X	-0.2613	-0.4053 to -0.1174	Yes
16869	15:40 X vs. 26:0 X	-1.116	-1.277 to -0.9552	Yes
16870	15:40 X vs. 26:1 X	-1.229	-1.390 to -1.068	Yes
16871	15:40 X vs. 26:10 X	-0.9157	-1.077 to -0.7547	Yes
16872	15:40 X vs. 26:40 X	-0.3613	-0.5053 to -0.2174	Yes
16873	15:40 X vs. 26:50 X	-0.2217	-0.3656 to -0.07771	Yes
16874	15:40 X vs. 27:0 X	-1.304	-1.448 to -1.160	Yes
16875	15:40 X vs. 27:1 X	-1.395	-1.556 to -1.234	Yes

2way ANOVA<br>Multiple comparisons	A<br>Data Set-A<br>Y	B<br>Data Set-B<br>Y	C<br>Data Set-C<br>Y
16876 15:40 X vs. 27:10 X	-1.207	-1.368 to -1.046	Yes
16877 15:40 X vs. 27:40 X	-0.3613	-0.5053 to -0.2174	Yes
16878 15:40 X vs. 27:50 X	-0.2210	-0.3650 to -0.07704	Yes
16879 15:40 X vs. 28:0 X	-1.253	-1.414 to -1.092	Yes
16880 15:40 X vs. 28:1 X	-1.294	-1.438 to -1.150	Yes
16881 15:40 X vs. 28:10 X	-1.096	-1.257 to -0.9347	Yes
16882 15:40 X vs. 28:40 X	-0.3737	-0.5176 to -0.2297	Yes
16883 15:40 X vs. 28:50 X	-0.2690	-0.4130 to -0.1250	Yes
16884 15:40 X vs. 29:0 X	-1.735	-1.896 to -1.574	Yes
16885 15:40 X vs. 29:1 X	-1.820	-1.981 to -1.659	Yes
16886 15:40 X vs. 29:10 X	-1.034	-1.195 to -0.8732	Yes
16887 15:40 X vs. 29:40 X	-0.4817	-0.6256 to -0.3377	Yes
16888 15:40 X vs. 29:50 X	-0.2703	-0.4143 to -0.1264	Yes
16889 15:40 X vs. 30:0 X	-1.449	-1.610 to -1.288	Yes
16890 15:40 X vs. 30:1 X	-1.771	-1.932 to -1.610	Yes
16891 15:40 X vs. 30:10 X	-1.109	-1.270 to -0.9477	Yes
16892 15:40 X vs. 30:40 X	-0.4087	-0.5526 to -0.2647	Yes
16893 15:40 X vs. 30:50 X	-0.3227	-0.4666 to -0.1787	Yes
16894 15:40 X vs. 31:0 X	-1.767	-1.928 to -1.606	Yes
16895 15:40 X vs. 31:1 X	-1.758	-1.919 to -1.597	Yes
16896 15:40 X vs. 31:10 X	-1.240	-1.401 to -1.079	Yes
16897 15:40 X vs. 31:40 X	-0.5003	-0.6443 to -0.3564	Yes
16898 15:40 X vs. 31:50 X	-0.3017	-0.4456 to -0.1577	Yes
16899 15:40 X vs. 32:0 X	-2.359	-2.503 to -2.215	Yes
16900 15:40 X vs. 32:1 X	-1.815	-1.976 to -1.654	Yes
16901 15:40 X vs. 32:10 X	-1.671	-1.815 to -1.527	Yes
16902 15:40 X vs. 32:40 X	-0.4967	-0.6406 to -0.3527	Yes
16903 15:40 X vs. 32:50 X	-0.3013	-0.4453 to -0.1574	Yes
16904 15:40 X vs. 33:0 X	-2.362	-2.506 to -2.218	Yes
16905 15:40 X vs. 33:1 X	-2.177	-2.321 to -2.033	Yes
16906 15:40 X vs. 33:10 X	-1.837	-1.981 to -1.693	Yes
16907 15:40 X vs. 33:40 X	-0.5153	-0.6593 to -0.3714	Yes
16908 15:40 X vs. 33:50 X	-0.3163	-0.4603 to -0.1724	Yes
16909 15:40 X vs. 34:0 X	-2.287	-2.431 to -2.143	Yes
16910 15:40 X vs. 34:1 X	-2.294	-2.438 to -2.150	Yes
16911 15:40 X vs. 34:10 X	-1.928	-2.072 to -1.784	Yes
16912 15:40 X vs. 34:40 X	-0.5273	-0.6713 to -0.3834	Yes
16913 15:40 X vs. 34:50 X	-0.4653	-0.6093 to -0.3214	Yes
16914 15:40 X vs. 35:0 X	-2.636	-2.780 to -2.492	Yes
16915 15:40 X vs. 35:1 X	-2.401	-2.545 to -2.257	Yes
16916 15:40 X vs. 35:10 X	-1.889	-2.033 to -1.745	Yes
16917 15:40 X vs. 35:40 X	-0.5363	-0.6803 to -0.3924	Yes
16918 15:40 X vs. 35:50 X	-0.4780	-0.6220 to -0.3340	Yes
16919 15:40 X vs. 36:0 X	-2.842	-3.003 to -2.681	Yes
16920 15:40 X vs. 36:1 X	-2.538	-2.682 to -2.394	Yes

	2way ANOVA<br>Multiple comparisons	A<br>Data Set-A<br>Y	B<br>Data Set-B<br>Y	C<br>Data Set-C<br>Y
16921	15:40 X vs. 36:10 X	-1.895	-2.039 to -1.751	Yes
16922	15:40 X vs. 36:40 X	-0.5917	-0.7356 to -0.4477	Yes
16923	15:40 X vs. 36:50 X	-0.4413	-0.5853 to -0.2974	Yes
16924	15:40 X vs. 37:0 X	-3.226	-3.370 to -3.082	Yes
16925	15:40 X vs. 37:1 X	-2.608	-2.752 to -2.464	Yes
16926	15:40 X vs. 37:10 X	-1.897	-2.041 to -1.753	Yes
16927	15:40 X vs. 37:40 X	-0.6290	-0.7730 to -0.4850	Yes
16928	15:40 X vs. 37:50 X	-0.4500	-0.5940 to -0.3060	Yes
16929	15:40 X vs. 38:0 X	-3.171	-3.315 to -3.027	Yes
16930	15:40 X vs. 38:1 X	-2.727	-2.871 to -2.583	Yes
16931	15:40 X vs. 38:10 X	-2.172	-2.316 to -2.028	Yes
16932	15:40 X vs. 38:40 X	-0.6333	-0.7773 to -0.4894	Yes
16933	15:40 X vs. 38:50 X	-0.4540	-0.5980 to -0.3100	Yes
16934	15:40 X vs. 39:0 X	-3.402	-3.546 to -3.258	Yes
16935	15:40 X vs. 39:1 X	-3.293	-3.437 to -3.149	Yes
16936	15:40 X vs. 39:10 X	-2.317	-2.461 to -2.173	Yes
16937	15:40 X vs. 39:40 X	-0.6393	-0.7833 to -0.4954	Yes
16938	15:40 X vs. 39:50 X	-0.4850	-0.6290 to -0.3410	Yes
16939	15:40 X vs. 40:0 X	-3.443	-3.587 to -3.299	Yes
16940	15:40 X vs. 40:1 X	-3.369	-3.513 to -3.225	Yes
16941	15:40 X vs. 40:10 X	-2.573	-2.717 to -2.429	Yes
16942	15:40 X vs. 40:40 X	-0.6440	-0.7880 to -0.5000	Yes
16943	15:40 X vs. 40:50 X	-0.4913	-0.6353 to -0.3474	Yes
16944	15:40 X vs. 41:0 X	-3.484	-3.628 to -3.340	Yes
16945	15:40 X vs. 41:1 X	-3.483	-3.627 to -3.339	Yes
16946	15:40 X vs. 41:10 X	-2.627	-2.771 to -2.483	Yes
16947	15:40 X vs. 41:40 X	-0.5913	-0.7353 to -0.4474	Yes
16948	15:40 X vs. 41:50 X	-0.5040	-0.6480 to -0.3600	Yes
16949	15:40 X vs. 42:0 X	-3.625	-3.769 to -3.481	Yes
16950	15:40 X vs. 42:1 X	-3.645	-3.789 to -3.501	Yes
16951	15:40 X vs. 42:10 X	-2.627	-2.771 to -2.483	Yes
16952	15:40 X vs. 42:40 X	-0.6040	-0.7480 to -0.4600	Yes
16953	15:40 X vs. 42:50 X	-0.5073	-0.6513 to -0.3634	Yes
16954	15:40 X vs. 43:0 X	-3.715	-3.859 to -3.571	Yes
16955	15:40 X vs. 43:1 X	-3.630	-3.774 to -3.486	Yes
16956	15:40 X vs. 43:10 X	-3.345	-3.489 to -3.201	Yes
16957	15:40 X vs. 43:40 X	-0.6023	-0.7463 to -0.4584	Yes
16958	15:40 X vs. 43:50 X	-0.5077	-0.6516 to -0.3637	Yes
16959	15:40 X vs. 44:0 X	-3.739	-3.883 to -3.595	Yes
16960	15:40 X vs. 44:1 X	-3.629	-3.773 to -3.485	Yes
16961	15:40 X vs. 44:10 X	-3.409	-3.553 to -3.265	Yes
16962	15:40 X vs. 44:40 X	-0.6043	-0.7483 to -0.4604	Yes
16963	15:40 X vs. 44:50 X	-0.4400	-0.5840 to -0.2960	Yes
16964	15:40 X vs. 45:0 X	-3.604	-3.748 to -3.460	Yes
16965	15:40 X vs. 45:1 X	-3.406	-3.550 to -3.262	Yes

	2way ANOVA<br>Multiple comparisons	A<br>Data Set-A<br>Y	B<br>Data Set-B<br>Y	C<br>Data Set-C<br>Y
16966	15:**40 X** vs. 45:**10 X**	-3.288	-3.432 to -3.144	Yes
16967	15:**40 X** vs. 45:**40 X**	-0.6057	-0.7496 to -0.4617	Yes
16968	15:**40 X** vs. 45:**50 X**	-0.4370	-0.5810 to -0.2930	Yes
16969	15:**40 X** vs. 46:**0 X**	-3.408	-3.552 to -3.264	Yes
16970	15:**40 X** vs. 46:**1 X**	-3.375	-3.519 to -3.231	Yes
16971	15:**40 X** vs. 46:**10 X**	-3.283	-3.427 to -3.139	Yes
16972	15:**40 X** vs. 46:**40 X**	-0.6193	-0.7633 to -0.4754	Yes
16973	15:**40 X** vs. 46:**50 X**	-0.4300	-0.5740 to -0.2860	Yes
16974	15:**40 X** vs. 47:**0 X**	-3.384	-3.528 to -3.240	Yes
16975	15:**40 X** vs. 47:**1 X**	-3.288	-3.432 to -3.144	Yes
16976	15:**40 X** vs. 47:**10 X**	-3.269	-3.413 to -3.125	Yes
16977	15:**40 X** vs. 47:**40 X**	-0.6293	-0.7733 to -0.4854	Yes
16978	15:**40 X** vs. 47:**50 X**	-0.3880	-0.5320 to -0.2440	Yes
16979	15:**40 X** vs. 48:**0 X**	-3.371	-3.532 to -3.210	Yes
16980	15:**40 X** vs. 48:**1 X**	-3.275	-3.419 to -3.131	Yes
16981	15:**40 X** vs. 48:**10 X**	-3.269	-3.413 to -3.125	Yes
16982	15:**40 X** vs. 48:**40 X**	-0.6357	-0.7796 to -0.4917	Yes
16983	15:**40 X** vs. 48:**50 X**	-0.3953	-0.5393 to -0.2514	Yes
16984	15:**40 X** vs. 49:**0 X**	-3.371	-3.515 to -3.227	Yes
16985	15:**40 X** vs. 49:**1 X**	-3.275	-3.419 to -3.131	Yes
16986	15:**40 X** vs. 49:**10 X**	-3.269	-3.413 to -3.125	Yes
16987	15:**40 X** vs. 49:**40 X**	-0.6400	-0.7840 to -0.4960	Yes
16988	15:**40 X** vs. 49:**50 X**	-0.3947	-0.5386 to -0.2507	Yes
16989	15:**40 X** vs. 50:**0 X**	-3.371	-3.515 to -3.227	Yes
16990	15:**40 X** vs. 50:**1 X**	-3.275	-3.419 to -3.131	Yes
16991	15:**40 X** vs. 50:**10 X**	-3.269	-3.413 to -3.125	Yes
16992	15:**40 X** vs. 50:**40 X**	-0.6477	-0.7916 to -0.5037	Yes
16993	15:**40 X** vs. 50:**50 X**	-0.3943	-0.5383 to -0.2504	Yes
16994	15:**50 X** vs. 16:**0 X**	-0.0850	-0.2290 to 0.05896	No
16995	15:**50 X** vs. 16:**1 X**	0.0240	-0.1200 to 0.1680	No
16996	15:**50 X** vs. 16:**10 X**	-0.001667	-0.1456 to 0.1423	No
16997	15:**50 X** vs. 16:**40 X**	-0.0210	-0.1650 to 0.1230	No
16998	15:**50 X** vs. 16:**50 X**	-0.06333	-0.2073 to 0.08063	No
16999	15:**50 X** vs. 17:**0 X**	-0.1377	-0.2816 to 0.006293	No
17000	15:**50 X** vs. 17:**1 X**	-0.0520	-0.1960 to 0.09196	No
17001	15:**50 X** vs. 17:**10 X**	-0.1087	-0.2526 to 0.03529	No
17002	15:**50 X** vs. 17:**40 X**	-0.0320	-0.1760 to 0.1120	No
17003	15:**50 X** vs. 17:**50 X**	-0.07733	-0.2213 to 0.06663	No
17004	15:**50 X** vs. 18:**0 X**	-0.1863	-0.3303 to -0.04237	Yes
17005	15:**50 X** vs. 18:**1 X**	-0.09467	-0.2386 to 0.04929	No
17006	15:**50 X** vs. 18:**10 X**	-0.1870	-0.3310 to -0.04304	Yes
17007	15:**50 X** vs. 18:**40 X**	-0.03633	-0.1803 to 0.1076	No
17008	15:**50 X** vs. 18:**50 X**	-0.08133	-0.2253 to 0.06263	No
17009	15:**50 X** vs. 19:**0 X**	-0.2213	-0.3653 to -0.07737	Yes
17010	15:**50 X** vs. 19:**1 X**	-0.2093	-0.3533 to -0.06537	Yes

2way ANOVA<br>Multiple comparisons	A<br>Data Set-A<br>Y	B<br>Data Set-B<br>Y	C<br>Data Set-C<br>Y
17011 15:50 X vs. 19:10 X	-0.2070	-0.3510 to -0.06304	Yes
17012 15:50 X vs. 19:40 X	-0.0650	-0.2090 to 0.07896	No
17013 15:50 X vs. 19:50 X	-0.0760	-0.2200 to 0.06796	No
17014 15:50 X vs. 20:0 X	-0.3010	-0.4450 to -0.1570	Yes
17015 15:50 X vs. 20:1 X	-0.1203	-0.2643 to 0.02363	No
17016 15:50 X vs. 20:10 X	-0.2093	-0.3533 to -0.06537	Yes
17017 15:50 X vs. 20:40 X	-0.07667	-0.2206 to 0.06729	No
17018 15:50 X vs. 20:50 X	-0.1207	-0.2646 to 0.02329	No
17019 15:50 X vs. 21:0 X	-0.3243	-0.4683 to -0.1804	Yes
17020 15:50 X vs. 21:1 X	-0.2773	-0.4213 to -0.1334	Yes
17021 15:50 X vs. 21:10 X	-0.2743	-0.4183 to -0.1304	Yes
17022 15:50 X vs. 21:40 X	-0.1497	-0.2936 to -0.005707	Yes
17023 15:50 X vs. 21:50 X	-0.2400	-0.3840 to -0.09604	Yes
17024 15:50 X vs. 22:0 X	-0.3660	-0.5100 to -0.2220	Yes
17025 15:50 X vs. 22:1 X	-0.3607	-0.5046 to -0.2167	Yes
17026 15:50 X vs. 22:10 X	-0.3560	-0.5000 to -0.2120	Yes
17027 15:50 X vs. 22:40 X	-0.2563	-0.4003 to -0.1124	Yes
17028 15:50 X vs. 22:50 X	-0.3190	-0.4630 to -0.1750	Yes
17029 15:50 X vs. 23:0 X	-0.4313	-0.5753 to -0.2874	Yes
17030 15:50 X vs. 23:1 X	-0.6210	-0.7820 to -0.4600	Yes
17031 15:50 X vs. 23:10 X	-0.3707	-0.5146 to -0.2267	Yes
17032 15:50 X vs. 23:40 X	-0.2510	-0.4120 to -0.09005	Yes
17033 15:50 X vs. 23:50 X	-0.2850	-0.4290 to -0.1410	Yes
17034 15:50 X vs. 24:0 X	-0.5487	-0.6926 to -0.4047	Yes
17035 15:50 X vs. 24:1 X	-0.7050	-0.8660 to -0.5440	Yes
17036 15:50 X vs. 24:10 X	-0.5325	-0.6935 to -0.3715	Yes
17037 15:50 X vs. 24:40 X	-0.2673	-0.4113 to -0.1234	Yes
17038 15:50 X vs. 24:50 X	-0.1890	-0.3330 to -0.04504	Yes
17039 15:50 X vs. 25:0 X	-0.8860	-1.047 to -0.7250	Yes
17040 15:50 X vs. 25:1 X	-1.076	-1.236 to -0.9145	Yes
17041 15:50 X vs. 25:10 X	-0.4370	-0.6406 to -0.2334	Yes
17042 15:50 X vs. 25:40 X	-0.2693	-0.4133 to -0.1254	Yes
17043 15:50 X vs. 25:50 X	-0.2437	-0.3876 to -0.09971	Yes
17044 15:50 X vs. 26:0 X	-1.099	-1.259 to -0.9375	Yes
17045 15:50 X vs. 26:1 X	-1.212	-1.372 to -1.051	Yes
17046 15:50 X vs. 26:10 X	-0.8980	-1.059 to -0.7370	Yes
17047 15:50 X vs. 26:40 X	-0.3437	-0.4876 to -0.1997	Yes
17048 15:50 X vs. 26:50 X	-0.2040	-0.3480 to -0.06004	Yes
17049 15:50 X vs. 27:0 X	-1.286	-1.430 to -1.142	Yes
17050 15:50 X vs. 27:1 X	-1.377	-1.538 to -1.216	Yes
17051 15:50 X vs. 27:10 X	-1.189	-1.350 to -1.028	Yes
17052 15:50 X vs. 27:40 X	-0.3437	-0.4876 to -0.1997	Yes
17053 15:50 X vs. 27:50 X	-0.2033	-0.3473 to -0.05937	Yes
17054 15:50 X vs. 28:0 X	-1.236	-1.396 to -1.075	Yes
17055 15:50 X vs. 28:1 X	-1.276	-1.420 to -1.132	Yes

	2way ANOVA<br>Multiple comparisons	A<br>Data Set-A<br>Y	B<br>Data Set-B<br>Y	C<br>Data Set-C<br>Y
17056	15:50 X vs. 28:10 X	-1.078	-1.239 to -0.9170	Yes
17057	15:50 X vs. 28:40 X	-0.3560	-0.5000 to -0.2120	Yes
17058	15:50 X vs. 28:50 X	-0.2513	-0.3953 to -0.1074	Yes
17059	15:50 X vs. 29:0 X	-1.717	-1.878 to -1.556	Yes
17060	15:50 X vs. 29:1 X	-1.802	-1.963 to -1.641	Yes
17061	15:50 X vs. 29:10 X	-1.017	-1.177 to -0.8555	Yes
17062	15:50 X vs. 29:40 X	-0.4640	-0.6080 to -0.3200	Yes
17063	15:50 X vs. 29:50 X	-0.2527	-0.3966 to -0.1087	Yes
17064	15:50 X vs. 30:0 X	-1.432	-1.592 to -1.271	Yes
17065	15:50 X vs. 30:1 X	-1.753	-1.914 to -1.592	Yes
17066	15:50 X vs. 30:10 X	-1.091	-1.252 to -0.9300	Yes
17067	15:50 X vs. 30:40 X	-0.3910	-0.5350 to -0.2470	Yes
17068	15:50 X vs. 30:50 X	-0.3050	-0.4490 to -0.1610	Yes
17069	15:50 X vs. 31:0 X	-1.750	-1.910 to -1.589	Yes
17070	15:50 X vs. 31:1 X	-1.740	-1.901 to -1.579	Yes
17071	15:50 X vs. 31:10 X	-1.223	-1.383 to -1.062	Yes
17072	15:50 X vs. 31:40 X	-0.4827	-0.6266 to -0.3387	Yes
17073	15:50 X vs. 31:50 X	-0.2840	-0.4280 to -0.1400	Yes
17074	15:50 X vs. 32:0 X	-2.341	-2.485 to -2.197	Yes
17075	15:50 X vs. 32:1 X	-1.797	-1.958 to -1.636	Yes
17076	15:50 X vs. 32:10 X	-1.653	-1.797 to -1.509	Yes
17077	15:50 X vs. 32:40 X	-0.4790	-0.6230 to -0.3350	Yes
17078	15:50 X vs. 32:50 X	-0.2837	-0.4276 to -0.1397	Yes
17079	15:50 X vs. 33:0 X	-2.345	-2.489 to -2.201	Yes
17080	15:50 X vs. 33:1 X	-2.159	-2.303 to -2.015	Yes
17081	15:50 X vs. 33:10 X	-1.820	-1.964 to -1.676	Yes
17082	15:50 X vs. 33:40 X	-0.4977	-0.6416 to -0.3537	Yes
17083	15:50 X vs. 33:50 X	-0.2987	-0.4426 to -0.1547	Yes
17084	15:50 X vs. 34:0 X	-2.269	-2.413 to -2.125	Yes
17085	15:50 X vs. 34:1 X	-2.277	-2.421 to -2.133	Yes
17086	15:50 X vs. 34:10 X	-1.910	-2.054 to -1.766	Yes
17087	15:50 X vs. 34:40 X	-0.5097	-0.6536 to -0.3657	Yes
17088	15:50 X vs. 34:50 X	-0.4477	-0.5916 to -0.3037	Yes
17089	15:50 X vs. 35:0 X	-2.618	-2.762 to -2.474	Yes
17090	15:50 X vs. 35:1 X	-2.384	-2.528 to -2.240	Yes
17091	15:50 X vs. 35:10 X	-1.872	-2.016 to -1.728	Yes
17092	15:50 X vs. 35:40 X	-0.5187	-0.6626 to -0.3747	Yes
17093	15:50 X vs. 35:50 X	-0.4603	-0.6043 to -0.3164	Yes
17094	15:50 X vs. 36:0 X	-2.825	-2.985 to -2.664	Yes
17095	15:50 X vs. 36:1 X	-2.520	-2.664 to -2.376	Yes
17096	15:50 X vs. 36:10 X	-1.877	-2.021 to -1.733	Yes
17097	15:50 X vs. 36:40 X	-0.5740	-0.7180 to -0.4300	Yes
17098	15:50 X vs. 36:50 X	-0.4237	-0.5676 to -0.2797	Yes
17099	15:50 X vs. 37:0 X	-3.208	-3.352 to -3.064	Yes
17100	15:50 X vs. 37:1 X	-2.590	-2.734 to -2.446	Yes

	2way ANOVA<br>Multiple comparisons	A<br>Data Set-A<br>Y	B<br>Data Set-B<br>Y	C<br>Data Set-C<br>Y
17101	15:**50 X** vs. 37:**10 X**	-1.879	-2.023 to -1.735	Yes
17102	15:**50 X** vs. 37:**40 X**	-0.6113	-0.7553 to -0.4674	Yes
17103	15:**50 X** vs. 37:**50 X**	-0.4323	-0.5763 to -0.2884	Yes
17104	15:**50 X** vs. 38:**0 X**	-3.153	-3.297 to -3.009	Yes
17105	15:**50 X** vs. 38:**1 X**	-2.709	-2.853 to -2.565	Yes
17106	15:**50 X** vs. 38:**10 X**	-2.154	-2.298 to -2.010	Yes
17107	15:**50 X** vs. 38:**40 X**	-0.6157	-0.7596 to -0.4717	Yes
17108	15:**50 X** vs. 38:**50 X**	-0.4363	-0.5803 to -0.2924	Yes
17109	15:**50 X** vs. 39:**0 X**	-3.384	-3.528 to -3.240	Yes
17110	15:**50 X** vs. 39:**1 X**	-3.276	-3.420 to -3.132	Yes
17111	15:**50 X** vs. 39:**10 X**	-2.299	-2.443 to -2.155	Yes
17112	15:**50 X** vs. 39:**40 X**	-0.6217	-0.7656 to -0.4777	Yes
17113	15:**50 X** vs. 39:**50 X**	-0.4673	-0.6113 to -0.3234	Yes
17114	15:**50 X** vs. 40:**0 X**	-3.425	-3.569 to -3.281	Yes
17115	15:**50 X** vs. 40:**1 X**	-3.351	-3.495 to -3.207	Yes
17116	15:**50 X** vs. 40:**10 X**	-2.556	-2.700 to -2.412	Yes
17117	15:**50 X** vs. 40:**40 X**	-0.6263	-0.7703 to -0.4824	Yes
17118	15:**50 X** vs. 40:**50 X**	-0.4737	-0.6176 to -0.3297	Yes
17119	15:**50 X** vs. 41:**0 X**	-3.466	-3.610 to -3.322	Yes
17120	15:**50 X** vs. 41:**1 X**	-3.465	-3.609 to -3.321	Yes
17121	15:**50 X** vs. 41:**10 X**	-2.609	-2.753 to -2.465	Yes
17122	15:**50 X** vs. 41:**40 X**	-0.5737	-0.7176 to -0.4297	Yes
17123	15:**50 X** vs. 41:**50 X**	-0.4863	-0.6303 to -0.3424	Yes
17124	15:**50 X** vs. 42:**0 X**	-3.608	-3.752 to -3.464	Yes
17125	15:**50 X** vs. 42:**1 X**	-3.627	-3.771 to -3.483	Yes
17126	15:**50 X** vs. 42:**10 X**	-2.610	-2.754 to -2.466	Yes
17127	15:**50 X** vs. 42:**40 X**	-0.5863	-0.7303 to -0.4424	Yes
17128	15:**50 X** vs. 42:**50 X**	-0.4897	-0.6336 to -0.3457	Yes
17129	15:**50 X** vs. 43:**0 X**	-3.698	-3.842 to -3.554	Yes
17130	15:**50 X** vs. 43:**1 X**	-3.612	-3.756 to -3.468	Yes
17131	15:**50 X** vs. 43:**10 X**	-3.328	-3.472 to -3.184	Yes
17132	15:**50 X** vs. 43:**40 X**	-0.5847	-0.7286 to -0.4407	Yes
17133	15:**50 X** vs. 43:**50 X**	-0.4900	-0.6340 to -0.3460	Yes
17134	15:**50 X** vs. 44:**0 X**	-3.722	-3.866 to -3.578	Yes
17135	15:**50 X** vs. 44:**1 X**	-3.611	-3.755 to -3.467	Yes
17136	15:**50 X** vs. 44:**10 X**	-3.391	-3.535 to -3.247	Yes
17137	15:**50 X** vs. 44:**40 X**	-0.5867	-0.7306 to -0.4427	Yes
17138	15:**50 X** vs. 44:**50 X**	-0.4223	-0.5663 to -0.2784	Yes
17139	15:**50 X** vs. 45:**0 X**	-3.586	-3.730 to -3.442	Yes
17140	15:**50 X** vs. 45:**1 X**	-3.388	-3.532 to -3.244	Yes
17141	15:**50 X** vs. 45:**10 X**	-3.271	-3.415 to -3.127	Yes
17142	15:**50 X** vs. 45:**40 X**	-0.5880	-0.7320 to -0.4440	Yes
17143	15:**50 X** vs. 45:**50 X**	-0.4193	-0.5633 to -0.2754	Yes
17144	15:**50 X** vs. 46:**0 X**	-3.391	-3.535 to -3.247	Yes
17145	15:**50 X** vs. 46:**1 X**	-3.358	-3.502 to -3.214	Yes

2way ANOVA<br>Multiple comparisons	A<br>Data Set-A<br>Y	B<br>Data Set-B<br>Y	C<br>Data Set-C<br>Y
17146 15:50 X vs. 46:10 X	-3.266	-3.410 to -3.122	Yes
17147 15:50 X vs. 46:40 X	-0.6017	-0.7456 to -0.4577	Yes
17148 15:50 X vs. 46:50 X	-0.4123	-0.5563 to -0.2684	Yes
17149 15:50 X vs. 47:0 X	-3.366	-3.510 to -3.222	Yes
17150 15:50 X vs. 47:1 X	-3.270	-3.414 to -3.126	Yes
17151 15:50 X vs. 47:10 X	-3.252	-3.396 to -3.108	Yes
17152 15:50 X vs. 47:40 X	-0.6117	-0.7556 to -0.4677	Yes
17153 15:50 X vs. 47:50 X	-0.3703	-0.5143 to -0.2264	Yes
17154 15:50 X vs. 48:0 X	-3.354	-3.514 to -3.193	Yes
17155 15:50 X vs. 48:1 X	-3.257	-3.401 to -3.113	Yes
17156 15:50 X vs. 48:10 X	-3.252	-3.396 to -3.108	Yes
17157 15:50 X vs. 48:40 X	-0.6180	-0.7620 to -0.4740	Yes
17158 15:50 X vs. 48:50 X	-0.3777	-0.5216 to -0.2337	Yes
17159 15:50 X vs. 49:0 X	-3.354	-3.498 to -3.210	Yes
17160 15:50 X vs. 49:1 X	-3.257	-3.401 to -3.113	Yes
17161 15:50 X vs. 49:10 X	-3.252	-3.396 to -3.108	Yes
17162 15:50 X vs. 49:40 X	-0.6223	-0.7663 to -0.4784	Yes
17163 15:50 X vs. 49:50 X	-0.3770	-0.5210 to -0.2330	Yes
17164 15:50 X vs. 50:0 X	-3.354	-3.498 to -3.210	Yes
17165 15:50 X vs. 50:1 X	-3.257	-3.401 to -3.113	Yes
17166 15:50 X vs. 50:10 X	-3.252	-3.396 to -3.108	Yes
17167 15:50 X vs. 50:40 X	-0.6300	-0.7740 to -0.4860	Yes
17168 15:50 X vs. 50:50 X	-0.3767	-0.5206 to -0.2327	Yes
17169 16:0 X vs. 16:1 X	0.1090	-0.03496 to 0.2530	No
17170 16:0 X vs. 16:10 X	0.08333	-0.06063 to 0.2273	No
17171 16:0 X vs. 16:40 X	0.0640	-0.07996 to 0.2080	No
17172 16:0 X vs. 16:50 X	0.02167	-0.1223 to 0.1656	No
17173 16:0 X vs. 17:0 X	-0.05267	-0.1966 to 0.09129	No
17174 16:0 X vs. 17:1 X	0.0330	-0.1110 to 0.1770	No
17175 16:0 X vs. 17:10 X	-0.02367	-0.1676 to 0.1203	No
17176 16:0 X vs. 17:40 X	0.0530	-0.09096 to 0.1970	No
17177 16:0 X vs. 17:50 X	0.007667	-0.1363 to 0.1516	No
17178 16:0 X vs. 18:0 X	-0.1013	-0.2453 to 0.04263	No
17179 16:0 X vs. 18:1 X	-0.009667	-0.1536 to 0.1343	No
17180 16:0 X vs. 18:10 X	-0.1020	-0.2460 to 0.04196	No
17181 16:0 X vs. 18:40 X	0.04867	-0.09529 to 0.1926	No
17182 16:0 X vs. 18:50 X	0.003667	-0.1403 to 0.1476	No
17183 16:0 X vs. 19:0 X	-0.1363	-0.2803 to 0.007626	No
17184 16:0 X vs. 19:1 X	-0.1243	-0.2683 to 0.01963	No
17185 16:0 X vs. 19:10 X	-0.1220	-0.2660 to 0.02196	No
17186 16:0 X vs. 19:40 X	0.0200	-0.1240 to 0.1640	No
17187 16:0 X vs. 19:50 X	0.009000	-0.1350 to 0.1530	No
17188 16:0 X vs. 20:0 X	-0.2160	-0.3600 to -0.07204	Yes
17189 16:0 X vs. 20:1 X	-0.03533	-0.1793 to 0.1086	No
17190 16:0 X vs. 20:10 X	-0.1243	-0.2683 to 0.01963	No

	2way ANOVA<br>Multiple comparisons	A<br>Data Set-A<br>Y	B<br>Data Set-B<br>Y	C<br>Data Set-C<br>Y
17191	16:0 X vs. 20:40 X	0.008333	-0.1356 to 0.1523	No
17192	16:0 X vs. 20:50 X	-0.03567	-0.1796 to 0.1083	No
17193	16:0 X vs. 21:0 X	-0.2393	-0.3833 to -0.09537	Yes
17194	16:0 X vs. 21:1 X	-0.1923	-0.3363 to -0.04837	Yes
17195	16:0 X vs. 21:10 X	-0.1893	-0.3333 to -0.04537	Yes
17196	16:0 X vs. 21:40 X	-0.06467	-0.2086 to 0.07929	No
17197	16:0 X vs. 21:50 X	-0.1550	-0.2990 to -0.01104	Yes
17198	16:0 X vs. 22:0 X	-0.2810	-0.4250 to -0.1370	Yes
17199	16:0 X vs. 22:1 X	-0.2757	-0.4196 to -0.1317	Yes
17200	16:0 X vs. 22:10 X	-0.2710	-0.4150 to -0.1270	Yes
17201	16:0 X vs. 22:40 X	-0.1713	-0.3153 to -0.02737	Yes
17202	16:0 X vs. 22:50 X	-0.2340	-0.3780 to -0.09004	Yes
17203	16:0 X vs. 23:0 X	-0.3463	-0.4903 to -0.2024	Yes
17204	16:0 X vs. 23:1 X	-0.5360	-0.6970 to -0.3750	Yes
17205	16:0 X vs. 23:10 X	-0.2857	-0.4296 to -0.1417	Yes
17206	16:0 X vs. 23:40 X	-0.1660	-0.3270 to -0.005048	Yes
17207	16:0 X vs. 23:50 X	-0.2000	-0.3440 to -0.05604	Yes
17208	16:0 X vs. 24:0 X	-0.4637	-0.6076 to -0.3197	Yes
17209	16:0 X vs. 24:1 X	-0.6200	-0.7810 to -0.4590	Yes
17210	16:0 X vs. 24:10 X	-0.4475	-0.6085 to -0.2865	Yes
17211	16:0 X vs. 24:40 X	-0.1823	-0.3263 to -0.03837	Yes
17212	16:0 X vs. 24:50 X	-0.1040	-0.2480 to 0.03996	No
17213	16:0 X vs. 25:0 X	-0.8010	-0.9620 to -0.6400	Yes
17214	16:0 X vs. 25:1 X	-0.9905	-1.151 to -0.8295	Yes
17215	16:0 X vs. 25:10 X	-0.3520	-0.5556 to -0.1484	Yes
17216	16:0 X vs. 25:40 X	-0.1843	-0.3283 to -0.04037	Yes
17217	16:0 X vs. 25:50 X	-0.1587	-0.3026 to -0.01471	Yes
17218	16:0 X vs. 26:0 X	-1.014	-1.174 to -0.8525	Yes
17219	16:0 X vs. 26:1 X	-1.127	-1.287 to -0.9655	Yes
17220	16:0 X vs. 26:10 X	-0.8130	-0.9740 to -0.6520	Yes
17221	16:0 X vs. 26:40 X	-0.2587	-0.4026 to -0.1147	Yes
17222	16:0 X vs. 26:50 X	-0.1190	-0.2630 to 0.02496	No
17223	16:0 X vs. 27:0 X	-1.201	-1.345 to -1.057	Yes
17224	16:0 X vs. 27:1 X	-1.292	-1.453 to -1.131	Yes
17225	16:0 X vs. 27:10 X	-1.104	-1.265 to -0.9430	Yes
17226	16:0 X vs. 27:40 X	-0.2587	-0.4026 to -0.1147	Yes
17227	16:0 X vs. 27:50 X	-0.1183	-0.2623 to 0.02563	No
17228	16:0 X vs. 28:0 X	-1.151	-1.311 to -0.9895	Yes
17229	16:0 X vs. 28:1 X	-1.191	-1.335 to -1.047	Yes
17230	16:0 X vs. 28:10 X	-0.9930	-1.154 to -0.8320	Yes
17231	16:0 X vs. 28:40 X	-0.2710	-0.4150 to -0.1270	Yes
17232	16:0 X vs. 28:50 X	-0.1663	-0.3103 to -0.02237	Yes
17233	16:0 X vs. 29:0 X	-1.632	-1.793 to -1.471	Yes
17234	16:0 X vs. 29:1 X	-1.717	-1.878 to -1.556	Yes
17235	16:0 X vs. 29:10 X	-0.9315	-1.092 to -0.7705	Yes

	2way ANOVA<br>Multiple comparisons	A<br>Data Set-A<br>Y	B<br>Data Set-B<br>Y	C<br>Data Set-C<br>Y
17236	16:**0 X** vs. 29:**40 X**	-0.3790	-0.5230 to -0.2350	Yes
17237	16:**0 X** vs. 29:**50 X**	-0.1677	-0.3116 to -0.02371	Yes
17238	16:**0 X** vs. 30:**0 X**	-1.347	-1.507 to -1.186	Yes
17239	16:**0 X** vs. 30:**1 X**	-1.668	-1.829 to -1.507	Yes
17240	16:**0 X** vs. 30:**10 X**	-1.006	-1.167 to -0.8450	Yes
17241	16:**0 X** vs. 30:**40 X**	-0.3060	-0.4500 to -0.1620	Yes
17242	16:**0 X** vs. 30:**50 X**	-0.2200	-0.3640 to -0.07604	Yes
17243	16:**0 X** vs. 31:**0 X**	-1.665	-1.825 to -1.504	Yes
17244	16:**0 X** vs. 31:**1 X**	-1.655	-1.816 to -1.494	Yes
17245	16:**0 X** vs. 31:**10 X**	-1.138	-1.298 to -0.9765	Yes
17246	16:**0 X** vs. 31:**40 X**	-0.3977	-0.5416 to -0.2537	Yes
17247	16:**0 X** vs. 31:**50 X**	-0.1990	-0.3430 to -0.05504	Yes
17248	16:**0 X** vs. 32:**0 X**	-2.256	-2.400 to -2.112	Yes
17249	16:**0 X** vs. 32:**1 X**	-1.712	-1.873 to -1.551	Yes
17250	16:**0 X** vs. 32:**10 X**	-1.568	-1.712 to -1.424	Yes
17251	16:**0 X** vs. 32:**40 X**	-0.3940	-0.5380 to -0.2500	Yes
17252	16:**0 X** vs. 32:**50 X**	-0.1987	-0.3426 to -0.05471	Yes
17253	16:**0 X** vs. 33:**0 X**	-2.260	-2.404 to -2.116	Yes
17254	16:**0 X** vs. 33:**1 X**	-2.074	-2.218 to -1.930	Yes
17255	16:**0 X** vs. 33:**10 X**	-1.735	-1.879 to -1.591	Yes
17256	16:**0 X** vs. 33:**40 X**	-0.4127	-0.5566 to -0.2687	Yes
17257	16:**0 X** vs. 33:**50 X**	-0.2137	-0.3576 to -0.06971	Yes
17258	16:**0 X** vs. 34:**0 X**	-2.184	-2.328 to -2.040	Yes
17259	16:**0 X** vs. 34:**1 X**	-2.192	-2.336 to -2.048	Yes
17260	16:**0 X** vs. 34:**10 X**	-1.825	-1.969 to -1.681	Yes
17261	16:**0 X** vs. 34:**40 X**	-0.4247	-0.5686 to -0.2807	Yes
17262	16:**0 X** vs. 34:**50 X**	-0.3627	-0.5066 to -0.2187	Yes
17263	16:**0 X** vs. 35:**0 X**	-2.533	-2.677 to -2.389	Yes
17264	16:**0 X** vs. 35:**1 X**	-2.299	-2.443 to -2.155	Yes
17265	16:**0 X** vs. 35:**10 X**	-1.787	-1.931 to -1.643	Yes
17266	16:**0 X** vs. 35:**40 X**	-0.4337	-0.5776 to -0.2897	Yes
17267	16:**0 X** vs. 35:**50 X**	-0.3753	-0.5193 to -0.2314	Yes
17268	16:**0 X** vs. 36:**0 X**	-2.740	-2.900 to -2.579	Yes
17269	16:**0 X** vs. 36:**1 X**	-2.435	-2.579 to -2.291	Yes
17270	16:**0 X** vs. 36:**10 X**	-1.792	-1.936 to -1.648	Yes
17271	16:**0 X** vs. 36:**40 X**	-0.4890	-0.6330 to -0.3450	Yes
17272	16:**0 X** vs. 36:**50 X**	-0.3387	-0.4826 to -0.1947	Yes
17273	16:**0 X** vs. 37:**0 X**	-3.123	-3.267 to -2.979	Yes
17274	16:**0 X** vs. 37:**1 X**	-2.505	-2.649 to -2.361	Yes
17275	16:**0 X** vs. 37:**10 X**	-1.794	-1.938 to -1.650	Yes
17276	16:**0 X** vs. 37:**40 X**	-0.5263	-0.6703 to -0.3824	Yes
17277	16:**0 X** vs. 37:**50 X**	-0.3473	-0.4913 to -0.2034	Yes
17278	16:**0 X** vs. 38:**0 X**	-3.068	-3.212 to -2.924	Yes
17279	16:**0 X** vs. 38:**1 X**	-2.624	-2.768 to -2.480	Yes
17280	16:**0 X** vs. 38:**10 X**	-2.069	-2.213 to -1.925	Yes

2way ANOVA Multiple comparisons	A Data Set-A Y	B Data Set-B Y	C Data Set-C Y
17281 16:0 X vs. 38:40 X	-0.5307	-0.6746 to -0.3867	Yes
17282 16:0 X vs. 38:50 X	-0.3513	-0.4953 to -0.2074	Yes
17283 16:0 X vs. 39:0 X	-3.299	-3.443 to -3.155	Yes
17284 16:0 X vs. 39:1 X	-3.191	-3.335 to -3.047	Yes
17285 16:0 X vs. 39:10 X	-2.214	-2.358 to -2.070	Yes
17286 16:0 X vs. 39:40 X	-0.5367	-0.6806 to -0.3927	Yes
17287 16:0 X vs. 39:50 X	-0.3823	-0.5263 to -0.2384	Yes
17288 16:0 X vs. 40:0 X	-3.340	-3.484 to -3.196	Yes
17289 16:0 X vs. 40:1 X	-3.266	-3.410 to -3.122	Yes
17290 16:0 X vs. 40:10 X	-2.471	-2.615 to -2.327	Yes
17291 16:0 X vs. 40:40 X	-0.5413	-0.6853 to -0.3974	Yes
17292 16:0 X vs. 40:50 X	-0.3887	-0.5326 to -0.2447	Yes
17293 16:0 X vs. 41:0 X	-3.381	-3.525 to -3.237	Yes
17294 16:0 X vs. 41:1 X	-3.380	-3.524 to -3.236	Yes
17295 16:0 X vs. 41:10 X	-2.524	-2.668 to -2.380	Yes
17296 16:0 X vs. 41:40 X	-0.4887	-0.6326 to -0.3447	Yes
17297 16:0 X vs. 41:50 X	-0.4013	-0.5453 to -0.2574	Yes
17298 16:0 X vs. 42:0 X	-3.523	-3.667 to -3.379	Yes
17299 16:0 X vs. 42:1 X	-3.542	-3.686 to -3.398	Yes
17300 16:0 X vs. 42:10 X	-2.525	-2.669 to -2.381	Yes
17301 16:0 X vs. 42:40 X	-0.5013	-0.6453 to -0.3574	Yes
17302 16:0 X vs. 42:50 X	-0.4047	-0.5486 to -0.2607	Yes
17303 16:0 X vs. 43:0 X	-3.613	-3.757 to -3.469	Yes
17304 16:0 X vs. 43:1 X	-3.527	-3.671 to -3.383	Yes
17305 16:0 X vs. 43:10 X	-3.243	-3.387 to -3.099	Yes
17306 16:0 X vs. 43:40 X	-0.4997	-0.6436 to -0.3557	Yes
17307 16:0 X vs. 43:50 X	-0.4050	-0.5490 to -0.2610	Yes
17308 16:0 X vs. 44:0 X	-3.637	-3.781 to -3.493	Yes
17309 16:0 X vs. 44:1 X	-3.526	-3.670 to -3.382	Yes
17310 16:0 X vs. 44:10 X	-3.306	-3.450 to -3.162	Yes
17311 16:0 X vs. 44:40 X	-0.5017	-0.6456 to -0.3577	Yes
17312 16:0 X vs. 44:50 X	-0.3373	-0.4813 to -0.1934	Yes
17313 16:0 X vs. 45:0 X	-3.501	-3.645 to -3.357	Yes
17314 16:0 X vs. 45:1 X	-3.303	-3.447 to -3.159	Yes
17315 16:0 X vs. 45:10 X	-3.186	-3.330 to -3.042	Yes
17316 16:0 X vs. 45:40 X	-0.5030	-0.6470 to -0.3590	Yes
17317 16:0 X vs. 45:50 X	-0.3343	-0.4783 to -0.1904	Yes
17318 16:0 X vs. 46:0 X	-3.306	-3.450 to -3.162	Yes
17319 16:0 X vs. 46:1 X	-3.273	-3.417 to -3.129	Yes
17320 16:0 X vs. 46:10 X	-3.181	-3.325 to -3.037	Yes
17321 16:0 X vs. 46:40 X	-0.5167	-0.6606 to -0.3727	Yes
17322 16:0 X vs. 46:50 X	-0.3273	-0.4713 to -0.1834	Yes
17323 16:0 X vs. 47:0 X	-3.281	-3.425 to -3.137	Yes
17324 16:0 X vs. 47:1 X	-3.185	-3.329 to -3.041	Yes
17325 16:0 X vs. 47:10 X	-3.167	-3.311 to -3.023	Yes

	2way ANOVA<br>Multiple comparisons	A<br>Data Set-A<br>Y	B<br>Data Set-B<br>Y	C<br>Data Set-C<br>Y
17326	16:**0 X** vs. 47:**40 X**	-0.5267	-0.6706 to -0.3827	Yes
17327	16:**0 X** vs. 47:**50 X**	-0.2853	-0.4293 to -0.1414	Yes
17328	16:**0 X** vs. 48:**0 X**	-3.269	-3.429 to -3.108	Yes
17329	16:**0 X** vs. 48:**1 X**	-3.172	-3.316 to -3.028	Yes
17330	16:**0 X** vs. 48:**10 X**	-3.167	-3.311 to -3.023	Yes
17331	16:**0 X** vs. 48:**40 X**	-0.5330	-0.6770 to -0.3890	Yes
17332	16:**0 X** vs. 48:**50 X**	-0.2927	-0.4366 to -0.1487	Yes
17333	16:**0 X** vs. 49:**0 X**	-3.269	-3.413 to -3.125	Yes
17334	16:**0 X** vs. 49:**1 X**	-3.172	-3.316 to -3.028	Yes
17335	16:**0 X** vs. 49:**10 X**	-3.167	-3.311 to -3.023	Yes
17336	16:**0 X** vs. 49:**40 X**	-0.5373	-0.6813 to -0.3934	Yes
17337	16:**0 X** vs. 49:**50 X**	-0.2920	-0.4360 to -0.1480	Yes
17338	16:**0 X** vs. 50:**0 X**	-3.269	-3.413 to -3.125	Yes
17339	16:**0 X** vs. 50:**1 X**	-3.172	-3.316 to -3.028	Yes
17340	16:**0 X** vs. 50:**10 X**	-3.167	-3.311 to -3.023	Yes
17341	16:**0 X** vs. 50:**40 X**	-0.5450	-0.6890 to -0.4010	Yes
17342	16:**0 X** vs. 50:**50 X**	-0.2917	-0.4356 to -0.1477	Yes
17343	16:**1 X** vs. 16:**10 X**	-0.02567	-0.1696 to 0.1183	No
17344	16:**1 X** vs. 16:**40 X**	-0.0450	-0.1890 to 0.09896	No
17345	16:**1 X** vs. 16:**50 X**	-0.08733	-0.2313 to 0.05663	No
17346	16:**1 X** vs. 17:**0 X**	-0.1617	-0.3056 to -0.01771	Yes
17347	16:**1 X** vs. 17:**1 X**	-0.07600	-0.2200 to 0.06796	No
17348	16:**1 X** vs. 17:**10 X**	-0.1327	-0.2766 to 0.01129	No
17349	16:**1 X** vs. 17:**40 X**	-0.0560	-0.2000 to 0.08796	No
17350	16:**1 X** vs. 17:**50 X**	-0.1013	-0.2453 to 0.04263	No
17351	16:**1 X** vs. 18:**0 X**	-0.2103	-0.3543 to -0.06637	Yes
17352	16:**1 X** vs. 18:**1 X**	-0.1187	-0.2626 to 0.02529	No
17353	16:**1 X** vs. 18:**10 X**	-0.2110	-0.3550 to -0.06704	Yes
17354	16:**1 X** vs. 18:**40 X**	-0.06033	-0.2043 to 0.08363	No
17355	16:**1 X** vs. 18:**50 X**	-0.1053	-0.2493 to 0.03863	No
17356	16:**1 X** vs. 19:**0 X**	-0.2453	-0.3893 to -0.1014	Yes
17357	16:**1 X** vs. 19:**1 X**	-0.2333	-0.3773 to -0.08937	Yes
17358	16:**1 X** vs. 19:**10 X**	-0.2310	-0.3750 to -0.08704	Yes
17359	16:**1 X** vs. 19:**40 X**	-0.0890	-0.2330 to 0.05496	No
17360	16:**1 X** vs. 19:**50 X**	-0.1000	-0.2440 to 0.04396	No
17361	16:**1 X** vs. 20:**0 X**	-0.3250	-0.4690 to -0.1810	Yes
17362	16:**1 X** vs. 20:**1 X**	-0.1443	-0.2883 to -0.0003735	Yes
17363	16:**1 X** vs. 20:**10 X**	-0.2333	-0.3773 to -0.08937	Yes
17364	16:**1 X** vs. 20:**40 X**	-0.1007	-0.2446 to 0.04329	No
17365	16:**1 X** vs. 20:**50 X**	-0.1447	-0.2886 to -0.0007068	Yes
17366	16:**1 X** vs. 21:**0 X**	-0.3483	-0.4923 to -0.2044	Yes
17367	16:**1 X** vs. 21:**1 X**	-0.3013	-0.4453 to -0.1574	Yes
17368	16:**1 X** vs. 21:**10 X**	-0.2983	-0.4423 to -0.1544	Yes
17369	16:**1 X** vs. 21:**40 X**	-0.1737	-0.3176 to -0.02971	Yes
17370	16:**1 X** vs. 21:**50 X**	-0.2640	-0.4080 to -0.1200	Yes

	2way ANOVA<br>Multiple comparisons	A<br>Data Set-A<br>Y	B<br>Data Set-B<br>Y	C<br>Data Set-C<br>Y
17371	16:1 X vs. 22:0 X	-0.3900	-0.5340 to -0.2460	Yes
17372	16:1 X vs. 22:1 X	-0.3847	-0.5286 to -0.2407	Yes
17373	16:1 X vs. 22:10 X	-0.3800	-0.5240 to -0.2360	Yes
17374	16:1 X vs. 22:40 X	-0.2803	-0.4243 to -0.1364	Yes
17375	16:1 X vs. 22:50 X	-0.3430	-0.4870 to -0.1990	Yes
17376	16:1 X vs. 23:0 X	-0.4553	-0.5993 to -0.3114	Yes
17377	16:1 X vs. 23:1 X	-0.6450	-0.8060 to -0.4840	Yes
17378	16:1 X vs. 23:10 X	-0.3947	-0.5386 to -0.2507	Yes
17379	16:1 X vs. 23:40 X	-0.2750	-0.4360 to -0.1140	Yes
17380	16:1 X vs. 23:50 X	-0.3090	-0.4530 to -0.1650	Yes
17381	16:1 X vs. 24:0 X	-0.5727	-0.7166 to -0.4287	Yes
17382	16:1 X vs. 24:1 X	-0.7290	-0.8900 to -0.5680	Yes
17383	16:1 X vs. 24:10 X	-0.5565	-0.7175 to -0.3955	Yes
17384	16:1 X vs. 24:40 X	-0.2913	-0.4353 to -0.1474	Yes
17385	16:1 X vs. 24:50 X	-0.2130	-0.3570 to -0.06904	Yes
17386	16:1 X vs. 25:0 X	-0.9100	-1.071 to -0.7490	Yes
17387	16:1 X vs. 25:1 X	-1.100	-1.260 to -0.9385	Yes
17388	16:1 X vs. 25:10 X	-0.4610	-0.6646 to -0.2574	Yes
17389	16:1 X vs. 25:40 X	-0.2933	-0.4373 to -0.1494	Yes
17390	16:1 X vs. 25:50 X	-0.2677	-0.4116 to -0.1237	Yes
17391	16:1 X vs. 26:0 X	-1.123	-1.283 to -0.9615	Yes
17392	16:1 X vs. 26:1 X	-1.236	-1.396 to -1.075	Yes
17393	16:1 X vs. 26:10 X	-0.9220	-1.083 to -0.7610	Yes
17394	16:1 X vs. 26:40 X	-0.3677	-0.5116 to -0.2237	Yes
17395	16:1 X vs. 26:50 X	-0.2280	-0.3720 to -0.08404	Yes
17396	16:1 X vs. 27:0 X	-1.310	-1.454 to -1.166	Yes
17397	16:1 X vs. 27:1 X	-1.401	-1.562 to -1.240	Yes
17398	16:1 X vs. 27:10 X	-1.213	-1.374 to -1.052	Yes
17399	16:1 X vs. 27:40 X	-0.3677	-0.5116 to -0.2237	Yes
17400	16:1 X vs. 27:50 X	-0.2273	-0.3713 to -0.08337	Yes
17401	16:1 X vs. 28:0 X	-1.260	-1.420 to -1.099	Yes
17402	16:1 X vs. 28:1 X	-1.300	-1.444 to -1.156	Yes
17403	16:1 X vs. 28:10 X	-1.102	-1.263 to -0.9410	Yes
17404	16:1 X vs. 28:40 X	-0.3800	-0.5240 to -0.2360	Yes
17405	16:1 X vs. 28:50 X	-0.2753	-0.4193 to -0.1314	Yes
17406	16:1 X vs. 29:0 X	-1.741	-1.902 to -1.580	Yes
17407	16:1 X vs. 29:1 X	-1.826	-1.987 to -1.665	Yes
17408	16:1 X vs. 29:10 X	-1.041	-1.201 to -0.8795	Yes
17409	16:1 X vs. 29:40 X	-0.4880	-0.6320 to -0.3440	Yes
17410	16:1 X vs. 29:50 X	-0.2767	-0.4206 to -0.1327	Yes
17411	16:1 X vs. 30:0 X	-1.456	-1.616 to -1.295	Yes
17412	16:1 X vs. 30:1 X	-1.777	-1.938 to -1.616	Yes
17413	16:1 X vs. 30:10 X	-1.115	-1.276 to -0.9540	Yes
17414	16:1 X vs. 30:40 X	-0.4150	-0.5590 to -0.2710	Yes
17415	16:1 X vs. 30:50 X	-0.3290	-0.4730 to -0.1850	Yes

	2way ANOVA<br>Multiple comparisons	A<br>Data Set-A<br>Y	B<br>Data Set-B<br>Y	C<br>Data Set-C<br>Y
17416	16:1 X vs. 31:0 X	-1.774	-1.934 to -1.613	Yes
17417	16:1 X vs. 31:1 X	-1.764	-1.925 to -1.603	Yes
17418	16:1 X vs. 31:10 X	-1.247	-1.407 to -1.086	Yes
17419	16:1 X vs. 31:40 X	-0.5067	-0.6506 to -0.3627	Yes
17420	16:1 X vs. 31:50 X	-0.3080	-0.4520 to -0.1640	Yes
17421	16:1 X vs. 32:0 X	-2.365	-2.509 to -2.221	Yes
17422	16:1 X vs. 32:1 X	-1.821	-1.982 to -1.660	Yes
17423	16:1 X vs. 32:10 X	-1.677	-1.821 to -1.533	Yes
17424	16:1 X vs. 32:40 X	-0.5030	-0.6470 to -0.3590	Yes
17425	16:1 X vs. 32:50 X	-0.3077	-0.4516 to -0.1637	Yes
17426	16:1 X vs. 33:0 X	-2.369	-2.513 to -2.225	Yes
17427	16:1 X vs. 33:1 X	-2.183	-2.327 to -2.039	Yes
17428	16:1 X vs. 33:10 X	-1.844	-1.988 to -1.700	Yes
17429	16:1 X vs. 33:40 X	-0.5217	-0.6656 to -0.3777	Yes
17430	16:1 X vs. 33:50 X	-0.3227	-0.4666 to -0.1787	Yes
17431	16:1 X vs. 34:0 X	-2.293	-2.437 to -2.149	Yes
17432	16:1 X vs. 34:1 X	-2.301	-2.445 to -2.157	Yes
17433	16:1 X vs. 34:10 X	-1.934	-2.078 to -1.790	Yes
17434	16:1 X vs. 34:40 X	-0.5337	-0.6776 to -0.3897	Yes
17435	16:1 X vs. 34:50 X	-0.4717	-0.6156 to -0.3277	Yes
17436	16:1 X vs. 35:0 X	-2.642	-2.786 to -2.498	Yes
17437	16:1 X vs. 35:1 X	-2.408	-2.552 to -2.264	Yes
17438	16:1 X vs. 35:10 X	-1.896	-2.040 to -1.752	Yes
17439	16:1 X vs. 35:40 X	-0.5427	-0.6866 to -0.3987	Yes
17440	16:1 X vs. 35:50 X	-0.4843	-0.6283 to -0.3404	Yes
17441	16:1 X vs. 36:0 X	-2.849	-3.009 to -2.688	Yes
17442	16:1 X vs. 36:1 X	-2.544	-2.688 to -2.400	Yes
17443	16:1 X vs. 36:10 X	-1.901	-2.045 to -1.757	Yes
17444	16:1 X vs. 36:40 X	-0.5980	-0.7420 to -0.4540	Yes
17445	16:1 X vs. 36:50 X	-0.4477	-0.5916 to -0.3037	Yes
17446	16:1 X vs. 37:0 X	-3.232	-3.376 to -3.088	Yes
17447	16:1 X vs. 37:1 X	-2.614	-2.758 to -2.470	Yes
17448	16:1 X vs. 37:10 X	-1.903	-2.047 to -1.759	Yes
17449	16:1 X vs. 37:40 X	-0.6353	-0.7793 to -0.4914	Yes
17450	16:1 X vs. 37:50 X	-0.4563	-0.6003 to -0.3124	Yes
17451	16:1 X vs. 38:0 X	-3.177	-3.321 to -3.033	Yes
17452	16:1 X vs. 38:1 X	-2.733	-2.877 to -2.589	Yes
17453	16:1 X vs. 38:10 X	-2.178	-2.322 to -2.034	Yes
17454	16:1 X vs. 38:40 X	-0.6397	-0.7836 to -0.4957	Yes
17455	16:1 X vs. 38:50 X	-0.4603	-0.6043 to -0.3164	Yes
17456	16:1 X vs. 39:0 X	-3.408	-3.552 to -3.264	Yes
17457	16:1 X vs. 39:1 X	-3.300	-3.444 to -3.156	Yes
17458	16:1 X vs. 39:10 X	-2.323	-2.467 to -2.179	Yes
17459	16:1 X vs. 39:40 X	-0.6457	-0.7896 to -0.5017	Yes
17460	16:1 X vs. 39:50 X	-0.4913	-0.6353 to -0.3474	Yes

2way ANOVA<br>Multiple comparisons	A<br>Data Set-A<br>Y	B<br>Data Set-B<br>Y	C<br>Data Set-C<br>Y
17461 16:1 X vs. 40:0 X	-3.449	-3.593 to -3.305	Yes
17462 16:1 X vs. 40:1 X	-3.375	-3.519 to -3.231	Yes
17463 16:1 X vs. 40:10 X	-2.580	-2.724 to -2.436	Yes
17464 16:1 X vs. 40:40 X	-0.6503	-0.7943 to -0.5064	Yes
17465 16:1 X vs. 40:50 X	-0.4977	-0.6416 to -0.3537	Yes
17466 16:1 X vs. 41:0 X	-3.490	-3.634 to -3.346	Yes
17467 16:1 X vs. 41:1 X	-3.489	-3.633 to -3.345	Yes
17468 16:1 X vs. 41:10 X	-2.633	-2.777 to -2.489	Yes
17469 16:1 X vs. 41:40 X	-0.5977	-0.7416 to -0.4537	Yes
17470 16:1 X vs. 41:50 X	-0.5103	-0.6543 to -0.3664	Yes
17471 16:1 X vs. 42:0 X	-3.632	-3.776 to -3.488	Yes
17472 16:1 X vs. 42:1 X	-3.651	-3.795 to -3.507	Yes
17473 16:1 X vs. 42:10 X	-2.634	-2.778 to -2.490	Yes
17474 16:1 X vs. 42:40 X	-0.6103	-0.7543 to -0.4664	Yes
17475 16:1 X vs. 42:50 X	-0.5137	-0.6576 to -0.3697	Yes
17476 16:1 X vs. 43:0 X	-3.722	-3.866 to -3.578	Yes
17477 16:1 X vs. 43:1 X	-3.636	-3.780 to -3.492	Yes
17478 16:1 X vs. 43:10 X	-3.352	-3.496 to -3.208	Yes
17479 16:1 X vs. 43:40 X	-0.6087	-0.7526 to -0.4647	Yes
17480 16:1 X vs. 43:50 X	-0.5140	-0.6580 to -0.3700	Yes
17481 16:1 X vs. 44:0 X	-3.746	-3.890 to -3.602	Yes
17482 16:1 X vs. 44:1 X	-3.635	-3.779 to -3.491	Yes
17483 16:1 X vs. 44:10 X	-3.415	-3.559 to -3.271	Yes
17484 16:1 X vs. 44:40 X	-0.6107	-0.7546 to -0.4667	Yes
17485 16:1 X vs. 44:50 X	-0.4463	-0.5903 to -0.3024	Yes
17486 16:1 X vs. 45:0 X	-3.610	-3.754 to -3.466	Yes
17487 16:1 X vs. 45:1 X	-3.412	-3.556 to -3.268	Yes
17488 16:1 X vs. 45:10 X	-3.295	-3.439 to -3.151	Yes
17489 16:1 X vs. 45:40 X	-0.6120	-0.7560 to -0.4680	Yes
17490 16:1 X vs. 45:50 X	-0.4433	-0.5873 to -0.2994	Yes
17491 16:1 X vs. 46:0 X	-3.415	-3.559 to -3.271	Yes
17492 16:1 X vs. 46:1 X	-3.382	-3.526 to -3.238	Yes
17493 16:1 X vs. 46:10 X	-3.290	-3.434 to -3.146	Yes
17494 16:1 X vs. 46:40 X	-0.6257	-0.7696 to -0.4817	Yes
17495 16:1 X vs. 46:50 X	-0.4363	-0.5803 to -0.2924	Yes
17496 16:1 X vs. 47:0 X	-3.390	-3.534 to -3.246	Yes
17497 16:1 X vs. 47:1 X	-3.294	-3.438 to -3.150	Yes
17498 16:1 X vs. 47:10 X	-3.276	-3.420 to -3.132	Yes
17499 16:1 X vs. 47:40 X	-0.6357	-0.7796 to -0.4917	Yes
17500 16:1 X vs. 47:50 X	-0.3943	-0.5383 to -0.2504	Yes
17501 16:1 X vs. 48:0 X	-3.378	-3.538 to -3.217	Yes
17502 16:1 X vs. 48:1 X	-3.281	-3.425 to -3.137	Yes
17503 16:1 X vs. 48:10 X	-3.276	-3.420 to -3.132	Yes
17504 16:1 X vs. 48:40 X	-0.6420	-0.7860 to -0.4980	Yes
17505 16:1 X vs. 48:50 X	-0.4017	-0.5456 to -0.2577	Yes

2way ANOVA<br>Multiple comparisons	A<br>Data Set-A<br>Y	B<br>Data Set-B<br>Y	C<br>Data Set-C<br>Y
17506 16:**1 X** vs. 49:**0 X**	-3.378	-3.522 to -3.234	Yes
17507 16:**1 X** vs. 49:**1 X**	-3.281	-3.425 to -3.137	Yes
17508 16:**1 X** vs. 49:**10 X**	-3.276	-3.420 to -3.132	Yes
17509 16:**1 X** vs. 49:**40 X**	-0.6463	-0.7903 to -0.5024	Yes
17510 16:**1 X** vs. 49:**50 X**	-0.4010	-0.5450 to -0.2570	Yes
17511 16:**1 X** vs. 50:**0 X**	-3.378	-3.522 to -3.234	Yes
17512 16:**1 X** vs. 50:**1 X**	-3.281	-3.425 to -3.137	Yes
17513 16:**1 X** vs. 50:**10 X**	-3.276	-3.420 to -3.132	Yes
17514 16:**1 X** vs. 50:**40 X**	-0.6540	-0.7980 to -0.5100	Yes
17515 16:**1 X** vs. 50:**50 X**	-0.4007	-0.5446 to -0.2567	Yes
17516 16:**10 X** vs. 16:**40 X**	-0.01933	-0.1633 to 0.1246	No
17517 16:**10 X** vs. 16:**50 X**	-0.06167	-0.2056 to 0.08229	No
17518 16:**10 X** vs. 17:**0 X**	-0.1360	-0.2800 to 0.007960	No
17519 16:**10 X** vs. 17:**1 X**	-0.05033	-0.1943 to 0.09363	No
17520 16:**10 X** vs. 17:**10 X**	-0.1070	-0.2510 to 0.03696	No
17521 16:**10 X** vs. 17:**40 X**	-0.03033	-0.1743 to 0.1136	No
17522 16:**10 X** vs. 17:**50 X**	-0.07567	-0.2196 to 0.06829	No
17523 16:**10 X** vs. 18:**0 X**	-0.1847	-0.3286 to -0.04071	Yes
17524 16:**10 X** vs. 18:**1 X**	-0.0930	-0.2370 to 0.05096	No
17525 16:**10 X** vs. 18:**10 X**	-0.1853	-0.3293 to -0.04137	Yes
17526 16:**10 X** vs. 18:**40 X**	-0.03467	-0.1786 to 0.1093	No
17527 16:**10 X** vs. 18:**50 X**	-0.07967	-0.2236 to 0.06429	No
17528 16:**10 X** vs. 19:**0 X**	-0.2197	-0.3636 to -0.07571	Yes
17529 16:**10 X** vs. 19:**1 X**	-0.2077	-0.3516 to -0.06371	Yes
17530 16:**10 X** vs. 19:**10 X**	-0.2053	-0.3493 to -0.06137	Yes
17531 16:**10 X** vs. 19:**40 X**	-0.06333	-0.2073 to 0.08063	No
17532 16:**10 X** vs. 19:**50 X**	-0.07433	-0.2183 to 0.06963	No
17533 16:**10 X** vs. 20:**0 X**	-0.2993	-0.4433 to -0.1554	Yes
17534 16:**10 X** vs. 20:**1 X**	-0.1187	-0.2626 to 0.02529	No
17535 16:**10 X** vs. 20:**10 X**	-0.2077	-0.3516 to -0.06371	Yes
17536 16:**10 X** vs. 20:**40 X**	-0.0750	-0.2190 to 0.06896	No
17537 16:**10 X** vs. 20:**50 X**	-0.1190	-0.2630 to 0.02496	No
17538 16:**10 X** vs. 21:**0 X**	-0.3227	-0.4666 to -0.1787	Yes
17539 16:**10 X** vs. 21:**1 X**	-0.2757	-0.4196 to -0.1317	Yes
17540 16:**10 X** vs. 21:**10 X**	-0.2727	-0.4166 to -0.1287	Yes
17541 16:**10 X** vs. 21:**40 X**	-0.1480	-0.2920 to -0.004040	Yes
17542 16:**10 X** vs. 21:**50 X**	-0.2383	-0.3823 to -0.09437	Yes
17543 16:**10 X** vs. 22:**0 X**	-0.3643	-0.5083 to -0.2204	Yes
17544 16:**10 X** vs. 22:**1 X**	-0.3590	-0.5030 to -0.2150	Yes
17545 16:**10 X** vs. 22:**10 X**	-0.3543	-0.4983 to -0.2104	Yes
17546 16:**10 X** vs. 22:**40 X**	-0.2547	-0.3986 to -0.1107	Yes
17547 16:**10 X** vs. 22:**50 X**	-0.3173	-0.4613 to -0.1734	Yes
17548 16:**10 X** vs. 23:**0 X**	-0.4297	-0.5736 to -0.2857	Yes
17549 16:**10 X** vs. 23:**1 X**	-0.6193	-0.7803 to -0.4584	Yes
17550 16:**10 X** vs. 23:**10 X**	-0.3690	-0.5130 to -0.2250	Yes

	2way ANOVA<br>Multiple comparisons	A<br>Data Set-A<br>Y	B<br>Data Set-B<br>Y	C<br>Data Set-C<br>Y
17551	16:10 X vs. 23:40 X	-0.2493	-0.4103 to -0.08838	Yes
17552	16:10 X vs. 23:50 X	-0.2833	-0.4273 to -0.1394	Yes
17553	16:10 X vs. 24:0 X	-0.5470	-0.6910 to -0.4030	Yes
17554	16:10 X vs. 24:1 X	-0.7033	-0.8643 to -0.5424	Yes
17555	16:10 X vs. 24:10 X	-0.5308	-0.6918 to -0.3699	Yes
17556	16:10 X vs. 24:40 X	-0.2657	-0.4096 to -0.1217	Yes
17557	16:10 X vs. 24:50 X	-0.1873	-0.3313 to -0.04337	Yes
17558	16:10 X vs. 25:0 X	-0.8843	-1.045 to -0.7234	Yes
17559	16:10 X vs. 25:1 X	-1.074	-1.235 to -0.9129	Yes
17560	16:10 X vs. 25:10 X	-0.4353	-0.6389 to -0.2317	Yes
17561	16:10 X vs. 25:40 X	-0.2677	-0.4116 to -0.1237	Yes
17562	16:10 X vs. 25:50 X	-0.2420	-0.3860 to -0.09804	Yes
17563	16:10 X vs. 26:0 X	-1.097	-1.258 to -0.9359	Yes
17564	16:10 X vs. 26:1 X	-1.210	-1.371 to -1.049	Yes
17565	16:10 X vs. 26:10 X	-0.8963	-1.057 to -0.7354	Yes
17566	16:10 X vs. 26:40 X	-0.3420	-0.4860 to -0.1980	Yes
17567	16:10 X vs. 26:50 X	-0.2023	-0.3463 to -0.05837	Yes
17568	16:10 X vs. 27:0 X	-1.285	-1.429 to -1.141	Yes
17569	16:10 X vs. 27:1 X	-1.375	-1.536 to -1.214	Yes
17570	16:10 X vs. 27:10 X	-1.187	-1.348 to -1.026	Yes
17571	16:10 X vs. 27:40 X	-0.3420	-0.4860 to -0.1980	Yes
17572	16:10 X vs. 27:50 X	-0.2017	-0.3456 to -0.05771	Yes
17573	16:10 X vs. 28:0 X	-1.234	-1.395 to -1.073	Yes
17574	16:10 X vs. 28:1 X	-1.275	-1.419 to -1.131	Yes
17575	16:10 X vs. 28:10 X	-1.076	-1.237 to -0.9154	Yes
17576	16:10 X vs. 28:40 X	-0.3543	-0.4983 to -0.2104	Yes
17577	16:10 X vs. 28:50 X	-0.2497	-0.3936 to -0.1057	Yes
17578	16:10 X vs. 29:0 X	-1.715	-1.876 to -1.554	Yes
17579	16:10 X vs. 29:1 X	-1.800	-1.961 to -1.639	Yes
17580	16:10 X vs. 29:10 X	-1.015	-1.176 to -0.8539	Yes
17581	16:10 X vs. 29:40 X	-0.4623	-0.6063 to -0.3184	Yes
17582	16:10 X vs. 29:50 X	-0.2510	-0.3950 to -0.1070	Yes
17583	16:10 X vs. 30:0 X	-1.430	-1.591 to -1.269	Yes
17584	16:10 X vs. 30:1 X	-1.751	-1.912 to -1.590	Yes
17585	16:10 X vs. 30:10 X	-1.089	-1.250 to -0.9284	Yes
17586	16:10 X vs. 30:40 X	-0.3893	-0.5333 to -0.2454	Yes
17587	16:10 X vs. 30:50 X	-0.3033	-0.4473 to -0.1594	Yes
17588	16:10 X vs. 31:0 X	-1.748	-1.909 to -1.587	Yes
17589	16:10 X vs. 31:1 X	-1.738	-1.899 to -1.577	Yes
17590	16:10 X vs. 31:10 X	-1.221	-1.382 to -1.060	Yes
17591	16:10 X vs. 31:40 X	-0.4810	-0.6250 to -0.3370	Yes
17592	16:10 X vs. 31:50 X	-0.2823	-0.4263 to -0.1384	Yes
17593	16:10 X vs. 32:0 X	-2.339	-2.483 to -2.195	Yes
17594	16:10 X vs. 32:1 X	-1.795	-1.956 to -1.634	Yes
17595	16:10 X vs. 32:10 X	-1.651	-1.795 to -1.507	Yes

	2way ANOVA<br>Multiple comparisons	A<br>Data Set-A<br>Y	B<br>Data Set-B<br>Y	C<br>Data Set-C<br>Y
17596	16:**10 X** vs. 32:**40 X**	-0.4773	-0.6213 to -0.3334	Yes
17597	16:**10 X** vs. 32:**50 X**	-0.2820	-0.4260 to -0.1380	Yes
17598	16:**10 X** vs. 33:**0 X**	-2.343	-2.487 to -2.199	Yes
17599	16:**10 X** vs. 33:**1 X**	-2.158	-2.302 to -2.014	Yes
17600	16:**10 X** vs. 33:**10 X**	-1.818	-1.962 to -1.674	Yes
17601	16:**10 X** vs. 33:**40 X**	-0.4960	-0.6400 to -0.3520	Yes
17602	16:**10 X** vs. 33:**50 X**	-0.2970	-0.4410 to -0.1530	Yes
17603	16:**10 X** vs. 34:**0 X**	-2.268	-2.412 to -2.124	Yes
17604	16:**10 X** vs. 34:**1 X**	-2.275	-2.419 to -2.131	Yes
17605	16:**10 X** vs. 34:**10 X**	-1.908	-2.052 to -1.764	Yes
17606	16:**10 X** vs. 34:**40 X**	-0.5080	-0.6520 to -0.3640	Yes
17607	16:**10 X** vs. 34:**50 X**	-0.4460	-0.5900 to -0.3020	Yes
17608	16:**10 X** vs. 35:**0 X**	-2.616	-2.760 to -2.472	Yes
17609	16:**10 X** vs. 35:**1 X**	-2.382	-2.526 to -2.238	Yes
17610	16:**10 X** vs. 35:**10 X**	-1.870	-2.014 to -1.726	Yes
17611	16:**10 X** vs. 35:**40 X**	-0.5170	-0.6610 to -0.3730	Yes
17612	16:**10 X** vs. 35:**50 X**	-0.4587	-0.6026 to -0.3147	Yes
17613	16:**10 X** vs. 36:**0 X**	-2.823	-2.984 to -2.662	Yes
17614	16:**10 X** vs. 36:**1 X**	-2.519	-2.663 to -2.375	Yes
17615	16:**10 X** vs. 36:**10 X**	-1.876	-2.020 to -1.732	Yes
17616	16:**10 X** vs. 36:**40 X**	-0.5723	-0.7163 to -0.4284	Yes
17617	16:**10 X** vs. 36:**50 X**	-0.4220	-0.5660 to -0.2780	Yes
17618	16:**10 X** vs. 37:**0 X**	-3.207	-3.351 to -3.063	Yes
17619	16:**10 X** vs. 37:**1 X**	-2.589	-2.733 to -2.445	Yes
17620	16:**10 X** vs. 37:**10 X**	-1.877	-2.021 to -1.733	Yes
17621	16:**10 X** vs. 37:**40 X**	-0.6097	-0.7536 to -0.4657	Yes
17622	16:**10 X** vs. 37:**50 X**	-0.4307	-0.5746 to -0.2867	Yes
17623	16:**10 X** vs. 38:**0 X**	-3.152	-3.296 to -3.008	Yes
17624	16:**10 X** vs. 38:**1 X**	-2.707	-2.851 to -2.563	Yes
17625	16:**10 X** vs. 38:**10 X**	-2.153	-2.297 to -2.009	Yes
17626	16:**10 X** vs. 38:**40 X**	-0.6140	-0.7580 to -0.4700	Yes
17627	16:**10 X** vs. 38:**50 X**	-0.4347	-0.5786 to -0.2907	Yes
17628	16:**10 X** vs. 39:**0 X**	-3.382	-3.526 to -3.238	Yes
17629	16:**10 X** vs. 39:**1 X**	-3.274	-3.418 to -3.130	Yes
17630	16:**10 X** vs. 39:**10 X**	-2.298	-2.442 to -2.154	Yes
17631	16:**10 X** vs. 39:**40 X**	-0.6200	-0.7640 to -0.4760	Yes
17632	16:**10 X** vs. 39:**50 X**	-0.4657	-0.6096 to -0.3217	Yes
17633	16:**10 X** vs. 40:**0 X**	-3.423	-3.567 to -3.279	Yes
17634	16:**10 X** vs. 40:**1 X**	-3.349	-3.493 to -3.205	Yes
17635	16:**10 X** vs. 40:**10 X**	-2.554	-2.698 to -2.410	Yes
17636	16:**10 X** vs. 40:**40 X**	-0.6247	-0.7686 to -0.4807	Yes
17637	16:**10 X** vs. 40:**50 X**	-0.4720	-0.6160 to -0.3280	Yes
17638	16:**10 X** vs. 41:**0 X**	-3.464	-3.608 to -3.320	Yes
17639	16:**10 X** vs. 41:**1 X**	-3.463	-3.607 to -3.319	Yes
17640	16:**10 X** vs. 41:**10 X**	-2.608	-2.752 to -2.464	Yes

2way ANOVA<br>Multiple comparisons	A<br>Data Set-A<br>Y	B<br>Data Set-B<br>Y	C<br>Data Set-C<br>Y
17641 16:10 X vs. 41:40 X	-0.5720	-0.7160 to -0.4280	Yes
17642 16:10 X vs. 41:50 X	-0.4847	-0.6286 to -0.3407	Yes
17643 16:10 X vs. 42:0 X	-3.606	-3.750 to -3.462	Yes
17644 16:10 X vs. 42:1 X	-3.626	-3.770 to -3.482	Yes
17645 16:10 X vs. 42:10 X	-2.608	-2.752 to -2.464	Yes
17646 16:10 X vs. 42:40 X	-0.5847	-0.7286 to -0.4407	Yes
17647 16:10 X vs. 42:50 X	-0.4880	-0.6320 to -0.3440	Yes
17648 16:10 X vs. 43:0 X	-3.696	-3.840 to -3.552	Yes
17649 16:10 X vs. 43:1 X	-3.610	-3.754 to -3.466	Yes
17650 16:10 X vs. 43:10 X	-3.326	-3.470 to -3.182	Yes
17651 16:10 X vs. 43:40 X	-0.5830	-0.7270 to -0.4390	Yes
17652 16:10 X vs. 43:50 X	-0.4883	-0.6323 to -0.3444	Yes
17653 16:10 X vs. 44:0 X	-3.720	-3.864 to -3.576	Yes
17654 16:10 X vs. 44:1 X	-3.610	-3.754 to -3.466	Yes
17655 16:10 X vs. 44:10 X	-3.389	-3.533 to -3.245	Yes
17656 16:10 X vs. 44:40 X	-0.5850	-0.7290 to -0.4410	Yes
17657 16:10 X vs. 44:50 X	-0.4207	-0.5646 to -0.2767	Yes
17658 16:10 X vs. 45:0 X	-3.585	-3.729 to -3.441	Yes
17659 16:10 X vs. 45:1 X	-3.387	-3.531 to -3.243	Yes
17660 16:10 X vs. 45:10 X	-3.269	-3.413 to -3.125	Yes
17661 16:10 X vs. 45:40 X	-0.5863	-0.7303 to -0.4424	Yes
17662 16:10 X vs. 45:50 X	-0.4177	-0.5616 to -0.2737	Yes
17663 16:10 X vs. 46:0 X	-3.389	-3.533 to -3.245	Yes
17664 16:10 X vs. 46:1 X	-3.356	-3.500 to -3.212	Yes
17665 16:10 X vs. 46:10 X	-3.264	-3.408 to -3.120	Yes
17666 16:10 X vs. 46:40 X	-0.6000	-0.7440 to -0.4560	Yes
17667 16:10 X vs. 46:50 X	-0.4107	-0.5546 to -0.2667	Yes
17668 16:10 X vs. 47:0 X	-3.365	-3.509 to -3.221	Yes
17669 16:10 X vs. 47:1 X	-3.269	-3.413 to -3.125	Yes
17670 16:10 X vs. 47:10 X	-3.250	-3.394 to -3.106	Yes
17671 16:10 X vs. 47:40 X	-0.6100	-0.7540 to -0.4660	Yes
17672 16:10 X vs. 47:50 X	-0.3687	-0.5126 to -0.2247	Yes
17673 16:10 X vs. 48:0 X	-3.352	-3.513 to -3.191	Yes
17674 16:10 X vs. 48:1 X	-3.256	-3.400 to -3.112	Yes
17675 16:10 X vs. 48:10 X	-3.250	-3.394 to -3.106	Yes
17676 16:10 X vs. 48:40 X	-0.6163	-0.7603 to -0.4724	Yes
17677 16:10 X vs. 48:50 X	-0.3760	-0.5200 to -0.2320	Yes
17678 16:10 X vs. 49:0 X	-3.352	-3.496 to -3.208	Yes
17679 16:10 X vs. 49:1 X	-3.256	-3.400 to -3.112	Yes
17680 16:10 X vs. 49:10 X	-3.250	-3.394 to -3.106	Yes
17681 16:10 X vs. 49:40 X	-0.6207	-0.7646 to -0.4767	Yes
17682 16:10 X vs. 49:50 X	-0.3753	-0.5193 to -0.2314	Yes
17683 16:10 X vs. 50:0 X	-3.352	-3.496 to -3.208	Yes
17684 16:10 X vs. 50:1 X	-3.256	-3.400 to -3.112	Yes
17685 16:10 X vs. 50:10 X	-3.250	-3.394 to -3.106	Yes

	2way ANOVA<br>Multiple comparisons	A<br>Data Set-A<br>Y	B<br>Data Set-B<br>Y	C<br>Data Set-C<br>Y
17686	16:10 X vs. 50:40 X	-0.6283	-0.7723 to -0.4844	Yes
17687	16:10 X vs. 50:50 X	-0.3750	-0.5190 to -0.2310	Yes
17688	16:40 X vs. 16:50 X	-0.04233	-0.1863 to 0.1016	No
17689	16:40 X vs. 17:0 X	-0.1167	-0.2606 to 0.02729	No
17690	16:40 X vs. 17:1 X	-0.0310	-0.1750 to 0.1130	No
17691	16:40 X vs. 17:10 X	-0.08767	-0.2316 to 0.05629	No
17692	16:40 X vs. 17:40 X	-0.0110	-0.1550 to 0.1330	No
17693	16:40 X vs. 17:50 X	-0.05633	-0.2003 to 0.08763	No
17694	16:40 X vs. 18:0 X	-0.1653	-0.3093 to -0.02137	Yes
17695	16:40 X vs. 18:1 X	-0.07367	-0.2176 to 0.07029	No
17696	16:40 X vs. 18:10 X	-0.1660	-0.3100 to -0.02204	Yes
17697	16:40 X vs. 18:40 X	-0.01533	-0.1593 to 0.1286	No
17698	16:40 X vs. 18:50 X	-0.06033	-0.2043 to 0.08363	No
17699	16:40 X vs. 19:0 X	-0.2003	-0.3443 to -0.05637	Yes
17700	16:40 X vs. 19:1 X	-0.1883	-0.3323 to -0.04437	Yes
17701	16:40 X vs. 19:10 X	-0.1860	-0.3300 to -0.04204	Yes
17702	16:40 X vs. 19:40 X	-0.0440	-0.1880 to 0.09996	No
17703	16:40 X vs. 19:50 X	-0.0550	-0.1990 to 0.08896	No
17704	16:40 X vs. 20:0 X	-0.2800	-0.4240 to -0.1360	Yes
17705	16:40 X vs. 20:1 X	-0.09933	-0.2433 to 0.04463	No
17706	16:40 X vs. 20:10 X	-0.1883	-0.3323 to -0.04437	Yes
17707	16:40 X vs. 20:40 X	-0.05567	-0.1996 to 0.08829	No
17708	16:40 X vs. 20:50 X	-0.09967	-0.2436 to 0.04429	No
17709	16:40 X vs. 21:0 X	-0.3033	-0.4473 to -0.1594	Yes
17710	16:40 X vs. 21:1 X	-0.2563	-0.4003 to -0.1124	Yes
17711	16:40 X vs. 21:10 X	-0.2533	-0.3973 to -0.1094	Yes
17712	16:40 X vs. 21:40 X	-0.1287	-0.2726 to 0.01529	No
17713	16:40 X vs. 21:50 X	-0.2190	-0.3630 to -0.07504	Yes
17714	16:40 X vs. 22:0 X	-0.3450	-0.4890 to -0.2010	Yes
17715	16:40 X vs. 22:1 X	-0.3397	-0.4836 to -0.1957	Yes
17716	16:40 X vs. 22:10 X	-0.3350	-0.4790 to -0.1910	Yes
17717	16:40 X vs. 22:40 X	-0.2353	-0.3793 to -0.09137	Yes
17718	16:40 X vs. 22:50 X	-0.2980	-0.4420 to -0.1540	Yes
17719	16:40 X vs. 23:0 X	-0.4103	-0.5543 to -0.2664	Yes
17720	16:40 X vs. 23:1 X	-0.6000	-0.7610 to -0.4390	Yes
17721	16:40 X vs. 23:10 X	-0.3497	-0.4936 to -0.2057	Yes
17722	16:40 X vs. 23:40 X	-0.2300	-0.3910 to -0.06905	Yes
17723	16:40 X vs. 23:50 X	-0.2640	-0.4080 to -0.1200	Yes
17724	16:40 X vs. 24:0 X	-0.5277	-0.6716 to -0.3837	Yes
17725	16:40 X vs. 24:1 X	-0.6840	-0.8450 to -0.5230	Yes
17726	16:40 X vs. 24:10 X	-0.5115	-0.6725 to -0.3505	Yes
17727	16:40 X vs. 24:40 X	-0.2463	-0.3903 to -0.1024	Yes
17728	16:40 X vs. 24:50 X	-0.1680	-0.3120 to -0.02404	Yes
17729	16:40 X vs. 25:0 X	-0.8650	-1.026 to -0.7040	Yes
17730	16:40 X vs. 25:1 X	-1.055	-1.215 to -0.8935	Yes

2way ANOVA<br>Multiple comparisons	A<br>Data Set-A<br>Y	B<br>Data Set-B<br>Y	C<br>Data Set-C<br>Y
17731 16:40 X vs. 25:10 X	-0.4160	-0.6196 to -0.2124	Yes
17732 16:40 X vs. 25:40 X	-0.2483	-0.3923 to -0.1044	Yes
17733 16:40 X vs. 25:50 X	-0.2227	-0.3666 to -0.07871	Yes
17734 16:40 X vs. 26:0 X	-1.078	-1.238 to -0.9165	Yes
17735 16:40 X vs. 26:1 X	-1.191	-1.351 to -1.030	Yes
17736 16:40 X vs. 26:10 X	-0.8770	-1.038 to -0.7160	Yes
17737 16:40 X vs. 26:40 X	-0.3227	-0.4666 to -0.1787	Yes
17738 16:40 X vs. 26:50 X	-0.1830	-0.3270 to -0.03904	Yes
17739 16:40 X vs. 27:0 X	-1.265	-1.409 to -1.121	Yes
17740 16:40 X vs. 27:1 X	-1.356	-1.517 to -1.195	Yes
17741 16:40 X vs. 27:10 X	-1.168	-1.329 to -1.007	Yes
17742 16:40 X vs. 27:40 X	-0.3227	-0.4666 to -0.1787	Yes
17743 16:40 X vs. 27:50 X	-0.1823	-0.3263 to -0.03837	Yes
17744 16:40 X vs. 28:0 X	-1.215	-1.375 to -1.054	Yes
17745 16:40 X vs. 28:1 X	-1.255	-1.399 to -1.111	Yes
17746 16:40 X vs. 28:10 X	-1.057	-1.218 to -0.8960	Yes
17747 16:40 X vs. 28:40 X	-0.3350	-0.4790 to -0.1910	Yes
17748 16:40 X vs. 28:50 X	-0.2303	-0.3743 to -0.08637	Yes
17749 16:40 X vs. 29:0 X	-1.696	-1.857 to -1.535	Yes
17750 16:40 X vs. 29:1 X	-1.781	-1.942 to -1.620	Yes
17751 16:40 X vs. 29:10 X	-0.9955	-1.156 to -0.8345	Yes
17752 16:40 X vs. 29:40 X	-0.4430	-0.5870 to -0.2990	Yes
17753 16:40 X vs. 29:50 X	-0.2317	-0.3756 to -0.08771	Yes
17754 16:40 X vs. 30:0 X	-1.411	-1.571 to -1.250	Yes
17755 16:40 X vs. 30:1 X	-1.732	-1.893 to -1.571	Yes
17756 16:40 X vs. 30:10 X	-1.070	-1.231 to -0.9090	Yes
17757 16:40 X vs. 30:40 X	-0.3700	-0.5140 to -0.2260	Yes
17758 16:40 X vs. 30:50 X	-0.2840	-0.4280 to -0.1400	Yes
17759 16:40 X vs. 31:0 X	-1.729	-1.889 to -1.568	Yes
17760 16:40 X vs. 31:1 X	-1.719	-1.880 to -1.558	Yes
17761 16:40 X vs. 31:10 X	-1.202	-1.362 to -1.041	Yes
17762 16:40 X vs. 31:40 X	-0.4617	-0.6056 to -0.3177	Yes
17763 16:40 X vs. 31:50 X	-0.2630	-0.4070 to -0.1190	Yes
17764 16:40 X vs. 32:0 X	-2.320	-2.464 to -2.176	Yes
17765 16:40 X vs. 32:1 X	-1.776	-1.937 to -1.615	Yes
17766 16:40 X vs. 32:10 X	-1.632	-1.776 to -1.488	Yes
17767 16:40 X vs. 32:40 X	-0.4580	-0.6020 to -0.3140	Yes
17768 16:40 X vs. 32:50 X	-0.2627	-0.4066 to -0.1187	Yes
17769 16:40 X vs. 33:0 X	-2.324	-2.468 to -2.180	Yes
17770 16:40 X vs. 33:1 X	-2.138	-2.282 to -1.994	Yes
17771 16:40 X vs. 33:10 X	-1.799	-1.943 to -1.655	Yes
17772 16:40 X vs. 33:40 X	-0.4767	-0.6206 to -0.3327	Yes
17773 16:40 X vs. 33:50 X	-0.2777	-0.4216 to -0.1337	Yes
17774 16:40 X vs. 34:0 X	-2.248	-2.392 to -2.104	Yes
17775 16:40 X vs. 34:1 X	-2.256	-2.400 to -2.112	Yes

	2way ANOVA<br>Multiple comparisons	A<br>Data Set-A<br>Y	B<br>Data Set-B<br>Y	C<br>Data Set-C<br>Y
17776	16:40 X vs. 34:10 X	-1.889	-2.033 to -1.745	Yes
17777	16:40 X vs. 34:40 X	-0.4887	-0.6326 to -0.3447	Yes
17778	16:40 X vs. 34:50 X	-0.4267	-0.5706 to -0.2827	Yes
17779	16:40 X vs. 35:0 X	-2.597	-2.741 to -2.453	Yes
17780	16:40 X vs. 35:1 X	-2.363	-2.507 to -2.219	Yes
17781	16:40 X vs. 35:10 X	-1.851	-1.995 to -1.707	Yes
17782	16:40 X vs. 35:40 X	-0.4977	-0.6416 to -0.3537	Yes
17783	16:40 X vs. 35:50 X	-0.4393	-0.5833 to -0.2954	Yes
17784	16:40 X vs. 36:0 X	-2.804	-2.964 to -2.643	Yes
17785	16:40 X vs. 36:1 X	-2.499	-2.643 to -2.355	Yes
17786	16:40 X vs. 36:10 X	-1.856	-2.000 to -1.712	Yes
17787	16:40 X vs. 36:40 X	-0.5530	-0.6970 to -0.4090	Yes
17788	16:40 X vs. 36:50 X	-0.4027	-0.5466 to -0.2587	Yes
17789	16:40 X vs. 37:0 X	-3.187	-3.331 to -3.043	Yes
17790	16:40 X vs. 37:1 X	-2.569	-2.713 to -2.425	Yes
17791	16:40 X vs. 37:10 X	-1.858	-2.002 to -1.714	Yes
17792	16:40 X vs. 37:40 X	-0.5903	-0.7343 to -0.4464	Yes
17793	16:40 X vs. 37:50 X	-0.4113	-0.5553 to -0.2674	Yes
17794	16:40 X vs. 38:0 X	-3.132	-3.276 to -2.988	Yes
17795	16:40 X vs. 38:1 X	-2.688	-2.832 to -2.544	Yes
17796	16:40 X vs. 38:10 X	-2.133	-2.277 to -1.989	Yes
17797	16:40 X vs. 38:40 X	-0.5947	-0.7386 to -0.4507	Yes
17798	16:40 X vs. 38:50 X	-0.4153	-0.5593 to -0.2714	Yes
17799	16:40 X vs. 39:0 X	-3.363	-3.507 to -3.219	Yes
17800	16:40 X vs. 39:1 X	-3.255	-3.399 to -3.111	Yes
17801	16:40 X vs. 39:10 X	-2.278	-2.422 to -2.134	Yes
17802	16:40 X vs. 39:40 X	-0.6007	-0.7446 to -0.4567	Yes
17803	16:40 X vs. 39:50 X	-0.4463	-0.5903 to -0.3024	Yes
17804	16:40 X vs. 40:0 X	-3.404	-3.548 to -3.260	Yes
17805	16:40 X vs. 40:1 X	-3.330	-3.474 to -3.186	Yes
17806	16:40 X vs. 40:10 X	-2.535	-2.679 to -2.391	Yes
17807	16:40 X vs. 40:40 X	-0.6053	-0.7493 to -0.4614	Yes
17808	16:40 X vs. 40:50 X	-0.4527	-0.5966 to -0.3087	Yes
17809	16:40 X vs. 41:0 X	-3.445	-3.589 to -3.301	Yes
17810	16:40 X vs. 41:1 X	-3.444	-3.588 to -3.300	Yes
17811	16:40 X vs. 41:10 X	-2.588	-2.732 to -2.444	Yes
17812	16:40 X vs. 41:40 X	-0.5527	-0.6966 to -0.4087	Yes
17813	16:40 X vs. 41:50 X	-0.4653	-0.6093 to -0.3214	Yes
17814	16:40 X vs. 42:0 X	-3.587	-3.731 to -3.443	Yes
17815	16:40 X vs. 42:1 X	-3.606	-3.750 to -3.462	Yes
17816	16:40 X vs. 42:10 X	-2.589	-2.733 to -2.445	Yes
17817	16:40 X vs. 42:40 X	-0.5653	-0.7093 to -0.4214	Yes
17818	16:40 X vs. 42:50 X	-0.4687	-0.6126 to -0.3247	Yes
17819	16:40 X vs. 43:0 X	-3.677	-3.821 to -3.533	Yes
17820	16:40 X vs. 43:1 X	-3.591	-3.735 to -3.447	Yes

	2way ANOVA<br>Multiple comparisons	A<br>Data Set-A<br>Y	B<br>Data Set-B<br>Y	C<br>Data Set-C<br>Y
17821	16:**40 X** vs. 43:**10 X**	-3.307	-3.451 to -3.163	Yes
17822	16:**40 X** vs. 43:**40 X**	-0.5637	-0.7076 to -0.4197	Yes
17823	16:**40 X** vs. 43:**50 X**	-0.4690	-0.6130 to -0.3250	Yes
17824	16:**40 X** vs. 44:**0 X**	-3.701	-3.845 to -3.557	Yes
17825	16:**40 X** vs. 44:**1 X**	-3.590	-3.734 to -3.446	Yes
17826	16:**40 X** vs. 44:**10 X**	-3.370	-3.514 to -3.226	Yes
17827	16:**40 X** vs. 44:**40 X**	-0.5657	-0.7096 to -0.4217	Yes
17828	16:**40 X** vs. 44:**50 X**	-0.4013	-0.5453 to -0.2574	Yes
17829	16:**40 X** vs. 45:**0 X**	-3.565	-3.709 to -3.421	Yes
17830	16:**40 X** vs. 45:**1 X**	-3.367	-3.511 to -3.223	Yes
17831	16:**40 X** vs. 45:**10 X**	-3.250	-3.394 to -3.106	Yes
17832	16:**40 X** vs. 45:**40 X**	-0.5670	-0.7110 to -0.4230	Yes
17833	16:**40 X** vs. 45:**50 X**	-0.3983	-0.5423 to -0.2544	Yes
17834	16:**40 X** vs. 46:**0 X**	-3.370	-3.514 to -3.226	Yes
17835	16:**40 X** vs. 46:**1 X**	-3.337	-3.481 to -3.193	Yes
17836	16:**40 X** vs. 46:**10 X**	-3.245	-3.389 to -3.101	Yes
17837	16:**40 X** vs. 46:**40 X**	-0.5807	-0.7246 to -0.4367	Yes
17838	16:**40 X** vs. 46:**50 X**	-0.3913	-0.5353 to -0.2474	Yes
17839	16:**40 X** vs. 47:**0 X**	-3.345	-3.489 to -3.201	Yes
17840	16:**40 X** vs. 47:**1 X**	-3.249	-3.393 to -3.105	Yes
17841	16:**40 X** vs. 47:**10 X**	-3.231	-3.375 to -3.087	Yes
17842	16:**40 X** vs. 47:**40 X**	-0.5907	-0.7346 to -0.4467	Yes
17843	16:**40 X** vs. 47:**50 X**	-0.3493	-0.4933 to -0.2054	Yes
17844	16:**40 X** vs. 48:**0 X**	-3.333	-3.493 to -3.172	Yes
17845	16:**40 X** vs. 48:**1 X**	-3.236	-3.380 to -3.092	Yes
17846	16:**40 X** vs. 48:**10 X**	-3.231	-3.375 to -3.087	Yes
17847	16:**40 X** vs. 48:**40 X**	-0.5970	-0.7410 to -0.4530	Yes
17848	16:**40 X** vs. 48:**50 X**	-0.3567	-0.5006 to -0.2127	Yes
17849	16:**40 X** vs. 49:**0 X**	-3.333	-3.477 to -3.189	Yes
17850	16:**40 X** vs. 49:**1 X**	-3.236	-3.380 to -3.092	Yes
17851	16:**40 X** vs. 49:**10 X**	-3.231	-3.375 to -3.087	Yes
17852	16:**40 X** vs. 49:**40 X**	-0.6013	-0.7453 to -0.4574	Yes
17853	16:**40 X** vs. 49:**50 X**	-0.3560	-0.5000 to -0.2120	Yes
17854	16:**40 X** vs. 50:**0 X**	-3.333	-3.477 to -3.189	Yes
17855	16:**40 X** vs. 50:**1 X**	-3.236	-3.380 to -3.092	Yes
17856	16:**40 X** vs. 50:**10 X**	-3.231	-3.375 to -3.087	Yes
17857	16:**40 X** vs. 50:**40 X**	-0.6090	-0.7530 to -0.4650	Yes
17858	16:**40 X** vs. 50:**50 X**	-0.3557	-0.4996 to -0.2117	Yes
17859	16:**50 X** vs. 17:**0 X**	-0.07433	-0.2183 to 0.06963	No
17860	16:**50 X** vs. 17:**1 X**	0.01133	-0.1326 to 0.1553	No
17861	16:**50 X** vs. 17:**10 X**	-0.04533	-0.1893 to 0.09863	No
17862	16:**50 X** vs. 17:**40 X**	0.03133	-0.1126 to 0.1753	No
17863	16:**50 X** vs. 17:**50 X**	-0.0140	-0.1580 to 0.1300	No
17864	16:**50 X** vs. 18:**0 X**	-0.1230	-0.2670 to 0.02096	No
17865	16:**50 X** vs. 18:**1 X**	-0.03133	-0.1753 to 0.1126	No

2way ANOVA<br>Multiple comparisons	A<br>Data Set-A<br>Y	B<br>Data Set-B<br>Y	C<br>Data Set-C<br>Y
17866 16:50 X vs. 18:10 X	-0.1237	-0.2676 to 0.02029	No
17867 16:50 X vs. 18:40 X	0.0270	-0.1170 to 0.1710	No
17868 16:50 X vs. 18:50 X	-0.01800	-0.1620 to 0.1260	No
17869 16:50 X vs. 19:0 X	-0.1580	-0.3020 to -0.01404	Yes
17870 16:50 X vs. 19:1 X	-0.1460	-0.2900 to -0.002040	Yes
17871 16:50 X vs. 19:10 X	-0.1437	-0.2876 to 0.0002932	No
17872 16:50 X vs. 19:40 X	-0.001667	-0.1456 to 0.1423	No
17873 16:50 X vs. 19:50 X	-0.01267	-0.1566 to 0.1313	No
17874 16:50 X vs. 20:0 X	-0.2377	-0.3816 to -0.09371	Yes
17875 16:50 X vs. 20:1 X	-0.05700	-0.2010 to 0.08696	No
17876 16:50 X vs. 20:10 X	-0.1460	-0.2900 to -0.002040	Yes
17877 16:50 X vs. 20:40 X	-0.01333	-0.1573 to 0.1306	No
17878 16:50 X vs. 20:50 X	-0.05733	-0.2013 to 0.08663	No
17879 16:50 X vs. 21:0 X	-0.2610	-0.4050 to -0.1170	Yes
17880 16:50 X vs. 21:1 X	-0.2140	-0.3580 to -0.07004	Yes
17881 16:50 X vs. 21:10 X	-0.2110	-0.3550 to -0.06704	Yes
17882 16:50 X vs. 21:40 X	-0.08633	-0.2303 to 0.05763	No
17883 16:50 X vs. 21:50 X	-0.1767	-0.3206 to -0.03271	Yes
17884 16:50 X vs. 22:0 X	-0.3027	-0.4466 to -0.1587	Yes
17885 16:50 X vs. 22:1 X	-0.2973	-0.4413 to -0.1534	Yes
17886 16:50 X vs. 22:10 X	-0.2927	-0.4366 to -0.1487	Yes
17887 16:50 X vs. 22:40 X	-0.1930	-0.3370 to -0.04904	Yes
17888 16:50 X vs. 22:50 X	-0.2557	-0.3996 to -0.1117	Yes
17889 16:50 X vs. 23:0 X	-0.3680	-0.5120 to -0.2240	Yes
17890 16:50 X vs. 23:1 X	-0.5577	-0.7186 to -0.3967	Yes
17891 16:50 X vs. 23:10 X	-0.3073	-0.4513 to -0.1634	Yes
17892 16:50 X vs. 23:40 X	-0.1877	-0.3486 to -0.02671	Yes
17893 16:50 X vs. 23:50 X	-0.2217	-0.3656 to -0.07771	Yes
17894 16:50 X vs. 24:0 X	-0.4853	-0.6293 to -0.3414	Yes
17895 16:50 X vs. 24:1 X	-0.6417	-0.8026 to -0.4807	Yes
17896 16:50 X vs. 24:10 X	-0.4692	-0.6301 to -0.3082	Yes
17897 16:50 X vs. 24:40 X	-0.2040	-0.3480 to -0.06004	Yes
17898 16:50 X vs. 24:50 X	-0.1257	-0.2696 to 0.01829	No
17899 16:50 X vs. 25:0 X	-0.8227	-0.9836 to -0.6617	Yes
17900 16:50 X vs. 25:1 X	-1.012	-1.173 to -0.8512	Yes
17901 16:50 X vs. 25:10 X	-0.3737	-0.5773 to -0.1701	Yes
17902 16:50 X vs. 25:40 X	-0.2060	-0.3500 to -0.06204	Yes
17903 16:50 X vs. 25:50 X	-0.1803	-0.3243 to -0.03637	Yes
17904 16:50 X vs. 26:0 X	-1.035	-1.196 to -0.8742	Yes
17905 16:50 X vs. 26:1 X	-1.148	-1.309 to -0.9872	Yes
17906 16:50 X vs. 26:10 X	-0.8347	-0.9956 to -0.6737	Yes
17907 16:50 X vs. 26:40 X	-0.2803	-0.4243 to -0.1364	Yes
17908 16:50 X vs. 26:50 X	-0.1407	-0.2846 to 0.003293	No
17909 16:50 X vs. 27:0 X	-1.223	-1.367 to -1.079	Yes
17910 16:50 X vs. 27:1 X	-1.314	-1.475 to -1.153	Yes

	2way ANOVA<br>Multiple comparisons	A<br>Data Set-A<br>Y	B<br>Data Set-B<br>Y	C<br>Data Set-C<br>Y
17911	16:50 X vs. 27:10 X	-1.126	-1.287 to -0.9647	Yes
17912	16:50 X vs. 27:40 X	-0.2803	-0.4243 to -0.1364	Yes
17913	16:50 X vs. 27:50 X	-0.1400	-0.2840 to 0.003960	No
17914	16:50 X vs. 28:0 X	-1.172	-1.333 to -1.011	Yes
17915	16:50 X vs. 28:1 X	-1.213	-1.357 to -1.069	Yes
17916	16:50 X vs. 28:10 X	-1.015	-1.176 to -0.8537	Yes
17917	16:50 X vs. 28:40 X	-0.2927	-0.4366 to -0.1487	Yes
17918	16:50 X vs. 28:50 X	-0.1880	-0.3320 to -0.04404	Yes
17919	16:50 X vs. 29:0 X	-1.654	-1.815 to -1.493	Yes
17920	16:50 X vs. 29:1 X	-1.739	-1.900 to -1.578	Yes
17921	16:50 X vs. 29:10 X	-0.9532	-1.114 to -0.7922	Yes
17922	16:50 X vs. 29:40 X	-0.4007	-0.5446 to -0.2567	Yes
17923	16:50 X vs. 29:50 X	-0.1893	-0.3333 to -0.04537	Yes
17924	16:50 X vs. 30:0 X	-1.368	-1.529 to -1.207	Yes
17925	16:50 X vs. 30:1 X	-1.690	-1.851 to -1.529	Yes
17926	16:50 X vs. 30:10 X	-1.028	-1.189 to -0.8667	Yes
17927	16:50 X vs. 30:40 X	-0.3277	-0.4716 to -0.1837	Yes
17928	16:50 X vs. 30:50 X	-0.2417	-0.3856 to -0.09771	Yes
17929	16:50 X vs. 31:0 X	-1.686	-1.847 to -1.525	Yes
17930	16:50 X vs. 31:1 X	-1.677	-1.838 to -1.516	Yes
17931	16:50 X vs. 31:10 X	-1.159	-1.320 to -0.9982	Yes
17932	16:50 X vs. 31:40 X	-0.4193	-0.5633 to -0.2754	Yes
17933	16:50 X vs. 31:50 X	-0.2207	-0.3646 to -0.07671	Yes
17934	16:50 X vs. 32:0 X	-2.278	-2.422 to -2.134	Yes
17935	16:50 X vs. 32:1 X	-1.734	-1.895 to -1.573	Yes
17936	16:50 X vs. 32:10 X	-1.590	-1.734 to -1.446	Yes
17937	16:50 X vs. 32:40 X	-0.4157	-0.5596 to -0.2717	Yes
17938	16:50 X vs. 32:50 X	-0.2203	-0.3643 to -0.07637	Yes
17939	16:50 X vs. 33:0 X	-2.281	-2.425 to -2.137	Yes
17940	16:50 X vs. 33:1 X	-2.096	-2.240 to -1.952	Yes
17941	16:50 X vs. 33:10 X	-1.756	-1.900 to -1.612	Yes
17942	16:50 X vs. 33:40 X	-0.4343	-0.5783 to -0.2904	Yes
17943	16:50 X vs. 33:50 X	-0.2353	-0.3793 to -0.09137	Yes
17944	16:50 X vs. 34:0 X	-2.206	-2.350 to -2.062	Yes
17945	16:50 X vs. 34:1 X	-2.213	-2.357 to -2.069	Yes
17946	16:50 X vs. 34:10 X	-1.847	-1.991 to -1.703	Yes
17947	16:50 X vs. 34:40 X	-0.4463	-0.5903 to -0.3024	Yes
17948	16:50 X vs. 34:50 X	-0.3843	-0.5283 to -0.2404	Yes
17949	16:50 X vs. 35:0 X	-2.555	-2.699 to -2.411	Yes
17950	16:50 X vs. 35:1 X	-2.320	-2.464 to -2.176	Yes
17951	16:50 X vs. 35:10 X	-1.808	-1.952 to -1.664	Yes
17952	16:50 X vs. 35:40 X	-0.4553	-0.5993 to -0.3114	Yes
17953	16:50 X vs. 35:50 X	-0.3970	-0.5410 to -0.2530	Yes
17954	16:50 X vs. 36:0 X	-2.761	-2.922 to -2.600	Yes
17955	16:50 X vs. 36:1 X	-2.457	-2.601 to -2.313	Yes

	2way ANOVA<br>Multiple comparisons	A<br>Data Set-A<br>Y	B<br>Data Set-B<br>Y	C<br>Data Set-C<br>Y
17956	16:50 X vs. 36:10 X	-1.814	-1.958 to -1.670	Yes
17957	16:50 X vs. 36:40 X	-0.5107	-0.6546 to -0.3667	Yes
17958	16:50 X vs. 36:50 X	-0.3603	-0.5043 to -0.2164	Yes
17959	16:50 X vs. 37:0 X	-3.145	-3.289 to -3.001	Yes
17960	16:50 X vs. 37:1 X	-2.527	-2.671 to -2.383	Yes
17961	16:50 X vs. 37:10 X	-1.816	-1.960 to -1.672	Yes
17962	16:50 X vs. 37:40 X	-0.5480	-0.6920 to -0.4040	Yes
17963	16:50 X vs. 37:50 X	-0.3690	-0.5130 to -0.2250	Yes
17964	16:50 X vs. 38:0 X	-3.090	-3.234 to -2.946	Yes
17965	16:50 X vs. 38:1 X	-2.646	-2.790 to -2.502	Yes
17966	16:50 X vs. 38:10 X	-2.091	-2.235 to -1.947	Yes
17967	16:50 X vs. 38:40 X	-0.5523	-0.6963 to -0.4084	Yes
17968	16:50 X vs. 38:50 X	-0.3730	-0.5170 to -0.2290	Yes
17969	16:50 X vs. 39:0 X	-3.321	-3.465 to -3.177	Yes
17970	16:50 X vs. 39:1 X	-3.212	-3.356 to -3.068	Yes
17971	16:50 X vs. 39:10 X	-2.236	-2.380 to -2.092	Yes
17972	16:50 X vs. 39:40 X	-0.5583	-0.7023 to -0.4144	Yes
17973	16:50 X vs. 39:50 X	-0.4040	-0.5480 to -0.2600	Yes
17974	16:50 X vs. 40:0 X	-3.362	-3.506 to -3.218	Yes
17975	16:50 X vs. 40:1 X	-3.288	-3.432 to -3.144	Yes
17976	16:50 X vs. 40:10 X	-2.492	-2.636 to -2.348	Yes
17977	16:50 X vs. 40:40 X	-0.5630	-0.7070 to -0.4190	Yes
17978	16:50 X vs. 40:50 X	-0.4103	-0.5543 to -0.2664	Yes
17979	16:50 X vs. 41:0 X	-3.403	-3.547 to -3.259	Yes
17980	16:50 X vs. 41:1 X	-3.402	-3.546 to -3.258	Yes
17981	16:50 X vs. 41:10 X	-2.546	-2.690 to -2.402	Yes
17982	16:50 X vs. 41:40 X	-0.5103	-0.6543 to -0.3664	Yes
17983	16:50 X vs. 41:50 X	-0.4230	-0.5670 to -0.2790	Yes
17984	16:50 X vs. 42:0 X	-3.544	-3.688 to -3.400	Yes
17985	16:50 X vs. 42:1 X	-3.564	-3.708 to -3.420	Yes
17986	16:50 X vs. 42:10 X	-2.546	-2.690 to -2.402	Yes
17987	16:50 X vs. 42:40 X	-0.5230	-0.6670 to -0.3790	Yes
17988	16:50 X vs. 42:50 X	-0.4263	-0.5703 to -0.2824	Yes
17989	16:50 X vs. 43:0 X	-3.634	-3.778 to -3.490	Yes
17990	16:50 X vs. 43:1 X	-3.549	-3.693 to -3.405	Yes
17991	16:50 X vs. 43:10 X	-3.264	-3.408 to -3.120	Yes
17992	16:50 X vs. 43:40 X	-0.5213	-0.6653 to -0.3774	Yes
17993	16:50 X vs. 43:50 X	-0.4267	-0.5706 to -0.2827	Yes
17994	16:50 X vs. 44:0 X	-3.658	-3.802 to -3.514	Yes
17995	16:50 X vs. 44:1 X	-3.548	-3.692 to -3.404	Yes
17996	16:50 X vs. 44:10 X	-3.328	-3.472 to -3.184	Yes
17997	16:50 X vs. 44:40 X	-0.5233	-0.6673 to -0.3794	Yes
17998	16:50 X vs. 44:50 X	-0.3590	-0.5030 to -0.2150	Yes
17999	16:50 X vs. 45:0 X	-3.523	-3.667 to -3.379	Yes
18000	16:50 X vs. 45:1 X	-3.325	-3.469 to -3.181	Yes

2way ANOVA<br>Multiple comparisons	A<br>Data Set-A<br>Y	B<br>Data Set-B<br>Y	C<br>Data Set-C<br>Y
18001 16:50 X vs. 45:10 X	-3.207	-3.351 to -3.063	Yes
18002 16:50 X vs. 45:40 X	-0.5247	-0.6686 to -0.3807	Yes
18003 16:50 X vs. 45:50 X	-0.3560	-0.5000 to -0.2120	Yes
18004 16:50 X vs. 46:0 X	-3.327	-3.471 to -3.183	Yes
18005 16:50 X vs. 46:1 X	-3.294	-3.438 to -3.150	Yes
18006 16:50 X vs. 46:10 X	-3.202	-3.346 to -3.058	Yes
18007 16:50 X vs. 46:40 X	-0.5383	-0.6823 to -0.3944	Yes
18008 16:50 X vs. 46:50 X	-0.3490	-0.4930 to -0.2050	Yes
18009 16:50 X vs. 47:0 X	-3.303	-3.447 to -3.159	Yes
18010 16:50 X vs. 47:1 X	-3.207	-3.351 to -3.063	Yes
18011 16:50 X vs. 47:10 X	-3.188	-3.332 to -3.044	Yes
18012 16:50 X vs. 47:40 X	-0.5483	-0.6923 to -0.4044	Yes
18013 16:50 X vs. 47:50 X	-0.3070	-0.4510 to -0.1630	Yes
18014 16:50 X vs. 48:0 X	-3.290	-3.451 to -3.129	Yes
18015 16:50 X vs. 48:1 X	-3.194	-3.338 to -3.050	Yes
18016 16:50 X vs. 48:10 X	-3.188	-3.332 to -3.044	Yes
18017 16:50 X vs. 48:40 X	-0.5547	-0.6986 to -0.4107	Yes
18018 16:50 X vs. 48:50 X	-0.3143	-0.4583 to -0.1704	Yes
18019 16:50 X vs. 49:0 X	-3.290	-3.434 to -3.146	Yes
18020 16:50 X vs. 49:1 X	-3.194	-3.338 to -3.050	Yes
18021 16:50 X vs. 49:10 X	-3.188	-3.332 to -3.044	Yes
18022 16:50 X vs. 49:40 X	-0.5590	-0.7030 to -0.4150	Yes
18023 16:50 X vs. 49:50 X	-0.3137	-0.4576 to -0.1697	Yes
18024 16:50 X vs. 50:0 X	-3.290	-3.434 to -3.146	Yes
18025 16:50 X vs. 50:1 X	-3.194	-3.338 to -3.050	Yes
18026 16:50 X vs. 50:10 X	-3.188	-3.332 to -3.044	Yes
18027 16:50 X vs. 50:40 X	-0.5667	-0.7106 to -0.4227	Yes
18028 16:50 X vs. 50:50 X	-0.3133	-0.4573 to -0.1694	Yes
18029 17:0 X vs. 17:1 X	0.08567	-0.05829 to 0.2296	No
18030 17:0 X vs. 17:10 X	0.0290	-0.1150 to 0.1730	No
18031 17:0 X vs. 17:40 X	0.1057	-0.03829 to 0.2496	No
18032 17:0 X vs. 17:50 X	0.06033	-0.08363 to 0.2043	No
18033 17:0 X vs. 18:0 X	-0.04867	-0.1926 to 0.09529	No
18034 17:0 X vs. 18:1 X	0.0430	-0.1010 to 0.1870	No
18035 17:0 X vs. 18:10 X	-0.04933	-0.1933 to 0.09463	No
18036 17:0 X vs. 18:40 X	0.1013	-0.04263 to 0.2453	No
18037 17:0 X vs. 18:50 X	0.05633	-0.08763 to 0.2003	No
18038 17:0 X vs. 19:0 X	-0.08367	-0.2276 to 0.06029	No
18039 17:0 X vs. 19:1 X	-0.07167	-0.2156 to 0.07229	No
18040 17:0 X vs. 19:10 X	-0.06933	-0.2133 to 0.07463	No
18041 17:0 X vs. 19:40 X	0.07267	-0.07129 to 0.2166	No
18042 17:0 X vs. 19:50 X	0.06167	-0.08229 to 0.2056	No
18043 17:0 X vs. 20:0 X	-0.1633	-0.3073 to -0.01937	Yes
18044 17:0 X vs. 20:1 X	0.01733	-0.1266 to 0.1613	No
18045 17:0 X vs. 20:10 X	-0.07167	-0.2156 to 0.07229	No

	2way ANOVA<br>Multiple comparisons	A<br>Data Set-A<br>Y	B<br>Data Set-B<br>Y	C<br>Data Set-C<br>Y
18046	17:**0 X** vs. 20:**40 X**	0.0610	-0.08296 to 0.2050	No
18047	17:**0 X** vs. 20:**50 X**	0.0170	-0.1270 to 0.1610	No
18048	17:**0 X** vs. 21:**0 X**	-0.1867	-0.3306 to -0.04271	Yes
18049	17:**0 X** vs. 21:**1 X**	-0.1397	-0.2836 to 0.004293	No
18050	17:**0 X** vs. 21:**10 X**	-0.1367	-0.2806 to 0.007293	No
18051	17:**0 X** vs. 21:**40 X**	-0.0120	-0.1560 to 0.1320	No
18052	17:**0 X** vs. 21:**50 X**	-0.1023	-0.2463 to 0.04163	No
18053	17:**0 X** vs. 22:**0 X**	-0.2283	-0.3723 to -0.08437	Yes
18054	17:**0 X** vs. 22:**1 X**	-0.2230	-0.3670 to -0.07904	Yes
18055	17:**0 X** vs. 22:**10 X**	-0.2183	-0.3623 to -0.07437	Yes
18056	17:**0 X** vs. 22:**40 X**	-0.1187	-0.2626 to 0.02529	No
18057	17:**0 X** vs. 22:**50 X**	-0.1813	-0.3253 to -0.03737	Yes
18058	17:**0 X** vs. 23:**0 X**	-0.2937	-0.4376 to -0.1497	Yes
18059	17:**0 X** vs. 23:**1 X**	-0.4833	-0.6443 to -0.3224	Yes
18060	17:**0 X** vs. 23:**10 X**	-0.2330	-0.3770 to -0.08904	Yes
18061	17:**0 X** vs. 23:**40 X**	-0.1133	-0.2743 to 0.04762	No
18062	17:**0 X** vs. 23:**50 X**	-0.1473	-0.2913 to -0.003374	Yes
18063	17:**0 X** vs. 24:**0 X**	-0.4110	-0.5550 to -0.2670	Yes
18064	17:**0 X** vs. 24:**1 X**	-0.5673	-0.7283 to -0.4064	Yes
18065	17:**0 X** vs. 24:**10 X**	-0.3948	-0.5558 to -0.2339	Yes
18066	17:**0 X** vs. 24:**40 X**	-0.1297	-0.2736 to 0.01429	No
18067	17:**0 X** vs. 24:**50 X**	-0.05133	-0.1953 to 0.09263	No
18068	17:**0 X** vs. 25:**0 X**	-0.7483	-0.9093 to -0.5874	Yes
18069	17:**0 X** vs. 25:**1 X**	-0.9378	-1.099 to -0.7769	Yes
18070	17:**0 X** vs. 25:**10 X**	-0.2993	-0.5029 to -0.09574	Yes
18071	17:**0 X** vs. 25:**40 X**	-0.1317	-0.2756 to 0.01229	No
18072	17:**0 X** vs. 25:**50 X**	-0.1060	-0.2500 to 0.03796	No
18073	17:**0 X** vs. 26:**0 X**	-0.9608	-1.122 to -0.7999	Yes
18074	17:**0 X** vs. 26:**1 X**	-1.074	-1.235 to -0.9129	Yes
18075	17:**0 X** vs. 26:**10 X**	-0.7603	-0.9213 to -0.5994	Yes
18076	17:**0 X** vs. 26:**40 X**	-0.2060	-0.3500 to -0.06204	Yes
18077	17:**0 X** vs. 26:**50 X**	-0.06633	-0.2103 to 0.07763	No
18078	17:**0 X** vs. 27:**0 X**	-1.149	-1.293 to -1.005	Yes
18079	17:**0 X** vs. 27:**1 X**	-1.239	-1.400 to -1.078	Yes
18080	17:**0 X** vs. 27:**10 X**	-1.051	-1.212 to -0.8904	Yes
18081	17:**0 X** vs. 27:**40 X**	-0.2060	-0.3500 to -0.06204	Yes
18082	17:**0 X** vs. 27:**50 X**	-0.06567	-0.2096 to 0.07829	No
18083	17:**0 X** vs. 28:**0 X**	-1.098	-1.259 to -0.9369	Yes
18084	17:**0 X** vs. 28:**1 X**	-1.139	-1.283 to -0.9947	Yes
18085	17:**0 X** vs. 28:**10 X**	-0.9403	-1.101 to -0.7794	Yes
18086	17:**0 X** vs. 28:**40 X**	-0.2183	-0.3623 to -0.07437	Yes
18087	17:**0 X** vs. 28:**50 X**	-0.1137	-0.2576 to 0.03029	No
18088	17:**0 X** vs. 29:**0 X**	-1.579	-1.740 to -1.418	Yes
18089	17:**0 X** vs. 29:**1 X**	-1.664	-1.825 to -1.503	Yes
18090	17:**0 X** vs. 29:**10 X**	-0.8788	-1.040 to -0.7179	Yes

	**2way ANOVA**<br>Multiple comparisons	**A**<br>Data Set-A<br>**Y**	**B**<br>Data Set-B<br>**Y**	**C**<br>Data Set-C<br>**Y**
**18091**	17:**0 X** vs. 29:**40 X**	-0.3263	-0.4703 to -0.1824	Yes
**18092**	17:**0 X** vs. 29:**50 X**	-0.1150	-0.2590 to 0.02896	No
**18093**	17:**0 X** vs. 30:**0 X**	-1.294	-1.455 to -1.133	Yes
**18094**	17:**0 X** vs. 30:**1 X**	-1.615	-1.776 to -1.454	Yes
**18095**	17:**0 X** vs. 30:**10 X**	-0.9533	-1.114 to -0.7924	Yes
**18096**	17:**0 X** vs. 30:**40 X**	-0.2533	-0.3973 to -0.1094	Yes
**18097**	17:**0 X** vs. 30:**50 X**	-0.1673	-0.3113 to -0.02337	Yes
**18098**	17:**0 X** vs. 31:**0 X**	-1.612	-1.773 to -1.451	Yes
**18099**	17:**0 X** vs. 31:**1 X**	-1.602	-1.763 to -1.441	Yes
**18100**	17:**0 X** vs. 31:**10 X**	-1.085	-1.246 to -0.9239	Yes
**18101**	17:**0 X** vs. 31:**40 X**	-0.3450	-0.4890 to -0.2010	Yes
**18102**	17:**0 X** vs. 31:**50 X**	-0.1463	-0.2903 to -0.002374	Yes
**18103**	17:**0 X** vs. 32:**0 X**	-2.203	-2.347 to -2.059	Yes
**18104**	17:**0 X** vs. 32:**1 X**	-1.659	-1.820 to -1.498	Yes
**18105**	17:**0 X** vs. 32:**10 X**	-1.515	-1.659 to -1.371	Yes
**18106**	17:**0 X** vs. 32:**40 X**	-0.3413	-0.4853 to -0.1974	Yes
**18107**	17:**0 X** vs. 32:**50 X**	-0.1460	-0.2900 to -0.002040	Yes
**18108**	17:**0 X** vs. 33:**0 X**	-2.207	-2.351 to -2.063	Yes
**18109**	17:**0 X** vs. 33:**1 X**	-2.022	-2.166 to -1.878	Yes
**18110**	17:**0 X** vs. 33:**10 X**	-1.682	-1.826 to -1.538	Yes
**18111**	17:**0 X** vs. 33:**40 X**	-0.3600	-0.5040 to -0.2160	Yes
**18112**	17:**0 X** vs. 33:**50 X**	-0.1610	-0.3050 to -0.01704	Yes
**18113**	17:**0 X** vs. 34:**0 X**	-2.132	-2.276 to -1.988	Yes
**18114**	17:**0 X** vs. 34:**1 X**	-2.139	-2.283 to -1.995	Yes
**18115**	17:**0 X** vs. 34:**10 X**	-1.772	-1.916 to -1.628	Yes
**18116**	17:**0 X** vs. 34:**40 X**	-0.3720	-0.5160 to -0.2280	Yes
**18117**	17:**0 X** vs. 34:**50 X**	-0.3100	-0.4540 to -0.1660	Yes
**18118**	17:**0 X** vs. 35:**0 X**	-2.480	-2.624 to -2.336	Yes
**18119**	17:**0 X** vs. 35:**1 X**	-2.246	-2.390 to -2.102	Yes
**18120**	17:**0 X** vs. 35:**10 X**	-1.734	-1.878 to -1.590	Yes
**18121**	17:**0 X** vs. 35:**40 X**	-0.3810	-0.5250 to -0.2370	Yes
**18122**	17:**0 X** vs. 35:**50 X**	-0.3227	-0.4666 to -0.1787	Yes
**18123**	17:**0 X** vs. 36:**0 X**	-2.687	-2.848 to -2.526	Yes
**18124**	17:**0 X** vs. 36:**1 X**	-2.383	-2.527 to -2.239	Yes
**18125**	17:**0 X** vs. 36:**10 X**	-1.740	-1.884 to -1.596	Yes
**18126**	17:**0 X** vs. 36:**40 X**	-0.4363	-0.5803 to -0.2924	Yes
**18127**	17:**0 X** vs. 36:**50 X**	-0.2860	-0.4300 to -0.1420	Yes
**18128**	17:**0 X** vs. 37:**0 X**	-3.071	-3.215 to -2.927	Yes
**18129**	17:**0 X** vs. 37:**1 X**	-2.453	-2.597 to -2.309	Yes
**18130**	17:**0 X** vs. 37:**10 X**	-1.741	-1.885 to -1.597	Yes
**18131**	17:**0 X** vs. 37:**40 X**	-0.4737	-0.6176 to -0.3297	Yes
**18132**	17:**0 X** vs. 37:**50 X**	-0.2947	-0.4386 to -0.1507	Yes
**18133**	17:**0 X** vs. 38:**0 X**	-3.016	-3.160 to -2.872	Yes
**18134**	17:**0 X** vs. 38:**1 X**	-2.571	-2.715 to -2.427	Yes
**18135**	17:**0 X** vs. 38:**10 X**	-2.017	-2.161 to -1.873	Yes

2way ANOVA Multiple comparisons	A Data Set-A Y	B Data Set-B Y	C Data Set-C Y
18136 17:0 X vs. 38:40 X	-0.4780	-0.6220 to -0.3340	Yes
18137 17:0 X vs. 38:50 X	-0.2987	-0.4426 to -0.1547	Yes
18138 17:0 X vs. 39:0 X	-3.246	-3.390 to -3.102	Yes
18139 17:0 X vs. 39:1 X	-3.138	-3.282 to -2.994	Yes
18140 17:0 X vs. 39:10 X	-2.162	-2.306 to -2.018	Yes
18141 17:0 X vs. 39:40 X	-0.4840	-0.6280 to -0.3400	Yes
18142 17:0 X vs. 39:50 X	-0.3297	-0.4736 to -0.1857	Yes
18143 17:0 X vs. 40:0 X	-3.287	-3.431 to -3.143	Yes
18144 17:0 X vs. 40:1 X	-3.213	-3.357 to -3.069	Yes
18145 17:0 X vs. 40:10 X	-2.418	-2.562 to -2.274	Yes
18146 17:0 X vs. 40:40 X	-0.4887	-0.6326 to -0.3447	Yes
18147 17:0 X vs. 40:50 X	-0.3360	-0.4800 to -0.1920	Yes
18148 17:0 X vs. 41:0 X	-3.328	-3.472 to -3.184	Yes
18149 17:0 X vs. 41:1 X	-3.327	-3.471 to -3.183	Yes
18150 17:0 X vs. 41:10 X	-2.472	-2.616 to -2.328	Yes
18151 17:0 X vs. 41:40 X	-0.4360	-0.5800 to -0.2920	Yes
18152 17:0 X vs. 41:50 X	-0.3487	-0.4926 to -0.2047	Yes
18153 17:0 X vs. 42:0 X	-3.470	-3.614 to -3.326	Yes
18154 17:0 X vs. 42:1 X	-3.490	-3.634 to -3.346	Yes
18155 17:0 X vs. 42:10 X	-2.472	-2.616 to -2.328	Yes
18156 17:0 X vs. 42:40 X	-0.4487	-0.5926 to -0.3047	Yes
18157 17:0 X vs. 42:50 X	-0.3520	-0.4960 to -0.2080	Yes
18158 17:0 X vs. 43:0 X	-3.560	-3.704 to -3.416	Yes
18159 17:0 X vs. 43:1 X	-3.474	-3.618 to -3.330	Yes
18160 17:0 X vs. 43:10 X	-3.190	-3.334 to -3.046	Yes
18161 17:0 X vs. 43:40 X	-0.4470	-0.5910 to -0.3030	Yes
18162 17:0 X vs. 43:50 X	-0.3523	-0.4963 to -0.2084	Yes
18163 17:0 X vs. 44:0 X	-3.584	-3.728 to -3.440	Yes
18164 17:0 X vs. 44:1 X	-3.474	-3.618 to -3.330	Yes
18165 17:0 X vs. 44:10 X	-3.253	-3.397 to -3.109	Yes
18166 17:0 X vs. 44:40 X	-0.4490	-0.5930 to -0.3050	Yes
18167 17:0 X vs. 44:50 X	-0.2847	-0.4286 to -0.1407	Yes
18168 17:0 X vs. 45:0 X	-3.449	-3.593 to -3.305	Yes
18169 17:0 X vs. 45:1 X	-3.251	-3.395 to -3.107	Yes
18170 17:0 X vs. 45:10 X	-3.133	-3.277 to -2.989	Yes
18171 17:0 X vs. 45:40 X	-0.4503	-0.5943 to -0.3064	Yes
18172 17:0 X vs. 45:50 X	-0.2817	-0.4256 to -0.1377	Yes
18173 17:0 X vs. 46:0 X	-3.253	-3.397 to -3.109	Yes
18174 17:0 X vs. 46:1 X	-3.220	-3.364 to -3.076	Yes
18175 17:0 X vs. 46:10 X	-3.128	-3.272 to -2.984	Yes
18176 17:0 X vs. 46:40 X	-0.4640	-0.6080 to -0.3200	Yes
18177 17:0 X vs. 46:50 X	-0.2747	-0.4186 to -0.1307	Yes
18178 17:0 X vs. 47:0 X	-3.229	-3.373 to -3.085	Yes
18179 17:0 X vs. 47:1 X	-3.133	-3.277 to -2.989	Yes
18180 17:0 X vs. 47:10 X	-3.114	-3.258 to -2.970	Yes

	2way ANOVA<br>Multiple comparisons	A<br>Data Set-A<br>Y	B<br>Data Set-B<br>Y	C<br>Data Set-C<br>Y
18181	17:0 X vs. 47:40 X	-0.4740	-0.6180 to -0.3300	Yes
18182	17:0 X vs. 47:50 X	-0.2327	-0.3766 to -0.08871	Yes
18183	17:0 X vs. 48:0 X	-3.216	-3.377 to -3.055	Yes
18184	17:0 X vs. 48:1 X	-3.120	-3.264 to -2.976	Yes
18185	17:0 X vs. 48:10 X	-3.114	-3.258 to -2.970	Yes
18186	17:0 X vs. 48:40 X	-0.4803	-0.6243 to -0.3364	Yes
18187	17:0 X vs. 48:50 X	-0.2400	-0.3840 to -0.09604	Yes
18188	17:0 X vs. 49:0 X	-3.216	-3.360 to -3.072	Yes
18189	17:0 X vs. 49:1 X	-3.120	-3.264 to -2.976	Yes
18190	17:0 X vs. 49:10 X	-3.114	-3.258 to -2.970	Yes
18191	17:0 X vs. 49:40 X	-0.4847	-0.6286 to -0.3407	Yes
18192	17:0 X vs. 49:50 X	-0.2393	-0.3833 to -0.09537	Yes
18193	17:0 X vs. 50:0 X	-3.216	-3.360 to -3.072	Yes
18194	17:0 X vs. 50:1 X	-3.120	-3.264 to -2.976	Yes
18195	17:0 X vs. 50:10 X	-3.114	-3.258 to -2.970	Yes
18196	17:0 X vs. 50:40 X	-0.4923	-0.6363 to -0.3484	Yes
18197	17:0 X vs. 50:50 X	-0.2390	-0.3830 to -0.09504	Yes
18198	17:1 X vs. 17:10 X	-0.05667	-0.2006 to 0.08729	No
18199	17:1 X vs. 17:40 X	0.0200	-0.1240 to 0.1640	No
18200	17:1 X vs. 17:50 X	-0.02533	-0.1693 to 0.1186	No
18201	17:1 X vs. 18:0 X	-0.1343	-0.2783 to 0.009626	No
18202	17:1 X vs. 18:1 X	-0.04267	-0.1866 to 0.1013	No
18203	17:1 X vs. 18:10 X	-0.1350	-0.2790 to 0.008960	No
18204	17:1 X vs. 18:40 X	0.01567	-0.1283 to 0.1596	No
18205	17:1 X vs. 18:50 X	-0.02933	-0.1733 to 0.1146	No
18206	17:1 X vs. 19:0 X	-0.1693	-0.3133 to -0.02537	Yes
18207	17:1 X vs. 19:1 X	-0.1573	-0.3013 to -0.01337	Yes
18208	17:1 X vs. 19:10 X	-0.1550	-0.2990 to -0.01104	Yes
18209	17:1 X vs. 19:40 X	-0.0130	-0.1570 to 0.1310	No
18210	17:1 X vs. 19:50 X	-0.0240	-0.1680 to 0.1200	No
18211	17:1 X vs. 20:0 X	-0.2490	-0.3930 to -0.1050	Yes
18212	17:1 X vs. 20:1 X	-0.06833	-0.2123 to 0.07563	No
18213	17:1 X vs. 20:10 X	-0.1573	-0.3013 to -0.01337	Yes
18214	17:1 X vs. 20:40 X	-0.02467	-0.1686 to 0.1193	No
18215	17:1 X vs. 20:50 X	-0.06867	-0.2126 to 0.07529	No
18216	17:1 X vs. 21:0 X	-0.2723	-0.4163 to -0.1284	Yes
18217	17:1 X vs. 21:1 X	-0.2253	-0.3693 to -0.08137	Yes
18218	17:1 X vs. 21:10 X	-0.2223	-0.3663 to -0.07837	Yes
18219	17:1 X vs. 21:40 X	-0.09767	-0.2416 to 0.04629	No
18220	17:1 X vs. 21:50 X	-0.1880	-0.3320 to -0.04404	Yes
18221	17:1 X vs. 22:0 X	-0.3140	-0.4580 to -0.1700	Yes
18222	17:1 X vs. 22:1 X	-0.3087	-0.4526 to -0.1647	Yes
18223	17:1 X vs. 22:10 X	-0.3040	-0.4480 to -0.1600	Yes
18224	17:1 X vs. 22:40 X	-0.2043	-0.3483 to -0.06037	Yes
18225	17:1 X vs. 22:50 X	-0.2670	-0.4110 to -0.1230	Yes

	2way ANOVA<br>Multiple comparisons	A<br>Data Set-A<br>Y	B<br>Data Set-B<br>Y	C<br>Data Set-C<br>Y
18226	17:**1 X** vs. 23:**0 X**	-0.3793	-0.5233 to -0.2354	Yes
18227	17:**1 X** vs. 23:**1 X**	-0.5690	-0.7300 to -0.4080	Yes
18228	17:**1 X** vs. 23:**10 X**	-0.3187	-0.4626 to -0.1747	Yes
18229	17:**1 X** vs. 23:**40 X**	-0.1990	-0.3600 to -0.03805	Yes
18230	17:**1 X** vs. 23:**50 X**	-0.2330	-0.3770 to -0.08904	Yes
18231	17:**1 X** vs. 24:**0 X**	-0.4967	-0.6406 to -0.3527	Yes
18232	17:**1 X** vs. 24:**1 X**	-0.6530	-0.8140 to -0.4920	Yes
18233	17:**1 X** vs. 24:**10 X**	-0.4805	-0.6415 to -0.3195	Yes
18234	17:**1 X** vs. 24:**40 X**	-0.2153	-0.3593 to -0.07137	Yes
18235	17:**1 X** vs. 24:**50 X**	-0.1370	-0.2810 to 0.006960	No
18236	17:**1 X** vs. 25:**0 X**	-0.8340	-0.9950 to -0.6730	Yes
18237	17:**1 X** vs. 25:**1 X**	-1.024	-1.184 to -0.8625	Yes
18238	17:**1 X** vs. 25:**10 X**	-0.3850	-0.5886 to -0.1814	Yes
18239	17:**1 X** vs. 25:**40 X**	-0.2173	-0.3613 to -0.07337	Yes
18240	17:**1 X** vs. 25:**50 X**	-0.1917	-0.3356 to -0.04771	Yes
18241	17:**1 X** vs. 26:**0 X**	-1.047	-1.207 to -0.8855	Yes
18242	17:**1 X** vs. 26:**1 X**	-1.160	-1.320 to -0.9985	Yes
18243	17:**1 X** vs. 26:**10 X**	-0.8460	-1.007 to -0.6850	Yes
18244	17:**1 X** vs. 26:**40 X**	-0.2917	-0.4356 to -0.1477	Yes
18245	17:**1 X** vs. 26:**50 X**	-0.1520	-0.2960 to -0.008040	Yes
18246	17:**1 X** vs. 27:**0 X**	-1.234	-1.378 to -1.090	Yes
18247	17:**1 X** vs. 27:**1 X**	-1.325	-1.486 to -1.164	Yes
18248	17:**1 X** vs. 27:**10 X**	-1.137	-1.298 to -0.9760	Yes
18249	17:**1 X** vs. 27:**40 X**	-0.2917	-0.4356 to -0.1477	Yes
18250	17:**1 X** vs. 27:**50 X**	-0.1513	-0.2953 to -0.007374	Yes
18251	17:**1 X** vs. 28:**0 X**	-1.184	-1.344 to -1.023	Yes
18252	17:**1 X** vs. 28:**1 X**	-1.224	-1.368 to -1.080	Yes
18253	17:**1 X** vs. 28:**10 X**	-1.026	-1.187 to -0.8650	Yes
18254	17:**1 X** vs. 28:**40 X**	-0.3040	-0.4480 to -0.1600	Yes
18255	17:**1 X** vs. 28:**50 X**	-0.1993	-0.3433 to -0.05537	Yes
18256	17:**1 X** vs. 29:**0 X**	-1.665	-1.826 to -1.504	Yes
18257	17:**1 X** vs. 29:**1 X**	-1.750	-1.911 to -1.589	Yes
18258	17:**1 X** vs. 29:**10 X**	-0.9645	-1.125 to -0.8035	Yes
18259	17:**1 X** vs. 29:**40 X**	-0.4120	-0.5560 to -0.2680	Yes
18260	17:**1 X** vs. 29:**50 X**	-0.2007	-0.3446 to -0.05671	Yes
18261	17:**1 X** vs. 30:**0 X**	-1.380	-1.540 to -1.219	Yes
18262	17:**1 X** vs. 30:**1 X**	-1.701	-1.862 to -1.540	Yes
18263	17:**1 X** vs. 30:**10 X**	-1.039	-1.200 to -0.8780	Yes
18264	17:**1 X** vs. 30:**40 X**	-0.3390	-0.4830 to -0.1950	Yes
18265	17:**1 X** vs. 30:**50 X**	-0.2530	-0.3970 to -0.1090	Yes
18266	17:**1 X** vs. 31:**0 X**	-1.698	-1.858 to -1.537	Yes
18267	17:**1 X** vs. 31:**1 X**	-1.688	-1.849 to -1.527	Yes
18268	17:**1 X** vs. 31:**10 X**	-1.171	-1.331 to -1.010	Yes
18269	17:**1 X** vs. 31:**40 X**	-0.4307	-0.5746 to -0.2867	Yes
18270	17:**1 X** vs. 31:**50 X**	-0.2320	-0.3760 to -0.08804	Yes

	2way ANOVA Multiple comparisons	A Data Set-A Y	B Data Set-B Y	C Data Set-C Y
18271	17:**1 X** vs. 32:**0 X**	-2.289	-2.433 to -2.145	Yes
18272	17:**1 X** vs. 32:**1 X**	-1.745	-1.906 to -1.584	Yes
18273	17:**1 X** vs. 32:**10 X**	-1.601	-1.745 to -1.457	Yes
18274	17:**1 X** vs. 32:**40 X**	-0.4270	-0.5710 to -0.2830	Yes
18275	17:**1 X** vs. 32:**50 X**	-0.2317	-0.3756 to -0.08771	Yes
18276	17:**1 X** vs. 33:**0 X**	-2.293	-2.437 to -2.149	Yes
18277	17:**1 X** vs. 33:**1 X**	-2.107	-2.251 to -1.963	Yes
18278	17:**1 X** vs. 33:**10 X**	-1.768	-1.912 to -1.624	Yes
18279	17:**1 X** vs. 33:**40 X**	-0.4457	-0.5896 to -0.3017	Yes
18280	17:**1 X** vs. 33:**50 X**	-0.2467	-0.3906 to -0.1027	Yes
18281	17:**1 X** vs. 34:**0 X**	-2.217	-2.361 to -2.073	Yes
18282	17:**1 X** vs. 34:**1 X**	-2.225	-2.369 to -2.081	Yes
18283	17:**1 X** vs. 34:**10 X**	-1.858	-2.002 to -1.714	Yes
18284	17:**1 X** vs. 34:**40 X**	-0.4577	-0.6016 to -0.3137	Yes
18285	17:**1 X** vs. 34:**50 X**	-0.3957	-0.5396 to -0.2517	Yes
18286	17:**1 X** vs. 35:**0 X**	-2.566	-2.710 to -2.422	Yes
18287	17:**1 X** vs. 35:**1 X**	-2.332	-2.476 to -2.188	Yes
18288	17:**1 X** vs. 35:**10 X**	-1.820	-1.964 to -1.676	Yes
18289	17:**1 X** vs. 35:**40 X**	-0.4667	-0.6106 to -0.3227	Yes
18290	17:**1 X** vs. 35:**50 X**	-0.4083	-0.5523 to -0.2644	Yes
18291	17:**1 X** vs. 36:**0 X**	-2.773	-2.933 to -2.612	Yes
18292	17:**1 X** vs. 36:**1 X**	-2.468	-2.612 to -2.324	Yes
18293	17:**1 X** vs. 36:**10 X**	-1.825	-1.969 to -1.681	Yes
18294	17:**1 X** vs. 36:**40 X**	-0.5220	-0.6660 to -0.3780	Yes
18295	17:**1 X** vs. 36:**50 X**	-0.3717	-0.5156 to -0.2277	Yes
18296	17:**1 X** vs. 37:**0 X**	-3.156	-3.300 to -3.012	Yes
18297	17:**1 X** vs. 37:**1 X**	-2.538	-2.682 to -2.394	Yes
18298	17:**1 X** vs. 37:**10 X**	-1.827	-1.971 to -1.683	Yes
18299	17:**1 X** vs. 37:**40 X**	-0.5593	-0.7033 to -0.4154	Yes
18300	17:**1 X** vs. 37:**50 X**	-0.3803	-0.5243 to -0.2364	Yes
18301	17:**1 X** vs. 38:**0 X**	-3.101	-3.245 to -2.957	Yes
18302	17:**1 X** vs. 38:**1 X**	-2.657	-2.801 to -2.513	Yes
18303	17:**1 X** vs. 38:**10 X**	-2.102	-2.246 to -1.958	Yes
18304	17:**1 X** vs. 38:**40 X**	-0.5637	-0.7076 to -0.4197	Yes
18305	17:**1 X** vs. 38:**50 X**	-0.3843	-0.5283 to -0.2404	Yes
18306	17:**1 X** vs. 39:**0 X**	-3.332	-3.476 to -3.188	Yes
18307	17:**1 X** vs. 39:**1 X**	-3.224	-3.368 to -3.080	Yes
18308	17:**1 X** vs. 39:**10 X**	-2.247	-2.391 to -2.103	Yes
18309	17:**1 X** vs. 39:**40 X**	-0.5697	-0.7136 to -0.4257	Yes
18310	17:**1 X** vs. 39:**50 X**	-0.4153	-0.5593 to -0.2714	Yes
18311	17:**1 X** vs. 40:**0 X**	-3.373	-3.517 to -3.229	Yes
18312	17:**1 X** vs. 40:**1 X**	-3.299	-3.443 to -3.155	Yes
18313	17:**1 X** vs. 40:**10 X**	-2.504	-2.648 to -2.360	Yes
18314	17:**1 X** vs. 40:**40 X**	-0.5743	-0.7183 to -0.4304	Yes
18315	17:**1 X** vs. 40:**50 X**	-0.4217	-0.5656 to -0.2777	Yes

	2way ANOVA<br>Multiple comparisons	A<br>Data Set-A<br>Y	B<br>Data Set-B<br>Y	C<br>Data Set-C<br>Y
18316	17:**1 X** vs. 41:**0 X**	-3.414	-3.558 to -3.270	Yes
18317	17:**1 X** vs. 41:**1 X**	-3.413	-3.557 to -3.269	Yes
18318	17:**1 X** vs. 41:**10 X**	-2.557	-2.701 to -2.413	Yes
18319	17:**1 X** vs. 41:**40 X**	-0.5217	-0.6656 to -0.3777	Yes
18320	17:**1 X** vs. 41:**50 X**	-0.4343	-0.5783 to -0.2904	Yes
18321	17:**1 X** vs. 42:**0 X**	-3.556	-3.700 to -3.412	Yes
18322	17:**1 X** vs. 42:**1 X**	-3.575	-3.719 to -3.431	Yes
18323	17:**1 X** vs. 42:**10 X**	-2.558	-2.702 to -2.414	Yes
18324	17:**1 X** vs. 42:**40 X**	-0.5343	-0.6783 to -0.3904	Yes
18325	17:**1 X** vs. 42:**50 X**	-0.4377	-0.5816 to -0.2937	Yes
18326	17:**1 X** vs. 43:**0 X**	-3.646	-3.790 to -3.502	Yes
18327	17:**1 X** vs. 43:**1 X**	-3.560	-3.704 to -3.416	Yes
18328	17:**1 X** vs. 43:**10 X**	-3.276	-3.420 to -3.132	Yes
18329	17:**1 X** vs. 43:**40 X**	-0.5327	-0.6766 to -0.3887	Yes
18330	17:**1 X** vs. 43:**50 X**	-0.4380	-0.5820 to -0.2940	Yes
18331	17:**1 X** vs. 44:**0 X**	-3.670	-3.814 to -3.526	Yes
18332	17:**1 X** vs. 44:**1 X**	-3.559	-3.703 to -3.415	Yes
18333	17:**1 X** vs. 44:**10 X**	-3.339	-3.483 to -3.195	Yes
18334	17:**1 X** vs. 44:**40 X**	-0.5347	-0.6786 to -0.3907	Yes
18335	17:**1 X** vs. 44:**50 X**	-0.3703	-0.5143 to -0.2264	Yes
18336	17:**1 X** vs. 45:**0 X**	-3.534	-3.678 to -3.390	Yes
18337	17:**1 X** vs. 45:**1 X**	-3.336	-3.480 to -3.192	Yes
18338	17:**1 X** vs. 45:**10 X**	-3.219	-3.363 to -3.075	Yes
18339	17:**1 X** vs. 45:**40 X**	-0.5360	-0.6800 to -0.3920	Yes
18340	17:**1 X** vs. 45:**50 X**	-0.3673	-0.5113 to -0.2234	Yes
18341	17:**1 X** vs. 46:**0 X**	-3.339	-3.483 to -3.195	Yes
18342	17:**1 X** vs. 46:**1 X**	-3.306	-3.450 to -3.162	Yes
18343	17:**1 X** vs. 46:**10 X**	-3.214	-3.358 to -3.070	Yes
18344	17:**1 X** vs. 46:**40 X**	-0.5497	-0.6936 to -0.4057	Yes
18345	17:**1 X** vs. 46:**50 X**	-0.3603	-0.5043 to -0.2164	Yes
18346	17:**1 X** vs. 47:**0 X**	-3.314	-3.458 to -3.170	Yes
18347	17:**1 X** vs. 47:**1 X**	-3.218	-3.362 to -3.074	Yes
18348	17:**1 X** vs. 47:**10 X**	-3.200	-3.344 to -3.056	Yes
18349	17:**1 X** vs. 47:**40 X**	-0.5597	-0.7036 to -0.4157	Yes
18350	17:**1 X** vs. 47:**50 X**	-0.3183	-0.4623 to -0.1744	Yes
18351	17:**1 X** vs. 48:**0 X**	-3.302	-3.462 to -3.141	Yes
18352	17:**1 X** vs. 48:**1 X**	-3.205	-3.349 to -3.061	Yes
18353	17:**1 X** vs. 48:**10 X**	-3.200	-3.344 to -3.056	Yes
18354	17:**1 X** vs. 48:**40 X**	-0.5660	-0.7100 to -0.4220	Yes
18355	17:**1 X** vs. 48:**50 X**	-0.3257	-0.4696 to -0.1817	Yes
18356	17:**1 X** vs. 49:**0 X**	-3.302	-3.446 to -3.158	Yes
18357	17:**1 X** vs. 49:**1 X**	-3.205	-3.349 to -3.061	Yes
18358	17:**1 X** vs. 49:**10 X**	-3.200	-3.344 to -3.056	Yes
18359	17:**1 X** vs. 49:**40 X**	-0.5703	-0.7143 to -0.4264	Yes
18360	17:**1 X** vs. 49:**50 X**	-0.3250	-0.4690 to -0.1810	Yes

	2way ANOVA Multiple comparisons	A Data Set-A Y	B Data Set-B Y	C Data Set-C Y
18361	17:**1 X** vs. 50:**0 X**	-3.302	-3.446 to -3.158	Yes
18362	17:**1 X** vs. 50:**1 X**	-3.205	-3.349 to -3.061	Yes
18363	17:**1 X** vs. 50:**10 X**	-3.200	-3.344 to -3.056	Yes
18364	17:**1 X** vs. 50:**40 X**	-0.5780	-0.7220 to -0.4340	Yes
18365	17:**1 X** vs. 50:**50 X**	-0.3247	-0.4686 to -0.1807	Yes
18366	17:**10 X** vs. 17:**40 X**	0.07667	-0.06729 to 0.2206	No
18367	17:**10 X** vs. 17:**50 X**	0.03133	-0.1126 to 0.1753	No
18368	17:**10 X** vs. 18:**0 X**	-0.07767	-0.2216 to 0.06629	No
18369	17:**10 X** vs. 18:**1 X**	0.0140	-0.1300 to 0.1580	No
18370	17:**10 X** vs. 18:**10 X**	-0.07833	-0.2223 to 0.06563	No
18371	17:**10 X** vs. 18:**40 X**	0.07233	-0.07163 to 0.2163	No
18372	17:**10 X** vs. 18:**50 X**	0.02733	-0.1166 to 0.1713	No
18373	17:**10 X** vs. 19:**0 X**	-0.1127	-0.2566 to 0.03129	No
18374	17:**10 X** vs. 19:**1 X**	-0.1007	-0.2446 to 0.04329	No
18375	17:**10 X** vs. 19:**10 X**	-0.09833	-0.2423 to 0.04563	No
18376	17:**10 X** vs. 19:**40 X**	0.04367	-0.1003 to 0.1876	No
18377	17:**10 X** vs. 19:**50 X**	0.03267	-0.1113 to 0.1766	No
18378	17:**10 X** vs. 20:**0 X**	-0.1923	-0.3363 to -0.04837	Yes
18379	17:**10 X** vs. 20:**1 X**	-0.01167	-0.1556 to 0.1323	No
18380	17:**10 X** vs. 20:**10 X**	-0.1007	-0.2446 to 0.04329	No
18381	17:**10 X** vs. 20:**40 X**	0.0320	-0.1120 to 0.1760	No
18382	17:**10 X** vs. 20:**50 X**	-0.0120	-0.1560 to 0.1320	No
18383	17:**10 X** vs. 21:**0 X**	-0.2157	-0.3596 to -0.07171	Yes
18384	17:**10 X** vs. 21:**1 X**	-0.1687	-0.3126 to -0.02471	Yes
18385	17:**10 X** vs. 21:**10 X**	-0.1657	-0.3096 to -0.02171	Yes
18386	17:**10 X** vs. 21:**40 X**	-0.0410	-0.1850 to 0.1030	No
18387	17:**10 X** vs. 21:**50 X**	-0.1313	-0.2753 to 0.01263	No
18388	17:**10 X** vs. 22:**0 X**	-0.2573	-0.4013 to -0.1134	Yes
18389	17:**10 X** vs. 22:**1 X**	-0.2520	-0.3960 to -0.1080	Yes
18390	17:**10 X** vs. 22:**10 X**	-0.2473	-0.3913 to -0.1034	Yes
18391	17:**10 X** vs. 22:**40 X**	-0.1477	-0.2916 to -0.003707	Yes
18392	17:**10 X** vs. 22:**50 X**	-0.2103	-0.3543 to -0.06637	Yes
18393	17:**10 X** vs. 23:**0 X**	-0.3227	-0.4666 to -0.1787	Yes
18394	17:**10 X** vs. 23:**1 X**	-0.5123	-0.6733 to -0.3514	Yes
18395	17:**10 X** vs. 23:**10 X**	-0.2620	-0.4060 to -0.1180	Yes
18396	17:**10 X** vs. 23:**40 X**	-0.1423	-0.3033 to 0.01862	No
18397	17:**10 X** vs. 23:**50 X**	-0.1763	-0.3203 to -0.03237	Yes
18398	17:**10 X** vs. 24:**0 X**	-0.4400	-0.5840 to -0.2960	Yes
18399	17:**10 X** vs. 24:**1 X**	-0.5963	-0.7573 to -0.4354	Yes
18400	17:**10 X** vs. 24:**10 X**	-0.4238	-0.5848 to -0.2629	Yes
18401	17:**10 X** vs. 24:**40 X**	-0.1587	-0.3026 to -0.01471	Yes
18402	17:**10 X** vs. 24:**50 X**	-0.08033	-0.2243 to 0.06363	No
18403	17:**10 X** vs. 25:**0 X**	-0.7773	-0.9383 to -0.6164	Yes
18404	17:**10 X** vs. 25:**1 X**	-0.9668	-1.128 to -0.8059	Yes
18405	17:**10 X** vs. 25:**10 X**	-0.3283	-0.5319 to -0.1247	Yes

	2way ANOVA<br>Multiple comparisons	A<br>Data Set-A<br>Y	B<br>Data Set-B<br>Y	C<br>Data Set-C<br>Y
18406	17:10 X vs. 25:40 X	-0.1607	-0.3046 to -0.01671	Yes
18407	17:10 X vs. 25:50 X	-0.1350	-0.2790 to 0.008960	No
18408	17:10 X vs. 26:0 X	-0.9898	-1.151 to -0.8289	Yes
18409	17:10 X vs. 26:1 X	-1.103	-1.264 to -0.9419	Yes
18410	17:10 X vs. 26:10 X	-0.7893	-0.9503 to -0.6284	Yes
18411	17:10 X vs. 26:40 X	-0.2350	-0.3790 to -0.09104	Yes
18412	17:10 X vs. 26:50 X	-0.09533	-0.2393 to 0.04863	No
18413	17:10 X vs. 27:0 X	-1.178	-1.322 to -1.034	Yes
18414	17:10 X vs. 27:1 X	-1.268	-1.429 to -1.107	Yes
18415	17:10 X vs. 27:10 X	-1.080	-1.241 to -0.9194	Yes
18416	17:10 X vs. 27:40 X	-0.2350	-0.3790 to -0.09104	Yes
18417	17:10 X vs. 27:50 X	-0.09467	-0.2386 to 0.04929	No
18418	17:10 X vs. 28:0 X	-1.127	-1.288 to -0.9659	Yes
18419	17:10 X vs. 28:1 X	-1.168	-1.312 to -1.024	Yes
18420	17:10 X vs. 28:10 X	-0.9693	-1.130 to -0.8084	Yes
18421	17:10 X vs. 28:40 X	-0.2473	-0.3913 to -0.1034	Yes
18422	17:10 X vs. 28:50 X	-0.1427	-0.2866 to 0.001293	No
18423	17:10 X vs. 29:0 X	-1.608	-1.769 to -1.447	Yes
18424	17:10 X vs. 29:1 X	-1.693	-1.854 to -1.532	Yes
18425	17:10 X vs. 29:10 X	-0.9078	-1.069 to -0.7469	Yes
18426	17:10 X vs. 29:40 X	-0.3553	-0.4993 to -0.2114	Yes
18427	17:10 X vs. 29:50 X	-0.1440	-0.2880 to -4.018e-005	Yes
18428	17:10 X vs. 30:0 X	-1.323	-1.484 to -1.162	Yes
18429	17:10 X vs. 30:1 X	-1.644	-1.805 to -1.483	Yes
18430	17:10 X vs. 30:10 X	-0.9823	-1.143 to -0.8214	Yes
18431	17:10 X vs. 30:40 X	-0.2823	-0.4263 to -0.1384	Yes
18432	17:10 X vs. 30:50 X	-0.1963	-0.3403 to -0.05237	Yes
18433	17:10 X vs. 31:0 X	-1.641	-1.802 to -1.480	Yes
18434	17:10 X vs. 31:1 X	-1.631	-1.792 to -1.470	Yes
18435	17:10 X vs. 31:10 X	-1.114	-1.275 to -0.9529	Yes
18436	17:10 X vs. 31:40 X	-0.3740	-0.5180 to -0.2300	Yes
18437	17:10 X vs. 31:50 X	-0.1753	-0.3193 to -0.03137	Yes
18438	17:10 X vs. 32:0 X	-2.232	-2.376 to -2.088	Yes
18439	17:10 X vs. 32:1 X	-1.688	-1.849 to -1.527	Yes
18440	17:10 X vs. 32:10 X	-1.544	-1.688 to -1.400	Yes
18441	17:10 X vs. 32:40 X	-0.3703	-0.5143 to -0.2264	Yes
18442	17:10 X vs. 32:50 X	-0.1750	-0.3190 to -0.03104	Yes
18443	17:10 X vs. 33:0 X	-2.236	-2.380 to -2.092	Yes
18444	17:10 X vs. 33:1 X	-2.051	-2.195 to -1.907	Yes
18445	17:10 X vs. 33:10 X	-1.711	-1.855 to -1.567	Yes
18446	17:10 X vs. 33:40 X	-0.3890	-0.5330 to -0.2450	Yes
18447	17:10 X vs. 33:50 X	-0.1900	-0.3340 to -0.04604	Yes
18448	17:10 X vs. 34:0 X	-2.161	-2.305 to -2.017	Yes
18449	17:10 X vs. 34:1 X	-2.168	-2.312 to -2.024	Yes
18450	17:10 X vs. 34:10 X	-1.801	-1.945 to -1.657	Yes

	2way ANOVA<br>Multiple comparisons	A<br>Data Set-A<br>Y	B<br>Data Set-B<br>Y	C<br>Data Set-C<br>Y
18451	17:10 X vs. 34:40 X	-0.4010	-0.5450 to -0.2570	Yes
18452	17:10 X vs. 34:50 X	-0.3390	-0.4830 to -0.1950	Yes
18453	17:10 X vs. 35:0 X	-2.509	-2.653 to -2.365	Yes
18454	17:10 X vs. 35:1 X	-2.275	-2.419 to -2.131	Yes
18455	17:10 X vs. 35:10 X	-1.763	-1.907 to -1.619	Yes
18456	17:10 X vs. 35:40 X	-0.4100	-0.5540 to -0.2660	Yes
18457	17:10 X vs. 35:50 X	-0.3517	-0.4956 to -0.2077	Yes
18458	17:10 X vs. 36:0 X	-2.716	-2.877 to -2.555	Yes
18459	17:10 X vs. 36:1 X	-2.412	-2.556 to -2.268	Yes
18460	17:10 X vs. 36:10 X	-1.769	-1.913 to -1.625	Yes
18461	17:10 X vs. 36:40 X	-0.4653	-0.6093 to -0.3214	Yes
18462	17:10 X vs. 36:50 X	-0.3150	-0.4590 to -0.1710	Yes
18463	17:10 X vs. 37:0 X	-3.100	-3.244 to -2.956	Yes
18464	17:10 X vs. 37:1 X	-2.482	-2.626 to -2.338	Yes
18465	17:10 X vs. 37:10 X	-1.770	-1.914 to -1.626	Yes
18466	17:10 X vs. 37:40 X	-0.5027	-0.6466 to -0.3587	Yes
18467	17:10 X vs. 37:50 X	-0.3237	-0.4676 to -0.1797	Yes
18468	17:10 X vs. 38:0 X	-3.045	-3.189 to -2.901	Yes
18469	17:10 X vs. 38:1 X	-2.600	-2.744 to -2.456	Yes
18470	17:10 X vs. 38:10 X	-2.046	-2.190 to -1.902	Yes
18471	17:10 X vs. 38:40 X	-0.5070	-0.6510 to -0.3630	Yes
18472	17:10 X vs. 38:50 X	-0.3277	-0.4716 to -0.1837	Yes
18473	17:10 X vs. 39:0 X	-3.275	-3.419 to -3.131	Yes
18474	17:10 X vs. 39:1 X	-3.167	-3.311 to -3.023	Yes
18475	17:10 X vs. 39:10 X	-2.191	-2.335 to -2.047	Yes
18476	17:10 X vs. 39:40 X	-0.5130	-0.6570 to -0.3690	Yes
18477	17:10 X vs. 39:50 X	-0.3587	-0.5026 to -0.2147	Yes
18478	17:10 X vs. 40:0 X	-3.316	-3.460 to -3.172	Yes
18479	17:10 X vs. 40:1 X	-3.242	-3.386 to -3.098	Yes
18480	17:10 X vs. 40:10 X	-2.447	-2.591 to -2.303	Yes
18481	17:10 X vs. 40:40 X	-0.5177	-0.6616 to -0.3737	Yes
18482	17:10 X vs. 40:50 X	-0.3650	-0.5090 to -0.2210	Yes
18483	17:10 X vs. 41:0 X	-3.357	-3.501 to -3.213	Yes
18484	17:10 X vs. 41:1 X	-3.356	-3.500 to -3.212	Yes
18485	17:10 X vs. 41:10 X	-2.501	-2.645 to -2.357	Yes
18486	17:10 X vs. 41:40 X	-0.4650	-0.6090 to -0.3210	Yes
18487	17:10 X vs. 41:50 X	-0.3777	-0.5216 to -0.2337	Yes
18488	17:10 X vs. 42:0 X	-3.499	-3.643 to -3.355	Yes
18489	17:10 X vs. 42:1 X	-3.519	-3.663 to -3.375	Yes
18490	17:10 X vs. 42:10 X	-2.501	-2.645 to -2.357	Yes
18491	17:10 X vs. 42:40 X	-0.4777	-0.6216 to -0.3337	Yes
18492	17:10 X vs. 42:50 X	-0.3810	-0.5250 to -0.2370	Yes
18493	17:10 X vs. 43:0 X	-3.589	-3.733 to -3.445	Yes
18494	17:10 X vs. 43:1 X	-3.503	-3.647 to -3.359	Yes
18495	17:10 X vs. 43:10 X	-3.219	-3.363 to -3.075	Yes

	2way ANOVA<br>Multiple comparisons	A<br>Data Set-A<br>Y	B<br>Data Set-B<br>Y	C<br>Data Set-C<br>Y
18496	17:**10 X** vs. 43:**40 X**	-0.4760	-0.6200 to -0.3320	Yes
18497	17:**10 X** vs. 43:**50 X**	-0.3813	-0.5253 to -0.2374	Yes
18498	17:**10 X** vs. 44:**0 X**	-3.613	-3.757 to -3.469	Yes
18499	17:**10 X** vs. 44:**1 X**	-3.503	-3.647 to -3.359	Yes
18500	17:**10 X** vs. 44:**10 X**	-3.282	-3.426 to -3.138	Yes
18501	17:**10 X** vs. 44:**40 X**	-0.4780	-0.6220 to -0.3340	Yes
18502	17:**10 X** vs. 44:**50 X**	-0.3137	-0.4576 to -0.1697	Yes
18503	17:**10 X** vs. 45:**0 X**	-3.478	-3.622 to -3.334	Yes
18504	17:**10 X** vs. 45:**1 X**	-3.280	-3.424 to -3.136	Yes
18505	17:**10 X** vs. 45:**10 X**	-3.162	-3.306 to -3.018	Yes
18506	17:**10 X** vs. 45:**40 X**	-0.4793	-0.6233 to -0.3354	Yes
18507	17:**10 X** vs. 45:**50 X**	-0.3107	-0.4546 to -0.1667	Yes
18508	17:**10 X** vs. 46:**0 X**	-3.282	-3.426 to -3.138	Yes
18509	17:**10 X** vs. 46:**1 X**	-3.249	-3.393 to -3.105	Yes
18510	17:**10 X** vs. 46:**10 X**	-3.157	-3.301 to -3.013	Yes
18511	17:**10 X** vs. 46:**40 X**	-0.4930	-0.6370 to -0.3490	Yes
18512	17:**10 X** vs. 46:**50 X**	-0.3037	-0.4476 to -0.1597	Yes
18513	17:**10 X** vs. 47:**0 X**	-3.258	-3.402 to -3.114	Yes
18514	17:**10 X** vs. 47:**1 X**	-3.162	-3.306 to -3.018	Yes
18515	17:**10 X** vs. 47:**10 X**	-3.143	-3.287 to -2.999	Yes
18516	17:**10 X** vs. 47:**40 X**	-0.5030	-0.6470 to -0.3590	Yes
18517	17:**10 X** vs. 47:**50 X**	-0.2617	-0.4056 to -0.1177	Yes
18518	17:**10 X** vs. 48:**0 X**	-3.245	-3.406 to -3.084	Yes
18519	17:**10 X** vs. 48:**1 X**	-3.149	-3.293 to -3.005	Yes
18520	17:**10 X** vs. 48:**10 X**	-3.143	-3.287 to -2.999	Yes
18521	17:**10 X** vs. 48:**40 X**	-0.5093	-0.6533 to -0.3654	Yes
18522	17:**10 X** vs. 48:**50 X**	-0.2690	-0.4130 to -0.1250	Yes
18523	17:**10 X** vs. 49:**0 X**	-3.245	-3.389 to -3.101	Yes
18524	17:**10 X** vs. 49:**1 X**	-3.149	-3.293 to -3.005	Yes
18525	17:**10 X** vs. 49:**10 X**	-3.143	-3.287 to -2.999	Yes
18526	17:**10 X** vs. 49:**40 X**	-0.5137	-0.6576 to -0.3697	Yes
18527	17:**10 X** vs. 49:**50 X**	-0.2683	-0.4123 to -0.1244	Yes
18528	17:**10 X** vs. 50:**0 X**	-3.245	-3.389 to -3.101	Yes
18529	17:**10 X** vs. 50:**1 X**	-3.149	-3.293 to -3.005	Yes
18530	17:**10 X** vs. 50:**10 X**	-3.143	-3.287 to -2.999	Yes
18531	17:**10 X** vs. 50:**40 X**	-0.5213	-0.6653 to -0.3774	Yes
18532	17:**10 X** vs. 50:**50 X**	-0.2680	-0.4120 to -0.1240	Yes
18533	17:**40 X** vs. 17:**50 X**	-0.04533	-0.1893 to 0.09863	No
18534	17:**40 X** vs. 18:**0 X**	-0.1543	-0.2983 to -0.01037	Yes
18535	17:**40 X** vs. 18:**1 X**	-0.06267	-0.2066 to 0.08129	No
18536	17:**40 X** vs. 18:**10 X**	-0.1550	-0.2990 to -0.01104	Yes
18537	17:**40 X** vs. 18:**40 X**	-0.004333	-0.1483 to 0.1396	No
18538	17:**40 X** vs. 18:**50 X**	-0.04933	-0.1933 to 0.09463	No
18539	17:**40 X** vs. 19:**0 X**	-0.1893	-0.3333 to -0.04537	Yes
18540	17:**40 X** vs. 19:**1 X**	-0.1773	-0.3213 to -0.03337	Yes

	2way ANOVA<br>Multiple comparisons	A<br>Data Set-A<br>Y	B<br>Data Set-B<br>Y	C<br>Data Set-C<br>Y
18541	17:40 X vs. 19:10 X	-0.1750	-0.3190 to -0.03104	Yes
18542	17:40 X vs. 19:40 X	-0.0330	-0.1770 to 0.1110	No
18543	17:40 X vs. 19:50 X	-0.0440	-0.1880 to 0.09996	No
18544	17:40 X vs. 20:0 X	-0.2690	-0.4130 to -0.1250	Yes
18545	17:40 X vs. 20:1 X	-0.08833	-0.2323 to 0.05563	No
18546	17:40 X vs. 20:10 X	-0.1773	-0.3213 to -0.03337	Yes
18547	17:40 X vs. 20:40 X	-0.04467	-0.1886 to 0.09929	No
18548	17:40 X vs. 20:50 X	-0.08867	-0.2326 to 0.05529	No
18549	17:40 X vs. 21:0 X	-0.2923	-0.4363 to -0.1484	Yes
18550	17:40 X vs. 21:1 X	-0.2453	-0.3893 to -0.1014	Yes
18551	17:40 X vs. 21:10 X	-0.2423	-0.3863 to -0.09837	Yes
18552	17:40 X vs. 21:40 X	-0.1177	-0.2616 to 0.02629	No
18553	17:40 X vs. 21:50 X	-0.2080	-0.3520 to -0.06404	Yes
18554	17:40 X vs. 22:0 X	-0.3340	-0.4780 to -0.1900	Yes
18555	17:40 X vs. 22:1 X	-0.3287	-0.4726 to -0.1847	Yes
18556	17:40 X vs. 22:10 X	-0.3240	-0.4680 to -0.1800	Yes
18557	17:40 X vs. 22:40 X	-0.2243	-0.3683 to -0.08037	Yes
18558	17:40 X vs. 22:50 X	-0.2870	-0.4310 to -0.1430	Yes
18559	17:40 X vs. 23:0 X	-0.3993	-0.5433 to -0.2554	Yes
18560	17:40 X vs. 23:1 X	-0.5890	-0.7500 to -0.4280	Yes
18561	17:40 X vs. 23:10 X	-0.3387	-0.4826 to -0.1947	Yes
18562	17:40 X vs. 23:40 X	-0.2190	-0.3800 to -0.05805	Yes
18563	17:40 X vs. 23:50 X	-0.2530	-0.3970 to -0.1090	Yes
18564	17:40 X vs. 24:0 X	-0.5167	-0.6606 to -0.3727	Yes
18565	17:40 X vs. 24:1 X	-0.6730	-0.8340 to -0.5120	Yes
18566	17:40 X vs. 24:10 X	-0.5005	-0.6615 to -0.3395	Yes
18567	17:40 X vs. 24:40 X	-0.2353	-0.3793 to -0.09137	Yes
18568	17:40 X vs. 24:50 X	-0.1570	-0.3010 to -0.01304	Yes
18569	17:40 X vs. 25:0 X	-0.8540	-1.015 to -0.6930	Yes
18570	17:40 X vs. 25:1 X	-1.044	-1.204 to -0.8825	Yes
18571	17:40 X vs. 25:10 X	-0.4050	-0.6086 to -0.2014	Yes
18572	17:40 X vs. 25:40 X	-0.2373	-0.3813 to -0.09337	Yes
18573	17:40 X vs. 25:50 X	-0.2117	-0.3556 to -0.06771	Yes
18574	17:40 X vs. 26:0 X	-1.067	-1.227 to -0.9055	Yes
18575	17:40 X vs. 26:1 X	-1.180	-1.340 to -1.019	Yes
18576	17:40 X vs. 26:10 X	-0.8660	-1.027 to -0.7050	Yes
18577	17:40 X vs. 26:40 X	-0.3117	-0.4556 to -0.1677	Yes
18578	17:40 X vs. 26:50 X	-0.1720	-0.3160 to -0.02804	Yes
18579	17:40 X vs. 27:0 X	-1.254	-1.398 to -1.110	Yes
18580	17:40 X vs. 27:1 X	-1.345	-1.506 to -1.184	Yes
18581	17:40 X vs. 27:10 X	-1.157	-1.318 to -0.9960	Yes
18582	17:40 X vs. 27:40 X	-0.3117	-0.4556 to -0.1677	Yes
18583	17:40 X vs. 27:50 X	-0.1713	-0.3153 to -0.02737	Yes
18584	17:40 X vs. 28:0 X	-1.204	-1.364 to -1.043	Yes
18585	17:40 X vs. 28:1 X	-1.244	-1.388 to -1.100	Yes

	2way ANOVA<br>Multiple comparisons	A<br>Data Set-A<br>Y	B<br>Data Set-B<br>Y	C<br>Data Set-C<br>Y
18586	17:40 X vs. 28:10 X	-1.046	-1.207 to -0.8850	Yes
18587	17:40 X vs. 28:40 X	-0.3240	-0.4680 to -0.1800	Yes
18588	17:40 X vs. 28:50 X	-0.2193	-0.3633 to -0.07537	Yes
18589	17:40 X vs. 29:0 X	-1.685	-1.846 to -1.524	Yes
18590	17:40 X vs. 29:1 X	-1.770	-1.931 to -1.609	Yes
18591	17:40 X vs. 29:10 X	-0.9845	-1.145 to -0.8235	Yes
18592	17:40 X vs. 29:40 X	-0.4320	-0.5760 to -0.2880	Yes
18593	17:40 X vs. 29:50 X	-0.2207	-0.3646 to -0.07671	Yes
18594	17:40 X vs. 30:0 X	-1.400	-1.560 to -1.239	Yes
18595	17:40 X vs. 30:1 X	-1.721	-1.882 to -1.560	Yes
18596	17:40 X vs. 30:10 X	-1.059	-1.220 to -0.8980	Yes
18597	17:40 X vs. 30:40 X	-0.3590	-0.5030 to -0.2150	Yes
18598	17:40 X vs. 30:50 X	-0.2730	-0.4170 to -0.1290	Yes
18599	17:40 X vs. 31:0 X	-1.718	-1.878 to -1.557	Yes
18600	17:40 X vs. 31:1 X	-1.708	-1.869 to -1.547	Yes
18601	17:40 X vs. 31:10 X	-1.191	-1.351 to -1.030	Yes
18602	17:40 X vs. 31:40 X	-0.4507	-0.5946 to -0.3067	Yes
18603	17:40 X vs. 31:50 X	-0.2520	-0.3960 to -0.1080	Yes
18604	17:40 X vs. 32:0 X	-2.309	-2.453 to -2.165	Yes
18605	17:40 X vs. 32:1 X	-1.765	-1.926 to -1.604	Yes
18606	17:40 X vs. 32:10 X	-1.621	-1.765 to -1.477	Yes
18607	17:40 X vs. 32:40 X	-0.4470	-0.5910 to -0.3030	Yes
18608	17:40 X vs. 32:50 X	-0.2517	-0.3956 to -0.1077	Yes
18609	17:40 X vs. 33:0 X	-2.313	-2.457 to -2.169	Yes
18610	17:40 X vs. 33:1 X	-2.127	-2.271 to -1.983	Yes
18611	17:40 X vs. 33:10 X	-1.788	-1.932 to -1.644	Yes
18612	17:40 X vs. 33:40 X	-0.4657	-0.6096 to -0.3217	Yes
18613	17:40 X vs. 33:50 X	-0.2667	-0.4106 to -0.1227	Yes
18614	17:40 X vs. 34:0 X	-2.237	-2.381 to -2.093	Yes
18615	17:40 X vs. 34:1 X	-2.245	-2.389 to -2.101	Yes
18616	17:40 X vs. 34:10 X	-1.878	-2.022 to -1.734	Yes
18617	17:40 X vs. 34:40 X	-0.4777	-0.6216 to -0.3337	Yes
18618	17:40 X vs. 34:50 X	-0.4157	-0.5596 to -0.2717	Yes
18619	17:40 X vs. 35:0 X	-2.586	-2.730 to -2.442	Yes
18620	17:40 X vs. 35:1 X	-2.352	-2.496 to -2.208	Yes
18621	17:40 X vs. 35:10 X	-1.840	-1.984 to -1.696	Yes
18622	17:40 X vs. 35:40 X	-0.4867	-0.6306 to -0.3427	Yes
18623	17:40 X vs. 35:50 X	-0.4283	-0.5723 to -0.2844	Yes
18624	17:40 X vs. 36:0 X	-2.793	-2.953 to -2.632	Yes
18625	17:40 X vs. 36:1 X	-2.488	-2.632 to -2.344	Yes
18626	17:40 X vs. 36:10 X	-1.845	-1.989 to -1.701	Yes
18627	17:40 X vs. 36:40 X	-0.5420	-0.6860 to -0.3980	Yes
18628	17:40 X vs. 36:50 X	-0.3917	-0.5356 to -0.2477	Yes
18629	17:40 X vs. 37:0 X	-3.176	-3.320 to -3.032	Yes
18630	17:40 X vs. 37:1 X	-2.558	-2.702 to -2.414	Yes

	2way ANOVA<br>Multiple comparisons	A<br>Data Set-A<br>Y	B<br>Data Set-B<br>Y	C<br>Data Set-C<br>Y
18631	17:40 X vs. 37:10 X	-1.847	-1.991 to -1.703	Yes
18632	17:40 X vs. 37:40 X	-0.5793	-0.7233 to -0.4354	Yes
18633	17:40 X vs. 37:50 X	-0.4003	-0.5443 to -0.2564	Yes
18634	17:40 X vs. 38:0 X	-3.121	-3.265 to -2.977	Yes
18635	17:40 X vs. 38:1 X	-2.677	-2.821 to -2.533	Yes
18636	17:40 X vs. 38:10 X	-2.122	-2.266 to -1.978	Yes
18637	17:40 X vs. 38:40 X	-0.5837	-0.7276 to -0.4397	Yes
18638	17:40 X vs. 38:50 X	-0.4043	-0.5483 to -0.2604	Yes
18639	17:40 X vs. 39:0 X	-3.352	-3.496 to -3.208	Yes
18640	17:40 X vs. 39:1 X	-3.244	-3.388 to -3.100	Yes
18641	17:40 X vs. 39:10 X	-2.267	-2.411 to -2.123	Yes
18642	17:40 X vs. 39:40 X	-0.5897	-0.7336 to -0.4457	Yes
18643	17:40 X vs. 39:50 X	-0.4353	-0.5793 to -0.2914	Yes
18644	17:40 X vs. 40:0 X	-3.393	-3.537 to -3.249	Yes
18645	17:40 X vs. 40:1 X	-3.319	-3.463 to -3.175	Yes
18646	17:40 X vs. 40:10 X	-2.524	-2.668 to -2.380	Yes
18647	17:40 X vs. 40:40 X	-0.5943	-0.7383 to -0.4504	Yes
18648	17:40 X vs. 40:50 X	-0.4417	-0.5856 to -0.2977	Yes
18649	17:40 X vs. 41:0 X	-3.434	-3.578 to -3.290	Yes
18650	17:40 X vs. 41:1 X	-3.433	-3.577 to -3.289	Yes
18651	17:40 X vs. 41:10 X	-2.577	-2.721 to -2.433	Yes
18652	17:40 X vs. 41:40 X	-0.5417	-0.6856 to -0.3977	Yes
18653	17:40 X vs. 41:50 X	-0.4543	-0.5983 to -0.3104	Yes
18654	17:40 X vs. 42:0 X	-3.576	-3.720 to -3.432	Yes
18655	17:40 X vs. 42:1 X	-3.595	-3.739 to -3.451	Yes
18656	17:40 X vs. 42:10 X	-2.578	-2.722 to -2.434	Yes
18657	17:40 X vs. 42:40 X	-0.5543	-0.6983 to -0.4104	Yes
18658	17:40 X vs. 42:50 X	-0.4577	-0.6016 to -0.3137	Yes
18659	17:40 X vs. 43:0 X	-3.666	-3.810 to -3.522	Yes
18660	17:40 X vs. 43:1 X	-3.580	-3.724 to -3.436	Yes
18661	17:40 X vs. 43:10 X	-3.296	-3.440 to -3.152	Yes
18662	17:40 X vs. 43:40 X	-0.5527	-0.6966 to -0.4087	Yes
18663	17:40 X vs. 43:50 X	-0.4580	-0.6020 to -0.3140	Yes
18664	17:40 X vs. 44:0 X	-3.690	-3.834 to -3.546	Yes
18665	17:40 X vs. 44:1 X	-3.579	-3.723 to -3.435	Yes
18666	17:40 X vs. 44:10 X	-3.359	-3.503 to -3.215	Yes
18667	17:40 X vs. 44:40 X	-0.5547	-0.6986 to -0.4107	Yes
18668	17:40 X vs. 44:50 X	-0.3903	-0.5343 to -0.2464	Yes
18669	17:40 X vs. 45:0 X	-3.554	-3.698 to -3.410	Yes
18670	17:40 X vs. 45:1 X	-3.356	-3.500 to -3.212	Yes
18671	17:40 X vs. 45:10 X	-3.239	-3.383 to -3.095	Yes
18672	17:40 X vs. 45:40 X	-0.5560	-0.7000 to -0.4120	Yes
18673	17:40 X vs. 45:50 X	-0.3873	-0.5313 to -0.2434	Yes
18674	17:40 X vs. 46:0 X	-3.359	-3.503 to -3.215	Yes
18675	17:40 X vs. 46:1 X	-3.326	-3.470 to -3.182	Yes

	2way ANOVA<br>Multiple comparisons	A<br>Data Set-A<br>Y	B<br>Data Set-B<br>Y	C<br>Data Set-C<br>Y
18676	17:40 X vs. 46:10 X	-3.234	-3.378 to -3.090	Yes
18677	17:40 X vs. 46:40 X	-0.5697	-0.7136 to -0.4257	Yes
18678	17:40 X vs. 46:50 X	-0.3803	-0.5243 to -0.2364	Yes
18679	17:40 X vs. 47:0 X	-3.334	-3.478 to -3.190	Yes
18680	17:40 X vs. 47:1 X	-3.238	-3.382 to -3.094	Yes
18681	17:40 X vs. 47:10 X	-3.220	-3.364 to -3.076	Yes
18682	17:40 X vs. 47:40 X	-0.5797	-0.7236 to -0.4357	Yes
18683	17:40 X vs. 47:50 X	-0.3383	-0.4823 to -0.1944	Yes
18684	17:40 X vs. 48:0 X	-3.322	-3.482 to -3.161	Yes
18685	17:40 X vs. 48:1 X	-3.225	-3.369 to -3.081	Yes
18686	17:40 X vs. 48:10 X	-3.220	-3.364 to -3.076	Yes
18687	17:40 X vs. 48:40 X	-0.5860	-0.7300 to -0.4420	Yes
18688	17:40 X vs. 48:50 X	-0.3457	-0.4896 to -0.2017	Yes
18689	17:40 X vs. 49:0 X	-3.322	-3.466 to -3.178	Yes
18690	17:40 X vs. 49:1 X	-3.225	-3.369 to -3.081	Yes
18691	17:40 X vs. 49:10 X	-3.220	-3.364 to -3.076	Yes
18692	17:40 X vs. 49:40 X	-0.5903	-0.7343 to -0.4464	Yes
18693	17:40 X vs. 49:50 X	-0.3450	-0.4890 to -0.2010	Yes
18694	17:40 X vs. 50:0 X	-3.322	-3.466 to -3.178	Yes
18695	17:40 X vs. 50:1 X	-3.225	-3.369 to -3.081	Yes
18696	17:40 X vs. 50:10 X	-3.220	-3.364 to -3.076	Yes
18697	17:40 X vs. 50:40 X	-0.5980	-0.7420 to -0.4540	Yes
18698	17:40 X vs. 50:50 X	-0.3447	-0.4886 to -0.2007	Yes
18699	17:50 X vs. 18:0 X	-0.1090	-0.2530 to 0.03496	No
18700	17:50 X vs. 18:1 X	-0.01733	-0.1613 to 0.1266	No
18701	17:50 X vs. 18:10 X	-0.1097	-0.2536 to 0.03429	No
18702	17:50 X vs. 18:40 X	0.0410	-0.1030 to 0.1850	No
18703	17:50 X vs. 18:50 X	-0.004000	-0.1480 to 0.1400	No
18704	17:50 X vs. 19:0 X	-0.1440	-0.2880 to -4.018e-005	Yes
18705	17:50 X vs. 19:1 X	-0.1320	-0.2760 to 0.01196	No
18706	17:50 X vs. 19:10 X	-0.1297	-0.2736 to 0.01429	No
18707	17:50 X vs. 19:40 X	0.01233	-0.1316 to 0.1563	No
18708	17:50 X vs. 19:50 X	0.001333	-0.1426 to 0.1453	No
18709	17:50 X vs. 20:0 X	-0.2237	-0.3676 to -0.07971	Yes
18710	17:50 X vs. 20:1 X	-0.0430	-0.1870 to 0.1010	No
18711	17:50 X vs. 20:10 X	-0.1320	-0.2760 to 0.01196	No
18712	17:50 X vs. 20:40 X	0.0006667	-0.1433 to 0.1446	No
18713	17:50 X vs. 20:50 X	-0.04333	-0.1873 to 0.1006	No
18714	17:50 X vs. 21:0 X	-0.2470	-0.3910 to -0.1030	Yes
18715	17:50 X vs. 21:1 X	-0.2000	-0.3440 to -0.05604	Yes
18716	17:50 X vs. 21:10 X	-0.1970	-0.3410 to -0.05304	Yes
18717	17:50 X vs. 21:40 X	-0.07233	-0.2163 to 0.07163	No
18718	17:50 X vs. 21:50 X	-0.1627	-0.3066 to -0.01871	Yes
18719	17:50 X vs. 22:0 X	-0.2887	-0.4326 to -0.1447	Yes
18720	17:50 X vs. 22:1 X	-0.2833	-0.4273 to -0.1394	Yes

2way ANOVA Multiple comparisons	A Data Set-A Y	B Data Set-B Y	C Data Set-C Y
18721 17:50 X vs. 22:10 X	-0.2787	-0.4226 to -0.1347	Yes
18722 17:50 X vs. 22:40 X	-0.1790	-0.3230 to -0.03504	Yes
18723 17:50 X vs. 22:50 X	-0.2417	-0.3856 to -0.09771	Yes
18724 17:50 X vs. 23:0 X	-0.3540	-0.4980 to -0.2100	Yes
18725 17:50 X vs. 23:1 X	-0.5437	-0.7046 to -0.3827	Yes
18726 17:50 X vs. 23:10 X	-0.2933	-0.4373 to -0.1494	Yes
18727 17:50 X vs. 23:40 X	-0.1737	-0.3346 to -0.01271	Yes
18728 17:50 X vs. 23:50 X	-0.2077	-0.3516 to -0.06371	Yes
18729 17:50 X vs. 24:0 X	-0.4713	-0.6153 to -0.3274	Yes
18730 17:50 X vs. 24:1 X	-0.6277	-0.7886 to -0.4667	Yes
18731 17:50 X vs. 24:10 X	-0.4552	-0.6161 to -0.2942	Yes
18732 17:50 X vs. 24:40 X	-0.1900	-0.3340 to -0.04604	Yes
18733 17:50 X vs. 24:50 X	-0.1117	-0.2556 to 0.03229	No
18734 17:50 X vs. 25:0 X	-0.8087	-0.9696 to -0.6477	Yes
18735 17:50 X vs. 25:1 X	-0.9982	-1.159 to -0.8372	Yes
18736 17:50 X vs. 25:10 X	-0.3597	-0.5633 to -0.1561	Yes
18737 17:50 X vs. 25:40 X	-0.1920	-0.3360 to -0.04804	Yes
18738 17:50 X vs. 25:50 X	-0.1663	-0.3103 to -0.02237	Yes
18739 17:50 X vs. 26:0 X	-1.021	-1.182 to -0.8602	Yes
18740 17:50 X vs. 26:1 X	-1.134	-1.295 to -0.9732	Yes
18741 17:50 X vs. 26:10 X	-0.8207	-0.9816 to -0.6597	Yes
18742 17:50 X vs. 26:40 X	-0.2663	-0.4103 to -0.1224	Yes
18743 17:50 X vs. 26:50 X	-0.1267	-0.2706 to 0.01729	No
18744 17:50 X vs. 27:0 X	-1.209	-1.353 to -1.065	Yes
18745 17:50 X vs. 27:1 X	-1.300	-1.461 to -1.139	Yes
18746 17:50 X vs. 27:10 X	-1.112	-1.273 to -0.9507	Yes
18747 17:50 X vs. 27:40 X	-0.2663	-0.4103 to -0.1224	Yes
18748 17:50 X vs. 27:50 X	-0.1260	-0.2700 to 0.01796	No
18749 17:50 X vs. 28:0 X	-1.158	-1.319 to -0.9972	Yes
18750 17:50 X vs. 28:1 X	-1.199	-1.343 to -1.055	Yes
18751 17:50 X vs. 28:10 X	-1.001	-1.162 to -0.8397	Yes
18752 17:50 X vs. 28:40 X	-0.2787	-0.4226 to -0.1347	Yes
18753 17:50 X vs. 28:50 X	-0.1740	-0.3180 to -0.03004	Yes
18754 17:50 X vs. 29:0 X	-1.640	-1.801 to -1.479	Yes
18755 17:50 X vs. 29:1 X	-1.725	-1.886 to -1.564	Yes
18756 17:50 X vs. 29:10 X	-0.9392	-1.100 to -0.7782	Yes
18757 17:50 X vs. 29:40 X	-0.3867	-0.5306 to -0.2427	Yes
18758 17:50 X vs. 29:50 X	-0.1753	-0.3193 to -0.03137	Yes
18759 17:50 X vs. 30:0 X	-1.354	-1.515 to -1.193	Yes
18760 17:50 X vs. 30:1 X	-1.676	-1.837 to -1.515	Yes
18761 17:50 X vs. 30:10 X	-1.014	-1.175 to -0.8527	Yes
18762 17:50 X vs. 30:40 X	-0.3137	-0.4576 to -0.1697	Yes
18763 17:50 X vs. 30:50 X	-0.2277	-0.3716 to -0.08371	Yes
18764 17:50 X vs. 31:0 X	-1.672	-1.833 to -1.511	Yes
18765 17:50 X vs. 31:1 X	-1.663	-1.824 to -1.502	Yes

	2way ANOVA<br>Multiple comparisons	A<br>Data Set-A<br>Y	B<br>Data Set-B<br>Y	C<br>Data Set-C<br>Y
18766	17:**50 X** vs. 31:**10 X**	-1.145	-1.306 to -0.9842	Yes
18767	17:**50 X** vs. 31:**40 X**	-0.4053	-0.5493 to -0.2614	Yes
18768	17:**50 X** vs. 31:**50 X**	-0.2067	-0.3506 to -0.06271	Yes
18769	17:**50 X** vs. 32:**0 X**	-2.264	-2.408 to -2.120	Yes
18770	17:**50 X** vs. 32:**1 X**	-1.720	-1.881 to -1.559	Yes
18771	17:**50 X** vs. 32:**10 X**	-1.576	-1.720 to -1.432	Yes
18772	17:**50 X** vs. 32:**40 X**	-0.4017	-0.5456 to -0.2577	Yes
18773	17:**50 X** vs. 32:**50 X**	-0.2063	-0.3503 to -0.06237	Yes
18774	17:**50 X** vs. 33:**0 X**	-2.267	-2.411 to -2.123	Yes
18775	17:**50 X** vs. 33:**1 X**	-2.082	-2.226 to -1.938	Yes
18776	17:**50 X** vs. 33:**10 X**	-1.742	-1.886 to -1.598	Yes
18777	17:**50 X** vs. 33:**40 X**	-0.4203	-0.5643 to -0.2764	Yes
18778	17:**50 X** vs. 33:**50 X**	-0.2213	-0.3653 to -0.07737	Yes
18779	17:**50 X** vs. 34:**0 X**	-2.192	-2.336 to -2.048	Yes
18780	17:**50 X** vs. 34:**1 X**	-2.199	-2.343 to -2.055	Yes
18781	17:**50 X** vs. 34:**10 X**	-1.833	-1.977 to -1.689	Yes
18782	17:**50 X** vs. 34:**40 X**	-0.4323	-0.5763 to -0.2884	Yes
18783	17:**50 X** vs. 34:**50 X**	-0.3703	-0.5143 to -0.2264	Yes
18784	17:**50 X** vs. 35:**0 X**	-2.541	-2.685 to -2.397	Yes
18785	17:**50 X** vs. 35:**1 X**	-2.306	-2.450 to -2.162	Yes
18786	17:**50 X** vs. 35:**10 X**	-1.794	-1.938 to -1.650	Yes
18787	17:**50 X** vs. 35:**40 X**	-0.4413	-0.5853 to -0.2974	Yes
18788	17:**50 X** vs. 35:**50 X**	-0.3830	-0.5270 to -0.2390	Yes
18789	17:**50 X** vs. 36:**0 X**	-2.747	-2.908 to -2.586	Yes
18790	17:**50 X** vs. 36:**1 X**	-2.443	-2.587 to -2.299	Yes
18791	17:**50 X** vs. 36:**10 X**	-1.800	-1.944 to -1.656	Yes
18792	17:**50 X** vs. 36:**40 X**	-0.4967	-0.6406 to -0.3527	Yes
18793	17:**50 X** vs. 36:**50 X**	-0.3463	-0.4903 to -0.2024	Yes
18794	17:**50 X** vs. 37:**0 X**	-3.131	-3.275 to -2.987	Yes
18795	17:**50 X** vs. 37:**1 X**	-2.513	-2.657 to -2.369	Yes
18796	17:**50 X** vs. 37:**10 X**	-1.802	-1.946 to -1.658	Yes
18797	17:**50 X** vs. 37:**40 X**	-0.5340	-0.6780 to -0.3900	Yes
18798	17:**50 X** vs. 37:**50 X**	-0.3550	-0.4990 to -0.2110	Yes
18799	17:**50 X** vs. 38:**0 X**	-3.076	-3.220 to -2.932	Yes
18800	17:**50 X** vs. 38:**1 X**	-2.632	-2.776 to -2.488	Yes
18801	17:**50 X** vs. 38:**10 X**	-2.077	-2.221 to -1.933	Yes
18802	17:**50 X** vs. 38:**40 X**	-0.5383	-0.6823 to -0.3944	Yes
18803	17:**50 X** vs. 38:**50 X**	-0.3590	-0.5030 to -0.2150	Yes
18804	17:**50 X** vs. 39:**0 X**	-3.307	-3.451 to -3.163	Yes
18805	17:**50 X** vs. 39:**1 X**	-3.198	-3.342 to -3.054	Yes
18806	17:**50 X** vs. 39:**10 X**	-2.222	-2.366 to -2.078	Yes
18807	17:**50 X** vs. 39:**40 X**	-0.5443	-0.6883 to -0.4004	Yes
18808	17:**50 X** vs. 39:**50 X**	-0.3900	-0.5340 to -0.2460	Yes
18809	17:**50 X** vs. 40:**0 X**	-3.348	-3.492 to -3.204	Yes
18810	17:**50 X** vs. 40:**1 X**	-3.274	-3.418 to -3.130	Yes

	2way ANOVA<br>Multiple comparisons	A<br>Data Set-A<br>Y	B<br>Data Set-B<br>Y	C<br>Data Set-C<br>Y
18811	17:50 X vs. 40:10 X	-2.478	-2.622 to -2.334	Yes
18812	17:50 X vs. 40:40 X	-0.5490	-0.6930 to -0.4050	Yes
18813	17:50 X vs. 40:50 X	-0.3963	-0.5403 to -0.2524	Yes
18814	17:50 X vs. 41:0 X	-3.389	-3.533 to -3.245	Yes
18815	17:50 X vs. 41:1 X	-3.388	-3.532 to -3.244	Yes
18816	17:50 X vs. 41:10 X	-2.532	-2.676 to -2.388	Yes
18817	17:50 X vs. 41:40 X	-0.4963	-0.6403 to -0.3524	Yes
18818	17:50 X vs. 41:50 X	-0.4090	-0.5530 to -0.2650	Yes
18819	17:50 X vs. 42:0 X	-3.530	-3.674 to -3.386	Yes
18820	17:50 X vs. 42:1 X	-3.550	-3.694 to -3.406	Yes
18821	17:50 X vs. 42:10 X	-2.532	-2.676 to -2.388	Yes
18822	17:50 X vs. 42:40 X	-0.5090	-0.6530 to -0.3650	Yes
18823	17:50 X vs. 42:50 X	-0.4123	-0.5563 to -0.2684	Yes
18824	17:50 X vs. 43:0 X	-3.620	-3.764 to -3.476	Yes
18825	17:50 X vs. 43:1 X	-3.535	-3.679 to -3.391	Yes
18826	17:50 X vs. 43:10 X	-3.250	-3.394 to -3.106	Yes
18827	17:50 X vs. 43:40 X	-0.5073	-0.6513 to -0.3634	Yes
18828	17:50 X vs. 43:50 X	-0.4127	-0.5566 to -0.2687	Yes
18829	17:50 X vs. 44:0 X	-3.644	-3.788 to -3.500	Yes
18830	17:50 X vs. 44:1 X	-3.534	-3.678 to -3.390	Yes
18831	17:50 X vs. 44:10 X	-3.314	-3.458 to -3.170	Yes
18832	17:50 X vs. 44:40 X	-0.5093	-0.6533 to -0.3654	Yes
18833	17:50 X vs. 44:50 X	-0.3450	-0.4890 to -0.2010	Yes
18834	17:50 X vs. 45:0 X	-3.509	-3.653 to -3.365	Yes
18835	17:50 X vs. 45:1 X	-3.311	-3.455 to -3.167	Yes
18836	17:50 X vs. 45:10 X	-3.193	-3.337 to -3.049	Yes
18837	17:50 X vs. 45:40 X	-0.5107	-0.6546 to -0.3667	Yes
18838	17:50 X vs. 45:50 X	-0.3420	-0.4860 to -0.1980	Yes
18839	17:50 X vs. 46:0 X	-3.313	-3.457 to -3.169	Yes
18840	17:50 X vs. 46:1 X	-3.280	-3.424 to -3.136	Yes
18841	17:50 X vs. 46:10 X	-3.188	-3.332 to -3.044	Yes
18842	17:50 X vs. 46:40 X	-0.5243	-0.6683 to -0.3804	Yes
18843	17:50 X vs. 46:50 X	-0.3350	-0.4790 to -0.1910	Yes
18844	17:50 X vs. 47:0 X	-3.289	-3.433 to -3.145	Yes
18845	17:50 X vs. 47:1 X	-3.193	-3.337 to -3.049	Yes
18846	17:50 X vs. 47:10 X	-3.174	-3.318 to -3.030	Yes
18847	17:50 X vs. 47:40 X	-0.5343	-0.6783 to -0.3904	Yes
18848	17:50 X vs. 47:50 X	-0.2930	-0.4370 to -0.1490	Yes
18849	17:50 X vs. 48:0 X	-3.276	-3.437 to -3.115	Yes
18850	17:50 X vs. 48:1 X	-3.180	-3.324 to -3.036	Yes
18851	17:50 X vs. 48:10 X	-3.174	-3.318 to -3.030	Yes
18852	17:50 X vs. 48:40 X	-0.5407	-0.6846 to -0.3967	Yes
18853	17:50 X vs. 48:50 X	-0.3003	-0.4443 to -0.1564	Yes
18854	17:50 X vs. 49:0 X	-3.276	-3.420 to -3.132	Yes
18855	17:50 X vs. 49:1 X	-3.180	-3.324 to -3.036	Yes

	2way ANOVA<br>Multiple comparisons	A<br>Data Set-A<br>Y	B<br>Data Set-B<br>Y	C<br>Data Set-C<br>Y
18856	17:**50 X** vs. 49:**10 X**	-3.174	-3.318 to -3.030	Yes
18857	17:**50 X** vs. 49:**40 X**	-0.5450	-0.6890 to -0.4010	Yes
18858	17:**50 X** vs. 49:**50 X**	-0.2997	-0.4436 to -0.1557	Yes
18859	17:**50 X** vs. 50:**0 X**	-3.276	-3.420 to -3.132	Yes
18860	17:**50 X** vs. 50:**1 X**	-3.180	-3.324 to -3.036	Yes
18861	17:**50 X** vs. 50:**10 X**	-3.174	-3.318 to -3.030	Yes
18862	17:**50 X** vs. 50:**40 X**	-0.5527	-0.6966 to -0.4087	Yes
18863	17:**50 X** vs. 50:**50 X**	-0.2993	-0.4433 to -0.1554	Yes
18864	18:**0 X** vs. 18:**1 X**	0.09167	-0.05229 to 0.2356	No
18865	18:**0 X** vs. 18:**10 X**	-0.0006667	-0.1446 to 0.1433	No
18866	18:**0 X** vs. 18:**40 X**	0.1500	0.006040 to 0.2940	Yes
18867	18:**0 X** vs. 18:**50 X**	0.1050	-0.03896 to 0.2490	No
18868	18:**0 X** vs. 19:**0 X**	-0.0350	-0.1790 to 0.1090	No
18869	18:**0 X** vs. 19:**1 X**	-0.02300	-0.1670 to 0.1210	No
18870	18:**0 X** vs. 19:**10 X**	-0.02067	-0.1646 to 0.1233	No
18871	18:**0 X** vs. 19:**40 X**	0.1213	-0.02263 to 0.2653	No
18872	18:**0 X** vs. 19:**50 X**	0.1103	-0.03363 to 0.2543	No
18873	18:**0 X** vs. 20:**0 X**	-0.1147	-0.2586 to 0.02929	No
18874	18:**0 X** vs. 20:**1 X**	0.06600	-0.07796 to 0.2100	No
18875	18:**0 X** vs. 20:**10 X**	-0.02300	-0.1670 to 0.1210	No
18876	18:**0 X** vs. 20:**40 X**	0.1097	-0.03429 to 0.2536	No
18877	18:**0 X** vs. 20:**50 X**	0.06567	-0.07829 to 0.2096	No
18878	18:**0 X** vs. 21:**0 X**	-0.1380	-0.2820 to 0.005960	No
18879	18:**0 X** vs. 21:**1 X**	-0.09100	-0.2350 to 0.05296	No
18880	18:**0 X** vs. 21:**10 X**	-0.0880	-0.2320 to 0.05596	No
18881	18:**0 X** vs. 21:**40 X**	0.03667	-0.1073 to 0.1806	No
18882	18:**0 X** vs. 21:**50 X**	-0.05367	-0.1976 to 0.09029	No
18883	18:**0 X** vs. 22:**0 X**	-0.1797	-0.3236 to -0.03571	Yes
18884	18:**0 X** vs. 22:**1 X**	-0.1743	-0.3183 to -0.03037	Yes
18885	18:**0 X** vs. 22:**10 X**	-0.1697	-0.3136 to -0.02571	Yes
18886	18:**0 X** vs. 22:**40 X**	-0.07000	-0.2140 to 0.07396	No
18887	18:**0 X** vs. 22:**50 X**	-0.1327	-0.2766 to 0.01129	No
18888	18:**0 X** vs. 23:**0 X**	-0.2450	-0.3890 to -0.1010	Yes
18889	18:**0 X** vs. 23:**1 X**	-0.4347	-0.5956 to -0.2737	Yes
18890	18:**0 X** vs. 23:**10 X**	-0.1843	-0.3283 to -0.04037	Yes
18891	18:**0 X** vs. 23:**40 X**	-0.06467	-0.2256 to 0.09629	No
18892	18:**0 X** vs. 23:**50 X**	-0.09867	-0.2426 to 0.04529	No
18893	18:**0 X** vs. 24:**0 X**	-0.3623	-0.5063 to -0.2184	Yes
18894	18:**0 X** vs. 24:**1 X**	-0.5187	-0.6796 to -0.3577	Yes
18895	18:**0 X** vs. 24:**10 X**	-0.3462	-0.5071 to -0.1852	Yes
18896	18:**0 X** vs. 24:**40 X**	-0.0810	-0.2250 to 0.06296	No
18897	18:**0 X** vs. 24:**50 X**	-0.002667	-0.1466 to 0.1413	No
18898	18:**0 X** vs. 25:**0 X**	-0.6997	-0.8606 to -0.5387	Yes
18899	18:**0 X** vs. 25:**1 X**	-0.8892	-1.050 to -0.7282	Yes
18900	18:**0 X** vs. 25:**10 X**	-0.2507	-0.4543 to -0.04708	Yes

	2way ANOVA<br>Multiple comparisons	A<br>Data Set-A<br>Y	B<br>Data Set-B<br>Y	C<br>Data Set-C<br>Y
18901	18:**0 X** vs. 25:**40 X**	-0.0830	-0.2270 to 0.06096	No
18902	18:**0 X** vs. 25:**50 X**	-0.05733	-0.2013 to 0.08663	No
18903	18:**0 X** vs. 26:**0 X**	-0.9122	-1.073 to -0.7512	Yes
18904	18:**0 X** vs. 26:**1 X**	-1.025	-1.186 to -0.8642	Yes
18905	18:**0 X** vs. 26:**10 X**	-0.7117	-0.8726 to -0.5507	Yes
18906	18:**0 X** vs. 26:**40 X**	-0.1573	-0.3013 to -0.01337	Yes
18907	18:**0 X** vs. 26:**50 X**	-0.01767	-0.1616 to 0.1263	No
18908	18:**0 X** vs. 27:**0 X**	-1.100	-1.244 to -0.9560	Yes
18909	18:**0 X** vs. 27:**1 X**	-1.191	-1.352 to -1.030	Yes
18910	18:**0 X** vs. 27:**10 X**	-1.003	-1.164 to -0.8417	Yes
18911	18:**0 X** vs. 27:**40 X**	-0.1573	-0.3013 to -0.01337	Yes
18912	18:**0 X** vs. 27:**50 X**	-0.0170	-0.1610 to 0.1270	No
18913	18:**0 X** vs. 28:**0 X**	-1.049	-1.210 to -0.8882	Yes
18914	18:**0 X** vs. 28:**1 X**	-1.090	-1.234 to -0.9460	Yes
18915	18:**0 X** vs. 28:**10 X**	-0.8917	-1.053 to -0.7307	Yes
18916	18:**0 X** vs. 28:**40 X**	-0.1697	-0.3136 to -0.02571	Yes
18917	18:**0 X** vs. 28:**50 X**	-0.06500	-0.2090 to 0.07896	No
18918	18:**0 X** vs. 29:**0 X**	-1.531	-1.692 to -1.370	Yes
18919	18:**0 X** vs. 29:**1 X**	-1.616	-1.777 to -1.455	Yes
18920	18:**0 X** vs. 29:**10 X**	-0.8302	-0.9911 to -0.6692	Yes
18921	18:**0 X** vs. 29:**40 X**	-0.2777	-0.4216 to -0.1337	Yes
18922	18:**0 X** vs. 29:**50 X**	-0.06633	-0.2103 to 0.07763	No
18923	18:**0 X** vs. 30:**0 X**	-1.245	-1.406 to -1.084	Yes
18924	18:**0 X** vs. 30:**1 X**	-1.567	-1.728 to -1.406	Yes
18925	18:**0 X** vs. 30:**10 X**	-0.9047	-1.066 to -0.7437	Yes
18926	18:**0 X** vs. 30:**40 X**	-0.2047	-0.3486 to -0.06071	Yes
18927	18:**0 X** vs. 30:**50 X**	-0.1187	-0.2626 to 0.02529	No
18928	18:**0 X** vs. 31:**0 X**	-1.563	-1.724 to -1.402	Yes
18929	18:**0 X** vs. 31:**1 X**	-1.554	-1.715 to -1.393	Yes
18930	18:**0 X** vs. 31:**10 X**	-1.036	-1.197 to -0.8752	Yes
18931	18:**0 X** vs. 31:**40 X**	-0.2963	-0.4403 to -0.1524	Yes
18932	18:**0 X** vs. 31:**50 X**	-0.09767	-0.2416 to 0.04629	No
18933	18:**0 X** vs. 32:**0 X**	-2.155	-2.299 to -2.011	Yes
18934	18:**0 X** vs. 32:**1 X**	-1.611	-1.772 to -1.450	Yes
18935	18:**0 X** vs. 32:**10 X**	-1.467	-1.611 to -1.323	Yes
18936	18:**0 X** vs. 32:**40 X**	-0.2927	-0.4366 to -0.1487	Yes
18937	18:**0 X** vs. 32:**50 X**	-0.09733	-0.2413 to 0.04663	No
18938	18:**0 X** vs. 33:**0 X**	-2.158	-2.302 to -2.014	Yes
18939	18:**0 X** vs. 33:**1 X**	-1.973	-2.117 to -1.829	Yes
18940	18:**0 X** vs. 33:**10 X**	-1.633	-1.777 to -1.489	Yes
18941	18:**0 X** vs. 33:**40 X**	-0.3113	-0.4553 to -0.1674	Yes
18942	18:**0 X** vs. 33:**50 X**	-0.1123	-0.2563 to 0.03163	No
18943	18:**0 X** vs. 34:**0 X**	-2.083	-2.227 to -1.939	Yes
18944	18:**0 X** vs. 34:**1 X**	-2.090	-2.234 to -1.946	Yes
18945	18:**0 X** vs. 34:**10 X**	-1.724	-1.868 to -1.580	Yes

	2way ANOVA<br>Multiple comparisons	A<br>Data Set-A<br>Y	B<br>Data Set-B<br>Y	C<br>Data Set-C<br>Y
18946	18:**0 X** vs. 34:**40 X**	-0.3233	-0.4673 to -0.1794	Yes
18947	18:**0 X** vs. 34:**50 X**	-0.2613	-0.4053 to -0.1174	Yes
18948	18:**0 X** vs. 35:**0 X**	-2.432	-2.576 to -2.288	Yes
18949	18:**0 X** vs. 35:**1 X**	-2.197	-2.341 to -2.053	Yes
18950	18:**0 X** vs. 35:**10 X**	-1.685	-1.829 to -1.541	Yes
18951	18:**0 X** vs. 35:**40 X**	-0.3323	-0.4763 to -0.1884	Yes
18952	18:**0 X** vs. 35:**50 X**	-0.2740	-0.4180 to -0.1300	Yes
18953	18:**0 X** vs. 36:**0 X**	-2.638	-2.799 to -2.477	Yes
18954	18:**0 X** vs. 36:**1 X**	-2.334	-2.478 to -2.190	Yes
18955	18:**0 X** vs. 36:**10 X**	-1.691	-1.835 to -1.547	Yes
18956	18:**0 X** vs. 36:**40 X**	-0.3877	-0.5316 to -0.2437	Yes
18957	18:**0 X** vs. 36:**50 X**	-0.2373	-0.3813 to -0.09337	Yes
18958	18:**0 X** vs. 37:**0 X**	-3.022	-3.166 to -2.878	Yes
18959	18:**0 X** vs. 37:**1 X**	-2.404	-2.548 to -2.260	Yes
18960	18:**0 X** vs. 37:**10 X**	-1.693	-1.837 to -1.549	Yes
18961	18:**0 X** vs. 37:**40 X**	-0.4250	-0.5690 to -0.2810	Yes
18962	18:**0 X** vs. 37:**50 X**	-0.2460	-0.3900 to -0.1020	Yes
18963	18:**0 X** vs. 38:**0 X**	-2.967	-3.111 to -2.823	Yes
18964	18:**0 X** vs. 38:**1 X**	-2.523	-2.667 to -2.379	Yes
18965	18:**0 X** vs. 38:**10 X**	-1.968	-2.112 to -1.824	Yes
18966	18:**0 X** vs. 38:**40 X**	-0.4293	-0.5733 to -0.2854	Yes
18967	18:**0 X** vs. 38:**50 X**	-0.2500	-0.3940 to -0.1060	Yes
18968	18:**0 X** vs. 39:**0 X**	-3.198	-3.342 to -3.054	Yes
18969	18:**0 X** vs. 39:**1 X**	-3.089	-3.233 to -2.945	Yes
18970	18:**0 X** vs. 39:**10 X**	-2.113	-2.257 to -1.969	Yes
18971	18:**0 X** vs. 39:**40 X**	-0.4353	-0.5793 to -0.2914	Yes
18972	18:**0 X** vs. 39:**50 X**	-0.2810	-0.4250 to -0.1370	Yes
18973	18:**0 X** vs. 40:**0 X**	-3.239	-3.383 to -3.095	Yes
18974	18:**0 X** vs. 40:**1 X**	-3.165	-3.309 to -3.021	Yes
18975	18:**0 X** vs. 40:**10 X**	-2.369	-2.513 to -2.225	Yes
18976	18:**0 X** vs. 40:**40 X**	-0.4400	-0.5840 to -0.2960	Yes
18977	18:**0 X** vs. 40:**50 X**	-0.2873	-0.4313 to -0.1434	Yes
18978	18:**0 X** vs. 41:**0 X**	-3.280	-3.424 to -3.136	Yes
18979	18:**0 X** vs. 41:**1 X**	-3.279	-3.423 to -3.135	Yes
18980	18:**0 X** vs. 41:**10 X**	-2.423	-2.567 to -2.279	Yes
18981	18:**0 X** vs. 41:**40 X**	-0.3873	-0.5313 to -0.2434	Yes
18982	18:**0 X** vs. 41:**50 X**	-0.3000	-0.4440 to -0.1560	Yes
18983	18:**0 X** vs. 42:**0 X**	-3.421	-3.565 to -3.277	Yes
18984	18:**0 X** vs. 42:**1 X**	-3.441	-3.585 to -3.297	Yes
18985	18:**0 X** vs. 42:**10 X**	-2.423	-2.567 to -2.279	Yes
18986	18:**0 X** vs. 42:**40 X**	-0.4000	-0.5440 to -0.2560	Yes
18987	18:**0 X** vs. 42:**50 X**	-0.3033	-0.4473 to -0.1594	Yes
18988	18:**0 X** vs. 43:**0 X**	-3.511	-3.655 to -3.367	Yes
18989	18:**0 X** vs. 43:**1 X**	-3.426	-3.570 to -3.282	Yes
18990	18:**0 X** vs. 43:**10 X**	-3.141	-3.285 to -2.997	Yes

2way ANOVA<br>Multiple comparisons		A<br>Data Set-A<br>Y	B<br>Data Set-B<br>Y	C<br>Data Set-C<br>Y
18991	18:0 X vs. 43:40 X	-0.3983	-0.5423 to -0.2544	Yes
18992	18:0 X vs. 43:50 X	-0.3037	-0.4476 to -0.1597	Yes
18993	18:0 X vs. 44:0 X	-3.535	-3.679 to -3.391	Yes
18994	18:0 X vs. 44:1 X	-3.425	-3.569 to -3.281	Yes
18995	18:0 X vs. 44:10 X	-3.205	-3.349 to -3.061	Yes
18996	18:0 X vs. 44:40 X	-0.4003	-0.5443 to -0.2564	Yes
18997	18:0 X vs. 44:50 X	-0.2360	-0.3800 to -0.09204	Yes
18998	18:0 X vs. 45:0 X	-3.400	-3.544 to -3.256	Yes
18999	18:0 X vs. 45:1 X	-3.202	-3.346 to -3.058	Yes
19000	18:0 X vs. 45:10 X	-3.084	-3.228 to -2.940	Yes
19001	18:0 X vs. 45:40 X	-0.4017	-0.5456 to -0.2577	Yes
19002	18:0 X vs. 45:50 X	-0.2330	-0.3770 to -0.08904	Yes
19003	18:0 X vs. 46:0 X	-3.204	-3.348 to -3.060	Yes
19004	18:0 X vs. 46:1 X	-3.171	-3.315 to -3.027	Yes
19005	18:0 X vs. 46:10 X	-3.079	-3.223 to -2.935	Yes
19006	18:0 X vs. 46:40 X	-0.4153	-0.5593 to -0.2714	Yes
19007	18:0 X vs. 46:50 X	-0.2260	-0.3700 to -0.08204	Yes
19008	18:0 X vs. 47:0 X	-3.180	-3.324 to -3.036	Yes
19009	18:0 X vs. 47:1 X	-3.084	-3.228 to -2.940	Yes
19010	18:0 X vs. 47:10 X	-3.065	-3.209 to -2.921	Yes
19011	18:0 X vs. 47:40 X	-0.4253	-0.5693 to -0.2814	Yes
19012	18:0 X vs. 47:50 X	-0.1840	-0.3280 to -0.04004	Yes
19013	18:0 X vs. 48:0 X	-3.167	-3.328 to -3.006	Yes
19014	18:0 X vs. 48:1 X	-3.071	-3.215 to -2.927	Yes
19015	18:0 X vs. 48:10 X	-3.065	-3.209 to -2.921	Yes
19016	18:0 X vs. 48:40 X	-0.4317	-0.5756 to -0.2877	Yes
19017	18:0 X vs. 48:50 X	-0.1913	-0.3353 to -0.04737	Yes
19018	18:0 X vs. 49:0 X	-3.167	-3.311 to -3.023	Yes
19019	18:0 X vs. 49:1 X	-3.071	-3.215 to -2.927	Yes
19020	18:0 X vs. 49:10 X	-3.065	-3.209 to -2.921	Yes
19021	18:0 X vs. 49:40 X	-0.4360	-0.5800 to -0.2920	Yes
19022	18:0 X vs. 49:50 X	-0.1907	-0.3346 to -0.04671	Yes
19023	18:0 X vs. 50:0 X	-3.167	-3.311 to -3.023	Yes
19024	18:0 X vs. 50:1 X	-3.071	-3.215 to -2.927	Yes
19025	18:0 X vs. 50:10 X	-3.065	-3.209 to -2.921	Yes
19026	18:0 X vs. 50:40 X	-0.4437	-0.5876 to -0.2997	Yes
19027	18:0 X vs. 50:50 X	-0.1903	-0.3343 to -0.04637	Yes
19028	18:1 X vs. 18:10 X	-0.09233	-0.2363 to 0.05163	No
19029	18:1 X vs. 18:40 X	0.05833	-0.08563 to 0.2023	No
19030	18:1 X vs. 18:50 X	0.01333	-0.1306 to 0.1573	No
19031	18:1 X vs. 19:0 X	-0.1267	-0.2706 to 0.01729	No
19032	18:1 X vs. 19:1 X	-0.1147	-0.2586 to 0.02929	No
19033	18:1 X vs. 19:10 X	-0.1123	-0.2563 to 0.03163	No
19034	18:1 X vs. 19:40 X	0.02967	-0.1143 to 0.1736	No
19035	18:1 X vs. 19:50 X	0.01867	-0.1253 to 0.1626	No

	2way ANOVA<br>Multiple comparisons	A<br>Data Set-A<br>Y	B<br>Data Set-B<br>Y	C<br>Data Set-C<br>Y
19036	18:**1 X** vs. 20:**0 X**	-0.2063	-0.3503 to -0.06237	Yes
19037	18:**1 X** vs. 20:**1 X**	-0.02567	-0.1696 to 0.1183	No
19038	18:**1 X** vs. 20:**10 X**	-0.1147	-0.2586 to 0.02929	No
19039	18:**1 X** vs. 20:**40 X**	0.0180	-0.1260 to 0.1620	No
19040	18:**1 X** vs. 20:**50 X**	-0.0260	-0.1700 to 0.1180	No
19041	18:**1 X** vs. 21:**0 X**	-0.2297	-0.3736 to -0.08571	Yes
19042	18:**1 X** vs. 21:**1 X**	-0.1827	-0.3266 to -0.03871	Yes
19043	18:**1 X** vs. 21:**10 X**	-0.1797	-0.3236 to -0.03571	Yes
19044	18:**1 X** vs. 21:**40 X**	-0.0550	-0.1990 to 0.08896	No
19045	18:**1 X** vs. 21:**50 X**	-0.1453	-0.2893 to -0.001374	Yes
19046	18:**1 X** vs. 22:**0 X**	-0.2713	-0.4153 to -0.1274	Yes
19047	18:**1 X** vs. 22:**1 X**	-0.2660	-0.4100 to -0.1220	Yes
19048	18:**1 X** vs. 22:**10 X**	-0.2613	-0.4053 to -0.1174	Yes
19049	18:**1 X** vs. 22:**40 X**	-0.1617	-0.3056 to -0.01771	Yes
19050	18:**1 X** vs. 22:**50 X**	-0.2243	-0.3683 to -0.08037	Yes
19051	18:**1 X** vs. 23:**0 X**	-0.3367	-0.4806 to -0.1927	Yes
19052	18:**1 X** vs. 23:**1 X**	-0.5263	-0.6873 to -0.3654	Yes
19053	18:**1 X** vs. 23:**10 X**	-0.2760	-0.4200 to -0.1320	Yes
19054	18:**1 X** vs. 23:**40 X**	-0.1563	-0.3173 to 0.004619	No
19055	18:**1 X** vs. 23:**50 X**	-0.1903	-0.3343 to -0.04637	Yes
19056	18:**1 X** vs. 24:**0 X**	-0.4540	-0.5980 to -0.3100	Yes
19057	18:**1 X** vs. 24:**1 X**	-0.6103	-0.7713 to -0.4494	Yes
19058	18:**1 X** vs. 24:**10 X**	-0.4378	-0.5988 to -0.2769	Yes
19059	18:**1 X** vs. 24:**40 X**	-0.1727	-0.3166 to -0.02871	Yes
19060	18:**1 X** vs. 24:**50 X**	-0.09433	-0.2383 to 0.04963	No
19061	18:**1 X** vs. 25:**0 X**	-0.7913	-0.9523 to -0.6304	Yes
19062	18:**1 X** vs. 25:**1 X**	-0.9808	-1.142 to -0.8199	Yes
19063	18:**1 X** vs. 25:**10 X**	-0.3423	-0.5459 to -0.1387	Yes
19064	18:**1 X** vs. 25:**40 X**	-0.1747	-0.3186 to -0.03071	Yes
19065	18:**1 X** vs. 25:**50 X**	-0.1490	-0.2930 to -0.005040	Yes
19066	18:**1 X** vs. 26:**0 X**	-1.004	-1.165 to -0.8429	Yes
19067	18:**1 X** vs. 26:**1 X**	-1.117	-1.278 to -0.9559	Yes
19068	18:**1 X** vs. 26:**10 X**	-0.8033	-0.9643 to -0.6424	Yes
19069	18:**1 X** vs. 26:**40 X**	-0.2490	-0.3930 to -0.1050	Yes
19070	18:**1 X** vs. 26:**50 X**	-0.1093	-0.2533 to 0.03463	No
19071	18:**1 X** vs. 27:**0 X**	-1.192	-1.336 to -1.048	Yes
19072	18:**1 X** vs. 27:**1 X**	-1.282	-1.443 to -1.121	Yes
19073	18:**1 X** vs. 27:**10 X**	-1.094	-1.255 to -0.9334	Yes
19074	18:**1 X** vs. 27:**40 X**	-0.2490	-0.3930 to -0.1050	Yes
19075	18:**1 X** vs. 27:**50 X**	-0.1087	-0.2526 to 0.03529	No
19076	18:**1 X** vs. 28:**0 X**	-1.141	-1.302 to -0.9799	Yes
19077	18:**1 X** vs. 28:**1 X**	-1.182	-1.326 to -1.038	Yes
19078	18:**1 X** vs. 28:**10 X**	-0.9833	-1.144 to -0.8224	Yes
19079	18:**1 X** vs. 28:**40 X**	-0.2613	-0.4053 to -0.1174	Yes
19080	18:**1 X** vs. 28:**50 X**	-0.1567	-0.3006 to -0.01271	Yes

	2way ANOVA<br>Multiple comparisons	A<br>Data Set-A<br>Y	B<br>Data Set-B<br>Y	C<br>Data Set-C<br>Y
19081	18:**1 X** vs. 29:**0 X**	-1.622	-1.783 to -1.461	Yes
19082	18:**1 X** vs. 29:**1 X**	-1.707	-1.868 to -1.546	Yes
19083	18:**1 X** vs. 29:**10 X**	-0.9218	-1.083 to -0.7609	Yes
19084	18:**1 X** vs. 29:**40 X**	-0.3693	-0.5133 to -0.2254	Yes
19085	18:**1 X** vs. 29:**50 X**	-0.1580	-0.3020 to -0.01404	Yes
19086	18:**1 X** vs. 30:**0 X**	-1.337	-1.498 to -1.176	Yes
19087	18:**1 X** vs. 30:**1 X**	-1.658	-1.819 to -1.497	Yes
19088	18:**1 X** vs. 30:**10 X**	-0.9963	-1.157 to -0.8354	Yes
19089	18:**1 X** vs. 30:**40 X**	-0.2963	-0.4403 to -0.1524	Yes
19090	18:**1 X** vs. 30:**50 X**	-0.2103	-0.3543 to -0.06637	Yes
19091	18:**1 X** vs. 31:**0 X**	-1.655	-1.816 to -1.494	Yes
19092	18:**1 X** vs. 31:**1 X**	-1.645	-1.806 to -1.484	Yes
19093	18:**1 X** vs. 31:**10 X**	-1.128	-1.289 to -0.9669	Yes
19094	18:**1 X** vs. 31:**40 X**	-0.3880	-0.5320 to -0.2440	Yes
19095	18:**1 X** vs. 31:**50 X**	-0.1893	-0.3333 to -0.04537	Yes
19096	18:**1 X** vs. 32:**0 X**	-2.246	-2.390 to -2.102	Yes
19097	18:**1 X** vs. 32:**1 X**	-1.702	-1.863 to -1.541	Yes
19098	18:**1 X** vs. 32:**10 X**	-1.558	-1.702 to -1.414	Yes
19099	18:**1 X** vs. 32:**40 X**	-0.3843	-0.5283 to -0.2404	Yes
19100	18:**1 X** vs. 32:**50 X**	-0.1890	-0.3330 to -0.04504	Yes
19101	18:**1 X** vs. 33:**0 X**	-2.250	-2.394 to -2.106	Yes
19102	18:**1 X** vs. 33:**1 X**	-2.065	-2.209 to -1.921	Yes
19103	18:**1 X** vs. 33:**10 X**	-1.725	-1.869 to -1.581	Yes
19104	18:**1 X** vs. 33:**40 X**	-0.4030	-0.5470 to -0.2590	Yes
19105	18:**1 X** vs. 33:**50 X**	-0.2040	-0.3480 to -0.06004	Yes
19106	18:**1 X** vs. 34:**0 X**	-2.175	-2.319 to -2.031	Yes
19107	18:**1 X** vs. 34:**1 X**	-2.182	-2.326 to -2.038	Yes
19108	18:**1 X** vs. 34:**10 X**	-1.815	-1.959 to -1.671	Yes
19109	18:**1 X** vs. 34:**40 X**	-0.4150	-0.5590 to -0.2710	Yes
19110	18:**1 X** vs. 34:**50 X**	-0.3530	-0.4970 to -0.2090	Yes
19111	18:**1 X** vs. 35:**0 X**	-2.523	-2.667 to -2.379	Yes
19112	18:**1 X** vs. 35:**1 X**	-2.289	-2.433 to -2.145	Yes
19113	18:**1 X** vs. 35:**10 X**	-1.777	-1.921 to -1.633	Yes
19114	18:**1 X** vs. 35:**40 X**	-0.4240	-0.5680 to -0.2800	Yes
19115	18:**1 X** vs. 35:**50 X**	-0.3657	-0.5096 to -0.2217	Yes
19116	18:**1 X** vs. 36:**0 X**	-2.730	-2.891 to -2.569	Yes
19117	18:**1 X** vs. 36:**1 X**	-2.426	-2.570 to -2.282	Yes
19118	18:**1 X** vs. 36:**10 X**	-1.783	-1.927 to -1.639	Yes
19119	18:**1 X** vs. 36:**40 X**	-0.4793	-0.6233 to -0.3354	Yes
19120	18:**1 X** vs. 36:**50 X**	-0.3290	-0.4730 to -0.1850	Yes
19121	18:**1 X** vs. 37:**0 X**	-3.114	-3.258 to -2.970	Yes
19122	18:**1 X** vs. 37:**1 X**	-2.496	-2.640 to -2.352	Yes
19123	18:**1 X** vs. 37:**10 X**	-1.784	-1.928 to -1.640	Yes
19124	18:**1 X** vs. 37:**40 X**	-0.5167	-0.6606 to -0.3727	Yes
19125	18:**1 X** vs. 37:**50 X**	-0.3377	-0.4816 to -0.1937	Yes

2way ANOVA<br>Multiple comparisons	A<br>Data Set-A<br>Y	B<br>Data Set-B<br>Y	C<br>Data Set-C<br>Y
19126 18:1 X vs. 38:0 X	-3.059	-3.203 to -2.915	Yes
19127 18:1 X vs. 38:1 X	-2.614	-2.758 to -2.470	Yes
19128 18:1 X vs. 38:10 X	-2.060	-2.204 to -1.916	Yes
19129 18:1 X vs. 38:40 X	-0.5210	-0.6650 to -0.3770	Yes
19130 18:1 X vs. 38:50 X	-0.3417	-0.4856 to -0.1977	Yes
19131 18:1 X vs. 39:0 X	-3.289	-3.433 to -3.145	Yes
19132 18:1 X vs. 39:1 X	-3.181	-3.325 to -3.037	Yes
19133 18:1 X vs. 39:10 X	-2.205	-2.349 to -2.061	Yes
19134 18:1 X vs. 39:40 X	-0.5270	-0.6710 to -0.3830	Yes
19135 18:1 X vs. 39:50 X	-0.3727	-0.5166 to -0.2287	Yes
19136 18:1 X vs. 40:0 X	-3.330	-3.474 to -3.186	Yes
19137 18:1 X vs. 40:1 X	-3.256	-3.400 to -3.112	Yes
19138 18:1 X vs. 40:10 X	-2.461	-2.605 to -2.317	Yes
19139 18:1 X vs. 40:40 X	-0.5317	-0.6756 to -0.3877	Yes
19140 18:1 X vs. 40:50 X	-0.3790	-0.5230 to -0.2350	Yes
19141 18:1 X vs. 41:0 X	-3.371	-3.515 to -3.227	Yes
19142 18:1 X vs. 41:1 X	-3.370	-3.514 to -3.226	Yes
19143 18:1 X vs. 41:10 X	-2.515	-2.659 to -2.371	Yes
19144 18:1 X vs. 41:40 X	-0.4790	-0.6230 to -0.3350	Yes
19145 18:1 X vs. 41:50 X	-0.3917	-0.5356 to -0.2477	Yes
19146 18:1 X vs. 42:0 X	-3.513	-3.657 to -3.369	Yes
19147 18:1 X vs. 42:1 X	-3.533	-3.677 to -3.389	Yes
19148 18:1 X vs. 42:10 X	-2.515	-2.659 to -2.371	Yes
19149 18:1 X vs. 42:40 X	-0.4917	-0.6356 to -0.3477	Yes
19150 18:1 X vs. 42:50 X	-0.3950	-0.5390 to -0.2510	Yes
19151 18:1 X vs. 43:0 X	-3.603	-3.747 to -3.459	Yes
19152 18:1 X vs. 43:1 X	-3.517	-3.661 to -3.373	Yes
19153 18:1 X vs. 43:10 X	-3.233	-3.377 to -3.089	Yes
19154 18:1 X vs. 43:40 X	-0.4900	-0.6340 to -0.3460	Yes
19155 18:1 X vs. 43:50 X	-0.3953	-0.5393 to -0.2514	Yes
19156 18:1 X vs. 44:0 X	-3.627	-3.771 to -3.483	Yes
19157 18:1 X vs. 44:1 X	-3.517	-3.661 to -3.373	Yes
19158 18:1 X vs. 44:10 X	-3.296	-3.440 to -3.152	Yes
19159 18:1 X vs. 44:40 X	-0.4920	-0.6360 to -0.3480	Yes
19160 18:1 X vs. 44:50 X	-0.3277	-0.4716 to -0.1837	Yes
19161 18:1 X vs. 45:0 X	-3.492	-3.636 to -3.348	Yes
19162 18:1 X vs. 45:1 X	-3.294	-3.438 to -3.150	Yes
19163 18:1 X vs. 45:10 X	-3.176	-3.320 to -3.032	Yes
19164 18:1 X vs. 45:40 X	-0.4933	-0.6373 to -0.3494	Yes
19165 18:1 X vs. 45:50 X	-0.3247	-0.4686 to -0.1807	Yes
19166 18:1 X vs. 46:0 X	-3.296	-3.440 to -3.152	Yes
19167 18:1 X vs. 46:1 X	-3.263	-3.407 to -3.119	Yes
19168 18:1 X vs. 46:10 X	-3.171	-3.315 to -3.027	Yes
19169 18:1 X vs. 46:40 X	-0.5070	-0.6510 to -0.3630	Yes
19170 18:1 X vs. 46:50 X	-0.3177	-0.4616 to -0.1737	Yes

2way ANOVA<br>Multiple comparisons	A<br>Data Set-A<br>Y	B<br>Data Set-B<br>Y	C<br>Data Set-C<br>Y
19171 18:**1 X** vs. 47:**0 X**	-3.272	-3.416 to -3.128	Yes
19172 18:**1 X** vs. 47:**1 X**	-3.176	-3.320 to -3.032	Yes
19173 18:**1 X** vs. 47:**10 X**	-3.157	-3.301 to -3.013	Yes
19174 18:**1 X** vs. 47:**40 X**	-0.5170	-0.6610 to -0.3730	Yes
19175 18:**1 X** vs. 47:**50 X**	-0.2757	-0.4196 to -0.1317	Yes
19176 18:**1 X** vs. 48:**0 X**	-3.259	-3.420 to -3.098	Yes
19177 18:**1 X** vs. 48:**1 X**	-3.163	-3.307 to -3.019	Yes
19178 18:**1 X** vs. 48:**10 X**	-3.157	-3.301 to -3.013	Yes
19179 18:**1 X** vs. 48:**40 X**	-0.5233	-0.6673 to -0.3794	Yes
19180 18:**1 X** vs. 48:**50 X**	-0.2830	-0.4270 to -0.1390	Yes
19181 18:**1 X** vs. 49:**0 X**	-3.259	-3.403 to -3.115	Yes
19182 18:**1 X** vs. 49:**1 X**	-3.163	-3.307 to -3.019	Yes
19183 18:**1 X** vs. 49:**10 X**	-3.157	-3.301 to -3.013	Yes
19184 18:**1 X** vs. 49:**40 X**	-0.5277	-0.6716 to -0.3837	Yes
19185 18:**1 X** vs. 49:**50 X**	-0.2823	-0.4263 to -0.1384	Yes
19186 18:**1 X** vs. 50:**0 X**	-3.259	-3.403 to -3.115	Yes
19187 18:**1 X** vs. 50:**1 X**	-3.163	-3.307 to -3.019	Yes
19188 18:**1 X** vs. 50:**10 X**	-3.157	-3.301 to -3.013	Yes
19189 18:**1 X** vs. 50:**40 X**	-0.5353	-0.6793 to -0.3914	Yes
19190 18:**1 X** vs. 50:**50 X**	-0.2820	-0.4260 to -0.1380	Yes
19191 18:**10 X** vs. 18:**40 X**	0.1507	0.006707 to 0.2946	Yes
19192 18:**10 X** vs. 18:**50 X**	0.1057	-0.03829 to 0.2496	No
19193 18:**10 X** vs. 19:**0 X**	-0.03433	-0.1783 to 0.1096	No
19194 18:**10 X** vs. 19:**1 X**	-0.02233	-0.1663 to 0.1216	No
19195 18:**10 X** vs. 19:**10 X**	-0.02000	-0.1640 to 0.1240	No
19196 18:**10 X** vs. 19:**40 X**	0.1220	-0.02196 to 0.2660	No
19197 18:**10 X** vs. 19:**50 X**	0.1110	-0.03296 to 0.2550	No
19198 18:**10 X** vs. 20:**0 X**	-0.1140	-0.2580 to 0.02996	No
19199 18:**10 X** vs. 20:**1 X**	0.06667	-0.07729 to 0.2106	No
19200 18:**10 X** vs. 20:**10 X**	-0.02233	-0.1663 to 0.1216	No
19201 18:**10 X** vs. 20:**40 X**	0.1103	-0.03363 to 0.2543	No
19202 18:**10 X** vs. 20:**50 X**	0.06633	-0.07763 to 0.2103	No
19203 18:**10 X** vs. 21:**0 X**	-0.1373	-0.2813 to 0.006626	No
19204 18:**10 X** vs. 21:**1 X**	-0.09033	-0.2343 to 0.05363	No
19205 18:**10 X** vs. 21:**10 X**	-0.08733	-0.2313 to 0.05663	No
19206 18:**10 X** vs. 21:**40 X**	0.03733	-0.1066 to 0.1813	No
19207 18:**10 X** vs. 21:**50 X**	-0.05300	-0.1970 to 0.09096	No
19208 18:**10 X** vs. 22:**0 X**	-0.1790	-0.3230 to -0.03504	Yes
19209 18:**10 X** vs. 22:**1 X**	-0.1737	-0.3176 to -0.02971	Yes
19210 18:**10 X** vs. 22:**10 X**	-0.1690	-0.3130 to -0.02504	Yes
19211 18:**10 X** vs. 22:**40 X**	-0.06933	-0.2133 to 0.07463	No
19212 18:**10 X** vs. 22:**50 X**	-0.1320	-0.2760 to 0.01196	No
19213 18:**10 X** vs. 23:**0 X**	-0.2443	-0.3883 to -0.1004	Yes
19214 18:**10 X** vs. 23:**1 X**	-0.4340	-0.5950 to -0.2730	Yes
19215 18:**10 X** vs. 23:**10 X**	-0.1837	-0.3276 to -0.03971	Yes

2way ANOVA Multiple comparisons		A Data Set-A Y	B Data Set-B Y	C Data Set-C Y
19216	18:**10 X** vs. 23:**40 X**	-0.06400	-0.2250 to 0.09695	No
19217	18:**10 X** vs. 23:**50 X**	-0.09800	-0.2420 to 0.04596	No
19218	18:**10 X** vs. 24:**0 X**	-0.3617	-0.5056 to -0.2177	Yes
19219	18:**10 X** vs. 24:**1 X**	-0.5180	-0.6790 to -0.3570	Yes
19220	18:**10 X** vs. 24:**10 X**	-0.3455	-0.5065 to -0.1845	Yes
19221	18:**10 X** vs. 24:**40 X**	-0.08033	-0.2243 to 0.06363	No
19222	18:**10 X** vs. 24:**50 X**	-0.002000	-0.1460 to 0.1420	No
19223	18:**10 X** vs. 25:**0 X**	-0.6990	-0.8600 to -0.5380	Yes
19224	18:**10 X** vs. 25:**1 X**	-0.8885	-1.049 to -0.7275	Yes
19225	18:**10 X** vs. 25:**10 X**	-0.2500	-0.4536 to -0.04641	Yes
19226	18:**10 X** vs. 25:**40 X**	-0.08233	-0.2263 to 0.06163	No
19227	18:**10 X** vs. 25:**50 X**	-0.05667	-0.2006 to 0.08729	No
19228	18:**10 X** vs. 26:**0 X**	-0.9115	-1.072 to -0.7505	Yes
19229	18:**10 X** vs. 26:**1 X**	-1.025	-1.185 to -0.8635	Yes
19230	18:**10 X** vs. 26:**10 X**	-0.7110	-0.8720 to -0.5500	Yes
19231	18:**10 X** vs. 26:**40 X**	-0.1567	-0.3006 to -0.01271	Yes
19232	18:**10 X** vs. 26:**50 X**	-0.0170	-0.1610 to 0.1270	No
19233	18:**10 X** vs. 27:**0 X**	-1.099	-1.243 to -0.9554	Yes
19234	18:**10 X** vs. 27:**1 X**	-1.190	-1.351 to -1.029	Yes
19235	18:**10 X** vs. 27:**10 X**	-1.002	-1.163 to -0.8410	Yes
19236	18:**10 X** vs. 27:**40 X**	-0.1567	-0.3006 to -0.01271	Yes
19237	18:**10 X** vs. 27:**50 X**	-0.01633	-0.1603 to 0.1276	No
19238	18:**10 X** vs. 28:**0 X**	-1.049	-1.209 to -0.8875	Yes
19239	18:**10 X** vs. 28:**1 X**	-1.089	-1.233 to -0.9454	Yes
19240	18:**10 X** vs. 28:**10 X**	-0.8910	-1.052 to -0.7300	Yes
19241	18:**10 X** vs. 28:**40 X**	-0.1690	-0.3130 to -0.02504	Yes
19242	18:**10 X** vs. 28:**50 X**	-0.06433	-0.2083 to 0.07963	No
19243	18:**10 X** vs. 29:**0 X**	-1.530	-1.691 to -1.369	Yes
19244	18:**10 X** vs. 29:**1 X**	-1.615	-1.776 to -1.454	Yes
19245	18:**10 X** vs. 29:**10 X**	-0.8295	-0.9905 to -0.6685	Yes
19246	18:**10 X** vs. 29:**40 X**	-0.2770	-0.4210 to -0.1330	Yes
19247	18:**10 X** vs. 29:**50 X**	-0.06567	-0.2096 to 0.07829	No
19248	18:**10 X** vs. 30:**0 X**	-1.245	-1.405 to -1.084	Yes
19249	18:**10 X** vs. 30:**1 X**	-1.566	-1.727 to -1.405	Yes
19250	18:**10 X** vs. 30:**10 X**	-0.9040	-1.065 to -0.7430	Yes
19251	18:**10 X** vs. 30:**40 X**	-0.2040	-0.3480 to -0.06004	Yes
19252	18:**10 X** vs. 30:**50 X**	-0.1180	-0.2620 to 0.02596	No
19253	18:**10 X** vs. 31:**0 X**	-1.563	-1.723 to -1.402	Yes
19254	18:**10 X** vs. 31:**1 X**	-1.553	-1.714 to -1.392	Yes
19255	18:**10 X** vs. 31:**10 X**	-1.036	-1.196 to -0.8745	Yes
19256	18:**10 X** vs. 31:**40 X**	-0.2957	-0.4396 to -0.1517	Yes
19257	18:**10 X** vs. 31:**50 X**	-0.0970	-0.2410 to 0.04696	No
19258	18:**10 X** vs. 32:**0 X**	-2.154	-2.298 to -2.010	Yes
19259	18:**10 X** vs. 32:**1 X**	-1.610	-1.771 to -1.449	Yes
19260	18:**10 X** vs. 32:**10 X**	-1.466	-1.610 to -1.322	Yes

	2way ANOVA<br>Multiple comparisons	A<br>Data Set-A<br>Y	B<br>Data Set-B<br>Y	C<br>Data Set-C<br>Y
19261	18:10 X vs. 32:40 X	-0.2920	-0.4360 to -0.1480	Yes
19262	18:10 X vs. 32:50 X	-0.09667	-0.2406 to 0.04729	No
19263	18:10 X vs. 33:0 X	-2.158	-2.302 to -2.014	Yes
19264	18:10 X vs. 33:1 X	-1.972	-2.116 to -1.828	Yes
19265	18:10 X vs. 33:10 X	-1.633	-1.777 to -1.489	Yes
19266	18:10 X vs. 33:40 X	-0.3107	-0.4546 to -0.1667	Yes
19267	18:10 X vs. 33:50 X	-0.1117	-0.2556 to 0.03229	No
19268	18:10 X vs. 34:0 X	-2.082	-2.226 to -1.938	Yes
19269	18:10 X vs. 34:1 X	-2.090	-2.234 to -1.946	Yes
19270	18:10 X vs. 34:10 X	-1.723	-1.867 to -1.579	Yes
19271	18:10 X vs. 34:40 X	-0.3227	-0.4666 to -0.1787	Yes
19272	18:10 X vs. 34:50 X	-0.2607	-0.4046 to -0.1167	Yes
19273	18:10 X vs. 35:0 X	-2.431	-2.575 to -2.287	Yes
19274	18:10 X vs. 35:1 X	-2.197	-2.341 to -2.053	Yes
19275	18:10 X vs. 35:10 X	-1.685	-1.829 to -1.541	Yes
19276	18:10 X vs. 35:40 X	-0.3317	-0.4756 to -0.1877	Yes
19277	18:10 X vs. 35:50 X	-0.2733	-0.4173 to -0.1294	Yes
19278	18:10 X vs. 36:0 X	-2.638	-2.798 to -2.477	Yes
19279	18:10 X vs. 36:1 X	-2.333	-2.477 to -2.189	Yes
19280	18:10 X vs. 36:10 X	-1.690	-1.834 to -1.546	Yes
19281	18:10 X vs. 36:40 X	-0.3870	-0.5310 to -0.2430	Yes
19282	18:10 X vs. 36:50 X	-0.2367	-0.3806 to -0.09271	Yes
19283	18:10 X vs. 37:0 X	-3.021	-3.165 to -2.877	Yes
19284	18:10 X vs. 37:1 X	-2.403	-2.547 to -2.259	Yes
19285	18:10 X vs. 37:10 X	-1.692	-1.836 to -1.548	Yes
19286	18:10 X vs. 37:40 X	-0.4243	-0.5683 to -0.2804	Yes
19287	18:10 X vs. 37:50 X	-0.2453	-0.3893 to -0.1014	Yes
19288	18:10 X vs. 38:0 X	-2.966	-3.110 to -2.822	Yes
19289	18:10 X vs. 38:1 X	-2.522	-2.666 to -2.378	Yes
19290	18:10 X vs. 38:10 X	-1.967	-2.111 to -1.823	Yes
19291	18:10 X vs. 38:40 X	-0.4287	-0.5726 to -0.2847	Yes
19292	18:10 X vs. 38:50 X	-0.2493	-0.3933 to -0.1054	Yes
19293	18:10 X vs. 39:0 X	-3.197	-3.341 to -3.053	Yes
19294	18:10 X vs. 39:1 X	-3.089	-3.233 to -2.945	Yes
19295	18:10 X vs. 39:10 X	-2.112	-2.256 to -1.968	Yes
19296	18:10 X vs. 39:40 X	-0.4347	-0.5786 to -0.2907	Yes
19297	18:10 X vs. 39:50 X	-0.2803	-0.4243 to -0.1364	Yes
19298	18:10 X vs. 40:0 X	-3.238	-3.382 to -3.094	Yes
19299	18:10 X vs. 40:1 X	-3.164	-3.308 to -3.020	Yes
19300	18:10 X vs. 40:10 X	-2.369	-2.513 to -2.225	Yes
19301	18:10 X vs. 40:40 X	-0.4393	-0.5833 to -0.2954	Yes
19302	18:10 X vs. 40:50 X	-0.2867	-0.4306 to -0.1427	Yes
19303	18:10 X vs. 41:0 X	-3.279	-3.423 to -3.135	Yes
19304	18:10 X vs. 41:1 X	-3.278	-3.422 to -3.134	Yes
19305	18:10 X vs. 41:10 X	-2.422	-2.566 to -2.278	Yes

2way ANOVA<br>Multiple comparisons		A<br>Data Set-A<br>Y	B<br>Data Set-B<br>Y	C<br>Data Set-C<br>Y
19306	18:**10 X** vs. 41:**40 X**	-0.3867	-0.5306 to -0.2427	Yes
19307	18:**10 X** vs. 41:**50 X**	-0.2993	-0.4433 to -0.1554	Yes
19308	18:**10 X** vs. 42:**0 X**	-3.421	-3.565 to -3.277	Yes
19309	18:**10 X** vs. 42:**1 X**	-3.440	-3.584 to -3.296	Yes
19310	18:**10 X** vs. 42:**10 X**	-2.423	-2.567 to -2.279	Yes
19311	18:**10 X** vs. 42:**40 X**	-0.3993	-0.5433 to -0.2554	Yes
19312	18:**10 X** vs. 42:**50 X**	-0.3027	-0.4466 to -0.1587	Yes
19313	18:**10 X** vs. 43:**0 X**	-3.511	-3.655 to -3.367	Yes
19314	18:**10 X** vs. 43:**1 X**	-3.425	-3.569 to -3.281	Yes
19315	18:**10 X** vs. 43:**10 X**	-3.141	-3.285 to -2.997	Yes
19316	18:**10 X** vs. 43:**40 X**	-0.3977	-0.5416 to -0.2537	Yes
19317	18:**10 X** vs. 43:**50 X**	-0.3030	-0.4470 to -0.1590	Yes
19318	18:**10 X** vs. 44:**0 X**	-3.535	-3.679 to -3.391	Yes
19319	18:**10 X** vs. 44:**1 X**	-3.424	-3.568 to -3.280	Yes
19320	18:**10 X** vs. 44:**10 X**	-3.204	-3.348 to -3.060	Yes
19321	18:**10 X** vs. 44:**40 X**	-0.3997	-0.5436 to -0.2557	Yes
19322	18:**10 X** vs. 44:**50 X**	-0.2353	-0.3793 to -0.09137	Yes
19323	18:**10 X** vs. 45:**0 X**	-3.399	-3.543 to -3.255	Yes
19324	18:**10 X** vs. 45:**1 X**	-3.201	-3.345 to -3.057	Yes
19325	18:**10 X** vs. 45:**10 X**	-3.084	-3.228 to -2.940	Yes
19326	18:**10 X** vs. 45:**40 X**	-0.4010	-0.5450 to -0.2570	Yes
19327	18:**10 X** vs. 45:**50 X**	-0.2323	-0.3763 to -0.08837	Yes
19328	18:**10 X** vs. 46:**0 X**	-3.204	-3.348 to -3.060	Yes
19329	18:**10 X** vs. 46:**1 X**	-3.171	-3.315 to -3.027	Yes
19330	18:**10 X** vs. 46:**10 X**	-3.079	-3.223 to -2.935	Yes
19331	18:**10 X** vs. 46:**40 X**	-0.4147	-0.5586 to -0.2707	Yes
19332	18:**10 X** vs. 46:**50 X**	-0.2253	-0.3693 to -0.08137	Yes
19333	18:**10 X** vs. 47:**0 X**	-3.179	-3.323 to -3.035	Yes
19334	18:**10 X** vs. 47:**1 X**	-3.083	-3.227 to -2.939	Yes
19335	18:**10 X** vs. 47:**10 X**	-3.065	-3.209 to -2.921	Yes
19336	18:**10 X** vs. 47:**40 X**	-0.4247	-0.5686 to -0.2807	Yes
19337	18:**10 X** vs. 47:**50 X**	-0.1833	-0.3273 to -0.03937	Yes
19338	18:**10 X** vs. 48:**0 X**	-3.167	-3.327 to -3.006	Yes
19339	18:**10 X** vs. 48:**1 X**	-3.070	-3.214 to -2.926	Yes
19340	18:**10 X** vs. 48:**10 X**	-3.065	-3.209 to -2.921	Yes
19341	18:**10 X** vs. 48:**40 X**	-0.4310	-0.5750 to -0.2870	Yes
19342	18:**10 X** vs. 48:**50 X**	-0.1907	-0.3346 to -0.04671	Yes
19343	18:**10 X** vs. 49:**0 X**	-3.167	-3.311 to -3.023	Yes
19344	18:**10 X** vs. 49:**1 X**	-3.070	-3.214 to -2.926	Yes
19345	18:**10 X** vs. 49:**10 X**	-3.065	-3.209 to -2.921	Yes
19346	18:**10 X** vs. 49:**40 X**	-0.4353	-0.5793 to -0.2914	Yes
19347	18:**10 X** vs. 49:**50 X**	-0.1900	-0.3340 to -0.04604	Yes
19348	18:**10 X** vs. 50:**0 X**	-3.167	-3.311 to -3.023	Yes
19349	18:**10 X** vs. 50:**1 X**	-3.070	-3.214 to -2.926	Yes
19350	18:**10 X** vs. 50:**10 X**	-3.065	-3.209 to -2.921	Yes

	2way ANOVA Multiple comparisons	A Data Set-A Y	B Data Set-B Y	C Data Set-C Y
19351	18:**10 X** vs. 50:**40 X**	-0.4430	-0.5870 to -0.2990	Yes
19352	18:**10 X** vs. 50:**50 X**	-0.1897	-0.3336 to -0.04571	Yes
19353	18:**40 X** vs. 18:**50 X**	-0.0450	-0.1890 to 0.09896	No
19354	18:**40 X** vs. 19:**0 X**	-0.1850	-0.3290 to -0.04104	Yes
19355	18:**40 X** vs. 19:**1 X**	-0.1730	-0.3170 to -0.02904	Yes
19356	18:**40 X** vs. 19:**10 X**	-0.1707	-0.3146 to -0.02671	Yes
19357	18:**40 X** vs. 19:**40 X**	-0.02867	-0.1726 to 0.1153	No
19358	18:**40 X** vs. 19:**50 X**	-0.03967	-0.1836 to 0.1043	No
19359	18:**40 X** vs. 20:**0 X**	-0.2647	-0.4086 to -0.1207	Yes
19360	18:**40 X** vs. 20:**1 X**	-0.0840	-0.2280 to 0.05996	No
19361	18:**40 X** vs. 20:**10 X**	-0.1730	-0.3170 to -0.02904	Yes
19362	18:**40 X** vs. 20:**40 X**	-0.04033	-0.1843 to 0.1036	No
19363	18:**40 X** vs. 20:**50 X**	-0.08433	-0.2283 to 0.05963	No
19364	18:**40 X** vs. 21:**0 X**	-0.2880	-0.4320 to -0.1440	Yes
19365	18:**40 X** vs. 21:**1 X**	-0.2410	-0.3850 to -0.09704	Yes
19366	18:**40 X** vs. 21:**10 X**	-0.2380	-0.3820 to -0.09404	Yes
19367	18:**40 X** vs. 21:**40 X**	-0.1133	-0.2573 to 0.03063	No
19368	18:**40 X** vs. 21:**50 X**	-0.2037	-0.3476 to -0.05971	Yes
19369	18:**40 X** vs. 22:**0 X**	-0.3297	-0.4736 to -0.1857	Yes
19370	18:**40 X** vs. 22:**1 X**	-0.3243	-0.4683 to -0.1804	Yes
19371	18:**40 X** vs. 22:**10 X**	-0.3197	-0.4636 to -0.1757	Yes
19372	18:**40 X** vs. 22:**40 X**	-0.2200	-0.3640 to -0.07604	Yes
19373	18:**40 X** vs. 22:**50 X**	-0.2827	-0.4266 to -0.1387	Yes
19374	18:**40 X** vs. 23:**0 X**	-0.3950	-0.5390 to -0.2510	Yes
19375	18:**40 X** vs. 23:**1 X**	-0.5847	-0.7456 to -0.4237	Yes
19376	18:**40 X** vs. 23:**10 X**	-0.3343	-0.4783 to -0.1904	Yes
19377	18:**40 X** vs. 23:**40 X**	-0.2147	-0.3756 to -0.05371	Yes
19378	18:**40 X** vs. 23:**50 X**	-0.2487	-0.3926 to -0.1047	Yes
19379	18:**40 X** vs. 24:**0 X**	-0.5123	-0.6563 to -0.3684	Yes
19380	18:**40 X** vs. 24:**1 X**	-0.6687	-0.8296 to -0.5077	Yes
19381	18:**40 X** vs. 24:**10 X**	-0.4962	-0.6571 to -0.3352	Yes
19382	18:**40 X** vs. 24:**40 X**	-0.2310	-0.3750 to -0.08704	Yes
19383	18:**40 X** vs. 24:**50 X**	-0.1527	-0.2966 to -0.008707	Yes
19384	18:**40 X** vs. 25:**0 X**	-0.8497	-1.011 to -0.6887	Yes
19385	18:**40 X** vs. 25:**1 X**	-1.039	-1.200 to -0.8782	Yes
19386	18:**40 X** vs. 25:**10 X**	-0.4007	-0.6043 to -0.1971	Yes
19387	18:**40 X** vs. 25:**40 X**	-0.2330	-0.3770 to -0.08904	Yes
19388	18:**40 X** vs. 25:**50 X**	-0.2073	-0.3513 to -0.06337	Yes
19389	18:**40 X** vs. 26:**0 X**	-1.062	-1.223 to -0.9012	Yes
19390	18:**40 X** vs. 26:**1 X**	-1.175	-1.336 to -1.014	Yes
19391	18:**40 X** vs. 26:**10 X**	-0.8617	-1.023 to -0.7007	Yes
19392	18:**40 X** vs. 26:**40 X**	-0.3073	-0.4513 to -0.1634	Yes
19393	18:**40 X** vs. 26:**50 X**	-0.1677	-0.3116 to -0.02371	Yes
19394	18:**40 X** vs. 27:**0 X**	-1.250	-1.394 to -1.106	Yes
19395	18:**40 X** vs. 27:**1 X**	-1.341	-1.502 to -1.180	Yes

	2way ANOVA<br>Multiple comparisons	A<br>Data Set-A<br>Y	B<br>Data Set-B<br>Y	C<br>Data Set-C<br>Y
19396	18:40 X vs. 27:10 X	-1.153	-1.314 to -0.9917	Yes
19397	18:40 X vs. 27:40 X	-0.3073	-0.4513 to -0.1634	Yes
19398	18:40 X vs. 27:50 X	-0.1670	-0.3110 to -0.02304	Yes
19399	18:40 X vs. 28:0 X	-1.199	-1.360 to -1.038	Yes
19400	18:40 X vs. 28:1 X	-1.240	-1.384 to -1.096	Yes
19401	18:40 X vs. 28:10 X	-1.042	-1.203 to -0.8807	Yes
19402	18:40 X vs. 28:40 X	-0.3197	-0.4636 to -0.1757	Yes
19403	18:40 X vs. 28:50 X	-0.2150	-0.3590 to -0.07104	Yes
19404	18:40 X vs. 29:0 X	-1.681	-1.842 to -1.520	Yes
19405	18:40 X vs. 29:1 X	-1.766	-1.927 to -1.605	Yes
19406	18:40 X vs. 29:10 X	-0.9802	-1.141 to -0.8192	Yes
19407	18:40 X vs. 29:40 X	-0.4277	-0.5716 to -0.2837	Yes
19408	18:40 X vs. 29:50 X	-0.2163	-0.3603 to -0.07237	Yes
19409	18:40 X vs. 30:0 X	-1.395	-1.556 to -1.234	Yes
19410	18:40 X vs. 30:1 X	-1.717	-1.878 to -1.556	Yes
19411	18:40 X vs. 30:10 X	-1.055	-1.216 to -0.8937	Yes
19412	18:40 X vs. 30:40 X	-0.3547	-0.4986 to -0.2107	Yes
19413	18:40 X vs. 30:50 X	-0.2687	-0.4126 to -0.1247	Yes
19414	18:40 X vs. 31:0 X	-1.713	-1.874 to -1.552	Yes
19415	18:40 X vs. 31:1 X	-1.704	-1.865 to -1.543	Yes
19416	18:40 X vs. 31:10 X	-1.186	-1.347 to -1.025	Yes
19417	18:40 X vs. 31:40 X	-0.4463	-0.5903 to -0.3024	Yes
19418	18:40 X vs. 31:50 X	-0.2477	-0.3916 to -0.1037	Yes
19419	18:40 X vs. 32:0 X	-2.305	-2.449 to -2.161	Yes
19420	18:40 X vs. 32:1 X	-1.761	-1.922 to -1.600	Yes
19421	18:40 X vs. 32:10 X	-1.617	-1.761 to -1.473	Yes
19422	18:40 X vs. 32:40 X	-0.4427	-0.5866 to -0.2987	Yes
19423	18:40 X vs. 32:50 X	-0.2473	-0.3913 to -0.1034	Yes
19424	18:40 X vs. 33:0 X	-2.308	-2.452 to -2.164	Yes
19425	18:40 X vs. 33:1 X	-2.123	-2.267 to -1.979	Yes
19426	18:40 X vs. 33:10 X	-1.783	-1.927 to -1.639	Yes
19427	18:40 X vs. 33:40 X	-0.4613	-0.6053 to -0.3174	Yes
19428	18:40 X vs. 33:50 X	-0.2623	-0.4063 to -0.1184	Yes
19429	18:40 X vs. 34:0 X	-2.233	-2.377 to -2.089	Yes
19430	18:40 X vs. 34:1 X	-2.240	-2.384 to -2.096	Yes
19431	18:40 X vs. 34:10 X	-1.874	-2.018 to -1.730	Yes
19432	18:40 X vs. 34:40 X	-0.4733	-0.6173 to -0.3294	Yes
19433	18:40 X vs. 34:50 X	-0.4113	-0.5553 to -0.2674	Yes
19434	18:40 X vs. 35:0 X	-2.582	-2.726 to -2.438	Yes
19435	18:40 X vs. 35:1 X	-2.347	-2.491 to -2.203	Yes
19436	18:40 X vs. 35:10 X	-1.835	-1.979 to -1.691	Yes
19437	18:40 X vs. 35:40 X	-0.4823	-0.6263 to -0.3384	Yes
19438	18:40 X vs. 35:50 X	-0.4240	-0.5680 to -0.2800	Yes
19439	18:40 X vs. 36:0 X	-2.788	-2.949 to -2.627	Yes
19440	18:40 X vs. 36:1 X	-2.484	-2.628 to -2.340	Yes

	2way ANOVA<br>Multiple comparisons	A<br>Data Set-A<br>Y	B<br>Data Set-B<br>Y	C<br>Data Set-C<br>Y
19441	18:40 X vs. 36:10 X	-1.841	-1.985 to -1.697	Yes
19442	18:40 X vs. 36:40 X	-0.5377	-0.6816 to -0.3937	Yes
19443	18:40 X vs. 36:50 X	-0.3873	-0.5313 to -0.2434	Yes
19444	18:40 X vs. 37:0 X	-3.172	-3.316 to -3.028	Yes
19445	18:40 X vs. 37:1 X	-2.554	-2.698 to -2.410	Yes
19446	18:40 X vs. 37:10 X	-1.843	-1.987 to -1.699	Yes
19447	18:40 X vs. 37:40 X	-0.5750	-0.7190 to -0.4310	Yes
19448	18:40 X vs. 37:50 X	-0.3960	-0.5400 to -0.2520	Yes
19449	18:40 X vs. 38:0 X	-3.117	-3.261 to -2.973	Yes
19450	18:40 X vs. 38:1 X	-2.673	-2.817 to -2.529	Yes
19451	18:40 X vs. 38:10 X	-2.118	-2.262 to -1.974	Yes
19452	18:40 X vs. 38:40 X	-0.5793	-0.7233 to -0.4354	Yes
19453	18:40 X vs. 38:50 X	-0.4000	-0.5440 to -0.2560	Yes
19454	18:40 X vs. 39:0 X	-3.348	-3.492 to -3.204	Yes
19455	18:40 X vs. 39:1 X	-3.239	-3.383 to -3.095	Yes
19456	18:40 X vs. 39:10 X	-2.263	-2.407 to -2.119	Yes
19457	18:40 X vs. 39:40 X	-0.5853	-0.7293 to -0.4414	Yes
19458	18:40 X vs. 39:50 X	-0.4310	-0.5750 to -0.2870	Yes
19459	18:40 X vs. 40:0 X	-3.389	-3.533 to -3.245	Yes
19460	18:40 X vs. 40:1 X	-3.315	-3.459 to -3.171	Yes
19461	18:40 X vs. 40:10 X	-2.519	-2.663 to -2.375	Yes
19462	18:40 X vs. 40:40 X	-0.5900	-0.7340 to -0.4460	Yes
19463	18:40 X vs. 40:50 X	-0.4373	-0.5813 to -0.2934	Yes
19464	18:40 X vs. 41:0 X	-3.430	-3.574 to -3.286	Yes
19465	18:40 X vs. 41:1 X	-3.429	-3.573 to -3.285	Yes
19466	18:40 X vs. 41:10 X	-2.573	-2.717 to -2.429	Yes
19467	18:40 X vs. 41:40 X	-0.5373	-0.6813 to -0.3934	Yes
19468	18:40 X vs. 41:50 X	-0.4500	-0.5940 to -0.3060	Yes
19469	18:40 X vs. 42:0 X	-3.571	-3.715 to -3.427	Yes
19470	18:40 X vs. 42:1 X	-3.591	-3.735 to -3.447	Yes
19471	18:40 X vs. 42:10 X	-2.573	-2.717 to -2.429	Yes
19472	18:40 X vs. 42:40 X	-0.5500	-0.6940 to -0.4060	Yes
19473	18:40 X vs. 42:50 X	-0.4533	-0.5973 to -0.3094	Yes
19474	18:40 X vs. 43:0 X	-3.661	-3.805 to -3.517	Yes
19475	18:40 X vs. 43:1 X	-3.576	-3.720 to -3.432	Yes
19476	18:40 X vs. 43:10 X	-3.291	-3.435 to -3.147	Yes
19477	18:40 X vs. 43:40 X	-0.5483	-0.6923 to -0.4044	Yes
19478	18:40 X vs. 43:50 X	-0.4537	-0.5976 to -0.3097	Yes
19479	18:40 X vs. 44:0 X	-3.685	-3.829 to -3.541	Yes
19480	18:40 X vs. 44:1 X	-3.575	-3.719 to -3.431	Yes
19481	18:40 X vs. 44:10 X	-3.355	-3.499 to -3.211	Yes
19482	18:40 X vs. 44:40 X	-0.5503	-0.6943 to -0.4064	Yes
19483	18:40 X vs. 44:50 X	-0.3860	-0.5300 to -0.2420	Yes
19484	18:40 X vs. 45:0 X	-3.550	-3.694 to -3.406	Yes
19485	18:40 X vs. 45:1 X	-3.352	-3.496 to -3.208	Yes

	2way ANOVA<br>Multiple comparisons	A<br>Data Set-A<br>Y	B<br>Data Set-B<br>Y	C<br>Data Set-C<br>Y
19486	18:**40 X** vs. 45:**10 X**	-3.234	-3.378 to -3.090	Yes
19487	18:**40 X** vs. 45:**40 X**	-0.5517	-0.6956 to -0.4077	Yes
19488	18:**40 X** vs. 45:**50 X**	-0.3830	-0.5270 to -0.2390	Yes
19489	18:**40 X** vs. 46:**0 X**	-3.354	-3.498 to -3.210	Yes
19490	18:**40 X** vs. 46:**1 X**	-3.321	-3.465 to -3.177	Yes
19491	18:**40 X** vs. 46:**10 X**	-3.229	-3.373 to -3.085	Yes
19492	18:**40 X** vs. 46:**40 X**	-0.5653	-0.7093 to -0.4214	Yes
19493	18:**40 X** vs. 46:**50 X**	-0.3760	-0.5200 to -0.2320	Yes
19494	18:**40 X** vs. 47:**0 X**	-3.330	-3.474 to -3.186	Yes
19495	18:**40 X** vs. 47:**1 X**	-3.234	-3.378 to -3.090	Yes
19496	18:**40 X** vs. 47:**10 X**	-3.215	-3.359 to -3.071	Yes
19497	18:**40 X** vs. 47:**40 X**	-0.5753	-0.7193 to -0.4314	Yes
19498	18:**40 X** vs. 47:**50 X**	-0.3340	-0.4780 to -0.1900	Yes
19499	18:**40 X** vs. 48:**0 X**	-3.317	-3.478 to -3.156	Yes
19500	18:**40 X** vs. 48:**1 X**	-3.221	-3.365 to -3.077	Yes
19501	18:**40 X** vs. 48:**10 X**	-3.215	-3.359 to -3.071	Yes
19502	18:**40 X** vs. 48:**40 X**	-0.5817	-0.7256 to -0.4377	Yes
19503	18:**40 X** vs. 48:**50 X**	-0.3413	-0.4853 to -0.1974	Yes
19504	18:**40 X** vs. 49:**0 X**	-3.317	-3.461 to -3.173	Yes
19505	18:**40 X** vs. 49:**1 X**	-3.221	-3.365 to -3.077	Yes
19506	18:**40 X** vs. 49:**10 X**	-3.215	-3.359 to -3.071	Yes
19507	18:**40 X** vs. 49:**40 X**	-0.5860	-0.7300 to -0.4420	Yes
19508	18:**40 X** vs. 49:**50 X**	-0.3407	-0.4846 to -0.1967	Yes
19509	18:**40 X** vs. 50:**0 X**	-3.317	-3.461 to -3.173	Yes
19510	18:**40 X** vs. 50:**1 X**	-3.221	-3.365 to -3.077	Yes
19511	18:**40 X** vs. 50:**10 X**	-3.215	-3.359 to -3.071	Yes
19512	18:**40 X** vs. 50:**40 X**	-0.5937	-0.7376 to -0.4497	Yes
19513	18:**40 X** vs. 50:**50 X**	-0.3403	-0.4843 to -0.1964	Yes
19514	18:**50 X** vs. 19:**0 X**	-0.1400	-0.2840 to 0.003960	No
19515	18:**50 X** vs. 19:**1 X**	-0.1280	-0.2720 to 0.01596	No
19516	18:**50 X** vs. 19:**10 X**	-0.1257	-0.2696 to 0.01829	No
19517	18:**50 X** vs. 19:**40 X**	0.01633	-0.1276 to 0.1603	No
19518	18:**50 X** vs. 19:**50 X**	0.005333	-0.1386 to 0.1493	No
19519	18:**50 X** vs. 20:**0 X**	-0.2197	-0.3636 to -0.07571	Yes
19520	18:**50 X** vs. 20:**1 X**	-0.0390	-0.1830 to 0.1050	No
19521	18:**50 X** vs. 20:**10 X**	-0.1280	-0.2720 to 0.01596	No
19522	18:**50 X** vs. 20:**40 X**	0.004667	-0.1393 to 0.1486	No
19523	18:**50 X** vs. 20:**50 X**	-0.03933	-0.1833 to 0.1046	No
19524	18:**50 X** vs. 21:**0 X**	-0.2430	-0.3870 to -0.09904	Yes
19525	18:**50 X** vs. 21:**1 X**	-0.1960	-0.3400 to -0.05204	Yes
19526	18:**50 X** vs. 21:**10 X**	-0.1930	-0.3370 to -0.04904	Yes
19527	18:**50 X** vs. 21:**40 X**	-0.06833	-0.2123 to 0.07563	No
19528	18:**50 X** vs. 21:**50 X**	-0.1587	-0.3026 to -0.01471	Yes
19529	18:**50 X** vs. 22:**0 X**	-0.2847	-0.4286 to -0.1407	Yes
19530	18:**50 X** vs. 22:**1 X**	-0.2793	-0.4233 to -0.1354	Yes

2way ANOVA<br>Multiple comparisons	A<br>Data Set-A<br>Y	B<br>Data Set-B<br>Y	C<br>Data Set-C<br>Y
19531 18:50 X vs. 22:10 X	-0.2747	-0.4186 to -0.1307	Yes
19532 18:50 X vs. 22:40 X	-0.1750	-0.3190 to -0.03104	Yes
19533 18:50 X vs. 22:50 X	-0.2377	-0.3816 to -0.09371	Yes
19534 18:50 X vs. 23:0 X	-0.3500	-0.4940 to -0.2060	Yes
19535 18:50 X vs. 23:1 X	-0.5397	-0.7006 to -0.3787	Yes
19536 18:50 X vs. 23:10 X	-0.2893	-0.4333 to -0.1454	Yes
19537 18:50 X vs. 23:40 X	-0.1697	-0.3306 to -0.008715	Yes
19538 18:50 X vs. 23:50 X	-0.2037	-0.3476 to -0.05971	Yes
19539 18:50 X vs. 24:0 X	-0.4673	-0.6113 to -0.3234	Yes
19540 18:50 X vs. 24:1 X	-0.6237	-0.7846 to -0.4627	Yes
19541 18:50 X vs. 24:10 X	-0.4512	-0.6121 to -0.2902	Yes
19542 18:50 X vs. 24:40 X	-0.1860	-0.3300 to -0.04204	Yes
19543 18:50 X vs. 24:50 X	-0.1077	-0.2516 to 0.03629	No
19544 18:50 X vs. 25:0 X	-0.8047	-0.9656 to -0.6437	Yes
19545 18:50 X vs. 25:1 X	-0.9942	-1.155 to -0.8332	Yes
19546 18:50 X vs. 25:10 X	-0.3557	-0.5593 to -0.1521	Yes
19547 18:50 X vs. 25:40 X	-0.1880	-0.3320 to -0.04404	Yes
19548 18:50 X vs. 25:50 X	-0.1623	-0.3063 to -0.01837	Yes
19549 18:50 X vs. 26:0 X	-1.017	-1.178 to -0.8562	Yes
19550 18:50 X vs. 26:1 X	-1.130	-1.291 to -0.9692	Yes
19551 18:50 X vs. 26:10 X	-0.8167	-0.9776 to -0.6557	Yes
19552 18:50 X vs. 26:40 X	-0.2623	-0.4063 to -0.1184	Yes
19553 18:50 X vs. 26:50 X	-0.1227	-0.2666 to 0.02129	No
19554 18:50 X vs. 27:0 X	-1.205	-1.349 to -1.061	Yes
19555 18:50 X vs. 27:1 X	-1.296	-1.457 to -1.135	Yes
19556 18:50 X vs. 27:10 X	-1.108	-1.269 to -0.9467	Yes
19557 18:50 X vs. 27:40 X	-0.2623	-0.4063 to -0.1184	Yes
19558 18:50 X vs. 27:50 X	-0.1220	-0.2660 to 0.02196	No
19559 18:50 X vs. 28:0 X	-1.154	-1.315 to -0.9932	Yes
19560 18:50 X vs. 28:1 X	-1.195	-1.339 to -1.051	Yes
19561 18:50 X vs. 28:10 X	-0.9967	-1.158 to -0.8357	Yes
19562 18:50 X vs. 28:40 X	-0.2747	-0.4186 to -0.1307	Yes
19563 18:50 X vs. 28:50 X	-0.1700	-0.3140 to -0.02604	Yes
19564 18:50 X vs. 29:0 X	-1.636	-1.797 to -1.475	Yes
19565 18:50 X vs. 29:1 X	-1.721	-1.882 to -1.560	Yes
19566 18:50 X vs. 29:10 X	-0.9352	-1.096 to -0.7742	Yes
19567 18:50 X vs. 29:40 X	-0.3827	-0.5266 to -0.2387	Yes
19568 18:50 X vs. 29:50 X	-0.1713	-0.3153 to -0.02737	Yes
19569 18:50 X vs. 30:0 X	-1.350	-1.511 to -1.189	Yes
19570 18:50 X vs. 30:1 X	-1.672	-1.833 to -1.511	Yes
19571 18:50 X vs. 30:10 X	-1.010	-1.171 to -0.8487	Yes
19572 18:50 X vs. 30:40 X	-0.3097	-0.4536 to -0.1657	Yes
19573 18:50 X vs. 30:50 X	-0.2237	-0.3676 to -0.07971	Yes
19574 18:50 X vs. 31:0 X	-1.668	-1.829 to -1.507	Yes
19575 18:50 X vs. 31:1 X	-1.659	-1.820 to -1.498	Yes

	2way ANOVA<br>Multiple comparisons	A<br>Data Set-A<br>Y	B<br>Data Set-B<br>Y	C<br>Data Set-C<br>Y
19576	18:50 X vs. 31:10 X	-1.141	-1.302 to -0.9802	Yes
19577	18:50 X vs. 31:40 X	-0.4013	-0.5453 to -0.2574	Yes
19578	18:50 X vs. 31:50 X	-0.2027	-0.3466 to -0.05871	Yes
19579	18:50 X vs. 32:0 X	-2.260	-2.404 to -2.116	Yes
19580	18:50 X vs. 32:1 X	-1.716	-1.877 to -1.555	Yes
19581	18:50 X vs. 32:10 X	-1.572	-1.716 to -1.428	Yes
19582	18:50 X vs. 32:40 X	-0.3977	-0.5416 to -0.2537	Yes
19583	18:50 X vs. 32:50 X	-0.2023	-0.3463 to -0.05837	Yes
19584	18:50 X vs. 33:0 X	-2.263	-2.407 to -2.119	Yes
19585	18:50 X vs. 33:1 X	-2.078	-2.222 to -1.934	Yes
19586	18:50 X vs. 33:10 X	-1.738	-1.882 to -1.594	Yes
19587	18:50 X vs. 33:40 X	-0.4163	-0.5603 to -0.2724	Yes
19588	18:50 X vs. 33:50 X	-0.2173	-0.3613 to -0.07337	Yes
19589	18:50 X vs. 34:0 X	-2.188	-2.332 to -2.044	Yes
19590	18:50 X vs. 34:1 X	-2.195	-2.339 to -2.051	Yes
19591	18:50 X vs. 34:10 X	-1.829	-1.973 to -1.685	Yes
19592	18:50 X vs. 34:40 X	-0.4283	-0.5723 to -0.2844	Yes
19593	18:50 X vs. 34:50 X	-0.3663	-0.5103 to -0.2224	Yes
19594	18:50 X vs. 35:0 X	-2.537	-2.681 to -2.393	Yes
19595	18:50 X vs. 35:1 X	-2.302	-2.446 to -2.158	Yes
19596	18:50 X vs. 35:10 X	-1.790	-1.934 to -1.646	Yes
19597	18:50 X vs. 35:40 X	-0.4373	-0.5813 to -0.2934	Yes
19598	18:50 X vs. 35:50 X	-0.3790	-0.5230 to -0.2350	Yes
19599	18:50 X vs. 36:0 X	-2.743	-2.904 to -2.582	Yes
19600	18:50 X vs. 36:1 X	-2.439	-2.583 to -2.295	Yes
19601	18:50 X vs. 36:10 X	-1.796	-1.940 to -1.652	Yes
19602	18:50 X vs. 36:40 X	-0.4927	-0.6366 to -0.3487	Yes
19603	18:50 X vs. 36:50 X	-0.3423	-0.4863 to -0.1984	Yes
19604	18:50 X vs. 37:0 X	-3.127	-3.271 to -2.983	Yes
19605	18:50 X vs. 37:1 X	-2.509	-2.653 to -2.365	Yes
19606	18:50 X vs. 37:10 X	-1.798	-1.942 to -1.654	Yes
19607	18:50 X vs. 37:40 X	-0.5300	-0.6740 to -0.3860	Yes
19608	18:50 X vs. 37:50 X	-0.3510	-0.4950 to -0.2070	Yes
19609	18:50 X vs. 38:0 X	-3.072	-3.216 to -2.928	Yes
19610	18:50 X vs. 38:1 X	-2.628	-2.772 to -2.484	Yes
19611	18:50 X vs. 38:10 X	-2.073	-2.217 to -1.929	Yes
19612	18:50 X vs. 38:40 X	-0.5343	-0.6783 to -0.3904	Yes
19613	18:50 X vs. 38:50 X	-0.3550	-0.4990 to -0.2110	Yes
19614	18:50 X vs. 39:0 X	-3.303	-3.447 to -3.159	Yes
19615	18:50 X vs. 39:1 X	-3.194	-3.338 to -3.050	Yes
19616	18:50 X vs. 39:10 X	-2.218	-2.362 to -2.074	Yes
19617	18:50 X vs. 39:40 X	-0.5403	-0.6843 to -0.3964	Yes
19618	18:50 X vs. 39:50 X	-0.3860	-0.5300 to -0.2420	Yes
19619	18:50 X vs. 40:0 X	-3.344	-3.488 to -3.200	Yes
19620	18:50 X vs. 40:1 X	-3.270	-3.414 to -3.126	Yes

	2way ANOVA<br>Multiple comparisons	A<br>Data Set-A<br>Y	B<br>Data Set-B<br>Y	C<br>Data Set-C<br>Y
19621	18:**50 X** vs. 40:**10 X**	-2.474	-2.618 to -2.330	Yes
19622	18:**50 X** vs. 40:**40 X**	-0.5450	-0.6890 to -0.4010	Yes
19623	18:**50 X** vs. 40:**50 X**	-0.3923	-0.5363 to -0.2484	Yes
19624	18:**50 X** vs. 41:**0 X**	-3.385	-3.529 to -3.241	Yes
19625	18:**50 X** vs. 41:**1 X**	-3.384	-3.528 to -3.240	Yes
19626	18:**50 X** vs. 41:**10 X**	-2.528	-2.672 to -2.384	Yes
19627	18:**50 X** vs. 41:**40 X**	-0.4923	-0.6363 to -0.3484	Yes
19628	18:**50 X** vs. 41:**50 X**	-0.4050	-0.5490 to -0.2610	Yes
19629	18:**50 X** vs. 42:**0 X**	-3.526	-3.670 to -3.382	Yes
19630	18:**50 X** vs. 42:**1 X**	-3.546	-3.690 to -3.402	Yes
19631	18:**50 X** vs. 42:**10 X**	-2.528	-2.672 to -2.384	Yes
19632	18:**50 X** vs. 42:**40 X**	-0.5050	-0.6490 to -0.3610	Yes
19633	18:**50 X** vs. 42:**50 X**	-0.4083	-0.5523 to -0.2644	Yes
19634	18:**50 X** vs. 43:**0 X**	-3.616	-3.760 to -3.472	Yes
19635	18:**50 X** vs. 43:**1 X**	-3.531	-3.675 to -3.387	Yes
19636	18:**50 X** vs. 43:**10 X**	-3.246	-3.390 to -3.102	Yes
19637	18:**50 X** vs. 43:**40 X**	-0.5033	-0.6473 to -0.3594	Yes
19638	18:**50 X** vs. 43:**50 X**	-0.4087	-0.5526 to -0.2647	Yes
19639	18:**50 X** vs. 44:**0 X**	-3.640	-3.784 to -3.496	Yes
19640	18:**50 X** vs. 44:**1 X**	-3.530	-3.674 to -3.386	Yes
19641	18:**50 X** vs. 44:**10 X**	-3.310	-3.454 to -3.166	Yes
19642	18:**50 X** vs. 44:**40 X**	-0.5053	-0.6493 to -0.3614	Yes
19643	18:**50 X** vs. 44:**50 X**	-0.3410	-0.4850 to -0.1970	Yes
19644	18:**50 X** vs. 45:**0 X**	-3.505	-3.649 to -3.361	Yes
19645	18:**50 X** vs. 45:**1 X**	-3.307	-3.451 to -3.163	Yes
19646	18:**50 X** vs. 45:**10 X**	-3.189	-3.333 to -3.045	Yes
19647	18:**50 X** vs. 45:**40 X**	-0.5067	-0.6506 to -0.3627	Yes
19648	18:**50 X** vs. 45:**50 X**	-0.3380	-0.4820 to -0.1940	Yes
19649	18:**50 X** vs. 46:**0 X**	-3.309	-3.453 to -3.165	Yes
19650	18:**50 X** vs. 46:**1 X**	-3.276	-3.420 to -3.132	Yes
19651	18:**50 X** vs. 46:**10 X**	-3.184	-3.328 to -3.040	Yes
19652	18:**50 X** vs. 46:**40 X**	-0.5203	-0.6643 to -0.3764	Yes
19653	18:**50 X** vs. 46:**50 X**	-0.3310	-0.4750 to -0.1870	Yes
19654	18:**50 X** vs. 47:**0 X**	-3.285	-3.429 to -3.141	Yes
19655	18:**50 X** vs. 47:**1 X**	-3.189	-3.333 to -3.045	Yes
19656	18:**50 X** vs. 47:**10 X**	-3.170	-3.314 to -3.026	Yes
19657	18:**50 X** vs. 47:**40 X**	-0.5303	-0.6743 to -0.3864	Yes
19658	18:**50 X** vs. 47:**50 X**	-0.2890	-0.4330 to -0.1450	Yes
19659	18:**50 X** vs. 48:**0 X**	-3.272	-3.433 to -3.111	Yes
19660	18:**50 X** vs. 48:**1 X**	-3.176	-3.320 to -3.032	Yes
19661	18:**50 X** vs. 48:**10 X**	-3.170	-3.314 to -3.026	Yes
19662	18:**50 X** vs. 48:**40 X**	-0.5367	-0.6806 to -0.3927	Yes
19663	18:**50 X** vs. 48:**50 X**	-0.2963	-0.4403 to -0.1524	Yes
19664	18:**50 X** vs. 49:**0 X**	-3.272	-3.416 to -3.128	Yes
19665	18:**50 X** vs. 49:**1 X**	-3.176	-3.320 to -3.032	Yes

	2way ANOVA<br>Multiple comparisons	A<br>Data Set-A<br>Y	B<br>Data Set-B<br>Y	C<br>Data Set-C<br>Y
19666	18:**50 X** vs. 49:**10 X**	-3.170	-3.314 to -3.026	Yes
19667	18:**50 X** vs. 49:**40 X**	-0.5410	-0.6850 to -0.3970	Yes
19668	18:**50 X** vs. 49:**50 X**	-0.2957	-0.4396 to -0.1517	Yes
19669	18:**50 X** vs. 50:**0 X**	-3.272	-3.416 to -3.128	Yes
19670	18:**50 X** vs. 50:**1 X**	-3.176	-3.320 to -3.032	Yes
19671	18:**50 X** vs. 50:**10 X**	-3.170	-3.314 to -3.026	Yes
19672	18:**50 X** vs. 50:**40 X**	-0.5487	-0.6926 to -0.4047	Yes
19673	18:**50 X** vs. 50:**50 X**	-0.2953	-0.4393 to -0.1514	Yes
19674	19:**0 X** vs. 19:**1 X**	0.0120	-0.1320 to 0.1560	No
19675	19:**0 X** vs. 19:**10 X**	0.01433	-0.1296 to 0.1583	No
19676	19:**0 X** vs. 19:**40 X**	0.1563	0.01237 to 0.3003	Yes
19677	19:**0 X** vs. 19:**50 X**	0.1453	0.001374 to 0.2893	Yes
19678	19:**0 X** vs. 20:**0 X**	-0.07967	-0.2236 to 0.06429	No
19679	19:**0 X** vs. 20:**1 X**	0.1010	-0.04296 to 0.2450	No
19680	19:**0 X** vs. 20:**10 X**	0.0120	-0.1320 to 0.1560	No
19681	19:**0 X** vs. 20:**40 X**	0.1447	0.0007068 to 0.2886	Yes
19682	19:**0 X** vs. 20:**50 X**	0.1007	-0.04329 to 0.2446	No
19683	19:**0 X** vs. 21:**0 X**	-0.1030	-0.2470 to 0.04096	No
19684	19:**0 X** vs. 21:**1 X**	-0.05600	-0.2000 to 0.08796	No
19685	19:**0 X** vs. 21:**10 X**	-0.0530	-0.1970 to 0.09096	No
19686	19:**0 X** vs. 21:**40 X**	0.07167	-0.07229 to 0.2156	No
19687	19:**0 X** vs. 21:**50 X**	-0.01867	-0.1626 to 0.1253	No
19688	19:**0 X** vs. 22:**0 X**	-0.1447	-0.2886 to -0.0007068	Yes
19689	19:**0 X** vs. 22:**1 X**	-0.1393	-0.2833 to 0.004626	No
19690	19:**0 X** vs. 22:**10 X**	-0.1347	-0.2786 to 0.009293	No
19691	19:**0 X** vs. 22:**40 X**	-0.03500	-0.1790 to 0.1090	No
19692	19:**0 X** vs. 22:**50 X**	-0.09767	-0.2416 to 0.04629	No
19693	19:**0 X** vs. 23:**0 X**	-0.2100	-0.3540 to -0.06604	Yes
19694	19:**0 X** vs. 23:**1 X**	-0.3997	-0.5606 to -0.2387	Yes
19695	19:**0 X** vs. 23:**10 X**	-0.1493	-0.2933 to -0.005374	Yes
19696	19:**0 X** vs. 23:**40 X**	-0.02967	-0.1906 to 0.1313	No
19697	19:**0 X** vs. 23:**50 X**	-0.06367	-0.2076 to 0.08029	No
19698	19:**0 X** vs. 24:**0 X**	-0.3273	-0.4713 to -0.1834	Yes
19699	19:**0 X** vs. 24:**1 X**	-0.4837	-0.6446 to -0.3227	Yes
19700	19:**0 X** vs. 24:**10 X**	-0.3112	-0.4721 to -0.1502	Yes
19701	19:**0 X** vs. 24:**40 X**	-0.0460	-0.1900 to 0.09796	No
19702	19:**0 X** vs. 24:**50 X**	0.03233	-0.1116 to 0.1763	No
19703	19:**0 X** vs. 25:**0 X**	-0.6647	-0.8256 to -0.5037	Yes
19704	19:**0 X** vs. 25:**1 X**	-0.8542	-1.015 to -0.6932	Yes
19705	19:**0 X** vs. 25:**10 X**	-0.2157	-0.4193 to -0.01208	Yes
19706	19:**0 X** vs. 25:**40 X**	-0.0480	-0.1920 to 0.09596	No
19707	19:**0 X** vs. 25:**50 X**	-0.02233	-0.1663 to 0.1216	No
19708	19:**0 X** vs. 26:**0 X**	-0.8772	-1.038 to -0.7162	Yes
19709	19:**0 X** vs. 26:**1 X**	-0.9902	-1.151 to -0.8292	Yes
19710	19:**0 X** vs. 26:**10 X**	-0.6767	-0.8376 to -0.5157	Yes

	2way ANOVA<br>Multiple comparisons	A<br>Data Set-A<br>Y	B<br>Data Set-B<br>Y	C<br>Data Set-C<br>Y
19711	19:**0 X** vs. 26:**40 X**	-0.1223	-0.2663 to 0.02163	No
19712	19:**0 X** vs. 26:**50 X**	0.01733	-0.1266 to 0.1613	No
19713	19:**0 X** vs. 27:**0 X**	-1.065	-1.209 to -0.9210	Yes
19714	19:**0 X** vs. 27:**1 X**	-1.156	-1.317 to -0.9947	Yes
19715	19:**0 X** vs. 27:**10 X**	-0.9677	-1.129 to -0.8067	Yes
19716	19:**0 X** vs. 27:**40 X**	-0.1223	-0.2663 to 0.02163	No
19717	19:**0 X** vs. 27:**50 X**	0.0180	-0.1260 to 0.1620	No
19718	19:**0 X** vs. 28:**0 X**	-1.014	-1.175 to -0.8532	Yes
19719	19:**0 X** vs. 28:**1 X**	-1.055	-1.199 to -0.9110	Yes
19720	19:**0 X** vs. 28:**10 X**	-0.8567	-1.018 to -0.6957	Yes
19721	19:**0 X** vs. 28:**40 X**	-0.1347	-0.2786 to 0.009293	No
19722	19:**0 X** vs. 28:**50 X**	-0.03000	-0.1740 to 0.1140	No
19723	19:**0 X** vs. 29:**0 X**	-1.496	-1.657 to -1.335	Yes
19724	19:**0 X** vs. 29:**1 X**	-1.581	-1.742 to -1.420	Yes
19725	19:**0 X** vs. 29:**10 X**	-0.7952	-0.9561 to -0.6342	Yes
19726	19:**0 X** vs. 29:**40 X**	-0.2427	-0.3866 to -0.09871	Yes
19727	19:**0 X** vs. 29:**50 X**	-0.03133	-0.1753 to 0.1126	No
19728	19:**0 X** vs. 30:**0 X**	-1.210	-1.371 to -1.049	Yes
19729	19:**0 X** vs. 30:**1 X**	-1.532	-1.693 to -1.371	Yes
19730	19:**0 X** vs. 30:**10 X**	-0.8697	-1.031 to -0.7087	Yes
19731	19:**0 X** vs. 30:**40 X**	-0.1697	-0.3136 to -0.02571	Yes
19732	19:**0 X** vs. 30:**50 X**	-0.08367	-0.2276 to 0.06029	No
19733	19:**0 X** vs. 31:**0 X**	-1.528	-1.689 to -1.367	Yes
19734	19:**0 X** vs. 31:**1 X**	-1.519	-1.680 to -1.358	Yes
19735	19:**0 X** vs. 31:**10 X**	-1.001	-1.162 to -0.8402	Yes
19736	19:**0 X** vs. 31:**40 X**	-0.2613	-0.4053 to -0.1174	Yes
19737	19:**0 X** vs. 31:**50 X**	-0.06267	-0.2066 to 0.08129	No
19738	19:**0 X** vs. 32:**0 X**	-2.120	-2.264 to -1.976	Yes
19739	19:**0 X** vs. 32:**1 X**	-1.576	-1.737 to -1.415	Yes
19740	19:**0 X** vs. 32:**10 X**	-1.432	-1.576 to -1.288	Yes
19741	19:**0 X** vs. 32:**40 X**	-0.2577	-0.4016 to -0.1137	Yes
19742	19:**0 X** vs. 32:**50 X**	-0.06233	-0.2063 to 0.08163	No
19743	19:**0 X** vs. 33:**0 X**	-2.123	-2.267 to -1.979	Yes
19744	19:**0 X** vs. 33:**1 X**	-1.938	-2.082 to -1.794	Yes
19745	19:**0 X** vs. 33:**10 X**	-1.598	-1.742 to -1.454	Yes
19746	19:**0 X** vs. 33:**40 X**	-0.2763	-0.4203 to -0.1324	Yes
19747	19:**0 X** vs. 33:**50 X**	-0.07733	-0.2213 to 0.06663	No
19748	19:**0 X** vs. 34:**0 X**	-2.048	-2.192 to -1.904	Yes
19749	19:**0 X** vs. 34:**1 X**	-2.055	-2.199 to -1.911	Yes
19750	19:**0 X** vs. 34:**10 X**	-1.689	-1.833 to -1.545	Yes
19751	19:**0 X** vs. 34:**40 X**	-0.2883	-0.4323 to -0.1444	Yes
19752	19:**0 X** vs. 34:**50 X**	-0.2263	-0.3703 to -0.08237	Yes
19753	19:**0 X** vs. 35:**0 X**	-2.397	-2.541 to -2.253	Yes
19754	19:**0 X** vs. 35:**1 X**	-2.162	-2.306 to -2.018	Yes
19755	19:**0 X** vs. 35:**10 X**	-1.650	-1.794 to -1.506	Yes

	2way ANOVA<br>Multiple comparisons	A<br>Data Set-A<br>Y	B<br>Data Set-B<br>Y	C<br>Data Set-C<br>Y
19756	19:0 X vs. 35:40 X	-0.2973	-0.4413 to -0.1534	Yes
19757	19:0 X vs. 35:50 X	-0.2390	-0.3830 to -0.09504	Yes
19758	19:0 X vs. 36:0 X	-2.603	-2.764 to -2.442	Yes
19759	19:0 X vs. 36:1 X	-2.299	-2.443 to -2.155	Yes
19760	19:0 X vs. 36:10 X	-1.656	-1.800 to -1.512	Yes
19761	19:0 X vs. 36:40 X	-0.3527	-0.4966 to -0.2087	Yes
19762	19:0 X vs. 36:50 X	-0.2023	-0.3463 to -0.05837	Yes
19763	19:0 X vs. 37:0 X	-2.987	-3.131 to -2.843	Yes
19764	19:0 X vs. 37:1 X	-2.369	-2.513 to -2.225	Yes
19765	19:0 X vs. 37:10 X	-1.658	-1.802 to -1.514	Yes
19766	19:0 X vs. 37:40 X	-0.3900	-0.5340 to -0.2460	Yes
19767	19:0 X vs. 37:50 X	-0.2110	-0.3550 to -0.06704	Yes
19768	19:0 X vs. 38:0 X	-2.932	-3.076 to -2.788	Yes
19769	19:0 X vs. 38:1 X	-2.488	-2.632 to -2.344	Yes
19770	19:0 X vs. 38:10 X	-1.933	-2.077 to -1.789	Yes
19771	19:0 X vs. 38:40 X	-0.3943	-0.5383 to -0.2504	Yes
19772	19:0 X vs. 38:50 X	-0.2150	-0.3590 to -0.07104	Yes
19773	19:0 X vs. 39:0 X	-3.163	-3.307 to -3.019	Yes
19774	19:0 X vs. 39:1 X	-3.054	-3.198 to -2.910	Yes
19775	19:0 X vs. 39:10 X	-2.078	-2.222 to -1.934	Yes
19776	19:0 X vs. 39:40 X	-0.4003	-0.5443 to -0.2564	Yes
19777	19:0 X vs. 39:50 X	-0.2460	-0.3900 to -0.1020	Yes
19778	19:0 X vs. 40:0 X	-3.204	-3.348 to -3.060	Yes
19779	19:0 X vs. 40:1 X	-3.130	-3.274 to -2.986	Yes
19780	19:0 X vs. 40:10 X	-2.334	-2.478 to -2.190	Yes
19781	19:0 X vs. 40:40 X	-0.4050	-0.5490 to -0.2610	Yes
19782	19:0 X vs. 40:50 X	-0.2523	-0.3963 to -0.1084	Yes
19783	19:0 X vs. 41:0 X	-3.245	-3.389 to -3.101	Yes
19784	19:0 X vs. 41:1 X	-3.244	-3.388 to -3.100	Yes
19785	19:0 X vs. 41:10 X	-2.388	-2.532 to -2.244	Yes
19786	19:0 X vs. 41:40 X	-0.3523	-0.4963 to -0.2084	Yes
19787	19:0 X vs. 41:50 X	-0.2650	-0.4090 to -0.1210	Yes
19788	19:0 X vs. 42:0 X	-3.386	-3.530 to -3.242	Yes
19789	19:0 X vs. 42:1 X	-3.406	-3.550 to -3.262	Yes
19790	19:0 X vs. 42:10 X	-2.388	-2.532 to -2.244	Yes
19791	19:0 X vs. 42:40 X	-0.3650	-0.5090 to -0.2210	Yes
19792	19:0 X vs. 42:50 X	-0.2683	-0.4123 to -0.1244	Yes
19793	19:0 X vs. 43:0 X	-3.476	-3.620 to -3.332	Yes
19794	19:0 X vs. 43:1 X	-3.391	-3.535 to -3.247	Yes
19795	19:0 X vs. 43:10 X	-3.106	-3.250 to -2.962	Yes
19796	19:0 X vs. 43:40 X	-0.3633	-0.5073 to -0.2194	Yes
19797	19:0 X vs. 43:50 X	-0.2687	-0.4126 to -0.1247	Yes
19798	19:0 X vs. 44:0 X	-3.500	-3.644 to -3.356	Yes
19799	19:0 X vs. 44:1 X	-3.390	-3.534 to -3.246	Yes
19800	19:0 X vs. 44:10 X	-3.170	-3.314 to -3.026	Yes

	2way ANOVA<br>Multiple comparisons	A<br>Data Set-A<br>Y	B<br>Data Set-B<br>Y	C<br>Data Set-C<br>Y
19801	19:**0 X** vs. 44:**40 X**	-0.3653	-0.5093 to -0.2214	Yes
19802	19:**0 X** vs. 44:**50 X**	-0.2010	-0.3450 to -0.05704	Yes
19803	19:**0 X** vs. 45:**0 X**	-3.365	-3.509 to -3.221	Yes
19804	19:**0 X** vs. 45:**1 X**	-3.167	-3.311 to -3.023	Yes
19805	19:**0 X** vs. 45:**10 X**	-3.049	-3.193 to -2.905	Yes
19806	19:**0 X** vs. 45:**40 X**	-0.3667	-0.5106 to -0.2227	Yes
19807	19:**0 X** vs. 45:**50 X**	-0.1980	-0.3420 to -0.05404	Yes
19808	19:**0 X** vs. 46:**0 X**	-3.169	-3.313 to -3.025	Yes
19809	19:**0 X** vs. 46:**1 X**	-3.136	-3.280 to -2.992	Yes
19810	19:**0 X** vs. 46:**10 X**	-3.044	-3.188 to -2.900	Yes
19811	19:**0 X** vs. 46:**40 X**	-0.3803	-0.5243 to -0.2364	Yes
19812	19:**0 X** vs. 46:**50 X**	-0.1910	-0.3350 to -0.04704	Yes
19813	19:**0 X** vs. 47:**0 X**	-3.145	-3.289 to -3.001	Yes
19814	19:**0 X** vs. 47:**1 X**	-3.049	-3.193 to -2.905	Yes
19815	19:**0 X** vs. 47:**10 X**	-3.030	-3.174 to -2.886	Yes
19816	19:**0 X** vs. 47:**40 X**	-0.3903	-0.5343 to -0.2464	Yes
19817	19:**0 X** vs. 47:**50 X**	-0.1490	-0.2930 to -0.005040	Yes
19818	19:**0 X** vs. 48:**0 X**	-3.132	-3.293 to -2.971	Yes
19819	19:**0 X** vs. 48:**1 X**	-3.036	-3.180 to -2.892	Yes
19820	19:**0 X** vs. 48:**10 X**	-3.030	-3.174 to -2.886	Yes
19821	19:**0 X** vs. 48:**40 X**	-0.3967	-0.5406 to -0.2527	Yes
19822	19:**0 X** vs. 48:**50 X**	-0.1563	-0.3003 to -0.01237	Yes
19823	19:**0 X** vs. 49:**0 X**	-3.132	-3.276 to -2.988	Yes
19824	19:**0 X** vs. 49:**1 X**	-3.036	-3.180 to -2.892	Yes
19825	19:**0 X** vs. 49:**10 X**	-3.030	-3.174 to -2.886	Yes
19826	19:**0 X** vs. 49:**40 X**	-0.4010	-0.5450 to -0.2570	Yes
19827	19:**0 X** vs. 49:**50 X**	-0.1557	-0.2996 to -0.01171	Yes
19828	19:**0 X** vs. 50:**0 X**	-3.132	-3.276 to -2.988	Yes
19829	19:**0 X** vs. 50:**1 X**	-3.036	-3.180 to -2.892	Yes
19830	19:**0 X** vs. 50:**10 X**	-3.030	-3.174 to -2.886	Yes
19831	19:**0 X** vs. 50:**40 X**	-0.4087	-0.5526 to -0.2647	Yes
19832	19:**0 X** vs. 50:**50 X**	-0.1553	-0.2993 to -0.01137	Yes
19833	19:**1 X** vs. 19:**10 X**	0.002333	-0.1416 to 0.1463	No
19834	19:**1 X** vs. 19:**40 X**	0.1443	0.0003735 to 0.2883	Yes
19835	19:**1 X** vs. 19:**50 X**	0.1333	-0.01063 to 0.2773	No
19836	19:**1 X** vs. 20:**0 X**	-0.09167	-0.2356 to 0.05229	No
19837	19:**1 X** vs. 20:**1 X**	0.08900	-0.05496 to 0.2330	No
19838	19:**1 X** vs. 20:**10 X**	0.0	-0.1440 to 0.1440	No
19839	19:**1 X** vs. 20:**40 X**	0.1327	-0.01129 to 0.2766	No
19840	19:**1 X** vs. 20:**50 X**	0.08867	-0.05529 to 0.2326	No
19841	19:**1 X** vs. 21:**0 X**	-0.1150	-0.2590 to 0.02896	No
19842	19:**1 X** vs. 21:**1 X**	-0.0680	-0.2120 to 0.07596	No
19843	19:**1 X** vs. 21:**10 X**	-0.06500	-0.2090 to 0.07896	No
19844	19:**1 X** vs. 21:**40 X**	0.05967	-0.08429 to 0.2036	No
19845	19:**1 X** vs. 21:**50 X**	-0.03067	-0.1746 to 0.1133	No

2way ANOVA<br>Multiple comparisons		A<br>Data Set-A<br>Y	B<br>Data Set-B<br>Y	C<br>Data Set-C<br>Y
19846	19:**1 X** vs. 22:**0 X**	-0.1567	-0.3006 to -0.01271	Yes
19847	19:**1 X** vs. 22:**1 X**	-0.1513	-0.2953 to -0.007374	Yes
19848	19:**1 X** vs. 22:**10 X**	-0.1467	-0.2906 to -0.002707	Yes
19849	19:**1 X** vs. 22:**40 X**	-0.04700	-0.1910 to 0.09696	No
19850	19:**1 X** vs. 22:**50 X**	-0.1097	-0.2536 to 0.03429	No
19851	19:**1 X** vs. 23:**0 X**	-0.2220	-0.3660 to -0.07804	Yes
19852	19:**1 X** vs. 23:**1 X**	-0.4117	-0.5726 to -0.2507	Yes
19853	19:**1 X** vs. 23:**10 X**	-0.1613	-0.3053 to -0.01737	Yes
19854	19:**1 X** vs. 23:**40 X**	-0.04167	-0.2026 to 0.1193	No
19855	19:**1 X** vs. 23:**50 X**	-0.07567	-0.2196 to 0.06829	No
19856	19:**1 X** vs. 24:**0 X**	-0.3393	-0.4833 to -0.1954	Yes
19857	19:**1 X** vs. 24:**1 X**	-0.4957	-0.6566 to -0.3347	Yes
19858	19:**1 X** vs. 24:**10 X**	-0.3232	-0.4841 to -0.1622	Yes
19859	19:**1 X** vs. 24:**40 X**	-0.05800	-0.2020 to 0.08596	No
19860	19:**1 X** vs. 24:**50 X**	0.02033	-0.1236 to 0.1643	No
19861	19:**1 X** vs. 25:**0 X**	-0.6767	-0.8376 to -0.5157	Yes
19862	19:**1 X** vs. 25:**1 X**	-0.8662	-1.027 to -0.7052	Yes
19863	19:**1 X** vs. 25:**10 X**	-0.2277	-0.4313 to -0.02408	Yes
19864	19:**1 X** vs. 25:**40 X**	-0.0600	-0.2040 to 0.08396	No
19865	19:**1 X** vs. 25:**50 X**	-0.03433	-0.1783 to 0.1096	No
19866	19:**1 X** vs. 26:**0 X**	-0.8892	-1.050 to -0.7282	Yes
19867	19:**1 X** vs. 26:**1 X**	-1.002	-1.163 to -0.8412	Yes
19868	19:**1 X** vs. 26:**10 X**	-0.6887	-0.8496 to -0.5277	Yes
19869	19:**1 X** vs. 26:**40 X**	-0.1343	-0.2783 to 0.009626	No
19870	19:**1 X** vs. 26:**50 X**	0.005333	-0.1386 to 0.1493	No
19871	19:**1 X** vs. 27:**0 X**	-1.077	-1.221 to -0.9330	Yes
19872	19:**1 X** vs. 27:**1 X**	-1.168	-1.329 to -1.007	Yes
19873	19:**1 X** vs. 27:**10 X**	-0.9797	-1.141 to -0.8187	Yes
19874	19:**1 X** vs. 27:**40 X**	-0.1343	-0.2783 to 0.009626	No
19875	19:**1 X** vs. 27:**50 X**	0.006000	-0.1380 to 0.1500	No
19876	19:**1 X** vs. 28:**0 X**	-1.026	-1.187 to -0.8652	Yes
19877	19:**1 X** vs. 28:**1 X**	-1.067	-1.211 to -0.9230	Yes
19878	19:**1 X** vs. 28:**10 X**	-0.8687	-1.030 to -0.7077	Yes
19879	19:**1 X** vs. 28:**40 X**	-0.1467	-0.2906 to -0.002707	Yes
19880	19:**1 X** vs. 28:**50 X**	-0.0420	-0.1860 to 0.1020	No
19881	19:**1 X** vs. 29:**0 X**	-1.508	-1.669 to -1.347	Yes
19882	19:**1 X** vs. 29:**1 X**	-1.593	-1.754 to -1.432	Yes
19883	19:**1 X** vs. 29:**10 X**	-0.8072	-0.9681 to -0.6462	Yes
19884	19:**1 X** vs. 29:**40 X**	-0.2547	-0.3986 to -0.1107	Yes
19885	19:**1 X** vs. 29:**50 X**	-0.04333	-0.1873 to 0.1006	No
19886	19:**1 X** vs. 30:**0 X**	-1.222	-1.383 to -1.061	Yes
19887	19:**1 X** vs. 30:**1 X**	-1.544	-1.705 to -1.383	Yes
19888	19:**1 X** vs. 30:**10 X**	-0.8817	-1.043 to -0.7207	Yes
19889	19:**1 X** vs. 30:**40 X**	-0.1817	-0.3256 to -0.03771	Yes
19890	19:**1 X** vs. 30:**50 X**	-0.09567	-0.2396 to 0.04829	No

2way ANOVA<br>Multiple comparisons	A<br>Data Set-A<br>Y	B<br>Data Set-B<br>Y	C<br>Data Set-C<br>Y
19891 19:**1 X** vs. 31:**0 X**	-1.540	-1.701 to -1.379	Yes
19892 19:**1 X** vs. 31:**1 X**	-1.531	-1.692 to -1.370	Yes
19893 19:**1 X** vs. 31:**10 X**	-1.013	-1.174 to -0.8522	Yes
19894 19:**1 X** vs. 31:**40 X**	-0.2733	-0.4173 to -0.1294	Yes
19895 19:**1 X** vs. 31:**50 X**	-0.07467	-0.2186 to 0.06929	No
19896 19:**1 X** vs. 32:**0 X**	-2.132	-2.276 to -1.988	Yes
19897 19:**1 X** vs. 32:**1 X**	-1.588	-1.749 to -1.427	Yes
19898 19:**1 X** vs. 32:**10 X**	-1.444	-1.588 to -1.300	Yes
19899 19:**1 X** vs. 32:**40 X**	-0.2697	-0.4136 to -0.1257	Yes
19900 19:**1 X** vs. 32:**50 X**	-0.07433	-0.2183 to 0.06963	No
19901 19:**1 X** vs. 33:**0 X**	-2.135	-2.279 to -1.991	Yes
19902 19:**1 X** vs. 33:**1 X**	-1.950	-2.094 to -1.806	Yes
19903 19:**1 X** vs. 33:**10 X**	-1.610	-1.754 to -1.466	Yes
19904 19:**1 X** vs. 33:**40 X**	-0.2883	-0.4323 to -0.1444	Yes
19905 19:**1 X** vs. 33:**50 X**	-0.08933	-0.2333 to 0.05463	No
19906 19:**1 X** vs. 34:**0 X**	-2.060	-2.204 to -1.916	Yes
19907 19:**1 X** vs. 34:**1 X**	-2.067	-2.211 to -1.923	Yes
19908 19:**1 X** vs. 34:**10 X**	-1.701	-1.845 to -1.557	Yes
19909 19:**1 X** vs. 34:**40 X**	-0.3003	-0.4443 to -0.1564	Yes
19910 19:**1 X** vs. 34:**50 X**	-0.2383	-0.3823 to -0.09437	Yes
19911 19:**1 X** vs. 35:**0 X**	-2.409	-2.553 to -2.265	Yes
19912 19:**1 X** vs. 35:**1 X**	-2.174	-2.318 to -2.030	Yes
19913 19:**1 X** vs. 35:**10 X**	-1.662	-1.806 to -1.518	Yes
19914 19:**1 X** vs. 35:**40 X**	-0.3093	-0.4533 to -0.1654	Yes
19915 19:**1 X** vs. 35:**50 X**	-0.2510	-0.3950 to -0.1070	Yes
19916 19:**1 X** vs. 36:**0 X**	-2.615	-2.776 to -2.454	Yes
19917 19:**1 X** vs. 36:**1 X**	-2.311	-2.455 to -2.167	Yes
19918 19:**1 X** vs. 36:**10 X**	-1.668	-1.812 to -1.524	Yes
19919 19:**1 X** vs. 36:**40 X**	-0.3647	-0.5086 to -0.2207	Yes
19920 19:**1 X** vs. 36:**50 X**	-0.2143	-0.3583 to -0.07037	Yes
19921 19:**1 X** vs. 37:**0 X**	-2.999	-3.143 to -2.855	Yes
19922 19:**1 X** vs. 37:**1 X**	-2.381	-2.525 to -2.237	Yes
19923 19:**1 X** vs. 37:**10 X**	-1.670	-1.814 to -1.526	Yes
19924 19:**1 X** vs. 37:**40 X**	-0.4020	-0.5460 to -0.2580	Yes
19925 19:**1 X** vs. 37:**50 X**	-0.2230	-0.3670 to -0.07904	Yes
19926 19:**1 X** vs. 38:**0 X**	-2.944	-3.088 to -2.800	Yes
19927 19:**1 X** vs. 38:**1 X**	-2.500	-2.644 to -2.356	Yes
19928 19:**1 X** vs. 38:**10 X**	-1.945	-2.089 to -1.801	Yes
19929 19:**1 X** vs. 38:**40 X**	-0.4063	-0.5503 to -0.2624	Yes
19930 19:**1 X** vs. 38:**50 X**	-0.2270	-0.3710 to -0.08304	Yes
19931 19:**1 X** vs. 39:**0 X**	-3.175	-3.319 to -3.031	Yes
19932 19:**1 X** vs. 39:**1 X**	-3.066	-3.210 to -2.922	Yes
19933 19:**1 X** vs. 39:**10 X**	-2.090	-2.234 to -1.946	Yes
19934 19:**1 X** vs. 39:**40 X**	-0.4123	-0.5563 to -0.2684	Yes
19935 19:**1 X** vs. 39:**50 X**	-0.2580	-0.4020 to -0.1140	Yes

2way ANOVA<br>Multiple comparisons		A<br>Data Set-A<br>Y	B<br>Data Set-B<br>Y	C<br>Data Set-C<br>Y
19936	19:**1 X** vs. 40:**0 X**	-3.216	-3.360 to -3.072	Yes
19937	19:**1 X** vs. 40:**1 X**	-3.142	-3.286 to -2.998	Yes
19938	19:**1 X** vs. 40:**10 X**	-2.346	-2.490 to -2.202	Yes
19939	19:**1 X** vs. 40:**40 X**	-0.4170	-0.5610 to -0.2730	Yes
19940	19:**1 X** vs. 40:**50 X**	-0.2643	-0.4083 to -0.1204	Yes
19941	19:**1 X** vs. 41:**0 X**	-3.257	-3.401 to -3.113	Yes
19942	19:**1 X** vs. 41:**1 X**	-3.256	-3.400 to -3.112	Yes
19943	19:**1 X** vs. 41:**10 X**	-2.400	-2.544 to -2.256	Yes
19944	19:**1 X** vs. 41:**40 X**	-0.3643	-0.5083 to -0.2204	Yes
19945	19:**1 X** vs. 41:**50 X**	-0.2770	-0.4210 to -0.1330	Yes
19946	19:**1 X** vs. 42:**0 X**	-3.398	-3.542 to -3.254	Yes
19947	19:**1 X** vs. 42:**1 X**	-3.418	-3.562 to -3.274	Yes
19948	19:**1 X** vs. 42:**10 X**	-2.400	-2.544 to -2.256	Yes
19949	19:**1 X** vs. 42:**40 X**	-0.3770	-0.5210 to -0.2330	Yes
19950	19:**1 X** vs. 42:**50 X**	-0.2803	-0.4243 to -0.1364	Yes
19951	19:**1 X** vs. 43:**0 X**	-3.488	-3.632 to -3.344	Yes
19952	19:**1 X** vs. 43:**1 X**	-3.403	-3.547 to -3.259	Yes
19953	19:**1 X** vs. 43:**10 X**	-3.118	-3.262 to -2.974	Yes
19954	19:**1 X** vs. 43:**40 X**	-0.3753	-0.5193 to -0.2314	Yes
19955	19:**1 X** vs. 43:**50 X**	-0.2807	-0.4246 to -0.1367	Yes
19956	19:**1 X** vs. 44:**0 X**	-3.512	-3.656 to -3.368	Yes
19957	19:**1 X** vs. 44:**1 X**	-3.402	-3.546 to -3.258	Yes
19958	19:**1 X** vs. 44:**10 X**	-3.182	-3.326 to -3.038	Yes
19959	19:**1 X** vs. 44:**40 X**	-0.3773	-0.5213 to -0.2334	Yes
19960	19:**1 X** vs. 44:**50 X**	-0.2130	-0.3570 to -0.06904	Yes
19961	19:**1 X** vs. 45:**0 X**	-3.377	-3.521 to -3.233	Yes
19962	19:**1 X** vs. 45:**1 X**	-3.179	-3.323 to -3.035	Yes
19963	19:**1 X** vs. 45:**10 X**	-3.061	-3.205 to -2.917	Yes
19964	19:**1 X** vs. 45:**40 X**	-0.3787	-0.5226 to -0.2347	Yes
19965	19:**1 X** vs. 45:**50 X**	-0.2100	-0.3540 to -0.06604	Yes
19966	19:**1 X** vs. 46:**0 X**	-3.181	-3.325 to -3.037	Yes
19967	19:**1 X** vs. 46:**1 X**	-3.148	-3.292 to -3.004	Yes
19968	19:**1 X** vs. 46:**10 X**	-3.056	-3.200 to -2.912	Yes
19969	19:**1 X** vs. 46:**40 X**	-0.3923	-0.5363 to -0.2484	Yes
19970	19:**1 X** vs. 46:**50 X**	-0.2030	-0.3470 to -0.05904	Yes
19971	19:**1 X** vs. 47:**0 X**	-3.157	-3.301 to -3.013	Yes
19972	19:**1 X** vs. 47:**1 X**	-3.061	-3.205 to -2.917	Yes
19973	19:**1 X** vs. 47:**10 X**	-3.042	-3.186 to -2.898	Yes
19974	19:**1 X** vs. 47:**40 X**	-0.4023	-0.5463 to -0.2584	Yes
19975	19:**1 X** vs. 47:**50 X**	-0.1610	-0.3050 to -0.01704	Yes
19976	19:**1 X** vs. 48:**0 X**	-3.144	-3.305 to -2.983	Yes
19977	19:**1 X** vs. 48:**1 X**	-3.048	-3.192 to -2.904	Yes
19978	19:**1 X** vs. 48:**10 X**	-3.042	-3.186 to -2.898	Yes
19979	19:**1 X** vs. 48:**40 X**	-0.4087	-0.5526 to -0.2647	Yes
19980	19:**1 X** vs. 48:**50 X**	-0.1683	-0.3123 to -0.02437	Yes

	2way ANOVA<br>Multiple comparisons	A<br>Data Set-A<br>Y	B<br>Data Set-B<br>Y	C<br>Data Set-C<br>Y
19981	19:1 X vs. 49:0 X	-3.144	-3.288 to -3.000	Yes
19982	19:1 X vs. 49:1 X	-3.048	-3.192 to -2.904	Yes
19983	19:1 X vs. 49:10 X	-3.042	-3.186 to -2.898	Yes
19984	19:1 X vs. 49:40 X	-0.4130	-0.5570 to -0.2690	Yes
19985	19:1 X vs. 49:50 X	-0.1677	-0.3116 to -0.02371	Yes
19986	19:1 X vs. 50:0 X	-3.144	-3.288 to -3.000	Yes
19987	19:1 X vs. 50:1 X	-3.048	-3.192 to -2.904	Yes
19988	19:1 X vs. 50:10 X	-3.042	-3.186 to -2.898	Yes
19989	19:1 X vs. 50:40 X	-0.4207	-0.5646 to -0.2767	Yes
19990	19:1 X vs. 50:50 X	-0.1673	-0.3113 to -0.02337	Yes
19991	19:10 X vs. 19:40 X	0.1420	-0.001960 to 0.2860	No
19992	19:10 X vs. 19:50 X	0.1310	-0.01296 to 0.2750	No
19993	19:10 X vs. 20:0 X	-0.0940	-0.2380 to 0.04996	No
19994	19:10 X vs. 20:1 X	0.08667	-0.05729 to 0.2306	No
19995	19:10 X vs. 20:10 X	-0.002333	-0.1463 to 0.1416	No
19996	19:10 X vs. 20:40 X	0.1303	-0.01363 to 0.2743	No
19997	19:10 X vs. 20:50 X	0.08633	-0.05763 to 0.2303	No
19998	19:10 X vs. 21:0 X	-0.1173	-0.2613 to 0.02663	No
19999	19:10 X vs. 21:1 X	-0.07033	-0.2143 to 0.07363	No
20000	19:10 X vs. 21:10 X	-0.06733	-0.2113 to 0.07663	No
20001	19:10 X vs. 21:40 X	0.05733	-0.08663 to 0.2013	No
20002	19:10 X vs. 21:50 X	-0.0330	-0.1770 to 0.1110	No
20003	19:10 X vs. 22:0 X	-0.1590	-0.3030 to -0.01504	Yes
20004	19:10 X vs. 22:1 X	-0.1537	-0.2976 to -0.009707	Yes
20005	19:10 X vs. 22:10 X	-0.1490	-0.2930 to -0.005040	Yes
20006	19:10 X vs. 22:40 X	-0.04933	-0.1933 to 0.09463	No
20007	19:10 X vs. 22:50 X	-0.1120	-0.2560 to 0.03196	No
20008	19:10 X vs. 23:0 X	-0.2243	-0.3683 to -0.08037	Yes
20009	19:10 X vs. 23:1 X	-0.4140	-0.5750 to -0.2530	Yes
20010	19:10 X vs. 23:10 X	-0.1637	-0.3076 to -0.01971	Yes
20011	19:10 X vs. 23:40 X	-0.04400	-0.2050 to 0.1170	No
20012	19:10 X vs. 23:50 X	-0.0780	-0.2220 to 0.06596	No
20013	19:10 X vs. 24:0 X	-0.3417	-0.4856 to -0.1977	Yes
20014	19:10 X vs. 24:1 X	-0.4980	-0.6590 to -0.3370	Yes
20015	19:10 X vs. 24:10 X	-0.3255	-0.4865 to -0.1645	Yes
20016	19:10 X vs. 24:40 X	-0.06033	-0.2043 to 0.08363	No
20017	19:10 X vs. 24:50 X	0.0180	-0.1260 to 0.1620	No
20018	19:10 X vs. 25:0 X	-0.6790	-0.8400 to -0.5180	Yes
20019	19:10 X vs. 25:1 X	-0.8685	-1.029 to -0.7075	Yes
20020	19:10 X vs. 25:10 X	-0.2300	-0.4336 to -0.02641	Yes
20021	19:10 X vs. 25:40 X	-0.06233	-0.2063 to 0.08163	No
20022	19:10 X vs. 25:50 X	-0.03667	-0.1806 to 0.1073	No
20023	19:10 X vs. 26:0 X	-0.8915	-1.052 to -0.7305	Yes
20024	19:10 X vs. 26:1 X	-1.005	-1.165 to -0.8435	Yes
20025	19:10 X vs. 26:10 X	-0.6910	-0.8520 to -0.5300	Yes

2way ANOVA<br>Multiple comparisons		A<br>Data Set-A<br>Y	B<br>Data Set-B<br>Y	C<br>Data Set-C<br>Y
20026	19:**10 X** vs. 26:**40 X**	-0.1367	-0.2806 to 0.007293	No
20027	19:**10 X** vs. 26:**50 X**	0.003000	-0.1410 to 0.1470	No
20028	19:**10 X** vs. 27:**0 X**	-1.079	-1.223 to -0.9354	Yes
20029	19:**10 X** vs. 27:**1 X**	-1.170	-1.331 to -1.009	Yes
20030	19:**10 X** vs. 27:**10 X**	-0.9820	-1.143 to -0.8210	Yes
20031	19:**10 X** vs. 27:**40 X**	-0.1367	-0.2806 to 0.007293	No
20032	19:**10 X** vs. 27:**50 X**	0.003667	-0.1403 to 0.1476	No
20033	19:**10 X** vs. 28:**0 X**	-1.029	-1.189 to -0.8675	Yes
20034	19:**10 X** vs. 28:**1 X**	-1.069	-1.213 to -0.9254	Yes
20035	19:**10 X** vs. 28:**10 X**	-0.8710	-1.032 to -0.7100	Yes
20036	19:**10 X** vs. 28:**40 X**	-0.1490	-0.2930 to -0.005040	Yes
20037	19:**10 X** vs. 28:**50 X**	-0.04433	-0.1883 to 0.09963	No
20038	19:**10 X** vs. 29:**0 X**	-1.510	-1.671 to -1.349	Yes
20039	19:**10 X** vs. 29:**1 X**	-1.595	-1.756 to -1.434	Yes
20040	19:**10 X** vs. 29:**10 X**	-0.8095	-0.9705 to -0.6485	Yes
20041	19:**10 X** vs. 29:**40 X**	-0.2570	-0.4010 to -0.1130	Yes
20042	19:**10 X** vs. 29:**50 X**	-0.04567	-0.1896 to 0.09829	No
20043	19:**10 X** vs. 30:**0 X**	-1.225	-1.385 to -1.064	Yes
20044	19:**10 X** vs. 30:**1 X**	-1.546	-1.707 to -1.385	Yes
20045	19:**10 X** vs. 30:**10 X**	-0.8840	-1.045 to -0.7230	Yes
20046	19:**10 X** vs. 30:**40 X**	-0.1840	-0.3280 to -0.04004	Yes
20047	19:**10 X** vs. 30:**50 X**	-0.09800	-0.2420 to 0.04596	No
20048	19:**10 X** vs. 31:**0 X**	-1.543	-1.703 to -1.382	Yes
20049	19:**10 X** vs. 31:**1 X**	-1.533	-1.694 to -1.372	Yes
20050	19:**10 X** vs. 31:**10 X**	-1.016	-1.176 to -0.8545	Yes
20051	19:**10 X** vs. 31:**40 X**	-0.2757	-0.4196 to -0.1317	Yes
20052	19:**10 X** vs. 31:**50 X**	-0.0770	-0.2210 to 0.06696	No
20053	19:**10 X** vs. 32:**0 X**	-2.134	-2.278 to -1.990	Yes
20054	19:**10 X** vs. 32:**1 X**	-1.590	-1.751 to -1.429	Yes
20055	19:**10 X** vs. 32:**10 X**	-1.446	-1.590 to -1.302	Yes
20056	19:**10 X** vs. 32:**40 X**	-0.2720	-0.4160 to -0.1280	Yes
20057	19:**10 X** vs. 32:**50 X**	-0.07667	-0.2206 to 0.06729	No
20058	19:**10 X** vs. 33:**0 X**	-2.138	-2.282 to -1.994	Yes
20059	19:**10 X** vs. 33:**1 X**	-1.952	-2.096 to -1.808	Yes
20060	19:**10 X** vs. 33:**10 X**	-1.613	-1.757 to -1.469	Yes
20061	19:**10 X** vs. 33:**40 X**	-0.2907	-0.4346 to -0.1467	Yes
20062	19:**10 X** vs. 33:**50 X**	-0.09167	-0.2356 to 0.05229	No
20063	19:**10 X** vs. 34:**0 X**	-2.062	-2.206 to -1.918	Yes
20064	19:**10 X** vs. 34:**1 X**	-2.070	-2.214 to -1.926	Yes
20065	19:**10 X** vs. 34:**10 X**	-1.703	-1.847 to -1.559	Yes
20066	19:**10 X** vs. 34:**40 X**	-0.3027	-0.4466 to -0.1587	Yes
20067	19:**10 X** vs. 34:**50 X**	-0.2407	-0.3846 to -0.09671	Yes
20068	19:**10 X** vs. 35:**0 X**	-2.411	-2.555 to -2.267	Yes
20069	19:**10 X** vs. 35:**1 X**	-2.177	-2.321 to -2.033	Yes
20070	19:**10 X** vs. 35:**10 X**	-1.665	-1.809 to -1.521	Yes

	2way ANOVA<br>Multiple comparisons	A<br>Data Set-A<br>Y	B<br>Data Set-B<br>Y	C<br>Data Set-C<br>Y
20071	19:**10 X** vs. 35:**40 X**	-0.3117	-0.4556 to -0.1677	Yes
20072	19:**10 X** vs. 35:**50 X**	-0.2533	-0.3973 to -0.1094	Yes
20073	19:**10 X** vs. 36:**0 X**	-2.618	-2.778 to -2.457	Yes
20074	19:**10 X** vs. 36:**1 X**	-2.313	-2.457 to -2.169	Yes
20075	19:**10 X** vs. 36:**10 X**	-1.670	-1.814 to -1.526	Yes
20076	19:**10 X** vs. 36:**40 X**	-0.3670	-0.5110 to -0.2230	Yes
20077	19:**10 X** vs. 36:**50 X**	-0.2167	-0.3606 to -0.07271	Yes
20078	19:**10 X** vs. 37:**0 X**	-3.001	-3.145 to -2.857	Yes
20079	19:**10 X** vs. 37:**1 X**	-2.383	-2.527 to -2.239	Yes
20080	19:**10 X** vs. 37:**10 X**	-1.672	-1.816 to -1.528	Yes
20081	19:**10 X** vs. 37:**40 X**	-0.4043	-0.5483 to -0.2604	Yes
20082	19:**10 X** vs. 37:**50 X**	-0.2253	-0.3693 to -0.08137	Yes
20083	19:**10 X** vs. 38:**0 X**	-2.946	-3.090 to -2.802	Yes
20084	19:**10 X** vs. 38:**1 X**	-2.502	-2.646 to -2.358	Yes
20085	19:**10 X** vs. 38:**10 X**	-1.947	-2.091 to -1.803	Yes
20086	19:**10 X** vs. 38:**40 X**	-0.4087	-0.5526 to -0.2647	Yes
20087	19:**10 X** vs. 38:**50 X**	-0.2293	-0.3733 to -0.08537	Yes
20088	19:**10 X** vs. 39:**0 X**	-3.177	-3.321 to -3.033	Yes
20089	19:**10 X** vs. 39:**1 X**	-3.069	-3.213 to -2.925	Yes
20090	19:**10 X** vs. 39:**10 X**	-2.092	-2.236 to -1.948	Yes
20091	19:**10 X** vs. 39:**40 X**	-0.4147	-0.5586 to -0.2707	Yes
20092	19:**10 X** vs. 39:**50 X**	-0.2603	-0.4043 to -0.1164	Yes
20093	19:**10 X** vs. 40:**0 X**	-3.218	-3.362 to -3.074	Yes
20094	19:**10 X** vs. 40:**1 X**	-3.144	-3.288 to -3.000	Yes
20095	19:**10 X** vs. 40:**10 X**	-2.349	-2.493 to -2.205	Yes
20096	19:**10 X** vs. 40:**40 X**	-0.4193	-0.5633 to -0.2754	Yes
20097	19:**10 X** vs. 40:**50 X**	-0.2667	-0.4106 to -0.1227	Yes
20098	19:**10 X** vs. 41:**0 X**	-3.259	-3.403 to -3.115	Yes
20099	19:**10 X** vs. 41:**1 X**	-3.258	-3.402 to -3.114	Yes
20100	19:**10 X** vs. 41:**10 X**	-2.402	-2.546 to -2.258	Yes
20101	19:**10 X** vs. 41:**40 X**	-0.3667	-0.5106 to -0.2227	Yes
20102	19:**10 X** vs. 41:**50 X**	-0.2793	-0.4233 to -0.1354	Yes
20103	19:**10 X** vs. 42:**0 X**	-3.401	-3.545 to -3.257	Yes
20104	19:**10 X** vs. 42:**1 X**	-3.420	-3.564 to -3.276	Yes
20105	19:**10 X** vs. 42:**10 X**	-2.403	-2.547 to -2.259	Yes
20106	19:**10 X** vs. 42:**40 X**	-0.3793	-0.5233 to -0.2354	Yes
20107	19:**10 X** vs. 42:**50 X**	-0.2827	-0.4266 to -0.1387	Yes
20108	19:**10 X** vs. 43:**0 X**	-3.491	-3.635 to -3.347	Yes
20109	19:**10 X** vs. 43:**1 X**	-3.405	-3.549 to -3.261	Yes
20110	19:**10 X** vs. 43:**10 X**	-3.121	-3.265 to -2.977	Yes
20111	19:**10 X** vs. 43:**40 X**	-0.3777	-0.5216 to -0.2337	Yes
20112	19:**10 X** vs. 43:**50 X**	-0.2830	-0.4270 to -0.1390	Yes
20113	19:**10 X** vs. 44:**0 X**	-3.515	-3.659 to -3.371	Yes
20114	19:**10 X** vs. 44:**1 X**	-3.404	-3.548 to -3.260	Yes
20115	19:**10 X** vs. 44:**10 X**	-3.184	-3.328 to -3.040	Yes

2way ANOVA<br>Multiple comparisons	A<br>Data Set-A<br>Y	B<br>Data Set-B<br>Y	C<br>Data Set-C<br>Y
20116 19:10 X vs. 44:40 X	-0.3797	-0.5236 to -0.2357	Yes
20117 19:10 X vs. 44:50 X	-0.2153	-0.3593 to -0.07137	Yes
20118 19:10 X vs. 45:0 X	-3.379	-3.523 to -3.235	Yes
20119 19:10 X vs. 45:1 X	-3.181	-3.325 to -3.037	Yes
20120 19:10 X vs. 45:10 X	-3.064	-3.208 to -2.920	Yes
20121 19:10 X vs. 45:40 X	-0.3810	-0.5250 to -0.2370	Yes
20122 19:10 X vs. 45:50 X	-0.2123	-0.3563 to -0.06837	Yes
20123 19:10 X vs. 46:0 X	-3.184	-3.328 to -3.040	Yes
20124 19:10 X vs. 46:1 X	-3.151	-3.295 to -3.007	Yes
20125 19:10 X vs. 46:10 X	-3.059	-3.203 to -2.915	Yes
20126 19:10 X vs. 46:40 X	-0.3947	-0.5386 to -0.2507	Yes
20127 19:10 X vs. 46:50 X	-0.2053	-0.3493 to -0.06137	Yes
20128 19:10 X vs. 47:0 X	-3.159	-3.303 to -3.015	Yes
20129 19:10 X vs. 47:1 X	-3.063	-3.207 to -2.919	Yes
20130 19:10 X vs. 47:10 X	-3.045	-3.189 to -2.901	Yes
20131 19:10 X vs. 47:40 X	-0.4047	-0.5486 to -0.2607	Yes
20132 19:10 X vs. 47:50 X	-0.1633	-0.3073 to -0.01937	Yes
20133 19:10 X vs. 48:0 X	-3.147	-3.307 to -2.986	Yes
20134 19:10 X vs. 48:1 X	-3.050	-3.194 to -2.906	Yes
20135 19:10 X vs. 48:10 X	-3.045	-3.189 to -2.901	Yes
20136 19:10 X vs. 48:40 X	-0.4110	-0.5550 to -0.2670	Yes
20137 19:10 X vs. 48:50 X	-0.1707	-0.3146 to -0.02671	Yes
20138 19:10 X vs. 49:0 X	-3.147	-3.291 to -3.003	Yes
20139 19:10 X vs. 49:1 X	-3.050	-3.194 to -2.906	Yes
20140 19:10 X vs. 49:10 X	-3.045	-3.189 to -2.901	Yes
20141 19:10 X vs. 49:40 X	-0.4153	-0.5593 to -0.2714	Yes
20142 19:10 X vs. 49:50 X	-0.1700	-0.3140 to -0.02604	Yes
20143 19:10 X vs. 50:0 X	-3.147	-3.291 to -3.003	Yes
20144 19:10 X vs. 50:1 X	-3.050	-3.194 to -2.906	Yes
20145 19:10 X vs. 50:10 X	-3.045	-3.189 to -2.901	Yes
20146 19:10 X vs. 50:40 X	-0.4230	-0.5670 to -0.2790	Yes
20147 19:10 X vs. 50:50 X	-0.1697	-0.3136 to -0.02571	Yes
20148 19:40 X vs. 19:50 X	-0.0110	-0.1550 to 0.1330	No
20149 19:40 X vs. 20:0 X	-0.2360	-0.3800 to -0.09204	Yes
20150 19:40 X vs. 20:1 X	-0.05533	-0.1993 to 0.08863	No
20151 19:40 X vs. 20:10 X	-0.1443	-0.2883 to -0.0003735	Yes
20152 19:40 X vs. 20:40 X	-0.01167	-0.1556 to 0.1323	No
20153 19:40 X vs. 20:50 X	-0.05567	-0.1996 to 0.08829	No
20154 19:40 X vs. 21:0 X	-0.2593	-0.4033 to -0.1154	Yes
20155 19:40 X vs. 21:1 X	-0.2123	-0.3563 to -0.06837	Yes
20156 19:40 X vs. 21:10 X	-0.2093	-0.3533 to -0.06537	Yes
20157 19:40 X vs. 21:40 X	-0.08467	-0.2286 to 0.05929	No
20158 19:40 X vs. 21:50 X	-0.1750	-0.3190 to -0.03104	Yes
20159 19:40 X vs. 22:0 X	-0.3010	-0.4450 to -0.1570	Yes
20160 19:40 X vs. 22:1 X	-0.2957	-0.4396 to -0.1517	Yes

	2way ANOVA<br>Multiple comparisons	A<br>Data Set-A<br>Y	B<br>Data Set-B<br>Y	C<br>Data Set-C<br>Y
20161	19:40 X vs. 22:10 X	-0.2910	-0.4350 to -0.1470	Yes
20162	19:40 X vs. 22:40 X	-0.1913	-0.3353 to -0.04737	Yes
20163	19:40 X vs. 22:50 X	-0.2540	-0.3980 to -0.1100	Yes
20164	19:40 X vs. 23:0 X	-0.3663	-0.5103 to -0.2224	Yes
20165	19:40 X vs. 23:1 X	-0.5560	-0.7170 to -0.3950	Yes
20166	19:40 X vs. 23:10 X	-0.3057	-0.4496 to -0.1617	Yes
20167	19:40 X vs. 23:40 X	-0.1860	-0.3470 to -0.02505	Yes
20168	19:40 X vs. 23:50 X	-0.2200	-0.3640 to -0.07604	Yes
20169	19:40 X vs. 24:0 X	-0.4837	-0.6276 to -0.3397	Yes
20170	19:40 X vs. 24:1 X	-0.6400	-0.8010 to -0.4790	Yes
20171	19:40 X vs. 24:10 X	-0.4675	-0.6285 to -0.3065	Yes
20172	19:40 X vs. 24:40 X	-0.2023	-0.3463 to -0.05837	Yes
20173	19:40 X vs. 24:50 X	-0.1240	-0.2680 to 0.01996	No
20174	19:40 X vs. 25:0 X	-0.8210	-0.9820 to -0.6600	Yes
20175	19:40 X vs. 25:1 X	-1.011	-1.171 to -0.8495	Yes
20176	19:40 X vs. 25:10 X	-0.3720	-0.5756 to -0.1684	Yes
20177	19:40 X vs. 25:40 X	-0.2043	-0.3483 to -0.06037	Yes
20178	19:40 X vs. 25:50 X	-0.1787	-0.3226 to -0.03471	Yes
20179	19:40 X vs. 26:0 X	-1.034	-1.194 to -0.8725	Yes
20180	19:40 X vs. 26:1 X	-1.147	-1.307 to -0.9855	Yes
20181	19:40 X vs. 26:10 X	-0.8330	-0.9940 to -0.6720	Yes
20182	19:40 X vs. 26:40 X	-0.2787	-0.4226 to -0.1347	Yes
20183	19:40 X vs. 26:50 X	-0.1390	-0.2830 to 0.004960	No
20184	19:40 X vs. 27:0 X	-1.221	-1.365 to -1.077	Yes
20185	19:40 X vs. 27:1 X	-1.312	-1.473 to -1.151	Yes
20186	19:40 X vs. 27:10 X	-1.124	-1.285 to -0.9630	Yes
20187	19:40 X vs. 27:40 X	-0.2787	-0.4226 to -0.1347	Yes
20188	19:40 X vs. 27:50 X	-0.1383	-0.2823 to 0.005626	No
20189	19:40 X vs. 28:0 X	-1.171	-1.331 to -1.010	Yes
20190	19:40 X vs. 28:1 X	-1.211	-1.355 to -1.067	Yes
20191	19:40 X vs. 28:10 X	-1.013	-1.174 to -0.8520	Yes
20192	19:40 X vs. 28:40 X	-0.2910	-0.4350 to -0.1470	Yes
20193	19:40 X vs. 28:50 X	-0.1863	-0.3303 to -0.04237	Yes
20194	19:40 X vs. 29:0 X	-1.652	-1.813 to -1.491	Yes
20195	19:40 X vs. 29:1 X	-1.737	-1.898 to -1.576	Yes
20196	19:40 X vs. 29:10 X	-0.9515	-1.112 to -0.7905	Yes
20197	19:40 X vs. 29:40 X	-0.3990	-0.5430 to -0.2550	Yes
20198	19:40 X vs. 29:50 X	-0.1877	-0.3316 to -0.04371	Yes
20199	19:40 X vs. 30:0 X	-1.367	-1.527 to -1.206	Yes
20200	19:40 X vs. 30:1 X	-1.688	-1.849 to -1.527	Yes
20201	19:40 X vs. 30:10 X	-1.026	-1.187 to -0.8650	Yes
20202	19:40 X vs. 30:40 X	-0.3260	-0.4700 to -0.1820	Yes
20203	19:40 X vs. 30:50 X	-0.2400	-0.3840 to -0.09604	Yes
20204	19:40 X vs. 31:0 X	-1.685	-1.845 to -1.524	Yes
20205	19:40 X vs. 31:1 X	-1.675	-1.836 to -1.514	Yes

	2way ANOVA<br>Multiple comparisons	A<br>Data Set-A<br>Y	B<br>Data Set-B<br>Y	C<br>Data Set-C<br>Y
20206	19:40 X vs. 31:10 X	-1.158	-1.318 to -0.9965	Yes
20207	19:40 X vs. 31:40 X	-0.4177	-0.5616 to -0.2737	Yes
20208	19:40 X vs. 31:50 X	-0.2190	-0.3630 to -0.07504	Yes
20209	19:40 X vs. 32:0 X	-2.276	-2.420 to -2.132	Yes
20210	19:40 X vs. 32:1 X	-1.732	-1.893 to -1.571	Yes
20211	19:40 X vs. 32:10 X	-1.588	-1.732 to -1.444	Yes
20212	19:40 X vs. 32:40 X	-0.4140	-0.5580 to -0.2700	Yes
20213	19:40 X vs. 32:50 X	-0.2187	-0.3626 to -0.07471	Yes
20214	19:40 X vs. 33:0 X	-2.280	-2.424 to -2.136	Yes
20215	19:40 X vs. 33:1 X	-2.094	-2.238 to -1.950	Yes
20216	19:40 X vs. 33:10 X	-1.755	-1.899 to -1.611	Yes
20217	19:40 X vs. 33:40 X	-0.4327	-0.5766 to -0.2887	Yes
20218	19:40 X vs. 33:50 X	-0.2337	-0.3776 to -0.08971	Yes
20219	19:40 X vs. 34:0 X	-2.204	-2.348 to -2.060	Yes
20220	19:40 X vs. 34:1 X	-2.212	-2.356 to -2.068	Yes
20221	19:40 X vs. 34:10 X	-1.845	-1.989 to -1.701	Yes
20222	19:40 X vs. 34:40 X	-0.4447	-0.5886 to -0.3007	Yes
20223	19:40 X vs. 34:50 X	-0.3827	-0.5266 to -0.2387	Yes
20224	19:40 X vs. 35:0 X	-2.553	-2.697 to -2.409	Yes
20225	19:40 X vs. 35:1 X	-2.319	-2.463 to -2.175	Yes
20226	19:40 X vs. 35:10 X	-1.807	-1.951 to -1.663	Yes
20227	19:40 X vs. 35:40 X	-0.4537	-0.5976 to -0.3097	Yes
20228	19:40 X vs. 35:50 X	-0.3953	-0.5393 to -0.2514	Yes
20229	19:40 X vs. 36:0 X	-2.760	-2.920 to -2.599	Yes
20230	19:40 X vs. 36:1 X	-2.455	-2.599 to -2.311	Yes
20231	19:40 X vs. 36:10 X	-1.812	-1.956 to -1.668	Yes
20232	19:40 X vs. 36:40 X	-0.5090	-0.6530 to -0.3650	Yes
20233	19:40 X vs. 36:50 X	-0.3587	-0.5026 to -0.2147	Yes
20234	19:40 X vs. 37:0 X	-3.143	-3.287 to -2.999	Yes
20235	19:40 X vs. 37:1 X	-2.525	-2.669 to -2.381	Yes
20236	19:40 X vs. 37:10 X	-1.814	-1.958 to -1.670	Yes
20237	19:40 X vs. 37:40 X	-0.5463	-0.6903 to -0.4024	Yes
20238	19:40 X vs. 37:50 X	-0.3673	-0.5113 to -0.2234	Yes
20239	19:40 X vs. 38:0 X	-3.088	-3.232 to -2.944	Yes
20240	19:40 X vs. 38:1 X	-2.644	-2.788 to -2.500	Yes
20241	19:40 X vs. 38:10 X	-2.089	-2.233 to -1.945	Yes
20242	19:40 X vs. 38:40 X	-0.5507	-0.6946 to -0.4067	Yes
20243	19:40 X vs. 38:50 X	-0.3713	-0.5153 to -0.2274	Yes
20244	19:40 X vs. 39:0 X	-3.319	-3.463 to -3.175	Yes
20245	19:40 X vs. 39:1 X	-3.211	-3.355 to -3.067	Yes
20246	19:40 X vs. 39:10 X	-2.234	-2.378 to -2.090	Yes
20247	19:40 X vs. 39:40 X	-0.5567	-0.7006 to -0.4127	Yes
20248	19:40 X vs. 39:50 X	-0.4023	-0.5463 to -0.2584	Yes
20249	19:40 X vs. 40:0 X	-3.360	-3.504 to -3.216	Yes
20250	19:40 X vs. 40:1 X	-3.286	-3.430 to -3.142	Yes

	2way ANOVA<br>Multiple comparisons	A<br>Data Set-A<br>Y	B<br>Data Set-B<br>Y	C<br>Data Set-C<br>Y
20251	19:**40 X** vs. 40:**10 X**	-2.491	-2.635 to -2.347	Yes
20252	19:**40 X** vs. 40:**40 X**	-0.5613	-0.7053 to -0.4174	Yes
20253	19:**40 X** vs. 40:**50 X**	-0.4087	-0.5526 to -0.2647	Yes
20254	19:**40 X** vs. 41:**0 X**	-3.401	-3.545 to -3.257	Yes
20255	19:**40 X** vs. 41:**1 X**	-3.400	-3.544 to -3.256	Yes
20256	19:**40 X** vs. 41:**10 X**	-2.544	-2.688 to -2.400	Yes
20257	19:**40 X** vs. 41:**40 X**	-0.5087	-0.6526 to -0.3647	Yes
20258	19:**40 X** vs. 41:**50 X**	-0.4213	-0.5653 to -0.2774	Yes
20259	19:**40 X** vs. 42:**0 X**	-3.543	-3.687 to -3.399	Yes
20260	19:**40 X** vs. 42:**1 X**	-3.562	-3.706 to -3.418	Yes
20261	19:**40 X** vs. 42:**10 X**	-2.545	-2.689 to -2.401	Yes
20262	19:**40 X** vs. 42:**40 X**	-0.5213	-0.6653 to -0.3774	Yes
20263	19:**40 X** vs. 42:**50 X**	-0.4247	-0.5686 to -0.2807	Yes
20264	19:**40 X** vs. 43:**0 X**	-3.633	-3.777 to -3.489	Yes
20265	19:**40 X** vs. 43:**1 X**	-3.547	-3.691 to -3.403	Yes
20266	19:**40 X** vs. 43:**10 X**	-3.263	-3.407 to -3.119	Yes
20267	19:**40 X** vs. 43:**40 X**	-0.5197	-0.6636 to -0.3757	Yes
20268	19:**40 X** vs. 43:**50 X**	-0.4250	-0.5690 to -0.2810	Yes
20269	19:**40 X** vs. 44:**0 X**	-3.657	-3.801 to -3.513	Yes
20270	19:**40 X** vs. 44:**1 X**	-3.546	-3.690 to -3.402	Yes
20271	19:**40 X** vs. 44:**10 X**	-3.326	-3.470 to -3.182	Yes
20272	19:**40 X** vs. 44:**40 X**	-0.5217	-0.6656 to -0.3777	Yes
20273	19:**40 X** vs. 44:**50 X**	-0.3573	-0.5013 to -0.2134	Yes
20274	19:**40 X** vs. 45:**0 X**	-3.521	-3.665 to -3.377	Yes
20275	19:**40 X** vs. 45:**1 X**	-3.323	-3.467 to -3.179	Yes
20276	19:**40 X** vs. 45:**10 X**	-3.206	-3.350 to -3.062	Yes
20277	19:**40 X** vs. 45:**40 X**	-0.5230	-0.6670 to -0.3790	Yes
20278	19:**40 X** vs. 45:**50 X**	-0.3543	-0.4983 to -0.2104	Yes
20279	19:**40 X** vs. 46:**0 X**	-3.326	-3.470 to -3.182	Yes
20280	19:**40 X** vs. 46:**1 X**	-3.293	-3.437 to -3.149	Yes
20281	19:**40 X** vs. 46:**10 X**	-3.201	-3.345 to -3.057	Yes
20282	19:**40 X** vs. 46:**40 X**	-0.5367	-0.6806 to -0.3927	Yes
20283	19:**40 X** vs. 46:**50 X**	-0.3473	-0.4913 to -0.2034	Yes
20284	19:**40 X** vs. 47:**0 X**	-3.301	-3.445 to -3.157	Yes
20285	19:**40 X** vs. 47:**1 X**	-3.205	-3.349 to -3.061	Yes
20286	19:**40 X** vs. 47:**10 X**	-3.187	-3.331 to -3.043	Yes
20287	19:**40 X** vs. 47:**40 X**	-0.5467	-0.6906 to -0.4027	Yes
20288	19:**40 X** vs. 47:**50 X**	-0.3053	-0.4493 to -0.1614	Yes
20289	19:**40 X** vs. 48:**0 X**	-3.289	-3.449 to -3.128	Yes
20290	19:**40 X** vs. 48:**1 X**	-3.192	-3.336 to -3.048	Yes
20291	19:**40 X** vs. 48:**10 X**	-3.187	-3.331 to -3.043	Yes
20292	19:**40 X** vs. 48:**40 X**	-0.5530	-0.6970 to -0.4090	Yes
20293	19:**40 X** vs. 48:**50 X**	-0.3127	-0.4566 to -0.1687	Yes
20294	19:**40 X** vs. 49:**0 X**	-3.289	-3.433 to -3.145	Yes
20295	19:**40 X** vs. 49:**1 X**	-3.192	-3.336 to -3.048	Yes

2way ANOVA<br>Multiple comparisons	A<br>Data Set-A<br>Y	B<br>Data Set-B<br>Y	C<br>Data Set-C<br>Y
20296 19:40 X vs. 49:10 X	-3.187	-3.331 to -3.043	Yes
20297 19:40 X vs. 49:40 X	-0.5573	-0.7013 to -0.4134	Yes
20298 19:40 X vs. 49:50 X	-0.3120	-0.4560 to -0.1680	Yes
20299 19:40 X vs. 50:0 X	-3.289	-3.433 to -3.145	Yes
20300 19:40 X vs. 50:1 X	-3.192	-3.336 to -3.048	Yes
20301 19:40 X vs. 50:10 X	-3.187	-3.331 to -3.043	Yes
20302 19:40 X vs. 50:40 X	-0.5650	-0.7090 to -0.4210	Yes
20303 19:40 X vs. 50:50 X	-0.3117	-0.4556 to -0.1677	Yes
20304 19:50 X vs. 20:0 X	-0.2250	-0.3690 to -0.08104	Yes
20305 19:50 X vs. 20:1 X	-0.04433	-0.1883 to 0.09963	No
20306 19:50 X vs. 20:10 X	-0.1333	-0.2773 to 0.01063	No
20307 19:50 X vs. 20:40 X	-0.0006667	-0.1446 to 0.1433	No
20308 19:50 X vs. 20:50 X	-0.04467	-0.1886 to 0.09929	No
20309 19:50 X vs. 21:0 X	-0.2483	-0.3923 to -0.1044	Yes
20310 19:50 X vs. 21:1 X	-0.2013	-0.3453 to -0.05737	Yes
20311 19:50 X vs. 21:10 X	-0.1983	-0.3423 to -0.05437	Yes
20312 19:50 X vs. 21:40 X	-0.07367	-0.2176 to 0.07029	No
20313 19:50 X vs. 21:50 X	-0.1640	-0.3080 to -0.02004	Yes
20314 19:50 X vs. 22:0 X	-0.2900	-0.4340 to -0.1460	Yes
20315 19:50 X vs. 22:1 X	-0.2847	-0.4286 to -0.1407	Yes
20316 19:50 X vs. 22:10 X	-0.2800	-0.4240 to -0.1360	Yes
20317 19:50 X vs. 22:40 X	-0.1803	-0.3243 to -0.03637	Yes
20318 19:50 X vs. 22:50 X	-0.2430	-0.3870 to -0.09904	Yes
20319 19:50 X vs. 23:0 X	-0.3553	-0.4993 to -0.2114	Yes
20320 19:50 X vs. 23:1 X	-0.5450	-0.7060 to -0.3840	Yes
20321 19:50 X vs. 23:10 X	-0.2947	-0.4386 to -0.1507	Yes
20322 19:50 X vs. 23:40 X	-0.1750	-0.3360 to -0.01405	Yes
20323 19:50 X vs. 23:50 X	-0.2090	-0.3530 to -0.06504	Yes
20324 19:50 X vs. 24:0 X	-0.4727	-0.6166 to -0.3287	Yes
20325 19:50 X vs. 24:1 X	-0.6290	-0.7900 to -0.4680	Yes
20326 19:50 X vs. 24:10 X	-0.4565	-0.6175 to -0.2955	Yes
20327 19:50 X vs. 24:40 X	-0.1913	-0.3353 to -0.04737	Yes
20328 19:50 X vs. 24:50 X	-0.1130	-0.2570 to 0.03096	No
20329 19:50 X vs. 25:0 X	-0.8100	-0.9710 to -0.6490	Yes
20330 19:50 X vs. 25:1 X	-0.9995	-1.160 to -0.8385	Yes
20331 19:50 X vs. 25:10 X	-0.3610	-0.5646 to -0.1574	Yes
20332 19:50 X vs. 25:40 X	-0.1933	-0.3373 to -0.04937	Yes
20333 19:50 X vs. 25:50 X	-0.1677	-0.3116 to -0.02371	Yes
20334 19:50 X vs. 26:0 X	-1.023	-1.183 to -0.8615	Yes
20335 19:50 X vs. 26:1 X	-1.136	-1.296 to -0.9745	Yes
20336 19:50 X vs. 26:10 X	-0.8220	-0.9830 to -0.6610	Yes
20337 19:50 X vs. 26:40 X	-0.2677	-0.4116 to -0.1237	Yes
20338 19:50 X vs. 26:50 X	-0.1280	-0.2720 to 0.01596	No
20339 19:50 X vs. 27:0 X	-1.210	-1.354 to -1.066	Yes
20340 19:50 X vs. 27:1 X	-1.301	-1.462 to -1.140	Yes

	2way ANOVA<br>Multiple comparisons	A<br>Data Set-A<br>Y	B<br>Data Set-B<br>Y	C<br>Data Set-C<br>Y
20341	19:**50 X** vs. 27:**10 X**	-1.113	-1.274 to -0.9520	Yes
20342	19:**50 X** vs. 27:**40 X**	-0.2677	-0.4116 to -0.1237	Yes
20343	19:**50 X** vs. 27:**50 X**	-0.1273	-0.2713 to 0.01663	No
20344	19:**50 X** vs. 28:**0 X**	-1.160	-1.320 to -0.9985	Yes
20345	19:**50 X** vs. 28:**1 X**	-1.200	-1.344 to -1.056	Yes
20346	19:**50 X** vs. 28:**10 X**	-1.002	-1.163 to -0.8410	Yes
20347	19:**50 X** vs. 28:**40 X**	-0.2800	-0.4240 to -0.1360	Yes
20348	19:**50 X** vs. 28:**50 X**	-0.1753	-0.3193 to -0.03137	Yes
20349	19:**50 X** vs. 29:**0 X**	-1.641	-1.802 to -1.480	Yes
20350	19:**50 X** vs. 29:**1 X**	-1.726	-1.887 to -1.565	Yes
20351	19:**50 X** vs. 29:**10 X**	-0.9405	-1.101 to -0.7795	Yes
20352	19:**50 X** vs. 29:**40 X**	-0.3880	-0.5320 to -0.2440	Yes
20353	19:**50 X** vs. 29:**50 X**	-0.1767	-0.3206 to -0.03271	Yes
20354	19:**50 X** vs. 30:**0 X**	-1.356	-1.516 to -1.195	Yes
20355	19:**50 X** vs. 30:**1 X**	-1.677	-1.838 to -1.516	Yes
20356	19:**50 X** vs. 30:**10 X**	-1.015	-1.176 to -0.8540	Yes
20357	19:**50 X** vs. 30:**40 X**	-0.3150	-0.4590 to -0.1710	Yes
20358	19:**50 X** vs. 30:**50 X**	-0.2290	-0.3730 to -0.08504	Yes
20359	19:**50 X** vs. 31:**0 X**	-1.674	-1.834 to -1.513	Yes
20360	19:**50 X** vs. 31:**1 X**	-1.664	-1.825 to -1.503	Yes
20361	19:**50 X** vs. 31:**10 X**	-1.147	-1.307 to -0.9855	Yes
20362	19:**50 X** vs. 31:**40 X**	-0.4067	-0.5506 to -0.2627	Yes
20363	19:**50 X** vs. 31:**50 X**	-0.2080	-0.3520 to -0.06404	Yes
20364	19:**50 X** vs. 32:**0 X**	-2.265	-2.409 to -2.121	Yes
20365	19:**50 X** vs. 32:**1 X**	-1.721	-1.882 to -1.560	Yes
20366	19:**50 X** vs. 32:**10 X**	-1.577	-1.721 to -1.433	Yes
20367	19:**50 X** vs. 32:**40 X**	-0.4030	-0.5470 to -0.2590	Yes
20368	19:**50 X** vs. 32:**50 X**	-0.2077	-0.3516 to -0.06371	Yes
20369	19:**50 X** vs. 33:**0 X**	-2.269	-2.413 to -2.125	Yes
20370	19:**50 X** vs. 33:**1 X**	-2.083	-2.227 to -1.939	Yes
20371	19:**50 X** vs. 33:**10 X**	-1.744	-1.888 to -1.600	Yes
20372	19:**50 X** vs. 33:**40 X**	-0.4217	-0.5656 to -0.2777	Yes
20373	19:**50 X** vs. 33:**50 X**	-0.2227	-0.3666 to -0.07871	Yes
20374	19:**50 X** vs. 34:**0 X**	-2.193	-2.337 to -2.049	Yes
20375	19:**50 X** vs. 34:**1 X**	-2.201	-2.345 to -2.057	Yes
20376	19:**50 X** vs. 34:**10 X**	-1.834	-1.978 to -1.690	Yes
20377	19:**50 X** vs. 34:**40 X**	-0.4337	-0.5776 to -0.2897	Yes
20378	19:**50 X** vs. 34:**50 X**	-0.3717	-0.5156 to -0.2277	Yes
20379	19:**50 X** vs. 35:**0 X**	-2.542	-2.686 to -2.398	Yes
20380	19:**50 X** vs. 35:**1 X**	-2.308	-2.452 to -2.164	Yes
20381	19:**50 X** vs. 35:**10 X**	-1.796	-1.940 to -1.652	Yes
20382	19:**50 X** vs. 35:**40 X**	-0.4427	-0.5866 to -0.2987	Yes
20383	19:**50 X** vs. 35:**50 X**	-0.3843	-0.5283 to -0.2404	Yes
20384	19:**50 X** vs. 36:**0 X**	-2.749	-2.909 to -2.588	Yes
20385	19:**50 X** vs. 36:**1 X**	-2.444	-2.588 to -2.300	Yes

2way ANOVA<br>Multiple comparisons	A<br>Data Set-A<br>Y	B<br>Data Set-B<br>Y	C<br>Data Set-C<br>Y
20386 19:50 X vs. 36:10 X	-1.801	-1.945 to -1.657	Yes
20387 19:50 X vs. 36:40 X	-0.4980	-0.6420 to -0.3540	Yes
20388 19:50 X vs. 36:50 X	-0.3477	-0.4916 to -0.2037	Yes
20389 19:50 X vs. 37:0 X	-3.132	-3.276 to -2.988	Yes
20390 19:50 X vs. 37:1 X	-2.514	-2.658 to -2.370	Yes
20391 19:50 X vs. 37:10 X	-1.803	-1.947 to -1.659	Yes
20392 19:50 X vs. 37:40 X	-0.5353	-0.6793 to -0.3914	Yes
20393 19:50 X vs. 37:50 X	-0.3563	-0.5003 to -0.2124	Yes
20394 19:50 X vs. 38:0 X	-3.077	-3.221 to -2.933	Yes
20395 19:50 X vs. 38:1 X	-2.633	-2.777 to -2.489	Yes
20396 19:50 X vs. 38:10 X	-2.078	-2.222 to -1.934	Yes
20397 19:50 X vs. 38:40 X	-0.5397	-0.6836 to -0.3957	Yes
20398 19:50 X vs. 38:50 X	-0.3603	-0.5043 to -0.2164	Yes
20399 19:50 X vs. 39:0 X	-3.308	-3.452 to -3.164	Yes
20400 19:50 X vs. 39:1 X	-3.200	-3.344 to -3.056	Yes
20401 19:50 X vs. 39:10 X	-2.223	-2.367 to -2.079	Yes
20402 19:50 X vs. 39:40 X	-0.5457	-0.6896 to -0.4017	Yes
20403 19:50 X vs. 39:50 X	-0.3913	-0.5353 to -0.2474	Yes
20404 19:50 X vs. 40:0 X	-3.349	-3.493 to -3.205	Yes
20405 19:50 X vs. 40:1 X	-3.275	-3.419 to -3.131	Yes
20406 19:50 X vs. 40:10 X	-2.480	-2.624 to -2.336	Yes
20407 19:50 X vs. 40:40 X	-0.5503	-0.6943 to -0.4064	Yes
20408 19:50 X vs. 40:50 X	-0.3977	-0.5416 to -0.2537	Yes
20409 19:50 X vs. 41:0 X	-3.390	-3.534 to -3.246	Yes
20410 19:50 X vs. 41:1 X	-3.389	-3.533 to -3.245	Yes
20411 19:50 X vs. 41:10 X	-2.533	-2.677 to -2.389	Yes
20412 19:50 X vs. 41:40 X	-0.4977	-0.6416 to -0.3537	Yes
20413 19:50 X vs. 41:50 X	-0.4103	-0.5543 to -0.2664	Yes
20414 19:50 X vs. 42:0 X	-3.532	-3.676 to -3.388	Yes
20415 19:50 X vs. 42:1 X	-3.551	-3.695 to -3.407	Yes
20416 19:50 X vs. 42:10 X	-2.534	-2.678 to -2.390	Yes
20417 19:50 X vs. 42:40 X	-0.5103	-0.6543 to -0.3664	Yes
20418 19:50 X vs. 42:50 X	-0.4137	-0.5576 to -0.2697	Yes
20419 19:50 X vs. 43:0 X	-3.622	-3.766 to -3.478	Yes
20420 19:50 X vs. 43:1 X	-3.536	-3.680 to -3.392	Yes
20421 19:50 X vs. 43:10 X	-3.252	-3.396 to -3.108	Yes
20422 19:50 X vs. 43:40 X	-0.5087	-0.6526 to -0.3647	Yes
20423 19:50 X vs. 43:50 X	-0.4140	-0.5580 to -0.2700	Yes
20424 19:50 X vs. 44:0 X	-3.646	-3.790 to -3.502	Yes
20425 19:50 X vs. 44:1 X	-3.535	-3.679 to -3.391	Yes
20426 19:50 X vs. 44:10 X	-3.315	-3.459 to -3.171	Yes
20427 19:50 X vs. 44:40 X	-0.5107	-0.6546 to -0.3667	Yes
20428 19:50 X vs. 44:50 X	-0.3463	-0.4903 to -0.2024	Yes
20429 19:50 X vs. 45:0 X	-3.510	-3.654 to -3.366	Yes
20430 19:50 X vs. 45:1 X	-3.312	-3.456 to -3.168	Yes

	2way ANOVA<br>Multiple comparisons	A<br>Data Set-A<br>Y	B<br>Data Set-B<br>Y	C<br>Data Set-C<br>Y
20431	19:**50 X** vs. 45:**10 X**	-3.195	-3.339 to -3.051	Yes
20432	19:**50 X** vs. 45:**40 X**	-0.5120	-0.6560 to -0.3680	Yes
20433	19:**50 X** vs. 45:**50 X**	-0.3433	-0.4873 to -0.1994	Yes
20434	19:**50 X** vs. 46:**0 X**	-3.315	-3.459 to -3.171	Yes
20435	19:**50 X** vs. 46:**1 X**	-3.282	-3.426 to -3.138	Yes
20436	19:**50 X** vs. 46:**10 X**	-3.190	-3.334 to -3.046	Yes
20437	19:**50 X** vs. 46:**40 X**	-0.5257	-0.6696 to -0.3817	Yes
20438	19:**50 X** vs. 46:**50 X**	-0.3363	-0.4803 to -0.1924	Yes
20439	19:**50 X** vs. 47:**0 X**	-3.290	-3.434 to -3.146	Yes
20440	19:**50 X** vs. 47:**1 X**	-3.194	-3.338 to -3.050	Yes
20441	19:**50 X** vs. 47:**10 X**	-3.176	-3.320 to -3.032	Yes
20442	19:**50 X** vs. 47:**40 X**	-0.5357	-0.6796 to -0.3917	Yes
20443	19:**50 X** vs. 47:**50 X**	-0.2943	-0.4383 to -0.1504	Yes
20444	19:**50 X** vs. 48:**0 X**	-3.278	-3.438 to -3.117	Yes
20445	19:**50 X** vs. 48:**1 X**	-3.181	-3.325 to -3.037	Yes
20446	19:**50 X** vs. 48:**10 X**	-3.176	-3.320 to -3.032	Yes
20447	19:**50 X** vs. 48:**40 X**	-0.5420	-0.6860 to -0.3980	Yes
20448	19:**50 X** vs. 48:**50 X**	-0.3017	-0.4456 to -0.1577	Yes
20449	19:**50 X** vs. 49:**0 X**	-3.278	-3.422 to -3.134	Yes
20450	19:**50 X** vs. 49:**1 X**	-3.181	-3.325 to -3.037	Yes
20451	19:**50 X** vs. 49:**10 X**	-3.176	-3.320 to -3.032	Yes
20452	19:**50 X** vs. 49:**40 X**	-0.5463	-0.6903 to -0.4024	Yes
20453	19:**50 X** vs. 49:**50 X**	-0.3010	-0.4450 to -0.1570	Yes
20454	19:**50 X** vs. 50:**0 X**	-3.278	-3.422 to -3.134	Yes
20455	19:**50 X** vs. 50:**1 X**	-3.181	-3.325 to -3.037	Yes
20456	19:**50 X** vs. 50:**10 X**	-3.176	-3.320 to -3.032	Yes
20457	19:**50 X** vs. 50:**40 X**	-0.5540	-0.6980 to -0.4100	Yes
20458	19:**50 X** vs. 50:**50 X**	-0.3007	-0.4446 to -0.1567	Yes
20459	20:**0 X** vs. 20:**1 X**	0.1807	0.03671 to 0.3246	Yes
20460	20:**0 X** vs. 20:**10 X**	0.09167	-0.05229 to 0.2356	No
20461	20:**0 X** vs. 20:**40 X**	0.2243	0.08037 to 0.3683	Yes
20462	20:**0 X** vs. 20:**50 X**	0.1803	0.03637 to 0.3243	Yes
20463	20:**0 X** vs. 21:**0 X**	-0.02333	-0.1673 to 0.1206	No
20464	20:**0 X** vs. 21:**1 X**	0.02367	-0.1203 to 0.1676	No
20465	20:**0 X** vs. 21:**10 X**	0.02667	-0.1173 to 0.1706	No
20466	20:**0 X** vs. 21:**40 X**	0.1513	0.007374 to 0.2953	Yes
20467	20:**0 X** vs. 21:**50 X**	0.0610	-0.08296 to 0.2050	No
20468	20:**0 X** vs. 22:**0 X**	-0.06500	-0.2090 to 0.07896	No
20469	20:**0 X** vs. 22:**1 X**	-0.05967	-0.2036 to 0.08429	No
20470	20:**0 X** vs. 22:**10 X**	-0.0550	-0.1990 to 0.08896	No
20471	20:**0 X** vs. 22:**40 X**	0.04467	-0.09929 to 0.1886	No
20472	20:**0 X** vs. 22:**50 X**	-0.0180	-0.1620 to 0.1260	No
20473	20:**0 X** vs. 23:**0 X**	-0.1303	-0.2743 to 0.01363	No
20474	20:**0 X** vs. 23:**1 X**	-0.3200	-0.4810 to -0.1590	Yes
20475	20:**0 X** vs. 23:**10 X**	-0.06967	-0.2136 to 0.07429	No

	2way ANOVA<br>Multiple comparisons	A<br>Data Set-A<br>Y	B<br>Data Set-B<br>Y	C<br>Data Set-C<br>Y
20476	20:0 X vs. 23:40 X	0.05000	-0.1110 to 0.2110	No
20477	20:0 X vs. 23:50 X	0.0160	-0.1280 to 0.1600	No
20478	20:0 X vs. 24:0 X	-0.2477	-0.3916 to -0.1037	Yes
20479	20:0 X vs. 24:1 X	-0.4040	-0.5650 to -0.2430	Yes
20480	20:0 X vs. 24:10 X	-0.2315	-0.3925 to -0.07055	Yes
20481	20:0 X vs. 24:40 X	0.03367	-0.1103 to 0.1776	No
20482	20:0 X vs. 24:50 X	0.1120	-0.03196 to 0.2560	No
20483	20:0 X vs. 25:0 X	-0.5850	-0.7460 to -0.4240	Yes
20484	20:0 X vs. 25:1 X	-0.7745	-0.9355 to -0.6135	Yes
20485	20:0 X vs. 25:10 X	-0.1360	-0.3396 to 0.06759	No
20486	20:0 X vs. 25:40 X	0.03167	-0.1123 to 0.1756	No
20487	20:0 X vs. 25:50 X	0.05733	-0.08663 to 0.2013	No
20488	20:0 X vs. 26:0 X	-0.7975	-0.9585 to -0.6365	Yes
20489	20:0 X vs. 26:1 X	-0.9105	-1.071 to -0.7495	Yes
20490	20:0 X vs. 26:10 X	-0.5970	-0.7580 to -0.4360	Yes
20491	20:0 X vs. 26:40 X	-0.04267	-0.1866 to 0.1013	No
20492	20:0 X vs. 26:50 X	0.0970	-0.04696 to 0.2410	No
20493	20:0 X vs. 27:0 X	-0.9853	-1.129 to -0.8414	Yes
20494	20:0 X vs. 27:1 X	-1.076	-1.237 to -0.9150	Yes
20495	20:0 X vs. 27:10 X	-0.8880	-1.049 to -0.7270	Yes
20496	20:0 X vs. 27:40 X	-0.04267	-0.1866 to 0.1013	No
20497	20:0 X vs. 27:50 X	0.09767	-0.04629 to 0.2416	No
20498	20:0 X vs. 28:0 X	-0.9345	-1.095 to -0.7735	Yes
20499	20:0 X vs. 28:1 X	-0.9753	-1.119 to -0.8314	Yes
20500	20:0 X vs. 28:10 X	-0.7770	-0.9380 to -0.6160	Yes
20501	20:0 X vs. 28:40 X	-0.0550	-0.1990 to 0.08896	No
20502	20:0 X vs. 28:50 X	0.04967	-0.09429 to 0.1936	No
20503	20:0 X vs. 29:0 X	-1.416	-1.577 to -1.255	Yes
20504	20:0 X vs. 29:1 X	-1.501	-1.662 to -1.340	Yes
20505	20:0 X vs. 29:10 X	-0.7155	-0.8765 to -0.5545	Yes
20506	20:0 X vs. 29:40 X	-0.1630	-0.3070 to -0.01904	Yes
20507	20:0 X vs. 29:50 X	0.04833	-0.09563 to 0.1923	No
20508	20:0 X vs. 30:0 X	-1.131	-1.291 to -0.9695	Yes
20509	20:0 X vs. 30:1 X	-1.452	-1.613 to -1.291	Yes
20510	20:0 X vs. 30:10 X	-0.7900	-0.9510 to -0.6290	Yes
20511	20:0 X vs. 30:40 X	-0.0900	-0.2340 to 0.05396	No
20512	20:0 X vs. 30:50 X	-0.004000	-0.1480 to 0.1400	No
20513	20:0 X vs. 31:0 X	-1.449	-1.609 to -1.288	Yes
20514	20:0 X vs. 31:1 X	-1.439	-1.600 to -1.278	Yes
20515	20:0 X vs. 31:10 X	-0.9215	-1.082 to -0.7605	Yes
20516	20:0 X vs. 31:40 X	-0.1817	-0.3256 to -0.03771	Yes
20517	20:0 X vs. 31:50 X	0.0170	-0.1270 to 0.1610	No
20518	20:0 X vs. 32:0 X	-2.040	-2.184 to -1.896	Yes
20519	20:0 X vs. 32:1 X	-1.496	-1.657 to -1.335	Yes
20520	20:0 X vs. 32:10 X	-1.352	-1.496 to -1.208	Yes

2way ANOVA<br>Multiple comparisons		A<br>Data Set-A<br>Y	B<br>Data Set-B<br>Y	C<br>Data Set-C<br>Y
20521	20:0 X vs. 32:40 X	-0.1780	-0.3220 to -0.03404	Yes
20522	20:0 X vs. 32:50 X	0.01733	-0.1266 to 0.1613	No
20523	20:0 X vs. 33:0 X	-2.044	-2.188 to -1.900	Yes
20524	20:0 X vs. 33:1 X	-1.858	-2.002 to -1.714	Yes
20525	20:0 X vs. 33:10 X	-1.519	-1.663 to -1.375	Yes
20526	20:0 X vs. 33:40 X	-0.1967	-0.3406 to -0.05271	Yes
20527	20:0 X vs. 33:50 X	0.002333	-0.1416 to 0.1463	No
20528	20:0 X vs. 34:0 X	-1.968	-2.112 to -1.824	Yes
20529	20:0 X vs. 34:1 X	-1.976	-2.120 to -1.832	Yes
20530	20:0 X vs. 34:10 X	-1.609	-1.753 to -1.465	Yes
20531	20:0 X vs. 34:40 X	-0.2087	-0.3526 to -0.06471	Yes
20532	20:0 X vs. 34:50 X	-0.1467	-0.2906 to -0.002707	Yes
20533	20:0 X vs. 35:0 X	-2.317	-2.461 to -2.173	Yes
20534	20:0 X vs. 35:1 X	-2.083	-2.227 to -1.939	Yes
20535	20:0 X vs. 35:10 X	-1.571	-1.715 to -1.427	Yes
20536	20:0 X vs. 35:40 X	-0.2177	-0.3616 to -0.07371	Yes
20537	20:0 X vs. 35:50 X	-0.1593	-0.3033 to -0.01537	Yes
20538	20:0 X vs. 36:0 X	-2.524	-2.684 to -2.363	Yes
20539	20:0 X vs. 36:1 X	-2.219	-2.363 to -2.075	Yes
20540	20:0 X vs. 36:10 X	-1.576	-1.720 to -1.432	Yes
20541	20:0 X vs. 36:40 X	-0.2730	-0.4170 to -0.1290	Yes
20542	20:0 X vs. 36:50 X	-0.1227	-0.2666 to 0.02129	No
20543	20:0 X vs. 37:0 X	-2.907	-3.051 to -2.763	Yes
20544	20:0 X vs. 37:1 X	-2.289	-2.433 to -2.145	Yes
20545	20:0 X vs. 37:10 X	-1.578	-1.722 to -1.434	Yes
20546	20:0 X vs. 37:40 X	-0.3103	-0.4543 to -0.1664	Yes
20547	20:0 X vs. 37:50 X	-0.1313	-0.2753 to 0.01263	No
20548	20:0 X vs. 38:0 X	-2.852	-2.996 to -2.708	Yes
20549	20:0 X vs. 38:1 X	-2.408	-2.552 to -2.264	Yes
20550	20:0 X vs. 38:10 X	-1.853	-1.997 to -1.709	Yes
20551	20:0 X vs. 38:40 X	-0.3147	-0.4586 to -0.1707	Yes
20552	20:0 X vs. 38:50 X	-0.1353	-0.2793 to 0.008626	No
20553	20:0 X vs. 39:0 X	-3.083	-3.227 to -2.939	Yes
20554	20:0 X vs. 39:1 X	-2.975	-3.119 to -2.831	Yes
20555	20:0 X vs. 39:10 X	-1.998	-2.142 to -1.854	Yes
20556	20:0 X vs. 39:40 X	-0.3207	-0.4646 to -0.1767	Yes
20557	20:0 X vs. 39:50 X	-0.1663	-0.3103 to -0.02237	Yes
20558	20:0 X vs. 40:0 X	-3.124	-3.268 to -2.980	Yes
20559	20:0 X vs. 40:1 X	-3.050	-3.194 to -2.906	Yes
20560	20:0 X vs. 40:10 X	-2.255	-2.399 to -2.111	Yes
20561	20:0 X vs. 40:40 X	-0.3253	-0.4693 to -0.1814	Yes
20562	20:0 X vs. 40:50 X	-0.1727	-0.3166 to -0.02871	Yes
20563	20:0 X vs. 41:0 X	-3.165	-3.309 to -3.021	Yes
20564	20:0 X vs. 41:1 X	-3.164	-3.308 to -3.020	Yes
20565	20:0 X vs. 41:10 X	-2.308	-2.452 to -2.164	Yes

	2way ANOVA<br>Multiple comparisons	A<br>Data Set-A<br>Y	B<br>Data Set-B<br>Y	C<br>Data Set-C<br>Y
20566	20:**0 X** vs. 41:**40 X**	-0.2727	-0.4166 to -0.1287	Yes
20567	20:**0 X** vs. 41:**50 X**	-0.1853	-0.3293 to -0.04137	Yes
20568	20:**0 X** vs. 42:**0 X**	-3.307	-3.451 to -3.163	Yes
20569	20:**0 X** vs. 42:**1 X**	-3.326	-3.470 to -3.182	Yes
20570	20:**0 X** vs. 42:**10 X**	-2.309	-2.453 to -2.165	Yes
20571	20:**0 X** vs. 42:**40 X**	-0.2853	-0.4293 to -0.1414	Yes
20572	20:**0 X** vs. 42:**50 X**	-0.1887	-0.3326 to -0.04471	Yes
20573	20:**0 X** vs. 43:**0 X**	-3.397	-3.541 to -3.253	Yes
20574	20:**0 X** vs. 43:**1 X**	-3.311	-3.455 to -3.167	Yes
20575	20:**0 X** vs. 43:**10 X**	-3.027	-3.171 to -2.883	Yes
20576	20:**0 X** vs. 43:**40 X**	-0.2837	-0.4276 to -0.1397	Yes
20577	20:**0 X** vs. 43:**50 X**	-0.1890	-0.3330 to -0.04504	Yes
20578	20:**0 X** vs. 44:**0 X**	-3.421	-3.565 to -3.277	Yes
20579	20:**0 X** vs. 44:**1 X**	-3.310	-3.454 to -3.166	Yes
20580	20:**0 X** vs. 44:**10 X**	-3.090	-3.234 to -2.946	Yes
20581	20:**0 X** vs. 44:**40 X**	-0.2857	-0.4296 to -0.1417	Yes
20582	20:**0 X** vs. 44:**50 X**	-0.1213	-0.2653 to 0.02263	No
20583	20:**0 X** vs. 45:**0 X**	-3.285	-3.429 to -3.141	Yes
20584	20:**0 X** vs. 45:**1 X**	-3.087	-3.231 to -2.943	Yes
20585	20:**0 X** vs. 45:**10 X**	-2.970	-3.114 to -2.826	Yes
20586	20:**0 X** vs. 45:**40 X**	-0.2870	-0.4310 to -0.1430	Yes
20587	20:**0 X** vs. 45:**50 X**	-0.1183	-0.2623 to 0.02563	No
20588	20:**0 X** vs. 46:**0 X**	-3.090	-3.234 to -2.946	Yes
20589	20:**0 X** vs. 46:**1 X**	-3.057	-3.201 to -2.913	Yes
20590	20:**0 X** vs. 46:**10 X**	-2.965	-3.109 to -2.821	Yes
20591	20:**0 X** vs. 46:**40 X**	-0.3007	-0.4446 to -0.1567	Yes
20592	20:**0 X** vs. 46:**50 X**	-0.1113	-0.2553 to 0.03263	No
20593	20:**0 X** vs. 47:**0 X**	-3.065	-3.209 to -2.921	Yes
20594	20:**0 X** vs. 47:**1 X**	-2.969	-3.113 to -2.825	Yes
20595	20:**0 X** vs. 47:**10 X**	-2.951	-3.095 to -2.807	Yes
20596	20:**0 X** vs. 47:**40 X**	-0.3107	-0.4546 to -0.1667	Yes
20597	20:**0 X** vs. 47:**50 X**	-0.06933	-0.2133 to 0.07463	No
20598	20:**0 X** vs. 48:**0 X**	-3.053	-3.213 to -2.892	Yes
20599	20:**0 X** vs. 48:**1 X**	-2.956	-3.100 to -2.812	Yes
20600	20:**0 X** vs. 48:**10 X**	-2.951	-3.095 to -2.807	Yes
20601	20:**0 X** vs. 48:**40 X**	-0.3170	-0.4610 to -0.1730	Yes
20602	20:**0 X** vs. 48:**50 X**	-0.07667	-0.2206 to 0.06729	No
20603	20:**0 X** vs. 49:**0 X**	-3.053	-3.197 to -2.909	Yes
20604	20:**0 X** vs. 49:**1 X**	-2.956	-3.100 to -2.812	Yes
20605	20:**0 X** vs. 49:**10 X**	-2.951	-3.095 to -2.807	Yes
20606	20:**0 X** vs. 49:**40 X**	-0.3213	-0.4653 to -0.1774	Yes
20607	20:**0 X** vs. 49:**50 X**	-0.0760	-0.2200 to 0.06796	No
20608	20:**0 X** vs. 50:**0 X**	-3.053	-3.197 to -2.909	Yes
20609	20:**0 X** vs. 50:**1 X**	-2.956	-3.100 to -2.812	Yes
20610	20:**0 X** vs. 50:**10 X**	-2.951	-3.095 to -2.807	Yes

	2way ANOVA Multiple comparisons	A Data Set-A Y	B Data Set-B Y	C Data Set-C Y
20611	20:**0 X** vs. 50:**40 X**	-0.3290	-0.4730 to -0.1850	Yes
20612	20:**0 X** vs. 50:**50 X**	-0.07567	-0.2196 to 0.06829	No
20613	20:**1 X** vs. 20:**10 X**	-0.08900	-0.2330 to 0.05496	No
20614	20:**1 X** vs. 20:**40 X**	0.04367	-0.1003 to 0.1876	No
20615	20:**1 X** vs. 20:**50 X**	-0.0003333	-0.1443 to 0.1436	No
20616	20:**1 X** vs. 21:**0 X**	-0.2040	-0.3480 to -0.06004	Yes
20617	20:**1 X** vs. 21:**1 X**	-0.1570	-0.3010 to -0.01304	Yes
20618	20:**1 X** vs. 21:**10 X**	-0.1540	-0.2980 to -0.01004	Yes
20619	20:**1 X** vs. 21:**40 X**	-0.02933	-0.1733 to 0.1146	No
20620	20:**1 X** vs. 21:**50 X**	-0.1197	-0.2636 to 0.02429	No
20621	20:**1 X** vs. 22:**0 X**	-0.2457	-0.3896 to -0.1017	Yes
20622	20:**1 X** vs. 22:**1 X**	-0.2403	-0.3843 to -0.09637	Yes
20623	20:**1 X** vs. 22:**10 X**	-0.2357	-0.3796 to -0.09171	Yes
20624	20:**1 X** vs. 22:**40 X**	-0.1360	-0.2800 to 0.007960	No
20625	20:**1 X** vs. 22:**50 X**	-0.1987	-0.3426 to -0.05471	Yes
20626	20:**1 X** vs. 23:**0 X**	-0.3110	-0.4550 to -0.1670	Yes
20627	20:**1 X** vs. 23:**1 X**	-0.5007	-0.6616 to -0.3397	Yes
20628	20:**1 X** vs. 23:**10 X**	-0.2503	-0.3943 to -0.1064	Yes
20629	20:**1 X** vs. 23:**40 X**	-0.1307	-0.2916 to 0.03029	No
20630	20:**1 X** vs. 23:**50 X**	-0.1647	-0.3086 to -0.02071	Yes
20631	20:**1 X** vs. 24:**0 X**	-0.4283	-0.5723 to -0.2844	Yes
20632	20:**1 X** vs. 24:**1 X**	-0.5847	-0.7456 to -0.4237	Yes
20633	20:**1 X** vs. 24:**10 X**	-0.4122	-0.5731 to -0.2512	Yes
20634	20:**1 X** vs. 24:**40 X**	-0.1470	-0.2910 to -0.003040	Yes
20635	20:**1 X** vs. 24:**50 X**	-0.06867	-0.2126 to 0.07529	No
20636	20:**1 X** vs. 25:**0 X**	-0.7657	-0.9266 to -0.6047	Yes
20637	20:**1 X** vs. 25:**1 X**	-0.9552	-1.116 to -0.7942	Yes
20638	20:**1 X** vs. 25:**10 X**	-0.3167	-0.5203 to -0.1131	Yes
20639	20:**1 X** vs. 25:**40 X**	-0.1490	-0.2930 to -0.005040	Yes
20640	20:**1 X** vs. 25:**50 X**	-0.1233	-0.2673 to 0.02063	No
20641	20:**1 X** vs. 26:**0 X**	-0.9782	-1.139 to -0.8172	Yes
20642	20:**1 X** vs. 26:**1 X**	-1.091	-1.252 to -0.9302	Yes
20643	20:**1 X** vs. 26:**10 X**	-0.7777	-0.9386 to -0.6167	Yes
20644	20:**1 X** vs. 26:**40 X**	-0.2233	-0.3673 to -0.07937	Yes
20645	20:**1 X** vs. 26:**50 X**	-0.08367	-0.2276 to 0.06029	No
20646	20:**1 X** vs. 27:**0 X**	-1.166	-1.310 to -1.022	Yes
20647	20:**1 X** vs. 27:**1 X**	-1.257	-1.418 to -1.096	Yes
20648	20:**1 X** vs. 27:**10 X**	-1.069	-1.230 to -0.9077	Yes
20649	20:**1 X** vs. 27:**40 X**	-0.2233	-0.3673 to -0.07937	Yes
20650	20:**1 X** vs. 27:**50 X**	-0.0830	-0.2270 to 0.06096	No
20651	20:**1 X** vs. 28:**0 X**	-1.115	-1.276 to -0.9542	Yes
20652	20:**1 X** vs. 28:**1 X**	-1.156	-1.300 to -1.012	Yes
20653	20:**1 X** vs. 28:**10 X**	-0.9577	-1.119 to -0.7967	Yes
20654	20:**1 X** vs. 28:**40 X**	-0.2357	-0.3796 to -0.09171	Yes
20655	20:**1 X** vs. 28:**50 X**	-0.1310	-0.2750 to 0.01296	No

	2way ANOVA<br>Multiple comparisons	A<br>Data Set-A<br>Y	B<br>Data Set-B<br>Y	C<br>Data Set-C<br>Y
20656	20:**1 X** vs. 29:**0 X**	-1.597	-1.758 to -1.436	Yes
20657	20:**1 X** vs. 29:**1 X**	-1.682	-1.843 to -1.521	Yes
20658	20:**1 X** vs. 29:**10 X**	-0.8962	-1.057 to -0.7352	Yes
20659	20:**1 X** vs. 29:**40 X**	-0.3437	-0.4876 to -0.1997	Yes
20660	20:**1 X** vs. 29:**50 X**	-0.1323	-0.2763 to 0.01163	No
20661	20:**1 X** vs. 30:**0 X**	-1.311	-1.472 to -1.150	Yes
20662	20:**1 X** vs. 30:**1 X**	-1.633	-1.794 to -1.472	Yes
20663	20:**1 X** vs. 30:**10 X**	-0.9707	-1.132 to -0.8097	Yes
20664	20:**1 X** vs. 30:**40 X**	-0.2707	-0.4146 to -0.1267	Yes
20665	20:**1 X** vs. 30:**50 X**	-0.1847	-0.3286 to -0.04071	Yes
20666	20:**1 X** vs. 31:**0 X**	-1.629	-1.790 to -1.468	Yes
20667	20:**1 X** vs. 31:**1 X**	-1.620	-1.781 to -1.459	Yes
20668	20:**1 X** vs. 31:**10 X**	-1.102	-1.263 to -0.9412	Yes
20669	20:**1 X** vs. 31:**40 X**	-0.3623	-0.5063 to -0.2184	Yes
20670	20:**1 X** vs. 31:**50 X**	-0.1637	-0.3076 to -0.01971	Yes
20671	20:**1 X** vs. 32:**0 X**	-2.221	-2.365 to -2.077	Yes
20672	20:**1 X** vs. 32:**1 X**	-1.677	-1.838 to -1.516	Yes
20673	20:**1 X** vs. 32:**10 X**	-1.533	-1.677 to -1.389	Yes
20674	20:**1 X** vs. 32:**40 X**	-0.3587	-0.5026 to -0.2147	Yes
20675	20:**1 X** vs. 32:**50 X**	-0.1633	-0.3073 to -0.01937	Yes
20676	20:**1 X** vs. 33:**0 X**	-2.224	-2.368 to -2.080	Yes
20677	20:**1 X** vs. 33:**1 X**	-2.039	-2.183 to -1.895	Yes
20678	20:**1 X** vs. 33:**10 X**	-1.699	-1.843 to -1.555	Yes
20679	20:**1 X** vs. 33:**40 X**	-0.3773	-0.5213 to -0.2334	Yes
20680	20:**1 X** vs. 33:**50 X**	-0.1783	-0.3223 to -0.03437	Yes
20681	20:**1 X** vs. 34:**0 X**	-2.149	-2.293 to -2.005	Yes
20682	20:**1 X** vs. 34:**1 X**	-2.156	-2.300 to -2.012	Yes
20683	20:**1 X** vs. 34:**10 X**	-1.790	-1.934 to -1.646	Yes
20684	20:**1 X** vs. 34:**40 X**	-0.3893	-0.5333 to -0.2454	Yes
20685	20:**1 X** vs. 34:**50 X**	-0.3273	-0.4713 to -0.1834	Yes
20686	20:**1 X** vs. 35:**0 X**	-2.498	-2.642 to -2.354	Yes
20687	20:**1 X** vs. 35:**1 X**	-2.263	-2.407 to -2.119	Yes
20688	20:**1 X** vs. 35:**10 X**	-1.751	-1.895 to -1.607	Yes
20689	20:**1 X** vs. 35:**40 X**	-0.3983	-0.5423 to -0.2544	Yes
20690	20:**1 X** vs. 35:**50 X**	-0.3400	-0.4840 to -0.1960	Yes
20691	20:**1 X** vs. 36:**0 X**	-2.704	-2.865 to -2.543	Yes
20692	20:**1 X** vs. 36:**1 X**	-2.400	-2.544 to -2.256	Yes
20693	20:**1 X** vs. 36:**10 X**	-1.757	-1.901 to -1.613	Yes
20694	20:**1 X** vs. 36:**40 X**	-0.4537	-0.5976 to -0.3097	Yes
20695	20:**1 X** vs. 36:**50 X**	-0.3033	-0.4473 to -0.1594	Yes
20696	20:**1 X** vs. 37:**0 X**	-3.088	-3.232 to -2.944	Yes
20697	20:**1 X** vs. 37:**1 X**	-2.470	-2.614 to -2.326	Yes
20698	20:**1 X** vs. 37:**10 X**	-1.759	-1.903 to -1.615	Yes
20699	20:**1 X** vs. 37:**40 X**	-0.4910	-0.6350 to -0.3470	Yes
20700	20:**1 X** vs. 37:**50 X**	-0.3120	-0.4560 to -0.1680	Yes

	2way ANOVA<br>Multiple comparisons	A<br>Data Set-A<br>Y	B<br>Data Set-B<br>Y	C<br>Data Set-C<br>Y
20701	20:**1 X** vs. 38:**0 X**	-3.033	-3.177 to -2.889	Yes
20702	20:**1 X** vs. 38:**1 X**	-2.589	-2.733 to -2.445	Yes
20703	20:**1 X** vs. 38:**10 X**	-2.034	-2.178 to -1.890	Yes
20704	20:**1 X** vs. 38:**40 X**	-0.4953	-0.6393 to -0.3514	Yes
20705	20:**1 X** vs. 38:**50 X**	-0.3160	-0.4600 to -0.1720	Yes
20706	20:**1 X** vs. 39:**0 X**	-3.264	-3.408 to -3.120	Yes
20707	20:**1 X** vs. 39:**1 X**	-3.155	-3.299 to -3.011	Yes
20708	20:**1 X** vs. 39:**10 X**	-2.179	-2.323 to -2.035	Yes
20709	20:**1 X** vs. 39:**40 X**	-0.5013	-0.6453 to -0.3574	Yes
20710	20:**1 X** vs. 39:**50 X**	-0.3470	-0.4910 to -0.2030	Yes
20711	20:**1 X** vs. 40:**0 X**	-3.305	-3.449 to -3.161	Yes
20712	20:**1 X** vs. 40:**1 X**	-3.231	-3.375 to -3.087	Yes
20713	20:**1 X** vs. 40:**10 X**	-2.435	-2.579 to -2.291	Yes
20714	20:**1 X** vs. 40:**40 X**	-0.5060	-0.6500 to -0.3620	Yes
20715	20:**1 X** vs. 40:**50 X**	-0.3533	-0.4973 to -0.2094	Yes
20716	20:**1 X** vs. 41:**0 X**	-3.346	-3.490 to -3.202	Yes
20717	20:**1 X** vs. 41:**1 X**	-3.345	-3.489 to -3.201	Yes
20718	20:**1 X** vs. 41:**10 X**	-2.489	-2.633 to -2.345	Yes
20719	20:**1 X** vs. 41:**40 X**	-0.4533	-0.5973 to -0.3094	Yes
20720	20:**1 X** vs. 41:**50 X**	-0.3660	-0.5100 to -0.2220	Yes
20721	20:**1 X** vs. 42:**0 X**	-3.487	-3.631 to -3.343	Yes
20722	20:**1 X** vs. 42:**1 X**	-3.507	-3.651 to -3.363	Yes
20723	20:**1 X** vs. 42:**10 X**	-2.489	-2.633 to -2.345	Yes
20724	20:**1 X** vs. 42:**40 X**	-0.4660	-0.6100 to -0.3220	Yes
20725	20:**1 X** vs. 42:**50 X**	-0.3693	-0.5133 to -0.2254	Yes
20726	20:**1 X** vs. 43:**0 X**	-3.577	-3.721 to -3.433	Yes
20727	20:**1 X** vs. 43:**1 X**	-3.492	-3.636 to -3.348	Yes
20728	20:**1 X** vs. 43:**10 X**	-3.207	-3.351 to -3.063	Yes
20729	20:**1 X** vs. 43:**40 X**	-0.4643	-0.6083 to -0.3204	Yes
20730	20:**1 X** vs. 43:**50 X**	-0.3697	-0.5136 to -0.2257	Yes
20731	20:**1 X** vs. 44:**0 X**	-3.601	-3.745 to -3.457	Yes
20732	20:**1 X** vs. 44:**1 X**	-3.491	-3.635 to -3.347	Yes
20733	20:**1 X** vs. 44:**10 X**	-3.271	-3.415 to -3.127	Yes
20734	20:**1 X** vs. 44:**40 X**	-0.4663	-0.6103 to -0.3224	Yes
20735	20:**1 X** vs. 44:**50 X**	-0.3020	-0.4460 to -0.1580	Yes
20736	20:**1 X** vs. 45:**0 X**	-3.466	-3.610 to -3.322	Yes
20737	20:**1 X** vs. 45:**1 X**	-3.268	-3.412 to -3.124	Yes
20738	20:**1 X** vs. 45:**10 X**	-3.150	-3.294 to -3.006	Yes
20739	20:**1 X** vs. 45:**40 X**	-0.4677	-0.6116 to -0.3237	Yes
20740	20:**1 X** vs. 45:**50 X**	-0.2990	-0.4430 to -0.1550	Yes
20741	20:**1 X** vs. 46:**0 X**	-3.270	-3.414 to -3.126	Yes
20742	20:**1 X** vs. 46:**1 X**	-3.237	-3.381 to -3.093	Yes
20743	20:**1 X** vs. 46:**10 X**	-3.145	-3.289 to -3.001	Yes
20744	20:**1 X** vs. 46:**40 X**	-0.4813	-0.6253 to -0.3374	Yes
20745	20:**1 X** vs. 46:**50 X**	-0.2920	-0.4360 to -0.1480	Yes

2way ANOVA<br>Multiple comparisons		A<br>Data Set-A<br>Y	B<br>Data Set-B<br>Y	C<br>Data Set-C<br>Y
20746	20:1 X vs. 47:0 X	-3.246	-3.390 to -3.102	Yes
20747	20:1 X vs. 47:1 X	-3.150	-3.294 to -3.006	Yes
20748	20:1 X vs. 47:10 X	-3.131	-3.275 to -2.987	Yes
20749	20:1 X vs. 47:40 X	-0.4913	-0.6353 to -0.3474	Yes
20750	20:1 X vs. 47:50 X	-0.2500	-0.3940 to -0.1060	Yes
20751	20:1 X vs. 48:0 X	-3.233	-3.394 to -3.072	Yes
20752	20:1 X vs. 48:1 X	-3.137	-3.281 to -2.993	Yes
20753	20:1 X vs. 48:10 X	-3.131	-3.275 to -2.987	Yes
20754	20:1 X vs. 48:40 X	-0.4977	-0.6416 to -0.3537	Yes
20755	20:1 X vs. 48:50 X	-0.2573	-0.4013 to -0.1134	Yes
20756	20:1 X vs. 49:0 X	-3.233	-3.377 to -3.089	Yes
20757	20:1 X vs. 49:1 X	-3.137	-3.281 to -2.993	Yes
20758	20:1 X vs. 49:10 X	-3.131	-3.275 to -2.987	Yes
20759	20:1 X vs. 49:40 X	-0.5020	-0.6460 to -0.3580	Yes
20760	20:1 X vs. 49:50 X	-0.2567	-0.4006 to -0.1127	Yes
20761	20:1 X vs. 50:0 X	-3.233	-3.377 to -3.089	Yes
20762	20:1 X vs. 50:1 X	-3.137	-3.281 to -2.993	Yes
20763	20:1 X vs. 50:10 X	-3.131	-3.275 to -2.987	Yes
20764	20:1 X vs. 50:40 X	-0.5097	-0.6536 to -0.3657	Yes
20765	20:1 X vs. 50:50 X	-0.2563	-0.4003 to -0.1124	Yes
20766	20:10 X vs. 20:40 X	0.1327	-0.01129 to 0.2766	No
20767	20:10 X vs. 20:50 X	0.08867	-0.05529 to 0.2326	No
20768	20:10 X vs. 21:0 X	-0.1150	-0.2590 to 0.02896	No
20769	20:10 X vs. 21:1 X	-0.0680	-0.2120 to 0.07596	No
20770	20:10 X vs. 21:10 X	-0.06500	-0.2090 to 0.07896	No
20771	20:10 X vs. 21:40 X	0.05967	-0.08429 to 0.2036	No
20772	20:10 X vs. 21:50 X	-0.03067	-0.1746 to 0.1133	No
20773	20:10 X vs. 22:0 X	-0.1567	-0.3006 to -0.01271	Yes
20774	20:10 X vs. 22:1 X	-0.1513	-0.2953 to -0.007374	Yes
20775	20:10 X vs. 22:10 X	-0.1467	-0.2906 to -0.002707	Yes
20776	20:10 X vs. 22:40 X	-0.04700	-0.1910 to 0.09696	No
20777	20:10 X vs. 22:50 X	-0.1097	-0.2536 to 0.03429	No
20778	20:10 X vs. 23:0 X	-0.2220	-0.3660 to -0.07804	Yes
20779	20:10 X vs. 23:1 X	-0.4117	-0.5726 to -0.2507	Yes
20780	20:10 X vs. 23:10 X	-0.1613	-0.3053 to -0.01737	Yes
20781	20:10 X vs. 23:40 X	-0.04167	-0.2026 to 0.1193	No
20782	20:10 X vs. 23:50 X	-0.07567	-0.2196 to 0.06829	No
20783	20:10 X vs. 24:0 X	-0.3393	-0.4833 to -0.1954	Yes
20784	20:10 X vs. 24:1 X	-0.4957	-0.6566 to -0.3347	Yes
20785	20:10 X vs. 24:10 X	-0.3232	-0.4841 to -0.1622	Yes
20786	20:10 X vs. 24:40 X	-0.05800	-0.2020 to 0.08596	No
20787	20:10 X vs. 24:50 X	0.02033	-0.1236 to 0.1643	No
20788	20:10 X vs. 25:0 X	-0.6767	-0.8376 to -0.5157	Yes
20789	20:10 X vs. 25:1 X	-0.8662	-1.027 to -0.7052	Yes
20790	20:10 X vs. 25:10 X	-0.2277	-0.4313 to -0.02408	Yes

2way ANOVA<br>Multiple comparisons		A<br>Data Set-A<br>Y	B<br>Data Set-B<br>Y	C<br>Data Set-C<br>Y
20791	20:**10 X** vs. 25:**40 X**	-0.0600	-0.2040 to 0.08396	No
20792	20:**10 X** vs. 25:**50 X**	-0.03433	-0.1783 to 0.1096	No
20793	20:**10 X** vs. 26:**0 X**	-0.8892	-1.050 to -0.7282	Yes
20794	20:**10 X** vs. 26:**1 X**	-1.002	-1.163 to -0.8412	Yes
20795	20:**10 X** vs. 26:**10 X**	-0.6887	-0.8496 to -0.5277	Yes
20796	20:**10 X** vs. 26:**40 X**	-0.1343	-0.2783 to 0.009626	No
20797	20:**10 X** vs. 26:**50 X**	0.005333	-0.1386 to 0.1493	No
20798	20:**10 X** vs. 27:**0 X**	-1.077	-1.221 to -0.9330	Yes
20799	20:**10 X** vs. 27:**1 X**	-1.168	-1.329 to -1.007	Yes
20800	20:**10 X** vs. 27:**10 X**	-0.9797	-1.141 to -0.8187	Yes
20801	20:**10 X** vs. 27:**40 X**	-0.1343	-0.2783 to 0.009626	No
20802	20:**10 X** vs. 27:**50 X**	0.006000	-0.1380 to 0.1500	No
20803	20:**10 X** vs. 28:**0 X**	-1.026	-1.187 to -0.8652	Yes
20804	20:**10 X** vs. 28:**1 X**	-1.067	-1.211 to -0.9230	Yes
20805	20:**10 X** vs. 28:**10 X**	-0.8687	-1.030 to -0.7077	Yes
20806	20:**10 X** vs. 28:**40 X**	-0.1467	-0.2906 to -0.002707	Yes
20807	20:**10 X** vs. 28:**50 X**	-0.0420	-0.1860 to 0.1020	No
20808	20:**10 X** vs. 29:**0 X**	-1.508	-1.669 to -1.347	Yes
20809	20:**10 X** vs. 29:**1 X**	-1.593	-1.754 to -1.432	Yes
20810	20:**10 X** vs. 29:**10 X**	-0.8072	-0.9681 to -0.6462	Yes
20811	20:**10 X** vs. 29:**40 X**	-0.2547	-0.3986 to -0.1107	Yes
20812	20:**10 X** vs. 29:**50 X**	-0.04333	-0.1873 to 0.1006	No
20813	20:**10 X** vs. 30:**0 X**	-1.222	-1.383 to -1.061	Yes
20814	20:**10 X** vs. 30:**1 X**	-1.544	-1.705 to -1.383	Yes
20815	20:**10 X** vs. 30:**10 X**	-0.8817	-1.043 to -0.7207	Yes
20816	20:**10 X** vs. 30:**40 X**	-0.1817	-0.3256 to -0.03771	Yes
20817	20:**10 X** vs. 30:**50 X**	-0.09567	-0.2396 to 0.04829	No
20818	20:**10 X** vs. 31:**0 X**	-1.540	-1.701 to -1.379	Yes
20819	20:**10 X** vs. 31:**1 X**	-1.531	-1.692 to -1.370	Yes
20820	20:**10 X** vs. 31:**10 X**	-1.013	-1.174 to -0.8522	Yes
20821	20:**10 X** vs. 31:**40 X**	-0.2733	-0.4173 to -0.1294	Yes
20822	20:**10 X** vs. 31:**50 X**	-0.07467	-0.2186 to 0.06929	No
20823	20:**10 X** vs. 32:**0 X**	-2.132	-2.276 to -1.988	Yes
20824	20:**10 X** vs. 32:**1 X**	-1.588	-1.749 to -1.427	Yes
20825	20:**10 X** vs. 32:**10 X**	-1.444	-1.588 to -1.300	Yes
20826	20:**10 X** vs. 32:**40 X**	-0.2697	-0.4136 to -0.1257	Yes
20827	20:**10 X** vs. 32:**50 X**	-0.07433	-0.2183 to 0.06963	No
20828	20:**10 X** vs. 33:**0 X**	-2.135	-2.279 to -1.991	Yes
20829	20:**10 X** vs. 33:**1 X**	-1.950	-2.094 to -1.806	Yes
20830	20:**10 X** vs. 33:**10 X**	-1.610	-1.754 to -1.466	Yes
20831	20:**10 X** vs. 33:**40 X**	-0.2883	-0.4323 to -0.1444	Yes
20832	20:**10 X** vs. 33:**50 X**	-0.08933	-0.2333 to 0.05463	No
20833	20:**10 X** vs. 34:**0 X**	-2.060	-2.204 to -1.916	Yes
20834	20:**10 X** vs. 34:**1 X**	-2.067	-2.211 to -1.923	Yes
20835	20:**10 X** vs. 34:**10 X**	-1.701	-1.845 to -1.557	Yes

	2way ANOVA<br>Multiple comparisons	A<br>Data Set-A<br>Y	B<br>Data Set-B<br>Y	C<br>Data Set-C<br>Y
20836	20:**10 X** vs. 34:**40 X**	-0.3003	-0.4443 to -0.1564	Yes
20837	20:**10 X** vs. 34:**50 X**	-0.2383	-0.3823 to -0.09437	Yes
20838	20:**10 X** vs. 35:**0 X**	-2.409	-2.553 to -2.265	Yes
20839	20:**10 X** vs. 35:**1 X**	-2.174	-2.318 to -2.030	Yes
20840	20:**10 X** vs. 35:**10 X**	-1.662	-1.806 to -1.518	Yes
20841	20:**10 X** vs. 35:**40 X**	-0.3093	-0.4533 to -0.1654	Yes
20842	20:**10 X** vs. 35:**50 X**	-0.2510	-0.3950 to -0.1070	Yes
20843	20:**10 X** vs. 36:**0 X**	-2.615	-2.776 to -2.454	Yes
20844	20:**10 X** vs. 36:**1 X**	-2.311	-2.455 to -2.167	Yes
20845	20:**10 X** vs. 36:**10 X**	-1.668	-1.812 to -1.524	Yes
20846	20:**10 X** vs. 36:**40 X**	-0.3647	-0.5086 to -0.2207	Yes
20847	20:**10 X** vs. 36:**50 X**	-0.2143	-0.3583 to -0.07037	Yes
20848	20:**10 X** vs. 37:**0 X**	-2.999	-3.143 to -2.855	Yes
20849	20:**10 X** vs. 37:**1 X**	-2.381	-2.525 to -2.237	Yes
20850	20:**10 X** vs. 37:**10 X**	-1.670	-1.814 to -1.526	Yes
20851	20:**10 X** vs. 37:**40 X**	-0.4020	-0.5460 to -0.2580	Yes
20852	20:**10 X** vs. 37:**50 X**	-0.2230	-0.3670 to -0.07904	Yes
20853	20:**10 X** vs. 38:**0 X**	-2.944	-3.088 to -2.800	Yes
20854	20:**10 X** vs. 38:**1 X**	-2.500	-2.644 to -2.356	Yes
20855	20:**10 X** vs. 38:**10 X**	-1.945	-2.089 to -1.801	Yes
20856	20:**10 X** vs. 38:**40 X**	-0.4063	-0.5503 to -0.2624	Yes
20857	20:**10 X** vs. 38:**50 X**	-0.2270	-0.3710 to -0.08304	Yes
20858	20:**10 X** vs. 39:**0 X**	-3.175	-3.319 to -3.031	Yes
20859	20:**10 X** vs. 39:**1 X**	-3.066	-3.210 to -2.922	Yes
20860	20:**10 X** vs. 39:**10 X**	-2.090	-2.234 to -1.946	Yes
20861	20:**10 X** vs. 39:**40 X**	-0.4123	-0.5563 to -0.2684	Yes
20862	20:**10 X** vs. 39:**50 X**	-0.2580	-0.4020 to -0.1140	Yes
20863	20:**10 X** vs. 40:**0 X**	-3.216	-3.360 to -3.072	Yes
20864	20:**10 X** vs. 40:**1 X**	-3.142	-3.286 to -2.998	Yes
20865	20:**10 X** vs. 40:**10 X**	-2.346	-2.490 to -2.202	Yes
20866	20:**10 X** vs. 40:**40 X**	-0.4170	-0.5610 to -0.2730	Yes
20867	20:**10 X** vs. 40:**50 X**	-0.2643	-0.4083 to -0.1204	Yes
20868	20:**10 X** vs. 41:**0 X**	-3.257	-3.401 to -3.113	Yes
20869	20:**10 X** vs. 41:**1 X**	-3.256	-3.400 to -3.112	Yes
20870	20:**10 X** vs. 41:**10 X**	-2.400	-2.544 to -2.256	Yes
20871	20:**10 X** vs. 41:**40 X**	-0.3643	-0.5083 to -0.2204	Yes
20872	20:**10 X** vs. 41:**50 X**	-0.2770	-0.4210 to -0.1330	Yes
20873	20:**10 X** vs. 42:**0 X**	-3.398	-3.542 to -3.254	Yes
20874	20:**10 X** vs. 42:**1 X**	-3.418	-3.562 to -3.274	Yes
20875	20:**10 X** vs. 42:**10 X**	-2.400	-2.544 to -2.256	Yes
20876	20:**10 X** vs. 42:**40 X**	-0.3770	-0.5210 to -0.2330	Yes
20877	20:**10 X** vs. 42:**50 X**	-0.2803	-0.4243 to -0.1364	Yes
20878	20:**10 X** vs. 43:**0 X**	-3.488	-3.632 to -3.344	Yes
20879	20:**10 X** vs. 43:**1 X**	-3.403	-3.547 to -3.259	Yes
20880	20:**10 X** vs. 43:**10 X**	-3.118	-3.262 to -2.974	Yes

2way ANOVA<br>Multiple comparisons		A<br>Data Set-A<br>Y	B<br>Data Set-B<br>Y	C<br>Data Set-C<br>Y
20881	20:10 X vs. 43:40 X	-0.3753	-0.5193 to -0.2314	Yes
20882	20:10 X vs. 43:50 X	-0.2807	-0.4246 to -0.1367	Yes
20883	20:10 X vs. 44:0 X	-3.512	-3.656 to -3.368	Yes
20884	20:10 X vs. 44:1 X	-3.402	-3.546 to -3.258	Yes
20885	20:10 X vs. 44:10 X	-3.182	-3.326 to -3.038	Yes
20886	20:10 X vs. 44:40 X	-0.3773	-0.5213 to -0.2334	Yes
20887	20:10 X vs. 44:50 X	-0.2130	-0.3570 to -0.06904	Yes
20888	20:10 X vs. 45:0 X	-3.377	-3.521 to -3.233	Yes
20889	20:10 X vs. 45:1 X	-3.179	-3.323 to -3.035	Yes
20890	20:10 X vs. 45:10 X	-3.061	-3.205 to -2.917	Yes
20891	20:10 X vs. 45:40 X	-0.3787	-0.5226 to -0.2347	Yes
20892	20:10 X vs. 45:50 X	-0.2100	-0.3540 to -0.06604	Yes
20893	20:10 X vs. 46:0 X	-3.181	-3.325 to -3.037	Yes
20894	20:10 X vs. 46:1 X	-3.148	-3.292 to -3.004	Yes
20895	20:10 X vs. 46:10 X	-3.056	-3.200 to -2.912	Yes
20896	20:10 X vs. 46:40 X	-0.3923	-0.5363 to -0.2484	Yes
20897	20:10 X vs. 46:50 X	-0.2030	-0.3470 to -0.05904	Yes
20898	20:10 X vs. 47:0 X	-3.157	-3.301 to -3.013	Yes
20899	20:10 X vs. 47:1 X	-3.061	-3.205 to -2.917	Yes
20900	20:10 X vs. 47:10 X	-3.042	-3.186 to -2.898	Yes
20901	20:10 X vs. 47:40 X	-0.4023	-0.5463 to -0.2584	Yes
20902	20:10 X vs. 47:50 X	-0.1610	-0.3050 to -0.01704	Yes
20903	20:10 X vs. 48:0 X	-3.144	-3.305 to -2.983	Yes
20904	20:10 X vs. 48:1 X	-3.048	-3.192 to -2.904	Yes
20905	20:10 X vs. 48:10 X	-3.042	-3.186 to -2.898	Yes
20906	20:10 X vs. 48:40 X	-0.4087	-0.5526 to -0.2647	Yes
20907	20:10 X vs. 48:50 X	-0.1683	-0.3123 to -0.02437	Yes
20908	20:10 X vs. 49:0 X	-3.144	-3.288 to -3.000	Yes
20909	20:10 X vs. 49:1 X	-3.048	-3.192 to -2.904	Yes
20910	20:10 X vs. 49:10 X	-3.042	-3.186 to -2.898	Yes
20911	20:10 X vs. 49:40 X	-0.4130	-0.5570 to -0.2690	Yes
20912	20:10 X vs. 49:50 X	-0.1677	-0.3116 to -0.02371	Yes
20913	20:10 X vs. 50:0 X	-3.144	-3.288 to -3.000	Yes
20914	20:10 X vs. 50:1 X	-3.048	-3.192 to -2.904	Yes
20915	20:10 X vs. 50:10 X	-3.042	-3.186 to -2.898	Yes
20916	20:10 X vs. 50:40 X	-0.4207	-0.5646 to -0.2767	Yes
20917	20:10 X vs. 50:50 X	-0.1673	-0.3113 to -0.02337	Yes
20918	20:40 X vs. 20:50 X	-0.0440	-0.1880 to 0.09996	No
20919	20:40 X vs. 21:0 X	-0.2477	-0.3916 to -0.1037	Yes
20920	20:40 X vs. 21:1 X	-0.2007	-0.3446 to -0.05671	Yes
20921	20:40 X vs. 21:10 X	-0.1977	-0.3416 to -0.05371	Yes
20922	20:40 X vs. 21:40 X	-0.0730	-0.2170 to 0.07096	No
20923	20:40 X vs. 21:50 X	-0.1633	-0.3073 to -0.01937	Yes
20924	20:40 X vs. 22:0 X	-0.2893	-0.4333 to -0.1454	Yes
20925	20:40 X vs. 22:1 X	-0.2840	-0.4280 to -0.1400	Yes

2way ANOVA<br>Multiple comparisons	A<br>Data Set-A<br>Y	B<br>Data Set-B<br>Y	C<br>Data Set-C<br>Y
20926 20:40 X vs. 22:10 X	-0.2793	-0.4233 to -0.1354	Yes
20927 20:40 X vs. 22:40 X	-0.1797	-0.3236 to -0.03571	Yes
20928 20:40 X vs. 22:50 X	-0.2423	-0.3863 to -0.09837	Yes
20929 20:40 X vs. 23:0 X	-0.3547	-0.4986 to -0.2107	Yes
20930 20:40 X vs. 23:1 X	-0.5443	-0.7053 to -0.3834	Yes
20931 20:40 X vs. 23:10 X	-0.2940	-0.4380 to -0.1500	Yes
20932 20:40 X vs. 23:40 X	-0.1743	-0.3353 to -0.01338	Yes
20933 20:40 X vs. 23:50 X	-0.2083	-0.3523 to -0.06437	Yes
20934 20:40 X vs. 24:0 X	-0.4720	-0.6160 to -0.3280	Yes
20935 20:40 X vs. 24:1 X	-0.6283	-0.7893 to -0.4674	Yes
20936 20:40 X vs. 24:10 X	-0.4558	-0.6168 to -0.2949	Yes
20937 20:40 X vs. 24:40 X	-0.1907	-0.3346 to -0.04671	Yes
20938 20:40 X vs. 24:50 X	-0.1123	-0.2563 to 0.03163	No
20939 20:40 X vs. 25:0 X	-0.8093	-0.9703 to -0.6484	Yes
20940 20:40 X vs. 25:1 X	-0.9988	-1.160 to -0.8379	Yes
20941 20:40 X vs. 25:10 X	-0.3603	-0.5639 to -0.1567	Yes
20942 20:40 X vs. 25:40 X	-0.1927	-0.3366 to -0.04871	Yes
20943 20:40 X vs. 25:50 X	-0.1670	-0.3110 to -0.02304	Yes
20944 20:40 X vs. 26:0 X	-1.022	-1.183 to -0.8609	Yes
20945 20:40 X vs. 26:1 X	-1.135	-1.296 to -0.9739	Yes
20946 20:40 X vs. 26:10 X	-0.8213	-0.9823 to -0.6604	Yes
20947 20:40 X vs. 26:40 X	-0.2670	-0.4110 to -0.1230	Yes
20948 20:40 X vs. 26:50 X	-0.1273	-0.2713 to 0.01663	No
20949 20:40 X vs. 27:0 X	-1.210	-1.354 to -1.066	Yes
20950 20:40 X vs. 27:1 X	-1.300	-1.461 to -1.139	Yes
20951 20:40 X vs. 27:10 X	-1.112	-1.273 to -0.9514	Yes
20952 20:40 X vs. 27:40 X	-0.2670	-0.4110 to -0.1230	Yes
20953 20:40 X vs. 27:50 X	-0.1267	-0.2706 to 0.01729	No
20954 20:40 X vs. 28:0 X	-1.159	-1.320 to -0.9979	Yes
20955 20:40 X vs. 28:1 X	-1.200	-1.344 to -1.056	Yes
20956 20:40 X vs. 28:10 X	-1.001	-1.162 to -0.8404	Yes
20957 20:40 X vs. 28:40 X	-0.2793	-0.4233 to -0.1354	Yes
20958 20:40 X vs. 28:50 X	-0.1747	-0.3186 to -0.03071	Yes
20959 20:40 X vs. 29:0 X	-1.640	-1.801 to -1.479	Yes
20960 20:40 X vs. 29:1 X	-1.725	-1.886 to -1.564	Yes
20961 20:40 X vs. 29:10 X	-0.9398	-1.101 to -0.7789	Yes
20962 20:40 X vs. 29:40 X	-0.3873	-0.5313 to -0.2434	Yes
20963 20:40 X vs. 29:50 X	-0.1760	-0.3200 to -0.03204	Yes
20964 20:40 X vs. 30:0 X	-1.355	-1.516 to -1.194	Yes
20965 20:40 X vs. 30:1 X	-1.676	-1.837 to -1.515	Yes
20966 20:40 X vs. 30:10 X	-1.014	-1.175 to -0.8534	Yes
20967 20:40 X vs. 30:40 X	-0.3143	-0.4583 to -0.1704	Yes
20968 20:40 X vs. 30:50 X	-0.2283	-0.3723 to -0.08437	Yes
20969 20:40 X vs. 31:0 X	-1.673	-1.834 to -1.512	Yes
20970 20:40 X vs. 31:1 X	-1.663	-1.824 to -1.502	Yes

	2way ANOVA<br>Multiple comparisons	A<br>Data Set-A<br>Y	B<br>Data Set-B<br>Y	C<br>Data Set-C<br>Y
20971	20:**40 X** vs. 31:**10 X**	-1.146	-1.307 to -0.9849	Yes
20972	20:**40 X** vs. 31:**40 X**	-0.4060	-0.5500 to -0.2620	Yes
20973	20:**40 X** vs. 31:**50 X**	-0.2073	-0.3513 to -0.06337	Yes
20974	20:**40 X** vs. 32:**0 X**	-2.264	-2.408 to -2.120	Yes
20975	20:**40 X** vs. 32:**1 X**	-1.720	-1.881 to -1.559	Yes
20976	20:**40 X** vs. 32:**10 X**	-1.576	-1.720 to -1.432	Yes
20977	20:**40 X** vs. 32:**40 X**	-0.4023	-0.5463 to -0.2584	Yes
20978	20:**40 X** vs. 32:**50 X**	-0.2070	-0.3510 to -0.06304	Yes
20979	20:**40 X** vs. 33:**0 X**	-2.268	-2.412 to -2.124	Yes
20980	20:**40 X** vs. 33:**1 X**	-2.083	-2.227 to -1.939	Yes
20981	20:**40 X** vs. 33:**10 X**	-1.743	-1.887 to -1.599	Yes
20982	20:**40 X** vs. 33:**40 X**	-0.4210	-0.5650 to -0.2770	Yes
20983	20:**40 X** vs. 33:**50 X**	-0.2220	-0.3660 to -0.07804	Yes
20984	20:**40 X** vs. 34:**0 X**	-2.193	-2.337 to -2.049	Yes
20985	20:**40 X** vs. 34:**1 X**	-2.200	-2.344 to -2.056	Yes
20986	20:**40 X** vs. 34:**10 X**	-1.833	-1.977 to -1.689	Yes
20987	20:**40 X** vs. 34:**40 X**	-0.4330	-0.5770 to -0.2890	Yes
20988	20:**40 X** vs. 34:**50 X**	-0.3710	-0.5150 to -0.2270	Yes
20989	20:**40 X** vs. 35:**0 X**	-2.541	-2.685 to -2.397	Yes
20990	20:**40 X** vs. 35:**1 X**	-2.307	-2.451 to -2.163	Yes
20991	20:**40 X** vs. 35:**10 X**	-1.795	-1.939 to -1.651	Yes
20992	20:**40 X** vs. 35:**40 X**	-0.4420	-0.5860 to -0.2980	Yes
20993	20:**40 X** vs. 35:**50 X**	-0.3837	-0.5276 to -0.2397	Yes
20994	20:**40 X** vs. 36:**0 X**	-2.748	-2.909 to -2.587	Yes
20995	20:**40 X** vs. 36:**1 X**	-2.444	-2.588 to -2.300	Yes
20996	20:**40 X** vs. 36:**10 X**	-1.801	-1.945 to -1.657	Yes
20997	20:**40 X** vs. 36:**40 X**	-0.4973	-0.6413 to -0.3534	Yes
20998	20:**40 X** vs. 36:**50 X**	-0.3470	-0.4910 to -0.2030	Yes
20999	20:**40 X** vs. 37:**0 X**	-3.132	-3.276 to -2.988	Yes
21000	20:**40 X** vs. 37:**1 X**	-2.514	-2.658 to -2.370	Yes
21001	20:**40 X** vs. 37:**10 X**	-1.802	-1.946 to -1.658	Yes
21002	20:**40 X** vs. 37:**40 X**	-0.5347	-0.6786 to -0.3907	Yes
21003	20:**40 X** vs. 37:**50 X**	-0.3557	-0.4996 to -0.2117	Yes
21004	20:**40 X** vs. 38:**0 X**	-3.077	-3.221 to -2.933	Yes
21005	20:**40 X** vs. 38:**1 X**	-2.632	-2.776 to -2.488	Yes
21006	20:**40 X** vs. 38:**10 X**	-2.078	-2.222 to -1.934	Yes
21007	20:**40 X** vs. 38:**40 X**	-0.5390	-0.6830 to -0.3950	Yes
21008	20:**40 X** vs. 38:**50 X**	-0.3597	-0.5036 to -0.2157	Yes
21009	20:**40 X** vs. 39:**0 X**	-3.307	-3.451 to -3.163	Yes
21010	20:**40 X** vs. 39:**1 X**	-3.199	-3.343 to -3.055	Yes
21011	20:**40 X** vs. 39:**10 X**	-2.223	-2.367 to -2.079	Yes
21012	20:**40 X** vs. 39:**40 X**	-0.5450	-0.6890 to -0.4010	Yes
21013	20:**40 X** vs. 39:**50 X**	-0.3907	-0.5346 to -0.2467	Yes
21014	20:**40 X** vs. 40:**0 X**	-3.348	-3.492 to -3.204	Yes
21015	20:**40 X** vs. 40:**1 X**	-3.274	-3.418 to -3.130	Yes

2way ANOVA<br>Multiple comparisons		A<br>Data Set-A<br>Y	B<br>Data Set-B<br>Y	C<br>Data Set-C<br>Y
21016	20:40 X vs. 40:10 X	-2.479	-2.623 to -2.335	Yes
21017	20:40 X vs. 40:40 X	-0.5497	-0.6936 to -0.4057	Yes
21018	20:40 X vs. 40:50 X	-0.3970	-0.5410 to -0.2530	Yes
21019	20:40 X vs. 41:0 X	-3.389	-3.533 to -3.245	Yes
21020	20:40 X vs. 41:1 X	-3.388	-3.532 to -3.244	Yes
21021	20:40 X vs. 41:10 X	-2.533	-2.677 to -2.389	Yes
21022	20:40 X vs. 41:40 X	-0.4970	-0.6410 to -0.3530	Yes
21023	20:40 X vs. 41:50 X	-0.4097	-0.5536 to -0.2657	Yes
21024	20:40 X vs. 42:0 X	-3.531	-3.675 to -3.387	Yes
21025	20:40 X vs. 42:1 X	-3.551	-3.695 to -3.407	Yes
21026	20:40 X vs. 42:10 X	-2.533	-2.677 to -2.389	Yes
21027	20:40 X vs. 42:40 X	-0.5097	-0.6536 to -0.3657	Yes
21028	20:40 X vs. 42:50 X	-0.4130	-0.5570 to -0.2690	Yes
21029	20:40 X vs. 43:0 X	-3.621	-3.765 to -3.477	Yes
21030	20:40 X vs. 43:1 X	-3.535	-3.679 to -3.391	Yes
21031	20:40 X vs. 43:10 X	-3.251	-3.395 to -3.107	Yes
21032	20:40 X vs. 43:40 X	-0.5080	-0.6520 to -0.3640	Yes
21033	20:40 X vs. 43:50 X	-0.4133	-0.5573 to -0.2694	Yes
21034	20:40 X vs. 44:0 X	-3.645	-3.789 to -3.501	Yes
21035	20:40 X vs. 44:1 X	-3.535	-3.679 to -3.391	Yes
21036	20:40 X vs. 44:10 X	-3.314	-3.458 to -3.170	Yes
21037	20:40 X vs. 44:40 X	-0.5100	-0.6540 to -0.3660	Yes
21038	20:40 X vs. 44:50 X	-0.3457	-0.4896 to -0.2017	Yes
21039	20:40 X vs. 45:0 X	-3.510	-3.654 to -3.366	Yes
21040	20:40 X vs. 45:1 X	-3.312	-3.456 to -3.168	Yes
21041	20:40 X vs. 45:10 X	-3.194	-3.338 to -3.050	Yes
21042	20:40 X vs. 45:40 X	-0.5113	-0.6553 to -0.3674	Yes
21043	20:40 X vs. 45:50 X	-0.3427	-0.4866 to -0.1987	Yes
21044	20:40 X vs. 46:0 X	-3.314	-3.458 to -3.170	Yes
21045	20:40 X vs. 46:1 X	-3.281	-3.425 to -3.137	Yes
21046	20:40 X vs. 46:10 X	-3.189	-3.333 to -3.045	Yes
21047	20:40 X vs. 46:40 X	-0.5250	-0.6690 to -0.3810	Yes
21048	20:40 X vs. 46:50 X	-0.3357	-0.4796 to -0.1917	Yes
21049	20:40 X vs. 47:0 X	-3.290	-3.434 to -3.146	Yes
21050	20:40 X vs. 47:1 X	-3.194	-3.338 to -3.050	Yes
21051	20:40 X vs. 47:10 X	-3.175	-3.319 to -3.031	Yes
21052	20:40 X vs. 47:40 X	-0.5350	-0.6790 to -0.3910	Yes
21053	20:40 X vs. 47:50 X	-0.2937	-0.4376 to -0.1497	Yes
21054	20:40 X vs. 48:0 X	-3.277	-3.438 to -3.116	Yes
21055	20:40 X vs. 48:1 X	-3.181	-3.325 to -3.037	Yes
21056	20:40 X vs. 48:10 X	-3.175	-3.319 to -3.031	Yes
21057	20:40 X vs. 48:40 X	-0.5413	-0.6853 to -0.3974	Yes
21058	20:40 X vs. 48:50 X	-0.3010	-0.4450 to -0.1570	Yes
21059	20:40 X vs. 49:0 X	-3.277	-3.421 to -3.133	Yes
21060	20:40 X vs. 49:1 X	-3.181	-3.325 to -3.037	Yes

2way ANOVA Multiple comparisons		A Data Set-A Y	B Data Set-B Y	C Data Set-C Y
21061	20:**40 X** vs. 49:**10 X**	-3.175	-3.319 to -3.031	Yes
21062	20:**40 X** vs. 49:**40 X**	-0.5457	-0.6896 to -0.4017	Yes
21063	20:**40 X** vs. 49:**50 X**	-0.3003	-0.4443 to -0.1564	Yes
21064	20:**40 X** vs. 50:**0 X**	-3.277	-3.421 to -3.133	Yes
21065	20:**40 X** vs. 50:**1 X**	-3.181	-3.325 to -3.037	Yes
21066	20:**40 X** vs. 50:**10 X**	-3.175	-3.319 to -3.031	Yes
21067	20:**40 X** vs. 50:**40 X**	-0.5533	-0.6973 to -0.4094	Yes
21068	20:**40 X** vs. 50:**50 X**	-0.3000	-0.4440 to -0.1560	Yes
21069	20:**50 X** vs. 21:**0 X**	-0.2037	-0.3476 to -0.05971	Yes
21070	20:**50 X** vs. 21:**1 X**	-0.1567	-0.3006 to -0.01271	Yes
21071	20:**50 X** vs. 21:**10 X**	-0.1537	-0.2976 to -0.009707	Yes
21072	20:**50 X** vs. 21:**40 X**	-0.02900	-0.1730 to 0.1150	No
21073	20:**50 X** vs. 21:**50 X**	-0.1193	-0.2633 to 0.02463	No
21074	20:**50 X** vs. 22:**0 X**	-0.2453	-0.3893 to -0.1014	Yes
21075	20:**50 X** vs. 22:**1 X**	-0.2400	-0.3840 to -0.09604	Yes
21076	20:**50 X** vs. 22:**10 X**	-0.2353	-0.3793 to -0.09137	Yes
21077	20:**50 X** vs. 22:**40 X**	-0.1357	-0.2796 to 0.008293	No
21078	20:**50 X** vs. 22:**50 X**	-0.1983	-0.3423 to -0.05437	Yes
21079	20:**50 X** vs. 23:**0 X**	-0.3107	-0.4546 to -0.1667	Yes
21080	20:**50 X** vs. 23:**1 X**	-0.5003	-0.6613 to -0.3394	Yes
21081	20:**50 X** vs. 23:**10 X**	-0.2500	-0.3940 to -0.1060	Yes
21082	20:**50 X** vs. 23:**40 X**	-0.1303	-0.2913 to 0.03062	No
21083	20:**50 X** vs. 23:**50 X**	-0.1643	-0.3083 to -0.02037	Yes
21084	20:**50 X** vs. 24:**0 X**	-0.4280	-0.5720 to -0.2840	Yes
21085	20:**50 X** vs. 24:**1 X**	-0.5843	-0.7453 to -0.4234	Yes
21086	20:**50 X** vs. 24:**10 X**	-0.4118	-0.5728 to -0.2509	Yes
21087	20:**50 X** vs. 24:**40 X**	-0.1467	-0.2906 to -0.002707	Yes
21088	20:**50 X** vs. 24:**50 X**	-0.06833	-0.2123 to 0.07563	No
21089	20:**50 X** vs. 25:**0 X**	-0.7653	-0.9263 to -0.6044	Yes
21090	20:**50 X** vs. 25:**1 X**	-0.9548	-1.116 to -0.7939	Yes
21091	20:**50 X** vs. 25:**10 X**	-0.3163	-0.5199 to -0.1127	Yes
21092	20:**50 X** vs. 25:**40 X**	-0.1487	-0.2926 to -0.004707	Yes
21093	20:**50 X** vs. 25:**50 X**	-0.1230	-0.2670 to 0.02096	No
21094	20:**50 X** vs. 26:**0 X**	-0.9778	-1.139 to -0.8169	Yes
21095	20:**50 X** vs. 26:**1 X**	-1.091	-1.252 to -0.9299	Yes
21096	20:**50 X** vs. 26:**10 X**	-0.7773	-0.9383 to -0.6164	Yes
21097	20:**50 X** vs. 26:**40 X**	-0.2230	-0.3670 to -0.07904	Yes
21098	20:**50 X** vs. 26:**50 X**	-0.08333	-0.2273 to 0.06063	No
21099	20:**50 X** vs. 27:**0 X**	-1.166	-1.310 to -1.022	Yes
21100	20:**50 X** vs. 27:**1 X**	-1.256	-1.417 to -1.095	Yes
21101	20:**50 X** vs. 27:**10 X**	-1.068	-1.229 to -0.9074	Yes
21102	20:**50 X** vs. 27:**40 X**	-0.2230	-0.3670 to -0.07904	Yes
21103	20:**50 X** vs. 27:**50 X**	-0.08267	-0.2266 to 0.06129	No
21104	20:**50 X** vs. 28:**0 X**	-1.115	-1.276 to -0.9539	Yes
21105	20:**50 X** vs. 28:**1 X**	-1.156	-1.300 to -1.012	Yes

2way ANOVA<br>Multiple comparisons	A<br>Data Set-A<br>Y	B<br>Data Set-B<br>Y	C<br>Data Set-C<br>Y
21106 20:50 X vs. 28:10 X	-0.9573	-1.118 to -0.7964	Yes
21107 20:50 X vs. 28:40 X	-0.2353	-0.3793 to -0.09137	Yes
21108 20:50 X vs. 28:50 X	-0.1307	-0.2746 to 0.01329	No
21109 20:50 X vs. 29:0 X	-1.596	-1.757 to -1.435	Yes
21110 20:50 X vs. 29:1 X	-1.681	-1.842 to -1.520	Yes
21111 20:50 X vs. 29:10 X	-0.8958	-1.057 to -0.7349	Yes
21112 20:50 X vs. 29:40 X	-0.3433	-0.4873 to -0.1994	Yes
21113 20:50 X vs. 29:50 X	-0.1320	-0.2760 to 0.01196	No
21114 20:50 X vs. 30:0 X	-1.311	-1.472 to -1.150	Yes
21115 20:50 X vs. 30:1 X	-1.632	-1.793 to -1.471	Yes
21116 20:50 X vs. 30:10 X	-0.9703	-1.131 to -0.8094	Yes
21117 20:50 X vs. 30:40 X	-0.2703	-0.4143 to -0.1264	Yes
21118 20:50 X vs. 30:50 X	-0.1843	-0.3283 to -0.04037	Yes
21119 20:50 X vs. 31:0 X	-1.629	-1.790 to -1.468	Yes
21120 20:50 X vs. 31:1 X	-1.619	-1.780 to -1.458	Yes
21121 20:50 X vs. 31:10 X	-1.102	-1.263 to -0.9409	Yes
21122 20:50 X vs. 31:40 X	-0.3620	-0.5060 to -0.2180	Yes
21123 20:50 X vs. 31:50 X	-0.1633	-0.3073 to -0.01937	Yes
21124 20:50 X vs. 32:0 X	-2.220	-2.364 to -2.076	Yes
21125 20:50 X vs. 32:1 X	-1.676	-1.837 to -1.515	Yes
21126 20:50 X vs. 32:10 X	-1.532	-1.676 to -1.388	Yes
21127 20:50 X vs. 32:40 X	-0.3583	-0.5023 to -0.2144	Yes
21128 20:50 X vs. 32:50 X	-0.1630	-0.3070 to -0.01904	Yes
21129 20:50 X vs. 33:0 X	-2.224	-2.368 to -2.080	Yes
21130 20:50 X vs. 33:1 X	-2.039	-2.183 to -1.895	Yes
21131 20:50 X vs. 33:10 X	-1.699	-1.843 to -1.555	Yes
21132 20:50 X vs. 33:40 X	-0.3770	-0.5210 to -0.2330	Yes
21133 20:50 X vs. 33:50 X	-0.1780	-0.3220 to -0.03404	Yes
21134 20:50 X vs. 34:0 X	-2.149	-2.293 to -2.005	Yes
21135 20:50 X vs. 34:1 X	-2.156	-2.300 to -2.012	Yes
21136 20:50 X vs. 34:10 X	-1.789	-1.933 to -1.645	Yes
21137 20:50 X vs. 34:40 X	-0.3890	-0.5330 to -0.2450	Yes
21138 20:50 X vs. 34:50 X	-0.3270	-0.4710 to -0.1830	Yes
21139 20:50 X vs. 35:0 X	-2.497	-2.641 to -2.353	Yes
21140 20:50 X vs. 35:1 X	-2.263	-2.407 to -2.119	Yes
21141 20:50 X vs. 35:10 X	-1.751	-1.895 to -1.607	Yes
21142 20:50 X vs. 35:40 X	-0.3980	-0.5420 to -0.2540	Yes
21143 20:50 X vs. 35:50 X	-0.3397	-0.4836 to -0.1957	Yes
21144 20:50 X vs. 36:0 X	-2.704	-2.865 to -2.543	Yes
21145 20:50 X vs. 36:1 X	-2.400	-2.544 to -2.256	Yes
21146 20:50 X vs. 36:10 X	-1.757	-1.901 to -1.613	Yes
21147 20:50 X vs. 36:40 X	-0.4533	-0.5973 to -0.3094	Yes
21148 20:50 X vs. 36:50 X	-0.3030	-0.4470 to -0.1590	Yes
21149 20:50 X vs. 37:0 X	-3.088	-3.232 to -2.944	Yes
21150 20:50 X vs. 37:1 X	-2.470	-2.614 to -2.326	Yes

2way ANOVA<br>Multiple comparisons	A<br>Data Set-A<br>Y	B<br>Data Set-B<br>Y	C<br>Data Set-C<br>Y
21151 20:**50 X** vs. 37:**10 X**	-1.758	-1.902 to -1.614	Yes
21152 20:**50 X** vs. 37:**40 X**	-0.4907	-0.6346 to -0.3467	Yes
21153 20:**50 X** vs. 37:**50 X**	-0.3117	-0.4556 to -0.1677	Yes
21154 20:**50 X** vs. 38:**0 X**	-3.033	-3.177 to -2.889	Yes
21155 20:**50 X** vs. 38:**1 X**	-2.588	-2.732 to -2.444	Yes
21156 20:**50 X** vs. 38:**10 X**	-2.034	-2.178 to -1.890	Yes
21157 20:**50 X** vs. 38:**40 X**	-0.4950	-0.6390 to -0.3510	Yes
21158 20:**50 X** vs. 38:**50 X**	-0.3157	-0.4596 to -0.1717	Yes
21159 20:**50 X** vs. 39:**0 X**	-3.263	-3.407 to -3.119	Yes
21160 20:**50 X** vs. 39:**1 X**	-3.155	-3.299 to -3.011	Yes
21161 20:**50 X** vs. 39:**10 X**	-2.179	-2.323 to -2.035	Yes
21162 20:**50 X** vs. 39:**40 X**	-0.5010	-0.6450 to -0.3570	Yes
21163 20:**50 X** vs. 39:**50 X**	-0.3467	-0.4906 to -0.2027	Yes
21164 20:**50 X** vs. 40:**0 X**	-3.304	-3.448 to -3.160	Yes
21165 20:**50 X** vs. 40:**1 X**	-3.230	-3.374 to -3.086	Yes
21166 20:**50 X** vs. 40:**10 X**	-2.435	-2.579 to -2.291	Yes
21167 20:**50 X** vs. 40:**40 X**	-0.5057	-0.6496 to -0.3617	Yes
21168 20:**50 X** vs. 40:**50 X**	-0.3530	-0.4970 to -0.2090	Yes
21169 20:**50 X** vs. 41:**0 X**	-3.345	-3.489 to -3.201	Yes
21170 20:**50 X** vs. 41:**1 X**	-3.344	-3.488 to -3.200	Yes
21171 20:**50 X** vs. 41:**10 X**	-2.489	-2.633 to -2.345	Yes
21172 20:**50 X** vs. 41:**40 X**	-0.4530	-0.5970 to -0.3090	Yes
21173 20:**50 X** vs. 41:**50 X**	-0.3657	-0.5096 to -0.2217	Yes
21174 20:**50 X** vs. 42:**0 X**	-3.487	-3.631 to -3.343	Yes
21175 20:**50 X** vs. 42:**1 X**	-3.507	-3.651 to -3.363	Yes
21176 20:**50 X** vs. 42:**10 X**	-2.489	-2.633 to -2.345	Yes
21177 20:**50 X** vs. 42:**40 X**	-0.4657	-0.6096 to -0.3217	Yes
21178 20:**50 X** vs. 42:**50 X**	-0.3690	-0.5130 to -0.2250	Yes
21179 20:**50 X** vs. 43:**0 X**	-3.577	-3.721 to -3.433	Yes
21180 20:**50 X** vs. 43:**1 X**	-3.491	-3.635 to -3.347	Yes
21181 20:**50 X** vs. 43:**10 X**	-3.207	-3.351 to -3.063	Yes
21182 20:**50 X** vs. 43:**40 X**	-0.4640	-0.6080 to -0.3200	Yes
21183 20:**50 X** vs. 43:**50 X**	-0.3693	-0.5133 to -0.2254	Yes
21184 20:**50 X** vs. 44:**0 X**	-3.601	-3.745 to -3.457	Yes
21185 20:**50 X** vs. 44:**1 X**	-3.491	-3.635 to -3.347	Yes
21186 20:**50 X** vs. 44:**10 X**	-3.270	-3.414 to -3.126	Yes
21187 20:**50 X** vs. 44:**40 X**	-0.4660	-0.6100 to -0.3220	Yes
21188 20:**50 X** vs. 44:**50 X**	-0.3017	-0.4456 to -0.1577	Yes
21189 20:**50 X** vs. 45:**0 X**	-3.466	-3.610 to -3.322	Yes
21190 20:**50 X** vs. 45:**1 X**	-3.268	-3.412 to -3.124	Yes
21191 20:**50 X** vs. 45:**10 X**	-3.150	-3.294 to -3.006	Yes
21192 20:**50 X** vs. 45:**40 X**	-0.4673	-0.6113 to -0.3234	Yes
21193 20:**50 X** vs. 45:**50 X**	-0.2987	-0.4426 to -0.1547	Yes
21194 20:**50 X** vs. 46:**0 X**	-3.270	-3.414 to -3.126	Yes
21195 20:**50 X** vs. 46:**1 X**	-3.237	-3.381 to -3.093	Yes

	2way ANOVA<br>Multiple comparisons	A<br>Data Set-A<br>Y	B<br>Data Set-B<br>Y	C<br>Data Set-C<br>Y
21196	20:50 X vs. 46:10 X	-3.145	-3.289 to -3.001	Yes
21197	20:50 X vs. 46:40 X	-0.4810	-0.6250 to -0.3370	Yes
21198	20:50 X vs. 46:50 X	-0.2917	-0.4356 to -0.1477	Yes
21199	20:50 X vs. 47:0 X	-3.246	-3.390 to -3.102	Yes
21200	20:50 X vs. 47:1 X	-3.150	-3.294 to -3.006	Yes
21201	20:50 X vs. 47:10 X	-3.131	-3.275 to -2.987	Yes
21202	20:50 X vs. 47:40 X	-0.4910	-0.6350 to -0.3470	Yes
21203	20:50 X vs. 47:50 X	-0.2497	-0.3936 to -0.1057	Yes
21204	20:50 X vs. 48:0 X	-3.233	-3.394 to -3.072	Yes
21205	20:50 X vs. 48:1 X	-3.137	-3.281 to -2.993	Yes
21206	20:50 X vs. 48:10 X	-3.131	-3.275 to -2.987	Yes
21207	20:50 X vs. 48:40 X	-0.4973	-0.6413 to -0.3534	Yes
21208	20:50 X vs. 48:50 X	-0.2570	-0.4010 to -0.1130	Yes
21209	20:50 X vs. 49:0 X	-3.233	-3.377 to -3.089	Yes
21210	20:50 X vs. 49:1 X	-3.137	-3.281 to -2.993	Yes
21211	20:50 X vs. 49:10 X	-3.131	-3.275 to -2.987	Yes
21212	20:50 X vs. 49:40 X	-0.5017	-0.6456 to -0.3577	Yes
21213	20:50 X vs. 49:50 X	-0.2563	-0.4003 to -0.1124	Yes
21214	20:50 X vs. 50:0 X	-3.233	-3.377 to -3.089	Yes
21215	20:50 X vs. 50:1 X	-3.137	-3.281 to -2.993	Yes
21216	20:50 X vs. 50:10 X	-3.131	-3.275 to -2.987	Yes
21217	20:50 X vs. 50:40 X	-0.5093	-0.6533 to -0.3654	Yes
21218	20:50 X vs. 50:50 X	-0.2560	-0.4000 to -0.1120	Yes
21219	21:0 X vs. 21:1 X	0.0470	-0.09696 to 0.1910	No
21220	21:0 X vs. 21:10 X	0.05000	-0.09396 to 0.1940	No
21221	21:0 X vs. 21:40 X	0.1747	0.03071 to 0.3186	Yes
21222	21:0 X vs. 21:50 X	0.08433	-0.05963 to 0.2283	No
21223	21:0 X vs. 22:0 X	-0.04167	-0.1856 to 0.1023	No
21224	21:0 X vs. 22:1 X	-0.03633	-0.1803 to 0.1076	No
21225	21:0 X vs. 22:10 X	-0.03167	-0.1756 to 0.1123	No
21226	21:0 X vs. 22:40 X	0.06800	-0.07596 to 0.2120	No
21227	21:0 X vs. 22:50 X	0.005333	-0.1386 to 0.1493	No
21228	21:0 X vs. 23:0 X	-0.1070	-0.2510 to 0.03696	No
21229	21:0 X vs. 23:1 X	-0.2967	-0.4576 to -0.1357	Yes
21230	21:0 X vs. 23:10 X	-0.04633	-0.1903 to 0.09763	No
21231	21:0 X vs. 23:40 X	0.07333	-0.08762 to 0.2343	No
21232	21:0 X vs. 23:50 X	0.03933	-0.1046 to 0.1833	No
21233	21:0 X vs. 24:0 X	-0.2243	-0.3683 to -0.08037	Yes
21234	21:0 X vs. 24:1 X	-0.3807	-0.5416 to -0.2197	Yes
21235	21:0 X vs. 24:10 X	-0.2082	-0.3691 to -0.04721	Yes
21236	21:0 X vs. 24:40 X	0.05700	-0.08696 to 0.2010	No
21237	21:0 X vs. 24:50 X	0.1353	-0.008626 to 0.2793	No
21238	21:0 X vs. 25:0 X	-0.5617	-0.7226 to -0.4007	Yes
21239	21:0 X vs. 25:1 X	-0.7512	-0.9121 to -0.5902	Yes
21240	21:0 X vs. 25:10 X	-0.1127	-0.3163 to 0.09092	No

	2way ANOVA<br>Multiple comparisons	A<br>Data Set-A<br>Y	B<br>Data Set-B<br>Y	C<br>Data Set-C<br>Y
21241	21:**0 X** vs. 25:**40 X**	0.0550	-0.08896 to 0.1990	No
21242	21:**0 X** vs. 25:**50 X**	0.08067	-0.06329 to 0.2246	No
21243	21:**0 X** vs. 26:**0 X**	-0.7742	-0.9351 to -0.6132	Yes
21244	21:**0 X** vs. 26:**1 X**	-0.8872	-1.048 to -0.7262	Yes
21245	21:**0 X** vs. 26:**10 X**	-0.5737	-0.7346 to -0.4127	Yes
21246	21:**0 X** vs. 26:**40 X**	-0.01933	-0.1633 to 0.1246	No
21247	21:**0 X** vs. 26:**50 X**	0.1203	-0.02363 to 0.2643	No
21248	21:**0 X** vs. 27:**0 X**	-0.9620	-1.106 to -0.8180	Yes
21249	21:**0 X** vs. 27:**1 X**	-1.053	-1.214 to -0.8917	Yes
21250	21:**0 X** vs. 27:**10 X**	-0.8647	-1.026 to -0.7037	Yes
21251	21:**0 X** vs. 27:**40 X**	-0.01933	-0.1633 to 0.1246	No
21252	21:**0 X** vs. 27:**50 X**	0.1210	-0.02296 to 0.2650	No
21253	21:**0 X** vs. 28:**0 X**	-0.9112	-1.072 to -0.7502	Yes
21254	21:**0 X** vs. 28:**1 X**	-0.9520	-1.096 to -0.8080	Yes
21255	21:**0 X** vs. 28:**10 X**	-0.7537	-0.9146 to -0.5927	Yes
21256	21:**0 X** vs. 28:**40 X**	-0.03167	-0.1756 to 0.1123	No
21257	21:**0 X** vs. 28:**50 X**	0.07300	-0.07096 to 0.2170	No
21258	21:**0 X** vs. 29:**0 X**	-1.393	-1.554 to -1.232	Yes
21259	21:**0 X** vs. 29:**1 X**	-1.478	-1.639 to -1.317	Yes
21260	21:**0 X** vs. 29:**10 X**	-0.6922	-0.8531 to -0.5312	Yes
21261	21:**0 X** vs. 29:**40 X**	-0.1397	-0.2836 to 0.004293	No
21262	21:**0 X** vs. 29:**50 X**	0.07167	-0.07229 to 0.2156	No
21263	21:**0 X** vs. 30:**0 X**	-1.107	-1.268 to -0.9462	Yes
21264	21:**0 X** vs. 30:**1 X**	-1.429	-1.590 to -1.268	Yes
21265	21:**0 X** vs. 30:**10 X**	-0.7667	-0.9276 to -0.6057	Yes
21266	21:**0 X** vs. 30:**40 X**	-0.06667	-0.2106 to 0.07729	No
21267	21:**0 X** vs. 30:**50 X**	0.01933	-0.1246 to 0.1633	No
21268	21:**0 X** vs. 31:**0 X**	-1.425	-1.586 to -1.264	Yes
21269	21:**0 X** vs. 31:**1 X**	-1.416	-1.577 to -1.255	Yes
21270	21:**0 X** vs. 31:**10 X**	-0.8982	-1.059 to -0.7372	Yes
21271	21:**0 X** vs. 31:**40 X**	-0.1583	-0.3023 to -0.01437	Yes
21272	21:**0 X** vs. 31:**50 X**	0.04033	-0.1036 to 0.1843	No
21273	21:**0 X** vs. 32:**0 X**	-2.017	-2.161 to -1.873	Yes
21274	21:**0 X** vs. 32:**1 X**	-1.473	-1.634 to -1.312	Yes
21275	21:**0 X** vs. 32:**10 X**	-1.329	-1.473 to -1.185	Yes
21276	21:**0 X** vs. 32:**40 X**	-0.1547	-0.2986 to -0.01071	Yes
21277	21:**0 X** vs. 32:**50 X**	0.04067	-0.1033 to 0.1846	No
21278	21:**0 X** vs. 33:**0 X**	-2.020	-2.164 to -1.876	Yes
21279	21:**0 X** vs. 33:**1 X**	-1.835	-1.979 to -1.691	Yes
21280	21:**0 X** vs. 33:**10 X**	-1.495	-1.639 to -1.351	Yes
21281	21:**0 X** vs. 33:**40 X**	-0.1733	-0.3173 to -0.02937	Yes
21282	21:**0 X** vs. 33:**50 X**	0.02567	-0.1183 to 0.1696	No
21283	21:**0 X** vs. 34:**0 X**	-1.945	-2.089 to -1.801	Yes
21284	21:**0 X** vs. 34:**1 X**	-1.952	-2.096 to -1.808	Yes
21285	21:**0 X** vs. 34:**10 X**	-1.586	-1.730 to -1.442	Yes

	2way ANOVA<br>Multiple comparisons	A<br>Data Set-A<br>Y	B<br>Data Set-B<br>Y	C<br>Data Set-C<br>Y
21286	21:**0 X** vs. 34:**40 X**	-0.1853	-0.3293 to -0.04137	Yes
21287	21:**0 X** vs. 34:**50 X**	-0.1233	-0.2673 to 0.02063	No
21288	21:**0 X** vs. 35:**0 X**	-2.294	-2.438 to -2.150	Yes
21289	21:**0 X** vs. 35:**1 X**	-2.059	-2.203 to -1.915	Yes
21290	21:**0 X** vs. 35:**10 X**	-1.547	-1.691 to -1.403	Yes
21291	21:**0 X** vs. 35:**40 X**	-0.1943	-0.3383 to -0.05037	Yes
21292	21:**0 X** vs. 35:**50 X**	-0.1360	-0.2800 to 0.007960	No
21293	21:**0 X** vs. 36:**0 X**	-2.500	-2.661 to -2.339	Yes
21294	21:**0 X** vs. 36:**1 X**	-2.196	-2.340 to -2.052	Yes
21295	21:**0 X** vs. 36:**10 X**	-1.553	-1.697 to -1.409	Yes
21296	21:**0 X** vs. 36:**40 X**	-0.2497	-0.3936 to -0.1057	Yes
21297	21:**0 X** vs. 36:**50 X**	-0.09933	-0.2433 to 0.04463	No
21298	21:**0 X** vs. 37:**0 X**	-2.884	-3.028 to -2.740	Yes
21299	21:**0 X** vs. 37:**1 X**	-2.266	-2.410 to -2.122	Yes
21300	21:**0 X** vs. 37:**10 X**	-1.555	-1.699 to -1.411	Yes
21301	21:**0 X** vs. 37:**40 X**	-0.2870	-0.4310 to -0.1430	Yes
21302	21:**0 X** vs. 37:**50 X**	-0.1080	-0.2520 to 0.03596	No
21303	21:**0 X** vs. 38:**0 X**	-2.829	-2.973 to -2.685	Yes
21304	21:**0 X** vs. 38:**1 X**	-2.385	-2.529 to -2.241	Yes
21305	21:**0 X** vs. 38:**10 X**	-1.830	-1.974 to -1.686	Yes
21306	21:**0 X** vs. 38:**40 X**	-0.2913	-0.4353 to -0.1474	Yes
21307	21:**0 X** vs. 38:**50 X**	-0.1120	-0.2560 to 0.03196	No
21308	21:**0 X** vs. 39:**0 X**	-3.060	-3.204 to -2.916	Yes
21309	21:**0 X** vs. 39:**1 X**	-2.951	-3.095 to -2.807	Yes
21310	21:**0 X** vs. 39:**10 X**	-1.975	-2.119 to -1.831	Yes
21311	21:**0 X** vs. 39:**40 X**	-0.2973	-0.4413 to -0.1534	Yes
21312	21:**0 X** vs. 39:**50 X**	-0.1430	-0.2870 to 0.0009598	No
21313	21:**0 X** vs. 40:**0 X**	-3.101	-3.245 to -2.957	Yes
21314	21:**0 X** vs. 40:**1 X**	-3.027	-3.171 to -2.883	Yes
21315	21:**0 X** vs. 40:**10 X**	-2.231	-2.375 to -2.087	Yes
21316	21:**0 X** vs. 40:**40 X**	-0.3020	-0.4460 to -0.1580	Yes
21317	21:**0 X** vs. 40:**50 X**	-0.1493	-0.2933 to -0.005374	Yes
21318	21:**0 X** vs. 41:**0 X**	-3.142	-3.286 to -2.998	Yes
21319	21:**0 X** vs. 41:**1 X**	-3.141	-3.285 to -2.997	Yes
21320	21:**0 X** vs. 41:**10 X**	-2.285	-2.429 to -2.141	Yes
21321	21:**0 X** vs. 41:**40 X**	-0.2493	-0.3933 to -0.1054	Yes
21322	21:**0 X** vs. 41:**50 X**	-0.1620	-0.3060 to -0.01804	Yes
21323	21:**0 X** vs. 42:**0 X**	-3.283	-3.427 to -3.139	Yes
21324	21:**0 X** vs. 42:**1 X**	-3.303	-3.447 to -3.159	Yes
21325	21:**0 X** vs. 42:**10 X**	-2.285	-2.429 to -2.141	Yes
21326	21:**0 X** vs. 42:**40 X**	-0.2620	-0.4060 to -0.1180	Yes
21327	21:**0 X** vs. 42:**50 X**	-0.1653	-0.3093 to -0.02137	Yes
21328	21:**0 X** vs. 43:**0 X**	-3.373	-3.517 to -3.229	Yes
21329	21:**0 X** vs. 43:**1 X**	-3.288	-3.432 to -3.144	Yes
21330	21:**0 X** vs. 43:**10 X**	-3.003	-3.147 to -2.859	Yes

2way ANOVA<br>Multiple comparisons	A<br>Data Set-A<br>Y	B<br>Data Set-B<br>Y	C<br>Data Set-C<br>Y
21331 21:**0 X** vs. 43:**40 X**	-0.2603	-0.4043 to -0.1164	Yes
21332 21:**0 X** vs. 43:**50 X**	-0.1657	-0.3096 to -0.02171	Yes
21333 21:**0 X** vs. 44:**0 X**	-3.397	-3.541 to -3.253	Yes
21334 21:**0 X** vs. 44:**1 X**	-3.287	-3.431 to -3.143	Yes
21335 21:**0 X** vs. 44:**10 X**	-3.067	-3.211 to -2.923	Yes
21336 21:**0 X** vs. 44:**40 X**	-0.2623	-0.4063 to -0.1184	Yes
21337 21:**0 X** vs. 44:**50 X**	-0.0980	-0.2420 to 0.04596	No
21338 21:**0 X** vs. 45:**0 X**	-3.262	-3.406 to -3.118	Yes
21339 21:**0 X** vs. 45:**1 X**	-3.064	-3.208 to -2.920	Yes
21340 21:**0 X** vs. 45:**10 X**	-2.946	-3.090 to -2.802	Yes
21341 21:**0 X** vs. 45:**40 X**	-0.2637	-0.4076 to -0.1197	Yes
21342 21:**0 X** vs. 45:**50 X**	-0.0950	-0.2390 to 0.04896	No
21343 21:**0 X** vs. 46:**0 X**	-3.066	-3.210 to -2.922	Yes
21344 21:**0 X** vs. 46:**1 X**	-3.033	-3.177 to -2.889	Yes
21345 21:**0 X** vs. 46:**10 X**	-2.941	-3.085 to -2.797	Yes
21346 21:**0 X** vs. 46:**40 X**	-0.2773	-0.4213 to -0.1334	Yes
21347 21:**0 X** vs. 46:**50 X**	-0.08800	-0.2320 to 0.05596	No
21348 21:**0 X** vs. 47:**0 X**	-3.042	-3.186 to -2.898	Yes
21349 21:**0 X** vs. 47:**1 X**	-2.946	-3.090 to -2.802	Yes
21350 21:**0 X** vs. 47:**10 X**	-2.927	-3.071 to -2.783	Yes
21351 21:**0 X** vs. 47:**40 X**	-0.2873	-0.4313 to -0.1434	Yes
21352 21:**0 X** vs. 47:**50 X**	-0.0460	-0.1900 to 0.09796	No
21353 21:**0 X** vs. 48:**0 X**	-3.029	-3.190 to -2.868	Yes
21354 21:**0 X** vs. 48:**1 X**	-2.933	-3.077 to -2.789	Yes
21355 21:**0 X** vs. 48:**10 X**	-2.927	-3.071 to -2.783	Yes
21356 21:**0 X** vs. 48:**40 X**	-0.2937	-0.4376 to -0.1497	Yes
21357 21:**0 X** vs. 48:**50 X**	-0.05333	-0.1973 to 0.09063	No
21358 21:**0 X** vs. 49:**0 X**	-3.029	-3.173 to -2.885	Yes
21359 21:**0 X** vs. 49:**1 X**	-2.933	-3.077 to -2.789	Yes
21360 21:**0 X** vs. 49:**10 X**	-2.927	-3.071 to -2.783	Yes
21361 21:**0 X** vs. 49:**40 X**	-0.2980	-0.4420 to -0.1540	Yes
21362 21:**0 X** vs. 49:**50 X**	-0.05267	-0.1966 to 0.09129	No
21363 21:**0 X** vs. 50:**0 X**	-3.029	-3.173 to -2.885	Yes
21364 21:**0 X** vs. 50:**1 X**	-2.933	-3.077 to -2.789	Yes
21365 21:**0 X** vs. 50:**10 X**	-2.927	-3.071 to -2.783	Yes
21366 21:**0 X** vs. 50:**40 X**	-0.3057	-0.4496 to -0.1617	Yes
21367 21:**0 X** vs. 50:**50 X**	-0.05233	-0.1963 to 0.09163	No
21368 21:**1 X** vs. 21:**10 X**	0.003000	-0.1410 to 0.1470	No
21369 21:**1 X** vs. 21:**40 X**	0.1277	-0.01629 to 0.2716	No
21370 21:**1 X** vs. 21:**50 X**	0.03733	-0.1066 to 0.1813	No
21371 21:**1 X** vs. 22:**0 X**	-0.08867	-0.2326 to 0.05529	No
21372 21:**1 X** vs. 22:**1 X**	-0.08333	-0.2273 to 0.06063	No
21373 21:**1 X** vs. 22:**10 X**	-0.07867	-0.2226 to 0.06529	No
21374 21:**1 X** vs. 22:**40 X**	0.02100	-0.1230 to 0.1650	No
21375 21:**1 X** vs. 22:**50 X**	-0.04167	-0.1856 to 0.1023	No

	2way ANOVA<br>Multiple comparisons	A<br>Data Set-A<br>Y	B<br>Data Set-B<br>Y	C<br>Data Set-C<br>Y
21376	21:**1 X** vs. 23:**0 X**	-0.1540	-0.2980 to -0.01004	Yes
21377	21:**1 X** vs. 23:**1 X**	-0.3437	-0.5046 to -0.1827	Yes
21378	21:**1 X** vs. 23:**10 X**	-0.09333	-0.2373 to 0.05063	No
21379	21:**1 X** vs. 23:**40 X**	0.02633	-0.1346 to 0.1873	No
21380	21:**1 X** vs. 23:**50 X**	-0.007667	-0.1516 to 0.1363	No
21381	21:**1 X** vs. 24:**0 X**	-0.2713	-0.4153 to -0.1274	Yes
21382	21:**1 X** vs. 24:**1 X**	-0.4277	-0.5886 to -0.2667	Yes
21383	21:**1 X** vs. 24:**10 X**	-0.2552	-0.4161 to -0.09421	Yes
21384	21:**1 X** vs. 24:**40 X**	0.01000	-0.1340 to 0.1540	No
21385	21:**1 X** vs. 24:**50 X**	0.08833	-0.05563 to 0.2323	No
21386	21:**1 X** vs. 25:**0 X**	-0.6087	-0.7696 to -0.4477	Yes
21387	21:**1 X** vs. 25:**1 X**	-0.7982	-0.9591 to -0.6372	Yes
21388	21:**1 X** vs. 25:**10 X**	-0.1597	-0.3633 to 0.04392	No
21389	21:**1 X** vs. 25:**40 X**	0.008000	-0.1360 to 0.1520	No
21390	21:**1 X** vs. 25:**50 X**	0.03367	-0.1103 to 0.1776	No
21391	21:**1 X** vs. 26:**0 X**	-0.8212	-0.9821 to -0.6602	Yes
21392	21:**1 X** vs. 26:**1 X**	-0.9342	-1.095 to -0.7732	Yes
21393	21:**1 X** vs. 26:**10 X**	-0.6207	-0.7816 to -0.4597	Yes
21394	21:**1 X** vs. 26:**40 X**	-0.06633	-0.2103 to 0.07763	No
21395	21:**1 X** vs. 26:**50 X**	0.07333	-0.07063 to 0.2173	No
21396	21:**1 X** vs. 27:**0 X**	-1.009	-1.153 to -0.8650	Yes
21397	21:**1 X** vs. 27:**1 X**	-1.100	-1.261 to -0.9387	Yes
21398	21:**1 X** vs. 27:**10 X**	-0.9117	-1.073 to -0.7507	Yes
21399	21:**1 X** vs. 27:**40 X**	-0.06633	-0.2103 to 0.07763	No
21400	21:**1 X** vs. 27:**50 X**	0.07400	-0.06996 to 0.2180	No
21401	21:**1 X** vs. 28:**0 X**	-0.9582	-1.119 to -0.7972	Yes
21402	21:**1 X** vs. 28:**1 X**	-0.9990	-1.143 to -0.8550	Yes
21403	21:**1 X** vs. 28:**10 X**	-0.8007	-0.9616 to -0.6397	Yes
21404	21:**1 X** vs. 28:**40 X**	-0.07867	-0.2226 to 0.06529	No
21405	21:**1 X** vs. 28:**50 X**	0.0260	-0.1180 to 0.1700	No
21406	21:**1 X** vs. 29:**0 X**	-1.440	-1.601 to -1.279	Yes
21407	21:**1 X** vs. 29:**1 X**	-1.525	-1.686 to -1.364	Yes
21408	21:**1 X** vs. 29:**10 X**	-0.7392	-0.9001 to -0.5782	Yes
21409	21:**1 X** vs. 29:**40 X**	-0.1867	-0.3306 to -0.04271	Yes
21410	21:**1 X** vs. 29:**50 X**	0.02467	-0.1193 to 0.1686	No
21411	21:**1 X** vs. 30:**0 X**	-1.154	-1.315 to -0.9932	Yes
21412	21:**1 X** vs. 30:**1 X**	-1.476	-1.637 to -1.315	Yes
21413	21:**1 X** vs. 30:**10 X**	-0.8137	-0.9746 to -0.6527	Yes
21414	21:**1 X** vs. 30:**40 X**	-0.1137	-0.2576 to 0.03029	No
21415	21:**1 X** vs. 30:**50 X**	-0.02767	-0.1716 to 0.1163	No
21416	21:**1 X** vs. 31:**0 X**	-1.472	-1.633 to -1.311	Yes
21417	21:**1 X** vs. 31:**1 X**	-1.463	-1.624 to -1.302	Yes
21418	21:**1 X** vs. 31:**10 X**	-0.9452	-1.106 to -0.7842	Yes
21419	21:**1 X** vs. 31:**40 X**	-0.2053	-0.3493 to -0.06137	Yes
21420	21:**1 X** vs. 31:**50 X**	-0.006667	-0.1506 to 0.1373	No

2way ANOVA<br>Multiple comparisons		A<br>Data Set-A<br>Y	B<br>Data Set-B<br>Y	C<br>Data Set-C<br>Y
21421	21:1 X vs. 32:0 X	-2.064	-2.208 to -1.920	Yes
21422	21:1 X vs. 32:1 X	-1.520	-1.681 to -1.359	Yes
21423	21:1 X vs. 32:10 X	-1.376	-1.520 to -1.232	Yes
21424	21:1 X vs. 32:40 X	-0.2017	-0.3456 to -0.05771	Yes
21425	21:1 X vs. 32:50 X	-0.006333	-0.1503 to 0.1376	No
21426	21:1 X vs. 33:0 X	-2.067	-2.211 to -1.923	Yes
21427	21:1 X vs. 33:1 X	-1.882	-2.026 to -1.738	Yes
21428	21:1 X vs. 33:10 X	-1.542	-1.686 to -1.398	Yes
21429	21:1 X vs. 33:40 X	-0.2203	-0.3643 to -0.07637	Yes
21430	21:1 X vs. 33:50 X	-0.02133	-0.1653 to 0.1226	No
21431	21:1 X vs. 34:0 X	-1.992	-2.136 to -1.848	Yes
21432	21:1 X vs. 34:1 X	-1.999	-2.143 to -1.855	Yes
21433	21:1 X vs. 34:10 X	-1.633	-1.777 to -1.489	Yes
21434	21:1 X vs. 34:40 X	-0.2323	-0.3763 to -0.08837	Yes
21435	21:1 X vs. 34:50 X	-0.1703	-0.3143 to -0.02637	Yes
21436	21:1 X vs. 35:0 X	-2.341	-2.485 to -2.197	Yes
21437	21:1 X vs. 35:1 X	-2.106	-2.250 to -1.962	Yes
21438	21:1 X vs. 35:10 X	-1.594	-1.738 to -1.450	Yes
21439	21:1 X vs. 35:40 X	-0.2413	-0.3853 to -0.09737	Yes
21440	21:1 X vs. 35:50 X	-0.1830	-0.3270 to -0.03904	Yes
21441	21:1 X vs. 36:0 X	-2.547	-2.708 to -2.386	Yes
21442	21:1 X vs. 36:1 X	-2.243	-2.387 to -2.099	Yes
21443	21:1 X vs. 36:10 X	-1.600	-1.744 to -1.456	Yes
21444	21:1 X vs. 36:40 X	-0.2967	-0.4406 to -0.1527	Yes
21445	21:1 X vs. 36:50 X	-0.1463	-0.2903 to -0.002374	Yes
21446	21:1 X vs. 37:0 X	-2.931	-3.075 to -2.787	Yes
21447	21:1 X vs. 37:1 X	-2.313	-2.457 to -2.169	Yes
21448	21:1 X vs. 37:10 X	-1.602	-1.746 to -1.458	Yes
21449	21:1 X vs. 37:40 X	-0.3340	-0.4780 to -0.1900	Yes
21450	21:1 X vs. 37:50 X	-0.1550	-0.2990 to -0.01104	Yes
21451	21:1 X vs. 38:0 X	-2.876	-3.020 to -2.732	Yes
21452	21:1 X vs. 38:1 X	-2.432	-2.576 to -2.288	Yes
21453	21:1 X vs. 38:10 X	-1.877	-2.021 to -1.733	Yes
21454	21:1 X vs. 38:40 X	-0.3383	-0.4823 to -0.1944	Yes
21455	21:1 X vs. 38:50 X	-0.1590	-0.3030 to -0.01504	Yes
21456	21:1 X vs. 39:0 X	-3.107	-3.251 to -2.963	Yes
21457	21:1 X vs. 39:1 X	-2.998	-3.142 to -2.854	Yes
21458	21:1 X vs. 39:10 X	-2.022	-2.166 to -1.878	Yes
21459	21:1 X vs. 39:40 X	-0.3443	-0.4883 to -0.2004	Yes
21460	21:1 X vs. 39:50 X	-0.1900	-0.3340 to -0.04604	Yes
21461	21:1 X vs. 40:0 X	-3.148	-3.292 to -3.004	Yes
21462	21:1 X vs. 40:1 X	-3.074	-3.218 to -2.930	Yes
21463	21:1 X vs. 40:10 X	-2.278	-2.422 to -2.134	Yes
21464	21:1 X vs. 40:40 X	-0.3490	-0.4930 to -0.2050	Yes
21465	21:1 X vs. 40:50 X	-0.1963	-0.3403 to -0.05237	Yes

	2way ANOVA<br>Multiple comparisons	A<br>Data Set-A<br>Y	B<br>Data Set-B<br>Y	C<br>Data Set-C<br>Y
21466	21:1 X vs. 41:0 X	-3.189	-3.333 to -3.045	Yes
21467	21:1 X vs. 41:1 X	-3.188	-3.332 to -3.044	Yes
21468	21:1 X vs. 41:10 X	-2.332	-2.476 to -2.188	Yes
21469	21:1 X vs. 41:40 X	-0.2963	-0.4403 to -0.1524	Yes
21470	21:1 X vs. 41:50 X	-0.2090	-0.3530 to -0.06504	Yes
21471	21:1 X vs. 42:0 X	-3.330	-3.474 to -3.186	Yes
21472	21:1 X vs. 42:1 X	-3.350	-3.494 to -3.206	Yes
21473	21:1 X vs. 42:10 X	-2.332	-2.476 to -2.188	Yes
21474	21:1 X vs. 42:40 X	-0.3090	-0.4530 to -0.1650	Yes
21475	21:1 X vs. 42:50 X	-0.2123	-0.3563 to -0.06837	Yes
21476	21:1 X vs. 43:0 X	-3.420	-3.564 to -3.276	Yes
21477	21:1 X vs. 43:1 X	-3.335	-3.479 to -3.191	Yes
21478	21:1 X vs. 43:10 X	-3.050	-3.194 to -2.906	Yes
21479	21:1 X vs. 43:40 X	-0.3073	-0.4513 to -0.1634	Yes
21480	21:1 X vs. 43:50 X	-0.2127	-0.3566 to -0.06871	Yes
21481	21:1 X vs. 44:0 X	-3.444	-3.588 to -3.300	Yes
21482	21:1 X vs. 44:1 X	-3.334	-3.478 to -3.190	Yes
21483	21:1 X vs. 44:10 X	-3.114	-3.258 to -2.970	Yes
21484	21:1 X vs. 44:40 X	-0.3093	-0.4533 to -0.1654	Yes
21485	21:1 X vs. 44:50 X	-0.1450	-0.2890 to -0.001040	Yes
21486	21:1 X vs. 45:0 X	-3.309	-3.453 to -3.165	Yes
21487	21:1 X vs. 45:1 X	-3.111	-3.255 to -2.967	Yes
21488	21:1 X vs. 45:10 X	-2.993	-3.137 to -2.849	Yes
21489	21:1 X vs. 45:40 X	-0.3107	-0.4546 to -0.1667	Yes
21490	21:1 X vs. 45:50 X	-0.1420	-0.2860 to 0.001960	No
21491	21:1 X vs. 46:0 X	-3.113	-3.257 to -2.969	Yes
21492	21:1 X vs. 46:1 X	-3.080	-3.224 to -2.936	Yes
21493	21:1 X vs. 46:10 X	-2.988	-3.132 to -2.844	Yes
21494	21:1 X vs. 46:40 X	-0.3243	-0.4683 to -0.1804	Yes
21495	21:1 X vs. 46:50 X	-0.1350	-0.2790 to 0.008960	No
21496	21:1 X vs. 47:0 X	-3.089	-3.233 to -2.945	Yes
21497	21:1 X vs. 47:1 X	-2.993	-3.137 to -2.849	Yes
21498	21:1 X vs. 47:10 X	-2.974	-3.118 to -2.830	Yes
21499	21:1 X vs. 47:40 X	-0.3343	-0.4783 to -0.1904	Yes
21500	21:1 X vs. 47:50 X	-0.0930	-0.2370 to 0.05096	No
21501	21:1 X vs. 48:0 X	-3.076	-3.237 to -2.915	Yes
21502	21:1 X vs. 48:1 X	-2.980	-3.124 to -2.836	Yes
21503	21:1 X vs. 48:10 X	-2.974	-3.118 to -2.830	Yes
21504	21:1 X vs. 48:40 X	-0.3407	-0.4846 to -0.1967	Yes
21505	21:1 X vs. 48:50 X	-0.1003	-0.2443 to 0.04363	No
21506	21:1 X vs. 49:0 X	-3.076	-3.220 to -2.932	Yes
21507	21:1 X vs. 49:1 X	-2.980	-3.124 to -2.836	Yes
21508	21:1 X vs. 49:10 X	-2.974	-3.118 to -2.830	Yes
21509	21:1 X vs. 49:40 X	-0.3450	-0.4890 to -0.2010	Yes
21510	21:1 X vs. 49:50 X	-0.09967	-0.2436 to 0.04429	No

2way ANOVA Multiple comparisons	A Data Set-A Y	B Data Set-B Y	C Data Set-C Y
21511 21:**1 X** vs. 50:**0 X**	-3.076	-3.220 to -2.932	Yes
21512 21:**1 X** vs. 50:**1 X**	-2.980	-3.124 to -2.836	Yes
21513 21:**1 X** vs. 50:**10 X**	-2.974	-3.118 to -2.830	Yes
21514 21:**1 X** vs. 50:**40 X**	-0.3527	-0.4966 to -0.2087	Yes
21515 21:**1 X** vs. 50:**50 X**	-0.09933	-0.2433 to 0.04463	No
21516 21:**10 X** vs. 21:**40 X**	0.1247	-0.01929 to 0.2686	No
21517 21:**10 X** vs. 21:**50 X**	0.03433	-0.1096 to 0.1783	No
21518 21:**10 X** vs. 22:**0 X**	-0.09167	-0.2356 to 0.05229	No
21519 21:**10 X** vs. 22:**1 X**	-0.08633	-0.2303 to 0.05763	No
21520 21:**10 X** vs. 22:**10 X**	-0.08167	-0.2256 to 0.06229	No
21521 21:**10 X** vs. 22:**40 X**	0.01800	-0.1260 to 0.1620	No
21522 21:**10 X** vs. 22:**50 X**	-0.04467	-0.1886 to 0.09929	No
21523 21:**10 X** vs. 23:**0 X**	-0.1570	-0.3010 to -0.01304	Yes
21524 21:**10 X** vs. 23:**1 X**	-0.3467	-0.5076 to -0.1857	Yes
21525 21:**10 X** vs. 23:**10 X**	-0.09633	-0.2403 to 0.04763	No
21526 21:**10 X** vs. 23:**40 X**	0.02333	-0.1376 to 0.1843	No
21527 21:**10 X** vs. 23:**50 X**	-0.01067	-0.1546 to 0.1333	No
21528 21:**10 X** vs. 24:**0 X**	-0.2743	-0.4183 to -0.1304	Yes
21529 21:**10 X** vs. 24:**1 X**	-0.4307	-0.5916 to -0.2697	Yes
21530 21:**10 X** vs. 24:**10 X**	-0.2582	-0.4191 to -0.09721	Yes
21531 21:**10 X** vs. 24:**40 X**	0.007000	-0.1370 to 0.1510	No
21532 21:**10 X** vs. 24:**50 X**	0.08533	-0.05863 to 0.2293	No
21533 21:**10 X** vs. 25:**0 X**	-0.6117	-0.7726 to -0.4507	Yes
21534 21:**10 X** vs. 25:**1 X**	-0.8012	-0.9621 to -0.6402	Yes
21535 21:**10 X** vs. 25:**10 X**	-0.1627	-0.3663 to 0.04092	No
21536 21:**10 X** vs. 25:**40 X**	0.005000	-0.1390 to 0.1490	No
21537 21:**10 X** vs. 25:**50 X**	0.03067	-0.1133 to 0.1746	No
21538 21:**10 X** vs. 26:**0 X**	-0.8242	-0.9851 to -0.6632	Yes
21539 21:**10 X** vs. 26:**1 X**	-0.9372	-1.098 to -0.7762	Yes
21540 21:**10 X** vs. 26:**10 X**	-0.6237	-0.7846 to -0.4627	Yes
21541 21:**10 X** vs. 26:**40 X**	-0.06933	-0.2133 to 0.07463	No
21542 21:**10 X** vs. 26:**50 X**	0.07033	-0.07363 to 0.2143	No
21543 21:**10 X** vs. 27:**0 X**	-1.012	-1.156 to -0.8680	Yes
21544 21:**10 X** vs. 27:**1 X**	-1.103	-1.264 to -0.9417	Yes
21545 21:**10 X** vs. 27:**10 X**	-0.9147	-1.076 to -0.7537	Yes
21546 21:**10 X** vs. 27:**40 X**	-0.06933	-0.2133 to 0.07463	No
21547 21:**10 X** vs. 27:**50 X**	0.0710	-0.07296 to 0.2150	No
21548 21:**10 X** vs. 28:**0 X**	-0.9612	-1.122 to -0.8002	Yes
21549 21:**10 X** vs. 28:**1 X**	-1.002	-1.146 to -0.8580	Yes
21550 21:**10 X** vs. 28:**10 X**	-0.8037	-0.9646 to -0.6427	Yes
21551 21:**10 X** vs. 28:**40 X**	-0.08167	-0.2256 to 0.06229	No
21552 21:**10 X** vs. 28:**50 X**	0.02300	-0.1210 to 0.1670	No
21553 21:**10 X** vs. 29:**0 X**	-1.443	-1.604 to -1.282	Yes
21554 21:**10 X** vs. 29:**1 X**	-1.528	-1.689 to -1.367	Yes
21555 21:**10 X** vs. 29:**10 X**	-0.7422	-0.9031 to -0.5812	Yes

	2way ANOVA<br>Multiple comparisons	A<br>Data Set-A<br>Y	B<br>Data Set-B<br>Y	C<br>Data Set-C<br>Y
21556	21:**10 X** vs. 29:**40 X**	-0.1897	-0.3336 to -0.04571	Yes
21557	21:**10 X** vs. 29:**50 X**	0.02167	-0.1223 to 0.1656	No
21558	21:**10 X** vs. 30:**0 X**	-1.157	-1.318 to -0.9962	Yes
21559	21:**10 X** vs. 30:**1 X**	-1.479	-1.640 to -1.318	Yes
21560	21:**10 X** vs. 30:**10 X**	-0.8167	-0.9776 to -0.6557	Yes
21561	21:**10 X** vs. 30:**40 X**	-0.1167	-0.2606 to 0.02729	No
21562	21:**10 X** vs. 30:**50 X**	-0.03067	-0.1746 to 0.1133	No
21563	21:**10 X** vs. 31:**0 X**	-1.475	-1.636 to -1.314	Yes
21564	21:**10 X** vs. 31:**1 X**	-1.466	-1.627 to -1.305	Yes
21565	21:**10 X** vs. 31:**10 X**	-0.9482	-1.109 to -0.7872	Yes
21566	21:**10 X** vs. 31:**40 X**	-0.2083	-0.3523 to -0.06437	Yes
21567	21:**10 X** vs. 31:**50 X**	-0.009667	-0.1536 to 0.1343	No
21568	21:**10 X** vs. 32:**0 X**	-2.067	-2.211 to -1.923	Yes
21569	21:**10 X** vs. 32:**1 X**	-1.523	-1.684 to -1.362	Yes
21570	21:**10 X** vs. 32:**10 X**	-1.379	-1.523 to -1.235	Yes
21571	21:**10 X** vs. 32:**40 X**	-0.2047	-0.3486 to -0.06071	Yes
21572	21:**10 X** vs. 32:**50 X**	-0.009333	-0.1533 to 0.1346	No
21573	21:**10 X** vs. 33:**0 X**	-2.070	-2.214 to -1.926	Yes
21574	21:**10 X** vs. 33:**1 X**	-1.885	-2.029 to -1.741	Yes
21575	21:**10 X** vs. 33:**10 X**	-1.545	-1.689 to -1.401	Yes
21576	21:**10 X** vs. 33:**40 X**	-0.2233	-0.3673 to -0.07937	Yes
21577	21:**10 X** vs. 33:**50 X**	-0.02433	-0.1683 to 0.1196	No
21578	21:**10 X** vs. 34:**0 X**	-1.995	-2.139 to -1.851	Yes
21579	21:**10 X** vs. 34:**1 X**	-2.002	-2.146 to -1.858	Yes
21580	21:**10 X** vs. 34:**10 X**	-1.636	-1.780 to -1.492	Yes
21581	21:**10 X** vs. 34:**40 X**	-0.2353	-0.3793 to -0.09137	Yes
21582	21:**10 X** vs. 34:**50 X**	-0.1733	-0.3173 to -0.02937	Yes
21583	21:**10 X** vs. 35:**0 X**	-2.344	-2.488 to -2.200	Yes
21584	21:**10 X** vs. 35:**1 X**	-2.109	-2.253 to -1.965	Yes
21585	21:**10 X** vs. 35:**10 X**	-1.597	-1.741 to -1.453	Yes
21586	21:**10 X** vs. 35:**40 X**	-0.2443	-0.3883 to -0.1004	Yes
21587	21:**10 X** vs. 35:**50 X**	-0.1860	-0.3300 to -0.04204	Yes
21588	21:**10 X** vs. 36:**0 X**	-2.550	-2.711 to -2.389	Yes
21589	21:**10 X** vs. 36:**1 X**	-2.246	-2.390 to -2.102	Yes
21590	21:**10 X** vs. 36:**10 X**	-1.603	-1.747 to -1.459	Yes
21591	21:**10 X** vs. 36:**40 X**	-0.2997	-0.4436 to -0.1557	Yes
21592	21:**10 X** vs. 36:**50 X**	-0.1493	-0.2933 to -0.005374	Yes
21593	21:**10 X** vs. 37:**0 X**	-2.934	-3.078 to -2.790	Yes
21594	21:**10 X** vs. 37:**1 X**	-2.316	-2.460 to -2.172	Yes
21595	21:**10 X** vs. 37:**10 X**	-1.605	-1.749 to -1.461	Yes
21596	21:**10 X** vs. 37:**40 X**	-0.3370	-0.4810 to -0.1930	Yes
21597	21:**10 X** vs. 37:**50 X**	-0.1580	-0.3020 to -0.01404	Yes
21598	21:**10 X** vs. 38:**0 X**	-2.879	-3.023 to -2.735	Yes
21599	21:**10 X** vs. 38:**1 X**	-2.435	-2.579 to -2.291	Yes
21600	21:**10 X** vs. 38:**10 X**	-1.880	-2.024 to -1.736	Yes

2way ANOVA Multiple comparisons	A Data Set-A Y	B Data Set-B Y	C Data Set-C Y
21601 21:10 X vs. 38:40 X	-0.3413	-0.4853 to -0.1974	Yes
21602 21:10 X vs. 38:50 X	-0.1620	-0.3060 to -0.01804	Yes
21603 21:10 X vs. 39:0 X	-3.110	-3.254 to -2.966	Yes
21604 21:10 X vs. 39:1 X	-3.001	-3.145 to -2.857	Yes
21605 21:10 X vs. 39:10 X	-2.025	-2.169 to -1.881	Yes
21606 21:10 X vs. 39:40 X	-0.3473	-0.4913 to -0.2034	Yes
21607 21:10 X vs. 39:50 X	-0.1930	-0.3370 to -0.04904	Yes
21608 21:10 X vs. 40:0 X	-3.151	-3.295 to -3.007	Yes
21609 21:10 X vs. 40:1 X	-3.077	-3.221 to -2.933	Yes
21610 21:10 X vs. 40:10 X	-2.281	-2.425 to -2.137	Yes
21611 21:10 X vs. 40:40 X	-0.3520	-0.4960 to -0.2080	Yes
21612 21:10 X vs. 40:50 X	-0.1993	-0.3433 to -0.05537	Yes
21613 21:10 X vs. 41:0 X	-3.192	-3.336 to -3.048	Yes
21614 21:10 X vs. 41:1 X	-3.191	-3.335 to -3.047	Yes
21615 21:10 X vs. 41:10 X	-2.335	-2.479 to -2.191	Yes
21616 21:10 X vs. 41:40 X	-0.2993	-0.4433 to -0.1554	Yes
21617 21:10 X vs. 41:50 X	-0.2120	-0.3560 to -0.06804	Yes
21618 21:10 X vs. 42:0 X	-3.333	-3.477 to -3.189	Yes
21619 21:10 X vs. 42:1 X	-3.353	-3.497 to -3.209	Yes
21620 21:10 X vs. 42:10 X	-2.335	-2.479 to -2.191	Yes
21621 21:10 X vs. 42:40 X	-0.3120	-0.4560 to -0.1680	Yes
21622 21:10 X vs. 42:50 X	-0.2153	-0.3593 to -0.07137	Yes
21623 21:10 X vs. 43:0 X	-3.423	-3.567 to -3.279	Yes
21624 21:10 X vs. 43:1 X	-3.338	-3.482 to -3.194	Yes
21625 21:10 X vs. 43:10 X	-3.053	-3.197 to -2.909	Yes
21626 21:10 X vs. 43:40 X	-0.3103	-0.4543 to -0.1664	Yes
21627 21:10 X vs. 43:50 X	-0.2157	-0.3596 to -0.07171	Yes
21628 21:10 X vs. 44:0 X	-3.447	-3.591 to -3.303	Yes
21629 21:10 X vs. 44:1 X	-3.337	-3.481 to -3.193	Yes
21630 21:10 X vs. 44:10 X	-3.117	-3.261 to -2.973	Yes
21631 21:10 X vs. 44:40 X	-0.3123	-0.4563 to -0.1684	Yes
21632 21:10 X vs. 44:50 X	-0.1480	-0.2920 to -0.004040	Yes
21633 21:10 X vs. 45:0 X	-3.312	-3.456 to -3.168	Yes
21634 21:10 X vs. 45:1 X	-3.114	-3.258 to -2.970	Yes
21635 21:10 X vs. 45:10 X	-2.996	-3.140 to -2.852	Yes
21636 21:10 X vs. 45:40 X	-0.3137	-0.4576 to -0.1697	Yes
21637 21:10 X vs. 45:50 X	-0.1450	-0.2890 to -0.001040	Yes
21638 21:10 X vs. 46:0 X	-3.116	-3.260 to -2.972	Yes
21639 21:10 X vs. 46:1 X	-3.083	-3.227 to -2.939	Yes
21640 21:10 X vs. 46:10 X	-2.991	-3.135 to -2.847	Yes
21641 21:10 X vs. 46:40 X	-0.3273	-0.4713 to -0.1834	Yes
21642 21:10 X vs. 46:50 X	-0.1380	-0.2820 to 0.005960	No
21643 21:10 X vs. 47:0 X	-3.092	-3.236 to -2.948	Yes
21644 21:10 X vs. 47:1 X	-2.996	-3.140 to -2.852	Yes
21645 21:10 X vs. 47:10 X	-2.977	-3.121 to -2.833	Yes

2way ANOVA Multiple comparisons		A Data Set-A Y	B Data Set-B Y	C Data Set-C Y
21646	21:10 X vs. 47:40 X	-0.3373	-0.4813 to -0.1934	Yes
21647	21:10 X vs. 47:50 X	-0.09600	-0.2400 to 0.04796	No
21648	21:10 X vs. 48:0 X	-3.079	-3.240 to -2.918	Yes
21649	21:10 X vs. 48:1 X	-2.983	-3.127 to -2.839	Yes
21650	21:10 X vs. 48:10 X	-2.977	-3.121 to -2.833	Yes
21651	21:10 X vs. 48:40 X	-0.3437	-0.4876 to -0.1997	Yes
21652	21:10 X vs. 48:50 X	-0.1033	-0.2473 to 0.04063	No
21653	21:10 X vs. 49:0 X	-3.079	-3.223 to -2.935	Yes
21654	21:10 X vs. 49:1 X	-2.983	-3.127 to -2.839	Yes
21655	21:10 X vs. 49:10 X	-2.977	-3.121 to -2.833	Yes
21656	21:10 X vs. 49:40 X	-0.3480	-0.4920 to -0.2040	Yes
21657	21:10 X vs. 49:50 X	-0.1027	-0.2466 to 0.04129	No
21658	21:10 X vs. 50:0 X	-3.079	-3.223 to -2.935	Yes
21659	21:10 X vs. 50:1 X	-2.983	-3.127 to -2.839	Yes
21660	21:10 X vs. 50:10 X	-2.977	-3.121 to -2.833	Yes
21661	21:10 X vs. 50:40 X	-0.3557	-0.4996 to -0.2117	Yes
21662	21:10 X vs. 50:50 X	-0.1023	-0.2463 to 0.04163	No
21663	21:40 X vs. 21:50 X	-0.09033	-0.2343 to 0.05363	No
21664	21:40 X vs. 22:0 X	-0.2163	-0.3603 to -0.07237	Yes
21665	21:40 X vs. 22:1 X	-0.2110	-0.3550 to -0.06704	Yes
21666	21:40 X vs. 22:10 X	-0.2063	-0.3503 to -0.06237	Yes
21667	21:40 X vs. 22:40 X	-0.1067	-0.2506 to 0.03729	No
21668	21:40 X vs. 22:50 X	-0.1693	-0.3133 to -0.02537	Yes
21669	21:40 X vs. 23:0 X	-0.2817	-0.4256 to -0.1377	Yes
21670	21:40 X vs. 23:1 X	-0.4713	-0.6323 to -0.3104	Yes
21671	21:40 X vs. 23:10 X	-0.2210	-0.3650 to -0.07704	Yes
21672	21:40 X vs. 23:40 X	-0.1013	-0.2623 to 0.05962	No
21673	21:40 X vs. 23:50 X	-0.1353	-0.2793 to 0.008626	No
21674	21:40 X vs. 24:0 X	-0.3990	-0.5430 to -0.2550	Yes
21675	21:40 X vs. 24:1 X	-0.5553	-0.7163 to -0.3944	Yes
21676	21:40 X vs. 24:10 X	-0.3828	-0.5438 to -0.2219	Yes
21677	21:40 X vs. 24:40 X	-0.1177	-0.2616 to 0.02629	No
21678	21:40 X vs. 24:50 X	-0.03933	-0.1833 to 0.1046	No
21679	21:40 X vs. 25:0 X	-0.7363	-0.8973 to -0.5754	Yes
21680	21:40 X vs. 25:1 X	-0.9258	-1.087 to -0.7649	Yes
21681	21:40 X vs. 25:10 X	-0.2873	-0.4909 to -0.08374	Yes
21682	21:40 X vs. 25:40 X	-0.1197	-0.2636 to 0.02429	No
21683	21:40 X vs. 25:50 X	-0.0940	-0.2380 to 0.04996	No
21684	21:40 X vs. 26:0 X	-0.9488	-1.110 to -0.7879	Yes
21685	21:40 X vs. 26:1 X	-1.062	-1.223 to -0.9009	Yes
21686	21:40 X vs. 26:10 X	-0.7483	-0.9093 to -0.5874	Yes
21687	21:40 X vs. 26:40 X	-0.1940	-0.3380 to -0.05004	Yes
21688	21:40 X vs. 26:50 X	-0.05433	-0.1983 to 0.08963	No
21689	21:40 X vs. 27:0 X	-1.137	-1.281 to -0.9927	Yes
21690	21:40 X vs. 27:1 X	-1.227	-1.388 to -1.066	Yes

2way ANOVA<br>Multiple comparisons		A<br>Data Set-A<br>Y	B<br>Data Set-B<br>Y	C<br>Data Set-C<br>Y
21691	21:40 X vs. 27:10 X	-1.039	-1.200 to -0.8784	Yes
21692	21:40 X vs. 27:40 X	-0.1940	-0.3380 to -0.05004	Yes
21693	21:40 X vs. 27:50 X	-0.05367	-0.1976 to 0.09029	No
21694	21:40 X vs. 28:0 X	-1.086	-1.247 to -0.9249	Yes
21695	21:40 X vs. 28:1 X	-1.127	-1.271 to -0.9827	Yes
21696	21:40 X vs. 28:10 X	-0.9283	-1.089 to -0.7674	Yes
21697	21:40 X vs. 28:40 X	-0.2063	-0.3503 to -0.06237	Yes
21698	21:40 X vs. 28:50 X	-0.1017	-0.2456 to 0.04229	No
21699	21:40 X vs. 29:0 X	-1.567	-1.728 to -1.406	Yes
21700	21:40 X vs. 29:1 X	-1.652	-1.813 to -1.491	Yes
21701	21:40 X vs. 29:10 X	-0.8668	-1.028 to -0.7059	Yes
21702	21:40 X vs. 29:40 X	-0.3143	-0.4583 to -0.1704	Yes
21703	21:40 X vs. 29:50 X	-0.1030	-0.2470 to 0.04096	No
21704	21:40 X vs. 30:0 X	-1.282	-1.443 to -1.121	Yes
21705	21:40 X vs. 30:1 X	-1.603	-1.764 to -1.442	Yes
21706	21:40 X vs. 30:10 X	-0.9413	-1.102 to -0.7804	Yes
21707	21:40 X vs. 30:40 X	-0.2413	-0.3853 to -0.09737	Yes
21708	21:40 X vs. 30:50 X	-0.1553	-0.2993 to -0.01137	Yes
21709	21:40 X vs. 31:0 X	-1.600	-1.761 to -1.439	Yes
21710	21:40 X vs. 31:1 X	-1.590	-1.751 to -1.429	Yes
21711	21:40 X vs. 31:10 X	-1.073	-1.234 to -0.9119	Yes
21712	21:40 X vs. 31:40 X	-0.3330	-0.4770 to -0.1890	Yes
21713	21:40 X vs. 31:50 X	-0.1343	-0.2783 to 0.009626	No
21714	21:40 X vs. 32:0 X	-2.191	-2.335 to -2.047	Yes
21715	21:40 X vs. 32:1 X	-1.647	-1.808 to -1.486	Yes
21716	21:40 X vs. 32:10 X	-1.503	-1.647 to -1.359	Yes
21717	21:40 X vs. 32:40 X	-0.3293	-0.4733 to -0.1854	Yes
21718	21:40 X vs. 32:50 X	-0.1340	-0.2780 to 0.009960	No
21719	21:40 X vs. 33:0 X	-2.195	-2.339 to -2.051	Yes
21720	21:40 X vs. 33:1 X	-2.010	-2.154 to -1.866	Yes
21721	21:40 X vs. 33:10 X	-1.670	-1.814 to -1.526	Yes
21722	21:40 X vs. 33:40 X	-0.3480	-0.4920 to -0.2040	Yes
21723	21:40 X vs. 33:50 X	-0.1490	-0.2930 to -0.005040	Yes
21724	21:40 X vs. 34:0 X	-2.120	-2.264 to -1.976	Yes
21725	21:40 X vs. 34:1 X	-2.127	-2.271 to -1.983	Yes
21726	21:40 X vs. 34:10 X	-1.760	-1.904 to -1.616	Yes
21727	21:40 X vs. 34:40 X	-0.3600	-0.5040 to -0.2160	Yes
21728	21:40 X vs. 34:50 X	-0.2980	-0.4420 to -0.1540	Yes
21729	21:40 X vs. 35:0 X	-2.468	-2.612 to -2.324	Yes
21730	21:40 X vs. 35:1 X	-2.234	-2.378 to -2.090	Yes
21731	21:40 X vs. 35:10 X	-1.722	-1.866 to -1.578	Yes
21732	21:40 X vs. 35:40 X	-0.3690	-0.5130 to -0.2250	Yes
21733	21:40 X vs. 35:50 X	-0.3107	-0.4546 to -0.1667	Yes
21734	21:40 X vs. 36:0 X	-2.675	-2.836 to -2.514	Yes
21735	21:40 X vs. 36:1 X	-2.371	-2.515 to -2.227	Yes

2way ANOVA<br>Multiple comparisons	A<br>Data Set-A<br>Y	B<br>Data Set-B<br>Y	C<br>Data Set-C<br>Y
21736 21:40 X vs. 36:10 X	-1.728	-1.872 to -1.584	Yes
21737 21:40 X vs. 36:40 X	-0.4243	-0.5683 to -0.2804	Yes
21738 21:40 X vs. 36:50 X	-0.2740	-0.4180 to -0.1300	Yes
21739 21:40 X vs. 37:0 X	-3.059	-3.203 to -2.915	Yes
21740 21:40 X vs. 37:1 X	-2.441	-2.585 to -2.297	Yes
21741 21:40 X vs. 37:10 X	-1.729	-1.873 to -1.585	Yes
21742 21:40 X vs. 37:40 X	-0.4617	-0.6056 to -0.3177	Yes
21743 21:40 X vs. 37:50 X	-0.2827	-0.4266 to -0.1387	Yes
21744 21:40 X vs. 38:0 X	-3.004	-3.148 to -2.860	Yes
21745 21:40 X vs. 38:1 X	-2.559	-2.703 to -2.415	Yes
21746 21:40 X vs. 38:10 X	-2.005	-2.149 to -1.861	Yes
21747 21:40 X vs. 38:40 X	-0.4660	-0.6100 to -0.3220	Yes
21748 21:40 X vs. 38:50 X	-0.2867	-0.4306 to -0.1427	Yes
21749 21:40 X vs. 39:0 X	-3.234	-3.378 to -3.090	Yes
21750 21:40 X vs. 39:1 X	-3.126	-3.270 to -2.982	Yes
21751 21:40 X vs. 39:10 X	-2.150	-2.294 to -2.006	Yes
21752 21:40 X vs. 39:40 X	-0.4720	-0.6160 to -0.3280	Yes
21753 21:40 X vs. 39:50 X	-0.3177	-0.4616 to -0.1737	Yes
21754 21:40 X vs. 40:0 X	-3.275	-3.419 to -3.131	Yes
21755 21:40 X vs. 40:1 X	-3.201	-3.345 to -3.057	Yes
21756 21:40 X vs. 40:10 X	-2.406	-2.550 to -2.262	Yes
21757 21:40 X vs. 40:40 X	-0.4767	-0.6206 to -0.3327	Yes
21758 21:40 X vs. 40:50 X	-0.3240	-0.4680 to -0.1800	Yes
21759 21:40 X vs. 41:0 X	-3.316	-3.460 to -3.172	Yes
21760 21:40 X vs. 41:1 X	-3.315	-3.459 to -3.171	Yes
21761 21:40 X vs. 41:10 X	-2.460	-2.604 to -2.316	Yes
21762 21:40 X vs. 41:40 X	-0.4240	-0.5680 to -0.2800	Yes
21763 21:40 X vs. 41:50 X	-0.3367	-0.4806 to -0.1927	Yes
21764 21:40 X vs. 42:0 X	-3.458	-3.602 to -3.314	Yes
21765 21:40 X vs. 42:1 X	-3.478	-3.622 to -3.334	Yes
21766 21:40 X vs. 42:10 X	-2.460	-2.604 to -2.316	Yes
21767 21:40 X vs. 42:40 X	-0.4367	-0.5806 to -0.2927	Yes
21768 21:40 X vs. 42:50 X	-0.3400	-0.4840 to -0.1960	Yes
21769 21:40 X vs. 43:0 X	-3.548	-3.692 to -3.404	Yes
21770 21:40 X vs. 43:1 X	-3.462	-3.606 to -3.318	Yes
21771 21:40 X vs. 43:10 X	-3.178	-3.322 to -3.034	Yes
21772 21:40 X vs. 43:40 X	-0.4350	-0.5790 to -0.2910	Yes
21773 21:40 X vs. 43:50 X	-0.3403	-0.4843 to -0.1964	Yes
21774 21:40 X vs. 44:0 X	-3.572	-3.716 to -3.428	Yes
21775 21:40 X vs. 44:1 X	-3.462	-3.606 to -3.318	Yes
21776 21:40 X vs. 44:10 X	-3.241	-3.385 to -3.097	Yes
21777 21:40 X vs. 44:40 X	-0.4370	-0.5810 to -0.2930	Yes
21778 21:40 X vs. 44:50 X	-0.2727	-0.4166 to -0.1287	Yes
21779 21:40 X vs. 45:0 X	-3.437	-3.581 to -3.293	Yes
21780 21:40 X vs. 45:1 X	-3.239	-3.383 to -3.095	Yes

	2way ANOVA<br>Multiple comparisons	A<br>Data Set-A<br>Y	B<br>Data Set-B<br>Y	C<br>Data Set-C<br>Y
21781	21:40 X vs. 45:10 X	-3.121	-3.265 to -2.977	Yes
21782	21:40 X vs. 45:40 X	-0.4383	-0.5823 to -0.2944	Yes
21783	21:40 X vs. 45:50 X	-0.2697	-0.4136 to -0.1257	Yes
21784	21:40 X vs. 46:0 X	-3.241	-3.385 to -3.097	Yes
21785	21:40 X vs. 46:1 X	-3.208	-3.352 to -3.064	Yes
21786	21:40 X vs. 46:10 X	-3.116	-3.260 to -2.972	Yes
21787	21:40 X vs. 46:40 X	-0.4520	-0.5960 to -0.3080	Yes
21788	21:40 X vs. 46:50 X	-0.2627	-0.4066 to -0.1187	Yes
21789	21:40 X vs. 47:0 X	-3.217	-3.361 to -3.073	Yes
21790	21:40 X vs. 47:1 X	-3.121	-3.265 to -2.977	Yes
21791	21:40 X vs. 47:10 X	-3.102	-3.246 to -2.958	Yes
21792	21:40 X vs. 47:40 X	-0.4620	-0.6060 to -0.3180	Yes
21793	21:40 X vs. 47:50 X	-0.2207	-0.3646 to -0.07671	Yes
21794	21:40 X vs. 48:0 X	-3.204	-3.365 to -3.043	Yes
21795	21:40 X vs. 48:1 X	-3.108	-3.252 to -2.964	Yes
21796	21:40 X vs. 48:10 X	-3.102	-3.246 to -2.958	Yes
21797	21:40 X vs. 48:40 X	-0.4683	-0.6123 to -0.3244	Yes
21798	21:40 X vs. 48:50 X	-0.2280	-0.3720 to -0.08404	Yes
21799	21:40 X vs. 49:0 X	-3.204	-3.348 to -3.060	Yes
21800	21:40 X vs. 49:1 X	-3.108	-3.252 to -2.964	Yes
21801	21:40 X vs. 49:10 X	-3.102	-3.246 to -2.958	Yes
21802	21:40 X vs. 49:40 X	-0.4727	-0.6166 to -0.3287	Yes
21803	21:40 X vs. 49:50 X	-0.2273	-0.3713 to -0.08337	Yes
21804	21:40 X vs. 50:0 X	-3.204	-3.348 to -3.060	Yes
21805	21:40 X vs. 50:1 X	-3.108	-3.252 to -2.964	Yes
21806	21:40 X vs. 50:10 X	-3.102	-3.246 to -2.958	Yes
21807	21:40 X vs. 50:40 X	-0.4803	-0.6243 to -0.3364	Yes
21808	21:40 X vs. 50:50 X	-0.2270	-0.3710 to -0.08304	Yes
21809	21:50 X vs. 22:0 X	-0.1260	-0.2700 to 0.01796	No
21810	21:50 X vs. 22:1 X	-0.1207	-0.2646 to 0.02329	No
21811	21:50 X vs. 22:10 X	-0.1160	-0.2600 to 0.02796	No
21812	21:50 X vs. 22:40 X	-0.01633	-0.1603 to 0.1276	No
21813	21:50 X vs. 22:50 X	-0.0790	-0.2230 to 0.06496	No
21814	21:50 X vs. 23:0 X	-0.1913	-0.3353 to -0.04737	Yes
21815	21:50 X vs. 23:1 X	-0.3810	-0.5420 to -0.2200	Yes
21816	21:50 X vs. 23:10 X	-0.1307	-0.2746 to 0.01329	No
21817	21:50 X vs. 23:40 X	-0.01100	-0.1720 to 0.1500	No
21818	21:50 X vs. 23:50 X	-0.0450	-0.1890 to 0.09896	No
21819	21:50 X vs. 24:0 X	-0.3087	-0.4526 to -0.1647	Yes
21820	21:50 X vs. 24:1 X	-0.4650	-0.6260 to -0.3040	Yes
21821	21:50 X vs. 24:10 X	-0.2925	-0.4535 to -0.1315	Yes
21822	21:50 X vs. 24:40 X	-0.02733	-0.1713 to 0.1166	No
21823	21:50 X vs. 24:50 X	0.0510	-0.09296 to 0.1950	No
21824	21:50 X vs. 25:0 X	-0.6460	-0.8070 to -0.4850	Yes
21825	21:50 X vs. 25:1 X	-0.8355	-0.9965 to -0.6745	Yes

2way ANOVA<br>Multiple comparisons		A<br>Data Set-A<br>Y	B<br>Data Set-B<br>Y	C<br>Data Set-C<br>Y
21826	21:50 X vs. 25:10 X	-0.1970	-0.4006 to 0.006590	No
21827	21:50 X vs. 25:40 X	-0.02933	-0.1733 to 0.1146	No
21828	21:50 X vs. 25:50 X	-0.003667	-0.1476 to 0.1403	No
21829	21:50 X vs. 26:0 X	-0.8585	-1.019 to -0.6975	Yes
21830	21:50 X vs. 26:1 X	-0.9715	-1.132 to -0.8105	Yes
21831	21:50 X vs. 26:10 X	-0.6580	-0.8190 to -0.4970	Yes
21832	21:50 X vs. 26:40 X	-0.1037	-0.2476 to 0.04029	No
21833	21:50 X vs. 26:50 X	0.03600	-0.1080 to 0.1800	No
21834	21:50 X vs. 27:0 X	-1.046	-1.190 to -0.9024	Yes
21835	21:50 X vs. 27:1 X	-1.137	-1.298 to -0.9760	Yes
21836	21:50 X vs. 27:10 X	-0.9490	-1.110 to -0.7880	Yes
21837	21:50 X vs. 27:40 X	-0.1037	-0.2476 to 0.04029	No
21838	21:50 X vs. 27:50 X	0.03667	-0.1073 to 0.1806	No
21839	21:50 X vs. 28:0 X	-0.9955	-1.156 to -0.8345	Yes
21840	21:50 X vs. 28:1 X	-1.036	-1.180 to -0.8924	Yes
21841	21:50 X vs. 28:10 X	-0.8380	-0.9990 to -0.6770	Yes
21842	21:50 X vs. 28:40 X	-0.1160	-0.2600 to 0.02796	No
21843	21:50 X vs. 28:50 X	-0.01133	-0.1553 to 0.1326	No
21844	21:50 X vs. 29:0 X	-1.477	-1.638 to -1.316	Yes
21845	21:50 X vs. 29:1 X	-1.562	-1.723 to -1.401	Yes
21846	21:50 X vs. 29:10 X	-0.7765	-0.9375 to -0.6155	Yes
21847	21:50 X vs. 29:40 X	-0.2240	-0.3680 to -0.08004	Yes
21848	21:50 X vs. 29:50 X	-0.01267	-0.1566 to 0.1313	No
21849	21:50 X vs. 30:0 X	-1.192	-1.352 to -1.031	Yes
21850	21:50 X vs. 30:1 X	-1.513	-1.674 to -1.352	Yes
21851	21:50 X vs. 30:10 X	-0.8510	-1.012 to -0.6900	Yes
21852	21:50 X vs. 30:40 X	-0.1510	-0.2950 to -0.007040	Yes
21853	21:50 X vs. 30:50 X	-0.06500	-0.2090 to 0.07896	No
21854	21:50 X vs. 31:0 X	-1.510	-1.670 to -1.349	Yes
21855	21:50 X vs. 31:1 X	-1.500	-1.661 to -1.339	Yes
21856	21:50 X vs. 31:10 X	-0.9825	-1.143 to -0.8215	Yes
21857	21:50 X vs. 31:40 X	-0.2427	-0.3866 to -0.09871	Yes
21858	21:50 X vs. 31:50 X	-0.0440	-0.1880 to 0.09996	No
21859	21:50 X vs. 32:0 X	-2.101	-2.245 to -1.957	Yes
21860	21:50 X vs. 32:1 X	-1.557	-1.718 to -1.396	Yes
21861	21:50 X vs. 32:10 X	-1.413	-1.557 to -1.269	Yes
21862	21:50 X vs. 32:40 X	-0.2390	-0.3830 to -0.09504	Yes
21863	21:50 X vs. 32:50 X	-0.04367	-0.1876 to 0.1003	No
21864	21:50 X vs. 33:0 X	-2.105	-2.249 to -1.961	Yes
21865	21:50 X vs. 33:1 X	-1.919	-2.063 to -1.775	Yes
21866	21:50 X vs. 33:10 X	-1.580	-1.724 to -1.436	Yes
21867	21:50 X vs. 33:40 X	-0.2577	-0.4016 to -0.1137	Yes
21868	21:50 X vs. 33:50 X	-0.05867	-0.2026 to 0.08529	No
21869	21:50 X vs. 34:0 X	-2.029	-2.173 to -1.885	Yes
21870	21:50 X vs. 34:1 X	-2.037	-2.181 to -1.893	Yes

	2way ANOVA<br>Multiple comparisons	A<br>Data Set-A<br>Y	B<br>Data Set-B<br>Y	C<br>Data Set-C<br>Y
21871	21:50 X vs. 34:10 X	-1.670	-1.814 to -1.526	Yes
21872	21:50 X vs. 34:40 X	-0.2697	-0.4136 to -0.1257	Yes
21873	21:50 X vs. 34:50 X	-0.2077	-0.3516 to -0.06371	Yes
21874	21:50 X vs. 35:0 X	-2.378	-2.522 to -2.234	Yes
21875	21:50 X vs. 35:1 X	-2.144	-2.288 to -2.000	Yes
21876	21:50 X vs. 35:10 X	-1.632	-1.776 to -1.488	Yes
21877	21:50 X vs. 35:40 X	-0.2787	-0.4226 to -0.1347	Yes
21878	21:50 X vs. 35:50 X	-0.2203	-0.3643 to -0.07637	Yes
21879	21:50 X vs. 36:0 X	-2.585	-2.745 to -2.424	Yes
21880	21:50 X vs. 36:1 X	-2.280	-2.424 to -2.136	Yes
21881	21:50 X vs. 36:10 X	-1.637	-1.781 to -1.493	Yes
21882	21:50 X vs. 36:40 X	-0.3340	-0.4780 to -0.1900	Yes
21883	21:50 X vs. 36:50 X	-0.1837	-0.3276 to -0.03971	Yes
21884	21:50 X vs. 37:0 X	-2.968	-3.112 to -2.824	Yes
21885	21:50 X vs. 37:1 X	-2.350	-2.494 to -2.206	Yes
21886	21:50 X vs. 37:10 X	-1.639	-1.783 to -1.495	Yes
21887	21:50 X vs. 37:40 X	-0.3713	-0.5153 to -0.2274	Yes
21888	21:50 X vs. 37:50 X	-0.1923	-0.3363 to -0.04837	Yes
21889	21:50 X vs. 38:0 X	-2.913	-3.057 to -2.769	Yes
21890	21:50 X vs. 38:1 X	-2.469	-2.613 to -2.325	Yes
21891	21:50 X vs. 38:10 X	-1.914	-2.058 to -1.770	Yes
21892	21:50 X vs. 38:40 X	-0.3757	-0.5196 to -0.2317	Yes
21893	21:50 X vs. 38:50 X	-0.1963	-0.3403 to -0.05237	Yes
21894	21:50 X vs. 39:0 X	-3.144	-3.288 to -3.000	Yes
21895	21:50 X vs. 39:1 X	-3.036	-3.180 to -2.892	Yes
21896	21:50 X vs. 39:10 X	-2.059	-2.203 to -1.915	Yes
21897	21:50 X vs. 39:40 X	-0.3817	-0.5256 to -0.2377	Yes
21898	21:50 X vs. 39:50 X	-0.2273	-0.3713 to -0.08337	Yes
21899	21:50 X vs. 40:0 X	-3.185	-3.329 to -3.041	Yes
21900	21:50 X vs. 40:1 X	-3.111	-3.255 to -2.967	Yes
21901	21:50 X vs. 40:10 X	-2.316	-2.460 to -2.172	Yes
21902	21:50 X vs. 40:40 X	-0.3863	-0.5303 to -0.2424	Yes
21903	21:50 X vs. 40:50 X	-0.2337	-0.3776 to -0.08971	Yes
21904	21:50 X vs. 41:0 X	-3.226	-3.370 to -3.082	Yes
21905	21:50 X vs. 41:1 X	-3.225	-3.369 to -3.081	Yes
21906	21:50 X vs. 41:10 X	-2.369	-2.513 to -2.225	Yes
21907	21:50 X vs. 41:40 X	-0.3337	-0.4776 to -0.1897	Yes
21908	21:50 X vs. 41:50 X	-0.2463	-0.3903 to -0.1024	Yes
21909	21:50 X vs. 42:0 X	-3.368	-3.512 to -3.224	Yes
21910	21:50 X vs. 42:1 X	-3.387	-3.531 to -3.243	Yes
21911	21:50 X vs. 42:10 X	-2.370	-2.514 to -2.226	Yes
21912	21:50 X vs. 42:40 X	-0.3463	-0.4903 to -0.2024	Yes
21913	21:50 X vs. 42:50 X	-0.2497	-0.3936 to -0.1057	Yes
21914	21:50 X vs. 43:0 X	-3.458	-3.602 to -3.314	Yes
21915	21:50 X vs. 43:1 X	-3.372	-3.516 to -3.228	Yes

	2way ANOVA<br>Multiple comparisons	A<br>Data Set-A<br>Y	B<br>Data Set-B<br>Y	C<br>Data Set-C<br>Y
21916	21:50 X vs. 43:10 X	-3.088	-3.232 to -2.944	Yes
21917	21:50 X vs. 43:40 X	-0.3447	-0.4886 to -0.2007	Yes
21918	21:50 X vs. 43:50 X	-0.2500	-0.3940 to -0.1060	Yes
21919	21:50 X vs. 44:0 X	-3.482	-3.626 to -3.338	Yes
21920	21:50 X vs. 44:1 X	-3.371	-3.515 to -3.227	Yes
21921	21:50 X vs. 44:10 X	-3.151	-3.295 to -3.007	Yes
21922	21:50 X vs. 44:40 X	-0.3467	-0.4906 to -0.2027	Yes
21923	21:50 X vs. 44:50 X	-0.1823	-0.3263 to -0.03837	Yes
21924	21:50 X vs. 45:0 X	-3.346	-3.490 to -3.202	Yes
21925	21:50 X vs. 45:1 X	-3.148	-3.292 to -3.004	Yes
21926	21:50 X vs. 45:10 X	-3.031	-3.175 to -2.887	Yes
21927	21:50 X vs. 45:40 X	-0.3480	-0.4920 to -0.2040	Yes
21928	21:50 X vs. 45:50 X	-0.1793	-0.3233 to -0.03537	Yes
21929	21:50 X vs. 46:0 X	-3.151	-3.295 to -3.007	Yes
21930	21:50 X vs. 46:1 X	-3.118	-3.262 to -2.974	Yes
21931	21:50 X vs. 46:10 X	-3.026	-3.170 to -2.882	Yes
21932	21:50 X vs. 46:40 X	-0.3617	-0.5056 to -0.2177	Yes
21933	21:50 X vs. 46:50 X	-0.1723	-0.3163 to -0.02837	Yes
21934	21:50 X vs. 47:0 X	-3.126	-3.270 to -2.982	Yes
21935	21:50 X vs. 47:1 X	-3.030	-3.174 to -2.886	Yes
21936	21:50 X vs. 47:10 X	-3.012	-3.156 to -2.868	Yes
21937	21:50 X vs. 47:40 X	-0.3717	-0.5156 to -0.2277	Yes
21938	21:50 X vs. 47:50 X	-0.1303	-0.2743 to 0.01363	No
21939	21:50 X vs. 48:0 X	-3.114	-3.274 to -2.953	Yes
21940	21:50 X vs. 48:1 X	-3.017	-3.161 to -2.873	Yes
21941	21:50 X vs. 48:10 X	-3.012	-3.156 to -2.868	Yes
21942	21:50 X vs. 48:40 X	-0.3780	-0.5220 to -0.2340	Yes
21943	21:50 X vs. 48:50 X	-0.1377	-0.2816 to 0.006293	No
21944	21:50 X vs. 49:0 X	-3.114	-3.258 to -2.970	Yes
21945	21:50 X vs. 49:1 X	-3.017	-3.161 to -2.873	Yes
21946	21:50 X vs. 49:10 X	-3.012	-3.156 to -2.868	Yes
21947	21:50 X vs. 49:40 X	-0.3823	-0.5263 to -0.2384	Yes
21948	21:50 X vs. 49:50 X	-0.1370	-0.2810 to 0.006960	No
21949	21:50 X vs. 50:0 X	-3.114	-3.258 to -2.970	Yes
21950	21:50 X vs. 50:1 X	-3.017	-3.161 to -2.873	Yes
21951	21:50 X vs. 50:10 X	-3.012	-3.156 to -2.868	Yes
21952	21:50 X vs. 50:40 X	-0.3900	-0.5340 to -0.2460	Yes
21953	21:50 X vs. 50:50 X	-0.1367	-0.2806 to 0.007293	No
21954	22:0 X vs. 22:1 X	0.005333	-0.1386 to 0.1493	No
21955	22:0 X vs. 22:10 X	0.01000	-0.1340 to 0.1540	No
21956	22:0 X vs. 22:40 X	0.1097	-0.03429 to 0.2536	No
21957	22:0 X vs. 22:50 X	0.04700	-0.09696 to 0.1910	No
21958	22:0 X vs. 23:0 X	-0.06533	-0.2093 to 0.07863	No
21959	22:0 X vs. 23:1 X	-0.2550	-0.4160 to -0.09405	Yes
21960	22:0 X vs. 23:10 X	-0.004667	-0.1486 to 0.1393	No

2way ANOVA<br>Multiple comparisons	A<br>Data Set-A<br>Y	B<br>Data Set-B<br>Y	C<br>Data Set-C<br>Y
21961 22:0 X vs. 23:40 X	0.1150	-0.04595 to 0.2760	No
21962 22:0 X vs. 23:50 X	0.08100	-0.06296 to 0.2250	No
21963 22:0 X vs. 24:0 X	-0.1827	-0.3266 to -0.03871	Yes
21964 22:0 X vs. 24:1 X	-0.3390	-0.5000 to -0.1780	Yes
21965 22:0 X vs. 24:10 X	-0.1665	-0.3275 to -0.005548	Yes
21966 22:0 X vs. 24:40 X	0.09867	-0.04529 to 0.2426	No
21967 22:0 X vs. 24:50 X	0.1770	0.03304 to 0.3210	Yes
21968 22:0 X vs. 25:0 X	-0.5200	-0.6810 to -0.3590	Yes
21969 22:0 X vs. 25:1 X	-0.7095	-0.8705 to -0.5485	Yes
21970 22:0 X vs. 25:10 X	-0.0710	-0.2746 to 0.1326	No
21971 22:0 X vs. 25:40 X	0.09667	-0.04729 to 0.2406	No
21972 22:0 X vs. 25:50 X	0.1223	-0.02163 to 0.2663	No
21973 22:0 X vs. 26:0 X	-0.7325	-0.8935 to -0.5715	Yes
21974 22:0 X vs. 26:1 X	-0.8455	-1.006 to -0.6845	Yes
21975 22:0 X vs. 26:10 X	-0.5320	-0.6930 to -0.3710	Yes
21976 22:0 X vs. 26:40 X	0.02233	-0.1216 to 0.1663	No
21977 22:0 X vs. 26:50 X	0.1620	0.01804 to 0.3060	Yes
21978 22:0 X vs. 27:0 X	-0.9203	-1.064 to -0.7764	Yes
21979 22:0 X vs. 27:1 X	-1.011	-1.172 to -0.8500	Yes
21980 22:0 X vs. 27:10 X	-0.8230	-0.9840 to -0.6620	Yes
21981 22:0 X vs. 27:40 X	0.02233	-0.1216 to 0.1663	No
21982 22:0 X vs. 27:50 X	0.1627	0.01871 to 0.3066	Yes
21983 22:0 X vs. 28:0 X	-0.8695	-1.030 to -0.7085	Yes
21984 22:0 X vs. 28:1 X	-0.9103	-1.054 to -0.7664	Yes
21985 22:0 X vs. 28:10 X	-0.7120	-0.8730 to -0.5510	Yes
21986 22:0 X vs. 28:40 X	0.01000	-0.1340 to 0.1540	No
21987 22:0 X vs. 28:50 X	0.1147	-0.02929 to 0.2586	No
21988 22:0 X vs. 29:0 X	-1.351	-1.512 to -1.190	Yes
21989 22:0 X vs. 29:1 X	-1.436	-1.597 to -1.275	Yes
21990 22:0 X vs. 29:10 X	-0.6505	-0.8115 to -0.4895	Yes
21991 22:0 X vs. 29:40 X	-0.09800	-0.2420 to 0.04596	No
21992 22:0 X vs. 29:50 X	0.1133	-0.03063 to 0.2573	No
21993 22:0 X vs. 30:0 X	-1.066	-1.226 to -0.9045	Yes
21994 22:0 X vs. 30:1 X	-1.387	-1.548 to -1.226	Yes
21995 22:0 X vs. 30:10 X	-0.7250	-0.8860 to -0.5640	Yes
21996 22:0 X vs. 30:40 X	-0.0250	-0.1690 to 0.1190	No
21997 22:0 X vs. 30:50 X	0.0610	-0.08296 to 0.2050	No
21998 22:0 X vs. 31:0 X	-1.384	-1.544 to -1.223	Yes
21999 22:0 X vs. 31:1 X	-1.374	-1.535 to -1.213	Yes
22000 22:0 X vs. 31:10 X	-0.8565	-1.017 to -0.6955	Yes
22001 22:0 X vs. 31:40 X	-0.1167	-0.2606 to 0.02729	No
22002 22:0 X vs. 31:50 X	0.08200	-0.06196 to 0.2260	No
22003 22:0 X vs. 32:0 X	-1.975	-2.119 to -1.831	Yes
22004 22:0 X vs. 32:1 X	-1.431	-1.592 to -1.270	Yes
22005 22:0 X vs. 32:10 X	-1.287	-1.431 to -1.143	Yes

2way ANOVA<br>Multiple comparisons	A<br>Data Set-A<br>Y	B<br>Data Set-B<br>Y	C<br>Data Set-C<br>Y
22006 22:0 X vs. 32:40 X	-0.1130	-0.2570 to 0.03096	No
22007 22:0 X vs. 32:50 X	0.08233	-0.06163 to 0.2263	No
22008 22:0 X vs. 33:0 X	-1.979	-2.123 to -1.835	Yes
22009 22:0 X vs. 33:1 X	-1.793	-1.937 to -1.649	Yes
22010 22:0 X vs. 33:10 X	-1.454	-1.598 to -1.310	Yes
22011 22:0 X vs. 33:40 X	-0.1317	-0.2756 to 0.01229	No
22012 22:0 X vs. 33:50 X	0.06733	-0.07663 to 0.2113	No
22013 22:0 X vs. 34:0 X	-1.903	-2.047 to -1.759	Yes
22014 22:0 X vs. 34:1 X	-1.911	-2.055 to -1.767	Yes
22015 22:0 X vs. 34:10 X	-1.544	-1.688 to -1.400	Yes
22016 22:0 X vs. 34:40 X	-0.1437	-0.2876 to 0.0002932	No
22017 22:0 X vs. 34:50 X	-0.08167	-0.2256 to 0.06229	No
22018 22:0 X vs. 35:0 X	-2.252	-2.396 to -2.108	Yes
22019 22:0 X vs. 35:1 X	-2.018	-2.162 to -1.874	Yes
22020 22:0 X vs. 35:10 X	-1.506	-1.650 to -1.362	Yes
22021 22:0 X vs. 35:40 X	-0.1527	-0.2966 to -0.008707	Yes
22022 22:0 X vs. 35:50 X	-0.09433	-0.2383 to 0.04963	No
22023 22:0 X vs. 36:0 X	-2.459	-2.619 to -2.298	Yes
22024 22:0 X vs. 36:1 X	-2.154	-2.298 to -2.010	Yes
22025 22:0 X vs. 36:10 X	-1.511	-1.655 to -1.367	Yes
22026 22:0 X vs. 36:40 X	-0.2080	-0.3520 to -0.06404	Yes
22027 22:0 X vs. 36:50 X	-0.05767	-0.2016 to 0.08629	No
22028 22:0 X vs. 37:0 X	-2.842	-2.986 to -2.698	Yes
22029 22:0 X vs. 37:1 X	-2.224	-2.368 to -2.080	Yes
22030 22:0 X vs. 37:10 X	-1.513	-1.657 to -1.369	Yes
22031 22:0 X vs. 37:40 X	-0.2453	-0.3893 to -0.1014	Yes
22032 22:0 X vs. 37:50 X	-0.06633	-0.2103 to 0.07763	No
22033 22:0 X vs. 38:0 X	-2.787	-2.931 to -2.643	Yes
22034 22:0 X vs. 38:1 X	-2.343	-2.487 to -2.199	Yes
22035 22:0 X vs. 38:10 X	-1.788	-1.932 to -1.644	Yes
22036 22:0 X vs. 38:40 X	-0.2497	-0.3936 to -0.1057	Yes
22037 22:0 X vs. 38:50 X	-0.07033	-0.2143 to 0.07363	No
22038 22:0 X vs. 39:0 X	-3.018	-3.162 to -2.874	Yes
22039 22:0 X vs. 39:1 X	-2.910	-3.054 to -2.766	Yes
22040 22:0 X vs. 39:10 X	-1.933	-2.077 to -1.789	Yes
22041 22:0 X vs. 39:40 X	-0.2557	-0.3996 to -0.1117	Yes
22042 22:0 X vs. 39:50 X	-0.1013	-0.2453 to 0.04263	No
22043 22:0 X vs. 40:0 X	-3.059	-3.203 to -2.915	Yes
22044 22:0 X vs. 40:1 X	-2.985	-3.129 to -2.841	Yes
22045 22:0 X vs. 40:10 X	-2.190	-2.334 to -2.046	Yes
22046 22:0 X vs. 40:40 X	-0.2603	-0.4043 to -0.1164	Yes
22047 22:0 X vs. 40:50 X	-0.1077	-0.2516 to 0.03629	No
22048 22:0 X vs. 41:0 X	-3.100	-3.244 to -2.956	Yes
22049 22:0 X vs. 41:1 X	-3.099	-3.243 to -2.955	Yes
22050 22:0 X vs. 41:10 X	-2.243	-2.387 to -2.099	Yes

	2way ANOVA<br>Multiple comparisons	A<br>Data Set-A<br>Y	B<br>Data Set-B<br>Y	C<br>Data Set-C<br>Y
22051	22:0 X vs. 41:40 X	-0.2077	-0.3516 to -0.06371	Yes
22052	22:0 X vs. 41:50 X	-0.1203	-0.2643 to 0.02363	No
22053	22:0 X vs. 42:0 X	-3.242	-3.386 to -3.098	Yes
22054	22:0 X vs. 42:1 X	-3.261	-3.405 to -3.117	Yes
22055	22:0 X vs. 42:10 X	-2.244	-2.388 to -2.100	Yes
22056	22:0 X vs. 42:40 X	-0.2203	-0.3643 to -0.07637	Yes
22057	22:0 X vs. 42:50 X	-0.1237	-0.2676 to 0.02029	No
22058	22:0 X vs. 43:0 X	-3.332	-3.476 to -3.188	Yes
22059	22:0 X vs. 43:1 X	-3.246	-3.390 to -3.102	Yes
22060	22:0 X vs. 43:10 X	-2.962	-3.106 to -2.818	Yes
22061	22:0 X vs. 43:40 X	-0.2187	-0.3626 to -0.07471	Yes
22062	22:0 X vs. 43:50 X	-0.1240	-0.2680 to 0.01996	No
22063	22:0 X vs. 44:0 X	-3.356	-3.500 to -3.212	Yes
22064	22:0 X vs. 44:1 X	-3.245	-3.389 to -3.101	Yes
22065	22:0 X vs. 44:10 X	-3.025	-3.169 to -2.881	Yes
22066	22:0 X vs. 44:40 X	-0.2207	-0.3646 to -0.07671	Yes
22067	22:0 X vs. 44:50 X	-0.05633	-0.2003 to 0.08763	No
22068	22:0 X vs. 45:0 X	-3.220	-3.364 to -3.076	Yes
22069	22:0 X vs. 45:1 X	-3.022	-3.166 to -2.878	Yes
22070	22:0 X vs. 45:10 X	-2.905	-3.049 to -2.761	Yes
22071	22:0 X vs. 45:40 X	-0.2220	-0.3660 to -0.07804	Yes
22072	22:0 X vs. 45:50 X	-0.05333	-0.1973 to 0.09063	No
22073	22:0 X vs. 46:0 X	-3.025	-3.169 to -2.881	Yes
22074	22:0 X vs. 46:1 X	-2.992	-3.136 to -2.848	Yes
22075	22:0 X vs. 46:10 X	-2.900	-3.044 to -2.756	Yes
22076	22:0 X vs. 46:40 X	-0.2357	-0.3796 to -0.09171	Yes
22077	22:0 X vs. 46:50 X	-0.04633	-0.1903 to 0.09763	No
22078	22:0 X vs. 47:0 X	-3.000	-3.144 to -2.856	Yes
22079	22:0 X vs. 47:1 X	-2.904	-3.048 to -2.760	Yes
22080	22:0 X vs. 47:10 X	-2.886	-3.030 to -2.742	Yes
22081	22:0 X vs. 47:40 X	-0.2457	-0.3896 to -0.1017	Yes
22082	22:0 X vs. 47:50 X	-0.004333	-0.1483 to 0.1396	No
22083	22:0 X vs. 48:0 X	-2.988	-3.148 to -2.827	Yes
22084	22:0 X vs. 48:1 X	-2.891	-3.035 to -2.747	Yes
22085	22:0 X vs. 48:10 X	-2.886	-3.030 to -2.742	Yes
22086	22:0 X vs. 48:40 X	-0.2520	-0.3960 to -0.1080	Yes
22087	22:0 X vs. 48:50 X	-0.01167	-0.1556 to 0.1323	No
22088	22:0 X vs. 49:0 X	-2.988	-3.132 to -2.844	Yes
22089	22:0 X vs. 49:1 X	-2.891	-3.035 to -2.747	Yes
22090	22:0 X vs. 49:10 X	-2.886	-3.030 to -2.742	Yes
22091	22:0 X vs. 49:40 X	-0.2563	-0.4003 to -0.1124	Yes
22092	22:0 X vs. 49:50 X	-0.01100	-0.1550 to 0.1330	No
22093	22:0 X vs. 50:0 X	-2.988	-3.132 to -2.844	Yes
22094	22:0 X vs. 50:1 X	-2.891	-3.035 to -2.747	Yes
22095	22:0 X vs. 50:10 X	-2.886	-3.030 to -2.742	Yes

	2way ANOVA<br>Multiple comparisons	A<br>Data Set-A<br>Y	B<br>Data Set-B<br>Y	C<br>Data Set-C<br>Y
22096	22:**0 X** vs. 50:**40 X**	-0.2640	-0.4080 to -0.1200	Yes
22097	22:**0 X** vs. 50:**50 X**	-0.01067	-0.1546 to 0.1333	No
22098	22:**1 X** vs. 22:**10 X**	0.004667	-0.1393 to 0.1486	No
22099	22:**1 X** vs. 22:**40 X**	0.1043	-0.03963 to 0.2483	No
22100	22:**1 X** vs. 22:**50 X**	0.04167	-0.1023 to 0.1856	No
22101	22:**1 X** vs. 23:**0 X**	-0.07067	-0.2146 to 0.07329	No
22102	22:**1 X** vs. 23:**1 X**	-0.2603	-0.4213 to -0.09938	Yes
22103	22:**1 X** vs. 23:**10 X**	-0.0100	-0.1540 to 0.1340	No
22104	22:**1 X** vs. 23:**40 X**	0.1097	-0.05129 to 0.2706	No
22105	22:**1 X** vs. 23:**50 X**	0.07567	-0.06829 to 0.2196	No
22106	22:**1 X** vs. 24:**0 X**	-0.1880	-0.3320 to -0.04404	Yes
22107	22:**1 X** vs. 24:**1 X**	-0.3443	-0.5053 to -0.1834	Yes
22108	22:**1 X** vs. 24:**10 X**	-0.1718	-0.3328 to -0.01088	Yes
22109	22:**1 X** vs. 24:**40 X**	0.09333	-0.05063 to 0.2373	No
22110	22:**1 X** vs. 24:**50 X**	0.1717	0.02771 to 0.3156	Yes
22111	22:**1 X** vs. 25:**0 X**	-0.5253	-0.6863 to -0.3644	Yes
22112	22:**1 X** vs. 25:**1 X**	-0.7148	-0.8758 to -0.5539	Yes
22113	22:**1 X** vs. 25:**10 X**	-0.07633	-0.2799 to 0.1273	No
22114	22:**1 X** vs. 25:**40 X**	0.09133	-0.05263 to 0.2353	No
22115	22:**1 X** vs. 25:**50 X**	0.1170	-0.02696 to 0.2610	No
22116	22:**1 X** vs. 26:**0 X**	-0.7378	-0.8988 to -0.5769	Yes
22117	22:**1 X** vs. 26:**1 X**	-0.8508	-1.012 to -0.6899	Yes
22118	22:**1 X** vs. 26:**10 X**	-0.5373	-0.6983 to -0.3764	Yes
22119	22:**1 X** vs. 26:**40 X**	0.0170	-0.1270 to 0.1610	No
22120	22:**1 X** vs. 26:**50 X**	0.1567	0.01271 to 0.3006	Yes
22121	22:**1 X** vs. 27:**0 X**	-0.9257	-1.070 to -0.7817	Yes
22122	22:**1 X** vs. 27:**1 X**	-1.016	-1.177 to -0.8554	Yes
22123	22:**1 X** vs. 27:**10 X**	-0.8283	-0.9893 to -0.6674	Yes
22124	22:**1 X** vs. 27:**40 X**	0.0170	-0.1270 to 0.1610	No
22125	22:**1 X** vs. 27:**50 X**	0.1573	0.01337 to 0.3013	Yes
22126	22:**1 X** vs. 28:**0 X**	-0.8748	-1.036 to -0.7139	Yes
22127	22:**1 X** vs. 28:**1 X**	-0.9157	-1.060 to -0.7717	Yes
22128	22:**1 X** vs. 28:**10 X**	-0.7173	-0.8783 to -0.5564	Yes
22129	22:**1 X** vs. 28:**40 X**	0.004667	-0.1393 to 0.1486	No
22130	22:**1 X** vs. 28:**50 X**	0.1093	-0.03463 to 0.2533	No
22131	22:**1 X** vs. 29:**0 X**	-1.356	-1.517 to -1.195	Yes
22132	22:**1 X** vs. 29:**1 X**	-1.441	-1.602 to -1.280	Yes
22133	22:**1 X** vs. 29:**10 X**	-0.6558	-0.8168 to -0.4949	Yes
22134	22:**1 X** vs. 29:**40 X**	-0.1033	-0.2473 to 0.04063	No
22135	22:**1 X** vs. 29:**50 X**	0.1080	-0.03596 to 0.2520	No
22136	22:**1 X** vs. 30:**0 X**	-1.071	-1.232 to -0.9099	Yes
22137	22:**1 X** vs. 30:**1 X**	-1.392	-1.553 to -1.231	Yes
22138	22:**1 X** vs. 30:**10 X**	-0.7303	-0.8913 to -0.5694	Yes
22139	22:**1 X** vs. 30:**40 X**	-0.03033	-0.1743 to 0.1136	No
22140	22:**1 X** vs. 30:**50 X**	0.05567	-0.08829 to 0.1996	No

	2way ANOVA<br>Multiple comparisons	A<br>Data Set-A<br>Y	B<br>Data Set-B<br>Y	C<br>Data Set-C<br>Y
22141	22:1 X vs. 31:0 X	-1.389	-1.550 to -1.228	Yes
22142	22:1 X vs. 31:1 X	-1.379	-1.540 to -1.218	Yes
22143	22:1 X vs. 31:10 X	-0.8618	-1.023 to -0.7009	Yes
22144	22:1 X vs. 31:40 X	-0.1220	-0.2660 to 0.02196	No
22145	22:1 X vs. 31:50 X	0.07667	-0.06729 to 0.2206	No
22146	22:1 X vs. 32:0 X	-1.980	-2.124 to -1.836	Yes
22147	22:1 X vs. 32:1 X	-1.436	-1.597 to -1.275	Yes
22148	22:1 X vs. 32:10 X	-1.292	-1.436 to -1.148	Yes
22149	22:1 X vs. 32:40 X	-0.1183	-0.2623 to 0.02563	No
22150	22:1 X vs. 32:50 X	0.07700	-0.06696 to 0.2210	No
22151	22:1 X vs. 33:0 X	-1.984	-2.128 to -1.840	Yes
22152	22:1 X vs. 33:1 X	-1.799	-1.943 to -1.655	Yes
22153	22:1 X vs. 33:10 X	-1.459	-1.603 to -1.315	Yes
22154	22:1 X vs. 33:40 X	-0.1370	-0.2810 to 0.006960	No
22155	22:1 X vs. 33:50 X	0.06200	-0.08196 to 0.2060	No
22156	22:1 X vs. 34:0 X	-1.909	-2.053 to -1.765	Yes
22157	22:1 X vs. 34:1 X	-1.916	-2.060 to -1.772	Yes
22158	22:1 X vs. 34:10 X	-1.549	-1.693 to -1.405	Yes
22159	22:1 X vs. 34:40 X	-0.1490	-0.2930 to -0.005040	Yes
22160	22:1 X vs. 34:50 X	-0.0870	-0.2310 to 0.05696	No
22161	22:1 X vs. 35:0 X	-2.257	-2.401 to -2.113	Yes
22162	22:1 X vs. 35:1 X	-2.023	-2.167 to -1.879	Yes
22163	22:1 X vs. 35:10 X	-1.511	-1.655 to -1.367	Yes
22164	22:1 X vs. 35:40 X	-0.1580	-0.3020 to -0.01404	Yes
22165	22:1 X vs. 35:50 X	-0.09967	-0.2436 to 0.04429	No
22166	22:1 X vs. 36:0 X	-2.464	-2.625 to -2.303	Yes
22167	22:1 X vs. 36:1 X	-2.160	-2.304 to -2.016	Yes
22168	22:1 X vs. 36:10 X	-1.517	-1.661 to -1.373	Yes
22169	22:1 X vs. 36:40 X	-0.2133	-0.3573 to -0.06937	Yes
22170	22:1 X vs. 36:50 X	-0.06300	-0.2070 to 0.08096	No
22171	22:1 X vs. 37:0 X	-2.848	-2.992 to -2.704	Yes
22172	22:1 X vs. 37:1 X	-2.230	-2.374 to -2.086	Yes
22173	22:1 X vs. 37:10 X	-1.518	-1.662 to -1.374	Yes
22174	22:1 X vs. 37:40 X	-0.2507	-0.3946 to -0.1067	Yes
22175	22:1 X vs. 37:50 X	-0.07167	-0.2156 to 0.07229	No
22176	22:1 X vs. 38:0 X	-2.793	-2.937 to -2.649	Yes
22177	22:1 X vs. 38:1 X	-2.348	-2.492 to -2.204	Yes
22178	22:1 X vs. 38:10 X	-1.794	-1.938 to -1.650	Yes
22179	22:1 X vs. 38:40 X	-0.2550	-0.3990 to -0.1110	Yes
22180	22:1 X vs. 38:50 X	-0.07567	-0.2196 to 0.06829	No
22181	22:1 X vs. 39:0 X	-3.023	-3.167 to -2.879	Yes
22182	22:1 X vs. 39:1 X	-2.915	-3.059 to -2.771	Yes
22183	22:1 X vs. 39:10 X	-1.939	-2.083 to -1.795	Yes
22184	22:1 X vs. 39:40 X	-0.2610	-0.4050 to -0.1170	Yes
22185	22:1 X vs. 39:50 X	-0.1067	-0.2506 to 0.03729	No

	2way ANOVA<br>Multiple comparisons	A<br>Data Set-A<br>Y	B<br>Data Set-B<br>Y	C<br>Data Set-C<br>Y
22186	22:1 X vs. 40:0 X	-3.064	-3.208 to -2.920	Yes
22187	22:1 X vs. 40:1 X	-2.990	-3.134 to -2.846	Yes
22188	22:1 X vs. 40:10 X	-2.195	-2.339 to -2.051	Yes
22189	22:1 X vs. 40:40 X	-0.2657	-0.4096 to -0.1217	Yes
22190	22:1 X vs. 40:50 X	-0.1130	-0.2570 to 0.03096	No
22191	22:1 X vs. 41:0 X	-3.105	-3.249 to -2.961	Yes
22192	22:1 X vs. 41:1 X	-3.104	-3.248 to -2.960	Yes
22193	22:1 X vs. 41:10 X	-2.249	-2.393 to -2.105	Yes
22194	22:1 X vs. 41:40 X	-0.2130	-0.3570 to -0.06904	Yes
22195	22:1 X vs. 41:50 X	-0.1257	-0.2696 to 0.01829	No
22196	22:1 X vs. 42:0 X	-3.247	-3.391 to -3.103	Yes
22197	22:1 X vs. 42:1 X	-3.267	-3.411 to -3.123	Yes
22198	22:1 X vs. 42:10 X	-2.249	-2.393 to -2.105	Yes
22199	22:1 X vs. 42:40 X	-0.2257	-0.3696 to -0.08171	Yes
22200	22:1 X vs. 42:50 X	-0.1290	-0.2730 to 0.01496	No
22201	22:1 X vs. 43:0 X	-3.337	-3.481 to -3.193	Yes
22202	22:1 X vs. 43:1 X	-3.251	-3.395 to -3.107	Yes
22203	22:1 X vs. 43:10 X	-2.967	-3.111 to -2.823	Yes
22204	22:1 X vs. 43:40 X	-0.2240	-0.3680 to -0.08004	Yes
22205	22:1 X vs. 43:50 X	-0.1293	-0.2733 to 0.01463	No
22206	22:1 X vs. 44:0 X	-3.361	-3.505 to -3.217	Yes
22207	22:1 X vs. 44:1 X	-3.251	-3.395 to -3.107	Yes
22208	22:1 X vs. 44:10 X	-3.030	-3.174 to -2.886	Yes
22209	22:1 X vs. 44:40 X	-0.2260	-0.3700 to -0.08204	Yes
22210	22:1 X vs. 44:50 X	-0.06167	-0.2056 to 0.08229	No
22211	22:1 X vs. 45:0 X	-3.226	-3.370 to -3.082	Yes
22212	22:1 X vs. 45:1 X	-3.028	-3.172 to -2.884	Yes
22213	22:1 X vs. 45:10 X	-2.910	-3.054 to -2.766	Yes
22214	22:1 X vs. 45:40 X	-0.2273	-0.3713 to -0.08337	Yes
22215	22:1 X vs. 45:50 X	-0.05867	-0.2026 to 0.08529	No
22216	22:1 X vs. 46:0 X	-3.030	-3.174 to -2.886	Yes
22217	22:1 X vs. 46:1 X	-2.997	-3.141 to -2.853	Yes
22218	22:1 X vs. 46:10 X	-2.905	-3.049 to -2.761	Yes
22219	22:1 X vs. 46:40 X	-0.2410	-0.3850 to -0.09704	Yes
22220	22:1 X vs. 46:50 X	-0.05167	-0.1956 to 0.09229	No
22221	22:1 X vs. 47:0 X	-3.006	-3.150 to -2.862	Yes
22222	22:1 X vs. 47:1 X	-2.910	-3.054 to -2.766	Yes
22223	22:1 X vs. 47:10 X	-2.891	-3.035 to -2.747	Yes
22224	22:1 X vs. 47:40 X	-0.2510	-0.3950 to -0.1070	Yes
22225	22:1 X vs. 47:50 X	-0.009667	-0.1536 to 0.1343	No
22226	22:1 X vs. 48:0 X	-2.993	-3.154 to -2.832	Yes
22227	22:1 X vs. 48:1 X	-2.897	-3.041 to -2.753	Yes
22228	22:1 X vs. 48:10 X	-2.891	-3.035 to -2.747	Yes
22229	22:1 X vs. 48:40 X	-0.2573	-0.4013 to -0.1134	Yes
22230	22:1 X vs. 48:50 X	-0.0170	-0.1610 to 0.1270	No

	2way ANOVA<br>Multiple comparisons	A<br>Data Set-A<br>Y	B<br>Data Set-B<br>Y	C<br>Data Set-C<br>Y
22231	22:1 X vs. 49:0 X	-2.993	-3.137 to -2.849	Yes
22232	22:1 X vs. 49:1 X	-2.897	-3.041 to -2.753	Yes
22233	22:1 X vs. 49:10 X	-2.891	-3.035 to -2.747	Yes
22234	22:1 X vs. 49:40 X	-0.2617	-0.4056 to -0.1177	Yes
22235	22:1 X vs. 49:50 X	-0.01633	-0.1603 to 0.1276	No
22236	22:1 X vs. 50:0 X	-2.993	-3.137 to -2.849	Yes
22237	22:1 X vs. 50:1 X	-2.897	-3.041 to -2.753	Yes
22238	22:1 X vs. 50:10 X	-2.891	-3.035 to -2.747	Yes
22239	22:1 X vs. 50:40 X	-0.2693	-0.4133 to -0.1254	Yes
22240	22:1 X vs. 50:50 X	-0.01600	-0.1600 to 0.1280	No
22241	22:10 X vs. 22:40 X	0.09967	-0.04429 to 0.2436	No
22242	22:10 X vs. 22:50 X	0.0370	-0.1070 to 0.1810	No
22243	22:10 X vs. 23:0 X	-0.07533	-0.2193 to 0.06863	No
22244	22:10 X vs. 23:1 X	-0.2650	-0.4260 to -0.1040	Yes
22245	22:10 X vs. 23:10 X	-0.01467	-0.1586 to 0.1293	No
22246	22:10 X vs. 23:40 X	0.1050	-0.05595 to 0.2660	No
22247	22:10 X vs. 23:50 X	0.0710	-0.07296 to 0.2150	No
22248	22:10 X vs. 24:0 X	-0.1927	-0.3366 to -0.04871	Yes
22249	22:10 X vs. 24:1 X	-0.3490	-0.5100 to -0.1880	Yes
22250	22:10 X vs. 24:10 X	-0.1765	-0.3375 to -0.01555	Yes
22251	22:10 X vs. 24:40 X	0.08867	-0.05529 to 0.2326	No
22252	22:10 X vs. 24:50 X	0.1670	0.02304 to 0.3110	Yes
22253	22:10 X vs. 25:0 X	-0.5300	-0.6910 to -0.3690	Yes
22254	22:10 X vs. 25:1 X	-0.7195	-0.8805 to -0.5585	Yes
22255	22:10 X vs. 25:10 X	-0.08100	-0.2846 to 0.1226	No
22256	22:10 X vs. 25:40 X	0.08667	-0.05729 to 0.2306	No
22257	22:10 X vs. 25:50 X	0.1123	-0.03163 to 0.2563	No
22258	22:10 X vs. 26:0 X	-0.7425	-0.9035 to -0.5815	Yes
22259	22:10 X vs. 26:1 X	-0.8555	-1.016 to -0.6945	Yes
22260	22:10 X vs. 26:10 X	-0.5420	-0.7030 to -0.3810	Yes
22261	22:10 X vs. 26:40 X	0.01233	-0.1316 to 0.1563	No
22262	22:10 X vs. 26:50 X	0.1520	0.008040 to 0.2960	Yes
22263	22:10 X vs. 27:0 X	-0.9303	-1.074 to -0.7864	Yes
22264	22:10 X vs. 27:1 X	-1.021	-1.182 to -0.8600	Yes
22265	22:10 X vs. 27:10 X	-0.8330	-0.9940 to -0.6720	Yes
22266	22:10 X vs. 27:40 X	0.01233	-0.1316 to 0.1563	No
22267	22:10 X vs. 27:50 X	0.1527	0.008707 to 0.2966	Yes
22268	22:10 X vs. 28:0 X	-0.8795	-1.040 to -0.7185	Yes
22269	22:10 X vs. 28:1 X	-0.9203	-1.064 to -0.7764	Yes
22270	22:10 X vs. 28:10 X	-0.7220	-0.8830 to -0.5610	Yes
22271	22:10 X vs. 28:40 X	0.0	-0.1440 to 0.1440	No
22272	22:10 X vs. 28:50 X	0.1047	-0.03929 to 0.2486	No
22273	22:10 X vs. 29:0 X	-1.361	-1.522 to -1.200	Yes
22274	22:10 X vs. 29:1 X	-1.446	-1.607 to -1.285	Yes
22275	22:10 X vs. 29:10 X	-0.6605	-0.8215 to -0.4995	Yes

	2way ANOVA<br>Multiple comparisons	A<br>Data Set-A<br>Y	B<br>Data Set-B<br>Y	C<br>Data Set-C<br>Y
22276	22:10 X vs. 29:40 X	-0.1080	-0.2520 to 0.03596	No
22277	22:10 X vs. 29:50 X	0.1033	-0.04063 to 0.2473	No
22278	22:10 X vs. 30:0 X	-1.076	-1.236 to -0.9145	Yes
22279	22:10 X vs. 30:1 X	-1.397	-1.558 to -1.236	Yes
22280	22:10 X vs. 30:10 X	-0.7350	-0.8960 to -0.5740	Yes
22281	22:10 X vs. 30:40 X	-0.03500	-0.1790 to 0.1090	No
22282	22:10 X vs. 30:50 X	0.05100	-0.09296 to 0.1950	No
22283	22:10 X vs. 31:0 X	-1.394	-1.554 to -1.233	Yes
22284	22:10 X vs. 31:1 X	-1.384	-1.545 to -1.223	Yes
22285	22:10 X vs. 31:10 X	-0.8665	-1.027 to -0.7055	Yes
22286	22:10 X vs. 31:40 X	-0.1267	-0.2706 to 0.01729	No
22287	22:10 X vs. 31:50 X	0.0720	-0.07196 to 0.2160	No
22288	22:10 X vs. 32:0 X	-1.985	-2.129 to -1.841	Yes
22289	22:10 X vs. 32:1 X	-1.441	-1.602 to -1.280	Yes
22290	22:10 X vs. 32:10 X	-1.297	-1.441 to -1.153	Yes
22291	22:10 X vs. 32:40 X	-0.1230	-0.2670 to 0.02096	No
22292	22:10 X vs. 32:50 X	0.07233	-0.07163 to 0.2163	No
22293	22:10 X vs. 33:0 X	-1.989	-2.133 to -1.845	Yes
22294	22:10 X vs. 33:1 X	-1.803	-1.947 to -1.659	Yes
22295	22:10 X vs. 33:10 X	-1.464	-1.608 to -1.320	Yes
22296	22:10 X vs. 33:40 X	-0.1417	-0.2856 to 0.002293	No
22297	22:10 X vs. 33:50 X	0.05733	-0.08663 to 0.2013	No
22298	22:10 X vs. 34:0 X	-1.913	-2.057 to -1.769	Yes
22299	22:10 X vs. 34:1 X	-1.921	-2.065 to -1.777	Yes
22300	22:10 X vs. 34:10 X	-1.554	-1.698 to -1.410	Yes
22301	22:10 X vs. 34:40 X	-0.1537	-0.2976 to -0.009707	Yes
22302	22:10 X vs. 34:50 X	-0.09167	-0.2356 to 0.05229	No
22303	22:10 X vs. 35:0 X	-2.262	-2.406 to -2.118	Yes
22304	22:10 X vs. 35:1 X	-2.028	-2.172 to -1.884	Yes
22305	22:10 X vs. 35:10 X	-1.516	-1.660 to -1.372	Yes
22306	22:10 X vs. 35:40 X	-0.1627	-0.3066 to -0.01871	Yes
22307	22:10 X vs. 35:50 X	-0.1043	-0.2483 to 0.03963	No
22308	22:10 X vs. 36:0 X	-2.469	-2.629 to -2.308	Yes
22309	22:10 X vs. 36:1 X	-2.164	-2.308 to -2.020	Yes
22310	22:10 X vs. 36:10 X	-1.521	-1.665 to -1.377	Yes
22311	22:10 X vs. 36:40 X	-0.2180	-0.3620 to -0.07404	Yes
22312	22:10 X vs. 36:50 X	-0.06767	-0.2116 to 0.07629	No
22313	22:10 X vs. 37:0 X	-2.852	-2.996 to -2.708	Yes
22314	22:10 X vs. 37:1 X	-2.234	-2.378 to -2.090	Yes
22315	22:10 X vs. 37:10 X	-1.523	-1.667 to -1.379	Yes
22316	22:10 X vs. 37:40 X	-0.2553	-0.3993 to -0.1114	Yes
22317	22:10 X vs. 37:50 X	-0.07633	-0.2203 to 0.06763	No
22318	22:10 X vs. 38:0 X	-2.797	-2.941 to -2.653	Yes
22319	22:10 X vs. 38:1 X	-2.353	-2.497 to -2.209	Yes
22320	22:10 X vs. 38:10 X	-1.798	-1.942 to -1.654	Yes

	2way ANOVA<br>Multiple comparisons	A<br>Data Set-A<br>Y	B<br>Data Set-B<br>Y	C<br>Data Set-C<br>Y
22321	22:**10 X** vs. 38:**40 X**	-0.2597	-0.4036 to -0.1157	Yes
22322	22:**10 X** vs. 38:**50 X**	-0.08033	-0.2243 to 0.06363	No
22323	22:**10 X** vs. 39:**0 X**	-3.028	-3.172 to -2.884	Yes
22324	22:**10 X** vs. 39:**1 X**	-2.920	-3.064 to -2.776	Yes
22325	22:**10 X** vs. 39:**10 X**	-1.943	-2.087 to -1.799	Yes
22326	22:**10 X** vs. 39:**40 X**	-0.2657	-0.4096 to -0.1217	Yes
22327	22:**10 X** vs. 39:**50 X**	-0.1113	-0.2553 to 0.03263	No
22328	22:**10 X** vs. 40:**0 X**	-3.069	-3.213 to -2.925	Yes
22329	22:**10 X** vs. 40:**1 X**	-2.995	-3.139 to -2.851	Yes
22330	22:**10 X** vs. 40:**10 X**	-2.200	-2.344 to -2.056	Yes
22331	22:**10 X** vs. 40:**40 X**	-0.2703	-0.4143 to -0.1264	Yes
22332	22:**10 X** vs. 40:**50 X**	-0.1177	-0.2616 to 0.02629	No
22333	22:**10 X** vs. 41:**0 X**	-3.110	-3.254 to -2.966	Yes
22334	22:**10 X** vs. 41:**1 X**	-3.109	-3.253 to -2.965	Yes
22335	22:**10 X** vs. 41:**10 X**	-2.253	-2.397 to -2.109	Yes
22336	22:**10 X** vs. 41:**40 X**	-0.2177	-0.3616 to -0.07371	Yes
22337	22:**10 X** vs. 41:**50 X**	-0.1303	-0.2743 to 0.01363	No
22338	22:**10 X** vs. 42:**0 X**	-3.252	-3.396 to -3.108	Yes
22339	22:**10 X** vs. 42:**1 X**	-3.271	-3.415 to -3.127	Yes
22340	22:**10 X** vs. 42:**10 X**	-2.254	-2.398 to -2.110	Yes
22341	22:**10 X** vs. 42:**40 X**	-0.2303	-0.3743 to -0.08637	Yes
22342	22:**10 X** vs. 42:**50 X**	-0.1337	-0.2776 to 0.01029	No
22343	22:**10 X** vs. 43:**0 X**	-3.342	-3.486 to -3.198	Yes
22344	22:**10 X** vs. 43:**1 X**	-3.256	-3.400 to -3.112	Yes
22345	22:**10 X** vs. 43:**10 X**	-2.972	-3.116 to -2.828	Yes
22346	22:**10 X** vs. 43:**40 X**	-0.2287	-0.3726 to -0.08471	Yes
22347	22:**10 X** vs. 43:**50 X**	-0.1340	-0.2780 to 0.009960	No
22348	22:**10 X** vs. 44:**0 X**	-3.366	-3.510 to -3.222	Yes
22349	22:**10 X** vs. 44:**1 X**	-3.255	-3.399 to -3.111	Yes
22350	22:**10 X** vs. 44:**10 X**	-3.035	-3.179 to -2.891	Yes
22351	22:**10 X** vs. 44:**40 X**	-0.2307	-0.3746 to -0.08671	Yes
22352	22:**10 X** vs. 44:**50 X**	-0.06633	-0.2103 to 0.07763	No
22353	22:**10 X** vs. 45:**0 X**	-3.230	-3.374 to -3.086	Yes
22354	22:**10 X** vs. 45:**1 X**	-3.032	-3.176 to -2.888	Yes
22355	22:**10 X** vs. 45:**10 X**	-2.915	-3.059 to -2.771	Yes
22356	22:**10 X** vs. 45:**40 X**	-0.2320	-0.3760 to -0.08804	Yes
22357	22:**10 X** vs. 45:**50 X**	-0.06333	-0.2073 to 0.08063	No
22358	22:**10 X** vs. 46:**0 X**	-3.035	-3.179 to -2.891	Yes
22359	22:**10 X** vs. 46:**1 X**	-3.002	-3.146 to -2.858	Yes
22360	22:**10 X** vs. 46:**10 X**	-2.910	-3.054 to -2.766	Yes
22361	22:**10 X** vs. 46:**40 X**	-0.2457	-0.3896 to -0.1017	Yes
22362	22:**10 X** vs. 46:**50 X**	-0.05633	-0.2003 to 0.08763	No
22363	22:**10 X** vs. 47:**0 X**	-3.010	-3.154 to -2.866	Yes
22364	22:**10 X** vs. 47:**1 X**	-2.914	-3.058 to -2.770	Yes
22365	22:**10 X** vs. 47:**10 X**	-2.896	-3.040 to -2.752	Yes

2way ANOVA<br>Multiple comparisons	A<br>Data Set-A<br>Y	B<br>Data Set-B<br>Y	C<br>Data Set-C<br>Y
22366 22:10 X vs. 47:40 X	-0.2557	-0.3996 to -0.1117	Yes
22367 22:10 X vs. 47:50 X	-0.01433	-0.1583 to 0.1296	No
22368 22:10 X vs. 48:0 X	-2.998	-3.158 to -2.837	Yes
22369 22:10 X vs. 48:1 X	-2.901	-3.045 to -2.757	Yes
22370 22:10 X vs. 48:10 X	-2.896	-3.040 to -2.752	Yes
22371 22:10 X vs. 48:40 X	-0.2620	-0.4060 to -0.1180	Yes
22372 22:10 X vs. 48:50 X	-0.02167	-0.1656 to 0.1223	No
22373 22:10 X vs. 49:0 X	-2.998	-3.142 to -2.854	Yes
22374 22:10 X vs. 49:1 X	-2.901	-3.045 to -2.757	Yes
22375 22:10 X vs. 49:10 X	-2.896	-3.040 to -2.752	Yes
22376 22:10 X vs. 49:40 X	-0.2663	-0.4103 to -0.1224	Yes
22377 22:10 X vs. 49:50 X	-0.0210	-0.1650 to 0.1230	No
22378 22:10 X vs. 50:0 X	-2.998	-3.142 to -2.854	Yes
22379 22:10 X vs. 50:1 X	-2.901	-3.045 to -2.757	Yes
22380 22:10 X vs. 50:10 X	-2.896	-3.040 to -2.752	Yes
22381 22:10 X vs. 50:40 X	-0.2740	-0.4180 to -0.1300	Yes
22382 22:10 X vs. 50:50 X	-0.02067	-0.1646 to 0.1233	No
22383 22:40 X vs. 22:50 X	-0.06267	-0.2066 to 0.08129	No
22384 22:40 X vs. 23:0 X	-0.1750	-0.3190 to -0.03104	Yes
22385 22:40 X vs. 23:1 X	-0.3647	-0.5256 to -0.2037	Yes
22386 22:40 X vs. 23:10 X	-0.1143	-0.2583 to 0.02963	No
22387 22:40 X vs. 23:40 X	0.005333	-0.1556 to 0.1663	No
22388 22:40 X vs. 23:50 X	-0.02867	-0.1726 to 0.1153	No
22389 22:40 X vs. 24:0 X	-0.2923	-0.4363 to -0.1484	Yes
22390 22:40 X vs. 24:1 X	-0.4487	-0.6096 to -0.2877	Yes
22391 22:40 X vs. 24:10 X	-0.2762	-0.4371 to -0.1152	Yes
22392 22:40 X vs. 24:40 X	-0.01100	-0.1550 to 0.1330	No
22393 22:40 X vs. 24:50 X	0.06733	-0.07663 to 0.2113	No
22394 22:40 X vs. 25:0 X	-0.6297	-0.7906 to -0.4687	Yes
22395 22:40 X vs. 25:1 X	-0.8192	-0.9801 to -0.6582	Yes
22396 22:40 X vs. 25:10 X	-0.1807	-0.3843 to 0.02292	No
22397 22:40 X vs. 25:40 X	-0.01300	-0.1570 to 0.1310	No
22398 22:40 X vs. 25:50 X	0.01267	-0.1313 to 0.1566	No
22399 22:40 X vs. 26:0 X	-0.8422	-1.003 to -0.6812	Yes
22400 22:40 X vs. 26:1 X	-0.9552	-1.116 to -0.7942	Yes
22401 22:40 X vs. 26:10 X	-0.6417	-0.8026 to -0.4807	Yes
22402 22:40 X vs. 26:40 X	-0.08733	-0.2313 to 0.05663	No
22403 22:40 X vs. 26:50 X	0.05233	-0.09163 to 0.1963	No
22404 22:40 X vs. 27:0 X	-1.030	-1.174 to -0.8860	Yes
22405 22:40 X vs. 27:1 X	-1.121	-1.282 to -0.9597	Yes
22406 22:40 X vs. 27:10 X	-0.9327	-1.094 to -0.7717	Yes
22407 22:40 X vs. 27:40 X	-0.08733	-0.2313 to 0.05663	No
22408 22:40 X vs. 27:50 X	0.05300	-0.09096 to 0.1970	No
22409 22:40 X vs. 28:0 X	-0.9792	-1.140 to -0.8182	Yes
22410 22:40 X vs. 28:1 X	-1.020	-1.164 to -0.8760	Yes

2way ANOVA<br>Multiple comparisons	A<br>Data Set-A<br>Y	B<br>Data Set-B<br>Y	C<br>Data Set-C<br>Y
22411 22:**40 X** vs. 28:**10 X**	-0.8217	-0.9826 to -0.6607	Yes
22412 22:**40 X** vs. 28:**40 X**	-0.09967	-0.2436 to 0.04429	No
22413 22:**40 X** vs. 28:**50 X**	0.005000	-0.1390 to 0.1490	No
22414 22:**40 X** vs. 29:**0 X**	-1.461	-1.622 to -1.300	Yes
22415 22:**40 X** vs. 29:**1 X**	-1.546	-1.707 to -1.385	Yes
22416 22:**40 X** vs. 29:**10 X**	-0.7602	-0.9211 to -0.5992	Yes
22417 22:**40 X** vs. 29:**40 X**	-0.2077	-0.3516 to -0.06371	Yes
22418 22:**40 X** vs. 29:**50 X**	0.003667	-0.1403 to 0.1476	No
22419 22:**40 X** vs. 30:**0 X**	-1.175	-1.336 to -1.014	Yes
22420 22:**40 X** vs. 30:**1 X**	-1.497	-1.658 to -1.336	Yes
22421 22:**40 X** vs. 30:**10 X**	-0.8347	-0.9956 to -0.6737	Yes
22422 22:**40 X** vs. 30:**40 X**	-0.1347	-0.2786 to 0.009293	No
22423 22:**40 X** vs. 30:**50 X**	-0.04867	-0.1926 to 0.09529	No
22424 22:**40 X** vs. 31:**0 X**	-1.493	-1.654 to -1.332	Yes
22425 22:**40 X** vs. 31:**1 X**	-1.484	-1.645 to -1.323	Yes
22426 22:**40 X** vs. 31:**10 X**	-0.9662	-1.127 to -0.8052	Yes
22427 22:**40 X** vs. 31:**40 X**	-0.2263	-0.3703 to -0.08237	Yes
22428 22:**40 X** vs. 31:**50 X**	-0.02767	-0.1716 to 0.1163	No
22429 22:**40 X** vs. 32:**0 X**	-2.085	-2.229 to -1.941	Yes
22430 22:**40 X** vs. 32:**1 X**	-1.541	-1.702 to -1.380	Yes
22431 22:**40 X** vs. 32:**10 X**	-1.397	-1.541 to -1.253	Yes
22432 22:**40 X** vs. 32:**40 X**	-0.2227	-0.3666 to -0.07871	Yes
22433 22:**40 X** vs. 32:**50 X**	-0.02733	-0.1713 to 0.1166	No
22434 22:**40 X** vs. 33:**0 X**	-2.088	-2.232 to -1.944	Yes
22435 22:**40 X** vs. 33:**1 X**	-1.903	-2.047 to -1.759	Yes
22436 22:**40 X** vs. 33:**10 X**	-1.563	-1.707 to -1.419	Yes
22437 22:**40 X** vs. 33:**40 X**	-0.2413	-0.3853 to -0.09737	Yes
22438 22:**40 X** vs. 33:**50 X**	-0.04233	-0.1863 to 0.1016	No
22439 22:**40 X** vs. 34:**0 X**	-2.013	-2.157 to -1.869	Yes
22440 22:**40 X** vs. 34:**1 X**	-2.020	-2.164 to -1.876	Yes
22441 22:**40 X** vs. 34:**10 X**	-1.654	-1.798 to -1.510	Yes
22442 22:**40 X** vs. 34:**40 X**	-0.2533	-0.3973 to -0.1094	Yes
22443 22:**40 X** vs. 34:**50 X**	-0.1913	-0.3353 to -0.04737	Yes
22444 22:**40 X** vs. 35:**0 X**	-2.362	-2.506 to -2.218	Yes
22445 22:**40 X** vs. 35:**1 X**	-2.127	-2.271 to -1.983	Yes
22446 22:**40 X** vs. 35:**10 X**	-1.615	-1.759 to -1.471	Yes
22447 22:**40 X** vs. 35:**40 X**	-0.2623	-0.4063 to -0.1184	Yes
22448 22:**40 X** vs. 35:**50 X**	-0.2040	-0.3480 to -0.06004	Yes
22449 22:**40 X** vs. 36:**0 X**	-2.568	-2.729 to -2.407	Yes
22450 22:**40 X** vs. 36:**1 X**	-2.264	-2.408 to -2.120	Yes
22451 22:**40 X** vs. 36:**10 X**	-1.621	-1.765 to -1.477	Yes
22452 22:**40 X** vs. 36:**40 X**	-0.3177	-0.4616 to -0.1737	Yes
22453 22:**40 X** vs. 36:**50 X**	-0.1673	-0.3113 to -0.02337	Yes
22454 22:**40 X** vs. 37:**0 X**	-2.952	-3.096 to -2.808	Yes
22455 22:**40 X** vs. 37:**1 X**	-2.334	-2.478 to -2.190	Yes

	2way ANOVA Multiple comparisons	A Data Set-A Y	B Data Set-B Y	C Data Set-C Y
22456	22:**40 X** vs. 37:**10 X**	-1.623	-1.767 to -1.479	Yes
22457	22:**40 X** vs. 37:**40 X**	-0.3550	-0.4990 to -0.2110	Yes
22458	22:**40 X** vs. 37:**50 X**	-0.1760	-0.3200 to -0.03204	Yes
22459	22:**40 X** vs. 38:**0 X**	-2.897	-3.041 to -2.753	Yes
22460	22:**40 X** vs. 38:**1 X**	-2.453	-2.597 to -2.309	Yes
22461	22:**40 X** vs. 38:**10 X**	-1.898	-2.042 to -1.754	Yes
22462	22:**40 X** vs. 38:**40 X**	-0.3593	-0.5033 to -0.2154	Yes
22463	22:**40 X** vs. 38:**50 X**	-0.1800	-0.3240 to -0.03604	Yes
22464	22:**40 X** vs. 39:**0 X**	-3.128	-3.272 to -2.984	Yes
22465	22:**40 X** vs. 39:**1 X**	-3.019	-3.163 to -2.875	Yes
22466	22:**40 X** vs. 39:**10 X**	-2.043	-2.187 to -1.899	Yes
22467	22:**40 X** vs. 39:**40 X**	-0.3653	-0.5093 to -0.2214	Yes
22468	22:**40 X** vs. 39:**50 X**	-0.2110	-0.3550 to -0.06704	Yes
22469	22:**40 X** vs. 40:**0 X**	-3.169	-3.313 to -3.025	Yes
22470	22:**40 X** vs. 40:**1 X**	-3.095	-3.239 to -2.951	Yes
22471	22:**40 X** vs. 40:**10 X**	-2.299	-2.443 to -2.155	Yes
22472	22:**40 X** vs. 40:**40 X**	-0.3700	-0.5140 to -0.2260	Yes
22473	22:**40 X** vs. 40:**50 X**	-0.2173	-0.3613 to -0.07337	Yes
22474	22:**40 X** vs. 41:**0 X**	-3.210	-3.354 to -3.066	Yes
22475	22:**40 X** vs. 41:**1 X**	-3.209	-3.353 to -3.065	Yes
22476	22:**40 X** vs. 41:**10 X**	-2.353	-2.497 to -2.209	Yes
22477	22:**40 X** vs. 41:**40 X**	-0.3173	-0.4613 to -0.1734	Yes
22478	22:**40 X** vs. 41:**50 X**	-0.2300	-0.3740 to -0.08604	Yes
22479	22:**40 X** vs. 42:**0 X**	-3.351	-3.495 to -3.207	Yes
22480	22:**40 X** vs. 42:**1 X**	-3.371	-3.515 to -3.227	Yes
22481	22:**40 X** vs. 42:**10 X**	-2.353	-2.497 to -2.209	Yes
22482	22:**40 X** vs. 42:**40 X**	-0.3300	-0.4740 to -0.1860	Yes
22483	22:**40 X** vs. 42:**50 X**	-0.2333	-0.3773 to -0.08937	Yes
22484	22:**40 X** vs. 43:**0 X**	-3.441	-3.585 to -3.297	Yes
22485	22:**40 X** vs. 43:**1 X**	-3.356	-3.500 to -3.212	Yes
22486	22:**40 X** vs. 43:**10 X**	-3.071	-3.215 to -2.927	Yes
22487	22:**40 X** vs. 43:**40 X**	-0.3283	-0.4723 to -0.1844	Yes
22488	22:**40 X** vs. 43:**50 X**	-0.2337	-0.3776 to -0.08971	Yes
22489	22:**40 X** vs. 44:**0 X**	-3.465	-3.609 to -3.321	Yes
22490	22:**40 X** vs. 44:**1 X**	-3.355	-3.499 to -3.211	Yes
22491	22:**40 X** vs. 44:**10 X**	-3.135	-3.279 to -2.991	Yes
22492	22:**40 X** vs. 44:**40 X**	-0.3303	-0.4743 to -0.1864	Yes
22493	22:**40 X** vs. 44:**50 X**	-0.1660	-0.3100 to -0.02204	Yes
22494	22:**40 X** vs. 45:**0 X**	-3.330	-3.474 to -3.186	Yes
22495	22:**40 X** vs. 45:**1 X**	-3.132	-3.276 to -2.988	Yes
22496	22:**40 X** vs. 45:**10 X**	-3.014	-3.158 to -2.870	Yes
22497	22:**40 X** vs. 45:**40 X**	-0.3317	-0.4756 to -0.1877	Yes
22498	22:**40 X** vs. 45:**50 X**	-0.1630	-0.3070 to -0.01904	Yes
22499	22:**40 X** vs. 46:**0 X**	-3.134	-3.278 to -2.990	Yes
22500	22:**40 X** vs. 46:**1 X**	-3.101	-3.245 to -2.957	Yes

	2way ANOVA<br>Multiple comparisons	A<br>Data Set-A<br>Y	B<br>Data Set-B<br>Y	C<br>Data Set-C<br>Y
22501	22:**40 X** vs. 46:**10 X**	-3.009	-3.153 to -2.865	Yes
22502	22:**40 X** vs. 46:**40 X**	-0.3453	-0.4893 to -0.2014	Yes
22503	22:**40 X** vs. 46:**50 X**	-0.1560	-0.3000 to -0.01204	Yes
22504	22:**40 X** vs. 47:**0 X**	-3.110	-3.254 to -2.966	Yes
22505	22:**40 X** vs. 47:**1 X**	-3.014	-3.158 to -2.870	Yes
22506	22:**40 X** vs. 47:**10 X**	-2.995	-3.139 to -2.851	Yes
22507	22:**40 X** vs. 47:**40 X**	-0.3553	-0.4993 to -0.2114	Yes
22508	22:**40 X** vs. 47:**50 X**	-0.1140	-0.2580 to 0.02996	No
22509	22:**40 X** vs. 48:**0 X**	-3.097	-3.258 to -2.936	Yes
22510	22:**40 X** vs. 48:**1 X**	-3.001	-3.145 to -2.857	Yes
22511	22:**40 X** vs. 48:**10 X**	-2.995	-3.139 to -2.851	Yes
22512	22:**40 X** vs. 48:**40 X**	-0.3617	-0.5056 to -0.2177	Yes
22513	22:**40 X** vs. 48:**50 X**	-0.1213	-0.2653 to 0.02263	No
22514	22:**40 X** vs. 49:**0 X**	-3.097	-3.241 to -2.953	Yes
22515	22:**40 X** vs. 49:**1 X**	-3.001	-3.145 to -2.857	Yes
22516	22:**40 X** vs. 49:**10 X**	-2.995	-3.139 to -2.851	Yes
22517	22:**40 X** vs. 49:**40 X**	-0.3660	-0.5100 to -0.2220	Yes
22518	22:**40 X** vs. 49:**50 X**	-0.1207	-0.2646 to 0.02329	No
22519	22:**40 X** vs. 50:**0 X**	-3.097	-3.241 to -2.953	Yes
22520	22:**40 X** vs. 50:**1 X**	-3.001	-3.145 to -2.857	Yes
22521	22:**40 X** vs. 50:**10 X**	-2.995	-3.139 to -2.851	Yes
22522	22:**40 X** vs. 50:**40 X**	-0.3737	-0.5176 to -0.2297	Yes
22523	22:**40 X** vs. 50:**50 X**	-0.1203	-0.2643 to 0.02363	No
22524	22:**50 X** vs. 23:**0 X**	-0.1123	-0.2563 to 0.03163	No
22525	22:**50 X** vs. 23:**1 X**	-0.3020	-0.4630 to -0.1410	Yes
22526	22:**50 X** vs. 23:**10 X**	-0.05167	-0.1956 to 0.09229	No
22527	22:**50 X** vs. 23:**40 X**	0.06800	-0.09295 to 0.2290	No
22528	22:**50 X** vs. 23:**50 X**	0.0340	-0.1100 to 0.1780	No
22529	22:**50 X** vs. 24:**0 X**	-0.2297	-0.3736 to -0.08571	Yes
22530	22:**50 X** vs. 24:**1 X**	-0.3860	-0.5470 to -0.2250	Yes
22531	22:**50 X** vs. 24:**10 X**	-0.2135	-0.3745 to -0.05255	Yes
22532	22:**50 X** vs. 24:**40 X**	0.05167	-0.09229 to 0.1956	No
22533	22:**50 X** vs. 24:**50 X**	0.1300	-0.01396 to 0.2740	No
22534	22:**50 X** vs. 25:**0 X**	-0.5670	-0.7280 to -0.4060	Yes
22535	22:**50 X** vs. 25:**1 X**	-0.7565	-0.9175 to -0.5955	Yes
22536	22:**50 X** vs. 25:**10 X**	-0.1180	-0.3216 to 0.08559	No
22537	22:**50 X** vs. 25:**40 X**	0.04967	-0.09429 to 0.1936	No
22538	22:**50 X** vs. 25:**50 X**	0.07533	-0.06863 to 0.2193	No
22539	22:**50 X** vs. 26:**0 X**	-0.7795	-0.9405 to -0.6185	Yes
22540	22:**50 X** vs. 26:**1 X**	-0.8925	-1.053 to -0.7315	Yes
22541	22:**50 X** vs. 26:**10 X**	-0.5790	-0.7400 to -0.4180	Yes
22542	22:**50 X** vs. 26:**40 X**	-0.02467	-0.1686 to 0.1193	No
22543	22:**50 X** vs. 26:**50 X**	0.1150	-0.02896 to 0.2590	No
22544	22:**50 X** vs. 27:**0 X**	-0.9673	-1.111 to -0.8234	Yes
22545	22:**50 X** vs. 27:**1 X**	-1.058	-1.219 to -0.8970	Yes

	2way ANOVA<br>Multiple comparisons	A<br>Data Set-A<br>Y	B<br>Data Set-B<br>Y	C<br>Data Set-C<br>Y
22546	22:50 X vs. 27:10 X	-0.8700	-1.031 to -0.7090	Yes
22547	22:50 X vs. 27:40 X	-0.02467	-0.1686 to 0.1193	No
22548	22:50 X vs. 27:50 X	0.1157	-0.02829 to 0.2596	No
22549	22:50 X vs. 28:0 X	-0.9165	-1.077 to -0.7555	Yes
22550	22:50 X vs. 28:1 X	-0.9573	-1.101 to -0.8134	Yes
22551	22:50 X vs. 28:10 X	-0.7590	-0.9200 to -0.5980	Yes
22552	22:50 X vs. 28:40 X	-0.0370	-0.1810 to 0.1070	No
22553	22:50 X vs. 28:50 X	0.06767	-0.07629 to 0.2116	No
22554	22:50 X vs. 29:0 X	-1.398	-1.559 to -1.237	Yes
22555	22:50 X vs. 29:1 X	-1.483	-1.644 to -1.322	Yes
22556	22:50 X vs. 29:10 X	-0.6975	-0.8585 to -0.5365	Yes
22557	22:50 X vs. 29:40 X	-0.1450	-0.2890 to -0.001040	Yes
22558	22:50 X vs. 29:50 X	0.06633	-0.07763 to 0.2103	No
22559	22:50 X vs. 30:0 X	-1.113	-1.273 to -0.9515	Yes
22560	22:50 X vs. 30:1 X	-1.434	-1.595 to -1.273	Yes
22561	22:50 X vs. 30:10 X	-0.7720	-0.9330 to -0.6110	Yes
22562	22:50 X vs. 30:40 X	-0.0720	-0.2160 to 0.07196	No
22563	22:50 X vs. 30:50 X	0.01400	-0.1300 to 0.1580	No
22564	22:50 X vs. 31:0 X	-1.431	-1.591 to -1.270	Yes
22565	22:50 X vs. 31:1 X	-1.421	-1.582 to -1.260	Yes
22566	22:50 X vs. 31:10 X	-0.9035	-1.064 to -0.7425	Yes
22567	22:50 X vs. 31:40 X	-0.1637	-0.3076 to -0.01971	Yes
22568	22:50 X vs. 31:50 X	0.0350	-0.1090 to 0.1790	No
22569	22:50 X vs. 32:0 X	-2.022	-2.166 to -1.878	Yes
22570	22:50 X vs. 32:1 X	-1.478	-1.639 to -1.317	Yes
22571	22:50 X vs. 32:10 X	-1.334	-1.478 to -1.190	Yes
22572	22:50 X vs. 32:40 X	-0.1600	-0.3040 to -0.01604	Yes
22573	22:50 X vs. 32:50 X	0.03533	-0.1086 to 0.1793	No
22574	22:50 X vs. 33:0 X	-2.026	-2.170 to -1.882	Yes
22575	22:50 X vs. 33:1 X	-1.840	-1.984 to -1.696	Yes
22576	22:50 X vs. 33:10 X	-1.501	-1.645 to -1.357	Yes
22577	22:50 X vs. 33:40 X	-0.1787	-0.3226 to -0.03471	Yes
22578	22:50 X vs. 33:50 X	0.02033	-0.1236 to 0.1643	No
22579	22:50 X vs. 34:0 X	-1.950	-2.094 to -1.806	Yes
22580	22:50 X vs. 34:1 X	-1.958	-2.102 to -1.814	Yes
22581	22:50 X vs. 34:10 X	-1.591	-1.735 to -1.447	Yes
22582	22:50 X vs. 34:40 X	-0.1907	-0.3346 to -0.04671	Yes
22583	22:50 X vs. 34:50 X	-0.1287	-0.2726 to 0.01529	No
22584	22:50 X vs. 35:0 X	-2.299	-2.443 to -2.155	Yes
22585	22:50 X vs. 35:1 X	-2.065	-2.209 to -1.921	Yes
22586	22:50 X vs. 35:10 X	-1.553	-1.697 to -1.409	Yes
22587	22:50 X vs. 35:40 X	-0.1997	-0.3436 to -0.05571	Yes
22588	22:50 X vs. 35:50 X	-0.1413	-0.2853 to 0.002626	No
22589	22:50 X vs. 36:0 X	-2.506	-2.666 to -2.345	Yes
22590	22:50 X vs. 36:1 X	-2.201	-2.345 to -2.057	Yes

	2way ANOVA Multiple comparisons	A Data Set-A Y	B Data Set-B Y	C Data Set-C Y
22591	22:**50 X** vs. 36:**10 X**	-1.558	-1.702 to -1.414	Yes
22592	22:**50 X** vs. 36:**40 X**	-0.2550	-0.3990 to -0.1110	Yes
22593	22:**50 X** vs. 36:**50 X**	-0.1047	-0.2486 to 0.03929	No
22594	22:**50 X** vs. 37:**0 X**	-2.889	-3.033 to -2.745	Yes
22595	22:**50 X** vs. 37:**1 X**	-2.271	-2.415 to -2.127	Yes
22596	22:**50 X** vs. 37:**10 X**	-1.560	-1.704 to -1.416	Yes
22597	22:**50 X** vs. 37:**40 X**	-0.2923	-0.4363 to -0.1484	Yes
22598	22:**50 X** vs. 37:**50 X**	-0.1133	-0.2573 to 0.03063	No
22599	22:**50 X** vs. 38:**0 X**	-2.834	-2.978 to -2.690	Yes
22600	22:**50 X** vs. 38:**1 X**	-2.390	-2.534 to -2.246	Yes
22601	22:**50 X** vs. 38:**10 X**	-1.835	-1.979 to -1.691	Yes
22602	22:**50 X** vs. 38:**40 X**	-0.2967	-0.4406 to -0.1527	Yes
22603	22:**50 X** vs. 38:**50 X**	-0.1173	-0.2613 to 0.02663	No
22604	22:**50 X** vs. 39:**0 X**	-3.065	-3.209 to -2.921	Yes
22605	22:**50 X** vs. 39:**1 X**	-2.957	-3.101 to -2.813	Yes
22606	22:**50 X** vs. 39:**10 X**	-1.980	-2.124 to -1.836	Yes
22607	22:**50 X** vs. 39:**40 X**	-0.3027	-0.4466 to -0.1587	Yes
22608	22:**50 X** vs. 39:**50 X**	-0.1483	-0.2923 to -0.004374	Yes
22609	22:**50 X** vs. 40:**0 X**	-3.106	-3.250 to -2.962	Yes
22610	22:**50 X** vs. 40:**1 X**	-3.032	-3.176 to -2.888	Yes
22611	22:**50 X** vs. 40:**10 X**	-2.237	-2.381 to -2.093	Yes
22612	22:**50 X** vs. 40:**40 X**	-0.3073	-0.4513 to -0.1634	Yes
22613	22:**50 X** vs. 40:**50 X**	-0.1547	-0.2986 to -0.01071	Yes
22614	22:**50 X** vs. 41:**0 X**	-3.147	-3.291 to -3.003	Yes
22615	22:**50 X** vs. 41:**1 X**	-3.146	-3.290 to -3.002	Yes
22616	22:**50 X** vs. 41:**10 X**	-2.290	-2.434 to -2.146	Yes
22617	22:**50 X** vs. 41:**40 X**	-0.2547	-0.3986 to -0.1107	Yes
22618	22:**50 X** vs. 41:**50 X**	-0.1673	-0.3113 to -0.02337	Yes
22619	22:**50 X** vs. 42:**0 X**	-3.289	-3.433 to -3.145	Yes
22620	22:**50 X** vs. 42:**1 X**	-3.308	-3.452 to -3.164	Yes
22621	22:**50 X** vs. 42:**10 X**	-2.291	-2.435 to -2.147	Yes
22622	22:**50 X** vs. 42:**40 X**	-0.2673	-0.4113 to -0.1234	Yes
22623	22:**50 X** vs. 42:**50 X**	-0.1707	-0.3146 to -0.02671	Yes
22624	22:**50 X** vs. 43:**0 X**	-3.379	-3.523 to -3.235	Yes
22625	22:**50 X** vs. 43:**1 X**	-3.293	-3.437 to -3.149	Yes
22626	22:**50 X** vs. 43:**10 X**	-3.009	-3.153 to -2.865	Yes
22627	22:**50 X** vs. 43:**40 X**	-0.2657	-0.4096 to -0.1217	Yes
22628	22:**50 X** vs. 43:**50 X**	-0.1710	-0.3150 to -0.02704	Yes
22629	22:**50 X** vs. 44:**0 X**	-3.403	-3.547 to -3.259	Yes
22630	22:**50 X** vs. 44:**1 X**	-3.292	-3.436 to -3.148	Yes
22631	22:**50 X** vs. 44:**10 X**	-3.072	-3.216 to -2.928	Yes
22632	22:**50 X** vs. 44:**40 X**	-0.2677	-0.4116 to -0.1237	Yes
22633	22:**50 X** vs. 44:**50 X**	-0.1033	-0.2473 to 0.04063	No
22634	22:**50 X** vs. 45:**0 X**	-3.267	-3.411 to -3.123	Yes
22635	22:**50 X** vs. 45:**1 X**	-3.069	-3.213 to -2.925	Yes

2way ANOVA<br>Multiple comparisons	A<br>Data Set-A<br>Y	B<br>Data Set-B<br>Y	C<br>Data Set-C<br>Y
22636 22:**50 X** vs. 45:**10 X**	-2.952	-3.096 to -2.808	Yes
22637 22:**50 X** vs. 45:**40 X**	-0.2690	-0.4130 to -0.1250	Yes
22638 22:**50 X** vs. 45:**50 X**	-0.1003	-0.2443 to 0.04363	No
22639 22:**50 X** vs. 46:**0 X**	-3.072	-3.216 to -2.928	Yes
22640 22:**50 X** vs. 46:**1 X**	-3.039	-3.183 to -2.895	Yes
22641 22:**50 X** vs. 46:**10 X**	-2.947	-3.091 to -2.803	Yes
22642 22:**50 X** vs. 46:**40 X**	-0.2827	-0.4266 to -0.1387	Yes
22643 22:**50 X** vs. 46:**50 X**	-0.09333	-0.2373 to 0.05063	No
22644 22:**50 X** vs. 47:**0 X**	-3.047	-3.191 to -2.903	Yes
22645 22:**50 X** vs. 47:**1 X**	-2.951	-3.095 to -2.807	Yes
22646 22:**50 X** vs. 47:**10 X**	-2.933	-3.077 to -2.789	Yes
22647 22:**50 X** vs. 47:**40 X**	-0.2927	-0.4366 to -0.1487	Yes
22648 22:**50 X** vs. 47:**50 X**	-0.05133	-0.1953 to 0.09263	No
22649 22:**50 X** vs. 48:**0 X**	-3.035	-3.195 to -2.874	Yes
22650 22:**50 X** vs. 48:**1 X**	-2.938	-3.082 to -2.794	Yes
22651 22:**50 X** vs. 48:**10 X**	-2.933	-3.077 to -2.789	Yes
22652 22:**50 X** vs. 48:**40 X**	-0.2990	-0.4430 to -0.1550	Yes
22653 22:**50 X** vs. 48:**50 X**	-0.05867	-0.2026 to 0.08529	No
22654 22:**50 X** vs. 49:**0 X**	-3.035	-3.179 to -2.891	Yes
22655 22:**50 X** vs. 49:**1 X**	-2.938	-3.082 to -2.794	Yes
22656 22:**50 X** vs. 49:**10 X**	-2.933	-3.077 to -2.789	Yes
22657 22:**50 X** vs. 49:**40 X**	-0.3033	-0.4473 to -0.1594	Yes
22658 22:**50 X** vs. 49:**50 X**	-0.0580	-0.2020 to 0.08596	No
22659 22:**50 X** vs. 50:**0 X**	-3.035	-3.179 to -2.891	Yes
22660 22:**50 X** vs. 50:**1 X**	-2.938	-3.082 to -2.794	Yes
22661 22:**50 X** vs. 50:**10 X**	-2.933	-3.077 to -2.789	Yes
22662 22:**50 X** vs. 50:**40 X**	-0.3110	-0.4550 to -0.1670	Yes
22663 22:**50 X** vs. 50:**50 X**	-0.05767	-0.2016 to 0.08629	No
22664 23:**0 X** vs. 23:**1 X**	-0.1897	-0.3506 to -0.02871	Yes
22665 23:**0 X** vs. 23:**10 X**	0.06067	-0.08329 to 0.2046	No
22666 23:**0 X** vs. 23:**40 X**	0.1803	0.01938 to 0.3413	Yes
22667 23:**0 X** vs. 23:**50 X**	0.1463	0.002374 to 0.2903	Yes
22668 23:**0 X** vs. 24:**0 X**	-0.1173	-0.2613 to 0.02663	No
22669 23:**0 X** vs. 24:**1 X**	-0.2737	-0.4346 to -0.1127	Yes
22670 23:**0 X** vs. 24:**10 X**	-0.1012	-0.2621 to 0.05979	No
22671 23:**0 X** vs. 24:**40 X**	0.1640	0.02004 to 0.3080	Yes
22672 23:**0 X** vs. 24:**50 X**	0.2423	0.09837 to 0.3863	Yes
22673 23:**0 X** vs. 25:**0 X**	-0.4547	-0.6156 to -0.2937	Yes
22674 23:**0 X** vs. 25:**1 X**	-0.6442	-0.8051 to -0.4832	Yes
22675 23:**0 X** vs. 25:**10 X**	-0.005667	-0.2093 to 0.1979	No
22676 23:**0 X** vs. 25:**40 X**	0.1620	0.01804 to 0.3060	Yes
22677 23:**0 X** vs. 25:**50 X**	0.1877	0.04371 to 0.3316	Yes
22678 23:**0 X** vs. 26:**0 X**	-0.6672	-0.8281 to -0.5062	Yes
22679 23:**0 X** vs. 26:**1 X**	-0.7802	-0.9411 to -0.6192	Yes
22680 23:**0 X** vs. 26:**10 X**	-0.4667	-0.6276 to -0.3057	Yes

	2way ANOVA<br>Multiple comparisons	A<br>Data Set-A<br>Y	B<br>Data Set-B<br>Y	C<br>Data Set-C<br>Y
22681	23:**0 X** vs. 26:**40 X**	0.08767	-0.05629 to 0.2316	No
22682	23:**0 X** vs. 26:**50 X**	0.2273	0.08337 to 0.3713	Yes
22683	23:**0 X** vs. 27:**0 X**	-0.8550	-0.9990 to -0.7110	Yes
22684	23:**0 X** vs. 27:**1 X**	-0.9457	-1.107 to -0.7847	Yes
22685	23:**0 X** vs. 27:**10 X**	-0.7577	-0.9186 to -0.5967	Yes
22686	23:**0 X** vs. 27:**40 X**	0.08767	-0.05629 to 0.2316	No
22687	23:**0 X** vs. 27:**50 X**	0.2280	0.08404 to 0.3720	Yes
22688	23:**0 X** vs. 28:**0 X**	-0.8042	-0.9651 to -0.6432	Yes
22689	23:**0 X** vs. 28:**1 X**	-0.8450	-0.9890 to -0.7010	Yes
22690	23:**0 X** vs. 28:**10 X**	-0.6467	-0.8076 to -0.4857	Yes
22691	23:**0 X** vs. 28:**40 X**	0.07533	-0.06863 to 0.2193	No
22692	23:**0 X** vs. 28:**50 X**	0.1800	0.03604 to 0.3240	Yes
22693	23:**0 X** vs. 29:**0 X**	-1.286	-1.447 to -1.125	Yes
22694	23:**0 X** vs. 29:**1 X**	-1.371	-1.532 to -1.210	Yes
22695	23:**0 X** vs. 29:**10 X**	-0.5852	-0.7461 to -0.4242	Yes
22696	23:**0 X** vs. 29:**40 X**	-0.03267	-0.1766 to 0.1113	No
22697	23:**0 X** vs. 29:**50 X**	0.1787	0.03471 to 0.3226	Yes
22698	23:**0 X** vs. 30:**0 X**	-1.000	-1.161 to -0.8392	Yes
22699	23:**0 X** vs. 30:**1 X**	-1.322	-1.483 to -1.161	Yes
22700	23:**0 X** vs. 30:**10 X**	-0.6597	-0.8206 to -0.4987	Yes
22701	23:**0 X** vs. 30:**40 X**	0.04033	-0.1036 to 0.1843	No
22702	23:**0 X** vs. 30:**50 X**	0.1263	-0.01763 to 0.2703	No
22703	23:**0 X** vs. 31:**0 X**	-1.318	-1.479 to -1.157	Yes
22704	23:**0 X** vs. 31:**1 X**	-1.309	-1.470 to -1.148	Yes
22705	23:**0 X** vs. 31:**10 X**	-0.7912	-0.9521 to -0.6302	Yes
22706	23:**0 X** vs. 31:**40 X**	-0.05133	-0.1953 to 0.09263	No
22707	23:**0 X** vs. 31:**50 X**	0.1473	0.003374 to 0.2913	Yes
22708	23:**0 X** vs. 32:**0 X**	-1.910	-2.054 to -1.766	Yes
22709	23:**0 X** vs. 32:**1 X**	-1.366	-1.527 to -1.205	Yes
22710	23:**0 X** vs. 32:**10 X**	-1.222	-1.366 to -1.078	Yes
22711	23:**0 X** vs. 32:**40 X**	-0.04767	-0.1916 to 0.09629	No
22712	23:**0 X** vs. 32:**50 X**	0.1477	0.003707 to 0.2916	Yes
22713	23:**0 X** vs. 33:**0 X**	-1.913	-2.057 to -1.769	Yes
22714	23:**0 X** vs. 33:**1 X**	-1.728	-1.872 to -1.584	Yes
22715	23:**0 X** vs. 33:**10 X**	-1.388	-1.532 to -1.244	Yes
22716	23:**0 X** vs. 33:**40 X**	-0.06633	-0.2103 to 0.07763	No
22717	23:**0 X** vs. 33:**50 X**	0.1327	-0.01129 to 0.2766	No
22718	23:**0 X** vs. 34:**0 X**	-1.838	-1.982 to -1.694	Yes
22719	23:**0 X** vs. 34:**1 X**	-1.845	-1.989 to -1.701	Yes
22720	23:**0 X** vs. 34:**10 X**	-1.479	-1.623 to -1.335	Yes
22721	23:**0 X** vs. 34:**40 X**	-0.07833	-0.2223 to 0.06563	No
22722	23:**0 X** vs. 34:**50 X**	-0.01633	-0.1603 to 0.1276	No
22723	23:**0 X** vs. 35:**0 X**	-2.187	-2.331 to -2.043	Yes
22724	23:**0 X** vs. 35:**1 X**	-1.952	-2.096 to -1.808	Yes
22725	23:**0 X** vs. 35:**10 X**	-1.440	-1.584 to -1.296	Yes

2way ANOVA<br>Multiple comparisons	A<br>Data Set-A<br>Y	B<br>Data Set-B<br>Y	C<br>Data Set-C<br>Y
22726 23:**0 X** vs. 35:**40 X**	-0.08733	-0.2313 to 0.05663	No
22727 23:**0 X** vs. 35:**50 X**	-0.02900	-0.1730 to 0.1150	No
22728 23:**0 X** vs. 36:**0 X**	-2.393	-2.554 to -2.232	Yes
22729 23:**0 X** vs. 36:**1 X**	-2.089	-2.233 to -1.945	Yes
22730 23:**0 X** vs. 36:**10 X**	-1.446	-1.590 to -1.302	Yes
22731 23:**0 X** vs. 36:**40 X**	-0.1427	-0.2866 to 0.001293	No
22732 23:**0 X** vs. 36:**50 X**	0.007667	-0.1363 to 0.1516	No
22733 23:**0 X** vs. 37:**0 X**	-2.777	-2.921 to -2.633	Yes
22734 23:**0 X** vs. 37:**1 X**	-2.159	-2.303 to -2.015	Yes
22735 23:**0 X** vs. 37:**10 X**	-1.448	-1.592 to -1.304	Yes
22736 23:**0 X** vs. 37:**40 X**	-0.1800	-0.3240 to -0.03604	Yes
22737 23:**0 X** vs. 37:**50 X**	-0.001000	-0.1450 to 0.1430	No
22738 23:**0 X** vs. 38:**0 X**	-2.722	-2.866 to -2.578	Yes
22739 23:**0 X** vs. 38:**1 X**	-2.278	-2.422 to -2.134	Yes
22740 23:**0 X** vs. 38:**10 X**	-1.723	-1.867 to -1.579	Yes
22741 23:**0 X** vs. 38:**40 X**	-0.1843	-0.3283 to -0.04037	Yes
22742 23:**0 X** vs. 38:**50 X**	-0.005000	-0.1490 to 0.1390	No
22743 23:**0 X** vs. 39:**0 X**	-2.953	-3.097 to -2.809	Yes
22744 23:**0 X** vs. 39:**1 X**	-2.844	-2.988 to -2.700	Yes
22745 23:**0 X** vs. 39:**10 X**	-1.868	-2.012 to -1.724	Yes
22746 23:**0 X** vs. 39:**40 X**	-0.1903	-0.3343 to -0.04637	Yes
22747 23:**0 X** vs. 39:**50 X**	-0.03600	-0.1800 to 0.1080	No
22748 23:**0 X** vs. 40:**0 X**	-2.994	-3.138 to -2.850	Yes
22749 23:**0 X** vs. 40:**1 X**	-2.920	-3.064 to -2.776	Yes
22750 23:**0 X** vs. 40:**10 X**	-2.124	-2.268 to -1.980	Yes
22751 23:**0 X** vs. 40:**40 X**	-0.1950	-0.3390 to -0.05104	Yes
22752 23:**0 X** vs. 40:**50 X**	-0.04233	-0.1863 to 0.1016	No
22753 23:**0 X** vs. 41:**0 X**	-3.035	-3.179 to -2.891	Yes
22754 23:**0 X** vs. 41:**1 X**	-3.034	-3.178 to -2.890	Yes
22755 23:**0 X** vs. 41:**10 X**	-2.178	-2.322 to -2.034	Yes
22756 23:**0 X** vs. 41:**40 X**	-0.1423	-0.2863 to 0.001626	No
22757 23:**0 X** vs. 41:**50 X**	-0.05500	-0.1990 to 0.08896	No
22758 23:**0 X** vs. 42:**0 X**	-3.176	-3.320 to -3.032	Yes
22759 23:**0 X** vs. 42:**1 X**	-3.196	-3.340 to -3.052	Yes
22760 23:**0 X** vs. 42:**10 X**	-2.178	-2.322 to -2.034	Yes
22761 23:**0 X** vs. 42:**40 X**	-0.1550	-0.2990 to -0.01104	Yes
22762 23:**0 X** vs. 42:**50 X**	-0.05833	-0.2023 to 0.08563	No
22763 23:**0 X** vs. 43:**0 X**	-3.266	-3.410 to -3.122	Yes
22764 23:**0 X** vs. 43:**1 X**	-3.181	-3.325 to -3.037	Yes
22765 23:**0 X** vs. 43:**10 X**	-2.896	-3.040 to -2.752	Yes
22766 23:**0 X** vs. 43:**40 X**	-0.1533	-0.2973 to -0.009374	Yes
22767 23:**0 X** vs. 43:**50 X**	-0.05867	-0.2026 to 0.08529	No
22768 23:**0 X** vs. 44:**0 X**	-3.290	-3.434 to -3.146	Yes
22769 23:**0 X** vs. 44:**1 X**	-3.180	-3.324 to -3.036	Yes
22770 23:**0 X** vs. 44:**10 X**	-2.960	-3.104 to -2.816	Yes

2way ANOVA<br>Multiple comparisons	A<br>Data Set-A<br>Y	B<br>Data Set-B<br>Y	C<br>Data Set-C<br>Y
22771 23:**0 X** vs. 44:**40 X**	-0.1553	-0.2993 to -0.01137	Yes
22772 23:**0 X** vs. 44:**50 X**	0.009000	-0.1350 to 0.1530	No
22773 23:**0 X** vs. 45:**0 X**	-3.155	-3.299 to -3.011	Yes
22774 23:**0 X** vs. 45:**1 X**	-2.957	-3.101 to -2.813	Yes
22775 23:**0 X** vs. 45:**10 X**	-2.839	-2.983 to -2.695	Yes
22776 23:**0 X** vs. 45:**40 X**	-0.1567	-0.3006 to -0.01271	Yes
22777 23:**0 X** vs. 45:**50 X**	0.0120	-0.1320 to 0.1560	No
22778 23:**0 X** vs. 46:**0 X**	-2.959	-3.103 to -2.815	Yes
22779 23:**0 X** vs. 46:**1 X**	-2.926	-3.070 to -2.782	Yes
22780 23:**0 X** vs. 46:**10 X**	-2.834	-2.978 to -2.690	Yes
22781 23:**0 X** vs. 46:**40 X**	-0.1703	-0.3143 to -0.02637	Yes
22782 23:**0 X** vs. 46:**50 X**	0.01900	-0.1250 to 0.1630	No
22783 23:**0 X** vs. 47:**0 X**	-2.935	-3.079 to -2.791	Yes
22784 23:**0 X** vs. 47:**1 X**	-2.839	-2.983 to -2.695	Yes
22785 23:**0 X** vs. 47:**10 X**	-2.820	-2.964 to -2.676	Yes
22786 23:**0 X** vs. 47:**40 X**	-0.1803	-0.3243 to -0.03637	Yes
22787 23:**0 X** vs. 47:**50 X**	0.0610	-0.08296 to 0.2050	No
22788 23:**0 X** vs. 48:**0 X**	-2.922	-3.083 to -2.761	Yes
22789 23:**0 X** vs. 48:**1 X**	-2.826	-2.970 to -2.682	Yes
22790 23:**0 X** vs. 48:**10 X**	-2.820	-2.964 to -2.676	Yes
22791 23:**0 X** vs. 48:**40 X**	-0.1867	-0.3306 to -0.04271	Yes
22792 23:**0 X** vs. 48:**50 X**	0.05367	-0.09029 to 0.1976	No
22793 23:**0 X** vs. 49:**0 X**	-2.922	-3.066 to -2.778	Yes
22794 23:**0 X** vs. 49:**1 X**	-2.826	-2.970 to -2.682	Yes
22795 23:**0 X** vs. 49:**10 X**	-2.820	-2.964 to -2.676	Yes
22796 23:**0 X** vs. 49:**40 X**	-0.1910	-0.3350 to -0.04704	Yes
22797 23:**0 X** vs. 49:**50 X**	0.05433	-0.08963 to 0.1983	No
22798 23:**0 X** vs. 50:**0 X**	-2.922	-3.066 to -2.778	Yes
22799 23:**0 X** vs. 50:**1 X**	-2.826	-2.970 to -2.682	Yes
22800 23:**0 X** vs. 50:**10 X**	-2.820	-2.964 to -2.676	Yes
22801 23:**0 X** vs. 50:**40 X**	-0.1987	-0.3426 to -0.05471	Yes
22802 23:**0 X** vs. 50:**50 X**	0.05467	-0.08929 to 0.1986	No
22803 23:**1 X** vs. 23:**10 X**	0.2503	0.08938 to 0.4113	Yes
22804 23:**1 X** vs. 23:**40 X**	0.3700	0.1937 to 0.5463	Yes
22805 23:**1 X** vs. 23:**50 X**	0.3360	0.1750 to 0.4970	Yes
22806 23:**1 X** vs. 24:**0 X**	0.07233	-0.08862 to 0.2333	No
22807 23:**1 X** vs. 24:**1 X**	-0.08400	-0.2603 to 0.09231	No
22808 23:**1 X** vs. 24:**10 X**	0.08850	-0.08781 to 0.2648	No
22809 23:**1 X** vs. 24:**40 X**	0.3537	0.1927 to 0.5146	Yes
22810 23:**1 X** vs. 24:**50 X**	0.4320	0.2710 to 0.5930	Yes
22811 23:**1 X** vs. 25:**0 X**	-0.2650	-0.4413 to -0.08869	Yes
22812 23:**1 X** vs. 25:**1 X**	-0.4545	-0.6308 to -0.2782	Yes
22813 23:**1 X** vs. 25:**10 X**	0.1840	-0.03194 to 0.3999	No
22814 23:**1 X** vs. 25:**40 X**	0.3517	0.1907 to 0.5126	Yes
22815 23:**1 X** vs. 25:**50 X**	0.3773	0.2164 to 0.5383	Yes

	2way ANOVA Multiple comparisons	A Data Set-A Y	B Data Set-B Y	C Data Set-C Y
22816	23:**1 X** vs. 26:**0 X**	-0.4775	-0.6538 to -0.3012	Yes
22817	23:**1 X** vs. 26:**1 X**	-0.5905	-0.7668 to -0.4142	Yes
22818	23:**1 X** vs. 26:**10 X**	-0.2770	-0.4533 to -0.1007	Yes
22819	23:**1 X** vs. 26:**40 X**	0.2773	0.1164 to 0.4383	Yes
22820	23:**1 X** vs. 26:**50 X**	0.4170	0.2560 to 0.5780	Yes
22821	23:**1 X** vs. 27:**0 X**	-0.6653	-0.8263 to -0.5044	Yes
22822	23:**1 X** vs. 27:**1 X**	-0.7560	-0.9323 to -0.5797	Yes
22823	23:**1 X** vs. 27:**10 X**	-0.5680	-0.7443 to -0.3917	Yes
22824	23:**1 X** vs. 27:**40 X**	0.2773	0.1164 to 0.4383	Yes
22825	23:**1 X** vs. 27:**50 X**	0.4177	0.2567 to 0.5786	Yes
22826	23:**1 X** vs. 28:**0 X**	-0.6145	-0.7908 to -0.4382	Yes
22827	23:**1 X** vs. 28:**1 X**	-0.6553	-0.8163 to -0.4944	Yes
22828	23:**1 X** vs. 28:**10 X**	-0.4570	-0.6333 to -0.2807	Yes
22829	23:**1 X** vs. 28:**40 X**	0.2650	0.1040 to 0.4260	Yes
22830	23:**1 X** vs. 28:**50 X**	0.3697	0.2087 to 0.5306	Yes
22831	23:**1 X** vs. 29:**0 X**	-1.096	-1.272 to -0.9197	Yes
22832	23:**1 X** vs. 29:**1 X**	-1.181	-1.357 to -1.005	Yes
22833	23:**1 X** vs. 29:**10 X**	-0.3955	-0.5718 to -0.2192	Yes
22834	23:**1 X** vs. 29:**40 X**	0.1570	-0.003952 to 0.3180	No
22835	23:**1 X** vs. 29:**50 X**	0.3683	0.2074 to 0.5293	Yes
22836	23:**1 X** vs. 30:**0 X**	-0.8105	-0.9868 to -0.6342	Yes
22837	23:**1 X** vs. 30:**1 X**	-1.132	-1.308 to -0.9557	Yes
22838	23:**1 X** vs. 30:**10 X**	-0.4700	-0.6463 to -0.2937	Yes
22839	23:**1 X** vs. 30:**40 X**	0.2300	0.06905 to 0.3910	Yes
22840	23:**1 X** vs. 30:**50 X**	0.3160	0.1550 to 0.4770	Yes
22841	23:**1 X** vs. 31:**0 X**	-1.129	-1.305 to -0.9522	Yes
22842	23:**1 X** vs. 31:**1 X**	-1.119	-1.295 to -0.9427	Yes
22843	23:**1 X** vs. 31:**10 X**	-0.6015	-0.7778 to -0.4252	Yes
22844	23:**1 X** vs. 31:**40 X**	0.1383	-0.02262 to 0.2993	No
22845	23:**1 X** vs. 31:**50 X**	0.3370	0.1760 to 0.4980	Yes
22846	23:**1 X** vs. 32:**0 X**	-1.720	-1.881 to -1.559	Yes
22847	23:**1 X** vs. 32:**1 X**	-1.176	-1.352 to -0.9997	Yes
22848	23:**1 X** vs. 32:**10 X**	-1.032	-1.193 to -0.8710	Yes
22849	23:**1 X** vs. 32:**40 X**	0.1420	-0.01895 to 0.3030	No
22850	23:**1 X** vs. 32:**50 X**	0.3373	0.1764 to 0.4983	Yes
22851	23:**1 X** vs. 33:**0 X**	-1.724	-1.885 to -1.563	Yes
22852	23:**1 X** vs. 33:**1 X**	-1.538	-1.699 to -1.377	Yes
22853	23:**1 X** vs. 33:**10 X**	-1.199	-1.360 to -1.038	Yes
22854	23:**1 X** vs. 33:**40 X**	0.1233	-0.03762 to 0.2843	No
22855	23:**1 X** vs. 33:**50 X**	0.3223	0.1614 to 0.4833	Yes
22856	23:**1 X** vs. 34:**0 X**	-1.648	-1.809 to -1.487	Yes
22857	23:**1 X** vs. 34:**1 X**	-1.656	-1.817 to -1.495	Yes
22858	23:**1 X** vs. 34:**10 X**	-1.289	-1.450 to -1.128	Yes
22859	23:**1 X** vs. 34:**40 X**	0.1113	-0.04962 to 0.2723	No
22860	23:**1 X** vs. 34:**50 X**	0.1733	0.01238 to 0.3343	Yes

	2way ANOVA<br>Multiple comparisons	A<br>Data Set-A<br>Y	B<br>Data Set-B<br>Y	C<br>Data Set-C<br>Y
22861	23:1 X vs. 35:0 X	-1.997	-2.158 to -1.836	Yes
22862	23:1 X vs. 35:1 X	-1.763	-1.924 to -1.602	Yes
22863	23:1 X vs. 35:10 X	-1.251	-1.412 to -1.090	Yes
22864	23:1 X vs. 35:40 X	0.1023	-0.05862 to 0.2633	No
22865	23:1 X vs. 35:50 X	0.1607	-0.0002853 to 0.3216	No
22866	23:1 X vs. 36:0 X	-2.204	-2.380 to -2.027	Yes
22867	23:1 X vs. 36:1 X	-1.899	-2.060 to -1.738	Yes
22868	23:1 X vs. 36:10 X	-1.256	-1.417 to -1.095	Yes
22869	23:1 X vs. 36:40 X	0.0470	-0.1140 to 0.2080	No
22870	23:1 X vs. 36:50 X	0.1973	0.03638 to 0.3583	Yes
22871	23:1 X vs. 37:0 X	-2.587	-2.748 to -2.426	Yes
22872	23:1 X vs. 37:1 X	-1.969	-2.130 to -1.808	Yes
22873	23:1 X vs. 37:10 X	-1.258	-1.419 to -1.097	Yes
22874	23:1 X vs. 37:40 X	0.009667	-0.1513 to 0.1706	No
22875	23:1 X vs. 37:50 X	0.1887	0.02771 to 0.3496	Yes
22876	23:1 X vs. 38:0 X	-2.532	-2.693 to -2.371	Yes
22877	23:1 X vs. 38:1 X	-2.088	-2.249 to -1.927	Yes
22878	23:1 X vs. 38:10 X	-1.533	-1.694 to -1.372	Yes
22879	23:1 X vs. 38:40 X	0.005333	-0.1556 to 0.1663	No
22880	23:1 X vs. 38:50 X	0.1847	0.02371 to 0.3456	Yes
22881	23:1 X vs. 39:0 X	-2.763	-2.924 to -2.602	Yes
22882	23:1 X vs. 39:1 X	-2.655	-2.816 to -2.494	Yes
22883	23:1 X vs. 39:10 X	-1.678	-1.839 to -1.517	Yes
22884	23:1 X vs. 39:40 X	-0.0006667	-0.1616 to 0.1603	No
22885	23:1 X vs. 39:50 X	0.1537	-0.007285 to 0.3146	No
22886	23:1 X vs. 40:0 X	-2.804	-2.965 to -2.643	Yes
22887	23:1 X vs. 40:1 X	-2.730	-2.891 to -2.569	Yes
22888	23:1 X vs. 40:10 X	-1.935	-2.096 to -1.774	Yes
22889	23:1 X vs. 40:40 X	-0.005333	-0.1663 to 0.1556	No
22890	23:1 X vs. 40:50 X	0.1473	-0.01362 to 0.3083	No
22891	23:1 X vs. 41:0 X	-2.845	-3.006 to -2.684	Yes
22892	23:1 X vs. 41:1 X	-2.844	-3.005 to -2.683	Yes
22893	23:1 X vs. 41:10 X	-1.988	-2.149 to -1.827	Yes
22894	23:1 X vs. 41:40 X	0.04733	-0.1136 to 0.2083	No
22895	23:1 X vs. 41:50 X	0.1347	-0.02629 to 0.2956	No
22896	23:1 X vs. 42:0 X	-2.987	-3.148 to -2.826	Yes
22897	23:1 X vs. 42:1 X	-3.006	-3.167 to -2.845	Yes
22898	23:1 X vs. 42:10 X	-1.989	-2.150 to -1.828	Yes
22899	23:1 X vs. 42:40 X	0.03467	-0.1263 to 0.1956	No
22900	23:1 X vs. 42:50 X	0.1313	-0.02962 to 0.2923	No
22901	23:1 X vs. 43:0 X	-3.077	-3.238 to -2.916	Yes
22902	23:1 X vs. 43:1 X	-2.991	-3.152 to -2.830	Yes
22903	23:1 X vs. 43:10 X	-2.707	-2.868 to -2.546	Yes
22904	23:1 X vs. 43:40 X	0.03633	-0.1246 to 0.1973	No
22905	23:1 X vs. 43:50 X	0.1310	-0.02995 to 0.2920	No

	2way ANOVA<br>Multiple comparisons	A<br>Data Set-A<br>Y	B<br>Data Set-B<br>Y	C<br>Data Set-C<br>Y
22906	23:**1 X** vs. 44:**0 X**	-3.101	-3.262 to -2.940	Yes
22907	23:**1 X** vs. 44:**1 X**	-2.990	-3.151 to -2.829	Yes
22908	23:**1 X** vs. 44:**10 X**	-2.770	-2.931 to -2.609	Yes
22909	23:**1 X** vs. 44:**40 X**	0.03433	-0.1266 to 0.1953	No
22910	23:**1 X** vs. 44:**50 X**	0.1987	0.03771 to 0.3596	Yes
22911	23:**1 X** vs. 45:**0 X**	-2.965	-3.126 to -2.804	Yes
22912	23:**1 X** vs. 45:**1 X**	-2.767	-2.928 to -2.606	Yes
22913	23:**1 X** vs. 45:**10 X**	-2.650	-2.811 to -2.489	Yes
22914	23:**1 X** vs. 45:**40 X**	0.0330	-0.1280 to 0.1940	No
22915	23:**1 X** vs. 45:**50 X**	0.2017	0.04071 to 0.3626	Yes
22916	23:**1 X** vs. 46:**0 X**	-2.770	-2.931 to -2.609	Yes
22917	23:**1 X** vs. 46:**1 X**	-2.737	-2.898 to -2.576	Yes
22918	23:**1 X** vs. 46:**10 X**	-2.645	-2.806 to -2.484	Yes
22919	23:**1 X** vs. 46:**40 X**	0.01933	-0.1416 to 0.1803	No
22920	23:**1 X** vs. 46:**50 X**	0.2087	0.04771 to 0.3696	Yes
22921	23:**1 X** vs. 47:**0 X**	-2.745	-2.906 to -2.584	Yes
22922	23:**1 X** vs. 47:**1 X**	-2.649	-2.810 to -2.488	Yes
22923	23:**1 X** vs. 47:**10 X**	-2.631	-2.792 to -2.470	Yes
22924	23:**1 X** vs. 47:**40 X**	0.009333	-0.1516 to 0.1703	No
22925	23:**1 X** vs. 47:**50 X**	0.2507	0.08971 to 0.4116	Yes
22926	23:**1 X** vs. 48:**0 X**	-2.733	-2.909 to -2.556	Yes
22927	23:**1 X** vs. 48:**1 X**	-2.636	-2.797 to -2.475	Yes
22928	23:**1 X** vs. 48:**10 X**	-2.631	-2.792 to -2.470	Yes
22929	23:**1 X** vs. 48:**40 X**	0.003000	-0.1580 to 0.1640	No
22930	23:**1 X** vs. 48:**50 X**	0.2433	0.08238 to 0.4043	Yes
22931	23:**1 X** vs. 49:**0 X**	-2.733	-2.894 to -2.572	Yes
22932	23:**1 X** vs. 49:**1 X**	-2.636	-2.797 to -2.475	Yes
22933	23:**1 X** vs. 49:**10 X**	-2.631	-2.792 to -2.470	Yes
22934	23:**1 X** vs. 49:**40 X**	-0.001333	-0.1623 to 0.1596	No
22935	23:**1 X** vs. 49:**50 X**	0.2440	0.08305 to 0.4050	Yes
22936	23:**1 X** vs. 50:**0 X**	-2.733	-2.894 to -2.572	Yes
22937	23:**1 X** vs. 50:**1 X**	-2.636	-2.797 to -2.475	Yes
22938	23:**1 X** vs. 50:**10 X**	-2.631	-2.792 to -2.470	Yes
22939	23:**1 X** vs. 50:**40 X**	-0.009000	-0.1700 to 0.1520	No
22940	23:**1 X** vs. 50:**50 X**	0.2443	0.08338 to 0.4053	Yes
22941	23:**10 X** vs. 23:**40 X**	0.1197	-0.04129 to 0.2806	No
22942	23:**10 X** vs. 23:**50 X**	0.08567	-0.05829 to 0.2296	No
22943	23:**10 X** vs. 24:**0 X**	-0.1780	-0.3220 to -0.03404	Yes
22944	23:**10 X** vs. 24:**1 X**	-0.3343	-0.4953 to -0.1734	Yes
22945	23:**10 X** vs. 24:**10 X**	-0.1618	-0.3228 to -0.0008814	Yes
22946	23:**10 X** vs. 24:**40 X**	0.1033	-0.04063 to 0.2473	No
22947	23:**10 X** vs. 24:**50 X**	0.1817	0.03771 to 0.3256	Yes
22948	23:**10 X** vs. 25:**0 X**	-0.5153	-0.6763 to -0.3544	Yes
22949	23:**10 X** vs. 25:**1 X**	-0.7048	-0.8658 to -0.5439	Yes
22950	23:**10 X** vs. 25:**10 X**	-0.06633	-0.2699 to 0.1373	No

2way ANOVA<br>Multiple comparisons		A<br>Data Set-A<br>Y	B<br>Data Set-B<br>Y	C<br>Data Set-C<br>Y
22951	23:10 X vs. 25:40 X	0.1013	-0.04263 to 0.2453	No
22952	23:10 X vs. 25:50 X	0.1270	-0.01696 to 0.2710	No
22953	23:10 X vs. 26:0 X	-0.7278	-0.8888 to -0.5669	Yes
22954	23:10 X vs. 26:1 X	-0.8408	-1.002 to -0.6799	Yes
22955	23:10 X vs. 26:10 X	-0.5273	-0.6883 to -0.3664	Yes
22956	23:10 X vs. 26:40 X	0.0270	-0.1170 to 0.1710	No
22957	23:10 X vs. 26:50 X	0.1667	0.02271 to 0.3106	Yes
22958	23:10 X vs. 27:0 X	-0.9157	-1.060 to -0.7717	Yes
22959	23:10 X vs. 27:1 X	-1.006	-1.167 to -0.8454	Yes
22960	23:10 X vs. 27:10 X	-0.8183	-0.9793 to -0.6574	Yes
22961	23:10 X vs. 27:40 X	0.0270	-0.1170 to 0.1710	No
22962	23:10 X vs. 27:50 X	0.1673	0.02337 to 0.3113	Yes
22963	23:10 X vs. 28:0 X	-0.8648	-1.026 to -0.7039	Yes
22964	23:10 X vs. 28:1 X	-0.9057	-1.050 to -0.7617	Yes
22965	23:10 X vs. 28:10 X	-0.7073	-0.8683 to -0.5464	Yes
22966	23:10 X vs. 28:40 X	0.01467	-0.1293 to 0.1586	No
22967	23:10 X vs. 28:50 X	0.1193	-0.02463 to 0.2633	No
22968	23:10 X vs. 29:0 X	-1.346	-1.507 to -1.185	Yes
22969	23:10 X vs. 29:1 X	-1.431	-1.592 to -1.270	Yes
22970	23:10 X vs. 29:10 X	-0.6458	-0.8068 to -0.4849	Yes
22971	23:10 X vs. 29:40 X	-0.09333	-0.2373 to 0.05063	No
22972	23:10 X vs. 29:50 X	0.1180	-0.02596 to 0.2620	No
22973	23:10 X vs. 30:0 X	-1.061	-1.222 to -0.8999	Yes
22974	23:10 X vs. 30:1 X	-1.382	-1.543 to -1.221	Yes
22975	23:10 X vs. 30:10 X	-0.7203	-0.8813 to -0.5594	Yes
22976	23:10 X vs. 30:40 X	-0.02033	-0.1643 to 0.1236	No
22977	23:10 X vs. 30:50 X	0.06567	-0.07829 to 0.2096	No
22978	23:10 X vs. 31:0 X	-1.379	-1.540 to -1.218	Yes
22979	23:10 X vs. 31:1 X	-1.369	-1.530 to -1.208	Yes
22980	23:10 X vs. 31:10 X	-0.8518	-1.013 to -0.6909	Yes
22981	23:10 X vs. 31:40 X	-0.1120	-0.2560 to 0.03196	No
22982	23:10 X vs. 31:50 X	0.08667	-0.05729 to 0.2306	No
22983	23:10 X vs. 32:0 X	-1.970	-2.114 to -1.826	Yes
22984	23:10 X vs. 32:1 X	-1.426	-1.587 to -1.265	Yes
22985	23:10 X vs. 32:10 X	-1.282	-1.426 to -1.138	Yes
22986	23:10 X vs. 32:40 X	-0.1083	-0.2523 to 0.03563	No
22987	23:10 X vs. 32:50 X	0.08700	-0.05696 to 0.2310	No
22988	23:10 X vs. 33:0 X	-1.974	-2.118 to -1.830	Yes
22989	23:10 X vs. 33:1 X	-1.789	-1.933 to -1.645	Yes
22990	23:10 X vs. 33:10 X	-1.449	-1.593 to -1.305	Yes
22991	23:10 X vs. 33:40 X	-0.1270	-0.2710 to 0.01696	No
22992	23:10 X vs. 33:50 X	0.07200	-0.07196 to 0.2160	No
22993	23:10 X vs. 34:0 X	-1.899	-2.043 to -1.755	Yes
22994	23:10 X vs. 34:1 X	-1.906	-2.050 to -1.762	Yes
22995	23:10 X vs. 34:10 X	-1.539	-1.683 to -1.395	Yes

	2way ANOVA Multiple comparisons	A Data Set-A Y	B Data Set-B Y	C Data Set-C Y
22996	23:10 X vs. 34:40 X	-0.1390	-0.2830 to 0.004960	No
22997	23:10 X vs. 34:50 X	-0.0770	-0.2210 to 0.06696	No
22998	23:10 X vs. 35:0 X	-2.247	-2.391 to -2.103	Yes
22999	23:10 X vs. 35:1 X	-2.013	-2.157 to -1.869	Yes
23000	23:10 X vs. 35:10 X	-1.501	-1.645 to -1.357	Yes
23001	23:10 X vs. 35:40 X	-0.1480	-0.2920 to -0.004040	Yes
23002	23:10 X vs. 35:50 X	-0.08967	-0.2336 to 0.05429	No
23003	23:10 X vs. 36:0 X	-2.454	-2.615 to -2.293	Yes
23004	23:10 X vs. 36:1 X	-2.150	-2.294 to -2.006	Yes
23005	23:10 X vs. 36:10 X	-1.507	-1.651 to -1.363	Yes
23006	23:10 X vs. 36:40 X	-0.2033	-0.3473 to -0.05937	Yes
23007	23:10 X vs. 36:50 X	-0.05300	-0.1970 to 0.09096	No
23008	23:10 X vs. 37:0 X	-2.838	-2.982 to -2.694	Yes
23009	23:10 X vs. 37:1 X	-2.220	-2.364 to -2.076	Yes
23010	23:10 X vs. 37:10 X	-1.508	-1.652 to -1.364	Yes
23011	23:10 X vs. 37:40 X	-0.2407	-0.3846 to -0.09671	Yes
23012	23:10 X vs. 37:50 X	-0.06167	-0.2056 to 0.08229	No
23013	23:10 X vs. 38:0 X	-2.783	-2.927 to -2.639	Yes
23014	23:10 X vs. 38:1 X	-2.338	-2.482 to -2.194	Yes
23015	23:10 X vs. 38:10 X	-1.784	-1.928 to -1.640	Yes
23016	23:10 X vs. 38:40 X	-0.2450	-0.3890 to -0.1010	Yes
23017	23:10 X vs. 38:50 X	-0.06567	-0.2096 to 0.07829	No
23018	23:10 X vs. 39:0 X	-3.013	-3.157 to -2.869	Yes
23019	23:10 X vs. 39:1 X	-2.905	-3.049 to -2.761	Yes
23020	23:10 X vs. 39:10 X	-1.929	-2.073 to -1.785	Yes
23021	23:10 X vs. 39:40 X	-0.2510	-0.3950 to -0.1070	Yes
23022	23:10 X vs. 39:50 X	-0.09667	-0.2406 to 0.04729	No
23023	23:10 X vs. 40:0 X	-3.054	-3.198 to -2.910	Yes
23024	23:10 X vs. 40:1 X	-2.980	-3.124 to -2.836	Yes
23025	23:10 X vs. 40:10 X	-2.185	-2.329 to -2.041	Yes
23026	23:10 X vs. 40:40 X	-0.2557	-0.3996 to -0.1117	Yes
23027	23:10 X vs. 40:50 X	-0.1030	-0.2470 to 0.04096	No
23028	23:10 X vs. 41:0 X	-3.095	-3.239 to -2.951	Yes
23029	23:10 X vs. 41:1 X	-3.094	-3.238 to -2.950	Yes
23030	23:10 X vs. 41:10 X	-2.239	-2.383 to -2.095	Yes
23031	23:10 X vs. 41:40 X	-0.2030	-0.3470 to -0.05904	Yes
23032	23:10 X vs. 41:50 X	-0.1157	-0.2596 to 0.02829	No
23033	23:10 X vs. 42:0 X	-3.237	-3.381 to -3.093	Yes
23034	23:10 X vs. 42:1 X	-3.257	-3.401 to -3.113	Yes
23035	23:10 X vs. 42:10 X	-2.239	-2.383 to -2.095	Yes
23036	23:10 X vs. 42:40 X	-0.2157	-0.3596 to -0.07171	Yes
23037	23:10 X vs. 42:50 X	-0.1190	-0.2630 to 0.02496	No
23038	23:10 X vs. 43:0 X	-3.327	-3.471 to -3.183	Yes
23039	23:10 X vs. 43:1 X	-3.241	-3.385 to -3.097	Yes
23040	23:10 X vs. 43:10 X	-2.957	-3.101 to -2.813	Yes

	2way ANOVA<br>Multiple comparisons	A<br>Data Set-A<br>Y	B<br>Data Set-B<br>Y	C<br>Data Set-C<br>Y
23041	23:**10 X** vs. 43:**40 X**	-0.2140	-0.3580 to -0.07004	Yes
23042	23:**10 X** vs. 43:**50 X**	-0.1193	-0.2633 to 0.02463	No
23043	23:**10 X** vs. 44:**0 X**	-3.351	-3.495 to -3.207	Yes
23044	23:**10 X** vs. 44:**1 X**	-3.241	-3.385 to -3.097	Yes
23045	23:**10 X** vs. 44:**10 X**	-3.020	-3.164 to -2.876	Yes
23046	23:**10 X** vs. 44:**40 X**	-0.2160	-0.3600 to -0.07204	Yes
23047	23:**10 X** vs. 44:**50 X**	-0.05167	-0.1956 to 0.09229	No
23048	23:**10 X** vs. 45:**0 X**	-3.216	-3.360 to -3.072	Yes
23049	23:**10 X** vs. 45:**1 X**	-3.018	-3.162 to -2.874	Yes
23050	23:**10 X** vs. 45:**10 X**	-2.900	-3.044 to -2.756	Yes
23051	23:**10 X** vs. 45:**40 X**	-0.2173	-0.3613 to -0.07337	Yes
23052	23:**10 X** vs. 45:**50 X**	-0.04867	-0.1926 to 0.09529	No
23053	23:**10 X** vs. 46:**0 X**	-3.020	-3.164 to -2.876	Yes
23054	23:**10 X** vs. 46:**1 X**	-2.987	-3.131 to -2.843	Yes
23055	23:**10 X** vs. 46:**10 X**	-2.895	-3.039 to -2.751	Yes
23056	23:**10 X** vs. 46:**40 X**	-0.2310	-0.3750 to -0.08704	Yes
23057	23:**10 X** vs. 46:**50 X**	-0.04167	-0.1856 to 0.1023	No
23058	23:**10 X** vs. 47:**0 X**	-2.996	-3.140 to -2.852	Yes
23059	23:**10 X** vs. 47:**1 X**	-2.900	-3.044 to -2.756	Yes
23060	23:**10 X** vs. 47:**10 X**	-2.881	-3.025 to -2.737	Yes
23061	23:**10 X** vs. 47:**40 X**	-0.2410	-0.3850 to -0.09704	Yes
23062	23:**10 X** vs. 47:**50 X**	0.0003333	-0.1436 to 0.1443	No
23063	23:**10 X** vs. 48:**0 X**	-2.983	-3.144 to -2.822	Yes
23064	23:**10 X** vs. 48:**1 X**	-2.887	-3.031 to -2.743	Yes
23065	23:**10 X** vs. 48:**10 X**	-2.881	-3.025 to -2.737	Yes
23066	23:**10 X** vs. 48:**40 X**	-0.2473	-0.3913 to -0.1034	Yes
23067	23:**10 X** vs. 48:**50 X**	-0.007000	-0.1510 to 0.1370	No
23068	23:**10 X** vs. 49:**0 X**	-2.983	-3.127 to -2.839	Yes
23069	23:**10 X** vs. 49:**1 X**	-2.887	-3.031 to -2.743	Yes
23070	23:**10 X** vs. 49:**10 X**	-2.881	-3.025 to -2.737	Yes
23071	23:**10 X** vs. 49:**40 X**	-0.2517	-0.3956 to -0.1077	Yes
23072	23:**10 X** vs. 49:**50 X**	-0.006333	-0.1503 to 0.1376	No
23073	23:**10 X** vs. 50:**0 X**	-2.983	-3.127 to -2.839	Yes
23074	23:**10 X** vs. 50:**1 X**	-2.887	-3.031 to -2.743	Yes
23075	23:**10 X** vs. 50:**10 X**	-2.881	-3.025 to -2.737	Yes
23076	23:**10 X** vs. 50:**40 X**	-0.2593	-0.4033 to -0.1154	Yes
23077	23:**10 X** vs. 50:**50 X**	-0.006000	-0.1500 to 0.1380	No
23078	23:**40 X** vs. 23:**50 X**	-0.03400	-0.1950 to 0.1270	No
23079	23:**40 X** vs. 24:**0 X**	-0.2977	-0.4586 to -0.1367	Yes
23080	23:**40 X** vs. 24:**1 X**	-0.4540	-0.6303 to -0.2777	Yes
23081	23:**40 X** vs. 24:**10 X**	-0.2815	-0.4578 to -0.1052	Yes
23082	23:**40 X** vs. 24:**40 X**	-0.01633	-0.1773 to 0.1446	No
23083	23:**40 X** vs. 24:**50 X**	0.06200	-0.09895 to 0.2230	No
23084	23:**40 X** vs. 25:**0 X**	-0.6350	-0.8113 to -0.4587	Yes
23085	23:**40 X** vs. 25:**1 X**	-0.8245	-1.001 to -0.6482	Yes

	2way ANOVA<br>Multiple comparisons	A<br>Data Set-A<br>Y	B<br>Data Set-B<br>Y	C<br>Data Set-C<br>Y
23086	23:40 X vs. 25:10 X	-0.1860	-0.4019 to 0.02994	No
23087	23:40 X vs. 25:40 X	-0.01833	-0.1793 to 0.1426	No
23088	23:40 X vs. 25:50 X	0.007333	-0.1536 to 0.1683	No
23089	23:40 X vs. 26:0 X	-0.8475	-1.024 to -0.6712	Yes
23090	23:40 X vs. 26:1 X	-0.9605	-1.137 to -0.7842	Yes
23091	23:40 X vs. 26:10 X	-0.6470	-0.8233 to -0.4707	Yes
23092	23:40 X vs. 26:40 X	-0.09267	-0.2536 to 0.06829	No
23093	23:40 X vs. 26:50 X	0.04700	-0.1140 to 0.2080	No
23094	23:40 X vs. 27:0 X	-1.035	-1.196 to -0.8744	Yes
23095	23:40 X vs. 27:1 X	-1.126	-1.302 to -0.9497	Yes
23096	23:40 X vs. 27:10 X	-0.9380	-1.114 to -0.7617	Yes
23097	23:40 X vs. 27:40 X	-0.09267	-0.2536 to 0.06829	No
23098	23:40 X vs. 27:50 X	0.04767	-0.1133 to 0.2086	No
23099	23:40 X vs. 28:0 X	-0.9845	-1.161 to -0.8082	Yes
23100	23:40 X vs. 28:1 X	-1.025	-1.186 to -0.8644	Yes
23101	23:40 X vs. 28:10 X	-0.8270	-1.003 to -0.6507	Yes
23102	23:40 X vs. 28:40 X	-0.1050	-0.2660 to 0.05595	No
23103	23:40 X vs. 28:50 X	-0.0003333	-0.1613 to 0.1606	No
23104	23:40 X vs. 29:0 X	-1.466	-1.642 to -1.290	Yes
23105	23:40 X vs. 29:1 X	-1.551	-1.727 to -1.375	Yes
23106	23:40 X vs. 29:10 X	-0.7655	-0.9418 to -0.5892	Yes
23107	23:40 X vs. 29:40 X	-0.2130	-0.3740 to -0.05205	Yes
23108	23:40 X vs. 29:50 X	-0.001667	-0.1626 to 0.1593	No
23109	23:40 X vs. 30:0 X	-1.181	-1.357 to -1.004	Yes
23110	23:40 X vs. 30:1 X	-1.502	-1.678 to -1.326	Yes
23111	23:40 X vs. 30:10 X	-0.8400	-1.016 to -0.6637	Yes
23112	23:40 X vs. 30:40 X	-0.1400	-0.3010 to 0.02095	No
23113	23:40 X vs. 30:50 X	-0.05400	-0.2150 to 0.1070	No
23114	23:40 X vs. 31:0 X	-1.499	-1.675 to -1.322	Yes
23115	23:40 X vs. 31:1 X	-1.489	-1.665 to -1.313	Yes
23116	23:40 X vs. 31:10 X	-0.9715	-1.148 to -0.7952	Yes
23117	23:40 X vs. 31:40 X	-0.2317	-0.3926 to -0.07071	Yes
23118	23:40 X vs. 31:50 X	-0.03300	-0.1940 to 0.1280	No
23119	23:40 X vs. 32:0 X	-2.090	-2.251 to -1.929	Yes
23120	23:40 X vs. 32:1 X	-1.546	-1.722 to -1.370	Yes
23121	23:40 X vs. 32:10 X	-1.402	-1.563 to -1.241	Yes
23122	23:40 X vs. 32:40 X	-0.2280	-0.3890 to -0.06705	Yes
23123	23:40 X vs. 32:50 X	-0.03267	-0.1936 to 0.1283	No
23124	23:40 X vs. 33:0 X	-2.094	-2.255 to -1.933	Yes
23125	23:40 X vs. 33:1 X	-1.908	-2.069 to -1.747	Yes
23126	23:40 X vs. 33:10 X	-1.569	-1.730 to -1.408	Yes
23127	23:40 X vs. 33:40 X	-0.2467	-0.4076 to -0.08571	Yes
23128	23:40 X vs. 33:50 X	-0.04767	-0.2086 to 0.1133	No
23129	23:40 X vs. 34:0 X	-2.018	-2.179 to -1.857	Yes
23130	23:40 X vs. 34:1 X	-2.026	-2.187 to -1.865	Yes

2way ANOVA<br>Multiple comparisons	A<br>Data Set-A<br>Y	B<br>Data Set-B<br>Y	C<br>Data Set-C<br>Y
23131 23:40 X vs. 34:10 X	-1.659	-1.820 to -1.498	Yes
23132 23:40 X vs. 34:40 X	-0.2587	-0.4196 to -0.09771	Yes
23133 23:40 X vs. 34:50 X	-0.1967	-0.3576 to -0.03571	Yes
23134 23:40 X vs. 35:0 X	-2.367	-2.528 to -2.206	Yes
23135 23:40 X vs. 35:1 X	-2.133	-2.294 to -1.972	Yes
23136 23:40 X vs. 35:10 X	-1.621	-1.782 to -1.460	Yes
23137 23:40 X vs. 35:40 X	-0.2677	-0.4286 to -0.1067	Yes
23138 23:40 X vs. 35:50 X	-0.2093	-0.3703 to -0.04838	Yes
23139 23:40 X vs. 36:0 X	-2.574	-2.750 to -2.397	Yes
23140 23:40 X vs. 36:1 X	-2.269	-2.430 to -2.108	Yes
23141 23:40 X vs. 36:10 X	-1.626	-1.787 to -1.465	Yes
23142 23:40 X vs. 36:40 X	-0.3230	-0.4840 to -0.1620	Yes
23143 23:40 X vs. 36:50 X	-0.1727	-0.3336 to -0.01171	Yes
23144 23:40 X vs. 37:0 X	-2.957	-3.118 to -2.796	Yes
23145 23:40 X vs. 37:1 X	-2.339	-2.500 to -2.178	Yes
23146 23:40 X vs. 37:10 X	-1.628	-1.789 to -1.467	Yes
23147 23:40 X vs. 37:40 X	-0.3603	-0.5213 to -0.1994	Yes
23148 23:40 X vs. 37:50 X	-0.1813	-0.3423 to -0.02038	Yes
23149 23:40 X vs. 38:0 X	-2.902	-3.063 to -2.741	Yes
23150 23:40 X vs. 38:1 X	-2.458	-2.619 to -2.297	Yes
23151 23:40 X vs. 38:10 X	-1.903	-2.064 to -1.742	Yes
23152 23:40 X vs. 38:40 X	-0.3647	-0.5256 to -0.2037	Yes
23153 23:40 X vs. 38:50 X	-0.1853	-0.3463 to -0.02438	Yes
23154 23:40 X vs. 39:0 X	-3.133	-3.294 to -2.972	Yes
23155 23:40 X vs. 39:1 X	-3.025	-3.186 to -2.864	Yes
23156 23:40 X vs. 39:10 X	-2.048	-2.209 to -1.887	Yes
23157 23:40 X vs. 39:40 X	-0.3707	-0.5316 to -0.2097	Yes
23158 23:40 X vs. 39:50 X	-0.2163	-0.3773 to -0.05538	Yes
23159 23:40 X vs. 40:0 X	-3.174	-3.335 to -3.013	Yes
23160 23:40 X vs. 40:1 X	-3.100	-3.261 to -2.939	Yes
23161 23:40 X vs. 40:10 X	-2.305	-2.466 to -2.144	Yes
23162 23:40 X vs. 40:40 X	-0.3753	-0.5363 to -0.2144	Yes
23163 23:40 X vs. 40:50 X	-0.2227	-0.3836 to -0.06171	Yes
23164 23:40 X vs. 41:0 X	-3.215	-3.376 to -3.054	Yes
23165 23:40 X vs. 41:1 X	-3.214	-3.375 to -3.053	Yes
23166 23:40 X vs. 41:10 X	-2.358	-2.519 to -2.197	Yes
23167 23:40 X vs. 41:40 X	-0.3227	-0.4836 to -0.1617	Yes
23168 23:40 X vs. 41:50 X	-0.2353	-0.3963 to -0.07438	Yes
23169 23:40 X vs. 42:0 X	-3.357	-3.518 to -3.196	Yes
23170 23:40 X vs. 42:1 X	-3.376	-3.537 to -3.215	Yes
23171 23:40 X vs. 42:10 X	-2.359	-2.520 to -2.198	Yes
23172 23:40 X vs. 42:40 X	-0.3353	-0.4963 to -0.1744	Yes
23173 23:40 X vs. 42:50 X	-0.2387	-0.3996 to -0.07771	Yes
23174 23:40 X vs. 43:0 X	-3.447	-3.608 to -3.286	Yes
23175 23:40 X vs. 43:1 X	-3.361	-3.522 to -3.200	Yes

2way ANOVA Multiple comparisons		A Data Set-A Y	B Data Set-B Y	C Data Set-C Y
23176	23:40 X vs. 43:10 X	-3.077	-3.238 to -2.916	Yes
23177	23:40 X vs. 43:40 X	-0.3337	-0.4946 to -0.1727	Yes
23178	23:40 X vs. 43:50 X	-0.2390	-0.4000 to -0.07805	Yes
23179	23:40 X vs. 44:0 X	-3.471	-3.632 to -3.310	Yes
23180	23:40 X vs. 44:1 X	-3.360	-3.521 to -3.199	Yes
23181	23:40 X vs. 44:10 X	-3.140	-3.301 to -2.979	Yes
23182	23:40 X vs. 44:40 X	-0.3357	-0.4966 to -0.1747	Yes
23183	23:40 X vs. 44:50 X	-0.1713	-0.3323 to -0.01038	Yes
23184	23:40 X vs. 45:0 X	-3.335	-3.496 to -3.174	Yes
23185	23:40 X vs. 45:1 X	-3.137	-3.298 to -2.976	Yes
23186	23:40 X vs. 45:10 X	-3.020	-3.181 to -2.859	Yes
23187	23:40 X vs. 45:40 X	-0.3370	-0.4980 to -0.1760	Yes
23188	23:40 X vs. 45:50 X	-0.1683	-0.3293 to -0.007381	Yes
23189	23:40 X vs. 46:0 X	-3.140	-3.301 to -2.979	Yes
23190	23:40 X vs. 46:1 X	-3.107	-3.268 to -2.946	Yes
23191	23:40 X vs. 46:10 X	-3.015	-3.176 to -2.854	Yes
23192	23:40 X vs. 46:40 X	-0.3507	-0.5116 to -0.1897	Yes
23193	23:40 X vs. 46:50 X	-0.1613	-0.3223 to -0.0003814	Yes
23194	23:40 X vs. 47:0 X	-3.115	-3.276 to -2.954	Yes
23195	23:40 X vs. 47:1 X	-3.019	-3.180 to -2.858	Yes
23196	23:40 X vs. 47:10 X	-3.001	-3.162 to -2.840	Yes
23197	23:40 X vs. 47:40 X	-0.3607	-0.5216 to -0.1997	Yes
23198	23:40 X vs. 47:50 X	-0.1193	-0.2803 to 0.04162	No
23199	23:40 X vs. 48:0 X	-3.103	-3.279 to -2.926	Yes
23200	23:40 X vs. 48:1 X	-3.006	-3.167 to -2.845	Yes
23201	23:40 X vs. 48:10 X	-3.001	-3.162 to -2.840	Yes
23202	23:40 X vs. 48:40 X	-0.3670	-0.5280 to -0.2060	Yes
23203	23:40 X vs. 48:50 X	-0.1267	-0.2876 to 0.03429	No
23204	23:40 X vs. 49:0 X	-3.103	-3.264 to -2.942	Yes
23205	23:40 X vs. 49:1 X	-3.006	-3.167 to -2.845	Yes
23206	23:40 X vs. 49:10 X	-3.001	-3.162 to -2.840	Yes
23207	23:40 X vs. 49:40 X	-0.3713	-0.5323 to -0.2104	Yes
23208	23:40 X vs. 49:50 X	-0.1260	-0.2870 to 0.03495	No
23209	23:40 X vs. 50:0 X	-3.103	-3.264 to -2.942	Yes
23210	23:40 X vs. 50:1 X	-3.006	-3.167 to -2.845	Yes
23211	23:40 X vs. 50:10 X	-3.001	-3.162 to -2.840	Yes
23212	23:40 X vs. 50:40 X	-0.3790	-0.5400 to -0.2180	Yes
23213	23:40 X vs. 50:50 X	-0.1257	-0.2866 to 0.03529	No
23214	23:50 X vs. 24:0 X	-0.2637	-0.4076 to -0.1197	Yes
23215	23:50 X vs. 24:1 X	-0.4200	-0.5810 to -0.2590	Yes
23216	23:50 X vs. 24:10 X	-0.2475	-0.4085 to -0.08655	Yes
23217	23:50 X vs. 24:40 X	0.01767	-0.1263 to 0.1616	No
23218	23:50 X vs. 24:50 X	0.09600	-0.04796 to 0.2400	No
23219	23:50 X vs. 25:0 X	-0.6010	-0.7620 to -0.4400	Yes
23220	23:50 X vs. 25:1 X	-0.7905	-0.9515 to -0.6295	Yes

	2way ANOVA<br>Multiple comparisons	A	B	C
		Data Set-A	Data Set-B	Data Set-C
		Y	Y	Y
23221	23:50 X vs. 25:10 X	-0.1520	-0.3556 to 0.05159	No
23222	23:50 X vs. 25:40 X	0.01567	-0.1283 to 0.1596	No
23223	23:50 X vs. 25:50 X	0.04133	-0.1026 to 0.1853	No
23224	23:50 X vs. 26:0 X	-0.8135	-0.9745 to -0.6525	Yes
23225	23:50 X vs. 26:1 X	-0.9265	-1.087 to -0.7655	Yes
23226	23:50 X vs. 26:10 X	-0.6130	-0.7740 to -0.4520	Yes
23227	23:50 X vs. 26:40 X	-0.05867	-0.2026 to 0.08529	No
23228	23:50 X vs. 26:50 X	0.08100	-0.06296 to 0.2250	No
23229	23:50 X vs. 27:0 X	-1.001	-1.145 to -0.8574	Yes
23230	23:50 X vs. 27:1 X	-1.092	-1.253 to -0.9310	Yes
23231	23:50 X vs. 27:10 X	-0.9040	-1.065 to -0.7430	Yes
23232	23:50 X vs. 27:40 X	-0.05867	-0.2026 to 0.08529	No
23233	23:50 X vs. 27:50 X	0.08167	-0.06229 to 0.2256	No
23234	23:50 X vs. 28:0 X	-0.9505	-1.111 to -0.7895	Yes
23235	23:50 X vs. 28:1 X	-0.9913	-1.135 to -0.8474	Yes
23236	23:50 X vs. 28:10 X	-0.7930	-0.9540 to -0.6320	Yes
23237	23:50 X vs. 28:40 X	-0.0710	-0.2150 to 0.07296	No
23238	23:50 X vs. 28:50 X	0.03367	-0.1103 to 0.1776	No
23239	23:50 X vs. 29:0 X	-1.432	-1.593 to -1.271	Yes
23240	23:50 X vs. 29:1 X	-1.517	-1.678 to -1.356	Yes
23241	23:50 X vs. 29:10 X	-0.7315	-0.8925 to -0.5705	Yes
23242	23:50 X vs. 29:40 X	-0.1790	-0.3230 to -0.03504	Yes
23243	23:50 X vs. 29:50 X	0.03233	-0.1116 to 0.1763	No
23244	23:50 X vs. 30:0 X	-1.147	-1.307 to -0.9855	Yes
23245	23:50 X vs. 30:1 X	-1.468	-1.629 to -1.307	Yes
23246	23:50 X vs. 30:10 X	-0.8060	-0.9670 to -0.6450	Yes
23247	23:50 X vs. 30:40 X	-0.1060	-0.2500 to 0.03796	No
23248	23:50 X vs. 30:50 X	-0.02000	-0.1640 to 0.1240	No
23249	23:50 X vs. 31:0 X	-1.465	-1.625 to -1.304	Yes
23250	23:50 X vs. 31:1 X	-1.455	-1.616 to -1.294	Yes
23251	23:50 X vs. 31:10 X	-0.9375	-1.098 to -0.7765	Yes
23252	23:50 X vs. 31:40 X	-0.1977	-0.3416 to -0.05371	Yes
23253	23:50 X vs. 31:50 X	0.001000	-0.1430 to 0.1450	No
23254	23:50 X vs. 32:0 X	-2.056	-2.200 to -1.912	Yes
23255	23:50 X vs. 32:1 X	-1.512	-1.673 to -1.351	Yes
23256	23:50 X vs. 32:10 X	-1.368	-1.512 to -1.224	Yes
23257	23:50 X vs. 32:40 X	-0.1940	-0.3380 to -0.05004	Yes
23258	23:50 X vs. 32:50 X	0.001333	-0.1426 to 0.1453	No
23259	23:50 X vs. 33:0 X	-2.060	-2.204 to -1.916	Yes
23260	23:50 X vs. 33:1 X	-1.874	-2.018 to -1.730	Yes
23261	23:50 X vs. 33:10 X	-1.535	-1.679 to -1.391	Yes
23262	23:50 X vs. 33:40 X	-0.2127	-0.3566 to -0.06871	Yes
23263	23:50 X vs. 33:50 X	-0.01367	-0.1576 to 0.1303	No
23264	23:50 X vs. 34:0 X	-1.984	-2.128 to -1.840	Yes
23265	23:50 X vs. 34:1 X	-1.992	-2.136 to -1.848	Yes

	2way ANOVA Multiple comparisons	A Data Set-A Y	B Data Set-B Y	C Data Set-C Y
23266	23:**50 X** vs. 34:**10 X**	-1.625	-1.769 to -1.481	Yes
23267	23:**50 X** vs. 34:**40 X**	-0.2247	-0.3686 to -0.08071	Yes
23268	23:**50 X** vs. 34:**50 X**	-0.1627	-0.3066 to -0.01871	Yes
23269	23:**50 X** vs. 35:**0 X**	-2.333	-2.477 to -2.189	Yes
23270	23:**50 X** vs. 35:**1 X**	-2.099	-2.243 to -1.955	Yes
23271	23:**50 X** vs. 35:**10 X**	-1.587	-1.731 to -1.443	Yes
23272	23:**50 X** vs. 35:**40 X**	-0.2337	-0.3776 to -0.08971	Yes
23273	23:**50 X** vs. 35:**50 X**	-0.1753	-0.3193 to -0.03137	Yes
23274	23:**50 X** vs. 36:**0 X**	-2.540	-2.700 to -2.379	Yes
23275	23:**50 X** vs. 36:**1 X**	-2.235	-2.379 to -2.091	Yes
23276	23:**50 X** vs. 36:**10 X**	-1.592	-1.736 to -1.448	Yes
23277	23:**50 X** vs. 36:**40 X**	-0.2890	-0.4330 to -0.1450	Yes
23278	23:**50 X** vs. 36:**50 X**	-0.1387	-0.2826 to 0.005293	No
23279	23:**50 X** vs. 37:**0 X**	-2.923	-3.067 to -2.779	Yes
23280	23:**50 X** vs. 37:**1 X**	-2.305	-2.449 to -2.161	Yes
23281	23:**50 X** vs. 37:**10 X**	-1.594	-1.738 to -1.450	Yes
23282	23:**50 X** vs. 37:**40 X**	-0.3263	-0.4703 to -0.1824	Yes
23283	23:**50 X** vs. 37:**50 X**	-0.1473	-0.2913 to -0.003374	Yes
23284	23:**50 X** vs. 38:**0 X**	-2.868	-3.012 to -2.724	Yes
23285	23:**50 X** vs. 38:**1 X**	-2.424	-2.568 to -2.280	Yes
23286	23:**50 X** vs. 38:**10 X**	-1.869	-2.013 to -1.725	Yes
23287	23:**50 X** vs. 38:**40 X**	-0.3307	-0.4746 to -0.1867	Yes
23288	23:**50 X** vs. 38:**50 X**	-0.1513	-0.2953 to -0.007374	Yes
23289	23:**50 X** vs. 39:**0 X**	-3.099	-3.243 to -2.955	Yes
23290	23:**50 X** vs. 39:**1 X**	-2.991	-3.135 to -2.847	Yes
23291	23:**50 X** vs. 39:**10 X**	-2.014	-2.158 to -1.870	Yes
23292	23:**50 X** vs. 39:**40 X**	-0.3367	-0.4806 to -0.1927	Yes
23293	23:**50 X** vs. 39:**50 X**	-0.1823	-0.3263 to -0.03837	Yes
23294	23:**50 X** vs. 40:**0 X**	-3.140	-3.284 to -2.996	Yes
23295	23:**50 X** vs. 40:**1 X**	-3.066	-3.210 to -2.922	Yes
23296	23:**50 X** vs. 40:**10 X**	-2.271	-2.415 to -2.127	Yes
23297	23:**50 X** vs. 40:**40 X**	-0.3413	-0.4853 to -0.1974	Yes
23298	23:**50 X** vs. 40:**50 X**	-0.1887	-0.3326 to -0.04471	Yes
23299	23:**50 X** vs. 41:**0 X**	-3.181	-3.325 to -3.037	Yes
23300	23:**50 X** vs. 41:**1 X**	-3.180	-3.324 to -3.036	Yes
23301	23:**50 X** vs. 41:**10 X**	-2.324	-2.468 to -2.180	Yes
23302	23:**50 X** vs. 41:**40 X**	-0.2887	-0.4326 to -0.1447	Yes
23303	23:**50 X** vs. 41:**50 X**	-0.2013	-0.3453 to -0.05737	Yes
23304	23:**50 X** vs. 42:**0 X**	-3.323	-3.467 to -3.179	Yes
23305	23:**50 X** vs. 42:**1 X**	-3.342	-3.486 to -3.198	Yes
23306	23:**50 X** vs. 42:**10 X**	-2.325	-2.469 to -2.181	Yes
23307	23:**50 X** vs. 42:**40 X**	-0.3013	-0.4453 to -0.1574	Yes
23308	23:**50 X** vs. 42:**50 X**	-0.2047	-0.3486 to -0.06071	Yes
23309	23:**50 X** vs. 43:**0 X**	-3.413	-3.557 to -3.269	Yes
23310	23:**50 X** vs. 43:**1 X**	-3.327	-3.471 to -3.183	Yes

2way ANOVA Multiple comparisons	A Data Set-A Y	B Data Set-B Y	C Data Set-C Y
23311 23:50 X vs. 43:10 X	-3.043	-3.187 to -2.899	Yes
23312 23:50 X vs. 43:40 X	-0.2997	-0.4436 to -0.1557	Yes
23313 23:50 X vs. 43:50 X	-0.2050	-0.3490 to -0.06104	Yes
23314 23:50 X vs. 44:0 X	-3.437	-3.581 to -3.293	Yes
23315 23:50 X vs. 44:1 X	-3.326	-3.470 to -3.182	Yes
23316 23:50 X vs. 44:10 X	-3.106	-3.250 to -2.962	Yes
23317 23:50 X vs. 44:40 X	-0.3017	-0.4456 to -0.1577	Yes
23318 23:50 X vs. 44:50 X	-0.1373	-0.2813 to 0.006626	No
23319 23:50 X vs. 45:0 X	-3.301	-3.445 to -3.157	Yes
23320 23:50 X vs. 45:1 X	-3.103	-3.247 to -2.959	Yes
23321 23:50 X vs. 45:10 X	-2.986	-3.130 to -2.842	Yes
23322 23:50 X vs. 45:40 X	-0.3030	-0.4470 to -0.1590	Yes
23323 23:50 X vs. 45:50 X	-0.1343	-0.2783 to 0.009626	No
23324 23:50 X vs. 46:0 X	-3.106	-3.250 to -2.962	Yes
23325 23:50 X vs. 46:1 X	-3.073	-3.217 to -2.929	Yes
23326 23:50 X vs. 46:10 X	-2.981	-3.125 to -2.837	Yes
23327 23:50 X vs. 46:40 X	-0.3167	-0.4606 to -0.1727	Yes
23328 23:50 X vs. 46:50 X	-0.1273	-0.2713 to 0.01663	No
23329 23:50 X vs. 47:0 X	-3.081	-3.225 to -2.937	Yes
23330 23:50 X vs. 47:1 X	-2.985	-3.129 to -2.841	Yes
23331 23:50 X vs. 47:10 X	-2.967	-3.111 to -2.823	Yes
23332 23:50 X vs. 47:40 X	-0.3267	-0.4706 to -0.1827	Yes
23333 23:50 X vs. 47:50 X	-0.08533	-0.2293 to 0.05863	No
23334 23:50 X vs. 48:0 X	-3.069	-3.229 to -2.908	Yes
23335 23:50 X vs. 48:1 X	-2.972	-3.116 to -2.828	Yes
23336 23:50 X vs. 48:10 X	-2.967	-3.111 to -2.823	Yes
23337 23:50 X vs. 48:40 X	-0.3330	-0.4770 to -0.1890	Yes
23338 23:50 X vs. 48:50 X	-0.09267	-0.2366 to 0.05129	No
23339 23:50 X vs. 49:0 X	-3.069	-3.213 to -2.925	Yes
23340 23:50 X vs. 49:1 X	-2.972	-3.116 to -2.828	Yes
23341 23:50 X vs. 49:10 X	-2.967	-3.111 to -2.823	Yes
23342 23:50 X vs. 49:40 X	-0.3373	-0.4813 to -0.1934	Yes
23343 23:50 X vs. 49:50 X	-0.0920	-0.2360 to 0.05196	No
23344 23:50 X vs. 50:0 X	-3.069	-3.213 to -2.925	Yes
23345 23:50 X vs. 50:1 X	-2.972	-3.116 to -2.828	Yes
23346 23:50 X vs. 50:10 X	-2.967	-3.111 to -2.823	Yes
23347 23:50 X vs. 50:40 X	-0.3450	-0.4890 to -0.2010	Yes
23348 23:50 X vs. 50:50 X	-0.09167	-0.2356 to 0.05229	No
23349 24:0 X vs. 24:1 X	-0.1563	-0.3173 to 0.004619	No
23350 24:0 X vs. 24:10 X	0.01617	-0.1448 to 0.1771	No
23351 24:0 X vs. 24:40 X	0.2813	0.1374 to 0.4253	Yes
23352 24:0 X vs. 24:50 X	0.3597	0.2157 to 0.5036	Yes
23353 24:0 X vs. 25:0 X	-0.3373	-0.4983 to -0.1764	Yes
23354 24:0 X vs. 25:1 X	-0.5268	-0.6878 to -0.3659	Yes
23355 24:0 X vs. 25:10 X	0.1117	-0.09192 to 0.3153	No

2way ANOVA Multiple comparisons	A Data Set-A Y	B Data Set-B Y	C Data Set-C Y
23356 24:0 X vs. 25:40 X	0.2793	0.1354 to 0.4233	Yes
23357 24:0 X vs. 25:50 X	0.3050	0.1610 to 0.4490	Yes
23358 24:0 X vs. 26:0 X	-0.5498	-0.7108 to -0.3889	Yes
23359 24:0 X vs. 26:1 X	-0.6628	-0.8238 to -0.5019	Yes
23360 24:0 X vs. 26:10 X	-0.3493	-0.5103 to -0.1884	Yes
23361 24:0 X vs. 26:40 X	0.2050	0.06104 to 0.3490	Yes
23362 24:0 X vs. 26:50 X	0.3447	0.2007 to 0.4886	Yes
23363 24:0 X vs. 27:0 X	-0.7377	-0.8816 to -0.5937	Yes
23364 24:0 X vs. 27:1 X	-0.8283	-0.9893 to -0.6674	Yes
23365 24:0 X vs. 27:10 X	-0.6403	-0.8013 to -0.4794	Yes
23366 24:0 X vs. 27:40 X	0.2050	0.06104 to 0.3490	Yes
23367 24:0 X vs. 27:50 X	0.3453	0.2014 to 0.4893	Yes
23368 24:0 X vs. 28:0 X	-0.6868	-0.8478 to -0.5259	Yes
23369 24:0 X vs. 28:1 X	-0.7277	-0.8716 to -0.5837	Yes
23370 24:0 X vs. 28:10 X	-0.5293	-0.6903 to -0.3684	Yes
23371 24:0 X vs. 28:40 X	0.1927	0.04871 to 0.3366	Yes
23372 24:0 X vs. 28:50 X	0.2973	0.1534 to 0.4413	Yes
23373 24:0 X vs. 29:0 X	-1.168	-1.329 to -1.007	Yes
23374 24:0 X vs. 29:1 X	-1.253	-1.414 to -1.092	Yes
23375 24:0 X vs. 29:10 X	-0.4678	-0.6288 to -0.3069	Yes
23376 24:0 X vs. 29:40 X	0.08467	-0.05929 to 0.2286	No
23377 24:0 X vs. 29:50 X	0.2960	0.1520 to 0.4400	Yes
23378 24:0 X vs. 30:0 X	-0.8828	-1.044 to -0.7219	Yes
23379 24:0 X vs. 30:1 X	-1.204	-1.365 to -1.043	Yes
23380 24:0 X vs. 30:10 X	-0.5423	-0.7033 to -0.3814	Yes
23381 24:0 X vs. 30:40 X	0.1577	0.01371 to 0.3016	Yes
23382 24:0 X vs. 30:50 X	0.2437	0.09971 to 0.3876	Yes
23383 24:0 X vs. 31:0 X	-1.201	-1.362 to -1.040	Yes
23384 24:0 X vs. 31:1 X	-1.191	-1.352 to -1.030	Yes
23385 24:0 X vs. 31:10 X	-0.6738	-0.8348 to -0.5129	Yes
23386 24:0 X vs. 31:40 X	0.06600	-0.07796 to 0.2100	No
23387 24:0 X vs. 31:50 X	0.2647	0.1207 to 0.4086	Yes
23388 24:0 X vs. 32:0 X	-1.792	-1.936 to -1.648	Yes
23389 24:0 X vs. 32:1 X	-1.248	-1.409 to -1.087	Yes
23390 24:0 X vs. 32:10 X	-1.104	-1.248 to -0.9604	Yes
23391 24:0 X vs. 32:40 X	0.06967	-0.07429 to 0.2136	No
23392 24:0 X vs. 32:50 X	0.2650	0.1210 to 0.4090	Yes
23393 24:0 X vs. 33:0 X	-1.796	-1.940 to -1.652	Yes
23394 24:0 X vs. 33:1 X	-1.611	-1.755 to -1.467	Yes
23395 24:0 X vs. 33:10 X	-1.271	-1.415 to -1.127	Yes
23396 24:0 X vs. 33:40 X	0.0510	-0.09296 to 0.1950	No
23397 24:0 X vs. 33:50 X	0.2500	0.1060 to 0.3940	Yes
23398 24:0 X vs. 34:0 X	-1.721	-1.865 to -1.577	Yes
23399 24:0 X vs. 34:1 X	-1.728	-1.872 to -1.584	Yes
23400 24:0 X vs. 34:10 X	-1.361	-1.505 to -1.217	Yes

	2way ANOVA Multiple comparisons	A Data Set-A Y	B Data Set-B Y	C Data Set-C Y
23401	24:**0 X** vs. 34:**40 X**	0.03900	-0.1050 to 0.1830	No
23402	24:**0 X** vs. 34:**50 X**	0.1010	-0.04296 to 0.2450	No
23403	24:**0 X** vs. 35:**0 X**	-2.069	-2.213 to -1.925	Yes
23404	24:**0 X** vs. 35:**1 X**	-1.835	-1.979 to -1.691	Yes
23405	24:**0 X** vs. 35:**10 X**	-1.323	-1.467 to -1.179	Yes
23406	24:**0 X** vs. 35:**40 X**	0.03000	-0.1140 to 0.1740	No
23407	24:**0 X** vs. 35:**50 X**	0.08833	-0.05563 to 0.2323	No
23408	24:**0 X** vs. 36:**0 X**	-2.276	-2.437 to -2.115	Yes
23409	24:**0 X** vs. 36:**1 X**	-1.972	-2.116 to -1.828	Yes
23410	24:**0 X** vs. 36:**10 X**	-1.329	-1.473 to -1.185	Yes
23411	24:**0 X** vs. 36:**40 X**	-0.02533	-0.1693 to 0.1186	No
23412	24:**0 X** vs. 36:**50 X**	0.1250	-0.01896 to 0.2690	No
23413	24:**0 X** vs. 37:**0 X**	-2.660	-2.804 to -2.516	Yes
23414	24:**0 X** vs. 37:**1 X**	-2.042	-2.186 to -1.898	Yes
23415	24:**0 X** vs. 37:**10 X**	-1.330	-1.474 to -1.186	Yes
23416	24:**0 X** vs. 37:**40 X**	-0.06267	-0.2066 to 0.08129	No
23417	24:**0 X** vs. 37:**50 X**	0.1163	-0.02763 to 0.2603	No
23418	24:**0 X** vs. 38:**0 X**	-2.605	-2.749 to -2.461	Yes
23419	24:**0 X** vs. 38:**1 X**	-2.160	-2.304 to -2.016	Yes
23420	24:**0 X** vs. 38:**10 X**	-1.606	-1.750 to -1.462	Yes
23421	24:**0 X** vs. 38:**40 X**	-0.06700	-0.2110 to 0.07696	No
23422	24:**0 X** vs. 38:**50 X**	0.1123	-0.03163 to 0.2563	No
23423	24:**0 X** vs. 39:**0 X**	-2.835	-2.979 to -2.691	Yes
23424	24:**0 X** vs. 39:**1 X**	-2.727	-2.871 to -2.583	Yes
23425	24:**0 X** vs. 39:**10 X**	-1.751	-1.895 to -1.607	Yes
23426	24:**0 X** vs. 39:**40 X**	-0.07300	-0.2170 to 0.07096	No
23427	24:**0 X** vs. 39:**50 X**	0.08133	-0.06263 to 0.2253	No
23428	24:**0 X** vs. 40:**0 X**	-2.876	-3.020 to -2.732	Yes
23429	24:**0 X** vs. 40:**1 X**	-2.802	-2.946 to -2.658	Yes
23430	24:**0 X** vs. 40:**10 X**	-2.007	-2.151 to -1.863	Yes
23431	24:**0 X** vs. 40:**40 X**	-0.07767	-0.2216 to 0.06629	No
23432	24:**0 X** vs. 40:**50 X**	0.07500	-0.06896 to 0.2190	No
23433	24:**0 X** vs. 41:**0 X**	-2.917	-3.061 to -2.773	Yes
23434	24:**0 X** vs. 41:**1 X**	-2.916	-3.060 to -2.772	Yes
23435	24:**0 X** vs. 41:**10 X**	-2.061	-2.205 to -1.917	Yes
23436	24:**0 X** vs. 41:**40 X**	-0.02500	-0.1690 to 0.1190	No
23437	24:**0 X** vs. 41:**50 X**	0.06233	-0.08163 to 0.2063	No
23438	24:**0 X** vs. 42:**0 X**	-3.059	-3.203 to -2.915	Yes
23439	24:**0 X** vs. 42:**1 X**	-3.079	-3.223 to -2.935	Yes
23440	24:**0 X** vs. 42:**10 X**	-2.061	-2.205 to -1.917	Yes
23441	24:**0 X** vs. 42:**40 X**	-0.03767	-0.1816 to 0.1063	No
23442	24:**0 X** vs. 42:**50 X**	0.0590	-0.08496 to 0.2030	No
23443	24:**0 X** vs. 43:**0 X**	-3.149	-3.293 to -3.005	Yes
23444	24:**0 X** vs. 43:**1 X**	-3.063	-3.207 to -2.919	Yes
23445	24:**0 X** vs. 43:**10 X**	-2.779	-2.923 to -2.635	Yes

	**2way ANOVA**<br>Multiple comparisons	**A**<br>Data Set-A<br>**Y**	**B**<br>Data Set-B<br>**Y**	**C**<br>Data Set-C<br>**Y**
23446	24:**0 X** vs. 43:**40 X**	-0.03600	-0.1800 to 0.1080	No
23447	24:**0 X** vs. 43:**50 X**	0.05867	-0.08529 to 0.2026	No
23448	24:**0 X** vs. 44:**0 X**	-3.173	-3.317 to -3.029	Yes
23449	24:**0 X** vs. 44:**1 X**	-3.063	-3.207 to -2.919	Yes
23450	24:**0 X** vs. 44:**10 X**	-2.842	-2.986 to -2.698	Yes
23451	24:**0 X** vs. 44:**40 X**	-0.03800	-0.1820 to 0.1060	No
23452	24:**0 X** vs. 44:**50 X**	0.1263	-0.01763 to 0.2703	No
23453	24:**0 X** vs. 45:**0 X**	-3.038	-3.182 to -2.894	Yes
23454	24:**0 X** vs. 45:**1 X**	-2.840	-2.984 to -2.696	Yes
23455	24:**0 X** vs. 45:**10 X**	-2.722	-2.866 to -2.578	Yes
23456	24:**0 X** vs. 45:**40 X**	-0.03933	-0.1833 to 0.1046	No
23457	24:**0 X** vs. 45:**50 X**	0.1293	-0.01463 to 0.2733	No
23458	24:**0 X** vs. 46:**0 X**	-2.842	-2.986 to -2.698	Yes
23459	24:**0 X** vs. 46:**1 X**	-2.809	-2.953 to -2.665	Yes
23460	24:**0 X** vs. 46:**10 X**	-2.717	-2.861 to -2.573	Yes
23461	24:**0 X** vs. 46:**40 X**	-0.05300	-0.1970 to 0.09096	No
23462	24:**0 X** vs. 46:**50 X**	0.1363	-0.007626 to 0.2803	No
23463	24:**0 X** vs. 47:**0 X**	-2.818	-2.962 to -2.674	Yes
23464	24:**0 X** vs. 47:**1 X**	-2.722	-2.866 to -2.578	Yes
23465	24:**0 X** vs. 47:**10 X**	-2.703	-2.847 to -2.559	Yes
23466	24:**0 X** vs. 47:**40 X**	-0.0630	-0.2070 to 0.08096	No
23467	24:**0 X** vs. 47:**50 X**	0.1783	0.03437 to 0.3223	Yes
23468	24:**0 X** vs. 48:**0 X**	-2.805	-2.966 to -2.644	Yes
23469	24:**0 X** vs. 48:**1 X**	-2.709	-2.853 to -2.565	Yes
23470	24:**0 X** vs. 48:**10 X**	-2.703	-2.847 to -2.559	Yes
23471	24:**0 X** vs. 48:**40 X**	-0.06933	-0.2133 to 0.07463	No
23472	24:**0 X** vs. 48:**50 X**	0.1710	0.02704 to 0.3150	Yes
23473	24:**0 X** vs. 49:**0 X**	-2.805	-2.949 to -2.661	Yes
23474	24:**0 X** vs. 49:**1 X**	-2.709	-2.853 to -2.565	Yes
23475	24:**0 X** vs. 49:**10 X**	-2.703	-2.847 to -2.559	Yes
23476	24:**0 X** vs. 49:**40 X**	-0.07367	-0.2176 to 0.07029	No
23477	24:**0 X** vs. 49:**50 X**	0.1717	0.02771 to 0.3156	Yes
23478	24:**0 X** vs. 50:**0 X**	-2.805	-2.949 to -2.661	Yes
23479	24:**0 X** vs. 50:**1 X**	-2.709	-2.853 to -2.565	Yes
23480	24:**0 X** vs. 50:**10 X**	-2.703	-2.847 to -2.559	Yes
23481	24:**0 X** vs. 50:**40 X**	-0.08133	-0.2253 to 0.06263	No
23482	24:**0 X** vs. 50:**50 X**	0.1720	0.02804 to 0.3160	Yes
23483	24:**1 X** vs. 24:**10 X**	0.1725	-0.003814 to 0.3488	No
23484	24:**1 X** vs. 24:**40 X**	0.4377	0.2767 to 0.5986	Yes
23485	24:**1 X** vs. 24:**50 X**	0.5160	0.3550 to 0.6770	Yes
23486	24:**1 X** vs. 25:**0 X**	-0.1810	-0.3573 to -0.004686	Yes
23487	24:**1 X** vs. 25:**1 X**	-0.3705	-0.5468 to -0.1942	Yes
23488	24:**1 X** vs. 25:**10 X**	0.2680	0.05206 to 0.4839	Yes
23489	24:**1 X** vs. 25:**40 X**	0.4357	0.2747 to 0.5966	Yes
23490	24:**1 X** vs. 25:**50 X**	0.4613	0.3004 to 0.6223	Yes

	2way ANOVA<br>Multiple comparisons	A<br>Data Set-A<br>Y	B<br>Data Set-B<br>Y	C<br>Data Set-C<br>Y
23491	24:1 X vs. 26:0 X	-0.3935	-0.5698 to -0.2172	Yes
23492	24:1 X vs. 26:1 X	-0.5065	-0.6828 to -0.3302	Yes
23493	24:1 X vs. 26:10 X	-0.1930	-0.3693 to -0.01669	Yes
23494	24:1 X vs. 26:40 X	0.3613	0.2004 to 0.5223	Yes
23495	24:1 X vs. 26:50 X	0.5010	0.3400 to 0.6620	Yes
23496	24:1 X vs. 27:0 X	-0.5813	-0.7423 to -0.4204	Yes
23497	24:1 X vs. 27:1 X	-0.6720	-0.8483 to -0.4957	Yes
23498	24:1 X vs. 27:10 X	-0.4840	-0.6603 to -0.3077	Yes
23499	24:1 X vs. 27:40 X	0.3613	0.2004 to 0.5223	Yes
23500	24:1 X vs. 27:50 X	0.5017	0.3407 to 0.6626	Yes
23501	24:1 X vs. 28:0 X	-0.5305	-0.7068 to -0.3542	Yes
23502	24:1 X vs. 28:1 X	-0.5713	-0.7323 to -0.4104	Yes
23503	24:1 X vs. 28:10 X	-0.3730	-0.5493 to -0.1967	Yes
23504	24:1 X vs. 28:40 X	0.3490	0.1880 to 0.5100	Yes
23505	24:1 X vs. 28:50 X	0.4537	0.2927 to 0.6146	Yes
23506	24:1 X vs. 29:0 X	-1.012	-1.188 to -0.8357	Yes
23507	24:1 X vs. 29:1 X	-1.097	-1.273 to -0.9207	Yes
23508	24:1 X vs. 29:10 X	-0.3115	-0.4878 to -0.1352	Yes
23509	24:1 X vs. 29:40 X	0.2410	0.08005 to 0.4020	Yes
23510	24:1 X vs. 29:50 X	0.4523	0.2914 to 0.6133	Yes
23511	24:1 X vs. 30:0 X	-0.7265	-0.9028 to -0.5502	Yes
23512	24:1 X vs. 30:1 X	-1.048	-1.224 to -0.8717	Yes
23513	24:1 X vs. 30:10 X	-0.3860	-0.5623 to -0.2097	Yes
23514	24:1 X vs. 30:40 X	0.3140	0.1530 to 0.4750	Yes
23515	24:1 X vs. 30:50 X	0.4000	0.2390 to 0.5610	Yes
23516	24:1 X vs. 31:0 X	-1.045	-1.221 to -0.8682	Yes
23517	24:1 X vs. 31:1 X	-1.035	-1.211 to -0.8587	Yes
23518	24:1 X vs. 31:10 X	-0.5175	-0.6938 to -0.3412	Yes
23519	24:1 X vs. 31:40 X	0.2223	0.06138 to 0.3833	Yes
23520	24:1 X vs. 31:50 X	0.4210	0.2600 to 0.5820	Yes
23521	24:1 X vs. 32:0 X	-1.636	-1.797 to -1.475	Yes
23522	24:1 X vs. 32:1 X	-1.092	-1.268 to -0.9157	Yes
23523	24:1 X vs. 32:10 X	-0.9480	-1.109 to -0.7870	Yes
23524	24:1 X vs. 32:40 X	0.2260	0.06505 to 0.3870	Yes
23525	24:1 X vs. 32:50 X	0.4213	0.2604 to 0.5823	Yes
23526	24:1 X vs. 33:0 X	-1.640	-1.801 to -1.479	Yes
23527	24:1 X vs. 33:1 X	-1.454	-1.615 to -1.293	Yes
23528	24:1 X vs. 33:10 X	-1.115	-1.276 to -0.9537	Yes
23529	24:1 X vs. 33:40 X	0.2073	0.04638 to 0.3683	Yes
23530	24:1 X vs. 33:50 X	0.4063	0.2454 to 0.5673	Yes
23531	24:1 X vs. 34:0 X	-1.564	-1.725 to -1.403	Yes
23532	24:1 X vs. 34:1 X	-1.572	-1.733 to -1.411	Yes
23533	24:1 X vs. 34:10 X	-1.205	-1.366 to -1.044	Yes
23534	24:1 X vs. 34:40 X	0.1953	0.03438 to 0.3563	Yes
23535	24:1 X vs. 34:50 X	0.2573	0.09638 to 0.4183	Yes

2way ANOVA Multiple comparisons	A Data Set-A Y	B Data Set-B Y	C Data Set-C Y
23536 24:1 X vs. 35:0 X	-1.913	-2.074 to -1.752	Yes
23537 24:1 X vs. 35:1 X	-1.679	-1.840 to -1.518	Yes
23538 24:1 X vs. 35:10 X	-1.167	-1.328 to -1.006	Yes
23539 24:1 X vs. 35:40 X	0.1863	0.02538 to 0.3473	Yes
23540 24:1 X vs. 35:50 X	0.2447	0.08371 to 0.4056	Yes
23541 24:1 X vs. 36:0 X	-2.120	-2.296 to -1.943	Yes
23542 24:1 X vs. 36:1 X	-1.815	-1.976 to -1.654	Yes
23543 24:1 X vs. 36:10 X	-1.172	-1.333 to -1.011	Yes
23544 24:1 X vs. 36:40 X	0.1310	-0.02995 to 0.2920	No
23545 24:1 X vs. 36:50 X	0.2813	0.1204 to 0.4423	Yes
23546 24:1 X vs. 37:0 X	-2.503	-2.664 to -2.342	Yes
23547 24:1 X vs. 37:1 X	-1.885	-2.046 to -1.724	Yes
23548 24:1 X vs. 37:10 X	-1.174	-1.335 to -1.013	Yes
23549 24:1 X vs. 37:40 X	0.09367	-0.06729 to 0.2546	No
23550 24:1 X vs. 37:50 X	0.2727	0.1117 to 0.4336	Yes
23551 24:1 X vs. 38:0 X	-2.448	-2.609 to -2.287	Yes
23552 24:1 X vs. 38:1 X	-2.004	-2.165 to -1.843	Yes
23553 24:1 X vs. 38:10 X	-1.449	-1.610 to -1.288	Yes
23554 24:1 X vs. 38:40 X	0.08933	-0.07162 to 0.2503	No
23555 24:1 X vs. 38:50 X	0.2687	0.1077 to 0.4296	Yes
23556 24:1 X vs. 39:0 X	-2.679	-2.840 to -2.518	Yes
23557 24:1 X vs. 39:1 X	-2.571	-2.732 to -2.410	Yes
23558 24:1 X vs. 39:10 X	-1.594	-1.755 to -1.433	Yes
23559 24:1 X vs. 39:40 X	0.08333	-0.07762 to 0.2443	No
23560 24:1 X vs. 39:50 X	0.2377	0.07671 to 0.3986	Yes
23561 24:1 X vs. 40:0 X	-2.720	-2.881 to -2.559	Yes
23562 24:1 X vs. 40:1 X	-2.646	-2.807 to -2.485	Yes
23563 24:1 X vs. 40:10 X	-1.851	-2.012 to -1.690	Yes
23564 24:1 X vs. 40:40 X	0.07867	-0.08229 to 0.2396	No
23565 24:1 X vs. 40:50 X	0.2313	0.07038 to 0.3923	Yes
23566 24:1 X vs. 41:0 X	-2.761	-2.922 to -2.600	Yes
23567 24:1 X vs. 41:1 X	-2.760	-2.921 to -2.599	Yes
23568 24:1 X vs. 41:10 X	-1.904	-2.065 to -1.743	Yes
23569 24:1 X vs. 41:40 X	0.1313	-0.02962 to 0.2923	No
23570 24:1 X vs. 41:50 X	0.2187	0.05771 to 0.3796	Yes
23571 24:1 X vs. 42:0 X	-2.903	-3.064 to -2.742	Yes
23572 24:1 X vs. 42:1 X	-2.922	-3.083 to -2.761	Yes
23573 24:1 X vs. 42:10 X	-1.905	-2.066 to -1.744	Yes
23574 24:1 X vs. 42:40 X	0.1187	-0.04229 to 0.2796	No
23575 24:1 X vs. 42:50 X	0.2153	0.05438 to 0.3763	Yes
23576 24:1 X vs. 43:0 X	-2.993	-3.154 to -2.832	Yes
23577 24:1 X vs. 43:1 X	-2.907	-3.068 to -2.746	Yes
23578 24:1 X vs. 43:10 X	-2.623	-2.784 to -2.462	Yes
23579 24:1 X vs. 43:40 X	0.1203	-0.04062 to 0.2813	No
23580 24:1 X vs. 43:50 X	0.2150	0.05405 to 0.3760	Yes

2way ANOVA<br>Multiple comparisons		A<br>Data Set-A<br>Y	B<br>Data Set-B<br>Y	C<br>Data Set-C<br>Y
23581	24:**1 X** vs. 44:**0 X**	-3.017	-3.178 to -2.856	Yes
23582	24:**1 X** vs. 44:**1 X**	-2.906	-3.067 to -2.745	Yes
23583	24:**1 X** vs. 44:**10 X**	-2.686	-2.847 to -2.525	Yes
23584	24:**1 X** vs. 44:**40 X**	0.1183	-0.04262 to 0.2793	No
23585	24:**1 X** vs. 44:**50 X**	0.2827	0.1217 to 0.4436	Yes
23586	24:**1 X** vs. 45:**0 X**	-2.881	-3.042 to -2.720	Yes
23587	24:**1 X** vs. 45:**1 X**	-2.683	-2.844 to -2.522	Yes
23588	24:**1 X** vs. 45:**10 X**	-2.566	-2.727 to -2.405	Yes
23589	24:**1 X** vs. 45:**40 X**	0.1170	-0.04395 to 0.2780	No
23590	24:**1 X** vs. 45:**50 X**	0.2857	0.1247 to 0.4466	Yes
23591	24:**1 X** vs. 46:**0 X**	-2.686	-2.847 to -2.525	Yes
23592	24:**1 X** vs. 46:**1 X**	-2.653	-2.814 to -2.492	Yes
23593	24:**1 X** vs. 46:**10 X**	-2.561	-2.722 to -2.400	Yes
23594	24:**1 X** vs. 46:**40 X**	0.1033	-0.05762 to 0.2643	No
23595	24:**1 X** vs. 46:**50 X**	0.2927	0.1317 to 0.4536	Yes
23596	24:**1 X** vs. 47:**0 X**	-2.661	-2.822 to -2.500	Yes
23597	24:**1 X** vs. 47:**1 X**	-2.565	-2.726 to -2.404	Yes
23598	24:**1 X** vs. 47:**10 X**	-2.547	-2.708 to -2.386	Yes
23599	24:**1 X** vs. 47:**40 X**	0.09333	-0.06762 to 0.2543	No
23600	24:**1 X** vs. 47:**50 X**	0.3347	0.1737 to 0.4956	Yes
23601	24:**1 X** vs. 48:**0 X**	-2.649	-2.825 to -2.472	Yes
23602	24:**1 X** vs. 48:**1 X**	-2.552	-2.713 to -2.391	Yes
23603	24:**1 X** vs. 48:**10 X**	-2.547	-2.708 to -2.386	Yes
23604	24:**1 X** vs. 48:**40 X**	0.08700	-0.07395 to 0.2480	No
23605	24:**1 X** vs. 48:**50 X**	0.3273	0.1664 to 0.4883	Yes
23606	24:**1 X** vs. 49:**0 X**	-2.649	-2.810 to -2.488	Yes
23607	24:**1 X** vs. 49:**1 X**	-2.552	-2.713 to -2.391	Yes
23608	24:**1 X** vs. 49:**10 X**	-2.547	-2.708 to -2.386	Yes
23609	24:**1 X** vs. 49:**40 X**	0.08267	-0.07829 to 0.2436	No
23610	24:**1 X** vs. 49:**50 X**	0.3280	0.1670 to 0.4890	Yes
23611	24:**1 X** vs. 50:**0 X**	-2.649	-2.810 to -2.488	Yes
23612	24:**1 X** vs. 50:**1 X**	-2.552	-2.713 to -2.391	Yes
23613	24:**1 X** vs. 50:**10 X**	-2.547	-2.708 to -2.386	Yes
23614	24:**1 X** vs. 50:**40 X**	0.07500	-0.08595 to 0.2360	No
23615	24:**1 X** vs. 50:**50 X**	0.3283	0.1674 to 0.4893	Yes
23616	24:**10 X** vs. 24:**40 X**	0.2652	0.1042 to 0.4261	Yes
23617	24:**10 X** vs. 24:**50 X**	0.3435	0.1825 to 0.5045	Yes
23618	24:**10 X** vs. 25:**0 X**	-0.3535	-0.5298 to -0.1772	Yes
23619	24:**10 X** vs. 25:**1 X**	-0.5430	-0.7193 to -0.3667	Yes
23620	24:**10 X** vs. 25:**10 X**	0.09550	-0.1204 to 0.3114	No
23621	24:**10 X** vs. 25:**40 X**	0.2632	0.1022 to 0.4241	Yes
23622	24:**10 X** vs. 25:**50 X**	0.2888	0.1279 to 0.4498	Yes
23623	24:**10 X** vs. 26:**0 X**	-0.5660	-0.7423 to -0.3897	Yes
23624	24:**10 X** vs. 26:**1 X**	-0.6790	-0.8553 to -0.5027	Yes
23625	24:**10 X** vs. 26:**10 X**	-0.3655	-0.5418 to -0.1892	Yes

2way ANOVA Multiple comparisons	A Data Set-A Y	B Data Set-B Y	C Data Set-C Y
23626 24:10 X vs. 26:40 X	0.1888	0.02788 to 0.3498	Yes
23627 24:10 X vs. 26:50 X	0.3285	0.1675 to 0.4895	Yes
23628 24:10 X vs. 27:0 X	-0.7538	-0.9148 to -0.5929	Yes
23629 24:10 X vs. 27:1 X	-0.8445	-1.021 to -0.6682	Yes
23630 24:10 X vs. 27:10 X	-0.6565	-0.8328 to -0.4802	Yes
23631 24:10 X vs. 27:40 X	0.1888	0.02788 to 0.3498	Yes
23632 24:10 X vs. 27:50 X	0.3292	0.1682 to 0.4901	Yes
23633 24:10 X vs. 28:0 X	-0.7030	-0.8793 to -0.5267	Yes
23634 24:10 X vs. 28:1 X	-0.7438	-0.9048 to -0.5829	Yes
23635 24:10 X vs. 28:10 X	-0.5455	-0.7218 to -0.3692	Yes
23636 24:10 X vs. 28:40 X	0.1765	0.01555 to 0.3375	Yes
23637 24:10 X vs. 28:50 X	0.2812	0.1202 to 0.4421	Yes
23638 24:10 X vs. 29:0 X	-1.185	-1.361 to -1.008	Yes
23639 24:10 X vs. 29:1 X	-1.270	-1.446 to -1.093	Yes
23640 24:10 X vs. 29:10 X	-0.4840	-0.6603 to -0.3077	Yes
23641 24:10 X vs. 29:40 X	0.0685	-0.09245 to 0.2295	No
23642 24:10 X vs. 29:50 X	0.2798	0.1189 to 0.4408	Yes
23643 24:10 X vs. 30:0 X	-0.8990	-1.075 to -0.7227	Yes
23644 24:10 X vs. 30:1 X	-1.221	-1.397 to -1.044	Yes
23645 24:10 X vs. 30:10 X	-0.5585	-0.7348 to -0.3822	Yes
23646 24:10 X vs. 30:40 X	0.1415	-0.01945 to 0.3025	No
23647 24:10 X vs. 30:50 X	0.2275	0.06655 to 0.3885	Yes
23648 24:10 X vs. 31:0 X	-1.217	-1.393 to -1.041	Yes
23649 24:10 X vs. 31:1 X	-1.208	-1.384 to -1.031	Yes
23650 24:10 X vs. 31:10 X	-0.6900	-0.8663 to -0.5137	Yes
23651 24:10 X vs. 31:40 X	0.04983	-0.1111 to 0.2108	No
23652 24:10 X vs. 31:50 X	0.2485	0.08755 to 0.4095	Yes
23653 24:10 X vs. 32:0 X	-1.809	-1.969 to -1.648	Yes
23654 24:10 X vs. 32:1 X	-1.265	-1.441 to -1.088	Yes
23655 24:10 X vs. 32:10 X	-1.121	-1.281 to -0.9595	Yes
23656 24:10 X vs. 32:40 X	0.0535	-0.1075 to 0.2145	No
23657 24:10 X vs. 32:50 X	0.2488	0.08788 to 0.4098	Yes
23658 24:10 X vs. 33:0 X	-1.812	-1.973 to -1.651	Yes
23659 24:10 X vs. 33:1 X	-1.627	-1.788 to -1.466	Yes
23660 24:10 X vs. 33:10 X	-1.287	-1.448 to -1.126	Yes
23661 24:10 X vs. 33:40 X	0.03483	-0.1261 to 0.1958	No
23662 24:10 X vs. 33:50 X	0.2338	0.07288 to 0.3948	Yes
23663 24:10 X vs. 34:0 X	-1.737	-1.898 to -1.576	Yes
23664 24:10 X vs. 34:1 X	-1.744	-1.905 to -1.583	Yes
23665 24:10 X vs. 34:10 X	-1.378	-1.538 to -1.217	Yes
23666 24:10 X vs. 34:40 X	0.02283	-0.1381 to 0.1838	No
23667 24:10 X vs. 34:50 X	0.08483	-0.07612 to 0.2458	No
23668 24:10 X vs. 35:0 X	-2.086	-2.246 to -1.925	Yes
23669 24:10 X vs. 35:1 X	-1.851	-2.012 to -1.690	Yes
23670 24:10 X vs. 35:10 X	-1.339	-1.500 to -1.178	Yes

	2way ANOVA<br>Multiple comparisons	A<br>Data Set-A<br>Y	B<br>Data Set-B<br>Y	C<br>Data Set-C<br>Y
23671	24:**10 X** vs. 35:**40 X**	0.01383	-0.1471 to 0.1748	No
23672	24:**10 X** vs. 35:**50 X**	0.07217	-0.08879 to 0.2331	No
23673	24:**10 X** vs. 36:**0 X**	-2.292	-2.468 to -2.116	Yes
23674	24:**10 X** vs. 36:**1 X**	-1.988	-2.149 to -1.827	Yes
23675	24:**10 X** vs. 36:**10 X**	-1.345	-1.506 to -1.184	Yes
23676	24:**10 X** vs. 36:**40 X**	-0.04150	-0.2025 to 0.1195	No
23677	24:**10 X** vs. 36:**50 X**	0.1088	-0.05212 to 0.2698	No
23678	24:**10 X** vs. 37:**0 X**	-2.676	-2.837 to -2.515	Yes
23679	24:**10 X** vs. 37:**1 X**	-2.058	-2.219 to -1.897	Yes
23680	24:**10 X** vs. 37:**10 X**	-1.347	-1.507 to -1.186	Yes
23681	24:**10 X** vs. 37:**40 X**	-0.07883	-0.2398 to 0.08212	No
23682	24:**10 X** vs. 37:**50 X**	0.1002	-0.06079 to 0.2611	No
23683	24:**10 X** vs. 38:**0 X**	-2.621	-2.782 to -2.460	Yes
23684	24:**10 X** vs. 38:**1 X**	-2.177	-2.337 to -2.016	Yes
23685	24:**10 X** vs. 38:**10 X**	-1.622	-1.783 to -1.461	Yes
23686	24:**10 X** vs. 38:**40 X**	-0.08317	-0.2441 to 0.07779	No
23687	24:**10 X** vs. 38:**50 X**	0.09617	-0.06479 to 0.2571	No
23688	24:**10 X** vs. 39:**0 X**	-2.852	-3.012 to -2.691	Yes
23689	24:**10 X** vs. 39:**1 X**	-2.743	-2.904 to -2.582	Yes
23690	24:**10 X** vs. 39:**10 X**	-1.767	-1.928 to -1.606	Yes
23691	24:**10 X** vs. 39:**40 X**	-0.08917	-0.2501 to 0.07179	No
23692	24:**10 X** vs. 39:**50 X**	0.06517	-0.09579 to 0.2261	No
23693	24:**10 X** vs. 40:**0 X**	-2.893	-3.053 to -2.732	Yes
23694	24:**10 X** vs. 40:**1 X**	-2.819	-2.979 to -2.658	Yes
23695	24:**10 X** vs. 40:**10 X**	-2.023	-2.184 to -1.862	Yes
23696	24:**10 X** vs. 40:**40 X**	-0.09383	-0.2548 to 0.06712	No
23697	24:**10 X** vs. 40:**50 X**	0.05883	-0.1021 to 0.2198	No
23698	24:**10 X** vs. 41:**0 X**	-2.934	-3.094 to -2.773	Yes
23699	24:**10 X** vs. 41:**1 X**	-2.933	-3.093 to -2.772	Yes
23700	24:**10 X** vs. 41:**10 X**	-2.077	-2.238 to -1.916	Yes
23701	24:**10 X** vs. 41:**40 X**	-0.04117	-0.2021 to 0.1198	No
23702	24:**10 X** vs. 41:**50 X**	0.04617	-0.1148 to 0.2071	No
23703	24:**10 X** vs. 42:**0 X**	-3.075	-3.236 to -2.914	Yes
23704	24:**10 X** vs. 42:**1 X**	-3.095	-3.256 to -2.934	Yes
23705	24:**10 X** vs. 42:**10 X**	-2.077	-2.238 to -1.916	Yes
23706	24:**10 X** vs. 42:**40 X**	-0.05383	-0.2148 to 0.1071	No
23707	24:**10 X** vs. 42:**50 X**	0.04283	-0.1181 to 0.2038	No
23708	24:**10 X** vs. 43:**0 X**	-3.165	-3.326 to -3.004	Yes
23709	24:**10 X** vs. 43:**1 X**	-3.080	-3.240 to -2.919	Yes
23710	24:**10 X** vs. 43:**10 X**	-2.795	-2.956 to -2.634	Yes
23711	24:**10 X** vs. 43:**40 X**	-0.05217	-0.2131 to 0.1088	No
23712	24:**10 X** vs. 43:**50 X**	0.0425	-0.1185 to 0.2035	No
23713	24:**10 X** vs. 44:**0 X**	-3.189	-3.350 to -3.028	Yes
23714	24:**10 X** vs. 44:**1 X**	-3.079	-3.240 to -2.918	Yes
23715	24:**10 X** vs. 44:**10 X**	-2.859	-3.019 to -2.698	Yes

2way ANOVA<br>Multiple comparisons	A<br>Data Set-A<br>Y	B<br>Data Set-B<br>Y	C<br>Data Set-C<br>Y
23716 24:10 X vs. 44:40 X	-0.05417	-0.2151 to 0.1068	No
23717 24:10 X vs. 44:50 X	0.1102	-0.05079 to 0.2711	No
23718 24:10 X vs. 45:0 X	-3.054	-3.215 to -2.893	Yes
23719 24:10 X vs. 45:1 X	-2.856	-3.017 to -2.695	Yes
23720 24:10 X vs. 45:10 X	-2.738	-2.899 to -2.577	Yes
23721 24:10 X vs. 45:40 X	-0.05550	-0.2165 to 0.1055	No
23722 24:10 X vs. 45:50 X	0.1132	-0.04779 to 0.2741	No
23723 24:10 X vs. 46:0 X	-2.858	-3.019 to -2.697	Yes
23724 24:10 X vs. 46:1 X	-2.825	-2.986 to -2.664	Yes
23725 24:10 X vs. 46:10 X	-2.733	-2.894 to -2.572	Yes
23726 24:10 X vs. 46:40 X	-0.06917	-0.2301 to 0.09179	No
23727 24:10 X vs. 46:50 X	0.1202	-0.04079 to 0.2811	No
23728 24:10 X vs. 47:0 X	-2.834	-2.995 to -2.673	Yes
23729 24:10 X vs. 47:1 X	-2.738	-2.899 to -2.577	Yes
23730 24:10 X vs. 47:10 X	-2.719	-2.880 to -2.558	Yes
23731 24:10 X vs. 47:40 X	-0.07917	-0.2401 to 0.08179	No
23732 24:10 X vs. 47:50 X	0.1622	0.001215 to 0.3231	Yes
23733 24:10 X vs. 48:0 X	-2.821	-2.997 to -2.645	Yes
23734 24:10 X vs. 48:1 X	-2.725	-2.886 to -2.564	Yes
23735 24:10 X vs. 48:10 X	-2.719	-2.880 to -2.558	Yes
23736 24:10 X vs. 48:40 X	-0.0855	-0.2465 to 0.07545	No
23737 24:10 X vs. 48:50 X	0.1548	-0.006119 to 0.3158	No
23738 24:10 X vs. 49:0 X	-2.821	-2.982 to -2.660	Yes
23739 24:10 X vs. 49:1 X	-2.725	-2.886 to -2.564	Yes
23740 24:10 X vs. 49:10 X	-2.719	-2.880 to -2.558	Yes
23741 24:10 X vs. 49:40 X	-0.08983	-0.2508 to 0.07112	No
23742 24:10 X vs. 49:50 X	0.1555	-0.005452 to 0.3165	No
23743 24:10 X vs. 50:0 X	-2.821	-2.982 to -2.660	Yes
23744 24:10 X vs. 50:1 X	-2.725	-2.886 to -2.564	Yes
23745 24:10 X vs. 50:10 X	-2.719	-2.880 to -2.558	Yes
23746 24:10 X vs. 50:40 X	-0.0975	-0.2585 to 0.06345	No
23747 24:10 X vs. 50:50 X	0.1558	-0.005119 to 0.3168	No
23748 24:40 X vs. 24:50 X	0.07833	-0.06563 to 0.2223	No
23749 24:40 X vs. 25:0 X	-0.6187	-0.7796 to -0.4577	Yes
23750 24:40 X vs. 25:1 X	-0.8082	-0.9691 to -0.6472	Yes
23751 24:40 X vs. 25:10 X	-0.1697	-0.3733 to 0.03392	No
23752 24:40 X vs. 25:40 X	-0.002000	-0.1460 to 0.1420	No
23753 24:40 X vs. 25:50 X	0.02367	-0.1203 to 0.1676	No
23754 24:40 X vs. 26:0 X	-0.8312	-0.9921 to -0.6702	Yes
23755 24:40 X vs. 26:1 X	-0.9442	-1.105 to -0.7832	Yes
23756 24:40 X vs. 26:10 X	-0.6307	-0.7916 to -0.4697	Yes
23757 24:40 X vs. 26:40 X	-0.07633	-0.2203 to 0.06763	No
23758 24:40 X vs. 26:50 X	0.06333	-0.08063 to 0.2073	No
23759 24:40 X vs. 27:0 X	-1.019	-1.163 to -0.8750	Yes
23760 24:40 X vs. 27:1 X	-1.110	-1.271 to -0.9487	Yes

2way ANOVA<br>Multiple comparisons	A<br>Data Set-A<br>Y	B<br>Data Set-B<br>Y	C<br>Data Set-C<br>Y
23761 24:40 X vs. 27:10 X	-0.9217	-1.083 to -0.7607	Yes
23762 24:40 X vs. 27:40 X	-0.07633	-0.2203 to 0.06763	No
23763 24:40 X vs. 27:50 X	0.0640	-0.07996 to 0.2080	No
23764 24:40 X vs. 28:0 X	-0.9682	-1.129 to -0.8072	Yes
23765 24:40 X vs. 28:1 X	-1.009	-1.153 to -0.8650	Yes
23766 24:40 X vs. 28:10 X	-0.8107	-0.9716 to -0.6497	Yes
23767 24:40 X vs. 28:40 X	-0.08867	-0.2326 to 0.05529	No
23768 24:40 X vs. 28:50 X	0.01600	-0.1280 to 0.1600	No
23769 24:40 X vs. 29:0 X	-1.450	-1.611 to -1.289	Yes
23770 24:40 X vs. 29:1 X	-1.535	-1.696 to -1.374	Yes
23771 24:40 X vs. 29:10 X	-0.7492	-0.9101 to -0.5882	Yes
23772 24:40 X vs. 29:40 X	-0.1967	-0.3406 to -0.05271	Yes
23773 24:40 X vs. 29:50 X	0.01467	-0.1293 to 0.1586	No
23774 24:40 X vs. 30:0 X	-1.164	-1.325 to -1.003	Yes
23775 24:40 X vs. 30:1 X	-1.486	-1.647 to -1.325	Yes
23776 24:40 X vs. 30:10 X	-0.8237	-0.9846 to -0.6627	Yes
23777 24:40 X vs. 30:40 X	-0.1237	-0.2676 to 0.02029	No
23778 24:40 X vs. 30:50 X	-0.03767	-0.1816 to 0.1063	No
23779 24:40 X vs. 31:0 X	-1.482	-1.643 to -1.321	Yes
23780 24:40 X vs. 31:1 X	-1.473	-1.634 to -1.312	Yes
23781 24:40 X vs. 31:10 X	-0.9552	-1.116 to -0.7942	Yes
23782 24:40 X vs. 31:40 X	-0.2153	-0.3593 to -0.07137	Yes
23783 24:40 X vs. 31:50 X	-0.01667	-0.1606 to 0.1273	No
23784 24:40 X vs. 32:0 X	-2.074	-2.218 to -1.930	Yes
23785 24:40 X vs. 32:1 X	-1.530	-1.691 to -1.369	Yes
23786 24:40 X vs. 32:10 X	-1.386	-1.530 to -1.242	Yes
23787 24:40 X vs. 32:40 X	-0.2117	-0.3556 to -0.06771	Yes
23788 24:40 X vs. 32:50 X	-0.01633	-0.1603 to 0.1276	No
23789 24:40 X vs. 33:0 X	-2.077	-2.221 to -1.933	Yes
23790 24:40 X vs. 33:1 X	-1.892	-2.036 to -1.748	Yes
23791 24:40 X vs. 33:10 X	-1.552	-1.696 to -1.408	Yes
23792 24:40 X vs. 33:40 X	-0.2303	-0.3743 to -0.08637	Yes
23793 24:40 X vs. 33:50 X	-0.03133	-0.1753 to 0.1126	No
23794 24:40 X vs. 34:0 X	-2.002	-2.146 to -1.858	Yes
23795 24:40 X vs. 34:1 X	-2.009	-2.153 to -1.865	Yes
23796 24:40 X vs. 34:10 X	-1.643	-1.787 to -1.499	Yes
23797 24:40 X vs. 34:40 X	-0.2423	-0.3863 to -0.09837	Yes
23798 24:40 X vs. 34:50 X	-0.1803	-0.3243 to -0.03637	Yes
23799 24:40 X vs. 35:0 X	-2.351	-2.495 to -2.207	Yes
23800 24:40 X vs. 35:1 X	-2.116	-2.260 to -1.972	Yes
23801 24:40 X vs. 35:10 X	-1.604	-1.748 to -1.460	Yes
23802 24:40 X vs. 35:40 X	-0.2513	-0.3953 to -0.1074	Yes
23803 24:40 X vs. 35:50 X	-0.1930	-0.3370 to -0.04904	Yes
23804 24:40 X vs. 36:0 X	-2.557	-2.718 to -2.396	Yes
23805 24:40 X vs. 36:1 X	-2.253	-2.397 to -2.109	Yes

2way ANOVA<br>Multiple comparisons	A<br>Data Set-A<br>Y	B<br>Data Set-B<br>Y	C<br>Data Set-C<br>Y
23806 24:40 X vs. 36:10 X	-1.610	-1.754 to -1.466	Yes
23807 24:40 X vs. 36:40 X	-0.3067	-0.4506 to -0.1627	Yes
23808 24:40 X vs. 36:50 X	-0.1563	-0.3003 to -0.01237	Yes
23809 24:40 X vs. 37:0 X	-2.941	-3.085 to -2.797	Yes
23810 24:40 X vs. 37:1 X	-2.323	-2.467 to -2.179	Yes
23811 24:40 X vs. 37:10 X	-1.612	-1.756 to -1.468	Yes
23812 24:40 X vs. 37:40 X	-0.3440	-0.4880 to -0.2000	Yes
23813 24:40 X vs. 37:50 X	-0.1650	-0.3090 to -0.02104	Yes
23814 24:40 X vs. 38:0 X	-2.886	-3.030 to -2.742	Yes
23815 24:40 X vs. 38:1 X	-2.442	-2.586 to -2.298	Yes
23816 24:40 X vs. 38:10 X	-1.887	-2.031 to -1.743	Yes
23817 24:40 X vs. 38:40 X	-0.3483	-0.4923 to -0.2044	Yes
23818 24:40 X vs. 38:50 X	-0.1690	-0.3130 to -0.02504	Yes
23819 24:40 X vs. 39:0 X	-3.117	-3.261 to -2.973	Yes
23820 24:40 X vs. 39:1 X	-3.008	-3.152 to -2.864	Yes
23821 24:40 X vs. 39:10 X	-2.032	-2.176 to -1.888	Yes
23822 24:40 X vs. 39:40 X	-0.3543	-0.4983 to -0.2104	Yes
23823 24:40 X vs. 39:50 X	-0.2000	-0.3440 to -0.05604	Yes
23824 24:40 X vs. 40:0 X	-3.158	-3.302 to -3.014	Yes
23825 24:40 X vs. 40:1 X	-3.084	-3.228 to -2.940	Yes
23826 24:40 X vs. 40:10 X	-2.288	-2.432 to -2.144	Yes
23827 24:40 X vs. 40:40 X	-0.3590	-0.5030 to -0.2150	Yes
23828 24:40 X vs. 40:50 X	-0.2063	-0.3503 to -0.06237	Yes
23829 24:40 X vs. 41:0 X	-3.199	-3.343 to -3.055	Yes
23830 24:40 X vs. 41:1 X	-3.198	-3.342 to -3.054	Yes
23831 24:40 X vs. 41:10 X	-2.342	-2.486 to -2.198	Yes
23832 24:40 X vs. 41:40 X	-0.3063	-0.4503 to -0.1624	Yes
23833 24:40 X vs. 41:50 X	-0.2190	-0.3630 to -0.07504	Yes
23834 24:40 X vs. 42:0 X	-3.340	-3.484 to -3.196	Yes
23835 24:40 X vs. 42:1 X	-3.360	-3.504 to -3.216	Yes
23836 24:40 X vs. 42:10 X	-2.342	-2.486 to -2.198	Yes
23837 24:40 X vs. 42:40 X	-0.3190	-0.4630 to -0.1750	Yes
23838 24:40 X vs. 42:50 X	-0.2223	-0.3663 to -0.07837	Yes
23839 24:40 X vs. 43:0 X	-3.430	-3.574 to -3.286	Yes
23840 24:40 X vs. 43:1 X	-3.345	-3.489 to -3.201	Yes
23841 24:40 X vs. 43:10 X	-3.060	-3.204 to -2.916	Yes
23842 24:40 X vs. 43:40 X	-0.3173	-0.4613 to -0.1734	Yes
23843 24:40 X vs. 43:50 X	-0.2227	-0.3666 to -0.07871	Yes
23844 24:40 X vs. 44:0 X	-3.454	-3.598 to -3.310	Yes
23845 24:40 X vs. 44:1 X	-3.344	-3.488 to -3.200	Yes
23846 24:40 X vs. 44:10 X	-3.124	-3.268 to -2.980	Yes
23847 24:40 X vs. 44:40 X	-0.3193	-0.4633 to -0.1754	Yes
23848 24:40 X vs. 44:50 X	-0.1550	-0.2990 to -0.01104	Yes
23849 24:40 X vs. 45:0 X	-3.319	-3.463 to -3.175	Yes
23850 24:40 X vs. 45:1 X	-3.121	-3.265 to -2.977	Yes

	2way ANOVA<br>Multiple comparisons	A<br>Data Set-A<br>Y	B<br>Data Set-B<br>Y	C<br>Data Set-C<br>Y
23851	24:**40 X** vs. 45:**10 X**	-3.003	-3.147 to -2.859	Yes
23852	24:**40 X** vs. 45:**40 X**	-0.3207	-0.4646 to -0.1767	Yes
23853	24:**40 X** vs. 45:**50 X**	-0.1520	-0.2960 to -0.008040	Yes
23854	24:**40 X** vs. 46:**0 X**	-3.123	-3.267 to -2.979	Yes
23855	24:**40 X** vs. 46:**1 X**	-3.090	-3.234 to -2.946	Yes
23856	24:**40 X** vs. 46:**10 X**	-2.998	-3.142 to -2.854	Yes
23857	24:**40 X** vs. 46:**40 X**	-0.3343	-0.4783 to -0.1904	Yes
23858	24:**40 X** vs. 46:**50 X**	-0.1450	-0.2890 to -0.001040	Yes
23859	24:**40 X** vs. 47:**0 X**	-3.099	-3.243 to -2.955	Yes
23860	24:**40 X** vs. 47:**1 X**	-3.003	-3.147 to -2.859	Yes
23861	24:**40 X** vs. 47:**10 X**	-2.984	-3.128 to -2.840	Yes
23862	24:**40 X** vs. 47:**40 X**	-0.3443	-0.4883 to -0.2004	Yes
23863	24:**40 X** vs. 47:**50 X**	-0.1030	-0.2470 to 0.04096	No
23864	24:**40 X** vs. 48:**0 X**	-3.086	-3.247 to -2.925	Yes
23865	24:**40 X** vs. 48:**1 X**	-2.990	-3.134 to -2.846	Yes
23866	24:**40 X** vs. 48:**10 X**	-2.984	-3.128 to -2.840	Yes
23867	24:**40 X** vs. 48:**40 X**	-0.3507	-0.4946 to -0.2067	Yes
23868	24:**40 X** vs. 48:**50 X**	-0.1103	-0.2543 to 0.03363	No
23869	24:**40 X** vs. 49:**0 X**	-3.086	-3.230 to -2.942	Yes
23870	24:**40 X** vs. 49:**1 X**	-2.990	-3.134 to -2.846	Yes
23871	24:**40 X** vs. 49:**10 X**	-2.984	-3.128 to -2.840	Yes
23872	24:**40 X** vs. 49:**40 X**	-0.3550	-0.4990 to -0.2110	Yes
23873	24:**40 X** vs. 49:**50 X**	-0.1097	-0.2536 to 0.03429	No
23874	24:**40 X** vs. 50:**0 X**	-3.086	-3.230 to -2.942	Yes
23875	24:**40 X** vs. 50:**1 X**	-2.990	-3.134 to -2.846	Yes
23876	24:**40 X** vs. 50:**10 X**	-2.984	-3.128 to -2.840	Yes
23877	24:**40 X** vs. 50:**40 X**	-0.3627	-0.5066 to -0.2187	Yes
23878	24:**40 X** vs. 50:**50 X**	-0.1093	-0.2533 to 0.03463	No
23879	24:**50 X** vs. 25:**0 X**	-0.6970	-0.8580 to -0.5360	Yes
23880	24:**50 X** vs. 25:**1 X**	-0.8865	-1.047 to -0.7255	Yes
23881	24:**50 X** vs. 25:**10 X**	-0.2480	-0.4516 to -0.04441	Yes
23882	24:**50 X** vs. 25:**40 X**	-0.08033	-0.2243 to 0.06363	No
23883	24:**50 X** vs. 25:**50 X**	-0.05467	-0.1986 to 0.08929	No
23884	24:**50 X** vs. 26:**0 X**	-0.9095	-1.070 to -0.7485	Yes
23885	24:**50 X** vs. 26:**1 X**	-1.023	-1.183 to -0.8615	Yes
23886	24:**50 X** vs. 26:**10 X**	-0.7090	-0.8700 to -0.5480	Yes
23887	24:**50 X** vs. 26:**40 X**	-0.1547	-0.2986 to -0.01071	Yes
23888	24:**50 X** vs. 26:**50 X**	-0.01500	-0.1590 to 0.1290	No
23889	24:**50 X** vs. 27:**0 X**	-1.097	-1.241 to -0.9534	Yes
23890	24:**50 X** vs. 27:**1 X**	-1.188	-1.349 to -1.027	Yes
23891	24:**50 X** vs. 27:**10 X**	-1.000	-1.161 to -0.8390	Yes
23892	24:**50 X** vs. 27:**40 X**	-0.1547	-0.2986 to -0.01071	Yes
23893	24:**50 X** vs. 27:**50 X**	-0.01433	-0.1583 to 0.1296	No
23894	24:**50 X** vs. 28:**0 X**	-1.047	-1.207 to -0.8855	Yes
23895	24:**50 X** vs. 28:**1 X**	-1.087	-1.231 to -0.9434	Yes

	2way ANOVA<br>Multiple comparisons	A<br>Data Set-A<br>Y	B<br>Data Set-B<br>Y	C<br>Data Set-C<br>Y
23896	24:**50 X** vs. 28:**10 X**	-0.8890	-1.050 to -0.7280	Yes
23897	24:**50 X** vs. 28:**40 X**	-0.1670	-0.3110 to -0.02304	Yes
23898	24:**50 X** vs. 28:**50 X**	-0.06233	-0.2063 to 0.08163	No
23899	24:**50 X** vs. 29:**0 X**	-1.528	-1.689 to -1.367	Yes
23900	24:**50 X** vs. 29:**1 X**	-1.613	-1.774 to -1.452	Yes
23901	24:**50 X** vs. 29:**10 X**	-0.8275	-0.9885 to -0.6665	Yes
23902	24:**50 X** vs. 29:**40 X**	-0.2750	-0.4190 to -0.1310	Yes
23903	24:**50 X** vs. 29:**50 X**	-0.06367	-0.2076 to 0.08029	No
23904	24:**50 X** vs. 30:**0 X**	-1.243	-1.403 to -1.082	Yes
23905	24:**50 X** vs. 30:**1 X**	-1.564	-1.725 to -1.403	Yes
23906	24:**50 X** vs. 30:**10 X**	-0.9020	-1.063 to -0.7410	Yes
23907	24:**50 X** vs. 30:**40 X**	-0.2020	-0.3460 to -0.05804	Yes
23908	24:**50 X** vs. 30:**50 X**	-0.1160	-0.2600 to 0.02796	No
23909	24:**50 X** vs. 31:**0 X**	-1.561	-1.721 to -1.400	Yes
23910	24:**50 X** vs. 31:**1 X**	-1.551	-1.712 to -1.390	Yes
23911	24:**50 X** vs. 31:**10 X**	-1.034	-1.194 to -0.8725	Yes
23912	24:**50 X** vs. 31:**40 X**	-0.2937	-0.4376 to -0.1497	Yes
23913	24:**50 X** vs. 31:**50 X**	-0.0950	-0.2390 to 0.04896	No
23914	24:**50 X** vs. 32:**0 X**	-2.152	-2.296 to -2.008	Yes
23915	24:**50 X** vs. 32:**1 X**	-1.608	-1.769 to -1.447	Yes
23916	24:**50 X** vs. 32:**10 X**	-1.464	-1.608 to -1.320	Yes
23917	24:**50 X** vs. 32:**40 X**	-0.2900	-0.4340 to -0.1460	Yes
23918	24:**50 X** vs. 32:**50 X**	-0.09467	-0.2386 to 0.04929	No
23919	24:**50 X** vs. 33:**0 X**	-2.156	-2.300 to -2.012	Yes
23920	24:**50 X** vs. 33:**1 X**	-1.970	-2.114 to -1.826	Yes
23921	24:**50 X** vs. 33:**10 X**	-1.631	-1.775 to -1.487	Yes
23922	24:**50 X** vs. 33:**40 X**	-0.3087	-0.4526 to -0.1647	Yes
23923	24:**50 X** vs. 33:**50 X**	-0.1097	-0.2536 to 0.03429	No
23924	24:**50 X** vs. 34:**0 X**	-2.080	-2.224 to -1.936	Yes
23925	24:**50 X** vs. 34:**1 X**	-2.088	-2.232 to -1.944	Yes
23926	24:**50 X** vs. 34:**10 X**	-1.721	-1.865 to -1.577	Yes
23927	24:**50 X** vs. 34:**40 X**	-0.3207	-0.4646 to -0.1767	Yes
23928	24:**50 X** vs. 34:**50 X**	-0.2587	-0.4026 to -0.1147	Yes
23929	24:**50 X** vs. 35:**0 X**	-2.429	-2.573 to -2.285	Yes
23930	24:**50 X** vs. 35:**1 X**	-2.195	-2.339 to -2.051	Yes
23931	24:**50 X** vs. 35:**10 X**	-1.683	-1.827 to -1.539	Yes
23932	24:**50 X** vs. 35:**40 X**	-0.3297	-0.4736 to -0.1857	Yes
23933	24:**50 X** vs. 35:**50 X**	-0.2713	-0.4153 to -0.1274	Yes
23934	24:**50 X** vs. 36:**0 X**	-2.636	-2.796 to -2.475	Yes
23935	24:**50 X** vs. 36:**1 X**	-2.331	-2.475 to -2.187	Yes
23936	24:**50 X** vs. 36:**10 X**	-1.688	-1.832 to -1.544	Yes
23937	24:**50 X** vs. 36:**40 X**	-0.3850	-0.5290 to -0.2410	Yes
23938	24:**50 X** vs. 36:**50 X**	-0.2347	-0.3786 to -0.09071	Yes
23939	24:**50 X** vs. 37:**0 X**	-3.019	-3.163 to -2.875	Yes
23940	24:**50 X** vs. 37:**1 X**	-2.401	-2.545 to -2.257	Yes

	2way ANOVA Multiple comparisons	A Data Set-A Y	B Data Set-B Y	C Data Set-C Y
23941	24:**50 X** vs. 37:**10 X**	-1.690	-1.834 to -1.546	Yes
23942	24:**50 X** vs. 37:**40 X**	-0.4223	-0.5663 to -0.2784	Yes
23943	24:**50 X** vs. 37:**50 X**	-0.2433	-0.3873 to -0.09937	Yes
23944	24:**50 X** vs. 38:**0 X**	-2.964	-3.108 to -2.820	Yes
23945	24:**50 X** vs. 38:**1 X**	-2.520	-2.664 to -2.376	Yes
23946	24:**50 X** vs. 38:**10 X**	-1.965	-2.109 to -1.821	Yes
23947	24:**50 X** vs. 38:**40 X**	-0.4267	-0.5706 to -0.2827	Yes
23948	24:**50 X** vs. 38:**50 X**	-0.2473	-0.3913 to -0.1034	Yes
23949	24:**50 X** vs. 39:**0 X**	-3.195	-3.339 to -3.051	Yes
23950	24:**50 X** vs. 39:**1 X**	-3.087	-3.231 to -2.943	Yes
23951	24:**50 X** vs. 39:**10 X**	-2.110	-2.254 to -1.966	Yes
23952	24:**50 X** vs. 39:**40 X**	-0.4327	-0.5766 to -0.2887	Yes
23953	24:**50 X** vs. 39:**50 X**	-0.2783	-0.4223 to -0.1344	Yes
23954	24:**50 X** vs. 40:**0 X**	-3.236	-3.380 to -3.092	Yes
23955	24:**50 X** vs. 40:**1 X**	-3.162	-3.306 to -3.018	Yes
23956	24:**50 X** vs. 40:**10 X**	-2.367	-2.511 to -2.223	Yes
23957	24:**50 X** vs. 40:**40 X**	-0.4373	-0.5813 to -0.2934	Yes
23958	24:**50 X** vs. 40:**50 X**	-0.2847	-0.4286 to -0.1407	Yes
23959	24:**50 X** vs. 41:**0 X**	-3.277	-3.421 to -3.133	Yes
23960	24:**50 X** vs. 41:**1 X**	-3.276	-3.420 to -3.132	Yes
23961	24:**50 X** vs. 41:**10 X**	-2.420	-2.564 to -2.276	Yes
23962	24:**50 X** vs. 41:**40 X**	-0.3847	-0.5286 to -0.2407	Yes
23963	24:**50 X** vs. 41:**50 X**	-0.2973	-0.4413 to -0.1534	Yes
23964	24:**50 X** vs. 42:**0 X**	-3.419	-3.563 to -3.275	Yes
23965	24:**50 X** vs. 42:**1 X**	-3.438	-3.582 to -3.294	Yes
23966	24:**50 X** vs. 42:**10 X**	-2.421	-2.565 to -2.277	Yes
23967	24:**50 X** vs. 42:**40 X**	-0.3973	-0.5413 to -0.2534	Yes
23968	24:**50 X** vs. 42:**50 X**	-0.3007	-0.4446 to -0.1567	Yes
23969	24:**50 X** vs. 43:**0 X**	-3.509	-3.653 to -3.365	Yes
23970	24:**50 X** vs. 43:**1 X**	-3.423	-3.567 to -3.279	Yes
23971	24:**50 X** vs. 43:**10 X**	-3.139	-3.283 to -2.995	Yes
23972	24:**50 X** vs. 43:**40 X**	-0.3957	-0.5396 to -0.2517	Yes
23973	24:**50 X** vs. 43:**50 X**	-0.3010	-0.4450 to -0.1570	Yes
23974	24:**50 X** vs. 44:**0 X**	-3.533	-3.677 to -3.389	Yes
23975	24:**50 X** vs. 44:**1 X**	-3.422	-3.566 to -3.278	Yes
23976	24:**50 X** vs. 44:**10 X**	-3.202	-3.346 to -3.058	Yes
23977	24:**50 X** vs. 44:**40 X**	-0.3977	-0.5416 to -0.2537	Yes
23978	24:**50 X** vs. 44:**50 X**	-0.2333	-0.3773 to -0.08937	Yes
23979	24:**50 X** vs. 45:**0 X**	-3.397	-3.541 to -3.253	Yes
23980	24:**50 X** vs. 45:**1 X**	-3.199	-3.343 to -3.055	Yes
23981	24:**50 X** vs. 45:**10 X**	-3.082	-3.226 to -2.938	Yes
23982	24:**50 X** vs. 45:**40 X**	-0.3990	-0.5430 to -0.2550	Yes
23983	24:**50 X** vs. 45:**50 X**	-0.2303	-0.3743 to -0.08637	Yes
23984	24:**50 X** vs. 46:**0 X**	-3.202	-3.346 to -3.058	Yes
23985	24:**50 X** vs. 46:**1 X**	-3.169	-3.313 to -3.025	Yes

2way ANOVA<br>Multiple comparisons	A<br>Data Set-A<br>Y	B<br>Data Set-B<br>Y	C<br>Data Set-C<br>Y
23986 24:**50 X** vs. 46:**10 X**	-3.077	-3.221 to -2.933	Yes
23987 24:**50 X** vs. 46:**40 X**	-0.4127	-0.5566 to -0.2687	Yes
23988 24:**50 X** vs. 46:**50 X**	-0.2233	-0.3673 to -0.07937	Yes
23989 24:**50 X** vs. 47:**0 X**	-3.177	-3.321 to -3.033	Yes
23990 24:**50 X** vs. 47:**1 X**	-3.081	-3.225 to -2.937	Yes
23991 24:**50 X** vs. 47:**10 X**	-3.063	-3.207 to -2.919	Yes
23992 24:**50 X** vs. 47:**40 X**	-0.4227	-0.5666 to -0.2787	Yes
23993 24:**50 X** vs. 47:**50 X**	-0.1813	-0.3253 to -0.03737	Yes
23994 24:**50 X** vs. 48:**0 X**	-3.165	-3.325 to -3.004	Yes
23995 24:**50 X** vs. 48:**1 X**	-3.068	-3.212 to -2.924	Yes
23996 24:**50 X** vs. 48:**10 X**	-3.063	-3.207 to -2.919	Yes
23997 24:**50 X** vs. 48:**40 X**	-0.4290	-0.5730 to -0.2850	Yes
23998 24:**50 X** vs. 48:**50 X**	-0.1887	-0.3326 to -0.04471	Yes
23999 24:**50 X** vs. 49:**0 X**	-3.165	-3.309 to -3.021	Yes
24000 24:**50 X** vs. 49:**1 X**	-3.068	-3.212 to -2.924	Yes
24001 24:**50 X** vs. 49:**10 X**	-3.063	-3.207 to -2.919	Yes
24002 24:**50 X** vs. 49:**40 X**	-0.4333	-0.5773 to -0.2894	Yes
24003 24:**50 X** vs. 49:**50 X**	-0.1880	-0.3320 to -0.04404	Yes
24004 24:**50 X** vs. 50:**0 X**	-3.165	-3.309 to -3.021	Yes
24005 24:**50 X** vs. 50:**1 X**	-3.068	-3.212 to -2.924	Yes
24006 24:**50 X** vs. 50:**10 X**	-3.063	-3.207 to -2.919	Yes
24007 24:**50 X** vs. 50:**40 X**	-0.4410	-0.5850 to -0.2970	Yes
24008 24:**50 X** vs. 50:**50 X**	-0.1877	-0.3316 to -0.04371	Yes
24009 25:**0 X** vs. 25:**1 X**	-0.1895	-0.3658 to -0.01319	Yes
24010 25:**0 X** vs. 25:**10 X**	0.4490	0.2331 to 0.6649	Yes
24011 25:**0 X** vs. 25:**40 X**	0.6167	0.4557 to 0.7776	Yes
24012 25:**0 X** vs. 25:**50 X**	0.6423	0.4814 to 0.8033	Yes
24013 25:**0 X** vs. 26:**0 X**	-0.2125	-0.3888 to -0.03619	Yes
24014 25:**0 X** vs. 26:**1 X**	-0.3255	-0.5018 to -0.1492	Yes
24015 25:**0 X** vs. 26:**10 X**	-0.01200	-0.1883 to 0.1643	No
24016 25:**0 X** vs. 26:**40 X**	0.5423	0.3814 to 0.7033	Yes
24017 25:**0 X** vs. 26:**50 X**	0.6820	0.5210 to 0.8430	Yes
24018 25:**0 X** vs. 27:**0 X**	-0.4003	-0.5613 to -0.2394	Yes
24019 25:**0 X** vs. 27:**1 X**	-0.4910	-0.6673 to -0.3147	Yes
24020 25:**0 X** vs. 27:**10 X**	-0.3030	-0.4793 to -0.1267	Yes
24021 25:**0 X** vs. 27:**40 X**	0.5423	0.3814 to 0.7033	Yes
24022 25:**0 X** vs. 27:**50 X**	0.6827	0.5217 to 0.8436	Yes
24023 25:**0 X** vs. 28:**0 X**	-0.3495	-0.5258 to -0.1732	Yes
24024 25:**0 X** vs. 28:**1 X**	-0.3903	-0.5513 to -0.2294	Yes
24025 25:**0 X** vs. 28:**10 X**	-0.1920	-0.3683 to -0.01569	Yes
24026 25:**0 X** vs. 28:**40 X**	0.5300	0.3690 to 0.6910	Yes
24027 25:**0 X** vs. 28:**50 X**	0.6347	0.4737 to 0.7956	Yes
24028 25:**0 X** vs. 29:**0 X**	-0.8310	-1.007 to -0.6547	Yes
24029 25:**0 X** vs. 29:**1 X**	-0.9160	-1.092 to -0.7397	Yes
24030 25:**0 X** vs. 29:**10 X**	-0.1305	-0.3068 to 0.04581	No

2way ANOVA Multiple comparisons	A Data Set-A Y	B Data Set-B Y	C Data Set-C Y
24031 25:**0 X** vs. 29:**40 X**	0.4220	0.2610 to 0.5830	Yes
24032 25:**0 X** vs. 29:**50 X**	0.6333	0.4724 to 0.7943	Yes
24033 25:**0 X** vs. 30:**0 X**	-0.5455	-0.7218 to -0.3692	Yes
24034 25:**0 X** vs. 30:**1 X**	-0.8670	-1.043 to -0.6907	Yes
24035 25:**0 X** vs. 30:**10 X**	-0.2050	-0.3813 to -0.02869	Yes
24036 25:**0 X** vs. 30:**40 X**	0.4950	0.3340 to 0.6560	Yes
24037 25:**0 X** vs. 30:**50 X**	0.5810	0.4200 to 0.7420	Yes
24038 25:**0 X** vs. 31:**0 X**	-0.8635	-1.040 to -0.6872	Yes
24039 25:**0 X** vs. 31:**1 X**	-0.8540	-1.030 to -0.6777	Yes
24040 25:**0 X** vs. 31:**10 X**	-0.3365	-0.5128 to -0.1602	Yes
24041 25:**0 X** vs. 31:**40 X**	0.4033	0.2424 to 0.5643	Yes
24042 25:**0 X** vs. 31:**50 X**	0.6020	0.4410 to 0.7630	Yes
24043 25:**0 X** vs. 32:**0 X**	-1.455	-1.616 to -1.294	Yes
24044 25:**0 X** vs. 32:**1 X**	-0.9110	-1.087 to -0.7347	Yes
24045 25:**0 X** vs. 32:**10 X**	-0.7670	-0.9280 to -0.6060	Yes
24046 25:**0 X** vs. 32:**40 X**	0.4070	0.2460 to 0.5680	Yes
24047 25:**0 X** vs. 32:**50 X**	0.6023	0.4414 to 0.7633	Yes
24048 25:**0 X** vs. 33:**0 X**	-1.459	-1.620 to -1.298	Yes
24049 25:**0 X** vs. 33:**1 X**	-1.273	-1.434 to -1.112	Yes
24050 25:**0 X** vs. 33:**10 X**	-0.9337	-1.095 to -0.7727	Yes
24051 25:**0 X** vs. 33:**40 X**	0.3883	0.2274 to 0.5493	Yes
24052 25:**0 X** vs. 33:**50 X**	0.5873	0.4264 to 0.7483	Yes
24053 25:**0 X** vs. 34:**0 X**	-1.383	-1.544 to -1.222	Yes
24054 25:**0 X** vs. 34:**1 X**	-1.391	-1.552 to -1.230	Yes
24055 25:**0 X** vs. 34:**10 X**	-1.024	-1.185 to -0.8630	Yes
24056 25:**0 X** vs. 34:**40 X**	0.3763	0.2154 to 0.5373	Yes
24057 25:**0 X** vs. 34:**50 X**	0.4383	0.2774 to 0.5993	Yes
24058 25:**0 X** vs. 35:**0 X**	-1.732	-1.893 to -1.571	Yes
24059 25:**0 X** vs. 35:**1 X**	-1.498	-1.659 to -1.337	Yes
24060 25:**0 X** vs. 35:**10 X**	-0.9857	-1.147 to -0.8247	Yes
24061 25:**0 X** vs. 35:**40 X**	0.3673	0.2064 to 0.5283	Yes
24062 25:**0 X** vs. 35:**50 X**	0.4257	0.2647 to 0.5866	Yes
24063 25:**0 X** vs. 36:**0 X**	-1.939	-2.115 to -1.762	Yes
24064 25:**0 X** vs. 36:**1 X**	-1.634	-1.795 to -1.473	Yes
24065 25:**0 X** vs. 36:**10 X**	-0.9913	-1.152 to -0.8304	Yes
24066 25:**0 X** vs. 36:**40 X**	0.3120	0.1510 to 0.4730	Yes
24067 25:**0 X** vs. 36:**50 X**	0.4623	0.3014 to 0.6233	Yes
24068 25:**0 X** vs. 37:**0 X**	-2.322	-2.483 to -2.161	Yes
24069 25:**0 X** vs. 37:**1 X**	-1.704	-1.865 to -1.543	Yes
24070 25:**0 X** vs. 37:**10 X**	-0.9930	-1.154 to -0.8320	Yes
24071 25:**0 X** vs. 37:**40 X**	0.2747	0.1137 to 0.4356	Yes
24072 25:**0 X** vs. 37:**50 X**	0.4537	0.2927 to 0.6146	Yes
24073 25:**0 X** vs. 38:**0 X**	-2.267	-2.428 to -2.106	Yes
24074 25:**0 X** vs. 38:**1 X**	-1.823	-1.984 to -1.662	Yes
24075 25:**0 X** vs. 38:**10 X**	-1.268	-1.429 to -1.107	Yes

	2way ANOVA<br>Multiple comparisons	A<br>Data Set-A<br>Y	B<br>Data Set-B<br>Y	C<br>Data Set-C<br>Y
24076	25:**0 X** vs. 38:**40 X**	0.2703	0.1094 to 0.4313	Yes
24077	25:**0 X** vs. 38:**50 X**	0.4497	0.2887 to 0.6106	Yes
24078	25:**0 X** vs. 39:**0 X**	-2.498	-2.659 to -2.337	Yes
24079	25:**0 X** vs. 39:**1 X**	-2.390	-2.551 to -2.229	Yes
24080	25:**0 X** vs. 39:**10 X**	-1.413	-1.574 to -1.252	Yes
24081	25:**0 X** vs. 39:**40 X**	0.2643	0.1034 to 0.4253	Yes
24082	25:**0 X** vs. 39:**50 X**	0.4187	0.2577 to 0.5796	Yes
24083	25:**0 X** vs. 40:**0 X**	-2.539	-2.700 to -2.378	Yes
24084	25:**0 X** vs. 40:**1 X**	-2.465	-2.626 to -2.304	Yes
24085	25:**0 X** vs. 40:**10 X**	-1.670	-1.831 to -1.509	Yes
24086	25:**0 X** vs. 40:**40 X**	0.2597	0.09871 to 0.4206	Yes
24087	25:**0 X** vs. 40:**50 X**	0.4123	0.2514 to 0.5733	Yes
24088	25:**0 X** vs. 41:**0 X**	-2.580	-2.741 to -2.419	Yes
24089	25:**0 X** vs. 41:**1 X**	-2.579	-2.740 to -2.418	Yes
24090	25:**0 X** vs. 41:**10 X**	-1.723	-1.884 to -1.562	Yes
24091	25:**0 X** vs. 41:**40 X**	0.3123	0.1514 to 0.4733	Yes
24092	25:**0 X** vs. 41:**50 X**	0.3997	0.2387 to 0.5606	Yes
24093	25:**0 X** vs. 42:**0 X**	-2.722	-2.883 to -2.561	Yes
24094	25:**0 X** vs. 42:**1 X**	-2.741	-2.902 to -2.580	Yes
24095	25:**0 X** vs. 42:**10 X**	-1.724	-1.885 to -1.563	Yes
24096	25:**0 X** vs. 42:**40 X**	0.2997	0.1387 to 0.4606	Yes
24097	25:**0 X** vs. 42:**50 X**	0.3963	0.2354 to 0.5573	Yes
24098	25:**0 X** vs. 43:**0 X**	-2.812	-2.973 to -2.651	Yes
24099	25:**0 X** vs. 43:**1 X**	-2.726	-2.887 to -2.565	Yes
24100	25:**0 X** vs. 43:**10 X**	-2.442	-2.603 to -2.281	Yes
24101	25:**0 X** vs. 43:**40 X**	0.3013	0.1404 to 0.4623	Yes
24102	25:**0 X** vs. 43:**50 X**	0.3960	0.2350 to 0.5570	Yes
24103	25:**0 X** vs. 44:**0 X**	-2.836	-2.997 to -2.675	Yes
24104	25:**0 X** vs. 44:**1 X**	-2.725	-2.886 to -2.564	Yes
24105	25:**0 X** vs. 44:**10 X**	-2.505	-2.666 to -2.344	Yes
24106	25:**0 X** vs. 44:**40 X**	0.2993	0.1384 to 0.4603	Yes
24107	25:**0 X** vs. 44:**50 X**	0.4637	0.3027 to 0.6246	Yes
24108	25:**0 X** vs. 45:**0 X**	-2.700	-2.861 to -2.539	Yes
24109	25:**0 X** vs. 45:**1 X**	-2.502	-2.663 to -2.341	Yes
24110	25:**0 X** vs. 45:**10 X**	-2.385	-2.546 to -2.224	Yes
24111	25:**0 X** vs. 45:**40 X**	0.2980	0.1370 to 0.4590	Yes
24112	25:**0 X** vs. 45:**50 X**	0.4667	0.3057 to 0.6276	Yes
24113	25:**0 X** vs. 46:**0 X**	-2.505	-2.666 to -2.344	Yes
24114	25:**0 X** vs. 46:**1 X**	-2.472	-2.633 to -2.311	Yes
24115	25:**0 X** vs. 46:**10 X**	-2.380	-2.541 to -2.219	Yes
24116	25:**0 X** vs. 46:**40 X**	0.2843	0.1234 to 0.4453	Yes
24117	25:**0 X** vs. 46:**50 X**	0.4737	0.3127 to 0.6346	Yes
24118	25:**0 X** vs. 47:**0 X**	-2.480	-2.641 to -2.319	Yes
24119	25:**0 X** vs. 47:**1 X**	-2.384	-2.545 to -2.223	Yes
24120	25:**0 X** vs. 47:**10 X**	-2.366	-2.527 to -2.205	Yes

2way ANOVA<br>Multiple comparisons	A<br>Data Set-A<br>Y	B<br>Data Set-B<br>Y	C<br>Data Set-C<br>Y
24121 25:0 X vs. 47:40 X	0.2743	0.1134 to 0.4353	Yes
24122 25:0 X vs. 47:50 X	0.5157	0.3547 to 0.6766	Yes
24123 25:0 X vs. 48:0 X	-2.468	-2.644 to -2.291	Yes
24124 25:0 X vs. 48:1 X	-2.371	-2.532 to -2.210	Yes
24125 25:0 X vs. 48:10 X	-2.366	-2.527 to -2.205	Yes
24126 25:0 X vs. 48:40 X	0.2680	0.1070 to 0.4290	Yes
24127 25:0 X vs. 48:50 X	0.5083	0.3474 to 0.6693	Yes
24128 25:0 X vs. 49:0 X	-2.468	-2.629 to -2.307	Yes
24129 25:0 X vs. 49:1 X	-2.371	-2.532 to -2.210	Yes
24130 25:0 X vs. 49:10 X	-2.366	-2.527 to -2.205	Yes
24131 25:0 X vs. 49:40 X	0.2637	0.1027 to 0.4246	Yes
24132 25:0 X vs. 49:50 X	0.5090	0.3480 to 0.6700	Yes
24133 25:0 X vs. 50:0 X	-2.468	-2.629 to -2.307	Yes
24134 25:0 X vs. 50:1 X	-2.371	-2.532 to -2.210	Yes
24135 25:0 X vs. 50:10 X	-2.366	-2.527 to -2.205	Yes
24136 25:0 X vs. 50:40 X	0.2560	0.09505 to 0.4170	Yes
24137 25:0 X vs. 50:50 X	0.5093	0.3484 to 0.6703	Yes
24138 25:1 X vs. 25:10 X	0.6385	0.4226 to 0.8544	Yes
24139 25:1 X vs. 25:40 X	0.8062	0.6452 to 0.9671	Yes
24140 25:1 X vs. 25:50 X	0.8318	0.6709 to 0.9928	Yes
24141 25:1 X vs. 26:0 X	-0.0230	-0.1993 to 0.1533	No
24142 25:1 X vs. 26:1 X	-0.1360	-0.3123 to 0.04031	No
24143 25:1 X vs. 26:10 X	0.1775	0.001186 to 0.3538	Yes
24144 25:1 X vs. 26:40 X	0.7318	0.5709 to 0.8928	Yes
24145 25:1 X vs. 26:50 X	0.8715	0.7105 to 1.032	Yes
24146 25:1 X vs. 27:0 X	-0.2108	-0.3718 to -0.04988	Yes
24147 25:1 X vs. 27:1 X	-0.3015	-0.4778 to -0.1252	Yes
24148 25:1 X vs. 27:10 X	-0.1135	-0.2898 to 0.06281	No
24149 25:1 X vs. 27:40 X	0.7318	0.5709 to 0.8928	Yes
24150 25:1 X vs. 27:50 X	0.8722	0.7112 to 1.033	Yes
24151 25:1 X vs. 28:0 X	-0.1600	-0.3363 to 0.01631	No
24152 25:1 X vs. 28:1 X	-0.2008	-0.3618 to -0.03988	Yes
24153 25:1 X vs. 28:10 X	-0.002500	-0.1788 to 0.1738	No
24154 25:1 X vs. 28:40 X	0.7195	0.5585 to 0.8805	Yes
24155 25:1 X vs. 28:50 X	0.8242	0.6632 to 0.9851	Yes
24156 25:1 X vs. 29:0 X	-0.6415	-0.8178 to -0.4652	Yes
24157 25:1 X vs. 29:1 X	-0.7265	-0.9028 to -0.5502	Yes
24158 25:1 X vs. 29:10 X	0.05900	-0.1173 to 0.2353	No
24159 25:1 X vs. 29:40 X	0.6115	0.4505 to 0.7725	Yes
24160 25:1 X vs. 29:50 X	0.8228	0.6619 to 0.9838	Yes
24161 25:1 X vs. 30:0 X	-0.3560	-0.5323 to -0.1797	Yes
24162 25:1 X vs. 30:1 X	-0.6775	-0.8538 to -0.5012	Yes
24163 25:1 X vs. 30:10 X	-0.01550	-0.1918 to 0.1608	No
24164 25:1 X vs. 30:40 X	0.6845	0.5235 to 0.8455	Yes
24165 25:1 X vs. 30:50 X	0.7705	0.6095 to 0.9315	Yes

	2way ANOVA<br>Multiple comparisons	A<br>Data Set-A<br>Y	B<br>Data Set-B<br>Y	C<br>Data Set-C<br>Y
24166	25:1 X vs. 31:0 X	-0.6740	-0.8503 to -0.4977	Yes
24167	25:1 X vs. 31:1 X	-0.6645	-0.8408 to -0.4882	Yes
24168	25:1 X vs. 31:10 X	-0.1470	-0.3233 to 0.02931	No
24169	25:1 X vs. 31:40 X	0.5928	0.4319 to 0.7538	Yes
24170	25:1 X vs. 31:50 X	0.7915	0.6305 to 0.9525	Yes
24171	25:1 X vs. 32:0 X	-1.266	-1.426 to -1.105	Yes
24172	25:1 X vs. 32:1 X	-0.7215	-0.8978 to -0.5452	Yes
24173	25:1 X vs. 32:10 X	-0.5775	-0.7385 to -0.4165	Yes
24174	25:1 X vs. 32:40 X	0.5965	0.4355 to 0.7575	Yes
24175	25:1 X vs. 32:50 X	0.7918	0.6309 to 0.9528	Yes
24176	25:1 X vs. 33:0 X	-1.269	-1.430 to -1.108	Yes
24177	25:1 X vs. 33:1 X	-1.084	-1.245 to -0.9229	Yes
24178	25:1 X vs. 33:10 X	-0.7442	-0.9051 to -0.5832	Yes
24179	25:1 X vs. 33:40 X	0.5778	0.4169 to 0.7388	Yes
24180	25:1 X vs. 33:50 X	0.7768	0.6159 to 0.9378	Yes
24181	25:1 X vs. 34:0 X	-1.194	-1.355 to -1.033	Yes
24182	25:1 X vs. 34:1 X	-1.201	-1.362 to -1.040	Yes
24183	25:1 X vs. 34:10 X	-0.8345	-0.9955 to -0.6735	Yes
24184	25:1 X vs. 34:40 X	0.5658	0.4049 to 0.7268	Yes
24185	25:1 X vs. 34:50 X	0.6278	0.4669 to 0.7888	Yes
24186	25:1 X vs. 35:0 X	-1.543	-1.703 to -1.382	Yes
24187	25:1 X vs. 35:1 X	-1.308	-1.469 to -1.147	Yes
24188	25:1 X vs. 35:10 X	-0.7962	-0.9571 to -0.6352	Yes
24189	25:1 X vs. 35:40 X	0.5568	0.3959 to 0.7178	Yes
24190	25:1 X vs. 35:50 X	0.6152	0.4542 to 0.7761	Yes
24191	25:1 X vs. 36:0 X	-1.749	-1.925 to -1.573	Yes
24192	25:1 X vs. 36:1 X	-1.445	-1.606 to -1.284	Yes
24193	25:1 X vs. 36:10 X	-0.8018	-0.9628 to -0.6409	Yes
24194	25:1 X vs. 36:40 X	0.5015	0.3405 to 0.6625	Yes
24195	25:1 X vs. 36:50 X	0.6518	0.4909 to 0.8128	Yes
24196	25:1 X vs. 37:0 X	-2.133	-2.294 to -1.972	Yes
24197	25:1 X vs. 37:1 X	-1.515	-1.676 to -1.354	Yes
24198	25:1 X vs. 37:10 X	-0.8035	-0.9645 to -0.6425	Yes
24199	25:1 X vs. 37:40 X	0.4642	0.3032 to 0.6251	Yes
24200	25:1 X vs. 37:50 X	0.6432	0.4822 to 0.8041	Yes
24201	25:1 X vs. 38:0 X	-2.078	-2.239 to -1.917	Yes
24202	25:1 X vs. 38:1 X	-1.634	-1.794 to -1.473	Yes
24203	25:1 X vs. 38:10 X	-1.079	-1.240 to -0.9179	Yes
24204	25:1 X vs. 38:40 X	0.4598	0.2989 to 0.6208	Yes
24205	25:1 X vs. 38:50 X	0.6392	0.4782 to 0.8001	Yes
24206	25:1 X vs. 39:0 X	-2.309	-2.469 to -2.148	Yes
24207	25:1 X vs. 39:1 X	-2.200	-2.361 to -2.039	Yes
24208	25:1 X vs. 39:10 X	-1.224	-1.385 to -1.063	Yes
24209	25:1 X vs. 39:40 X	0.4538	0.2929 to 0.6148	Yes
24210	25:1 X vs. 39:50 X	0.6082	0.4472 to 0.7691	Yes

	2way ANOVA<br>Multiple comparisons	A<br>Data Set-A<br>Y	B<br>Data Set-B<br>Y	C<br>Data Set-C<br>Y
24211	25:**1 X** vs. 40:**0 X**	-2.350	-2.510 to -2.189	Yes
24212	25:**1 X** vs. 40:**1 X**	-2.276	-2.436 to -2.115	Yes
24213	25:**1 X** vs. 40:**10 X**	-1.480	-1.641 to -1.319	Yes
24214	25:**1 X** vs. 40:**40 X**	0.4492	0.2882 to 0.6101	Yes
24215	25:**1 X** vs. 40:**50 X**	0.6018	0.4409 to 0.7628	Yes
24216	25:**1 X** vs. 41:**0 X**	-2.391	-2.551 to -2.230	Yes
24217	25:**1 X** vs. 41:**1 X**	-2.390	-2.550 to -2.229	Yes
24218	25:**1 X** vs. 41:**10 X**	-1.534	-1.695 to -1.373	Yes
24219	25:**1 X** vs. 41:**40 X**	0.5018	0.3409 to 0.6628	Yes
24220	25:**1 X** vs. 41:**50 X**	0.5892	0.4282 to 0.7501	Yes
24221	25:**1 X** vs. 42:**0 X**	-2.532	-2.693 to -2.371	Yes
24222	25:**1 X** vs. 42:**1 X**	-2.552	-2.713 to -2.391	Yes
24223	25:**1 X** vs. 42:**10 X**	-1.534	-1.695 to -1.373	Yes
24224	25:**1 X** vs. 42:**40 X**	0.4892	0.3282 to 0.6501	Yes
24225	25:**1 X** vs. 42:**50 X**	0.5858	0.4249 to 0.7468	Yes
24226	25:**1 X** vs. 43:**0 X**	-2.622	-2.783 to -2.461	Yes
24227	25:**1 X** vs. 43:**1 X**	-2.537	-2.697 to -2.376	Yes
24228	25:**1 X** vs. 43:**10 X**	-2.252	-2.413 to -2.091	Yes
24229	25:**1 X** vs. 43:**40 X**	0.4908	0.3299 to 0.6518	Yes
24230	25:**1 X** vs. 43:**50 X**	0.5855	0.4245 to 0.7465	Yes
24231	25:**1 X** vs. 44:**0 X**	-2.646	-2.807 to -2.485	Yes
24232	25:**1 X** vs. 44:**1 X**	-2.536	-2.697 to -2.375	Yes
24233	25:**1 X** vs. 44:**10 X**	-2.316	-2.476 to -2.155	Yes
24234	25:**1 X** vs. 44:**40 X**	0.4888	0.3279 to 0.6498	Yes
24235	25:**1 X** vs. 44:**50 X**	0.6532	0.4922 to 0.8141	Yes
24236	25:**1 X** vs. 45:**0 X**	-2.511	-2.672 to -2.350	Yes
24237	25:**1 X** vs. 45:**1 X**	-2.313	-2.474 to -2.152	Yes
24238	25:**1 X** vs. 45:**10 X**	-2.195	-2.356 to -2.034	Yes
24239	25:**1 X** vs. 45:**40 X**	0.4875	0.3265 to 0.6485	Yes
24240	25:**1 X** vs. 45:**50 X**	0.6562	0.4952 to 0.8171	Yes
24241	25:**1 X** vs. 46:**0 X**	-2.315	-2.476 to -2.154	Yes
24242	25:**1 X** vs. 46:**1 X**	-2.282	-2.443 to -2.121	Yes
24243	25:**1 X** vs. 46:**10 X**	-2.190	-2.351 to -2.029	Yes
24244	25:**1 X** vs. 46:**40 X**	0.4738	0.3129 to 0.6348	Yes
24245	25:**1 X** vs. 46:**50 X**	0.6632	0.5022 to 0.8241	Yes
24246	25:**1 X** vs. 47:**0 X**	-2.291	-2.452 to -2.130	Yes
24247	25:**1 X** vs. 47:**1 X**	-2.195	-2.356 to -2.034	Yes
24248	25:**1 X** vs. 47:**10 X**	-2.176	-2.337 to -2.015	Yes
24249	25:**1 X** vs. 47:**40 X**	0.4638	0.3029 to 0.6248	Yes
24250	25:**1 X** vs. 47:**50 X**	0.7052	0.5442 to 0.8661	Yes
24251	25:**1 X** vs. 48:**0 X**	-2.278	-2.454 to -2.102	Yes
24252	25:**1 X** vs. 48:**1 X**	-2.182	-2.343 to -2.021	Yes
24253	25:**1 X** vs. 48:**10 X**	-2.176	-2.337 to -2.015	Yes
24254	25:**1 X** vs. 48:**40 X**	0.4575	0.2965 to 0.6185	Yes
24255	25:**1 X** vs. 48:**50 X**	0.6978	0.5369 to 0.8588	Yes

	2way ANOVA Multiple comparisons	A Data Set-A Y	B Data Set-B Y	C Data Set-C Y
24256	25:**1 X** vs. 49:**0 X**	-2.278	-2.439 to -2.117	Yes
24257	25:**1 X** vs. 49:**1 X**	-2.182	-2.343 to -2.021	Yes
24258	25:**1 X** vs. 49:**10 X**	-2.176	-2.337 to -2.015	Yes
24259	25:**1 X** vs. 49:**40 X**	0.4532	0.2922 to 0.6141	Yes
24260	25:**1 X** vs. 49:**50 X**	0.6985	0.5375 to 0.8595	Yes
24261	25:**1 X** vs. 50:**0 X**	-2.278	-2.439 to -2.117	Yes
24262	25:**1 X** vs. 50:**1 X**	-2.182	-2.343 to -2.021	Yes
24263	25:**1 X** vs. 50:**10 X**	-2.176	-2.337 to -2.015	Yes
24264	25:**1 X** vs. 50:**40 X**	0.4455	0.2845 to 0.6065	Yes
24265	25:**1 X** vs. 50:**50 X**	0.6988	0.5379 to 0.8598	Yes
24266	25:**10 X** vs. 25:**40 X**	0.1677	-0.03592 to 0.3713	No
24267	25:**10 X** vs. 25:**50 X**	0.1933	-0.01026 to 0.3969	No
24268	25:**10 X** vs. 26:**0 X**	-0.6615	-0.8774 to -0.4456	Yes
24269	25:**10 X** vs. 26:**1 X**	-0.7745	-0.9904 to -0.5586	Yes
24270	25:**10 X** vs. 26:**10 X**	-0.4610	-0.6769 to -0.2451	Yes
24271	25:**10 X** vs. 26:**40 X**	0.09333	-0.1103 to 0.2969	No
24272	25:**10 X** vs. 26:**50 X**	0.2330	0.02941 to 0.4366	Yes
24273	25:**10 X** vs. 27:**0 X**	-0.8493	-1.053 to -0.6457	Yes
24274	25:**10 X** vs. 27:**1 X**	-0.9400	-1.156 to -0.7241	Yes
24275	25:**10 X** vs. 27:**10 X**	-0.7520	-0.9679 to -0.5361	Yes
24276	25:**10 X** vs. 27:**40 X**	0.09333	-0.1103 to 0.2969	No
24277	25:**10 X** vs. 27:**50 X**	0.2337	0.03008 to 0.4373	Yes
24278	25:**10 X** vs. 28:**0 X**	-0.7985	-1.014 to -0.5826	Yes
24279	25:**10 X** vs. 28:**1 X**	-0.8393	-1.043 to -0.6357	Yes
24280	25:**10 X** vs. 28:**10 X**	-0.6410	-0.8569 to -0.4251	Yes
24281	25:**10 X** vs. 28:**40 X**	0.08100	-0.1226 to 0.2846	No
24282	25:**10 X** vs. 28:**50 X**	0.1857	-0.01792 to 0.3893	No
24283	25:**10 X** vs. 29:**0 X**	-1.280	-1.496 to -1.064	Yes
24284	25:**10 X** vs. 29:**1 X**	-1.365	-1.581 to -1.149	Yes
24285	25:**10 X** vs. 29:**10 X**	-0.5795	-0.7954 to -0.3636	Yes
24286	25:**10 X** vs. 29:**40 X**	-0.02700	-0.2306 to 0.1766	No
24287	25:**10 X** vs. 29:**50 X**	0.1843	-0.01926 to 0.3879	No
24288	25:**10 X** vs. 30:**0 X**	-0.9945	-1.210 to -0.7786	Yes
24289	25:**10 X** vs. 30:**1 X**	-1.316	-1.532 to -1.100	Yes
24290	25:**10 X** vs. 30:**10 X**	-0.6540	-0.8699 to -0.4381	Yes
24291	25:**10 X** vs. 30:**40 X**	0.0460	-0.1576 to 0.2496	No
24292	25:**10 X** vs. 30:**50 X**	0.1320	-0.07159 to 0.3356	No
24293	25:**10 X** vs. 31:**0 X**	-1.313	-1.528 to -1.097	Yes
24294	25:**10 X** vs. 31:**1 X**	-1.303	-1.519 to -1.087	Yes
24295	25:**10 X** vs. 31:**10 X**	-0.7855	-1.001 to -0.5696	Yes
24296	25:**10 X** vs. 31:**40 X**	-0.04567	-0.2493 to 0.1579	No
24297	25:**10 X** vs. 31:**50 X**	0.1530	-0.05059 to 0.3566	No
24298	25:**10 X** vs. 32:**0 X**	-1.904	-2.108 to -1.700	Yes
24299	25:**10 X** vs. 32:**1 X**	-1.360	-1.576 to -1.144	Yes
24300	25:**10 X** vs. 32:**10 X**	-1.216	-1.420 to -1.012	Yes

	2way ANOVA<br>Multiple comparisons	A<br>Data Set-A<br>Y	B<br>Data Set-B<br>Y	C<br>Data Set-C<br>Y
24301	25:**10 X** vs. 32:**40 X**	-0.0420	-0.2456 to 0.1616	No
24302	25:**10 X** vs. 32:**50 X**	0.1533	-0.05026 to 0.3569	No
24303	25:**10 X** vs. 33:**0 X**	-1.908	-2.111 to -1.704	Yes
24304	25:**10 X** vs. 33:**1 X**	-1.722	-1.926 to -1.519	Yes
24305	25:**10 X** vs. 33:**10 X**	-1.383	-1.586 to -1.179	Yes
24306	25:**10 X** vs. 33:**40 X**	-0.06067	-0.2643 to 0.1429	No
24307	25:**10 X** vs. 33:**50 X**	0.1383	-0.06526 to 0.3419	No
24308	25:**10 X** vs. 34:**0 X**	-1.832	-2.036 to -1.629	Yes
24309	25:**10 X** vs. 34:**1 X**	-1.840	-2.043 to -1.636	Yes
24310	25:**10 X** vs. 34:**10 X**	-1.473	-1.677 to -1.269	Yes
24311	25:**10 X** vs. 34:**40 X**	-0.07267	-0.2763 to 0.1309	No
24312	25:**10 X** vs. 34:**50 X**	-0.01067	-0.2143 to 0.1929	No
24313	25:**10 X** vs. 35:**0 X**	-2.181	-2.385 to -1.977	Yes
24314	25:**10 X** vs. 35:**1 X**	-1.947	-2.150 to -1.743	Yes
24315	25:**10 X** vs. 35:**10 X**	-1.435	-1.638 to -1.231	Yes
24316	25:**10 X** vs. 35:**40 X**	-0.08167	-0.2853 to 0.1219	No
24317	25:**10 X** vs. 35:**50 X**	-0.02333	-0.2269 to 0.1803	No
24318	25:**10 X** vs. 36:**0 X**	-2.388	-2.603 to -2.172	Yes
24319	25:**10 X** vs. 36:**1 X**	-2.083	-2.287 to -1.880	Yes
24320	25:**10 X** vs. 36:**10 X**	-1.440	-1.644 to -1.237	Yes
24321	25:**10 X** vs. 36:**40 X**	-0.1370	-0.3406 to 0.06659	No
24322	25:**10 X** vs. 36:**50 X**	0.01333	-0.1903 to 0.2169	No
24323	25:**10 X** vs. 37:**0 X**	-2.771	-2.975 to -2.568	Yes
24324	25:**10 X** vs. 37:**1 X**	-2.153	-2.357 to -1.950	Yes
24325	25:**10 X** vs. 37:**10 X**	-1.442	-1.646 to -1.238	Yes
24326	25:**10 X** vs. 37:**40 X**	-0.1743	-0.3779 to 0.02926	No
24327	25:**10 X** vs. 37:**50 X**	0.004667	-0.1989 to 0.2083	No
24328	25:**10 X** vs. 38:**0 X**	-2.716	-2.920 to -2.513	Yes
24329	25:**10 X** vs. 38:**1 X**	-2.272	-2.476 to -2.068	Yes
24330	25:**10 X** vs. 38:**10 X**	-1.717	-1.921 to -1.514	Yes
24331	25:**10 X** vs. 38:**40 X**	-0.1787	-0.3823 to 0.02492	No
24332	25:**10 X** vs. 38:**50 X**	0.0006667	-0.2029 to 0.2043	No
24333	25:**10 X** vs. 39:**0 X**	-2.947	-3.151 to -2.743	Yes
24334	25:**10 X** vs. 39:**1 X**	-2.839	-3.042 to -2.635	Yes
24335	25:**10 X** vs. 39:**10 X**	-1.862	-2.066 to -1.659	Yes
24336	25:**10 X** vs. 39:**40 X**	-0.1847	-0.3883 to 0.01892	No
24337	25:**10 X** vs. 39:**50 X**	-0.03033	-0.2339 to 0.1733	No
24338	25:**10 X** vs. 40:**0 X**	-2.988	-3.192 to -2.784	Yes
24339	25:**10 X** vs. 40:**1 X**	-2.914	-3.118 to -2.710	Yes
24340	25:**10 X** vs. 40:**10 X**	-2.119	-2.322 to -1.915	Yes
24341	25:**10 X** vs. 40:**40 X**	-0.1893	-0.3929 to 0.01426	No
24342	25:**10 X** vs. 40:**50 X**	-0.03667	-0.2403 to 0.1669	No
24343	25:**10 X** vs. 41:**0 X**	-3.029	-3.233 to -2.825	Yes
24344	25:**10 X** vs. 41:**1 X**	-3.028	-3.232 to -2.824	Yes
24345	25:**10 X** vs. 41:**10 X**	-2.172	-2.376 to -1.969	Yes

2way ANOVA<br>Multiple comparisons		A<br>Data Set-A<br>Y	B<br>Data Set-B<br>Y	C<br>Data Set-C<br>Y
24346	25:10 X vs. 41:40 X	-0.1367	-0.3403 to 0.06692	No
24347	25:10 X vs. 41:50 X	-0.04933	-0.2529 to 0.1543	No
24348	25:10 X vs. 42:0 X	-3.171	-3.374 to -2.967	Yes
24349	25:10 X vs. 42:1 X	-3.190	-3.394 to -2.987	Yes
24350	25:10 X vs. 42:10 X	-2.173	-2.376 to -1.969	Yes
24351	25:10 X vs. 42:40 X	-0.1493	-0.3529 to 0.05426	No
24352	25:10 X vs. 42:50 X	-0.05267	-0.2563 to 0.1509	No
24353	25:10 X vs. 43:0 X	-3.261	-3.464 to -3.057	Yes
24354	25:10 X vs. 43:1 X	-3.175	-3.379 to -2.971	Yes
24355	25:10 X vs. 43:10 X	-2.891	-3.094 to -2.687	Yes
24356	25:10 X vs. 43:40 X	-0.1477	-0.3513 to 0.05592	No
24357	25:10 X vs. 43:50 X	-0.05300	-0.2566 to 0.1506	No
24358	25:10 X vs. 44:0 X	-3.285	-3.488 to -3.081	Yes
24359	25:10 X vs. 44:1 X	-3.174	-3.378 to -2.971	Yes
24360	25:10 X vs. 44:10 X	-2.954	-3.158 to -2.750	Yes
24361	25:10 X vs. 44:40 X	-0.1497	-0.3533 to 0.05392	No
24362	25:10 X vs. 44:50 X	0.01467	-0.1889 to 0.2183	No
24363	25:10 X vs. 45:0 X	-3.149	-3.353 to -2.946	Yes
24364	25:10 X vs. 45:1 X	-2.951	-3.155 to -2.748	Yes
24365	25:10 X vs. 45:10 X	-2.834	-3.037 to -2.630	Yes
24366	25:10 X vs. 45:40 X	-0.1510	-0.3546 to 0.05259	No
24367	25:10 X vs. 45:50 X	0.01767	-0.1859 to 0.2213	No
24368	25:10 X vs. 46:0 X	-2.954	-3.157 to -2.750	Yes
24369	25:10 X vs. 46:1 X	-2.921	-3.124 to -2.717	Yes
24370	25:10 X vs. 46:10 X	-2.829	-3.032 to -2.625	Yes
24371	25:10 X vs. 46:40 X	-0.1647	-0.3683 to 0.03892	No
24372	25:10 X vs. 46:50 X	0.02467	-0.1789 to 0.2283	No
24373	25:10 X vs. 47:0 X	-2.929	-3.133 to -2.726	Yes
24374	25:10 X vs. 47:1 X	-2.833	-3.037 to -2.630	Yes
24375	25:10 X vs. 47:10 X	-2.815	-3.018 to -2.611	Yes
24376	25:10 X vs. 47:40 X	-0.1747	-0.3783 to 0.02892	No
24377	25:10 X vs. 47:50 X	0.06667	-0.1369 to 0.2703	No
24378	25:10 X vs. 48:0 X	-2.917	-3.132 to -2.701	Yes
24379	25:10 X vs. 48:1 X	-2.820	-3.024 to -2.617	Yes
24380	25:10 X vs. 48:10 X	-2.815	-3.018 to -2.611	Yes
24381	25:10 X vs. 48:40 X	-0.1810	-0.3846 to 0.02259	No
24382	25:10 X vs. 48:50 X	0.05933	-0.1443 to 0.2629	No
24383	25:10 X vs. 49:0 X	-2.917	-3.120 to -2.713	Yes
24384	25:10 X vs. 49:1 X	-2.820	-3.024 to -2.617	Yes
24385	25:10 X vs. 49:10 X	-2.815	-3.018 to -2.611	Yes
24386	25:10 X vs. 49:40 X	-0.1853	-0.3889 to 0.01826	No
24387	25:10 X vs. 49:50 X	0.06000	-0.1436 to 0.2636	No
24388	25:10 X vs. 50:0 X	-2.917	-3.120 to -2.713	Yes
24389	25:10 X vs. 50:1 X	-2.820	-3.024 to -2.617	Yes
24390	25:10 X vs. 50:10 X	-2.815	-3.018 to -2.611	Yes

	2way ANOVA Multiple comparisons	A Data Set-A Y	B Data Set-B Y	C Data Set-C Y
24391	25:10 X vs. 50:40 X	-0.1930	-0.3966 to 0.01059	No
24392	25:10 X vs. 50:50 X	0.06033	-0.1433 to 0.2639	No
24393	25:40 X vs. 25:50 X	0.02567	-0.1183 to 0.1696	No
24394	25:40 X vs. 26:0 X	-0.8292	-0.9901 to -0.6682	Yes
24395	25:40 X vs. 26:1 X	-0.9422	-1.103 to -0.7812	Yes
24396	25:40 X vs. 26:10 X	-0.6287	-0.7896 to -0.4677	Yes
24397	25:40 X vs. 26:40 X	-0.07433	-0.2183 to 0.06963	No
24398	25:40 X vs. 26:50 X	0.06533	-0.07863 to 0.2093	No
24399	25:40 X vs. 27:0 X	-1.017	-1.161 to -0.8730	Yes
24400	25:40 X vs. 27:1 X	-1.108	-1.269 to -0.9467	Yes
24401	25:40 X vs. 27:10 X	-0.9197	-1.081 to -0.7587	Yes
24402	25:40 X vs. 27:40 X	-0.07433	-0.2183 to 0.06963	No
24403	25:40 X vs. 27:50 X	0.06600	-0.07796 to 0.2100	No
24404	25:40 X vs. 28:0 X	-0.9662	-1.127 to -0.8052	Yes
24405	25:40 X vs. 28:1 X	-1.007	-1.151 to -0.8630	Yes
24406	25:40 X vs. 28:10 X	-0.8087	-0.9696 to -0.6477	Yes
24407	25:40 X vs. 28:40 X	-0.08667	-0.2306 to 0.05729	No
24408	25:40 X vs. 28:50 X	0.01800	-0.1260 to 0.1620	No
24409	25:40 X vs. 29:0 X	-1.448	-1.609 to -1.287	Yes
24410	25:40 X vs. 29:1 X	-1.533	-1.694 to -1.372	Yes
24411	25:40 X vs. 29:10 X	-0.7472	-0.9081 to -0.5862	Yes
24412	25:40 X vs. 29:40 X	-0.1947	-0.3386 to -0.05071	Yes
24413	25:40 X vs. 29:50 X	0.01667	-0.1273 to 0.1606	No
24414	25:40 X vs. 30:0 X	-1.162	-1.323 to -1.001	Yes
24415	25:40 X vs. 30:1 X	-1.484	-1.645 to -1.323	Yes
24416	25:40 X vs. 30:10 X	-0.8217	-0.9826 to -0.6607	Yes
24417	25:40 X vs. 30:40 X	-0.1217	-0.2656 to 0.02229	No
24418	25:40 X vs. 30:50 X	-0.03567	-0.1796 to 0.1083	No
24419	25:40 X vs. 31:0 X	-1.480	-1.641 to -1.319	Yes
24420	25:40 X vs. 31:1 X	-1.471	-1.632 to -1.310	Yes
24421	25:40 X vs. 31:10 X	-0.9532	-1.114 to -0.7922	Yes
24422	25:40 X vs. 31:40 X	-0.2133	-0.3573 to -0.06937	Yes
24423	25:40 X vs. 31:50 X	-0.01467	-0.1586 to 0.1293	No
24424	25:40 X vs. 32:0 X	-2.072	-2.216 to -1.928	Yes
24425	25:40 X vs. 32:1 X	-1.528	-1.689 to -1.367	Yes
24426	25:40 X vs. 32:10 X	-1.384	-1.528 to -1.240	Yes
24427	25:40 X vs. 32:40 X	-0.2097	-0.3536 to -0.06571	Yes
24428	25:40 X vs. 32:50 X	-0.01433	-0.1583 to 0.1296	No
24429	25:40 X vs. 33:0 X	-2.075	-2.219 to -1.931	Yes
24430	25:40 X vs. 33:1 X	-1.890	-2.034 to -1.746	Yes
24431	25:40 X vs. 33:10 X	-1.550	-1.694 to -1.406	Yes
24432	25:40 X vs. 33:40 X	-0.2283	-0.3723 to -0.08437	Yes
24433	25:40 X vs. 33:50 X	-0.02933	-0.1733 to 0.1146	No
24434	25:40 X vs. 34:0 X	-2.000	-2.144 to -1.856	Yes
24435	25:40 X vs. 34:1 X	-2.007	-2.151 to -1.863	Yes

2way ANOVA<br>Multiple comparisons	A<br>Data Set-A<br>Y	B<br>Data Set-B<br>Y	C<br>Data Set-C<br>Y
24436 25:40 X vs. 34:10 X	-1.641	-1.785 to -1.497	Yes
24437 25:40 X vs. 34:40 X	-0.2403	-0.3843 to -0.09637	Yes
24438 25:40 X vs. 34:50 X	-0.1783	-0.3223 to -0.03437	Yes
24439 25:40 X vs. 35:0 X	-2.349	-2.493 to -2.205	Yes
24440 25:40 X vs. 35:1 X	-2.114	-2.258 to -1.970	Yes
24441 25:40 X vs. 35:10 X	-1.602	-1.746 to -1.458	Yes
24442 25:40 X vs. 35:40 X	-0.2493	-0.3933 to -0.1054	Yes
24443 25:40 X vs. 35:50 X	-0.1910	-0.3350 to -0.04704	Yes
24444 25:40 X vs. 36:0 X	-2.555	-2.716 to -2.394	Yes
24445 25:40 X vs. 36:1 X	-2.251	-2.395 to -2.107	Yes
24446 25:40 X vs. 36:10 X	-1.608	-1.752 to -1.464	Yes
24447 25:40 X vs. 36:40 X	-0.3047	-0.4486 to -0.1607	Yes
24448 25:40 X vs. 36:50 X	-0.1543	-0.2983 to -0.01037	Yes
24449 25:40 X vs. 37:0 X	-2.939	-3.083 to -2.795	Yes
24450 25:40 X vs. 37:1 X	-2.321	-2.465 to -2.177	Yes
24451 25:40 X vs. 37:10 X	-1.610	-1.754 to -1.466	Yes
24452 25:40 X vs. 37:40 X	-0.3420	-0.4860 to -0.1980	Yes
24453 25:40 X vs. 37:50 X	-0.1630	-0.3070 to -0.01904	Yes
24454 25:40 X vs. 38:0 X	-2.884	-3.028 to -2.740	Yes
24455 25:40 X vs. 38:1 X	-2.440	-2.584 to -2.296	Yes
24456 25:40 X vs. 38:10 X	-1.885	-2.029 to -1.741	Yes
24457 25:40 X vs. 38:40 X	-0.3463	-0.4903 to -0.2024	Yes
24458 25:40 X vs. 38:50 X	-0.1670	-0.3110 to -0.02304	Yes
24459 25:40 X vs. 39:0 X	-3.115	-3.259 to -2.971	Yes
24460 25:40 X vs. 39:1 X	-3.006	-3.150 to -2.862	Yes
24461 25:40 X vs. 39:10 X	-2.030	-2.174 to -1.886	Yes
24462 25:40 X vs. 39:40 X	-0.3523	-0.4963 to -0.2084	Yes
24463 25:40 X vs. 39:50 X	-0.1980	-0.3420 to -0.05404	Yes
24464 25:40 X vs. 40:0 X	-3.156	-3.300 to -3.012	Yes
24465 25:40 X vs. 40:1 X	-3.082	-3.226 to -2.938	Yes
24466 25:40 X vs. 40:10 X	-2.286	-2.430 to -2.142	Yes
24467 25:40 X vs. 40:40 X	-0.3570	-0.5010 to -0.2130	Yes
24468 25:40 X vs. 40:50 X	-0.2043	-0.3483 to -0.06037	Yes
24469 25:40 X vs. 41:0 X	-3.197	-3.341 to -3.053	Yes
24470 25:40 X vs. 41:1 X	-3.196	-3.340 to -3.052	Yes
24471 25:40 X vs. 41:10 X	-2.340	-2.484 to -2.196	Yes
24472 25:40 X vs. 41:40 X	-0.3043	-0.4483 to -0.1604	Yes
24473 25:40 X vs. 41:50 X	-0.2170	-0.3610 to -0.07304	Yes
24474 25:40 X vs. 42:0 X	-3.338	-3.482 to -3.194	Yes
24475 25:40 X vs. 42:1 X	-3.358	-3.502 to -3.214	Yes
24476 25:40 X vs. 42:10 X	-2.340	-2.484 to -2.196	Yes
24477 25:40 X vs. 42:40 X	-0.3170	-0.4610 to -0.1730	Yes
24478 25:40 X vs. 42:50 X	-0.2203	-0.3643 to -0.07637	Yes
24479 25:40 X vs. 43:0 X	-3.428	-3.572 to -3.284	Yes
24480 25:40 X vs. 43:1 X	-3.343	-3.487 to -3.199	Yes

2way ANOVA<br>Multiple comparisons	A<br>Data Set-A<br>Y	B<br>Data Set-B<br>Y	C<br>Data Set-C<br>Y
24481 25:40 X vs. 43:10 X	-3.058	-3.202 to -2.914	Yes
24482 25:40 X vs. 43:40 X	-0.3153	-0.4593 to -0.1714	Yes
24483 25:40 X vs. 43:50 X	-0.2207	-0.3646 to -0.07671	Yes
24484 25:40 X vs. 44:0 X	-3.452	-3.596 to -3.308	Yes
24485 25:40 X vs. 44:1 X	-3.342	-3.486 to -3.198	Yes
24486 25:40 X vs. 44:10 X	-3.122	-3.266 to -2.978	Yes
24487 25:40 X vs. 44:40 X	-0.3173	-0.4613 to -0.1734	Yes
24488 25:40 X vs. 44:50 X	-0.1530	-0.2970 to -0.009040	Yes
24489 25:40 X vs. 45:0 X	-3.317	-3.461 to -3.173	Yes
24490 25:40 X vs. 45:1 X	-3.119	-3.263 to -2.975	Yes
24491 25:40 X vs. 45:10 X	-3.001	-3.145 to -2.857	Yes
24492 25:40 X vs. 45:40 X	-0.3187	-0.4626 to -0.1747	Yes
24493 25:40 X vs. 45:50 X	-0.1500	-0.2940 to -0.006040	Yes
24494 25:40 X vs. 46:0 X	-3.121	-3.265 to -2.977	Yes
24495 25:40 X vs. 46:1 X	-3.088	-3.232 to -2.944	Yes
24496 25:40 X vs. 46:10 X	-2.996	-3.140 to -2.852	Yes
24497 25:40 X vs. 46:40 X	-0.3323	-0.4763 to -0.1884	Yes
24498 25:40 X vs. 46:50 X	-0.1430	-0.2870 to 0.0009598	No
24499 25:40 X vs. 47:0 X	-3.097	-3.241 to -2.953	Yes
24500 25:40 X vs. 47:1 X	-3.001	-3.145 to -2.857	Yes
24501 25:40 X vs. 47:10 X	-2.982	-3.126 to -2.838	Yes
24502 25:40 X vs. 47:40 X	-0.3423	-0.4863 to -0.1984	Yes
24503 25:40 X vs. 47:50 X	-0.1010	-0.2450 to 0.04296	No
24504 25:40 X vs. 48:0 X	-3.084	-3.245 to -2.923	Yes
24505 25:40 X vs. 48:1 X	-2.988	-3.132 to -2.844	Yes
24506 25:40 X vs. 48:10 X	-2.982	-3.126 to -2.838	Yes
24507 25:40 X vs. 48:40 X	-0.3487	-0.4926 to -0.2047	Yes
24508 25:40 X vs. 48:50 X	-0.1083	-0.2523 to 0.03563	No
24509 25:40 X vs. 49:0 X	-3.084	-3.228 to -2.940	Yes
24510 25:40 X vs. 49:1 X	-2.988	-3.132 to -2.844	Yes
24511 25:40 X vs. 49:10 X	-2.982	-3.126 to -2.838	Yes
24512 25:40 X vs. 49:40 X	-0.3530	-0.4970 to -0.2090	Yes
24513 25:40 X vs. 49:50 X	-0.1077	-0.2516 to 0.03629	No
24514 25:40 X vs. 50:0 X	-3.084	-3.228 to -2.940	Yes
24515 25:40 X vs. 50:1 X	-2.988	-3.132 to -2.844	Yes
24516 25:40 X vs. 50:10 X	-2.982	-3.126 to -2.838	Yes
24517 25:40 X vs. 50:40 X	-0.3607	-0.5046 to -0.2167	Yes
24518 25:40 X vs. 50:50 X	-0.1073	-0.2513 to 0.03663	No
24519 25:50 X vs. 26:0 X	-0.8548	-1.016 to -0.6939	Yes
24520 25:50 X vs. 26:1 X	-0.9678	-1.129 to -0.8069	Yes
24521 25:50 X vs. 26:10 X	-0.6543	-0.8153 to -0.4934	Yes
24522 25:50 X vs. 26:40 X	-0.1000	-0.2440 to 0.04396	No
24523 25:50 X vs. 26:50 X	0.03967	-0.1043 to 0.1836	No
24524 25:50 X vs. 27:0 X	-1.043	-1.187 to -0.8987	Yes
24525 25:50 X vs. 27:1 X	-1.133	-1.294 to -0.9724	Yes

2way ANOVA<br>Multiple comparisons	A<br>Data Set-A<br>Y	B<br>Data Set-B<br>Y	C<br>Data Set-C<br>Y
24526 25:**50 X** vs. 27:**10 X**	-0.9453	-1.106 to -0.7844	Yes
24527 25:**50 X** vs. 27:**40 X**	-0.1000	-0.2440 to 0.04396	No
24528 25:**50 X** vs. 27:**50 X**	0.04033	-0.1036 to 0.1843	No
24529 25:**50 X** vs. 28:**0 X**	-0.9918	-1.153 to -0.8309	Yes
24530 25:**50 X** vs. 28:**1 X**	-1.033	-1.177 to -0.8887	Yes
24531 25:**50 X** vs. 28:**10 X**	-0.8343	-0.9953 to -0.6734	Yes
24532 25:**50 X** vs. 28:**40 X**	-0.1123	-0.2563 to 0.03163	No
24533 25:**50 X** vs. 28:**50 X**	-0.007667	-0.1516 to 0.1363	No
24534 25:**50 X** vs. 29:**0 X**	-1.473	-1.634 to -1.312	Yes
24535 25:**50 X** vs. 29:**1 X**	-1.558	-1.719 to -1.397	Yes
24536 25:**50 X** vs. 29:**10 X**	-0.7728	-0.9338 to -0.6119	Yes
24537 25:**50 X** vs. 29:**40 X**	-0.2203	-0.3643 to -0.07637	Yes
24538 25:**50 X** vs. 29:**50 X**	-0.009000	-0.1530 to 0.1350	No
24539 25:**50 X** vs. 30:**0 X**	-1.188	-1.349 to -1.027	Yes
24540 25:**50 X** vs. 30:**1 X**	-1.509	-1.670 to -1.348	Yes
24541 25:**50 X** vs. 30:**10 X**	-0.8473	-1.008 to -0.6864	Yes
24542 25:**50 X** vs. 30:**40 X**	-0.1473	-0.2913 to -0.003374	Yes
24543 25:**50 X** vs. 30:**50 X**	-0.06133	-0.2053 to 0.08263	No
24544 25:**50 X** vs. 31:**0 X**	-1.506	-1.667 to -1.345	Yes
24545 25:**50 X** vs. 31:**1 X**	-1.496	-1.657 to -1.335	Yes
24546 25:**50 X** vs. 31:**10 X**	-0.9788	-1.140 to -0.8179	Yes
24547 25:**50 X** vs. 31:**40 X**	-0.2390	-0.3830 to -0.09504	Yes
24548 25:**50 X** vs. 31:**50 X**	-0.04033	-0.1843 to 0.1036	No
24549 25:**50 X** vs. 32:**0 X**	-2.097	-2.241 to -1.953	Yes
24550 25:**50 X** vs. 32:**1 X**	-1.553	-1.714 to -1.392	Yes
24551 25:**50 X** vs. 32:**10 X**	-1.409	-1.553 to -1.265	Yes
24552 25:**50 X** vs. 32:**40 X**	-0.2353	-0.3793 to -0.09137	Yes
24553 25:**50 X** vs. 32:**50 X**	-0.0400	-0.1840 to 0.1040	No
24554 25:**50 X** vs. 33:**0 X**	-2.101	-2.245 to -1.957	Yes
24555 25:**50 X** vs. 33:**1 X**	-1.916	-2.060 to -1.772	Yes
24556 25:**50 X** vs. 33:**10 X**	-1.576	-1.720 to -1.432	Yes
24557 25:**50 X** vs. 33:**40 X**	-0.2540	-0.3980 to -0.1100	Yes
24558 25:**50 X** vs. 33:**50 X**	-0.05500	-0.1990 to 0.08896	No
24559 25:**50 X** vs. 34:**0 X**	-2.026	-2.170 to -1.882	Yes
24560 25:**50 X** vs. 34:**1 X**	-2.033	-2.177 to -1.889	Yes
24561 25:**50 X** vs. 34:**10 X**	-1.666	-1.810 to -1.522	Yes
24562 25:**50 X** vs. 34:**40 X**	-0.2660	-0.4100 to -0.1220	Yes
24563 25:**50 X** vs. 34:**50 X**	-0.2040	-0.3480 to -0.06004	Yes
24564 25:**50 X** vs. 35:**0 X**	-2.374	-2.518 to -2.230	Yes
24565 25:**50 X** vs. 35:**1 X**	-2.140	-2.284 to -1.996	Yes
24566 25:**50 X** vs. 35:**10 X**	-1.628	-1.772 to -1.484	Yes
24567 25:**50 X** vs. 35:**40 X**	-0.2750	-0.4190 to -0.1310	Yes
24568 25:**50 X** vs. 35:**50 X**	-0.2167	-0.3606 to -0.07271	Yes
24569 25:**50 X** vs. 36:**0 X**	-2.581	-2.742 to -2.420	Yes
24570 25:**50 X** vs. 36:**1 X**	-2.277	-2.421 to -2.133	Yes

2way ANOVA<br>Multiple comparisons	A<br>Data Set-A<br>Y	B<br>Data Set-B<br>Y	C<br>Data Set-C<br>Y
24571 25:**50 X** vs. 36:**10 X**	-1.634	-1.778 to -1.490	Yes
24572 25:**50 X** vs. 36:**40 X**	-0.3303	-0.4743 to -0.1864	Yes
24573 25:**50 X** vs. 36:**50 X**	-0.1800	-0.3240 to -0.03604	Yes
24574 25:**50 X** vs. 37:**0 X**	-2.965	-3.109 to -2.821	Yes
24575 25:**50 X** vs. 37:**1 X**	-2.347	-2.491 to -2.203	Yes
24576 25:**50 X** vs. 37:**10 X**	-1.635	-1.779 to -1.491	Yes
24577 25:**50 X** vs. 37:**40 X**	-0.3677	-0.5116 to -0.2237	Yes
24578 25:**50 X** vs. 37:**50 X**	-0.1887	-0.3326 to -0.04471	Yes
24579 25:**50 X** vs. 38:**0 X**	-2.910	-3.054 to -2.766	Yes
24580 25:**50 X** vs. 38:**1 X**	-2.465	-2.609 to -2.321	Yes
24581 25:**50 X** vs. 38:**10 X**	-1.911	-2.055 to -1.767	Yes
24582 25:**50 X** vs. 38:**40 X**	-0.3720	-0.5160 to -0.2280	Yes
24583 25:**50 X** vs. 38:**50 X**	-0.1927	-0.3366 to -0.04871	Yes
24584 25:**50 X** vs. 39:**0 X**	-3.140	-3.284 to -2.996	Yes
24585 25:**50 X** vs. 39:**1 X**	-3.032	-3.176 to -2.888	Yes
24586 25:**50 X** vs. 39:**10 X**	-2.056	-2.200 to -1.912	Yes
24587 25:**50 X** vs. 39:**40 X**	-0.3780	-0.5220 to -0.2340	Yes
24588 25:**50 X** vs. 39:**50 X**	-0.2237	-0.3676 to -0.07971	Yes
24589 25:**50 X** vs. 40:**0 X**	-3.181	-3.325 to -3.037	Yes
24590 25:**50 X** vs. 40:**1 X**	-3.107	-3.251 to -2.963	Yes
24591 25:**50 X** vs. 40:**10 X**	-2.312	-2.456 to -2.168	Yes
24592 25:**50 X** vs. 40:**40 X**	-0.3827	-0.5266 to -0.2387	Yes
24593 25:**50 X** vs. 40:**50 X**	-0.2300	-0.3740 to -0.08604	Yes
24594 25:**50 X** vs. 41:**0 X**	-3.222	-3.366 to -3.078	Yes
24595 25:**50 X** vs. 41:**1 X**	-3.221	-3.365 to -3.077	Yes
24596 25:**50 X** vs. 41:**10 X**	-2.366	-2.510 to -2.222	Yes
24597 25:**50 X** vs. 41:**40 X**	-0.3300	-0.4740 to -0.1860	Yes
24598 25:**50 X** vs. 41:**50 X**	-0.2427	-0.3866 to -0.09871	Yes
24599 25:**50 X** vs. 42:**0 X**	-3.364	-3.508 to -3.220	Yes
24600 25:**50 X** vs. 42:**1 X**	-3.384	-3.528 to -3.240	Yes
24601 25:**50 X** vs. 42:**10 X**	-2.366	-2.510 to -2.222	Yes
24602 25:**50 X** vs. 42:**40 X**	-0.3427	-0.4866 to -0.1987	Yes
24603 25:**50 X** vs. 42:**50 X**	-0.2460	-0.3900 to -0.1020	Yes
24604 25:**50 X** vs. 43:**0 X**	-3.454	-3.598 to -3.310	Yes
24605 25:**50 X** vs. 43:**1 X**	-3.368	-3.512 to -3.224	Yes
24606 25:**50 X** vs. 43:**10 X**	-3.084	-3.228 to -2.940	Yes
24607 25:**50 X** vs. 43:**40 X**	-0.3410	-0.4850 to -0.1970	Yes
24608 25:**50 X** vs. 43:**50 X**	-0.2463	-0.3903 to -0.1024	Yes
24609 25:**50 X** vs. 44:**0 X**	-3.478	-3.622 to -3.334	Yes
24610 25:**50 X** vs. 44:**1 X**	-3.368	-3.512 to -3.224	Yes
24611 25:**50 X** vs. 44:**10 X**	-3.147	-3.291 to -3.003	Yes
24612 25:**50 X** vs. 44:**40 X**	-0.3430	-0.4870 to -0.1990	Yes
24613 25:**50 X** vs. 44:**50 X**	-0.1787	-0.3226 to -0.03471	Yes
24614 25:**50 X** vs. 45:**0 X**	-3.343	-3.487 to -3.199	Yes
24615 25:**50 X** vs. 45:**1 X**	-3.145	-3.289 to -3.001	Yes

2way ANOVA<br>Multiple comparisons	A<br>Data Set-A<br>Y	B<br>Data Set-B<br>Y	C<br>Data Set-C<br>Y
24616 25:50 X vs. 45:10 X	-3.027	-3.171 to -2.883	Yes
24617 25:50 X vs. 45:40 X	-0.3443	-0.4883 to -0.2004	Yes
24618 25:50 X vs. 45:50 X	-0.1757	-0.3196 to -0.03171	Yes
24619 25:50 X vs. 46:0 X	-3.147	-3.291 to -3.003	Yes
24620 25:50 X vs. 46:1 X	-3.114	-3.258 to -2.970	Yes
24621 25:50 X vs. 46:10 X	-3.022	-3.166 to -2.878	Yes
24622 25:50 X vs. 46:40 X	-0.3580	-0.5020 to -0.2140	Yes
24623 25:50 X vs. 46:50 X	-0.1687	-0.3126 to -0.02471	Yes
24624 25:50 X vs. 47:0 X	-3.123	-3.267 to -2.979	Yes
24625 25:50 X vs. 47:1 X	-3.027	-3.171 to -2.883	Yes
24626 25:50 X vs. 47:10 X	-3.008	-3.152 to -2.864	Yes
24627 25:50 X vs. 47:40 X	-0.3680	-0.5120 to -0.2240	Yes
24628 25:50 X vs. 47:50 X	-0.1267	-0.2706 to 0.01729	No
24629 25:50 X vs. 48:0 X	-3.110	-3.271 to -2.949	Yes
24630 25:50 X vs. 48:1 X	-3.014	-3.158 to -2.870	Yes
24631 25:50 X vs. 48:10 X	-3.008	-3.152 to -2.864	Yes
24632 25:50 X vs. 48:40 X	-0.3743	-0.5183 to -0.2304	Yes
24633 25:50 X vs. 48:50 X	-0.1340	-0.2780 to 0.009960	No
24634 25:50 X vs. 49:0 X	-3.110	-3.254 to -2.966	Yes
24635 25:50 X vs. 49:1 X	-3.014	-3.158 to -2.870	Yes
24636 25:50 X vs. 49:10 X	-3.008	-3.152 to -2.864	Yes
24637 25:50 X vs. 49:40 X	-0.3787	-0.5226 to -0.2347	Yes
24638 25:50 X vs. 49:50 X	-0.1333	-0.2773 to 0.01063	No
24639 25:50 X vs. 50:0 X	-3.110	-3.254 to -2.966	Yes
24640 25:50 X vs. 50:1 X	-3.014	-3.158 to -2.870	Yes
24641 25:50 X vs. 50:10 X	-3.008	-3.152 to -2.864	Yes
24642 25:50 X vs. 50:40 X	-0.3863	-0.5303 to -0.2424	Yes
24643 25:50 X vs. 50:50 X	-0.1330	-0.2770 to 0.01096	No
24644 26:0 X vs. 26:1 X	-0.1130	-0.2893 to 0.06331	No
24645 26:0 X vs. 26:10 X	0.2005	0.02419 to 0.3768	Yes
24646 26:0 X vs. 26:40 X	0.7548	0.5939 to 0.9158	Yes
24647 26:0 X vs. 26:50 X	0.8945	0.7335 to 1.055	Yes
24648 26:0 X vs. 27:0 X	-0.1878	-0.3488 to -0.02688	Yes
24649 26:0 X vs. 27:1 X	-0.2785	-0.4548 to -0.1022	Yes
24650 26:0 X vs. 27:10 X	-0.09050	-0.2668 to 0.08581	No
24651 26:0 X vs. 27:40 X	0.7548	0.5939 to 0.9158	Yes
24652 26:0 X vs. 27:50 X	0.8952	0.7342 to 1.056	Yes
24653 26:0 X vs. 28:0 X	-0.1370	-0.3133 to 0.03931	No
24654 26:0 X vs. 28:1 X	-0.1778	-0.3388 to -0.01688	Yes
24655 26:0 X vs. 28:10 X	0.02050	-0.1558 to 0.1968	No
24656 26:0 X vs. 28:40 X	0.7425	0.5815 to 0.9035	Yes
24657 26:0 X vs. 28:50 X	0.8472	0.6862 to 1.008	Yes
24658 26:0 X vs. 29:0 X	-0.6185	-0.7948 to -0.4422	Yes
24659 26:0 X vs. 29:1 X	-0.7035	-0.8798 to -0.5272	Yes
24660 26:0 X vs. 29:10 X	0.08200	-0.09431 to 0.2583	No

2way ANOVA<br>Multiple comparisons		A<br>Data Set-A<br>Y	B<br>Data Set-B<br>Y	C<br>Data Set-C<br>Y
24661	26:0 X vs. 29:40 X	0.6345	0.4735 to 0.7955	Yes
24662	26:0 X vs. 29:50 X	0.8458	0.6849 to 1.007	Yes
24663	26:0 X vs. 30:0 X	-0.3330	-0.5093 to -0.1567	Yes
24664	26:0 X vs. 30:1 X	-0.6545	-0.8308 to -0.4782	Yes
24665	26:0 X vs. 30:10 X	0.007500	-0.1688 to 0.1838	No
24666	26:0 X vs. 30:40 X	0.7075	0.5465 to 0.8685	Yes
24667	26:0 X vs. 30:50 X	0.7935	0.6325 to 0.9545	Yes
24668	26:0 X vs. 31:0 X	-0.6510	-0.8273 to -0.4747	Yes
24669	26:0 X vs. 31:1 X	-0.6415	-0.8178 to -0.4652	Yes
24670	26:0 X vs. 31:10 X	-0.1240	-0.3003 to 0.05231	No
24671	26:0 X vs. 31:40 X	0.6158	0.4549 to 0.7768	Yes
24672	26:0 X vs. 31:50 X	0.8145	0.6535 to 0.9755	Yes
24673	26:0 X vs. 32:0 X	-1.243	-1.403 to -1.082	Yes
24674	26:0 X vs. 32:1 X	-0.6985	-0.8748 to -0.5222	Yes
24675	26:0 X vs. 32:10 X	-0.5545	-0.7155 to -0.3935	Yes
24676	26:0 X vs. 32:40 X	0.6195	0.4585 to 0.7805	Yes
24677	26:0 X vs. 32:50 X	0.8148	0.6539 to 0.9758	Yes
24678	26:0 X vs. 33:0 X	-1.246	-1.407 to -1.085	Yes
24679	26:0 X vs. 33:1 X	-1.061	-1.222 to -0.8999	Yes
24680	26:0 X vs. 33:10 X	-0.7212	-0.8821 to -0.5602	Yes
24681	26:0 X vs. 33:40 X	0.6008	0.4399 to 0.7618	Yes
24682	26:0 X vs. 33:50 X	0.7998	0.6389 to 0.9608	Yes
24683	26:0 X vs. 34:0 X	-1.171	-1.332 to -1.010	Yes
24684	26:0 X vs. 34:1 X	-1.178	-1.339 to -1.017	Yes
24685	26:0 X vs. 34:10 X	-0.8115	-0.9725 to -0.6505	Yes
24686	26:0 X vs. 34:40 X	0.5888	0.4279 to 0.7498	Yes
24687	26:0 X vs. 34:50 X	0.6508	0.4899 to 0.8118	Yes
24688	26:0 X vs. 35:0 X	-1.520	-1.680 to -1.359	Yes
24689	26:0 X vs. 35:1 X	-1.285	-1.446 to -1.124	Yes
24690	26:0 X vs. 35:10 X	-0.7732	-0.9341 to -0.6122	Yes
24691	26:0 X vs. 35:40 X	0.5798	0.4189 to 0.7408	Yes
24692	26:0 X vs. 35:50 X	0.6382	0.4772 to 0.7991	Yes
24693	26:0 X vs. 36:0 X	-1.726	-1.902 to -1.550	Yes
24694	26:0 X vs. 36:1 X	-1.422	-1.583 to -1.261	Yes
24695	26:0 X vs. 36:10 X	-0.7788	-0.9398 to -0.6179	Yes
24696	26:0 X vs. 36:40 X	0.5245	0.3635 to 0.6855	Yes
24697	26:0 X vs. 36:50 X	0.6748	0.5139 to 0.8358	Yes
24698	26:0 X vs. 37:0 X	-2.110	-2.271 to -1.949	Yes
24699	26:0 X vs. 37:1 X	-1.492	-1.653 to -1.331	Yes
24700	26:0 X vs. 37:10 X	-0.7805	-0.9415 to -0.6195	Yes
24701	26:0 X vs. 37:40 X	0.4872	0.3262 to 0.6481	Yes
24702	26:0 X vs. 37:50 X	0.6662	0.5052 to 0.8271	Yes
24703	26:0 X vs. 38:0 X	-2.055	-2.216 to -1.894	Yes
24704	26:0 X vs. 38:1 X	-1.611	-1.771 to -1.450	Yes
24705	26:0 X vs. 38:10 X	-1.056	-1.217 to -0.8949	Yes

2way ANOVA<br>Multiple comparisons	A<br>Data Set-A<br>Y	B<br>Data Set-B<br>Y	C<br>Data Set-C<br>Y
24706 26:0 X vs. 38:40 X	0.4828	0.3219 to 0.6438	Yes
24707 26:0 X vs. 38:50 X	0.6622	0.5012 to 0.8231	Yes
24708 26:0 X vs. 39:0 X	-2.286	-2.446 to -2.125	Yes
24709 26:0 X vs. 39:1 X	-2.177	-2.338 to -2.016	Yes
24710 26:0 X vs. 39:10 X	-1.201	-1.362 to -1.040	Yes
24711 26:0 X vs. 39:40 X	0.4768	0.3159 to 0.6378	Yes
24712 26:0 X vs. 39:50 X	0.6312	0.4702 to 0.7921	Yes
24713 26:0 X vs. 40:0 X	-2.327	-2.487 to -2.166	Yes
24714 26:0 X vs. 40:1 X	-2.253	-2.413 to -2.092	Yes
24715 26:0 X vs. 40:10 X	-1.457	-1.618 to -1.296	Yes
24716 26:0 X vs. 40:40 X	0.4722	0.3112 to 0.6331	Yes
24717 26:0 X vs. 40:50 X	0.6248	0.4639 to 0.7858	Yes
24718 26:0 X vs. 41:0 X	-2.368	-2.528 to -2.207	Yes
24719 26:0 X vs. 41:1 X	-2.367	-2.527 to -2.206	Yes
24720 26:0 X vs. 41:10 X	-1.511	-1.672 to -1.350	Yes
24721 26:0 X vs. 41:40 X	0.5248	0.3639 to 0.6858	Yes
24722 26:0 X vs. 41:50 X	0.6122	0.4512 to 0.7731	Yes
24723 26:0 X vs. 42:0 X	-2.509	-2.670 to -2.348	Yes
24724 26:0 X vs. 42:1 X	-2.529	-2.690 to -2.368	Yes
24725 26:0 X vs. 42:10 X	-1.511	-1.672 to -1.350	Yes
24726 26:0 X vs. 42:40 X	0.5122	0.3512 to 0.6731	Yes
24727 26:0 X vs. 42:50 X	0.6088	0.4479 to 0.7698	Yes
24728 26:0 X vs. 43:0 X	-2.599	-2.760 to -2.438	Yes
24729 26:0 X vs. 43:1 X	-2.514	-2.674 to -2.353	Yes
24730 26:0 X vs. 43:10 X	-2.229	-2.390 to -2.068	Yes
24731 26:0 X vs. 43:40 X	0.5138	0.3529 to 0.6748	Yes
24732 26:0 X vs. 43:50 X	0.6085	0.4475 to 0.7695	Yes
24733 26:0 X vs. 44:0 X	-2.623	-2.784 to -2.462	Yes
24734 26:0 X vs. 44:1 X	-2.513	-2.674 to -2.352	Yes
24735 26:0 X vs. 44:10 X	-2.293	-2.453 to -2.132	Yes
24736 26:0 X vs. 44:40 X	0.5118	0.3509 to 0.6728	Yes
24737 26:0 X vs. 44:50 X	0.6762	0.5152 to 0.8371	Yes
24738 26:0 X vs. 45:0 X	-2.488	-2.649 to -2.327	Yes
24739 26:0 X vs. 45:1 X	-2.290	-2.451 to -2.129	Yes
24740 26:0 X vs. 45:10 X	-2.172	-2.333 to -2.011	Yes
24741 26:0 X vs. 45:40 X	0.5105	0.3495 to 0.6715	Yes
24742 26:0 X vs. 45:50 X	0.6792	0.5182 to 0.8401	Yes
24743 26:0 X vs. 46:0 X	-2.292	-2.453 to -2.131	Yes
24744 26:0 X vs. 46:1 X	-2.259	-2.420 to -2.098	Yes
24745 26:0 X vs. 46:10 X	-2.167	-2.328 to -2.006	Yes
24746 26:0 X vs. 46:40 X	0.4968	0.3359 to 0.6578	Yes
24747 26:0 X vs. 46:50 X	0.6862	0.5252 to 0.8471	Yes
24748 26:0 X vs. 47:0 X	-2.268	-2.429 to -2.107	Yes
24749 26:0 X vs. 47:1 X	-2.172	-2.333 to -2.011	Yes
24750 26:0 X vs. 47:10 X	-2.153	-2.314 to -1.992	Yes

2way ANOVA<br>Multiple comparisons	A<br>Data Set-A<br>Y	B<br>Data Set-B<br>Y	C<br>Data Set-C<br>Y
24751 26:0 X vs. 47:40 X	0.4868	0.3259 to 0.6478	Yes
24752 26:0 X vs. 47:50 X	0.7282	0.5672 to 0.8891	Yes
24753 26:0 X vs. 48:0 X	-2.255	-2.431 to -2.079	Yes
24754 26:0 X vs. 48:1 X	-2.159	-2.320 to -1.998	Yes
24755 26:0 X vs. 48:10 X	-2.153	-2.314 to -1.992	Yes
24756 26:0 X vs. 48:40 X	0.4805	0.3195 to 0.6415	Yes
24757 26:0 X vs. 48:50 X	0.7208	0.5599 to 0.8818	Yes
24758 26:0 X vs. 49:0 X	-2.255	-2.416 to -2.094	Yes
24759 26:0 X vs. 49:1 X	-2.159	-2.320 to -1.998	Yes
24760 26:0 X vs. 49:10 X	-2.153	-2.314 to -1.992	Yes
24761 26:0 X vs. 49:40 X	0.4762	0.3152 to 0.6371	Yes
24762 26:0 X vs. 49:50 X	0.7215	0.5605 to 0.8825	Yes
24763 26:0 X vs. 50:0 X	-2.255	-2.416 to -2.094	Yes
24764 26:0 X vs. 50:1 X	-2.159	-2.320 to -1.998	Yes
24765 26:0 X vs. 50:10 X	-2.153	-2.314 to -1.992	Yes
24766 26:0 X vs. 50:40 X	0.4685	0.3075 to 0.6295	Yes
24767 26:0 X vs. 50:50 X	0.7218	0.5609 to 0.8828	Yes
24768 26:1 X vs. 26:10 X	0.3135	0.1372 to 0.4898	Yes
24769 26:1 X vs. 26:40 X	0.8678	0.7069 to 1.029	Yes
24770 26:1 X vs. 26:50 X	1.008	0.8465 to 1.168	Yes
24771 26:1 X vs. 27:0 X	-0.07483	-0.2358 to 0.08612	No
24772 26:1 X vs. 27:1 X	-0.1655	-0.3418 to 0.01081	No
24773 26:1 X vs. 27:10 X	0.02250	-0.1538 to 0.1988	No
24774 26:1 X vs. 27:40 X	0.8678	0.7069 to 1.029	Yes
24775 26:1 X vs. 27:50 X	1.008	0.8472 to 1.169	Yes
24776 26:1 X vs. 28:0 X	-0.02400	-0.2003 to 0.1523	No
24777 26:1 X vs. 28:1 X	-0.06483	-0.2258 to 0.09612	No
24778 26:1 X vs. 28:10 X	0.1335	-0.04281 to 0.3098	No
24779 26:1 X vs. 28:40 X	0.8555	0.6945 to 1.016	Yes
24780 26:1 X vs. 28:50 X	0.9602	0.7992 to 1.121	Yes
24781 26:1 X vs. 29:0 X	-0.5055	-0.6818 to -0.3292	Yes
24782 26:1 X vs. 29:1 X	-0.5905	-0.7668 to -0.4142	Yes
24783 26:1 X vs. 29:10 X	0.1950	0.01869 to 0.3713	Yes
24784 26:1 X vs. 29:40 X	0.7475	0.5865 to 0.9085	Yes
24785 26:1 X vs. 29:50 X	0.9588	0.7979 to 1.120	Yes
24786 26:1 X vs. 30:0 X	-0.2200	-0.3963 to -0.04369	Yes
24787 26:1 X vs. 30:1 X	-0.5415	-0.7178 to -0.3652	Yes
24788 26:1 X vs. 30:10 X	0.1205	-0.05581 to 0.2968	No
24789 26:1 X vs. 30:40 X	0.8205	0.6595 to 0.9815	Yes
24790 26:1 X vs. 30:50 X	0.9065	0.7455 to 1.067	Yes
24791 26:1 X vs. 31:0 X	-0.5380	-0.7143 to -0.3617	Yes
24792 26:1 X vs. 31:1 X	-0.5285	-0.7048 to -0.3522	Yes
24793 26:1 X vs. 31:10 X	-0.01100	-0.1873 to 0.1653	No
24794 26:1 X vs. 31:40 X	0.7288	0.5679 to 0.8898	Yes
24795 26:1 X vs. 31:50 X	0.9275	0.7665 to 1.088	Yes

2way ANOVA<br>Multiple comparisons	A<br>Data Set-A<br>Y	B<br>Data Set-B<br>Y	C<br>Data Set-C<br>Y
24796 26:**1 X** vs. 32:**0 X**	-1.130	-1.290 to -0.9685	Yes
24797 26:**1 X** vs. 32:**1 X**	-0.5855	-0.7618 to -0.4092	Yes
24798 26:**1 X** vs. 32:**10 X**	-0.4415	-0.6025 to -0.2805	Yes
24799 26:**1 X** vs. 32:**40 X**	0.7325	0.5715 to 0.8935	Yes
24800 26:**1 X** vs. 32:**50 X**	0.9278	0.7669 to 1.089	Yes
24801 26:**1 X** vs. 33:**0 X**	-1.133	-1.294 to -0.9722	Yes
24802 26:**1 X** vs. 33:**1 X**	-0.9478	-1.109 to -0.7869	Yes
24803 26:**1 X** vs. 33:**10 X**	-0.6082	-0.7691 to -0.4472	Yes
24804 26:**1 X** vs. 33:**40 X**	0.7138	0.5529 to 0.8748	Yes
24805 26:**1 X** vs. 33:**50 X**	0.9128	0.7519 to 1.074	Yes
24806 26:**1 X** vs. 34:**0 X**	-1.058	-1.219 to -0.8969	Yes
24807 26:**1 X** vs. 34:**1 X**	-1.065	-1.226 to -0.9042	Yes
24808 26:**1 X** vs. 34:**10 X**	-0.6985	-0.8595 to -0.5375	Yes
24809 26:**1 X** vs. 34:**40 X**	0.7018	0.5409 to 0.8628	Yes
24810 26:**1 X** vs. 34:**50 X**	0.7638	0.6029 to 0.9248	Yes
24811 26:**1 X** vs. 35:**0 X**	-1.407	-1.567 to -1.246	Yes
24812 26:**1 X** vs. 35:**1 X**	-1.172	-1.333 to -1.011	Yes
24813 26:**1 X** vs. 35:**10 X**	-0.6602	-0.8211 to -0.4992	Yes
24814 26:**1 X** vs. 35:**40 X**	0.6928	0.5319 to 0.8538	Yes
24815 26:**1 X** vs. 35:**50 X**	0.7512	0.5902 to 0.9121	Yes
24816 26:**1 X** vs. 36:**0 X**	-1.613	-1.789 to -1.437	Yes
24817 26:**1 X** vs. 36:**1 X**	-1.309	-1.470 to -1.148	Yes
24818 26:**1 X** vs. 36:**10 X**	-0.6658	-0.8268 to -0.5049	Yes
24819 26:**1 X** vs. 36:**40 X**	0.6375	0.4765 to 0.7985	Yes
24820 26:**1 X** vs. 36:**50 X**	0.7878	0.6269 to 0.9488	Yes
24821 26:**1 X** vs. 37:**0 X**	-1.997	-2.158 to -1.836	Yes
24822 26:**1 X** vs. 37:**1 X**	-1.379	-1.540 to -1.218	Yes
24823 26:**1 X** vs. 37:**10 X**	-0.6675	-0.8285 to -0.5065	Yes
24824 26:**1 X** vs. 37:**40 X**	0.6002	0.4392 to 0.7611	Yes
24825 26:**1 X** vs. 37:**50 X**	0.7792	0.6182 to 0.9401	Yes
24826 26:**1 X** vs. 38:**0 X**	-1.942	-2.103 to -1.781	Yes
24827 26:**1 X** vs. 38:**1 X**	-1.498	-1.658 to -1.337	Yes
24828 26:**1 X** vs. 38:**10 X**	-0.9428	-1.104 to -0.7819	Yes
24829 26:**1 X** vs. 38:**40 X**	0.5958	0.4349 to 0.7568	Yes
24830 26:**1 X** vs. 38:**50 X**	0.7752	0.6142 to 0.9361	Yes
24831 26:**1 X** vs. 39:**0 X**	-2.173	-2.333 to -2.012	Yes
24832 26:**1 X** vs. 39:**1 X**	-2.064	-2.225 to -1.903	Yes
24833 26:**1 X** vs. 39:**10 X**	-1.088	-1.249 to -0.9269	Yes
24834 26:**1 X** vs. 39:**40 X**	0.5898	0.4289 to 0.7508	Yes
24835 26:**1 X** vs. 39:**50 X**	0.7442	0.5832 to 0.9051	Yes
24836 26:**1 X** vs. 40:**0 X**	-2.214	-2.374 to -2.053	Yes
24837 26:**1 X** vs. 40:**1 X**	-2.140	-2.300 to -1.979	Yes
24838 26:**1 X** vs. 40:**10 X**	-1.344	-1.505 to -1.183	Yes
24839 26:**1 X** vs. 40:**40 X**	0.5852	0.4242 to 0.7461	Yes
24840 26:**1 X** vs. 40:**50 X**	0.7378	0.5769 to 0.8988	Yes

2way ANOVA<br>Multiple comparisons	A<br>Data Set-A<br>Y	B<br>Data Set-B<br>Y	C<br>Data Set-C<br>Y
24841 26:1 X vs. 41:0 X	-2.255	-2.415 to -2.094	Yes
24842 26:1 X vs. 41:1 X	-2.254	-2.414 to -2.093	Yes
24843 26:1 X vs. 41:10 X	-1.398	-1.559 to -1.237	Yes
24844 26:1 X vs. 41:40 X	0.6378	0.4769 to 0.7988	Yes
24845 26:1 X vs. 41:50 X	0.7252	0.5642 to 0.8861	Yes
24846 26:1 X vs. 42:0 X	-2.396	-2.557 to -2.235	Yes
24847 26:1 X vs. 42:1 X	-2.416	-2.577 to -2.255	Yes
24848 26:1 X vs. 42:10 X	-1.398	-1.559 to -1.237	Yes
24849 26:1 X vs. 42:40 X	0.6252	0.4642 to 0.7861	Yes
24850 26:1 X vs. 42:50 X	0.7218	0.5609 to 0.8828	Yes
24851 26:1 X vs. 43:0 X	-2.486	-2.647 to -2.325	Yes
24852 26:1 X vs. 43:1 X	-2.401	-2.561 to -2.240	Yes
24853 26:1 X vs. 43:10 X	-2.116	-2.277 to -1.955	Yes
24854 26:1 X vs. 43:40 X	0.6268	0.4659 to 0.7878	Yes
24855 26:1 X vs. 43:50 X	0.7215	0.5605 to 0.8825	Yes
24856 26:1 X vs. 44:0 X	-2.510	-2.671 to -2.349	Yes
24857 26:1 X vs. 44:1 X	-2.400	-2.561 to -2.239	Yes
24858 26:1 X vs. 44:10 X	-2.180	-2.340 to -2.019	Yes
24859 26:1 X vs. 44:40 X	0.6248	0.4639 to 0.7858	Yes
24860 26:1 X vs. 44:50 X	0.7892	0.6282 to 0.9501	Yes
24861 26:1 X vs. 45:0 X	-2.375	-2.536 to -2.214	Yes
24862 26:1 X vs. 45:1 X	-2.177	-2.338 to -2.016	Yes
24863 26:1 X vs. 45:10 X	-2.059	-2.220 to -1.898	Yes
24864 26:1 X vs. 45:40 X	0.6235	0.4625 to 0.7845	Yes
24865 26:1 X vs. 45:50 X	0.7922	0.6312 to 0.9531	Yes
24866 26:1 X vs. 46:0 X	-2.179	-2.340 to -2.018	Yes
24867 26:1 X vs. 46:1 X	-2.146	-2.307 to -1.985	Yes
24868 26:1 X vs. 46:10 X	-2.054	-2.215 to -1.893	Yes
24869 26:1 X vs. 46:40 X	0.6098	0.4489 to 0.7708	Yes
24870 26:1 X vs. 46:50 X	0.7992	0.6382 to 0.9601	Yes
24871 26:1 X vs. 47:0 X	-2.155	-2.316 to -1.994	Yes
24872 26:1 X vs. 47:1 X	-2.059	-2.220 to -1.898	Yes
24873 26:1 X vs. 47:10 X	-2.040	-2.201 to -1.879	Yes
24874 26:1 X vs. 47:40 X	0.5998	0.4389 to 0.7608	Yes
24875 26:1 X vs. 47:50 X	0.8412	0.6802 to 1.002	Yes
24876 26:1 X vs. 48:0 X	-2.142	-2.318 to -1.966	Yes
24877 26:1 X vs. 48:1 X	-2.046	-2.207 to -1.885	Yes
24878 26:1 X vs. 48:10 X	-2.040	-2.201 to -1.879	Yes
24879 26:1 X vs. 48:40 X	0.5935	0.4325 to 0.7545	Yes
24880 26:1 X vs. 48:50 X	0.8338	0.6729 to 0.9948	Yes
24881 26:1 X vs. 49:0 X	-2.142	-2.303 to -1.981	Yes
24882 26:1 X vs. 49:1 X	-2.046	-2.207 to -1.885	Yes
24883 26:1 X vs. 49:10 X	-2.040	-2.201 to -1.879	Yes
24884 26:1 X vs. 49:40 X	0.5892	0.4282 to 0.7501	Yes
24885 26:1 X vs. 49:50 X	0.8345	0.6735 to 0.9955	Yes

	2way ANOVA<br>Multiple comparisons	A<br>Data Set-A<br>Y	B<br>Data Set-B<br>Y	C<br>Data Set-C<br>Y
24886	26:1 X vs. 50:0 X	-2.142	-2.303 to -1.981	Yes
24887	26:1 X vs. 50:1 X	-2.046	-2.207 to -1.885	Yes
24888	26:1 X vs. 50:10 X	-2.040	-2.201 to -1.879	Yes
24889	26:1 X vs. 50:40 X	0.5815	0.4205 to 0.7425	Yes
24890	26:1 X vs. 50:50 X	0.8348	0.6739 to 0.9958	Yes
24891	26:10 X vs. 26:40 X	0.5543	0.3934 to 0.7153	Yes
24892	26:10 X vs. 26:50 X	0.6940	0.5330 to 0.8550	Yes
24893	26:10 X vs. 27:0 X	-0.3883	-0.5493 to -0.2274	Yes
24894	26:10 X vs. 27:1 X	-0.4790	-0.6553 to -0.3027	Yes
24895	26:10 X vs. 27:10 X	-0.2910	-0.4673 to -0.1147	Yes
24896	26:10 X vs. 27:40 X	0.5543	0.3934 to 0.7153	Yes
24897	26:10 X vs. 27:50 X	0.6947	0.5337 to 0.8556	Yes
24898	26:10 X vs. 28:0 X	-0.3375	-0.5138 to -0.1612	Yes
24899	26:10 X vs. 28:1 X	-0.3783	-0.5393 to -0.2174	Yes
24900	26:10 X vs. 28:10 X	-0.1800	-0.3563 to -0.003686	Yes
24901	26:10 X vs. 28:40 X	0.5420	0.3810 to 0.7030	Yes
24902	26:10 X vs. 28:50 X	0.6467	0.4857 to 0.8076	Yes
24903	26:10 X vs. 29:0 X	-0.8190	-0.9953 to -0.6427	Yes
24904	26:10 X vs. 29:1 X	-0.9040	-1.080 to -0.7277	Yes
24905	26:10 X vs. 29:10 X	-0.1185	-0.2948 to 0.05781	No
24906	26:10 X vs. 29:40 X	0.4340	0.2730 to 0.5950	Yes
24907	26:10 X vs. 29:50 X	0.6453	0.4844 to 0.8063	Yes
24908	26:10 X vs. 30:0 X	-0.5335	-0.7098 to -0.3572	Yes
24909	26:10 X vs. 30:1 X	-0.8550	-1.031 to -0.6787	Yes
24910	26:10 X vs. 30:10 X	-0.1930	-0.3693 to -0.01669	Yes
24911	26:10 X vs. 30:40 X	0.5070	0.3460 to 0.6680	Yes
24912	26:10 X vs. 30:50 X	0.5930	0.4320 to 0.7540	Yes
24913	26:10 X vs. 31:0 X	-0.8515	-1.028 to -0.6752	Yes
24914	26:10 X vs. 31:1 X	-0.8420	-1.018 to -0.6657	Yes
24915	26:10 X vs. 31:10 X	-0.3245	-0.5008 to -0.1482	Yes
24916	26:10 X vs. 31:40 X	0.4153	0.2544 to 0.5763	Yes
24917	26:10 X vs. 31:50 X	0.6140	0.4530 to 0.7750	Yes
24918	26:10 X vs. 32:0 X	-1.443	-1.604 to -1.282	Yes
24919	26:10 X vs. 32:1 X	-0.8990	-1.075 to -0.7227	Yes
24920	26:10 X vs. 32:10 X	-0.7550	-0.9160 to -0.5940	Yes
24921	26:10 X vs. 32:40 X	0.4190	0.2580 to 0.5800	Yes
24922	26:10 X vs. 32:50 X	0.6143	0.4534 to 0.7753	Yes
24923	26:10 X vs. 33:0 X	-1.447	-1.608 to -1.286	Yes
24924	26:10 X vs. 33:1 X	-1.261	-1.422 to -1.100	Yes
24925	26:10 X vs. 33:10 X	-0.9217	-1.083 to -0.7607	Yes
24926	26:10 X vs. 33:40 X	0.4003	0.2394 to 0.5613	Yes
24927	26:10 X vs. 33:50 X	0.5993	0.4384 to 0.7603	Yes
24928	26:10 X vs. 34:0 X	-1.371	-1.532 to -1.210	Yes
24929	26:10 X vs. 34:1 X	-1.379	-1.540 to -1.218	Yes
24930	26:10 X vs. 34:10 X	-1.012	-1.173 to -0.8510	Yes

2way ANOVA Multiple comparisons	A Data Set-A Y	B Data Set-B Y	C Data Set-C Y
24931 26:10 X vs. 34:40 X	0.3883	0.2274 to 0.5493	Yes
24932 26:10 X vs. 34:50 X	0.4503	0.2894 to 0.6113	Yes
24933 26:10 X vs. 35:0 X	-1.720	-1.881 to -1.559	Yes
24934 26:10 X vs. 35:1 X	-1.486	-1.647 to -1.325	Yes
24935 26:10 X vs. 35:10 X	-0.9737	-1.135 to -0.8127	Yes
24936 26:10 X vs. 35:40 X	0.3793	0.2184 to 0.5403	Yes
24937 26:10 X vs. 35:50 X	0.4377	0.2767 to 0.5986	Yes
24938 26:10 X vs. 36:0 X	-1.927	-2.103 to -1.750	Yes
24939 26:10 X vs. 36:1 X	-1.622	-1.783 to -1.461	Yes
24940 26:10 X vs. 36:10 X	-0.9793	-1.140 to -0.8184	Yes
24941 26:10 X vs. 36:40 X	0.3240	0.1630 to 0.4850	Yes
24942 26:10 X vs. 36:50 X	0.4743	0.3134 to 0.6353	Yes
24943 26:10 X vs. 37:0 X	-2.310	-2.471 to -2.149	Yes
24944 26:10 X vs. 37:1 X	-1.692	-1.853 to -1.531	Yes
24945 26:10 X vs. 37:10 X	-0.9810	-1.142 to -0.8200	Yes
24946 26:10 X vs. 37:40 X	0.2867	0.1257 to 0.4476	Yes
24947 26:10 X vs. 37:50 X	0.4657	0.3047 to 0.6266	Yes
24948 26:10 X vs. 38:0 X	-2.255	-2.416 to -2.094	Yes
24949 26:10 X vs. 38:1 X	-1.811	-1.972 to -1.650	Yes
24950 26:10 X vs. 38:10 X	-1.256	-1.417 to -1.095	Yes
24951 26:10 X vs. 38:40 X	0.2823	0.1214 to 0.4433	Yes
24952 26:10 X vs. 38:50 X	0.4617	0.3007 to 0.6226	Yes
24953 26:10 X vs. 39:0 X	-2.486	-2.647 to -2.325	Yes
24954 26:10 X vs. 39:1 X	-2.378	-2.539 to -2.217	Yes
24955 26:10 X vs. 39:10 X	-1.401	-1.562 to -1.240	Yes
24956 26:10 X vs. 39:40 X	0.2763	0.1154 to 0.4373	Yes
24957 26:10 X vs. 39:50 X	0.4307	0.2697 to 0.5916	Yes
24958 26:10 X vs. 40:0 X	-2.527	-2.688 to -2.366	Yes
24959 26:10 X vs. 40:1 X	-2.453	-2.614 to -2.292	Yes
24960 26:10 X vs. 40:10 X	-1.658	-1.819 to -1.497	Yes
24961 26:10 X vs. 40:40 X	0.2717	0.1107 to 0.4326	Yes
24962 26:10 X vs. 40:50 X	0.4243	0.2634 to 0.5853	Yes
24963 26:10 X vs. 41:0 X	-2.568	-2.729 to -2.407	Yes
24964 26:10 X vs. 41:1 X	-2.567	-2.728 to -2.406	Yes
24965 26:10 X vs. 41:10 X	-1.711	-1.872 to -1.550	Yes
24966 26:10 X vs. 41:40 X	0.3243	0.1634 to 0.4853	Yes
24967 26:10 X vs. 41:50 X	0.4117	0.2507 to 0.5726	Yes
24968 26:10 X vs. 42:0 X	-2.710	-2.871 to -2.549	Yes
24969 26:10 X vs. 42:1 X	-2.729	-2.890 to -2.568	Yes
24970 26:10 X vs. 42:10 X	-1.712	-1.873 to -1.551	Yes
24971 26:10 X vs. 42:40 X	0.3117	0.1507 to 0.4726	Yes
24972 26:10 X vs. 42:50 X	0.4083	0.2474 to 0.5693	Yes
24973 26:10 X vs. 43:0 X	-2.800	-2.961 to -2.639	Yes
24974 26:10 X vs. 43:1 X	-2.714	-2.875 to -2.553	Yes
24975 26:10 X vs. 43:10 X	-2.430	-2.591 to -2.269	Yes

	2way ANOVA<br>Multiple comparisons	A<br>Data Set-A<br>Y	B<br>Data Set-B<br>Y	C<br>Data Set-C<br>Y
24976	26:**10 X** vs. 43:**40 X**	0.3133	0.1524 to 0.4743	Yes
24977	26:**10 X** vs. 43:**50 X**	0.4080	0.2470 to 0.5690	Yes
24978	26:**10 X** vs. 44:**0 X**	-2.824	-2.985 to -2.663	Yes
24979	26:**10 X** vs. 44:**1 X**	-2.713	-2.874 to -2.552	Yes
24980	26:**10 X** vs. 44:**10 X**	-2.493	-2.654 to -2.332	Yes
24981	26:**10 X** vs. 44:**40 X**	0.3113	0.1504 to 0.4723	Yes
24982	26:**10 X** vs. 44:**50 X**	0.4757	0.3147 to 0.6366	Yes
24983	26:**10 X** vs. 45:**0 X**	-2.688	-2.849 to -2.527	Yes
24984	26:**10 X** vs. 45:**1 X**	-2.490	-2.651 to -2.329	Yes
24985	26:**10 X** vs. 45:**10 X**	-2.373	-2.534 to -2.212	Yes
24986	26:**10 X** vs. 45:**40 X**	0.3100	0.1490 to 0.4710	Yes
24987	26:**10 X** vs. 45:**50 X**	0.4787	0.3177 to 0.6396	Yes
24988	26:**10 X** vs. 46:**0 X**	-2.493	-2.654 to -2.332	Yes
24989	26:**10 X** vs. 46:**1 X**	-2.460	-2.621 to -2.299	Yes
24990	26:**10 X** vs. 46:**10 X**	-2.368	-2.529 to -2.207	Yes
24991	26:**10 X** vs. 46:**40 X**	0.2963	0.1354 to 0.4573	Yes
24992	26:**10 X** vs. 46:**50 X**	0.4857	0.3247 to 0.6466	Yes
24993	26:**10 X** vs. 47:**0 X**	-2.468	-2.629 to -2.307	Yes
24994	26:**10 X** vs. 47:**1 X**	-2.372	-2.533 to -2.211	Yes
24995	26:**10 X** vs. 47:**10 X**	-2.354	-2.515 to -2.193	Yes
24996	26:**10 X** vs. 47:**40 X**	0.2863	0.1254 to 0.4473	Yes
24997	26:**10 X** vs. 47:**50 X**	0.5277	0.3667 to 0.6886	Yes
24998	26:**10 X** vs. 48:**0 X**	-2.456	-2.632 to -2.279	Yes
24999	26:**10 X** vs. 48:**1 X**	-2.359	-2.520 to -2.198	Yes
25000	26:**10 X** vs. 48:**10 X**	-2.354	-2.515 to -2.193	Yes
25001	26:**10 X** vs. 48:**40 X**	0.2800	0.1190 to 0.4410	Yes
25002	26:**10 X** vs. 48:**50 X**	0.5203	0.3594 to 0.6813	Yes
25003	26:**10 X** vs. 49:**0 X**	-2.456	-2.617 to -2.295	Yes
25004	26:**10 X** vs. 49:**1 X**	-2.359	-2.520 to -2.198	Yes
25005	26:**10 X** vs. 49:**10 X**	-2.354	-2.515 to -2.193	Yes
25006	26:**10 X** vs. 49:**40 X**	0.2757	0.1147 to 0.4366	Yes
25007	26:**10 X** vs. 49:**50 X**	0.5210	0.3600 to 0.6820	Yes
25008	26:**10 X** vs. 50:**0 X**	-2.456	-2.617 to -2.295	Yes
25009	26:**10 X** vs. 50:**1 X**	-2.359	-2.520 to -2.198	Yes
25010	26:**10 X** vs. 50:**10 X**	-2.354	-2.515 to -2.193	Yes
25011	26:**10 X** vs. 50:**40 X**	0.2680	0.1070 to 0.4290	Yes
25012	26:**10 X** vs. 50:**50 X**	0.5213	0.3604 to 0.6823	Yes
25013	26:**40 X** vs. 26:**50 X**	0.1397	-0.004293 to 0.2836	No
25014	26:**40 X** vs. 27:**0 X**	-0.9427	-1.087 to -0.7987	Yes
25015	26:**40 X** vs. 27:**1 X**	-1.033	-1.194 to -0.8724	Yes
25016	26:**40 X** vs. 27:**10 X**	-0.8453	-1.006 to -0.6844	Yes
25017	26:**40 X** vs. 27:**40 X**	0.0	-0.1440 to 0.1440	No
25018	26:**40 X** vs. 27:**50 X**	0.1403	-0.003626 to 0.2843	No
25019	26:**40 X** vs. 28:**0 X**	-0.8918	-1.053 to -0.7309	Yes
25020	26:**40 X** vs. 28:**1 X**	-0.9327	-1.077 to -0.7887	Yes

	2way ANOVA<br>Multiple comparisons	A<br>Data Set-A<br>Y	B<br>Data Set-B<br>Y	C<br>Data Set-C<br>Y
25021	26:40 X vs. 28:10 X	-0.7343	-0.8953 to -0.5734	Yes
25022	26:40 X vs. 28:40 X	-0.01233	-0.1563 to 0.1316	No
25023	26:40 X vs. 28:50 X	0.09233	-0.05163 to 0.2363	No
25024	26:40 X vs. 29:0 X	-1.373	-1.534 to -1.212	Yes
25025	26:40 X vs. 29:1 X	-1.458	-1.619 to -1.297	Yes
25026	26:40 X vs. 29:10 X	-0.6728	-0.8338 to -0.5119	Yes
25027	26:40 X vs. 29:40 X	-0.1203	-0.2643 to 0.02363	No
25028	26:40 X vs. 29:50 X	0.0910	-0.05296 to 0.2350	No
25029	26:40 X vs. 30:0 X	-1.088	-1.249 to -0.9269	Yes
25030	26:40 X vs. 30:1 X	-1.409	-1.570 to -1.248	Yes
25031	26:40 X vs. 30:10 X	-0.7473	-0.9083 to -0.5864	Yes
25032	26:40 X vs. 30:40 X	-0.04733	-0.1913 to 0.09663	No
25033	26:40 X vs. 30:50 X	0.03867	-0.1053 to 0.1826	No
25034	26:40 X vs. 31:0 X	-1.406	-1.567 to -1.245	Yes
25035	26:40 X vs. 31:1 X	-1.396	-1.557 to -1.235	Yes
25036	26:40 X vs. 31:10 X	-0.8788	-1.040 to -0.7179	Yes
25037	26:40 X vs. 31:40 X	-0.1390	-0.2830 to 0.004960	No
25038	26:40 X vs. 31:50 X	0.05967	-0.08429 to 0.2036	No
25039	26:40 X vs. 32:0 X	-1.997	-2.141 to -1.853	Yes
25040	26:40 X vs. 32:1 X	-1.453	-1.614 to -1.292	Yes
25041	26:40 X vs. 32:10 X	-1.309	-1.453 to -1.165	Yes
25042	26:40 X vs. 32:40 X	-0.1353	-0.2793 to 0.008626	No
25043	26:40 X vs. 32:50 X	0.06000	-0.08396 to 0.2040	No
25044	26:40 X vs. 33:0 X	-2.001	-2.145 to -1.857	Yes
25045	26:40 X vs. 33:1 X	-1.816	-1.960 to -1.672	Yes
25046	26:40 X vs. 33:10 X	-1.476	-1.620 to -1.332	Yes
25047	26:40 X vs. 33:40 X	-0.1540	-0.2980 to -0.01004	Yes
25048	26:40 X vs. 33:50 X	0.04500	-0.09896 to 0.1890	No
25049	26:40 X vs. 34:0 X	-1.926	-2.070 to -1.782	Yes
25050	26:40 X vs. 34:1 X	-1.933	-2.077 to -1.789	Yes
25051	26:40 X vs. 34:10 X	-1.566	-1.710 to -1.422	Yes
25052	26:40 X vs. 34:40 X	-0.1660	-0.3100 to -0.02204	Yes
25053	26:40 X vs. 34:50 X	-0.1040	-0.2480 to 0.03996	No
25054	26:40 X vs. 35:0 X	-2.274	-2.418 to -2.130	Yes
25055	26:40 X vs. 35:1 X	-2.040	-2.184 to -1.896	Yes
25056	26:40 X vs. 35:10 X	-1.528	-1.672 to -1.384	Yes
25057	26:40 X vs. 35:40 X	-0.1750	-0.3190 to -0.03104	Yes
25058	26:40 X vs. 35:50 X	-0.1167	-0.2606 to 0.02729	No
25059	26:40 X vs. 36:0 X	-2.481	-2.642 to -2.320	Yes
25060	26:40 X vs. 36:1 X	-2.177	-2.321 to -2.033	Yes
25061	26:40 X vs. 36:10 X	-1.534	-1.678 to -1.390	Yes
25062	26:40 X vs. 36:40 X	-0.2303	-0.3743 to -0.08637	Yes
25063	26:40 X vs. 36:50 X	-0.08000	-0.2240 to 0.06396	No
25064	26:40 X vs. 37:0 X	-2.865	-3.009 to -2.721	Yes
25065	26:40 X vs. 37:1 X	-2.247	-2.391 to -2.103	Yes

2way ANOVA<br>Multiple comparisons	A<br>Data Set-A<br>Y	B<br>Data Set-B<br>Y	C<br>Data Set-C<br>Y
25066 26:40 X vs. 37:10 X	-1.535	-1.679 to -1.391	Yes
25067 26:40 X vs. 37:40 X	-0.2677	-0.4116 to -0.1237	Yes
25068 26:40 X vs. 37:50 X	-0.08867	-0.2326 to 0.05529	No
25069 26:40 X vs. 38:0 X	-2.810	-2.954 to -2.666	Yes
25070 26:40 X vs. 38:1 X	-2.365	-2.509 to -2.221	Yes
25071 26:40 X vs. 38:10 X	-1.811	-1.955 to -1.667	Yes
25072 26:40 X vs. 38:40 X	-0.2720	-0.4160 to -0.1280	Yes
25073 26:40 X vs. 38:50 X	-0.09267	-0.2366 to 0.05129	No
25074 26:40 X vs. 39:0 X	-3.040	-3.184 to -2.896	Yes
25075 26:40 X vs. 39:1 X	-2.932	-3.076 to -2.788	Yes
25076 26:40 X vs. 39:10 X	-1.956	-2.100 to -1.812	Yes
25077 26:40 X vs. 39:40 X	-0.2780	-0.4220 to -0.1340	Yes
25078 26:40 X vs. 39:50 X	-0.1237	-0.2676 to 0.02029	No
25079 26:40 X vs. 40:0 X	-3.081	-3.225 to -2.937	Yes
25080 26:40 X vs. 40:1 X	-3.007	-3.151 to -2.863	Yes
25081 26:40 X vs. 40:10 X	-2.212	-2.356 to -2.068	Yes
25082 26:40 X vs. 40:40 X	-0.2827	-0.4266 to -0.1387	Yes
25083 26:40 X vs. 40:50 X	-0.1300	-0.2740 to 0.01396	No
25084 26:40 X vs. 41:0 X	-3.122	-3.266 to -2.978	Yes
25085 26:40 X vs. 41:1 X	-3.121	-3.265 to -2.977	Yes
25086 26:40 X vs. 41:10 X	-2.266	-2.410 to -2.122	Yes
25087 26:40 X vs. 41:40 X	-0.2300	-0.3740 to -0.08604	Yes
25088 26:40 X vs. 41:50 X	-0.1427	-0.2866 to 0.001293	No
25089 26:40 X vs. 42:0 X	-3.264	-3.408 to -3.120	Yes
25090 26:40 X vs. 42:1 X	-3.284	-3.428 to -3.140	Yes
25091 26:40 X vs. 42:10 X	-2.266	-2.410 to -2.122	Yes
25092 26:40 X vs. 42:40 X	-0.2427	-0.3866 to -0.09871	Yes
25093 26:40 X vs. 42:50 X	-0.1460	-0.2900 to -0.002040	Yes
25094 26:40 X vs. 43:0 X	-3.354	-3.498 to -3.210	Yes
25095 26:40 X vs. 43:1 X	-3.268	-3.412 to -3.124	Yes
25096 26:40 X vs. 43:10 X	-2.984	-3.128 to -2.840	Yes
25097 26:40 X vs. 43:40 X	-0.2410	-0.3850 to -0.09704	Yes
25098 26:40 X vs. 43:50 X	-0.1463	-0.2903 to -0.002374	Yes
25099 26:40 X vs. 44:0 X	-3.378	-3.522 to -3.234	Yes
25100 26:40 X vs. 44:1 X	-3.268	-3.412 to -3.124	Yes
25101 26:40 X vs. 44:10 X	-3.047	-3.191 to -2.903	Yes
25102 26:40 X vs. 44:40 X	-0.2430	-0.3870 to -0.09904	Yes
25103 26:40 X vs. 44:50 X	-0.07867	-0.2226 to 0.06529	No
25104 26:40 X vs. 45:0 X	-3.243	-3.387 to -3.099	Yes
25105 26:40 X vs. 45:1 X	-3.045	-3.189 to -2.901	Yes
25106 26:40 X vs. 45:10 X	-2.927	-3.071 to -2.783	Yes
25107 26:40 X vs. 45:40 X	-0.2443	-0.3883 to -0.1004	Yes
25108 26:40 X vs. 45:50 X	-0.07567	-0.2196 to 0.06829	No
25109 26:40 X vs. 46:0 X	-3.047	-3.191 to -2.903	Yes
25110 26:40 X vs. 46:1 X	-3.014	-3.158 to -2.870	Yes

2way ANOVA<br>Multiple comparisons	A<br>Data Set-A<br>Y	B<br>Data Set-B<br>Y	C<br>Data Set-C<br>Y
25111 26:40 X vs. 46:10 X	-2.922	-3.066 to -2.778	Yes
25112 26:40 X vs. 46:40 X	-0.2580	-0.4020 to -0.1140	Yes
25113 26:40 X vs. 46:50 X	-0.06867	-0.2126 to 0.07529	No
25114 26:40 X vs. 47:0 X	-3.023	-3.167 to -2.879	Yes
25115 26:40 X vs. 47:1 X	-2.927	-3.071 to -2.783	Yes
25116 26:40 X vs. 47:10 X	-2.908	-3.052 to -2.764	Yes
25117 26:40 X vs. 47:40 X	-0.2680	-0.4120 to -0.1240	Yes
25118 26:40 X vs. 47:50 X	-0.02667	-0.1706 to 0.1173	No
25119 26:40 X vs. 48:0 X	-3.010	-3.171 to -2.849	Yes
25120 26:40 X vs. 48:1 X	-2.914	-3.058 to -2.770	Yes
25121 26:40 X vs. 48:10 X	-2.908	-3.052 to -2.764	Yes
25122 26:40 X vs. 48:40 X	-0.2743	-0.4183 to -0.1304	Yes
25123 26:40 X vs. 48:50 X	-0.0340	-0.1780 to 0.1100	No
25124 26:40 X vs. 49:0 X	-3.010	-3.154 to -2.866	Yes
25125 26:40 X vs. 49:1 X	-2.914	-3.058 to -2.770	Yes
25126 26:40 X vs. 49:10 X	-2.908	-3.052 to -2.764	Yes
25127 26:40 X vs. 49:40 X	-0.2787	-0.4226 to -0.1347	Yes
25128 26:40 X vs. 49:50 X	-0.03333	-0.1773 to 0.1106	No
25129 26:40 X vs. 50:0 X	-3.010	-3.154 to -2.866	Yes
25130 26:40 X vs. 50:1 X	-2.914	-3.058 to -2.770	Yes
25131 26:40 X vs. 50:10 X	-2.908	-3.052 to -2.764	Yes
25132 26:40 X vs. 50:40 X	-0.2863	-0.4303 to -0.1424	Yes
25133 26:40 X vs. 50:50 X	-0.03300	-0.1770 to 0.1110	No
25134 26:50 X vs. 27:0 X	-1.082	-1.226 to -0.9384	Yes
25135 26:50 X vs. 27:1 X	-1.173	-1.334 to -1.012	Yes
25136 26:50 X vs. 27:10 X	-0.9850	-1.146 to -0.8240	Yes
25137 26:50 X vs. 27:40 X	-0.1397	-0.2836 to 0.004293	No
25138 26:50 X vs. 27:50 X	0.0006667	-0.1433 to 0.1446	No
25139 26:50 X vs. 28:0 X	-1.032	-1.192 to -0.8705	Yes
25140 26:50 X vs. 28:1 X	-1.072	-1.216 to -0.9284	Yes
25141 26:50 X vs. 28:10 X	-0.8740	-1.035 to -0.7130	Yes
25142 26:50 X vs. 28:40 X	-0.1520	-0.2960 to -0.008040	Yes
25143 26:50 X vs. 28:50 X	-0.04733	-0.1913 to 0.09663	No
25144 26:50 X vs. 29:0 X	-1.513	-1.674 to -1.352	Yes
25145 26:50 X vs. 29:1 X	-1.598	-1.759 to -1.437	Yes
25146 26:50 X vs. 29:10 X	-0.8125	-0.9735 to -0.6515	Yes
25147 26:50 X vs. 29:40 X	-0.2600	-0.4040 to -0.1160	Yes
25148 26:50 X vs. 29:50 X	-0.04867	-0.1926 to 0.09529	No
25149 26:50 X vs. 30:0 X	-1.228	-1.388 to -1.067	Yes
25150 26:50 X vs. 30:1 X	-1.549	-1.710 to -1.388	Yes
25151 26:50 X vs. 30:10 X	-0.8870	-1.048 to -0.7260	Yes
25152 26:50 X vs. 30:40 X	-0.1870	-0.3310 to -0.04304	Yes
25153 26:50 X vs. 30:50 X	-0.1010	-0.2450 to 0.04296	No
25154 26:50 X vs. 31:0 X	-1.546	-1.706 to -1.385	Yes
25155 26:50 X vs. 31:1 X	-1.536	-1.697 to -1.375	Yes

2way ANOVA<br>Multiple comparisons	A<br>Data Set-A<br>Y	B<br>Data Set-B<br>Y	C<br>Data Set-C<br>Y
25156 26:50 X vs. 31:10 X	-1.019	-1.179 to -0.8575	Yes
25157 26:50 X vs. 31:40 X	-0.2787	-0.4226 to -0.1347	Yes
25158 26:50 X vs. 31:50 X	-0.08000	-0.2240 to 0.06396	No
25159 26:50 X vs. 32:0 X	-2.137	-2.281 to -1.993	Yes
25160 26:50 X vs. 32:1 X	-1.593	-1.754 to -1.432	Yes
25161 26:50 X vs. 32:10 X	-1.449	-1.593 to -1.305	Yes
25162 26:50 X vs. 32:40 X	-0.2750	-0.4190 to -0.1310	Yes
25163 26:50 X vs. 32:50 X	-0.07967	-0.2236 to 0.06429	No
25164 26:50 X vs. 33:0 X	-2.141	-2.285 to -1.997	Yes
25165 26:50 X vs. 33:1 X	-1.955	-2.099 to -1.811	Yes
25166 26:50 X vs. 33:10 X	-1.616	-1.760 to -1.472	Yes
25167 26:50 X vs. 33:40 X	-0.2937	-0.4376 to -0.1497	Yes
25168 26:50 X vs. 33:50 X	-0.09467	-0.2386 to 0.04929	No
25169 26:50 X vs. 34:0 X	-2.065	-2.209 to -1.921	Yes
25170 26:50 X vs. 34:1 X	-2.073	-2.217 to -1.929	Yes
25171 26:50 X vs. 34:10 X	-1.706	-1.850 to -1.562	Yes
25172 26:50 X vs. 34:40 X	-0.3057	-0.4496 to -0.1617	Yes
25173 26:50 X vs. 34:50 X	-0.2437	-0.3876 to -0.09971	Yes
25174 26:50 X vs. 35:0 X	-2.414	-2.558 to -2.270	Yes
25175 26:50 X vs. 35:1 X	-2.180	-2.324 to -2.036	Yes
25176 26:50 X vs. 35:10 X	-1.668	-1.812 to -1.524	Yes
25177 26:50 X vs. 35:40 X	-0.3147	-0.4586 to -0.1707	Yes
25178 26:50 X vs. 35:50 X	-0.2563	-0.4003 to -0.1124	Yes
25179 26:50 X vs. 36:0 X	-2.621	-2.781 to -2.460	Yes
25180 26:50 X vs. 36:1 X	-2.316	-2.460 to -2.172	Yes
25181 26:50 X vs. 36:10 X	-1.673	-1.817 to -1.529	Yes
25182 26:50 X vs. 36:40 X	-0.3700	-0.5140 to -0.2260	Yes
25183 26:50 X vs. 36:50 X	-0.2197	-0.3636 to -0.07571	Yes
25184 26:50 X vs. 37:0 X	-3.004	-3.148 to -2.860	Yes
25185 26:50 X vs. 37:1 X	-2.386	-2.530 to -2.242	Yes
25186 26:50 X vs. 37:10 X	-1.675	-1.819 to -1.531	Yes
25187 26:50 X vs. 37:40 X	-0.4073	-0.5513 to -0.2634	Yes
25188 26:50 X vs. 37:50 X	-0.2283	-0.3723 to -0.08437	Yes
25189 26:50 X vs. 38:0 X	-2.949	-3.093 to -2.805	Yes
25190 26:50 X vs. 38:1 X	-2.505	-2.649 to -2.361	Yes
25191 26:50 X vs. 38:10 X	-1.950	-2.094 to -1.806	Yes
25192 26:50 X vs. 38:40 X	-0.4117	-0.5556 to -0.2677	Yes
25193 26:50 X vs. 38:50 X	-0.2323	-0.3763 to -0.08837	Yes
25194 26:50 X vs. 39:0 X	-3.180	-3.324 to -3.036	Yes
25195 26:50 X vs. 39:1 X	-3.072	-3.216 to -2.928	Yes
25196 26:50 X vs. 39:10 X	-2.095	-2.239 to -1.951	Yes
25197 26:50 X vs. 39:40 X	-0.4177	-0.5616 to -0.2737	Yes
25198 26:50 X vs. 39:50 X	-0.2633	-0.4073 to -0.1194	Yes
25199 26:50 X vs. 40:0 X	-3.221	-3.365 to -3.077	Yes
25200 26:50 X vs. 40:1 X	-3.147	-3.291 to -3.003	Yes

	2way ANOVA<br>Multiple comparisons	A<br>Data Set-A<br>Y	B<br>Data Set-B<br>Y	C<br>Data Set-C<br>Y
25201	26:**50 X** vs. 40:**10 X**	-2.352	-2.496 to -2.208	Yes
25202	26:**50 X** vs. 40:**40 X**	-0.4223	-0.5663 to -0.2784	Yes
25203	26:**50 X** vs. 40:**50 X**	-0.2697	-0.4136 to -0.1257	Yes
25204	26:**50 X** vs. 41:**0 X**	-3.262	-3.406 to -3.118	Yes
25205	26:**50 X** vs. 41:**1 X**	-3.261	-3.405 to -3.117	Yes
25206	26:**50 X** vs. 41:**10 X**	-2.405	-2.549 to -2.261	Yes
25207	26:**50 X** vs. 41:**40 X**	-0.3697	-0.5136 to -0.2257	Yes
25208	26:**50 X** vs. 41:**50 X**	-0.2823	-0.4263 to -0.1384	Yes
25209	26:**50 X** vs. 42:**0 X**	-3.404	-3.548 to -3.260	Yes
25210	26:**50 X** vs. 42:**1 X**	-3.423	-3.567 to -3.279	Yes
25211	26:**50 X** vs. 42:**10 X**	-2.406	-2.550 to -2.262	Yes
25212	26:**50 X** vs. 42:**40 X**	-0.3823	-0.5263 to -0.2384	Yes
25213	26:**50 X** vs. 42:**50 X**	-0.2857	-0.4296 to -0.1417	Yes
25214	26:**50 X** vs. 43:**0 X**	-3.494	-3.638 to -3.350	Yes
25215	26:**50 X** vs. 43:**1 X**	-3.408	-3.552 to -3.264	Yes
25216	26:**50 X** vs. 43:**10 X**	-3.124	-3.268 to -2.980	Yes
25217	26:**50 X** vs. 43:**40 X**	-0.3807	-0.5246 to -0.2367	Yes
25218	26:**50 X** vs. 43:**50 X**	-0.2860	-0.4300 to -0.1420	Yes
25219	26:**50 X** vs. 44:**0 X**	-3.518	-3.662 to -3.374	Yes
25220	26:**50 X** vs. 44:**1 X**	-3.407	-3.551 to -3.263	Yes
25221	26:**50 X** vs. 44:**10 X**	-3.187	-3.331 to -3.043	Yes
25222	26:**50 X** vs. 44:**40 X**	-0.3827	-0.5266 to -0.2387	Yes
25223	26:**50 X** vs. 44:**50 X**	-0.2183	-0.3623 to -0.07437	Yes
25224	26:**50 X** vs. 45:**0 X**	-3.382	-3.526 to -3.238	Yes
25225	26:**50 X** vs. 45:**1 X**	-3.184	-3.328 to -3.040	Yes
25226	26:**50 X** vs. 45:**10 X**	-3.067	-3.211 to -2.923	Yes
25227	26:**50 X** vs. 45:**40 X**	-0.3840	-0.5280 to -0.2400	Yes
25228	26:**50 X** vs. 45:**50 X**	-0.2153	-0.3593 to -0.07137	Yes
25229	26:**50 X** vs. 46:**0 X**	-3.187	-3.331 to -3.043	Yes
25230	26:**50 X** vs. 46:**1 X**	-3.154	-3.298 to -3.010	Yes
25231	26:**50 X** vs. 46:**10 X**	-3.062	-3.206 to -2.918	Yes
25232	26:**50 X** vs. 46:**40 X**	-0.3977	-0.5416 to -0.2537	Yes
25233	26:**50 X** vs. 46:**50 X**	-0.2083	-0.3523 to -0.06437	Yes
25234	26:**50 X** vs. 47:**0 X**	-3.162	-3.306 to -3.018	Yes
25235	26:**50 X** vs. 47:**1 X**	-3.066	-3.210 to -2.922	Yes
25236	26:**50 X** vs. 47:**10 X**	-3.048	-3.192 to -2.904	Yes
25237	26:**50 X** vs. 47:**40 X**	-0.4077	-0.5516 to -0.2637	Yes
25238	26:**50 X** vs. 47:**50 X**	-0.1663	-0.3103 to -0.02237	Yes
25239	26:**50 X** vs. 48:**0 X**	-3.150	-3.310 to -2.989	Yes
25240	26:**50 X** vs. 48:**1 X**	-3.053	-3.197 to -2.909	Yes
25241	26:**50 X** vs. 48:**10 X**	-3.048	-3.192 to -2.904	Yes
25242	26:**50 X** vs. 48:**40 X**	-0.4140	-0.5580 to -0.2700	Yes
25243	26:**50 X** vs. 48:**50 X**	-0.1737	-0.3176 to -0.02971	Yes
25244	26:**50 X** vs. 49:**0 X**	-3.150	-3.294 to -3.006	Yes
25245	26:**50 X** vs. 49:**1 X**	-3.053	-3.197 to -2.909	Yes

	2way ANOVA<br>Multiple comparisons	A<br>Data Set-A<br>Y	B<br>Data Set-B<br>Y	C<br>Data Set-C<br>Y
25246	26:**50 X** vs. 49:**10 X**	-3.048	-3.192 to -2.904	Yes
25247	26:**50 X** vs. 49:**40 X**	-0.4183	-0.5623 to -0.2744	Yes
25248	26:**50 X** vs. 49:**50 X**	-0.1730	-0.3170 to -0.02904	Yes
25249	26:**50 X** vs. 50:**0 X**	-3.150	-3.294 to -3.006	Yes
25250	26:**50 X** vs. 50:**1 X**	-3.053	-3.197 to -2.909	Yes
25251	26:**50 X** vs. 50:**10 X**	-3.048	-3.192 to -2.904	Yes
25252	26:**50 X** vs. 50:**40 X**	-0.4260	-0.5700 to -0.2820	Yes
25253	26:**50 X** vs. 50:**50 X**	-0.1727	-0.3166 to -0.02871	Yes
25254	27:**0 X** vs. 27:**1 X**	-0.09067	-0.2516 to 0.07029	No
25255	27:**0 X** vs. 27:**10 X**	0.09733	-0.06362 to 0.2583	No
25256	27:**0 X** vs. 27:**40 X**	0.9427	0.7987 to 1.087	Yes
25257	27:**0 X** vs. 27:**50 X**	1.083	0.9390 to 1.227	Yes
25258	27:**0 X** vs. 28:**0 X**	0.05083	-0.1101 to 0.2118	No
25259	27:**0 X** vs. 28:**1 X**	0.01000	-0.1340 to 0.1540	No
25260	27:**0 X** vs. 28:**10 X**	0.2083	0.04738 to 0.3693	Yes
25261	27:**0 X** vs. 28:**40 X**	0.9303	0.7864 to 1.074	Yes
25262	27:**0 X** vs. 28:**50 X**	1.035	0.8910 to 1.179	Yes
25263	27:**0 X** vs. 29:**0 X**	-0.4307	-0.5916 to -0.2697	Yes
25264	27:**0 X** vs. 29:**1 X**	-0.5157	-0.6766 to -0.3547	Yes
25265	27:**0 X** vs. 29:**10 X**	0.2698	0.1089 to 0.4308	Yes
25266	27:**0 X** vs. 29:**40 X**	0.8223	0.6784 to 0.9663	Yes
25267	27:**0 X** vs. 29:**50 X**	1.034	0.8897 to 1.178	Yes
25268	27:**0 X** vs. 30:**0 X**	-0.1452	-0.3061 to 0.01579	No
25269	27:**0 X** vs. 30:**1 X**	-0.4667	-0.6276 to -0.3057	Yes
25270	27:**0 X** vs. 30:**10 X**	0.1953	0.03438 to 0.3563	Yes
25271	27:**0 X** vs. 30:**40 X**	0.8953	0.7514 to 1.039	Yes
25272	27:**0 X** vs. 30:**50 X**	0.9813	0.8374 to 1.125	Yes
25273	27:**0 X** vs. 31:**0 X**	-0.4632	-0.6241 to -0.3022	Yes
25274	27:**0 X** vs. 31:**1 X**	-0.4537	-0.6146 to -0.2927	Yes
25275	27:**0 X** vs. 31:**10 X**	0.06383	-0.09712 to 0.2248	No
25276	27:**0 X** vs. 31:**40 X**	0.8037	0.6597 to 0.9476	Yes
25277	27:**0 X** vs. 31:**50 X**	1.002	0.8584 to 1.146	Yes
25278	27:**0 X** vs. 32:**0 X**	-1.055	-1.199 to -0.9107	Yes
25279	27:**0 X** vs. 32:**1 X**	-0.5107	-0.6716 to -0.3497	Yes
25280	27:**0 X** vs. 32:**10 X**	-0.3667	-0.5106 to -0.2227	Yes
25281	27:**0 X** vs. 32:**40 X**	0.8073	0.6634 to 0.9513	Yes
25282	27:**0 X** vs. 32:**50 X**	1.003	0.8587 to 1.147	Yes
25283	27:**0 X** vs. 33:**0 X**	-1.058	-1.202 to -0.9144	Yes
25284	27:**0 X** vs. 33:**1 X**	-0.8730	-1.017 to -0.7290	Yes
25285	27:**0 X** vs. 33:**10 X**	-0.5333	-0.6773 to -0.3894	Yes
25286	27:**0 X** vs. 33:**40 X**	0.7887	0.6447 to 0.9326	Yes
25287	27:**0 X** vs. 33:**50 X**	0.9877	0.8437 to 1.132	Yes
25288	27:**0 X** vs. 34:**0 X**	-0.9830	-1.127 to -0.8390	Yes
25289	27:**0 X** vs. 34:**1 X**	-0.9903	-1.134 to -0.8464	Yes
25290	27:**0 X** vs. 34:**10 X**	-0.6237	-0.7676 to -0.4797	Yes

	2way ANOVA<br>Multiple comparisons	A<br>Data Set-A<br>Y	B<br>Data Set-B<br>Y	C<br>Data Set-C<br>Y
25291	27:**0 X** vs. 34:**40 X**	0.7767	0.6327 to 0.9206	Yes
25292	27:**0 X** vs. 34:**50 X**	0.8387	0.6947 to 0.9826	Yes
25293	27:**0 X** vs. 35:**0 X**	-1.332	-1.476 to -1.188	Yes
25294	27:**0 X** vs. 35:**1 X**	-1.097	-1.241 to -0.9534	Yes
25295	27:**0 X** vs. 35:**10 X**	-0.5853	-0.7293 to -0.4414	Yes
25296	27:**0 X** vs. 35:**40 X**	0.7677	0.6237 to 0.9116	Yes
25297	27:**0 X** vs. 35:**50 X**	0.8260	0.6820 to 0.9700	Yes
25298	27:**0 X** vs. 36:**0 X**	-1.538	-1.699 to -1.377	Yes
25299	27:**0 X** vs. 36:**1 X**	-1.234	-1.378 to -1.090	Yes
25300	27:**0 X** vs. 36:**10 X**	-0.5910	-0.7350 to -0.4470	Yes
25301	27:**0 X** vs. 36:**40 X**	0.7123	0.5684 to 0.8563	Yes
25302	27:**0 X** vs. 36:**50 X**	0.8627	0.7187 to 1.007	Yes
25303	27:**0 X** vs. 37:**0 X**	-1.922	-2.066 to -1.778	Yes
25304	27:**0 X** vs. 37:**1 X**	-1.304	-1.448 to -1.160	Yes
25305	27:**0 X** vs. 37:**10 X**	-0.5927	-0.7366 to -0.4487	Yes
25306	27:**0 X** vs. 37:**40 X**	0.6750	0.5310 to 0.8190	Yes
25307	27:**0 X** vs. 37:**50 X**	0.8540	0.7100 to 0.9980	Yes
25308	27:**0 X** vs. 38:**0 X**	-1.867	-2.011 to -1.723	Yes
25309	27:**0 X** vs. 38:**1 X**	-1.423	-1.567 to -1.279	Yes
25310	27:**0 X** vs. 38:**10 X**	-0.8680	-1.012 to -0.7240	Yes
25311	27:**0 X** vs. 38:**40 X**	0.6707	0.5267 to 0.8146	Yes
25312	27:**0 X** vs. 38:**50 X**	0.8500	0.7060 to 0.9940	Yes
25313	27:**0 X** vs. 39:**0 X**	-2.098	-2.242 to -1.954	Yes
25314	27:**0 X** vs. 39:**1 X**	-1.989	-2.133 to -1.845	Yes
25315	27:**0 X** vs. 39:**10 X**	-1.013	-1.157 to -0.8690	Yes
25316	27:**0 X** vs. 39:**40 X**	0.6647	0.5207 to 0.8086	Yes
25317	27:**0 X** vs. 39:**50 X**	0.8190	0.6750 to 0.9630	Yes
25318	27:**0 X** vs. 40:**0 X**	-2.139	-2.283 to -1.995	Yes
25319	27:**0 X** vs. 40:**1 X**	-2.065	-2.209 to -1.921	Yes
25320	27:**0 X** vs. 40:**10 X**	-1.269	-1.413 to -1.125	Yes
25321	27:**0 X** vs. 40:**40 X**	0.6600	0.5160 to 0.8040	Yes
25322	27:**0 X** vs. 40:**50 X**	0.8127	0.6687 to 0.9566	Yes
25323	27:**0 X** vs. 41:**0 X**	-2.180	-2.324 to -2.036	Yes
25324	27:**0 X** vs. 41:**1 X**	-2.179	-2.323 to -2.035	Yes
25325	27:**0 X** vs. 41:**10 X**	-1.323	-1.467 to -1.179	Yes
25326	27:**0 X** vs. 41:**40 X**	0.7127	0.5687 to 0.8566	Yes
25327	27:**0 X** vs. 41:**50 X**	0.8000	0.6560 to 0.9440	Yes
25328	27:**0 X** vs. 42:**0 X**	-2.321	-2.465 to -2.177	Yes
25329	27:**0 X** vs. 42:**1 X**	-2.341	-2.485 to -2.197	Yes
25330	27:**0 X** vs. 42:**10 X**	-1.323	-1.467 to -1.179	Yes
25331	27:**0 X** vs. 42:**40 X**	0.7000	0.5560 to 0.8440	Yes
25332	27:**0 X** vs. 42:**50 X**	0.7967	0.6527 to 0.9406	Yes
25333	27:**0 X** vs. 43:**0 X**	-2.411	-2.555 to -2.267	Yes
25334	27:**0 X** vs. 43:**1 X**	-2.326	-2.470 to -2.182	Yes
25335	27:**0 X** vs. 43:**10 X**	-2.041	-2.185 to -1.897	Yes

	2way ANOVA Multiple comparisons	A Data Set-A Y	B Data Set-B Y	C Data Set-C Y
25336	27:**0 X** vs. 43:**40 X**	0.7017	0.5577 to 0.8456	Yes
25337	27:**0 X** vs. 43:**50 X**	0.7963	0.6524 to 0.9403	Yes
25338	27:**0 X** vs. 44:**0 X**	-2.435	-2.579 to -2.291	Yes
25339	27:**0 X** vs. 44:**1 X**	-2.325	-2.469 to -2.181	Yes
25340	27:**0 X** vs. 44:**10 X**	-2.105	-2.249 to -1.961	Yes
25341	27:**0 X** vs. 44:**40 X**	0.6997	0.5557 to 0.8436	Yes
25342	27:**0 X** vs. 44:**50 X**	0.8640	0.7200 to 1.008	Yes
25343	27:**0 X** vs. 45:**0 X**	-2.300	-2.444 to -2.156	Yes
25344	27:**0 X** vs. 45:**1 X**	-2.102	-2.246 to -1.958	Yes
25345	27:**0 X** vs. 45:**10 X**	-1.984	-2.128 to -1.840	Yes
25346	27:**0 X** vs. 45:**40 X**	0.6983	0.5544 to 0.8423	Yes
25347	27:**0 X** vs. 45:**50 X**	0.8670	0.7230 to 1.011	Yes
25348	27:**0 X** vs. 46:**0 X**	-2.104	-2.248 to -1.960	Yes
25349	27:**0 X** vs. 46:**1 X**	-2.071	-2.215 to -1.927	Yes
25350	27:**0 X** vs. 46:**10 X**	-1.979	-2.123 to -1.835	Yes
25351	27:**0 X** vs. 46:**40 X**	0.6847	0.5407 to 0.8286	Yes
25352	27:**0 X** vs. 46:**50 X**	0.8740	0.7300 to 1.018	Yes
25353	27:**0 X** vs. 47:**0 X**	-2.080	-2.224 to -1.936	Yes
25354	27:**0 X** vs. 47:**1 X**	-1.984	-2.128 to -1.840	Yes
25355	27:**0 X** vs. 47:**10 X**	-1.965	-2.109 to -1.821	Yes
25356	27:**0 X** vs. 47:**40 X**	0.6747	0.5307 to 0.8186	Yes
25357	27:**0 X** vs. 47:**50 X**	0.9160	0.7720 to 1.060	Yes
25358	27:**0 X** vs. 48:**0 X**	-2.067	-2.228 to -1.906	Yes
25359	27:**0 X** vs. 48:**1 X**	-1.971	-2.115 to -1.827	Yes
25360	27:**0 X** vs. 48:**10 X**	-1.965	-2.109 to -1.821	Yes
25361	27:**0 X** vs. 48:**40 X**	0.6683	0.5244 to 0.8123	Yes
25362	27:**0 X** vs. 48:**50 X**	0.9087	0.7647 to 1.053	Yes
25363	27:**0 X** vs. 49:**0 X**	-2.067	-2.211 to -1.923	Yes
25364	27:**0 X** vs. 49:**1 X**	-1.971	-2.115 to -1.827	Yes
25365	27:**0 X** vs. 49:**10 X**	-1.965	-2.109 to -1.821	Yes
25366	27:**0 X** vs. 49:**40 X**	0.6640	0.5200 to 0.8080	Yes
25367	27:**0 X** vs. 49:**50 X**	0.9093	0.7654 to 1.053	Yes
25368	27:**0 X** vs. 50:**0 X**	-2.067	-2.211 to -1.923	Yes
25369	27:**0 X** vs. 50:**1 X**	-1.971	-2.115 to -1.827	Yes
25370	27:**0 X** vs. 50:**10 X**	-1.965	-2.109 to -1.821	Yes
25371	27:**0 X** vs. 50:**40 X**	0.6563	0.5124 to 0.8003	Yes
25372	27:**0 X** vs. 50:**50 X**	0.9097	0.7657 to 1.054	Yes
25373	27:**1 X** vs. 27:**10 X**	0.1880	0.01169 to 0.3643	Yes
25374	27:**1 X** vs. 27:**40 X**	1.033	0.8724 to 1.194	Yes
25375	27:**1 X** vs. 27:**50 X**	1.174	1.013 to 1.335	Yes
25376	27:**1 X** vs. 28:**0 X**	0.1415	-0.03481 to 0.3178	No
25377	27:**1 X** vs. 28:**1 X**	0.1007	-0.06029 to 0.2616	No
25378	27:**1 X** vs. 28:**10 X**	0.2990	0.1227 to 0.4753	Yes
25379	27:**1 X** vs. 28:**40 X**	1.021	0.8600 to 1.182	Yes
25380	27:**1 X** vs. 28:**50 X**	1.126	0.9647 to 1.287	Yes

2way ANOVA<br>Multiple comparisons	A<br>Data Set-A<br>Y	B<br>Data Set-B<br>Y	C<br>Data Set-C<br>Y
25381 27:1 X vs. 29:0 X	-0.3400	-0.5163 to -0.1637	Yes
25382 27:1 X vs. 29:1 X	-0.4250	-0.6013 to -0.2487	Yes
25383 27:1 X vs. 29:10 X	0.3605	0.1842 to 0.5368	Yes
25384 27:1 X vs. 29:40 X	0.9130	0.7520 to 1.074	Yes
25385 27:1 X vs. 29:50 X	1.124	0.9634 to 1.285	Yes
25386 27:1 X vs. 30:0 X	-0.05450	-0.2308 to 0.1218	No
25387 27:1 X vs. 30:1 X	-0.3760	-0.5523 to -0.1997	Yes
25388 27:1 X vs. 30:10 X	0.2860	0.1097 to 0.4623	Yes
25389 27:1 X vs. 30:40 X	0.9860	0.8250 to 1.147	Yes
25390 27:1 X vs. 30:50 X	1.072	0.9110 to 1.233	Yes
25391 27:1 X vs. 31:0 X	-0.3725	-0.5488 to -0.1962	Yes
25392 27:1 X vs. 31:1 X	-0.3630	-0.5393 to -0.1867	Yes
25393 27:1 X vs. 31:10 X	0.1545	-0.02181 to 0.3308	No
25394 27:1 X vs. 31:40 X	0.8943	0.7334 to 1.055	Yes
25395 27:1 X vs. 31:50 X	1.093	0.9320 to 1.254	Yes
25396 27:1 X vs. 32:0 X	-0.9640	-1.125 to -0.8030	Yes
25397 27:1 X vs. 32:1 X	-0.4200	-0.5963 to -0.2437	Yes
25398 27:1 X vs. 32:10 X	-0.2760	-0.4370 to -0.1150	Yes
25399 27:1 X vs. 32:40 X	0.8980	0.7370 to 1.059	Yes
25400 27:1 X vs. 32:50 X	1.093	0.9324 to 1.254	Yes
25401 27:1 X vs. 33:0 X	-0.9677	-1.129 to -0.8067	Yes
25402 27:1 X vs. 33:1 X	-0.7823	-0.9433 to -0.6214	Yes
25403 27:1 X vs. 33:10 X	-0.4427	-0.6036 to -0.2817	Yes
25404 27:1 X vs. 33:40 X	0.8793	0.7184 to 1.040	Yes
25405 27:1 X vs. 33:50 X	1.078	0.9174 to 1.239	Yes
25406 27:1 X vs. 34:0 X	-0.8923	-1.053 to -0.7314	Yes
25407 27:1 X vs. 34:1 X	-0.8997	-1.061 to -0.7387	Yes
25408 27:1 X vs. 34:10 X	-0.5330	-0.6940 to -0.3720	Yes
25409 27:1 X vs. 34:40 X	0.8673	0.7064 to 1.028	Yes
25410 27:1 X vs. 34:50 X	0.9293	0.7684 to 1.090	Yes
25411 27:1 X vs. 35:0 X	-1.241	-1.402 to -1.080	Yes
25412 27:1 X vs. 35:1 X	-1.007	-1.168 to -0.8457	Yes
25413 27:1 X vs. 35:10 X	-0.4947	-0.6556 to -0.3337	Yes
25414 27:1 X vs. 35:40 X	0.8583	0.6974 to 1.019	Yes
25415 27:1 X vs. 35:50 X	0.9167	0.7557 to 1.078	Yes
25416 27:1 X vs. 36:0 X	-1.448	-1.624 to -1.271	Yes
25417 27:1 X vs. 36:1 X	-1.143	-1.304 to -0.9824	Yes
25418 27:1 X vs. 36:10 X	-0.5003	-0.6613 to -0.3394	Yes
25419 27:1 X vs. 36:40 X	0.8030	0.6420 to 0.9640	Yes
25420 27:1 X vs. 36:50 X	0.9533	0.7924 to 1.114	Yes
25421 27:1 X vs. 37:0 X	-1.831	-1.992 to -1.670	Yes
25422 27:1 X vs. 37:1 X	-1.213	-1.374 to -1.052	Yes
25423 27:1 X vs. 37:10 X	-0.5020	-0.6630 to -0.3410	Yes
25424 27:1 X vs. 37:40 X	0.7657	0.6047 to 0.9266	Yes
25425 27:1 X vs. 37:50 X	0.9447	0.7837 to 1.106	Yes

	2way ANOVA Multiple comparisons	A Data Set-A Y	B Data Set-B Y	C Data Set-C Y
25426	27:1 X vs. 38:0 X	-1.776	-1.937 to -1.615	Yes
25427	27:1 X vs. 38:1 X	-1.332	-1.493 to -1.171	Yes
25428	27:1 X vs. 38:10 X	-0.7773	-0.9383 to -0.6164	Yes
25429	27:1 X vs. 38:40 X	0.7613	0.6004 to 0.9223	Yes
25430	27:1 X vs. 38:50 X	0.9407	0.7797 to 1.102	Yes
25431	27:1 X vs. 39:0 X	-2.007	-2.168 to -1.846	Yes
25432	27:1 X vs. 39:1 X	-1.899	-2.060 to -1.738	Yes
25433	27:1 X vs. 39:10 X	-0.9223	-1.083 to -0.7614	Yes
25434	27:1 X vs. 39:40 X	0.7553	0.5944 to 0.9163	Yes
25435	27:1 X vs. 39:50 X	0.9097	0.7487 to 1.071	Yes
25436	27:1 X vs. 40:0 X	-2.048	-2.209 to -1.887	Yes
25437	27:1 X vs. 40:1 X	-1.974	-2.135 to -1.813	Yes
25438	27:1 X vs. 40:10 X	-1.179	-1.340 to -1.018	Yes
25439	27:1 X vs. 40:40 X	0.7507	0.5897 to 0.9116	Yes
25440	27:1 X vs. 40:50 X	0.9033	0.7424 to 1.064	Yes
25441	27:1 X vs. 41:0 X	-2.089	-2.250 to -1.928	Yes
25442	27:1 X vs. 41:1 X	-2.088	-2.249 to -1.927	Yes
25443	27:1 X vs. 41:10 X	-1.232	-1.393 to -1.071	Yes
25444	27:1 X vs. 41:40 X	0.8033	0.6424 to 0.9643	Yes
25445	27:1 X vs. 41:50 X	0.8907	0.7297 to 1.052	Yes
25446	27:1 X vs. 42:0 X	-2.231	-2.392 to -2.070	Yes
25447	27:1 X vs. 42:1 X	-2.250	-2.411 to -2.089	Yes
25448	27:1 X vs. 42:10 X	-1.233	-1.394 to -1.072	Yes
25449	27:1 X vs. 42:40 X	0.7907	0.6297 to 0.9516	Yes
25450	27:1 X vs. 42:50 X	0.8873	0.7264 to 1.048	Yes
25451	27:1 X vs. 43:0 X	-2.321	-2.482 to -2.160	Yes
25452	27:1 X vs. 43:1 X	-2.235	-2.396 to -2.074	Yes
25453	27:1 X vs. 43:10 X	-1.951	-2.112 to -1.790	Yes
25454	27:1 X vs. 43:40 X	0.7923	0.6314 to 0.9533	Yes
25455	27:1 X vs. 43:50 X	0.8870	0.7260 to 1.048	Yes
25456	27:1 X vs. 44:0 X	-2.345	-2.506 to -2.184	Yes
25457	27:1 X vs. 44:1 X	-2.234	-2.395 to -2.073	Yes
25458	27:1 X vs. 44:10 X	-2.014	-2.175 to -1.853	Yes
25459	27:1 X vs. 44:40 X	0.7903	0.6294 to 0.9513	Yes
25460	27:1 X vs. 44:50 X	0.9547	0.7937 to 1.116	Yes
25461	27:1 X vs. 45:0 X	-2.209	-2.370 to -2.048	Yes
25462	27:1 X vs. 45:1 X	-2.011	-2.172 to -1.850	Yes
25463	27:1 X vs. 45:10 X	-1.894	-2.055 to -1.733	Yes
25464	27:1 X vs. 45:40 X	0.7890	0.6280 to 0.9500	Yes
25465	27:1 X vs. 45:50 X	0.9577	0.7967 to 1.119	Yes
25466	27:1 X vs. 46:0 X	-2.014	-2.175 to -1.853	Yes
25467	27:1 X vs. 46:1 X	-1.981	-2.142 to -1.820	Yes
25468	27:1 X vs. 46:10 X	-1.889	-2.050 to -1.728	Yes
25469	27:1 X vs. 46:40 X	0.7753	0.6144 to 0.9363	Yes
25470	27:1 X vs. 46:50 X	0.9647	0.8037 to 1.126	Yes

	2way ANOVA<br>Multiple comparisons	A<br>Data Set-A<br>Y	B<br>Data Set-B<br>Y	C<br>Data Set-C<br>Y
25471	27:1 X vs. 47:0 X	-1.989	-2.150 to -1.828	Yes
25472	27:1 X vs. 47:1 X	-1.893	-2.054 to -1.732	Yes
25473	27:1 X vs. 47:10 X	-1.875	-2.036 to -1.714	Yes
25474	27:1 X vs. 47:40 X	0.7653	0.6044 to 0.9263	Yes
25475	27:1 X vs. 47:50 X	1.007	0.8457 to 1.168	Yes
25476	27:1 X vs. 48:0 X	-1.977	-2.153 to -1.800	Yes
25477	27:1 X vs. 48:1 X	-1.880	-2.041 to -1.719	Yes
25478	27:1 X vs. 48:10 X	-1.875	-2.036 to -1.714	Yes
25479	27:1 X vs. 48:40 X	0.7590	0.5980 to 0.9200	Yes
25480	27:1 X vs. 48:50 X	0.9993	0.8384 to 1.160	Yes
25481	27:1 X vs. 49:0 X	-1.977	-2.138 to -1.816	Yes
25482	27:1 X vs. 49:1 X	-1.880	-2.041 to -1.719	Yes
25483	27:1 X vs. 49:10 X	-1.875	-2.036 to -1.714	Yes
25484	27:1 X vs. 49:40 X	0.7547	0.5937 to 0.9156	Yes
25485	27:1 X vs. 49:50 X	1.000	0.8390 to 1.161	Yes
25486	27:1 X vs. 50:0 X	-1.977	-2.138 to -1.816	Yes
25487	27:1 X vs. 50:1 X	-1.880	-2.041 to -1.719	Yes
25488	27:1 X vs. 50:10 X	-1.875	-2.036 to -1.714	Yes
25489	27:1 X vs. 50:40 X	0.7470	0.5860 to 0.9080	Yes
25490	27:1 X vs. 50:50 X	1.000	0.8394 to 1.161	Yes
25491	27:10 X vs. 27:40 X	0.8453	0.6844 to 1.006	Yes
25492	27:10 X vs. 27:50 X	0.9857	0.8247 to 1.147	Yes
25493	27:10 X vs. 28:0 X	-0.04650	-0.2228 to 0.1298	No
25494	27:10 X vs. 28:1 X	-0.08733	-0.2483 to 0.07362	No
25495	27:10 X vs. 28:10 X	0.1110	-0.06531 to 0.2873	No
25496	27:10 X vs. 28:40 X	0.8330	0.6720 to 0.9940	Yes
25497	27:10 X vs. 28:50 X	0.9377	0.7767 to 1.099	Yes
25498	27:10 X vs. 29:0 X	-0.5280	-0.7043 to -0.3517	Yes
25499	27:10 X vs. 29:1 X	-0.6130	-0.7893 to -0.4367	Yes
25500	27:10 X vs. 29:10 X	0.1725	-0.003814 to 0.3488	No
25501	27:10 X vs. 29:40 X	0.7250	0.5640 to 0.8860	Yes
25502	27:10 X vs. 29:50 X	0.9363	0.7754 to 1.097	Yes
25503	27:10 X vs. 30:0 X	-0.2425	-0.4188 to -0.06619	Yes
25504	27:10 X vs. 30:1 X	-0.5640	-0.7403 to -0.3877	Yes
25505	27:10 X vs. 30:10 X	0.09800	-0.07831 to 0.2743	No
25506	27:10 X vs. 30:40 X	0.7980	0.6370 to 0.9590	Yes
25507	27:10 X vs. 30:50 X	0.8840	0.7230 to 1.045	Yes
25508	27:10 X vs. 31:0 X	-0.5605	-0.7368 to -0.3842	Yes
25509	27:10 X vs. 31:1 X	-0.5510	-0.7273 to -0.3747	Yes
25510	27:10 X vs. 31:10 X	-0.03350	-0.2098 to 0.1428	No
25511	27:10 X vs. 31:40 X	0.7063	0.5454 to 0.8673	Yes
25512	27:10 X vs. 31:50 X	0.9050	0.7440 to 1.066	Yes
25513	27:10 X vs. 32:0 X	-1.152	-1.313 to -0.9910	Yes
25514	27:10 X vs. 32:1 X	-0.6080	-0.7843 to -0.4317	Yes
25515	27:10 X vs. 32:10 X	-0.4640	-0.6250 to -0.3030	Yes

	2way ANOVA<br>Multiple comparisons	A<br>Data Set-A<br>Y	B<br>Data Set-B<br>Y	C<br>Data Set-C<br>Y
25516	27:10 X vs. 32:40 X	0.7100	0.5490 to 0.8710	Yes
25517	27:10 X vs. 32:50 X	0.9053	0.7444 to 1.066	Yes
25518	27:10 X vs. 33:0 X	-1.156	-1.317 to -0.9947	Yes
25519	27:10 X vs. 33:1 X	-0.9703	-1.131 to -0.8094	Yes
25520	27:10 X vs. 33:10 X	-0.6307	-0.7916 to -0.4697	Yes
25521	27:10 X vs. 33:40 X	0.6913	0.5304 to 0.8523	Yes
25522	27:10 X vs. 33:50 X	0.8903	0.7294 to 1.051	Yes
25523	27:10 X vs. 34:0 X	-1.080	-1.241 to -0.9194	Yes
25524	27:10 X vs. 34:1 X	-1.088	-1.249 to -0.9267	Yes
25525	27:10 X vs. 34:10 X	-0.7210	-0.8820 to -0.5600	Yes
25526	27:10 X vs. 34:40 X	0.6793	0.5184 to 0.8403	Yes
25527	27:10 X vs. 34:50 X	0.7413	0.5804 to 0.9023	Yes
25528	27:10 X vs. 35:0 X	-1.429	-1.590 to -1.268	Yes
25529	27:10 X vs. 35:1 X	-1.195	-1.356 to -1.034	Yes
25530	27:10 X vs. 35:10 X	-0.6827	-0.8436 to -0.5217	Yes
25531	27:10 X vs. 35:40 X	0.6703	0.5094 to 0.8313	Yes
25532	27:10 X vs. 35:50 X	0.7287	0.5677 to 0.8896	Yes
25533	27:10 X vs. 36:0 X	-1.636	-1.812 to -1.459	Yes
25534	27:10 X vs. 36:1 X	-1.331	-1.492 to -1.170	Yes
25535	27:10 X vs. 36:10 X	-0.6883	-0.8493 to -0.5274	Yes
25536	27:10 X vs. 36:40 X	0.6150	0.4540 to 0.7760	Yes
25537	27:10 X vs. 36:50 X	0.7653	0.6044 to 0.9263	Yes
25538	27:10 X vs. 37:0 X	-2.019	-2.180 to -1.858	Yes
25539	27:10 X vs. 37:1 X	-1.401	-1.562 to -1.240	Yes
25540	27:10 X vs. 37:10 X	-0.6900	-0.8510 to -0.5290	Yes
25541	27:10 X vs. 37:40 X	0.5777	0.4167 to 0.7386	Yes
25542	27:10 X vs. 37:50 X	0.7567	0.5957 to 0.9176	Yes
25543	27:10 X vs. 38:0 X	-1.964	-2.125 to -1.803	Yes
25544	27:10 X vs. 38:1 X	-1.520	-1.681 to -1.359	Yes
25545	27:10 X vs. 38:10 X	-0.9653	-1.126 to -0.8044	Yes
25546	27:10 X vs. 38:40 X	0.5733	0.4124 to 0.7343	Yes
25547	27:10 X vs. 38:50 X	0.7527	0.5917 to 0.9136	Yes
25548	27:10 X vs. 39:0 X	-2.195	-2.356 to -2.034	Yes
25549	27:10 X vs. 39:1 X	-2.087	-2.248 to -1.926	Yes
25550	27:10 X vs. 39:10 X	-1.110	-1.271 to -0.9494	Yes
25551	27:10 X vs. 39:40 X	0.5673	0.4064 to 0.7283	Yes
25552	27:10 X vs. 39:50 X	0.7217	0.5607 to 0.8826	Yes
25553	27:10 X vs. 40:0 X	-2.236	-2.397 to -2.075	Yes
25554	27:10 X vs. 40:1 X	-2.162	-2.323 to -2.001	Yes
25555	27:10 X vs. 40:10 X	-1.367	-1.528 to -1.206	Yes
25556	27:10 X vs. 40:40 X	0.5627	0.4017 to 0.7236	Yes
25557	27:10 X vs. 40:50 X	0.7153	0.5544 to 0.8763	Yes
25558	27:10 X vs. 41:0 X	-2.277	-2.438 to -2.116	Yes
25559	27:10 X vs. 41:1 X	-2.276	-2.437 to -2.115	Yes
25560	27:10 X vs. 41:10 X	-1.420	-1.581 to -1.259	Yes

	2way ANOVA<br>Multiple comparisons	A<br>Data Set-A<br>Y	B<br>Data Set-B<br>Y	C<br>Data Set-C<br>Y
25561	27:**10 X** vs. 41:**40 X**	0.6153	0.4544 to 0.7763	Yes
25562	27:**10 X** vs. 41:**50 X**	0.7027	0.5417 to 0.8636	Yes
25563	27:**10 X** vs. 42:**0 X**	-2.419	-2.580 to -2.258	Yes
25564	27:**10 X** vs. 42:**1 X**	-2.438	-2.599 to -2.277	Yes
25565	27:**10 X** vs. 42:**10 X**	-1.421	-1.582 to -1.260	Yes
25566	27:**10 X** vs. 42:**40 X**	0.6027	0.4417 to 0.7636	Yes
25567	27:**10 X** vs. 42:**50 X**	0.6993	0.5384 to 0.8603	Yes
25568	27:**10 X** vs. 43:**0 X**	-2.509	-2.670 to -2.348	Yes
25569	27:**10 X** vs. 43:**1 X**	-2.423	-2.584 to -2.262	Yes
25570	27:**10 X** vs. 43:**10 X**	-2.139	-2.300 to -1.978	Yes
25571	27:**10 X** vs. 43:**40 X**	0.6043	0.4434 to 0.7653	Yes
25572	27:**10 X** vs. 43:**50 X**	0.6990	0.5380 to 0.8600	Yes
25573	27:**10 X** vs. 44:**0 X**	-2.533	-2.694 to -2.372	Yes
25574	27:**10 X** vs. 44:**1 X**	-2.422	-2.583 to -2.261	Yes
25575	27:**10 X** vs. 44:**10 X**	-2.202	-2.363 to -2.041	Yes
25576	27:**10 X** vs. 44:**40 X**	0.6023	0.4414 to 0.7633	Yes
25577	27:**10 X** vs. 44:**50 X**	0.7667	0.6057 to 0.9276	Yes
25578	27:**10 X** vs. 45:**0 X**	-2.397	-2.558 to -2.236	Yes
25579	27:**10 X** vs. 45:**1 X**	-2.199	-2.360 to -2.038	Yes
25580	27:**10 X** vs. 45:**10 X**	-2.082	-2.243 to -1.921	Yes
25581	27:**10 X** vs. 45:**40 X**	0.6010	0.4400 to 0.7620	Yes
25582	27:**10 X** vs. 45:**50 X**	0.7697	0.6087 to 0.9306	Yes
25583	27:**10 X** vs. 46:**0 X**	-2.202	-2.363 to -2.041	Yes
25584	27:**10 X** vs. 46:**1 X**	-2.169	-2.330 to -2.008	Yes
25585	27:**10 X** vs. 46:**10 X**	-2.077	-2.238 to -1.916	Yes
25586	27:**10 X** vs. 46:**40 X**	0.5873	0.4264 to 0.7483	Yes
25587	27:**10 X** vs. 46:**50 X**	0.7767	0.6157 to 0.9376	Yes
25588	27:**10 X** vs. 47:**0 X**	-2.177	-2.338 to -2.016	Yes
25589	27:**10 X** vs. 47:**1 X**	-2.081	-2.242 to -1.920	Yes
25590	27:**10 X** vs. 47:**10 X**	-2.063	-2.224 to -1.902	Yes
25591	27:**10 X** vs. 47:**40 X**	0.5773	0.4164 to 0.7383	Yes
25592	27:**10 X** vs. 47:**50 X**	0.8187	0.6577 to 0.9796	Yes
25593	27:**10 X** vs. 48:**0 X**	-2.165	-2.341 to -1.988	Yes
25594	27:**10 X** vs. 48:**1 X**	-2.068	-2.229 to -1.907	Yes
25595	27:**10 X** vs. 48:**10 X**	-2.063	-2.224 to -1.902	Yes
25596	27:**10 X** vs. 48:**40 X**	0.5710	0.4100 to 0.7320	Yes
25597	27:**10 X** vs. 48:**50 X**	0.8113	0.6504 to 0.9723	Yes
25598	27:**10 X** vs. 49:**0 X**	-2.165	-2.326 to -2.004	Yes
25599	27:**10 X** vs. 49:**1 X**	-2.068	-2.229 to -1.907	Yes
25600	27:**10 X** vs. 49:**10 X**	-2.063	-2.224 to -1.902	Yes
25601	27:**10 X** vs. 49:**40 X**	0.5667	0.4057 to 0.7276	Yes
25602	27:**10 X** vs. 49:**50 X**	0.8120	0.6510 to 0.9730	Yes
25603	27:**10 X** vs. 50:**0 X**	-2.165	-2.326 to -2.004	Yes
25604	27:**10 X** vs. 50:**1 X**	-2.068	-2.229 to -1.907	Yes
25605	27:**10 X** vs. 50:**10 X**	-2.063	-2.224 to -1.902	Yes

	2way ANOVA<br>Multiple comparisons	A<br>Data Set-A<br>Y	B<br>Data Set-B<br>Y	C<br>Data Set-C<br>Y
25606	27:10 X vs. 50:40 X	0.5590	0.3980 to 0.7200	Yes
25607	27:10 X vs. 50:50 X	0.8123	0.6514 to 0.9733	Yes
25608	27:40 X vs. 27:50 X	0.1403	-0.003626 to 0.2843	No
25609	27:40 X vs. 28:0 X	-0.8918	-1.053 to -0.7309	Yes
25610	27:40 X vs. 28:1 X	-0.9327	-1.077 to -0.7887	Yes
25611	27:40 X vs. 28:10 X	-0.7343	-0.8953 to -0.5734	Yes
25612	27:40 X vs. 28:40 X	-0.01233	-0.1563 to 0.1316	No
25613	27:40 X vs. 28:50 X	0.09233	-0.05163 to 0.2363	No
25614	27:40 X vs. 29:0 X	-1.373	-1.534 to -1.212	Yes
25615	27:40 X vs. 29:1 X	-1.458	-1.619 to -1.297	Yes
25616	27:40 X vs. 29:10 X	-0.6728	-0.8338 to -0.5119	Yes
25617	27:40 X vs. 29:40 X	-0.1203	-0.2643 to 0.02363	No
25618	27:40 X vs. 29:50 X	0.0910	-0.05296 to 0.2350	No
25619	27:40 X vs. 30:0 X	-1.088	-1.249 to -0.9269	Yes
25620	27:40 X vs. 30:1 X	-1.409	-1.570 to -1.248	Yes
25621	27:40 X vs. 30:10 X	-0.7473	-0.9083 to -0.5864	Yes
25622	27:40 X vs. 30:40 X	-0.04733	-0.1913 to 0.09663	No
25623	27:40 X vs. 30:50 X	0.03867	-0.1053 to 0.1826	No
25624	27:40 X vs. 31:0 X	-1.406	-1.567 to -1.245	Yes
25625	27:40 X vs. 31:1 X	-1.396	-1.557 to -1.235	Yes
25626	27:40 X vs. 31:10 X	-0.8788	-1.040 to -0.7179	Yes
25627	27:40 X vs. 31:40 X	-0.1390	-0.2830 to 0.004960	No
25628	27:40 X vs. 31:50 X	0.05967	-0.08429 to 0.2036	No
25629	27:40 X vs. 32:0 X	-1.997	-2.141 to -1.853	Yes
25630	27:40 X vs. 32:1 X	-1.453	-1.614 to -1.292	Yes
25631	27:40 X vs. 32:10 X	-1.309	-1.453 to -1.165	Yes
25632	27:40 X vs. 32:40 X	-0.1353	-0.2793 to 0.008626	No
25633	27:40 X vs. 32:50 X	0.06000	-0.08396 to 0.2040	No
25634	27:40 X vs. 33:0 X	-2.001	-2.145 to -1.857	Yes
25635	27:40 X vs. 33:1 X	-1.816	-1.960 to -1.672	Yes
25636	27:40 X vs. 33:10 X	-1.476	-1.620 to -1.332	Yes
25637	27:40 X vs. 33:40 X	-0.1540	-0.2980 to -0.01004	Yes
25638	27:40 X vs. 33:50 X	0.04500	-0.09896 to 0.1890	No
25639	27:40 X vs. 34:0 X	-1.926	-2.070 to -1.782	Yes
25640	27:40 X vs. 34:1 X	-1.933	-2.077 to -1.789	Yes
25641	27:40 X vs. 34:10 X	-1.566	-1.710 to -1.422	Yes
25642	27:40 X vs. 34:40 X	-0.1660	-0.3100 to -0.02204	Yes
25643	27:40 X vs. 34:50 X	-0.1040	-0.2480 to 0.03996	No
25644	27:40 X vs. 35:0 X	-2.274	-2.418 to -2.130	Yes
25645	27:40 X vs. 35:1 X	-2.040	-2.184 to -1.896	Yes
25646	27:40 X vs. 35:10 X	-1.528	-1.672 to -1.384	Yes
25647	27:40 X vs. 35:40 X	-0.1750	-0.3190 to -0.03104	Yes
25648	27:40 X vs. 35:50 X	-0.1167	-0.2606 to 0.02729	No
25649	27:40 X vs. 36:0 X	-2.481	-2.642 to -2.320	Yes
25650	27:40 X vs. 36:1 X	-2.177	-2.321 to -2.033	Yes

	2way ANOVA<br>Multiple comparisons	A<br>Data Set-A<br>Y	B<br>Data Set-B<br>Y	C<br>Data Set-C<br>Y
25651	27:40 X vs. 36:10 X	-1.534	-1.678 to -1.390	Yes
25652	27:40 X vs. 36:40 X	-0.2303	-0.3743 to -0.08637	Yes
25653	27:40 X vs. 36:50 X	-0.08000	-0.2240 to 0.06396	No
25654	27:40 X vs. 37:0 X	-2.865	-3.009 to -2.721	Yes
25655	27:40 X vs. 37:1 X	-2.247	-2.391 to -2.103	Yes
25656	27:40 X vs. 37:10 X	-1.535	-1.679 to -1.391	Yes
25657	27:40 X vs. 37:40 X	-0.2677	-0.4116 to -0.1237	Yes
25658	27:40 X vs. 37:50 X	-0.08867	-0.2326 to 0.05529	No
25659	27:40 X vs. 38:0 X	-2.810	-2.954 to -2.666	Yes
25660	27:40 X vs. 38:1 X	-2.365	-2.509 to -2.221	Yes
25661	27:40 X vs. 38:10 X	-1.811	-1.955 to -1.667	Yes
25662	27:40 X vs. 38:40 X	-0.2720	-0.4160 to -0.1280	Yes
25663	27:40 X vs. 38:50 X	-0.09267	-0.2366 to 0.05129	No
25664	27:40 X vs. 39:0 X	-3.040	-3.184 to -2.896	Yes
25665	27:40 X vs. 39:1 X	-2.932	-3.076 to -2.788	Yes
25666	27:40 X vs. 39:10 X	-1.956	-2.100 to -1.812	Yes
25667	27:40 X vs. 39:40 X	-0.2780	-0.4220 to -0.1340	Yes
25668	27:40 X vs. 39:50 X	-0.1237	-0.2676 to 0.02029	No
25669	27:40 X vs. 40:0 X	-3.081	-3.225 to -2.937	Yes
25670	27:40 X vs. 40:1 X	-3.007	-3.151 to -2.863	Yes
25671	27:40 X vs. 40:10 X	-2.212	-2.356 to -2.068	Yes
25672	27:40 X vs. 40:40 X	-0.2827	-0.4266 to -0.1387	Yes
25673	27:40 X vs. 40:50 X	-0.1300	-0.2740 to 0.01396	No
25674	27:40 X vs. 41:0 X	-3.122	-3.266 to -2.978	Yes
25675	27:40 X vs. 41:1 X	-3.121	-3.265 to -2.977	Yes
25676	27:40 X vs. 41:10 X	-2.266	-2.410 to -2.122	Yes
25677	27:40 X vs. 41:40 X	-0.2300	-0.3740 to -0.08604	Yes
25678	27:40 X vs. 41:50 X	-0.1427	-0.2866 to 0.001293	No
25679	27:40 X vs. 42:0 X	-3.264	-3.408 to -3.120	Yes
25680	27:40 X vs. 42:1 X	-3.284	-3.428 to -3.140	Yes
25681	27:40 X vs. 42:10 X	-2.266	-2.410 to -2.122	Yes
25682	27:40 X vs. 42:40 X	-0.2427	-0.3866 to -0.09871	Yes
25683	27:40 X vs. 42:50 X	-0.1460	-0.2900 to -0.002040	Yes
25684	27:40 X vs. 43:0 X	-3.354	-3.498 to -3.210	Yes
25685	27:40 X vs. 43:1 X	-3.268	-3.412 to -3.124	Yes
25686	27:40 X vs. 43:10 X	-2.984	-3.128 to -2.840	Yes
25687	27:40 X vs. 43:40 X	-0.2410	-0.3850 to -0.09704	Yes
25688	27:40 X vs. 43:50 X	-0.1463	-0.2903 to -0.002374	Yes
25689	27:40 X vs. 44:0 X	-3.378	-3.522 to -3.234	Yes
25690	27:40 X vs. 44:1 X	-3.268	-3.412 to -3.124	Yes
25691	27:40 X vs. 44:10 X	-3.047	-3.191 to -2.903	Yes
25692	27:40 X vs. 44:40 X	-0.2430	-0.3870 to -0.09904	Yes
25693	27:40 X vs. 44:50 X	-0.07867	-0.2226 to 0.06529	No
25694	27:40 X vs. 45:0 X	-3.243	-3.387 to -3.099	Yes
25695	27:40 X vs. 45:1 X	-3.045	-3.189 to -2.901	Yes

	2way ANOVA<br>Multiple comparisons	A<br>Data Set-A<br>Y	B<br>Data Set-B<br>Y	C<br>Data Set-C<br>Y
25696	27:**40 X** vs. 45:**10 X**	-2.927	-3.071 to -2.783	Yes
25697	27:**40 X** vs. 45:**40 X**	-0.2443	-0.3883 to -0.1004	Yes
25698	27:**40 X** vs. 45:**50 X**	-0.07567	-0.2196 to 0.06829	No
25699	27:**40 X** vs. 46:**0 X**	-3.047	-3.191 to -2.903	Yes
25700	27:**40 X** vs. 46:**1 X**	-3.014	-3.158 to -2.870	Yes
25701	27:**40 X** vs. 46:**10 X**	-2.922	-3.066 to -2.778	Yes
25702	27:**40 X** vs. 46:**40 X**	-0.2580	-0.4020 to -0.1140	Yes
25703	27:**40 X** vs. 46:**50 X**	-0.06867	-0.2126 to 0.07529	No
25704	27:**40 X** vs. 47:**0 X**	-3.023	-3.167 to -2.879	Yes
25705	27:**40 X** vs. 47:**1 X**	-2.927	-3.071 to -2.783	Yes
25706	27:**40 X** vs. 47:**10 X**	-2.908	-3.052 to -2.764	Yes
25707	27:**40 X** vs. 47:**40 X**	-0.2680	-0.4120 to -0.1240	Yes
25708	27:**40 X** vs. 47:**50 X**	-0.02667	-0.1706 to 0.1173	No
25709	27:**40 X** vs. 48:**0 X**	-3.010	-3.171 to -2.849	Yes
25710	27:**40 X** vs. 48:**1 X**	-2.914	-3.058 to -2.770	Yes
25711	27:**40 X** vs. 48:**10 X**	-2.908	-3.052 to -2.764	Yes
25712	27:**40 X** vs. 48:**40 X**	-0.2743	-0.4183 to -0.1304	Yes
25713	27:**40 X** vs. 48:**50 X**	-0.0340	-0.1780 to 0.1100	No
25714	27:**40 X** vs. 49:**0 X**	-3.010	-3.154 to -2.866	Yes
25715	27:**40 X** vs. 49:**1 X**	-2.914	-3.058 to -2.770	Yes
25716	27:**40 X** vs. 49:**10 X**	-2.908	-3.052 to -2.764	Yes
25717	27:**40 X** vs. 49:**40 X**	-0.2787	-0.4226 to -0.1347	Yes
25718	27:**40 X** vs. 49:**50 X**	-0.03333	-0.1773 to 0.1106	No
25719	27:**40 X** vs. 50:**0 X**	-3.010	-3.154 to -2.866	Yes
25720	27:**40 X** vs. 50:**1 X**	-2.914	-3.058 to -2.770	Yes
25721	27:**40 X** vs. 50:**10 X**	-2.908	-3.052 to -2.764	Yes
25722	27:**40 X** vs. 50:**40 X**	-0.2863	-0.4303 to -0.1424	Yes
25723	27:**40 X** vs. 50:**50 X**	-0.03300	-0.1770 to 0.1110	No
25724	27:**50 X** vs. 28:**0 X**	-1.032	-1.193 to -0.8712	Yes
25725	27:**50 X** vs. 28:**1 X**	-1.073	-1.217 to -0.9290	Yes
25726	27:**50 X** vs. 28:**10 X**	-0.8747	-1.036 to -0.7137	Yes
25727	27:**50 X** vs. 28:**40 X**	-0.1527	-0.2966 to -0.008707	Yes
25728	27:**50 X** vs. 28:**50 X**	-0.04800	-0.1920 to 0.09596	No
25729	27:**50 X** vs. 29:**0 X**	-1.514	-1.675 to -1.353	Yes
25730	27:**50 X** vs. 29:**1 X**	-1.599	-1.760 to -1.438	Yes
25731	27:**50 X** vs. 29:**10 X**	-0.8132	-0.9741 to -0.6522	Yes
25732	27:**50 X** vs. 29:**40 X**	-0.2607	-0.4046 to -0.1167	Yes
25733	27:**50 X** vs. 29:**50 X**	-0.04933	-0.1933 to 0.09463	No
25734	27:**50 X** vs. 30:**0 X**	-1.228	-1.389 to -1.067	Yes
25735	27:**50 X** vs. 30:**1 X**	-1.550	-1.711 to -1.389	Yes
25736	27:**50 X** vs. 30:**10 X**	-0.8877	-1.049 to -0.7267	Yes
25737	27:**50 X** vs. 30:**40 X**	-0.1877	-0.3316 to -0.04371	Yes
25738	27:**50 X** vs. 30:**50 X**	-0.1017	-0.2456 to 0.04229	No
25739	27:**50 X** vs. 31:**0 X**	-1.546	-1.707 to -1.385	Yes
25740	27:**50 X** vs. 31:**1 X**	-1.537	-1.698 to -1.376	Yes

2way ANOVA Multiple comparisons	A Data Set-A Y	B Data Set-B Y	C Data Set-C Y
25741 27:50 X vs. 31:10 X	-1.019	-1.180 to -0.8582	Yes
25742 27:50 X vs. 31:40 X	-0.2793	-0.4233 to -0.1354	Yes
25743 27:50 X vs. 31:50 X	-0.08067	-0.2246 to 0.06329	No
25744 27:50 X vs. 32:0 X	-2.138	-2.282 to -1.994	Yes
25745 27:50 X vs. 32:1 X	-1.594	-1.755 to -1.433	Yes
25746 27:50 X vs. 32:10 X	-1.450	-1.594 to -1.306	Yes
25747 27:50 X vs. 32:40 X	-0.2757	-0.4196 to -0.1317	Yes
25748 27:50 X vs. 32:50 X	-0.08033	-0.2243 to 0.06363	No
25749 27:50 X vs. 33:0 X	-2.141	-2.285 to -1.997	Yes
25750 27:50 X vs. 33:1 X	-1.956	-2.100 to -1.812	Yes
25751 27:50 X vs. 33:10 X	-1.616	-1.760 to -1.472	Yes
25752 27:50 X vs. 33:40 X	-0.2943	-0.4383 to -0.1504	Yes
25753 27:50 X vs. 33:50 X	-0.09533	-0.2393 to 0.04863	No
25754 27:50 X vs. 34:0 X	-2.066	-2.210 to -1.922	Yes
25755 27:50 X vs. 34:1 X	-2.073	-2.217 to -1.929	Yes
25756 27:50 X vs. 34:10 X	-1.707	-1.851 to -1.563	Yes
25757 27:50 X vs. 34:40 X	-0.3063	-0.4503 to -0.1624	Yes
25758 27:50 X vs. 34:50 X	-0.2443	-0.3883 to -0.1004	Yes
25759 27:50 X vs. 35:0 X	-2.415	-2.559 to -2.271	Yes
25760 27:50 X vs. 35:1 X	-2.180	-2.324 to -2.036	Yes
25761 27:50 X vs. 35:10 X	-1.668	-1.812 to -1.524	Yes
25762 27:50 X vs. 35:40 X	-0.3153	-0.4593 to -0.1714	Yes
25763 27:50 X vs. 35:50 X	-0.2570	-0.4010 to -0.1130	Yes
25764 27:50 X vs. 36:0 X	-2.621	-2.782 to -2.460	Yes
25765 27:50 X vs. 36:1 X	-2.317	-2.461 to -2.173	Yes
25766 27:50 X vs. 36:10 X	-1.674	-1.818 to -1.530	Yes
25767 27:50 X vs. 36:40 X	-0.3707	-0.5146 to -0.2267	Yes
25768 27:50 X vs. 36:50 X	-0.2203	-0.3643 to -0.07637	Yes
25769 27:50 X vs. 37:0 X	-3.005	-3.149 to -2.861	Yes
25770 27:50 X vs. 37:1 X	-2.387	-2.531 to -2.243	Yes
25771 27:50 X vs. 37:10 X	-1.676	-1.820 to -1.532	Yes
25772 27:50 X vs. 37:40 X	-0.4080	-0.5520 to -0.2640	Yes
25773 27:50 X vs. 37:50 X	-0.2290	-0.3730 to -0.08504	Yes
25774 27:50 X vs. 38:0 X	-2.950	-3.094 to -2.806	Yes
25775 27:50 X vs. 38:1 X	-2.506	-2.650 to -2.362	Yes
25776 27:50 X vs. 38:10 X	-1.951	-2.095 to -1.807	Yes
25777 27:50 X vs. 38:40 X	-0.4123	-0.5563 to -0.2684	Yes
25778 27:50 X vs. 38:50 X	-0.2330	-0.3770 to -0.08904	Yes
25779 27:50 X vs. 39:0 X	-3.181	-3.325 to -3.037	Yes
25780 27:50 X vs. 39:1 X	-3.072	-3.216 to -2.928	Yes
25781 27:50 X vs. 39:10 X	-2.096	-2.240 to -1.952	Yes
25782 27:50 X vs. 39:40 X	-0.4183	-0.5623 to -0.2744	Yes
25783 27:50 X vs. 39:50 X	-0.2640	-0.4080 to -0.1200	Yes
25784 27:50 X vs. 40:0 X	-3.222	-3.366 to -3.078	Yes
25785 27:50 X vs. 40:1 X	-3.148	-3.292 to -3.004	Yes

	2way ANOVA<br>Multiple comparisons	A<br>Data Set-A<br>Y	B<br>Data Set-B<br>Y	C<br>Data Set-C<br>Y
25786	27:**50 X** vs. 40:**10 X**	-2.352	-2.496 to -2.208	Yes
25787	27:**50 X** vs. 40:**40 X**	-0.4230	-0.5670 to -0.2790	Yes
25788	27:**50 X** vs. 40:**50 X**	-0.2703	-0.4143 to -0.1264	Yes
25789	27:**50 X** vs. 41:**0 X**	-3.263	-3.407 to -3.119	Yes
25790	27:**50 X** vs. 41:**1 X**	-3.262	-3.406 to -3.118	Yes
25791	27:**50 X** vs. 41:**10 X**	-2.406	-2.550 to -2.262	Yes
25792	27:**50 X** vs. 41:**40 X**	-0.3703	-0.5143 to -0.2264	Yes
25793	27:**50 X** vs. 41:**50 X**	-0.2830	-0.4270 to -0.1390	Yes
25794	27:**50 X** vs. 42:**0 X**	-3.404	-3.548 to -3.260	Yes
25795	27:**50 X** vs. 42:**1 X**	-3.424	-3.568 to -3.280	Yes
25796	27:**50 X** vs. 42:**10 X**	-2.406	-2.550 to -2.262	Yes
25797	27:**50 X** vs. 42:**40 X**	-0.3830	-0.5270 to -0.2390	Yes
25798	27:**50 X** vs. 42:**50 X**	-0.2863	-0.4303 to -0.1424	Yes
25799	27:**50 X** vs. 43:**0 X**	-3.494	-3.638 to -3.350	Yes
25800	27:**50 X** vs. 43:**1 X**	-3.409	-3.553 to -3.265	Yes
25801	27:**50 X** vs. 43:**10 X**	-3.124	-3.268 to -2.980	Yes
25802	27:**50 X** vs. 43:**40 X**	-0.3813	-0.5253 to -0.2374	Yes
25803	27:**50 X** vs. 43:**50 X**	-0.2867	-0.4306 to -0.1427	Yes
25804	27:**50 X** vs. 44:**0 X**	-3.518	-3.662 to -3.374	Yes
25805	27:**50 X** vs. 44:**1 X**	-3.408	-3.552 to -3.264	Yes
25806	27:**50 X** vs. 44:**10 X**	-3.188	-3.332 to -3.044	Yes
25807	27:**50 X** vs. 44:**40 X**	-0.3833	-0.5273 to -0.2394	Yes
25808	27:**50 X** vs. 44:**50 X**	-0.2190	-0.3630 to -0.07504	Yes
25809	27:**50 X** vs. 45:**0 X**	-3.383	-3.527 to -3.239	Yes
25810	27:**50 X** vs. 45:**1 X**	-3.185	-3.329 to -3.041	Yes
25811	27:**50 X** vs. 45:**10 X**	-3.067	-3.211 to -2.923	Yes
25812	27:**50 X** vs. 45:**40 X**	-0.3847	-0.5286 to -0.2407	Yes
25813	27:**50 X** vs. 45:**50 X**	-0.2160	-0.3600 to -0.07204	Yes
25814	27:**50 X** vs. 46:**0 X**	-3.187	-3.331 to -3.043	Yes
25815	27:**50 X** vs. 46:**1 X**	-3.154	-3.298 to -3.010	Yes
25816	27:**50 X** vs. 46:**10 X**	-3.062	-3.206 to -2.918	Yes
25817	27:**50 X** vs. 46:**40 X**	-0.3983	-0.5423 to -0.2544	Yes
25818	27:**50 X** vs. 46:**50 X**	-0.2090	-0.3530 to -0.06504	Yes
25819	27:**50 X** vs. 47:**0 X**	-3.163	-3.307 to -3.019	Yes
25820	27:**50 X** vs. 47:**1 X**	-3.067	-3.211 to -2.923	Yes
25821	27:**50 X** vs. 47:**10 X**	-3.048	-3.192 to -2.904	Yes
25822	27:**50 X** vs. 47:**40 X**	-0.4083	-0.5523 to -0.2644	Yes
25823	27:**50 X** vs. 47:**50 X**	-0.1670	-0.3110 to -0.02304	Yes
25824	27:**50 X** vs. 48:**0 X**	-3.150	-3.311 to -2.989	Yes
25825	27:**50 X** vs. 48:**1 X**	-3.054	-3.198 to -2.910	Yes
25826	27:**50 X** vs. 48:**10 X**	-3.048	-3.192 to -2.904	Yes
25827	27:**50 X** vs. 48:**40 X**	-0.4147	-0.5586 to -0.2707	Yes
25828	27:**50 X** vs. 48:**50 X**	-0.1743	-0.3183 to -0.03037	Yes
25829	27:**50 X** vs. 49:**0 X**	-3.150	-3.294 to -3.006	Yes
25830	27:**50 X** vs. 49:**1 X**	-3.054	-3.198 to -2.910	Yes

	2way ANOVA<br>Multiple comparisons	A<br>Data Set-A<br>Y	B<br>Data Set-B<br>Y	C<br>Data Set-C<br>Y
25831	27:**50 X** vs. 49:**10 X**	-3.048	-3.192 to -2.904	Yes
25832	27:**50 X** vs. 49:**40 X**	-0.4190	-0.5630 to -0.2750	Yes
25833	27:**50 X** vs. 49:**50 X**	-0.1737	-0.3176 to -0.02971	Yes
25834	27:**50 X** vs. 50:**0 X**	-3.150	-3.294 to -3.006	Yes
25835	27:**50 X** vs. 50:**1 X**	-3.054	-3.198 to -2.910	Yes
25836	27:**50 X** vs. 50:**10 X**	-3.048	-3.192 to -2.904	Yes
25837	27:**50 X** vs. 50:**40 X**	-0.4267	-0.5706 to -0.2827	Yes
25838	27:**50 X** vs. 50:**50 X**	-0.1733	-0.3173 to -0.02937	Yes
25839	28:**0 X** vs. 28:**1 X**	-0.04083	-0.2018 to 0.1201	No
25840	28:**0 X** vs. 28:**10 X**	0.1575	-0.01881 to 0.3338	No
25841	28:**0 X** vs. 28:**40 X**	0.8795	0.7185 to 1.040	Yes
25842	28:**0 X** vs. 28:**50 X**	0.9842	0.8232 to 1.145	Yes
25843	28:**0 X** vs. 29:**0 X**	-0.4815	-0.6578 to -0.3052	Yes
25844	28:**0 X** vs. 29:**1 X**	-0.5665	-0.7428 to -0.3902	Yes
25845	28:**0 X** vs. 29:**10 X**	0.2190	0.04269 to 0.3953	Yes
25846	28:**0 X** vs. 29:**40 X**	0.7715	0.6105 to 0.9325	Yes
25847	28:**0 X** vs. 29:**50 X**	0.9828	0.8219 to 1.144	Yes
25848	28:**0 X** vs. 30:**0 X**	-0.1960	-0.3723 to -0.01969	Yes
25849	28:**0 X** vs. 30:**1 X**	-0.5175	-0.6938 to -0.3412	Yes
25850	28:**0 X** vs. 30:**10 X**	0.1445	-0.03181 to 0.3208	No
25851	28:**0 X** vs. 30:**40 X**	0.8445	0.6835 to 1.005	Yes
25852	28:**0 X** vs. 30:**50 X**	0.9305	0.7695 to 1.091	Yes
25853	28:**0 X** vs. 31:**0 X**	-0.5140	-0.6903 to -0.3377	Yes
25854	28:**0 X** vs. 31:**1 X**	-0.5045	-0.6808 to -0.3282	Yes
25855	28:**0 X** vs. 31:**10 X**	0.01300	-0.1633 to 0.1893	No
25856	28:**0 X** vs. 31:**40 X**	0.7528	0.5919 to 0.9138	Yes
25857	28:**0 X** vs. 31:**50 X**	0.9515	0.7905 to 1.112	Yes
25858	28:**0 X** vs. 32:**0 X**	-1.106	-1.266 to -0.9445	Yes
25859	28:**0 X** vs. 32:**1 X**	-0.5615	-0.7378 to -0.3852	Yes
25860	28:**0 X** vs. 32:**10 X**	-0.4175	-0.5785 to -0.2565	Yes
25861	28:**0 X** vs. 32:**40 X**	0.7565	0.5955 to 0.9175	Yes
25862	28:**0 X** vs. 32:**50 X**	0.9518	0.7909 to 1.113	Yes
25863	28:**0 X** vs. 33:**0 X**	-1.109	-1.270 to -0.9482	Yes
25864	28:**0 X** vs. 33:**1 X**	-0.9238	-1.085 to -0.7629	Yes
25865	28:**0 X** vs. 33:**10 X**	-0.5842	-0.7451 to -0.4232	Yes
25866	28:**0 X** vs. 33:**40 X**	0.7378	0.5769 to 0.8988	Yes
25867	28:**0 X** vs. 33:**50 X**	0.9368	0.7759 to 1.098	Yes
25868	28:**0 X** vs. 34:**0 X**	-1.034	-1.195 to -0.8729	Yes
25869	28:**0 X** vs. 34:**1 X**	-1.041	-1.202 to -0.8802	Yes
25870	28:**0 X** vs. 34:**10 X**	-0.6745	-0.8355 to -0.5135	Yes
25871	28:**0 X** vs. 34:**40 X**	0.7258	0.5649 to 0.8868	Yes
25872	28:**0 X** vs. 34:**50 X**	0.7878	0.6269 to 0.9488	Yes
25873	28:**0 X** vs. 35:**0 X**	-1.383	-1.543 to -1.222	Yes
25874	28:**0 X** vs. 35:**1 X**	-1.148	-1.309 to -0.9872	Yes
25875	28:**0 X** vs. 35:**10 X**	-0.6362	-0.7971 to -0.4752	Yes

	2way ANOVA<br>Multiple comparisons	A<br>Data Set-A<br>Y	B<br>Data Set-B<br>Y	C<br>Data Set-C<br>Y
25876	28:**0 X** vs. 35:**40 X**	0.7168	0.5559 to 0.8778	Yes
25877	28:**0 X** vs. 35:**50 X**	0.7752	0.6142 to 0.9361	Yes
25878	28:**0 X** vs. 36:**0 X**	-1.589	-1.765 to -1.413	Yes
25879	28:**0 X** vs. 36:**1 X**	-1.285	-1.446 to -1.124	Yes
25880	28:**0 X** vs. 36:**10 X**	-0.6418	-0.8028 to -0.4809	Yes
25881	28:**0 X** vs. 36:**40 X**	0.6615	0.5005 to 0.8225	Yes
25882	28:**0 X** vs. 36:**50 X**	0.8118	0.6509 to 0.9728	Yes
25883	28:**0 X** vs. 37:**0 X**	-1.973	-2.134 to -1.812	Yes
25884	28:**0 X** vs. 37:**1 X**	-1.355	-1.516 to -1.194	Yes
25885	28:**0 X** vs. 37:**10 X**	-0.6435	-0.8045 to -0.4825	Yes
25886	28:**0 X** vs. 37:**40 X**	0.6242	0.4632 to 0.7851	Yes
25887	28:**0 X** vs. 37:**50 X**	0.8032	0.6422 to 0.9641	Yes
25888	28:**0 X** vs. 38:**0 X**	-1.918	-2.079 to -1.757	Yes
25889	28:**0 X** vs. 38:**1 X**	-1.474	-1.634 to -1.313	Yes
25890	28:**0 X** vs. 38:**10 X**	-0.9188	-1.080 to -0.7579	Yes
25891	28:**0 X** vs. 38:**40 X**	0.6198	0.4589 to 0.7808	Yes
25892	28:**0 X** vs. 38:**50 X**	0.7992	0.6382 to 0.9601	Yes
25893	28:**0 X** vs. 39:**0 X**	-2.149	-2.309 to -1.988	Yes
25894	28:**0 X** vs. 39:**1 X**	-2.040	-2.201 to -1.879	Yes
25895	28:**0 X** vs. 39:**10 X**	-1.064	-1.225 to -0.9029	Yes
25896	28:**0 X** vs. 39:**40 X**	0.6138	0.4529 to 0.7748	Yes
25897	28:**0 X** vs. 39:**50 X**	0.7682	0.6072 to 0.9291	Yes
25898	28:**0 X** vs. 40:**0 X**	-2.190	-2.350 to -2.029	Yes
25899	28:**0 X** vs. 40:**1 X**	-2.116	-2.276 to -1.955	Yes
25900	28:**0 X** vs. 40:**10 X**	-1.320	-1.481 to -1.159	Yes
25901	28:**0 X** vs. 40:**40 X**	0.6092	0.4482 to 0.7701	Yes
25902	28:**0 X** vs. 40:**50 X**	0.7618	0.6009 to 0.9228	Yes
25903	28:**0 X** vs. 41:**0 X**	-2.231	-2.391 to -2.070	Yes
25904	28:**0 X** vs. 41:**1 X**	-2.230	-2.390 to -2.069	Yes
25905	28:**0 X** vs. 41:**10 X**	-1.374	-1.535 to -1.213	Yes
25906	28:**0 X** vs. 41:**40 X**	0.6618	0.5009 to 0.8228	Yes
25907	28:**0 X** vs. 41:**50 X**	0.7492	0.5882 to 0.9101	Yes
25908	28:**0 X** vs. 42:**0 X**	-2.372	-2.533 to -2.211	Yes
25909	28:**0 X** vs. 42:**1 X**	-2.392	-2.553 to -2.231	Yes
25910	28:**0 X** vs. 42:**10 X**	-1.374	-1.535 to -1.213	Yes
25911	28:**0 X** vs. 42:**40 X**	0.6492	0.4882 to 0.8101	Yes
25912	28:**0 X** vs. 42:**50 X**	0.7458	0.5849 to 0.9068	Yes
25913	28:**0 X** vs. 43:**0 X**	-2.462	-2.623 to -2.301	Yes
25914	28:**0 X** vs. 43:**1 X**	-2.377	-2.537 to -2.216	Yes
25915	28:**0 X** vs. 43:**10 X**	-2.092	-2.253 to -1.931	Yes
25916	28:**0 X** vs. 43:**40 X**	0.6508	0.4899 to 0.8118	Yes
25917	28:**0 X** vs. 43:**50 X**	0.7455	0.5845 to 0.9065	Yes
25918	28:**0 X** vs. 44:**0 X**	-2.486	-2.647 to -2.325	Yes
25919	28:**0 X** vs. 44:**1 X**	-2.376	-2.537 to -2.215	Yes
25920	28:**0 X** vs. 44:**10 X**	-2.156	-2.316 to -1.995	Yes

2way ANOVA<br>Multiple comparisons		A<br>Data Set-A<br>Y	B<br>Data Set-B<br>Y	C<br>Data Set-C<br>Y
25921	28:0 X vs. 44:40 X	0.6488	0.4879 to 0.8098	Yes
25922	28:0 X vs. 44:50 X	0.8132	0.6522 to 0.9741	Yes
25923	28:0 X vs. 45:0 X	-2.351	-2.512 to -2.190	Yes
25924	28:0 X vs. 45:1 X	-2.153	-2.314 to -1.992	Yes
25925	28:0 X vs. 45:10 X	-2.035	-2.196 to -1.874	Yes
25926	28:0 X vs. 45:40 X	0.6475	0.4865 to 0.8085	Yes
25927	28:0 X vs. 45:50 X	0.8162	0.6552 to 0.9771	Yes
25928	28:0 X vs. 46:0 X	-2.155	-2.316 to -1.994	Yes
25929	28:0 X vs. 46:1 X	-2.122	-2.283 to -1.961	Yes
25930	28:0 X vs. 46:10 X	-2.030	-2.191 to -1.869	Yes
25931	28:0 X vs. 46:40 X	0.6338	0.4729 to 0.7948	Yes
25932	28:0 X vs. 46:50 X	0.8232	0.6622 to 0.9841	Yes
25933	28:0 X vs. 47:0 X	-2.131	-2.292 to -1.970	Yes
25934	28:0 X vs. 47:1 X	-2.035	-2.196 to -1.874	Yes
25935	28:0 X vs. 47:10 X	-2.016	-2.177 to -1.855	Yes
25936	28:0 X vs. 47:40 X	0.6238	0.4629 to 0.7848	Yes
25937	28:0 X vs. 47:50 X	0.8652	0.7042 to 1.026	Yes
25938	28:0 X vs. 48:0 X	-2.118	-2.294 to -1.942	Yes
25939	28:0 X vs. 48:1 X	-2.022	-2.183 to -1.861	Yes
25940	28:0 X vs. 48:10 X	-2.016	-2.177 to -1.855	Yes
25941	28:0 X vs. 48:40 X	0.6175	0.4565 to 0.7785	Yes
25942	28:0 X vs. 48:50 X	0.8578	0.6969 to 1.019	Yes
25943	28:0 X vs. 49:0 X	-2.118	-2.279 to -1.957	Yes
25944	28:0 X vs. 49:1 X	-2.022	-2.183 to -1.861	Yes
25945	28:0 X vs. 49:10 X	-2.016	-2.177 to -1.855	Yes
25946	28:0 X vs. 49:40 X	0.6132	0.4522 to 0.7741	Yes
25947	28:0 X vs. 49:50 X	0.8585	0.6975 to 1.019	Yes
25948	28:0 X vs. 50:0 X	-2.118	-2.279 to -1.957	Yes
25949	28:0 X vs. 50:1 X	-2.022	-2.183 to -1.861	Yes
25950	28:0 X vs. 50:10 X	-2.016	-2.177 to -1.855	Yes
25951	28:0 X vs. 50:40 X	0.6055	0.4445 to 0.7665	Yes
25952	28:0 X vs. 50:50 X	0.8588	0.6979 to 1.020	Yes
25953	28:1 X vs. 28:10 X	0.1983	0.03738 to 0.3593	Yes
25954	28:1 X vs. 28:40 X	0.9203	0.7764 to 1.064	Yes
25955	28:1 X vs. 28:50 X	1.025	0.8810 to 1.169	Yes
25956	28:1 X vs. 29:0 X	-0.4407	-0.6016 to -0.2797	Yes
25957	28:1 X vs. 29:1 X	-0.5257	-0.6866 to -0.3647	Yes
25958	28:1 X vs. 29:10 X	0.2598	0.09888 to 0.4208	Yes
25959	28:1 X vs. 29:40 X	0.8123	0.6684 to 0.9563	Yes
25960	28:1 X vs. 29:50 X	1.024	0.8797 to 1.168	Yes
25961	28:1 X vs. 30:0 X	-0.1552	-0.3161 to 0.005785	No
25962	28:1 X vs. 30:1 X	-0.4767	-0.6376 to -0.3157	Yes
25963	28:1 X vs. 30:10 X	0.1853	0.02438 to 0.3463	Yes
25964	28:1 X vs. 30:40 X	0.8853	0.7414 to 1.029	Yes
25965	28:1 X vs. 30:50 X	0.9713	0.8274 to 1.115	Yes

	2way ANOVA<br>Multiple comparisons	A<br>Data Set-A<br>Y	B<br>Data Set-B<br>Y	C<br>Data Set-C<br>Y
25966	28:**1 X** vs. 31:**0 X**	-0.4732	-0.6341 to -0.3122	Yes
25967	28:**1 X** vs. 31:**1 X**	-0.4637	-0.6246 to -0.3027	Yes
25968	28:**1 X** vs. 31:**10 X**	0.05383	-0.1071 to 0.2148	No
25969	28:**1 X** vs. 31:**40 X**	0.7937	0.6497 to 0.9376	Yes
25970	28:**1 X** vs. 31:**50 X**	0.9923	0.8484 to 1.136	Yes
25971	28:**1 X** vs. 32:**0 X**	-1.065	-1.209 to -0.9207	Yes
25972	28:**1 X** vs. 32:**1 X**	-0.5207	-0.6816 to -0.3597	Yes
25973	28:**1 X** vs. 32:**10 X**	-0.3767	-0.5206 to -0.2327	Yes
25974	28:**1 X** vs. 32:**40 X**	0.7973	0.6534 to 0.9413	Yes
25975	28:**1 X** vs. 32:**50 X**	0.9927	0.8487 to 1.137	Yes
25976	28:**1 X** vs. 33:**0 X**	-1.068	-1.212 to -0.9244	Yes
25977	28:**1 X** vs. 33:**1 X**	-0.8830	-1.027 to -0.7390	Yes
25978	28:**1 X** vs. 33:**10 X**	-0.5433	-0.6873 to -0.3994	Yes
25979	28:**1 X** vs. 33:**40 X**	0.7787	0.6347 to 0.9226	Yes
25980	28:**1 X** vs. 33:**50 X**	0.9777	0.8337 to 1.122	Yes
25981	28:**1 X** vs. 34:**0 X**	-0.9930	-1.137 to -0.8490	Yes
25982	28:**1 X** vs. 34:**1 X**	-1.000	-1.144 to -0.8564	Yes
25983	28:**1 X** vs. 34:**10 X**	-0.6337	-0.7776 to -0.4897	Yes
25984	28:**1 X** vs. 34:**40 X**	0.7667	0.6227 to 0.9106	Yes
25985	28:**1 X** vs. 34:**50 X**	0.8287	0.6847 to 0.9726	Yes
25986	28:**1 X** vs. 35:**0 X**	-1.342	-1.486 to -1.198	Yes
25987	28:**1 X** vs. 35:**1 X**	-1.107	-1.251 to -0.9634	Yes
25988	28:**1 X** vs. 35:**10 X**	-0.5953	-0.7393 to -0.4514	Yes
25989	28:**1 X** vs. 35:**40 X**	0.7577	0.6137 to 0.9016	Yes
25990	28:**1 X** vs. 35:**50 X**	0.8160	0.6720 to 0.9600	Yes
25991	28:**1 X** vs. 36:**0 X**	-1.548	-1.709 to -1.387	Yes
25992	28:**1 X** vs. 36:**1 X**	-1.244	-1.388 to -1.100	Yes
25993	28:**1 X** vs. 36:**10 X**	-0.6010	-0.7450 to -0.4570	Yes
25994	28:**1 X** vs. 36:**40 X**	0.7023	0.5584 to 0.8463	Yes
25995	28:**1 X** vs. 36:**50 X**	0.8527	0.7087 to 0.9966	Yes
25996	28:**1 X** vs. 37:**0 X**	-1.932	-2.076 to -1.788	Yes
25997	28:**1 X** vs. 37:**1 X**	-1.314	-1.458 to -1.170	Yes
25998	28:**1 X** vs. 37:**10 X**	-0.6027	-0.7466 to -0.4587	Yes
25999	28:**1 X** vs. 37:**40 X**	0.6650	0.5210 to 0.8090	Yes
26000	28:**1 X** vs. 37:**50 X**	0.8440	0.7000 to 0.9880	Yes
26001	28:**1 X** vs. 38:**0 X**	-1.877	-2.021 to -1.733	Yes
26002	28:**1 X** vs. 38:**1 X**	-1.433	-1.577 to -1.289	Yes
26003	28:**1 X** vs. 38:**10 X**	-0.8780	-1.022 to -0.7340	Yes
26004	28:**1 X** vs. 38:**40 X**	0.6607	0.5167 to 0.8046	Yes
26005	28:**1 X** vs. 38:**50 X**	0.8400	0.6960 to 0.9840	Yes
26006	28:**1 X** vs. 39:**0 X**	-2.108	-2.252 to -1.964	Yes
26007	28:**1 X** vs. 39:**1 X**	-1.999	-2.143 to -1.855	Yes
26008	28:**1 X** vs. 39:**10 X**	-1.023	-1.167 to -0.8790	Yes
26009	28:**1 X** vs. 39:**40 X**	0.6547	0.5107 to 0.7986	Yes
26010	28:**1 X** vs. 39:**50 X**	0.8090	0.6650 to 0.9530	Yes

2way ANOVA<br>Multiple comparisons	A<br>Data Set-A<br>Y	B<br>Data Set-B<br>Y	C<br>Data Set-C<br>Y
26011 28:1 X vs. 40:0 X	-2.149	-2.293 to -2.005	Yes
26012 28:1 X vs. 40:1 X	-2.075	-2.219 to -1.931	Yes
26013 28:1 X vs. 40:10 X	-1.279	-1.423 to -1.135	Yes
26014 28:1 X vs. 40:40 X	0.6500	0.5060 to 0.7940	Yes
26015 28:1 X vs. 40:50 X	0.8027	0.6587 to 0.9466	Yes
26016 28:1 X vs. 41:0 X	-2.190	-2.334 to -2.046	Yes
26017 28:1 X vs. 41:1 X	-2.189	-2.333 to -2.045	Yes
26018 28:1 X vs. 41:10 X	-1.333	-1.477 to -1.189	Yes
26019 28:1 X vs. 41:40 X	0.7027	0.5587 to 0.8466	Yes
26020 28:1 X vs. 41:50 X	0.7900	0.6460 to 0.9340	Yes
26021 28:1 X vs. 42:0 X	-2.331	-2.475 to -2.187	Yes
26022 28:1 X vs. 42:1 X	-2.351	-2.495 to -2.207	Yes
26023 28:1 X vs. 42:10 X	-1.333	-1.477 to -1.189	Yes
26024 28:1 X vs. 42:40 X	0.6900	0.5460 to 0.8340	Yes
26025 28:1 X vs. 42:50 X	0.7867	0.6427 to 0.9306	Yes
26026 28:1 X vs. 43:0 X	-2.421	-2.565 to -2.277	Yes
26027 28:1 X vs. 43:1 X	-2.336	-2.480 to -2.192	Yes
26028 28:1 X vs. 43:10 X	-2.051	-2.195 to -1.907	Yes
26029 28:1 X vs. 43:40 X	0.6917	0.5477 to 0.8356	Yes
26030 28:1 X vs. 43:50 X	0.7863	0.6424 to 0.9303	Yes
26031 28:1 X vs. 44:0 X	-2.445	-2.589 to -2.301	Yes
26032 28:1 X vs. 44:1 X	-2.335	-2.479 to -2.191	Yes
26033 28:1 X vs. 44:10 X	-2.115	-2.259 to -1.971	Yes
26034 28:1 X vs. 44:40 X	0.6897	0.5457 to 0.8336	Yes
26035 28:1 X vs. 44:50 X	0.8540	0.7100 to 0.9980	Yes
26036 28:1 X vs. 45:0 X	-2.310	-2.454 to -2.166	Yes
26037 28:1 X vs. 45:1 X	-2.112	-2.256 to -1.968	Yes
26038 28:1 X vs. 45:10 X	-1.994	-2.138 to -1.850	Yes
26039 28:1 X vs. 45:40 X	0.6883	0.5444 to 0.8323	Yes
26040 28:1 X vs. 45:50 X	0.8570	0.7130 to 1.001	Yes
26041 28:1 X vs. 46:0 X	-2.114	-2.258 to -1.970	Yes
26042 28:1 X vs. 46:1 X	-2.081	-2.225 to -1.937	Yes
26043 28:1 X vs. 46:10 X	-1.989	-2.133 to -1.845	Yes
26044 28:1 X vs. 46:40 X	0.6747	0.5307 to 0.8186	Yes
26045 28:1 X vs. 46:50 X	0.8640	0.7200 to 1.008	Yes
26046 28:1 X vs. 47:0 X	-2.090	-2.234 to -1.946	Yes
26047 28:1 X vs. 47:1 X	-1.994	-2.138 to -1.850	Yes
26048 28:1 X vs. 47:10 X	-1.975	-2.119 to -1.831	Yes
26049 28:1 X vs. 47:40 X	0.6647	0.5207 to 0.8086	Yes
26050 28:1 X vs. 47:50 X	0.9060	0.7620 to 1.050	Yes
26051 28:1 X vs. 48:0 X	-2.077	-2.238 to -1.916	Yes
26052 28:1 X vs. 48:1 X	-1.981	-2.125 to -1.837	Yes
26053 28:1 X vs. 48:10 X	-1.975	-2.119 to -1.831	Yes
26054 28:1 X vs. 48:40 X	0.6583	0.5144 to 0.8023	Yes
26055 28:1 X vs. 48:50 X	0.8987	0.7547 to 1.043	Yes

	2way ANOVA Multiple comparisons	A Data Set-A Y	B Data Set-B Y	C Data Set-C Y
26056	28:**1 X** vs. 49:**0 X**	-2.077	-2.221 to -1.933	Yes
26057	28:**1 X** vs. 49:**1 X**	-1.981	-2.125 to -1.837	Yes
26058	28:**1 X** vs. 49:**10 X**	-1.975	-2.119 to -1.831	Yes
26059	28:**1 X** vs. 49:**40 X**	0.6540	0.5100 to 0.7980	Yes
26060	28:**1 X** vs. 49:**50 X**	0.8993	0.7554 to 1.043	Yes
26061	28:**1 X** vs. 50:**0 X**	-2.077	-2.221 to -1.933	Yes
26062	28:**1 X** vs. 50:**1 X**	-1.981	-2.125 to -1.837	Yes
26063	28:**1 X** vs. 50:**10 X**	-1.975	-2.119 to -1.831	Yes
26064	28:**1 X** vs. 50:**40 X**	0.6463	0.5024 to 0.7903	Yes
26065	28:**1 X** vs. 50:**50 X**	0.8997	0.7557 to 1.044	Yes
26066	28:**10 X** vs. 28:**40 X**	0.7220	0.5610 to 0.8830	Yes
26067	28:**10 X** vs. 28:**50 X**	0.8267	0.6657 to 0.9876	Yes
26068	28:**10 X** vs. 29:**0 X**	-0.6390	-0.8153 to -0.4627	Yes
26069	28:**10 X** vs. 29:**1 X**	-0.7240	-0.9003 to -0.5477	Yes
26070	28:**10 X** vs. 29:**10 X**	0.06150	-0.1148 to 0.2378	No
26071	28:**10 X** vs. 29:**40 X**	0.6140	0.4530 to 0.7750	Yes
26072	28:**10 X** vs. 29:**50 X**	0.8253	0.6644 to 0.9863	Yes
26073	28:**10 X** vs. 30:**0 X**	-0.3535	-0.5298 to -0.1772	Yes
26074	28:**10 X** vs. 30:**1 X**	-0.6750	-0.8513 to -0.4987	Yes
26075	28:**10 X** vs. 30:**10 X**	-0.01300	-0.1893 to 0.1633	No
26076	28:**10 X** vs. 30:**40 X**	0.6870	0.5260 to 0.8480	Yes
26077	28:**10 X** vs. 30:**50 X**	0.7730	0.6120 to 0.9340	Yes
26078	28:**10 X** vs. 31:**0 X**	-0.6715	-0.8478 to -0.4952	Yes
26079	28:**10 X** vs. 31:**1 X**	-0.6620	-0.8383 to -0.4857	Yes
26080	28:**10 X** vs. 31:**10 X**	-0.1445	-0.3208 to 0.03181	No
26081	28:**10 X** vs. 31:**40 X**	0.5953	0.4344 to 0.7563	Yes
26082	28:**10 X** vs. 31:**50 X**	0.7940	0.6330 to 0.9550	Yes
26083	28:**10 X** vs. 32:**0 X**	-1.263	-1.424 to -1.102	Yes
26084	28:**10 X** vs. 32:**1 X**	-0.7190	-0.8953 to -0.5427	Yes
26085	28:**10 X** vs. 32:**10 X**	-0.5750	-0.7360 to -0.4140	Yes
26086	28:**10 X** vs. 32:**40 X**	0.5990	0.4380 to 0.7600	Yes
26087	28:**10 X** vs. 32:**50 X**	0.7943	0.6334 to 0.9553	Yes
26088	28:**10 X** vs. 33:**0 X**	-1.267	-1.428 to -1.106	Yes
26089	28:**10 X** vs. 33:**1 X**	-1.081	-1.242 to -0.9204	Yes
26090	28:**10 X** vs. 33:**10 X**	-0.7417	-0.9026 to -0.5807	Yes
26091	28:**10 X** vs. 33:**40 X**	0.5803	0.4194 to 0.7413	Yes
26092	28:**10 X** vs. 33:**50 X**	0.7793	0.6184 to 0.9403	Yes
26093	28:**10 X** vs. 34:**0 X**	-1.191	-1.352 to -1.030	Yes
26094	28:**10 X** vs. 34:**1 X**	-1.199	-1.360 to -1.038	Yes
26095	28:**10 X** vs. 34:**10 X**	-0.8320	-0.9930 to -0.6710	Yes
26096	28:**10 X** vs. 34:**40 X**	0.5683	0.4074 to 0.7293	Yes
26097	28:**10 X** vs. 34:**50 X**	0.6303	0.4694 to 0.7913	Yes
26098	28:**10 X** vs. 35:**0 X**	-1.540	-1.701 to -1.379	Yes
26099	28:**10 X** vs. 35:**1 X**	-1.306	-1.467 to -1.145	Yes
26100	28:**10 X** vs. 35:**10 X**	-0.7937	-0.9546 to -0.6327	Yes

	2way ANOVA<br>Multiple comparisons	A<br>Data Set-A<br>Y	B<br>Data Set-B<br>Y	C<br>Data Set-C<br>Y
26101	28:10 X vs. 35:40 X	0.5593	0.3984 to 0.7203	Yes
26102	28:10 X vs. 35:50 X	0.6177	0.4567 to 0.7786	Yes
26103	28:10 X vs. 36:0 X	-1.747	-1.923 to -1.570	Yes
26104	28:10 X vs. 36:1 X	-1.442	-1.603 to -1.281	Yes
26105	28:10 X vs. 36:10 X	-0.7993	-0.9603 to -0.6384	Yes
26106	28:10 X vs. 36:40 X	0.5040	0.3430 to 0.6650	Yes
26107	28:10 X vs. 36:50 X	0.6543	0.4934 to 0.8153	Yes
26108	28:10 X vs. 37:0 X	-2.130	-2.291 to -1.969	Yes
26109	28:10 X vs. 37:1 X	-1.512	-1.673 to -1.351	Yes
26110	28:10 X vs. 37:10 X	-0.8010	-0.9620 to -0.6400	Yes
26111	28:10 X vs. 37:40 X	0.4667	0.3057 to 0.6276	Yes
26112	28:10 X vs. 37:50 X	0.6457	0.4847 to 0.8066	Yes
26113	28:10 X vs. 38:0 X	-2.075	-2.236 to -1.914	Yes
26114	28:10 X vs. 38:1 X	-1.631	-1.792 to -1.470	Yes
26115	28:10 X vs. 38:10 X	-1.076	-1.237 to -0.9154	Yes
26116	28:10 X vs. 38:40 X	0.4623	0.3014 to 0.6233	Yes
26117	28:10 X vs. 38:50 X	0.6417	0.4807 to 0.8026	Yes
26118	28:10 X vs. 39:0 X	-2.306	-2.467 to -2.145	Yes
26119	28:10 X vs. 39:1 X	-2.198	-2.359 to -2.037	Yes
26120	28:10 X vs. 39:10 X	-1.221	-1.382 to -1.060	Yes
26121	28:10 X vs. 39:40 X	0.4563	0.2954 to 0.6173	Yes
26122	28:10 X vs. 39:50 X	0.6107	0.4497 to 0.7716	Yes
26123	28:10 X vs. 40:0 X	-2.347	-2.508 to -2.186	Yes
26124	28:10 X vs. 40:1 X	-2.273	-2.434 to -2.112	Yes
26125	28:10 X vs. 40:10 X	-1.478	-1.639 to -1.317	Yes
26126	28:10 X vs. 40:40 X	0.4517	0.2907 to 0.6126	Yes
26127	28:10 X vs. 40:50 X	0.6043	0.4434 to 0.7653	Yes
26128	28:10 X vs. 41:0 X	-2.388	-2.549 to -2.227	Yes
26129	28:10 X vs. 41:1 X	-2.387	-2.548 to -2.226	Yes
26130	28:10 X vs. 41:10 X	-1.531	-1.692 to -1.370	Yes
26131	28:10 X vs. 41:40 X	0.5043	0.3434 to 0.6653	Yes
26132	28:10 X vs. 41:50 X	0.5917	0.4307 to 0.7526	Yes
26133	28:10 X vs. 42:0 X	-2.530	-2.691 to -2.369	Yes
26134	28:10 X vs. 42:1 X	-2.549	-2.710 to -2.388	Yes
26135	28:10 X vs. 42:10 X	-1.532	-1.693 to -1.371	Yes
26136	28:10 X vs. 42:40 X	0.4917	0.3307 to 0.6526	Yes
26137	28:10 X vs. 42:50 X	0.5883	0.4274 to 0.7493	Yes
26138	28:10 X vs. 43:0 X	-2.620	-2.781 to -2.459	Yes
26139	28:10 X vs. 43:1 X	-2.534	-2.695 to -2.373	Yes
26140	28:10 X vs. 43:10 X	-2.250	-2.411 to -2.089	Yes
26141	28:10 X vs. 43:40 X	0.4933	0.3324 to 0.6543	Yes
26142	28:10 X vs. 43:50 X	0.5880	0.4270 to 0.7490	Yes
26143	28:10 X vs. 44:0 X	-2.644	-2.805 to -2.483	Yes
26144	28:10 X vs. 44:1 X	-2.533	-2.694 to -2.372	Yes
26145	28:10 X vs. 44:10 X	-2.313	-2.474 to -2.152	Yes

	2way ANOVA<br>Multiple comparisons	A<br>Data Set-A<br>Y	B<br>Data Set-B<br>Y	C<br>Data Set-C<br>Y
26146	28:10 X vs. 44:40 X	0.4913	0.3304 to 0.6523	Yes
26147	28:10 X vs. 44:50 X	0.6557	0.4947 to 0.8166	Yes
26148	28:10 X vs. 45:0 X	-2.508	-2.669 to -2.347	Yes
26149	28:10 X vs. 45:1 X	-2.310	-2.471 to -2.149	Yes
26150	28:10 X vs. 45:10 X	-2.193	-2.354 to -2.032	Yes
26151	28:10 X vs. 45:40 X	0.4900	0.3290 to 0.6510	Yes
26152	28:10 X vs. 45:50 X	0.6587	0.4977 to 0.8196	Yes
26153	28:10 X vs. 46:0 X	-2.313	-2.474 to -2.152	Yes
26154	28:10 X vs. 46:1 X	-2.280	-2.441 to -2.119	Yes
26155	28:10 X vs. 46:10 X	-2.188	-2.349 to -2.027	Yes
26156	28:10 X vs. 46:40 X	0.4763	0.3154 to 0.6373	Yes
26157	28:10 X vs. 46:50 X	0.6657	0.5047 to 0.8266	Yes
26158	28:10 X vs. 47:0 X	-2.288	-2.449 to -2.127	Yes
26159	28:10 X vs. 47:1 X	-2.192	-2.353 to -2.031	Yes
26160	28:10 X vs. 47:10 X	-2.174	-2.335 to -2.013	Yes
26161	28:10 X vs. 47:40 X	0.4663	0.3054 to 0.6273	Yes
26162	28:10 X vs. 47:50 X	0.7077	0.5467 to 0.8686	Yes
26163	28:10 X vs. 48:0 X	-2.276	-2.452 to -2.099	Yes
26164	28:10 X vs. 48:1 X	-2.179	-2.340 to -2.018	Yes
26165	28:10 X vs. 48:10 X	-2.174	-2.335 to -2.013	Yes
26166	28:10 X vs. 48:40 X	0.4600	0.2990 to 0.6210	Yes
26167	28:10 X vs. 48:50 X	0.7003	0.5394 to 0.8613	Yes
26168	28:10 X vs. 49:0 X	-2.276	-2.437 to -2.115	Yes
26169	28:10 X vs. 49:1 X	-2.179	-2.340 to -2.018	Yes
26170	28:10 X vs. 49:10 X	-2.174	-2.335 to -2.013	Yes
26171	28:10 X vs. 49:40 X	0.4557	0.2947 to 0.6166	Yes
26172	28:10 X vs. 49:50 X	0.7010	0.5400 to 0.8620	Yes
26173	28:10 X vs. 50:0 X	-2.276	-2.437 to -2.115	Yes
26174	28:10 X vs. 50:1 X	-2.179	-2.340 to -2.018	Yes
26175	28:10 X vs. 50:10 X	-2.174	-2.335 to -2.013	Yes
26176	28:10 X vs. 50:40 X	0.4480	0.2870 to 0.6090	Yes
26177	28:10 X vs. 50:50 X	0.7013	0.5404 to 0.8623	Yes
26178	28:40 X vs. 28:50 X	0.1047	-0.03929 to 0.2486	No
26179	28:40 X vs. 29:0 X	-1.361	-1.522 to -1.200	Yes
26180	28:40 X vs. 29:1 X	-1.446	-1.607 to -1.285	Yes
26181	28:40 X vs. 29:10 X	-0.6605	-0.8215 to -0.4995	Yes
26182	28:40 X vs. 29:40 X	-0.1080	-0.2520 to 0.03596	No
26183	28:40 X vs. 29:50 X	0.1033	-0.04063 to 0.2473	No
26184	28:40 X vs. 30:0 X	-1.076	-1.236 to -0.9145	Yes
26185	28:40 X vs. 30:1 X	-1.397	-1.558 to -1.236	Yes
26186	28:40 X vs. 30:10 X	-0.7350	-0.8960 to -0.5740	Yes
26187	28:40 X vs. 30:40 X	-0.03500	-0.1790 to 0.1090	No
26188	28:40 X vs. 30:50 X	0.05100	-0.09296 to 0.1950	No
26189	28:40 X vs. 31:0 X	-1.394	-1.554 to -1.233	Yes
26190	28:40 X vs. 31:1 X	-1.384	-1.545 to -1.223	Yes

	2way ANOVA<br>Multiple comparisons	A<br>Data Set-A<br>Y	B<br>Data Set-B<br>Y	C<br>Data Set-C<br>Y
26191	28:40 X vs. 31:10 X	-0.8665	-1.027 to -0.7055	Yes
26192	28:40 X vs. 31:40 X	-0.1267	-0.2706 to 0.01729	No
26193	28:40 X vs. 31:50 X	0.0720	-0.07196 to 0.2160	No
26194	28:40 X vs. 32:0 X	-1.985	-2.129 to -1.841	Yes
26195	28:40 X vs. 32:1 X	-1.441	-1.602 to -1.280	Yes
26196	28:40 X vs. 32:10 X	-1.297	-1.441 to -1.153	Yes
26197	28:40 X vs. 32:40 X	-0.1230	-0.2670 to 0.02096	No
26198	28:40 X vs. 32:50 X	0.07233	-0.07163 to 0.2163	No
26199	28:40 X vs. 33:0 X	-1.989	-2.133 to -1.845	Yes
26200	28:40 X vs. 33:1 X	-1.803	-1.947 to -1.659	Yes
26201	28:40 X vs. 33:10 X	-1.464	-1.608 to -1.320	Yes
26202	28:40 X vs. 33:40 X	-0.1417	-0.2856 to 0.002293	No
26203	28:40 X vs. 33:50 X	0.05733	-0.08663 to 0.2013	No
26204	28:40 X vs. 34:0 X	-1.913	-2.057 to -1.769	Yes
26205	28:40 X vs. 34:1 X	-1.921	-2.065 to -1.777	Yes
26206	28:40 X vs. 34:10 X	-1.554	-1.698 to -1.410	Yes
26207	28:40 X vs. 34:40 X	-0.1537	-0.2976 to -0.009707	Yes
26208	28:40 X vs. 34:50 X	-0.09167	-0.2356 to 0.05229	No
26209	28:40 X vs. 35:0 X	-2.262	-2.406 to -2.118	Yes
26210	28:40 X vs. 35:1 X	-2.028	-2.172 to -1.884	Yes
26211	28:40 X vs. 35:10 X	-1.516	-1.660 to -1.372	Yes
26212	28:40 X vs. 35:40 X	-0.1627	-0.3066 to -0.01871	Yes
26213	28:40 X vs. 35:50 X	-0.1043	-0.2483 to 0.03963	No
26214	28:40 X vs. 36:0 X	-2.469	-2.629 to -2.308	Yes
26215	28:40 X vs. 36:1 X	-2.164	-2.308 to -2.020	Yes
26216	28:40 X vs. 36:10 X	-1.521	-1.665 to -1.377	Yes
26217	28:40 X vs. 36:40 X	-0.2180	-0.3620 to -0.07404	Yes
26218	28:40 X vs. 36:50 X	-0.06767	-0.2116 to 0.07629	No
26219	28:40 X vs. 37:0 X	-2.852	-2.996 to -2.708	Yes
26220	28:40 X vs. 37:1 X	-2.234	-2.378 to -2.090	Yes
26221	28:40 X vs. 37:10 X	-1.523	-1.667 to -1.379	Yes
26222	28:40 X vs. 37:40 X	-0.2553	-0.3993 to -0.1114	Yes
26223	28:40 X vs. 37:50 X	-0.07633	-0.2203 to 0.06763	No
26224	28:40 X vs. 38:0 X	-2.797	-2.941 to -2.653	Yes
26225	28:40 X vs. 38:1 X	-2.353	-2.497 to -2.209	Yes
26226	28:40 X vs. 38:10 X	-1.798	-1.942 to -1.654	Yes
26227	28:40 X vs. 38:40 X	-0.2597	-0.4036 to -0.1157	Yes
26228	28:40 X vs. 38:50 X	-0.08033	-0.2243 to 0.06363	No
26229	28:40 X vs. 39:0 X	-3.028	-3.172 to -2.884	Yes
26230	28:40 X vs. 39:1 X	-2.920	-3.064 to -2.776	Yes
26231	28:40 X vs. 39:10 X	-1.943	-2.087 to -1.799	Yes
26232	28:40 X vs. 39:40 X	-0.2657	-0.4096 to -0.1217	Yes
26233	28:40 X vs. 39:50 X	-0.1113	-0.2553 to 0.03263	No
26234	28:40 X vs. 40:0 X	-3.069	-3.213 to -2.925	Yes
26235	28:40 X vs. 40:1 X	-2.995	-3.139 to -2.851	Yes

2way ANOVA Multiple comparisons		A Data Set-A Y	B Data Set-B Y	C Data Set-C Y
26236	28:40 X vs. 40:10 X	-2.200	-2.344 to -2.056	Yes
26237	28:40 X vs. 40:40 X	-0.2703	-0.4143 to -0.1264	Yes
26238	28:40 X vs. 40:50 X	-0.1177	-0.2616 to 0.02629	No
26239	28:40 X vs. 41:0 X	-3.110	-3.254 to -2.966	Yes
26240	28:40 X vs. 41:1 X	-3.109	-3.253 to -2.965	Yes
26241	28:40 X vs. 41:10 X	-2.253	-2.397 to -2.109	Yes
26242	28:40 X vs. 41:40 X	-0.2177	-0.3616 to -0.07371	Yes
26243	28:40 X vs. 41:50 X	-0.1303	-0.2743 to 0.01363	No
26244	28:40 X vs. 42:0 X	-3.252	-3.396 to -3.108	Yes
26245	28:40 X vs. 42:1 X	-3.271	-3.415 to -3.127	Yes
26246	28:40 X vs. 42:10 X	-2.254	-2.398 to -2.110	Yes
26247	28:40 X vs. 42:40 X	-0.2303	-0.3743 to -0.08637	Yes
26248	28:40 X vs. 42:50 X	-0.1337	-0.2776 to 0.01029	No
26249	28:40 X vs. 43:0 X	-3.342	-3.486 to -3.198	Yes
26250	28:40 X vs. 43:1 X	-3.256	-3.400 to -3.112	Yes
26251	28:40 X vs. 43:10 X	-2.972	-3.116 to -2.828	Yes
26252	28:40 X vs. 43:40 X	-0.2287	-0.3726 to -0.08471	Yes
26253	28:40 X vs. 43:50 X	-0.1340	-0.2780 to 0.009960	No
26254	28:40 X vs. 44:0 X	-3.366	-3.510 to -3.222	Yes
26255	28:40 X vs. 44:1 X	-3.255	-3.399 to -3.111	Yes
26256	28:40 X vs. 44:10 X	-3.035	-3.179 to -2.891	Yes
26257	28:40 X vs. 44:40 X	-0.2307	-0.3746 to -0.08671	Yes
26258	28:40 X vs. 44:50 X	-0.06633	-0.2103 to 0.07763	No
26259	28:40 X vs. 45:0 X	-3.230	-3.374 to -3.086	Yes
26260	28:40 X vs. 45:1 X	-3.032	-3.176 to -2.888	Yes
26261	28:40 X vs. 45:10 X	-2.915	-3.059 to -2.771	Yes
26262	28:40 X vs. 45:40 X	-0.2320	-0.3760 to -0.08804	Yes
26263	28:40 X vs. 45:50 X	-0.06333	-0.2073 to 0.08063	No
26264	28:40 X vs. 46:0 X	-3.035	-3.179 to -2.891	Yes
26265	28:40 X vs. 46:1 X	-3.002	-3.146 to -2.858	Yes
26266	28:40 X vs. 46:10 X	-2.910	-3.054 to -2.766	Yes
26267	28:40 X vs. 46:40 X	-0.2457	-0.3896 to -0.1017	Yes
26268	28:40 X vs. 46:50 X	-0.05633	-0.2003 to 0.08763	No
26269	28:40 X vs. 47:0 X	-3.010	-3.154 to -2.866	Yes
26270	28:40 X vs. 47:1 X	-2.914	-3.058 to -2.770	Yes
26271	28:40 X vs. 47:10 X	-2.896	-3.040 to -2.752	Yes
26272	28:40 X vs. 47:40 X	-0.2557	-0.3996 to -0.1117	Yes
26273	28:40 X vs. 47:50 X	-0.01433	-0.1583 to 0.1296	No
26274	28:40 X vs. 48:0 X	-2.998	-3.158 to -2.837	Yes
26275	28:40 X vs. 48:1 X	-2.901	-3.045 to -2.757	Yes
26276	28:40 X vs. 48:10 X	-2.896	-3.040 to -2.752	Yes
26277	28:40 X vs. 48:40 X	-0.2620	-0.4060 to -0.1180	Yes
26278	28:40 X vs. 48:50 X	-0.02167	-0.1656 to 0.1223	No
26279	28:40 X vs. 49:0 X	-2.998	-3.142 to -2.854	Yes
26280	28:40 X vs. 49:1 X	-2.901	-3.045 to -2.757	Yes

	2way ANOVA<br>Multiple comparisons	A<br>Data Set-A<br>Y	B<br>Data Set-B<br>Y	C<br>Data Set-C<br>Y
26281	28:40 X vs. 49:10 X	-2.896	-3.040 to -2.752	Yes
26282	28:40 X vs. 49:40 X	-0.2663	-0.4103 to -0.1224	Yes
26283	28:40 X vs. 49:50 X	-0.0210	-0.1650 to 0.1230	No
26284	28:40 X vs. 50:0 X	-2.998	-3.142 to -2.854	Yes
26285	28:40 X vs. 50:1 X	-2.901	-3.045 to -2.757	Yes
26286	28:40 X vs. 50:10 X	-2.896	-3.040 to -2.752	Yes
26287	28:40 X vs. 50:40 X	-0.2740	-0.4180 to -0.1300	Yes
26288	28:40 X vs. 50:50 X	-0.02067	-0.1646 to 0.1233	No
26289	28:50 X vs. 29:0 X	-1.466	-1.627 to -1.305	Yes
26290	28:50 X vs. 29:1 X	-1.551	-1.712 to -1.390	Yes
26291	28:50 X vs. 29:10 X	-0.7652	-0.9261 to -0.6042	Yes
26292	28:50 X vs. 29:40 X	-0.2127	-0.3566 to -0.06871	Yes
26293	28:50 X vs. 29:50 X	-0.001333	-0.1453 to 0.1426	No
26294	28:50 X vs. 30:0 X	-1.180	-1.341 to -1.019	Yes
26295	28:50 X vs. 30:1 X	-1.502	-1.663 to -1.341	Yes
26296	28:50 X vs. 30:10 X	-0.8397	-1.001 to -0.6787	Yes
26297	28:50 X vs. 30:40 X	-0.1397	-0.2836 to 0.004293	No
26298	28:50 X vs. 30:50 X	-0.05367	-0.1976 to 0.09029	No
26299	28:50 X vs. 31:0 X	-1.498	-1.659 to -1.337	Yes
26300	28:50 X vs. 31:1 X	-1.489	-1.650 to -1.328	Yes
26301	28:50 X vs. 31:10 X	-0.9712	-1.132 to -0.8102	Yes
26302	28:50 X vs. 31:40 X	-0.2313	-0.3753 to -0.08737	Yes
26303	28:50 X vs. 31:50 X	-0.03267	-0.1766 to 0.1113	No
26304	28:50 X vs. 32:0 X	-2.090	-2.234 to -1.946	Yes
26305	28:50 X vs. 32:1 X	-1.546	-1.707 to -1.385	Yes
26306	28:50 X vs. 32:10 X	-1.402	-1.546 to -1.258	Yes
26307	28:50 X vs. 32:40 X	-0.2277	-0.3716 to -0.08371	Yes
26308	28:50 X vs. 32:50 X	-0.03233	-0.1763 to 0.1116	No
26309	28:50 X vs. 33:0 X	-2.093	-2.237 to -1.949	Yes
26310	28:50 X vs. 33:1 X	-1.908	-2.052 to -1.764	Yes
26311	28:50 X vs. 33:10 X	-1.568	-1.712 to -1.424	Yes
26312	28:50 X vs. 33:40 X	-0.2463	-0.3903 to -0.1024	Yes
26313	28:50 X vs. 33:50 X	-0.04733	-0.1913 to 0.09663	No
26314	28:50 X vs. 34:0 X	-2.018	-2.162 to -1.874	Yes
26315	28:50 X vs. 34:1 X	-2.025	-2.169 to -1.881	Yes
26316	28:50 X vs. 34:10 X	-1.659	-1.803 to -1.515	Yes
26317	28:50 X vs. 34:40 X	-0.2583	-0.4023 to -0.1144	Yes
26318	28:50 X vs. 34:50 X	-0.1963	-0.3403 to -0.05237	Yes
26319	28:50 X vs. 35:0 X	-2.367	-2.511 to -2.223	Yes
26320	28:50 X vs. 35:1 X	-2.132	-2.276 to -1.988	Yes
26321	28:50 X vs. 35:10 X	-1.620	-1.764 to -1.476	Yes
26322	28:50 X vs. 35:40 X	-0.2673	-0.4113 to -0.1234	Yes
26323	28:50 X vs. 35:50 X	-0.2090	-0.3530 to -0.06504	Yes
26324	28:50 X vs. 36:0 X	-2.573	-2.734 to -2.412	Yes
26325	28:50 X vs. 36:1 X	-2.269	-2.413 to -2.125	Yes

2way ANOVA<br>Multiple comparisons	A<br>Data Set-A<br>Y	B<br>Data Set-B<br>Y	C<br>Data Set-C<br>Y
26326 28:50 X vs. 36:10 X	-1.626	-1.770 to -1.482	Yes
26327 28:50 X vs. 36:40 X	-0.3227	-0.4666 to -0.1787	Yes
26328 28:50 X vs. 36:50 X	-0.1723	-0.3163 to -0.02837	Yes
26329 28:50 X vs. 37:0 X	-2.957	-3.101 to -2.813	Yes
26330 28:50 X vs. 37:1 X	-2.339	-2.483 to -2.195	Yes
26331 28:50 X vs. 37:10 X	-1.628	-1.772 to -1.484	Yes
26332 28:50 X vs. 37:40 X	-0.3600	-0.5040 to -0.2160	Yes
26333 28:50 X vs. 37:50 X	-0.1810	-0.3250 to -0.03704	Yes
26334 28:50 X vs. 38:0 X	-2.902	-3.046 to -2.758	Yes
26335 28:50 X vs. 38:1 X	-2.458	-2.602 to -2.314	Yes
26336 28:50 X vs. 38:10 X	-1.903	-2.047 to -1.759	Yes
26337 28:50 X vs. 38:40 X	-0.3643	-0.5083 to -0.2204	Yes
26338 28:50 X vs. 38:50 X	-0.1850	-0.3290 to -0.04104	Yes
26339 28:50 X vs. 39:0 X	-3.133	-3.277 to -2.989	Yes
26340 28:50 X vs. 39:1 X	-3.024	-3.168 to -2.880	Yes
26341 28:50 X vs. 39:10 X	-2.048	-2.192 to -1.904	Yes
26342 28:50 X vs. 39:40 X	-0.3703	-0.5143 to -0.2264	Yes
26343 28:50 X vs. 39:50 X	-0.2160	-0.3600 to -0.07204	Yes
26344 28:50 X vs. 40:0 X	-3.174	-3.318 to -3.030	Yes
26345 28:50 X vs. 40:1 X	-3.100	-3.244 to -2.956	Yes
26346 28:50 X vs. 40:10 X	-2.304	-2.448 to -2.160	Yes
26347 28:50 X vs. 40:40 X	-0.3750	-0.5190 to -0.2310	Yes
26348 28:50 X vs. 40:50 X	-0.2223	-0.3663 to -0.07837	Yes
26349 28:50 X vs. 41:0 X	-3.215	-3.359 to -3.071	Yes
26350 28:50 X vs. 41:1 X	-3.214	-3.358 to -3.070	Yes
26351 28:50 X vs. 41:10 X	-2.358	-2.502 to -2.214	Yes
26352 28:50 X vs. 41:40 X	-0.3223	-0.4663 to -0.1784	Yes
26353 28:50 X vs. 41:50 X	-0.2350	-0.3790 to -0.09104	Yes
26354 28:50 X vs. 42:0 X	-3.356	-3.500 to -3.212	Yes
26355 28:50 X vs. 42:1 X	-3.376	-3.520 to -3.232	Yes
26356 28:50 X vs. 42:10 X	-2.358	-2.502 to -2.214	Yes
26357 28:50 X vs. 42:40 X	-0.3350	-0.4790 to -0.1910	Yes
26358 28:50 X vs. 42:50 X	-0.2383	-0.3823 to -0.09437	Yes
26359 28:50 X vs. 43:0 X	-3.446	-3.590 to -3.302	Yes
26360 28:50 X vs. 43:1 X	-3.361	-3.505 to -3.217	Yes
26361 28:50 X vs. 43:10 X	-3.076	-3.220 to -2.932	Yes
26362 28:50 X vs. 43:40 X	-0.3333	-0.4773 to -0.1894	Yes
26363 28:50 X vs. 43:50 X	-0.2387	-0.3826 to -0.09471	Yes
26364 28:50 X vs. 44:0 X	-3.470	-3.614 to -3.326	Yes
26365 28:50 X vs. 44:1 X	-3.360	-3.504 to -3.216	Yes
26366 28:50 X vs. 44:10 X	-3.140	-3.284 to -2.996	Yes
26367 28:50 X vs. 44:40 X	-0.3353	-0.4793 to -0.1914	Yes
26368 28:50 X vs. 44:50 X	-0.1710	-0.3150 to -0.02704	Yes
26369 28:50 X vs. 45:0 X	-3.335	-3.479 to -3.191	Yes
26370 28:50 X vs. 45:1 X	-3.137	-3.281 to -2.993	Yes

2way ANOVA<br>Multiple comparisons		A<br>Data Set-A<br>Y	B<br>Data Set-B<br>Y	C<br>Data Set-C<br>Y
26371	28:**50 X** vs. 45:**10 X**	-3.019	-3.163 to -2.875	Yes
26372	28:**50 X** vs. 45:**40 X**	-0.3367	-0.4806 to -0.1927	Yes
26373	28:**50 X** vs. 45:**50 X**	-0.1680	-0.3120 to -0.02404	Yes
26374	28:**50 X** vs. 46:**0 X**	-3.139	-3.283 to -2.995	Yes
26375	28:**50 X** vs. 46:**1 X**	-3.106	-3.250 to -2.962	Yes
26376	28:**50 X** vs. 46:**10 X**	-3.014	-3.158 to -2.870	Yes
26377	28:**50 X** vs. 46:**40 X**	-0.3503	-0.4943 to -0.2064	Yes
26378	28:**50 X** vs. 46:**50 X**	-0.1610	-0.3050 to -0.01704	Yes
26379	28:**50 X** vs. 47:**0 X**	-3.115	-3.259 to -2.971	Yes
26380	28:**50 X** vs. 47:**1 X**	-3.019	-3.163 to -2.875	Yes
26381	28:**50 X** vs. 47:**10 X**	-3.000	-3.144 to -2.856	Yes
26382	28:**50 X** vs. 47:**40 X**	-0.3603	-0.5043 to -0.2164	Yes
26383	28:**50 X** vs. 47:**50 X**	-0.1190	-0.2630 to 0.02496	No
26384	28:**50 X** vs. 48:**0 X**	-3.102	-3.263 to -2.941	Yes
26385	28:**50 X** vs. 48:**1 X**	-3.006	-3.150 to -2.862	Yes
26386	28:**50 X** vs. 48:**10 X**	-3.000	-3.144 to -2.856	Yes
26387	28:**50 X** vs. 48:**40 X**	-0.3667	-0.5106 to -0.2227	Yes
26388	28:**50 X** vs. 48:**50 X**	-0.1263	-0.2703 to 0.01763	No
26389	28:**50 X** vs. 49:**0 X**	-3.102	-3.246 to -2.958	Yes
26390	28:**50 X** vs. 49:**1 X**	-3.006	-3.150 to -2.862	Yes
26391	28:**50 X** vs. 49:**10 X**	-3.000	-3.144 to -2.856	Yes
26392	28:**50 X** vs. 49:**40 X**	-0.3710	-0.5150 to -0.2270	Yes
26393	28:**50 X** vs. 49:**50 X**	-0.1257	-0.2696 to 0.01829	No
26394	28:**50 X** vs. 50:**0 X**	-3.102	-3.246 to -2.958	Yes
26395	28:**50 X** vs. 50:**1 X**	-3.006	-3.150 to -2.862	Yes
26396	28:**50 X** vs. 50:**10 X**	-3.000	-3.144 to -2.856	Yes
26397	28:**50 X** vs. 50:**40 X**	-0.3787	-0.5226 to -0.2347	Yes
26398	28:**50 X** vs. 50:**50 X**	-0.1253	-0.2693 to 0.01863	No
26399	29:**0 X** vs. 29:**1 X**	-0.08500	-0.2613 to 0.09131	No
26400	29:**0 X** vs. 29:**10 X**	0.7005	0.5242 to 0.8768	Yes
26401	29:**0 X** vs. 29:**40 X**	1.253	1.092 to 1.414	Yes
26402	29:**0 X** vs. 29:**50 X**	1.464	1.303 to 1.625	Yes
26403	29:**0 X** vs. 30:**0 X**	0.2855	0.1092 to 0.4618	Yes
26404	29:**0 X** vs. 30:**1 X**	-0.03600	-0.2123 to 0.1403	No
26405	29:**0 X** vs. 30:**10 X**	0.6260	0.4497 to 0.8023	Yes
26406	29:**0 X** vs. 30:**40 X**	1.326	1.165 to 1.487	Yes
26407	29:**0 X** vs. 30:**50 X**	1.412	1.251 to 1.573	Yes
26408	29:**0 X** vs. 31:**0 X**	-0.03250	-0.2088 to 0.1438	No
26409	29:**0 X** vs. 31:**1 X**	-0.0230	-0.1993 to 0.1533	No
26410	29:**0 X** vs. 31:**10 X**	0.4945	0.3182 to 0.6708	Yes
26411	29:**0 X** vs. 31:**40 X**	1.234	1.073 to 1.395	Yes
26412	29:**0 X** vs. 31:**50 X**	1.433	1.272 to 1.594	Yes
26413	29:**0 X** vs. 32:**0 X**	-0.6240	-0.7850 to -0.4630	Yes
26414	29:**0 X** vs. 32:**1 X**	-0.08000	-0.2563 to 0.09631	No
26415	29:**0 X** vs. 32:**10 X**	0.06400	-0.09695 to 0.2250	No

	2way ANOVA<br>Multiple comparisons	A<br>Data Set-A<br>Y	B<br>Data Set-B<br>Y	C<br>Data Set-C<br>Y
26416	29:**0 X** vs. 32:**40 X**	1.238	1.077 to 1.399	Yes
26417	29:**0 X** vs. 32:**50 X**	1.433	1.272 to 1.594	Yes
26418	29:**0 X** vs. 33:**0 X**	-0.6277	-0.7886 to -0.4667	Yes
26419	29:**0 X** vs. 33:**1 X**	-0.4423	-0.6033 to -0.2814	Yes
26420	29:**0 X** vs. 33:**10 X**	-0.1027	-0.2636 to 0.05829	No
26421	29:**0 X** vs. 33:**40 X**	1.219	1.058 to 1.380	Yes
26422	29:**0 X** vs. 33:**50 X**	1.418	1.257 to 1.579	Yes
26423	29:**0 X** vs. 34:**0 X**	-0.5523	-0.7133 to -0.3914	Yes
26424	29:**0 X** vs. 34:**1 X**	-0.5597	-0.7206 to -0.3987	Yes
26425	29:**0 X** vs. 34:**10 X**	-0.1930	-0.3540 to -0.03205	Yes
26426	29:**0 X** vs. 34:**40 X**	1.207	1.046 to 1.368	Yes
26427	29:**0 X** vs. 34:**50 X**	1.269	1.108 to 1.430	Yes
26428	29:**0 X** vs. 35:**0 X**	-0.9010	-1.062 to -0.7400	Yes
26429	29:**0 X** vs. 35:**1 X**	-0.6667	-0.8276 to -0.5057	Yes
26430	29:**0 X** vs. 35:**10 X**	-0.1547	-0.3156 to 0.006285	No
26431	29:**0 X** vs. 35:**40 X**	1.198	1.037 to 1.359	Yes
26432	29:**0 X** vs. 35:**50 X**	1.257	1.096 to 1.418	Yes
26433	29:**0 X** vs. 36:**0 X**	-1.108	-1.284 to -0.9312	Yes
26434	29:**0 X** vs. 36:**1 X**	-0.8033	-0.9643 to -0.6424	Yes
26435	29:**0 X** vs. 36:**10 X**	-0.1603	-0.3213 to 0.0006186	No
26436	29:**0 X** vs. 36:**40 X**	1.143	0.9820 to 1.304	Yes
26437	29:**0 X** vs. 36:**50 X**	1.293	1.132 to 1.454	Yes
26438	29:**0 X** vs. 37:**0 X**	-1.491	-1.652 to -1.330	Yes
26439	29:**0 X** vs. 37:**1 X**	-0.8733	-1.034 to -0.7124	Yes
26440	29:**0 X** vs. 37:**10 X**	-0.1620	-0.3230 to -0.001048	Yes
26441	29:**0 X** vs. 37:**40 X**	1.106	0.9447 to 1.267	Yes
26442	29:**0 X** vs. 37:**50 X**	1.285	1.124 to 1.446	Yes
26443	29:**0 X** vs. 38:**0 X**	-1.436	-1.597 to -1.275	Yes
26444	29:**0 X** vs. 38:**1 X**	-0.9920	-1.153 to -0.8310	Yes
26445	29:**0 X** vs. 38:**10 X**	-0.4373	-0.5983 to -0.2764	Yes
26446	29:**0 X** vs. 38:**40 X**	1.101	0.9404 to 1.262	Yes
26447	29:**0 X** vs. 38:**50 X**	1.281	1.120 to 1.442	Yes
26448	29:**0 X** vs. 39:**0 X**	-1.667	-1.828 to -1.506	Yes
26449	29:**0 X** vs. 39:**1 X**	-1.559	-1.720 to -1.398	Yes
26450	29:**0 X** vs. 39:**10 X**	-0.5823	-0.7433 to -0.4214	Yes
26451	29:**0 X** vs. 39:**40 X**	1.095	0.9344 to 1.256	Yes
26452	29:**0 X** vs. 39:**50 X**	1.250	1.089 to 1.411	Yes
26453	29:**0 X** vs. 40:**0 X**	-1.708	-1.869 to -1.547	Yes
26454	29:**0 X** vs. 40:**1 X**	-1.634	-1.795 to -1.473	Yes
26455	29:**0 X** vs. 40:**10 X**	-0.8387	-0.9996 to -0.6777	Yes
26456	29:**0 X** vs. 40:**40 X**	1.091	0.9297 to 1.252	Yes
26457	29:**0 X** vs. 40:**50 X**	1.243	1.082 to 1.404	Yes
26458	29:**0 X** vs. 41:**0 X**	-1.749	-1.910 to -1.588	Yes
26459	29:**0 X** vs. 41:**1 X**	-1.748	-1.909 to -1.587	Yes
26460	29:**0 X** vs. 41:**10 X**	-0.8923	-1.053 to -0.7314	Yes

2way ANOVA<br>Multiple comparisons		A<br>Data Set-A<br>Y	B<br>Data Set-B<br>Y	C<br>Data Set-C<br>Y
26461	29:**0 X** vs. 41:**40 X**	1.143	0.9824 to 1.304	Yes
26462	29:**0 X** vs. 41:**50 X**	1.231	1.070 to 1.392	Yes
26463	29:**0 X** vs. 42:**0 X**	-1.891	-2.052 to -1.730	Yes
26464	29:**0 X** vs. 42:**1 X**	-1.910	-2.071 to -1.749	Yes
26465	29:**0 X** vs. 42:**10 X**	-0.8927	-1.054 to -0.7317	Yes
26466	29:**0 X** vs. 42:**40 X**	1.131	0.9697 to 1.292	Yes
26467	29:**0 X** vs. 42:**50 X**	1.227	1.066 to 1.388	Yes
26468	29:**0 X** vs. 43:**0 X**	-1.981	-2.142 to -1.820	Yes
26469	29:**0 X** vs. 43:**1 X**	-1.895	-2.056 to -1.734	Yes
26470	29:**0 X** vs. 43:**10 X**	-1.611	-1.772 to -1.450	Yes
26471	29:**0 X** vs. 43:**40 X**	1.132	0.9714 to 1.293	Yes
26472	29:**0 X** vs. 43:**50 X**	1.227	1.066 to 1.388	Yes
26473	29:**0 X** vs. 44:**0 X**	-2.005	-2.166 to -1.844	Yes
26474	29:**0 X** vs. 44:**1 X**	-1.894	-2.055 to -1.733	Yes
26475	29:**0 X** vs. 44:**10 X**	-1.674	-1.835 to -1.513	Yes
26476	29:**0 X** vs. 44:**40 X**	1.130	0.9694 to 1.291	Yes
26477	29:**0 X** vs. 44:**50 X**	1.295	1.134 to 1.456	Yes
26478	29:**0 X** vs. 45:**0 X**	-1.869	-2.030 to -1.708	Yes
26479	29:**0 X** vs. 45:**1 X**	-1.671	-1.832 to -1.510	Yes
26480	29:**0 X** vs. 45:**10 X**	-1.554	-1.715 to -1.393	Yes
26481	29:**0 X** vs. 45:**40 X**	1.129	0.9680 to 1.290	Yes
26482	29:**0 X** vs. 45:**50 X**	1.298	1.137 to 1.459	Yes
26483	29:**0 X** vs. 46:**0 X**	-1.674	-1.835 to -1.513	Yes
26484	29:**0 X** vs. 46:**1 X**	-1.641	-1.802 to -1.480	Yes
26485	29:**0 X** vs. 46:**10 X**	-1.549	-1.710 to -1.388	Yes
26486	29:**0 X** vs. 46:**40 X**	1.115	0.9544 to 1.276	Yes
26487	29:**0 X** vs. 46:**50 X**	1.305	1.144 to 1.466	Yes
26488	29:**0 X** vs. 47:**0 X**	-1.649	-1.810 to -1.488	Yes
26489	29:**0 X** vs. 47:**1 X**	-1.553	-1.714 to -1.392	Yes
26490	29:**0 X** vs. 47:**10 X**	-1.535	-1.696 to -1.374	Yes
26491	29:**0 X** vs. 47:**40 X**	1.105	0.9444 to 1.266	Yes
26492	29:**0 X** vs. 47:**50 X**	1.347	1.186 to 1.508	Yes
26493	29:**0 X** vs. 48:**0 X**	-1.637	-1.813 to -1.460	Yes
26494	29:**0 X** vs. 48:**1 X**	-1.540	-1.701 to -1.379	Yes
26495	29:**0 X** vs. 48:**10 X**	-1.535	-1.696 to -1.374	Yes
26496	29:**0 X** vs. 48:**40 X**	1.099	0.9380 to 1.260	Yes
26497	29:**0 X** vs. 48:**50 X**	1.339	1.178 to 1.500	Yes
26498	29:**0 X** vs. 49:**0 X**	-1.637	-1.798 to -1.476	Yes
26499	29:**0 X** vs. 49:**1 X**	-1.540	-1.701 to -1.379	Yes
26500	29:**0 X** vs. 49:**10 X**	-1.535	-1.696 to -1.374	Yes
26501	29:**0 X** vs. 49:**40 X**	1.095	0.9337 to 1.256	Yes
26502	29:**0 X** vs. 49:**50 X**	1.340	1.179 to 1.501	Yes
26503	29:**0 X** vs. 50:**0 X**	-1.637	-1.798 to -1.476	Yes
26504	29:**0 X** vs. 50:**1 X**	-1.540	-1.701 to -1.379	Yes
26505	29:**0 X** vs. 50:**10 X**	-1.535	-1.696 to -1.374	Yes

2way ANOVA<br>Multiple comparisons	A<br>Data Set-A<br>Y	B<br>Data Set-B<br>Y	C<br>Data Set-C<br>Y
26506 29:**0 X** vs. 50:**40 X**	1.087	0.9260 to 1.248	Yes
26507 29:**0 X** vs. 50:**50 X**	1.340	1.179 to 1.501	Yes
26508 29:**1 X** vs. 29:**10 X**	0.7855	0.6092 to 0.9618	Yes
26509 29:**1 X** vs. 29:**40 X**	1.338	1.177 to 1.499	Yes
26510 29:**1 X** vs. 29:**50 X**	1.549	1.388 to 1.710	Yes
26511 29:**1 X** vs. 30:**0 X**	0.3705	0.1942 to 0.5468	Yes
26512 29:**1 X** vs. 30:**1 X**	0.04900	-0.1273 to 0.2253	No
26513 29:**1 X** vs. 30:**10 X**	0.7110	0.5347 to 0.8873	Yes
26514 29:**1 X** vs. 30:**40 X**	1.411	1.250 to 1.572	Yes
26515 29:**1 X** vs. 30:**50 X**	1.497	1.336 to 1.658	Yes
26516 29:**1 X** vs. 31:**0 X**	0.05250	-0.1238 to 0.2288	No
26517 29:**1 X** vs. 31:**1 X**	0.06200	-0.1143 to 0.2383	No
26518 29:**1 X** vs. 31:**10 X**	0.5795	0.4032 to 0.7558	Yes
26519 29:**1 X** vs. 31:**40 X**	1.319	1.158 to 1.480	Yes
26520 29:**1 X** vs. 31:**50 X**	1.518	1.357 to 1.679	Yes
26521 29:**1 X** vs. 32:**0 X**	-0.5390	-0.7000 to -0.3780	Yes
26522 29:**1 X** vs. 32:**1 X**	0.005000	-0.1713 to 0.1813	No
26523 29:**1 X** vs. 32:**10 X**	0.1490	-0.01195 to 0.3100	No
26524 29:**1 X** vs. 32:**40 X**	1.323	1.162 to 1.484	Yes
26525 29:**1 X** vs. 32:**50 X**	1.518	1.357 to 1.679	Yes
26526 29:**1 X** vs. 33:**0 X**	-0.5427	-0.7036 to -0.3817	Yes
26527 29:**1 X** vs. 33:**1 X**	-0.3573	-0.5183 to -0.1964	Yes
26528 29:**1 X** vs. 33:**10 X**	-0.01767	-0.1786 to 0.1433	No
26529 29:**1 X** vs. 33:**40 X**	1.304	1.143 to 1.465	Yes
26530 29:**1 X** vs. 33:**50 X**	1.503	1.342 to 1.664	Yes
26531 29:**1 X** vs. 34:**0 X**	-0.4673	-0.6283 to -0.3064	Yes
26532 29:**1 X** vs. 34:**1 X**	-0.4747	-0.6356 to -0.3137	Yes
26533 29:**1 X** vs. 34:**10 X**	-0.1080	-0.2690 to 0.05295	No
26534 29:**1 X** vs. 34:**40 X**	1.292	1.131 to 1.453	Yes
26535 29:**1 X** vs. 34:**50 X**	1.354	1.193 to 1.515	Yes
26536 29:**1 X** vs. 35:**0 X**	-0.8160	-0.9770 to -0.6550	Yes
26537 29:**1 X** vs. 35:**1 X**	-0.5817	-0.7426 to -0.4207	Yes
26538 29:**1 X** vs. 35:**10 X**	-0.06967	-0.2306 to 0.09129	No
26539 29:**1 X** vs. 35:**40 X**	1.283	1.122 to 1.444	Yes
26540 29:**1 X** vs. 35:**50 X**	1.342	1.181 to 1.503	Yes
26541 29:**1 X** vs. 36:**0 X**	-1.023	-1.199 to -0.8462	Yes
26542 29:**1 X** vs. 36:**1 X**	-0.7183	-0.8793 to -0.5574	Yes
26543 29:**1 X** vs. 36:**10 X**	-0.07533	-0.2363 to 0.08562	No
26544 29:**1 X** vs. 36:**40 X**	1.228	1.067 to 1.389	Yes
26545 29:**1 X** vs. 36:**50 X**	1.378	1.217 to 1.539	Yes
26546 29:**1 X** vs. 37:**0 X**	-1.406	-1.567 to -1.245	Yes
26547 29:**1 X** vs. 37:**1 X**	-0.7883	-0.9493 to -0.6274	Yes
26548 29:**1 X** vs. 37:**10 X**	-0.0770	-0.2380 to 0.08395	No
26549 29:**1 X** vs. 37:**40 X**	1.191	1.030 to 1.352	Yes
26550 29:**1 X** vs. 37:**50 X**	1.370	1.209 to 1.531	Yes

	2way ANOVA Multiple comparisons	A Data Set-A Y	B Data Set-B Y	C Data Set-C Y
26551	29:1 X vs. 38:0 X	-1.351	-1.512 to -1.190	Yes
26552	29:1 X vs. 38:1 X	-0.9070	-1.068 to -0.7460	Yes
26553	29:1 X vs. 38:10 X	-0.3523	-0.5133 to -0.1914	Yes
26554	29:1 X vs. 38:40 X	1.186	1.025 to 1.347	Yes
26555	29:1 X vs. 38:50 X	1.366	1.205 to 1.527	Yes
26556	29:1 X vs. 39:0 X	-1.582	-1.743 to -1.421	Yes
26557	29:1 X vs. 39:1 X	-1.474	-1.635 to -1.313	Yes
26558	29:1 X vs. 39:10 X	-0.4973	-0.6583 to -0.3364	Yes
26559	29:1 X vs. 39:40 X	1.180	1.019 to 1.341	Yes
26560	29:1 X vs. 39:50 X	1.335	1.174 to 1.496	Yes
26561	29:1 X vs. 40:0 X	-1.623	-1.784 to -1.462	Yes
26562	29:1 X vs. 40:1 X	-1.549	-1.710 to -1.388	Yes
26563	29:1 X vs. 40:10 X	-0.7537	-0.9146 to -0.5927	Yes
26564	29:1 X vs. 40:40 X	1.176	1.015 to 1.337	Yes
26565	29:1 X vs. 40:50 X	1.328	1.167 to 1.489	Yes
26566	29:1 X vs. 41:0 X	-1.664	-1.825 to -1.503	Yes
26567	29:1 X vs. 41:1 X	-1.663	-1.824 to -1.502	Yes
26568	29:1 X vs. 41:10 X	-0.8073	-0.9683 to -0.6464	Yes
26569	29:1 X vs. 41:40 X	1.228	1.067 to 1.389	Yes
26570	29:1 X vs. 41:50 X	1.316	1.155 to 1.477	Yes
26571	29:1 X vs. 42:0 X	-1.806	-1.967 to -1.645	Yes
26572	29:1 X vs. 42:1 X	-1.825	-1.986 to -1.664	Yes
26573	29:1 X vs. 42:10 X	-0.8077	-0.9686 to -0.6467	Yes
26574	29:1 X vs. 42:40 X	1.216	1.055 to 1.377	Yes
26575	29:1 X vs. 42:50 X	1.312	1.151 to 1.473	Yes
26576	29:1 X vs. 43:0 X	-1.896	-2.057 to -1.735	Yes
26577	29:1 X vs. 43:1 X	-1.810	-1.971 to -1.649	Yes
26578	29:1 X vs. 43:10 X	-1.526	-1.687 to -1.365	Yes
26579	29:1 X vs. 43:40 X	1.217	1.056 to 1.378	Yes
26580	29:1 X vs. 43:50 X	1.312	1.151 to 1.473	Yes
26581	29:1 X vs. 44:0 X	-1.920	-2.081 to -1.759	Yes
26582	29:1 X vs. 44:1 X	-1.809	-1.970 to -1.648	Yes
26583	29:1 X vs. 44:10 X	-1.589	-1.750 to -1.428	Yes
26584	29:1 X vs. 44:40 X	1.215	1.054 to 1.376	Yes
26585	29:1 X vs. 44:50 X	1.380	1.219 to 1.541	Yes
26586	29:1 X vs. 45:0 X	-1.784	-1.945 to -1.623	Yes
26587	29:1 X vs. 45:1 X	-1.586	-1.747 to -1.425	Yes
26588	29:1 X vs. 45:10 X	-1.469	-1.630 to -1.308	Yes
26589	29:1 X vs. 45:40 X	1.214	1.053 to 1.375	Yes
26590	29:1 X vs. 45:50 X	1.383	1.222 to 1.544	Yes
26591	29:1 X vs. 46:0 X	-1.589	-1.750 to -1.428	Yes
26592	29:1 X vs. 46:1 X	-1.556	-1.717 to -1.395	Yes
26593	29:1 X vs. 46:10 X	-1.464	-1.625 to -1.303	Yes
26594	29:1 X vs. 46:40 X	1.200	1.039 to 1.361	Yes
26595	29:1 X vs. 46:50 X	1.390	1.229 to 1.551	Yes

2way ANOVA<br>Multiple comparisons	A<br>Data Set-A<br>Y	B<br>Data Set-B<br>Y	C<br>Data Set-C<br>Y
26596 29:**1 X** vs. 47:**0 X**	-1.564	-1.725 to -1.403	Yes
26597 29:**1 X** vs. 47:**1 X**	-1.468	-1.629 to -1.307	Yes
26598 29:**1 X** vs. 47:**10 X**	-1.450	-1.611 to -1.289	Yes
26599 29:**1 X** vs. 47:**40 X**	1.190	1.029 to 1.351	Yes
26600 29:**1 X** vs. 47:**50 X**	1.432	1.271 to 1.593	Yes
26601 29:**1 X** vs. 48:**0 X**	-1.552	-1.728 to -1.375	Yes
26602 29:**1 X** vs. 48:**1 X**	-1.455	-1.616 to -1.294	Yes
26603 29:**1 X** vs. 48:**10 X**	-1.450	-1.611 to -1.289	Yes
26604 29:**1 X** vs. 48:**40 X**	1.184	1.023 to 1.345	Yes
26605 29:**1 X** vs. 48:**50 X**	1.424	1.263 to 1.585	Yes
26606 29:**1 X** vs. 49:**0 X**	-1.552	-1.713 to -1.391	Yes
26607 29:**1 X** vs. 49:**1 X**	-1.455	-1.616 to -1.294	Yes
26608 29:**1 X** vs. 49:**10 X**	-1.450	-1.611 to -1.289	Yes
26609 29:**1 X** vs. 49:**40 X**	1.180	1.019 to 1.341	Yes
26610 29:**1 X** vs. 49:**50 X**	1.425	1.264 to 1.586	Yes
26611 29:**1 X** vs. 50:**0 X**	-1.552	-1.713 to -1.391	Yes
26612 29:**1 X** vs. 50:**1 X**	-1.455	-1.616 to -1.294	Yes
26613 29:**1 X** vs. 50:**10 X**	-1.450	-1.611 to -1.289	Yes
26614 29:**1 X** vs. 50:**40 X**	1.172	1.011 to 1.333	Yes
26615 29:**1 X** vs. 50:**50 X**	1.425	1.264 to 1.586	Yes
26616 29:**10 X** vs. 29:**40 X**	0.5525	0.3915 to 0.7135	Yes
26617 29:**10 X** vs. 29:**50 X**	0.7638	0.6029 to 0.9248	Yes
26618 29:**10 X** vs. 30:**0 X**	-0.4150	-0.5913 to -0.2387	Yes
26619 29:**10 X** vs. 30:**1 X**	-0.7365	-0.9128 to -0.5602	Yes
26620 29:**10 X** vs. 30:**10 X**	-0.07450	-0.2508 to 0.1018	No
26621 29:**10 X** vs. 30:**40 X**	0.6255	0.4645 to 0.7865	Yes
26622 29:**10 X** vs. 30:**50 X**	0.7115	0.5505 to 0.8725	Yes
26623 29:**10 X** vs. 31:**0 X**	-0.7330	-0.9093 to -0.5567	Yes
26624 29:**10 X** vs. 31:**1 X**	-0.7235	-0.8998 to -0.5472	Yes
26625 29:**10 X** vs. 31:**10 X**	-0.2060	-0.3823 to -0.02969	Yes
26626 29:**10 X** vs. 31:**40 X**	0.5338	0.3729 to 0.6948	Yes
26627 29:**10 X** vs. 31:**50 X**	0.7325	0.5715 to 0.8935	Yes
26628 29:**10 X** vs. 32:**0 X**	-1.325	-1.485 to -1.164	Yes
26629 29:**10 X** vs. 32:**1 X**	-0.7805	-0.9568 to -0.6042	Yes
26630 29:**10 X** vs. 32:**10 X**	-0.6365	-0.7975 to -0.4755	Yes
26631 29:**10 X** vs. 32:**40 X**	0.5375	0.3765 to 0.6985	Yes
26632 29:**10 X** vs. 32:**50 X**	0.7328	0.5719 to 0.8938	Yes
26633 29:**10 X** vs. 33:**0 X**	-1.328	-1.489 to -1.167	Yes
26634 29:**10 X** vs. 33:**1 X**	-1.143	-1.304 to -0.9819	Yes
26635 29:**10 X** vs. 33:**10 X**	-0.8032	-0.9641 to -0.6422	Yes
26636 29:**10 X** vs. 33:**40 X**	0.5188	0.3579 to 0.6798	Yes
26637 29:**10 X** vs. 33:**50 X**	0.7178	0.5569 to 0.8788	Yes
26638 29:**10 X** vs. 34:**0 X**	-1.253	-1.414 to -1.092	Yes
26639 29:**10 X** vs. 34:**1 X**	-1.260	-1.421 to -1.099	Yes
26640 29:**10 X** vs. 34:**10 X**	-0.8935	-1.054 to -0.7325	Yes

2way ANOVA<br>Multiple comparisons	A<br>Data Set-A<br>Y	B<br>Data Set-B<br>Y	C<br>Data Set-C<br>Y
26641 29:10 X vs. 34:40 X	0.5068	0.3459 to 0.6678	Yes
26642 29:10 X vs. 34:50 X	0.5688	0.4079 to 0.7298	Yes
26643 29:10 X vs. 35:0 X	-1.602	-1.762 to -1.441	Yes
26644 29:10 X vs. 35:1 X	-1.367	-1.528 to -1.206	Yes
26645 29:10 X vs. 35:10 X	-0.8552	-1.016 to -0.6942	Yes
26646 29:10 X vs. 35:40 X	0.4978	0.3369 to 0.6588	Yes
26647 29:10 X vs. 35:50 X	0.5562	0.3952 to 0.7171	Yes
26648 29:10 X vs. 36:0 X	-1.808	-1.984 to -1.632	Yes
26649 29:10 X vs. 36:1 X	-1.504	-1.665 to -1.343	Yes
26650 29:10 X vs. 36:10 X	-0.8608	-1.022 to -0.6999	Yes
26651 29:10 X vs. 36:40 X	0.4425	0.2815 to 0.6035	Yes
26652 29:10 X vs. 36:50 X	0.5928	0.4319 to 0.7538	Yes
26653 29:10 X vs. 37:0 X	-2.192	-2.353 to -2.031	Yes
26654 29:10 X vs. 37:1 X	-1.574	-1.735 to -1.413	Yes
26655 29:10 X vs. 37:10 X	-0.8625	-1.023 to -0.7015	Yes
26656 29:10 X vs. 37:40 X	0.4052	0.2442 to 0.5661	Yes
26657 29:10 X vs. 37:50 X	0.5842	0.4232 to 0.7451	Yes
26658 29:10 X vs. 38:0 X	-2.137	-2.298 to -1.976	Yes
26659 29:10 X vs. 38:1 X	-1.693	-1.853 to -1.532	Yes
26660 29:10 X vs. 38:10 X	-1.138	-1.299 to -0.9769	Yes
26661 29:10 X vs. 38:40 X	0.4008	0.2399 to 0.5618	Yes
26662 29:10 X vs. 38:50 X	0.5802	0.4192 to 0.7411	Yes
26663 29:10 X vs. 39:0 X	-2.368	-2.528 to -2.207	Yes
26664 29:10 X vs. 39:1 X	-2.259	-2.420 to -2.098	Yes
26665 29:10 X vs. 39:10 X	-1.283	-1.444 to -1.122	Yes
26666 29:10 X vs. 39:40 X	0.3948	0.2339 to 0.5558	Yes
26667 29:10 X vs. 39:50 X	0.5492	0.3882 to 0.7101	Yes
26668 29:10 X vs. 40:0 X	-2.409	-2.569 to -2.248	Yes
26669 29:10 X vs. 40:1 X	-2.335	-2.495 to -2.174	Yes
26670 29:10 X vs. 40:10 X	-1.539	-1.700 to -1.378	Yes
26671 29:10 X vs. 40:40 X	0.3902	0.2292 to 0.5511	Yes
26672 29:10 X vs. 40:50 X	0.5428	0.3819 to 0.7038	Yes
26673 29:10 X vs. 41:0 X	-2.450	-2.610 to -2.289	Yes
26674 29:10 X vs. 41:1 X	-2.449	-2.609 to -2.288	Yes
26675 29:10 X vs. 41:10 X	-1.593	-1.754 to -1.432	Yes
26676 29:10 X vs. 41:40 X	0.4428	0.2819 to 0.6038	Yes
26677 29:10 X vs. 41:50 X	0.5302	0.3692 to 0.6911	Yes
26678 29:10 X vs. 42:0 X	-2.591	-2.752 to -2.430	Yes
26679 29:10 X vs. 42:1 X	-2.611	-2.772 to -2.450	Yes
26680 29:10 X vs. 42:10 X	-1.593	-1.754 to -1.432	Yes
26681 29:10 X vs. 42:40 X	0.4302	0.2692 to 0.5911	Yes
26682 29:10 X vs. 42:50 X	0.5268	0.3659 to 0.6878	Yes
26683 29:10 X vs. 43:0 X	-2.681	-2.842 to -2.520	Yes
26684 29:10 X vs. 43:1 X	-2.596	-2.756 to -2.435	Yes
26685 29:10 X vs. 43:10 X	-2.311	-2.472 to -2.150	Yes

	2way ANOVA<br>Multiple comparisons	A<br>Data Set-A<br>Y	B<br>Data Set-B<br>Y	C<br>Data Set-C<br>Y
26686	29:10 X vs. 43:40 X	0.4318	0.2709 to 0.5928	Yes
26687	29:10 X vs. 43:50 X	0.5265	0.3655 to 0.6875	Yes
26688	29:10 X vs. 44:0 X	-2.705	-2.866 to -2.544	Yes
26689	29:10 X vs. 44:1 X	-2.595	-2.756 to -2.434	Yes
26690	29:10 X vs. 44:10 X	-2.375	-2.535 to -2.214	Yes
26691	29:10 X vs. 44:40 X	0.4298	0.2689 to 0.5908	Yes
26692	29:10 X vs. 44:50 X	0.5942	0.4332 to 0.7551	Yes
26693	29:10 X vs. 45:0 X	-2.570	-2.731 to -2.409	Yes
26694	29:10 X vs. 45:1 X	-2.372	-2.533 to -2.211	Yes
26695	29:10 X vs. 45:10 X	-2.254	-2.415 to -2.093	Yes
26696	29:10 X vs. 45:40 X	0.4285	0.2675 to 0.5895	Yes
26697	29:10 X vs. 45:50 X	0.5972	0.4362 to 0.7581	Yes
26698	29:10 X vs. 46:0 X	-2.374	-2.535 to -2.213	Yes
26699	29:10 X vs. 46:1 X	-2.341	-2.502 to -2.180	Yes
26700	29:10 X vs. 46:10 X	-2.249	-2.410 to -2.088	Yes
26701	29:10 X vs. 46:40 X	0.4148	0.2539 to 0.5758	Yes
26702	29:10 X vs. 46:50 X	0.6042	0.4432 to 0.7651	Yes
26703	29:10 X vs. 47:0 X	-2.350	-2.511 to -2.189	Yes
26704	29:10 X vs. 47:1 X	-2.254	-2.415 to -2.093	Yes
26705	29:10 X vs. 47:10 X	-2.235	-2.396 to -2.074	Yes
26706	29:10 X vs. 47:40 X	0.4048	0.2439 to 0.5658	Yes
26707	29:10 X vs. 47:50 X	0.6462	0.4852 to 0.8071	Yes
26708	29:10 X vs. 48:0 X	-2.337	-2.513 to -2.161	Yes
26709	29:10 X vs. 48:1 X	-2.241	-2.402 to -2.080	Yes
26710	29:10 X vs. 48:10 X	-2.235	-2.396 to -2.074	Yes
26711	29:10 X vs. 48:40 X	0.3985	0.2375 to 0.5595	Yes
26712	29:10 X vs. 48:50 X	0.6388	0.4779 to 0.7998	Yes
26713	29:10 X vs. 49:0 X	-2.337	-2.498 to -2.176	Yes
26714	29:10 X vs. 49:1 X	-2.241	-2.402 to -2.080	Yes
26715	29:10 X vs. 49:10 X	-2.235	-2.396 to -2.074	Yes
26716	29:10 X vs. 49:40 X	0.3942	0.2332 to 0.5551	Yes
26717	29:10 X vs. 49:50 X	0.6395	0.4785 to 0.8005	Yes
26718	29:10 X vs. 50:0 X	-2.337	-2.498 to -2.176	Yes
26719	29:10 X vs. 50:1 X	-2.241	-2.402 to -2.080	Yes
26720	29:10 X vs. 50:10 X	-2.235	-2.396 to -2.074	Yes
26721	29:10 X vs. 50:40 X	0.3865	0.2255 to 0.5475	Yes
26722	29:10 X vs. 50:50 X	0.6398	0.4789 to 0.8008	Yes
26723	29:40 X vs. 29:50 X	0.2113	0.06737 to 0.3553	Yes
26724	29:40 X vs. 30:0 X	-0.9675	-1.128 to -0.8065	Yes
26725	29:40 X vs. 30:1 X	-1.289	-1.450 to -1.128	Yes
26726	29:40 X vs. 30:10 X	-0.6270	-0.7880 to -0.4660	Yes
26727	29:40 X vs. 30:40 X	0.07300	-0.07096 to 0.2170	No
26728	29:40 X vs. 30:50 X	0.1590	0.01504 to 0.3030	Yes
26729	29:40 X vs. 31:0 X	-1.286	-1.446 to -1.125	Yes
26730	29:40 X vs. 31:1 X	-1.276	-1.437 to -1.115	Yes

2way ANOVA Multiple comparisons	A Data Set-A Y	B Data Set-B Y	C Data Set-C Y
26731 29:40 X vs. 31:10 X	-0.7585	-0.9195 to -0.5975	Yes
26732 29:40 X vs. 31:40 X	-0.01867	-0.1626 to 0.1253	No
26733 29:40 X vs. 31:50 X	0.1800	0.03604 to 0.3240	Yes
26734 29:40 X vs. 32:0 X	-1.877	-2.021 to -1.733	Yes
26735 29:40 X vs. 32:1 X	-1.333	-1.494 to -1.172	Yes
26736 29:40 X vs. 32:10 X	-1.189	-1.333 to -1.045	Yes
26737 29:40 X vs. 32:40 X	-0.01500	-0.1590 to 0.1290	No
26738 29:40 X vs. 32:50 X	0.1803	0.03637 to 0.3243	Yes
26739 29:40 X vs. 33:0 X	-1.881	-2.025 to -1.737	Yes
26740 29:40 X vs. 33:1 X	-1.695	-1.839 to -1.551	Yes
26741 29:40 X vs. 33:10 X	-1.356	-1.500 to -1.212	Yes
26742 29:40 X vs. 33:40 X	-0.03367	-0.1776 to 0.1103	No
26743 29:40 X vs. 33:50 X	0.1653	0.02137 to 0.3093	Yes
26744 29:40 X vs. 34:0 X	-1.805	-1.949 to -1.661	Yes
26745 29:40 X vs. 34:1 X	-1.813	-1.957 to -1.669	Yes
26746 29:40 X vs. 34:10 X	-1.446	-1.590 to -1.302	Yes
26747 29:40 X vs. 34:40 X	-0.04567	-0.1896 to 0.09829	No
26748 29:40 X vs. 34:50 X	0.01633	-0.1276 to 0.1603	No
26749 29:40 X vs. 35:0 X	-2.154	-2.298 to -2.010	Yes
26750 29:40 X vs. 35:1 X	-1.920	-2.064 to -1.776	Yes
26751 29:40 X vs. 35:10 X	-1.408	-1.552 to -1.264	Yes
26752 29:40 X vs. 35:40 X	-0.05467	-0.1986 to 0.08929	No
26753 29:40 X vs. 35:50 X	0.003667	-0.1403 to 0.1476	No
26754 29:40 X vs. 36:0 X	-2.361	-2.521 to -2.200	Yes
26755 29:40 X vs. 36:1 X	-2.056	-2.200 to -1.912	Yes
26756 29:40 X vs. 36:10 X	-1.413	-1.557 to -1.269	Yes
26757 29:40 X vs. 36:40 X	-0.1100	-0.2540 to 0.03396	No
26758 29:40 X vs. 36:50 X	0.04033	-0.1036 to 0.1843	No
26759 29:40 X vs. 37:0 X	-2.744	-2.888 to -2.600	Yes
26760 29:40 X vs. 37:1 X	-2.126	-2.270 to -1.982	Yes
26761 29:40 X vs. 37:10 X	-1.415	-1.559 to -1.271	Yes
26762 29:40 X vs. 37:40 X	-0.1473	-0.2913 to -0.003374	Yes
26763 29:40 X vs. 37:50 X	0.03167	-0.1123 to 0.1756	No
26764 29:40 X vs. 38:0 X	-2.689	-2.833 to -2.545	Yes
26765 29:40 X vs. 38:1 X	-2.245	-2.389 to -2.101	Yes
26766 29:40 X vs. 38:10 X	-1.690	-1.834 to -1.546	Yes
26767 29:40 X vs. 38:40 X	-0.1517	-0.2956 to -0.007707	Yes
26768 29:40 X vs. 38:50 X	0.02767	-0.1163 to 0.1716	No
26769 29:40 X vs. 39:0 X	-2.920	-3.064 to -2.776	Yes
26770 29:40 X vs. 39:1 X	-2.812	-2.956 to -2.668	Yes
26771 29:40 X vs. 39:10 X	-1.835	-1.979 to -1.691	Yes
26772 29:40 X vs. 39:40 X	-0.1577	-0.3016 to -0.01371	Yes
26773 29:40 X vs. 39:50 X	-0.003333	-0.1473 to 0.1406	No
26774 29:40 X vs. 40:0 X	-2.961	-3.105 to -2.817	Yes
26775 29:40 X vs. 40:1 X	-2.887	-3.031 to -2.743	Yes

2way ANOVA<br>Multiple comparisons		A<br>Data Set-A<br>Y	B<br>Data Set-B<br>Y	C<br>Data Set-C<br>Y
26776	29:40 X vs. 40:10 X	-2.092	-2.236 to -1.948	Yes
26777	29:40 X vs. 40:40 X	-0.1623	-0.3063 to -0.01837	Yes
26778	29:40 X vs. 40:50 X	-0.009667	-0.1536 to 0.1343	No
26779	29:40 X vs. 41:0 X	-3.002	-3.146 to -2.858	Yes
26780	29:40 X vs. 41:1 X	-3.001	-3.145 to -2.857	Yes
26781	29:40 X vs. 41:10 X	-2.145	-2.289 to -2.001	Yes
26782	29:40 X vs. 41:40 X	-0.1097	-0.2536 to 0.03429	No
26783	29:40 X vs. 41:50 X	-0.02233	-0.1663 to 0.1216	No
26784	29:40 X vs. 42:0 X	-3.144	-3.288 to -3.000	Yes
26785	29:40 X vs. 42:1 X	-3.163	-3.307 to -3.019	Yes
26786	29:40 X vs. 42:10 X	-2.146	-2.290 to -2.002	Yes
26787	29:40 X vs. 42:40 X	-0.1223	-0.2663 to 0.02163	No
26788	29:40 X vs. 42:50 X	-0.02567	-0.1696 to 0.1183	No
26789	29:40 X vs. 43:0 X	-3.234	-3.378 to -3.090	Yes
26790	29:40 X vs. 43:1 X	-3.148	-3.292 to -3.004	Yes
26791	29:40 X vs. 43:10 X	-2.864	-3.008 to -2.720	Yes
26792	29:40 X vs. 43:40 X	-0.1207	-0.2646 to 0.02329	No
26793	29:40 X vs. 43:50 X	-0.0260	-0.1700 to 0.1180	No
26794	29:40 X vs. 44:0 X	-3.258	-3.402 to -3.114	Yes
26795	29:40 X vs. 44:1 X	-3.147	-3.291 to -3.003	Yes
26796	29:40 X vs. 44:10 X	-2.927	-3.071 to -2.783	Yes
26797	29:40 X vs. 44:40 X	-0.1227	-0.2666 to 0.02129	No
26798	29:40 X vs. 44:50 X	0.04167	-0.1023 to 0.1856	No
26799	29:40 X vs. 45:0 X	-3.122	-3.266 to -2.978	Yes
26800	29:40 X vs. 45:1 X	-2.924	-3.068 to -2.780	Yes
26801	29:40 X vs. 45:10 X	-2.807	-2.951 to -2.663	Yes
26802	29:40 X vs. 45:40 X	-0.1240	-0.2680 to 0.01996	No
26803	29:40 X vs. 45:50 X	0.04467	-0.09929 to 0.1886	No
26804	29:40 X vs. 46:0 X	-2.927	-3.071 to -2.783	Yes
26805	29:40 X vs. 46:1 X	-2.894	-3.038 to -2.750	Yes
26806	29:40 X vs. 46:10 X	-2.802	-2.946 to -2.658	Yes
26807	29:40 X vs. 46:40 X	-0.1377	-0.2816 to 0.006293	No
26808	29:40 X vs. 46:50 X	0.05167	-0.09229 to 0.1956	No
26809	29:40 X vs. 47:0 X	-2.902	-3.046 to -2.758	Yes
26810	29:40 X vs. 47:1 X	-2.806	-2.950 to -2.662	Yes
26811	29:40 X vs. 47:10 X	-2.788	-2.932 to -2.644	Yes
26812	29:40 X vs. 47:40 X	-0.1477	-0.2916 to -0.003707	Yes
26813	29:40 X vs. 47:50 X	0.09367	-0.05029 to 0.2376	No
26814	29:40 X vs. 48:0 X	-2.890	-3.050 to -2.729	Yes
26815	29:40 X vs. 48:1 X	-2.793	-2.937 to -2.649	Yes
26816	29:40 X vs. 48:10 X	-2.788	-2.932 to -2.644	Yes
26817	29:40 X vs. 48:40 X	-0.1540	-0.2980 to -0.01004	Yes
26818	29:40 X vs. 48:50 X	0.08633	-0.05763 to 0.2303	No
26819	29:40 X vs. 49:0 X	-2.890	-3.034 to -2.746	Yes
26820	29:40 X vs. 49:1 X	-2.793	-2.937 to -2.649	Yes

2way ANOVA<br>Multiple comparisons	A<br>Data Set-A<br>Y	B<br>Data Set-B<br>Y	C<br>Data Set-C<br>Y
26821 29:40 X vs. 49:10 X	-2.788	-2.932 to -2.644	Yes
26822 29:40 X vs. 49:40 X	-0.1583	-0.3023 to -0.01437	Yes
26823 29:40 X vs. 49:50 X	0.0870	-0.05696 to 0.2310	No
26824 29:40 X vs. 50:0 X	-2.890	-3.034 to -2.746	Yes
26825 29:40 X vs. 50:1 X	-2.793	-2.937 to -2.649	Yes
26826 29:40 X vs. 50:10 X	-2.788	-2.932 to -2.644	Yes
26827 29:40 X vs. 50:40 X	-0.1660	-0.3100 to -0.02204	Yes
26828 29:40 X vs. 50:50 X	0.08733	-0.05663 to 0.2313	No
26829 29:50 X vs. 30:0 X	-1.179	-1.340 to -1.018	Yes
26830 29:50 X vs. 30:1 X	-1.500	-1.661 to -1.339	Yes
26831 29:50 X vs. 30:10 X	-0.8383	-0.9993 to -0.6774	Yes
26832 29:50 X vs. 30:40 X	-0.1383	-0.2823 to 0.005626	No
26833 29:50 X vs. 30:50 X	-0.05233	-0.1963 to 0.09163	No
26834 29:50 X vs. 31:0 X	-1.497	-1.658 to -1.336	Yes
26835 29:50 X vs. 31:1 X	-1.487	-1.648 to -1.326	Yes
26836 29:50 X vs. 31:10 X	-0.9698	-1.131 to -0.8089	Yes
26837 29:50 X vs. 31:40 X	-0.2300	-0.3740 to -0.08604	Yes
26838 29:50 X vs. 31:50 X	-0.03133	-0.1753 to 0.1126	No
26839 29:50 X vs. 32:0 X	-2.088	-2.232 to -1.944	Yes
26840 29:50 X vs. 32:1 X	-1.544	-1.705 to -1.383	Yes
26841 29:50 X vs. 32:10 X	-1.400	-1.544 to -1.256	Yes
26842 29:50 X vs. 32:40 X	-0.2263	-0.3703 to -0.08237	Yes
26843 29:50 X vs. 32:50 X	-0.03100	-0.1750 to 0.1130	No
26844 29:50 X vs. 33:0 X	-2.092	-2.236 to -1.948	Yes
26845 29:50 X vs. 33:1 X	-1.907	-2.051 to -1.763	Yes
26846 29:50 X vs. 33:10 X	-1.567	-1.711 to -1.423	Yes
26847 29:50 X vs. 33:40 X	-0.2450	-0.3890 to -0.1010	Yes
26848 29:50 X vs. 33:50 X	-0.04600	-0.1900 to 0.09796	No
26849 29:50 X vs. 34:0 X	-2.017	-2.161 to -1.873	Yes
26850 29:50 X vs. 34:1 X	-2.024	-2.168 to -1.880	Yes
26851 29:50 X vs. 34:10 X	-1.657	-1.801 to -1.513	Yes
26852 29:50 X vs. 34:40 X	-0.2570	-0.4010 to -0.1130	Yes
26853 29:50 X vs. 34:50 X	-0.1950	-0.3390 to -0.05104	Yes
26854 29:50 X vs. 35:0 X	-2.365	-2.509 to -2.221	Yes
26855 29:50 X vs. 35:1 X	-2.131	-2.275 to -1.987	Yes
26856 29:50 X vs. 35:10 X	-1.619	-1.763 to -1.475	Yes
26857 29:50 X vs. 35:40 X	-0.2660	-0.4100 to -0.1220	Yes
26858 29:50 X vs. 35:50 X	-0.2077	-0.3516 to -0.06371	Yes
26859 29:50 X vs. 36:0 X	-2.572	-2.733 to -2.411	Yes
26860 29:50 X vs. 36:1 X	-2.268	-2.412 to -2.124	Yes
26861 29:50 X vs. 36:10 X	-1.625	-1.769 to -1.481	Yes
26862 29:50 X vs. 36:40 X	-0.3213	-0.4653 to -0.1774	Yes
26863 29:50 X vs. 36:50 X	-0.1710	-0.3150 to -0.02704	Yes
26864 29:50 X vs. 37:0 X	-2.956	-3.100 to -2.812	Yes
26865 29:50 X vs. 37:1 X	-2.338	-2.482 to -2.194	Yes

	2way ANOVA<br>Multiple comparisons	A<br>Data Set-A<br>Y	B<br>Data Set-B<br>Y	C<br>Data Set-C<br>Y
26866	29:**50 X** vs. 37:**10 X**	-1.626	-1.770 to -1.482	Yes
26867	29:**50 X** vs. 37:**40 X**	-0.3587	-0.5026 to -0.2147	Yes
26868	29:**50 X** vs. 37:**50 X**	-0.1797	-0.3236 to -0.03571	Yes
26869	29:**50 X** vs. 38:**0 X**	-2.901	-3.045 to -2.757	Yes
26870	29:**50 X** vs. 38:**1 X**	-2.456	-2.600 to -2.312	Yes
26871	29:**50 X** vs. 38:**10 X**	-1.902	-2.046 to -1.758	Yes
26872	29:**50 X** vs. 38:**40 X**	-0.3630	-0.5070 to -0.2190	Yes
26873	29:**50 X** vs. 38:**50 X**	-0.1837	-0.3276 to -0.03971	Yes
26874	29:**50 X** vs. 39:**0 X**	-3.131	-3.275 to -2.987	Yes
26875	29:**50 X** vs. 39:**1 X**	-3.023	-3.167 to -2.879	Yes
26876	29:**50 X** vs. 39:**10 X**	-2.047	-2.191 to -1.903	Yes
26877	29:**50 X** vs. 39:**40 X**	-0.3690	-0.5130 to -0.2250	Yes
26878	29:**50 X** vs. 39:**50 X**	-0.2147	-0.3586 to -0.07071	Yes
26879	29:**50 X** vs. 40:**0 X**	-3.172	-3.316 to -3.028	Yes
26880	29:**50 X** vs. 40:**1 X**	-3.098	-3.242 to -2.954	Yes
26881	29:**50 X** vs. 40:**10 X**	-2.303	-2.447 to -2.159	Yes
26882	29:**50 X** vs. 40:**40 X**	-0.3737	-0.5176 to -0.2297	Yes
26883	29:**50 X** vs. 40:**50 X**	-0.2210	-0.3650 to -0.07704	Yes
26884	29:**50 X** vs. 41:**0 X**	-3.213	-3.357 to -3.069	Yes
26885	29:**50 X** vs. 41:**1 X**	-3.212	-3.356 to -3.068	Yes
26886	29:**50 X** vs. 41:**10 X**	-2.357	-2.501 to -2.213	Yes
26887	29:**50 X** vs. 41:**40 X**	-0.3210	-0.4650 to -0.1770	Yes
26888	29:**50 X** vs. 41:**50 X**	-0.2337	-0.3776 to -0.08971	Yes
26889	29:**50 X** vs. 42:**0 X**	-3.355	-3.499 to -3.211	Yes
26890	29:**50 X** vs. 42:**1 X**	-3.375	-3.519 to -3.231	Yes
26891	29:**50 X** vs. 42:**10 X**	-2.357	-2.501 to -2.213	Yes
26892	29:**50 X** vs. 42:**40 X**	-0.3337	-0.4776 to -0.1897	Yes
26893	29:**50 X** vs. 42:**50 X**	-0.2370	-0.3810 to -0.09304	Yes
26894	29:**50 X** vs. 43:**0 X**	-3.445	-3.589 to -3.301	Yes
26895	29:**50 X** vs. 43:**1 X**	-3.359	-3.503 to -3.215	Yes
26896	29:**50 X** vs. 43:**10 X**	-3.075	-3.219 to -2.931	Yes
26897	29:**50 X** vs. 43:**40 X**	-0.3320	-0.4760 to -0.1880	Yes
26898	29:**50 X** vs. 43:**50 X**	-0.2373	-0.3813 to -0.09337	Yes
26899	29:**50 X** vs. 44:**0 X**	-3.469	-3.613 to -3.325	Yes
26900	29:**50 X** vs. 44:**1 X**	-3.359	-3.503 to -3.215	Yes
26901	29:**50 X** vs. 44:**10 X**	-3.138	-3.282 to -2.994	Yes
26902	29:**50 X** vs. 44:**40 X**	-0.3340	-0.4780 to -0.1900	Yes
26903	29:**50 X** vs. 44:**50 X**	-0.1697	-0.3136 to -0.02571	Yes
26904	29:**50 X** vs. 45:**0 X**	-3.334	-3.478 to -3.190	Yes
26905	29:**50 X** vs. 45:**1 X**	-3.136	-3.280 to -2.992	Yes
26906	29:**50 X** vs. 45:**10 X**	-3.018	-3.162 to -2.874	Yes
26907	29:**50 X** vs. 45:**40 X**	-0.3353	-0.4793 to -0.1914	Yes
26908	29:**50 X** vs. 45:**50 X**	-0.1667	-0.3106 to -0.02271	Yes
26909	29:**50 X** vs. 46:**0 X**	-3.138	-3.282 to -2.994	Yes
26910	29:**50 X** vs. 46:**1 X**	-3.105	-3.249 to -2.961	Yes

2way ANOVA<br>Multiple comparisons	A<br>Data Set-A<br>Y	B<br>Data Set-B<br>Y	C<br>Data Set-C<br>Y
26911 29:50 X vs. 46:10 X	-3.013	-3.157 to -2.869	Yes
26912 29:50 X vs. 46:40 X	-0.3490	-0.4930 to -0.2050	Yes
26913 29:50 X vs. 46:50 X	-0.1597	-0.3036 to -0.01571	Yes
26914 29:50 X vs. 47:0 X	-3.114	-3.258 to -2.970	Yes
26915 29:50 X vs. 47:1 X	-3.018	-3.162 to -2.874	Yes
26916 29:50 X vs. 47:10 X	-2.999	-3.143 to -2.855	Yes
26917 29:50 X vs. 47:40 X	-0.3590	-0.5030 to -0.2150	Yes
26918 29:50 X vs. 47:50 X	-0.1177	-0.2616 to 0.02629	No
26919 29:50 X vs. 48:0 X	-3.101	-3.262 to -2.940	Yes
26920 29:50 X vs. 48:1 X	-3.005	-3.149 to -2.861	Yes
26921 29:50 X vs. 48:10 X	-2.999	-3.143 to -2.855	Yes
26922 29:50 X vs. 48:40 X	-0.3653	-0.5093 to -0.2214	Yes
26923 29:50 X vs. 48:50 X	-0.1250	-0.2690 to 0.01896	No
26924 29:50 X vs. 49:0 X	-3.101	-3.245 to -2.957	Yes
26925 29:50 X vs. 49:1 X	-3.005	-3.149 to -2.861	Yes
26926 29:50 X vs. 49:10 X	-2.999	-3.143 to -2.855	Yes
26927 29:50 X vs. 49:40 X	-0.3697	-0.5136 to -0.2257	Yes
26928 29:50 X vs. 49:50 X	-0.1243	-0.2683 to 0.01963	No
26929 29:50 X vs. 50:0 X	-3.101	-3.245 to -2.957	Yes
26930 29:50 X vs. 50:1 X	-3.005	-3.149 to -2.861	Yes
26931 29:50 X vs. 50:10 X	-2.999	-3.143 to -2.855	Yes
26932 29:50 X vs. 50:40 X	-0.3773	-0.5213 to -0.2334	Yes
26933 29:50 X vs. 50:50 X	-0.1240	-0.2680 to 0.01996	No
26934 30:0 X vs. 30:1 X	-0.3215	-0.4978 to -0.1452	Yes
26935 30:0 X vs. 30:10 X	0.3405	0.1642 to 0.5168	Yes
26936 30:0 X vs. 30:40 X	1.041	0.8795 to 1.201	Yes
26937 30:0 X vs. 30:50 X	1.127	0.9655 to 1.287	Yes
26938 30:0 X vs. 31:0 X	-0.3180	-0.4943 to -0.1417	Yes
26939 30:0 X vs. 31:1 X	-0.3085	-0.4848 to -0.1322	Yes
26940 30:0 X vs. 31:10 X	0.2090	0.03269 to 0.3853	Yes
26941 30:0 X vs. 31:40 X	0.9488	0.7879 to 1.110	Yes
26942 30:0 X vs. 31:50 X	1.148	0.9865 to 1.308	Yes
26943 30:0 X vs. 32:0 X	-0.9095	-1.070 to -0.7485	Yes
26944 30:0 X vs. 32:1 X	-0.3655	-0.5418 to -0.1892	Yes
26945 30:0 X vs. 32:10 X	-0.2215	-0.3825 to -0.06055	Yes
26946 30:0 X vs. 32:40 X	0.9525	0.7915 to 1.113	Yes
26947 30:0 X vs. 32:50 X	1.148	0.9869 to 1.309	Yes
26948 30:0 X vs. 33:0 X	-0.9132	-1.074 to -0.7522	Yes
26949 30:0 X vs. 33:1 X	-0.7278	-0.8888 to -0.5669	Yes
26950 30:0 X vs. 33:10 X	-0.3882	-0.5491 to -0.2272	Yes
26951 30:0 X vs. 33:40 X	0.9338	0.7729 to 1.095	Yes
26952 30:0 X vs. 33:50 X	1.133	0.9719 to 1.294	Yes
26953 30:0 X vs. 34:0 X	-0.8378	-0.9988 to -0.6769	Yes
26954 30:0 X vs. 34:1 X	-0.8452	-1.006 to -0.6842	Yes
26955 30:0 X vs. 34:10 X	-0.4785	-0.6395 to -0.3175	Yes

	2way ANOVA Multiple comparisons	A Data Set-A Y	B Data Set-B Y	C Data Set-C Y
26956	30:**0 X** vs. 34:**40 X**	0.9218	0.7609 to 1.083	Yes
26957	30:**0 X** vs. 34:**50 X**	0.9838	0.8229 to 1.145	Yes
26958	30:**0 X** vs. 35:**0 X**	-1.187	-1.347 to -1.026	Yes
26959	30:**0 X** vs. 35:**1 X**	-0.9522	-1.113 to -0.7912	Yes
26960	30:**0 X** vs. 35:**10 X**	-0.4402	-0.6011 to -0.2792	Yes
26961	30:**0 X** vs. 35:**40 X**	0.9128	0.7519 to 1.074	Yes
26962	30:**0 X** vs. 35:**50 X**	0.9712	0.8102 to 1.132	Yes
26963	30:**0 X** vs. 36:**0 X**	-1.393	-1.569 to -1.217	Yes
26964	30:**0 X** vs. 36:**1 X**	-1.089	-1.250 to -0.9279	Yes
26965	30:**0 X** vs. 36:**10 X**	-0.4458	-0.6068 to -0.2849	Yes
26966	30:**0 X** vs. 36:**40 X**	0.8575	0.6965 to 1.018	Yes
26967	30:**0 X** vs. 36:**50 X**	1.008	0.8469 to 1.169	Yes
26968	30:**0 X** vs. 37:**0 X**	-1.777	-1.938 to -1.616	Yes
26969	30:**0 X** vs. 37:**1 X**	-1.159	-1.320 to -0.9979	Yes
26970	30:**0 X** vs. 37:**10 X**	-0.4475	-0.6085 to -0.2865	Yes
26971	30:**0 X** vs. 37:**40 X**	0.8202	0.6592 to 0.9811	Yes
26972	30:**0 X** vs. 37:**50 X**	0.9992	0.8382 to 1.160	Yes
26973	30:**0 X** vs. 38:**0 X**	-1.722	-1.883 to -1.561	Yes
26974	30:**0 X** vs. 38:**1 X**	-1.278	-1.438 to -1.117	Yes
26975	30:**0 X** vs. 38:**10 X**	-0.7228	-0.8838 to -0.5619	Yes
26976	30:**0 X** vs. 38:**40 X**	0.8158	0.6549 to 0.9768	Yes
26977	30:**0 X** vs. 38:**50 X**	0.9952	0.8342 to 1.156	Yes
26978	30:**0 X** vs. 39:**0 X**	-1.953	-2.113 to -1.792	Yes
26979	30:**0 X** vs. 39:**1 X**	-1.844	-2.005 to -1.683	Yes
26980	30:**0 X** vs. 39:**10 X**	-0.8678	-1.029 to -0.7069	Yes
26981	30:**0 X** vs. 39:**40 X**	0.8098	0.6489 to 0.9708	Yes
26982	30:**0 X** vs. 39:**50 X**	0.9642	0.8032 to 1.125	Yes
26983	30:**0 X** vs. 40:**0 X**	-1.994	-2.154 to -1.833	Yes
26984	30:**0 X** vs. 40:**1 X**	-1.920	-2.080 to -1.759	Yes
26985	30:**0 X** vs. 40:**10 X**	-1.124	-1.285 to -0.9632	Yes
26986	30:**0 X** vs. 40:**40 X**	0.8052	0.6442 to 0.9661	Yes
26987	30:**0 X** vs. 40:**50 X**	0.9578	0.7969 to 1.119	Yes
26988	30:**0 X** vs. 41:**0 X**	-2.035	-2.195 to -1.874	Yes
26989	30:**0 X** vs. 41:**1 X**	-2.034	-2.194 to -1.873	Yes
26990	30:**0 X** vs. 41:**10 X**	-1.178	-1.339 to -1.017	Yes
26991	30:**0 X** vs. 41:**40 X**	0.8578	0.6969 to 1.019	Yes
26992	30:**0 X** vs. 41:**50 X**	0.9452	0.7842 to 1.106	Yes
26993	30:**0 X** vs. 42:**0 X**	-2.176	-2.337 to -2.015	Yes
26994	30:**0 X** vs. 42:**1 X**	-2.196	-2.357 to -2.035	Yes
26995	30:**0 X** vs. 42:**10 X**	-1.178	-1.339 to -1.017	Yes
26996	30:**0 X** vs. 42:**40 X**	0.8452	0.6842 to 1.006	Yes
26997	30:**0 X** vs. 42:**50 X**	0.9418	0.7809 to 1.103	Yes
26998	30:**0 X** vs. 43:**0 X**	-2.266	-2.427 to -2.105	Yes
26999	30:**0 X** vs. 43:**1 X**	-2.181	-2.341 to -2.020	Yes
27000	30:**0 X** vs. 43:**10 X**	-1.896	-2.057 to -1.735	Yes

2way ANOVA<br>Multiple comparisons	A<br>Data Set-A<br>Y	B<br>Data Set-B<br>Y	C<br>Data Set-C<br>Y
27001 30:0 X vs. 43:40 X	0.8468	0.6859 to 1.008	Yes
27002 30:0 X vs. 43:50 X	0.9415	0.7805 to 1.102	Yes
27003 30:0 X vs. 44:0 X	-2.290	-2.451 to -2.129	Yes
27004 30:0 X vs. 44:1 X	-2.180	-2.341 to -2.019	Yes
27005 30:0 X vs. 44:10 X	-1.960	-2.120 to -1.799	Yes
27006 30:0 X vs. 44:40 X	0.8448	0.6839 to 1.006	Yes
27007 30:0 X vs. 44:50 X	1.009	0.8482 to 1.170	Yes
27008 30:0 X vs. 45:0 X	-2.155	-2.316 to -1.994	Yes
27009 30:0 X vs. 45:1 X	-1.957	-2.118 to -1.796	Yes
27010 30:0 X vs. 45:10 X	-1.839	-2.000 to -1.678	Yes
27011 30:0 X vs. 45:40 X	0.8435	0.6825 to 1.004	Yes
27012 30:0 X vs. 45:50 X	1.012	0.8512 to 1.173	Yes
27013 30:0 X vs. 46:0 X	-1.959	-2.120 to -1.798	Yes
27014 30:0 X vs. 46:1 X	-1.926	-2.087 to -1.765	Yes
27015 30:0 X vs. 46:10 X	-1.834	-1.995 to -1.673	Yes
27016 30:0 X vs. 46:40 X	0.8298	0.6689 to 0.9908	Yes
27017 30:0 X vs. 46:50 X	1.019	0.8582 to 1.180	Yes
27018 30:0 X vs. 47:0 X	-1.935	-2.096 to -1.774	Yes
27019 30:0 X vs. 47:1 X	-1.839	-2.000 to -1.678	Yes
27020 30:0 X vs. 47:10 X	-1.820	-1.981 to -1.659	Yes
27021 30:0 X vs. 47:40 X	0.8198	0.6589 to 0.9808	Yes
27022 30:0 X vs. 47:50 X	1.061	0.9002 to 1.222	Yes
27023 30:0 X vs. 48:0 X	-1.922	-2.098 to -1.746	Yes
27024 30:0 X vs. 48:1 X	-1.826	-1.987 to -1.665	Yes
27025 30:0 X vs. 48:10 X	-1.820	-1.981 to -1.659	Yes
27026 30:0 X vs. 48:40 X	0.8135	0.6525 to 0.9745	Yes
27027 30:0 X vs. 48:50 X	1.054	0.8929 to 1.215	Yes
27028 30:0 X vs. 49:0 X	-1.922	-2.083 to -1.761	Yes
27029 30:0 X vs. 49:1 X	-1.826	-1.987 to -1.665	Yes
27030 30:0 X vs. 49:10 X	-1.820	-1.981 to -1.659	Yes
27031 30:0 X vs. 49:40 X	0.8092	0.6482 to 0.9701	Yes
27032 30:0 X vs. 49:50 X	1.055	0.8935 to 1.215	Yes
27033 30:0 X vs. 50:0 X	-1.922	-2.083 to -1.761	Yes
27034 30:0 X vs. 50:1 X	-1.826	-1.987 to -1.665	Yes
27035 30:0 X vs. 50:10 X	-1.820	-1.981 to -1.659	Yes
27036 30:0 X vs. 50:40 X	0.8015	0.6405 to 0.9625	Yes
27037 30:0 X vs. 50:50 X	1.055	0.8939 to 1.216	Yes
27038 30:1 X vs. 30:10 X	0.6620	0.4857 to 0.8383	Yes
27039 30:1 X vs. 30:40 X	1.362	1.201 to 1.523	Yes
27040 30:1 X vs. 30:50 X	1.448	1.287 to 1.609	Yes
27041 30:1 X vs. 31:0 X	0.003500	-0.1728 to 0.1798	No
27042 30:1 X vs. 31:1 X	0.01300	-0.1633 to 0.1893	No
27043 30:1 X vs. 31:10 X	0.5305	0.3542 to 0.7068	Yes
27044 30:1 X vs. 31:40 X	1.270	1.109 to 1.431	Yes
27045 30:1 X vs. 31:50 X	1.469	1.308 to 1.630	Yes

2way ANOVA<br>Multiple comparisons	A<br>Data Set-A<br>Y	B<br>Data Set-B<br>Y	C<br>Data Set-C<br>Y
27046 30:1 X vs. 32:0 X	-0.5880	-0.7490 to -0.4270	Yes
27047 30:1 X vs. 32:1 X	-0.04400	-0.2203 to 0.1323	No
27048 30:1 X vs. 32:10 X	0.1000	-0.06095 to 0.2610	No
27049 30:1 X vs. 32:40 X	1.274	1.113 to 1.435	Yes
27050 30:1 X vs. 32:50 X	1.469	1.308 to 1.630	Yes
27051 30:1 X vs. 33:0 X	-0.5917	-0.7526 to -0.4307	Yes
27052 30:1 X vs. 33:1 X	-0.4063	-0.5673 to -0.2454	Yes
27053 30:1 X vs. 33:10 X	-0.06667	-0.2276 to 0.09429	No
27054 30:1 X vs. 33:40 X	1.255	1.094 to 1.416	Yes
27055 30:1 X vs. 33:50 X	1.454	1.293 to 1.615	Yes
27056 30:1 X vs. 34:0 X	-0.5163	-0.6773 to -0.3554	Yes
27057 30:1 X vs. 34:1 X	-0.5237	-0.6846 to -0.3627	Yes
27058 30:1 X vs. 34:10 X	-0.1570	-0.3180 to 0.003952	No
27059 30:1 X vs. 34:40 X	1.243	1.082 to 1.404	Yes
27060 30:1 X vs. 34:50 X	1.305	1.144 to 1.466	Yes
27061 30:1 X vs. 35:0 X	-0.8650	-1.026 to -0.7040	Yes
27062 30:1 X vs. 35:1 X	-0.6307	-0.7916 to -0.4697	Yes
27063 30:1 X vs. 35:10 X	-0.1187	-0.2796 to 0.04229	No
27064 30:1 X vs. 35:40 X	1.234	1.073 to 1.395	Yes
27065 30:1 X vs. 35:50 X	1.293	1.132 to 1.454	Yes
27066 30:1 X vs. 36:0 X	-1.072	-1.248 to -0.8952	Yes
27067 30:1 X vs. 36:1 X	-0.7673	-0.9283 to -0.6064	Yes
27068 30:1 X vs. 36:10 X	-0.1243	-0.2853 to 0.03662	No
27069 30:1 X vs. 36:40 X	1.179	1.018 to 1.340	Yes
27070 30:1 X vs. 36:50 X	1.329	1.168 to 1.490	Yes
27071 30:1 X vs. 37:0 X	-1.455	-1.616 to -1.294	Yes
27072 30:1 X vs. 37:1 X	-0.8373	-0.9983 to -0.6764	Yes
27073 30:1 X vs. 37:10 X	-0.1260	-0.2870 to 0.03495	No
27074 30:1 X vs. 37:40 X	1.142	0.9807 to 1.303	Yes
27075 30:1 X vs. 37:50 X	1.321	1.160 to 1.482	Yes
27076 30:1 X vs. 38:0 X	-1.400	-1.561 to -1.239	Yes
27077 30:1 X vs. 38:1 X	-0.9560	-1.117 to -0.7950	Yes
27078 30:1 X vs. 38:10 X	-0.4013	-0.5623 to -0.2404	Yes
27079 30:1 X vs. 38:40 X	1.137	0.9764 to 1.298	Yes
27080 30:1 X vs. 38:50 X	1.317	1.156 to 1.478	Yes
27081 30:1 X vs. 39:0 X	-1.631	-1.792 to -1.470	Yes
27082 30:1 X vs. 39:1 X	-1.523	-1.684 to -1.362	Yes
27083 30:1 X vs. 39:10 X	-0.5463	-0.7073 to -0.3854	Yes
27084 30:1 X vs. 39:40 X	1.131	0.9704 to 1.292	Yes
27085 30:1 X vs. 39:50 X	1.286	1.125 to 1.447	Yes
27086 30:1 X vs. 40:0 X	-1.672	-1.833 to -1.511	Yes
27087 30:1 X vs. 40:1 X	-1.598	-1.759 to -1.437	Yes
27088 30:1 X vs. 40:10 X	-0.8027	-0.9636 to -0.6417	Yes
27089 30:1 X vs. 40:40 X	1.127	0.9657 to 1.288	Yes
27090 30:1 X vs. 40:50 X	1.279	1.118 to 1.440	Yes

2way ANOVA<br>Multiple comparisons		A<br>Data Set-A<br>Y	B<br>Data Set-B<br>Y	C<br>Data Set-C<br>Y
27091	30:**1 X** vs. 41:**0 X**	-1.713	-1.874 to -1.552	Yes
27092	30:**1 X** vs. 41:**1 X**	-1.712	-1.873 to -1.551	Yes
27093	30:**1 X** vs. 41:**10 X**	-0.8563	-1.017 to -0.6954	Yes
27094	30:**1 X** vs. 41:**40 X**	1.179	1.018 to 1.340	Yes
27095	30:**1 X** vs. 41:**50 X**	1.267	1.106 to 1.428	Yes
27096	30:**1 X** vs. 42:**0 X**	-1.855	-2.016 to -1.694	Yes
27097	30:**1 X** vs. 42:**1 X**	-1.874	-2.035 to -1.713	Yes
27098	30:**1 X** vs. 42:**10 X**	-0.8567	-1.018 to -0.6957	Yes
27099	30:**1 X** vs. 42:**40 X**	1.167	1.006 to 1.328	Yes
27100	30:**1 X** vs. 42:**50 X**	1.263	1.102 to 1.424	Yes
27101	30:**1 X** vs. 43:**0 X**	-1.945	-2.106 to -1.784	Yes
27102	30:**1 X** vs. 43:**1 X**	-1.859	-2.020 to -1.698	Yes
27103	30:**1 X** vs. 43:**10 X**	-1.575	-1.736 to -1.414	Yes
27104	30:**1 X** vs. 43:**40 X**	1.168	1.007 to 1.329	Yes
27105	30:**1 X** vs. 43:**50 X**	1.263	1.102 to 1.424	Yes
27106	30:**1 X** vs. 44:**0 X**	-1.969	-2.130 to -1.808	Yes
27107	30:**1 X** vs. 44:**1 X**	-1.858	-2.019 to -1.697	Yes
27108	30:**1 X** vs. 44:**10 X**	-1.638	-1.799 to -1.477	Yes
27109	30:**1 X** vs. 44:**40 X**	1.166	1.005 to 1.327	Yes
27110	30:**1 X** vs. 44:**50 X**	1.331	1.170 to 1.492	Yes
27111	30:**1 X** vs. 45:**0 X**	-1.833	-1.994 to -1.672	Yes
27112	30:**1 X** vs. 45:**1 X**	-1.635	-1.796 to -1.474	Yes
27113	30:**1 X** vs. 45:**10 X**	-1.518	-1.679 to -1.357	Yes
27114	30:**1 X** vs. 45:**40 X**	1.165	1.004 to 1.326	Yes
27115	30:**1 X** vs. 45:**50 X**	1.334	1.173 to 1.495	Yes
27116	30:**1 X** vs. 46:**0 X**	-1.638	-1.799 to -1.477	Yes
27117	30:**1 X** vs. 46:**1 X**	-1.605	-1.766 to -1.444	Yes
27118	30:**1 X** vs. 46:**10 X**	-1.513	-1.674 to -1.352	Yes
27119	30:**1 X** vs. 46:**40 X**	1.151	0.9904 to 1.312	Yes
27120	30:**1 X** vs. 46:**50 X**	1.341	1.180 to 1.502	Yes
27121	30:**1 X** vs. 47:**0 X**	-1.613	-1.774 to -1.452	Yes
27122	30:**1 X** vs. 47:**1 X**	-1.517	-1.678 to -1.356	Yes
27123	30:**1 X** vs. 47:**10 X**	-1.499	-1.660 to -1.338	Yes
27124	30:**1 X** vs. 47:**40 X**	1.141	0.9804 to 1.302	Yes
27125	30:**1 X** vs. 47:**50 X**	1.383	1.222 to 1.544	Yes
27126	30:**1 X** vs. 48:**0 X**	-1.601	-1.777 to -1.424	Yes
27127	30:**1 X** vs. 48:**1 X**	-1.504	-1.665 to -1.343	Yes
27128	30:**1 X** vs. 48:**10 X**	-1.499	-1.660 to -1.338	Yes
27129	30:**1 X** vs. 48:**40 X**	1.135	0.9740 to 1.296	Yes
27130	30:**1 X** vs. 48:**50 X**	1.375	1.214 to 1.536	Yes
27131	30:**1 X** vs. 49:**0 X**	-1.601	-1.762 to -1.440	Yes
27132	30:**1 X** vs. 49:**1 X**	-1.504	-1.665 to -1.343	Yes
27133	30:**1 X** vs. 49:**10 X**	-1.499	-1.660 to -1.338	Yes
27134	30:**1 X** vs. 49:**40 X**	1.131	0.9697 to 1.292	Yes
27135	30:**1 X** vs. 49:**50 X**	1.376	1.215 to 1.537	Yes

	2way ANOVA<br>Multiple comparisons	A<br>Data Set-A<br>Y	B<br>Data Set-B<br>Y	C<br>Data Set-C<br>Y
27136	30:**1 X** vs. 50:**0 X**	-1.601	-1.762 to -1.440	Yes
27137	30:**1 X** vs. 50:**1 X**	-1.504	-1.665 to -1.343	Yes
27138	30:**1 X** vs. 50:**10 X**	-1.499	-1.660 to -1.338	Yes
27139	30:**1 X** vs. 50:**40 X**	1.123	0.9620 to 1.284	Yes
27140	30:**1 X** vs. 50:**50 X**	1.376	1.215 to 1.537	Yes
27141	30:**10 X** vs. 30:**40 X**	0.7000	0.5390 to 0.8610	Yes
27142	30:**10 X** vs. 30:**50 X**	0.7860	0.6250 to 0.9470	Yes
27143	30:**10 X** vs. 31:**0 X**	-0.6585	-0.8348 to -0.4822	Yes
27144	30:**10 X** vs. 31:**1 X**	-0.6490	-0.8253 to -0.4727	Yes
27145	30:**10 X** vs. 31:**10 X**	-0.1315	-0.3078 to 0.04481	No
27146	30:**10 X** vs. 31:**40 X**	0.6083	0.4474 to 0.7693	Yes
27147	30:**10 X** vs. 31:**50 X**	0.8070	0.6460 to 0.9680	Yes
27148	30:**10 X** vs. 32:**0 X**	-1.250	-1.411 to -1.089	Yes
27149	30:**10 X** vs. 32:**1 X**	-0.7060	-0.8823 to -0.5297	Yes
27150	30:**10 X** vs. 32:**10 X**	-0.5620	-0.7230 to -0.4010	Yes
27151	30:**10 X** vs. 32:**40 X**	0.6120	0.4510 to 0.7730	Yes
27152	30:**10 X** vs. 32:**50 X**	0.8073	0.6464 to 0.9683	Yes
27153	30:**10 X** vs. 33:**0 X**	-1.254	-1.415 to -1.093	Yes
27154	30:**10 X** vs. 33:**1 X**	-1.068	-1.229 to -0.9074	Yes
27155	30:**10 X** vs. 33:**10 X**	-0.7287	-0.8896 to -0.5677	Yes
27156	30:**10 X** vs. 33:**40 X**	0.5933	0.4324 to 0.7543	Yes
27157	30:**10 X** vs. 33:**50 X**	0.7923	0.6314 to 0.9533	Yes
27158	30:**10 X** vs. 34:**0 X**	-1.178	-1.339 to -1.017	Yes
27159	30:**10 X** vs. 34:**1 X**	-1.186	-1.347 to -1.025	Yes
27160	30:**10 X** vs. 34:**10 X**	-0.8190	-0.9800 to -0.6580	Yes
27161	30:**10 X** vs. 34:**40 X**	0.5813	0.4204 to 0.7423	Yes
27162	30:**10 X** vs. 34:**50 X**	0.6433	0.4824 to 0.8043	Yes
27163	30:**10 X** vs. 35:**0 X**	-1.527	-1.688 to -1.366	Yes
27164	30:**10 X** vs. 35:**1 X**	-1.293	-1.454 to -1.132	Yes
27165	30:**10 X** vs. 35:**10 X**	-0.7807	-0.9416 to -0.6197	Yes
27166	30:**10 X** vs. 35:**40 X**	0.5723	0.4114 to 0.7333	Yes
27167	30:**10 X** vs. 35:**50 X**	0.6307	0.4697 to 0.7916	Yes
27168	30:**10 X** vs. 36:**0 X**	-1.734	-1.910 to -1.557	Yes
27169	30:**10 X** vs. 36:**1 X**	-1.429	-1.590 to -1.268	Yes
27170	30:**10 X** vs. 36:**10 X**	-0.7863	-0.9473 to -0.6254	Yes
27171	30:**10 X** vs. 36:**40 X**	0.5170	0.3560 to 0.6780	Yes
27172	30:**10 X** vs. 36:**50 X**	0.6673	0.5064 to 0.8283	Yes
27173	30:**10 X** vs. 37:**0 X**	-2.117	-2.278 to -1.956	Yes
27174	30:**10 X** vs. 37:**1 X**	-1.499	-1.660 to -1.338	Yes
27175	30:**10 X** vs. 37:**10 X**	-0.7880	-0.9490 to -0.6270	Yes
27176	30:**10 X** vs. 37:**40 X**	0.4797	0.3187 to 0.6406	Yes
27177	30:**10 X** vs. 37:**50 X**	0.6587	0.4977 to 0.8196	Yes
27178	30:**10 X** vs. 38:**0 X**	-2.062	-2.223 to -1.901	Yes
27179	30:**10 X** vs. 38:**1 X**	-1.618	-1.779 to -1.457	Yes
27180	30:**10 X** vs. 38:**10 X**	-1.063	-1.224 to -0.9024	Yes

2way ANOVA Multiple comparisons		A Data Set-A	B Data Set-B	C Data Set-C
		Y	Y	Y
27181	30:10 X vs. 38:40 X	0.4753	0.3144 to 0.6363	Yes
27182	30:10 X vs. 38:50 X	0.6547	0.4937 to 0.8156	Yes
27183	30:10 X vs. 39:0 X	-2.293	-2.454 to -2.132	Yes
27184	30:10 X vs. 39:1 X	-2.185	-2.346 to -2.024	Yes
27185	30:10 X vs. 39:10 X	-1.208	-1.369 to -1.047	Yes
27186	30:10 X vs. 39:40 X	0.4693	0.3084 to 0.6303	Yes
27187	30:10 X vs. 39:50 X	0.6237	0.4627 to 0.7846	Yes
27188	30:10 X vs. 40:0 X	-2.334	-2.495 to -2.173	Yes
27189	30:10 X vs. 40:1 X	-2.260	-2.421 to -2.099	Yes
27190	30:10 X vs. 40:10 X	-1.465	-1.626 to -1.304	Yes
27191	30:10 X vs. 40:40 X	0.4647	0.3037 to 0.6256	Yes
27192	30:10 X vs. 40:50 X	0.6173	0.4564 to 0.7783	Yes
27193	30:10 X vs. 41:0 X	-2.375	-2.536 to -2.214	Yes
27194	30:10 X vs. 41:1 X	-2.374	-2.535 to -2.213	Yes
27195	30:10 X vs. 41:10 X	-1.518	-1.679 to -1.357	Yes
27196	30:10 X vs. 41:40 X	0.5173	0.3564 to 0.6783	Yes
27197	30:10 X vs. 41:50 X	0.6047	0.4437 to 0.7656	Yes
27198	30:10 X vs. 42:0 X	-2.517	-2.678 to -2.356	Yes
27199	30:10 X vs. 42:1 X	-2.536	-2.697 to -2.375	Yes
27200	30:10 X vs. 42:10 X	-1.519	-1.680 to -1.358	Yes
27201	30:10 X vs. 42:40 X	0.5047	0.3437 to 0.6656	Yes
27202	30:10 X vs. 42:50 X	0.6013	0.4404 to 0.7623	Yes
27203	30:10 X vs. 43:0 X	-2.607	-2.768 to -2.446	Yes
27204	30:10 X vs. 43:1 X	-2.521	-2.682 to -2.360	Yes
27205	30:10 X vs. 43:10 X	-2.237	-2.398 to -2.076	Yes
27206	30:10 X vs. 43:40 X	0.5063	0.3454 to 0.6673	Yes
27207	30:10 X vs. 43:50 X	0.6010	0.4400 to 0.7620	Yes
27208	30:10 X vs. 44:0 X	-2.631	-2.792 to -2.470	Yes
27209	30:10 X vs. 44:1 X	-2.520	-2.681 to -2.359	Yes
27210	30:10 X vs. 44:10 X	-2.300	-2.461 to -2.139	Yes
27211	30:10 X vs. 44:40 X	0.5043	0.3434 to 0.6653	Yes
27212	30:10 X vs. 44:50 X	0.6687	0.5077 to 0.8296	Yes
27213	30:10 X vs. 45:0 X	-2.495	-2.656 to -2.334	Yes
27214	30:10 X vs. 45:1 X	-2.297	-2.458 to -2.136	Yes
27215	30:10 X vs. 45:10 X	-2.180	-2.341 to -2.019	Yes
27216	30:10 X vs. 45:40 X	0.5030	0.3420 to 0.6640	Yes
27217	30:10 X vs. 45:50 X	0.6717	0.5107 to 0.8326	Yes
27218	30:10 X vs. 46:0 X	-2.300	-2.461 to -2.139	Yes
27219	30:10 X vs. 46:1 X	-2.267	-2.428 to -2.106	Yes
27220	30:10 X vs. 46:10 X	-2.175	-2.336 to -2.014	Yes
27221	30:10 X vs. 46:40 X	0.4893	0.3284 to 0.6503	Yes
27222	30:10 X vs. 46:50 X	0.6787	0.5177 to 0.8396	Yes
27223	30:10 X vs. 47:0 X	-2.275	-2.436 to -2.114	Yes
27224	30:10 X vs. 47:1 X	-2.179	-2.340 to -2.018	Yes
27225	30:10 X vs. 47:10 X	-2.161	-2.322 to -2.000	Yes

	2way ANOVA<br>Multiple comparisons	A<br>Data Set-A<br>Y	B<br>Data Set-B<br>Y	C<br>Data Set-C<br>Y
27226	30:10 X vs. 47:40 X	0.4793	0.3184 to 0.6403	Yes
27227	30:10 X vs. 47:50 X	0.7207	0.5597 to 0.8816	Yes
27228	30:10 X vs. 48:0 X	-2.263	-2.439 to -2.086	Yes
27229	30:10 X vs. 48:1 X	-2.166	-2.327 to -2.005	Yes
27230	30:10 X vs. 48:10 X	-2.161	-2.322 to -2.000	Yes
27231	30:10 X vs. 48:40 X	0.4730	0.3120 to 0.6340	Yes
27232	30:10 X vs. 48:50 X	0.7133	0.5524 to 0.8743	Yes
27233	30:10 X vs. 49:0 X	-2.263	-2.424 to -2.102	Yes
27234	30:10 X vs. 49:1 X	-2.166	-2.327 to -2.005	Yes
27235	30:10 X vs. 49:10 X	-2.161	-2.322 to -2.000	Yes
27236	30:10 X vs. 49:40 X	0.4687	0.3077 to 0.6296	Yes
27237	30:10 X vs. 49:50 X	0.7140	0.5530 to 0.8750	Yes
27238	30:10 X vs. 50:0 X	-2.263	-2.424 to -2.102	Yes
27239	30:10 X vs. 50:1 X	-2.166	-2.327 to -2.005	Yes
27240	30:10 X vs. 50:10 X	-2.161	-2.322 to -2.000	Yes
27241	30:10 X vs. 50:40 X	0.4610	0.3000 to 0.6220	Yes
27242	30:10 X vs. 50:50 X	0.7143	0.5534 to 0.8753	Yes
27243	30:40 X vs. 30:50 X	0.0860	-0.05796 to 0.2300	No
27244	30:40 X vs. 31:0 X	-1.359	-1.519 to -1.198	Yes
27245	30:40 X vs. 31:1 X	-1.349	-1.510 to -1.188	Yes
27246	30:40 X vs. 31:10 X	-0.8315	-0.9925 to -0.6705	Yes
27247	30:40 X vs. 31:40 X	-0.09167	-0.2356 to 0.05229	No
27248	30:40 X vs. 31:50 X	0.1070	-0.03696 to 0.2510	No
27249	30:40 X vs. 32:0 X	-1.950	-2.094 to -1.806	Yes
27250	30:40 X vs. 32:1 X	-1.406	-1.567 to -1.245	Yes
27251	30:40 X vs. 32:10 X	-1.262	-1.406 to -1.118	Yes
27252	30:40 X vs. 32:40 X	-0.0880	-0.2320 to 0.05596	No
27253	30:40 X vs. 32:50 X	0.1073	-0.03663 to 0.2513	No
27254	30:40 X vs. 33:0 X	-1.954	-2.098 to -1.810	Yes
27255	30:40 X vs. 33:1 X	-1.768	-1.912 to -1.624	Yes
27256	30:40 X vs. 33:10 X	-1.429	-1.573 to -1.285	Yes
27257	30:40 X vs. 33:40 X	-0.1067	-0.2506 to 0.03729	No
27258	30:40 X vs. 33:50 X	0.09233	-0.05163 to 0.2363	No
27259	30:40 X vs. 34:0 X	-1.878	-2.022 to -1.734	Yes
27260	30:40 X vs. 34:1 X	-1.886	-2.030 to -1.742	Yes
27261	30:40 X vs. 34:10 X	-1.519	-1.663 to -1.375	Yes
27262	30:40 X vs. 34:40 X	-0.1187	-0.2626 to 0.02529	No
27263	30:40 X vs. 34:50 X	-0.05667	-0.2006 to 0.08729	No
27264	30:40 X vs. 35:0 X	-2.227	-2.371 to -2.083	Yes
27265	30:40 X vs. 35:1 X	-1.993	-2.137 to -1.849	Yes
27266	30:40 X vs. 35:10 X	-1.481	-1.625 to -1.337	Yes
27267	30:40 X vs. 35:40 X	-0.1277	-0.2716 to 0.01629	No
27268	30:40 X vs. 35:50 X	-0.06933	-0.2133 to 0.07463	No
27269	30:40 X vs. 36:0 X	-2.434	-2.594 to -2.273	Yes
27270	30:40 X vs. 36:1 X	-2.129	-2.273 to -1.985	Yes

	2way ANOVA Multiple comparisons	A Data Set-A Y	B Data Set-B Y	C Data Set-C Y
27271	30:**40 X** vs. 36:**10 X**	-1.486	-1.630 to -1.342	Yes
27272	30:**40 X** vs. 36:**40 X**	-0.1830	-0.3270 to -0.03904	Yes
27273	30:**40 X** vs. 36:**50 X**	-0.03267	-0.1766 to 0.1113	No
27274	30:**40 X** vs. 37:**0 X**	-2.817	-2.961 to -2.673	Yes
27275	30:**40 X** vs. 37:**1 X**	-2.199	-2.343 to -2.055	Yes
27276	30:**40 X** vs. 37:**10 X**	-1.488	-1.632 to -1.344	Yes
27277	30:**40 X** vs. 37:**40 X**	-0.2203	-0.3643 to -0.07637	Yes
27278	30:**40 X** vs. 37:**50 X**	-0.04133	-0.1853 to 0.1026	No
27279	30:**40 X** vs. 38:**0 X**	-2.762	-2.906 to -2.618	Yes
27280	30:**40 X** vs. 38:**1 X**	-2.318	-2.462 to -2.174	Yes
27281	30:**40 X** vs. 38:**10 X**	-1.763	-1.907 to -1.619	Yes
27282	30:**40 X** vs. 38:**40 X**	-0.2247	-0.3686 to -0.08071	Yes
27283	30:**40 X** vs. 38:**50 X**	-0.04533	-0.1893 to 0.09863	No
27284	30:**40 X** vs. 39:**0 X**	-2.993	-3.137 to -2.849	Yes
27285	30:**40 X** vs. 39:**1 X**	-2.885	-3.029 to -2.741	Yes
27286	30:**40 X** vs. 39:**10 X**	-1.908	-2.052 to -1.764	Yes
27287	30:**40 X** vs. 39:**40 X**	-0.2307	-0.3746 to -0.08671	Yes
27288	30:**40 X** vs. 39:**50 X**	-0.07633	-0.2203 to 0.06763	No
27289	30:**40 X** vs. 40:**0 X**	-3.034	-3.178 to -2.890	Yes
27290	30:**40 X** vs. 40:**1 X**	-2.960	-3.104 to -2.816	Yes
27291	30:**40 X** vs. 40:**10 X**	-2.165	-2.309 to -2.021	Yes
27292	30:**40 X** vs. 40:**40 X**	-0.2353	-0.3793 to -0.09137	Yes
27293	30:**40 X** vs. 40:**50 X**	-0.08267	-0.2266 to 0.06129	No
27294	30:**40 X** vs. 41:**0 X**	-3.075	-3.219 to -2.931	Yes
27295	30:**40 X** vs. 41:**1 X**	-3.074	-3.218 to -2.930	Yes
27296	30:**40 X** vs. 41:**10 X**	-2.218	-2.362 to -2.074	Yes
27297	30:**40 X** vs. 41:**40 X**	-0.1827	-0.3266 to -0.03871	Yes
27298	30:**40 X** vs. 41:**50 X**	-0.09533	-0.2393 to 0.04863	No
27299	30:**40 X** vs. 42:**0 X**	-3.217	-3.361 to -3.073	Yes
27300	30:**40 X** vs. 42:**1 X**	-3.236	-3.380 to -3.092	Yes
27301	30:**40 X** vs. 42:**10 X**	-2.219	-2.363 to -2.075	Yes
27302	30:**40 X** vs. 42:**40 X**	-0.1953	-0.3393 to -0.05137	Yes
27303	30:**40 X** vs. 42:**50 X**	-0.09867	-0.2426 to 0.04529	No
27304	30:**40 X** vs. 43:**0 X**	-3.307	-3.451 to -3.163	Yes
27305	30:**40 X** vs. 43:**1 X**	-3.221	-3.365 to -3.077	Yes
27306	30:**40 X** vs. 43:**10 X**	-2.937	-3.081 to -2.793	Yes
27307	30:**40 X** vs. 43:**40 X**	-0.1937	-0.3376 to -0.04971	Yes
27308	30:**40 X** vs. 43:**50 X**	-0.0990	-0.2430 to 0.04496	No
27309	30:**40 X** vs. 44:**0 X**	-3.331	-3.475 to -3.187	Yes
27310	30:**40 X** vs. 44:**1 X**	-3.220	-3.364 to -3.076	Yes
27311	30:**40 X** vs. 44:**10 X**	-3.000	-3.144 to -2.856	Yes
27312	30:**40 X** vs. 44:**40 X**	-0.1957	-0.3396 to -0.05171	Yes
27313	30:**40 X** vs. 44:**50 X**	-0.03133	-0.1753 to 0.1126	No
27314	30:**40 X** vs. 45:**0 X**	-3.195	-3.339 to -3.051	Yes
27315	30:**40 X** vs. 45:**1 X**	-2.997	-3.141 to -2.853	Yes

	2way ANOVA<br>Multiple comparisons	A<br>Data Set-A<br>Y	B<br>Data Set-B<br>Y	C<br>Data Set-C<br>Y
27316	30:40 X vs. 45:10 X	-2.880	-3.024 to -2.736	Yes
27317	30:40 X vs. 45:40 X	-0.1970	-0.3410 to -0.05304	Yes
27318	30:40 X vs. 45:50 X	-0.02833	-0.1723 to 0.1156	No
27319	30:40 X vs. 46:0 X	-3.000	-3.144 to -2.856	Yes
27320	30:40 X vs. 46:1 X	-2.967	-3.111 to -2.823	Yes
27321	30:40 X vs. 46:10 X	-2.875	-3.019 to -2.731	Yes
27322	30:40 X vs. 46:40 X	-0.2107	-0.3546 to -0.06671	Yes
27323	30:40 X vs. 46:50 X	-0.02133	-0.1653 to 0.1226	No
27324	30:40 X vs. 47:0 X	-2.975	-3.119 to -2.831	Yes
27325	30:40 X vs. 47:1 X	-2.879	-3.023 to -2.735	Yes
27326	30:40 X vs. 47:10 X	-2.861	-3.005 to -2.717	Yes
27327	30:40 X vs. 47:40 X	-0.2207	-0.3646 to -0.07671	Yes
27328	30:40 X vs. 47:50 X	0.02067	-0.1233 to 0.1646	No
27329	30:40 X vs. 48:0 X	-2.963	-3.123 to -2.802	Yes
27330	30:40 X vs. 48:1 X	-2.866	-3.010 to -2.722	Yes
27331	30:40 X vs. 48:10 X	-2.861	-3.005 to -2.717	Yes
27332	30:40 X vs. 48:40 X	-0.2270	-0.3710 to -0.08304	Yes
27333	30:40 X vs. 48:50 X	0.01333	-0.1306 to 0.1573	No
27334	30:40 X vs. 49:0 X	-2.963	-3.107 to -2.819	Yes
27335	30:40 X vs. 49:1 X	-2.866	-3.010 to -2.722	Yes
27336	30:40 X vs. 49:10 X	-2.861	-3.005 to -2.717	Yes
27337	30:40 X vs. 49:40 X	-0.2313	-0.3753 to -0.08737	Yes
27338	30:40 X vs. 49:50 X	0.01400	-0.1300 to 0.1580	No
27339	30:40 X vs. 50:0 X	-2.963	-3.107 to -2.819	Yes
27340	30:40 X vs. 50:1 X	-2.866	-3.010 to -2.722	Yes
27341	30:40 X vs. 50:10 X	-2.861	-3.005 to -2.717	Yes
27342	30:40 X vs. 50:40 X	-0.2390	-0.3830 to -0.09504	Yes
27343	30:40 X vs. 50:50 X	0.01433	-0.1296 to 0.1583	No
27344	30:50 X vs. 31:0 X	-1.445	-1.605 to -1.284	Yes
27345	30:50 X vs. 31:1 X	-1.435	-1.596 to -1.274	Yes
27346	30:50 X vs. 31:10 X	-0.9175	-1.078 to -0.7565	Yes
27347	30:50 X vs. 31:40 X	-0.1777	-0.3216 to -0.03371	Yes
27348	30:50 X vs. 31:50 X	0.02100	-0.1230 to 0.1650	No
27349	30:50 X vs. 32:0 X	-2.036	-2.180 to -1.892	Yes
27350	30:50 X vs. 32:1 X	-1.492	-1.653 to -1.331	Yes
27351	30:50 X vs. 32:10 X	-1.348	-1.492 to -1.204	Yes
27352	30:50 X vs. 32:40 X	-0.1740	-0.3180 to -0.03004	Yes
27353	30:50 X vs. 32:50 X	0.02133	-0.1226 to 0.1653	No
27354	30:50 X vs. 33:0 X	-2.040	-2.184 to -1.896	Yes
27355	30:50 X vs. 33:1 X	-1.854	-1.998 to -1.710	Yes
27356	30:50 X vs. 33:10 X	-1.515	-1.659 to -1.371	Yes
27357	30:50 X vs. 33:40 X	-0.1927	-0.3366 to -0.04871	Yes
27358	30:50 X vs. 33:50 X	0.006333	-0.1376 to 0.1503	No
27359	30:50 X vs. 34:0 X	-1.964	-2.108 to -1.820	Yes
27360	30:50 X vs. 34:1 X	-1.972	-2.116 to -1.828	Yes

	2way ANOVA<br>Multiple comparisons	A<br>Data Set-A<br>Y	B<br>Data Set-B<br>Y	C<br>Data Set-C<br>Y
27361	30:50 X vs. 34:10 X	-1.605	-1.749 to -1.461	Yes
27362	30:50 X vs. 34:40 X	-0.2047	-0.3486 to -0.06071	Yes
27363	30:50 X vs. 34:50 X	-0.1427	-0.2866 to 0.001293	No
27364	30:50 X vs. 35:0 X	-2.313	-2.457 to -2.169	Yes
27365	30:50 X vs. 35:1 X	-2.079	-2.223 to -1.935	Yes
27366	30:50 X vs. 35:10 X	-1.567	-1.711 to -1.423	Yes
27367	30:50 X vs. 35:40 X	-0.2137	-0.3576 to -0.06971	Yes
27368	30:50 X vs. 35:50 X	-0.1553	-0.2993 to -0.01137	Yes
27369	30:50 X vs. 36:0 X	-2.520	-2.680 to -2.359	Yes
27370	30:50 X vs. 36:1 X	-2.215	-2.359 to -2.071	Yes
27371	30:50 X vs. 36:10 X	-1.572	-1.716 to -1.428	Yes
27372	30:50 X vs. 36:40 X	-0.2690	-0.4130 to -0.1250	Yes
27373	30:50 X vs. 36:50 X	-0.1187	-0.2626 to 0.02529	No
27374	30:50 X vs. 37:0 X	-2.903	-3.047 to -2.759	Yes
27375	30:50 X vs. 37:1 X	-2.285	-2.429 to -2.141	Yes
27376	30:50 X vs. 37:10 X	-1.574	-1.718 to -1.430	Yes
27377	30:50 X vs. 37:40 X	-0.3063	-0.4503 to -0.1624	Yes
27378	30:50 X vs. 37:50 X	-0.1273	-0.2713 to 0.01663	No
27379	30:50 X vs. 38:0 X	-2.848	-2.992 to -2.704	Yes
27380	30:50 X vs. 38:1 X	-2.404	-2.548 to -2.260	Yes
27381	30:50 X vs. 38:10 X	-1.849	-1.993 to -1.705	Yes
27382	30:50 X vs. 38:40 X	-0.3107	-0.4546 to -0.1667	Yes
27383	30:50 X vs. 38:50 X	-0.1313	-0.2753 to 0.01263	No
27384	30:50 X vs. 39:0 X	-3.079	-3.223 to -2.935	Yes
27385	30:50 X vs. 39:1 X	-2.971	-3.115 to -2.827	Yes
27386	30:50 X vs. 39:10 X	-1.994	-2.138 to -1.850	Yes
27387	30:50 X vs. 39:40 X	-0.3167	-0.4606 to -0.1727	Yes
27388	30:50 X vs. 39:50 X	-0.1623	-0.3063 to -0.01837	Yes
27389	30:50 X vs. 40:0 X	-3.120	-3.264 to -2.976	Yes
27390	30:50 X vs. 40:1 X	-3.046	-3.190 to -2.902	Yes
27391	30:50 X vs. 40:10 X	-2.251	-2.395 to -2.107	Yes
27392	30:50 X vs. 40:40 X	-0.3213	-0.4653 to -0.1774	Yes
27393	30:50 X vs. 40:50 X	-0.1687	-0.3126 to -0.02471	Yes
27394	30:50 X vs. 41:0 X	-3.161	-3.305 to -3.017	Yes
27395	30:50 X vs. 41:1 X	-3.160	-3.304 to -3.016	Yes
27396	30:50 X vs. 41:10 X	-2.304	-2.448 to -2.160	Yes
27397	30:50 X vs. 41:40 X	-0.2687	-0.4126 to -0.1247	Yes
27398	30:50 X vs. 41:50 X	-0.1813	-0.3253 to -0.03737	Yes
27399	30:50 X vs. 42:0 X	-3.303	-3.447 to -3.159	Yes
27400	30:50 X vs. 42:1 X	-3.322	-3.466 to -3.178	Yes
27401	30:50 X vs. 42:10 X	-2.305	-2.449 to -2.161	Yes
27402	30:50 X vs. 42:40 X	-0.2813	-0.4253 to -0.1374	Yes
27403	30:50 X vs. 42:50 X	-0.1847	-0.3286 to -0.04071	Yes
27404	30:50 X vs. 43:0 X	-3.393	-3.537 to -3.249	Yes
27405	30:50 X vs. 43:1 X	-3.307	-3.451 to -3.163	Yes

2way ANOVA<br>Multiple comparisons	A<br>Data Set-A<br>Y	B<br>Data Set-B<br>Y	C<br>Data Set-C<br>Y
27406 30:**50 X** vs. 43:**10 X**	-3.023	-3.167 to -2.879	Yes
27407 30:**50 X** vs. 43:**40 X**	-0.2797	-0.4236 to -0.1357	Yes
27408 30:**50 X** vs. 43:**50 X**	-0.1850	-0.3290 to -0.04104	Yes
27409 30:**50 X** vs. 44:**0 X**	-3.417	-3.561 to -3.273	Yes
27410 30:**50 X** vs. 44:**1 X**	-3.306	-3.450 to -3.162	Yes
27411 30:**50 X** vs. 44:**10 X**	-3.086	-3.230 to -2.942	Yes
27412 30:**50 X** vs. 44:**40 X**	-0.2817	-0.4256 to -0.1377	Yes
27413 30:**50 X** vs. 44:**50 X**	-0.1173	-0.2613 to 0.02663	No
27414 30:**50 X** vs. 45:**0 X**	-3.281	-3.425 to -3.137	Yes
27415 30:**50 X** vs. 45:**1 X**	-3.083	-3.227 to -2.939	Yes
27416 30:**50 X** vs. 45:**10 X**	-2.966	-3.110 to -2.822	Yes
27417 30:**50 X** vs. 45:**40 X**	-0.2830	-0.4270 to -0.1390	Yes
27418 30:**50 X** vs. 45:**50 X**	-0.1143	-0.2583 to 0.02963	No
27419 30:**50 X** vs. 46:**0 X**	-3.086	-3.230 to -2.942	Yes
27420 30:**50 X** vs. 46:**1 X**	-3.053	-3.197 to -2.909	Yes
27421 30:**50 X** vs. 46:**10 X**	-2.961	-3.105 to -2.817	Yes
27422 30:**50 X** vs. 46:**40 X**	-0.2967	-0.4406 to -0.1527	Yes
27423 30:**50 X** vs. 46:**50 X**	-0.1073	-0.2513 to 0.03663	No
27424 30:**50 X** vs. 47:**0 X**	-3.061	-3.205 to -2.917	Yes
27425 30:**50 X** vs. 47:**1 X**	-2.965	-3.109 to -2.821	Yes
27426 30:**50 X** vs. 47:**10 X**	-2.947	-3.091 to -2.803	Yes
27427 30:**50 X** vs. 47:**40 X**	-0.3067	-0.4506 to -0.1627	Yes
27428 30:**50 X** vs. 47:**50 X**	-0.06533	-0.2093 to 0.07863	No
27429 30:**50 X** vs. 48:**0 X**	-3.049	-3.209 to -2.888	Yes
27430 30:**50 X** vs. 48:**1 X**	-2.952	-3.096 to -2.808	Yes
27431 30:**50 X** vs. 48:**10 X**	-2.947	-3.091 to -2.803	Yes
27432 30:**50 X** vs. 48:**40 X**	-0.3130	-0.4570 to -0.1690	Yes
27433 30:**50 X** vs. 48:**50 X**	-0.07267	-0.2166 to 0.07129	No
27434 30:**50 X** vs. 49:**0 X**	-3.049	-3.193 to -2.905	Yes
27435 30:**50 X** vs. 49:**1 X**	-2.952	-3.096 to -2.808	Yes
27436 30:**50 X** vs. 49:**10 X**	-2.947	-3.091 to -2.803	Yes
27437 30:**50 X** vs. 49:**40 X**	-0.3173	-0.4613 to -0.1734	Yes
27438 30:**50 X** vs. 49:**50 X**	-0.07200	-0.2160 to 0.07196	No
27439 30:**50 X** vs. 50:**0 X**	-3.049	-3.193 to -2.905	Yes
27440 30:**50 X** vs. 50:**1 X**	-2.952	-3.096 to -2.808	Yes
27441 30:**50 X** vs. 50:**10 X**	-2.947	-3.091 to -2.803	Yes
27442 30:**50 X** vs. 50:**40 X**	-0.3250	-0.4690 to -0.1810	Yes
27443 30:**50 X** vs. 50:**50 X**	-0.07167	-0.2156 to 0.07229	No
27444 31:**0 X** vs. 31:**1 X**	0.009500	-0.1668 to 0.1858	No
27445 31:**0 X** vs. 31:**10 X**	0.5270	0.3507 to 0.7033	Yes
27446 31:**0 X** vs. 31:**40 X**	1.267	1.106 to 1.428	Yes
27447 31:**0 X** vs. 31:**50 X**	1.466	1.305 to 1.626	Yes
27448 31:**0 X** vs. 32:**0 X**	-0.5915	-0.7525 to -0.4305	Yes
27449 31:**0 X** vs. 32:**1 X**	-0.04750	-0.2238 to 0.1288	No
27450 31:**0 X** vs. 32:**10 X**	0.09650	-0.06445 to 0.2575	No

2way ANOVA<br>Multiple comparisons	A<br>Data Set-A<br>Y	B<br>Data Set-B<br>Y	C<br>Data Set-C<br>Y
27451 31:0 X vs. 32:40 X	1.271	1.110 to 1.431	Yes
27452 31:0 X vs. 32:50 X	1.466	1.305 to 1.627	Yes
27453 31:0 X vs. 33:0 X	-0.5952	-0.7561 to -0.4342	Yes
27454 31:0 X vs. 33:1 X	-0.4098	-0.5708 to -0.2489	Yes
27455 31:0 X vs. 33:10 X	-0.07017	-0.2311 to 0.09079	No
27456 31:0 X vs. 33:40 X	1.252	1.091 to 1.413	Yes
27457 31:0 X vs. 33:50 X	1.451	1.290 to 1.612	Yes
27458 31:0 X vs. 34:0 X	-0.5198	-0.6808 to -0.3589	Yes
27459 31:0 X vs. 34:1 X	-0.5272	-0.6881 to -0.3662	Yes
27460 31:0 X vs. 34:10 X	-0.1605	-0.3215 to 0.0004520	No
27461 31:0 X vs. 34:40 X	1.240	1.079 to 1.401	Yes
27462 31:0 X vs. 34:50 X	1.302	1.141 to 1.463	Yes
27463 31:0 X vs. 35:0 X	-0.8685	-1.029 to -0.7075	Yes
27464 31:0 X vs. 35:1 X	-0.6342	-0.7951 to -0.4732	Yes
27465 31:0 X vs. 35:10 X	-0.1222	-0.2831 to 0.03879	No
27466 31:0 X vs. 35:40 X	1.231	1.070 to 1.392	Yes
27467 31:0 X vs. 35:50 X	1.289	1.128 to 1.450	Yes
27468 31:0 X vs. 36:0 X	-1.075	-1.251 to -0.8987	Yes
27469 31:0 X vs. 36:1 X	-0.7708	-0.9318 to -0.6099	Yes
27470 31:0 X vs. 36:10 X	-0.1278	-0.2888 to 0.03312	No
27471 31:0 X vs. 36:40 X	1.176	1.015 to 1.336	Yes
27472 31:0 X vs. 36:50 X	1.326	1.165 to 1.487	Yes
27473 31:0 X vs. 37:0 X	-1.459	-1.620 to -1.298	Yes
27474 31:0 X vs. 37:1 X	-0.8408	-1.002 to -0.6799	Yes
27475 31:0 X vs. 37:10 X	-0.1295	-0.2905 to 0.03145	No
27476 31:0 X vs. 37:40 X	1.138	0.9772 to 1.299	Yes
27477 31:0 X vs. 37:50 X	1.317	1.156 to 1.478	Yes
27478 31:0 X vs. 38:0 X	-1.404	-1.565 to -1.243	Yes
27479 31:0 X vs. 38:1 X	-0.9595	-1.120 to -0.7985	Yes
27480 31:0 X vs. 38:10 X	-0.4048	-0.5658 to -0.2439	Yes
27481 31:0 X vs. 38:40 X	1.134	0.9729 to 1.295	Yes
27482 31:0 X vs. 38:50 X	1.313	1.152 to 1.474	Yes
27483 31:0 X vs. 39:0 X	-1.635	-1.795 to -1.474	Yes
27484 31:0 X vs. 39:1 X	-1.526	-1.687 to -1.365	Yes
27485 31:0 X vs. 39:10 X	-0.5498	-0.7108 to -0.3889	Yes
27486 31:0 X vs. 39:40 X	1.128	0.9669 to 1.289	Yes
27487 31:0 X vs. 39:50 X	1.282	1.121 to 1.443	Yes
27488 31:0 X vs. 40:0 X	-1.676	-1.836 to -1.515	Yes
27489 31:0 X vs. 40:1 X	-1.602	-1.762 to -1.441	Yes
27490 31:0 X vs. 40:10 X	-0.8062	-0.9671 to -0.6452	Yes
27491 31:0 X vs. 40:40 X	1.123	0.9622 to 1.284	Yes
27492 31:0 X vs. 40:50 X	1.276	1.115 to 1.437	Yes
27493 31:0 X vs. 41:0 X	-1.717	-1.877 to -1.556	Yes
27494 31:0 X vs. 41:1 X	-1.716	-1.876 to -1.555	Yes
27495 31:0 X vs. 41:10 X	-0.8598	-1.021 to -0.6989	Yes

	2way ANOVA<br>Multiple comparisons	A<br>Data Set-A<br>Y	B<br>Data Set-B<br>Y	C<br>Data Set-C<br>Y
27496	31:0 X vs. 41:40 X	1.176	1.015 to 1.337	Yes
27497	31:0 X vs. 41:50 X	1.263	1.102 to 1.424	Yes
27498	31:0 X vs. 42:0 X	-1.858	-2.019 to -1.697	Yes
27499	31:0 X vs. 42:1 X	-1.878	-2.039 to -1.717	Yes
27500	31:0 X vs. 42:10 X	-0.8602	-1.021 to -0.6992	Yes
27501	31:0 X vs. 42:40 X	1.163	1.002 to 1.324	Yes
27502	31:0 X vs. 42:50 X	1.260	1.099 to 1.421	Yes
27503	31:0 X vs. 43:0 X	-1.948	-2.109 to -1.787	Yes
27504	31:0 X vs. 43:1 X	-1.863	-2.023 to -1.702	Yes
27505	31:0 X vs. 43:10 X	-1.578	-1.739 to -1.417	Yes
27506	31:0 X vs. 43:40 X	1.165	1.004 to 1.326	Yes
27507	31:0 X vs. 43:50 X	1.260	1.099 to 1.420	Yes
27508	31:0 X vs. 44:0 X	-1.972	-2.133 to -1.811	Yes
27509	31:0 X vs. 44:1 X	-1.862	-2.023 to -1.701	Yes
27510	31:0 X vs. 44:10 X	-1.642	-1.802 to -1.481	Yes
27511	31:0 X vs. 44:40 X	1.163	1.002 to 1.324	Yes
27512	31:0 X vs. 44:50 X	1.327	1.166 to 1.488	Yes
27513	31:0 X vs. 45:0 X	-1.837	-1.998 to -1.676	Yes
27514	31:0 X vs. 45:1 X	-1.639	-1.800 to -1.478	Yes
27515	31:0 X vs. 45:10 X	-1.521	-1.682 to -1.360	Yes
27516	31:0 X vs. 45:40 X	1.162	1.001 to 1.322	Yes
27517	31:0 X vs. 45:50 X	1.330	1.169 to 1.491	Yes
27518	31:0 X vs. 46:0 X	-1.641	-1.802 to -1.480	Yes
27519	31:0 X vs. 46:1 X	-1.608	-1.769 to -1.447	Yes
27520	31:0 X vs. 46:10 X	-1.516	-1.677 to -1.355	Yes
27521	31:0 X vs. 46:40 X	1.148	0.9869 to 1.309	Yes
27522	31:0 X vs. 46:50 X	1.337	1.176 to 1.498	Yes
27523	31:0 X vs. 47:0 X	-1.617	-1.778 to -1.456	Yes
27524	31:0 X vs. 47:1 X	-1.521	-1.682 to -1.360	Yes
27525	31:0 X vs. 47:10 X	-1.502	-1.663 to -1.341	Yes
27526	31:0 X vs. 47:40 X	1.138	0.9769 to 1.299	Yes
27527	31:0 X vs. 47:50 X	1.379	1.218 to 1.540	Yes
27528	31:0 X vs. 48:0 X	-1.604	-1.780 to -1.428	Yes
27529	31:0 X vs. 48:1 X	-1.508	-1.669 to -1.347	Yes
27530	31:0 X vs. 48:10 X	-1.502	-1.663 to -1.341	Yes
27531	31:0 X vs. 48:40 X	1.132	0.9705 to 1.292	Yes
27532	31:0 X vs. 48:50 X	1.372	1.211 to 1.533	Yes
27533	31:0 X vs. 49:0 X	-1.604	-1.765 to -1.443	Yes
27534	31:0 X vs. 49:1 X	-1.508	-1.669 to -1.347	Yes
27535	31:0 X vs. 49:10 X	-1.502	-1.663 to -1.341	Yes
27536	31:0 X vs. 49:40 X	1.127	0.9662 to 1.288	Yes
27537	31:0 X vs. 49:50 X	1.373	1.212 to 1.533	Yes
27538	31:0 X vs. 50:0 X	-1.604	-1.765 to -1.443	Yes
27539	31:0 X vs. 50:1 X	-1.508	-1.669 to -1.347	Yes
27540	31:0 X vs. 50:10 X	-1.502	-1.663 to -1.341	Yes

2way ANOVA<br>Multiple comparisons	A<br>Data Set-A<br>Y	B<br>Data Set-B<br>Y	C<br>Data Set-C<br>Y
27541 31:0 X vs. 50:40 X	1.120	0.9585 to 1.280	Yes
27542 31:0 X vs. 50:50 X	1.373	1.212 to 1.534	Yes
27543 31:1 X vs. 31:10 X	0.5175	0.3412 to 0.6938	Yes
27544 31:1 X vs. 31:40 X	1.257	1.096 to 1.418	Yes
27545 31:1 X vs. 31:50 X	1.456	1.295 to 1.617	Yes
27546 31:1 X vs. 32:0 X	-0.6010	-0.7620 to -0.4400	Yes
27547 31:1 X vs. 32:1 X	-0.05700	-0.2333 to 0.1193	No
27548 31:1 X vs. 32:10 X	0.08700	-0.07395 to 0.2480	No
27549 31:1 X vs. 32:40 X	1.261	1.100 to 1.422	Yes
27550 31:1 X vs. 32:50 X	1.456	1.295 to 1.617	Yes
27551 31:1 X vs. 33:0 X	-0.6047	-0.7656 to -0.4437	Yes
27552 31:1 X vs. 33:1 X	-0.4193	-0.5803 to -0.2584	Yes
27553 31:1 X vs. 33:10 X	-0.07967	-0.2406 to 0.08129	No
27554 31:1 X vs. 33:40 X	1.242	1.081 to 1.403	Yes
27555 31:1 X vs. 33:50 X	1.441	1.280 to 1.602	Yes
27556 31:1 X vs. 34:0 X	-0.5293	-0.6903 to -0.3684	Yes
27557 31:1 X vs. 34:1 X	-0.5367	-0.6976 to -0.3757	Yes
27558 31:1 X vs. 34:10 X	-0.1700	-0.3310 to -0.009048	Yes
27559 31:1 X vs. 34:40 X	1.230	1.069 to 1.391	Yes
27560 31:1 X vs. 34:50 X	1.292	1.131 to 1.453	Yes
27561 31:1 X vs. 35:0 X	-0.8780	-1.039 to -0.7170	Yes
27562 31:1 X vs. 35:1 X	-0.6437	-0.8046 to -0.4827	Yes
27563 31:1 X vs. 35:10 X	-0.1317	-0.2926 to 0.02929	No
27564 31:1 X vs. 35:40 X	1.221	1.060 to 1.382	Yes
27565 31:1 X vs. 35:50 X	1.280	1.119 to 1.441	Yes
27566 31:1 X vs. 36:0 X	-1.085	-1.261 to -0.9082	Yes
27567 31:1 X vs. 36:1 X	-0.7803	-0.9413 to -0.6194	Yes
27568 31:1 X vs. 36:10 X	-0.1373	-0.2983 to 0.02362	No
27569 31:1 X vs. 36:40 X	1.166	1.005 to 1.327	Yes
27570 31:1 X vs. 36:50 X	1.316	1.155 to 1.477	Yes
27571 31:1 X vs. 37:0 X	-1.468	-1.629 to -1.307	Yes
27572 31:1 X vs. 37:1 X	-0.8503	-1.011 to -0.6894	Yes
27573 31:1 X vs. 37:10 X	-0.1390	-0.3000 to 0.02195	No
27574 31:1 X vs. 37:40 X	1.129	0.9677 to 1.290	Yes
27575 31:1 X vs. 37:50 X	1.308	1.147 to 1.469	Yes
27576 31:1 X vs. 38:0 X	-1.413	-1.574 to -1.252	Yes
27577 31:1 X vs. 38:1 X	-0.9690	-1.130 to -0.8080	Yes
27578 31:1 X vs. 38:10 X	-0.4143	-0.5753 to -0.2534	Yes
27579 31:1 X vs. 38:40 X	1.124	0.9634 to 1.285	Yes
27580 31:1 X vs. 38:50 X	1.304	1.143 to 1.465	Yes
27581 31:1 X vs. 39:0 X	-1.644	-1.805 to -1.483	Yes
27582 31:1 X vs. 39:1 X	-1.536	-1.697 to -1.375	Yes
27583 31:1 X vs. 39:10 X	-0.5593	-0.7203 to -0.3984	Yes
27584 31:1 X vs. 39:40 X	1.118	0.9574 to 1.279	Yes
27585 31:1 X vs. 39:50 X	1.273	1.112 to 1.434	Yes

	2way ANOVA<br>Multiple comparisons	A<br>Data Set-A<br>Y	B<br>Data Set-B<br>Y	C<br>Data Set-C<br>Y
27586	31:**1 X** vs. 40:**0 X**	-1.685	-1.846 to -1.524	Yes
27587	31:**1 X** vs. 40:**1 X**	-1.611	-1.772 to -1.450	Yes
27588	31:**1 X** vs. 40:**10 X**	-0.8157	-0.9766 to -0.6547	Yes
27589	31:**1 X** vs. 40:**40 X**	1.114	0.9527 to 1.275	Yes
27590	31:**1 X** vs. 40:**50 X**	1.266	1.105 to 1.427	Yes
27591	31:**1 X** vs. 41:**0 X**	-1.726	-1.887 to -1.565	Yes
27592	31:**1 X** vs. 41:**1 X**	-1.725	-1.886 to -1.564	Yes
27593	31:**1 X** vs. 41:**10 X**	-0.8693	-1.030 to -0.7084	Yes
27594	31:**1 X** vs. 41:**40 X**	1.166	1.005 to 1.327	Yes
27595	31:**1 X** vs. 41:**50 X**	1.254	1.093 to 1.415	Yes
27596	31:**1 X** vs. 42:**0 X**	-1.868	-2.029 to -1.707	Yes
27597	31:**1 X** vs. 42:**1 X**	-1.887	-2.048 to -1.726	Yes
27598	31:**1 X** vs. 42:**10 X**	-0.8697	-1.031 to -0.7087	Yes
27599	31:**1 X** vs. 42:**40 X**	1.154	0.9927 to 1.315	Yes
27600	31:**1 X** vs. 42:**50 X**	1.250	1.089 to 1.411	Yes
27601	31:**1 X** vs. 43:**0 X**	-1.958	-2.119 to -1.797	Yes
27602	31:**1 X** vs. 43:**1 X**	-1.872	-2.033 to -1.711	Yes
27603	31:**1 X** vs. 43:**10 X**	-1.588	-1.749 to -1.427	Yes
27604	31:**1 X** vs. 43:**40 X**	1.155	0.9944 to 1.316	Yes
27605	31:**1 X** vs. 43:**50 X**	1.250	1.089 to 1.411	Yes
27606	31:**1 X** vs. 44:**0 X**	-1.982	-2.143 to -1.821	Yes
27607	31:**1 X** vs. 44:**1 X**	-1.871	-2.032 to -1.710	Yes
27608	31:**1 X** vs. 44:**10 X**	-1.651	-1.812 to -1.490	Yes
27609	31:**1 X** vs. 44:**40 X**	1.153	0.9924 to 1.314	Yes
27610	31:**1 X** vs. 44:**50 X**	1.318	1.157 to 1.479	Yes
27611	31:**1 X** vs. 45:**0 X**	-1.846	-2.007 to -1.685	Yes
27612	31:**1 X** vs. 45:**1 X**	-1.648	-1.809 to -1.487	Yes
27613	31:**1 X** vs. 45:**10 X**	-1.531	-1.692 to -1.370	Yes
27614	31:**1 X** vs. 45:**40 X**	1.152	0.9910 to 1.313	Yes
27615	31:**1 X** vs. 45:**50 X**	1.321	1.160 to 1.482	Yes
27616	31:**1 X** vs. 46:**0 X**	-1.651	-1.812 to -1.490	Yes
27617	31:**1 X** vs. 46:**1 X**	-1.618	-1.779 to -1.457	Yes
27618	31:**1 X** vs. 46:**10 X**	-1.526	-1.687 to -1.365	Yes
27619	31:**1 X** vs. 46:**40 X**	1.138	0.9774 to 1.299	Yes
27620	31:**1 X** vs. 46:**50 X**	1.328	1.167 to 1.489	Yes
27621	31:**1 X** vs. 47:**0 X**	-1.626	-1.787 to -1.465	Yes
27622	31:**1 X** vs. 47:**1 X**	-1.530	-1.691 to -1.369	Yes
27623	31:**1 X** vs. 47:**10 X**	-1.512	-1.673 to -1.351	Yes
27624	31:**1 X** vs. 47:**40 X**	1.128	0.9674 to 1.289	Yes
27625	31:**1 X** vs. 47:**50 X**	1.370	1.209 to 1.531	Yes
27626	31:**1 X** vs. 48:**0 X**	-1.614	-1.790 to -1.437	Yes
27627	31:**1 X** vs. 48:**1 X**	-1.517	-1.678 to -1.356	Yes
27628	31:**1 X** vs. 48:**10 X**	-1.512	-1.673 to -1.351	Yes
27629	31:**1 X** vs. 48:**40 X**	1.122	0.9610 to 1.283	Yes
27630	31:**1 X** vs. 48:**50 X**	1.362	1.201 to 1.523	Yes

2way ANOVA<br>Multiple comparisons		A<br>Data Set-A<br>Y	B<br>Data Set-B<br>Y	C<br>Data Set-C<br>Y
27631	31:**1 X** vs. 49:**0 X**	-1.614	-1.775 to -1.453	Yes
27632	31:**1 X** vs. 49:**1 X**	-1.517	-1.678 to -1.356	Yes
27633	31:**1 X** vs. 49:**10 X**	-1.512	-1.673 to -1.351	Yes
27634	31:**1 X** vs. 49:**40 X**	1.118	0.9567 to 1.279	Yes
27635	31:**1 X** vs. 49:**50 X**	1.363	1.202 to 1.524	Yes
27636	31:**1 X** vs. 50:**0 X**	-1.614	-1.775 to -1.453	Yes
27637	31:**1 X** vs. 50:**1 X**	-1.517	-1.678 to -1.356	Yes
[truncated: 334,624 more chars]
