# Supplementary material for: Changes in fatty acid composition as a response to glyphosate toxicity in Pseudomonas fluorescens
Source: Heliyon. 2022 Jul 13;8(8):e09938. doi: 10.1016/j.heliyon.2022.e09938 (PMC9364109; doi:10.1016/j.heliyon.2022.e09938)
Supplement: Multimedia component 2 [file mmc2.pdf]

Cell Viability

(2way ANOVA) Multiple Comparisons.

	**2way ANOVA**<br>Multiple comparisons	**A**<br>Data Set-A<br>**Y**	**B**<br>Data Set-B<br>**Y**	**C**<br>Data Set-C<br>**Y**
**1**	Compare cell means regardless of rows and columns			
**2**				
**3**	Number of families	1		
**4**	Number of comparisons per family	105		
**5**	Alpha	0.05		
**6**				
**7**	Tukey's multiple comparisons test	Mean Diff.	95% CI of diff.	Significant?
**8**				
**9**	Early log:**0 X** vs. Early log:**1 X**	47.44	11.91 to 82.97	Yes
**10**	Early log:**0 X** vs. Early log:**10 X**	49.22	13.69 to 84.75	Yes
**11**	Early log:**0 X** vs. Early log:**40 X**	54.11	18.58 to 89.64	Yes
**12**	Early log:**0 X** vs. Early log:**50 X**	79.33	43.80 to 114.9	Yes
**13**	Early log:**0 X** vs. Mid log:**0 X**	-22.78	-58.31 to 12.75	No
**14**	Early log:**0 X** vs. Mid log:**1 X**	49.44	9.721 to 89.17	Yes
**15**	Early log:**0 X** vs. Mid log:**10 X**	38.28	-1.446 to 78.00	No
**16**	Early log:**0 X** vs. Mid log:**40 X**	57.61	17.89 to 97.33	Yes
**17**	Early log:**0 X** vs. Mid log:**50 X**	64.89	29.36 to 100.4	Yes
**18**	Early log:**0 X** vs. Stationary:**0 X**	41.89	6.359 to 77.42	Yes
**19**	Early log:**0 X** vs. Stationary:**1 X**	48.89	13.36 to 84.42	Yes
**20**	Early log:**0 X** vs. Stationary:**10 X**	54.56	19.03 to 90.09	Yes
**21**	Early log:**0 X** vs. Stationary:**40 X**	59.33	23.80 to 94.86	Yes
**22**	Early log:**0 X** vs. Stationary:**50 X**	60.22	24.69 to 95.75	Yes
**23**	Early log:**1 X** vs. Early log:**10 X**	1.778	-33.75 to 37.31	No
**24**	Early log:**1 X** vs. Early log:**40 X**	6.667	-28.86 to 42.20	No
**25**	Early log:**1 X** vs. Early log:**50 X**	31.89	-3.641 to 67.42	No
**26**	Early log:**1 X** vs. Mid log:**0 X**	-70.22	-105.8 to -34.69	Yes
**27**	Early log:**1 X** vs. Mid log:**1 X**	2.000	-37.72 to 41.72	No
**28**	Early log:**1 X** vs. Mid log:**10 X**	-9.167	-48.89 to 30.56	No
**29**	Early log:**1 X** vs. Mid log:**40 X**	10.17	-29.56 to 49.89	No
**30**	Early log:**1 X** vs. Mid log:**50 X**	17.44	-18.09 to 52.97	No
**31**	Early log:**1 X** vs. Stationary:**0 X**	-5.556	-41.09 to 29.97	No
**32**	Early log:**1 X** vs. Stationary:**1 X**	1.444	-34.09 to 36.97	No
**33**	Early log:**1 X** vs. Stationary:**10 X**	7.111	-28.42 to 42.64	No
**34**	Early log:**1 X** vs. Stationary:**40 X**	11.89	-23.64 to 47.42	No
**35**	Early log:**1 X** vs. Stationary:**50 X**	12.78	-22.75 to 48.31	No
**36**	Early log:**10 X** vs. Early log:**40 X**	4.889	-30.64 to 40.42	No
**37**	Early log:**10 X** vs. Early log:**50 X**	30.11	-5.419 to 65.64	No
**38**	Early log:**10 X** vs. Mid log:**0 X**	-72.00	-107.5 to -36.47	Yes
**39**	Early log:**10 X** vs. Mid log:**1 X**	0.2222	-39.50 to 39.95	No
**40**	Early log:**10 X** vs. Mid log:**10 X**	-10.94	-50.67 to 28.78	No
**41**	Early log:**10 X** vs. Mid log:**40 X**	8.389	-31.33 to 48.11	No
**42**	Early log:**10 X** vs. Mid log:**50 X**	15.67	-19.86 to 51.20	No
**43**	Early log:**10 X** vs. Stationary:**0 X**	-7.333	-42.86 to 28.20	No
**44**	Early log:**10 X** vs. Stationary:**1 X**	-0.3333	-35.86 to 35.20	No
**45**	Early log:**10 X** vs. Stationary:**10 X**	5.333	-30.20 to 40.86	No

	2way ANOVA<br>Multiple comparisons	A<br>Data Set-A<br>Y	B<br>Data Set-B<br>Y	C<br>Data Set-C<br>Y
**46**	Early log:**10 X** vs. Stationary:**40 X**	10.11	-25.42 to 45.64	No
**47**	Early log:**10 X** vs. Stationary:**50 X**	11.00	-24.53 to 46.53	No
**48**	Early log:**40 X** vs. Early log:**50 X**	25.22	-10.31 to 60.75	No
**49**	Early log:**40 X** vs. Mid log:**0 X**	-76.89	-112.4 to -41.36	Yes
**50**	Early log:**40 X** vs. Mid log:**1 X**	-4.667	-44.39 to 35.06	No
**51**	Early log:**40 X** vs. Mid log:**10 X**	-15.83	-55.56 to 23.89	No
**52**	Early log:**40 X** vs. Mid log:**40 X**	3.500	-36.22 to 43.22	No
**53**	Early log:**40 X** vs. Mid log:**50 X**	10.78	-24.75 to 46.31	No
**54**	Early log:**40 X** vs. Stationary:**0 X**	-12.22	-47.75 to 23.31	No
**55**	Early log:**40 X** vs. Stationary:**1 X**	-5.222	-40.75 to 30.31	No
**56**	Early log:**40 X** vs. Stationary:**10 X**	0.4444	-35.09 to 35.97	No
**57**	Early log:**40 X** vs. Stationary:**40 X**	5.222	-30.31 to 40.75	No
**58**	Early log:**40 X** vs. Stationary:**50 X**	6.111	-29.42 to 41.64	No
**59**	Early log:**50 X** vs. Mid log:**0 X**	-102.1	-137.6 to -66.58	Yes
**60**	Early log:**50 X** vs. Mid log:**1 X**	-29.89	-69.61 to 9.835	No
**61**	Early log:**50 X** vs. Mid log:**10 X**	-41.06	-80.78 to -1.332	Yes
**62**	Early log:**50 X** vs. Mid log:**40 X**	-21.72	-61.45 to 18.00	No
**63**	Early log:**50 X** vs. Mid log:**50 X**	-14.44	-49.97 to 21.09	No
**64**	Early log:**50 X** vs. Stationary:**0 X**	-37.44	-72.97 to -1.915	Yes
**65**	Early log:**50 X** vs. Stationary:**1 X**	-30.44	-65.97 to 5.085	No
**66**	Early log:**50 X** vs. Stationary:**10 X**	-24.78	-60.31 to 10.75	No
**67**	Early log:**50 X** vs. Stationary:**40 X**	-20.00	-55.53 to 15.53	No
**68**	Early log:**50 X** vs. Stationary:**50 X**	-19.11	-54.64 to 16.42	No
**69**	Mid log:**0 X** vs. Mid log:**1 X**	72.22	32.50 to 111.9	Yes
**70**	Mid log:**0 X** vs. Mid log:**10 X**	61.06	21.33 to 100.8	Yes
**71**	Mid log:**0 X** vs. Mid log:**40 X**	80.39	40.67 to 120.1	Yes
**72**	Mid log:**0 X** vs. Mid log:**50 X**	87.67	52.14 to 123.2	Yes
**73**	Mid log:**0 X** vs. Stationary:**0 X**	64.67	29.14 to 100.2	Yes
**74**	Mid log:**0 X** vs. Stationary:**1 X**	71.67	36.14 to 107.2	Yes
**75**	Mid log:**0 X** vs. Stationary:**10 X**	77.33	41.80 to 112.9	Yes
**76**	Mid log:**0 X** vs. Stationary:**40 X**	82.11	46.58 to 117.6	Yes
**77**	Mid log:**0 X** vs. Stationary:**50 X**	83.00	47.47 to 118.5	Yes
**78**	Mid log:**1 X** vs. Mid log:**10 X**	-11.17	-54.68 to 32.35	No
**79**	Mid log:**1 X** vs. Mid log:**40 X**	8.167	-35.35 to 51.68	No
**80**	Mid log:**1 X** vs. Mid log:**50 X**	15.44	-24.28 to 55.17	No
**81**	Mid log:**1 X** vs. Stationary:**0 X**	-7.556	-47.28 to 32.17	No
**82**	Mid log:**1 X** vs. Stationary:**1 X**	-0.5556	-40.28 to 39.17	No
**83**	Mid log:**1 X** vs. Stationary:**10 X**	5.111	-34.61 to 44.83	No
**84**	Mid log:**1 X** vs. Stationary:**40 X**	9.889	-29.83 to 49.61	No
**85**	Mid log:**1 X** vs. Stationary:**50 X**	10.78	-28.95 to 50.50	No
**86**	Mid log:**10 X** vs. Mid log:**40 X**	19.33	-24.18 to 62.85	No
**87**	Mid log:**10 X** vs. Mid log:**50 X**	26.61	-13.11 to 66.33	No
**88**	Mid log:**10 X** vs. Stationary:**0 X**	3.611	-36.11 to 43.33	No
**89**	Mid log:**10 X** vs. Stationary:**1 X**	10.61	-29.11 to 50.33	No
**90**	Mid log:**10 X** vs. Stationary:**10 X**	16.28	-23.45 to 56.00	No

	**2way ANOVA** Multiple comparisons	**A** Data Set-A **Y**	**B** Data Set-B **Y**	**C** Data Set-C **Y**
**91**	Mid log:**10 X** vs. Stationary:**40 X**	21.06	-18.67 to 60.78	No
**92**	Mid log:**10 X** vs. Stationary:**50 X**	21.94	-17.78 to 61.67	No
**93**	Mid log:**40 X** vs. Mid log:**50 X**	7.278	-32.45 to 47.00	No
**94**	Mid log:**40 X** vs. Stationary:**0 X**	-15.72	-55.45 to 24.00	No
**95**	Mid log:**40 X** vs. Stationary:**1 X**	-8.722	-48.45 to 31.00	No
**96**	Mid log:**40 X** vs. Stationary:**10 X**	-3.056	-42.78 to 36.67	No
**97**	Mid log:**40 X** vs. Stationary:**40 X**	1.722	-38.00 to 41.45	No
**98**	Mid log:**40 X** vs. Stationary:**50 X**	2.611	-37.11 to 42.33	No
**99**	Mid log:**50 X** vs. Stationary:**0 X**	-23.00	-58.53 to 12.53	No
**100**	Mid log:**50 X** vs. Stationary:**1 X**	-16.00	-51.53 to 19.53	No
**101**	Mid log:**50 X** vs. Stationary:**10 X**	-10.33	-45.86 to 25.20	No
**102**	Mid log:**50 X** vs. Stationary:**40 X**	-5.556	-41.09 to 29.97	No
**103**	Mid log:**50 X** vs. Stationary:**50 X**	-4.667	-40.20 to 30.86	No
**104**	Stationary:**0 X** vs. Stationary:**1 X**	7.000	-28.53 to 42.53	No
**105**	Stationary:**0 X** vs. Stationary:**10 X**	12.67	-22.86 to 48.20	No
**106**	Stationary:**0 X** vs. Stationary:**40 X**	17.44	-18.09 to 52.97	No
**107**	Stationary:**0 X** vs. Stationary:**50 X**	18.33	-17.20 to 53.86	No
**108**	Stationary:**1 X** vs. Stationary:**10 X**	5.667	-29.86 to 41.20	No
**109**	Stationary:**1 X** vs. Stationary:**40 X**	10.44	-25.09 to 45.97	No
**110**	Stationary:**1 X** vs. Stationary:**50 X**	11.33	-24.20 to 46.86	No
**111**	Stationary:**10 X** vs. Stationary:**40 X**	4.778	-30.75 to 40.31	No
**112**	Stationary:**10 X** vs. Stationary:**50 X**	5.667	-29.86 to 41.20	No
**113**	Stationary:**40 X** vs. Stationary:**50 X**	0.8889	-34.64 to 36.42	No
**114**				
**115**				
**116**	Test details	Mean 1	Mean 2	Mean Diff.
**117**				
**118**	Early log:**0 X** vs. Early log:**1 X**	122.8	75.33	47.44
**119**	Early log:**0 X** vs. Early log:**10 X**	122.8	73.56	49.22
**120**	Early log:**0 X** vs. Early log:**40 X**	122.8	68.67	54.11
**121**	Early log:**0 X** vs. Early log:**50 X**	122.8	43.44	79.33
**122**	Early log:**0 X** vs. Mid log:**0 X**	122.8	145.6	-22.78
**123**	Early log:**0 X** vs. Mid log:**1 X**	122.8	73.33	49.44
**124**	Early log:**0 X** vs. Mid log:**10 X**	122.8	84.50	38.28
**125**	Early log:**0 X** vs. Mid log:**40 X**	122.8	65.17	57.61
**126**	Early log:**0 X** vs. Mid log:**50 X**	122.8	57.89	64.89
**127**	Early log:**0 X** vs. Stationary:**0 X**	122.8	80.89	41.89
**128**	Early log:**0 X** vs. Stationary:**1 X**	122.8	73.89	48.89
**129**	Early log:**0 X** vs. Stationary:**10 X**	122.8	68.22	54.56
**130**	Early log:**0 X** vs. Stationary:**40 X**	122.8	63.44	59.33
**131**	Early log:**0 X** vs. Stationary:**50 X**	122.8	62.56	60.22
**132**	Early log:**1 X** vs. Early log:**10 X**	75.33	73.56	1.778
**133**	Early log:**1 X** vs. Early log:**40 X**	75.33	68.67	6.667
**134**	Early log:**1 X** vs. Early log:**50 X**	75.33	43.44	31.89
**135**	Early log:**1 X** vs. Mid log:**0 X**	75.33	145.6	-70.22

	**2way ANOVA** Multiple comparisons	**A** Data Set-A **Y**	**B** Data Set-B **Y**	**C** Data Set-C **Y**
**136**	Early log:**1 X** vs. Mid log:**1 X**	75.33	73.33	2.000
**137**	Early log:**1 X** vs. Mid log:**10 X**	75.33	84.50	-9.167
**138**	Early log:**1 X** vs. Mid log:**40 X**	75.33	65.17	10.17
**139**	Early log:**1 X** vs. Mid log:**50 X**	75.33	57.89	17.44
**140**	Early log:**1 X** vs. Stationary:**0 X**	75.33	80.89	-5.556
**141**	Early log:**1 X** vs. Stationary:**1 X**	75.33	73.89	1.444
**142**	Early log:**1 X** vs. Stationary:**10 X**	75.33	68.22	7.111
**143**	Early log:**1 X** vs. Stationary:**40 X**	75.33	63.44	11.89
**144**	Early log:**1 X** vs. Stationary:**50 X**	75.33	62.56	12.78
**145**	Early log:**10 X** vs. Early log:**40 X**	73.56	68.67	4.889
**146**	Early log:**10 X** vs. Early log:**50 X**	73.56	43.44	30.11
**147**	Early log:**10 X** vs. Mid log:**0 X**	73.56	145.6	-72.00
**148**	Early log:**10 X** vs. Mid log:**1 X**	73.56	73.33	0.2222
**149**	Early log:**10 X** vs. Mid log:**10 X**	73.56	84.50	-10.94
**150**	Early log:**10 X** vs. Mid log:**40 X**	73.56	65.17	8.389
**151**	Early log:**10 X** vs. Mid log:**50 X**	73.56	57.89	15.67
**152**	Early log:**10 X** vs. Stationary:**0 X**	73.56	80.89	-7.333
**153**	Early log:**10 X** vs. Stationary:**1 X**	73.56	73.89	-0.3333
**154**	Early log:**10 X** vs. Stationary:**10 X**	73.56	68.22	5.333
**155**	Early log:**10 X** vs. Stationary:**40 X**	73.56	63.44	10.11
**156**	Early log:**10 X** vs. Stationary:**50 X**	73.56	62.56	11.00
**157**	Early log:**40 X** vs. Early log:**50 X**	68.67	43.44	25.22
**158**	Early log:**40 X** vs. Mid log:**0 X**	68.67	145.6	-76.89
**159**	Early log:**40 X** vs. Mid log:**1 X**	68.67	73.33	-4.667
**160**	Early log:**40 X** vs. Mid log:**10 X**	68.67	84.50	-15.83
**161**	Early log:**40 X** vs. Mid log:**40 X**	68.67	65.17	3.500
**162**	Early log:**40 X** vs. Mid log:**50 X**	68.67	57.89	10.78
**163**	Early log:**40 X** vs. Stationary:**0 X**	68.67	80.89	-12.22
**164**	Early log:**40 X** vs. Stationary:**1 X**	68.67	73.89	-5.222
**165**	Early log:**40 X** vs. Stationary:**10 X**	68.67	68.22	0.4444
**166**	Early log:**40 X** vs. Stationary:**40 X**	68.67	63.44	5.222
**167**	Early log:**40 X** vs. Stationary:**50 X**	68.67	62.56	6.111
**168**	Early log:**50 X** vs. Mid log:**0 X**	43.44	145.6	-102.1
**169**	Early log:**50 X** vs. Mid log:**1 X**	43.44	73.33	-29.89
**170**	Early log:**50 X** vs. Mid log:**10 X**	43.44	84.50	-41.06
**171**	Early log:**50 X** vs. Mid log:**40 X**	43.44	65.17	-21.72
**172**	Early log:**50 X** vs. Mid log:**50 X**	43.44	57.89	-14.44
**173**	Early log:**50 X** vs. Stationary:**0 X**	43.44	80.89	-37.44
**174**	Early log:**50 X** vs. Stationary:**1 X**	43.44	73.89	-30.44
**175**	Early log:**50 X** vs. Stationary:**10 X**	43.44	68.22	-24.78
**176**	Early log:**50 X** vs. Stationary:**40 X**	43.44	63.44	-20.00
**177**	Early log:**50 X** vs. Stationary:**50 X**	43.44	62.56	-19.11
**178**	Mid log:**0 X** vs. Mid log:**1 X**	145.6	73.33	72.22
**179**	Mid log:**0 X** vs. Mid log:**10 X**	145.6	84.50	61.06
**180**	Mid log:**0 X** vs. Mid log:**40 X**	145.6	65.17	80.39

	2way ANOVA<br>Multiple comparisons	A<br>Data Set-A<br>Y	B<br>Data Set-B<br>Y	C<br>Data Set-C<br>Y
**181**	Mid log:**0 X** vs. Mid log:**50 X**	145.6	57.89	87.67
**182**	Mid log:**0 X** vs. Stationary:**0 X**	145.6	80.89	64.67
**183**	Mid log:**0 X** vs. Stationary:**1 X**	145.6	73.89	71.67
**184**	Mid log:**0 X** vs. Stationary:**10 X**	145.6	68.22	77.33
**185**	Mid log:**0 X** vs. Stationary:**40 X**	145.6	63.44	82.11
**186**	Mid log:**0 X** vs. Stationary:**50 X**	145.6	62.56	83.00
**187**	Mid log:**1 X** vs. Mid log:**10 X**	73.33	84.50	-11.17
**188**	Mid log:**1 X** vs. Mid log:**40 X**	73.33	65.17	8.167
**189**	Mid log:**1 X** vs. Mid log:**50 X**	73.33	57.89	15.44
**190**	Mid log:**1 X** vs. Stationary:**0 X**	73.33	80.89	-7.556
**191**	Mid log:**1 X** vs. Stationary:**1 X**	73.33	73.89	-0.5556
**192**	Mid log:**1 X** vs. Stationary:**10 X**	73.33	68.22	5.111
**193**	Mid log:**1 X** vs. Stationary:**40 X**	73.33	63.44	9.889
**194**	Mid log:**1 X** vs. Stationary:**50 X**	73.33	62.56	10.78
**195**	Mid log:**10 X** vs. Mid log:**40 X**	84.50	65.17	19.33
**196**	Mid log:**10 X** vs. Mid log:**50 X**	84.50	57.89	26.61
**197**	Mid log:**10 X** vs. Stationary:**0 X**	84.50	80.89	3.611
**198**	Mid log:**10 X** vs. Stationary:**1 X**	84.50	73.89	10.61
**199**	Mid log:**10 X** vs. Stationary:**10 X**	84.50	68.22	16.28
**200**	Mid log:**10 X** vs. Stationary:**40 X**	84.50	63.44	21.06
**201**	Mid log:**10 X** vs. Stationary:**50 X**	84.50	62.56	21.94
**202**	Mid log:**40 X** vs. Mid log:**50 X**	65.17	57.89	7.278
**203**	Mid log:**40 X** vs. Stationary:**0 X**	65.17	80.89	-15.72
**204**	Mid log:**40 X** vs. Stationary:**1 X**	65.17	73.89	-8.722
**205**	Mid log:**40 X** vs. Stationary:**10 X**	65.17	68.22	-3.056
**206**	Mid log:**40 X** vs. Stationary:**40 X**	65.17	63.44	1.722
**207**	Mid log:**40 X** vs. Stationary:**50 X**	65.17	62.56	2.611
**208**	Mid log:**50 X** vs. Stationary:**0 X**	57.89	80.89	-23.00
**209**	Mid log:**50 X** vs. Stationary:**1 X**	57.89	73.89	-16.00
**210**	Mid log:**50 X** vs. Stationary:**10 X**	57.89	68.22	-10.33
**211**	Mid log:**50 X** vs. Stationary:**40 X**	57.89	63.44	-5.556
**212**	Mid log:**50 X** vs. Stationary:**50 X**	57.89	62.56	-4.667
**213**	Stationary:**0 X** vs. Stationary:**1 X**	80.89	73.89	7.000
**214**	Stationary:**0 X** vs. Stationary:**10 X**	80.89	68.22	12.67
**215**	Stationary:**0 X** vs. Stationary:**40 X**	80.89	63.44	17.44
**216**	Stationary:**0 X** vs. Stationary:**50 X**	80.89	62.56	18.33
**217**	Stationary:**1 X** vs. Stationary:**10 X**	73.89	68.22	5.667
**218**	Stationary:**1 X** vs. Stationary:**40 X**	73.89	63.44	10.44
**219**	Stationary:**1 X** vs. Stationary:**50 X**	73.89	62.56	11.33
**220**	Stationary:**10 X** vs. Stationary:**40 X**	68.22	63.44	4.778
**221**	Stationary:**10 X** vs. Stationary:**50 X**	68.22	62.56	5.667
**222**	Stationary:**40 X** vs. Stationary:**50 X**	63.44	62.56	0.8889

Quantification of hydrogen peroxide
(2way ANOVA) Multiple Comparisons.

	**2way ANOVA**<br>Multiple comparisons	**A**<br>Data Set-A<br>**Y**	**B**<br>Data Set-B<br>**Y**	**C**<br>Data Set-C<br>**Y**	**D**<br>Data Set-D<br>**Y**
**1**	Compare cell means regardless of rows and columns				
**2**					
**3**	Number of families	1			
**4**	Number of comparisons per family	105			
**5**	Alpha	0.05			
**6**					
**7**	Tukey's multiple comparisons test	Mean Diff.	95% CI of diff.	Significant?	Summary
**8**					
**9**	Earl log:**0 X** vs. Earl log:**1 X**	-892.8	-1779 to -6.842	Yes	*
**10**	Earl log:**0 X** vs. Earl log:**10 X**	-524.1	-1410 to 361.8	No	ns
**11**	Earl log:**0 X** vs. Earl log:**40 X**	-1009	-1895 to -123.1	Yes	*
**12**	Earl log:**0 X** vs. Earl log:**50 X**	-1423	-2309 to -537.1	Yes	****
**13**	Earl log:**0 X** vs. Mid log:**0 X**	-737.6	-1623 to 148.4	No	ns
**14**	Earl log:**0 X** vs. Mid log:**1 X**	-1359	-2306 to -412.0	Yes	***
**15**	Earl log:**0 X** vs. Mid log:**10 X**	-873.3	-1759 to 12.60	No	ns
**16**	Earl log:**0 X** vs. Mid log:**40 X**	-297.6	-1183 to 588.4	No	ns
**17**	Earl log:**0 X** vs. Mid log:**50 X**	-1637	-2627 to -646.2	Yes	****
**18**	Earl log:**0 X** vs. Late log:**0 X**	-693.8	-1641 to 253.3	No	ns
**19**	Earl log:**0 X** vs. Late log:**1 X**	-750.4	-1636 to 135.5	No	ns
**20**	Earl log:**0 X** vs. Late log:**10 X**	-591.2	-1504 to 322.0	No	ns
**21**	Earl log:**0 X** vs. Late log:**40 X**	-912.2	-1798 to -26.29	Yes	*
**22**	Earl log:**0 X** vs. Late log:**50 X**	-899.0	-1785 to -13.06	Yes	*
**23**	Earl log:**1 X** vs. Earl log:**10 X**	368.7	-517.3 to 1255	No	ns
**24**	Earl log:**1 X** vs. Earl log:**40 X**	-116.2	-1002 to 769.7	No	ns
**25**	Earl log:**1 X** vs. Earl log:**50 X**	-530.2	-1416 to 355.7	No	ns
**26**	Earl log:**1 X** vs. Mid log:**0 X**	155.2	-730.7 to 1041	No	ns
**27**	Earl log:**1 X** vs. Mid log:**1 X**	-466.3	-1413 to 480.8	No	ns
**28**	Earl log:**1 X** vs. Mid log:**10 X**	19.44	-866.5 to 905.4	No	ns
**29**	Earl log:**1 X** vs. Mid log:**40 X**	595.2	-290.7 to 1481	No	ns
**30**	Earl log:**1 X** vs. Mid log:**50 X**	-743.9	-1734 to 246.6	No	ns
**31**	Earl log:**1 X** vs. Late log:**0 X**	199.0	-748.1 to 1146	No	ns
**32**	Earl log:**1 X** vs. Late log:**1 X**	142.3	-743.6 to 1028	No	ns
**33**	Earl log:**1 X** vs. Late log:**10 X**	301.6	-611.6 to 1215	No	ns
**34**	Earl log:**1 X** vs. Late log:**40 X**	-19.44	-905.4 to 866.5	No	ns
**35**	Earl log:**1 X** vs. Late log:**50 X**	-6.222	-892.2 to 879.7	No	ns
**36**	Earl log:**10 X** vs. Earl log:**40 X**	-484.9	-1371 to 401.0	No	ns
**37**	Earl log:**10 X** vs. Earl log:**50 X**	-898.9	-1785 to -12.95	Yes	*
**38**	Earl log:**10 X** vs. Mid log:**0 X**	-213.4	-1099 to 672.5	No	ns
**39**	Earl log:**10 X** vs. Mid log:**1 X**	-835.0	-1782 to 112.1	No	ns
**40**	Earl log:**10 X** vs. Mid log:**10 X**	-349.2	-1235 to 536.7	No	ns
**41**	Earl log:**10 X** vs. Mid log:**40 X**	226.6	-659.4 to 1112	No	ns
**42**	Earl log:**10 X** vs. Mid log:**50 X**	-1113	-2103 to -122.0	Yes	*
**43**	Earl log:**10 X** vs. Late log:**0 X**	-169.7	-1117 to 777.4	No	ns
**44**	Earl log:**10 X** vs. Late log:**1 X**	-226.3	-1112 to 659.6	No	ns
**45**	Earl log:**10 X** vs. Late log:**10 X**	-67.06	-980.3 to 846.1	No	ns
**46**	Earl log:**10 X** vs. Late log:**40 X**	-388.1	-1274 to 497.8	No	ns
**47**	Earl log:**10 X** vs. Late log:**50 X**	-374.9	-1261 to 511.0	No	ns
**48**	Earl log:**40 X** vs. Earl log:**50 X**	-414.0	-1300 to 471.9	No	ns
**49**	Earl log:**40 X** vs. Mid log:**0 X**	271.4	-614.5 to 1157	No	ns
**50**	Earl log:**40 X** vs. Mid log:**1 X**	-350.1	-1297 to 597.0	No	ns

	2way ANOVA<br>Multiple comparisons	A<br>Data Set-A<br>Y	B<br>Data Set-B<br>Y	C<br>Data Set-C<br>Y	D<br>Data Set-D<br>Y
**51**	Earl log:**40 X** vs. Mid log:**10 X**	135.7	-750.3 to 1022	No	ns
**52**	Earl log:**40 X** vs. Mid log:**40 X**	711.4	-174.5 to 1597	No	ns
**53**	Earl log:**40 X** vs. Mid log:**50 X**	-627.7	-1618 to 362.8	No	ns
**54**	Earl log:**40 X** vs. Late log:**0 X**	315.2	-631.9 to 1262	No	ns
**55**	Earl log:**40 X** vs. Late log:**1 X**	258.6	-627.4 to 1144	No	ns
**56**	Earl log:**40 X** vs. Late log:**10 X**	417.8	-495.4 to 1331	No	ns
**57**	Earl log:**40 X** vs. Late log:**40 X**	96.78	-789.2 to 982.7	No	ns
**58**	Earl log:**40 X** vs. Late log:**50 X**	110.0	-775.9 to 995.9	No	ns
**59**	Earl log:**50 X** vs. Mid log:**0 X**	685.4	-200.5 to 1571	No	ns
**60**	Earl log:**50 X** vs. Mid log:**1 X**	63.90	-883.2 to 1011	No	ns
**61**	Earl log:**50 X** vs. Mid log:**10 X**	549.7	-336.3 to 1436	No	ns
**62**	Earl log:**50 X** vs. Mid log:**40 X**	1125	239.5 to 2011	Yes	**
**63**	Earl log:**50 X** vs. Mid log:**50 X**	-213.7	-1204 to 776.8	No	ns
**64**	Earl log:**50 X** vs. Late log:**0 X**	729.2	-217.9 to 1676	No	ns
**65**	Earl log:**50 X** vs. Late log:**1 X**	672.6	-213.4 to 1558	No	ns
**66**	Earl log:**50 X** vs. Late log:**10 X**	831.8	-81.37 to 1745	No	ns
**67**	Earl log:**50 X** vs. Late log:**40 X**	510.8	-375.2 to 1397	No	ns
**68**	Earl log:**50 X** vs. Late log:**50 X**	524.0	-361.9 to 1410	No	ns
**69**	Mid log:**0 X** vs. Mid log:**1 X**	-621.5	-1569 to 325.6	No	ns
**70**	Mid log:**0 X** vs. Mid log:**10 X**	-135.8	-1022 to 750.2	No	ns
**71**	Mid log:**0 X** vs. Mid log:**40 X**	440.0	-445.9 to 1326	No	ns
**72**	Mid log:**0 X** vs. Mid log:**50 X**	-899.1	-1890 to 91.40	No	ns
**73**	Mid log:**0 X** vs. Late log:**0 X**	43.75	-903.4 to 990.9	No	ns
**74**	Mid log:**0 X** vs. Late log:**1 X**	-12.89	-898.8 to 873.0	No	ns
**75**	Mid log:**0 X** vs. Late log:**10 X**	146.4	-766.8 to 1060	No	ns
**76**	Mid log:**0 X** vs. Late log:**40 X**	-174.7	-1061 to 711.3	No	ns
**77**	Mid log:**0 X** vs. Late log:**50 X**	-161.4	-1047 to 724.5	No	ns
**78**	Mid log:**1 X** vs. Mid log:**10 X**	485.8	-461.3 to 1433	No	ns
**79**	Mid log:**1 X** vs. Mid log:**40 X**	1062	114.4 to 2009	Yes	*
**80**	Mid log:**1 X** vs. Mid log:**50 X**	-277.6	-1323 to 768.0	No	ns
**81**	Mid log:**1 X** vs. Late log:**0 X**	665.3	-339.3 to 1670	No	ns
**82**	Mid log:**1 X** vs. Late log:**1 X**	608.7	-338.5 to 1556	No	ns
**83**	Mid log:**1 X** vs. Late log:**10 X**	767.9	-204.7 to 1741	No	ns
**84**	Mid log:**1 X** vs. Late log:**40 X**	446.9	-500.2 to 1394	No	ns
**85**	Mid log:**1 X** vs. Late log:**50 X**	460.1	-487.0 to 1407	No	ns
**86**	Mid log:**10 X** vs. Mid log:**40 X**	575.8	-310.2 to 1462	No	ns
**87**	Mid log:**10 X** vs. Mid log:**50 X**	-763.3	-1754 to 227.2	No	ns
**88**	Mid log:**10 X** vs. Late log:**0 X**	179.5	-767.6 to 1127	No	ns
**89**	Mid log:**10 X** vs. Late log:**1 X**	122.9	-763.0 to 1009	No	ns
**90**	Mid log:**10 X** vs. Late log:**10 X**	282.2	-631.0 to 1195	No	ns
**91**	Mid log:**10 X** vs. Late log:**40 X**	-38.89	-924.8 to 847.0	No	ns
**92**	Mid log:**10 X** vs. Late log:**50 X**	-25.67	-911.6 to 860.3	No	ns
**93**	Mid log:**40 X** vs. Mid log:**50 X**	-1339	-2330 to -348.6	Yes	***
**94**	Mid log:**40 X** vs. Late log:**0 X**	-396.3	-1343 to 550.9	No	ns
**95**	Mid log:**40 X** vs. Late log:**1 X**	-452.9	-1339 to 433.0	No	ns
**96**	Mid log:**40 X** vs. Late log:**10 X**	-293.6	-1207 to 619.6	No	ns
**97**	Mid log:**40 X** vs. Late log:**40 X**	-614.7	-1501 to 271.3	No	ns
**98**	Mid log:**40 X** vs. Late log:**50 X**	-601.4	-1487 to 284.5	No	ns
**99**	Mid log:**50 X** vs. Late log:**0 X**	942.9	-102.7 to 1988	No	ns
**100**	Mid log:**50 X** vs. Late log:**1 X**	886.2	-104.3 to 1877	No	ns

	2way ANOVA<br>Multiple comparisons	A<br>Data Set-A<br>Y	B<br>Data Set-B<br>Y	C<br>Data Set-C<br>Y	D<br>Data Set-D<br>Y
101	Mid log:50 X vs. Late log:10 X	1046	30.53 to 2060	Yes	*
102	Mid log:50 X vs. Late log:40 X	724.4	-266.1 to 1715	No	ns
103	Mid log:50 X vs. Late log:50 X	737.7	-252.8 to 1728	No	ns
104	Late log:0 X vs. Late log:1 X	-56.63	-1004 to 890.5	No	ns
105	Late log:0 X vs. Late log:10 X	102.6	-870.0 to 1075	No	ns
106	Late log:0 X vs. Late log:40 X	-218.4	-1166 to 728.7	No	ns
107	Late log:0 X vs. Late log:50 X	-205.2	-1152 to 741.9	No	ns
108	Late log:1 X vs. Late log:10 X	159.3	-753.9 to 1072	No	ns
109	Late log:1 X vs. Late log:40 X	-161.8	-1048 to 724.2	No	ns
110	Late log:1 X vs. Late log:50 X	-148.6	-1034 to 737.4	No	ns
111	Late log:10 X vs. Late log:40 X	-321.1	-1234 to 592.1	No	ns
112	Late log:10 X vs. Late log:50 X	-307.8	-1221 to 605.4	No	ns
113	Late log:40 X vs. Late log:50 X	13.22	-872.7 to 899.2	No	ns
114					
115					
116	Test details	Mean 1	Mean 2	Mean Diff.	SE of diff.
117					
118	Earl log:0 X vs. Earl log:1 X	2283	3176	-892.8	255.4
119	Earl log:0 X vs. Earl log:10 X	2283	2807	-524.1	255.4
120	Earl log:0 X vs. Earl log:40 X	2283	3292	-1009	255.4
121	Earl log:0 X vs. Earl log:50 X	2283	3706	-1423	255.4
122	Earl log:0 X vs. Mid log:0 X	2283	3021	-737.6	255.4
123	Earl log:0 X vs. Mid log:1 X	2283	3642	-1359	273.1
124	Earl log:0 X vs. Mid log:10 X	2283	3157	-873.3	255.4
125	Earl log:0 X vs. Mid log:40 X	2283	2581	-297.6	255.4
126	Earl log:0 X vs. Mid log:50 X	2283	3920	-1637	285.6
127	Earl log:0 X vs. Late log:0 X	2283	2977	-693.8	273.1
128	Earl log:0 X vs. Late log:1 X	2283	3034	-750.4	255.4
129	Earl log:0 X vs. Late log:10 X	2283	2875	-591.2	263.3
130	Earl log:0 X vs. Late log:40 X	2283	3196	-912.2	255.4
131	Earl log:0 X vs. Late log:50 X	2283	3182	-899.0	255.4
132	Earl log:1 X vs. Earl log:10 X	3176	2807	368.7	255.4
133	Earl log:1 X vs. Earl log:40 X	3176	3292	-116.2	255.4
134	Earl log:1 X vs. Earl log:50 X	3176	3706	-530.2	255.4
135	Earl log:1 X vs. Mid log:0 X	3176	3021	155.2	255.4
136	Earl log:1 X vs. Mid log:1 X	3176	3642	-466.3	273.1
137	Earl log:1 X vs. Mid log:10 X	3176	3157	19.44	255.4
138	Earl log:1 X vs. Mid log:40 X	3176	2581	595.2	255.4
139	Earl log:1 X vs. Mid log:50 X	3176	3920	-743.9	285.6
140	Earl log:1 X vs. Late log:0 X	3176	2977	199.0	273.1
141	Earl log:1 X vs. Late log:1 X	3176	3034	142.3	255.4
142	Earl log:1 X vs. Late log:10 X	3176	2875	301.6	263.3
143	Earl log:1 X vs. Late log:40 X	3176	3196	-19.44	255.4
144	Earl log:1 X vs. Late log:50 X	3176	3182	-6.222	255.4
145	Earl log:10 X vs. Earl log:40 X	2807	3292	-484.9	255.4
146	Earl log:10 X vs. Earl log:50 X	2807	3706	-898.9	255.4
147	Earl log:10 X vs. Mid log:0 X	2807	3021	-213.4	255.4
148	Earl log:10 X vs. Mid log:1 X	2807	3642	-835.0	273.1
149	Earl log:10 X vs. Mid log:10 X	2807	3157	-349.2	255.4
150	Earl log:10 X vs. Mid log:40 X	2807	2581	226.6	255.4

	2way ANOVA<br>Multiple comparisons	A<br>Data Set-A<br>Y	B<br>Data Set-B<br>Y	C<br>Data Set-C<br>Y	D<br>Data Set-D<br>Y
**151**	Earl log:**10 X** vs. Mid log:**50 X**	2807	3920	-1113	285.6
**152**	Earl log:**10 X** vs. Late log:**0 X**	2807	2977	-169.7	273.1
**153**	Earl log:**10 X** vs. Late log:**1 X**	2807	3034	-226.3	255.4
**154**	Earl log:**10 X** vs. Late log:**10 X**	2807	2875	-67.06	263.3
**155**	Earl log:**10 X** vs. Late log:**40 X**	2807	3196	-388.1	255.4
**156**	Earl log:**10 X** vs. Late log:**50 X**	2807	3182	-374.9	255.4
**157**	Earl log:**40 X** vs. Earl log:**50 X**	3292	3706	-414.0	255.4
**158**	Earl log:**40 X** vs. Mid log:**0 X**	3292	3021	271.4	255.4
**159**	Earl log:**40 X** vs. Mid log:**1 X**	3292	3642	-350.1	273.1
**160**	Earl log:**40 X** vs. Mid log:**10 X**	3292	3157	135.7	255.4
**161**	Earl log:**40 X** vs. Mid log:**40 X**	3292	2581	711.4	255.4
**162**	Earl log:**40 X** vs. Mid log:**50 X**	3292	3920	-627.7	285.6
**163**	Earl log:**40 X** vs. Late log:**0 X**	3292	2977	315.2	273.1
**164**	Earl log:**40 X** vs. Late log:**1 X**	3292	3034	258.6	255.4
**165**	Earl log:**40 X** vs. Late log:**10 X**	3292	2875	417.8	263.3
**166**	Earl log:**40 X** vs. Late log:**40 X**	3292	3196	96.78	255.4
**167**	Earl log:**40 X** vs. Late log:**50 X**	3292	3182	110.0	255.4
**168**	Earl log:**50 X** vs. Mid log:**0 X**	3706	3021	685.4	255.4
**169**	Earl log:**50 X** vs. Mid log:**1 X**	3706	3642	63.90	273.1
**170**	Earl log:**50 X** vs. Mid log:**10 X**	3706	3157	549.7	255.4
**171**	Earl log:**50 X** vs. Mid log:**40 X**	3706	2581	1125	255.4
**172**	Earl log:**50 X** vs. Mid log:**50 X**	3706	3920	-213.7	285.6
**173**	Earl log:**50 X** vs. Late log:**0 X**	3706	2977	729.2	273.1
**174**	Earl log:**50 X** vs. Late log:**1 X**	3706	3034	672.6	255.4
**175**	Earl log:**50 X** vs. Late log:**10 X**	3706	2875	831.8	263.3
**176**	Earl log:**50 X** vs. Late log:**40 X**	3706	3196	510.8	255.4
**177**	Earl log:**50 X** vs. Late log:**50 X**	3706	3182	524.0	255.4
**178**	Mid log:**0 X** vs. Mid log:**1 X**	3021	3642	-621.5	273.1
**179**	Mid log:**0 X** vs. Mid log:**10 X**	3021	3157	-135.8	255.4
**180**	Mid log:**0 X** vs. Mid log:**40 X**	3021	2581	440.0	255.4
**181**	Mid log:**0 X** vs. Mid log:**50 X**	3021	3920	-899.1	285.6
**182**	Mid log:**0 X** vs. Late log:**0 X**	3021	2977	43.75	273.1
**183**	Mid log:**0 X** vs. Late log:**1 X**	3021	3034	-12.89	255.4
**184**	Mid log:**0 X** vs. Late log:**10 X**	3021	2875	146.4	263.3
**185**	Mid log:**0 X** vs. Late log:**40 X**	3021	3196	-174.7	255.4
**186**	Mid log:**0 X** vs. Late log:**50 X**	3021	3182	-161.4	255.4
**187**	Mid log:**1 X** vs. Mid log:**10 X**	3642	3157	485.8	273.1
**188**	Mid log:**1 X** vs. Mid log:**40 X**	3642	2581	1062	273.1
**189**	Mid log:**1 X** vs. Mid log:**50 X**	3642	3920	-277.6	301.4
**190**	Mid log:**1 X** vs. Late log:**0 X**	3642	2977	665.3	289.6
**191**	Mid log:**1 X** vs. Late log:**1 X**	3642	3034	608.7	273.1
**192**	Mid log:**1 X** vs. Late log:**10 X**	3642	2875	767.9	280.4
**193**	Mid log:**1 X** vs. Late log:**40 X**	3642	3196	446.9	273.1
**194**	Mid log:**1 X** vs. Late log:**50 X**	3642	3182	460.1	273.1
**195**	Mid log:**10 X** vs. Mid log:**40 X**	3157	2581	575.8	255.4
**196**	Mid log:**10 X** vs. Mid log:**50 X**	3157	3920	-763.3	285.6
**197**	Mid log:**10 X** vs. Late log:**0 X**	3157	2977	179.5	273.1
**198**	Mid log:**10 X** vs. Late log:**1 X**	3157	3034	122.9	255.4
**199**	Mid log:**10 X** vs. Late log:**10 X**	3157	2875	282.2	263.3
**200**	Mid log:**10 X** vs. Late log:**40 X**	3157	3196	-38.89	255.4

2way ANOVA<br>Multiple comparisons		A<br>Data Set-A<br>Y	B<br>Data Set-B<br>Y	C<br>Data Set-C<br>Y	D<br>Data Set-D<br>Y
**201**	Mid log:**10 X** vs. Late log:**50 X**	3157	3182	-25.67	255.4
**202**	Mid log:**40 X** vs. Mid log:**50 X**	2581	3920	-1339	285.6
**203**	Mid log:**40 X** vs. Late log:**0 X**	2581	2977	-396.3	273.1
**204**	Mid log:**40 X** vs. Late log:**1 X**	2581	3034	-452.9	255.4
**205**	Mid log:**40 X** vs. Late log:**10 X**	2581	2875	-293.6	263.3
**206**	Mid log:**40 X** vs. Late log:**40 X**	2581	3196	-614.7	255.4
**207**	Mid log:**40 X** vs. Late log:**50 X**	2581	3182	-601.4	255.4
**208**	Mid log:**50 X** vs. Late log:**0 X**	3920	2977	942.9	301.4
**209**	Mid log:**50 X** vs. Late log:**1 X**	3920	3034	886.2	285.6
**210**	Mid log:**50 X** vs. Late log:**10 X**	3920	2875	1046	292.6
**211**	Mid log:**50 X** vs. Late log:**40 X**	3920	3196	724.4	285.6
**212**	Mid log:**50 X** vs. Late log:**50 X**	3920	3182	737.7	285.6
**213**	Late log:**0 X** vs. Late log:**1 X**	2977	3034	-56.63	273.1
**214**	Late log:**0 X** vs. Late log:**10 X**	2977	2875	102.6	280.4
**215**	Late log:**0 X** vs. Late log:**40 X**	2977	3196	-218.4	273.1
**216**	Late log:**0 X** vs. Late log:**50 X**	2977	3182	-205.2	273.1
**217**	Late log:**1 X** vs. Late log:**10 X**	3034	2875	159.3	263.3
**218**	Late log:**1 X** vs. Late log:**40 X**	3034	3196	-161.8	255.4
**219**	Late log:**1 X** vs. Late log:**50 X**	3034	3182	-148.6	255.4
**220**	Late log:**10 X** vs. Late log:**40 X**	2875	3196	-321.1	263.3
**221**	Late log:**10 X** vs. Late log:**50 X**	2875	3182	-307.8	263.3
**222**	Late log:**40 X** vs. Late log:**50 X**	3196	3182	13.22	255.4

Quantification of Malondialdehyde (MDA)

(2way ANOVA) Multiple Comparisons.

	**2way ANOVA**<br>Multiple comparisons	**A**<br>Data Set-A<br>**Y**	**B**<br>Data Set-B<br>**Y**	**C**<br>Data Set-C<br>**Y**	**D**<br>Data Set-D<br>**Y**
**1**	Compare cell means regardless of rows and columns				
**2**					
**3**	Number of families	1			
**4**	Number of comparisons per family	105			
**5**	Alpha	0.05			
**6**					
**7**	Tukey's multiple comparisons test	Mean Diff.	95% CI of diff.	Significant?	Summary
**8**					
**9**	Mid Log:**0 X** vs. Mid Log:**1 X**	-0.3875	-1.668 to 0.8935	No	ns
**10**	Mid Log:**0 X** vs. Mid Log:**10 X**	-5.270	-6.839 to -3.701	Yes	****
**11**	Mid Log:**0 X** vs. Mid Log:**40 X**	-0.4650	-2.034 to 1.104	No	ns
**12**	Mid Log:**0 X** vs. Mid Log:**50 X**	-2.093	-3.373 to -0.8115	Yes	**
**13**	Mid Log:**0 X** vs. Late Log:**0 X**	-0.4650	-2.034 to 1.104	No	ns
**14**	Mid Log:**0 X** vs. Late Log:**1 X**	5.960e-008	-1.569 to 1.569	No	ns
**15**	Mid Log:**0 X** vs. Late Log:**10 X**	0.3100	-1.259 to 1.879	No	ns
**16**	Mid Log:**0 X** vs. Late Log:**40 X**	-0.6200	-1.901 to 0.6610	No	ns
**17**	Mid Log:**0 X** vs. Late Log:**50 X**	-1.395	-2.676 to -0.1140	Yes	*
**18**	Mid Log:**0 X** vs. Stationary:**0 X**	-0.6975	-1.978 to 0.5835	No	ns
**19**	Mid Log:**0 X** vs. Stationary:**1 X**	-1.085	-2.366 to 0.1960	No	ns
**20**	Mid Log:**0 X** vs. Stationary:**10 X**	-3.410	-4.979 to -1.841	Yes	***
**21**	Mid Log:**0 X** vs. Stationary:**40 X**	-8.370	-9.939 to -6.801	Yes	****
**22**	Mid Log:**0 X** vs. Stationary:**50 X**	-1.318	-2.598 to -0.03653	Yes	*
**23**	Mid Log:**1 X** vs. Mid Log:**10 X**	-4.883	-6.451 to -3.314	Yes	****
**24**	Mid Log:**1 X** vs. Mid Log:**40 X**	-0.07750	-1.646 to 1.491	No	ns
**25**	Mid Log:**1 X** vs. Mid Log:**50 X**	-1.705	-2.986 to -0.4240	Yes	**
**26**	Mid Log:**1 X** vs. Late Log:**0 X**	-0.07750	-1.646 to 1.491	No	ns
**27**	Mid Log:**1 X** vs. Late Log:**1 X**	0.3875	-1.181 to 1.956	No	ns
**28**	Mid Log:**1 X** vs. Late Log:**10 X**	0.6975	-0.8714 to 2.266	No	ns
**29**	Mid Log:**1 X** vs. Late Log:**40 X**	-0.2325	-1.513 to 1.048	No	ns
**30**	Mid Log:**1 X** vs. Late Log:**50 X**	-1.008	-2.288 to 0.2735	No	ns
**31**	Mid Log:**1 X** vs. Stationary:**0 X**	-0.3100	-1.591 to 0.9710	No	ns
**32**	Mid Log:**1 X** vs. Stationary:**1 X**	-0.6975	-1.978 to 0.5835	No	ns
**33**	Mid Log:**1 X** vs. Stationary:**10 X**	-3.023	-4.591 to -1.454	Yes	***
**34**	Mid Log:**1 X** vs. Stationary:**40 X**	-7.983	-9.551 to -6.414	Yes	****
**35**	Mid Log:**1 X** vs. Stationary:**50 X**	-0.9300	-2.211 to 0.3510	No	ns
**36**	Mid Log:**10 X** vs. Mid Log:**40 X**	4.805	2.993 to 6.617	Yes	****
**37**	Mid Log:**10 X** vs. Mid Log:**50 X**	3.178	1.609 to 4.746	Yes	***
**38**	Mid Log:**10 X** vs. Late Log:**0 X**	4.805	2.993 to 6.617	Yes	****
**39**	Mid Log:**10 X** vs. Late Log:**1 X**	5.270	3.458 to 7.082	Yes	****
**40**	Mid Log:**10 X** vs. Late Log:**10 X**	5.580	3.768 to 7.392	Yes	****
**41**	Mid Log:**10 X** vs. Late Log:**40 X**	4.650	3.081 to 6.219	Yes	****
**42**	Mid Log:**10 X** vs. Late Log:**50 X**	3.875	2.306 to 5.444	Yes	***
**43**	Mid Log:**10 X** vs. Stationary:**0 X**	4.573	3.004 to 6.141	Yes	****
**44**	Mid Log:**10 X** vs. Stationary:**1 X**	4.185	2.616 to 5.754	Yes	****
**45**	Mid Log:**10 X** vs. Stationary:**10 X**	1.860	0.04843 to 3.672	Yes	*
**46**	Mid Log:**10 X** vs. Stationary:**40 X**	-3.100	-4.912 to -1.288	Yes	**
**47**	Mid Log:**10 X** vs. Stationary:**50 X**	3.953	2.384 to 5.521	Yes	***
**48**	Mid Log:**40 X** vs. Mid Log:**50 X**	-1.628	-3.196 to -0.05864	Yes	*
**49**	Mid Log:**40 X** vs. Late Log:**0 X**	0.0	-1.812 to 1.812	No	ns
**50**	Mid Log:**40 X** vs. Late Log:**1 X**	0.4650	-1.347 to 2.277	No	ns

	2way ANOVA<br>Multiple comparisons	A<br>Data Set-A<br>Y	B<br>Data Set-B<br>Y	C<br>Data Set-C<br>Y	D<br>Data Set-D<br>Y
**51**	Mid Log:**40 X** vs. Late Log:**10 X**	0.7750	-1.037 to 2.587	No	ns
**52**	Mid Log:**40 X** vs. Late Log:**40 X**	-0.1550	-1.724 to 1.414	No	ns
**53**	Mid Log:**40 X** vs. Late Log:**50 X**	-0.9300	-2.499 to 0.6389	No	ns
**54**	Mid Log:**40 X** vs. Stationary:**0 X**	-0.2325	-1.801 to 1.336	No	ns
**55**	Mid Log:**40 X** vs. Stationary:**1 X**	-0.6200	-2.189 to 0.9489	No	ns
**56**	Mid Log:**40 X** vs. Stationary:**10 X**	-2.945	-4.757 to -1.133	Yes	**
**57**	Mid Log:**40 X** vs. Stationary:**40 X**	-7.905	-9.717 to -6.093	Yes	****
**58**	Mid Log:**40 X** vs. Stationary:**50 X**	-0.8525	-2.421 to 0.7164	No	ns
**59**	Mid Log:**50 X** vs. Late Log:**0 X**	1.628	0.05864 to 3.196	Yes	*
**60**	Mid Log:**50 X** vs. Late Log:**1 X**	2.093	0.5236 to 3.661	Yes	**
**61**	Mid Log:**50 X** vs. Late Log:**10 X**	2.403	0.8336 to 3.971	Yes	**
**62**	Mid Log:**50 X** vs. Late Log:**40 X**	1.473	0.1915 to 2.753	Yes	*
**63**	Mid Log:**50 X** vs. Late Log:**50 X**	0.6975	-0.5835 to 1.978	No	ns
**64**	Mid Log:**50 X** vs. Stationary:**0 X**	1.395	0.1140 to 2.676	Yes	*
**65**	Mid Log:**50 X** vs. Stationary:**1 X**	1.008	-0.2735 to 2.288	No	ns
**66**	Mid Log:**50 X** vs. Stationary:**10 X**	-1.318	-2.886 to 0.2514	No	ns
**67**	Mid Log:**50 X** vs. Stationary:**40 X**	-6.278	-7.846 to -4.709	Yes	****
**68**	Mid Log:**50 X** vs. Stationary:**50 X**	0.7750	-0.5060 to 2.056	No	ns
**69**	Late Log:**0 X** vs. Late Log:**1 X**	0.4650	-1.347 to 2.277	No	ns
**70**	Late Log:**0 X** vs. Late Log:**10 X**	0.7750	-1.037 to 2.587	No	ns
**71**	Late Log:**0 X** vs. Late Log:**40 X**	-0.1550	-1.724 to 1.414	No	ns
**72**	Late Log:**0 X** vs. Late Log:**50 X**	-0.9300	-2.499 to 0.6389	No	ns
**73**	Late Log:**0 X** vs. Stationary:**0 X**	-0.2325	-1.801 to 1.336	No	ns
**74**	Late Log:**0 X** vs. Stationary:**1 X**	-0.6200	-2.189 to 0.9489	No	ns
**75**	Late Log:**0 X** vs. Stationary:**10 X**	-2.945	-4.757 to -1.133	Yes	**
**76**	Late Log:**0 X** vs. Stationary:**40 X**	-7.905	-9.717 to -6.093	Yes	****
**77**	Late Log:**0 X** vs. Stationary:**50 X**	-0.8525	-2.421 to 0.7164	No	ns
**78**	Late Log:**1 X** vs. Late Log:**10 X**	0.3100	-1.502 to 2.122	No	ns
**79**	Late Log:**1 X** vs. Late Log:**40 X**	-0.6200	-2.189 to 0.9489	No	ns
**80**	Late Log:**1 X** vs. Late Log:**50 X**	-1.395	-2.964 to 0.1739	No	ns
**81**	Late Log:**1 X** vs. Stationary:**0 X**	-0.6975	-2.266 to 0.8714	No	ns
**82**	Late Log:**1 X** vs. Stationary:**1 X**	-1.085	-2.654 to 0.4839	No	ns
**83**	Late Log:**1 X** vs. Stationary:**10 X**	-3.410	-5.222 to -1.598	Yes	***
**84**	Late Log:**1 X** vs. Stationary:**40 X**	-8.370	-10.18 to -6.558	Yes	****
**85**	Late Log:**1 X** vs. Stationary:**50 X**	-1.318	-2.886 to 0.2514	No	ns
**86**	Late Log:**10 X** vs. Late Log:**40 X**	-0.9300	-2.499 to 0.6389	No	ns
**87**	Late Log:**10 X** vs. Late Log:**50 X**	-1.705	-3.274 to -0.1361	Yes	*
**88**	Late Log:**10 X** vs. Stationary:**0 X**	-1.008	-2.576 to 0.5614	No	ns
**89**	Late Log:**10 X** vs. Stationary:**1 X**	-1.395	-2.964 to 0.1739	No	ns
**90**	Late Log:**10 X** vs. Stationary:**10 X**	-3.720	-5.532 to -1.908	Yes	***
**91**	Late Log:**10 X** vs. Stationary:**40 X**	-8.680	-10.49 to -6.868	Yes	****
**92**	Late Log:**10 X** vs. Stationary:**50 X**	-1.628	-3.196 to -0.05864	Yes	*
**93**	Late Log:**40 X** vs. Late Log:**50 X**	-0.7750	-2.056 to 0.5060	No	ns
**94**	Late Log:**40 X** vs. Stationary:**0 X**	-0.07750	-1.358 to 1.203	No	ns
**95**	Late Log:**40 X** vs. Stationary:**1 X**	-0.4650	-1.746 to 0.8160	No	ns
**96**	Late Log:**40 X** vs. Stationary:**10 X**	-2.790	-4.359 to -1.221	Yes	**
**97**	Late Log:**40 X** vs. Stationary:**40 X**	-7.750	-9.319 to -6.181	Yes	****
**98**	Late Log:**40 X** vs. Stationary:**50 X**	-0.6975	-1.978 to 0.5835	No	ns
**99**	Late Log:**50 X** vs. Stationary:**0 X**	0.6975	-0.5835 to 1.978	No	ns
**100**	Late Log:**50 X** vs. Stationary:**1 X**	0.3100	-0.9710 to 1.591	No	ns

	2way ANOVA<br>Multiple comparisons	A<br>Data Set-A<br>Y	B<br>Data Set-B<br>Y	C<br>Data Set-C<br>Y	D<br>Data Set-D<br>Y
101	Late Log:**50 X** vs. Stationary:**10 X**	-2.015	-3.584 to -0.4461	Yes	*
102	Late Log:**50 X** vs. Stationary:**40 X**	-6.975	-8.544 to -5.406	Yes	****
103	Late Log:**50 X** vs. Stationary:**50 X**	0.07750	-1.203 to 1.358	No	ns
104	Stationary:**0 X** vs. Stationary:**1 X**	-0.3875	-1.668 to 0.8935	No	ns
105	Stationary:**0 X** vs. Stationary:**10 X**	-2.713	-4.281 to -1.144	Yes	**
106	Stationary:**0 X** vs. Stationary:**40 X**	-7.673	-9.241 to -6.104	Yes	****
107	Stationary:**0 X** vs. Stationary:**50 X**	-0.6200	-1.901 to 0.6610	No	ns
108	Stationary:**1 X** vs. Stationary:**10 X**	-2.325	-3.894 to -0.7561	Yes	**
109	Stationary:**1 X** vs. Stationary:**40 X**	-7.285	-8.854 to -5.716	Yes	****
110	Stationary:**1 X** vs. Stationary:**50 X**	-0.2325	-1.513 to 1.048	No	ns
111	Stationary:**10 X** vs. Stationary:**40 X**	-4.960	-6.772 to -3.148	Yes	****
112	Stationary:**10 X** vs. Stationary:**50 X**	2.093	0.5236 to 3.661	Yes	**
113	Stationary:**40 X** vs. Stationary:**50 X**	7.053	5.484 to 8.621	Yes	****
114					
115					
116	Test details	Mean 1	Mean 2	Mean Diff.	SE of diff.
117					
118	Mid Log:**0 X** vs. Mid Log:**1 X**	1.550	1.938	-0.3875	0.2794
119	Mid Log:**0 X** vs. Mid Log:**10 X**	1.550	6.820	-5.270	0.3422
120	Mid Log:**0 X** vs. Mid Log:**40 X**	1.550	2.015	-0.4650	0.3422
121	Mid Log:**0 X** vs. Mid Log:**50 X**	1.550	3.643	-2.093	0.2794
122	Mid Log:**0 X** vs. Late Log:**0 X**	1.550	2.015	-0.4650	0.3422
123	Mid Log:**0 X** vs. Late Log:**1 X**	1.550	1.550	5.960e-008	0.3422
124	Mid Log:**0 X** vs. Late Log:**10 X**	1.550	1.240	0.3100	0.3422
125	Mid Log:**0 X** vs. Late Log:**40 X**	1.550	2.170	-0.6200	0.2794
126	Mid Log:**0 X** vs. Late Log:**50 X**	1.550	2.945	-1.395	0.2794
127	Mid Log:**0 X** vs. Stationary:**0 X**	1.550	2.248	-0.6975	0.2794
128	Mid Log:**0 X** vs. Stationary:**1 X**	1.550	2.635	-1.085	0.2794
129	Mid Log:**0 X** vs. Stationary:**10 X**	1.550	4.960	-3.410	0.3422
130	Mid Log:**0 X** vs. Stationary:**40 X**	1.550	9.920	-8.370	0.3422
131	Mid Log:**0 X** vs. Stationary:**50 X**	1.550	2.868	-1.318	0.2794
132	Mid Log:**1 X** vs. Mid Log:**10 X**	1.938	6.820	-4.883	0.3422
133	Mid Log:**1 X** vs. Mid Log:**40 X**	1.938	2.015	-0.07750	0.3422
134	Mid Log:**1 X** vs. Mid Log:**50 X**	1.938	3.643	-1.705	0.2794
135	Mid Log:**1 X** vs. Late Log:**0 X**	1.938	2.015	-0.07750	0.3422
136	Mid Log:**1 X** vs. Late Log:**1 X**	1.938	1.550	0.3875	0.3422
137	Mid Log:**1 X** vs. Late Log:**10 X**	1.938	1.240	0.6975	0.3422
138	Mid Log:**1 X** vs. Late Log:**40 X**	1.938	2.170	-0.2325	0.2794
139	Mid Log:**1 X** vs. Late Log:**50 X**	1.938	2.945	-1.008	0.2794
140	Mid Log:**1 X** vs. Stationary:**0 X**	1.938	2.248	-0.3100	0.2794
141	Mid Log:**1 X** vs. Stationary:**1 X**	1.938	2.635	-0.6975	0.2794
142	Mid Log:**1 X** vs. Stationary:**10 X**	1.938	4.960	-3.023	0.3422
143	Mid Log:**1 X** vs. Stationary:**40 X**	1.938	9.920	-7.983	0.3422
144	Mid Log:**1 X** vs. Stationary:**50 X**	1.938	2.868	-0.9300	0.2794
145	Mid Log:**10 X** vs. Mid Log:**40 X**	6.820	2.015	4.805	0.3952
146	Mid Log:**10 X** vs. Mid Log:**50 X**	6.820	3.643	3.178	0.3422
147	Mid Log:**10 X** vs. Late Log:**0 X**	6.820	2.015	4.805	0.3952
148	Mid Log:**10 X** vs. Late Log:**1 X**	6.820	1.550	5.270	0.3952
149	Mid Log:**10 X** vs. Late Log:**10 X**	6.820	1.240	5.580	0.3952
150	Mid Log:**10 X** vs. Late Log:**40 X**	6.820	2.170	4.650	0.3422

	2way ANOVA<br>Multiple comparisons	A<br>Data Set-A<br>Y	B<br>Data Set-B<br>Y	C<br>Data Set-C<br>Y	D<br>Data Set-D<br>Y
**151**	Mid Log:**10 X** vs. Late Log:**50 X**	6.820	2.945	3.875	0.3422
**152**	Mid Log:**10 X** vs. Stationary:**0 X**	6.820	2.248	4.573	0.3422
**153**	Mid Log:**10 X** vs. Stationary:**1 X**	6.820	2.635	4.185	0.3422
**154**	Mid Log:**10 X** vs. Stationary:**10 X**	6.820	4.960	1.860	0.3952
**155**	Mid Log:**10 X** vs. Stationary:**40 X**	6.820	9.920	-3.100	0.3952
**156**	Mid Log:**10 X** vs. Stationary:**50 X**	6.820	2.868	3.953	0.3422
**157**	Mid Log:**40 X** vs. Mid Log:**50 X**	2.015	3.643	-1.628	0.3422
**158**	Mid Log:**40 X** vs. Late Log:**0 X**	2.015	2.015	0.0	0.3952
**159**	Mid Log:**40 X** vs. Late Log:**1 X**	2.015	1.550	0.4650	0.3952
**160**	Mid Log:**40 X** vs. Late Log:**10 X**	2.015	1.240	0.7750	0.3952
**161**	Mid Log:**40 X** vs. Late Log:**40 X**	2.015	2.170	-0.1550	0.3422
**162**	Mid Log:**40 X** vs. Late Log:**50 X**	2.015	2.945	-0.9300	0.3422
**163**	Mid Log:**40 X** vs. Stationary:**0 X**	2.015	2.248	-0.2325	0.3422
**164**	Mid Log:**40 X** vs. Stationary:**1 X**	2.015	2.635	-0.6200	0.3422
**165**	Mid Log:**40 X** vs. Stationary:**10 X**	2.015	4.960	-2.945	0.3952
**166**	Mid Log:**40 X** vs. Stationary:**40 X**	2.015	9.920	-7.905	0.3952
**167**	Mid Log:**40 X** vs. Stationary:**50 X**	2.015	2.868	-0.8525	0.3422
**168**	Mid Log:**50 X** vs. Late Log:**0 X**	3.643	2.015	1.628	0.3422
**169**	Mid Log:**50 X** vs. Late Log:**1 X**	3.643	1.550	2.093	0.3422
**170**	Mid Log:**50 X** vs. Late Log:**10 X**	3.643	1.240	2.403	0.3422
**171**	Mid Log:**50 X** vs. Late Log:**40 X**	3.643	2.170	1.473	0.2794
**172**	Mid Log:**50 X** vs. Late Log:**50 X**	3.643	2.945	0.6975	0.2794
**173**	Mid Log:**50 X** vs. Stationary:**0 X**	3.643	2.248	1.395	0.2794
**174**	Mid Log:**50 X** vs. Stationary:**1 X**	3.643	2.635	1.008	0.2794
**175**	Mid Log:**50 X** vs. Stationary:**10 X**	3.643	4.960	-1.318	0.3422
**176**	Mid Log:**50 X** vs. Stationary:**40 X**	3.643	9.920	-6.278	0.3422
**177**	Mid Log:**50 X** vs. Stationary:**50 X**	3.643	2.868	0.7750	0.2794
**178**	Late Log:**0 X** vs. Late Log:**1 X**	2.015	1.550	0.4650	0.3952
**179**	Late Log:**0 X** vs. Late Log:**10 X**	2.015	1.240	0.7750	0.3952
**180**	Late Log:**0 X** vs. Late Log:**40 X**	2.015	2.170	-0.1550	0.3422
**181**	Late Log:**0 X** vs. Late Log:**50 X**	2.015	2.945	-0.9300	0.3422
**182**	Late Log:**0 X** vs. Stationary:**0 X**	2.015	2.248	-0.2325	0.3422
**183**	Late Log:**0 X** vs. Stationary:**1 X**	2.015	2.635	-0.6200	0.3422
**184**	Late Log:**0 X** vs. Stationary:**10 X**	2.015	4.960	-2.945	0.3952
**185**	Late Log:**0 X** vs. Stationary:**40 X**	2.015	9.920	-7.905	0.3952
**186**	Late Log:**0 X** vs. Stationary:**50 X**	2.015	2.868	-0.8525	0.3422
**187**	Late Log:**1 X** vs. Late Log:**10 X**	1.550	1.240	0.3100	0.3952
**188**	Late Log:**1 X** vs. Late Log:**40 X**	1.550	2.170	-0.6200	0.3422
**189**	Late Log:**1 X** vs. Late Log:**50 X**	1.550	2.945	-1.395	0.3422
**190**	Late Log:**1 X** vs. Stationary:**0 X**	1.550	2.248	-0.6975	0.3422
**191**	Late Log:**1 X** vs. Stationary:**1 X**	1.550	2.635	-1.085	0.3422
**192**	Late Log:**1 X** vs. Stationary:**10 X**	1.550	4.960	-3.410	0.3952
**193**	Late Log:**1 X** vs. Stationary:**40 X**	1.550	9.920	-8.370	0.3952
**194**	Late Log:**1 X** vs. Stationary:**50 X**	1.550	2.868	-1.318	0.3422
**195**	Late Log:**10 X** vs. Late Log:**40 X**	1.240	2.170	-0.9300	0.3422
**196**	Late Log:**10 X** vs. Late Log:**50 X**	1.240	2.945	-1.705	0.3422
**197**	Late Log:**10 X** vs. Stationary:**0 X**	1.240	2.248	-1.008	0.3422
**198**	Late Log:**10 X** vs. Stationary:**1 X**	1.240	2.635	-1.395	0.3422
**199**	Late Log:**10 X** vs. Stationary:**10 X**	1.240	4.960	-3.720	0.3952
**200**	Late Log:**10 X** vs. Stationary:**40 X**	1.240	9.920	-8.680	0.3952

	2way ANOVA<br>Multiple comparisons	A<br>Data Set-A<br>Y	B<br>Data Set-B<br>Y	C<br>Data Set-C<br>Y	D<br>Data Set-D<br>Y
**201**	Late Log:**10 X** vs. Stationary:**50 X**	1.240	2.868	-1.628	0.3422
**202**	Late Log:**40 X** vs. Late Log:**50 X**	2.170	2.945	-0.7750	0.2794
**203**	Late Log:**40 X** vs. Stationary:**0 X**	2.170	2.248	-0.07750	0.2794
**204**	Late Log:**40 X** vs. Stationary:**1 X**	2.170	2.635	-0.4650	0.2794
**205**	Late Log:**40 X** vs. Stationary:**10 X**	2.170	4.960	-2.790	0.3422
**206**	Late Log:**40 X** vs. Stationary:**40 X**	2.170	9.920	-7.750	0.3422
**207**	Late Log:**40 X** vs. Stationary:**50 X**	2.170	2.868	-0.6975	0.2794
**208**	Late Log:**50 X** vs. Stationary:**0 X**	2.945	2.248	0.6975	0.2794
**209**	Late Log:**50 X** vs. Stationary:**1 X**	2.945	2.635	0.3100	0.2794
**210**	Late Log:**50 X** vs. Stationary:**10 X**	2.945	4.960	-2.015	0.3422
**211**	Late Log:**50 X** vs. Stationary:**40 X**	2.945	9.920	-6.975	0.3422
**212**	Late Log:**50 X** vs. Stationary:**50 X**	2.945	2.868	0.07750	0.2794
**213**	Stationary:**0 X** vs. Stationary:**1 X**	2.248	2.635	-0.3875	0.2794
**214**	Stationary:**0 X** vs. Stationary:**10 X**	2.248	4.960	-2.713	0.3422
**215**	Stationary:**0 X** vs. Stationary:**40 X**	2.248	9.920	-7.673	0.3422
**216**	Stationary:**0 X** vs. Stationary:**50 X**	2.248	2.868	-0.6200	0.2794
**217**	Stationary:**1 X** vs. Stationary:**10 X**	2.635	4.960	-2.325	0.3422
**218**	Stationary:**1 X** vs. Stationary:**40 X**	2.635	9.920	-7.285	0.3422
**219**	Stationary:**1 X** vs. Stationary:**50 X**	2.635	2.868	-0.2325	0.2794
**220**	Stationary:**10 X** vs. Stationary:**40 X**	4.960	9.920	-4.960	0.3952
**221**	Stationary:**10 X** vs. Stationary:**50 X**	4.960	2.868	2.093	0.3422
**222**	Stationary:**40 X** vs. Stationary:**50 X**	9.920	2.868	7.053	0.3422
