# Supplementary material for: Changes in fatty acid composition as a response to glyphosate toxicity in Pseudomonas fluorescens
Source: Heliyon. 2022 Jul 13;8(8):e09938. doi: 10.1016/j.heliyon.2022.e09938 (PMC9364109; doi:10.1016/j.heliyon.2022.e09938)
Supplement: Multimedia component 3 [file mmc3.docx]

**Supplementary Material 3**: Correlation of treatments 20 h, 30 h and 40 h, corresponding to early-log, mid-log and stationary growing phases, respectively.


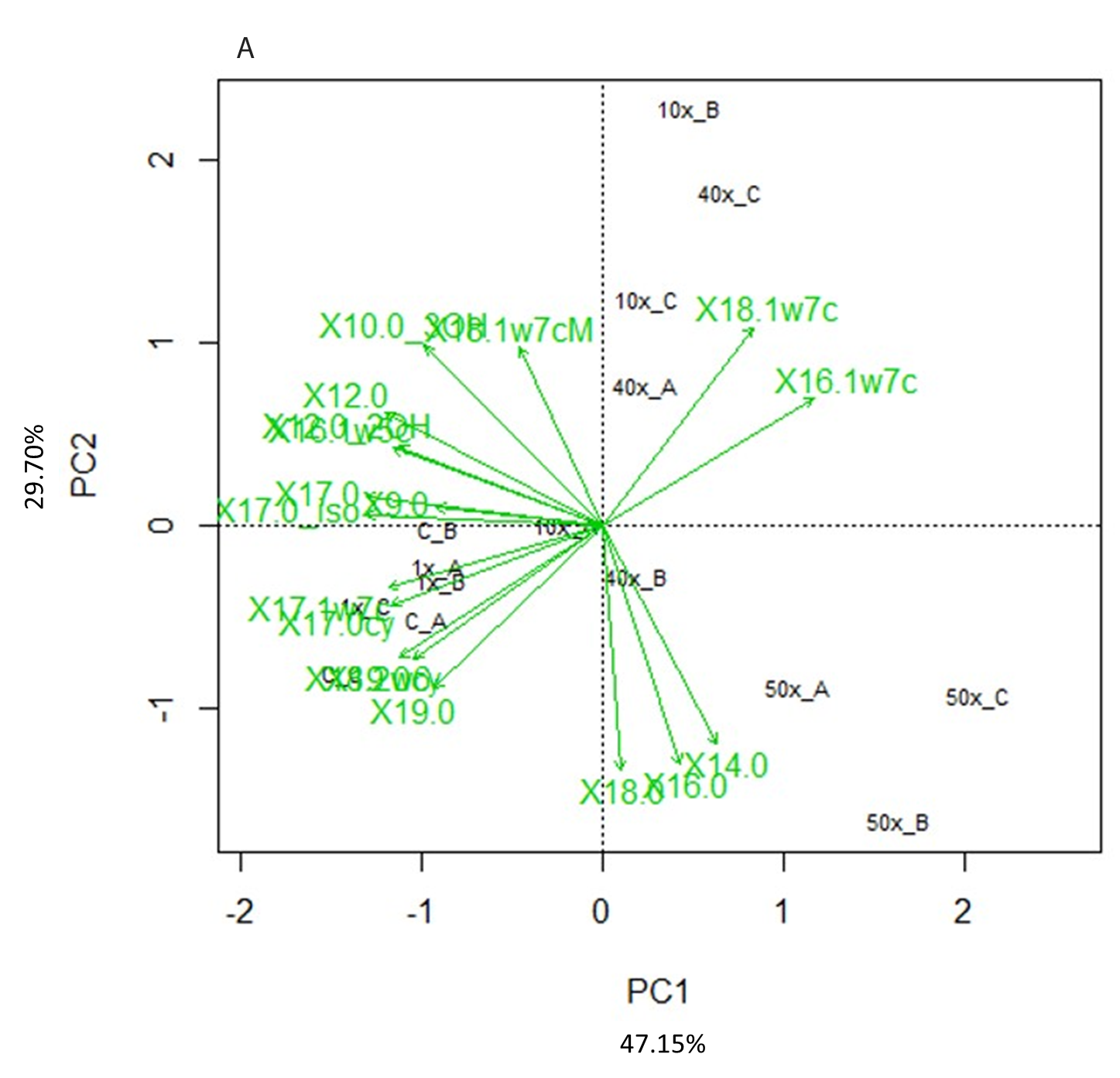


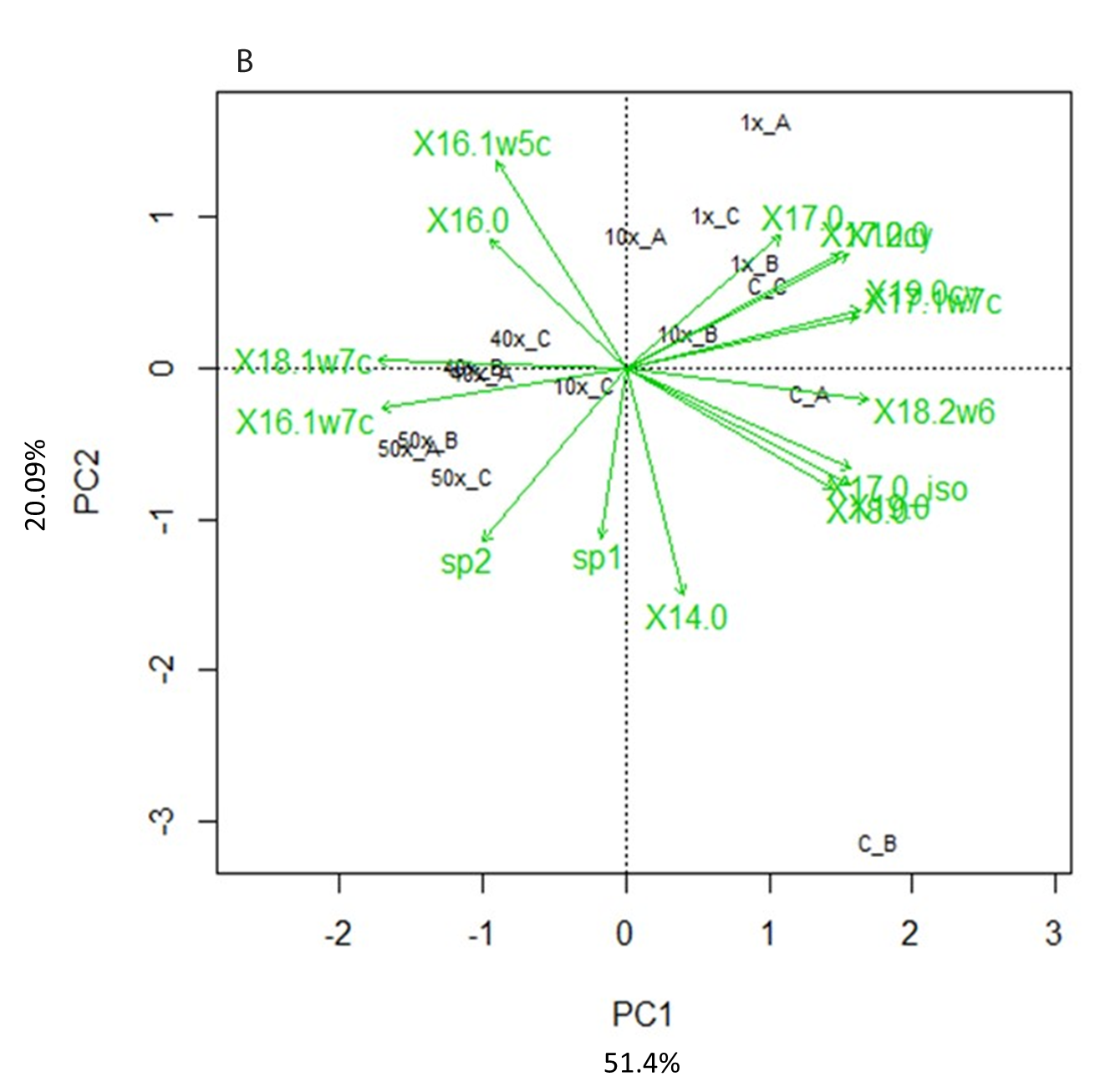


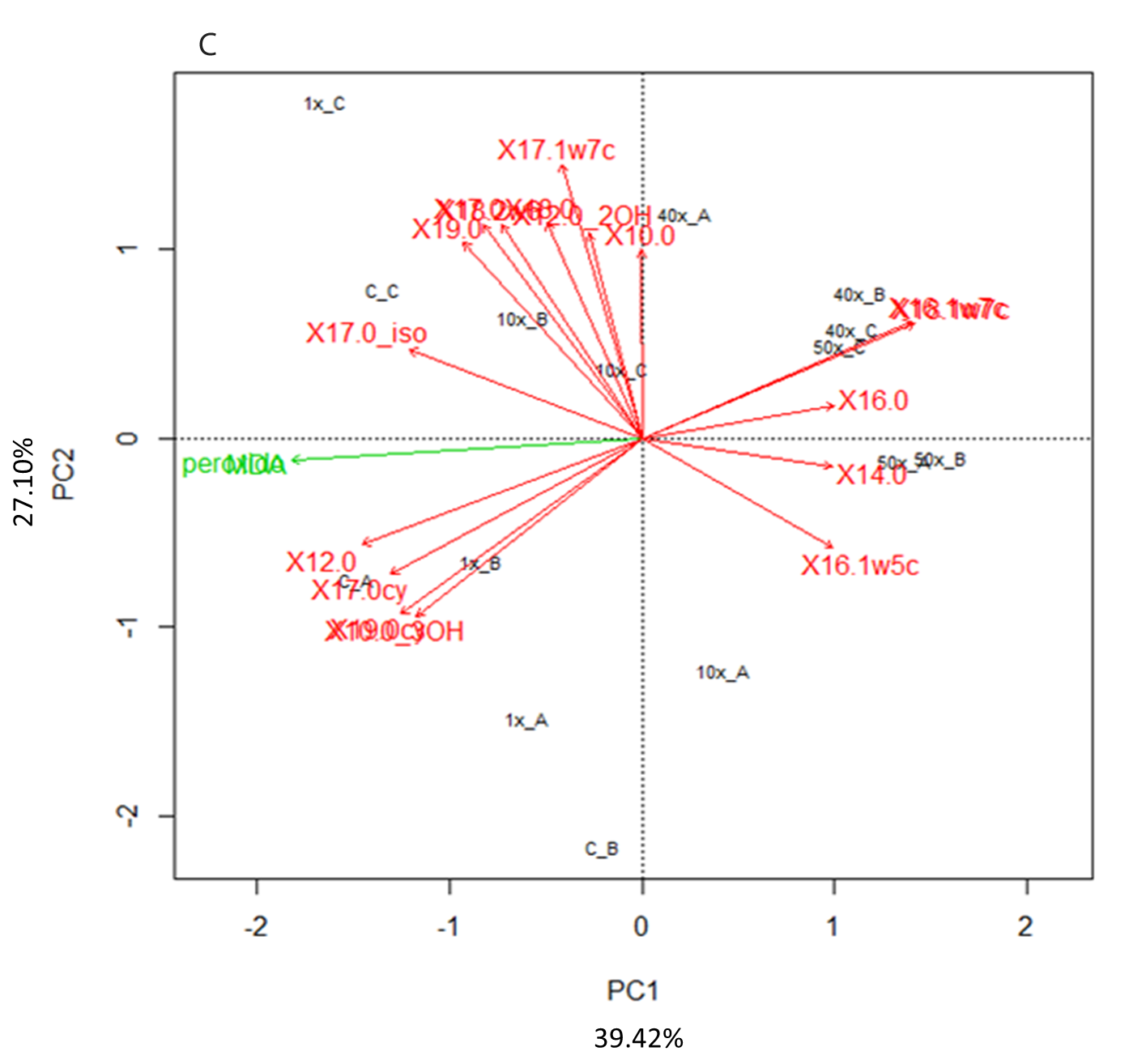


39.42%

Supplementary Material 3: Ordering of bacterial treatment with glyphosate herbicide based on fatty acid composition, H_2_O_2_, and MDA. Only parameters with P <0.05 for significance after 999 permutations are printed. Each vector indicates a direction of increase of a variable, and its length indicates the strength of correlation between a variable and an ordering score. Groups according to the order of glyphosate treatments in correlation with the types of identified lipids. Panel A: early-log growing phase; Panel B: mid-log growing phase; Panel C: stationary growing phase.
