# Supplementary material for: Changes in fatty acid composition as a response to glyphosate toxicity in Pseudomonas fluorescens
Source: Heliyon. 2022 Jul 13;8(8):e09938. doi: 10.1016/j.heliyon.2022.e09938 (PMC9364109; doi:10.1016/j.heliyon.2022.e09938)
Supplement: Multimedia component 4 [file mmc4.docx]

**Supplementary Material 4**

0x 1x 10x 40x 50x





Mn-SOD

Early-log

0x 1x 10x 40x 50x





Mn-SOD

Mid-log

0x 1x 10x 40x 50x



Mn-SOD

Stationary

**Supplementary Material for Figure 5.** Non-adjusted images for characterization of SOD isoforms in PAGE, which were obtained from the extracts of *P. fluorescens* CMA-55, in PAGE, in the treatments with 0x, 1x, 10x, 40x, and 50x glyphosate concentrations, in the early-log, mid-log, and stationary growing phases.
