# Supplementary material for: Changes in fatty acid composition as a response to glyphosate toxicity in Pseudomonas fluorescens
Source: Heliyon. 2022 Jul 13;8(8):e09938. doi: 10.1016/j.heliyon.2022.e09938 (PMC9364109; doi:10.1016/j.heliyon.2022.e09938)
Supplement: Multimedia component 5 [file mmc5.docx]

**Supplementary Material 5**

0x 1x 10x 40x 50x


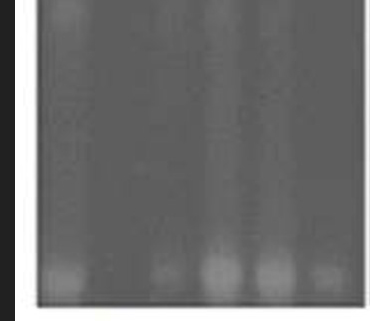
KatB

KatC

KatA

Early-log

0x 1x 10x 40x 50x


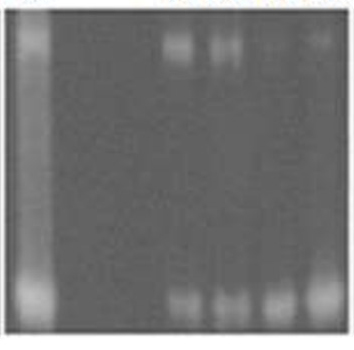


KatB

KatC

KatA

Mid-log

0x 1x 10x 40x 50x


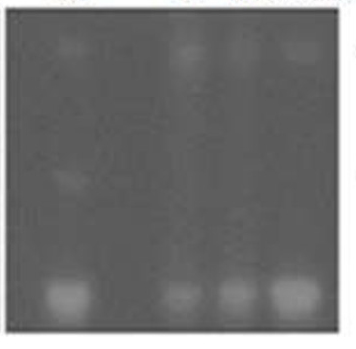
KatB

KatC

KatA

Stationary

**Supplementary Material for Figure 6.** Non-adjusted images for CAT gel activity in 0x, 1x, 10x, 40x, and 50x treatments, in the early-log and mid-log growing phases of *P. fluorescens* CMA-55. No band was observed at the 50x stationary growth phase. KatA, KatB, and additional band KatC isoforms were observed.
